# Differential long non-coding RNA expression profiles in human oocytes and cumulus cells

**DOI:** 10.1038/s41598-018-20727-0

**Published:** 2018-02-02

**Authors:** Julien Bouckenheimer, Patricia Fauque, Charles-Henri Lecellier, Céline Bruno, Thérèse Commes, Jean-Marc Lemaître, John De Vos, Said Assou

**Affiliations:** 1 IRMB, Univ Montpellier, INSERM, CHU Montpellier, Montpellier, F34000 France; 2 Laboratoire de Biologie de la Reproduction, Hôpital François Mitterrand, Université de Bourgogne, Dijon, France; 3 Institut de Biologie Computationnelle, IGMM, CNRS, Montpellier, F34000 France; 4 Stem Cell Core Facility SAFE-iPSC, INGESTEM, Saint-Eloi Hospital Montpellier, Montpellier, F34000 France; 5 Department of Cell and Tissue Engineering, Saint-Eloi Hospital, Montpellier, F34000 France

## Abstract

Progress in assisted reproductive technologies strongly relies on understanding the regulation of the dialogue between oocyte and cumulus cells (CCs). Little is known about the role of long non-coding RNAs (lncRNAs) in the human cumulus-oocyte complex (COC). To this aim, publicly available RNA-sequencing data were analyzed to identify lncRNAs that were abundant in metaphase II (MII) oocytes (*BCAR4*, *C3orf56, TUNAR, OOEP-AS1*, *CASC18*, and *LINC01118*) and CCs (*NEAT1*, *MALAT1*, *ANXA2P2, MEG3*, *IL6STP1*, and *VIM-AS1*). These data were validated by RT-qPCR analysis using independent oocytes and CC samples. The functions of the identified lncRNAs were then predicted by constructing lncRNA-mRNA co-expression networks. This analysis suggested that MII oocyte lncRNAs could be involved in chromatin remodeling, cell pluripotency and in driving early embryonic development. CC lncRNAs were co-expressed with genes involved in apoptosis and extracellular matrix-related functions. A bioinformatic analysis of RNA-sequencing data to identify CC lncRNAs that are affected by maternal age showed that lncRNAs with age-related altered expression in CCs are essential for oocyte growth. This comprehensive analysis of lncRNAs expressed in human MII oocytes and CCs could provide biomarkers of oocyte quality for the development of non-invasive tests to identify embryos with high developmental potential.

## Introduction

The main function of ovarian folliculogenesis is to generate mature and competent oocytes for reproduction. To achieve this, the dialogue between follicle somatic and germinal cells must be precisely coordinated. Two somatic cell populations can be distinguished in the ovarian follicle: mural granulosa cells that line the wall of antral follicle, and cumulus cells (CCs) that are in physical contact with the oocyte, thus creating a specialized structure, named cumulus–oocyte complex (COC)^1^. The bidirectional communication between the oocyte and the surrounding CCs through gap junctions is crucial for the development of the follicular compartments and the acquisition of oocyte competence^2–4^. Indeed, CCs provide several trophic and metabolic factors to the pre-ovulatory oocyte^5^. The oocyte regulates CC metabolism and synthesizes and accumulates transcripts that are essential for embryo development^6,7^. Many studies on human oocytes and CCs have provided information on cell-type specific coding RNAs, and have suggested that CC transcriptomic analysis could be used as a non-invasive tool to assess oocyte quality and pregnancy outcome [for review see ref.^8–10^].

Substantial advances in next-generation sequencing technologies have revolutionized our understanding of the human genome architecture. Particularly, it is now clear that a significant portion of our genome consists of non-coding RNAs^11,12^. Non-coding RNAs can be grouped in two main classes according to their length: small non-coding RNAs (less than 200 nucleotides; for instance, microRNAs, piRNA, snoRNA) and long non-coding RNAs (lncRNAs; more than 200 nucleotides)^13,14^. High-throughput microRNA profile analysis of human ovarian somatic cell samples showed that small non-coding RNAs could play an important role in modulating gonadotropin signaling^15^. The expression of lncRNAs is strikingly more tissue-specific than that of coding RNAs^16^ and they have been found in a stable form in many tissues and body fluids^17–19^. Increasing evidence indicates that lncRNAs can play critical regulatory roles in many biological processes, including the modulation of chromatin states, transcription and post-transcriptional processing^20–23^. In human CCs, it was reported that lncRNAs and coding RNAs are differentially expressed in poor and high-quality embryos, using microarray analysis^24,25^, and in expanded and compact CCs, using global transcriptome sequencing^26^. Moreover, some lncRNAs are aberrantly expressed in CCs of women with polycystic ovary syndrome compared with controls^27^. However, our knowledge of the lncRNA expression profile in human COC is still limited.

In this study, we used publicly available RNA-seq data to investigate COC lncRNA expression profiles. The objectives were: (i) to identify lncRNAs that were differentially expressed between human metaphase II (MII) oocytes and ovarian somatic cells and explore their putative function and regulatory networks, (ii) to investigate the expression profile of MII oocyte lncRNAs during early embryonic development, and (iii) to evaluate the effect of female age on the CC lncRNA expression profile. This study should help us to understand the role of lncRNAs during the acquisition of oocyte competence within the follicle. This knowledge could be used to develop new non-invasive methods based on CC lncRNA expression for embryo selection with the aim of improving *in vitro* fertilization (IVF) outcome.

## Results

### Global gene expression patterns of mature MII oocytes and cumulus granulosa cells

**Figure 1:**
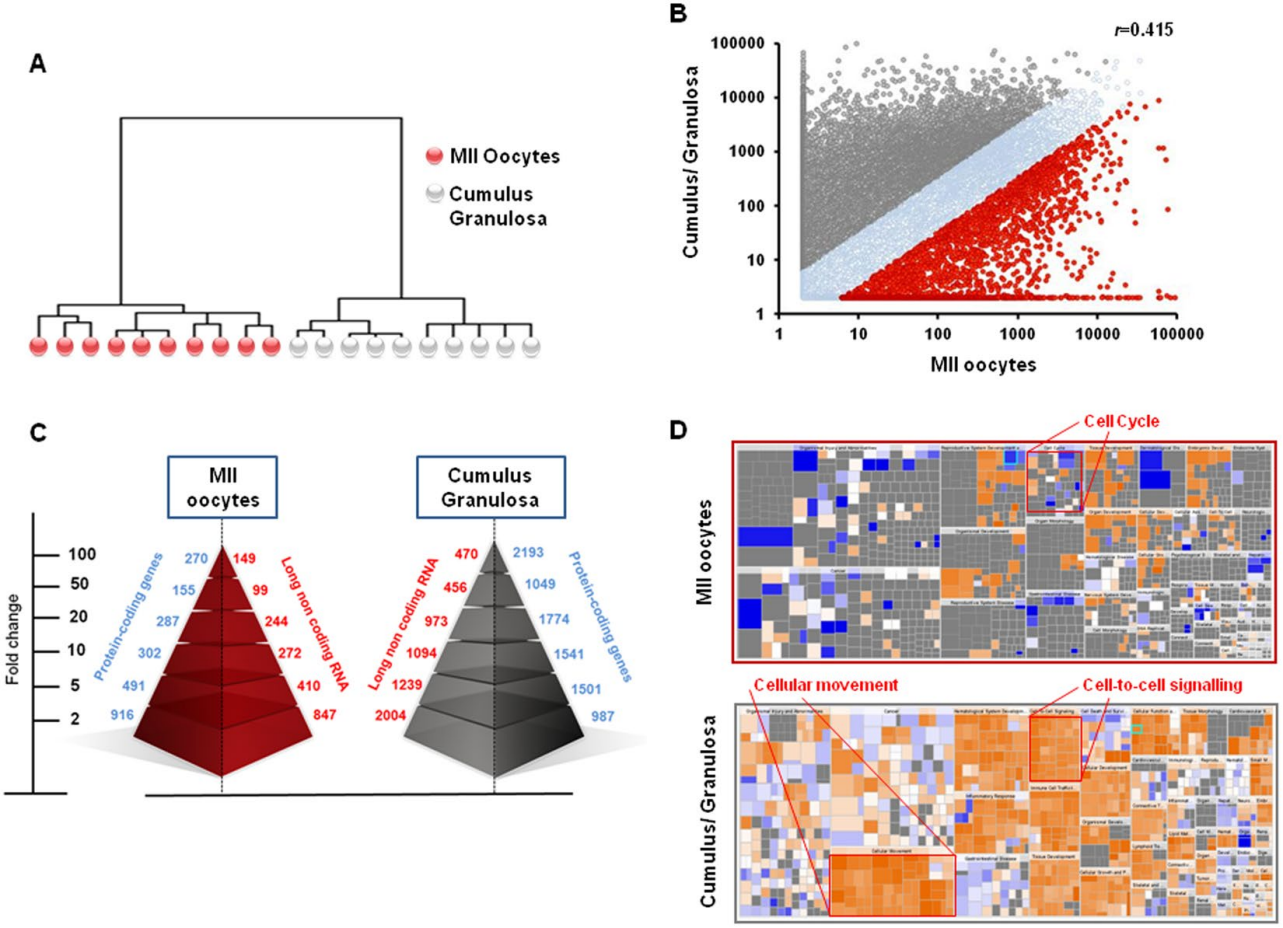
Differences in the global gene expression profiles of human mature MII oocytes and cumulus granulosa samples. (**A**) Unsupervised hierarchical clustering the 10,000 genes with the highest variation coefficient. The dendrogram shows that their expression profiles cluster cumulus granulosa cell and MII oocyte samples (n = 10/each group) in two distinct groups. The 10 oocytes used for RNA-seq were obtained from nine 25- to 35-year-old patients. (**B**) Scatter plot showing the distribution of gene expression fold changes. The Pearson correlation coefficient (*r*) value is shown in the plot. Gene expression data were normalized as Reads Per Kilobase Million (RPKM). (**C**) Number of upregulated protein-coding genes and lncRNAs in MII oocyte and cumulus granulosa samples. SAM analysis identified 2,422 protein-coding genes that are upregulated in MII oocytes and 9,045 protein-coding genes that are upregulated in cumulus granulosa samples. Of these, 270 and 2,193 coding genes showed a fold change ≥100 in MII oocytes and cumulus granulosa cells, respectively. Moreover, 2,021 and 6,236 lncRNAs were upregulated in MII oocytes and in cumulus granulosa samples, respectively. Of these, 149 and 470 lncRNAs showed a fold change ≥100 in MII oocytes and cumulus granulosa cells, respectively. (**D**) Analysis of significantly represented Gene Ontology (GO) terms. Results were generated by IPA of differentially expressed genes. The heat map colors correspond to the relative expression of genes represented in the subgroups of the major annotations terms. For instance, most ‘cell cycle’ genes were upregulated in MII oocytes, whereas most ‘cell-to-cell signaling, cellular movement’ genes were upregulated in cumulus granulosa cells. Orange, upregulated genes; blue, downregulated gene expression.

To determine the global variation of the transcriptome profiles of MII oocyte (i.e., mature oocytes ready for fertilization) and cumulus granulosa samples, we first analyzed RNA-seq data by hierarchical clustering (Fig. 1A). The results showed that all MII oocyte samples clustered together and were well separated from all ovarian somatic cell samples, demonstrating a strong cell type-specific expression profile for each group. This result was also supported by scatter plot analysis (Fig. 1B) that showed a low correlation coefficient (*r* = 0.415) between oocyte and cumulus granulosa samples. To determine the expression profile differences between MII oocytes and cumulus granulosa samples, we performed a differential gene expression analysis. In agreement with previous results^28^, cumulus granulosa cell expression profile included a larger number of protein-coding genes than the oocyte profile (Fig. 1C). Specifically, 2,421 protein-coding genes were significantly upregulated in MII oocytes (“Oocyte protein-coding gene signature”) and 9,045 in cumulus granulosa cells (“CC protein-coding gene signature”) (the transcripts lists are in Supplementary Tables S1 and S2). The oocyte signature included the known oocyte-specific genes *H1FOO*, *DAZL*, *MOS*, *FIGLA*, *GDF9*, *BMP15*, the zona pellucida genes 1 to 4 (*ZP1*, 2, 3 and 4), the developmental pluripotency-associated genes 3 and 5 (*DPPA3*, *DPPA5*), the NLR family (*NLRP2*, 4, 5, 7, 8, 9, 11, 12, 13 and 14) and Aurora kinases (*AURKA*, *AURKB*, *AURKC*). Conversely, the “CC protein-coding gene signature” included stearoyl-CoA desaturase (*SCD*), steroidogenic acute regulatory protein (*STAR*), pentraxin 3 (*PTX3*), gap junction protein alpha (*GJA1*, 4 and 5), inhibins (*INHA*, *INHBA*), receptors (*LHCGR* and *FSHR*), *S100* calcium-binding proteins (*S100A1*, 2, 4, 6, 8, 9, 10, 11, 12, 13 and 16), hydroxysteroid dehydrogenases [(*HSD3B2* and 7; *HSD11B1* and 2; *HSD17B1*, 4, 7, 8, 10, 11, 12 and 14)] and peroxiredoxins (*PRDX1*, 2, 4, 5 and 6). The expression analysis of some of these well-known markers in MII oocyte and cumulus granulosa samples is shown in Supplementary Figure S1. We then performed gene ontology (GO) analysis of these two molecular signatures to identify potential biological processes associated with the MII oocyte- and cumulus granulosa cell-specific profiles. Enriched GO terms revealed that some functional annotations were more represented in the “oocyte protein-coding gene signature”: meiotic cell cycle (GO:0051321; *p*-value = 2.81E-15), mitotic cell cycle (GO:1903047; *p*-value = 3.03E-16), oogenesis (GO:0048477, *p*-value = 2.03E-04), centromere complex assembly (GO:0034508; *p*-value = 5.87E-04), and meiotic chromosome segregation (GO:0045132; *p*-value = 1.73E-07). Conversely, transcripts involved in response to stimulus (GO:0071216; *p*-value = 9.99E-04), protein transport (GO:0015031; *p*-value = 2.35E-11), extracellular matrix organization (GO:0030198; *p*-value = 8.32E-03), apoptosis (GO:0006915; *p*-value = 2.89E-07) and metabolic processes (GO:0043436; *p*-value = 5.52E-10) were more represented in the “CC protein-coding gene signature”. We obtained similar results also when assessing regulatory networks (Fig. 1D). Taken together, these results are in line with the major processes known to occur during folliculogenesis and validated the dataset.

### Differential lncRNA expression in MII oocytes and cumulus granulosa cells

**Figure 2:**
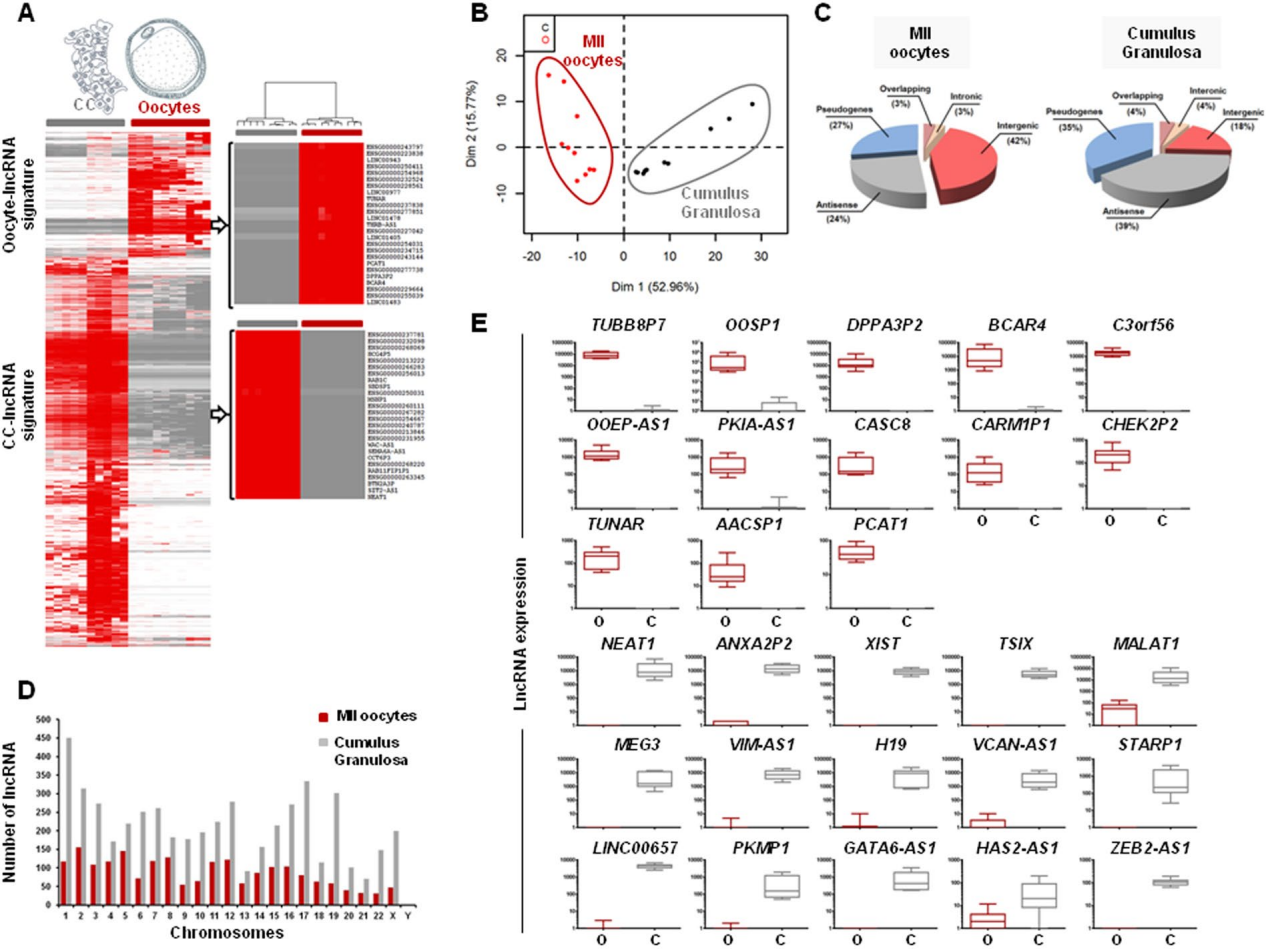
Characterization of lncRNAs that are upregulated in MII oocyte or cumulus granulosa samples. (**A**) Hierarchical clustering of lncRNAs that are differentially expressed in oocytes and cumulus granulosa samples. The color scale illustrates the relative expression level of lncRNAs in the different samples. Red, upregulated genes; gray, downregulated genes. (**B**) Two-dimensional scatter plots (PCA) representing the top 150 lncRNAs that are differentially expressed in MII oocytes and cumulus granulosa cells. Each dot represents a sample; red, MII oocytes; black, cumulus granulosa cells. Samples could be divided in two distinct groups (oocytes or somatic cells) based on their lncRNA expression profiles. (**C**) Pie charts representing lncRNA class distribution (intergenic, antisense, intronic, overlapping and pseudogenes) in MII oocyte and cumulus granulosa samples. (**D**) LncRNA distribution in human chromosomes. The *x-axis* indicates the different human chromosomes, and the *y-axis* indicates the number of differentially expressed lncRNAs transcribed from each chromosome. (**E**) Box-and-whisker plots comparing lncRNAs that are differently expressed (FDR ≤0.05) in MII oocyte (n = 10) and cumulus granulosa samples (n = 10) based on the SAM analysis of the RNA-seq data. O: Oocytes, C: Cumulus granulosa cells.

We then focused on lncRNA expression in MII oocyte and cumulus granulosa samples. We identified 8,257 differentially expressed lncRNAs with a fold change (FC) >2. Hierarchical clustering and principal component analysis (PCA) based on these differentially expressed lncRNAs separated perfectly the MII oocyte and somatic cell compartments of ovarian follicles (Fig. 2A,B). The classification of these differentially expressed lncRNAs in different subgroups according to their position relative to protein-coding genes revealed a greater proportion of intergenic lncRNAs (42% of all upregulated lncRNAs) in MII oocytes, and of antisense lncRNAs (39% of all upregulated lncRNAs) in cumulus granulosa cells (Fig. 2C). Noteworthy, differentially expressed lncRNAs were widely distributed along all human chromosomes, including the sex chromosome (X) (Fig. 2D). The “MII oocyte lncRNA signature” included 2,021 lncRNAs (Supplementary Table S3), among which *TUBB8P7* (FC = 71 259), *OOSP1* (FC = 43 555), *DPPA3P2* (FC = 24 554), *C3orf56* (FC = 19 086), *BCAR4* (FC = 14 488) and *OOEP-AS1* (FC = 1 764) were the most significantly upregulated. The “CC lncRNA signature” included 6,236 lncRNAs (Supplementary Table S4), among which 48% displayed a FC >10 (up to 18 883). *NEAT1* (FC = 18 883), *XIST* (FC = 9 253), *TSIX* (FC = 6 409), *VIM-AS1* (FC = 6 248), *MEG3* (FC = 5 217) and *H19* (FC = 4 333) were the most significantly upregulated lncRNAs in cumulus granulosa samples. The expression levels of the lncRNAs that best represented MII oocytes and cumulus granulosa samples are shown in Fig. 2E.

### Validation of differentially expressed lncRNAs by quantitative PCR in an independent cohort

**Figure 3:**
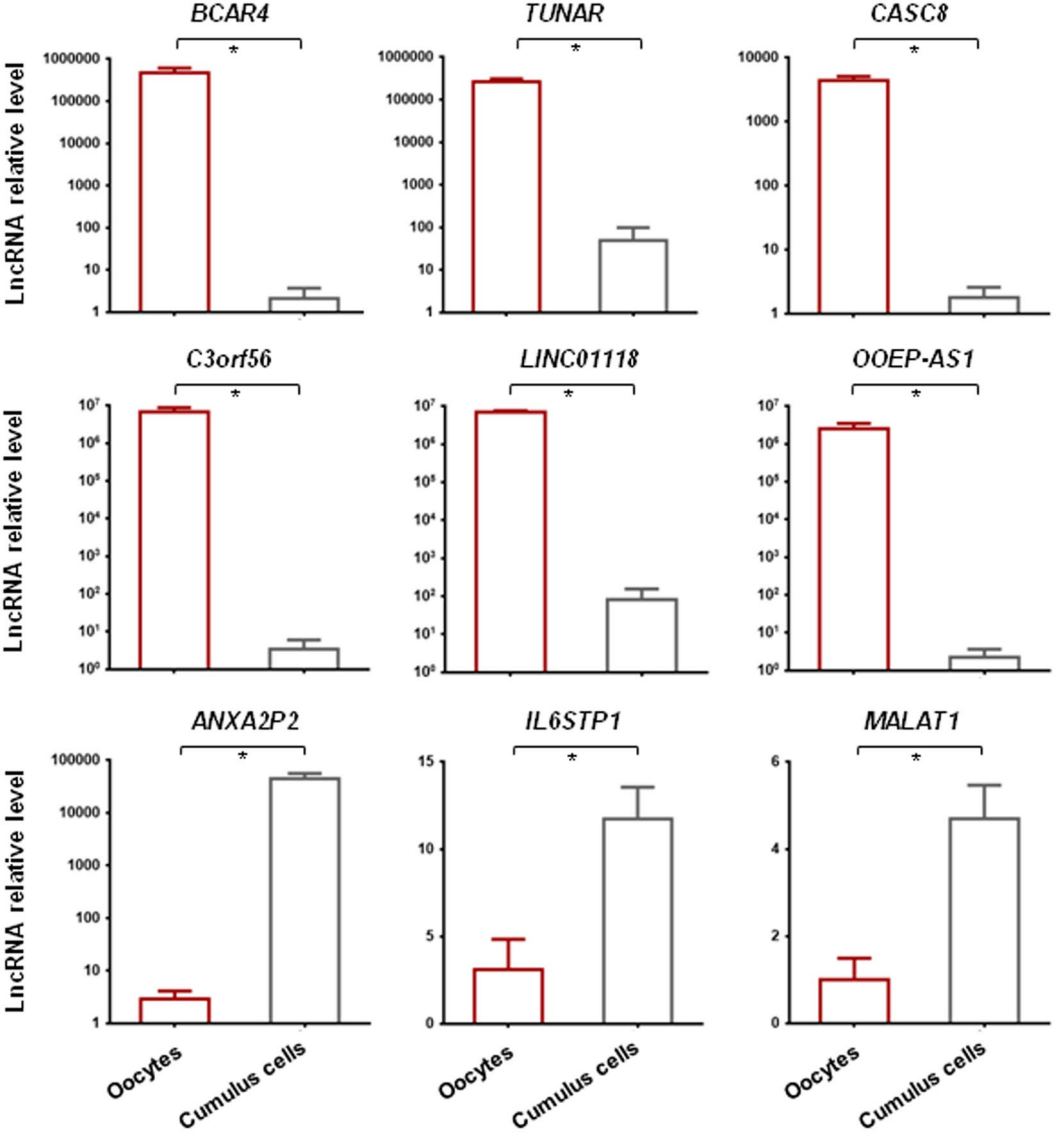
RT-qPCR validation of lncRNAs that are differentially expressed in human MII oocytes and cumulus cells. The results of RT–qPCR using three pools of oocytes and three pools of cumulus cells (CCs) validated the RNA-seq data. The lncRNA expression levels were normalized to endogenous *GAPDH*. The relative abundance of each lncRNA is shown on the *y* axis in arbitrary units. Results are presented as the mean ± SEM. *P value < 0.05 (Student-*t* test).

To validate the RNA-seq data, we analyzed the expression levels of ten lncRNAs using three pools of MII oocytes and three pools of cumulus cells (CC) samples by RT-PCR (Fig. 3). This analysis confirmed that some lncRNAs were specifically expressed in MII oocytes (*BCAR4*, *TUNAR*, *CASC8*, *C3orf56*, *LINC01118* and *OOEP-AS1*) or in CCs (*ANXA2P2*, *IL6STP1* and *MALAT1*). These results were consistent with the RNA-seq data.

### Regulatory roles of the identified lncRNAs

**Figure 4:**
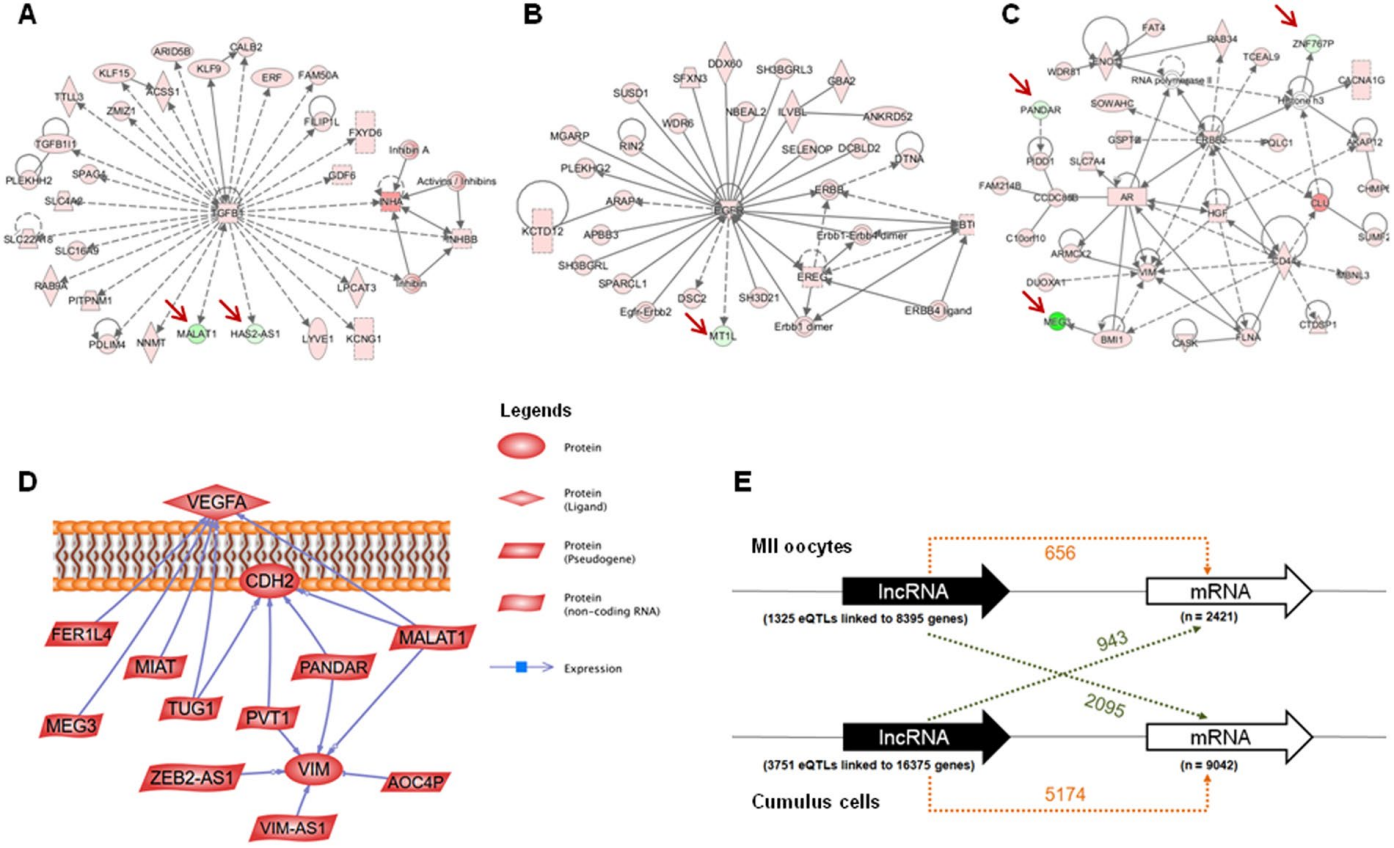
Co-expression of lncRNAs and mRNAs. (**A**–**C**) CC lncRNA–mRNA interaction networks were constructed using the IPA software. Nodes shaded in pink represent protein-coding genes that are upregulated in CCs, and green nodes are lncRNAs that are upregulated in CCs. The intensity of the node color indicates the degree of up-regulation. Edges (lines) and nodes are annotated with labels that illustrate the nature of the relationship between genes and their functions. A solid line represents a direct interaction and a dotted line an indirect interaction. (**D**) Pathway studio analysis of lncRNA–mRNA interactions using lncRNAs and mRNAs that are differentially expressed in CCs. Each node represents a gene entity or a control mechanism of the interaction. LncRNAs–*VEGFA* subnetwork: *VEGFA* was related to approximately five different lncRNAs. LncRNAs–VIM subnetwork: *VIM* was related to approximately six different lncRNAs. (**E**) Number of mRNAs linked to GTEx eQTL SNPs located in lncRNAs (cis-eQTL). The numbers of eQTL-linked mRNAs expressed in the same tissue (orange dotted arrow) or in a different tissue (green dotted arrow) are indicated. For instance, 943 of the 2,421 (39%) mRNAs expressed in MII oocytes are eQTL-linked to lncRNAs expressed in CCs, while 5,174 of the 9,042 (57%) mRNAs expressed in CCs are eQTL-linked to CC-expressed lncRNAs (Fisher’s exact test p-value < 2.2e-16).

To explore the putative functions of lncRNAs that are upregulated in MII oocytes or CCs, we identified networks that were enriched in transcripts overexpressed in the CC compartment. This analysis revealed that the lncRNAs *MALAT1* and *HAS2-AS1* were connected to *TGFB1* and that *MT1L* was associated with *EGFR*, suggesting an operative role of CC-lncRNAs in essential cell signaling pathways. In addition, the lncRNAs *MEG3* and *PANDAR* displayed a functional interaction with *BMI1* and *PIDD1*, forming a tightly connected network (Fig. 4A–C). Moreover, six CC-lncRNAs (*MALAT1*, *VIM-AS1*, *PANDAR*, *PVT1*, *ZEB2-AS1* and *AOC4P*) converged to *VIM* (vimentin), five (*MEG3*, *MALAT1*, *FER1L4*, *MIAT* and *TUG1*) to *VEGFA*, and four (*MALAT1*, *MEG3*, *PANDAR*, *PVT1* and *TUG1*) to *CDH2* (N-cadherin) (Fig. 4D). By contrast, only one MII oocyte-lncRNA (*LINC-ROR*) was highly connected to the network of the pluripotency marker *POU5F1*. Overall, these results show that CC-lncRNAs are highly linked to protein-coding genes involved in intercellular communication: growth factor receptors, growth factors, one adhesion molecule and one intermediate filament.

### Relationship between differentially expressed lncRNAs and COC-related protein-coding genes

Next, we asked whether lncRNAs could regulate COC-related protein-coding genes. We first looked at expression quantitative trait loci acting in *cis* (cis-eQTLS) present in lncRNAs and linked to mRNAs expressed in MII oocytes or CCs v6p GTEx release^29^. LncRNAs overexpressed in CCs contained 3,751 eQTLs linked to 16,375 genes among which 943 were overexpressed in MII oocytes (total number of protein-coding genes overexpressed in MII oocytes = 2,421) and 5,174 in CCs (total number of protein-coding genes overexpressed in CCs = 9,042) (Fig. 4E). This indicated that lncRNAs overexpressed in CCs preferentially contain cis-eQTLs linked to mRNAs expressed in CCs (943/2,421 genes *vs*. 5,174/9,042; Fisher’s exact test p-value < 2.2e-16). On the other hand, lncRNAs overexpressed in MII oocytes contained 1,325 cis-eQTLs linked to 8,395 genes, among which 656 were overexpressed in MII oocytes and 2,095 in CCs (Fig. 4E). Hence, as observed in CCs, lncRNAs overexpressed in MII oocytes preferentially contained cis-eQTLs linked to MII oocyte-overexpressed mRNAs (656/2,421 genes *vs*. 2095/9042; Fisher’s exact test p-value = 7.275e-05).

We then sought to functionally annotate lncRNAs using cis-eQTLs-associated mRNAs. We generated a list of the cis-eQTL-linked lncRNA-mRNA pairs (Supplementary Table S5) and annotated lncRNAs according to the associated mRNAs using the Gorilla tool^30^. We did not find any significant GO enrichment for CC-overexpressed lncRNAs compared with MII oocyte-overexpressed lncRNAs (Supplementary Table S6). Conversely, oocyte-overexpressed lncRNAs were associated with cell cycle process (false discovery rate, FDR, q-value = 3e-5), mitotic cell cycle process (FDR q-value = 1.32e-4) and reproductive process (FDR q-value = 1.37E-3) (Supplementary Table S7).

We then investigated the genomic organization of the lncRNAs and mRNAs that were overexpressed in MII oocyte and CC samples. The coordinates of 2,582 of the 6,199 lncRNAs overexpressed in CC samples (42%) intersected that of mRNAs overexpressed in CC samples. Conversely, only 259 CC-overexpressed lncRNAs (4%) overlapped MII oocyte-overexpressed protein-coding genes. Moreover, 2,137 of the 2,582 mRNA-overlapping lncRNAs (83%) were located on the opposite strand of the associated mRNA. On the other hand, the coordinates of 173 of the 2,421 MII oocyte-overexpressed lncRNAs (7%) intersected those of CC-overexpressed protein-coding genes, while 373 MII oocyte-overexpressed lncRNAs (15%) overlapped with MII oocyte-overexpressed mRNAs. As observed in CC samples, most of the mRNA-overlapping MII oocyte-overexpressed lncRNAs (311 out of 373; 83%) were located on the opposite strand of the associated mRNA. Together these results suggested the existence of mRNA regulations mediated by lncRNAs through sense/antisense pairing. Note that these regulations, which appear specific to MII oocytes or to CCs, may occur at the transcriptional and/or post-transcriptional levels. Dedicated experiments are necessary to validate this hypothesis.

### MII oocyte-specific lncRNA dynamics during early embryonic development

**Figure 5:**
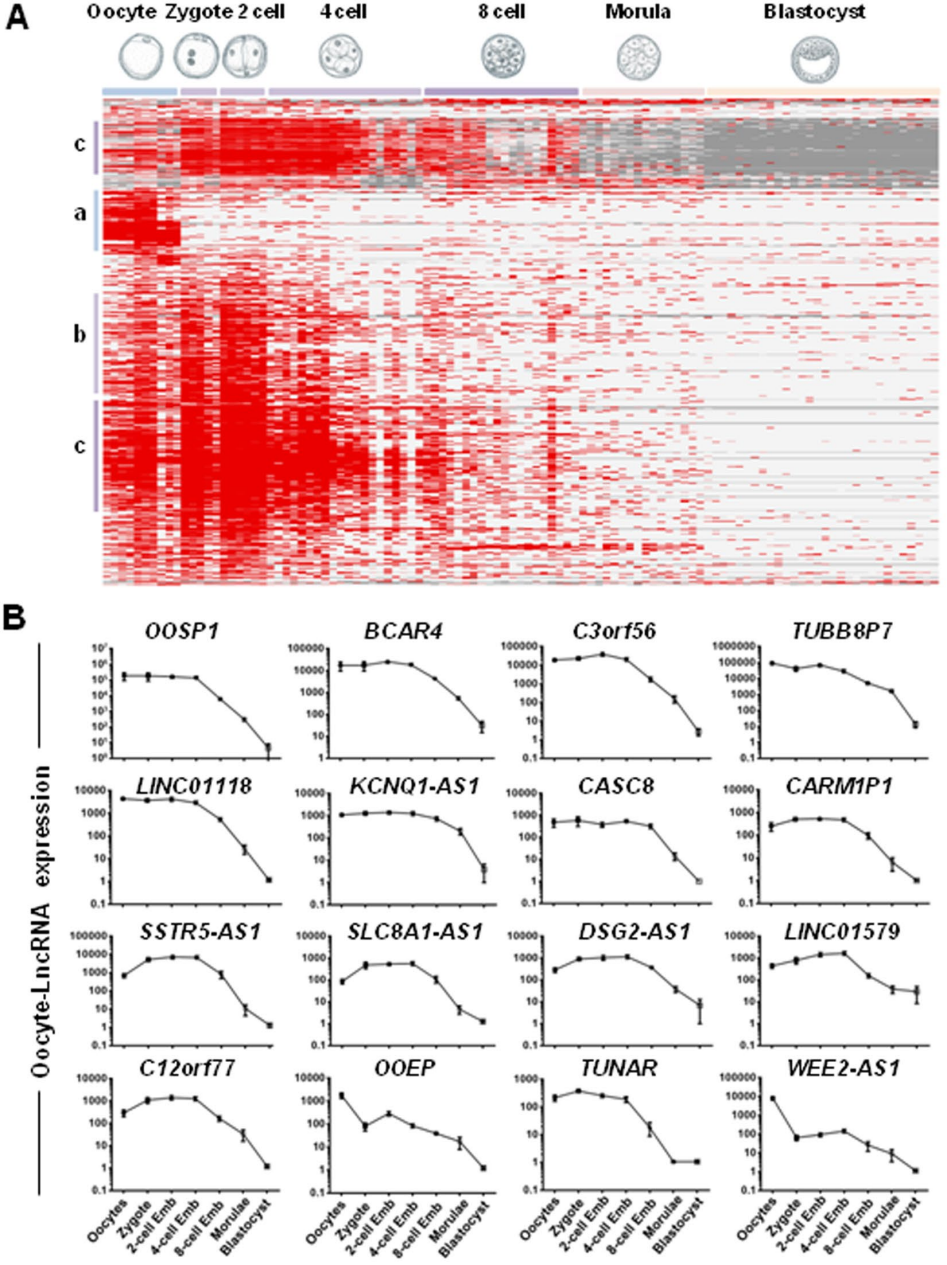
Dynamic changes in MII oocyte-lncRNAs expression during human early embryo development. (**A**) Heat map of the expression of the MII oocyte-lncRNAs in 97 embryonic samples (Table S8) analyzed by RNA-seq. Samples are arranged in columns and transcripts in rows. Clustering was carried out using the CLUSTER and TREEVIEW software; red and gray indicate expression above and below the mean, respectively, and white represents the mean expression. Average-link hierarchical clustering delineated three major clusters: (a) lncRNAs specific to MII oocytes, (b) lncRNAs specific to MII oocyte and early cleavage stages (zygote, 2-cell, 4-cell embryos), and (c) lncRNAs specific to oocyte, zygote, 2-cell, 4-cell and 8-cell embryos. (**B**). Expression profiles of selected lncRNAs in MII oocytes, zygote, 2-cell-, 4-cell-, 8-cell-stage embryos, morulae and blastocyst stages. The figure shows that the expression level of 16 MII oocyte-lncRNAs gradually decreases during human early embryonic development.

To investigate the expression kinetics of MII oocyte-specific lncRNAs during human early embryonic development, we analyzed additional RNA-seq data from human pre-implantation embryos (five zygotes, six 2-cell stage embryos, twenty 4-cell stage embryos, twenty 8-cell stage embryos, sixteen morulae and thirty late blastocysts) (see Materials and Methods and Supplementary Table S8). By analyzing the distribution of RNA-seq reads for the MII oocyte-specific lncRNAs during early embryonic development, we could define three major lncRNA groups (Fig. 5A): (a) lncRNAs specific to MII oocytes; (b) lncRNAs preferentially expressed in MII oocytes and early cleavage stages (zygote, 2-cell and 4-cell embryos), and (c) lncRNAs expressed in MII oocytes, zygotes, 2-cell, 4-cell and 8-cell embryos. The expression kinetics of MII oocyte-lncRNAs in the different samples (Fig. 5B) indicated that some MII oocyte-lncRNAs (*OOSP1*, *BCAR4*, *C3orf56*, *TUBB8P7*, *LINC01118*, *KCNQ1-AS1*, *CASC8*, *CARM1P1*, *SSTR5-AS1*, *SLC8A1-AS1*, *DSG2-AS1*, *LINC01579*, *C12orf77*, *OOEP*, *TUNAR* and *WEE2-AS1*) were degraded during progression to the blastocyst stage, specifically between the 4-cell and 8-cell stage.

### CC-lncRNA expression profile variations according to the women’s age

**Figure 6:**
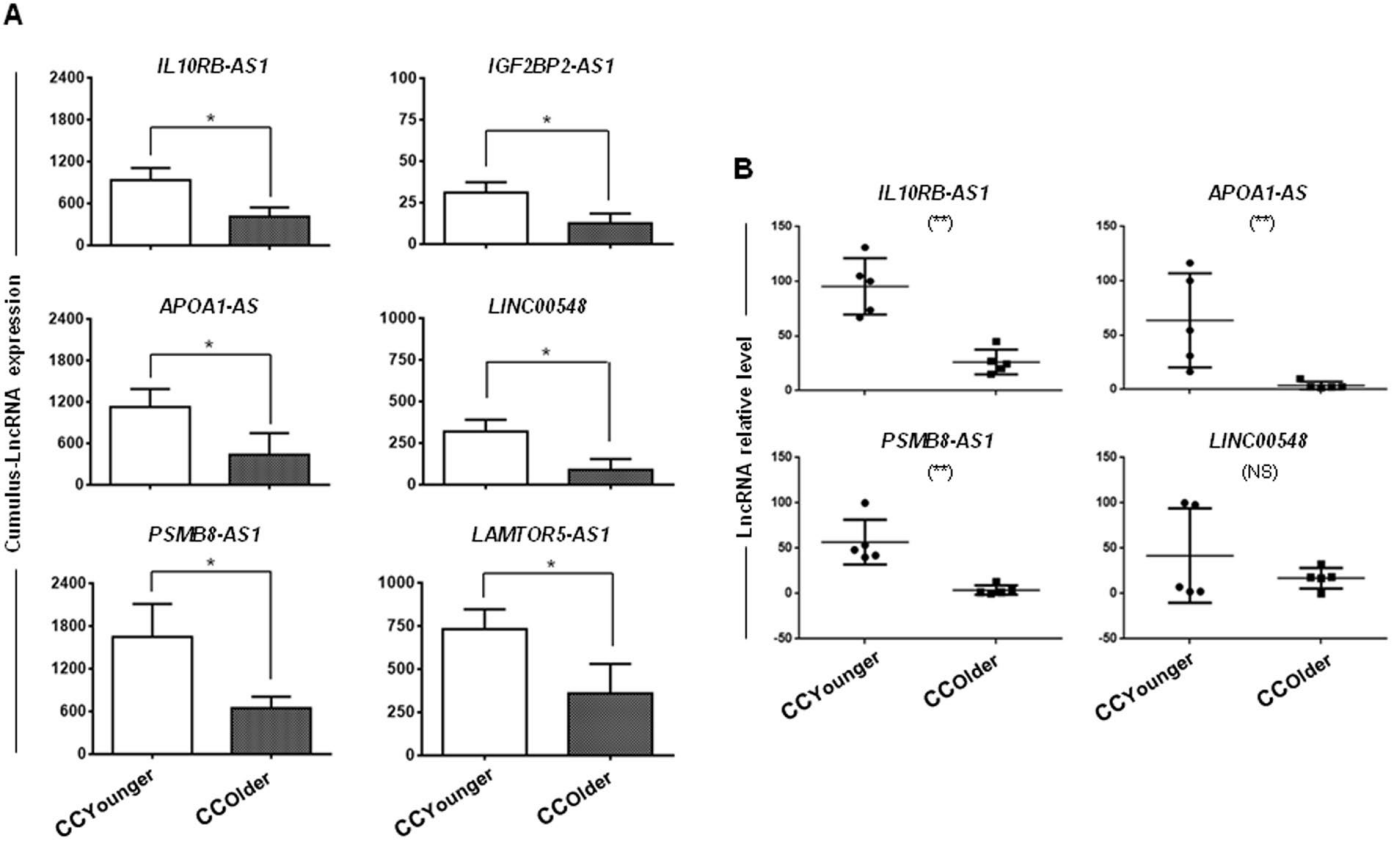
Expression profiles of CC-lncRNAs according to the donors’ age. (**A**) Histograms show the expression level of six lncRNAs (*IL10RB-AS1*, *APOA1-AS*, *IGF2BP2-AS1*, *LINC00548*, *PSMB8-AS1* and *LAMTOR5-AS1*) in CC samples from young (<35 years) and older patients (>40 years of age) (n = 9 sample/group). The number of reads is shown on the *y*-axis as arbitrary units determined by the RNA-seq analysis. *Significant difference with FDR ≤0.05 according to SAM. (**B**) Validation by qRT-PCR analysis of the expression profile of some lncRNAs that are differentially expressed in older and younger patients (n = 5 samples/group). The signal intensity (arbitrary units) for each lncRNA is shown on the Y-axis; **Significant difference with *p*-value = 0.0079. NS: not significant.

To evaluate the influence of women’s age on CC-lncRNA expression, we compared the transcriptome profile of CCs from younger (CCyounger, <35 years of age; n = 9 samples) and older (CColder, >40 years of age; n = 9 samples) women (see Material and Methods). We identified 147 lncRNAs (FDR <5% and FC ≥2) that were significantly downregulated in CColder (comprehensive list in Supplementary Table S9), suggesting that the women’s age influences the lncRNA expression profile in CCs. Some lncRNAs that were annotated as playing a role in the CC-oocyte dialogue and that may be important for oocyte quality showed lower expression levels in CColder than in CCyounger samples, particularly *IL10RB-AS1*, *APOA1-AS*, *IGF2BP2-AS1*, *LINC00548*, *PSMB8-AS1* and *LAMTOR5-AS1* (Fig. 6A). The IPA analysis of the molecular and cellular functions of these age-related lncRNAs showed that they were significantly (*P* < 0.05) involved in lipid metabolism, small molecule biochemistry, molecular transport, carbohydrate metabolism and cell cycle (Supplementary Figure S2). Finally, using qRT-PCR approach, we assessed the expression pattern of four lncRNAs (*IL10RB-AS1*, *APOA1-AS*, *PSMB8-AS1* and *LINC00548*) in independent samples [five CC samples from younger patients (age <35 years) and five CC samples from older patients (age >40 years)]. The obtained results were in agreement with the sequencing data (Fig. 6B).

## Discussion

Human oocytes and cumulus cells have been widely studied in the last decades, but their detailed molecular features remain incomplete. Our previous efforts were focused on profiling mRNA and microRNA expression in COC samples^28,31–34^. Here, we determined the lncRNA expression profiles of human MII oocytes and CCs. First, we show by RNA-seq data analysis that these two cell types have very different lncRNA profiles. Particularly, more lncRNAs are overexpressed in CC samples than in MII oocytes. This is in line with our previous observation of a lower number of other non-coding RNAs, such as microRNAs, in germinal (MII oocytes) than somatic (CCs) cells^33^. In addition, the expression level of most lncRNAs is substantially lower than that of protein-coding genes in both MII oocyte and CC samples, in agreement with previous studies^35^. Moreover, the expression profile of protein-coding genes, such as *H1FOO*, *DPPA3*, *DAZL*, *ZP2*, *FIGLA*, *MOS* and *GDF9*, is in agreement with the results of previous microarray-based studies in humans and other species, thus perfectly validating our RNA-seq analysis.

The “MII oocyte lncRNA signature” included the lncRNAs *BCAR4*, *PCAT1*, *WEE2-AS1* and *TUNAR* that are involved in cancer, cell cycle and pluripotency. *BCAR4* is a lncRNA involved in cancer progression^36–39^ and is expressed in bovine and human oocytes^40,41^. *PCAT1* promotes cell proliferation in different cancers^42–44^, but its role in germinal cells is unknown. *WEE2* plays a crucial role in maintaining meiosis arrest in rhesus macaque^45^ and mouse oocytes^46^. *TUNAR* is required for the maintenance of the pluripotency and self-renewal features of mouse embryonic stem cells (ESCs)^47^. As ESCs and oocytes share a common transcriptome signature^48^ and can reprogram somatic cells into pluripotent cells^49,50^, the identification of common key lncRNAs (for instance *TUNAR*) could contribute to understanding the mechanisms underlying their reprogramming properties. The “MII oocyte lncRNA signature” included also the *TUBB8* (tubulin, beta 8 class VIII) pseudogene that might play an important role in oocyte maturation, fertilization and early embryonic development^51^. Recently, Feng and colleagues found that *TUBB8* mutations lead to human oocyte maturation arrest and cause multiple phenotypes in human oocytes and early embryos^52,53^. Nevertheless, the functions of many lncRNAs of the MII oocyte lncRNA signature, such as *LINC01118*, *LINC01467*, *LINC01579*, *LINC01498*, *LINC00943*, *LINC00371* and *LINC01483*, remain largely unknown.

Moreover, we found that some MII oocyte-lncRNAs have a dynamic expression that is stable during the first early cleavage stages, but then strongly declines between the 4-cell and 8-cell stage, at the time of the maternal genome degradation and embryonic genome activation (EGA). This suggests that these lncRNAs could play a role during the maternal to embryonic genome transition and in EGA^54–57^. Based on their proliferative effect in cancer and stem cells, we hypothesize that the MII oocyte-lncRNAs *BCAR4*, *WEE2-AS1* and *TUNAR* could be involved in cell division, until the human embryo can transcribe its own factors. Future studies should be oriented toward investigating whether altered expression of these lncRNAs affect human early embryo development.

Human CC samples were enriched in lncRNAs (*NEAT1*, *MALAT1* and *MEG3*) that have been already detected in various human malignancies, including ovarian carcinoma^58–60^. Genetic ablation of *Neat1* in mice^61^ showed that this lncRNA is essential for corpus luteum formation and pregnancy establishment in suboptimal conditions. Previous studies have described complementary binding and synergistic roles between *NEAT1* and *MALAT1*^62,63^. *MEG3* is highly expressed in normal ovarian tissues^60^ and targets *p53* indirectly by suppressing *MDM2* in ovarian granulosa cells^64^. Additional studies on the effects of *NEAT1, MALAT1* and *MEG3* aberrant expression in CCs should bring critical insights into their function during oocyte maturation and in ovarian carcinoma. Although the function of most of the identified lncRNAs has not been determined yet, it could be inferred by annotation of the protein-coding genes that interact or are co-expressed with them. Our analysis showed that *MEG3*, *MALAT1*, *TUG1* and *MIAT* lncRNAs interact with *VEGFA* in a network pathway. *VEGF* is a potent angiogenic factor that promotes vascularization^65^ and a critical regulator of angiogenesis during ovarian follicular growth^66^. Several studies examined VEGF concentration in follicular fluid in relation with the ovarian response to gonadotropin stimulation^67^, follicle development^68^, oocyte maturity and fertilization^69^, embryo quality^70^ and pregnancy outcome^71^. They found that dysregulation of the *VEGF* pathway may negatively affect oocyte competence and embryo quality. We also found that the lncRNAs *MALAT1*, *PANDAR* and *PVT1* are associated with *VIM* (vimentin) and *CDH2* (N-cadherin) in networks interconnected with the *VEGFA* network. Vimentin and N-cadherin are key proteins involved in cell-cell adhesion and cytoskeleton regulation in granulosa cells^72,73^. *Vim* ablation leads to defective steroid hormone production in mouse ovaries^74^. In the developing ovary, N-cadherin might interact with basic fibroblast growth factor (*bFGF*) to promote granulosa cell survival and ovarian cell viability^75^. Therefore, we can hypothesize that the lncRNAs found in these networks could contribute to the regulation of the cytoskeletal architecture and cell-cell adhesion in early developing human follicles. Considering the critical role of *VEGF*, N-cadherin and vimentin in the ovulatory follicle, these putative regulator lncRNAs could be considered as candidate biomarkers of IVF success.

Previous reports have shown that maternal age could influence the mRNA, protein and microRNA expression patterns in human CC samples^76–78^. Specifically, age affects the expression of genes involved in many functional processes, such as angiogenesis, fatty acid metabolism, inflammation, DNA methylation profile, energy production and hypoxia stress responses^76,77,79,80^. Here, we found marked differences in lncRNA expression according to the donors’ age. Some lncRNAs involved in processes that govern oocyte maturation and follicle development are under-expressed in CC samples from women older than 40 years of age. Specifically, lncRNAs with age-related reduced expression were mostly associated with angiogenesis, lipid transport, cell cycle regulation and transcriptional control (*APOA1-AS*, *IGF2BP2-AS1* and *PSMB8-AS1*). The lncRNA apolipoprotein A1-antisense (*APOA1-AS*) acts as a transcriptional regulator of *APOA1*^81^ that is the major component in high-density lipoprotein (HDL) particles and plays a key role in cholesterol trafficking^82–84^. HDL in follicular fluid could play a role in follicle development by stimulating angiogenesis^85^ and *APOA1* could affect oocyte maturation and fertility potential^86^. Furthermore, variations in the follicular apolipoprotein content have been associated with age-related fertility decline^86^. *APOA1-AS* decrease in CCs with age could also be involved in the regulation of angiogenesis in aging follicles. *IGF2BP2-AS1* is another antisense lncRNA that is under-expressed in CC samples from older women. *IGF2BP2-AS1*, which is located in the *IGF2BP2* antisense strand^87^, modulates *IGF* signaling and has anti-angiogenic activity^87^. The lncRNA *PSMB8-AS1* (proteasome activator subunit 1 *PSMB8* antisense RNA 1) also is downregulated in CC samples from older women. The proteasome contributes to the maintenance of cell homeostasis and is a regulator of essential processes, such as transcription, cell division, and signaling^88^. A failure of the “proteasome machinery” might have detrimental effects on CC physiology and consequently on fertility, especially in older women. In conclusion, the identification of CC lncRNAs the expression of which is affected by the women’s age represent a valuable resource for future investigations on the biology of aging oocytes and could become candidate biomarkers to evaluate IVF feasibility in aged couples.

## Conclusion

This work is the first comprehensive description of lncRNA expression profile in COC. By identifying lncRNAs that are differentially expressed in mature MII oocytes and CCs or the expression of which decreases with age, these data will be a valuable resource for future studies. These lncRNAs could be involved in the many processes that regulate folliculogenesis. Future investigations are needed to elucidate their physiological roles and their potential as diagnostic/research biomarkers.

## Methods

### RNA-seq data selection and analysis

Publicly available RNA-seq data were downloaded from the European Nucleotide Archive (ENA) database. We included 135 human samples: 10 MII oocytes, 10 cumulus granulosa samples and 97 pre-implantation embryos. In addition, we used 9 cumulus cells (CC) samples from younger patients and 9 CC from older patients. The accession numbers and detailed information on the RNA-seq data are listed in Supplementary Table S8.

Data were processed using the open Galaxy platform GalaxEast (http://galaxeast.fr). Raw RNA-seq reads were mapped to the human reference genome (GRCh37/hg19) using TopHat2^89^ and reads were counted with HTSeq^90^ using the galaxeast default parameters, as reported^91^. We used Ensembl annotations (http://www.ensembl.org/index.html) for protein-coding genes and long non-coding transcripts that included lncRNAs, long intergenic non-coding RNAs (lincRNAs), sense and antisense overlapping transcripts and pseudogenes. Small non-coding RNAs, such as microRNAs, and ribosomal RNAs, which are not classified as lncRNAs, were removed from the analysis. Transcripts with a read detection in less than 50% of samples for each sample category were also removed. Dendrograms and heat maps were produced by using the Cluster and Treeview software packages^92^. The average linkage method and the branching pattern in which the similarity between the lncRNA expression profiles could be visually assessed were used. Significant Analysis of Microarrays (SAM) (http://statweb.stanford.edu/~tibs/SAM/) with the Wilcoxon test and sample label permutation (*n* = 300) was performed to identify differentially expressed genes (lncRNAs and protein- coding mRNAs) in MII oocyte and cumulus granulosa samples. Genes were considered as differentially expressed at FDR < 0.05 and with an absolute FC > 2. R studio (https://www.rstudio.com/) was used to run custom R scripts for the principal component analysis (PCA). Scatter plots were generated using Excel, based on the differentially expressed gene analysis and the color was determined according to the filtering criteria.

### Gene ontology (GO) and network analysis

LncRNA and mRNA functions were assessed with Gene ontology (GO) and the PANTHER tool (http://pantherdb.org) and the Ingenuity Pathway Analysis (IPA) platform (http://www.ingenuity.com). LncRNA and mRNA data were integrated with molecular interactions using IPA. LncRNAs and mRNAs (with known gene symbols) and their corresponding expression values were uploaded in the IPA software. Each gene symbol was mapped to the corresponding gene object in the Ingenuity Pathways Knowledge Base. Gene networks were algorithmically generated based on their connectivity and assigned a score. The score is a numerical value used to rank networks according to how relevant they are to the genes in the input dataset, but may not be an indication of the network quality or significance. The score takes into account the number of focus genes in the network and the network size to approximate how relevant the network is to the original list of focus genes.

### Prediction of binding sites on lncRNA targets

The Single Tissue cis-eQTL data were downloaded from the GTEx website (v6p GTEx release, GTEx_Analysis_v6p_eQTL.tar)^29^ using the UCSC liftover tool. Then, eQTL SNPs were located in lncRNAs and mRNAs using BedTools^93^. Fisher’s exact tests were performed using R.

### Oocyte and CC samples for RT-PCR

For data validation by RT-PCR, human oocyte and CC samples were collected from women (without endometriosis and polycystic ovary syndrome) who underwent conventional IVF or intracytoplasmic sperm injection (ICSI) at Laboratoire de Biologie de la Reproduction, François Mitterrand Hospital, Dijon, France. All patients signed an informed consent form. Moreover, the material used in the present study would have been discarded. This study was approved by the Ethical Committee of DIJON (authorization number #DC-2011-1332) and the methods were carried out in accordance with the approved guidelines.

Oocytes were retrieved transvaginally 36 h after human chorionic gonadotropin (hCG) injection under ultrasound guidance. Immature and unfertilized MII oocytes were collected 24 h post-insemination and CCs were mechanically removed from MII oocytes before ICSI, as previously described^28,33^. Oocytes or CC samples were pooled and immediately frozen at −80 °C in RLT buffer (ref: 74004; Qiagen) before RNA extraction. Three pools of 4 oocytes and three pools of 3 CCs (from six patients, age <37 years) were used for lncRNA RT–qPCR validation. We also did qRT-PCR experiments using an independent cohort of patients [5 CC samples from younger patients (age <35 years) and 5 CC samples from older patients (age >40 years)] to assess the expression pattern of age-related lncRNAs.

### Quantitative PCR validation of lncRNAs

Total RNA was extracted from pools of MII oocytes or CCs using the RNeasy Micro Kit (Qiagen), according to the manufacturer’s instructions. Reverse transcription (RT) was performed in a 20 μL reaction volume that included the SuperScript^®^ First-Strand Synthesis System (ref. 18064-014, Invitrogen), oligo-dT primer, dNTP mixture, MgCl_2_ and RNase inhibitor, according to the manufacturer’s instructions. Quantitative PCR was performed in 384-well plates (Sorenson BioScience, Inc.) on a Lightcycler^®^ 480 Real-Time PCR System using the SYBR Green I Master Kit (Roche Diagnostics, Mannheim, Germany). Each sample was run in triplicate. The cycling conditions included an initial cycle (95 °C for 3 min) that was followed by 40 cycles (95 °C for 15 s; 60 °C for 15 s; 72 °C for 20 s). The primers used for lncRNA expression validation are listed in Supplementary Table S10. LncRNA expression levels were normalized to that of the housekeeping gene *GAPDH*. The relative lncRNA expression level was calculated with the 2−ΔΔCt method.

### Statistical analysis

All values are presented as the mean ± SEM. Statistical differences between groups were analyzed by one-way ANOVA and the Student’s *t*-test using GraphPad Prism 6. A value of p < 0.05 was considered significant.

### Data availability

All data generated or analyzed during this study are included in this published article and its Supplementary Information files.

## Electronic supplementary material

Supplementary Information

Supplementary Information

Dataset 1

Dataset 2

Dataset 3

Dataset 4

Dataset 5

Dataset 6

Dataset 7

Dataset 8

Dataset 9

Dataset 10
