# Supplementary material for: Differential long non-coding RNA expression profiles in human oocytes and cumulus cells
Source: Sci Rep. 2018 Feb 2;8:2202. doi: 10.1038/s41598-018-20727-0 (PMC5797088; doi:10.1038/s41598-018-20727-0)
Supplement: Supplementary file 1 — Supplementary Information [file 41598_2018_20727_MOESM1_ESM.pdf]

Differential long non-coding RNA expression profiles in human oocytes and cumulus cells

Julien Bouckenheimer, Patricia Fauque, Charles-Henri Lecellier, Céline Bruno, Thérèse Commes, Jean-Marc Lemaître, John De Vos, Said Assou

Supplementary Figure S1: Expression of known markers of MII oocytes and cumulus granulosa cells. Box-and-whisker plots showing the expression level of MII oocyte markers (*NLRP4*, *WEE2*, *AURKC*, *OOEP*, *DPPA5*, *DNMT1*, *ZP4* and SOX30) and cumulus cell markers (*SCD*, *StAR*, *HSPG2*, *GJA1*, *LHCGR*, *INHBA*, *ADAMTS1* and *IL1R1*) in MII oocyte (n=10) and cumulus granulosa samples (n=10), based on the SAM analysis of RNA-seq expression data. The resulting data demonstrated the high and specific expression pattern of these markers. O: Oocytes, C: Cumulus granulosa cells.

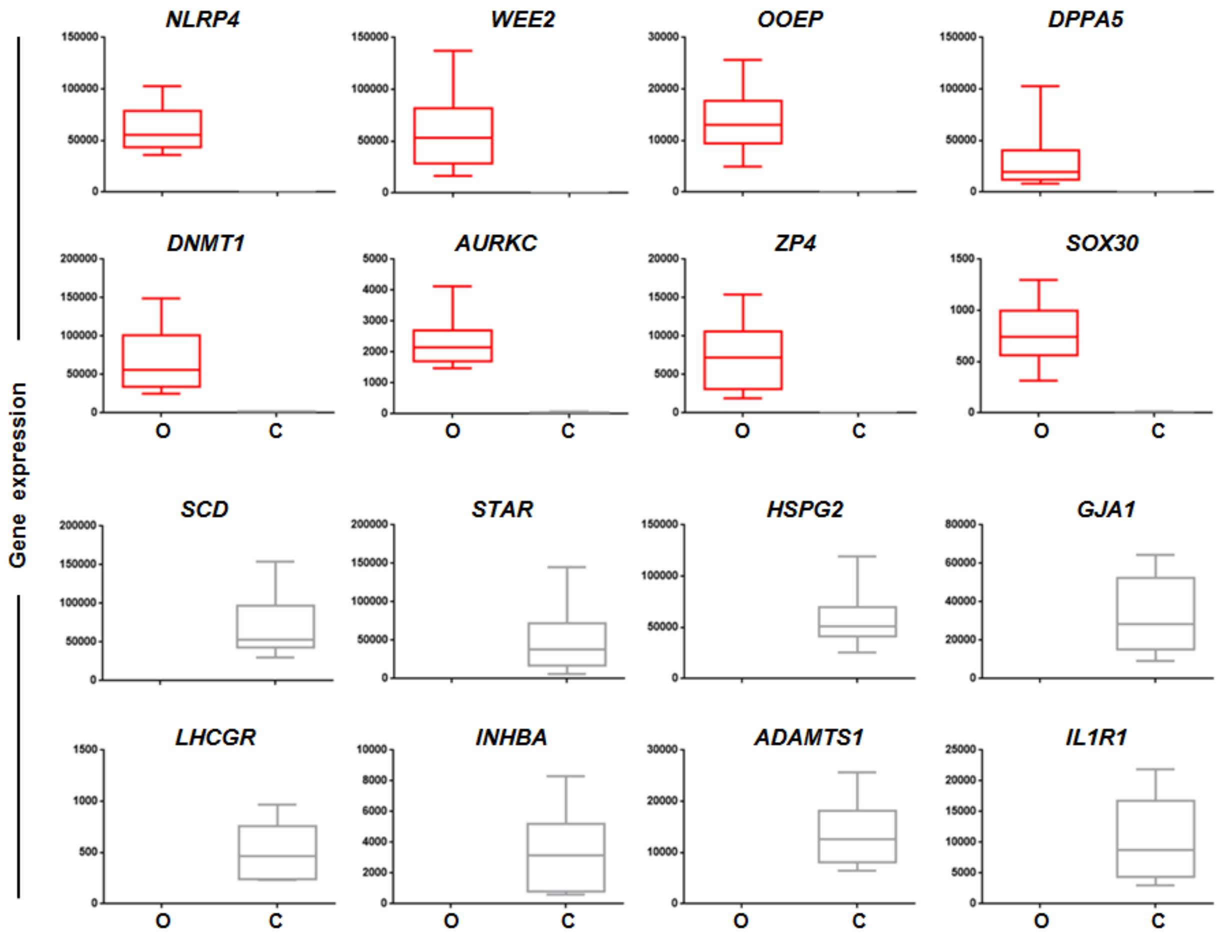

Supplementary Figure S2: Ingenuity Pathway Analysis (IPA) showing the molecular and cellular functions of age-related lncRNAs.

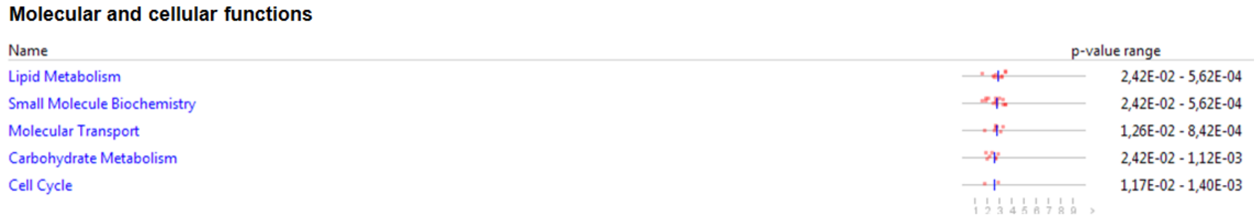

Molecular and cellular functions

Name	p-value range
Lipid Metabolism	2,42E-02 - 5,62E-04
Small Molecule Biochemistry	2,42E-02 - 5,62E-04
Molecular Transport	1,26E-02 - 8,42E-04
Carbohydrate Metabolism	2,42E-02 - 1,12E-03
Cell Cycle	1,17E-02 - 1,40E-03
