# Supplementary material for: Differential long non-coding RNA expression profiles in human oocytes and cumulus cells
Source: Sci Rep. 2018 Feb 2;8:2202. doi: 10.1038/s41598-018-20727-0 (PMC5797088; doi:10.1038/s41598-018-20727-0)
Supplement: Supplementary file 2 — Supplementary Information [file 41598_2018_20727_MOESM2_ESM.pdf]

Differential long non-coding RNA expression profiles in human oocytes and cumulus cells

Julien Bouckenheimer, Patricia Fauque, Charles-Henri Lecellier, Céline Bruno, Thérèse Commes, Jean-Marc Lemaître, John De Vos, Said Assou

Supplementary Table S1: List of the 2,421 protein-coding genes upregulated in MII oocytes

Gene ID	Gene Name	Chromoso	Start_position	End_position	Gene_biotype	Fold Change	q-value(%)
ENSG00000178804	H1FOO	3	129543214	129551467	protein_coding	74217,18	0,0000000
ENSG00000187569	DPPA3	12	7711454	7717559	protein_coding	58356,00	0,0000000
ENSG00000160505	NLRP4	19	55836578	55881854	protein_coding	56065,82	0,0000000
ENSG00000261456	TUBB8	10	46892	74163	protein_coding	33504,36	0,0000000
ENSG00000185467	KPNA7	7	99173574	99207506	protein_coding	33463,61	0,0000000
ENSG00000100721	TCL1A	14	95709967	95714196	protein_coding	32215,40	0,0000000
ENSG00000203909	DPPA5	6	73353062	73354295	protein_coding	30731,00	0,0000000
ENSG00000173572	NLRP13	19	55891699	55932336	protein_coding	23823,10	0,0000000
ENSG00000214102	WEE2	7	141708353	141731271	protein_coding	21431,22	0,0000000
ENSG00000149507	OOSP2	11	60040275	60048044	protein_coding	16859,40	0,0000000
ENSG00000189167	ZAR1L	13	32303700	32315344	protein_coding	11219,60	0,0000000
ENSG00000117148	ACTL8	1	17755313	17827063	protein_coding	8839,67	0,0000000
ENSG00000103310	ZP2	16	21197450	21214510	protein_coding	8571,30	0,0000000
ENSG00000203907	OOEP	6	73368555	73395133	protein_coding	7564,72	0,0000000
ENSG00000116996	ZP4	1	237877864	237890922	protein_coding	7444,40	0,0000000
ENSG00000187690	CXorf67	X	51406915	51408843	protein_coding	6624,55	0,0000000
ENSG00000223601	EBLN1	10	22208814	22210021	protein_coding	6453,20	0,0000000
ENSG00000137875	BCL2L10	15	52109263	52112775	protein_coding	6383,54	0,0000000
ENSG00000203908	KHDC3L	6	73362677	73364171	protein_coding	5558,95	0,0000000
ENSG00000165588	OTX2	14	56799905	56810479	protein_coding	4931,20	0,0000000
ENSG00000248485	PCP4L1	1	161258727	161285450	protein_coding	4165,22	0,0000000
ENSG00000179873	NLRP11	19	55785397	55836800	protein_coding	3383,97	0,0000000
ENSG00000137707	BTG4	11	111467526	111512354	protein_coding	3346,52	0,0000000
ENSG00000180438	TPRXL	3	13937256	14082811	protein_coding	3071,22	0,0000000
ENSG00000205022	PABPN1L	16	88863333	88866660	protein_coding	2886,10	0,0000000
ENSG00000137766	UNC13C	15	54012904	54633414	protein_coding	2813,75	0,0000000
ENSG00000141738	GRB7	17	39737927	39747291	protein_coding	2718,28	0,0000000
ENSG00000102290	PCDH11X	X	91779261	92623230	protein_coding	2392,86	0,0000000
ENSG00000172680	MOS	8	56112942	56113982	protein_coding	2392,40	0,0000000
ENSG00000104413	ESRP1	8	94641074	94707466	protein_coding	2206,58	0,0000000
ENSG00000114455	HHLA2	3	108296490	108378285	protein_coding	2060,80	0,0000000
ENSG00000152670	DDX4	5	55738017	55817157	protein_coding	1971,90	0,0000000
ENSG00000092345	DAZL	3	16586792	16670306	protein_coding	1906,00	0,0000000
ENSG00000124610	HIST1H1A	6	26017085	26017732	protein_coding	1866,80	0,0006696
ENSG00000185155	MIXL1	1	226223618	226227054	protein_coding	1847,59	0,0000000
ENSG00000149506	ZP1	11	60867562	60875693	protein_coding	1815,09	0,0000000
ENSG00000143194	MAEL	1	166975582	167022214	protein_coding	1812,71	0,0000000
ENSG00000214414	TRIM77	11	89710299	89717872	protein_coding	1795,50	0,0000000
ENSG00000276747	PADI6	1	17372196	17401699	protein_coding	1614,00	0,0000000
ENSG00000167634	NLRP7	19	54923509	54966312	protein_coding	1592,13	0,0000000
ENSG00000143006	DMRTB1	1	53459399	53467488	protein_coding	1498,10	0,0000000
ENSG00000221818	EBF2	8	25841730	26045397	protein_coding	1300,27	0,0000000
ENSG00000205126	ACCSL	11	44047981	44059977	protein_coding	1249,74	0,0000000
ENSG00000120051	CFAP58	10	104353764	104455090	protein_coding	1223,48	0,0000000
ENSG00000165970	SLC6A5	11	20599400	20659285	protein_coding	1088,65	0,0000000
ENSG00000127743	IL17B	5	149371324	149404202	protein_coding	1071,92	0,0000000
ENSG00000104953	TLE6	19	2977446	2995184	protein_coding	1055,38	0,0000000
ENSG00000008196	TFAP2B	6	50818723	50847613	protein_coding	1035,20	0,0006696
ENSG00000159166	LAD1	1	201373244	201399915	protein_coding	1017,90	0,0000000
ENSG00000112333	NR2E1	6	108166058	108188809	protein_coding	1012,75	0,0000000
ENSG00000137434	C6orf52	6	10671418	10694797	protein_coding	997,85	0,0000000
ENSG00000162949	CAPN13	2	30722771	30820542	protein_coding	993,55	0,0000000

ENSG00000161905	ALOX15	17	4630902	4642294	protein_coding	947,32	0,0000000
ENSG00000154102	C16orf74	16	85690084	85751129	protein_coding	929,26	0,0000000
ENSG00000178796	RIIAD1	1	151710433	151729805	protein_coding	925,33	0,0000000
ENSG00000121690	DEPDC7	11	33015864	33033582	protein_coding	887,73	0,0000000
ENSG00000146038	DCDC2	6	24171756	24358052	protein_coding	882,93	0,0000000
ENSG00000164611	PTTG1	5	160421822	160428744	protein_coding	867,31	0,0000000
ENSG00000137948	BRDT	1	91949371	92014426	protein_coding	793,70	0,0000000
ENSG00000087495	PHACTR3	20	59577509	59847711	protein_coding	750,58	0,0000000
ENSG00000147655	RSPO2	8	107899316	108083648	protein_coding	744,21	0,0000000
ENSG00000112494	UNC93A	6	167271169	167316019	protein_coding	743,30	0,0000000
ENSG00000184571	PIWIL3	22	24719034	24774720	protein_coding	732,10	0,0000000
ENSG00000104321	TRPA1	8	72019917	72075617	protein_coding	728,50	0,0000000
ENSG00000185792	NLRP9	19	55708432	55738402	protein_coding	726,93	0,0000000
ENSG00000148702	HABP2	10	113550837	113589602	protein_coding	725,17	0,0000000
ENSG00000176979	TRIM60	4	165031953	165041744	protein_coding	709,30	0,0000000
ENSG00000154319	FAM167A	8	11421463	11474715	protein_coding	674,64	0,0000000
ENSG00000183185	GABRR3	3	97986673	98035304	protein_coding	667,30	0,0000000
ENSG00000124092	CTCFL	20	57495966	57525652	protein_coding	666,44	0,0000000
ENSG00000176204	LRRTM4	2	76747719	77593319	protein_coding	663,40	0,0000000
ENSG00000253313	C1orf210	1	43281883	43285617	protein_coding	636,82	0,0000000
ENSG00000163380	LMOD3	3	69106872	69123032	protein_coding	627,91	0,0000000
ENSG00000164113	ADAD1	4	122378966	122429802	protein_coding	626,92	0,0000000
ENSG00000188372	ZP3	7	76397518	76442071	protein_coding	613,16	0,0000000
ENSG00000183324	REC114	15	73443158	73560014	protein_coding	605,33	0,0000000
ENSG00000144583	04-mars	2	216257865	216372027	protein_coding	575,41	0,0000000
ENSG00000255298	OR8G5	11	124264827	124265867	protein_coding	573,90	0,0000000
ENSG00000157927	RADIL	7	4797055	4883719	protein_coding	571,96	0,0000000
ENSG00000120669	SOHLH2	13	36168208	36214615	protein_coding	558,21	0,0000000
ENSG00000158486	DNAH3	16	20933111	21159441	protein_coding	556,13	0,0000000
ENSG00000204710	SPDYC	11	65170154	65173244	protein_coding	545,40	0,0000000
ENSG00000176302	FOXR1	11	118971707	118981291	protein_coding	534,47	0,0000000
ENSG00000117322	CR2	1	207454230	207489895	protein_coding	534,00	0,0000000
ENSG00000171487	NLRP5	19	55999726	56061813	protein_coding	524,71	0,0000000
ENSG00000130385	BMP15	X	50910784	50916607	protein_coding	516,50	0,0006696
ENSG00000095627	TDRD1	10	114179270	114232304	protein_coding	495,42	0,0000000
ENSG00000016082	ISL1	5	51383391	51394738	protein_coding	484,09	0,0000000
ENSG00000163377	FAM19A4	3	68731766	68953297	protein_coding	479,70	0,0000000
ENSG00000166840	GLYATL1	11	58905398	59043527	protein_coding	472,91	0,0000000
ENSG00000089225	TBX5	12	114353931	114408442	protein_coding	469,50	0,0000000
ENSG00000075340	ADD2	2	70607618	70768225	protein_coding	464,06	0,0000000
ENSG00000198054	DSCR8	21	38121451	38188016	protein_coding	463,10	0,0000000
ENSG00000140538	NTRK3	15	87859751	88256768	protein_coding	456,71	0,0000000
ENSG00000079102	RUNX1T1	8	91954967	92103286	protein_coding	456,41	0,0000000
ENSG00000077800	FKBP6	7	73328164	73358637	protein_coding	456,40	0,0000000
ENSG00000103528	SYT17	16	19167971	19268334	protein_coding	442,58	0,0012807
ENSG00000179709	NLRP8	19	55947832	55988629	protein_coding	442,10	0,0000000
ENSG00000164265	SCGB3A2	5	147870682	147882191	protein_coding	436,30	0,0000000
ENSG00000155875	SAXO1	9	18927658	19049354	protein_coding	429,42	0,0000000
ENSG00000148123	PLPPR1	9	101028709	101325135	protein_coding	427,60	0,0000000
ENSG00000165973	NELL1	11	20669551	21575681	protein_coding	421,90	0,0000000
ENSG00000001626	CFTR	7	117465784	117715971	protein_coding	417,13	0,0000000
ENSG00000175077	RTP1	3	187197486	187201465	protein_coding	412,90	0,0000000
ENSG00000102466	FGF14	13	101710804	102402457	protein_coding	406,98	0,0000000
ENSG00000206557	TRIM71	3	32818018	32897826	protein_coding	400,19	0,0000000
ENSG00000171987	C11orf40	11	4571423	4577820	protein_coding	400,10	0,0000000

ENSG00000184221	OLIG1	21	33070144	33072420	protein_coding	397,86	0,0000000
ENSG00000183625	CCR3	3	46163604	46266706	protein_coding	396,63	0,0000000
ENSG00000101443	WFDC2	20	45469706	45481532	protein_coding	396,50	0,0000000
ENSG00000164049	FBXW12	3	48372219	48401259	protein_coding	396,16	0,0000000
ENSG00000237521	OR7E24	19	9250930	9252479	protein_coding	384,20	0,0000000
ENSG00000197893	NRAP	10	113588716	113664127	protein_coding	369,50	0,0000000
ENSG00000005471	ABCB4	7	87401697	87480435	protein_coding	369,32	0,0000000
ENSG00000162592	CCDC27	1	3752398	3771645	protein_coding	366,18	0,0000000
ENSG00000132164	SLC6A11	3	10816200	10940733	protein_coding	364,40	0,0000000
ENSG00000087586	AURKA	20	56369389	56392337	protein_coding	359,04	0,0000000
ENSG00000162624	LHX8	1	75128434	75161533	protein_coding	357,20	0,0000000
ENSG00000078725	BRINP1	9	119153458	119369467	protein_coding	350,04	0,0000000
ENSG00000114656	KIAA1257	3	128909866	129002690	protein_coding	346,15	0,0000000
ENSG00000083307	GRHL2	8	101492432	101669726	protein_coding	343,75	0,0012807
ENSG00000105251	SHD	19	4278601	4290724	protein_coding	339,85	0,0000000
ENSG00000174498	IGDCC3	15	65327127	65378040	protein_coding	338,16	0,0000000
ENSG00000156269	NAA11	4	79225694	79326050	protein_coding	334,40	0,0000000
ENSG00000008197	TFAP2D	6	50713828	50772988	protein_coding	323,60	0,0006696
ENSG00000175766	EIF4E1B	5	176630682	176646641	protein_coding	312,40	0,0000000
ENSG00000073282	TP63	3	189631416	189897279	protein_coding	309,82	0,0000000
ENSG00000156395	SORCS3	10	104641101	105265235	protein_coding	308,80	0,0000000
ENSG00000149735	GPHA2	11	64934471	64935888	protein_coding	305,90	0,0000000
ENSG00000187546	AGMO	7	15200318	15562015	protein_coding	305,40	0,0000000
ENSG00000136231	IGF2BP3	7	23310209	23470467	protein_coding	300,89	0,0000000
ENSG00000183733	FIGLA	2	70777310	70790643	protein_coding	300,60	0,0000000
ENSG00000047936	ROS1	6	117288300	117425855	protein_coding	299,20	0,0000000
ENSG00000106689	LHX2	9	124001670	124033301	protein_coding	296,50	0,0006696
ENSG00000170627	GTSF1	12	54455950	54473602	protein_coding	295,26	0,0000000
ENSG00000164675	IQUB	7	123452400	123535077	protein_coding	281,42	0,0000000
ENSG00000165623	UCMA	10	13221767	13234334	protein_coding	279,40	0,0000000
ENSG00000089169	RPH3A	12	112570380	112898881	protein_coding	274,23	0,0000000
ENSG00000196335	STK31	7	23710167	23832513	protein_coding	270,58	0,0000000
ENSG00000135638	EMX1	2	72916260	72936071	protein_coding	266,81	0,0000000
ENSG00000203910	C1orf146	1	92217940	92245813	protein_coding	264,29	0,0000000
ENSG00000176571	CNBD1	8	86866442	87615219	protein_coding	259,00	0,0000000
ENSG00000080166	DCT	13	94436808	94479682	protein_coding	257,19	0,0000000
ENSG00000179796	LRRC3B	3	26622806	26710776	protein_coding	255,50	0,0000000
ENSG00000177414	UBE2U	1	64203627	64267368	protein_coding	247,70	0,0000000
ENSG00000137392	CLPS	6	35794982	35797344	protein_coding	247,40	0,0000000
ENSG00000149548	CCDC15	11	124954121	125041489	protein_coding	244,54	0,0000000
ENSG00000158865	SLC5A11	16	24845841	24911628	protein_coding	244,20	0,0000000
ENSG00000182223	ZAR1	4	48490252	48494389	protein_coding	242,45	0,0000000
ENSG00000147676	MAL2	8	119165034	119245673	protein_coding	241,00	0,0000000
ENSG00000129354	AP1M2	19	10572671	10587315	protein_coding	235,60	0,0000000
ENSG00000148584	A1CF	10	50799409	50885675	protein_coding	231,10	0,0006696
ENSG00000156509	FBXO43	8	100133360	100145800	protein_coding	228,74	0,0000000
ENSG00000104313	EYA1	8	71197433	71362232	protein_coding	226,73	0,0000000
ENSG00000122592	HOXA7	7	27153716	27157936	protein_coding	226,31	0,0000000
ENSG00000154274	C4orf19	4	37453941	37623495	protein_coding	225,18	0,0000000
ENSG00000150275	PCDH15	10	53802771	55627942	protein_coding	223,20	0,0000000
ENSG00000164330	EBF1	5	158695916	159099761	protein_coding	220,90	0,0000000
ENSG00000178462	TUBAL3	10	5393098	5404830	protein_coding	216,80	0,0000000
ENSG00000183508	FAM46C	1	117605934	117628372	protein_coding	216,09	0,0000000
ENSG00000206075	SERPINB5	18	63476761	63505085	protein_coding	216,00	0,0000000
ENSG00000066468	FGFR2	10	121478334	121598458	protein_coding	215,06	0,0000000

ENSG00000105894	PTN	7	137227341	137343865	protein_coding	214,93	0,0000000
ENSG00000173769	TOPAZ1	3	44241886	44332098	protein_coding	212,20	0,0006696
ENSG00000125207	PIWIL1	12	130337887	130372637	protein_coding	211,00	0,0000000
ENSG00000131914	LIN28A	1	26410778	26429722	protein_coding	210,90	0,0000000
ENSG00000250254	PTTG2	4	37960435	37961125	protein_coding	210,54	0,2229774
ENSG00000174898	CATSPERD	19	5720677	5778734	protein_coding	209,91	0,0000000
ENSG00000113520	IL4	5	132673986	132682676	protein_coding	209,36	0,0000000
ENSG00000276043	UHRF1	19	4903080	4962154	protein_coding	205,52	0,0000000
ENSG00000173406	DAB1	1	56994778	58546734	protein_coding	205,20	0,0000000
ENSG00000039600	SOX30	5	157625679	157671480	protein_coding	204,03	0,0000000
ENSG00000206384	COL6A6	3	130560334	130678155	protein_coding	199,69	0,0000000
ENSG00000273045	C2orf15	2	99141485	99151487	protein_coding	199,18	0,0000000
ENSG00000240204	SMKR1	7	129502479	129512932	protein_coding	198,04	0,0000000
ENSG00000156049	GNA14	9	77423079	77648307	protein_coding	197,81	0,0000000
ENSG00000157703	SVOPL	7	138594285	138701352	protein_coding	194,94	0,0000000
ENSG00000144285	SCN1A	2	165989160	166128047	protein_coding	193,40	0,0006696
ENSG00000179915	NRXN1	2	49918505	51225564	protein_coding	192,18	0,0000000
ENSG00000091704	CPA1	7	130380339	130388114	protein_coding	190,83	0,0000000
ENSG00000197561	ELANE	19	851014	856247	protein_coding	188,00	0,0006696
ENSG00000015413	DPEP1	16	89613308	89638456	protein_coding	186,62	0,0012807
ENSG00000176399	DMRTA1	9	22446841	22455740	protein_coding	184,67	0,0000000
ENSG00000166796	LDHC	11	18412307	18452058	protein_coding	184,61	0,0000000
ENSG00000138795	LEF1	4	108047545	108168956	protein_coding	183,12	0,0000000
ENSG00000107018	RLN1	9	5334969	5339873	protein_coding	183,07	0,0000000
ENSG00000183117	CSMD1	8	2935353	4994972	protein_coding	182,40	0,0000000
ENSG00000181847	TIGIT	3	114276913	114310288	protein_coding	177,00	0,0012807
ENSG00000273079	GRIN2B	12	13537337	13981957	protein_coding	174,30	0,0000000
ENSG00000043039	BARX2	11	129375940	129452279	protein_coding	173,50	0,0006696
ENSG00000101746	NOL4	18	33851100	34224952	protein_coding	172,00	0,0000000
ENSG00000188032	C19orf67	19	14081619	14085875	protein_coding	169,17	0,0012807
ENSG00000172987	HPSE2	10	98457077	99235862	protein_coding	168,69	0,0000000
ENSG00000183166	CALN1	7	71779491	72447151	protein_coding	168,18	0,0000000
ENSG00000149054	ZNF215	11	6926404	6984632	protein_coding	167,97	0,0000000
ENSG00000131055	COX4I2	20	31637888	31645006	protein_coding	166,40	0,0000000
ENSG00000102104	RS1	X	18639910	18672109	protein_coding	163,47	0,0000000
ENSG00000148482	SLC39A12	10	17951839	18043292	protein_coding	162,40	0,0000000
ENSG00000196277	GRM7	3	6770001	7741533	protein_coding	160,36	0,0000000
ENSG00000118307	CASC1	12	25108420	25195162	protein_coding	159,66	0,0000000
ENSG00000158806	NPM2	8	22024125	22036897	protein_coding	159,65	0,0012807
ENSG00000167889	MGAT5B	17	76868456	76950393	protein_coding	157,36	0,0000000
ENSG00000145721	LIX1	5	97091867	97142872	protein_coding	156,30	0,0006696
ENSG00000070808	CAMK2A	5	150219491	150290291	protein_coding	155,90	0,0000000
ENSG00000198178	CLEC4C	12	7729415	7751605	protein_coding	154,70	0,0000000
ENSG00000158077	NLRP14	11	7020446	7071308	protein_coding	153,31	0,0000000
ENSG00000133808	MICALCL	11	12276080	12359144	protein_coding	153,24	0,0000000
ENSG00000109794	FAM149A	4	186104419	186172667	protein_coding	151,80	0,0000000
ENSG00000152953	STK32B	4	5051442	5500998	protein_coding	149,77	0,0000000
ENSG00000152669	CCNO	5	55231152	55233680	protein_coding	149,06	0,0000000
ENSG00000124493	GRM4	6	34018645	34155622	protein_coding	148,80	0,0000000
ENSG00000164404	GDF9	5	132861181	132866884	protein_coding	147,06	0,0000000
ENSG00000169862	CTNND2	5	10971840	11904043	protein_coding	146,91	0,0000000
ENSG00000159495	TGM7	15	43276280	43302255	protein_coding	144,40	0,0000000
ENSG00000168702	LRP1B	2	140231423	142131701	protein_coding	143,88	0,0000000
ENSG00000162639	HENMT1	1	108648290	108661526	protein_coding	143,45	0,0000000
ENSG00000168843	FSTL5	4	161383897	162164035	protein_coding	142,40	0,0006696

ENSG00000156218	ADAMTSL3	15	83654086	84039842	protein_coding	142,31	0,0000000
ENSG00000185518	SV2B	15	91099950	91301309	protein_coding	142,00	0,0000000
ENSG00000102468	HTR2A	13	46831550	46897076	protein_coding	141,80	0,0000000
ENSG00000146049	KAAG1	6	24356903	24358284	protein_coding	141,08	0,0050271
ENSG00000040608	RTN4R	22	20241415	20283246	protein_coding	140,88	0,0000000
ENSG00000135960	EDAR	2	108894471	108989372	protein_coding	140,25	0,0000000
ENSG00000188886	ASTL	2	96123850	96138436	protein_coding	140,19	0,0012807
ENSG00000176566	DCAF4L2	8	87870743	87874068	protein_coding	138,65	0,0000000
ENSG00000138798	EGF	4	109912884	110012266	protein_coding	135,88	0,0000000
ENSG00000198807	PAX9	14	36657568	36679715	protein_coding	135,55	0,0000000
ENSG00000132514	CLEC10A	17	7074537	7080307	protein_coding	135,51	0,0000000
ENSG00000134258	VTCN1	1	117143587	117210960	protein_coding	135,42	0,0000000
ENSG00000069018	TRPC7	5	136213320	136365537	protein_coding	133,90	0,0012807
ENSG00000132972	RNF17	13	24764152	24879921	protein_coding	132,75	0,0000000
ENSG00000151952	TMEM132D	12	129071725	129903666	protein_coding	132,50	0,0006696
ENSG00000178217	SH2D4B	10	80537902	80646560	protein_coding	129,18	0,0006696
ENSG00000058335	RASGRF1	15	78959947	79090773	protein_coding	128,92	0,0000000
ENSG00000187889	C1orf168	1	56718804	56819696	protein_coding	127,70	0,0006696
ENSG00000275896	PRSS2	7	142748662	142774564	protein_coding	127,40	0,0000000
ENSG00000173207	CKS1B	1	154974653	154979249	protein_coding	126,64	0,0000000
ENSG00000139287	TPH2	12	71938846	72186618	protein_coding	126,58	0,0000000
ENSG00000126787	DLGAP5	14	55148112	55191678	protein_coding	124,20	0,0000000
ENSG00000080709	KCNN2	5	114360945	114496500	protein_coding	123,72	0,0000000
ENSG00000149972	CNTN5	11	99020953	100358885	protein_coding	123,45	0,0012807
ENSG00000170927	PKHD1	6	51615300	52087625	protein_coding	121,50	0,0000000
ENSG00000126016	AMOT	X	112774503	112840815	protein_coding	120,31	0,0000000
ENSG00000187094	CCK	3	42257825	42266207	protein_coding	118,40	0,0000000
ENSG00000141255	SPATA22	17	3440019	3513852	protein_coding	116,21	0,0000000
ENSG00000269964	MEI4	6	77690658	77926974	protein_coding	114,79	0,0000000
ENSG00000179023	KLHDC7A	1	18480982	18486126	protein_coding	113,40	0,0000000
ENSG00000243910	TUBA4B	2	219253243	219272188	protein_coding	112,00	0,0000000
ENSG00000132698	RAB25	1	156061160	156070514	protein_coding	110,06	0,0000000
ENSG00000196074	SYCP2	20	59863563	59933655	protein_coding	109,83	0,0000000
ENSG00000177398	UMODL1	21	42062959	42143453	protein_coding	109,59	0,0000000
ENSG00000153237	CCDC148	2	158171081	158456753	protein_coding	109,22	0,0000000
ENSG00000080007	DDX43	6	73394748	73417569	protein_coding	108,31	0,0000000
ENSG00000169594	BNC1	15	83255903	83284716	protein_coding	107,93	0,0000000
ENSG00000112273	HDGFL1	6	22569493	22571666	protein_coding	107,10	0,0000000
ENSG00000102837	OLFM4	13	53028759	53052057	protein_coding	107,09	0,0000000
ENSG00000180113	TDRD6	6	46687875	46704319	protein_coding	106,74	0,0000000
ENSG00000152503	TRIM36	5	115124762	115180546	protein_coding	106,67	0,0000000
ENSG00000203805	PLPP4	10	120456954	120589855	protein_coding	106,59	0,0000000
ENSG00000187123	LYPD6	2	149329985	149474148	protein_coding	106,49	0,0000000
ENSG00000106927	AMBP	9	114060127	114078472	protein_coding	105,43	0,0000000
ENSG00000205129	C4orf47	4	185426249	185449826	protein_coding	105,31	0,0000000
ENSG00000166800	LDHAL6A	11	18455824	18479600	protein_coding	104,81	0,0000000
ENSG00000060709	RIMBP2	12	130396137	130716281	protein_coding	104,22	0,0000000
ENSG00000118308	LRMP	12	25021002	25108334	protein_coding	103,04	0,0000000
ENSG00000132321	IQCA1	2	236324147	236507542	protein_coding	101,63	0,0000000
ENSG00000137473	TTC29	4	146706638	146945882	protein_coding	101,40	0,0000000
ENSG00000157219	HTR5A	7	155070324	155085749	protein_coding	101,00	0,0012807
ENSG00000256463	SALL3	18	78980275	79002677	protein_coding	100,89	0,0012807
ENSG00000077152	UBE2T	1	202331657	202341980	protein_coding	100,43	0,0000000
ENSG00000132646	PCNA	20	5114953	5126626	protein_coding	99,73	0,0000000
ENSG00000149403	GRIK4	11	120511759	120988904	protein_coding	99,39	0,0000000

ENSG00000215018	COL28A1	7	7356203	7535853	protein_coding	99,29	0,0000000
ENSG00000105146	AURKC	19	57230802	57235548	protein_coding	99,19	0,0000000
ENSG00000187695	ENSG000001	3	128909874	128971330	protein_coding	98,46	0,0111464
ENSG00000157168	NRG1	8	31639386	32767959	protein_coding	97,68	0,0000000
ENSG00000177181	RIMKLA	1	42380795	42422578	protein_coding	97,63	0,0000000
ENSG00000108849	PPY	17	43940804	43942468	protein_coding	97,60	0,0000000
ENSG00000188869	TMC3	15	81331217	81374213	protein_coding	97,47	0,0012807
ENSG00000179148	ALOXE3	17	8095900	8119047	protein_coding	97,07	0,0000000
ENSG00000269699	ZIM2	19	56774552	56840729	protein_coding	96,53	0,0000000
ENSG00000244476	ERVFRD-1	6	11102489	11111732	protein_coding	95,60	0,0000000
ENSG00000169427	KCNK9	8	139600838	139703056	protein_coding	94,83	0,0105771
ENSG00000169836	TACR3	4	103586031	103719816	protein_coding	94,38	0,0000000
ENSG00000082126	MPP4	2	201644870	201698694	protein_coding	94,04	0,0000000
ENSG00000156299	TIAM1	21	31118416	31559977	protein_coding	93,16	0,0000000
ENSG00000214866	DCDC2C	2	3703592	3847408	protein_coding	92,50	0,0006696
ENSG00000197181	PIWIL2	8	22275297	22357563	protein_coding	92,35	0,0000000
ENSG00000259040	BLOC1S5-TX	6	7881522	8064364	protein_coding	92,04	0,0000000
ENSG00000180828	BHLHE22	8	64580367	64583628	protein_coding	91,40	0,0000000
ENSG00000276087	ENSG000002	2	24124366	24190436	protein_coding	91,31	0,0012807
ENSG00000048052	HDAC9	7	18086949	19002416	protein_coding	90,87	0,0000000
ENSG00000163092	XIRP2	2	166888487	167259753	protein_coding	90,30	0,0025348
ENSG00000215915	ATAD3C	1	1449689	1470158	protein_coding	90,10	0,0000000
ENSG00000128645	HOXD1	2	176188579	176190907	protein_coding	89,23	0,0000000
ENSG00000180210	F2	11	46719180	46739506	protein_coding	89,09	0,0012807
ENSG00000039068	CDH1	16	68737225	68835548	protein_coding	88,99	0,0012807
ENSG00000134057	CCNB1	5	69167010	69178245	protein_coding	88,50	0,0000000
ENSG00000197361	FBXL22	15	63597353	63602428	protein_coding	88,26	0,0000000
ENSG00000171505	OR1N1	9	122526358	122527296	protein_coding	88,20	0,0000000
ENSG00000101115	SALL4	20	51782331	51802520	protein_coding	87,60	0,0012807
ENSG00000214575	CPEB1	15	82543201	82648861	protein_coding	87,13	0,0000000
ENSG00000147082	CCNB3	X	50202713	50351910	protein_coding	87,11	0,0012807
ENSG00000185053	SGCZ	8	14089864	15238339	protein_coding	86,73	0,0000000
ENSG00000119915	ELOVL3	10	102226328	102229589	protein_coding	86,06	0,0012807
ENSG00000101405	OXT	20	3071620	3072517	protein_coding	86,05	0,0000000
ENSG00000113905	HRG	3	186660216	186678240	protein_coding	85,30	0,0000000
ENSG00000134460	IL2RA	10	6010689	6062325	protein_coding	84,95	0,0000000
ENSG00000196944	OR2T4	1	248361581	248362627	protein_coding	84,90	0,0000000
ENSG00000056050	C4orf27	4	169729465	169757953	protein_coding	84,25	0,0000000
ENSG00000164841	TMEM74	8	108606850	108787615	protein_coding	84,20	0,0012807
ENSG00000118690	ARMC2	6	108848416	108974472	protein_coding	82,83	0,0000000
ENSG00000172244	C5orf34	5	43486701	43515145	protein_coding	82,17	0,0000000
ENSG00000186487	MYT1L	2	1789113	2331260	protein_coding	82,00	0,0000000
ENSG00000036672	USP2	11	119355215	119381726	protein_coding	81,19	0,0000000
ENSG00000186075	ZPBP2	17	39868164	39877896	protein_coding	81,10	0,0006696
ENSG00000112499	SLC22A2	6	160171061	160277638	protein_coding	80,40	0,0000000
ENSG00000160396	HIPK4	19	40379271	40390187	protein_coding	79,84	0,0012807
ENSG00000279956	ENSG000002	2	48632291	48755724	protein_coding	78,93	0,0000000
ENSG00000189108	IL1RAPL2	X	104566315	105767829	protein_coding	78,25	0,0012807
ENSG00000140955	ADAD2	16	84191138	84197168	protein_coding	78,13	0,0012807
ENSG00000111249	CUX2	12	111034024	111350554	protein_coding	77,90	0,0000000
ENSG00000144821	MYH15	3	108380369	108529322	protein_coding	77,78	0,0012807
ENSG00000154118	JPH3	16	87601835	87698156	protein_coding	77,63	0,0000000
ENSG00000130201	EXOC3L2	19	45212621	45234211	protein_coding	77,14	0,0000000
ENSG00000166578	IQCD	12	113195441	113221094	protein_coding	77,13	0,0000000
ENSG00000178397	FAM220A	7	6329409	6348981	protein_coding	76,44	0,0000000

ENSG00000260027	HOXB7	17	48607227	48633572	protein_coding	76,20	0,0012807
ENSG00000175063	UBE2C	20	45812576	45816957	protein_coding	75,21	0,0000000
ENSG00000181778	TMEM252	9	68536580	68540867	protein_coding	75,13	0,0012807
ENSG00000141431	ASXL3	18	33578615	33751192	protein_coding	74,94	0,0000000
ENSG00000169402	RSPH10B2	7	6754109	6799365	protein_coding	74,65	0,0012807
ENSG00000154975	CA10	17	51630313	52160017	protein_coding	74,60	0,0000000
ENSG00000054803	CBLN4	20	55997440	56005472	protein_coding	73,75	0,0000000
ENSG00000013810	TACC3	4	1721490	1745176	protein_coding	73,10	0,0000000
ENSG00000094963	FMO2	1	171185208	171211230	protein_coding	72,08	0,0012807
ENSG00000106128	GHRHR	7	30938669	30993254	protein_coding	69,70	0,0006696
ENSG00000123977	DAW1	2	227871054	227924344	protein_coding	69,48	0,0000000
ENSG00000102678	FGF9	13	21671383	21704498	protein_coding	69,15	0,0012807
ENSG00000130226	DPP6	7	153887097	154894285	protein_coding	69,15	0,0000000
ENSG00000079112	CDH17	8	94127171	94217303	protein_coding	68,97	0,0000000
ENSG00000178222	RNF212	4	1056250	1113562	protein_coding	68,97	0,0000000
ENSG00000074211	PPP2R2C	4	6320578	6563600	protein_coding	68,65	0,0012807
ENSG00000188523	CFAP77	9	132410043	132573317	protein_coding	68,38	0,0000000
ENSG00000159374	M1AP	2	74557883	74648338	protein_coding	68,23	0,0000000
ENSG00000196139	AKR1C3	10	5035354	5107686	protein_coding	68,10	0,0000000
ENSG00000092054	MYH7	14	23412738	23435718	protein_coding	68,09	0,0012807
ENSG00000179314	WSCD1	17	5772234	6124427	protein_coding	67,80	0,0000000
ENSG00000071909	MYO3B	2	170178145	170655171	protein_coding	67,72	0,0000000
ENSG00000104147	OIP5	15	41309268	41332621	protein_coding	67,34	0,0000000
ENSG00000173432	SAA1	11	18266174	18269977	protein_coding	67,27	0,0000000
ENSG00000092853	CLSPN	1	35720218	35769967	protein_coding	67,09	0,0012807
ENSG00000175600	SUGCT	7	40134977	40860763	protein_coding	66,91	0,0000000
ENSG00000186377	CYP4X1	1	47023568	47050751	protein_coding	66,83	0,0000000
ENSG00000187997	C17orf99	17	78146353	78166177	protein_coding	66,70	0,0012807
ENSG00000184672	RALYL	8	84182787	84921844	protein_coding	65,75	0,0000000
ENSG00000086717	PPEF1	X	18675909	18827921	protein_coding	65,16	0,0000000
ENSG00000019186	CYP24A1	20	54153449	54173973	protein_coding	64,73	0,0000000
ENSG00000176771	NCKAP5	2	132671799	133568463	protein_coding	63,86	0,0000000
ENSG00000168070	C11orf85	11	64937517	64972085	protein_coding	63,67	0,0000000
ENSG00000126778	SIX1	14	60643415	60658259	protein_coding	63,50	0,0025348
ENSG00000229453	SPINK8	3	48306842	48328341	protein_coding	63,20	0,0000000
ENSG00000183742	MACC1	7	20134655	20217404	protein_coding	63,18	0,0012807
ENSG00000145604	SKP2	5	36151989	36184319	protein_coding	63,10	0,0000000
ENSG00000128606	LRRC17	7	102912991	102944949	protein_coding	63,05	0,0000000
ENSG00000133937	GSC	14	94768216	94770230	protein_coding	62,58	0,0000000
ENSG00000184185	KCNJ12	17	21376197	21419872	protein_coding	62,45	0,0184474
ENSG00000135175	OCM2	7	97984684	97991169	protein_coding	62,30	0,0006696
ENSG00000145832	SLC25A48	5	135834649	135888637	protein_coding	62,00	0,0000000
ENSG00000146453	PNLDC1	6	159800249	159820704	protein_coding	61,25	0,0000000
ENSG00000135451	TROAP	12	49323236	49331731	protein_coding	61,22	0,0000000
ENSG00000164920	OSR2	8	98944403	98952104	protein_coding	61,06	0,0000000
ENSG00000179284	DAND5	19	12965159	12974762	protein_coding	59,64	0,0025348
ENSG00000237136	C4orf51	4	145680204	145771032	protein_coding	59,40	0,0000000
ENSG00000152804	HHEX	10	92689951	92695646	protein_coding	59,21	0,0000000
ENSG00000137261	KIAA0319	6	24544104	24646155	protein_coding	58,78	0,0000000
ENSG00000066405	CLDN18	3	137998735	138033655	protein_coding	58,23	0,0012807
ENSG00000151090	THRB	3	24117160	24495282	protein_coding	58,15	0,0000000
ENSG00000147724	FAM135B	8	138130023	138496822	protein_coding	58,10	0,0000000
ENSG00000104722	NEFM	8	24913012	24919098	protein_coding	58,04	0,0062591
ENSG00000165490	DDIAS	11	82899975	82958277	protein_coding	57,98	0,0000000
ENSG00000131620	ANO1	11	70078302	70189528	protein_coding	57,92	0,0000000

ENSG00000281370	ENSG000002	12	102075068	102075229	protein_coding	57,75	0,0000000
ENSG00000130816	DNMT1	19	10133345	10231286	protein_coding	57,62	0,0000000
ENSG00000263513	FAM72C	1	143955364	143971965	protein_coding	57,43	0,0000000
ENSG00000134020	PEBP4	8	22713251	23000000	protein_coding	57,32	0,0012807
ENSG00000173976	RAX2	19	3769089	3772221	protein_coding	56,90	0,0012807
ENSG00000132446	FTHL17	X	31071241	31072053	protein_coding	56,70	0,0006696
ENSG00000249961	CCDC79	16	66754976	66801620	protein_coding	56,36	0,0012807
ENSG00000144460	NYAP2	2	225400648	225654018	protein_coding	56,32	0,0000000
ENSG00000105877	DNAH11	7	21543215	21901839	protein_coding	56,02	0,0000000
ENSG00000143032	BARHL2	1	90711539	90717237	protein_coding	56,00	0,0012807
ENSG00000143228	NUF2	1	163266576	163355764	protein_coding	55,80	0,0012807
ENSG00000164089	ETNPPL	4	108742040	108763054	protein_coding	55,79	0,0025348
ENSG00000106484	MEST	7	130486171	130506296	protein_coding	55,63	0,0000000
ENSG00000128408	RIBC2	22	45413691	45432496	protein_coding	55,61	0,0148097
ENSG00000136542	GALNT5	2	157257598	157314211	protein_coding	55,60	0,0000000
ENSG00000164109	MAD2L1	4	120055608	120067074	protein_coding	55,34	0,0000000
ENSG00000042781	USH2A	1	215622894	216423396	protein_coding	55,24	0,0000000
ENSG00000127780	OR1E2	17	3432870	3433841	protein_coding	55,00	0,0000000
ENSG00000168298	HIST1H1E	6	26156354	26157107	protein_coding	54,37	0,0609506
ENSG00000152214	RIT2	18	42743227	43115691	protein_coding	54,30	0,0000000
ENSG00000165304	MELK	9	36572862	36677683	protein_coding	54,14	0,0000000
ENSG00000151812	SLC35F4	14	57563922	57982194	protein_coding	54,00	0,0000000
ENSG00000198010	DLGAP2	8	1501366	1708476	protein_coding	54,00	0,0012807
ENSG00000094755	GABRP	5	170763350	170814047	protein_coding	54,00	0,0006696
ENSG00000156475	PPP2R2B	5	146581146	147084784	protein_coding	53,36	0,0012807
ENSG00000095464	PDE6C	10	93612588	93666010	protein_coding	52,60	0,0050271
ENSG00000173805	HAP1	17	41717742	41734644	protein_coding	52,23	0,0012807
ENSG00000261949	GFY	19	49423749	49428818	protein_coding	52,20	0,0006696
ENSG00000171848	RRM2	2	10122328	10131419	protein_coding	52,02	0,0000000
ENSG00000007001	UPP2	2	157876702	158136154	protein_coding	51,90	0,0000000
ENSG00000156265	MAP3K7CL	21	29077471	29175889	protein_coding	51,69	0,0000000
ENSG00000140015	KCNH5	14	62699454	63102037	protein_coding	51,60	0,0006696
ENSG00000122674	CCZ1	7	5898725	5926550	protein_coding	51,52	0,0000000
ENSG00000171587	DSCAM	21	40010999	40847139	protein_coding	51,50	0,0006696
ENSG00000182968	SOX1	13	112067647	112070488	protein_coding	51,50	0,0006696
ENSG00000182308	DCAF4L1	4	41981696	41986467	protein_coding	51,44	0,0000000
ENSG00000131979	GCH1	14	54842008	54902852	protein_coding	51,28	0,0000000
ENSG00000145919	BOD1	5	173607514	173616660	protein_coding	51,07	0,0000000
ENSG00000166509	CLEC3A	16	78022515	78066761	protein_coding	51,00	0,0025348
ENSG00000151322	NPAS3	14	32934933	33804176	protein_coding	50,89	0,0000000
ENSG00000139865	TTC6	14	37595847	38041442	protein_coding	50,70	0,0006696
ENSG00000174844	DNAH12	3	57293699	57544344	protein_coding	50,43	0,0000000
ENSG00000165105	RASEF	9	82979585	83063177	protein_coding	50,34	0,0000000
ENSG00000168928	CTRB2	16	75204096	75207185	protein_coding	49,86	0,0000000
ENSG00000229474	PATL2	15	44665732	44711316	protein_coding	49,62	0,0012807
ENSG00000168959	GRM5	11	88504576	89065945	protein_coding	49,58	0,0012807
ENSG00000166415	WDR72	15	53513741	53762878	protein_coding	49,00	0,0000000
ENSG00000090889	KIF4A	X	70290090	70420832	protein_coding	48,82	0,0000000
ENSG00000172320	OR5A1	11	59443144	59444194	protein_coding	48,80	0,0006696
ENSG00000204711	C9orf135	9	69820793	69906232	protein_coding	48,54	0,0012807
ENSG00000162782	TDRD5	1	179591613	179691272	protein_coding	48,46	0,0012807
ENSG00000143921	ABCG8	2	43838964	43878466	protein_coding	48,33	0,0000000
ENSG00000105523	FAM83E	19	48600810	48614854	protein_coding	48,12	0,0062591
ENSG00000189056	RELN	7	103471784	103989516	protein_coding	48,09	0,0000000
ENSG00000156006	NAT2	8	18391245	18401218	protein_coding	48,00	0,0000000

ENSG00000185272	RBM11	21	14216130	14228372	protein_coding	47,98	0,0000000
ENSG00000184661	CDCA2	8	25458997	25507920	protein_coding	47,66	0,0012807
ENSG00000183317	EPHA10	1	37713880	37765133	protein_coding	47,64	0,0000000
ENSG00000155511	GRIA1	5	153489615	153813869	protein_coding	47,50	0,0012807
ENSG00000273111	LYPD4	19	41837074	41844697	protein_coding	47,40	0,0000000
ENSG00000183560	IZUMO1R	11	94305593	94307721	protein_coding	47,15	0,0012807
ENSG00000104213	PDGFRL	8	17576433	17644071	protein_coding	47,14	0,0000000
ENSG00000163817	SLC6A20	3	45755450	45796535	protein_coding	46,97	0,0006696
ENSG00000206102	KRTAP19-8	21	31038159	31038476	protein_coding	46,90	0,0006696
ENSG00000167741	GGT6	17	4556927	4560818	protein_coding	46,80	0,0006696
ENSG00000198331	HYLS1	11	125883614	125900648	protein_coding	46,62	0,0000000
ENSG00000153233	PTPRR	12	70638073	70920843	protein_coding	46,61	0,0000000
ENSG00000006047	YBX2	17	7288252	7294615	protein_coding	46,33	0,0000000
ENSG00000124391	IL17C	16	88638591	88640473	protein_coding	46,30	0,0012807
ENSG00000164756	SLC30A8	8	116950273	117176714	protein_coding	46,21	0,0000000
ENSG00000117399	CDC20	1	43358955	43363203	protein_coding	45,95	0,0000000
ENSG00000157111	TMEM171	5	73120292	73131817	protein_coding	45,94	0,0000000
ENSG00000171243	SOSTDC1	7	16461481	16530580	protein_coding	45,87	0,0000000
ENSG00000205549	C9orf92	9	16203935	16276313	protein_coding	45,20	0,0000000
ENSG00000157152	SYN2	3	12004402	12191400	protein_coding	45,08	0,0000000
ENSG00000077935	SMC1B	22	45344063	45413619	protein_coding	44,85	0,0012807
ENSG00000128040	SPINK2	4	56809860	56821742	protein_coding	44,30	0,0012807
ENSG00000165828	PRAP1	10	133309410	133352683	protein_coding	44,18	0,0000000
ENSG00000153898	MCOLN2	1	84925583	84997113	protein_coding	44,13	0,0000000
ENSG00000181143	MUC16	19	8848844	8981342	protein_coding	44,00	0,0012807
ENSG00000181541	MAB21L2	4	150581922	150584693	protein_coding	43,83	0,0184474
ENSG00000153162	BMP6	6	7726797	7881422	protein_coding	43,81	0,0000000
ENSG00000119125	GDA	9	72114595	72257193	protein_coding	43,46	0,0012807
ENSG00000133980	VRTN	14	74303069	74360008	protein_coding	43,30	0,0006696
ENSG00000156970	BUB1B	15	40161023	40221136	protein_coding	43,04	0,0000000
ENSG00000070501	POLB	8	42338454	42371808	protein_coding	42,83	0,0000000
ENSG00000172331	BPGM	7	134646808	134679813	protein_coding	42,75	0,0000000
ENSG00000006747	SCIN	7	12570577	12660179	protein_coding	42,65	0,0000000
ENSG00000174527	MYO1H	12	109347903	109455523	protein_coding	42,62	0,0037996
ENSG00000140563	MCTP2	15	94231538	94483952	protein_coding	42,50	0,0000000
ENSG00000155026	RSPH10B	7	5925550	5970683	protein_coding	42,32	0,0000000
ENSG00000158813	EDA	X	69616067	70039469	protein_coding	42,21	0,0000000
ENSG00000138778	CENPE	4	103105806	103198409	protein_coding	42,13	0,0000000
ENSG00000109927	TECTA	11	121101173	121191493	protein_coding	42,08	0,0000000
ENSG00000123485	HJURP	2	233833416	233854566	protein_coding	42,03	0,0000000
ENSG00000165863	C10orf82	10	116663696	116670264	protein_coding	41,76	0,0000000
ENSG00000274997	HIST1H2AH	6	27147129	27147515	protein_coding	41,71	0,0012807
ENSG00000115163	CENPA	2	26764289	26801067	protein_coding	41,55	0,0000000
ENSG00000164508	HIST1H2AA	6	25726132	25726527	protein_coding	41,40	0,0418702
ENSG00000185056	C5orf47	5	173973779	174006140	protein_coding	41,37	0,0000000
ENSG00000093009	CDC45	22	19479459	19520612	protein_coding	41,35	0,0000000
ENSG00000143333	RGS16	1	182598623	182604408	protein_coding	41,04	0,0000000
ENSG00000007944	MYLIP	6	16129125	16148248	protein_coding	40,87	0,0000000
ENSG00000121152	NCAPH	2	96335787	96373845	protein_coding	40,43	0,0075075
ENSG00000144893	MED12L	3	151085697	151437072	protein_coding	40,40	0,0000000
ENSG00000183801	OLFML1	11	7485388	7511377	protein_coding	40,40	0,0000000
ENSG00000187021	PNLIPRP1	10	116590385	116609175	protein_coding	40,00	0,0006696
ENSG00000215784	FAM72D	1	145096000	145112696	protein_coding	40,00	0,0000000
ENSG00000157456	CCNB2	15	59105078	59125045	protein_coding	39,91	0,0000000
ENSG00000142789	CELA3A	1	22001656	22012539	protein_coding	39,90	0,0418702

ENSG00000154227	CERS3	15	100400395	100544995	protein_coding	39,30	0,0006696
ENSG00000277481	PKD1L3	16	71929538	71999978	protein_coding	39,00	0,0000000
ENSG00000153157	SYCP2L	6	10886831	10979320	protein_coding	39,00	0,0000000
ENSG00000134215	VAV3	1	107571160	107965144	protein_coding	38,64	0,0000000
ENSG00000119973	PRLHR	10	118589989	118595699	protein_coding	38,45	0,0000000
ENSG00000121211	MND1	4	153344649	153415118	protein_coding	38,35	0,0000000
ENSG00000121236	TRIM6	11	5596109	5612958	protein_coding	38,26	0,0000000
ENSG00000105011	ASF1B	19	14119509	14136956	protein_coding	38,20	0,0000000
ENSG00000149554	CHEK1	11	125625136	125676255	protein_coding	38,17	0,0000000
ENSG00000138617	PARP16	15	65234460	65300618	protein_coding	38,00	0,0000000
ENSG00000154493	C10orf90	10	126424997	126670446	protein_coding	38,00	0,0000000
ENSG00000105173	CCNE1	19	29811898	29824308	protein_coding	37,54	0,0000000
ENSG00000158089	GALNT14	2	30910467	31155202	protein_coding	37,27	0,0000000
ENSG00000184611	KCNH7	2	162371407	162838730	protein_coding	36,43	0,0012807
ENSG00000156876	SASS6	1	100083563	100132955	protein_coding	36,40	0,0000000
ENSG00000183559	C10orf120	10	122697709	122699822	protein_coding	36,40	0,0000000
ENSG00000169436	COL22A1	8	138588235	138914006	protein_coding	36,27	0,0075075
ENSG00000065328	MCM10	10	13161554	13211104	protein_coding	35,94	0,0000000
ENSG00000171435	KSR2	12	117453012	117968983	protein_coding	35,80	0,0012807
ENSG00000273983	HIST1H3G	6	26269405	26271815	protein_coding	35,63	0,0012807
ENSG00000182256	GABRG3	15	26971282	27541991	protein_coding	35,50	0,0000000
ENSG00000062038	CDH3	16	68636189	68722616	protein_coding	35,42	0,0000000
ENSG00000182334	OR5P3	11	7825037	7825972	protein_coding	35,40	0,0012807
ENSG00000131773	KHDRBS3	8	135457457	135656722	protein_coding	35,40	0,0000000
ENSG00000021826	CPS1	2	210477682	210679107	protein_coding	35,34	0,0000000
ENSG00000006788	MYH13	17	10298084	10373130	protein_coding	35,27	0,0012807
ENSG00000203995	ZYG11A	1	52842511	52894998	protein_coding	35,03	0,0000000
ENSG00000039139	DNAH5	5	13690331	13944543	protein_coding	34,95	0,0012807
ENSG00000204595	DPRX	19	53632056	53637009	protein_coding	34,90	0,0000000
ENSG00000175879	HOXD8	2	176129694	176132695	protein_coding	34,86	0,0000000
ENSG00000157303	SUSD3	9	93058688	93085138	protein_coding	34,76	0,0000000
ENSG00000112706	IMPG1	6	75921115	76072678	protein_coding	34,72	0,0000000
ENSG00000138376	BARD1	2	214725646	214809711	protein_coding	34,65	0,0000000
ENSG00000179603	GRM8	7	126438598	127253294	protein_coding	34,63	0,0000000
ENSG00000165899	OTOGL	12	80209453	80379090	protein_coding	34,50	0,0000000
ENSG00000112941	PAPD7	5	6713007	6757048	protein_coding	34,38	0,0000000
ENSG00000183273	CCDC60	12	119334712	119541047	protein_coding	34,36	0,0000000
ENSG00000171643	S100Z	5	76850001	76921650	protein_coding	34,28	0,0012807
ENSG00000085552	IGSF9	1	159927039	159945604	protein_coding	34,25	0,0000000
ENSG00000164674	SYTL3	6	158650014	158764876	protein_coding	34,13	0,0000000
ENSG00000239642	MEIKIN	5	131806993	131945698	protein_coding	34,00	0,0006696
ENSG00000151475	SLC25A31	4	127730378	127774299	protein_coding	33,82	0,0000000
ENSG00000166748	AGBL1	15	86141996	87029052	protein_coding	33,80	0,0000000
ENSG00000160188	RSPH1	21	42472486	42496354	protein_coding	33,76	0,0000000
ENSG00000183840	GPR39	2	132416574	132646559	protein_coding	33,68	0,0000000
ENSG00000157542	KCNJ6	21	37607376	37916446	protein_coding	33,67	0,0000000
ENSG00000132801	ZSWIM3	20	45857617	45879122	protein_coding	33,66	0,0000000
ENSG00000153266	FEZF2	3	62369681	62374324	protein_coding	33,44	0,0025348
ENSG00000172073	TEX37	2	88524651	88529584	protein_coding	33,30	0,0025348
ENSG00000077279	DCX	X	111293780	111412375	protein_coding	33,30	0,0025348
ENSG00000184029	DSCR4	21	37951425	38121360	protein_coding	33,20	0,0006696
ENSG00000229544	NKX1-2	10	124445239	124450184	protein_coding	33,17	0,0000000
ENSG00000139618	BRCA2	13	32315474	32400266	protein_coding	32,97	0,0012807
ENSG00000181215	C4orf50	4	5897591	5989818	protein_coding	32,58	0,0025348
ENSG00000180785	OR51E1	11	4643420	4655488	protein_coding	32,12	0,0012807

ENSG00000144550	CPNE9	3	9703807	9729908	protein_coding	32,02	0,0000000
ENSG00000165023	DIRAS2	9	90609832	90643104	protein_coding	32,00	0,0050271
ENSG00000082516	GEMIN5	5	154887416	154938209	protein_coding	31,86	0,0000000
ENSG00000131153	GINS2	16	85676198	85690073	protein_coding	31,85	0,0000000
ENSG00000184305	CCSER1	4	90127535	91601913	protein_coding	31,73	0,0000000
ENSG00000170289	CNGB3	8	86553977	86743675	protein_coding	31,64	0,0335607
ENSG00000249751	ECSCR	5	139448556	139462743	protein_coding	31,64	0,0012807
ENSG00000143473	KCNH1	1	210678315	211134115	protein_coding	31,62	0,0000000
ENSG00000178999	AURKB	17	8204733	8210600	protein_coding	31,51	0,0000000
ENSG00000154027	AK5	1	77282051	77559969	protein_coding	31,48	0,0000000
ENSG00000131730	CKMT2	5	81233285	81266397	protein_coding	31,41	0,0000000
ENSG00000183378	OVCH2	11	7689438	7706421	protein_coding	31,40	0,0006696
ENSG00000170775	GPR37	7	124745997	124765627	protein_coding	31,37	0,0000000
ENSG00000088992	TESC	12	117038923	117099479	protein_coding	31,29	0,0000000
ENSG00000171320	ESCO2	8	27771949	27812640	protein_coding	30,98	0,0000000
ENSG00000176092	AIM1L	1	26321859	26354130	protein_coding	30,82	0,0050271
ENSG00000160145	KALRN	3	124080023	124726325	protein_coding	30,56	0,0000000
ENSG00000147246	HTR2C	X	114584078	114910061	protein_coding	30,55	0,0012807
ENSG00000132297	HHLA1	8	132061486	132111159	protein_coding	30,50	0,0006696
ENSG00000142025	DMRTC2	19	41844743	41852333	protein_coding	30,40	0,0006696
ENSG00000150394	CDH8	16	61647242	62037035	protein_coding	30,34	0,0012807
ENSG00000188487	INSC	11	15112424	15247208	protein_coding	30,27	0,0000000
ENSG00000134690	CDCA8	1	37692418	37709719	protein_coding	30,16	0,0000000
ENSG00000118322	ATP10B	5	160563120	160852214	protein_coding	30,10	0,0006696
ENSG00000197430	OPALIN	10	96343216	96359365	protein_coding	30,08	0,0012807
ENSG00000158825	CDA	1	20588948	20618908	protein_coding	30,00	0,0000000
ENSG00000177570	SAMD12	8	118189459	118621995	protein_coding	29,99	0,0000000
ENSG00000129810	SGOL1	3	20160593	20186292	protein_coding	29,94	0,0000000
ENSG00000146574	CCZ1B	7	6794134	6826770	protein_coding	29,92	0,0000000
ENSG00000176273	SLC35G1	10	93893973	93956062	protein_coding	29,91	0,0000000
ENSG00000043355	ZIC2	13	99981772	99986773	protein_coding	29,90	0,0000000
ENSG00000186795	KCNK18	10	117197489	117210299	protein_coding	29,70	0,0012807
ENSG00000204531	POU5F1	6	31164337	31180731	protein_coding	29,70	0,0000000
ENSG00000226650	KIF4B	5	155013755	155018132	protein_coding	29,68	0,0012807
ENSG00000277586	NEFL	8	24950955	24957110	protein_coding	29,50	0,0012807
ENSG00000155465	SLC7A7	14	22773222	22829820	protein_coding	29,46	0,0000000
ENSG00000120675	DNAJC15	13	43023203	43114224	protein_coding	29,43	0,0000000
ENSG00000134058	CDK7	5	69234795	69277430	protein_coding	29,35	0,0000000
ENSG00000186871	ERCC6L	X	72204657	72239047	protein_coding	29,34	0,0111464
ENSG00000168546	GFRA2	8	21690403	21812357	protein_coding	29,23	0,0000000
ENSG00000274286	ADRA2B	2	96112875	96116245	protein_coding	29,22	0,0012807
ENSG00000107672	NSMCE4A	10	121957088	121975217	protein_coding	29,19	0,0000000
ENSG00000143476	DTL	1	212035553	212107400	protein_coding	28,88	0,0000000
ENSG00000119888	EPCAM	2	47345158	47387601	protein_coding	28,82	0,0000000
ENSG00000140968	IRF8	16	85898803	85922609	protein_coding	28,75	0,0000000
ENSG00000163808	KIF15	3	44761717	44873376	protein_coding	28,63	0,0000000
ENSG00000111404	RERGL	12	18080869	18320107	protein_coding	28,60	0,0006696
ENSG00000140025	EFCAB11	14	89794669	89954777	protein_coding	28,55	0,0000000
ENSG00000187151	ANGPTL5	11	101890674	101916522	protein_coding	28,50	0,0000000
ENSG00000168672	FAM84B	8	126552442	126558393	protein_coding	28,39	0,0000000
ENSG00000115468	EFHD1	2	232606057	232682781	protein_coding	28,39	0,0000000
ENSG00000204740	MALRD1	10	19048771	19790401	protein_coding	28,17	0,0000000
ENSG00000151365	THRSP	11	78063861	78068351	protein_coding	28,06	0,0012807
ENSG00000174607	UGT8	4	114598455	114678224	protein_coding	28,04	0,0000000
ENSG00000176714	CCDC121	2	27625639	27629012	protein_coding	27,99	0,0000000

ENSG00000163002	NUP35	2	183117513	183161680	protein_coding	27,63	0,0000000
ENSG00000198542	ITGBL1	13	101452593	101720856	protein_coding	27,56	0,0000000
ENSG00000173258	ZNF483	9	111525159	111577844	protein_coding	27,36	0,0184474
ENSG00000168772	CXXC4	4	104468312	104494901	protein_coding	27,36	0,0000000
ENSG00000021488	SLC7A9	19	32830509	32869766	protein_coding	27,14	0,0000000
ENSG00000160191	PDE9A	21	42653636	42775509	protein_coding	27,12	0,0012807
ENSG00000172572	PDE3A	12	20369245	20684381	protein_coding	27,09	0,0000000
ENSG00000187416	LHFPL3	7	104328656	104907232	protein_coding	26,69	0,0012807
ENSG00000188580	NKAIN2	6	123804141	124825657	protein_coding	26,69	0,0012807
ENSG00000172159	FRMD3	9	83242990	83538546	protein_coding	26,57	0,0012807
ENSG00000150551	LYPD1	2	132644853	132671579	protein_coding	26,56	0,0000000
ENSG00000103534	TMC5	16	19410496	19499113	protein_coding	26,53	0,0000000
ENSG00000145703	IQGAP2	5	76403249	76708132	protein_coding	26,47	0,0000000
ENSG00000174015	SPERT	13	45702311	45714559	protein_coding	26,40	0,0012807
ENSG00000176160	HSF5	17	58420167	58488384	protein_coding	26,37	0,0000000
ENSG00000188779	SKOR1	15	67819704	67834561	protein_coding	26,23	0,0000000
ENSG00000139973	SYT16	14	61995823	62112550	protein_coding	26,16	0,0000000
ENSG00000189057	FAM111B	11	59107185	59127410	protein_coding	26,06	0,0111464
ENSG00000138640	FAM13A	4	88725955	89111398	protein_coding	26,05	0,0000000
ENSG00000239887	C1orf226	1	162366908	162386818	protein_coding	25,96	0,0000000
ENSG00000070182	SPTB	14	64746283	64879883	protein_coding	25,81	0,0000000
ENSG00000237693	IRGM	5	150846523	150900736	protein_coding	25,80	0,0000000
ENSG00000170633	RNF34	12	121400041	121430623	protein_coding	25,69	0,0000000
ENSG00000183629	GOLGA8G	15	28519611	28533014	protein_coding	25,40	0,1245284
ENSG00000070159	PTPN3	9	109375466	109498313	protein_coding	25,24	0,0000000
ENSG00000107105	ELAVL2	9	23690104	23826337	protein_coding	25,19	0,0000000
ENSG00000111247	RAD51AP1	12	4538798	4560048	protein_coding	25,14	0,0000000
ENSG00000172602	RND1	12	48857145	48865898	protein_coding	25,13	0,0062591
ENSG00000136267	DGKB	7	14145049	14974777	protein_coding	25,09	0,0000000
ENSG00000124678	TCP11	6	35118071	35148610	protein_coding	24,71	0,0000000
ENSG00000155816	FMN2	1	240014348	240475189	protein_coding	24,71	0,0000000
ENSG00000144339	TMEFF2	2	191949043	192195709	protein_coding	24,66	0,0000000
ENSG00000142945	KIF2C	1	44739818	44767767	protein_coding	24,65	0,0075075
ENSG00000119865	CNRIP1	2	68284171	68320051	protein_coding	24,62	0,0000000
ENSG00000275674	ENSG00000	15	90266317	90314499	protein_coding	24,60	0,0012807
ENSG00000280188	ENSG00000	14	71965306	71966899	protein_coding	24,60	0,0006696
ENSG00000154175	ABI3BP	3	100749156	100993515	protein_coding	24,56	0,0000000
ENSG00000112742	TTK	6	80003887	80042527	protein_coding	24,49	0,0000000
ENSG00000005421	PON1	7	95297676	95324707	protein_coding	24,45	0,0012807
ENSG00000077092	RARB	3	25174332	25597932	protein_coding	24,45	0,0000000
ENSG00000214336	FOXI3	2	88446787	88452656	protein_coding	24,33	0,0062591
ENSG00000162999	DUSP19	2	183078559	183100005	protein_coding	24,29	0,0000000
ENSG00000156575	PRG3	11	57376769	57381150	protein_coding	24,20	0,0000000
ENSG00000166450	PRTG	15	55611546	55743090	protein_coding	24,07	0,0000000
ENSG00000214511	HIGD1C	12	50953922	50970506	protein_coding	23,90	0,0111464
ENSG00000175311	ANKS4B	16	21233665	21253845	protein_coding	23,83	0,0012807
ENSG00000155269	GPR78	4	8558725	8619761	protein_coding	23,78	0,0025348
ENSG00000103316	CRYM	16	21238874	21303083	protein_coding	23,72	0,0000000
ENSG00000182175	RGMA	15	93035273	93089204	protein_coding	23,71	0,0012807
ENSG00000136982	DSCC1	8	119833976	119856010	protein_coding	23,27	0,0000000
ENSG00000175707	KDF1	1	26949562	26960406	protein_coding	23,20	0,0025348
ENSG00000141665	FBXO15	18	74073353	74147865	protein_coding	23,08	0,0000000
ENSG00000197826	C4orf22	4	80335720	80963756	protein_coding	23,08	0,0000000
ENSG00000163632	C3orf49	3	63819362	63848636	protein_coding	23,04	0,4808196
ENSG00000275004	ZNF280B	22	22484421	22509154	protein_coding	22,93	0,0000000

ENSG00000165120	SSMEM1	7	130207860	130216843	protein_coding	22,90	0,0012807
ENSG00000181965	NEUROG1	5	135534282	135535949	protein_coding	22,80	0,0418702
ENSG00000137821	LRRC49	15	70853239	71053657	protein_coding	22,78	0,0000000
ENSG00000083454	P2RX5	17	3672199	3696404	protein_coding	22,71	0,0025348
ENSG00000105289	TJP3	19	3708109	3750813	protein_coding	22,70	0,0006696
ENSG00000126583	PRKCG	19	53879190	53907652	protein_coding	22,68	0,0000000
ENSG00000182521	TBPL2	14	55413541	55456726	protein_coding	22,64	0,0012807
ENSG00000110427	KIAA1549L	11	33542072	33674102	protein_coding	22,62	0,0000000
ENSG00000187772	LIN28B	6	104957048	105083332	protein_coding	22,60	0,0012807
ENSG00000187581	COX8C	14	93347191	93348356	protein_coding	22,55	0,0012807
ENSG00000101938	CHRDL1	X	110673856	110795819	protein_coding	22,48	0,0111464
ENSG00000101951	PAGE4	X	49829260	49833973	protein_coding	22,40	0,0111464
ENSG00000154438	ASZ1	7	117363222	117428123	protein_coding	22,30	0,0006696
ENSG00000117650	NEK2	1	211658657	211675630	protein_coding	22,25	0,0075075
ENSG00000160207	HSF2BP	21	43529192	43659493	protein_coding	22,25	0,0000000
ENSG00000075188	NUP37	12	102073103	102120124	protein_coding	22,22	0,0000000
ENSG00000040275	SPDL1	5	169583634	169604778	protein_coding	22,07	0,0000000
ENSG00000234965	SHISA8	22	41909554	41914667	protein_coding	22,00	0,0000000
ENSG00000170743	SYT9	11	7238778	7469042	protein_coding	22,00	0,0000000
ENSG00000129194	SOX15	17	7588178	7590170	protein_coding	21,99	0,0000000
ENSG00000176532	PRR15	7	29563811	29567295	protein_coding	21,90	0,0000000
ENSG00000166960	CCDC178	18	32937402	33441101	protein_coding	21,77	0,0148097
ENSG00000164142	FAM160A1	4	151409216	151663632	protein_coding	21,72	0,0075075
ENSG00000197410	DCHS2	4	154232037	154491716	protein_coding	21,72	0,0000000
ENSG00000182348	ZNF804B	7	88759368	89337057	protein_coding	21,70	0,0025348
ENSG00000175806	MSRA	8	10054268	10428891	protein_coding	21,67	0,0000000
ENSG00000104499	GML	8	142834247	142916506	protein_coding	21,50	0,0006696
ENSG00000111875	ASF1A	6	118894220	118909167	protein_coding	21,42	0,0000000
ENSG00000174145	NWD2	4	37244220	37449465	protein_coding	21,42	0,0012807
ENSG00000108242	CYP2C18	10	94683621	94736190	protein_coding	21,40	0,0025348
ENSG00000183347	GBP6	1	89364058	89386461	protein_coding	21,25	0,0793786
ENSG00000138308	PLA2G12B	10	72935170	72954778	protein_coding	21,23	0,0000000
ENSG00000167670	CHAF1A	19	4402662	4445018	protein_coding	21,22	0,0037996
ENSG00000168754	FAM178B	2	96875882	96986592	protein_coding	21,20	0,0006696
ENSG00000145850	TIMD4	5	156919282	156963255	protein_coding	21,17	0,0000000
ENSG00000180090	OR3A1	17	3291635	3292600	protein_coding	21,10	0,0025348
ENSG00000059145	UNKL	16	1363205	1414751	protein_coding	21,04	0,0000000
ENSG00000182667	NTM	11	131370478	132336822	protein_coding	21,00	0,0006696
ENSG00000118492	ADGB	6	146598965	146815462	protein_coding	21,00	0,0006696
ENSG00000178084	HTR3C	3	184053047	184060671	protein_coding	20,82	0,0012807
ENSG00000111863	ADTRP	6	11712054	11807046	protein_coding	20,71	0,0012807
ENSG00000184258	CDR1	X	140782405	140784871	protein_coding	20,70	0,0586426
ENSG00000188596	CFAP54	12	96489571	96875555	protein_coding	20,68	0,0012807
ENSG00000048540	LMO3	12	16548373	16610594	protein_coding	20,63	0,0000000
ENSG00000105549	THEG	19	361747	376670	protein_coding	20,60	0,0111464
ENSG00000165125	TRPV6	7	142871203	142885762	protein_coding	20,53	0,0105771
ENSG00000134317	GRHL1	2	9951693	10002277	protein_coding	20,52	0,0000000
ENSG00000275714	HIST1H3A	6	26020490	26020900	protein_coding	20,26	0,0000000
ENSG00000168826	ZBTB49	4	4290251	4321786	protein_coding	20,26	0,0025348
ENSG00000138162	TACC2	10	121989174	122254545	protein_coding	20,20	0,0000000
ENSG00000105605	CACNG7	19	53909335	53943941	protein_coding	20,17	0,0012807
ENSG00000161243	FBXO27	19	38990714	39032785	protein_coding	20,09	0,0012807
ENSG00000177602	GSG2	17	3723903	3726699	protein_coding	20,01	0,0000000
ENSG00000213231	TCL1B	14	95686417	95692643	protein_coding	20,00	0,0000000
ENSG00000170381	SEMA3E	7	83363906	83649010	protein_coding	20,00	0,0012807

ENSG00000175471	MCTP1	5	94703741	95284575	protein_coding	19,95	0,0000000
ENSG00000122547	EEPD1	7	36153149	36301543	protein_coding	19,94	0,0000000
ENSG00000152969	JAKMIP1	4	6026199	6200591	protein_coding	19,86	0,0000000
ENSG00000148019	CEP78	9	78236062	78279690	protein_coding	19,84	0,0000000
ENSG00000164626	KCNK5	6	39188973	39229450	protein_coding	19,83	0,0000000
ENSG00000183844	FAM3B	21	41304212	41357431	protein_coding	19,82	0,0012807
ENSG00000129038	LOXL1	15	73925989	73952137	protein_coding	19,78	0,0000000
ENSG00000166558	SLC38A8	16	84009667	84042636	protein_coding	19,76	0,0006696
ENSG00000122735	DNAI1	9	34457414	34520989	protein_coding	19,60	0,0012807
ENSG00000150054	MPP7	10	28050993	28334486	protein_coding	19,45	0,0000000
ENSG00000091262	ABCC6	16	16148928	16223522	protein_coding	19,40	0,0000000
ENSG00000184811	TUSC5	17	1279663	1300987	protein_coding	19,36	0,0000000
ENSG00000122584	NXPH1	7	8433955	8752963	protein_coding	19,30	0,0025348
ENSG00000184368	MAP7D2	X	20006713	20116917	protein_coding	19,28	0,0000000
ENSG00000064270	ATP2C2	16	84368527	84464187	protein_coding	19,27	0,0000000
ENSG00000212993	POU5F1B	8	127414290	127420069	protein_coding	19,25	0,0062591
ENSG00000007174	DNAH9	17	11598431	11969748	protein_coding	19,24	0,0000000
ENSG00000164045	CDC25A	3	48157146	48188402	protein_coding	19,21	0,0075075
ENSG00000186188	FFAR4	10	93566665	93604480	protein_coding	19,10	0,0025348
ENSG00000131126	TEX101	19	43401496	43418597	protein_coding	19,07	0,0000000
ENSG00000162594	IL23R	1	67166400	67259979	protein_coding	19,02	0,0000000
ENSG00000163116	STPG2	4	97184093	98143240	protein_coding	18,97	0,0012807
ENSG00000159625	DRC7	16	57694793	57731805	protein_coding	18,93	0,0000000
ENSG00000121210	KIAA0922	4	153466346	153636711	protein_coding	18,92	0,0000000
ENSG00000264424	MYH4	17	10443290	10469559	protein_coding	18,90	0,0012807
ENSG00000273706	LHX1	17	36936785	36944612	protein_coding	18,90	0,0012807
ENSG00000174776	WDR49	3	167478684	167653983	protein_coding	18,90	0,0012807
ENSG00000146755	TRIM50	7	73312539	73328082	protein_coding	18,80	0,0012807
ENSG00000185737	NRG3	10	81875314	82987179	protein_coding	18,70	0,0006696
ENSG00000144820	ADGRG7	3	100609589	100695479	protein_coding	18,70	0,0111464
ENSG00000157404	KIT	4	54657918	54740715	protein_coding	18,69	0,0000000
ENSG00000176029	C11orf16	11	8920076	8933006	protein_coding	18,68	0,0000000
ENSG00000042088	TDP1	14	89954939	90044768	protein_coding	18,61	0,0000000
ENSG00000153930	ANKFN1	17	56110958	56511659	protein_coding	18,56	0,0012807
ENSG00000152377	SPOCK1	5	136975298	137598379	protein_coding	18,51	0,0000000
ENSG00000198901	PRC1	15	90966038	90995629	protein_coding	18,47	0,0000000
ENSG00000101144	BMP7	20	57168748	57266629	protein_coding	18,46	0,0012807
ENSG00000213185	FAM24B	10	122849078	122879641	protein_coding	18,44	0,0012807
ENSG00000130957	FBP2	9	94558720	94593793	protein_coding	18,40	0,0025348
ENSG00000175193	PARL	3	183829385	183884933	protein_coding	18,36	0,0000000
ENSG00000110675	ELMOD1	11	107591091	107666779	protein_coding	18,34	0,0000000
ENSG00000121005	CRISPLD1	8	74984515	75034558	protein_coding	18,34	0,0000000
ENSG00000106013	ANKRD7	7	118214669	118496171	protein_coding	18,33	0,0075075
ENSG00000158055	GRHL3	1	24319322	24364482	protein_coding	18,31	0,0000000
ENSG00000155890	TRIM42	3	140678039	140701150	protein_coding	18,26	0,0012807
ENSG00000155886	SLC24A2	9	19507452	19786928	protein_coding	18,19	0,0012807
ENSG00000035499	DEPDC1B	5	60596912	60700190	protein_coding	18,12	0,0000000
ENSG00000006377	DLX6	7	97005548	97011039	protein_coding	18,07	0,0025348
ENSG00000159055	MIS18A	21	32268219	32279069	protein_coding	17,98	0,0000000
ENSG00000168079	SCARA5	8	27869882	27992727	protein_coding	17,97	0,0000000
ENSG00000145365	TIFA	4	112274542	112285903	protein_coding	17,88	0,0000000
ENSG00000179520	SLC17A8	12	100357079	100422059	protein_coding	17,83	0,0012807
ENSG00000146410	MTFR2	6	136231024	136250335	protein_coding	17,81	0,0037996
ENSG00000161609	CCDC155	19	49388218	49417994	protein_coding	17,80	0,0012807
ENSG00000101850	GPR143	X	9725346	9786297	protein_coding	17,66	0,0000000

ENSG00000144362	PHOSPHO2	2	169694454	169701708	protein_coding	17,64	0,0012807
ENSG00000168952	STXBP6	14	24809656	25050297	protein_coding	17,56	0,0000000
ENSG00000175262	C1orf127	1	10946471	10982037	protein_coding	17,55	0,0012807
ENSG00000169306	IL1RAPL1	X	28587399	29956723	protein_coding	17,51	0,0000000
ENSG00000145386	CCNA2	4	121816444	121823933	protein_coding	17,51	0,0000000
ENSG00000091128	LAMB4	7	108023548	108130356	protein_coding	17,47	0,0000000
ENSG00000165084	C8orf34	8	68330722	68819022	protein_coding	17,45	0,0000000
ENSG00000183032	SLC25A21	14	36677921	37172866	protein_coding	17,40	0,0006696
ENSG00000075218	GTSE1	22	46296741	46330810	protein_coding	17,33	0,0012807
ENSG00000086570	FAT2	5	151504093	151568944	protein_coding	17,32	0,0000000
ENSG00000145839	IL9	5	135892246	135895827	protein_coding	17,30	0,0025348
ENSG00000177275	OR2AJ1	1	247933769	247934755	protein_coding	17,30	0,0418702
ENSG00000095981	KCNK16	6	39314698	39322968	protein_coding	17,20	0,0012807
ENSG00000198929	NOS1AP	1	162069774	162370475	protein_coding	17,18	0,0148097
ENSG00000134873	CLDN10	13	95433604	95579759	protein_coding	16,86	0,0000000
ENSG00000198028	ZNF560	19	9466507	9498607	protein_coding	16,86	0,0000000
ENSG00000186314	PRELID2	5	145471799	145835369	protein_coding	16,84	0,0000000
ENSG00000164440	TXLNB	6	139240061	139292139	protein_coding	16,82	0,0000000
ENSG00000149488	TMC2	20	2536607	2641784	protein_coding	16,80	0,0000000
ENSG00000166426	CRABP1	15	78340324	78348230	protein_coding	16,76	0,0000000
ENSG00000153779	TGIF2LX	X	89921882	89922883	protein_coding	16,70	0,0012807
ENSG00000123219	CENPK	5	65517766	65563171	protein_coding	16,55	0,0000000
ENSG00000248905	FMN1	15	32765545	33194733	protein_coding	16,54	0,0000000
ENSG00000173175	ADCY5	3	123282296	123449758	protein_coding	16,54	0,0012807
ENSG00000261652	C15orf65	15	55408548	55418764	protein_coding	16,52	0,0300092
ENSG00000180044	C3orf80	3	160225636	160228213	protein_coding	16,50	0,0012807
ENSG00000091651	ORC6	16	46689643	46698394	protein_coding	16,38	0,0000000
ENSG00000134343	ANO3	11	26309599	26663288	protein_coding	16,34	0,0300092
ENSG00000197721	CR1L	1	207645113	207738416	protein_coding	16,30	0,0025348
ENSG00000129173	E2F8	11	19224063	19241620	protein_coding	16,28	0,0000000
ENSG00000066279	ASPM	1	197084128	197146694	protein_coding	16,20	0,0000000
ENSG00000163535	SGOL2	2	200510008	200583782	protein_coding	16,12	0,0000000
ENSG00000189320	FAM180A	7	135728348	135748846	protein_coding	16,09	0,0793786
ENSG00000101958	GLRA2	X	14529298	14731812	protein_coding	16,05	0,0012807
ENSG00000105357	MYH14	19	50188186	50310545	protein_coding	16,00	0,0184474
ENSG00000142920	AZIN2	1	33081104	33120530	protein_coding	15,92	0,0000000
ENSG00000197849	OR8G1	11	124249676	124250611	protein_coding	15,90	0,0012807
ENSG00000177186	OR2M7	1	248323630	248324568	protein_coding	15,90	0,0006696
ENSG00000169247	SH3TC2	5	148923639	149063163	protein_coding	15,88	0,0000000
ENSG00000125878	TCF15	20	603797	610398	protein_coding	15,81	0,0000000
ENSG00000110195	FOLR1	11	72189558	72196301	protein_coding	15,80	0,0025348
ENSG00000134533	RERG	12	15107783	15348675	protein_coding	15,76	0,0012807
ENSG00000170044	ZPLD1	3	102099244	102479841	protein_coding	15,73	0,0012807
ENSG00000105852	PON3	7	95359944	95396368	protein_coding	15,67	0,0000000
ENSG00000135476	ESPL1	12	53268299	53293643	protein_coding	15,60	0,0000000
ENSG00000187527	ATP13A5	3	193274790	193378843	protein_coding	15,55	0,0050271
ENSG00000180116	C12orf40	12	39626167	39908300	protein_coding	15,53	0,0012807
ENSG00000167014	C15orf43	15	44956702	44979229	protein_coding	15,50	0,0006696
ENSG00000175183	CSRP2	12	76858715	76879060	protein_coding	15,45	0,0000000
ENSG00000104814	MAP4K1	19	38587641	38618882	protein_coding	15,35	0,0012807
ENSG00000175305	CCNE2	8	94879770	94896678	protein_coding	15,34	0,0025348
ENSG00000159259	CHAF1B	21	36385378	36419015	protein_coding	15,28	0,0000000
ENSG00000165730	STOX1	10	68827541	68895432	protein_coding	15,25	0,0012807
ENSG00000203972	GLYATL3	6	49499958	49527047	protein_coding	15,20	0,0000000
ENSG00000228198	OR2M3	1	248203068	248204006	protein_coding	15,20	0,0012807

ENSG00000119938	PPP1R3C	10	91628442	91633054	protein_coding	15,11	0,0000000
ENSG00000180347	CCDC129	7	31514071	31658720	protein_coding	15,10	0,0006696
ENSG00000149527	PLCH2	1	2425980	2505530	protein_coding	15,07	0,0000000
ENSG00000145491	ROPN1L	5	10441524	10472029	protein_coding	15,07	0,0111464
ENSG00000237515	SHISA9	16	12901620	13240413	protein_coding	15,00	0,0012807
ENSG00000185038	MROH2A	2	233775679	233833423	protein_coding	15,00	0,0111464
ENSG00000165124	SVEP1	9	110365251	110579880	protein_coding	14,98	0,0000000
ENSG00000120685	PROSER1	13	39009866	39038076	protein_coding	14,93	0,0000000
ENSG00000183654	11-mars	5	16067139	16180762	protein_coding	14,91	0,0012807
ENSG00000074317	SNCB	5	176620084	176630556	protein_coding	14,91	0,0000000
ENSG00000015133	CCDC88C	14	91271323	91417844	protein_coding	14,82	0,0000000
ENSG00000112984	KIF20A	5	138178719	138187715	protein_coding	14,64	0,0000000
ENSG00000157087	ATP2B2	3	10324023	10708031	protein_coding	14,63	0,0012807
ENSG00000066923	STAG3	7	100177563	100221488	protein_coding	14,45	0,0000000
ENSG00000105219	CNTD2	19	40222208	40226690	protein_coding	14,43	0,0000000
ENSG00000163281	GNPDA2	4	44682200	44726595	protein_coding	14,43	0,0006696
ENSG00000156469	MTERF3	8	96239398	96261610	protein_coding	14,37	0,0000000
ENSG00000164086	DUSP7	3	52048919	52056550	protein_coding	14,36	0,0000000
ENSG00000111783	RFX4	12	106582907	106762803	protein_coding	14,36	0,0062591
ENSG00000022556	NLRP2	19	54953130	55001142	protein_coding	14,35	0,0000000
ENSG00000152580	IGSF10	3	151425384	151458709	protein_coding	14,32	0,0000000
ENSG00000167346	MMP26	11	4704927	4992429	protein_coding	14,30	0,0111464
ENSG00000138669	PRKG2	4	81087370	81215117	protein_coding	14,29	0,0025348
ENSG00000132825	PPP1R3D	20	59936668	59940297	protein_coding	14,25	0,0000000
ENSG00000169064	ZBBX	3	167240287	167381346	protein_coding	14,19	0,0062591
ENSG00000112214	FHL5	6	96562548	96616636	protein_coding	14,14	0,0012807
ENSG00000164123	C4orf45	4	158893134	159038760	protein_coding	14,12	0,0012807
ENSG00000184347	SLIT3	5	168661733	169301129	protein_coding	14,12	0,0012807
ENSG00000214107	MAGEB1	X	30243730	30252038	protein_coding	14,10	0,0025348
ENSG00000135362	PRR5L	11	36296288	36465204	protein_coding	14,05	0,0000000
ENSG00000182010	RTKN2	10	62183035	62268707	protein_coding	14,01	0,0000000
ENSG00000152910	CNTNAP4	16	76277278	76559238	protein_coding	13,94	0,0000000
ENSG00000104381	GDAP1	8	74321130	74488872	protein_coding	13,80	0,0000000
ENSG00000075043	KCNQ2	20	63400210	63472677	protein_coding	13,80	0,0025348
ENSG00000011426	ANLN	7	36389806	36453791	protein_coding	13,69	0,0000000
ENSG00000133958	UNC79	14	93333219	93707876	protein_coding	13,62	0,0000000
ENSG00000142512	SIGLEC10	19	51410021	51417803	protein_coding	13,61	0,0000000
ENSG00000187323	DCC	18	52340172	53535903	protein_coding	13,58	0,0012807
ENSG00000142973	CYP4B1	1	46757838	46819413	protein_coding	13,55	0,0050271
ENSG00000132386	SERPINF1	17	1761959	1777574	protein_coding	13,53	0,0000000
ENSG00000086506	HBQ1	16	180453	181181	protein_coding	13,53	0,0148097
ENSG00000139351	SYCP3	12	101728648	101739472	protein_coding	13,51	0,0000000
ENSG00000033122	LRRC7	1	69568398	70151945	protein_coding	13,45	0,0000000
ENSG00000280148	ENSG00000	6	143857318	143938471	protein_coding	13,40	0,0111464
ENSG00000173890	GPR160	3	170037929	170085403	protein_coding	13,34	0,0012807
ENSG00000153347	FAM81B	5	95391344	95450454	protein_coding	13,25	0,0037996
ENSG00000214050	FBXO16	8	28348287	28490318	protein_coding	13,21	0,0000000
ENSG00000101695	RNF125	18	32018372	32073213	protein_coding	13,19	0,0006696
ENSG00000172239	PAIP1	5	43526267	43557758	protein_coding	13,19	0,0000000
ENSG00000054282	SDCCAG8	1	243256034	243500092	protein_coding	13,19	0,0000000
ENSG00000205358	MT1H	16	56669814	56671129	protein_coding	13,16	0,0062591
ENSG00000163449	TMEM169	2	216081866	216102783	protein_coding	13,16	0,0012807
ENSG00000136943	CTSV	9	97029679	97039643	protein_coding	13,11	0,0000000
ENSG00000182698	RESP18	2	219327409	219333177	protein_coding	13,10	0,0418702
ENSG00000183831	ANKRD45	1	173609561	173669862	protein_coding	13,02	0,0012807

ENSG00000176225	RTTN	18	70003793	70205945	protein_coding	13,02	0,0000000
ENSG00000109674	NEIL3	4	177309836	177362943	protein_coding	13,01	0,0000000
ENSG00000115194	SLC30A3	2	27253684	27275817	protein_coding	12,99	0,0012807
ENSG00000135127	CCDC64	12	119989869	120094494	protein_coding	12,94	0,0000000
ENSG00000174016	FAM46D	X	80335504	80445311	protein_coding	12,93	0,0012807
ENSG00000135218	CD36	7	80369575	80679277	protein_coding	12,90	0,0000000
ENSG00000187672	ERC2	3	55508308	56468363	protein_coding	12,88	0,0000000
ENSG00000164451	FAM26D	6	116529013	116558868	protein_coding	12,86	0,1775672
ENSG00000154479	CCDC173	2	169645425	169694433	protein_coding	12,81	0,0062591
ENSG00000064692	SNCAIP	5	122311354	122464219	protein_coding	12,79	0,0025348
ENSG00000113231	PDE8B	5	77210449	77429807	protein_coding	12,73	0,0012807
ENSG00000175866	BAIAP2	17	81035122	81117432	protein_coding	12,72	0,0000000
ENSG00000263715	ENSG00000	17	45620344	45835826	protein_coding	12,70	0,0012807
ENSG00000189350	FAM179A	2	28956611	29061373	protein_coding	12,70	0,0000000
ENSG00000150361	KLHL1	13	69700594	70108493	protein_coding	12,67	0,0012807
ENSG00000164379	FOXQ1	6	1312473	1314187	protein_coding	12,64	0,0012807
ENSG00000181690	PLAG1	8	56160904	56211324	protein_coding	12,62	0,0000000
ENSG00000166569	CPLX4	18	59275156	59318649	protein_coding	12,58	0,0335607
ENSG00000080644	CHRNA3	15	78593052	78621295	protein_coding	12,43	0,0000000
ENSG00000109832	DDX25	11	125903376	125928829	protein_coding	12,41	0,0012807
ENSG00000106018	VIPR2	7	159028175	159144957	protein_coding	12,38	0,0335607
ENSG00000174514	MFSD4	1	205568885	205602918	protein_coding	12,38	0,0006696
ENSG00000124140	SLC12A5	20	46021690	46060152	protein_coding	12,35	0,0000000
ENSG00000187950	OVCH1	12	29412474	29497686	protein_coding	12,28	0,0075075
ENSG00000155719	OTOA	16	21678514	21760729	protein_coding	12,27	0,0000000
ENSG00000185352	HS6ST3	13	96090839	96839562	protein_coding	12,27	0,0012807
ENSG00000178795	GDPD4	11	77216558	77301687	protein_coding	12,25	0,0050271
ENSG00000165553	NGB	14	77265483	77271312	protein_coding	12,23	0,0062591
ENSG00000157851	DPYSL5	2	26847747	26950351	protein_coding	12,23	0,0335607
ENSG00000134864	GGACT	13	100530164	100589528	protein_coding	12,21	0,0000000
ENSG00000138075	ABCG5	2	43812472	43838865	protein_coding	12,21	0,0012807
ENSG00000126337	KRT36	17	41486136	41492546	protein_coding	12,20	0,0012807
ENSG00000112038	OPRM1	6	154010496	154246867	protein_coding	12,20	0,0111464
ENSG00000168903	BTNL3	5	180988845	181006727	protein_coding	12,20	0,1245284
ENSG00000111665	CDCA3	12	6844793	6852066	protein_coding	12,13	0,0000000
ENSG00000029993	HMGB3	X	150980509	150990775	protein_coding	12,12	0,0000000
ENSG00000004948	CALCR	7	93424487	93574730	protein_coding	12,10	0,6142622
ENSG00000203760	CENPW	6	126340174	126348875	protein_coding	12,08	0,0000000
ENSG00000185532	PRKG1	10	50991358	52298350	protein_coding	12,06	0,0000000
ENSG00000169635	HIC2	22	21417404	21451463	protein_coding	12,03	0,0025348
ENSG00000166387	PPFIBP2	11	7513298	7657127	protein_coding	12,01	0,0012807
ENSG00000178184	PARD6G	18	80157232	80247546	protein_coding	12,01	0,0000000
ENSG00000150656	CNDP1	18	74534440	74587212	protein_coding	12,00	0,0025348
ENSG00000136155	SCEL	13	77535674	77645263	protein_coding	12,00	0,0012807
ENSG00000037241	RPL26L1	5	172958729	172969771	protein_coding	11,95	0,0000000
ENSG00000136250	AOAH	7	36512949	36724549	protein_coding	11,94	0,0000000
ENSG00000215475	SIAH3	13	45777243	45851736	protein_coding	11,93	0,0111464
ENSG00000151962	RBM46	4	154781213	154828813	protein_coding	11,91	0,0000000
ENSG00000129422	MTUS1	8	17643795	17800917	protein_coding	11,89	0,0000000
ENSG00000241484	ARHGAP8	22	44752558	44862788	protein_coding	11,87	0,0012807
ENSG00000162849	KIF26B	1	245154985	245709431	protein_coding	11,84	0,0000000
ENSG00000248746	ACTN3	11	66546395	66563329	protein_coding	11,82	0,6003007
ENSG00000173894	CBX2	17	79778132	79787983	protein_coding	11,80	0,0000000
ENSG00000145321	GC	4	71741693	71804041	protein_coding	11,80	0,0012807
ENSG00000100749	VRK1	14	96797304	96931722	protein_coding	11,74	0,0000000

ENSG00000196511	TPK1	7	144451941	144836395	protein_coding	11,72	0,0000000
ENSG00000164627	KIF6	6	39329990	39725405	protein_coding	11,71	0,0012807
ENSG00000067715	SYT1	12	78863993	79452008	protein_coding	11,69	0,0062591
ENSG00000115289	PCGF1	2	74505043	74508580	protein_coding	11,67	0,0000000
ENSG00000155275	TRMT44	4	8436140	8493531	protein_coding	11,66	0,0000000
ENSG00000079101	CLUL1	18	596988	650334	protein_coding	11,52	0,0012807
ENSG00000157212	PAXIP1	7	154943687	155003084	protein_coding	11,50	0,0000000
ENSG00000108423	TUBD1	17	59859482	59892945	protein_coding	11,43	0,0000000
ENSG00000166971	AKTIP	16	53491040	53504411	protein_coding	11,43	0,0000000
ENSG00000135697	BCO1	16	81238448	81291142	protein_coding	11,43	0,0012807
ENSG00000205089	CCNI2	5	132747445	132754403	protein_coding	11,41	0,0012807
ENSG00000104369	JPH1	8	74234700	74321328	protein_coding	11,39	0,0012807
ENSG00000139187	KLRG1	12	8950044	9010760	protein_coding	11,38	0,0012807
ENSG00000158246	FAM46B	1	27005020	27012836	protein_coding	11,35	0,0050271
ENSG00000108852	MPP2	17	43875357	43909711	protein_coding	11,32	0,0050271
ENSG00000180287	PLD5	1	242082986	242524696	protein_coding	11,29	0,0012807
ENSG00000157423	HYDIN	16	70807378	71230722	protein_coding	11,28	0,0012807
ENSG00000138079	SLC3A1	2	44275458	44321494	protein_coding	11,27	0,0000000
ENSG00000051341	POLQ	3	121431427	121546641	protein_coding	11,26	0,0000000
ENSG00000134874	DZIP1	13	95578202	95644703	protein_coding	11,23	0,0000000
ENSG00000198846	TOX	8	58805418	59119208	protein_coding	11,20	0,0000000
ENSG00000188610	FAM72B	1	121167646	121185539	protein_coding	11,18	0,0000000
ENSG00000151575	TEX9	15	56244009	56445997	protein_coding	11,16	0,0000000
ENSG00000151136	BTBD11	12	107318413	107659642	protein_coding	11,15	0,0000000
ENSG00000157388	CACNA1D	3	53494656	53813733	protein_coding	11,12	0,0000000
ENSG00000089116	LHX5	12	113462034	113472280	protein_coding	11,10	0,0418702
ENSG00000165078	CPA6	8	67422125	67746345	protein_coding	11,10	0,0012807
ENSG00000172752	COL6A5	3	130345516	130484844	protein_coding	11,10	0,0012807
ENSG00000183631	PRR32	X	126819764	126821786	protein_coding	11,10	0,0025348
ENSG00000137947	GTF2B	1	88852932	88891944	protein_coding	11,07	0,0000000
ENSG00000133302	SLF1	5	94618347	94739436	protein_coding	11,07	0,0000000
ENSG00000121101	TEX14	17	58556678	58692055	protein_coding	11,06	0,0000000
ENSG00000135063	FAM189A2	9	69324572	69392455	protein_coding	11,03	0,0000000
ENSG00000197653	DNAH10	12	123762188	123936206	protein_coding	11,03	0,0000000
ENSG00000205212	CCDC144NL	17	20836447	20896140	protein_coding	11,00	0,0025348
ENSG00000164815	ORC5	7	104126341	104208047	protein_coding	10,99	0,0000000
ENSG00000182329	KIAA2012	2	202073255	202197862	protein_coding	10,98	0,0105771
ENSG00000144278	GALNT13	2	153871913	154453849	protein_coding	10,97	0,0025348
ENSG00000141052	MYOCD	17	12665890	12768949	protein_coding	10,96	0,0012807
ENSG00000127337	YEATS4	12	69359703	69390796	protein_coding	10,93	0,0012807
ENSG00000153044	CENPH	5	69189548	69210357	protein_coding	10,92	0,0012807
ENSG00000205409	OR52E6	11	5840928	5841952	protein_coding	10,90	0,0111464
ENSG00000100628	ASB2	14	93934153	93976791	protein_coding	10,88	0,0012807
ENSG00000137767	SQRDL	15	45631148	45691294	protein_coding	10,88	0,0006696
ENSG00000111664	GNB3	12	6839954	6847393	protein_coding	10,81	0,0012807
ENSG00000012048	BRCA1	17	43044295	43170245	protein_coding	10,80	0,0012807
ENSG00000166352	C11orf74	11	36594493	36659290	protein_coding	10,79	0,0025348
ENSG00000163624	CDS1	4	84582979	84651338	protein_coding	10,79	0,0000000
ENSG00000168925	CTRB1	16	75219000	75224924	protein_coding	10,75	0,0025348
ENSG00000065057	NTHL1	16	2039815	2047866	protein_coding	10,74	0,0012807
ENSG00000181355	OFCC1	6	9596110	10211608	protein_coding	10,70	0,0006696
ENSG00000064655	EYA2	20	46894624	47188844	protein_coding	10,68	0,0000000
ENSG00000075884	ARHGAP15	2	143091362	143768352	protein_coding	10,67	0,0012807
ENSG00000046651	OFD1	X	13734745	13769353	protein_coding	10,64	0,0418702
ENSG00000145375	SPATA5	4	122923074	123319450	protein_coding	10,61	0,0000000

ENSG00000140859	KIFC3	16	57758217	57863053	protein_coding	10,59	0,0012807
ENSG00000145506	NKD2	5	1008829	1038943	protein_coding	10,59	0,0418702
ENSG00000085117	CD82	11	44564427	44620363	protein_coding	10,55	0,0257457
ENSG00000112053	SLC26A8	6	35943514	36024868	protein_coding	10,54	0,0050271
ENSG00000144559	TAMM41	3	11790442	11846919	protein_coding	10,51	0,0000000
ENSG00000078053	AMPH	7	38383704	38631567	protein_coding	10,51	0,0012807
ENSG00000050405	LIMA1	12	50175788	50283546	protein_coding	10,50	0,0000000
ENSG00000111837	MAK	6	10762723	10838555	protein_coding	10,50	0,0000000
ENSG00000142408	CACNG8	19	53963040	53990215	protein_coding	10,50	0,0075075
ENSG00000140534	TICRR	15	89575482	89631056	protein_coding	10,44	0,0000000
ENSG00000076043	REXO2	11	114439386	114450279	protein_coding	10,42	0,0012807
ENSG00000180176	TH	11	2163929	2171877	protein_coding	10,40	0,1245284
ENSG00000225830	ERCC6	10	49455368	49539538	protein_coding	10,31	0,0000000
ENSG00000184156	KCNQ3	8	132120859	132480953	protein_coding	10,25	0,0006696
ENSG00000167311	ART5	11	3638503	3642316	protein_coding	10,20	0,0012807
ENSG00000114805	PLCH1	3	155375580	155745067	protein_coding	10,20	0,0184474
ENSG00000172935	MRGPRF	11	69004395	69013409	protein_coding	10,18	0,0012807
ENSG00000141452	C18orf8	18	23503470	23531807	protein_coding	10,18	0,0000000
ENSG00000167281	RBFOX3	17	79089345	79516148	protein_coding	10,18	0,0012807
ENSG00000135951	TSGA10	2	98997261	99154964	protein_coding	10,18	0,0000000
ENSG00000182111	ZNF716	7	57450177	57473546	protein_coding	10,17	0,0062591
ENSG00000140743	CDR2	16	22345936	22437165	protein_coding	10,14	0,0000000
ENSG00000175894	TSPEAR	21	44497892	44711580	protein_coding	10,13	0,0037996
ENSG00000166823	MESP1	15	89748661	89751310	protein_coding	10,11	0,0012807
ENSG00000139151	PLCZ1	12	18683169	18738057	protein_coding	10,10	0,1245284
ENSG00000150750	C11orf53	11	111255982	111286401	protein_coding	10,05	0,2600087
ENSG00000164941	INTS8	8	94813311	94881746	protein_coding	10,02	0,0012807
ENSG00000115425	PECR	2	215996329	216082955	protein_coding	9,99	0,0000000
ENSG00000133640	LRRIQ1	12	85036314	85263224	protein_coding	9,97	0,0075075
ENSG00000175449	RFESD	5	95646754	95684773	protein_coding	9,97	0,0012807
ENSG00000180305	WFDC10A	20	45629526	45631196	protein_coding	9,91	0,0184474
ENSG00000106648	GALNTL5	7	151956379	152019934	protein_coding	9,90	0,3269660
ENSG00000157445	CACNA2D3	3	54122547	55074557	protein_coding	9,88	0,0000000
ENSG00000070882	OSBPL3	7	24796539	24981634	protein_coding	9,87	0,0000000
ENSG00000148513	ANKRD30A	10	37125788	37384111	protein_coding	9,80	0,0418702
ENSG00000177174	OR14C36	1	248348775	248349713	protein_coding	9,80	0,1245284
ENSG00000085871	MGST2	4	139665768	139740745	protein_coding	9,73	0,0000000
ENSG00000121057	AKAP1	17	57085092	57121349	protein_coding	9,72	0,0075075
ENSG00000164270	HTR4	5	148451032	148677235	protein_coding	9,70	0,0025348
ENSG00000128594	LRRC4	7	128027071	128032107	protein_coding	9,70	0,1245284
ENSG00000103056	SMPD3	16	68358325	68448688	protein_coding	9,64	0,0012807
ENSG00000162755	KLHDC9	1	161098361	161100346	protein_coding	9,62	0,0012807
ENSG00000164162	ANAPC10	4	144967112	145098541	protein_coding	9,60	0,0050271
ENSG00000143355	LHX9	1	197911902	197935478	protein_coding	9,60	0,0012807
ENSG00000147262	GPR119	X	130384440	130385447	protein_coding	9,60	0,0418702
ENSG00000143507	DUSP10	1	221701424	221742176	protein_coding	9,60	0,0012807
ENSG00000127423	AUNIP	1	25831913	25859458	protein_coding	9,56	0,0000000
ENSG00000145700	ANKRD31	5	75068275	75236878	protein_coding	9,53	0,0012807
ENSG00000178997	EXD1	15	41182725	41230743	protein_coding	9,53	0,0000000
ENSG00000042062	FAM65C	20	50586108	50691528	protein_coding	9,48	0,0000000
ENSG00000151725	CENPU	4	184694618	184734133	protein_coding	9,46	0,0000000
ENSG00000206043	C18orf63	18	74315813	74359187	protein_coding	9,45	0,2229774
ENSG00000177675	CD163L1	12	7346685	7479897	protein_coding	9,42	0,0012807
ENSG00000167550	RHEBL1	12	49064685	49070025	protein_coding	9,42	0,0012807
ENSG00000244020	MT1HL1	1	237004103	237004418	protein_coding	9,36	0,0062591

ENSG00000135423	GLS2	12	56470944	56488414	protein_coding	9,36	0,0012807
ENSG00000148200	NR6A1	9	124517275	124771310	protein_coding	9,35	0,0000000
ENSG00000164430	MB21D1	6	73413515	73452276	protein_coding	9,31	0,0000000
ENSG00000138182	KIF20B	10	89701610	89774939	protein_coding	9,29	0,0000000
ENSG00000129048	ACKR4	3	132597237	132618967	protein_coding	9,28	0,0012807
ENSG00000111799	COL12A1	6	75084326	75206051	protein_coding	9,26	0,0000000
ENSG00000182459	TEX19	17	82359247	82363776	protein_coding	9,25	0,0418702
ENSG00000137501	SYTL2	11	85694224	85811159	protein_coding	9,25	0,0000000
ENSG00000173578	XCR1	3	46017024	46027742	protein_coding	9,24	0,0050271
ENSG00000171804	WDR87	19	37884823	37906677	protein_coding	9,23	0,0050271
ENSG00000160801	PTH1R	3	46877746	46903799	protein_coding	9,23	0,0012807
ENSG00000038295	TLL1	4	165873258	166103895	protein_coding	9,19	0,0000000
ENSG00000130167	TSPAN16	19	11296139	11326996	protein_coding	9,17	0,0062591
ENSG00000122543	OCM	7	5879827	5886362	protein_coding	9,17	0,2600087
ENSG00000214814	FER1L6	8	123851987	124120061	protein_coding	9,16	0,0025348
ENSG00000134709	HOOK1	1	59814786	59876378	protein_coding	9,15	0,0000000
ENSG00000186710	CFAP73	12	113149858	113159276	protein_coding	9,15	0,0012807
ENSG00000174469	CNTNAP2	7	146116002	148420998	protein_coding	9,14	0,0000000
ENSG00000142405	NLRP12	19	53793603	53824394	protein_coding	9,12	0,0012807
ENSG00000129007	CALML4	15	68190705	68206110	protein_coding	9,11	0,0538744
ENSG00000177103	DSCAML1	11	117427773	117817525	protein_coding	9,11	0,0050271
ENSG00000100354	TNRC6B	22	40044817	40335808	protein_coding	9,09	0,0538744
ENSG00000123473	STIL	1	47250139	47314147	protein_coding	9,06	0,0000000
ENSG00000197734	C14orf178	14	77760830	77769742	protein_coding	9,05	0,0609506
ENSG00000077327	SPAG6	10	22345445	22454224	protein_coding	9,04	0,0000000
ENSG00000172673	THEMIS	6	127708072	127918631	protein_coding	9,03	0,0000000
ENSG00000115297	TLX2	2	74513463	74517147	protein_coding	9,00	0,0025348
ENSG00000075275	CELSR1	22	46360834	46537170	protein_coding	8,99	0,0000000
ENSG00000172000	ZNF556	19	2867335	2883445	protein_coding	8,98	0,0075075
ENSG00000148835	TAF5	10	103367967	103389065	protein_coding	8,96	0,0012807
ENSG00000183690	EFHC2	X	44147882	44343672	protein_coding	8,96	0,0000000
ENSG00000150628	SPATA4	4	176184638	176195671	protein_coding	8,94	0,0000000
ENSG00000145220	LYAR	4	4267701	4290169	protein_coding	8,92	0,0012807
ENSG00000145934	TENM2	5	167284799	168264157	protein_coding	8,92	0,0062591
ENSG00000140876	NUDT7	16	77722492	77742260	protein_coding	8,92	0,0012807
ENSG00000189252	SPANXN3	X	143508735	143517475	protein_coding	8,90	0,0025348
ENSG00000140367	UBE2Q2	15	75843285	75901078	protein_coding	8,90	0,0006696
ENSG00000105929	ATP6V0A4	7	138706295	138799560	protein_coding	8,85	0,0062591
ENSG00000168062	BATF2	11	64987943	64997045	protein_coding	8,83	0,0012807
ENSG00000257743	MGAM2	7	142111749	142222324	protein_coding	8,83	0,0586426
ENSG00000102452	NALCN	13	101053776	101416492	protein_coding	8,82	0,0000000
ENSG00000173702	MUC13	3	124905442	124953819	protein_coding	8,82	0,0717418
ENSG00000174567	GOLT1A	1	204198160	204214092	protein_coding	8,82	0,0184474
ENSG00000124019	FAM124B	2	224378698	224402085	protein_coding	8,81	0,0012807
ENSG00000036448	MYOM2	8	2045040	2165552	protein_coding	8,80	0,0111464
ENSG00000151789	ZNF385D	3	21412222	22373321	protein_coding	8,75	0,0006696
ENSG00000164929	BAALC	8	103140710	103230305	protein_coding	8,75	0,0000000
ENSG00000169567	HINT1	5	131159027	131171735	protein_coding	8,71	0,0000000
ENSG00000113302	IL12B	5	159314783	159330887	protein_coding	8,70	0,0012807
ENSG00000163132	MSX1	4	4859666	4863936	protein_coding	8,70	0,0012807
ENSG00000150672	DLG2	11	83455012	85627922	protein_coding	8,69	0,0000000
ENSG00000134183	GNAT2	1	109603267	109619929	protein_coding	8,68	0,0012807
ENSG00000005075	POLR2J	7	102473118	102478907	protein_coding	8,66	0,0012807
ENSG00000148948	LRRC4C	11	40114203	41459773	protein_coding	8,64	0,0717418
ENSG00000162643	WDR63	1	84999147	85133138	protein_coding	8,61	0,0172684

ENSG00000167210	LOXHD1	18	46476972	46657033	protein_coding	8,60	0,0012807
ENSG00000197971	MBP	18	76978827	77133683	protein_coding	8,57	0,0000000
ENSG00000169933	FRMPD4	X	12138466	12724523	protein_coding	8,57	0,0012807
ENSG00000142609	CFAP74	1	1921951	2003837	protein_coding	8,57	0,0025348
ENSG00000186453	FAM228A	2	24175069	24200849	protein_coding	8,56	0,0148097
ENSG00000128944	KNSTRN	15	40382721	40394246	protein_coding	8,55	0,0000000
ENSG00000124641	MED20	6	41905354	41921139	protein_coding	8,54	0,0006696
ENSG00000132749	MTL5	11	68707440	68751564	protein_coding	8,51	0,0006696
ENSG00000204952	FBXO47	17	38936432	38967402	protein_coding	8,50	0,0111464
ENSG00000188124	OR2AG2	11	6767987	6769055	protein_coding	8,50	0,0111464
ENSG00000083457	ITGAE	17	3714628	3801243	protein_coding	8,48	0,0012807
ENSG00000112312	GMNN	6	24774931	24786099	protein_coding	8,46	0,0000000
ENSG00000174928	C3orf33	3	155762617	155806351	protein_coding	8,46	0,0000000
ENSG00000189046	ALKBH2	12	109088188	109093631	protein_coding	8,45	0,0012807
ENSG00000135090	TAOK3	12	118149801	118372945	protein_coding	8,44	0,0012807
ENSG00000147419	CCDC25	8	27733311	27772653	protein_coding	8,42	0,0000000
ENSG00000231274	SBK3	19	55540656	55545543	protein_coding	8,40	0,0418702
ENSG00000167941	SOST	17	43753731	43758788	protein_coding	8,38	0,0172684
ENSG00000073111	MCM2	3	127598223	127622436	protein_coding	8,36	0,0000000
ENSG00000178752	FAM132B	2	238158982	238168900	protein_coding	8,34	0,0000000
ENSG00000151655	ITIH2	10	7703269	7749520	protein_coding	8,33	0,0012807
ENSG00000124784	RIOK1	6	7389496	7418037	protein_coding	8,32	0,0000000
ENSG00000171729	TMEM51	1	15152532	15220480	protein_coding	8,32	0,0050271
ENSG00000187720	THSD4	15	71096952	71783383	protein_coding	8,32	0,0000000
ENSG00000122550	KLHL7	7	23105758	23177914	protein_coding	8,31	0,0000000
ENSG00000186832	KRT16	17	41609778	41615899	protein_coding	8,30	0,1245284
ENSG00000007933	FMO3	1	171090877	171117819	protein_coding	8,30	0,1245284
ENSG00000113356	POLR3G	5	90471748	90514553	protein_coding	8,28	0,0000000
ENSG00000197769	MAP1LC3C	1	241995490	241999073	protein_coding	8,27	0,0418702
ENSG00000088002	SULT2B1	19	48552075	48599425	protein_coding	8,26	0,0037996
ENSG00000105499	PLA2G4C	19	48047843	48110817	protein_coding	8,25	0,0000000
ENSG00000198825	INPP5F	10	119726097	119829278	protein_coding	8,24	0,0012807
ENSG00000161040	FBXL13	7	102813230	103074843	protein_coding	8,23	0,0335607
ENSG00000169856	ONECUT1	15	52756989	52791078	protein_coding	8,22	0,0025348
ENSG00000180016	OR1E1	17	3397104	3398410	protein_coding	8,20	0,1245284
ENSG00000166596	CFAP52	17	9576627	9643459	protein_coding	8,17	0,0509539
ENSG00000103995	CEP152	15	48712928	48811146	protein_coding	8,16	0,0000000
ENSG00000171817	ZNF540	19	37551406	37614097	protein_coding	8,16	0,0012807
ENSG00000214694	ARHGEF33	2	38889880	38975449	protein_coding	8,15	0,0012807
ENSG00000122691	TWIST1	7	19020991	19117672	protein_coding	8,15	0,0000000
ENSG00000106603	COA1	7	43608456	43729717	protein_coding	8,15	0,0000000
ENSG00000167077	MEI1	22	41699499	41799456	protein_coding	8,13	0,0012807
ENSG00000182103	FAM181B	11	82729941	82733864	protein_coding	8,12	0,0012807
ENSG00000059378	PARP12	7	140023744	140063721	protein_coding	8,11	0,0000000
ENSG00000100024	UPB1	22	24494107	24528390	protein_coding	8,11	0,0257457
ENSG00000135931	ARMC9	2	231198546	231374837	protein_coding	8,10	0,0000000
ENSG00000148156	ACTL7B	9	108854589	108856967	protein_coding	8,07	0,0586426
ENSG00000107371	EXOSC3	9	37766978	37801437	protein_coding	8,01	0,0012807
ENSG00000186231	KLHL32	6	96924620	97140754	protein_coding	8,01	0,0000000
ENSG00000204086	RPA4	X	96883908	96885467	protein_coding	8,01	0,0012807
ENSG00000070190	DAPP1	4	99816833	99870154	protein_coding	8,01	0,6606414
ENSG00000144401	METTL21A	2	207580631	207625928	protein_coding	8,00	0,0000000
ENSG00000153684	GOLGA8F	15	28378621	28392018	protein_coding	8,00	0,3269660
ENSG00000139610	CELA1	12	51328443	51346679	protein_coding	8,00	0,0012807
ENSG00000187790	FANCM	14	45135940	45200890	protein_coding	7,99	0,0000000

ENSG00000096433	ITPR3	6	33620365	33696574	protein_coding	7,98	0,0000000
ENSG00000104290	FZD3	8	28494205	28574268	protein_coding	7,97	0,0000000
ENSG00000066629	EML1	14	99737693	99942060	protein_coding	7,97	0,0000000
ENSG00000148798	INA	10	103277163	103290351	protein_coding	7,96	0,0075075
ENSG00000163950	SLBP	4	1692800	1712555	protein_coding	7,96	0,0000000
ENSG00000233757	ENSG00000[illegible]	2	95207535	95259774	protein_coding	7,95	0,1245284
ENSG00000121104	FAM117A	17	49710332	49789180	protein_coding	7,92	0,0000000
ENSG00000056277	ZNF280C	X	130202711	130268899	protein_coding	7,92	0,0000000
ENSG00000112029	FBXO5	6	152970519	152983579	protein_coding	7,91	0,1245284
ENSG00000072657	TRHDE	12	72087266	72670757	protein_coding	7,91	0,0012807
ENSG00000177301	KCNA2	1	110593580	110631474	protein_coding	7,91	0,0184474
ENSG00000145808	ADAMTS19	5	129460265	129738683	protein_coding	7,90	0,6142622
ENSG00000256061	DYX1C1	15	55410525	55508234	protein_coding	7,90	0,0012807
ENSG00000168078	PBK	8	27809620	27838095	protein_coding	7,90	0,0012807
ENSG00000136014	USP44	12	95516560	95551490	protein_coding	7,89	0,0012807
ENSG00000156374	PCGF6	10	103302796	103351134	protein_coding	7,89	0,0025348
ENSG00000008083	JARID2	6	15246296	15522040	protein_coding	7,86	0,0000000
ENSG00000109458	GAB1	4	143336762	143474568	protein_coding	7,82	0,0000000
ENSG00000090932	DLL3	19	39498895	39508481	protein_coding	7,82	0,0012807
ENSG00000173610	UGT2A1	4	69588417	69653249	protein_coding	7,80	0,0418702
ENSG00000156853	ZNF689	16	30602558	30624012	protein_coding	7,75	0,1598647
ENSG00000071539	TRIP13	5	892643	919357	protein_coding	7,75	0,0000000
ENSG00000164104	HMGB2	4	173331695	173335125	protein_coding	7,73	0,0000000
ENSG00000183571	PGPEP1L	15	98968230	99007795	protein_coding	7,72	0,0184474
ENSG00000119801	YPEL5	2	30146941	30160533	protein_coding	7,71	0,0012807
ENSG00000143033	MTF2	1	93079235	93139081	protein_coding	7,71	0,0025348
ENSG00000109881	CCDC34	11	27330827	27363868	protein_coding	7,71	0,0257457
ENSG00000135346	CGA	6	87085498	87095406	protein_coding	7,70	0,0418702
ENSG00000172179	PRL	6	22287244	22297501	protein_coding	7,70	0,0111464
ENSG00000169752	NRG4	15	75935969	76059795	protein_coding	7,69	0,0000000
ENSG00000168785	TSPAN5	4	98470367	98658629	protein_coding	7,65	0,0000000
ENSG00000187537	POTEG	14	19402486	19434341	protein_coding	7,62	0,1245284
ENSG00000164362	TERT	5	1253147	1295069	protein_coding	7,60	0,0418702
ENSG00000162460	TMEM82	1	15742422	15747982	protein_coding	7,60	0,1245284
ENSG00000073350	LLGL2	17	75525080	75575208	protein_coding	7,58	0,0050271
ENSG00000180448	HMHA1	19	1065923	1086628	protein_coding	7,54	0,0000000
ENSG00000153993	SEMA3D	7	84995553	85186855	protein_coding	7,50	0,0012807
ENSG00000237452	BHMG1	19	45733251	45764534	protein_coding	7,50	0,3269660
ENSG00000198759	EGFL6	X	13569605	13633575	protein_coding	7,50	0,2229774
ENSG00000196550	FAM72A	1	206186179	206204414	protein_coding	7,48	0,0000000
ENSG00000101384	JAG1	20	10637684	10674107	protein_coding	7,48	0,0062591
ENSG00000167977	KCTD5	16	2682475	2709030	protein_coding	7,47	0,0012807
ENSG00000156697	UTP14A	X	129906121	129929761	protein_coding	7,46	0,0000000
ENSG00000188452	CERKL	2	181536676	181680665	protein_coding	7,41	0,0000000
ENSG00000136319	TTC5	14	20256558	20305994	protein_coding	7,37	0,0000000
ENSG00000178031	ADAMTSL1	9	18473894	18910950	protein_coding	7,37	0,0012807
ENSG00000144161	ZC3H8	2	112211525	112255136	protein_coding	7,36	0,0000000
ENSG00000112414	ADGRG6	6	142301854	142446266	protein_coding	7,34	0,0012807
ENSG00000127585	FBXL16	16	692498	705829	protein_coding	7,33	0,0012807
ENSG00000137807	KIF23	15	69414246	69448427	protein_coding	7,32	0,0000000
ENSG00000165480	SKA3	13	21153595	21176602	protein_coding	7,32	0,0000000
ENSG00000185306	C12orf56	12	64264762	64391192	protein_coding	7,30	0,1245284
ENSG00000183775	KCTD16	5	144170832	144485686	protein_coding	7,28	0,1867869
ENSG00000115687	PASK	2	241106099	241150264	protein_coding	7,27	0,0000000
ENSG00000164287	CDC20B	5	55112995	55173175	protein_coding	7,27	0,0717418

ENSG00000134597	RBMX2	X	130401969	130413343	protein_coding	7,27	0,0012807
ENSG00000133106	EPSTI1	13	42886388	42992271	protein_coding	7,26	0,0000000
ENSG00000106415	GLCCI1	7	7968794	8094272	protein_coding	7,26	0,0000000
ENSG00000186094	AGBL4	1	48532855	50023913	protein_coding	7,25	0,0111464
ENSG00000072571	HMMR	5	163460203	163491945	protein_coding	7,23	0,0609506
ENSG00000184990	SIVA1	14	104753100	104768494	protein_coding	7,22	0,0012807
ENSG00000164414	SLC35A1	6	87470623	87512336	protein_coding	7,22	0,6702615
ENSG00000034971	MYOC	1	171635417	171652683	protein_coding	7,21	0,0105771
ENSG00000184933	OR6A2	11	6794627	6795783	protein_coding	7,20	0,3269660
ENSG00000167487	KLHL26	19	18636965	18671714	protein_coding	7,19	0,0000000
ENSG00000166860	ZBTB39	12	56998834	57006446	protein_coding	7,19	0,0075075
ENSG00000137812	CASC5	15	40594020	40664342	protein_coding	7,17	0,0111464
ENSG00000187398	LUZP2	11	24496970	25082631	protein_coding	7,16	0,0012807
ENSG00000172301	COPRS	17	31851864	31859337	protein_coding	7,15	0,0012807
ENSG00000198691	ABCA4	1	93992835	94121132	protein_coding	7,14	0,0257457
ENSG00000198121	LPAR1	9	110873263	111038458	protein_coding	7,13	0,0025348
ENSG00000151611	MMAA	4	145618263	145660035	protein_coding	7,11	0,0006696
ENSG00000162961	DPY30	2	31867809	32039812	protein_coding	7,11	0,0025348
ENSG00000168418	KCNG4	16	84218667	84239750	protein_coding	7,10	0,1245284
ENSG00000171815	PCDHB1	5	141051135	141059344	protein_coding	7,10	0,0012807
ENSG00000157890	MEGF11	15	65895079	66253747	protein_coding	7,10	0,0012807
ENSG00000091181	IL5RA	3	3066326	3126613	protein_coding	7,07	0,0037996
ENSG00000166535	A2ML1	12	8822472	8887001	protein_coding	7,06	0,0075075
ENSG00000263155	MYZAP	15	57591941	57685364	protein_coding	7,06	0,0000000
ENSG00000217442	SYCE3	22	50551112	50562905	protein_coding	7,06	0,0012807
ENSG00000092470	WDR76	15	43826963	43868419	protein_coding	7,05	0,0000000
ENSG00000130762	ARHGEF16	1	3454426	3481113	protein_coding	7,04	0,0012807
ENSG00000268744	ENSG00000	19	12379189	12401274	protein_coding	7,00	0,0012807
ENSG00000133710	SPINK5	5	148025683	148137289	protein_coding	7,00	0,3269660
ENSG00000189023	MAGEB16	X	35798342	35803735	protein_coding	7,00	0,0418702
ENSG00000154237	LRRK1	15	100919215	101078254	protein_coding	6,99	0,0000000
ENSG00000204564	C6orf136	6	30647039	30653210	protein_coding	6,99	0,0000000
ENSG00000189227	C15orf61	15	67521068	67530143	protein_coding	6,98	0,0012807
ENSG00000158402	CDC25C	5	138285265	138338355	protein_coding	6,95	0,0111464
ENSG00000196132	MYT1	20	64151791	64242253	protein_coding	6,94	0,0184474
ENSG00000137210	TMEM14B	6	10747759	10852753	protein_coding	6,94	0,0012807
ENSG00000197299	BLM	15	90717327	90816165	protein_coding	6,93	0,0000000
ENSG00000125872	LRRN4	20	6040778	6054049	protein_coding	6,90	0,0012807
ENSG00000259120	SMIM6	17	75646243	75647977	protein_coding	6,90	0,0012807
ENSG00000170325	PRDM10	11	129899706	130002835	protein_coding	6,89	0,0000000
ENSG00000175279	APITD1	1	10430443	10442809	protein_coding	6,89	0,0418702
ENSG00000165164	CFAP47	X	35919734	36385319	protein_coding	6,88	0,0012807
ENSG00000184860	SDR42E1	16	81988855	82011488	protein_coding	6,88	0,0050271
ENSG00000112293	GPLD1	6	24424565	24495205	protein_coding	6,85	0,0300092
ENSG00000143842	SOX13	1	204073115	204127743	protein_coding	6,84	0,0000000
ENSG00000162385	MAGOH	1	53226892	53238610	protein_coding	6,82	0,0000000
ENSG00000198478	SH3BGRL2	6	79631283	79703659	protein_coding	6,81	0,0025348
ENSG00000196581	AJAP1	1	4654732	4792534	protein_coding	6,80	0,0418702
ENSG00000181938	GINS3	16	58295080	58406144	protein_coding	6,79	0,0000000
ENSG00000188611	ASAH2	10	50182778	50248610	protein_coding	6,78	0,0000000
ENSG00000186952	TMEM232	5	110289233	110738956	protein_coding	6,78	0,0012807
ENSG00000198633	ZNF534	19	52429187	52452315	protein_coding	6,77	0,0335607
ENSG00000100592	DAAM1	14	59188646	59371405	protein_coding	6,77	0,0012807
ENSG00000224689	ZNF812	19	9689924	9700817	protein_coding	6,77	0,6606414
ENSG00000157368	IL34	16	70579895	70660682	protein_coding	6,77	0,0062591

ENSG00000144962	SPATA16	3	172889358	173141268	protein_coding	6,77	0,0062591
ENSG00000165959	CLMN	14	95181940	95319906	protein_coding	6,76	0,0025348
ENSG00000185442	FAM174B	15	92617443	92809884	protein_coding	6,75	0,0000000
ENSG00000089101	CFAP61	20	20052514	20360702	protein_coding	6,75	0,0000000
ENSG00000100600	LGMN	14	92703807	92748702	protein_coding	6,74	0,0000000
ENSG00000157657	ZNF618	9	113876282	114056591	protein_coding	6,74	0,1245284
ENSG00000011332	DPF1	19	38211006	38229714	protein_coding	6,74	0,2600087
ENSG00000161944	ASGR2	17	7101322	7115700	protein_coding	6,73	0,1867869
ENSG00000187240	DYNC2H1	11	103109431	103479863	protein_coding	6,72	0,0000000
ENSG00000178381	ZFAND2A	7	1152071	1160759	protein_coding	6,70	0,0000000
ENSG00000154548	SRSF12	6	89095959	89118081	protein_coding	6,70	0,0006696
ENSG00000130347	RTN4IP1	6	106571971	106629487	protein_coding	6,69	0,0000000
ENSG00000186281	GPAT2	2	96021946	96039451	protein_coding	6,69	0,1775672
ENSG00000256525	POLG2	17	64477785	64497036	protein_coding	6,67	0,0000000
ENSG00000145526	CDH18	5	19472951	20575873	protein_coding	6,66	0,0012807
ENSG00000164758	MED30	8	117520713	117540262	protein_coding	6,66	0,0012807
ENSG00000091073	DTX2	7	76461676	76505995	protein_coding	6,66	0,0012807
ENSG00000152193	RNF219	13	78614291	78659179	protein_coding	6,65	0,0000000
ENSG00000144407	PTH2R	2	208359714	208854503	protein_coding	6,64	0,0111464
ENSG00000120332	TNN	1	175067858	175148066	protein_coding	6,64	0,0050271
ENSG00000087086	FTL	19	48965301	48966878	protein_coding	6,64	0,0000000
ENSG00000004468	CD38	4	15778275	15853230	protein_coding	6,63	0,0012807
ENSG00000180263	FGD6	12	95076749	95217482	protein_coding	6,63	0,0000000
ENSG00000142449	FBN3	19	8065402	8149846	protein_coding	6,63	0,0717418
ENSG00000166619	BLCAP	20	37492472	37527931	protein_coding	6,61	0,0000000
ENSG00000136573	BLK	8	11494001	11564604	protein_coding	6,60	0,0538744
ENSG00000146143	PRIM2	6	57314805	57646849	protein_coding	6,59	0,0000000
ENSG00000110944	IL23A	12	56334174	56340410	protein_coding	6,58	0,0012807
ENSG00000154646	TMPRSS15	21	18269116	18485879	protein_coding	6,57	0,0012807
ENSG00000114346	ECT2	3	172750682	172821474	protein_coding	6,57	0,0000000
ENSG00000180855	ZNF443	19	12429707	12441112	protein_coding	6,56	0,0012807
ENSG00000165632	TAF3	10	7818504	8016627	protein_coding	6,54	0,1245284
ENSG00000122952	ZWINT	10	56357228	56361275	protein_coding	6,54	0,0000000
ENSG00000130475	FCHO1	19	17747718	17788568	protein_coding	6,53	0,0012807
ENSG00000120647	CCDC77	12	389273	442645	protein_coding	6,52	0,0000000
ENSG00000057757	PITHD1	1	23778405	23788232	protein_coding	6,51	0,0000000
ENSG00000140374	ETFA	15	76215355	76311472	protein_coding	6,50	0,0006696
ENSG00000218819	TDRD15	2	21123917	21143272	protein_coding	6,50	0,0025348
ENSG00000004848	ARX	X	25003694	25015948	protein_coding	6,50	0,3269660
ENSG00000203952	CCDC160	X	134237047	134246207	protein_coding	6,48	0,0012807
ENSG00000134070	IRAK2	3	10164865	10243743	protein_coding	6,47	0,0000000
ENSG00000076248	UNG	12	109097574	109110992	protein_coding	6,44	0,0000000
ENSG00000115539	PDCL3	2	100562690	100576735	protein_coding	6,43	0,0012807
ENSG00000133641	C12orf29	12	88033846	88050160	protein_coding	6,42	0,0025348
ENSG00000066032	CTNNA2	2	79185231	80648861	protein_coding	6,42	0,0000000
ENSG00000150625	GPM6A	4	175632934	176002664	protein_coding	6,39	0,0012807
ENSG00000173409	ARV1	1	230978981	231000595	protein_coding	6,39	0,0075075
ENSG00000100629	CEP128	14	80476983	80959517	protein_coding	6,38	0,0000000
ENSG00000065609	SNAP91	6	83552880	83709691	protein_coding	6,36	0,0012807
ENSG00000139835	GRTP1	13	113324164	113364148	protein_coding	6,34	0,0000000
ENSG00000163508	EOMES	3	27715949	27722711	protein_coding	6,32	0,2600087
ENSG00000153140	CETN3	5	90392261	90409786	protein_coding	6,31	0,1245284
ENSG00000163249	CCNYL1	2	207711540	207761839	protein_coding	6,31	0,0012807
ENSG00000118194	TNNT2	1	201359008	201377762	protein_coding	6,31	0,0335607
ENSG00000128683	GAD1	2	170813213	170861151	protein_coding	6,31	0,0012807

ENSG00000101624	CEP76	18	12661833	12702777	protein_coding	6,29	0,0000000
ENSG00000183765	CHEK2	22	28687743	28742422	protein_coding	6,28	0,0012807
ENSG00000141469	SLC14A1	18	45724127	45752520	protein_coding	6,28	0,0012807
ENSG00000213551	DNAJC9	10	73183362	73248862	protein_coding	6,27	0,0012807
ENSG00000187862	TTC24	1	156579727	156586770	protein_coding	6,26	0,0111464
ENSG00000100330	MTMR3	22	29883155	30030866	protein_coding	6,25	0,0000000
ENSG00000105278	ZFR2	19	3804024	3869032	protein_coding	6,23	0,2127456
ENSG00000113749	HRH2	5	175658030	175686242	protein_coding	6,21	0,0012807
ENSG00000066379	ZNRD1	6	30058899	30064909	protein_coding	6,21	0,0000000
ENSG00000154040	CABYR	18	24138956	24161603	protein_coding	6,21	0,0000000
ENSG00000164532	TBX20	7	35202430	35254147	protein_coding	6,20	0,0418702
ENSG00000257365	FNTB	14	64986720	65062652	protein_coding	6,19	0,0012807
ENSG00000133863	TEX15	8	30831544	30890606	protein_coding	6,17	0,0012807
ENSG00000056291	NPFFR2	4	72031804	72148067	protein_coding	6,17	0,0995111
ENSG00000078487	ZCWPW1	7	100400826	100428992	protein_coding	6,14	0,0538744
ENSG00000127824	TUBA4A	2	219249711	219278170	protein_coding	6,14	0,0012807
ENSG00000090581	GNPTG	16	1351923	1364113	protein_coding	6,13	0,0000000
ENSG00000214491	SEC14L6	22	30522799	30546682	protein_coding	6,12	0,0012807
ENSG00000158516	CPA2	7	130266827	130289798	protein_coding	6,09	0,0012807
ENSG00000169946	ZFPM2	8	104590733	105804532	protein_coding	6,09	0,0000000
ENSG00000197124	ZNF682	19	19997058	20039506	protein_coding	6,09	0,0012807
ENSG00000106069	CHN2	7	29122274	29514328	protein_coding	6,08	0,0000000
ENSG00000164070	HSPA4L	4	127781821	127840733	protein_coding	6,08	0,0012807
ENSG00000166664	CHRFAM7A	15	30357766	30393849	protein_coding	6,07	0,0000000
ENSG00000081059	TCF7	5	134114711	134151865	protein_coding	6,06	0,0000000
ENSG00000173846	PLK3	1	44800225	44805990	protein_coding	6,05	0,0012807
ENSG00000065320	NTN1	17	9021542	9244000	protein_coding	6,04	0,0012807
ENSG00000140835	CHST4	16	71525233	71538746	protein_coding	6,04	0,0538744
ENSG00000176769	TCERG1L	10	131092391	131311721	protein_coding	6,04	0,1598647
ENSG00000072832	CRMP1	4	5748084	5893058	protein_coding	6,03	0,0000000
ENSG00000121621	KIF18A	11	28020620	28108308	protein_coding	6,03	0,0000000
ENSG00000137601	NEK1	4	169393275	169612629	protein_coding	6,03	0,0000000
ENSG00000111199	TRPV4	12	109783085	109833401	protein_coding	6,03	0,1598647
ENSG00000118402	ELOVL4	6	79914812	79947580	protein_coding	6,02	0,0012807
ENSG00000169894	MUC3A	7	100949555	100968346	protein_coding	6,02	0,0006696
ENSG00000186732	MPPED1	22	43411196	43507848	protein_coding	6,00	0,0025348
ENSG00000140798	ABCC12	16	48082973	48156018	protein_coding	6,00	0,0111464
ENSG00000156206	CFAP161	15	81007033	81149175	protein_coding	6,00	0,0062591
ENSG00000105996	HOXA2	7	27100354	27102811	protein_coding	6,00	0,0062591
ENSG00000186352	ANKRD37	4	185396021	185400628	protein_coding	6,00	0,0000000
ENSG00000063127	SLC6A16	19	49289638	49325225	protein_coding	5,99	0,0000000
ENSG00000171365	CLCN5	X	49922615	50099235	protein_coding	5,99	0,0111464
ENSG00000117477	CCDC181	1	169394870	169460669	protein_coding	5,98	0,0062591
ENSG00000118197	DDX59	1	200623896	200669969	protein_coding	5,98	0,0012807
ENSG00000176371	ZSCAN2	15	84600986	84627796	protein_coding	5,97	0,0000000
ENSG00000186153	WWOX	16	78099413	79212667	protein_coding	5,96	0,0075075
ENSG00000163864	NMNAT3	3	139560180	139678017	protein_coding	5,95	0,0000000
ENSG00000261115	TMEM178B	7	141074232	141480380	protein_coding	5,95	0,0012807
ENSG00000185947	ZNF267	16	31873758	31917357	protein_coding	5,94	0,0000000
ENSG00000111145	ELK3	12	96194382	96269835	protein_coding	5,94	0,0012807
ENSG00000149050	ZNF214	11	6999318	7020368	protein_coding	5,93	0,0000000
ENSG00000186777	ZNF732	4	270675	305321	protein_coding	5,92	0,0000000
ENSG00000184349	EFNA5	5	107376889	107670895	protein_coding	5,91	0,0037996
ENSG00000165471	MBL2	10	52765380	52771700	protein_coding	5,90	0,6142622
ENSG00000166897	ELFN2	22	37367960	37427470	protein_coding	5,89	0,0793786

ENSG00000100253	MIOX	22	50486784	50490648	protein_coding	5,88	0,0793786
ENSG00000196653	ZNF502	3	44712643	44723831	protein_coding	5,86	0,0012807
ENSG00000130176	CNN1	19	11538717	11550323	protein_coding	5,85	0,0012807
ENSG00000136928	GABBR2	9	98288082	98709197	protein_coding	5,84	0,0006696
ENSG00000174371	EXO1	1	241847967	241895148	protein_coding	5,84	0,0050271
ENSG00000104848	KCNA7	19	49067418	49072941	protein_coding	5,83	0,0793786
ENSG00000164989	CCDC171	9	15552897	16061663	protein_coding	5,83	0,0111464
ENSG00000187268	FAM9C	X	13035618	13044682	protein_coding	5,81	0,0012807
ENSG00000130545	CRB3	19	6463777	6467221	protein_coding	5,80	0,0418702
ENSG00000214216	IQCJ	3	158962928	159266307	protein_coding	5,80	0,0111464
ENSG00000003147	ICA1	7	8113184	8262687	protein_coding	5,80	0,0012807
ENSG00000184992	BRI3BP	12	124993700	125031231	protein_coding	5,79	0,0000000
ENSG00000151500	THYN1	11	134248279	134253370	protein_coding	5,77	0,0012807
ENSG00000214456	PLIN5	19	4522531	4535224	protein_coding	5,77	0,3963183
ENSG00000078246	TULP3	12	2877223	2941140	protein_coding	5,77	0,0000000
ENSG00000124143	ARHGAP40	20	38601934	38651035	protein_coding	5,77	0,0062591
ENSG00000077514	POLD3	11	74493851	74669117	protein_coding	5,76	0,0012807
ENSG00000163507	KIAA1524	3	108549869	108589644	protein_coding	5,76	0,1168222
ENSG00000167553	TUBA1C	12	49188736	49274603	protein_coding	5,74	0,0012807
ENSG00000119147	C2orf40	2	106063246	106078159	protein_coding	5,73	0,0184474
ENSG00000213123	TCTEX1D2	3	196291219	196318299	protein_coding	5,72	0,0586426
ENSG00000183960	KCNH8	3	19148454	19535646	protein_coding	5,71	0,0012807
ENSG00000100601	ALKBH1	14	77672404	77708020	protein_coding	5,70	0,0000000
ENSG00000198208	RPS6KL1	14	74903954	74923396	protein_coding	5,68	0,0025348
ENSG00000078081	LAMP3	3	183122213	183163839	protein_coding	5,68	0,0012807
ENSG00000137804	NUSAP1	15	41332694	41381050	protein_coding	5,67	0,0000000
ENSG00000119969	HELLS	10	94501434	94613905	protein_coding	5,66	0,0000000
ENSG00000114124	GRK7	3	141778148	141818490	protein_coding	5,66	0,0793786
ENSG00000120210	INSL6	9	5131979	5185668	protein_coding	5,64	0,0995111
ENSG00000113430	IRX4	5	1877413	1887236	protein_coding	5,64	0,0184474
ENSG00000221866	PLXNA4	7	132123332	132648688	protein_coding	5,64	0,0111464
ENSG00000178404	CEP295NL	17	78870910	78903217	protein_coding	5,63	0,0335607
ENSG00000104626	ERI1	8	9002147	9116746	protein_coding	5,63	0,0012807
ENSG00000137962	ARHGAP29	1	94148988	94275068	protein_coding	5,62	0,0300092
ENSG00000152359	POC5	5	75674124	75717481	protein_coding	5,62	0,0000000
ENSG00000125144	MT1G	16	56666731	56668065	protein_coding	5,62	0,0300092
ENSG00000166153	DEPDC4	12	100203669	100267079	protein_coding	5,61	0,0012807
ENSG00000182606	TRAK1	3	42013802	42225889	protein_coding	5,60	0,0000000
ENSG00000069248	NUP133	1	229440260	229508341	protein_coding	5,60	0,0012807
ENSG00000106536	POU6F2	7	38977998	39493095	protein_coding	5,59	0,0335607
ENSG00000124226	RNF114	20	49936336	49953892	protein_coding	5,58	0,0000000
ENSG00000167034	NKX3-1	8	23678693	23682927	protein_coding	5,58	0,0148097
ENSG00000266265	KLF14	7	130731235	130734061	protein_coding	5,57	0,0025348
ENSG00000144152	FBLN7	2	112138385	112188216	protein_coding	5,57	0,0025348
ENSG00000186976	EFCAB6	22	43528744	43812337	protein_coding	5,56	0,0006696
ENSG00000122507	BBS9	7	33129244	33606068	protein_coding	5,55	0,0012807
ENSG00000117595	IRF6	1	209785623	209806175	protein_coding	5,55	0,0000000
ENSG00000168297	PXK	3	58332880	58426126	protein_coding	5,55	0,0793786
ENSG00000160282	FTCD	21	46136262	46155567	protein_coding	5,54	0,0538744
ENSG00000084453	SLCO1A2	12	21264600	21419594	protein_coding	5,54	0,0037996
ENSG00000129028	THAP10	15	70881342	70892785	protein_coding	5,53	0,0000000
ENSG00000177042	TMEM80	11	695428	705028	protein_coding	5,53	0,0006696
ENSG00000158480	SPATA2	20	49903391	49915508	protein_coding	5,52	0,0000000
ENSG00000111845	PAK1IP1	6	10694695	10709782	protein_coding	5,51	0,0025348
ENSG00000155849	ELMO1	7	36854361	37449249	protein_coding	5,51	0,0000000

ENSG00000181963	OR52K2	11	4449295	4450361	protein_coding	5,50	0,1245284
ENSG00000140386	SCAPER	15	76347904	76905444	protein_coding	5,49	0,0062591
ENSG00000132554	RGS22	8	99960936	100131268	protein_coding	5,49	0,0075075
ENSG00000120696	KBTBD7	13	41189833	41194566	protein_coding	5,46	0,0000000
ENSG00000167797	CDK2AP2	11	67506497	67508649	protein_coding	5,46	0,0000000
ENSG00000140297	GCNT3	15	59594875	59640239	protein_coding	5,46	0,0025348
ENSG00000076554	TPD52	8	80034745	80231232	protein_coding	5,45	0,0172684
ENSG00000188312	CENPP	9	92325484	92620533	protein_coding	5,45	0,0012807
ENSG00000115232	ITGA4	2	181457202	181536187	protein_coding	5,45	0,0012807
ENSG00000122966	CIT	12	119685790	119877291	protein_coding	5,44	0,0000000
ENSG00000135211	TMEM60	7	77793728	77798580	protein_coding	5,44	0,0037996
ENSG00000113396	SLC27A6	5	128538013	129033642	protein_coding	5,44	0,0000000
ENSG00000151623	NR3C2	4	148078762	148444698	protein_coding	5,43	0,0012807
ENSG00000088305	DNMT3B	20	32762385	32809356	protein_coding	5,43	0,0000000
ENSG00000159079	C21orf59	21	32592079	32612866	protein_coding	5,42	0,0050271
ENSG00000131941	RHPN2	19	32978593	33064888	protein_coding	5,42	0,0012807
ENSG00000180767	CHST13	3	126524283	126543291	protein_coding	5,41	0,0148097
ENSG00000197275	RAD54B	8	94371960	94475109	protein_coding	5,41	0,0012807
ENSG00000143106	PSMA5	1	109399031	109426427	protein_coding	5,40	0,0012807
ENSG00000104044	OCA2	15	27754875	28099358	protein_coding	5,39	0,0012807
ENSG00000140527	WDR93	15	89690797	89743638	protein_coding	5,39	0,5364729
ENSG00000196071	OR2L13	1	247937191	248100922	protein_coding	5,39	0,0148097
ENSG00000163793	DNAJC5G	2	27275421	27281499	protein_coding	5,38	0,0148097
ENSG00000187288	CIDEC	3	9866711	9880254	protein_coding	5,35	0,2600087
ENSG00000168263	KCNV2	9	2717502	2730037	protein_coding	5,34	0,0586426
ENSG00000104611	SH2D4A	8	19313617	19396218	protein_coding	5,34	0,0000000
ENSG00000185736	ADARB2	10	1177318	1737476	protein_coding	5,33	0,2127456
ENSG00000184838	PRR16	5	120464278	120687332	protein_coding	5,33	0,0012807
ENSG00000081479	LRP2	2	169127109	169362685	protein_coding	5,33	0,1245284
ENSG00000161381	PLXDC1	17	39063303	39154394	protein_coding	5,32	0,0025348
ENSG00000131437	KIF3A	5	132692628	132737638	protein_coding	5,32	0,0025348
ENSG00000114854	TNNC1	3	52451102	52454070	protein_coding	5,30	0,0418702
ENSG00000159173	TNNI1	1	201403768	201429866	protein_coding	5,30	0,1245284
ENSG00000273274	ZBTB8B	1	32465069	32496686	protein_coding	5,29	0,0012807
ENSG00000180611	MB21D2	3	192796815	192918161	protein_coding	5,28	0,0012807
ENSG00000118193	KIF14	1	200551497	200620734	protein_coding	5,27	0,0012807
ENSG00000036530	CYP46A1	14	99684304	99727301	protein_coding	5,26	0,0012807
ENSG00000187726	DNAJB13	11	73950319	73970366	protein_coding	5,26	0,0050271
ENSG00000166407	LMO1	11	8224304	8268716	protein_coding	5,26	0,0012807
ENSG00000095002	MSH2	2	47402969	47562311	protein_coding	5,25	0,0000000
ENSG00000185798	WDR53	3	196554177	196568674	protein_coding	5,24	0,0012807
ENSG00000129993	CBFA2T3	16	88874858	88977204	protein_coding	5,24	0,0418702
ENSG00000198650	TAT	16	71565660	71577130	protein_coding	5,24	0,0037996
ENSG00000178568	ERBB4	2	211375717	212538841	protein_coding	5,23	0,0000000
ENSG00000146263	MMS22L	6	97142161	97283217	protein_coding	5,23	0,0000000
ENSG00000135040	NAA35	9	85941146	86022298	protein_coding	5,22	0,0012807
ENSG00000131018	SYNE1	6	152121684	152637801	protein_coding	5,20	0,0000000
ENSG00000165097	KDM1B	6	18155329	18223853	protein_coding	5,18	0,0012807
ENSG00000175344	CHRNA7	15	32030488	32172521	protein_coding	5,18	0,0012807
ENSG00000213066	FGFR1OP	6	166999182	167052713	protein_coding	5,17	0,0012807
ENSG00000100351	GRAP2	22	39901082	39973721	protein_coding	5,14	0,2725752
ENSG00000136842	TMOD1	9	97501180	97601748	protein_coding	5,11	0,0012807
ENSG00000143633	C1orf131	1	231223763	231241187	protein_coding	5,11	0,0012807
ENSG00000167703	SLC43A2	17	1569267	1628886	protein_coding	5,11	0,0012807
ENSG00000197006	METTL9	16	21597218	21657473	protein_coding	5,10	0,0012807

ENSG00000104442	ARMC1	8	65602456	65634217	protein_coding	5,10	0,0000000
ENSG00000196169	KIF19	17	74326210	74355820	protein_coding	5,10	0,1245284
ENSG00000214215	C12orf74	12	92702843	92708549	protein_coding	5,10	0,0418702
ENSG00000188958	UTS2B	3	191267168	191330536	protein_coding	5,10	0,2229774
ENSG00000129221	AIPL1	17	6393693	6435199	protein_coding	5,09	0,0717418
ENSG00000120656	TAF12	1	28589323	28643085	protein_coding	5,09	0,0000000
ENSG00000077063	CTTNBP2	7	117710651	117874139	protein_coding	5,09	0,0012807
ENSG00000123737	EXOSC9	4	121801317	121817021	protein_coding	5,09	0,0012807
ENSG00000146243	IRAK1BP1	6	78867472	78946440	protein_coding	5,07	0,0000000
ENSG00000198924	DCLRE1A	10	113834725	113854383	protein_coding	5,07	0,0995111
ENSG00000111834	RSPH4A	6	116616479	116632985	protein_coding	5,06	0,0012807
ENSG00000077585	GPR137B	1	236142505	236221865	protein_coding	5,06	0,0000000
ENSG00000128340	RAC2	22	37225261	37244448	protein_coding	5,06	0,0012807
ENSG00000170965	PLAC1	X	134565838	134764322	protein_coding	5,05	0,0012807
ENSG00000037042	TUBG2	17	42659305	42667006	protein_coding	5,05	0,0000000
ENSG00000125319	C17orf53	17	44141906	44162476	protein_coding	5,05	0,0012807
ENSG00000074319	TSG101	11	18468336	18527232	protein_coding	5,04	0,0006696
ENSG00000105143	SLC1A6	19	14950034	15022990	protein_coding	5,04	0,0012807
ENSG00000075914	EXOSC7	3	44975241	45036066	protein_coding	5,04	0,0000000
ENSG00000106399	RPA3	7	7636518	7718607	protein_coding	5,02	0,0335607
ENSG00000173681	CXorf23	X	19912860	19970298	protein_coding	5,00	0,0012807
ENSG00000175318	GRAMD2	15	72159807	72197785	protein_coding	5,00	0,0105771
ENSG00000181609	OR52D1	11	5488685	5489749	protein_coding	5,00	0,1245284
ENSG00000148426	PROSER2	10	11823339	11872277	protein_coding	4,99	0,0184474
ENSG00000112118	MCM3	6	52264009	52284881	protein_coding	4,98	0,0335607
ENSG00000185989	RASA3	13	113977783	114132611	protein_coding	4,98	0,0000000
ENSG00000139220	PPFIA2	12	81257975	81759553	protein_coding	4,98	0,0012807
ENSG00000148925	BTBD10	11	13388001	13463297	protein_coding	4,97	0,0012807
ENSG00000138433	CIR1	2	174348022	174395715	protein_coding	4,97	0,1867869
ENSG00000105784	RUNDC3B	7	87627548	87832296	protein_coding	4,96	0,0075075
ENSG00000139915	MDGA2	14	46839629	47674954	protein_coding	4,95	0,0012807
ENSG00000171094	ALK	2	29192774	29921566	protein_coding	4,95	0,0148097
ENSG00000170448	NFXL1	4	47847233	47914667	protein_coding	4,93	0,0012807
ENSG00000188738	FSIP2	2	185738628	185833290	protein_coding	4,92	0,0586426
ENSG00000169914	OTUD3	1	19882513	19912945	protein_coding	4,92	0,0000000
ENSG00000133398	MED10	5	6371881	6378594	protein_coding	4,91	0,0012807
ENSG00000185087	FAM169B	15	98437162	98514382	protein_coding	4,90	0,0025348
ENSG00000152944	MED21	12	27022546	27066343	protein_coding	4,89	0,0793786
ENSG00000158023	WDR66	12	121917862	122003927	protein_coding	4,88	0,0012807
ENSG00000258986	TMEM179	14	104474678	104605647	protein_coding	4,88	0,0184474
ENSG00000099958	DERL3	22	23834503	23839128	protein_coding	4,88	0,0012807
ENSG00000132436	FIGNL1	7	50444128	50542535	protein_coding	4,88	0,0012807
ENSG00000170390	DCLK2	4	150078274	150257457	protein_coding	4,87	0,0012807
ENSG00000148660	CAMK2G	10	73812501	73874591	protein_coding	4,87	0,0335607
ENSG00000143001	TMEM61	1	54980792	54992293	protein_coding	4,87	0,0335607
ENSG00000145757	SPATA9	5	95652181	95698711	protein_coding	4,86	0,0025348
ENSG00000033867	SLC4A7	3	27372721	27484420	protein_coding	4,86	0,0025348
ENSG00000213186	TRIM59	3	160432445	160485773	protein_coding	4,85	0,0793786
ENSG00000112167	SAYSD1	6	39104064	39115189	protein_coding	4,84	0,0006696
ENSG00000070718	AP3M2	8	42152946	42171673	protein_coding	4,81	0,0012807
ENSG00000121898	CPXM2	10	123706207	123940267	protein_coding	4,81	0,0184474
ENSG00000179930	ZNF648	1	182054570	182061712	protein_coding	4,80	0,6142622
ENSG00000198729	PPP1R14C	6	150143076	150250357	protein_coding	4,79	0,0012807
ENSG00000187098	MITF	3	69739435	69968337	protein_coding	4,78	0,0012807
ENSG00000196466	ZNF799	19	12390016	12401271	protein_coding	4,78	0,0012807

ENSG00000149418	ST14	11	130159562	130210376	protein_coding	4,78	0,0184474
ENSG00000165181	C9orf84	9	111686173	111795008	protein_coding	4,77	0,2725752
ENSG00000105865	DUS4L	7	107563484	107578464	protein_coding	4,77	0,0000000
ENSG00000145781	COMMD10	5	116084991	116412762	protein_coding	4,76	0,0418702
ENSG00000260314	MRC1	10	17809344	17911170	protein_coding	4,75	0,0012807
ENSG00000129235	TXNDC17	17	6640758	6644541	protein_coding	4,75	0,0050271
ENSG00000173698	ADGRG2	X	18989309	19122637	protein_coding	4,74	0,0006696
ENSG00000135540	NHSL1	6	138422043	138692571	protein_coding	4,71	0,0000000
ENSG00000059588	TARBP1	1	234391313	234479103	protein_coding	4,71	0,0050271
ENSG00000136011	STAB2	12	103587273	103766727	protein_coding	4,70	0,0012807
ENSG00000132780	NASP	1	45583846	45618904	protein_coding	4,70	0,0000000
ENSG00000170584	NUDCD2	5	163446526	163460140	protein_coding	4,69	0,0012807
ENSG00000100479	POLE2	14	49643555	49688422	protein_coding	4,69	0,0609506
ENSG00000170807	LMOD2	7	123655807	123664290	protein_coding	4,68	0,3785161
ENSG00000131944	FAAP24	19	32972209	32978222	protein_coding	4,67	0,1488645
ENSG00000188385	JAKMIP3	10	132104671	132184809	protein_coding	4,66	0,0037996
ENSG00000132874	SLC14A2	18	45212995	45683686	protein_coding	4,66	0,0012807
ENSG00000041353	RAB27B	18	54717860	54895516	protein_coding	4,65	0,0012807
ENSG00000172209	GPR22	7	107470018	107475659	protein_coding	4,64	0,0184474
ENSG00000151332	MBIP	14	36298558	36320676	protein_coding	4,63	0,0012807
ENSG00000172687	ZNF738	19	21358930	21379302	protein_coding	4,62	0,0012807
ENSG00000214189	ZNF788	19	12092263	12137235	protein_coding	4,60	0,0050271
ENSG00000134049	IER3IP1	18	47152998	47176374	protein_coding	4,59	0,1245284
ENSG00000177459	ERICH5	8	98064311	98093610	protein_coding	4,58	0,0000000
ENSG00000188613	NANOS1	10	119029716	119033732	protein_coding	4,57	0,0012807
ENSG00000072952	MRVI1	11	10573091	10693988	protein_coding	4,57	0,0006696
ENSG00000160813	PPP1R35	7	100435282	100436565	protein_coding	4,57	0,0012807
ENSG00000165929	TC2N	14	91779751	91867536	protein_coding	4,57	0,0006696
ENSG00000182004	SNRPE	1	203861603	203870550	protein_coding	4,57	0,0257457
ENSG00000101003	GINS1	20	25407727	25452628	protein_coding	4,55	0,0012807
ENSG00000156831	NSMCE2	8	125091679	125367120	protein_coding	4,54	0,0062591
ENSG00000134758	RNF138	18	32091855	32131561	protein_coding	4,53	0,2127456
ENSG00000186625	KATNA1	6	149594873	149648972	protein_coding	4,53	0,0012807
ENSG00000143375	CGN	1	151510510	151538692	protein_coding	4,53	0,0012807
ENSG00000203965	EFCAB7	1	63523372	63572693	protein_coding	4,52	0,6142622
ENSG00000135045	C9orf40	9	74946581	74952886	protein_coding	4,52	0,0050271
ENSG00000106034	CPED1	7	120988677	121297444	protein_coding	4,52	0,0000000
ENSG00000109452	INPP4B	4	142023160	142847432	protein_coding	4,51	0,0012807
ENSG00000113734	BNIP1	5	173144442	173164387	protein_coding	4,51	0,0000000
ENSG00000120156	TEK	9	27109141	27230175	protein_coding	4,51	0,0012807
ENSG00000166266	CUL5	11	108008733	108107776	protein_coding	4,51	0,0000000
ENSG00000175779	C15orf53	15	38696598	38700038	protein_coding	4,50	0,6142622
ENSG00000179088	C12orf42	12	103237591	103496010	protein_coding	4,50	0,0111464
ENSG00000146013	GFRA3	5	138252379	138274671	protein_coding	4,50	0,0006696
ENSG00000071243	ING3	7	120950749	120977216	protein_coding	4,50	0,0793786
ENSG00000147905	ZCCHC7	9	37120539	37358149	protein_coding	4,49	0,0335607
ENSG00000242950	ERVW-1	7	92468380	92477986	protein_coding	4,49	0,0075075
ENSG00000205111	CDKL4	2	39175646	39229588	protein_coding	4,48	0,0075075
ENSG00000149636	DSN1	20	36751791	36773818	protein_coding	4,48	0,4999662
ENSG00000140284	SLC27A2	15	50182196	50236395	protein_coding	4,47	0,0012807
ENSG00000154277	UCHL1	4	41256413	41268455	protein_coding	4,47	0,0257457
ENSG00000101811	CSTF2	X	100820359	100840932	protein_coding	4,47	0,0000000
ENSG00000051596	THOC3	5	175917873	176034680	protein_coding	4,46	0,0006696
ENSG00000107833	NPM3	10	101781325	101783413	protein_coding	4,45	0,0037996
ENSG00000177138	FAM9B	X	9024232	9164639	protein_coding	4,44	0,0335607

ENSG00000163166	IWS1	2	127436207	127526886	protein_coding	4,44	0,1598647
ENSG00000136824	SMC2	9	104094260	104141417	protein_coding	4,44	0,0050271
ENSG00000120533	ENY2	8	109334324	109345953	protein_coding	4,43	0,0111464
ENSG00000055609	KMT2C	7	152134922	152436005	protein_coding	4,43	0,0012807
ENSG00000083635	NUFIP1	13	44939249	44989483	protein_coding	4,43	0,0062591
ENSG00000115252	PDE1A	2	182140036	182523192	protein_coding	4,42	0,0000000
ENSG00000116525	TRIM62	1	33145402	33182059	protein_coding	4,42	0,0000000
ENSG00000183527	PSMG1	21	39174769	39183851	protein_coding	4,42	0,0012807
ENSG00000088325	TPX2	20	31739271	31801805	protein_coding	4,40	0,0000000
ENSG00000054796	SPO11	20	57329759	57343994	protein_coding	4,40	0,0418702
ENSG00000088256	GNA11	19	3094410	3124004	protein_coding	4,39	0,3963183
ENSG00000257093	KIAA1147	7	141656728	141702153	protein_coding	4,39	0,0000000
ENSG00000272886	DCP1A	3	53283428	53347610	protein_coding	4,38	0,0000000
ENSG00000172817	CYP7B1	8	64587763	64798761	protein_coding	4,38	0,0105771
ENSG00000106686	SPATA6L	9	4553386	4666674	protein_coding	4,38	0,0012807
ENSG00000135334	AKIRIN2	6	87675072	87702209	protein_coding	4,38	0,0037996
ENSG00000166529	ZSCAN21	7	100049774	100065038	protein_coding	4,38	0,0000000
ENSG00000248710	ENSG00000	3	160227454	160449829	protein_coding	4,38	0,6142622
ENSG00000120306	CYSTM1	5	140174642	140282052	protein_coding	4,37	0,0000000
ENSG00000166851	PLK1	16	23677656	23690367	protein_coding	4,37	0,0148097
ENSG00000111554	MDM1	12	68272443	68332381	protein_coding	4,36	0,0012807
ENSG00000137393	RNF144B	6	18387350	18468874	protein_coding	4,36	0,0012807
ENSG00000196876	SCN8A	12	51590266	51812864	protein_coding	4,35	0,0012807
ENSG00000115705	TPO	2	1374223	1543711	protein_coding	4,34	0,0717418
ENSG00000135094	SDS	12	113392445	113426301	protein_coding	4,33	0,0012807
ENSG00000169379	ARL13B	3	93980139	94055668	protein_coding	4,32	0,0025348
ENSG00000172530	BANP	16	87949244	88118422	protein_coding	4,32	0,0257457
ENSG00000135205	CCDC146	7	77122434	77329533	protein_coding	4,32	0,0538744
ENSG00000058729	RIOK2	5	97160867	97183260	protein_coding	4,30	0,0025348
ENSG00000168907	PLA2G4F	15	42139034	42156636	protein_coding	4,30	0,0111464
ENSG00000235711	ANKRD34C	15	79293285	79298235	protein_coding	4,30	0,0012807
ENSG00000162456	KNCN	1	46545644	46551527	protein_coding	4,30	0,0418702
ENSG00000099715	PCDH11Y	X	5000226	5742224	protein_coding	4,30	0,0012807
ENSG00000147003	TMEM27	X	15627318	15665031	protein_coding	4,29	0,0793786
ENSG00000197128	ZNF772	19	57466663	57477570	protein_coding	4,28	0,0012807
ENSG00000095777	MYO3A	10	25934267	26212527	protein_coding	4,27	0,2229774
ENSG00000112759	SLC29A1	6	44219505	44234151	protein_coding	4,27	0,0012807
ENSG00000174473	GALNTL6	4	171812254	173041559	protein_coding	4,26	0,0717418
ENSG00000173065	FAM222B	17	28755978	28855232	protein_coding	4,26	0,0012807
ENSG00000086967	MYBPC2	19	50432903	50466321	protein_coding	4,25	0,0148097
ENSG00000112379	ARFGEF3	6	138161921	138344663	protein_coding	4,24	0,0172684
ENSG00000132383	RPA1	17	1829702	1900082	protein_coding	4,24	0,0006696
ENSG00000154783	FGD5	3	14818962	14934565	protein_coding	4,23	0,0075075
ENSG00000011083	SLC6A7	5	150189957	150222788	protein_coding	4,23	0,1245284
ENSG00000116741	RGS2	1	192809039	192812283	protein_coding	4,22	0,0012807
ENSG00000174667	OR7D4	19	9213850	9214871	protein_coding	4,20	0,0418702
ENSG00000176239	OR51B6	11	5351508	5352446	protein_coding	4,20	0,1245284
ENSG00000182674	KCNB2	8	72537391	72938349	protein_coding	4,20	0,0111464
ENSG00000278705	HIST1H4B	6	26026815	26027252	protein_coding	4,20	0,0418702
ENSG00000104327	CALB1	8	90058608	90095475	protein_coding	4,20	0,0335607
ENSG00000137692	DCUN1D5	11	103062076	103092215	protein_coding	4,19	0,0062591
ENSG00000175097	RAG2	11	36575574	36598279	protein_coding	4,19	0,0025348
ENSG00000163012	ZSWIM2	2	186827835	186849208	protein_coding	4,18	0,0184474
ENSG00000078401	EDN1	6	12290363	12297194	protein_coding	4,18	0,0184474
ENSG00000149308	NPAT	11	108157215	108222642	protein_coding	4,18	0,1245284

ENSG00000028839	TBPL1	6	133952170	133990432	protein_coding	4,17	0,0012807
ENSG00000183569	SERHL2	22	42553617	42574382	protein_coding	4,16	0,0012807
ENSG00000032389	TSSC1	2	3188925	3377882	protein_coding	4,16	0,0012807
ENSG00000146267	FAXC	6	99271169	99350062	protein_coding	4,15	0,0300092
ENSG00000116273	PHF13	1	6613685	6624033	protein_coding	4,15	0,0000000
ENSG00000165643	SOHLH1	9	135693407	135699528	protein_coding	4,15	0,4999662
ENSG00000181378	CCDC108	2	219002846	219041527	protein_coding	4,14	0,0075075
ENSG00000164253	WDR41	5	77425970	77620611	protein_coding	4,13	0,0148097
ENSG00000174013	FBXO45	3	196568611	196589059	protein_coding	4,13	0,0075075
ENSG00000162607	USP1	1	62436297	62451804	protein_coding	4,13	0,0012807
ENSG00000105849	TWISTNB	7	19695462	19709087	protein_coding	4,12	0,0075075
ENSG00000267909	CCDC177	14	69569816	69574837	protein_coding	4,12	0,0012807
ENSG00000064652	SNX24	5	122843439	123029354	protein_coding	4,11	0,0062591
ENSG00000110108	TMEM109	11	60913874	60923443	protein_coding	4,11	0,0000000
ENSG00000163159	VPS72	1	151169987	151195321	protein_coding	4,10	0,0418702
ENSG00000136944	LMX1B	9	126614443	126701032	protein_coding	4,10	0,0418702
ENSG00000131686	CA6	1	8945867	8975092	protein_coding	4,10	0,0418702
ENSG00000176208	ATAD5	17	30831970	30895869	protein_coding	4,09	0,0538744
ENSG00000175267	VWA3A	16	22092538	22156966	protein_coding	4,09	0,0012807
ENSG00000158435	CNOT11	2	101252802	101270316	protein_coding	4,09	0,0025348
ENSG00000135100	HNF1A	12	120978543	121002512	protein_coding	4,08	0,0111464
ENSG00000107864	CPEB3	10	92046692	92291087	protein_coding	4,08	0,0062591
ENSG00000198948	MFAP3L	4	169986597	170033031	protein_coding	4,08	0,0062591
ENSG00000088926	F11	4	186265945	186288806	protein_coding	4,08	0,0075075
ENSG00000135838	NPL	1	182789293	182830384	protein_coding	4,07	0,2229774
ENSG00000159596	TMEM69	1	45687214	45694443	protein_coding	4,07	0,0012807
ENSG00000239521	GATS	7	100200653	100272218	protein_coding	4,07	0,0012807
ENSG00000137075	RNF38	9	36336396	36487548	protein_coding	4,05	0,0012807
ENSG00000163935	SFMBT1	3	52903572	53046750	protein_coding	4,05	0,0012807
ENSG00000152382	TADA1	1	166856510	166876327	protein_coding	4,05	0,0050271
ENSG00000108001	EBF3	10	129835283	129963841	protein_coding	4,05	0,0184474
ENSG00000176896	TCEANC	X	13653189	13681964	protein_coding	4,05	0,0050271
ENSG00000116016	EPAS1	2	46293667	46386703	protein_coding	4,05	0,0000000
ENSG00000151892	GFRA1	10	116056925	116273467	protein_coding	4,04	0,0111464
ENSG00000166920	C15orf48	15	45430529	45448761	protein_coding	4,04	0,0025348
ENSG00000103942	HOMER2	15	82836946	82986153	protein_coding	4,04	0,0000000
ENSG00000130349	C6orf203	6	107028213	107051342	protein_coding	4,03	0,0257457
ENSG00000167600	CYP2S1	19	41193049	41207539	protein_coding	4,03	0,1488645
ENSG00000147010	SH3KBP1	X	19533975	19887601	protein_coding	4,02	0,0000000
ENSG00000065154	OAT	10	124397303	124418976	protein_coding	4,01	0,0012807
ENSG00000169607	CKAP2L	2	112736607	112764677	protein_coding	4,01	0,0172684
ENSG00000172728	FUT10	8	33370824	33473422	protein_coding	4,00	0,0000000
ENSG00000108055	SMC3	10	110567691	110604636	protein_coding	4,00	0,0000000
ENSG00000169032	MAP2K1	15	66386817	66492312	protein_coding	4,00	0,0012807
ENSG00000096092	TMEM14A	6	52671109	52686588	protein_coding	4,00	0,0062591
ENSG00000161973	CCDC42	17	8729934	8745219	protein_coding	4,00	0,3269660
ENSG00000139767	SRRM4	12	118981495	119163051	protein_coding	4,00	0,1245284
ENSG00000259305	ZHX1-C8orf7	8	123226189	123274284	protein_coding	4,00	0,3785161
ENSG00000272636	DOC2B	17	142789	181636	protein_coding	3,98	0,0050271
ENSG00000065457	ADAT1	16	75596981	75623300	protein_coding	3,98	0,0000000
ENSG00000059728	MXD1	2	69897688	69942945	protein_coding	3,98	0,0000000
ENSG00000137880	GCHFR	15	40764020	40767710	protein_coding	3,97	0,1598647
ENSG00000141854	ENSG000001	19	14072536	14075062	protein_coding	3,96	0,0075075
ENSG00000214960	ISPD	7	16087527	16421322	protein_coding	3,96	0,0012807
ENSG00000070019	GUCY2C	12	14612632	14696585	protein_coding	3,96	0,0012807

ENSG00000157570	TSPAN18	11	44726465	44932421	protein_coding	3,95	0,0793786
ENSG00000112110	MRPL18	6	159789812	159798436	protein_coding	3,95	0,0006696
ENSG00000161920	MED11	17	4731428	4733610	protein_coding	3,95	0,0012807
ENSG00000127152	BCL11B	14	99169287	99271524	protein_coding	3,95	0,1598647
ENSG00000170365	SMAD1	4	145481194	145558079	protein_coding	3,94	0,1867869
ENSG00000162493	PDPN	1	13583465	13617957	protein_coding	3,93	0,0012807
ENSG00000174827	PDZK1	1	145670852	145708148	protein_coding	3,92	0,0000000
ENSG00000255529	POLR2M	15	57706629	57782762	protein_coding	3,92	0,0000000
ENSG00000131148	EMC8	16	85771758	85799608	protein_coding	3,91	0,0000000
ENSG00000176890	TYMS	18	657604	673578	protein_coding	3,90	0,0025348
ENSG00000111046	MYF6	12	80707498	80709474	protein_coding	3,90	0,3269660
ENSG00000186280	KDM4D	11	94973681	94999518	protein_coding	3,89	0,0335607
ENSG00000137273	FOXF2	6	1389834	1395597	protein_coding	3,88	0,0335607
ENSG00000129295	LRRC6	8	132571953	132675617	protein_coding	3,87	0,0105771
ENSG00000138346	DNA2	10	68414064	68472121	protein_coding	3,87	0,0000000
ENSG00000176124	DLEU1	13	50082171	50723236	protein_coding	3,87	0,0257457
ENSG00000080986	NDC80	18	2571511	2616635	protein_coding	3,86	0,0050271
ENSG00000166831	RBPMS2	15	64739892	64775587	protein_coding	3,86	0,0050271
ENSG00000143195	ILDR2	1	166895711	166975482	protein_coding	3,86	0,0012807
ENSG00000122873	CISD1	10	58269058	58289586	protein_coding	3,86	0,4999662
ENSG00000205464	ATP6AP1L	5	82279462	82386977	protein_coding	3,86	0,0609506
ENSG00000171984	C20orf196	20	5750393	5863912	protein_coding	3,85	0,0037996
ENSG00000129460	NGDN	14	23469688	23509862	protein_coding	3,83	0,0025348
ENSG00000215421	ZNF407	18	74597870	75065671	protein_coding	3,83	0,0000000
ENSG00000182732	RGS6	14	71932439	72566529	protein_coding	3,82	0,0025348
ENSG00000141664	ZCCHC2	18	62523007	62587709	protein_coding	3,82	0,0257457
ENSG00000105185	PDCD5	19	32581068	32587452	protein_coding	3,80	0,1867869
ENSG00000136153	LMO7	13	75620434	75859870	protein_coding	3,80	0,0000000
ENSG00000141458	NPC1	18	23506184	23586898	protein_coding	3,80	0,0000000
ENSG00000177047	IFNW1	9	21140214	21142145	protein_coding	3,80	0,1245284
ENSG00000106541	AGR2	7	16791811	16833433	protein_coding	3,80	0,0111464
ENSG00000197323	TRIM33	1	114392777	114511160	protein_coding	3,79	0,0012807
ENSG00000115271	GCA	2	162318840	162371595	protein_coding	3,79	0,1598647
ENSG00000102054	RBBP7	X	16839283	16870414	protein_coding	3,79	0,0012807
ENSG00000148950	IMMP1L	11	31432401	31509645	protein_coding	3,77	0,0050271
ENSG00000185666	SYN3	22	32512552	33058372	protein_coding	3,77	0,0335607
ENSG00000184277	TM2D3	15	101621444	101652391	protein_coding	3,76	0,0012807
ENSG00000135900	MRPL44	2	223957404	223967714	protein_coding	3,76	0,0012807
ENSG00000164900	GBX1	7	151148589	151174745	protein_coding	3,75	0,0062591
ENSG00000277149	TYW1B	7	72558744	72828198	protein_coding	3,75	0,0300092
ENSG00000141540	TTYH2	17	74213514	74262020	protein_coding	3,74	0,0148097
ENSG00000175691	ZNF77	19	2933218	2944971	protein_coding	3,74	0,0012807
ENSG00000129170	CSRP3	11	19182030	19210573	protein_coding	3,74	0,6606414
ENSG00000125898	FAM110A	20	833715	857463	protein_coding	3,74	0,0012807
ENSG00000109685	WHSC1	4	1871424	1982207	protein_coding	3,72	0,0012807
ENSG00000099282	TSPAN15	10	69451473	69507669	protein_coding	3,72	0,0257457
ENSG00000123612	ACVR1C	2	157526767	157629005	protein_coding	3,72	0,0075075
ENSG00000221988	PPT2	6	32153441	32163680	protein_coding	3,71	0,0012807
ENSG00000101331	CCM2L	20	32010450	32032180	protein_coding	3,70	0,0184474
ENSG00000158901	WFDC8	20	45551153	45579326	protein_coding	3,70	0,1245284
ENSG00000223638	RFPL4A	19	55759014	55763175	protein_coding	3,70	0,6142622
ENSG00000166368	OR2D2	11	6891490	6892599	protein_coding	3,70	0,0418702
ENSG00000170819	BFSP2	3	133399995	133475222	protein_coding	3,70	0,1245284
ENSG00000133773	CCDC59	12	82223681	82358805	protein_coding	3,69	0,1598647
ENSG00000132938	MTUS2	13	28820348	29505947	protein_coding	3,69	0,0586426

ENSG00000139131	YARS2	12	32727490	32755902	protein_coding	3,69	0,0012807
ENSG00000157680	DGKI	7	137381037	137847092	protein_coding	3,69	0,0050271
ENSG00000132016	C19orf57	19	13882348	13906452	protein_coding	3,68	0,0012807
ENSG00000088766	CRLS1	20	6006090	6040053	protein_coding	3,68	0,0995111
ENSG00000163634	THOC7	3	63833870	63863903	protein_coding	3,68	0,0995111
ENSG00000121851	POLR3GL	1	145964702	145978848	protein_coding	3,67	0,1867869
ENSG00000165113	GKAP1	9	83739421	83829516	protein_coding	3,67	0,0335607
ENSG00000137221	TJAP1	6	43477523	43506556	protein_coding	3,67	0,0000000
ENSG00000143190	POU2F1	1	167220829	167427345	protein_coding	3,66	0,1245284
ENSG00000172361	CFAP53	18	50227193	50266522	protein_coding	3,65	0,0050271
ENSG00000108984	MAP2K6	17	69414698	69543331	protein_coding	3,64	0,0335607
ENSG00000158715	SLC45A3	1	205657851	205680459	protein_coding	3,64	0,0000000
ENSG00000130695	CEP85	1	26234153	26279038	protein_coding	3,63	0,0012807
ENSG00000196660	SLC30A10	1	219685427	219958647	protein_coding	3,63	0,4463415
ENSG00000153012	LGI2	4	24998847	25030879	protein_coding	3,62	0,0172684
ENSG00000102738	MRPS31	13	40729135	40771173	protein_coding	3,62	0,2725752
ENSG00000138801	PAPSS1	4	107590276	107720452	protein_coding	3,62	0,0000000
ENSG00000273611	ZNHIT3	17	36486629	36499310	protein_coding	3,62	0,0012807
ENSG00000273559	CWC25	17	38800434	38825481	protein_coding	3,61	0,0793786
ENSG00000145979	TBC1D7	6	13266542	13328583	protein_coding	3,61	0,0012807
ENSG00000148700	ADD3	10	109996368	110135565	protein_coding	3,60	0,0012807
ENSG00000138136	LBX1	10	101226195	101229794	protein_coding	3,60	0,6142622
ENSG00000101981	F9	X	139530758	139563458	protein_coding	3,60	0,3269660
ENSG00000054654	SYNE2	14	63852983	64226433	protein_coding	3,60	0,1245284
ENSG00000171202	TMEM126A	11	85647967	85656547	protein_coding	3,59	0,0418702
ENSG00000156273	BACH1	21	29194071	29630751	protein_coding	3,59	0,0000000
ENSG00000151572	ANO4	12	100717526	101128641	protein_coding	3,58	0,0000000
ENSG00000144395	CCDC150	2	196639554	196763490	protein_coding	3,58	0,0000000
ENSG00000109944	C11orf63	11	122882528	122959798	protein_coding	3,58	0,2127456
ENSG00000149970	CNKSR2	X	21374418	21654695	protein_coding	3,58	0,0012807
ENSG00000214212	C19orf38	19	10836575	10869790	protein_coding	3,58	0,4999662
ENSG00000121039	RDH10	8	73294612	73325281	protein_coding	3,57	0,0025348
ENSG00000158545	ZC3H18	16	88570381	88631966	protein_coding	3,57	0,4999662
ENSG00000116791	CRYZ	1	74705482	74733408	protein_coding	3,57	0,0012807
ENSG00000164778	EN2	7	155458129	155464831	protein_coding	3,57	0,0509539
ENSG00000144554	FANCD2	3	10026414	10101930	protein_coding	3,56	0,0538744
ENSG00000152192	POU4F1	13	78598362	78603560	protein_coding	3,56	0,1245284
ENSG00000112394	SLC16A10	6	111087503	111231194	protein_coding	3,56	0,0012807
ENSG00000166455	C16orf46	16	81053497	81077267	protein_coding	3,56	0,0050271
ENSG00000167065	DUSP18	22	30652051	30667890	protein_coding	3,54	0,0037996
ENSG00000177483	RBM44	2	237798389	237842808	protein_coding	3,54	0,1867869
ENSG00000160588	MPZL3	11	118226690	118252350	protein_coding	3,54	0,0586426
ENSG00000115363	EVA1A	2	75469302	75569722	protein_coding	3,54	0,3963183
ENSG00000187908	DMBT1	10	122560665	122643736	protein_coding	3,53	0,4808196
ENSG00000204842	ATXN2	12	111452214	111599676	protein_coding	3,53	0,1245284
ENSG00000178966	RMI1	9	83980711	84004074	protein_coding	3,52	0,0012807
ENSG00000166452	AKIP1	11	8911139	8920084	protein_coding	3,52	0,0012807
ENSG00000168589	DYNLRB2	16	80540734	80550760	protein_coding	3,51	0,1598647
ENSG00000134222	PSRC1	1	109279556	109283186	protein_coding	3,51	0,0000000
ENSG00000183431	SF3A3	1	37956975	37990921	protein_coding	3,50	0,0012807
ENSG00000164105	SAP30	4	173369969	173377532	protein_coding	3,50	0,0257457
ENSG00000176177	ENTHD1	22	39743044	39893864	protein_coding	3,50	0,1245284
ENSG00000137090	DMRT1	9	841690	969090	protein_coding	3,50	0,1245284
ENSG00000168913	ENHO	9	34521040	34523041	protein_coding	3,50	0,1775672
ENSG00000178919	FOXE1	9	97853254	97856715	protein_coding	3,50	0,0418702

ENSG00000130368	MAS1	6	159906690	159916530	protein_coding	3,50	0,0111464
ENSG00000065534	MYLK	3	123610049	123884331	protein_coding	3,49	0,0050271
ENSG00000169375	SIN3A	15	75369379	75455842	protein_coding	3,49	0,0000000
ENSG00000067798	NAV3	12	77830905	78213008	protein_coding	3,49	0,0012807
ENSG00000198604	BAZ1A	14	34752731	34875647	protein_coding	3,48	0,0075075
ENSG00000085840	ORC1	1	52372829	52404459	protein_coding	3,48	0,1867869
ENSG00000121274	PAPD5	16	50152918	50235310	protein_coding	3,47	0,0793786
ENSG00000115758	ODC1	2	10439968	10448504	protein_coding	3,47	0,0012807
ENSG00000181333	HEPHL1	11	94021361	94113751	protein_coding	3,47	0,0111464
ENSG00000163618	CADPS	3	62398346	62875389	protein_coding	3,46	0,0012807
ENSG00000105991	HOXA1	7	27092993	27095996	protein_coding	3,45	0,0075075
ENSG00000151164	RAD9B	12	110501655	110532086	protein_coding	3,45	0,3096079
ENSG00000159208	CIART	1	150282543	150287093	protein_coding	3,44	0,0012807
ENSG00000179270	C2orf71	2	29060976	29074261	protein_coding	3,43	0,0062591
ENSG00000139168	ZCRB1	12	42312078	42326118	protein_coding	3,43	0,2229774
ENSG00000131747	TOP2A	17	40388516	40417950	protein_coding	3,42	0,0335607
ENSG00000183023	SLC8A1	2	40097270	40611053	protein_coding	3,41	0,0012807
ENSG00000081870	HSPB11	1	53916574	53945929	protein_coding	3,41	0,0538744
ENSG00000121542	SEC22A	3	123201927	123274130	protein_coding	3,41	0,0075075
ENSG00000139350	NEDD1	12	96907223	96953777	protein_coding	3,40	0,0075075
ENSG00000186106	ANKRD46	8	100509752	100559784	protein_coding	3,40	0,0184474
ENSG00000182578	CSF1R	5	150053291	150113372	protein_coding	3,40	0,0012807
ENSG00000197977	ELOVL2	6	10980759	11044314	protein_coding	3,39	0,0012807
ENSG00000113318	MSH3	5	80654648	80876460	protein_coding	3,39	0,0025348
ENSG00000187775	DNAH17	17	78423697	78577394	protein_coding	3,38	0,0300092
ENSG00000135945	REV1	2	99400475	99490035	protein_coding	3,38	0,0012807
ENSG00000151948	GLT1D1	12	128853427	128984968	protein_coding	3,38	0,0012807
ENSG00000167554	ZNF610	19	52336245	52367778	protein_coding	3,37	0,1245284
ENSG00000185480	PARPBP	12	102120185	102197520	protein_coding	3,37	0,0000000
ENSG00000154611	PSMA8	18	26133852	26193355	protein_coding	3,37	0,0300092
ENSG00000154914	USP43	17	9644698	9729691	protein_coding	3,37	0,2725752
ENSG00000146857	STRA8	7	135231979	135258492	protein_coding	3,36	0,2229774
ENSG00000102984	ZNF821	16	71859680	71895336	protein_coding	3,36	0,0012807
ENSG00000086061	DNAJA1	9	33025211	33039907	protein_coding	3,35	0,0075075
ENSG00000169564	PCBP1	2	70087454	70089203	protein_coding	3,34	0,0012807
ENSG00000131626	PPFIA1	11	70270700	70384403	protein_coding	3,34	0,0000000
ENSG00000085449	WDFY1	2	223855716	223945387	protein_coding	3,34	0,0025348
ENSG00000205038	PKHD1L1	8	109362477	109530330	protein_coding	3,33	0,3785161
ENSG00000157191	NECAP2	1	16440672	16460078	protein_coding	3,33	0,0012807
ENSG00000172575	RASGRP1	15	38488103	38565575	protein_coding	3,33	0,0000000
ENSG00000130560	UBAC1	9	135932969	135961380	protein_coding	3,32	0,0012807
ENSG00000133477	FAM83F	22	39994949	40043529	protein_coding	3,32	0,1488645
ENSG00000141858	SAMD1	19	14087840	14091036	protein_coding	3,32	0,0717418
ENSG00000162244	RPL29	3	51993600	51995942	protein_coding	3,30	0,0335607
ENSG00000124196	GTSF1L	20	43726164	43726998	protein_coding	3,30	0,0418702
ENSG00000204671	IL31	12	122172030	122174199	protein_coding	3,30	0,3269660
ENSG00000122194	PLG	6	160702238	160753315	protein_coding	3,30	0,0111464
ENSG00000181867	FTMT	5	121851955	121852833	protein_coding	3,30	0,1245284
ENSG00000162490	DRAXIN	1	11691729	11725857	protein_coding	3,30	0,0418702
ENSG00000268988	SPANXN2	X	143711955	143721423	protein_coding	3,30	0,1245284
ENSG00000197928	ZNF677	19	53235381	53254898	protein_coding	3,30	0,0075075
ENSG00000089006	SNX5	20	17941597	17968980	protein_coding	3,30	0,0012807
ENSG00000138193	PLCE1	10	93993989	94332823	protein_coding	3,30	0,1867869
ENSG00000094804	CDC6	17	40287633	40304657	protein_coding	3,29	0,1867869
ENSG00000183814	LIN9	1	226231149	226309869	protein_coding	3,29	0,0012807

ENSG00000186184	POLR1D	13	27620742	27667415	protein_coding	3,28	0,0335607
ENSG00000141576	RNF157	17	76142453	76240373	protein_coding	3,28	0,0793786
ENSG00000006634	DBF4	7	87876216	87909541	protein_coding	3,28	0,1867869
ENSG00000167513	CDT1	16	88803213	88809258	protein_coding	3,28	0,0037996
ENSG00000075035	WSCD2	12	108129471	108250537	protein_coding	3,27	0,0793786
ENSG00000148942	SLC5A12	11	26667019	26723427	protein_coding	3,27	0,2600087
ENSG00000197077	KIAA1671	22	24952730	25197448	protein_coding	3,27	0,0000000
ENSG00000164114	MAP9	4	155342658	155376970	protein_coding	3,27	0,1245284
ENSG00000001460	STPG1	1	24356999	24416934	protein_coding	3,26	0,0000000
ENSG00000173253	DMRT2	9	1049858	1057552	protein_coding	3,26	0,3269660
ENSG00000185267	CDNF	10	14819250	14838575	protein_coding	3,26	0,0793786
ENSG00000241978	AKAP2	9	110048598	110172512	protein_coding	3,26	0,1598647
ENSG00000120833	SOCS2	12	93569814	93583487	protein_coding	3,25	0,0012807
ENSG00000115109	EPB41L5	2	120013005	120179119	protein_coding	3,24	0,0025348
ENSG00000149596	JPH2	20	44111695	44187578	protein_coding	3,24	0,2127456
ENSG00000042813	ZPBP	7	49850421	50121329	protein_coding	3,24	0,1598647
ENSG00000032219	ARID4A	14	58298385	58373887	protein_coding	3,22	0,1867869
ENSG00000151748	SAV1	14	50632058	50668331	protein_coding	3,22	0,0012807
ENSG00000182759	MAFA	8	143419182	143430406	protein_coding	3,21	0,1775672
ENSG00000144645	OSBPL10	3	31657890	32077580	protein_coding	3,21	0,0012807
ENSG00000131351	HAUS8	19	17049729	17075625	protein_coding	3,21	0,0418702
ENSG00000004779	NDUFAB1	16	23581002	23596356	protein_coding	3,21	0,0025348
ENSG00000139445	FOXN4	12	109277979	109309220	protein_coding	3,21	0,1168222
ENSG00000133020	MYH8	17	10390322	10421950	protein_coding	3,20	0,1245284
ENSG00000103355	PRSS33	16	2783953	2787948	protein_coding	3,20	0,3269660
ENSG00000149452	SLC22A8	11	62989154	63015839	protein_coding	3,20	0,0418702
ENSG00000168267	PTF1A	10	23192327	23194252	protein_coding	3,20	0,3269660
ENSG00000260220	CCDC187	9	136280942	136306901	protein_coding	3,20	0,6142622
ENSG00000106302	HYAL4	7	123828983	123877478	protein_coding	3,20	0,6142622
ENSG00000156925	ZIC3	X	137566142	137577691	protein_coding	3,20	0,0111464
ENSG00000069020	MAST4	5	66596361	67169595	protein_coding	3,19	0,0184474
ENSG00000124596	OARD1	6	41033627	41097787	protein_coding	3,19	0,0012807
ENSG00000263809	ENSG00000[illegible]	17	8368638	8383187	protein_coding	3,19	0,4999662
ENSG00000144452	ABCA12	2	214931542	215138428	protein_coding	3,19	0,0000000
ENSG00000205838	TTC23L	5	34838833	34899456	protein_coding	3,18	0,2600087
ENSG00000215186	GOLGA6B	15	72654738	72666394	protein_coding	3,18	0,2229774
ENSG00000140543	DET1	15	88511559	88546675	protein_coding	3,18	0,0012807
ENSG00000168216	LMBRD1	6	69675802	69797111	protein_coding	3,17	0,1245284
ENSG00000120149	MSX2	5	174724533	174730893	protein_coding	3,17	0,0050271
ENSG00000241186	TDGF1	3	46574555	46626543	protein_coding	3,17	0,3269660
ENSG00000005108	THSD7A	7	11370357	11832198	protein_coding	3,16	0,1245284
ENSG00000136925	TSTD2	9	97600080	97633575	protein_coding	3,16	0,0012807
ENSG00000197808	ZNF461	19	36637192	36666853	protein_coding	3,16	0,0335607
ENSG00000146707	POMZP3	7	76609986	76627261	protein_coding	3,16	0,0025348
ENSG00000183150	GPR19	12	12660891	12696207	protein_coding	3,16	0,0062591
ENSG00000151849	CENPJ	13	24882284	24922889	protein_coding	3,16	0,0717418
ENSG00000087302	C14orf166	14	51989475	52010691	protein_coding	3,16	0,0111464
ENSG00000160886	LY6K	8	142700111	142705127	protein_coding	3,15	0,0148097
ENSG00000168658	VWA3B	2	98087116	98313299	protein_coding	3,15	0,3963183
ENSG00000157470	FAM81A	15	59372693	59523549	protein_coding	3,15	0,0012807
ENSG00000106305	AIMP2	7	6009245	6023834	protein_coding	3,14	0,0012807
ENSG00000175354	PTPN2	18	12785478	12929643	protein_coding	3,14	0,0025348
ENSG00000131969	ABHD12B	14	50872160	50904970	protein_coding	3,13	0,0335607
ENSG00000156983	BRPF1	3	9731729	9748018	protein_coding	3,13	0,4999662
ENSG00000184635	ZNF93	19	19900913	19935575	protein_coding	3,13	0,0012807

ENSG00000144451	SPAG16	2	213284379	214410501	protein_coding	3,13	0,0012807
ENSG00000149658	YTHDF1	20	63195429	63216234	protein_coding	3,12	0,0012807
ENSG00000114737	CISH	3	50606490	50611831	protein_coding	3,12	0,0184474
ENSG00000115128	SF3B6	2	24067584	24076443	protein_coding	3,10	0,3269660
ENSG00000166004	CEP295	11	93661639	93730358	protein_coding	3,10	0,0012807
ENSG00000196504	PRPF40A	2	152651593	152717997	protein_coding	3,10	0,2229774
ENSG00000169129	AFAP1L2	10	114294824	114404756	protein_coding	3,10	0,1168222
ENSG00000181035	SLC25A42	19	19063999	19112888	protein_coding	3,10	0,0012807
ENSG00000151025	GPR158	10	25175062	25602226	protein_coding	3,09	0,0335607
ENSG00000134852	CLOCK	4	55427903	55547138	protein_coding	3,09	0,0111464
ENSG00000262655	SPON1	11	13962689	14267884	protein_coding	3,09	0,0012807
ENSG00000196470	SIAH1	16	48356364	48448402	protein_coding	3,09	0,0111464
ENSG00000181826	RELL1	4	37590800	37686376	protein_coding	3,08	0,0995111
ENSG00000137513	NARS2	11	78435961	78574874	protein_coding	3,08	0,0025348
ENSG00000111913	FAM65B	6	24804282	25042168	protein_coding	3,07	0,0335607
ENSG00000005020	SKAP2	7	26667062	26995239	protein_coding	3,07	0,0111464
ENSG00000179399	GPC5	13	91398607	92873682	protein_coding	3,07	0,0184474
ENSG00000006757	PNPLA4	X	7898247	7927739	protein_coding	3,06	0,0012807
ENSG00000164236	ANKRD33B	5	10564330	10657816	protein_coding	3,06	0,0793786
ENSG00000170854	MINA	3	97941818	97972457	protein_coding	3,06	0,0012807
ENSG00000186812	ZNF397	18	35241030	35267133	protein_coding	3,06	0,0609506
ENSG00000065717	TLE2	19	2997638	3047635	protein_coding	3,05	0,2725752
ENSG00000139988	RDH12	14	67701886	67734452	protein_coding	3,05	0,1775672
ENSG00000132819	RBM38	20	57391407	57409333	protein_coding	3,04	0,1245284
ENSG00000186868	MAPT	17	45894382	46028334	protein_coding	3,04	0,2725752
ENSG00000169599	NFU1	2	69395750	69437628	protein_coding	3,03	0,0075075
ENSG00000154839	SKA1	18	50374995	50394173	protein_coding	3,03	0,1168222
ENSG00000197576	HOXA4	7	27128507	27130799	protein_coding	3,02	0,0509539
ENSG00000169155	ZBTB43	9	126805006	126838210	protein_coding	3,02	0,0184474
ENSG00000166477	LEO1	15	51938025	51971806	protein_coding	3,02	0,0025348
ENSG00000181274	FRAT2	10	97332497	97334709	protein_coding	3,02	0,0012807
ENSG00000128276	RFPL3	22	32354885	32361161	protein_coding	3,01	0,0025348
ENSG00000153064	BANK1	4	101411286	102074812	protein_coding	3,01	0,0075075
ENSG00000183230	CTNNA3	10	65912518	67696169	protein_coding	3,01	0,0025348
ENSG00000136045	PWP1	12	107685732	107713167	protein_coding	3,01	0,0075075
ENSG00000102781	KATNAL1	13	30202630	30307484	protein_coding	3,00	0,0012807
ENSG00000138767	CNOT6L	4	77713387	77819615	protein_coding	3,00	0,0012807
ENSG00000196091	MYBPC1	12	101568353	101686018	protein_coding	3,00	0,1245284
ENSG00000170290	SLN	11	107707378	107719693	protein_coding	3,00	0,0184474
ENSG00000183715	OPCML	11	132414977	133532519	protein_coding	3,00	0,0418702
ENSG00000183621	ZNF438	10	30820207	31031937	protein_coding	3,00	0,0012807
ENSG00000013293	SLC7A14	3	170459584	170586074	protein_coding	3,00	0,3269660
ENSG00000205086	C2orf91	2	41935368	41954266	protein_coding	3,00	0,1245284
ENSG00000170340	B3GNT2	2	62196113	62224731	protein_coding	2,99	0,0075075
ENSG00000160563	MED27	9	131860107	132079908	protein_coding	2,99	0,0012807
ENSG00000164116	GUCY1A3	4	155666711	155732349	protein_coding	2,99	0,2600087
ENSG00000112182	BACH2	6	89926529	90296908	protein_coding	2,97	0,0335607
ENSG00000111581	NUP107	12	68686734	68745809	protein_coding	2,97	0,1245284
ENSG00000198780	FAM169A	5	74777574	74866951	protein_coding	2,96	0,0012807
ENSG00000171914	TLN2	15	62390526	62844631	protein_coding	2,95	0,0418702
ENSG00000105928	DFNA5	7	24698353	24758113	protein_coding	2,95	0,0012807
ENSG00000132326	PER2	2	238244038	238290102	protein_coding	2,95	0,0012807
ENSG00000100281	HMGXB4	22	35257452	35295807	protein_coding	2,94	0,0184474
ENSG00000114648	KLHL18	3	47282917	47346816	protein_coding	2,94	0,0111464
ENSG00000261794	GOLGA8H	15	30604126	30614561	protein_coding	2,94	0,5364729

ENSG00000164615	CAMLG	5	134738501	134752160	protein_coding	2,94	0,0418702
ENSG00000183780	SLC35F3	1	233904933	234324516	protein_coding	2,94	0,0257457
ENSG00000175906	ARL4D	17	43398959	43401137	protein_coding	2,93	0,0148097
ENSG00000170310	STX8	17	9250471	9576591	protein_coding	2,93	0,1867869
ENSG00000156076	WIF1	12	65050626	65121566	protein_coding	2,93	0,0995111
ENSG00000171055	FEZ2	2	36531805	36646087	protein_coding	2,93	0,0335607
ENSG00000109762	SNX25	4	185204237	185370185	protein_coding	2,93	0,0012807
ENSG00000266094	RASSF5	1	206507530	206589283	protein_coding	2,92	0,0793786
ENSG00000184949	FAM227A	22	38578120	38656629	protein_coding	2,92	0,0335607
ENSG00000136492	BRIP1	17	61681266	61863521	protein_coding	2,91	0,0037996
ENSG00000115652	UXS1	2	106093303	106194339	protein_coding	2,90	0,0012807
ENSG00000215455	KRTAP10-1	21	44538981	44540195	protein_coding	2,90	0,6142622
ENSG00000185372	OR2V1	5	181124357	181125304	protein_coding	2,90	0,0418702
ENSG00000184022	OR2T10	1	248592830	248593768	protein_coding	2,90	0,3269660
ENSG00000138400	MDH1B	2	206737763	206765547	protein_coding	2,90	0,0148097
ENSG00000170322	NFRKB	11	129863636	129895590	protein_coding	2,89	0,0012807
ENSG00000076650	GPATCH1	19	33080880	33130542	protein_coding	2,89	0,1867869
ENSG00000172795	DCP2	5	112976702	113020970	protein_coding	2,89	0,0000000
ENSG00000146670	CDCA5	11	65066300	65084164	protein_coding	2,89	0,0037996
ENSG00000119397	CNTRL	9	121074863	121177610	protein_coding	2,89	0,0793786
ENSG00000083093	PALB2	16	23603160	23641310	protein_coding	2,89	0,0012807
ENSG00000186469	GNG2	14	51826195	51979342	protein_coding	2,89	0,0075075
ENSG00000213390	ARHGAP19	10	97222173	97292673	protein_coding	2,88	0,0075075
ENSG00000114790	ARHGEF26	3	154121003	154257827	protein_coding	2,88	0,1598647
ENSG00000135823	STX6	1	180972712	181023121	protein_coding	2,88	0,0012807
ENSG00000113558	SKP1	5	134148935	134177038	protein_coding	2,87	0,1598647
ENSG00000126226	PCID2	13	113177539	113208715	protein_coding	2,87	0,0012807
ENSG00000065600	TMEM206	1	212363931	212414901	protein_coding	2,87	0,0012807
ENSG00000100625	SIX4	14	60709528	60724348	protein_coding	2,87	0,5364729
ENSG00000159197	KCNE2	21	34364024	34371389	protein_coding	2,86	0,0586426
ENSG00000127903	ZNF835	19	56661981	56671783	protein_coding	2,86	0,0793786
ENSG00000162396	PARS2	1	54756898	54764514	protein_coding	2,86	0,0025348
ENSG00000140416	TPM1	15	63042632	63071915	protein_coding	2,85	0,0335607
ENSG00000132475	H3F3B	17	75776434	75785893	protein_coding	2,85	0,0037996
ENSG00000010539	ZNF200	16	3222325	3236221	protein_coding	2,85	0,0062591
ENSG00000166546	BEAN1	16	66427297	66493529	protein_coding	2,85	0,0995111
ENSG00000131931	THAP1	8	42836674	42843325	protein_coding	2,84	0,0184474
ENSG00000151552	QDPR	4	17460261	17512234	protein_coding	2,84	0,0062591
ENSG00000152348	ATG10	5	81972025	82276857	protein_coding	2,84	0,3269660
ENSG00000126249	PDCD2L	19	34404384	34426168	protein_coding	2,84	0,0793786
ENSG00000150556	LYPD6B	2	149038107	149215262	protein_coding	2,83	0,0717418
ENSG00000162076	FLYWCH2	16	2883186	2899382	protein_coding	2,83	0,4999662
ENSG00000205853	RFPL3S	22	32359906	32382052	protein_coding	2,82	0,0012807
ENSG00000250091	DNAH10OS	12	123926424	123934984	protein_coding	2,82	0,0006696
ENSG00000174720	LARP7	4	112636964	112657592	protein_coding	2,81	0,4463415
ENSG00000144895	EIF2A	3	150546678	150584242	protein_coding	2,80	0,1598647
ENSG00000133115	STOML3	13	38965925	38991066	protein_coding	2,80	0,3269660
ENSG00000187918	OR51I2	11	5453489	5454427	protein_coding	2,80	0,3269660
ENSG00000030304	MUSK	9	110668771	110801620	protein_coding	2,80	0,1245284
ENSG00000183148	ANKRD20A2	9	40223285	40266392	protein_coding	2,80	0,6142622
ENSG00000163666	HESX1	3	57197843	57226521	protein_coding	2,80	0,4463415
ENSG00000064933	PMS1	2	189784085	189877629	protein_coding	2,80	0,0037996
ENSG00000118655	DCLRE1B	1	113905141	113914086	protein_coding	2,79	0,0148097
ENSG00000131044	TTLL9	20	31870702	31944963	protein_coding	2,79	0,0012807
ENSG00000157554	ERG	21	38380027	38661780	protein_coding	2,79	0,0050271

ENSG00000100162	CENPM	22	41938721	41947164	protein_coding	2,78	0,1168222
ENSG00000148384	INPP5E	9	136428619	136439822	protein_coding	2,78	0,0050271
ENSG00000183091	NEB	2	151485336	151734487	protein_coding	2,78	0,0793786
ENSG00000204920	ZNF155	19	43967862	43998325	protein_coding	2,78	0,0050271
ENSG00000138279	ANXA7	10	73375101	73414076	protein_coding	2,78	0,0025348
ENSG00000047579	DTNBP1	6	15522801	15663058	protein_coding	2,78	0,0012807
ENSG00000130921	C12orf65	12	123232916	123257959	protein_coding	2,77	0,1245284
ENSG00000108733	PEX12	17	35574795	35578863	protein_coding	2,77	0,0184474
ENSG00000204116	CHIC1	X	73563200	73687102	protein_coding	2,77	0,2229774
ENSG00000169918	OTUD7A	15	31475398	31870789	protein_coding	2,76	0,2600087
ENSG00000114739	ACVR2B	3	38453851	38493142	protein_coding	2,76	0,0609506
ENSG00000100276	RASL10A	22	29312933	29319679	protein_coding	2,76	0,4307702
ENSG00000183723	CMTM4	16	66614750	66696707	protein_coding	2,75	0,0012807
ENSG00000136141	LRCH1	13	46553168	46753040	protein_coding	2,75	0,0000000
ENSG00000108684	ASIC2	17	33013087	34174964	protein_coding	2,75	0,0793786
ENSG00000197579	TOPORS	9	32540544	32552553	protein_coding	2,75	0,0995111
ENSG00000169087	HSPBAP1	3	122740003	122793824	protein_coding	2,74	0,0418702
ENSG00000140600	SH3GL3	15	83447228	83618743	protein_coding	2,74	0,0075075
ENSG00000204147	ASAH2B	10	50739318	50816495	protein_coding	2,74	0,0025348
ENSG00000173914	RBM4B	11	66664998	66677921	protein_coding	2,74	0,3785161
ENSG00000196976	LAGE3	X	154477769	154479257	protein_coding	2,74	0,0050271
ENSG00000166478	ZNF143	11	9460319	9528524	protein_coding	2,74	0,0012807
ENSG00000140368	PSTPIP1	15	76993359	77037332	protein_coding	2,74	0,0538744
ENSG00000185046	ANKS1B	12	98726457	99984654	protein_coding	2,73	0,0075075
ENSG00000184486	POU3F2	6	98834592	98839490	protein_coding	2,73	0,0793786
ENSG00000198740	ZNF652	17	49289206	49362473	protein_coding	2,72	0,2725752
ENSG00000211456	SACM1L	3	45689056	45745424	protein_coding	2,71	0,0609506
ENSG00000188001	TPRG1	3	188947214	189325304	protein_coding	2,71	0,0012807
ENSG00000133704	IPO8	12	30628988	30695986	protein_coding	2,71	0,0000000
ENSG00000172765	TMCC1	3	129647792	129893576	protein_coding	2,71	0,0012807
ENSG00000181472	ZBTB2	6	151364117	151391548	protein_coding	2,71	0,0025348
ENSG00000155827	RNF20	9	101533851	101563344	protein_coding	2,71	0,1245284
ENSG00000120526	NUDCD1	8	109240919	109334385	protein_coding	2,71	0,0012807
ENSG00000095585	BLNK	10	96191702	96271587	protein_coding	2,71	0,0509539
ENSG00000204389	HSPA1A	6	31815464	31817946	protein_coding	2,71	0,0995111
ENSG00000221947	XKR9	8	70669365	70790371	protein_coding	2,71	0,3785161
ENSG00000147257	GPC3	X	133535745	133985895	protein_coding	2,70	0,4999662
ENSG00000150527	CTAGE5	14	39265284	39388513	protein_coding	2,70	0,0025348
ENSG00000279219	ENSG000002	22	32191094	32191452	protein_coding	2,70	0,0025348
ENSG00000187791	FAM205C	9	34889064	34895778	protein_coding	2,70	0,3269660
ENSG00000128872	TMOD2	15	51751561	51816368	protein_coding	2,70	0,0037996
ENSG00000244405	ETV5	3	186046308	186110318	protein_coding	2,70	0,0111464
ENSG00000189195	BTBD8	1	92080305	92147836	protein_coding	2,70	0,2229774
ENSG00000100027	YPEL1	22	21697544	21735834	protein_coding	2,69	0,0050271
ENSG00000141837	CACNA1A	19	13206442	13623990	protein_coding	2,69	0,0418702
ENSG00000124659	TBCC	6	42744481	42746096	protein_coding	2,69	0,0793786
ENSG00000178105	DDX10	11	108665025	108940930	protein_coding	2,69	0,0995111
ENSG00000006530	AGK	7	141551189	141655244	protein_coding	2,69	0,0037996
ENSG00000235631	RNF148	7	122701664	122702967	protein_coding	2,69	0,5364729
ENSG00000172059	KLF11	2	10042849	10054836	protein_coding	2,68	0,0012807
ENSG00000203724	C1orf53	1	197902647	197907367	protein_coding	2,67	0,2229774
ENSG00000119946	CNNM1	10	99329099	99394330	protein_coding	2,67	0,0995111
ENSG00000151320	AKAP6	14	32329273	32837681	protein_coding	2,67	0,1598647
ENSG00000168497	SDPR	2	191834302	191847255	protein_coding	2,67	0,0050271
ENSG00000278311	GGNBP2	17	36544888	36589848	protein_coding	2,67	0,0609506

ENSG00000135148	TRAFD1	12	112125501	112153609	protein_coding	2,67	0,0012807
ENSG00000007038	PRSS21	16	2817180	2826304	protein_coding	2,67	0,5736135
ENSG00000100884	CPNE6	14	24070837	24078100	protein_coding	2,67	0,3963183
ENSG00000100246	DNAL4	22	38778508	38794198	protein_coding	2,66	0,0148097
ENSG00000198826	ARHGAP11A	15	32615144	32639949	protein_coding	2,66	0,0012807
ENSG00000008405	CRY1	12	106991364	107093829	protein_coding	2,66	0,0012807
ENSG00000055130	CUL1	7	148697914	148801036	protein_coding	2,66	0,0012807
ENSG00000157110	RBPMS	8	30384479	30572261	protein_coding	2,66	0,0050271
ENSG00000143498	TAF1A	1	222557902	222589933	protein_coding	2,66	0,0037996
ENSG00000169760	NLGN1	3	173396284	174286644	protein_coding	2,66	0,0075075
ENSG00000155085	AK9	6	109492856	109691217	protein_coding	2,65	0,0148097
ENSG00000034693	PEX3	6	143450807	143490010	protein_coding	2,65	0,5736135
ENSG00000185774	KCNIP4	4	20728616	21948799	protein_coding	2,65	0,0148097
ENSG00000106588	PSMA2	7	42916857	42932223	protein_coding	2,64	0,4307702
ENSG00000186446	ZNF501	3	44729596	44737083	protein_coding	2,64	0,0012807
ENSG00000152409	JMY	5	79236189	79327215	protein_coding	2,64	0,0012807
ENSG00000149929	HIRIP3	16	29992321	29996436	protein_coding	2,64	0,0257457
ENSG00000144644	GADL1	3	30726200	30894765	protein_coding	2,64	0,2229774
ENSG00000107951	MTPAP	10	30309801	30374448	protein_coding	2,63	0,0257457
ENSG00000086619	ERO1B	1	236215555	236281985	protein_coding	2,63	0,0995111
ENSG00000143858	SYT2	1	202590596	202710417	protein_coding	2,63	0,0075075
ENSG00000100906	NFKBIA	14	35401511	35404749	protein_coding	2,62	0,0025348
ENSG00000010318	PHF7	3	52410561	52423641	protein_coding	2,62	0,0105771
ENSG00000125245	GPR18	13	99254714	99261744	protein_coding	2,62	0,1867869
ENSG00000075429	CACNG5	17	66835117	66885486	protein_coding	2,62	0,5364729
ENSG00000152454	ZNF256	19	57940833	57947675	protein_coding	2,61	0,0037996
ENSG00000160404	TOR2A	9	127731524	127735317	protein_coding	2,61	0,0335607
ENSG00000119698	PPP4R4	14	94146128	94279735	protein_coding	2,61	0,0050271
ENSG00000133884	DPF2	11	65333754	65353249	protein_coding	2,60	0,0025348
ENSG00000274391	TPTE	21	10521553	10606140	protein_coding	2,60	0,6142622
ENSG00000161652	IZUMO2	19	50152548	50163195	protein_coding	2,60	0,6142622
ENSG00000178690	DYNAP	18	54587757	54599493	protein_coding	2,60	0,3269660
ENSG00000188716	DUPD1	10	75037836	75058514	protein_coding	2,60	0,6142622
ENSG00000171855	IFNB1	9	21077105	21077963	protein_coding	2,60	0,0111464
ENSG00000173612	GPRC6A	6	116792085	116829037	protein_coding	2,60	0,3269660
ENSG00000263020	ENSG000002	6	31666102	31673546	protein_coding	2,60	0,1245284
ENSG00000169783	LINGO1	15	77613027	77820900	protein_coding	2,60	0,6606414
ENSG00000158411	MITD1	2	99161427	99181058	protein_coding	2,60	0,0717418
ENSG00000145819	ARHGAP26	5	142770384	143229011	protein_coding	2,59	0,0012807
ENSG00000049759	NEDD4L	18	58044367	58401540	protein_coding	2,59	0,0025348
ENSG00000008324	SS18L2	3	42581840	42595114	protein_coding	2,59	0,0111464
ENSG00000113593	PPWD1	5	65563236	65587549	protein_coding	2,59	0,4463415
ENSG00000141639	MAPK4	18	50560078	50731824	protein_coding	2,59	0,0012807
ENSG00000092036	HAUS4	14	22946228	22957161	protein_coding	2,59	0,2725752
ENSG00000187231	SESTD1	2	179101692	179264790	protein_coding	2,58	0,0418702
ENSG00000105641	SLC5A5	19	17871973	17895174	protein_coding	2,58	0,3096079
ENSG00000188243	COMMD6	13	75525219	75549439	protein_coding	2,58	0,0995111
ENSG00000206559	ZCWPW2	3	28349146	28538122	protein_coding	2,58	0,0609506
ENSG00000205456	TP53TG3D	16	32252719	32255922	protein_coding	2,57	0,0586426
ENSG00000197943	PLCG2	16	81739097	81962693	protein_coding	2,57	0,0418702
ENSG00000116830	TTF2	1	117060303	117107453	protein_coding	2,56	0,2725752
ENSG00000075702	WDR62	19	36054881	36105106	protein_coding	2,55	0,0335607
ENSG00000105072	C19orf44	19	16496311	16521352	protein_coding	2,55	0,0012807
ENSG00000140009	ESR2	14	64084232	64338112	protein_coding	2,55	0,0105771
ENSG00000127564	PKMYT1	16	2968024	2980539	protein_coding	2,55	0,0586426

ENSG00000152128	TMEM163	2	134455759	134719000	protein_coding	2,55	0,0012807
ENSG00000101138	CSTF1	20	56392371	56406369	protein_coding	2,54	0,0012807
ENSG00000115295	CLIP4	2	29097705	29189643	protein_coding	2,54	0,0335607
ENSG00000140463	BBS4	15	72686179	72738476	protein_coding	2,54	0,0012807
ENSG00000066651	TRMT11	6	125986430	126039276	protein_coding	2,54	0,0418702
ENSG00000054392	HHAT	1	210328252	210676296	protein_coding	2,54	0,0012807
ENSG00000168038	ULK4	3	41246599	41962430	protein_coding	2,53	0,0050271
ENSG00000196683	TOMM7	7	22812628	22822852	protein_coding	2,53	0,0050271
ENSG00000157578	LCA5L	21	39405844	39445805	protein_coding	2,53	0,0335607
ENSG00000144619	CNTN4	3	2098813	3057956	protein_coding	2,53	0,0184474
ENSG00000128918	ALDH1A2	15	57953424	58497866	protein_coding	2,53	0,0012807
ENSG00000181915	ADO	10	62804857	62808483	protein_coding	2,53	0,3269660
ENSG00000103540	CCP110	16	19523811	19553408	protein_coding	2,52	0,0075075
ENSG00000171475	WIPF2	17	40219304	40284136	protein_coding	2,52	0,1775672
ENSG00000120262	CCDC170	6	151494030	151621193	protein_coding	2,51	0,0184474
ENSG00000164707	SLC13A4	7	135681237	135729258	protein_coding	2,51	0,1488645
ENSG00000144485	HES6	2	238238267	238240662	protein_coding	2,51	0,0335607
ENSG00000125851	PCSK2	20	17226107	17484578	protein_coding	2,50	0,3269660
ENSG00000134538	SLCO1B1	12	21131202	21239246	protein_coding	2,50	0,1245284
ENSG00000108018	SORCS1	10	106573663	107164534	protein_coding	2,50	0,0062591
ENSG00000082458	DLG3	X	70444861	70505490	protein_coding	2,50	0,0111464
ENSG00000177981	ASB8	12	48147788	48181213	protein_coding	2,49	0,0538744
ENSG00000132849	INADL	1	61742477	62178675	protein_coding	2,49	0,0037996
ENSG00000146592	CREB5	7	28299321	28825894	protein_coding	2,49	0,0025348
ENSG00000118496	FBXO30	6	145793502	145814753	protein_coding	2,49	0,4463415
ENSG00000182963	GJC1	17	44798448	44830816	protein_coding	2,49	0,0793786
ENSG00000009780	FAM76A	1	27725979	27763122	protein_coding	2,49	0,0335607
ENSG00000132388	UBE2G1	17	4269259	4366628	protein_coding	2,49	0,3963183
ENSG00000120334	CENPL	1	173799550	173824720	protein_coding	2,49	0,0111464
ENSG00000156787	TBC1D31	8	123041968	123152153	protein_coding	2,49	0,0012807
ENSG00000214595	EML6	2	54723499	54972025	protein_coding	2,48	0,0012807
ENSG00000144677	CTDSPL	3	37861960	37984469	protein_coding	2,48	0,3269660
ENSG00000151651	ADAM8	10	133262403	133276868	protein_coding	2,48	0,0335607
ENSG00000163754	GYG1	3	148991341	149027668	protein_coding	2,48	0,0050271
ENSG00000181007	ZFP82	19	36383120	36418656	protein_coding	2,48	0,0025348
ENSG00000133069	TMCC2	1	205228176	205273343	protein_coding	2,48	0,0111464
ENSG00000103044	HAS3	16	69105564	69118719	protein_coding	2,48	0,0148097
ENSG00000173275	ZNF449	X	135344796	135363152	protein_coding	2,48	0,0257457
ENSG00000159593	NAE1	16	66802875	66873256	protein_coding	2,47	0,1245284
ENSG00000170293	CMTM8	3	32238679	32370325	protein_coding	2,47	0,0012807
ENSG00000156298	TSPAN7	X	38561370	38688920	protein_coding	2,47	0,0335607
ENSG00000066185	ZMYND12	1	42430329	42456267	protein_coding	2,46	0,0538744
ENSG00000171649	ZIK1	19	57578456	57593777	protein_coding	2,46	0,0148097
ENSG00000141552	ANAPC11	17	81890790	81900991	protein_coding	2,46	0,0995111
ENSG00000138587	MNS1	15	56421544	56465137	protein_coding	2,46	0,4999662
ENSG00000248383	PCDHAC1	5	140926369	141012344	protein_coding	2,46	0,1488645
ENSG00000171772	SYCE1	10	133553901	133569835	protein_coding	2,45	0,3096079
ENSG00000198515	CNGA1	4	47935977	48016672	protein_coding	2,45	0,5364729
ENSG00000119929	CUTC	10	99702558	99756134	protein_coding	2,45	0,0418702
ENSG00000219438	FAM19A5	22	48489460	48850912	protein_coding	2,45	0,0793786
ENSG00000110958	PTGES3	12	56663341	56688408	protein_coding	2,45	0,0609506
ENSG00000117643	MAN1C1	1	25617468	25786207	protein_coding	2,44	0,1598647
ENSG00000115368	WDR75	2	189441433	189475565	protein_coding	2,44	0,0111464
ENSG00000122870	BICC1	10	58513140	58831437	protein_coding	2,44	0,0793786
ENSG00000204815	TTC25	17	41930635	41965651	protein_coding	2,44	0,4999662

ENSG00000110700	RPS13	11	17074389	17077787	protein_coding	2,43	0,0793786
ENSG00000153006	SREK1IP1	5	64718144	64768685	protein_coding	2,43	0,0995111
ENSG00000131196	NFATC1	18	79395856	79529325	protein_coding	2,43	0,0418702
ENSG00000118160	SLC8A2	19	47428017	47472168	protein_coding	2,42	0,1168222
ENSG00000180061	TMEM150B	19	55312801	55334048	protein_coding	2,42	0,5364729
ENSG00000100784	RPS6KA5	14	90847862	91060636	protein_coding	2,42	0,0257457
ENSG00000072864	NDE1	16	15643267	15726353	protein_coding	2,41	0,2725752
ENSG00000163995	ABLIM2	4	7965310	8158832	protein_coding	2,41	0,0609506
ENSG00000172995	ARPP21	3	35638945	35794496	protein_coding	2,40	0,1775672
ENSG00000205268	PDE7A	8	65717510	65842322	protein_coding	2,40	0,0025348
ENSG00000186509	OR9Q1	11	58023881	58181616	protein_coding	2,40	0,6142622
ENSG00000163746	PLSCR2	3	146391421	146495991	protein_coding	2,40	0,3269660
ENSG00000071677	PRLH	2	237566574	237567175	protein_coding	2,40	0,6142622
ENSG00000174306	ZHX3	20	41178448	41317672	protein_coding	2,40	0,0609506
ENSG00000051180	RAD51	15	40694774	40732339	protein_coding	2,40	0,0062591
ENSG00000138028	CGREF1	2	27098889	27119115	protein_coding	2,40	0,1598647
ENSG00000232040	ZBED9	6	28571630	28616212	protein_coding	2,39	0,0037996
ENSG00000154429	CCSAP	1	229321005	229343294	protein_coding	2,39	0,0995111
ENSG00000143156	NME7	1	169132531	169367967	protein_coding	2,38	0,3963183
ENSG00000163492	CCDC141	2	178829757	179050086	protein_coding	2,38	0,0335607
ENSG00000077943	ITGA8	10	15513949	15720125	protein_coding	2,38	0,0184474
ENSG00000031691	CENPQ	6	49463378	49493107	protein_coding	2,38	0,2725752
ENSG00000182253	SYNM	15	99098217	99135593	protein_coding	2,37	0,2229774
ENSG00000164919	COX6C	8	99873200	99894062	protein_coding	2,37	0,2229774
ENSG00000213988	ZNF90	19	20077994	20127076	protein_coding	2,37	0,0037996
ENSG00000165995	CACNB2	10	18140677	18541869	protein_coding	2,36	0,5736135
ENSG00000151150	ANK3	10	60026298	60733490	protein_coding	2,36	0,0538744
ENSG00000107295	SH3GL2	9	17579082	17797129	protein_coding	2,36	0,1598647
ENSG00000125351	UPF3B	X	119834022	119852998	protein_coding	2,36	0,0793786
ENSG00000047346	FAM214A	15	52581317	52709817	protein_coding	2,36	0,0111464
ENSG00000155011	DKK2	4	106921802	107283806	protein_coding	2,36	0,0586426
ENSG00000131462	TUBG1	17	42609676	42615234	protein_coding	2,36	0,0037996
ENSG00000118997	DNAH7	2	195737703	196068812	protein_coding	2,36	0,0184474
ENSG00000021300	PLEKHB1	11	73646178	73662819	protein_coding	2,35	0,0111464
ENSG00000023330	ALAS1	3	52198086	52214327	protein_coding	2,35	0,0335607
ENSG00000101871	MID1	X	10445310	10833654	protein_coding	2,35	0,0025348
ENSG00000171316	CHD7	8	60678778	60868028	protein_coding	2,34	0,1245284
ENSG00000205659	LIN52	14	74084796	74201235	protein_coding	2,33	0,1598647
ENSG00000212935	KRTAP10-3	21	44557790	44558760	protein_coding	2,33	0,0995111
ENSG00000174990	CA5A	16	87881546	87936529	protein_coding	2,33	0,1488645
ENSG00000204538	PSORS1C2	6	31137536	31139350	protein_coding	2,33	0,3269660
ENSG00000185055	EFCAB10	7	105565120	105600875	protein_coding	2,33	0,0075075
ENSG00000144730	IL17RD	3	57089982	57170306	protein_coding	2,33	0,0012807
ENSG00000130255	RPL36	19	5674947	5691876	protein_coding	2,33	0,0257457
ENSG00000196116	TDRD7	9	97411950	97496125	protein_coding	2,33	0,0037996
ENSG00000010810	FYN	6	111660332	111873452	protein_coding	2,33	0,2229774
ENSG00000276234	TADA2A	17	37406874	37479730	protein_coding	2,32	0,0609506
ENSG00000181773	GPR3	1	27392644	27395814	protein_coding	2,32	0,0609506
ENSG00000120539	MASTL	10	27154824	27186924	protein_coding	2,32	0,0184474
ENSG00000132964	CDK8	13	26254104	26405238	protein_coding	2,32	0,0257457
ENSG00000070886	EPHA8	1	22563564	22603594	protein_coding	2,31	0,4307702
ENSG00000147854	UHRF2	9	6413151	6507054	protein_coding	2,30	0,0257457
ENSG00000049656	CLPTM1L	5	1317744	1345099	protein_coding	2,30	0,0148097
ENSG00000070814	TCOF1	5	150357639	150400308	protein_coding	2,30	0,4463415
ENSG00000108468	CBX1	17	48070052	48101521	protein_coding	2,30	0,0335607

ENSG00000128253	RFPL2	22	32190435	32203477	protein_coding	2,30	0,6702615
ENSG00000163286	ALPPL2	2	232406843	232410714	protein_coding	2,30	0,3269660
ENSG00000148053	NTRK2	9	84668551	85027070	protein_coding	2,29	0,0111464
ENSG00000128487	SPECC1	17	20009344	20319026	protein_coding	2,29	0,0025348
ENSG00000121073	SLC35B1	17	49700943	49709014	protein_coding	2,29	0,0037996
ENSG00000106052	TAX1BP1	7	27739331	27844564	protein_coding	2,29	0,0418702
ENSG00000249222	ATP5L2	22	42639803	42640601	protein_coding	2,29	0,6003007
ENSG00000215182	MUC5AC	11	1157953	1201138	protein_coding	2,29	0,3096079
ENSG00000130559	CAMSAP1	9	135808487	135907228	protein_coding	2,28	0,0012807
ENSG00000204947	ZNF425	7	149102784	149126346	protein_coding	2,28	0,1598647
ENSG00000058272	PPP1R12A	12	79773563	79935460	protein_coding	2,28	0,0538744
ENSG00000005194	CIAPIN1	16	57428169	57447528	protein_coding	2,27	0,0025348
ENSG00000140873	ADAMTS18	16	77247813	77435114	protein_coding	2,27	0,0075075
ENSG00000125482	TTF1	9	132375548	132406851	protein_coding	2,27	0,0609506
ENSG00000100711	ZFYVE21	14	103715730	103733668	protein_coding	2,27	0,0050271
ENSG00000115112	TFCP2L1	2	121216587	121285207	protein_coding	2,26	0,1245284
ENSG00000155858	LSM11	5	157743695	157760709	protein_coding	2,26	0,2725752
ENSG00000113319	RASGRF2	5	80960672	81230156	protein_coding	2,26	0,0050271
ENSG00000186472	PCLO	7	82754013	83162930	protein_coding	2,26	0,0012807
ENSG00000144868	TMEM108	3	133038391	133397792	protein_coding	2,25	0,0793786
ENSG00000006128	TAC1	7	97731908	97740472	protein_coding	2,25	0,3963183
ENSG00000139354	GAS2L3	12	100573683	100628286	protein_coding	2,25	0,6702615
ENSG00000136161	RCBTB2	13	48488959	48533256	protein_coding	2,25	0,0257457
ENSG00000143353	LYPLAL1	1	219173844	219212865	protein_coding	2,24	0,2725752
ENSG00000204390	HSPA1L	6	31809619	31815065	protein_coding	2,24	0,6606414
ENSG00000184983	NDUFA6	22	42085525	42090955	protein_coding	2,24	0,2725752
ENSG00000126246	IGFLR1	19	35738801	35742453	protein_coding	2,24	0,0148097
ENSG00000138964	PARVG	22	44172956	44219533	protein_coding	2,24	0,0075075
ENSG00000188396	TCTEX1D4	1	44805913	44806675	protein_coding	2,24	0,0075075
ENSG00000105732	ZNF574	19	42068477	42081565	protein_coding	2,24	0,3963183
ENSG00000213190	MLLT11	1	151057758	151068497	protein_coding	2,23	0,0793786
ENSG00000171056	SOX7	8	10723768	10730512	protein_coding	2,23	0,0586426
ENSG00000145416	01-mars	4	163524298	164384050	protein_coding	2,22	0,0418702
ENSG00000109445	ZNF330	4	141220887	141234697	protein_coding	2,22	0,0184474
ENSG00000156574	NODAL	10	70432315	70447951	protein_coding	2,22	0,0995111
ENSG00000168890	TMEM150A	2	85598548	85603196	protein_coding	2,22	0,0075075
ENSG00000121989	ACVR2A	2	147844517	147930826	protein_coding	2,22	0,0025348
ENSG00000205209	SCGB2B2	19	34593329	34675699	protein_coding	2,22	0,6702615
ENSG00000152689	RASGRP3	2	33436324	33564750	protein_coding	2,22	0,0075075
ENSG00000076321	KLHL20	1	173714941	173786702	protein_coding	2,21	0,0538744
ENSG00000112149	CD83	6	14117256	14136918	protein_coding	2,21	0,0184474
ENSG00000107249	GLIS3	9	3824127	4348392	protein_coding	2,21	0,3269660
ENSG00000100473	COCH	14	30874514	30895065	protein_coding	2,21	0,0609506
ENSG00000175093	SPSB4	3	141051402	141148611	protein_coding	2,21	0,6003007
ENSG00000173013	CCDC96	4	7040849	7042939	protein_coding	2,21	0,2600087
ENSG00000197472	ZNF695	1	246945547	247008093	protein_coding	2,21	0,2725752
ENSG00000108244	KRT23	17	40922696	40937634	protein_coding	2,21	0,4999662
ENSG00000164713	BRI3	7	98252379	98310441	protein_coding	2,21	0,0335607
ENSG00000163521	GLB1L	2	219236606	219245478	protein_coding	2,20	0,0793786
ENSG00000196646	ZNF136	19	12163064	12189881	protein_coding	2,20	0,5736135
ENSG00000187689	AMTN	4	70518540	70532742	protein_coding	2,20	0,6142622
ENSG00000114204	SERPINI2	3	167441789	167479004	protein_coding	2,20	0,1245284
ENSG00000066583	ISOC1	5	129094751	129114028	protein_coding	2,20	0,0995111
ENSG00000128242	GAL3ST1	22	30554635	30574587	protein_coding	2,19	0,1488645
ENSG00000152380	FAM151B	5	80487969	80542563	protein_coding	2,19	0,4999662

ENSG00000182117	NOP10	15	34341713	34343177	protein_coding	2,19	0,0538744
ENSG00000166526	ZNF3	7	100064033	100082548	protein_coding	2,19	0,0148097
ENSG00000105649	RAB3A	19	18196784	18204074	protein_coding	2,18	0,0148097
ENSG00000125703	ATG4C	1	62784135	62865513	protein_coding	2,18	0,4999662
ENSG00000141384	TAF4B	18	26225936	26391685	protein_coding	2,18	0,3963183
ENSG00000153574	RPIA	2	88691644	88750935	protein_coding	2,18	0,0538744
ENSG00000158423	RIBC1	X	53422690	53431120	protein_coding	2,18	0,4808196
ENSG00000112592	TBP	6	170554302	170572870	protein_coding	2,18	0,0062591
ENSG00000169758	TMEM266	15	76059837	76229121	protein_coding	2,17	0,2127456
ENSG00000135972	MRPS9	2	105037983	105099960	protein_coding	2,17	0,0037996
ENSG00000039560	RAI14	5	34656237	34832627	protein_coding	2,17	0,0037996
ENSG00000183647	ZNF530	19	57599885	57612722	protein_coding	2,17	0,6899576
ENSG00000116213	WRAP73	1	3630767	3652761	protein_coding	2,17	0,0257457
ENSG00000214941	ZSWIM7	17	15976560	15999717	protein_coding	2,16	0,0418702
ENSG00000119688	ABCD4	14	74285423	74303056	protein_coding	2,16	0,0335607
ENSG00000101040	ZMYND8	20	47209214	47356889	protein_coding	2,16	0,2725752
ENSG00000196368	NUDT11	X	51490011	51496596	protein_coding	2,16	0,0300092
ENSG00000151693	ASAP2	2	9206765	9405683	protein_coding	2,16	0,0586426
ENSG00000140153	WDR20	14	102139503	102224847	protein_coding	2,15	0,0075075
ENSG00000169876	MUC17	7	101020072	101058745	protein_coding	2,15	0,3096079
ENSG00000124126	PREX1	20	48624252	48827883	protein_coding	2,15	0,3963183
ENSG00000164002	EXO5	1	40508741	40516556	protein_coding	2,15	0,0184474
ENSG00000176927	EFCAB5	17	29929200	30108452	protein_coding	2,15	0,2127456
ENSG00000136940	PDCL	9	122798389	122828631	protein_coding	2,15	0,0012807
ENSG00000168350	DEGS2	14	100143957	100160163	protein_coding	2,15	0,4307702
ENSG00000183439	TRIM61	4	164954446	164977668	protein_coding	2,14	0,1168222
ENSG00000187815	ZFP69	1	40477215	40496343	protein_coding	2,14	0,0257457
ENSG00000111596	CNOT2	12	70242994	70354993	protein_coding	2,14	0,1867869
ENSG00000171960	PPIH	1	42658425	42676758	protein_coding	2,14	0,0995111
ENSG00000129071	MBD4	3	129430944	129440179	protein_coding	2,14	0,0111464
ENSG00000106443	PHF14	7	10973872	11169630	protein_coding	2,14	0,0609506
ENSG00000169612	FAM103A1	15	82986207	82991057	protein_coding	2,13	0,0609506
ENSG00000137656	BUD13	11	116748170	116772988	protein_coding	2,13	0,0025348
ENSG00000078304	PPP2R5C	14	101761798	101927989	protein_coding	2,13	0,0184474
ENSG00000140807	NKD1	16	50548330	50640739	protein_coding	2,12	0,0012807
ENSG00000117697	NSL1	1	212726153	212791782	protein_coding	2,12	0,0418702
ENSG00000162976	PQLC3	2	11155198	11178874	protein_coding	2,12	0,4999662
ENSG00000065675	PRKCQ	10	6427143	6580301	protein_coding	2,12	0,0075075
ENSG00000136960	ENPP2	8	119557086	119673453	protein_coding	2,12	0,0257457
ENSG00000070018	LRP6	12	12116025	12267012	protein_coding	2,12	0,0793786
ENSG00000076641	PAG1	8	80967810	81112068	protein_coding	2,11	0,0538744
ENSG00000229859	PGA3	11	61203307	61213098	protein_coding	2,11	0,3785161
ENSG00000106993	CDC37L1	9	4679559	4708398	protein_coding	2,11	0,0111464
ENSG00000110002	VWA5A	11	124115362	124147721	protein_coding	2,11	0,0012807
ENSG00000187109	NAP1L1	12	76036587	76085033	protein_coding	2,11	0,2229774
ENSG00000183134	PTGDR2	11	60850940	60855971	protein_coding	2,10	0,4307702
ENSG00000187258	NPSR1	7	34658239	34878332	protein_coding	2,10	0,0418702
ENSG00000278637	HIST1H4A	6	26021679	26021990	protein_coding	2,10	0,3269660
ENSG00000088726	TMEM40	3	12733525	12769457	protein_coding	2,10	0,6142622
ENSG00000020922	MRE11A	11	94415578	94493908	protein_coding	2,10	0,0418702
ENSG00000086475	SEPHS1	10	13317424	13348298	protein_coding	2,10	0,0050271
ENSG00000158417	EIF5B	2	99337353	99401326	protein_coding	2,10	0,0184474
ENSG00000171827	ZNF570	19	37467585	37488652	protein_coding	2,10	0,0062591
ENSG00000126216	TUBGCP3	13	112485005	112588167	protein_coding	2,10	0,0012807
ENSG00000101146	RAE1	20	57351010	57379211	protein_coding	2,09	0,0062591

ENSG00000138101	DTNB	2	25377198	25673647	protein_coding	2,09	0,0111464
ENSG00000204524	ZNF805	19	57240685	57255135	protein_coding	2,09	0,0184474
ENSG00000138395	CDK15	2	201790461	201895550	protein_coding	2,09	0,0538744
ENSG00000118276	B4GALT6	18	31622247	31685836	protein_coding	2,09	0,2229774
ENSG00000152049	KCNE4	2	223051814	223198399	protein_coding	2,08	0,4999662
ENSG00000163026	C2orf44	2	24029340	24049575	protein_coding	2,08	0,0995111
ENSG00000138286	FAM149B1	10	73168166	73244504	protein_coding	2,08	0,2725752
ENSG00000197168	NEK5	13	52033611	52129078	protein_coding	2,08	0,0111464
ENSG00000255150	EID3	12	104303739	104305202	protein_coding	2,07	0,0062591
ENSG00000107485	GATA3	10	8053604	8075198	protein_coding	2,07	0,0793786
ENSG00000111877	MCM9	6	118813442	118935162	protein_coding	2,07	0,4999662
ENSG00000087338	GMCL1	2	69829642	69881396	protein_coding	2,07	0,0025348
ENSG00000146122	DAAM2	6	39792298	39904877	protein_coding	2,06	0,1867869
ENSG00000171450	CDK5R2	2	218959655	218962162	protein_coding	2,06	0,0793786
ENSG00000170315	UBB	17	16380798	16382745	protein_coding	2,06	0,0075075
ENSG00000101182	PSMA7	20	62136735	62143440	protein_coding	2,06	0,5736135
ENSG00000166192	SENP8	15	72114258	72143688	protein_coding	2,06	0,0050271
ENSG00000173145	NOC3L	10	94333226	94362959	protein_coding	2,06	0,4463415
ENSG00000177058	SLC38A9	5	55625845	55773194	protein_coding	2,05	0,1598647
ENSG00000164597	COG5	7	107201555	107564514	protein_coding	2,05	0,0037996
ENSG00000173226	IQCB1	3	121769763	121835079	protein_coding	2,05	0,0148097
ENSG00000173113	TRMT112	11	64316460	64318084	protein_coding	2,05	0,0111464
ENSG00000101187	SLCO4A1	20	62642445	62685785	protein_coding	2,04	0,6899576
ENSG00000127720	METTL25	12	82358497	82479236	protein_coding	2,04	0,2229774
ENSG00000101412	E2F1	20	33675683	33686404	protein_coding	2,04	0,1775672
ENSG00000103599	IQCH	15	67254800	67502260	protein_coding	2,04	0,0793786
ENSG00000092871	RFFL	17	35005990	35089319	protein_coding	2,04	0,2725752
ENSG00000089048	ESF1	20	13714322	13784886	protein_coding	2,04	0,4999662
ENSG00000169193	CCDC126	7	23597379	23644708	protein_coding	2,03	0,0538744
ENSG00000205683	DPF3	14	72619296	72894116	protein_coding	2,03	0,1598647
ENSG00000147654	EBAG9	8	109539711	109565996	protein_coding	2,03	0,2229774
ENSG00000123064	DDX54	12	113157174	113185479	protein_coding	2,03	0,6142622
ENSG00000242866	STRC	15	43599398	43618800	protein_coding	2,03	0,6003007
ENSG00000023608	SNAPC1	14	61762357	61796428	protein_coding	2,03	0,6702615
ENSG00000158201	ABHD3	18	21650897	21704805	protein_coding	2,02	0,0793786
ENSG00000149089	APIP	11	34853094	34916499	protein_coding	2,02	0,3963183
ENSG00000083097	DOPEY1	6	83067666	83171350	protein_coding	2,02	0,2229774
ENSG00000187959	CPSF4L	17	73248449	73262352	protein_coding	2,02	0,0012807
ENSG00000145075	CCDC39	3	180602858	180871005	protein_coding	2,02	0,0538744
ENSG00000088448	ANKRD10	13	110878540	110915069	protein_coding	2,02	0,0148097
ENSG00000101224	CDC25B	20	3786772	3806121	protein_coding	2,02	0,0609506
ENSG00000136108	CKAP2	13	52455429	52476628	protein_coding	2,02	0,0037996
ENSG00000103174	NAGPA	16	5024844	5034141	protein_coding	2,02	0,0075075
ENSG00000063245	EPN1	19	55675226	55709858	protein_coding	2,02	0,0995111
ENSG00000100567	PSMA3	14	58244831	58272012	protein_coding	2,02	0,1245284
ENSG00000154678	PDE1C	7	31751179	32299329	protein_coding	2,02	0,1245284
ENSG00000181481	RNF135	17	30968785	30999911	protein_coding	2,01	0,0050271
ENSG00000117020	AKT3	1	243488233	243851079	protein_coding	2,01	0,0184474
ENSG00000153786	ZDHHC7	16	84974181	85011535	protein_coding	2,01	0,0050271
ENSG00000179021	C3orf38	3	88149743	88168729	protein_coding	2,01	0,0012807
ENSG00000196104	SPOCK3	4	166733384	167234796	protein_coding	2,00	0,0538744
ENSG00000205856	C22orf42	22	32149006	32159322	protein_coding	2,00	0,6142622
ENSG00000196218	RYR1	19	38433699	38587564	protein_coding	2,00	0,5364729
ENSG00000185974	GRK1	13	113667155	113737735	protein_coding	2,00	0,5736135
ENSG00000254466	OR4D10	11	59477373	59478396	protein_coding	2,00	0,6142622

ENSG00000170279	C7orf33	7	148590565	148615860	protein_coding	2,00	0,3269660
ENSG00000272514	CFAP206	6	87407983	87464465	protein_coding	2,00	0,1245284
ENSG00000163376	KBTBD8	3	66998307	67011210	protein_coding	2,00	0,0793786
ENSG00000173699	SPATA3	2	230996121	231025055	protein_coding	2,00	0,1245284

Supplementary Table S2: List of the 9,045 protein-coding genes upregulated in cumulus granulosa cells

Gene ID	Gene Name	Chromosome	Start_position	End_position	Gene_biotype	Fold Change	q-value(%)
ENSG00000099194	SCD	10	100347124	100364834	protein_coding	62207,91	0
ENSG00000147465	STAR	8	38143649	38151265	protein_coding	48811,50	0
ENSG00000166710	B2M	15	44711477	44718877	protein_coding	43073,00	0
ENSG00000142798	HSPG2	1	21822245	21937297	protein_coding	36646,63	0
ENSG00000152661	GJA1	6	121435692	121449727	protein_coding	33062,80	0
ENSG00000117594	HSD11B1	1	209686178	209734950	protein_coding	30510,33	0
ENSG00000102038	SMARCA1	X	129446501	129523500	protein_coding	30296,38	0
ENSG00000106366	SERPINE1	7	101127089	101139266	protein_coding	29155,10	0
ENSG00000041982	TNC	9	115019578	115118257	protein_coding	28368,50	0
ENSG00000023171	GRAMD1B	11	123454398	123627774	protein_coding	26643,80	0
ENSG00000187498	COL4A1	13	110148963	110307149	protein_coding	25779,40	0
ENSG00000142494	SLC47A1	17	19495385	19579034	protein_coding	23234,10	0
ENSG00000124942	AHNAK	11	62433542	62556235	protein_coding	22059,55	0
ENSG00000172201	ID4	6	19837386	19840684	protein_coding	20956,70	0
ENSG00000185070	FLRT2	14	85530144	85654426	protein_coding	20326,80	0
ENSG00000197747	S100A10	1	151982915	151994390	protein_coding	18689,91	0
ENSG00000140459	CYP11A1	15	74337759	74367740	protein_coding	18656,10	0
ENSG00000147872	PLIN2	9	19108375	19149290	protein_coding	18403,75	0
ENSG00000140279	DUOX2	15	45092650	45114344	protein_coding	16442,30	0
ENSG00000203859	HSD3B2	1	119414931	119423035	protein_coding	16001,40	0
ENSG00000120885	CLU	8	27596917	27615031	protein_coding	14190,94	0
ENSG00000073756	PTGS2	1	186671791	186680427	protein_coding	14000,90	0
ENSG00000177606	JUN	1	58780788	58784327	protein_coding	13614,60	0
ENSG00000154734	ADAMTS1	21	26835747	26845409	protein_coding	13530,80	0
ENSG00000143878	RHOB	2	20447074	20449445	protein_coding	13305,70	0
ENSG00000169908	TM4SF1	3	149369022	149377865	protein_coding	13015,20	0
ENSG00000034510	TMSB10	2	84905625	84906675	protein_coding	12722,91	0
ENSG00000182718	ANXA2	15	60347134	60402883	protein_coding	12628,91	0
ENSG00000137801	THBS1	15	39581079	39599466	protein_coding	12458,67	0
ENSG00000123999	INHA	2	219569162	219575713	protein_coding	12421,42	0
ENSG00000102271	KLHL4	X	87517749	87670050	protein_coding	12077,60	0
ENSG00000204262	COL5A2	2	189031896	189179879	protein_coding	12073,30	0
ENSG00000172037	LAMB2	3	49121114	49133118	protein_coding	11669,30	0
ENSG00000123560	PLP1	X	103773718	103792619	protein_coding	11373,36	0
ENSG00000139329	LUM	12	91102629	91111831	protein_coding	11134,50	0
ENSG00000167460	TPM4	19	16067021	16103005	protein_coding	10791,00	0
ENSG00000108679	LGALS3BP	17	78971238	78980109	protein_coding	10724,00	0
ENSG00000102265	TIMP1	X	47582313	47586789	protein_coding	10633,57	0
ENSG00000187942	LDLRAD2	1	21812265	21825221	protein_coding	10493,20	0
ENSG00000204525	HLA-C	6	31268749	31272130	protein_coding	10441,10	0
ENSG00000115594	IL1R1	2	102064544	102179874	protein_coding	10398,00	0
ENSG00000099250	NRP1	10	33177492	33336262	protein_coding	10000,60	0
ENSG00000115380	EFEMP1	2	55865967	55924139	protein_coding	9517,73	0
ENSG00000113657	DPYSL3	5	147390811	147510056	protein_coding	9269,66	0
ENSG00000122863	CHST3	10	71964365	72013564	protein_coding	9073,80	0
ENSG00000166033	HTRA1	10	122461525	122514908	protein_coding	8813,00	0
ENSG00000113140	SPARC	5	151661096	151687165	protein_coding	8733,44	0
ENSG00000141756	FKBP10	17	41812680	41823217	protein_coding	8639,80	0
ENSG00000163430	FSTL1	3	120392293	120451253	protein_coding	8486,80	0
ENSG00000163661	PTX3	3	157436789	157443628	protein_coding	8423,40	0
ENSG00000060982	BCAT1	12	24810022	24949459	protein_coding	8184,26	0
ENSG00000112936	C7	5	40909252	40982939	protein_coding	8080,40	0
ENSG00000164125	FAM198B	4	158124474	158173318	protein_coding	8018,60	0
ENSG00000102316	MAGED2	X	54807599	54816012	protein_coding	7970,71	0
ENSG00000187479	C11orf96	11	43925342	43944338	protein_coding	7810,70	0

ENSG00000107742	SPOCK2	10	72059035	72089032	protein_coding	7399,20	0
ENSG00000206503	HLA-A	6	29941260	29945884	protein_coding	7260,50	0
ENSG00000141448	GATA6	18	22169443	22202528	protein_coding	7144,30	0
ENSG00000014257	ACPP	3	132317367	132368298	protein_coding	7042,90	0
ENSG00000159403	C1R	12	7080209	7092607	protein_coding	7028,40	0
ENSG00000092421	SEMA6A	5	116443616	116574934	protein_coding	6855,00	0
ENSG00000164171	ITGA2	5	52989326	53094779	protein_coding	6819,27	0
ENSG00000134285	FKBP11	12	48921518	48926474	protein_coding	6766,55	0
ENSG00000105825	TFPI2	7	93885397	93890991	protein_coding	6753,20	0
ENSG00000110148	CCKBR	11	6259736	6272127	protein_coding	6451,40	0
ENSG00000171223	JUNB	19	12791496	12793315	protein_coding	6423,40	0
ENSG00000272398	CD24	6	106969831	106975627	protein_coding	6392,18	0
ENSG00000156515	HK1	10	69269984	69401882	protein_coding	6386,00	0
ENSG00000147065	MSN	X	65588377	65741931	protein_coding	6351,90	0
ENSG00000234745	HLA-B	6	31353872	31357188	protein_coding	6338,35	0
ENSG00000122862	SRGN	10	69088106	69104811	protein_coding	6237,80	0
ENSG00000135404	CD63	12	55725323	55729707	protein_coding	6135,70	0
ENSG00000145730	PAM	5	102753981	103031105	protein_coding	6069,64	0
ENSG00000177469	PTRF	17	42402452	42423517	protein_coding	5805,60	0
ENSG00000101439	CST3	20	23626706	23638473	protein_coding	5799,80	0
ENSG00000179388	EGR3	8	22687659	22693302	protein_coding	5727,30	0
ENSG00000124813	RUNX2	6	45328157	45664349	protein_coding	5722,20	0
ENSG00000185650	ZFP36L1	14	68787660	68796253	protein_coding	5653,53	0
ENSG00000186854	TRABD2A	2	84821650	84907008	protein_coding	5620,10	0
ENSG00000136717	BIN1	2	127048027	127107355	protein_coding	5595,90	0
ENSG00000166681	NGFRAP1	X	103376340	103378077	protein_coding	5590,80	0
ENSG00000089199	CHGB	20	5911430	5925361	protein_coding	5580,60	0
ENSG00000171303	KCNK3	2	26692690	26733420	protein_coding	5563,20	0
ENSG00000113389	NPR3	5	32689070	32791724	protein_coding	5368,27	0
ENSG00000204592	HLA-E	6	30489467	30494205	protein_coding	5363,50	0
ENSG00000108821	COL1A1	17	50183289	50201632	protein_coding	5333,00	0
ENSG00000114115	RBP1	3	139517434	139539829	protein_coding	5199,78	0
ENSG00000130203	APOE	19	44905754	44909393	protein_coding	5037,16	0
ENSG00000122574	WIPF3	7	29806486	29917066	protein_coding	5009,20	0
ENSG00000011465	DCN	12	91140484	91183123	protein_coding	4997,10	0
ENSG00000064666	CNN2	19	1026581	1039068	protein_coding	4926,60	0
ENSG00000049449	RCN1	11	31812391	32105755	protein_coding	4814,20	0
ENSG00000188643	S100A16	1	153606886	153613145	protein_coding	4730,86	0
ENSG00000084628	NKAIN1	1	31179745	31239554	protein_coding	4664,90	0
ENSG00000111716	LDHB	12	21635342	21757857	protein_coding	4602,56	0
ENSG00000130202	PVRL2	19	44846175	44889228	protein_coding	4563,00	0
ENSG00000168461	RAB31	18	9708165	9862551	protein_coding	4447,40	0
ENSG00000145284	SCD5	4	82629539	82798857	protein_coding	4437,20	0
ENSG00000185222	WBP5	X	103356445	103358469	protein_coding	4418,80	0
ENSG00000178695	KCTD12	13	76880166	76886390	protein_coding	4339,10	0
ENSG00000116133	DHCR24	1	54849633	54887218	protein_coding	4260,04	0
ENSG00000148344	PTGES	9	129738331	129753047	protein_coding	4131,09	0
ENSG00000106236	NPTX2	7	98617297	98629868	protein_coding	4092,69	0
ENSG00000250722	SEPP1	5	42799880	42887392	protein_coding	4033,60	0
ENSG00000050165	DKK3	11	11963106	12009769	protein_coding	4031,80	0
ENSG00000024422	EHD2	19	47713343	47743134	protein_coding	3968,70	0
ENSG00000114771	AADAC	3	151814037	151828488	protein_coding	3931,00	0
ENSG00000079432	CIC	19	42268537	42295797	protein_coding	3930,50	0
ENSG00000256235	SMIM3	5	150777946	150796734	protein_coding	3919,50	0
ENSG00000184731	FAM110C	2	38814	46870	protein_coding	3918,90	0
ENSG00000136002	ARHGEF4	2	130836916	131047263	protein_coding	3910,10	0
ENSG00000108551	RASD1	17	17494437	17496395	protein_coding	3907,92	0
ENSG00000112715	VEGFA	6	43770184	43786487	protein_coding	3907,20	0

ENSG00000134824	FADS2	11	61792980	61867354	protein_coding	3905,42	0
ENSG00000133169	BEX1	X	103062651	103064240	protein_coding	3847,90	0
ENSG00000102359	SRPX2	X	100644218	100671299	protein_coding	3808,50	0
ENSG00000142089	IFITM3	11	319669	327537	protein_coding	3711,46	0
ENSG00000101335	MYL9	20	36541484	36551447	protein_coding	3684,30	0
ENSG00000134352	IL6ST	5	55935095	55994993	protein_coding	3643,50	0
ENSG00000108786	HSD17B1	17	42549214	42555213	protein_coding	3632,00	0
ENSG00000149257	SERPINH1	11	75562056	75572783	protein_coding	3600,21	0
ENSG00000168542	COL3A1	2	188974320	189012746	protein_coding	3584,70	0
ENSG00000075618	FSCN1	7	5592823	5606655	protein_coding	3536,20	0
ENSG00000141753	IGFBP4	17	40443461	40457731	protein_coding	3518,70	0
ENSG00000176978	DPP7	9	137110542	137115177	protein_coding	3509,91	0
ENSG00000154930	ACSS1	20	25006230	25058980	protein_coding	3506,60	0
ENSG00000083444	PLOD1	1	11934205	11975538	protein_coding	3475,63	0
ENSG00000124882	EREG	4	74365143	74388751	protein_coding	3450,20	0
ENSG00000115641	FHL2	2	105357712	105438513	protein_coding	3418,64	0
ENSG00000135272	MDFIC	7	114922154	115019916	protein_coding	3401,20	0
ENSG00000106624	AEBP1	7	44104361	44114562	protein_coding	3396,60	0
ENSG00000116983	HPCAL4	1	39678648	39691689	protein_coding	3394,75	0
ENSG00000169116	PARM1	4	74933095	75050115	protein_coding	3389,80	0
ENSG00000197956	S100A6	1	153534599	153536244	protein_coding	3361,25	0
ENSG00000135424	ITGA7	12	55684568	55716043	protein_coding	3316,27	0
ENSG00000122641	INHBA	7	41685114	41703108	protein_coding	3305,70	0
ENSG00000171227	TMEM37	2	119429901	119438520	protein_coding	3271,30	0
ENSG00000165757	KIAA1462	10	30012800	30115494	protein_coding	3270,78	0
ENSG00000163565	IFI16	1	158999968	159055155	protein_coding	3195,90	0
ENSG00000109072	VTN	17	28367276	28373091	protein_coding	3192,30	0
ENSG00000140961	OSGIN1	16	83948282	83966332	protein_coding	3176,30	0
ENSG00000167601	AXL	19	41219203	41261766	protein_coding	3170,50	0
ENSG00000139514	SLC7A1	13	29509410	29595688	protein_coding	3146,70	0
ENSG00000053747	LAMA3	18	23689443	23955066	protein_coding	3119,80	0
ENSG00000000971	CFH	1	196651878	196747504	protein_coding	3083,60	0
ENSG00000067182	TNFRSF1A	12	6328757	6342114	protein_coding	3057,64	0
ENSG00000108175	ZMIZ1	10	79069035	79316528	protein_coding	3054,80	0
ENSG00000169871	TRIM56	7	101085439	101097967	protein_coding	3050,60	0
ENSG00000171992	SYNPO	5	150601080	150659220	protein_coding	3041,58	0
ENSG00000196507	TCEAL3	X	103607451	103629690	protein_coding	3035,80	0
ENSG00000012779	ALOX5	10	45374176	45446119	protein_coding	3027,80	0
ENSG00000130822	PNCK	X	153669730	153689010	protein_coding	3027,70	0
ENSG00000205403	CFI	4	109740694	109802179	protein_coding	2991,70	0
ENSG00000076770	MBNL3	X	132369317	132489968	protein_coding	2972,45	0
ENSG00000122786	CALD1	7	134744252	134970728	protein_coding	2967,00	0
ENSG00000061273	HDAC7	12	47782722	47833132	protein_coding	2936,50	0
ENSG00000175445	LPL	8	19901717	19967258	protein_coding	2910,45	0
ENSG00000111321	LTBR	12	6375045	6391571	protein_coding	2873,70	0
ENSG00000249242	TMEM150C	4	82483170	82562357	protein_coding	2865,20	0
ENSG00000120057	SFRP5	10	97766751	97771952	protein_coding	2817,32	0
ENSG00000179222	MAGED1	X	51803007	51902357	protein_coding	2811,67	0
ENSG00000106571	GLI3	7	41960950	42237870	protein_coding	2788,20	0
ENSG00000175164	ABO	9	133250401	133275214	protein_coding	2783,40	0
ENSG00000164692	COL1A2	7	94394561	94431232	protein_coding	2765,56	0
ENSG00000104415	WISP1	8	133191039	133230344	protein_coding	2747,80	0
ENSG00000166888	STAT6	12	57095408	57132139	protein_coding	2722,60	0
ENSG00000166250	CLMP	11	123069865	123195281	protein_coding	2701,90	0
ENSG00000196924	FLNA	X	154348524	154374638	protein_coding	2686,88	0
ENSG00000198865	CCDC152	5	42756801	42802360	protein_coding	2674,30	0
ENSG00000124145	SDC4	20	45325288	45348424	protein_coding	2672,00	0
ENSG00000123689	G0S2	1	209675420	209676388	protein_coding	2637,00	0

ENSG00000146676	PURB	7	44876293	44885361	protein_coding	2634,27	0
ENSG00000102024	PLS3	X	115561174	115650861	protein_coding	2634,00	0
ENSG00000198467	TPM2	9	35681992	35691020	protein_coding	2619,64	0
ENSG00000160360	GPSM1	9	136327476	136359605	protein_coding	2617,00	0
ENSG00000026025	VIM	10	17228259	17237593	protein_coding	2616,51	0
ENSG00000117525	F3	1	94529225	94541800	protein_coding	2601,30	0
ENSG00000122877	EGR2	10	62811996	62919900	protein_coding	2592,90	0
ENSG00000171867	PRNP	20	4686236	4701590	protein_coding	2590,90	0
ENSG00000167580	AQP2	12	49950741	49958881	protein_coding	2587,00	0
ENSG00000109107	ALDOC	17	28573115	28577264	protein_coding	2580,30	0
ENSG00000164442	CITED2	6	139371807	139374620	protein_coding	2577,70	0
ENSG00000157227	MMP14	14	22836557	22849027	protein_coding	2561,90	0
ENSG00000197122	SRC	20	37344685	37406050	protein_coding	2554,50	0
ENSG00000038427	VCAN	5	83471465	83582303	protein_coding	2543,69	0
ENSG00000100403	ZC3H7B	22	41301522	41360147	protein_coding	2525,70	0
ENSG00000146648	EGFR	7	55019021	55256620	protein_coding	2524,80	0
ENSG00000136158	SPRY2	13	80335976	80340951	protein_coding	2507,90	0
ENSG00000159388	BTG2	1	203305491	203309602	protein_coding	2493,80	0
ENSG00000019582	CD74	5	150401637	150412929	protein_coding	2479,30	0
ENSG00000159164	SV2A	1	149903318	149917882	protein_coding	2477,00	0
ENSG00000102007	PLP2	X	49171926	49175239	protein_coding	2462,73	0
ENSG00000120659	TNFSF11	13	42562736	42608013	protein_coding	2454,70	0
ENSG00000169710	FASN	17	82078333	82098332	protein_coding	2434,14	0
ENSG00000124570	SERPINB6	6	2948159	2972165	protein_coding	2425,90	0
ENSG00000162772	ATF3	1	212565334	212620777	protein_coding	2424,80	0
ENSG00000002587	HS3ST1	4	11393150	11429765	protein_coding	2402,50	0
ENSG00000197635	DPP4	2	161992241	162074542	protein_coding	2402,20	0
ENSG00000099949	LZTR1	22	20979462	20999038	protein_coding	2377,82	0
ENSG00000184897	H1FX	3	129314771	129316277	protein_coding	2370,90	0
ENSG00000170899	GSTA4	6	52977948	52995378	protein_coding	2352,00	0
ENSG00000186432	KPNA4	3	160494995	160565588	protein_coding	2340,45	0
ENSG00000169554	ZEB2	2	144384081	144524583	protein_coding	2333,57	0
ENSG00000163083	INHBB	2	120346143	120351808	protein_coding	2319,90	0
ENSG00000103855	CD276	15	73683966	73714518	protein_coding	2318,85	0
ENSG00000182871	COL18A1	21	45405137	45513720	protein_coding	2296,70	0
ENSG00000196230	TUBB	6	30720201	30725426	protein_coding	2293,98	0
ENSG00000159069	FBXW5	9	136940435	136944696	protein_coding	2279,42	0
ENSG00000144655	CSRNP1	3	39141855	39154562	protein_coding	2262,00	0
ENSG00000102409	BEX4	X	103215092	103217246	protein_coding	2259,50	0
ENSG00000140682	TGFB1I1	16	31471585	31477960	protein_coding	2253,36	0
ENSG00000004139	SARM1	17	28364356	28404049	protein_coding	2237,90	0
ENSG00000172638	EFEMP2	11	65866441	65873592	protein_coding	2227,10	0
ENSG00000163191	S100A11	1	152032506	152047907	protein_coding	2225,67	0
ENSG00000136931	NR5A1	9	124481236	124507430	protein_coding	2225,10	0
ENSG00000123358	NR4A1	12	52022832	52059507	protein_coding	2219,70	0
ENSG00000231925	TAPBP	6	33299694	33314387	protein_coding	2214,85	0
ENSG00000153721	CNKSR3	6	154159828	154510659	protein_coding	2210,92	0
ENSG00000119138	KLF9	9	70384597	70414624	protein_coding	2209,10	0
ENSG00000118418	HMGN3	6	79201245	79234738	protein_coding	2208,30	0
ENSG00000147119	CHST7	X	46573784	46598408	protein_coding	2189,45	0
ENSG00000102401	ARMCX3	X	101622797	101627843	protein_coding	2183,30	0
ENSG00000012171	SEMA3B	3	50267558	50277546	protein_coding	2178,60	0
ENSG00000171310	CHST11	12	104455295	104762014	protein_coding	2163,50	0
ENSG00000148926	ADM	11	10304680	10307397	protein_coding	2147,40	0
ENSG00000185559	DLK1	14	100725705	100738224	protein_coding	2145,90	0
ENSG00000161638	ITGA5	12	54395261	54419460	protein_coding	2130,77	0
ENSG00000186635	ARAP1	11	72685069	72793599	protein_coding	2112,58	0
ENSG00000134121	CHL1	3	196596	409417	protein_coding	2107,20	0

ENSG00000177697	CD151	11	832843	839831	protein_coding	2093,56	0
ENSG00000137463	MGARP	4	139266163	139280338	protein_coding	2077,27	0
ENSG00000082212	ME2	18	50879049	50954257	protein_coding	2072,52	0
ENSG00000091428	RAPGEF4	2	172735274	173052893	protein_coding	2068,20	0
ENSG00000198563	DDX39B	6	31530219	31542448	protein_coding	2049,50	0
ENSG00000198300	PEG3	19	56810083	56840728	protein_coding	2040,59	0
ENSG00000140931	CMTM3	16	66603874	66613892	protein_coding	2035,91	0
ENSG00000165507	C10orf10	10	44970981	44978810	protein_coding	2033,70	0
ENSG00000003436	TFPI	2	187464230	187565760	protein_coding	2032,59	0
ENSG00000139289	PHLDA1	12	76025447	76033932	protein_coding	2030,54	0
ENSG00000109787	KLF3	4	38664196	38701042	protein_coding	1990,40	0
ENSG00000149639	SOGA1	20	36777442	36863686	protein_coding	1988,83	0
ENSG00000111674	ENO2	12	6913745	6923698	protein_coding	1980,80	0
ENSG00000168394	TAP1	6	32845209	32853978	protein_coding	1957,70	0
ENSG00000279576	ENSG00000	11	65502034	65503622	protein_coding	1957,63	0
ENSG00000143515	ATP8B2	1	154325553	154351307	protein_coding	1956,00	0
ENSG00000072422	RHOBTB1	10	60869438	61001440	protein_coding	1933,70	0
ENSG00000107796	ACTA2	10	88935074	88991390	protein_coding	1930,18	0
ENSG00000076351	SLC46A1	17	28394756	28407197	protein_coding	1920,50	0
ENSG00000100003	SEC14L2	22	30396857	30425317	protein_coding	1906,14	0
ENSG00000136295	TTYH3	7	2631951	2664802	protein_coding	1905,83	0
ENSG00000082175	PGR	11	101029624	101130524	protein_coding	1903,60	0
ENSG00000134871	COL4A2	13	110305812	110513027	protein_coding	1898,52	0
ENSG00000111684	LPCAT3	12	6976186	7018510	protein_coding	1881,62	0
ENSG00000116711	PLA2G4A	1	186828953	186988981	protein_coding	1879,50	0
ENSG00000118137	APOA1	11	116835751	116837950	protein_coding	1850,08	0
ENSG00000136997	MYC	8	127735434	127741434	protein_coding	1837,00	0
ENSG00000165801	ARHGEF40	14	21070270	21090240	protein_coding	1833,60	0
ENSG00000184557	SOCS3	17	78356778	78360077	protein_coding	1821,60	0
ENSG00000107819	SFXN3	10	101031234	101041244	protein_coding	1815,60	0
ENSG00000171812	COL8A2	1	36095236	36125220	protein_coding	1797,90	0
ENSG00000130589	HELZ2	20	63558086	63574239	protein_coding	1781,23	0
ENSG00000140263	SORD	15	45023104	45077185	protein_coding	1765,86	0
ENSG00000118523	CTGF	6	131948176	131951373	protein_coding	1763,18	0
ENSG00000121060	TRIM25	17	56887909	56914038	protein_coding	1762,30	0
ENSG00000265107	GJA5	1	147756199	147773362	protein_coding	1757,20	0
ENSG00000120129	DUSP1	5	172768090	172771195	protein_coding	1754,15	0
ENSG00000165169	DYNLT3	X	37836757	37847637	protein_coding	1744,20	0
ENSG00000169429	CXCL8	4	73740506	73743716	protein_coding	1740,70	0
ENSG00000162745	OLFML2B	1	161983192	162023854	protein_coding	1740,10	0
ENSG00000140937	CDH11	16	64943753	65126112	protein_coding	1719,60	0
ENSG00000144802	NFKBIZ	3	101827991	101861022	protein_coding	1718,70	0
ENSG00000243955	GSTA1	6	52791664	52803910	protein_coding	1712,30	0
ENSG00000173334	TRIB1	8	125430321	125438405	protein_coding	1709,93	0
ENSG00000196576	PLXNB2	22	50274979	50307627	protein_coding	1705,08	0
ENSG00000134250	NOTCH2	1	119911553	120069626	protein_coding	1703,02	0
ENSG00000130821	SLC6A8	X	153688099	153696593	protein_coding	1682,08	0
ENSG00000101680	LAMA1	18	6941744	7117814	protein_coding	1676,11	0
ENSG00000197586	ENTPD6	20	25195693	25226729	protein_coding	1668,33	0
ENSG00000152785	BMP3	4	81030965	81057531	protein_coding	1662,64	0
ENSG00000145391	SETD7	4	139495941	139606699	protein_coding	1649,20	0
ENSG00000128342	LIF	22	30240447	30246851	protein_coding	1643,30	0
ENSG00000182636	NDN	15	23685400	23687330	protein_coding	1637,10	0
ENSG00000140853	NLRC5	16	56989485	57083531	protein_coding	1628,30	0
ENSG00000122642	FKBP9	7	32957404	33006931	protein_coding	1621,80	0
ENSG00000143384	MCL1	1	150574551	150579738	protein_coding	1613,10	0
ENSG00000111371	SLC38A1	12	46183063	46270017	protein_coding	1606,50	0
ENSG00000130222	GADD45G	9	89605013	89606555	protein_coding	1601,40	0

ENSG00000100242	SUN2	22	38734725	38794143	protein_coding	1582,67	0
ENSG00000117114	ADGRL2	1	81306160	81992436	protein_coding	1576,17	0
ENSG00000100441	KHNYN	14	24429286	24441834	protein_coding	1573,61	0
ENSG00000147044	CASK	X	41514934	41923463	protein_coding	1569,24	0
ENSG00000107731	UNC5B	10	71212570	71302864	protein_coding	1561,40	0
ENSG00000170989	S1PR1	1	101236888	101241518	protein_coding	1559,10	0
ENSG00000124225	PMEPA1	20	57648392	57711536	protein_coding	1554,80	0
ENSG00000100504	PYGL	14	50857891	50944736	protein_coding	1550,80	0
ENSG00000109113	RAB34	17	28714281	28718429	protein_coding	1538,00	0
ENSG00000078018	MAP2	2	209424058	209734118	protein_coding	1528,70	0
ENSG00000205903	ZNF316	7	6637322	6656432	protein_coding	1521,40	0
ENSG00000137331	IER3	6	30743199	30744554	protein_coding	1518,91	0
ENSG00000213965	NUDT19	19	32691961	32713796	protein_coding	1515,50	0
ENSG00000187688	TRPV2	17	16415542	16437003	protein_coding	1512,60	0
ENSG00000170017	ALCAM	3	105366909	105576900	protein_coding	1499,70	0
ENSG00000205678	TECRL	4	64275257	64409468	protein_coding	1491,90	0
ENSG00000081189	MEF2C	5	88717117	88904257	protein_coding	1481,58	0
ENSG00000133636	NTS	12	85874295	85882992	protein_coding	1481,40	0
ENSG00000188313	PLSCR1	3	146515180	146544864	protein_coding	1474,00	0
ENSG00000198734	F5	1	169514166	169586588	protein_coding	1470,70	0
ENSG00000145431	PDGFC	4	156760454	156971394	protein_coding	1467,80	0
ENSG00000204681	GABBR1	6	29555629	29633976	protein_coding	1453,10	0
ENSG00000167716	WDR81	17	1716523	1738599	protein_coding	1448,60	0
ENSG00000129675	ARHGEF6	X	136665547	136782088	protein_coding	1446,71	0
ENSG00000137509	PRCP	11	82823502	82970584	protein_coding	1446,64	0
ENSG00000143390	RFX5	1	151340640	151347357	protein_coding	1444,60	0
ENSG00000139116	KIF21A	12	39293228	39443390	protein_coding	1440,00	0
ENSG00000163389	POGLUT1	3	119468938	119494708	protein_coding	1431,80	0
ENSG00000168685	IL7R	5	35852695	35879603	protein_coding	1425,70	0
ENSG00000077522	ACTN2	1	236686454	236764631	protein_coding	1421,09	0
ENSG00000141338	ABCA8	17	68867292	68955392	protein_coding	1418,31	0
ENSG00000090924	PLEKHG2	19	39412585	39428415	protein_coding	1416,10	0
ENSG00000090376	IRAK3	12	66188879	66254622	protein_coding	1416,00	0
ENSG00000225614	ZNF469	16	88427471	88440757	protein_coding	1399,30	0
ENSG00000100968	NFATC4	14	24365673	24379604	protein_coding	1394,14	0
ENSG00000104964	AES	19	3052910	3063107	protein_coding	1375,58	0
ENSG00000266714	MYO15B	17	75588058	75626501	protein_coding	1373,68	0
ENSG00000026508	CD44	11	35138870	35232402	protein_coding	1366,10	0
ENSG00000140332	TLE3	15	70047790	70098176	protein_coding	1363,36	0
ENSG00000180914	OXTR	3	8750408	8769628	protein_coding	1361,60	0
ENSG00000000003	TSPAN6	X	100627109	100639991	protein_coding	1360,55	0
ENSG00000189186	DCAF8L2	X	27590382	27748821	protein_coding	1359,10	0
ENSG00000074800	ENO1	1	8861002	8879249	protein_coding	1357,95	0
ENSG00000153714	LURAP1L	9	12775021	12822131	protein_coding	1356,60	0
ENSG00000170369	CST2	20	23823769	23826731	protein_coding	1354,00	0
ENSG00000134042	MRO	18	50795120	50825402	protein_coding	1353,17	0
ENSG00000165802	NSMF	9	137447570	137459334	protein_coding	1350,86	0
ENSG00000141504	SAT2	17	7626234	7627876	protein_coding	1349,80	0
ENSG00000166825	ANPEP	15	89784889	89815401	protein_coding	1344,50	0
ENSG00000221972	C3orf36	3	133928145	133929812	protein_coding	1339,00	0
ENSG00000102034	ELF4	X	130064874	130110716	protein_coding	1333,00	0
ENSG00000131171	SH3BGRL	X	81201943	81298547	protein_coding	1318,00	0
ENSG00000064726	BTBD1	15	83016422	83067354	protein_coding	1313,50	0
ENSG00000197872	FAM49A	2	16549459	16666331	protein_coding	1312,20	0
ENSG00000019549	SNAI2	8	48917690	48921740	protein_coding	1311,80	0
ENSG00000142227	EMP3	19	48321509	48330553	protein_coding	1296,08	0
ENSG00000143382	ADAMTSL4	1	150549369	150560937	protein_coding	1289,30	0
ENSG00000182326	C1S	12	6988259	7071032	protein_coding	1286,28	0

ENSG00000184613	NELL2	12	44508275	44921848	protein_coding	1282,20	0
ENSG00000113916	BCL6	3	187721377	187745727	protein_coding	1276,86	0
ENSG00000105767	CADM4	19	43622368	43639839	protein_coding	1264,80	0
ENSG00000116717	GADD45A	1	67685061	67688338	protein_coding	1263,95	0
ENSG00000139211	AMIGO2	12	47075707	47079951	protein_coding	1263,50	0
ENSG00000085491	SLC25A24	1	108134036	108200849	protein_coding	1256,40	0
ENSG00000172465	TCEAL1	X	103628704	103630953	protein_coding	1253,00	0
ENSG00000079308	TNS1	2	217799789	218002995	protein_coding	1251,08	0
ENSG00000160752	FDPS	1	155308748	155320666	protein_coding	1250,88	0
ENSG00000176155	CCDC57	17	82101460	82212830	protein_coding	1250,38	0
ENSG00000196954	CASP4	11	104942866	104969436	protein_coding	1248,40	0
ENSG00000131435	PDLIM4	5	132257671	132273454	protein_coding	1248,00	0
ENSG00000180921	FAM83H	8	143723933	143733801	protein_coding	1246,15	0
ENSG00000105939	ZC3HAV1	7	139043520	139109719	protein_coding	1238,52	0
ENSG00000144843	ADPRH	3	119579268	119589945	protein_coding	1229,30	0
ENSG00000198753	PLXNB3	X	153764196	153779346	protein_coding	1227,70	0
ENSG00000031081	ARHGAP31	3	119294373	119420714	protein_coding	1222,68	0
ENSG00000181458	TMEM45A	3	100492619	100577444	protein_coding	1218,30	0
ENSG00000086289	EPDR1	7	37683843	37951941	protein_coding	1214,30	0
ENSG00000157766	ACAN	15	88803443	88875354	protein_coding	1213,74	0
ENSG00000106868	SUSD1	9	112040785	112175408	protein_coding	1209,10	0
ENSG00000181751	C5orf30	5	103258702	103278660	protein_coding	1202,90	0
ENSG00000163840	DTX3L	3	122564238	122575203	protein_coding	1191,80	0
ENSG00000275832	ARHGAP23	17	38419280	38512392	protein_coding	1188,90	0
ENSG00000197043	ANXA6	5	151100712	151157882	protein_coding	1187,82	0
ENSG00000104881	PPP1R13L	19	45379634	45406349	protein_coding	1186,00	0
ENSG00000185565	LSAMP	3	115802363	117139389	protein_coding	1184,57	0
ENSG00000182087	TMEM259	19	1009648	1021179	protein_coding	1180,98	0
ENSG00000188522	FAM83G	17	18968789	19004804	protein_coding	1179,20	0
ENSG00000165449	SLC16A9	10	59650761	59736002	protein_coding	1170,50	0
ENSG00000165795	NDRG2	14	21016763	21070872	protein_coding	1169,40	0
ENSG00000124920	MYRF	11	61752642	61788518	protein_coding	1168,70	0
ENSG00000078596	ITM2A	X	79360384	79367667	protein_coding	1168,50	0
ENSG00000178951	ZBTB7A	19	4044364	4066945	protein_coding	1166,09	0
ENSG00000125398	SOX9	17	72121020	72126420	protein_coding	1164,03	0
ENSG00000178814	OPLAH	8	144051266	144063832	protein_coding	1158,50	0
ENSG00000117152	RGS4	1	163068775	163076802	protein_coding	1157,69	0
ENSG00000109099	PMP22	17	15229777	15265326	protein_coding	1154,20	0
ENSG00000112343	TRIM38	6	25962802	25991226	protein_coding	1149,10	0
ENSG00000156587	UBE2L6	11	57551656	57568284	protein_coding	1148,90	0
ENSG00000111981	ULBP1	6	149964007	149973710	protein_coding	1146,60	0
ENSG00000204287	HLA-DRA	6	32439842	32445046	protein_coding	1142,10	0
ENSG00000169499	PLEKHA2	8	38901235	38973909	protein_coding	1140,00	0
ENSG00000244509	APOBEC3C	22	39014083	39020352	protein_coding	1130,00	0
ENSG00000176049	JAKMIP2	5	147585439	147782848	protein_coding	1129,80	0
ENSG00000166340	TPP1	11	6612763	6619461	protein_coding	1128,69	0
ENSG00000132793	LPIN3	20	41340920	41360582	protein_coding	1120,10	0
ENSG00000054356	PTPRN	2	219289623	219309648	protein_coding	1114,52	0
ENSG00000003402	CFLAR	2	201116104	201176687	protein_coding	1112,82	0
ENSG00000133121	STARD13	13	33103135	33350630	protein_coding	1106,29	0
ENSG00000140320	BAHD1	15	40439721	40468242	protein_coding	1106,20	0
ENSG00000178209	PLEC	8	143915147	143976734	protein_coding	1104,43	0
ENSG00000086544	ITPKC	19	40717103	40740860	protein_coding	1102,69	0
ENSG00000134668	SPOCD1	1	31790422	31816051	protein_coding	1101,33	0
ENSG00000162413	KLHL21	1	6590724	6614607	protein_coding	1092,10	0
ENSG00000118596	SLC16A7	12	59596067	59789855	protein_coding	1091,60	0
ENSG00000164251	F2RL1	5	76818933	76835315	protein_coding	1091,50	0
ENSG00000157514	TSC22D3	X	107713221	107777342	protein_coding	1089,71	0

ENSG00000180964	TCEAL8	X	103252995	103255203	protein_coding	1089,36	0
ENSG00000126709	IFI6	1	27666061	27672218	protein_coding	1087,20	0
ENSG00000044090	CUL7	6	43037617	43053945	protein_coding	1086,29	0
ENSG00000146833	TRIM4	7	99876958	99919600	protein_coding	1085,18	0
ENSG00000149289	ZC3H12C	11	110093361	110171841	protein_coding	1083,46	0
ENSG00000184500	PROS1	3	93873033	93974066	protein_coding	1082,90	0
ENSG00000115850	LCT	2	135787840	135837180	protein_coding	1070,30	0
ENSG00000110719	TCIRG1	11	68039016	68050895	protein_coding	1069,25	0
ENSG00000104774	MAN2B1	19	12646511	12666742	protein_coding	1065,62	0
ENSG00000183943	PRKX	X	3604370	3713608	protein_coding	1064,14	0
ENSG00000166922	SCG5	15	32641676	32697098	protein_coding	1063,50	0
ENSG00000165175	MID1IP1	X	38801432	38806537	protein_coding	1062,70	0
ENSG00000177426	TGIF1	18	3411608	3459978	protein_coding	1062,00	0
ENSG00000185880	TRIM69	15	44728988	44767829	protein_coding	1061,80	0
ENSG00000152766	ANKRD22	10	88822132	88851818	protein_coding	1059,92	0
ENSG00000165476	REEP3	10	63521363	63625123	protein_coding	1058,41	0
ENSG00000087152	ATXN7L3	17	44191805	44200113	protein_coding	1053,88	0
ENSG00000183853	KIRREL	1	157993273	158100262	protein_coding	1050,90	0
ENSG00000136720	HS6ST1	2	128236716	128318577	protein_coding	1046,29	0
ENSG00000010818	HIVEP2	6	142751467	142945201	protein_coding	1045,40	0
ENSG00000134294	SLC38A2	12	46358189	46372867	protein_coding	1044,57	0
ENSG00000171451	DSEL	18	67506582	67516980	protein_coding	1042,00	0
ENSG00000188488	SERPINA5	14	94561442	94593120	protein_coding	1035,25	0
ENSG00000005486	RHBDD2	7	75842602	75888926	protein_coding	1029,46	0
ENSG00000166341	DCHS1	11	6621323	6655854	protein_coding	1028,23	0
ENSG00000138834	MAPK8IP3	16	1706183	1770317	protein_coding	1019,68	0
ENSG00000143862	ARL8A	1	202133404	202144743	protein_coding	1018,60	0
ENSG00000115290	GRB14	2	164492812	164621848	protein_coding	1016,73	0
ENSG00000139178	C1RL	12	7089587	7109273	protein_coding	1015,69	0
ENSG00000134504	KCTD1	18	26454910	26657401	protein_coding	1005,20	0
ENSG00000185340	GAS2L1	22	29306582	29312785	protein_coding	1003,20	0
ENSG00000178685	PARP10	8	143977153	144012772	protein_coding	1000,60	0
ENSG00000168283	BMI1	10	22321211	22331484	protein_coding	996,59	0
ENSG00000109321	AREG	4	74445134	74455009	protein_coding	985,95	0
ENSG00000173621	LRFN4	11	66856647	66860475	protein_coding	985,40	0
ENSG00000163359	COL6A3	2	237324003	237414375	protein_coding	976,79	0
ENSG00000006283	CACNA1G	17	50561068	50627474	protein_coding	974,10	0
ENSG00000091972	CD200	3	112332347	112362812	protein_coding	971,40	0
ENSG00000039523	FAM65A	16	67518418	67546788	protein_coding	967,10	0
ENSG00000175592	FOSL1	11	65892049	65900573	protein_coding	965,75	0
ENSG00000158769	F11R	1	160995211	161021348	protein_coding	963,10	0
ENSG00000171551	ECEL1	2	232479827	232487828	protein_coding	958,60	0
ENSG00000126458	RRAS	19	49635292	49640201	protein_coding	954,54	0
ENSG00000140274	DUOXA2	15	45114321	45118421	protein_coding	953,10	0
ENSG00000136147	PHF11	13	49495610	49528987	protein_coding	951,90	0
ENSG00000171557	FGG	4	154604134	154612967	protein_coding	950,20	0
ENSG00000131069	ACSS2	20	34872146	34927962	protein_coding	947,91	0
ENSG00000113369	ARRDC3	5	91368724	91383359	protein_coding	940,24	0
ENSG00000101160	CTSZ	20	58995185	59007247	protein_coding	939,43	0
ENSG00000102119	EMD	X	154379197	154381523	protein_coding	925,54	0
ENSG00000163297	ANTXR2	4	79901149	80125454	protein_coding	924,66	0
ENSG00000153071	DAB2	5	39371675	39462300	protein_coding	923,00	0
ENSG00000067445	TRO	X	54920462	54931431	protein_coding	921,90	0
ENSG00000179431	FJX1	11	35618419	35620868	protein_coding	921,36	0
ENSG00000173083	HPSE	4	83292461	83335153	protein_coding	918,40	0
ENSG00000160796	NBEAL2	3	46979683	47009703	protein_coding	916,90	0
ENSG00000117228	GBP1	1	89052319	89065360	protein_coding	916,90	0
ENSG00000141526	SLC16A3	17	82228397	82261129	protein_coding	915,33	0

ENSG00000123342	MMP19	12	55835433	55842966	protein_coding	914,67	0
ENSG00000163347	CLDN1	3	190305701	190322475	protein_coding	914,40	0
ENSG00000006042	TMEM98	17	32927910	32945106	protein_coding	914,30	0
ENSG00000163694	RBM47	4	40423267	40630875	protein_coding	912,80	0
ENSG00000177679	SRRM3	7	76201900	76287288	protein_coding	912,70	0
ENSG00000153443	UBALD1	16	4608883	4615027	protein_coding	910,58	0
ENSG00000161513	FDXR	17	74862497	74873031	protein_coding	910,46	0
ENSG00000131471	AOC3	17	42851184	42858130	protein_coding	906,48	0
ENSG00000197457	STMN3	20	63639705	63657682	protein_coding	904,75	0
ENSG00000104899	AMH	19	2249309	2252073	protein_coding	904,60	0
ENSG00000008056	SYN1	X	47571898	47619853	protein_coding	903,35	0
ENSG00000114019	AMOTL2	3	134355874	134375479	protein_coding	901,92	0
ENSG00000146067	FAM193B	5	177519788	177554541	protein_coding	901,56	0
ENSG00000136167	LCP1	13	46125920	46211871	protein_coding	899,75	0
ENSG00000129566	TEP1	14	20365667	20413429	protein_coding	895,80	0
ENSG00000173706	HEG1	3	124965710	125055958	protein_coding	893,38	0
ENSG00000112763	BTN2A1	6	26457904	26476621	protein_coding	892,60	0
ENSG00000013288	MAN2B2	4	6575175	6623362	protein_coding	892,37	0
ENSG00000151176	PLBD2	12	113358566	113391625	protein_coding	887,74	0
ENSG00000181104	F2R	5	76716043	76735781	protein_coding	886,60	0
ENSG00000118503	TNFAIP3	6	137867188	137883312	protein_coding	880,09	0
ENSG00000184254	ALDH1A3	15	100877714	100916626	protein_coding	877,60	0
ENSG00000144057	ST6GAL2	2	106801600	106887108	protein_coding	877,40	0
ENSG00000119508	NR4A3	9	99821855	99866891	protein_coding	874,60	0
ENSG00000178467	P4HTM	3	48989886	49007154	protein_coding	873,60	0
ENSG00000111859	NEDD9	6	11183298	11382348	protein_coding	871,40	0
ENSG00000143622	RIT1	1	155897808	155911404	protein_coding	868,09	0
ENSG00000112419	PHACTR2	6	143536845	143831185	protein_coding	867,14	0
ENSG00000213949	ITGA1	5	52787896	52959210	protein_coding	865,59	0
ENSG00000126522	ASL	7	66075798	66093558	protein_coding	865,50	0
ENSG00000116574	RHOU	1	228735077	228746669	protein_coding	862,59	0
ENSG00000143344	RGL1	1	183636085	183928531	protein_coding	855,55	0
ENSG00000185201	IFITM2	11	307631	315272	protein_coding	853,21	0
ENSG00000179532	DNHD1	11	6497260	6593758	protein_coding	852,75	0
ENSG00000100911	PSME2	14	24143362	24147570	protein_coding	848,09	0
ENSG00000142552	RCN3	19	49527618	49546962	protein_coding	847,90	0
ENSG00000125931	CITED1	X	72301638	72307187	protein_coding	847,40	0
ENSG00000105974	CAV1	7	116524785	116561184	protein_coding	847,00	0
ENSG00000070404	FSTL3	19	676365	683399	protein_coding	845,20	0
ENSG00000018408	WWTR1	3	149517235	149736714	protein_coding	844,43	0
ENSG00000100234	TIMP3	22	32801701	32863043	protein_coding	844,32	0
ENSG00000073060	SCARB1	12	124776856	124882668	protein_coding	840,57	0
ENSG00000204264	PSMB8	6	32840717	32844703	protein_coding	834,50	0
ENSG00000099326	MZF1	19	58561931	58573575	protein_coding	833,10	0
ENSG00000188641	DPYD	1	97077743	97921049	protein_coding	829,26	0
ENSG00000100445	SDR39U1	14	24439766	24442905	protein_coding	828,30	0
ENSG00000118515	SGK1	6	134169246	134318112	protein_coding	825,25	0
ENSG00000134755	DSC2	18	31058840	31102415	protein_coding	821,70	0
ENSG00000168916	ZNF608	5	124636913	124748807	protein_coding	813,10	0
ENSG00000130303	BST2	19	17402939	17405648	protein_coding	811,10	0
ENSG00000122824	NUDT10	X	51332231	51337525	protein_coding	809,73	0
ENSG00000119900	OGFRL1	6	71288803	71308950	protein_coding	802,91	0
ENSG00000089597	GANAB	11	62624826	62646726	protein_coding	799,96	0
ENSG00000162302	RPS6KA4	11	64359148	64372215	protein_coding	799,20	0
ENSG00000096696	DSP	6	7541575	7586717	protein_coding	794,78	0
ENSG00000141696	P3H4	17	41801947	41812604	protein_coding	791,40	0
ENSG00000035681	NSMAF	8	58583504	58659844	protein_coding	786,31	0
ENSG00000167508	MVD	16	88651935	88663161	protein_coding	785,31	0

ENSG00000198624	CCDC69	5	151181052	151224145	protein_coding	785,03	0
ENSG00000103257	SLC7A5	16	87830023	87869488	protein_coding	784,06	0
ENSG00000186919	ZACN	17	76071961	76083666	protein_coding	782,69	0
ENSG00000174640	SLCO2A1	3	133932696	134052184	protein_coding	780,75	0
ENSG00000115884	SDC1	2	20200797	20225433	protein_coding	778,10	0
ENSG00000164050	PLXNB1	3	48403854	48430051	protein_coding	776,80	0
ENSG00000165168	CYBB	X	37780011	37813461	protein_coding	775,80	0
ENSG00000108963	DPH1	17	2030110	2043430	protein_coding	774,40	0
ENSG00000196878	LAMB3	1	209614870	209652466	protein_coding	773,10	0
ENSG00000159674	SPON2	4	1166932	1208962	protein_coding	771,97	0
ENSG00000134138	MEIS2	15	36889204	37101299	protein_coding	771,62	0
ENSG00000102181	CD99L2	X	150766337	150898816	protein_coding	771,61	0
ENSG00000256043	CTSO	4	155924118	155953917	protein_coding	768,50	0
ENSG00000162734	PEA15	1	160205337	160215376	protein_coding	766,86	0
ENSG00000160408	ST6GALNAC	9	127885321	127905408	protein_coding	764,15	0
ENSG00000168386	FILIP1L	3	99830141	100114513	protein_coding	761,90	0
ENSG00000159899	NPR2	9	35792154	35809732	protein_coding	761,09	0
ENSG00000132669	RIN2	20	19886521	20002457	protein_coding	759,69	0
ENSG00000138735	PDE5A	4	119494395	119628991	protein_coding	752,62	0
ENSG00000198833	UBE2J1	6	89326625	89352848	protein_coding	748,09	0
ENSG00000004799	PDK4	7	95583499	95596491	protein_coding	747,10	0
ENSG00000162511	LAPTM5	1	30732469	30757820	protein_coding	747,00	0
ENSG00000111275	ALDH2	12	111766887	111817529	protein_coding	743,86	0
ENSG00000144579	CTDSP1	2	218398256	218405941	protein_coding	743,58	0
ENSG00000133800	LYVE1	11	10556966	10611689	protein_coding	742,00	0
ENSG00000198932	GPRASP1	X	102651366	102659083	protein_coding	741,00	0
ENSG00000142156	COL6A1	21	45981737	46005050	protein_coding	740,87	0
ENSG00000137267	TUBB2A	6	3153669	3157526	protein_coding	740,14	0
ENSG00000106483	SFRP4	7	37905932	38025695	protein_coding	730,40	0
ENSG00000196159	FAT4	4	125316399	125492932	protein_coding	730,10	0
ENSG00000101955	SRPX	X	38149336	38220924	protein_coding	729,83	0
ENSG00000171517	LPAR3	1	84811602	84893213	protein_coding	729,80	0
ENSG00000162873	KLHDC8A	1	205336065	205357090	protein_coding	728,00	0
ENSG00000184867	ARMCX2	X	101655281	101659891	protein_coding	725,80	0
ENSG00000125848	FLRT3	20	14322988	14337616	protein_coding	724,80	0
ENSG00000114923	SLC4A3	2	219627327	219641980	protein_coding	721,55	0
ENSG00000135625	EGR4	2	73290929	73293705	protein_coding	718,00	0
ENSG00000134531	EMP1	12	13196716	13219939	protein_coding	717,60	0
ENSG00000146112	PPP1R18	6	30676389	30687895	protein_coding	713,59	0
ENSG00000115461	IGFBP5	2	216672105	216695525	protein_coding	712,29	0
ENSG00000099377	HSD3B7	16	30985207	30989152	protein_coding	710,20	0
ENSG00000115155	OTOF	2	26457203	26558698	protein_coding	708,50	0
ENSG00000103154	NECAB2	16	83968632	84002776	protein_coding	705,73	0
ENSG00000203963	C1orf141	1	67092165	67231853	protein_coding	702,00	0
ENSG00000100439	ABHD4	14	22598237	22613215	protein_coding	700,92	0
ENSG00000116584	ARHGEF2	1	155946851	156007070	protein_coding	699,30	0
ENSG00000026103	FAS	10	88990531	89015785	protein_coding	696,00	0
ENSG00000013297	CLDN11	3	170418865	170860380	protein_coding	695,45	0
ENSG00000177943	MAMDC4	9	136850943	136860799	protein_coding	695,00	0
ENSG00000182752	PAPPA	9	116153804	116402322	protein_coding	694,78	0
ENSG00000237441	RGL2	6	33291654	33299324	protein_coding	694,59	0
ENSG00000138615	CILP	15	65194758	65211488	protein_coding	693,67	0
ENSG00000025708	TYMP	22	50525752	50530056	protein_coding	693,30	0
ENSG00000130513	GDF15	19	18374731	18389176	protein_coding	692,18	0
ENSG00000168477	TNXB	6	32041154	32115334	protein_coding	690,90	0
ENSG00000185924	RTN4RL1	17	1934677	2025345	protein_coding	685,90	0
ENSG00000153029	MR1	1	181033425	181061938	protein_coding	685,60	0
ENSG00000152092	ASTN1	1	176857302	177164973	protein_coding	684,80	0

ENSG00000126947	ARMCX1	X	101550531	101554700	protein_coding	684,10	0
ENSG00000147140	NONO	X	71283192	71301168	protein_coding	682,24	0
ENSG00000143768	LEFTY2	1	225936598	225941489	protein_coding	679,90	0
ENSG00000204520	MICA	6	31399784	31415315	protein_coding	677,70	0
ENSG00000143869	GDF7	2	20666664	20679245	protein_coding	675,90	0
ENSG00000142669	SH3BGRL3	1	26279176	26281522	protein_coding	674,00	0
ENSG00000147576	ADHFE1	8	66430185	66471601	protein_coding	673,40	0
ENSG00000102178	UBL4A	X	154483717	154486670	protein_coding	673,10	0
ENSG00000101199	ARFGAP1	20	63272785	63289793	protein_coding	672,61	0
ENSG00000068079	IFI35	17	43006725	43014456	protein_coding	672,10	0
ENSG00000213462	ERV3-1	7	64990809	65006743	protein_coding	672,10	0
ENSG00000244734	HBB	11	5225464	5229395	protein_coding	670,30	0
ENSG00000071889	FAM3A	X	154506159	154516242	protein_coding	670,15	0
ENSG00000085719	CPNE3	8	86484830	86561498	protein_coding	665,05	0
ENSG00000185149	NPY2R	4	155208629	155217078	protein_coding	663,10	0
ENSG00000073737	DHRS9	2	169064789	169096167	protein_coding	662,91	0
ENSG00000196411	EPHB4	7	100802565	100827521	protein_coding	662,80	0
ENSG00000197283	SYNGAP1	6	33420070	33457541	protein_coding	662,45	0
ENSG00000173281	PPP1R3B	8	9136255	9151574	protein_coding	662,28	0
ENSG00000087077	TRIP6	7	100867138	100873454	protein_coding	661,04	0
ENSG00000214021	TTLL3	3	9808086	9855138	protein_coding	660,86	0
ENSG00000167615	LENG8	19	54448887	54462037	protein_coding	660,38	0
ENSG00000167543	TP53I13	17	29566052	29573157	protein_coding	657,10	0
ENSG00000167925	GHDC	17	42188799	42194532	protein_coding	656,90	0
ENSG00000135083	CCNJL	5	160251652	160345396	protein_coding	656,20	0
ENSG00000005238	FAM214B	9	35104112	35116341	protein_coding	654,27	0
ENSG00000105971	CAV2	7	116287380	116508541	protein_coding	654,11	0
ENSG00000198108	CHSY3	5	129904472	130186634	protein_coding	653,50	0
ENSG00000071655	MBD3	19	1573596	1592801	protein_coding	652,93	0
ENSG00000140553	UNC45A	15	90930180	90954093	protein_coding	652,75	0
ENSG00000230657	PRB4	12	11307083	11310435	protein_coding	652,50	0
ENSG00000125817	CENPB	20	3783851	3786690	protein_coding	651,61	0
ENSG00000175899	A2M	12	9067664	9116229	protein_coding	649,50	0
ENSG00000133083	DCLK1	13	35768652	36131306	protein_coding	645,80	0
ENSG00000213366	GSTM2	1	109668022	109709551	protein_coding	645,40	0
ENSG00000140691	ARMC5	16	31458080	31467166	protein_coding	645,00	0
ENSG00000077713	SLC25A43	X	119399060	119454478	protein_coding	641,18	0
ENSG00000148730	EIF4EBP2	10	70404379	70428618	protein_coding	640,89	0
ENSG00000175602	CCDC85B	11	65890112	65891635	protein_coding	640,00	0
ENSG00000138449	SLC40A1	2	189560579	189583758	protein_coding	637,90	0
ENSG00000120594	PLXDC2	10	19816239	20289856	protein_coding	636,88	0
ENSG00000118785	SPP1	4	87975650	87983426	protein_coding	636,50	0
ENSG00000141736	ERBB2	17	39687914	39730426	protein_coding	635,95	0
ENSG00000137869	CYP19A1	15	51208057	51338610	protein_coding	633,73	0
ENSG00000162378	ZYG11B	1	52726467	52827342	protein_coding	633,56	0
ENSG00000093010	COMT	22	19941607	19969975	protein_coding	633,03	0
ENSG00000231389	HLA-DPA1	6	33064569	33080775	protein_coding	630,90	0
ENSG00000157214	STEAP2	7	90167590	90238137	protein_coding	630,50	0
ENSG00000132274	TRIM22	11	5689689	5737089	protein_coding	630,40	0
ENSG00000174808	BTC	4	74744759	74794686	protein_coding	628,80	0
ENSG00000239713	APOBEC3G	22	39040961	39087743	protein_coding	626,80	0
ENSG00000157873	TNFRSF14	1	2555639	2565382	protein_coding	625,70	0
ENSG00000169083	AR	X	67544032	67730619	protein_coding	623,50	0
ENSG00000155287	SLC25A28	10	99610522	99620609	protein_coding	621,69	0
ENSG00000177732	SOX12	20	325401	330224	protein_coding	621,41	0
ENSG00000163932	PRKCD	3	53156009	53192717	protein_coding	620,74	0
ENSG00000140400	MAN2C1	15	75355207	75368630	protein_coding	619,91	0
ENSG00000114841	DNAH1	3	52316319	52400491	protein_coding	618,17	0

ENSG00000182179	UBA7	3	49805207	49813946	protein_coding	616,10	0
ENSG00000120708	TGFBI	5	136028895	136063818	protein_coding	615,80	0
ENSG00000067082	KLF6	10	3775996	3785281	protein_coding	615,53	0
ENSG00000180155	LYNX1	8	142764334	142778224	protein_coding	615,40	0
ENSG00000177575	CD163	12	7470813	7503893	protein_coding	610,60	0
ENSG00000147650	LRP12	8	104489231	104589024	protein_coding	610,37	0
ENSG00000162733	DDR2	1	162631373	162787400	protein_coding	606,49	0
ENSG00000140479	PCSK6	15	101297142	101525202	protein_coding	604,85	0
ENSG00000176170	SPHK1	17	76376584	76387860	protein_coding	602,50	0
ENSG00000180304	OAZ2	15	64687573	64703281	protein_coding	601,33	0
ENSG00000096060	FKBP5	6	35573585	35728583	protein_coding	601,13	0
ENSG00000177225	PDDC1	11	767220	777488	protein_coding	601,00	0
ENSG00000130449	ZSWIM6	5	61332273	61546170	protein_coding	600,40	0
ENSG00000167107	ACSF2	17	50426158	50474845	protein_coding	599,60	0
ENSG00000064205	WISP2	20	44714844	44728509	protein_coding	599,52	0
ENSG00000105429	MEGF8	19	42325609	42378769	protein_coding	594,53	0
ENSG00000125384	PTGER2	14	52314305	52328606	protein_coding	593,30	0
ENSG00000099960	SLC7A4	22	21028718	21032840	protein_coding	591,70	0
ENSG00000115129	TP53I3	2	24077433	24085861	protein_coding	590,70	0
ENSG00000137460	FHDC1	4	152936352	152979696	protein_coding	590,55	0
ENSG00000118762	PKD2	4	88007668	88077777	protein_coding	589,10	0
ENSG00000162687	KCNT2	1	196225779	196609225	protein_coding	587,50	0
ENSG00000176046	NUPR1	16	28532708	28539174	protein_coding	586,10	0
ENSG00000093072	CECR1	22	17178790	17221989	protein_coding	584,50	0
ENSG00000177628	GBA	1	155234452	155244699	protein_coding	584,50	0
ENSG00000183741	CBX6	22	38861450	38872314	protein_coding	580,13	0
ENSG00000132535	DLG4	17	7189890	7219702	protein_coding	580,10	0
ENSG00000220205	VAMP2	17	8159149	8163546	protein_coding	577,12	0
ENSG00000162241	SLC25A45	11	65375192	65383701	protein_coding	576,69	0
ENSG00000189077	TMEM120A	7	75986837	75994659	protein_coding	575,92	0
ENSG00000151883	PARP8	5	50665899	50846522	protein_coding	573,70	0
ENSG00000177432	NAP1L5	4	88695915	88698235	protein_coding	572,50	0
ENSG00000197496	SLC2A10	20	46709487	46736347	protein_coding	571,10	0
ENSG00000135452	TSPAN31	12	57738013	57750211	protein_coding	570,58	0
ENSG00000154262	ABCA6	17	69078702	69141888	protein_coding	570,38	0
ENSG00000106605	BLVRA	7	43758680	43807342	protein_coding	570,30	0
ENSG00000182492	BGN	X	153494939	153509554	protein_coding	568,70	0
ENSG00000131981	LGALS3	14	55124110	55145413	protein_coding	568,65	0
ENSG00000067066	SP100	2	230415942	230544090	protein_coding	568,29	0
ENSG00000173442	EHBP1L1	11	65576038	65592650	protein_coding	566,60	0
ENSG00000103264	FBXO31	16	87326987	87392142	protein_coding	566,57	0
ENSG00000124253	PCK1	20	57561080	57568112	protein_coding	564,70	0
ENSG00000165891	E2F7	12	77021247	77065580	protein_coding	563,19	0
ENSG00000160959	LRRC14	8	144517992	144525178	protein_coding	562,81	0
ENSG00000184384	MAML2	11	95976598	96343180	protein_coding	562,05	0
ENSG00000010704	HFE	6	26087281	26098343	protein_coding	560,60	0
ENSG00000198286	CARD11	7	2906141	3043945	protein_coding	560,20	0
ENSG00000110237	ARHGEF17	11	73308289	73369091	protein_coding	559,24	0
ENSG00000140254	DUOXA1	15	45117367	45129938	protein_coding	558,10	0
ENSG00000111344	RASAL1	12	113098819	113136239	protein_coding	557,60	0
ENSG00000148677	ANKRD1	10	90912096	90921276	protein_coding	557,30	0
ENSG00000277443	MARCKS	6	113857362	113863471	protein_coding	556,77	0
ENSG00000165215	CLDN3	7	73768997	73770270	protein_coding	555,80	0
ENSG00000197355	UAP1L1	9	137077501	137084539	protein_coding	554,50	0
ENSG00000076928	ARHGEF1	19	41883161	41930150	protein_coding	553,53	0
ENSG00000204439	C6orf47	6	31658298	31660772	protein_coding	551,40	0
ENSG00000115226	FNDC4	2	27491883	27495245	protein_coding	551,35	0
ENSG00000156869	FRRS1	1	99708703	99766631	protein_coding	550,70	0

ENSG00000168310	IRF2	4	184387713	184474580	protein_coding	549,89	0
ENSG00000129103	SUMF2	7	56064002	56080670	protein_coding	549,52	0
ENSG00000165633	VSTM4	10	49014245	49115509	protein_coding	549,10	0
ENSG00000123595	RAB9A	X	13689121	13710506	protein_coding	546,38	0
ENSG00000130518	KIAA1683	19	18257097	18274509	protein_coding	543,60	0
ENSG00000144815	NXPE3	3	101779202	101828229	protein_coding	543,22	0
ENSG00000124067	SLC12A4	16	67943474	67969601	protein_coding	541,21	0
ENSG00000149591	TAGLN	11	117199321	117204782	protein_coding	541,20	0
ENSG00000183337	BCOR	X	40049815	40177329	protein_coding	541,00	0
ENSG00000006118	TMEM132A	11	60924463	60937159	protein_coding	538,31	0
ENSG00000196123	KIAA0895L	16	67175602	67184040	protein_coding	537,74	0
ENSG00000114251	WNT5A	3	55465715	55490539	protein_coding	537,38	0
ENSG00000075399	VPS9D1	16	89707134	89720986	protein_coding	533,88	0
ENSG00000166886	NAB2	12	57088894	57095476	protein_coding	533,75	0
ENSG00000152926	ZNF117	7	64971776	65006684	protein_coding	533,52	0
ENSG00000011478	QPCTL	19	45692483	45703989	protein_coding	532,70	0
ENSG00000198959	TGM2	20	38127387	38166578	protein_coding	532,40	0
ENSG00000138134	STAMBPL1	10	88879734	88975153	protein_coding	530,90	0
ENSG00000214193	SH3D21	1	36306387	36324886	protein_coding	529,36	0
ENSG00000119314	PTBP3	9	112217716	112333667	protein_coding	526,89	0
ENSG00000169604	ANTXR1	2	69013178	69249327	protein_coding	526,25	0
ENSG00000115107	STEAP3	2	119223831	119265652	protein_coding	526,10	0
ENSG00000213722	DDAH2	6	31727038	31730617	protein_coding	525,76	0
ENSG00000156466	GDF6	8	96142330	96160792	protein_coding	525,09	0
ENSG00000110697	PITPNM1	11	67491768	67506263	protein_coding	525,00	0
ENSG00000149809	TM7SF2	11	65111845	65116384	protein_coding	524,40	0
ENSG00000105204	DYRK1B	19	39825350	39834201	protein_coding	523,90	0
ENSG00000101665	SMAD7	18	48919853	48950711	protein_coding	523,70	0
ENSG00000163898	LIPH	3	185506262	185552613	protein_coding	523,55	0
ENSG00000138039	LHCGR	2	48686775	48755730	protein_coding	523,10	0
ENSG00000174233	ADCY6	12	48766194	48789037	protein_coding	522,55	0
ENSG00000088899	ENSG000000	20	3162617	3173592	protein_coding	521,92	0
ENSG00000149131	SERPING1	11	57597387	57614853	protein_coding	521,70	0
ENSG00000186310	NAP1L3	X	93670930	93673568	protein_coding	521,60	0
ENSG00000167552	TUBA1A	12	49184796	49189324	protein_coding	521,49	0
ENSG00000114270	COL7A1	3	48564073	48595267	protein_coding	520,69	0
ENSG00000137124	ALDH1B1	9	38392664	38398661	protein_coding	520,67	0
ENSG00000105701	FKBP8	19	18531751	18544077	protein_coding	520,51	0
ENSG00000177613	CSTF2T	10	51695487	51699591	protein_coding	518,12	0
ENSG00000155252	PI4K2A	10	97640686	97676434	protein_coding	518,00	0
ENSG00000188157	AGRN	1	1020123	1056118	protein_coding	516,68	0
ENSG00000179862	CITED4	1	40861051	40862366	protein_coding	515,06	0
ENSG00000059804	SLC2A3	12	7919230	7936275	protein_coding	514,47	0
ENSG00000158301	GPRASP2	X	102712176	102717733	protein_coding	514,30	0
ENSG00000162236	STX5	11	62806897	62832088	protein_coding	512,80	0
ENSG00000214026	MRPL23	11	1947278	1984522	protein_coding	512,64	0
ENSG00000160712	IL6R	1	154405193	154469450	protein_coding	510,20	0
ENSG00000189369	GSPT2	X	51743431	51746232	protein_coding	509,80	0
ENSG00000014914	MTMR11	1	149928651	149936898	protein_coding	508,82	0
ENSG00000105137	SYDE1	19	15107403	15114988	protein_coding	507,40	0
ENSG00000184905	TCEAL2	X	102125688	102127711	protein_coding	506,90	0
ENSG00000065357	DGKA	12	55927319	55954027	protein_coding	505,79	0
ENSG00000105135	ILVBL	19	15114984	15125785	protein_coding	505,22	0
ENSG00000173548	SNX33	15	75647906	75662301	protein_coding	504,90	0
ENSG00000159348	CYB5R1	1	202961869	202967280	protein_coding	504,70	0
ENSG00000155090	KLF10	8	102648779	102655902	protein_coding	503,13	0
ENSG00000173230	GOLGB1	3	121663199	121749767	protein_coding	501,23	0
ENSG00000173114	LRRN3	7	111091006	111125454	protein_coding	498,40	0

ENSG00000254470	AP5B1	11	65775893	65780802	protein_coding	496,60	0
ENSG00000196126	HLA-DRB1	6	32552990	32589848	protein_coding	496,40	0
ENSG00000183770	FOXL2	3	138944224	138947140	protein_coding	495,90	0
ENSG00000178252	WDR6	3	49007062	49015953	protein_coding	494,23	0
ENSG00000160255	ITGB2	21	44885953	44931989	protein_coding	493,50	0
ENSG00000176165	FOXG1	14	28765388	28770277	protein_coding	492,50	0
ENSG00000105329	TGFB1	19	41301587	41353911	protein_coding	492,37	0
ENSG00000105810	CDK6	7	92604921	92836594	protein_coding	491,40	0
ENSG00000186470	BTN3A2	6	26365159	26378320	protein_coding	489,00	0
ENSG00000103066	PLA2G15	16	68245304	68261062	protein_coding	488,80	0
ENSG00000099860	GADD45B	19	2476122	2478259	protein_coding	488,46	0
ENSG00000140939	NOL3	16	67170154	67175735	protein_coding	486,86	0
ENSG00000104936	DMPK	19	45769717	45782552	protein_coding	486,76	0
ENSG00000172315	TP53RK	20	46684365	46689779	protein_coding	486,20	0
ENSG00000117298	ECE1	1	21217247	21345504	protein_coding	484,58	0
ENSG00000163884	KLF15	3	126342635	126357442	protein_coding	484,14	0
ENSG00000179094	PER1	17	8140472	8156506	protein_coding	483,70	0
ENSG00000082684	SEMA5B	3	122909193	123028605	protein_coding	482,90	0
ENSG00000143147	GPR161	1	168079543	168137667	protein_coding	481,92	0
ENSG00000160683	CXCR5	11	118883766	118897799	protein_coding	481,16	0
ENSG00000055118	KCNH2	7	150944961	150978315	protein_coding	480,08	0
ENSG00000196155	PLEKHG4	16	67277510	67289499	protein_coding	479,40	0
ENSG00000177595	PIDD1	11	799179	809753	protein_coding	479,23	0
ENSG00000110455	ACCS	11	44065925	44084222	protein_coding	479,10	0
ENSG00000117335	CD46	1	207752057	207795513	protein_coding	478,73	0
ENSG00000057019	DCBLD2	3	98795941	98901689	protein_coding	478,66	0
ENSG00000164897	TMUB1	7	151081080	151083546	protein_coding	477,79	0
ENSG00000146373	RNF217	6	124962545	125092633	protein_coding	476,80	0
ENSG00000130066	SAT1	X	23783173	23786226	protein_coding	476,59	0
ENSG00000184271	POU6F1	12	51186936	51217693	protein_coding	476,10	0
ENSG00000139266	09-mars	12	57755098	57760407	protein_coding	474,50	0
ENSG00000100596	SPTLC2	14	77505997	77616773	protein_coding	474,49	0
ENSG00000051523	CYBA	16	88643283	88651152	protein_coding	470,50	0
ENSG00000163739	CXCL1	4	73869393	73871242	protein_coding	470,40	0
ENSG00000139567	ACVRL1	12	51906908	51923361	protein_coding	469,60	0
ENSG00000013619	MAMLD1	X	150361422	150514178	protein_coding	468,80	0
ENSG00000137857	DUOX1	15	45129933	45165576	protein_coding	468,70	0
ENSG00000143847	PPFIA4	1	203026498	203078740	protein_coding	465,20	0
ENSG00000125810	CD93	20	23079349	23086340	protein_coding	463,30	0
ENSG00000109323	MANBA	4	102631488	102760994	protein_coding	462,60	0
ENSG00000164128	NPY1R	4	163323961	163344832	protein_coding	461,50	0
ENSG00000205413	SAMD9	7	93099513	93118023	protein_coding	461,18	0
ENSG00000137628	DDX60	4	168216293	168318807	protein_coding	460,70	0
ENSG00000121966	CXCR4	2	136114349	136118165	protein_coding	460,62	0
ENSG00000099999	RNF215	22	30368811	30421771	protein_coding	460,50	0
ENSG00000110628	SLC22A18	11	2899721	2925246	protein_coding	458,70	0
ENSG00000198354	DCAF12L2	X	126164531	126165951	protein_coding	457,80	0
ENSG00000129925	TMEM8A	16	370773	387113	protein_coding	457,34	0
ENSG00000137872	SEMA6D	15	47184101	47774223	protein_coding	456,91	0
ENSG00000128335	APOL2	22	36226203	36239954	protein_coding	456,70	0
ENSG00000124479	NDP	X	43948776	43973504	protein_coding	455,40	0
ENSG00000113108	APBB3	5	140558268	140593752	protein_coding	455,33	0
ENSG00000111676	ATN1	12	6924463	6942321	protein_coding	455,17	0
ENSG00000147174	ACRC	X	71578411	71613583	protein_coding	455,00	0
ENSG00000171864	PRND	20	4721910	4728460	protein_coding	454,60	0
ENSG00000180447	GAS1	9	86944363	86947189	protein_coding	454,50	0
ENSG00000132821	VSTM2L	20	37903104	37945350	protein_coding	453,50	0
ENSG00000142686	C1orf216	1	35713875	35719472	protein_coding	453,00	0

ENSG00000168758	SEMA4C	2	96859716	96870757	protein_coding	450,10	0
ENSG00000153294	ADGRF4	6	47685864	47722021	protein_coding	449,90	0
ENSG00000154655	L3MBTL4	18	5954706	6415237	protein_coding	449,18	0
ENSG00000159167	STC1	8	23841915	23854807	protein_coding	447,80	0
ENSG00000133142	TCEAL4	X	103576231	103587736	protein_coding	447,35	0
ENSG00000103249	CLCN7	16	1444934	1475580	protein_coding	446,64	0
ENSG00000160072	ATAD3B	1	1471769	1497848	protein_coding	446,54	0
ENSG00000135046	ANXA1	9	73151757	73170393	protein_coding	446,30	0
ENSG00000158859	ADAMTS4	1	161184308	161199056	protein_coding	445,00	0
ENSG00000152583	SPARCL1	4	87473335	87531061	protein_coding	444,90	0
ENSG00000162692	VCAM1	1	100719742	100739045	protein_coding	444,32	0
ENSG00000218336	TENM3	4	182143987	182803024	protein_coding	442,73	0
ENSG00000164889	SLC4A2	7	151057210	151076527	protein_coding	442,61	0
ENSG00000125746	EML2	19	45606994	45645629	protein_coding	442,27	0
ENSG00000123815	ADCK4	19	40691529	40718207	protein_coding	442,23	0
ENSG00000157350	ST3GAL2	16	70375978	70439237	protein_coding	440,19	0
ENSG00000092098	RNF31	14	24146683	24160661	protein_coding	438,30	0
ENSG00000142961	MOB3C	1	46607715	46616891	protein_coding	436,90	0
ENSG00000128283	CDC42EP1	22	37560447	37569405	protein_coding	436,70	0
ENSG00000090565	RAB11FIP3	16	425619	523011	protein_coding	434,89	0
ENSG00000140983	RHOT2	16	668086	674174	protein_coding	434,73	0
ENSG00000108352	RAPGEFL1	17	40177010	40195656	protein_coding	432,70	0
ENSG00000134853	PDGFRA	4	54229097	54298247	protein_coding	431,90	0
ENSG00000162415	ZSWIM5	1	45016399	45306209	protein_coding	431,75	0
ENSG00000129473	BCL2L2	14	23298790	23311759	protein_coding	431,52	0
ENSG00000071859	FAM50A	X	154444126	154450654	protein_coding	429,26	0
ENSG00000139645	ANKRD52	12	56237807	56258391	protein_coding	428,78	0
ENSG00000007541	PIGQ	16	566995	584136	protein_coding	428,62	0
ENSG00000134684	YARS	1	32775237	32818153	protein_coding	428,15	0
ENSG00000137842	TMEM62	15	43123279	43185146	protein_coding	426,90	0
ENSG00000240972	MIF	22	23894004	23895227	protein_coding	426,66	0
ENSG00000137094	DNAJB5	9	34989641	34998900	protein_coding	426,03	0
ENSG00000115602	IL1RL1	2	102311502	102352037	protein_coding	425,40	0
ENSG00000143126	CELSR2	1	109250019	109275750	protein_coding	425,40	0
ENSG00000196263	ZNF471	19	56507843	56530221	protein_coding	425,30	0
ENSG00000198142	SOWAHC	2	109614334	109618990	protein_coding	424,59	0
ENSG00000116157	GPX7	1	52602372	52609051	protein_coding	424,00	0
ENSG00000212747	FAM127C	X	135020504	135022529	protein_coding	423,80	0
ENSG00000257704	INAFM1	19	47274453	47275707	protein_coding	423,62	0
ENSG00000129538	RNASE1	14	20801228	20803278	protein_coding	423,50	0
ENSG00000145335	SNCA	4	89724099	89838315	protein_coding	423,40	0
ENSG00000148399	DPH7	9	137554904	137578935	protein_coding	421,70	0
ENSG00000127472	PLA2G5	1	20028179	20091190	protein_coding	419,50	0
ENSG00000106404	CLDN15	7	101232092	101238820	protein_coding	418,08	0
ENSG00000160233	LRRC3	21	44455486	44462196	protein_coding	416,00	0
ENSG00000006282	SPATA20	17	50543058	50555852	protein_coding	415,71	0
ENSG00000136305	CIDEB	14	24305096	24311430	protein_coding	415,69	0
ENSG00000163131	CTSS	1	150730196	150765957	protein_coding	415,64	0
ENSG00000110315	RNF141	11	10511678	10541230	protein_coding	415,11	0
ENSG00000069535	MAOB	X	43766611	43882447	protein_coding	415,00	0
ENSG00000134184	GSTM1	1	109687814	109709039	protein_coding	414,90	0
ENSG00000075213	SEMA3A	7	83955777	84492724	protein_coding	414,23	0
ENSG00000158792	SPATA2L	16	89696343	89701705	protein_coding	413,80	0
ENSG00000198963	RORB	9	74497365	74693177	protein_coding	413,60	0
ENSG00000095739	BAMBI	10	28677342	28682939	protein_coding	412,57	0
ENSG00000037757	MRI1	19	13764532	13774282	protein_coding	412,10	0
ENSG00000161996	WDR90	16	649311	667833	protein_coding	412,10	0
ENSG00000026950	BTN3A1	6	26402237	26415216	protein_coding	411,20	0

ENSG00000114738	MAPKAPK3	3	50611520	50649297	protein_coding	410,69	0
ENSG00000143387	CTSK	1	150796208	150808323	protein_coding	410,57	0
ENSG00000172137	CALB2	16	71358713	71390438	protein_coding	407,98	0
ENSG00000250479	CHCHD10	22	23765834	23768443	protein_coding	407,90	0
ENSG00000131016	AKAP12	6	151239999	151358557	protein_coding	407,45	0
ENSG00000176845	METRNL	17	83079691	83095119	protein_coding	406,12	0
ENSG00000058453	CROCC	1	16740273	16972979	protein_coding	404,80	0
ENSG00000185818	NAT8L	4	2059512	2069089	protein_coding	404,70	0
ENSG00000197629	MPEG1	11	59208510	59212951	protein_coding	404,00	0
ENSG00000101276	SLC52A3	20	760080	776015	protein_coding	403,80	0
ENSG00000182173	TSEN54	17	75516060	75524739	protein_coding	403,50	0
ENSG00000166592	RRAD	16	66921679	66925644	protein_coding	403,20	0
ENSG00000130038	CRACR2A	12	3606633	3764819	protein_coding	401,51	0
ENSG00000169271	HSPB3	5	54455601	54456384	protein_coding	400,80	0
ENSG00000169992	NLGN2	17	7404874	7419860	protein_coding	399,29	0
ENSG00000160326	SLC2A6	9	133471095	133479137	protein_coding	398,60	0
ENSG00000104805	NUCB1	19	48900050	48923372	protein_coding	397,75	0
ENSG00000164032	H2AFZ	4	99948086	99950388	protein_coding	397,65	0
ENSG00000070610	GBA2	9	35736866	35749228	protein_coding	397,01	0
ENSG00000155760	FZD7	2	202034587	202038445	protein_coding	396,64	0
ENSG00000155256	ZFYVE27	10	97737121	97760907	protein_coding	394,30	0
ENSG00000100612	DHRS7	14	60144120	60169856	protein_coding	393,71	0
ENSG00000185432	METTL7A	12	50923472	50932517	protein_coding	392,40	0
ENSG00000135446	CDK4	12	57747727	57756013	protein_coding	391,39	0
ENSG00000123609	NMI	2	151270465	151290057	protein_coding	391,10	0
ENSG00000139531	SUOX	12	55997180	56006641	protein_coding	390,30	0
ENSG00000165282	PIGO	9	35088688	35096601	protein_coding	390,13	0
ENSG00000100342	APOL1	22	36253010	36267530	protein_coding	389,70	0
ENSG00000107551	RASSF4	10	44959407	44995891	protein_coding	389,60	0
ENSG00000162981	FAM84A	2	14632686	14650814	protein_coding	388,40	0
ENSG00000141959	PFKL	21	44300051	44327376	protein_coding	388,27	0
ENSG00000172216	CEBPB	20	50190734	50192689	protein_coding	388,06	0
ENSG00000184194	GPR173	X	53049091	53080615	protein_coding	387,90	0
ENSG00000084652	TXLNA	1	32179686	32198285	protein_coding	387,66	0
ENSG00000170439	METTL7B	12	55681546	55684611	protein_coding	387,60	0
ENSG00000196440	ARMCX4	X	101418287	101533459	protein_coding	387,57	0
ENSG00000168899	VAMP5	2	85584408	85593412	protein_coding	386,70	0
ENSG00000152778	IFIT5	10	89414586	89421001	protein_coding	385,83	0
ENSG00000176108	CHMP6	17	80991598	81009517	protein_coding	385,62	0
ENSG00000170004	CHD3	17	7884806	7912760	protein_coding	385,37	0
ENSG00000161960	EIF4A1	17	7572706	7579005	protein_coding	384,27	0
ENSG00000198816	ZNF358	19	7515292	7521026	protein_coding	384,06	0
ENSG00000048140	TSPAN17	5	176647387	176659054	protein_coding	383,84	0
ENSG00000187134	AKR1C1	10	4963253	5107522	protein_coding	382,80	0
ENSG00000144115	THNSL2	2	88170295	88186636	protein_coding	382,80	0
ENSG00000101194	SLC17A9	20	62952647	62969585	protein_coding	382,15	0
ENSG00000074181	NOTCH3	19	15159038	15200981	protein_coding	381,10	0
ENSG00000176248	ANAPC2	9	137174784	137188549	protein_coding	380,83	0
ENSG00000137076	TLN1	9	35696948	35732395	protein_coding	380,53	0
ENSG00000198892	SHISA4	1	201888680	201892306	protein_coding	380,30	0
ENSG00000133134	BEX2	X	103309346	103311046	protein_coding	380,23	0
ENSG00000185324	CDK10	16	89680737	89696364	protein_coding	378,49	0
ENSG00000104824	HNRNPL	19	38836388	38852347	protein_coding	377,80	0
ENSG00000141026	MED9	17	17476986	17493226	protein_coding	376,40	0
ENSG00000185909	KLHDC8B	3	49171611	49176486	protein_coding	376,40	0
ENSG00000182916	TCEAL7	X	103330196	103332326	protein_coding	375,50	0
ENSG00000127129	EDN2	1	41478775	41484673	protein_coding	375,20	0
ENSG00000173349	SFT2D3	2	127701508	127705242	protein_coding	374,25	0

ENSG00000030110	BAK1	6	33572547	33580293	protein_coding	374,00	0
ENSG00000064989	CALCRL	2	187343129	187448460	protein_coding	373,80	0
ENSG00000068366	ACSL4	X	109624244	109733403	protein_coding	372,91	0
ENSG00000119986	AVPI1	10	97677424	97687323	protein_coding	372,86	0
ENSG00000185189	NRBP2	8	143833594	143840974	protein_coding	372,72	0
ENSG00000019991	HGF	7	81699006	81770438	protein_coding	372,69	0
ENSG00000150347	ARID5B	10	61901300	62096944	protein_coding	371,93	0
ENSG00000069399	BCL3	19	44747705	44760044	protein_coding	371,85	0
ENSG00000090382	LYZ	12	69348341	69354234	protein_coding	371,50	0
ENSG00000150687	PRSS23	11	86791059	86952910	protein_coding	370,14	0
ENSG00000164068	RNF123	3	49689499	49721529	protein_coding	369,76	0
ENSG00000165272	AQP3	9	33441154	33447611	protein_coding	369,75	0
ENSG00000179542	SLITRK4	X	143622790	143635777	protein_coding	369,30	0
ENSG00000127920	GNG11	7	93921699	93928610	protein_coding	368,80	0
ENSG00000050327	ARHGEF5	7	144355288	144380632	protein_coding	368,50	0
ENSG00000258839	MC1R	16	89912119	89920977	protein_coding	368,40	0
ENSG00000123143	PKN1	19	14433053	14471867	protein_coding	368,39	0
ENSG00000162729	IGSF8	1	160091340	160098943	protein_coding	368,00	0
ENSG00000130775	THEMIS2	1	27872543	27886685	protein_coding	367,87	0
ENSG00000074590	NUAK1	12	106063340	106140033	protein_coding	367,60	0
ENSG00000225828	FAM229A	1	32361270	32364278	protein_coding	366,70	0
ENSG00000135828	RNASEL	1	182573634	182589256	protein_coding	365,20	0
ENSG00000196961	AP2A1	19	49766968	49807113	protein_coding	364,62	0
ENSG00000136279	DBNL	7	44044640	44069456	protein_coding	363,84	0
ENSG00000131584	ACAP3	1	1292376	1309609	protein_coding	362,73	0
ENSG00000196923	PDLIM7	5	177483394	177497606	protein_coding	362,68	0
ENSG00000134769	DTNA	18	34493290	34891844	protein_coding	360,00	0
ENSG00000149532	CPSF7	11	61402641	61430031	protein_coding	359,65	0
ENSG00000126524	SBDS	7	66987677	66995601	protein_coding	359,58	0
ENSG00000103245	NARFL	16	729753	741329	protein_coding	359,41	0
ENSG00000012124	CD22	19	35319261	35347355	protein_coding	358,60	0
ENSG00000107968	MAP3K8	10	30433937	30461833	protein_coding	358,60	0
ENSG00000170231	FABP6	5	160187367	160238735	protein_coding	358,60	0
ENSG00000145214	DGKQ	4	958887	986895	protein_coding	358,40	0
ENSG00000182095	TNRC18	7	5306790	5425414	protein_coding	357,94	0
ENSG00000184678	HIST2H2BE	1	149842204	149886652	protein_coding	357,93	0
ENSG00000170801	HTRA3	4	8269765	8307111	protein_coding	357,72	0
ENSG00000072310	SREBF1	17	17810399	17837002	protein_coding	357,25	0
ENSG00000090975	PITPNM2	12	122983480	123150015	protein_coding	356,90	0
ENSG00000125430	HS3ST3B1	17	14301083	14349404	protein_coding	356,60	0
ENSG00000026559	KCNG1	20	51003656	51023129	protein_coding	355,70	0
ENSG00000175482	POLD4	11	67350777	67356972	protein_coding	355,50	0
ENSG00000105722	ERF	19	42247572	42255157	protein_coding	355,48	0
ENSG00000160284	SPATC1L	21	46161148	46184476	protein_coding	355,27	0
ENSG00000169242	EFNA1	1	155127460	155134857	protein_coding	354,30	0
ENSG00000127928	GNGT1	7	93591573	93911265	protein_coding	354,20	0
ENSG00000167702	KIFC2	8	144466043	144474202	protein_coding	354,11	0
ENSG00000152527	PLEKHH2	2	43637273	43767987	protein_coding	353,95	0
ENSG00000213694	S1PR3	9	88991447	89005010	protein_coding	353,70	0
ENSG00000100359	SGSM3	22	40370591	40410289	protein_coding	353,67	0
ENSG00000179588	ZFPM1	16	88453317	88537016	protein_coding	353,67	0
ENSG00000084734	GCKR	2	27496842	27523684	protein_coding	353,24	0
ENSG00000087250	MT3	16	56589074	56591088	protein_coding	352,43	0
ENSG00000169957	ZNF768	16	30524001	30526821	protein_coding	352,05	0
ENSG00000104325	DECR1	8	90001405	90052092	protein_coding	352,00	0
ENSG00000122490	PQLC1	18	79902420	79951664	protein_coding	351,49	0
ENSG00000162654	GBP4	1	89181148	89198932	protein_coding	351,30	0
ENSG00000168487	BMP1	8	22164736	22212326	protein_coding	351,08	0

ENSG00000169213	RAB3B	1	51907956	51990764	protein_coding	349,53	0
ENSG00000119535	CSF3R	1	36466043	36483278	protein_coding	349,20	0
ENSG00000091986	CCDC80	3	112596794	112649530	protein_coding	348,08	0
ENSG00000166741	NNMT	11	114257787	114313285	protein_coding	346,40	0
ENSG00000136048	DRAM1	12	101877351	102012130	protein_coding	346,15	0
ENSG00000049089	COL9A2	1	40300487	40317816	protein_coding	346,10	0
ENSG00000100994	PYGB	20	25248069	25298014	protein_coding	344,38	0
ENSG00000171813	PWWP2B	10	132397168	132417863	protein_coding	343,25	0
ENSG00000170379	TCAF2	7	143620950	143730409	protein_coding	340,79	0
ENSG00000112972	HMGCS1	5	43289395	43313512	protein_coding	340,56	0
ENSG00000072042	RDH11	14	67676801	67695814	protein_coding	340,51	0
ENSG00000061656	SPAG4	20	35615892	35621049	protein_coding	339,30	0
ENSG00000071575	TRIB2	2	12716889	12742734	protein_coding	338,96	0
ENSG00000197296	FITM2	20	44302838	44311169	protein_coding	338,75	0
ENSG00000137411	VARS2	6	30908242	30926459	protein_coding	338,63	0
ENSG00000155111	CDK19	6	110609978	110815958	protein_coding	337,84	0
ENSG00000130175	PRKCSH	19	11435288	11450968	protein_coding	337,57	0
ENSG00000198455	ZXDB	X	57591652	57597545	protein_coding	337,36	0
ENSG00000159792	PSKH1	16	67893272	67929678	protein_coding	337,05	0
ENSG00000159496	RGL4	22	23688136	23699176	protein_coding	337,00	0
ENSG00000167549	CORO6	17	29614756	29622907	protein_coding	337,00	0
ENSG00000181234	TMEM132C	12	128267403	128707915	protein_coding	336,95	0
ENSG00000159335	PTMS	12	6765516	6770952	protein_coding	336,91	0
ENSG00000132530	XAF1	17	6755447	6775647	protein_coding	335,60	0
ENSG00000090097	PCBP4	3	51957454	51974016	protein_coding	335,06	0
ENSG00000126391	FRMD8	11	65386599	65413525	protein_coding	334,91	0
ENSG00000104833	TUBB4A	19	6494319	6502848	protein_coding	334,00	0
ENSG00000180902	D2HGDH	2	241734579	241768816	protein_coding	333,95	0
ENSG00000197019	SERTAD1	19	40421592	40426025	protein_coding	333,85	0
ENSG00000222009	BTBD19	1	44808482	44815585	protein_coding	333,28	0
ENSG00000197774	EME2	16	1773207	1781708	protein_coding	333,10	0
ENSG00000137726	FXYD6	11	117836976	117877486	protein_coding	333,00	0
ENSG00000134590	FAM127A	X	135032366	135033546	protein_coding	333,00	0
ENSG00000183722	LHFP	13	39342892	39603528	protein_coding	332,88	0
ENSG00000125637	PSD4	2	113157325	113209396	protein_coding	332,50	0
ENSG00000139192	TAPBPL	12	6451690	6466517	protein_coding	331,60	0
ENSG00000185483	ROR1	1	63774022	64181498	protein_coding	330,75	0
ENSG00000144476	ACKR3	2	236567787	236582358	protein_coding	330,60	0
ENSG00000188211	NCR3LG1	11	17351726	17377341	protein_coding	329,88	0
ENSG00000197249	SERPINA1	14	94376747	94390693	protein_coding	329,60	0
ENSG00000204267	TAP2	6	32821833	32838780	protein_coding	329,60	0
ENSG00000129968	ABHD17A	19	1876810	1885547	protein_coding	329,29	0
ENSG00000221963	APOL6	22	35648395	35668409	protein_coding	328,25	0
ENSG00000102780	DGKH	13	42040036	42256578	protein_coding	327,73	0
ENSG00000173530	TNFRSF10D	8	23135588	23164030	protein_coding	327,00	0
ENSG00000115275	MOGS	2	74461057	74465410	protein_coding	326,98	0
ENSG00000118508	RAB32	6	146543693	146554965	protein_coding	326,60	0
ENSG00000104524	PYCRL	8	143603913	143609773	protein_coding	326,40	0
ENSG00000182141	ZNF708	19	21221500	21329425	protein_coding	325,90	0
ENSG00000124875	CXCL6	4	73836497	73849064	protein_coding	325,80	0
ENSG00000116962	NID1	1	235975830	236065162	protein_coding	325,74	0
ENSG00000170049	KCNAB3	17	7921859	7929803	protein_coding	325,20	0
ENSG00000115255	REEP6	19	1490747	1497927	protein_coding	324,84	0
ENSG00000105737	GRIK5	19	41998321	42069498	protein_coding	324,71	0
ENSG00000189171	S100A13	1	153618787	153631360	protein_coding	324,70	0
ENSG00000089060	SLC8B1	12	113298759	113359493	protein_coding	324,62	0
ENSG00000178947	SMIM10L2A	X	135421943	135428074	protein_coding	324,30	0
ENSG00000178719	GRINA	8	143990058	143993415	protein_coding	324,20	0

ENSG00000146278	PNRC1	6	89080751	89085160	protein_coding	324,19	0
ENSG00000126759	CFP	X	47624213	47630305	protein_coding	323,70	0
ENSG00000128159	TUBGCP6	22	50217689	50244992	protein_coding	323,34	0
ENSG00000086504	MRPL28	16	367384	370527	protein_coding	322,96	0
ENSG00000215041	NEURL4	17	7315628	7329393	protein_coding	322,13	0
ENSG00000161618	ALDH16A1	19	49453169	49471048	protein_coding	321,85	0
ENSG00000165915	SLC39A13	11	47407132	47416501	protein_coding	321,03	0
ENSG00000099385	BCL7C	16	30833626	30894960	protein_coding	321,00	0
ENSG00000101493	ZNF516	18	76358190	76495190	protein_coding	320,56	0
ENSG00000100321	SYNGR1	22	39349925	39385588	protein_coding	320,23	0
ENSG00000197712	FAM114A1	4	38867677	38945739	protein_coding	320,00	0
ENSG00000130224	LRCH2	X	115110616	115234072	protein_coding	319,60	0
ENSG00000088882	CPXM1	20	2794069	2800637	protein_coding	319,40	0
ENSG00000103485	QPRT	16	29679008	29698699	protein_coding	319,40	0
ENSG00000092621	PHGDH	1	119659798	119744215	protein_coding	318,60	0
ENSG00000187601	MAGEH1	X	55452105	55453566	protein_coding	317,90	0
ENSG00000167291	TBC1D16	17	79932343	80035848	protein_coding	317,69	0
ENSG00000141258	SGSM2	17	2337498	2381058	protein_coding	317,68	0
ENSG00000196372	ASB13	10	5638867	5666595	protein_coding	317,40	0
ENSG00000169122	FAM110B	8	57994509	58204279	protein_coding	316,90	0
ENSG00000114166	KAT2B	3	20040023	20154404	protein_coding	316,23	0
ENSG00000102225	CDK16	X	47217860	47229997	protein_coding	316,03	0
ENSG00000094631	HDAC6	X	48801377	48824982	protein_coding	315,83	0
ENSG00000203772	SPRN	10	133420666	133424572	protein_coding	315,30	0
ENSG00000127415	IDUA	4	986997	1004506	protein_coding	314,50	0
ENSG00000134698	AGO4	1	35808172	35857890	protein_coding	314,33	0
ENSG00000149948	HMGA2	12	65824131	65966295	protein_coding	313,40	0
ENSG00000173531	MST1	3	49683947	49689501	protein_coding	313,00	0
ENSG00000254402	LRRC24	8	144522377	144527032	protein_coding	312,64	0
ENSG00000178538	CA8	8	60187347	60281412	protein_coding	312,40	0
ENSG00000185561	TLCD2	17	1702790	1710438	protein_coding	312,17	0
ENSG00000114698	PLSCR4	3	146192339	146251179	protein_coding	311,58	0
ENSG00000099899	TRMT2A	22	20111866	20117392	protein_coding	311,27	0
ENSG00000215252	GOLGA8B	15	34525207	34588503	protein_coding	310,95	0
ENSG00000173193	PARP14	3	122680618	122730840	protein_coding	309,55	0
ENSG00000169016	E2F6	2	11444375	11466177	protein_coding	309,38	0
ENSG00000186174	BCL9L	11	118893875	118925608	protein_coding	309,31	0
ENSG00000169026	MFSD7	4	681829	689441	protein_coding	309,00	0
ENSG00000137504	CREBZF	11	85659708	85682908	protein_coding	308,53	0
ENSG00000134884	ARGLU1	13	106541673	106568164	protein_coding	308,21	0
ENSG00000178773	CPNE7	16	89575768	89597246	protein_coding	307,56	0
ENSG00000214655	ZSWIM8	10	73785582	73801797	protein_coding	307,53	0
ENSG00000183578	TNFAIP8L3	15	51056598	51105276	protein_coding	307,20	0
ENSG00000206262	FOXL2NB	3	138947234	138953451	protein_coding	307,00	0
ENSG00000204394	VARS	6	31777518	31795953	protein_coding	306,63	0
ENSG00000196943	NOP9	14	24299862	24309124	protein_coding	306,05	0
ENSG00000114853	ZBTB47	3	42653684	42665854	protein_coding	305,80	0
ENSG00000101825	MXRA5	X	3308565	3346641	protein_coding	305,80	0
ENSG00000047230	CTPS2	X	16588003	16712936	protein_coding	305,27	0
ENSG00000127863	TNFRSF19	13	23570370	23676104	protein_coding	305,20	0
ENSG00000205978	NYNRIN	14	24398786	24419288	protein_coding	304,40	0
ENSG00000160932	LY6E	8	143017982	143023832	protein_coding	304,30	0
ENSG00000105327	BBC3	19	47220822	47232766	protein_coding	303,90	0
ENSG00000122420	PTGFR	1	78303884	78539749	protein_coding	303,90	0
ENSG00000178802	MPI	15	74890005	74902219	protein_coding	303,64	0
ENSG00000136378	ADAMTS7	15	78759203	78811431	protein_coding	302,90	0
ENSG00000236609	ZNF853	7	6615617	6624290	protein_coding	302,90	0
ENSG00000196208	GREB1	2	11482341	11642788	protein_coding	302,74	0

ENSG00000157617	C2CD2	21	41885112	41953890	protein_coding	302,71	0
ENSG00000124507	PACSIN1	6	34466061	34535231	protein_coding	302,50	0
ENSG00000068137	PLEKHH3	17	42667914	42676994	protein_coding	302,09	0
ENSG00000121716	PILRB	7	100352176	100367733	protein_coding	301,90	0
ENSG00000145247	OCIAD2	4	48885019	48906937	protein_coding	301,75	0
ENSG00000054690	PLEKHH1	14	67533301	67589612	protein_coding	301,73	0
ENSG00000174749	C4orf32	4	112145397	112195256	protein_coding	301,60	0
ENSG00000146386	ABRACL	6	139028682	139043302	protein_coding	301,35	0
ENSG00000161904	LEMD2	6	33771202	33789136	protein_coding	301,24	0
ENSG00000127586	CHTF18	16	788046	800737	protein_coding	301,20	0
ENSG00000138316	ADAMTS14	10	70672803	70762441	protein_coding	300,91	0
ENSG00000006534	ALDH3B1	11	68008578	68029282	protein_coding	300,60	0
ENSG00000120738	EGR1	5	138465490	138469315	protein_coding	300,54	0
ENSG00000116985	BMP8B	1	39757182	39788861	protein_coding	299,69	0
ENSG00000144935	TRPC1	3	142724074	142807888	protein_coding	299,42	0
ENSG00000112242	E2F3	6	20401906	20493715	protein_coding	299,00	0
ENSG00000198960	ARMCX6	X	101615118	101618001	protein_coding	298,40	0
ENSG00000123329	ARHGAP9	12	57472255	57488814	protein_coding	298,38	0
ENSG00000142046	TMEM91	19	41350911	41384083	protein_coding	298,35	0
ENSG00000163040	CCDC74A	2	131527675	131533666	protein_coding	298,00	0
ENSG00000130707	ASS1	9	130444929	130501274	protein_coding	297,40	0
ENSG00000173511	VEGFB	11	64234538	64238793	protein_coding	297,14	0
ENSG00000185950	IRS2	13	109752698	109786568	protein_coding	296,87	0
ENSG00000198464	ZNF480	19	52297177	52325922	protein_coding	296,70	0
ENSG00000166189	HPS6	10	102065390	102068038	protein_coding	295,50	0
ENSG00000172828	CES3	16	66961237	66975148	protein_coding	295,20	0
ENSG00000148218	ALAD	9	113386317	113401333	protein_coding	295,01	0
ENSG00000157193	LRP8	1	53242364	53328070	protein_coding	294,83	0
ENSG00000223865	HLA-DPB1	6	33075926	33087201	protein_coding	294,70	0
ENSG00000185813	PCYT2	17	81900965	81911464	protein_coding	294,42	0
ENSG00000049283	EPN3	17	50532543	50543750	protein_coding	294,27	0
ENSG00000130204	TOMM40	19	44890569	44903689	protein_coding	294,14	0
ENSG00000213859	KCTD11	17	7351889	7354944	protein_coding	293,65	0
ENSG00000183963	SMTN	22	31064105	31104757	protein_coding	293,60	0
ENSG00000170604	IRF2BP1	19	45883607	45886170	protein_coding	292,93	0
ENSG00000139372	TDG	12	103965804	103988874	protein_coding	292,63	0
ENSG00000178301	AQP11	11	77589391	77610355	protein_coding	292,54	0
ENSG00000171476	HOPX	4	56647988	56681899	protein_coding	292,20	0
ENSG00000146587	RBAK	7	5045821	5069488	protein_coding	292,15	0
ENSG00000138650	PCDH10	4	133149315	133208606	protein_coding	292,10	0
ENSG00000108379	WNT3	17	46762506	46833154	protein_coding	291,88	0
ENSG00000196739	COL27A1	9	114155560	114312511	protein_coding	291,09	0
ENSG00000186416	NKRF	X	119588337	119605895	protein_coding	290,90	0
ENSG00000105612	DNASE2	19	12875211	12881468	protein_coding	290,73	0
ENSG00000172936	MYD88	3	38138478	38143022	protein_coding	289,48	0
ENSG00000143319	ISG20L2	1	156721891	156728799	protein_coding	288,19	0
ENSG00000142192	APP	21	25880550	26171128	protein_coding	287,38	0
ENSG00000104332	SFRP1	8	41261958	41309497	protein_coding	286,67	0
ENSG00000185507	IRF7	11	612553	615999	protein_coding	286,40	0
ENSG00000167394	ZNF668	16	31060843	31074320	protein_coding	285,50	0
ENSG00000118526	TCF21	6	133889138	133895553	protein_coding	285,30	0
ENSG00000142102	ATHL1	11	289135	296107	protein_coding	283,90	0
ENSG00000185022	MAFF	22	38200767	38216511	protein_coding	283,78	0
ENSG00000149499	EML3	11	62602218	62612765	protein_coding	282,82	0
ENSG00000104361	NIPAL2	8	98189833	98294393	protein_coding	282,60	0
ENSG00000176842	IRX5	16	54930862	54934485	protein_coding	281,80	0
ENSG00000178662	CSRNP3	2	165469647	165689407	protein_coding	281,17	0
ENSG00000130522	JUND	19	18279760	18281622	protein_coding	280,79	0

ENSG00000138496	PARP9	3	122527924	122564577	protein_coding	280,69	0
ENSG00000088881	EBF4	20	2692878	2760108	protein_coding	280,46	0
ENSG00000243449	C4orf48	4	2041993	2043970	protein_coding	279,27	0
ENSG00000113494	PRLR	5	35048756	35230589	protein_coding	279,07	0
ENSG00000125733	TRIP10	19	6737925	6751526	protein_coding	278,86	0
ENSG00000188760	TMEM198	2	219543663	219550595	protein_coding	278,80	0
ENSG00000128000	ZNF780B	19	40028260	40056209	protein_coding	278,50	0
ENSG00000140678	ITGAX	16	31355134	31382997	protein_coding	277,64	0
ENSG00000183779	ZNF703	8	37695751	37700021	protein_coding	277,60	0
ENSG00000123989	CHPF	2	219538947	219543787	protein_coding	276,54	0
ENSG00000184584	TMEM173	5	139475534	139482935	protein_coding	276,30	0
ENSG00000112655	PTK7	6	43076268	43161719	protein_coding	275,56	0
ENSG00000109790	KLHL5	4	39045039	39126857	protein_coding	275,53	0
ENSG00000204103	MAFB	20	40685848	40689240	protein_coding	275,18	0
ENSG00000204305	AGER	6	32180968	32184324	protein_coding	275,00	0
ENSG00000103876	FAH	15	80152490	80186946	protein_coding	274,18	0
ENSG00000110074	FOXRED1	11	126269055	126278131	protein_coding	273,67	0
ENSG00000085265	FCN1	9	134905890	134917963	protein_coding	273,20	0
ENSG00000186350	RXRA	9	134317098	134440585	protein_coding	273,15	0
ENSG00000142632	ARHGEF19	1	16197854	16212609	protein_coding	273,00	0
ENSG00000162542	TMCO4	1	19682213	19799945	protein_coding	272,90	0
ENSG00000174705	SH3PXD2B	5	172325181	172454523	protein_coding	272,87	0
ENSG00000106009	BRAT1	7	2537877	2555727	protein_coding	272,66	0
ENSG00000165458	INPPL1	11	72223701	72239105	protein_coding	272,55	0
ENSG00000161642	ZNF385A	12	54369133	54391298	protein_coding	272,40	0
ENSG00000065183	WDR3	1	117929720	117966542	protein_coding	272,16	0
ENSG00000135439	AGAP2	12	57723761	57742157	protein_coding	272,00	0
ENSG00000089057	SLC23A2	20	4852356	5010293	protein_coding	271,43	0
ENSG00000181191	PJA1	X	69160851	69165793	protein_coding	271,11	0
ENSG00000161547	SRSF2	17	76734115	76737374	protein_coding	270,99	0
ENSG00000171659	GPR34	X	41688973	41697277	protein_coding	270,90	0
ENSG00000109472	CPE	4	165361194	165498320	protein_coding	269,54	0
ENSG00000176928	GCNT4	5	75025346	75030899	protein_coding	269,50	0
ENSG00000173545	ZNF622	5	16451519	16465792	protein_coding	268,27	0
ENSG00000158773	USF1	1	161039251	161045977	protein_coding	267,51	0
ENSG00000137944	CCBL2	1	88935773	88992953	protein_coding	266,44	0
ENSG00000101255	TRIB3	20	362835	397559	protein_coding	265,90	0
ENSG00000164308	ERAP2	5	96875939	96919716	protein_coding	265,80	0
ENSG00000102871	TRADD	16	67154180	67160298	protein_coding	265,18	0
ENSG00000124508	BTN2A2	6	26383096	26394874	protein_coding	265,10	0
ENSG00000148803	FUOM	10	133355154	133358035	protein_coding	264,50	0
ENSG00000239264	TXNDC5	6	7881517	7910814	protein_coding	264,30	0
ENSG00000149115	TNKS1BP1	11	57299638	57324952	protein_coding	264,00	0
ENSG00000100726	TELO2	16	1493344	1510457	protein_coding	263,23	0
ENSG00000166483	WEE1	11	9573681	9593457	protein_coding	263,00	0
ENSG00000170458	CD14	5	140631728	140633701	protein_coding	262,80	0
ENSG00000136111	TBC1D4	13	75284665	75482114	protein_coding	262,70	0
ENSG00000143248	RGS5	1	163111121	163321791	protein_coding	262,40	0
ENSG00000131019	ULBP3	6	150063150	150069095	protein_coding	262,10	0
ENSG00000197226	TBC1D9B	5	179862066	179907859	protein_coding	261,64	0
ENSG00000204642	HLA-F	6	29722775	29738528	protein_coding	261,40	0
ENSG00000174684	B4GAT1	11	66345372	66347692	protein_coding	261,36	0
ENSG00000008517	IL32	16	3065297	3082192	protein_coding	260,90	0
ENSG00000134575	ACP2	11	47239302	47248906	protein_coding	259,85	0
ENSG00000017483	SLC38A5	X	48458537	48470256	protein_coding	259,00	0
ENSG00000158106	RHPN1	8	143368855	143384220	protein_coding	258,55	0
ENSG00000240184	PCDHGC3	5	141475947	141512979	protein_coding	258,50	0
ENSG00000117226	GBP3	1	89006666	89022894	protein_coding	258,50	0

ENSG00000079435	LIPE	19	42401507	42427426	protein_coding	258,41	0
ENSG00000063587	ZNF275	X	153334155	153360110	protein_coding	258,32	0
ENSG00000135763	URB2	1	229626234	229660199	protein_coding	258,30	0
ENSG00000213199	ASIC3	7	151048292	151052756	protein_coding	257,57	0
ENSG00000143774	GUK1	1	228139962	228148984	protein_coding	257,53	0
ENSG00000100889	PCK2	14	24094053	24110598	protein_coding	257,40	0
ENSG00000115267	IFIH1	2	162267079	162318703	protein_coding	257,30	0
ENSG00000167772	ANGPTL4	19	8363289	8374373	protein_coding	257,21	0
ENSG00000205336	ADGRG1	16	57610652	57665580	protein_coding	255,15	0
ENSG00000163702	IL17RC	3	9917074	9933630	protein_coding	254,65	0
ENSG00000089639	GMIP	19	19629476	19643667	protein_coding	254,60	0
ENSG00000115935	WIPF1	2	174559572	174682916	protein_coding	253,36	0
ENSG00000115828	QPCT	2	37344574	37373322	protein_coding	253,30	0
ENSG00000041880	PARP3	3	51942345	51948867	protein_coding	252,40	0
ENSG00000110344	UBE4A	11	118359585	118399211	protein_coding	252,28	0
ENSG00000113721	PDGFRB	5	150113837	150155872	protein_coding	252,00	0
ENSG00000126243	LRFN3	19	35935358	35945767	protein_coding	251,62	0
ENSG00000147027	TMEM47	X	34627064	34657288	protein_coding	251,60	0
ENSG00000102390	PBDC1	X	76172936	76178204	protein_coding	250,67	0
ENSG00000125534	PPDPF	20	63520724	63522206	protein_coding	250,47	0
ENSG00000167996	FTH1	11	61959718	61967660	protein_coding	250,07	0
ENSG00000176903	PNMA1	14	73711783	73714372	protein_coding	249,54	0
ENSG00000169047	IRS1	2	226731317	226799759	protein_coding	249,42	0
ENSG00000226742	HSBP1L1	18	79964561	79970822	protein_coding	249,20	0
ENSG00000127951	FGL2	7	77193371	77199826	protein_coding	249,10	0
ENSG00000165996	HACD1	10	17589032	17617377	protein_coding	249,09	0
ENSG00000143851	PTPN7	1	202147013	202161588	protein_coding	249,00	0
ENSG00000175634	RPS6KB2	11	67428460	67435408	protein_coding	248,98	0
ENSG00000198931	APRT	16	88809339	88811944	protein_coding	248,74	0
ENSG00000185122	HSF1	8	144291591	144314722	protein_coding	248,69	0
ENSG00000147124	ZNF41	X	47445879	47482946	protein_coding	248,65	0
ENSG00000075426	FOSL2	2	28392448	28417312	protein_coding	248,56	0
ENSG00000173638	SLC19A1	21	45493572	45544411	protein_coding	248,29	0
ENSG00000167778	SPRYD3	12	53064316	53079420	protein_coding	248,24	0
ENSG00000143891	GALM	2	38665910	38741237	protein_coding	248,12	0
ENSG00000137070	IL11RA	9	34650702	34661892	protein_coding	248,10	0
ENSG00000149925	ALDOA	16	30053090	30070457	protein_coding	247,30	0
ENSG00000092096	SLC22A17	14	23346306	23352912	protein_coding	246,80	0
ENSG00000116455	WDR77	1	111439890	111449376	protein_coding	246,77	0
ENSG00000112562	SMOC2	6	168441151	168673445	protein_coding	246,71	0
ENSG00000178921	PFAS	17	8247618	8270491	protein_coding	245,23	0
ENSG00000186297	GABRA5	15	26866363	26949207	protein_coding	245,20	0
ENSG00000156103	MMP16	8	88032009	88328025	protein_coding	244,77	0
ENSG00000247596	TWF2	3	52228610	52239260	protein_coding	244,74	0
ENSG00000005700	IBTK	6	82169983	82247754	protein_coding	244,64	0
ENSG00000160307	S100B	21	46598962	46605208	protein_coding	244,50	0
ENSG00000091490	SEL1L3	4	25747427	25863760	protein_coding	244,00	0
ENSG00000243978	RGAG1	X	110358816	110456334	protein_coding	243,95	0
ENSG00000150893	FREM2	13	38687129	38887131	protein_coding	243,73	0
ENSG00000105711	SCN1B	19	35030684	35040448	protein_coding	243,70	0
ENSG00000160469	BRSK1	19	55282072	55312533	protein_coding	243,18	0
ENSG00000116260	QSOX1	1	180154834	180204030	protein_coding	243,05	0
ENSG00000213398	LCAT	16	67939750	67944131	protein_coding	242,60	0
ENSG00000240771	ARHGEF25	12	57610180	57619379	protein_coding	242,20	0
ENSG00000177409	SAMD9L	7	93130055	93148369	protein_coding	242,20	0
ENSG00000161509	GRIN2C	17	74842023	74861504	protein_coding	242,00	0
ENSG00000132965	ALOX5AP	13	30713478	30764426	protein_coding	241,70	0
ENSG00000163563	MNDA	1	158831317	158849506	protein_coding	241,60	0

ENSG00000130749	ZC3H4	19	47064187	47113752	protein_coding	241,30	0
ENSG00000106636	YKT6	7	44200968	44214294	protein_coding	241,25	0
ENSG00000004399	PLXND1	3	129555175	129606818	protein_coding	240,85	0
ENSG00000163686	ABHD6	3	58237506	58295693	protein_coding	240,10	0
ENSG00000213445	SIPA1	11	65585395	65650930	protein_coding	240,00	0
ENSG00000159714	ZDHHC1	16	67394419	67416833	protein_coding	239,80	0
ENSG00000213246	SUPT4H1	17	58345175	58353093	protein_coding	238,83	0
ENSG00000112874	NUDT12	5	103548855	103562793	protein_coding	238,46	0
ENSG00000001630	CYP51A1	7	92112151	92142952	protein_coding	237,79	0
ENSG00000049130	KITLG	12	88492793	88580851	protein_coding	237,00	0
ENSG00000099821	POLRMT	19	617224	633604	protein_coding	236,43	0
ENSG00000135750	KCNK1	1	233614004	233672512	protein_coding	236,00	0
ENSG00000134318	ROCK2	2	11179761	11348330	protein_coding	235,99	0
ENSG00000134815	DHX34	19	47349281	47382704	protein_coding	235,96	0
ENSG00000068724	TTC7A	2	46916157	47076137	protein_coding	235,90	0
ENSG00000271601	LIX1L	1	145933423	145958001	protein_coding	235,62	0
ENSG00000104059	FAM189A1	15	29120254	29570723	protein_coding	235,22	0
ENSG00000174307	PHLDA3	1	201464383	201469237	protein_coding	234,73	0
ENSG00000109654	TRIM2	4	153152342	153339320	protein_coding	234,46	0
ENSG00000105643	ARRDC2	19	18001132	18014102	protein_coding	234,29	0
ENSG00000183597	TANGO2	22	20017014	20065926	protein_coding	234,25	0
ENSG00000102699	PARP4	13	24420926	24512810	protein_coding	233,93	0
ENSG00000197162	ZNF785	16	30573740	30585771	protein_coding	233,92	0
ENSG00000152518	ZFP36L2	2	43222402	43226609	protein_coding	233,66	0
ENSG00000139874	SSTR1	14	38207999	38213067	protein_coding	233,60	0
ENSG00000116815	CD58	1	116514535	116571039	protein_coding	233,60	0
ENSG00000204498	NFKBIL1	6	31546870	31558829	protein_coding	233,20	0
ENSG00000185339	TCN2	22	30606838	30627278	protein_coding	233,00	0
ENSG00000110446	SLC15A3	11	60937083	60952530	protein_coding	232,60	0
ENSG00000130881	LRP3	19	33177603	33208867	protein_coding	232,50	0
ENSG00000164823	OSGIN2	8	89901859	89927888	protein_coding	232,37	0
ENSG00000169750	RAC3	17	82031624	82034204	protein_coding	232,30	0
ENSG00000177875	CCDC184	12	48183584	48185926	protein_coding	232,23	0
ENSG00000166147	FBN1	15	48408306	48645849	protein_coding	231,75	0
ENSG00000165688	PMPCA	9	136410570	136423761	protein_coding	231,51	0
ENSG00000048342	CC2D2A	4	15469865	15601557	protein_coding	231,50	0
ENSG00000198925	ATG9A	2	219209772	219229717	protein_coding	231,45	0
ENSG00000162139	NEU3	11	74988134	75018893	protein_coding	230,60	0
ENSG00000127220	ABHD8	19	17292131	17310236	protein_coding	230,50	0
ENSG00000122386	ZNF205	16	3112560	3120517	protein_coding	230,50	0
ENSG00000197580	BCO2	11	112175467	112224699	protein_coding	229,90	0
ENSG00000002933	TMEM176A	7	150800403	150805120	protein_coding	229,90	0
ENSG00000013583	HEBP1	12	12974864	13000273	protein_coding	229,64	0
ENSG00000148334	PTGES2	9	128120693	128128462	protein_coding	229,37	0
ENSG00000124749	COL21A1	6	56056590	56394094	protein_coding	229,30	0
ENSG00000187961	KLHL17	1	960587	965715	protein_coding	229,29	0
ENSG00000213096	ZNF254	19	24033405	24129961	protein_coding	229,00	0
ENSG00000145349	CAMK2D	4	113451032	113761927	protein_coding	228,43	0
ENSG00000173156	RHOD	11	67056818	67072013	protein_coding	228,30	0
ENSG00000182473	EXOC7	17	76081017	76121576	protein_coding	227,49	0
ENSG00000177096	FAM109B	22	42074251	42079441	protein_coding	227,40	0
ENSG00000162526	TSSK3	1	32351521	32364312	protein_coding	227,10	0
ENSG00000213719	CLIC1	6	31730581	31739763	protein_coding	226,91	0
ENSG00000229809	ZNF688	16	30569346	30572734	protein_coding	226,60	0
ENSG00000012211	PRICKLE3	X	49175264	49186528	protein_coding	226,30	0
ENSG00000198682	PAPSS2	10	87659613	87747705	protein_coding	225,81	0
ENSG00000163071	SPATA18	4	52051331	52097292	protein_coding	225,73	0
ENSG00000184261	KCNK12	2	47516581	47570939	protein_coding	225,62	0

ENSG00000185215	TNFAIP2	14	103123442	103137439	protein_coding	225,30	0
ENSG00000239305	RNF103	2	86603393	86623866	protein_coding	224,47	0
ENSG00000197136	PCNXL3	11	65615773	65637439	protein_coding	224,29	0
ENSG00000160803	UBQLN4	1	156035301	156053794	protein_coding	224,23	0
ENSG00000206561	COLQ	3	15450133	15521751	protein_coding	224,20	0
ENSG00000170581	STAT2	12	56341597	56360155	protein_coding	224,13	0
ENSG00000180573	HIST1H2AC	6	26124145	26139116	protein_coding	224,10	0
ENSG00000088888	MAVS	20	3846799	3876123	protein_coding	223,97	0
ENSG00000168010	ATG16L2	11	72814308	72843674	protein_coding	223,65	0
ENSG00000013588	GPRC5A	12	12890782	12917937	protein_coding	223,65	0
ENSG00000196422	PPP1R26	9	135479079	135488893	protein_coding	223,30	0
ENSG00000250067	YJEFN3	19	19528861	19537581	protein_coding	223,20	0
ENSG00000123338	NCKAP1L	12	54497711	54548238	protein_coding	223,20	0
ENSG00000102882	MAPK3	16	30114105	30123506	protein_coding	223,18	0
ENSG00000168961	LGALS9	17	27629798	27649560	protein_coding	222,70	0
ENSG00000139793	MBNL2	13	97221434	97394120	protein_coding	222,20	0
ENSG00000121691	CAT	11	34438925	34472062	protein_coding	222,19	0
ENSG00000142279	WTIP	19	34481638	34512304	protein_coding	222,13	0
ENSG00000183798	EMILIN3	20	41359966	41366827	protein_coding	222,10	0
ENSG00000167992	VWCE	11	61258286	61295424	protein_coding	221,90	0
ENSG00000163879	DNALI1	1	37556919	37566857	protein_coding	221,48	0
ENSG00000160703	NLRX1	11	119166568	119184016	protein_coding	221,40	0
ENSG00000179715	PCED1B	12	47079603	47236662	protein_coding	221,30	0
ENSG00000136448	NMT1	17	45051610	45109016	protein_coding	221,30	0
ENSG00000179627	ZBTB42	14	104800596	104804712	protein_coding	221,20	0
ENSG00000010327	STAB1	3	52495338	52524495	protein_coding	221,09	0
ENSG00000180776	ZDHHC20	13	21372573	21459370	protein_coding	220,91	0
ENSG00000134333	LDHA	11	18394388	18408425	protein_coding	220,76	0
ENSG00000111077	TNS2	12	53046969	53064372	protein_coding	219,88	0
ENSG00000178033	FAM26E	6	116511646	116524792	protein_coding	219,30	0
ENSG00000033100	CHPF2	7	151232489	151238827	protein_coding	219,27	0
ENSG00000070614	NDST1	5	150485818	150558211	protein_coding	219,25	0
ENSG00000092094	OSGEP	14	20446411	20455105	protein_coding	219,13	0
ENSG00000158966	CACHD1	1	64470792	64693058	protein_coding	218,77	0
ENSG00000204272	LINC01420	X	56729259	56818380	protein_coding	217,56	0
ENSG00000171105	INSR	19	7112255	7294034	protein_coding	217,16	0
ENSG00000143409	FAM63A	1	150996086	151008375	protein_coding	217,00	0
ENSG00000239857	GET4	7	876552	896436	protein_coding	216,60	0
ENSG00000111801	BTN3A3	6	26440472	26453415	protein_coding	216,40	0
ENSG00000125740	FOSB	19	45467995	45475179	protein_coding	216,03	0
ENSG00000101856	PGRMC1	X	119236245	119244466	protein_coding	216,02	0
ENSG00000171314	PGAM1	10	97426160	97433441	protein_coding	215,91	0
ENSG00000136274	NACAD	7	45080438	45088914	protein_coding	215,84	0
ENSG00000184371	CSF1	1	109910242	109930992	protein_coding	215,60	0
ENSG00000131669	NINJ1	9	93121489	93134288	protein_coding	215,55	0
ENSG00000177548	RABEP2	16	28904421	28936526	protein_coding	215,44	0
ENSG00000172432	GTPBP2	6	43605316	43629162	protein_coding	215,38	0
ENSG00000149541	B3GAT3	11	62615296	62622175	protein_coding	215,38	0
ENSG00000112139	MDGA1	6	37630679	37699306	protein_coding	215,20	0
ENSG00000014216	CAPN1	11	65180566	65212006	protein_coding	215,13	0
ENSG00000139636	LMBR1L	12	49096551	49110900	protein_coding	215,05	0
ENSG00000119632	IFI27L2	14	94127779	94130253	protein_coding	213,78	0
ENSG00000112473	SLC39A7	6	33200445	33204439	protein_coding	213,60	0
ENSG00000169851	PCDH7	4	30720415	31146805	protein_coding	213,50	0
ENSG00000160113	NR2F6	19	17231883	17245940	protein_coding	212,88	0
ENSG00000066322	ELOVL1	1	43363397	43368074	protein_coding	212,83	0
ENSG00000160781	PAQR6	1	156243321	156248117	protein_coding	212,70	0
ENSG00000169515	CCDC8	19	46410372	46413584	protein_coding	212,60	0

ENSG00000165916	PSMC3	11	47418769	47426473	protein_coding	212,34	0
ENSG00000007129	CEACAM21	19	41549518	41586844	protein_coding	212,10	0
ENSG00000175264	CHST1	11	45648877	45665622	protein_coding	211,80	0
ENSG00000143772	ITPKB	1	226631690	226739323	protein_coding	211,70	0
ENSG00000166670	MMP10	11	102770503	102780628	protein_coding	211,40	0
ENSG00000108840	HDAC5	17	44076746	44123702	protein_coding	211,38	0
ENSG00000155254	MARVELD1	10	97713173	97718152	protein_coding	211,27	0
ENSG00000127507	ADGRE2	19	14732393	14778541	protein_coding	211,10	0
ENSG00000073605	GSDMB	17	39904595	39919854	protein_coding	210,80	0
ENSG00000107738	C10orf54	10	71747559	71773498	protein_coding	210,60	0
ENSG00000106479	ZNF862	7	149838367	149867479	protein_coding	210,58	0
ENSG00000088827	SIGLEC1	20	3686970	3707128	protein_coding	210,40	0
ENSG00000183397	C19orf71	19	3539154	3544030	protein_coding	210,20	0
ENSG00000165424	ZCCHC24	10	79382325	79445627	protein_coding	209,89	0
ENSG00000068903	SIRT2	19	38878555	38899862	protein_coding	209,83	0
ENSG00000113719	ERGIC1	5	172834275	172952685	protein_coding	209,76	0
ENSG00000106077	ABHD11	7	73736094	73738867	protein_coding	209,72	0
ENSG00000163344	PMVK	1	154924734	154936991	protein_coding	209,69	0
ENSG00000174738	NR1D2	3	23945260	23980618	protein_coding	209,60	0
ENSG00000165194	PCDH19	X	100291644	100410273	protein_coding	209,52	0
ENSG00000176022	B3GALT6	1	1232265	1235041	protein_coding	208,68	0
ENSG00000155008	APOOL	X	85003826	85093316	protein_coding	208,65	0
ENSG00000142871	CYR61	1	85580761	85583962	protein_coding	208,50	0
ENSG00000189339	SLC35E2B	1	1659529	1692728	protein_coding	208,38	0
ENSG00000163346	PBXIP1	1	154944076	154956123	protein_coding	207,95	0
ENSG00000185499	MUC1	1	155185824	155192916	protein_coding	207,80	0
ENSG00000203747	FCGR3A	1	161541759	161550737	protein_coding	207,80	0
ENSG00000162572	SCNN1D	1	1280436	1292029	protein_coding	207,60	0
ENSG00000213853	EMP2	16	10528422	10580698	protein_coding	207,14	0
ENSG00000089050	RBBP9	20	18486540	18497243	protein_coding	207,00	0
ENSG00000136193	SCRN1	7	29920103	29990289	protein_coding	206,66	0
ENSG00000026036	RTEL1-TNFR	20	63659300	63698684	protein_coding	206,30	0
ENSG00000184207	PGP	16	2211997	2214807	protein_coding	206,28	0
ENSG00000239779	WBP1	2	74458329	74460891	protein_coding	206,00	0
ENSG00000169239	CA5B	X	15688830	15788409	protein_coding	205,90	0
ENSG00000109846	CRYAB	11	111908565	111923722	protein_coding	205,90	0
ENSG00000185404	SP140L	2	230327184	230403732	protein_coding	205,46	0
ENSG00000124762	CDKN1A	6	36676460	36687339	protein_coding	205,18	0
ENSG00000155393	HEATR3	16	50065941	50106387	protein_coding	205,12	0
ENSG00000119326	CTNNAL1	9	108942569	109013529	protein_coding	205,11	0
ENSG00000253276	CCDC71L	7	106656765	106660996	protein_coding	204,80	0
ENSG00000179909	ZNF154	19	57697367	57709194	protein_coding	204,70	0
ENSG00000138131	LOXL4	10	98247690	98268250	protein_coding	204,50	0
ENSG00000110080	ST3GAL4	11	126355640	126440344	protein_coding	204,49	0
ENSG00000018699	TTC27	2	32628032	32821051	protein_coding	204,46	0
ENSG00000271383	NBPF19	1	149390623	149556361	protein_coding	204,40	0
ENSG00000165682	CLEC1B	12	9985642	10013424	protein_coding	204,30	0
ENSG00000011028	MRC2	17	62627401	62693597	protein_coding	204,21	0
ENSG00000148600	CDHR1	10	84194635	84219621	protein_coding	203,80	0
ENSG00000136205	TNS3	7	47275154	47582558	protein_coding	203,80	0
ENSG00000242732	RGAG4	X	72127110	72131901	protein_coding	203,40	0
ENSG00000136732	GYPC	2	126655933	126696675	protein_coding	203,10	0
ENSG00000138172	CALHM2	10	103446786	103452402	protein_coding	203,08	0
ENSG00000177352	CCDC71	3	49162535	49166321	protein_coding	202,81	0
ENSG00000102924	CBLN1	16	49277917	49281831	protein_coding	202,79	0
ENSG00000244094	SPRR2F	1	153112114	153113515	protein_coding	202,30	0
ENSG00000163220	S100A9	1	153357854	153361027	protein_coding	201,90	0
ENSG00000178726	THBD	20	23045633	23049741	protein_coding	201,80	0

ENSG00000166188	ZNF319	16	57994668	58000453	protein_coding	201,40	0
ENSG00000170915	PAQR8	6	52361421	52407777	protein_coding	201,11	0
ENSG00000151640	DPYSL4	10	132186900	132205776	protein_coding	201,10	0
ENSG00000105227	PRX	19	40393768	40413366	protein_coding	200,80	0
ENSG00000133027	PEMT	17	17505563	17591708	protein_coding	200,30	0
ENSG00000150990	DHX37	12	124946825	124989122	protein_coding	199,55	0
ENSG00000119403	PHF19	9	120855652	120894896	protein_coding	199,00	0
ENSG00000137033	IL33	9	6215786	6257983	protein_coding	199,00	0
ENSG00000137965	IFI44	1	78649796	78664078	protein_coding	198,90	0
ENSG00000171791	BCL2	18	63123346	63320128	protein_coding	198,70	0
ENSG00000163873	GRIK3	1	36795527	37034129	protein_coding	198,53	0
ENSG00000160445	ZER1	9	128729786	128772414	protein_coding	198,50	0
ENSG00000124217	MOCS3	20	50958826	50963931	protein_coding	198,25	0
ENSG00000067191	CACNB1	17	39173456	39197703	protein_coding	198,21	0
ENSG00000100139	MICALL1	22	37905657	37942822	protein_coding	198,20	0
ENSG00000121577	POPDC2	3	119636457	119665324	protein_coding	198,17	0
ENSG00000112787	FBRSL1	12	132489551	132585188	protein_coding	198,07	0
ENSG00000240065	PSMB9	6	32844136	32859585	protein_coding	197,50	0
ENSG00000155966	AFF2	X	148500619	149000663	protein_coding	196,57	0
ENSG00000150540	HNMT	2	137964020	138016364	protein_coding	196,10	0
ENSG00000166148	AVPR1A	12	63142759	63150942	protein_coding	195,94	0
ENSG00000204673	AKT1S1	19	49869033	49878459	protein_coding	195,71	0
ENSG00000100897	DCAF11	14	24114195	24125242	protein_coding	195,65	0
ENSG00000224877	C17orf89	17	81239239	81241281	protein_coding	195,40	0
ENSG00000126821	SGPP1	14	63684214	63728039	protein_coding	195,32	0
ENSG00000078061	ARAF	X	47561100	47571920	protein_coding	195,25	0
ENSG00000182446	NPLOC4	17	81556887	81648465	protein_coding	195,10	0
ENSG00000099256	PRTFDC1	10	24848607	24952604	protein_coding	195,10	0
ENSG00000130653	PNPLA7	9	137459953	137550534	protein_coding	195,07	0
ENSG00000008710	PKD1	16	2088710	2135898	protein_coding	194,99	0
ENSG00000162614	NEXN	1	77888513	77943895	protein_coding	194,80	0
ENSG00000154025	SLC5A10	17	18950345	19022595	protein_coding	194,50	0
ENSG00000172578	KLHL6	3	183487531	183555689	protein_coding	193,30	0
ENSG00000138821	SLC39A8	4	102251041	102431258	protein_coding	193,21	0
ENSG00000006025	OSBPL7	17	47807372	47821834	protein_coding	193,00	0
ENSG00000198898	CAPZA2	7	116811070	116922049	protein_coding	192,98	0
ENSG00000170234	PWWP2A	5	160061801	160119423	protein_coding	192,98	0
ENSG00000128394	APOBEC3F	22	39040604	39053910	protein_coding	192,80	0
ENSG00000120458	MSANTD2	11	124766498	124800673	protein_coding	192,64	0
ENSG00000169896	ITGAM	16	31259990	31332892	protein_coding	192,60	0
ENSG00000148824	MTG1	10	133394094	133421307	protein_coding	192,55	0
ENSG00000177508	IRX3	16	54283304	54286763	protein_coding	192,20	0
ENSG00000174775	HRAS	11	532242	537287	protein_coding	191,89	0
ENSG00000139546	TARBP2	12	53500921	53506431	protein_coding	191,09	0
ENSG00000119280	C1orf198	1	230837119	230869589	protein_coding	191,07	0
ENSG00000142396	ERVK3-1	19	58305319	58315663	protein_coding	190,80	0
ENSG00000182836	PLCXD3	5	41306954	41510628	protein_coding	190,74	0
ENSG00000111348	ARHGDIB	12	14942017	14961728	protein_coding	190,10	0
ENSG00000198797	BRINP2	1	177171497	177282422	protein_coding	190,00	0
ENSG00000165186	PTCHD1	X	23334015	23404372	protein_coding	190,00	0
ENSG00000167995	BEST1	11	61949821	61965515	protein_coding	189,87	0
ENSG00000137338	PGBD1	6	28281572	28302549	protein_coding	189,85	0
ENSG00000204351	SKIV2L	6	31959080	31969755	protein_coding	189,83	0
ENSG00000168792	ABHD15	17	29560547	29567137	protein_coding	189,60	0
ENSG00000158373	HIST1H2BD	6	26158146	26171349	protein_coding	189,60	0
ENSG00000129691	ASH2L	8	38105242	38144076	protein_coding	189,59	0
ENSG00000140688	C16orf58	16	31489471	31509309	protein_coding	189,54	0
ENSG00000149781	FERMT3	11	64206678	64223886	protein_coding	189,50	0

ENSG00000173540	GMPPB	3	49716844	49723951	protein_coding	189,24	0
ENSG00000165804	ZNF219	14	21090046	21104722	protein_coding	189,10	0
ENSG00000218891	ZNF579	19	55576770	55580845	protein_coding	189,00	0
ENSG00000107130	NCS1	9	130172578	130237304	protein_coding	188,82	0
ENSG00000032444	PNPLA6	19	7534004	7561764	protein_coding	188,81	0
ENSG00000119917	IFIT3	10	89327894	89340971	protein_coding	188,80	0
ENSG00000215271	HOMEZ	14	23272422	23299447	protein_coding	188,52	0
ENSG00000139899	CBLN3	14	24426532	24430954	protein_coding	188,00	0
ENSG00000138606	SHF	15	45167214	45201175	protein_coding	187,62	0
ENSG00000141682	PMAIP1	18	59899948	59904306	protein_coding	187,50	0
ENSG00000204065	TCEAL5	X	103273691	103276872	protein_coding	187,20	0
ENSG00000104870	FCGRT	19	49506816	49526333	protein_coding	186,49	0
ENSG00000072778	ACADVL	17	7217125	7225273	protein_coding	186,30	0
ENSG00000099625	CBARP	19	1228287	1238027	protein_coding	186,21	0
ENSG00000165949	IFI27	14	94104836	94116698	protein_coding	185,93	0
ENSG00000099337	KCNK6	19	38319844	38332076	protein_coding	185,88	0
ENSG00000185686	PRAME	22	22547701	22559361	protein_coding	185,75	0
ENSG00000140545	MFGE8	15	88898683	88913411	protein_coding	185,16	0
ENSG00000156113	KCNMA1	10	76869601	77638595	protein_coding	184,83	0
ENSG00000100908	EMC9	14	24138959	24141588	protein_coding	184,79	0
ENSG00000156381	ANKRD9	14	102501760	102509799	protein_coding	184,65	0
ENSG00000164294	GPX8	5	55160118	55167071	protein_coding	184,50	0
ENSG00000111652	COPS7A	12	6723741	6731875	protein_coding	184,48	0
ENSG00000134369	NAV1	1	201622885	201826969	protein_coding	184,46	0
ENSG00000163644	PPM1K	4	88257620	88284769	protein_coding	184,40	0
ENSG00000139641	ESYT1	12	56118250	56144671	protein_coding	184,30	0
ENSG00000177283	FZD8	10	35638249	35642278	protein_coding	184,10	0
ENSG00000180353	HCLS1	3	121631399	121660927	protein_coding	183,50	0
ENSG00000132357	CARD6	5	40841184	40860175	protein_coding	183,40	0
ENSG00000101265	RASSF2	20	4780023	4823645	protein_coding	183,35	0
ENSG00000198768	APCDD1L	20	58459101	58515131	protein_coding	183,20	0
ENSG00000076716	GPC4	X	133300103	133415490	protein_coding	183,14	0
ENSG00000198952	SMG5	1	156249224	156282825	protein_coding	183,13	0
ENSG00000211445	GPX3	5	151020438	151028993	protein_coding	182,95	0
ENSG00000166848	TERF2IP	16	75647786	75761872	protein_coding	182,85	0
ENSG00000198889	DCAF12L1	X	126549383	126552851	protein_coding	182,40	0
ENSG00000105339	DENND3	8	141117278	141195808	protein_coding	182,39	0
ENSG00000114735	HEMK1	3	50569152	50596168	protein_coding	181,94	0
ENSG00000095321	CRAT	9	129094810	129111189	protein_coding	181,81	0
ENSG00000168781	PPIP5K1	15	43533462	43590253	protein_coding	181,73	0
ENSG00000119943	PYROXD2	10	98383565	98415184	protein_coding	181,60	0
ENSG00000187837	HIST1H1C	6	26055787	26056428	protein_coding	181,57	0
ENSG00000162747	FCGR3B	1	161623196	161631963	protein_coding	181,40	0
ENSG00000189292	FAM150B	2	279558	288851	protein_coding	181,30	0
ENSG00000174151	CYB561D1	1	109494052	109502932	protein_coding	180,90	0
ENSG00000138835	RGS3	9	113444731	113597743	protein_coding	180,87	0
ENSG00000169981	ZNF35	3	44648727	44660791	protein_coding	180,82	0
ENSG00000100151	PICK1	22	38056311	38075701	protein_coding	180,42	0
ENSG00000254585	MAGEL2	15	23643544	23647841	protein_coding	180,40	0
ENSG00000130988	RGN	X	47078355	47093314	protein_coding	180,40	0
ENSG00000178691	SUZ12	17	31937018	32001045	protein_coding	180,17	0
ENSG00000141698	NT5C3B	17	41825181	41836263	protein_coding	179,90	0
ENSG00000164880	INTS1	7	1470277	1504367	protein_coding	179,82	0
ENSG00000198055	GRK6	5	177403204	177442901	protein_coding	179,61	0
ENSG00000185985	SLITRK2	X	145817832	145825842	protein_coding	179,40	0
ENSG00000082014	SMARCD3	7	151238764	151277896	protein_coding	179,38	0
ENSG00000130731	C16orf13	16	634427	636366	protein_coding	179,24	0
ENSG00000185920	PTCH1	9	95442980	95517057	protein_coding	179,05	0

ENSG00000100918	REC8	14	24171853	24180257	protein_coding	179,00	0
ENSG00000213047	DENND1B	1	197504748	197775696	protein_coding	179,00	0
ENSG00000169231	THBS3	1	155195588	155209051	protein_coding	178,69	0
ENSG00000007392	LUC7L	16	188969	229463	protein_coding	178,42	0
ENSG00000124212	PTGIS	20	49503874	49568146	protein_coding	178,40	0
ENSG00000170296	GABARAP	17	7240014	7242770	protein_coding	177,95	0
ENSG00000154556	SORBS2	4	185585444	185956652	protein_coding	177,79	0
ENSG00000174227	PIGG	4	499210	540196	protein_coding	177,69	0
ENSG00000155926	SLA	8	133036724	133103054	protein_coding	177,50	0
ENSG00000107957	SH3PXD2A	10	103594027	103855543	protein_coding	177,40	0
ENSG00000254087	LYN	8	55879813	56014168	protein_coding	177,20	0
ENSG00000103187	COTL1	16	84565594	84618077	protein_coding	177,07	0
ENSG00000164543	STK17A	7	43582758	43626786	protein_coding	176,69	0
ENSG00000137941	TTLL7	1	83865028	83999150	protein_coding	176,64	0
ENSG00000235173	HGH1	8	144137769	144140843	protein_coding	176,50	0
ENSG00000180398	MCFD2	2	46901870	46941855	protein_coding	176,34	0
ENSG00000100478	AP4S1	14	31025106	31096450	protein_coding	175,80	0
ENSG00000136840	ST6GALNAC	9	127907886	127917038	protein_coding	175,70	0
ENSG00000163428	LRRC58	3	120324509	120349339	protein_coding	175,36	0
ENSG00000042286	AIFM2	10	70098223	70132934	protein_coding	175,30	0
ENSG00000130962	PRRG1	X	37349275	37457295	protein_coding	175,26	0
ENSG00000099364	FBXL19	16	30923055	30948783	protein_coding	175,02	0
ENSG00000184216	IRAK1	X	154010500	154019980	protein_coding	174,97	0
ENSG00000198502	HLA-DRB5	6	32517343	32530287	protein_coding	174,80	0
ENSG00000128602	SMO	7	129188872	129213545	protein_coding	174,50	0
ENSG00000143554	SLC27A3	1	153774354	153780157	protein_coding	174,20	0
ENSG00000100083	GGA1	22	37608475	37633564	protein_coding	174,11	0
ENSG00000137486	ARRB1	11	75264182	75351705	protein_coding	174,09	0
ENSG00000127124	HIVEP3	1	41506365	42035925	protein_coding	173,91	0
ENSG00000124313	IQSEC2	X	53232876	53321324	protein_coding	173,80	0
ENSG00000138061	CYP1B1	2	38066973	38109902	protein_coding	173,74	0
ENSG00000161640	SIGLEC11	19	49948985	49961172	protein_coding	173,60	0
ENSG00000085644	ZNF213	16	3129777	3142805	protein_coding	173,48	0
ENSG00000188803	SHISA6	17	11241263	11564063	protein_coding	173,40	0
ENSG00000090013	BLVRB	19	40447789	40465840	protein_coding	173,36	0
ENSG00000102309	PIN4	X	72181353	72302926	protein_coding	173,33	0
ENSG00000141994	DUS3L	19	5784832	5791238	protein_coding	173,28	0
ENSG00000157502	MUM1L1	X	106168305	106208956	protein_coding	173,24	0
ENSG00000131591	C1orf159	1	1081818	1116361	protein_coding	173,24	0
ENSG00000162931	TRIM17	1	228407940	228416861	protein_coding	173,20	0
ENSG00000166265	CYYR1	21	26466209	26573284	protein_coding	173,10	0
ENSG00000138111	TMEM180	10	102461395	102477045	protein_coding	172,80	0
ENSG00000179593	ALOX15B	17	8039017	8049134	protein_coding	172,40	0
ENSG00000197093	GAL3ST4	7	100159244	100168750	protein_coding	172,40	0
ENSG00000174939	ASPHD1	16	29900375	29919864	protein_coding	172,33	0
ENSG00000165359	DDX26B	X	135520659	135582510	protein_coding	172,10	0
ENSG00000127463	EMC1	1	19215664	19251552	protein_coding	171,64	0
ENSG00000180773	SLC36A4	11	93144171	93197964	protein_coding	171,58	0
ENSG00000102057	KCND1	X	48961378	48971569	protein_coding	170,90	0
ENSG00000189143	CLDN4	7	73799542	73832693	protein_coding	170,73	0
ENSG00000177106	EPS8L2	11	694438	727727	protein_coding	170,50	0
ENSG00000275074	NUDT18	8	22106872	22109419	protein_coding	170,50	0
ENSG00000079215	SLC1A3	5	36606355	36688334	protein_coding	170,50	0
ENSG00000161956	SENP3	17	7561875	7571969	protein_coding	170,40	0
ENSG00000124587	PEX6	6	42963870	42979220	protein_coding	170,28	0
ENSG00000113083	LOX	5	122063195	122078285	protein_coding	170,20	0
ENSG00000147041	SYTL5	X	38006582	38128819	protein_coding	169,90	0
ENSG00000088280	ASAP3	1	23428563	23484568	protein_coding	169,77	0

ENSG00000100241	SBF1	22	50445000	50475024	protein_coding	169,51	0
ENSG00000204839	MROH6	8	143566187	143572971	protein_coding	169,36	0
ENSG00000165140	FBP1	9	94603133	94640249	protein_coding	169,30	0
ENSG00000125538	IL1B	2	112829751	112836903	protein_coding	169,20	0
ENSG00000135409	AMHR2	12	53423855	53431534	protein_coding	168,50	0
ENSG00000166432	ZMAT1	X	101882288	101932031	protein_coding	168,43	0
ENSG00000111671	SPSB2	12	6870935	6889358	protein_coding	168,29	0
ENSG00000166923	GREM1	15	32717974	32745107	protein_coding	168,12	0
ENSG00000187474	FPR3	19	51795163	51826189	protein_coding	167,90	0
ENSG00000132879	FBXO44	1	11654375	11663327	protein_coding	167,80	0
ENSG00000159212	CLIC6	21	34669389	34718227	protein_coding	167,70	0
ENSG00000130511	SSBP4	19	18418864	18434562	protein_coding	167,67	0
ENSG00000165030	NFIL3	9	91409045	91423862	protein_coding	167,43	0
ENSG00000099849	RASSF7	11	560404	564021	protein_coding	167,42	0
ENSG00000110324	IL10RA	11	117986348	118001483	protein_coding	167,42	0
ENSG00000187244	BCAM	19	44809059	44821420	protein_coding	167,30	0
ENSG00000170262	MRAP	21	32291813	32314784	protein_coding	167,20	0
ENSG00000110090	CPT1A	11	68754620	68844410	protein_coding	167,20	0
ENSG00000130270	ATP8B3	19	1782075	1812276	protein_coding	167,10	0
ENSG00000164221	CCDC112	5	115267188	115296831	protein_coding	167,09	0
ENSG00000136010	ALDH1L2	12	105019784	105084577	protein_coding	167,08	0
ENSG00000156804	FBXO32	8	123497889	123541206	protein_coding	166,99	0
ENSG00000125841	NRSN2	20	346782	359660	protein_coding	166,99	0
ENSG00000107816	LZTS2	10	100996618	101007836	protein_coding	166,70	0
ENSG00000188647	PTAR1	9	69709522	69759959	protein_coding	166,28	0
ENSG00000123427	METTL21B	12	57771492	57782798	protein_coding	166,00	0
ENSG00000143369	ECM1	1	150508062	150513789	protein_coding	166,00	0
ENSG00000196544	BORCS6	17	8188333	8190907	protein_coding	165,92	0
ENSG00000164237	CMBL	5	10275875	10308026	protein_coding	165,91	0
ENSG00000167244	IGF2	11	2129112	2141238	protein_coding	165,82	0
ENSG00000011347	SYT7	11	61515313	61581148	protein_coding	165,59	0
ENSG00000076555	ACACB	12	109116595	109268226	protein_coding	165,32	0
ENSG00000183258	DDX41	5	177511577	177517469	protein_coding	165,30	0
ENSG00000232434	C9orf172	9	136844415	136848801	protein_coding	165,28	0
ENSG00000125354	09/01/2006	X	119615724	119693370	protein_coding	165,04	0
ENSG00000163975	MFI2	3	196988621	197029816	protein_coding	165,00	0
ENSG00000119457	SLC46A2	9	112878920	112890913	protein_coding	164,80	0
ENSG00000064932	SBNO2	19	1107636	1174283	protein_coding	164,50	0
ENSG00000163453	IGFBP7	4	57030773	57110385	protein_coding	164,46	0
ENSG00000213699	SLC35F6	2	26764284	26781231	protein_coding	164,23	0
ENSG00000010610	CD4	12	6786858	6820808	protein_coding	164,10	0
ENSG00000173898	SPTBN2	11	66685248	66729226	protein_coding	164,07	0
ENSG00000141933	TPGS1	19	507497	519654	protein_coding	164,00	0
ENSG00000177427	MIEF2	17	18260534	18266552	protein_coding	163,90	0
ENSG00000114023	FAM162A	3	122384176	122412334	protein_coding	163,81	0
ENSG00000170545	SMAGP	12	51244558	51270890	protein_coding	163,81	0
ENSG00000136059	VILL	3	37988059	38007188	protein_coding	163,73	0
ENSG00000186814	ZSCAN30	18	35251058	35290245	protein_coding	163,73	0
ENSG00000025039	RRAGD	6	89364636	89412270	protein_coding	163,45	0
ENSG00000106948	AKNA	9	114334156	114394405	protein_coding	162,95	0
ENSG00000144681	STAC	3	36380344	36548007	protein_coding	162,89	0
ENSG00000081237	PTPRC	1	198638671	198757283	protein_coding	162,76	0
ENSG00000126091	ST3GAL3	1	43705824	43931165	protein_coding	162,74	0
ENSG00000166750	SLFN5	17	35243036	35273655	protein_coding	162,73	0
ENSG00000155659	VSIG4	X	66021738	66040125	protein_coding	162,60	0
ENSG00000182511	FES	15	90883695	90895776	protein_coding	162,42	0
ENSG00000081014	AP4E1	15	50908672	51005900	protein_coding	162,25	0
ENSG00000151690	MFSD6	2	190408355	190509205	protein_coding	162,05	0

ENSG00000140450	ARRDC4	15	97960698	97973838	protein_coding	161,78	0
ENSG00000160219	GAB3	X	154675249	154751583	protein_coding	161,64	0
ENSG00000157870	FAM213B	1	2586491	2591469	protein_coding	161,07	0
ENSG00000137491	SLCO2B1	11	75100563	75206549	protein_coding	161,00	0
ENSG00000168765	GSTM4	1	109656081	109674836	protein_coding	161,00	0
ENSG00000063660	GPC1	2	240435671	240468078	protein_coding	160,83	0
ENSG00000118946	PCDH17	13	57631810	57729311	protein_coding	160,50	0
ENSG00000188906	LRRK2	12	40196744	40369285	protein_coding	160,20	0
ENSG00000164111	ANXA5	4	121667955	121697113	protein_coding	159,89	0
ENSG00000108387	09/01/2004	17	58520250	58540818	protein_coding	159,70	0
ENSG00000276600	RAB7B	1	205976740	206003461	protein_coding	159,70	0
ENSG00000130717	UCK1	9	131523801	131531268	protein_coding	159,65	0
ENSG00000130827	PLXNA3	X	154458281	154477779	protein_coding	159,37	0
ENSG00000197763	TXNRD3	3	126571779	126655155	protein_coding	159,18	0
ENSG00000184588	PDE4B	1	65792514	66374579	protein_coding	159,10	0
ENSG00000158163	DZIP1L	3	138061990	138115818	protein_coding	158,80	0
ENSG00000172403	SYNPO2	4	118850688	119061247	protein_coding	158,64	0
ENSG00000128564	VGF	7	101162509	101165593	protein_coding	158,30	0
ENSG00000215183	MSMP	9	35752990	35756613	protein_coding	157,83	0
ENSG00000185477	GPRIN3	4	89236386	89308010	protein_coding	157,70	0
ENSG00000183617	MRPL54	19	3762664	3768575	protein_coding	157,66	0
ENSG00000144810	COL8A1	3	99638475	99799226	protein_coding	157,60	0
ENSG00000081985	IL12RB2	1	67307364	67396900	protein_coding	157,50	0
ENSG00000100299	ARSA	22	50622754	50628173	protein_coding	157,46	0
ENSG00000181744	C3orf58	3	143971798	144048719	protein_coding	157,45	0
ENSG00000108784	NAGLU	17	42536172	42544449	protein_coding	157,44	0
ENSG00000146072	TNFRSF21	6	47231532	47309905	protein_coding	157,36	0
ENSG00000007520	TSR3	16	1349240	1351911	protein_coding	157,33	0
ENSG00000267796	LIN37	19	35748361	35754519	protein_coding	157,10	0
ENSG00000204381	LAYN	11	111540280	111561745	protein_coding	157,10	0
ENSG00000148356	LRSAM1	9	127451486	127503501	protein_coding	157,03	0
ENSG00000185885	IFITM1	11	313506	315272	protein_coding	156,70	0
ENSG00000158560	DYNC1I1	7	95772506	96110322	protein_coding	156,70	0
ENSG00000179846	NKPD1	19	45149750	45160150	protein_coding	156,60	0
ENSG00000243678	NME2	17	51165435	51171747	protein_coding	156,42	0
ENSG00000183979	NPB	17	81900745	81902905	protein_coding	156,30	0
ENSG00000168056	LTBP3	11	65538560	65558930	protein_coding	156,26	0
ENSG00000177239	MAN1B1	9	137086927	137109187	protein_coding	155,97	0
ENSG00000164088	PPM1M	3	52245793	52250597	protein_coding	155,90	0
ENSG00000164849	GPR146	7	1044576	1059261	protein_coding	155,70	0
ENSG00000184702	09/01/2005	22	19714464	19724772	protein_coding	155,60	0
ENSG00000073169	ENSG00000(	22	50200979	50217616	protein_coding	155,39	0
ENSG00000175265	GOLGA8A	15	34379068	34437466	protein_coding	155,37	0
ENSG00000083814	ZNF671	19	57719751	57727624	protein_coding	155,27	0
ENSG00000103254	FAM173A	16	720581	722601	protein_coding	154,75	0
ENSG00000171617	ENC1	5	74627406	74641424	protein_coding	154,72	0
ENSG00000212864	RNF208	9	137220247	137221581	protein_coding	154,60	0
ENSG00000103226	NOMO3	16	16232495	16294814	protein_coding	154,40	0
ENSG00000123095	BHLHE41	12	26120026	26125127	protein_coding	154,40	0
ENSG00000215440	NPEPL1	20	58689131	58719238	protein_coding	154,38	0
ENSG00000184675	AMER1	X	64185117	64205744	protein_coding	154,30	0
ENSG00000136877	FPGS	9	127794597	127814327	protein_coding	153,95	0
ENSG00000214357	NEURL1B	5	172641266	172691540	protein_coding	153,90	0
ENSG00000183837	PNMA3	X	153056409	153060467	protein_coding	153,73	0
ENSG00000145242	EPHA5	4	65319563	65670495	protein_coding	153,70	0
ENSG00000105639	JAK3	19	17824780	17848071	protein_coding	153,64	0
ENSG00000005379	BZRAP1	17	58301228	58328760	protein_coding	153,60	0
ENSG00000111669	TPI1	12	6867119	6870948	protein_coding	153,31	0

ENSG00000214706	IFRD2	3	50287732	50292918	protein_coding	153,30	0
ENSG00000163902	RPN1	3	128619970	128681075	protein_coding	153,12	0
ENSG00000182902	SLC25A18	22	17563439	17590994	protein_coding	152,64	0
ENSG00000160271	RALGDS	9	133097720	133149334	protein_coding	152,29	0
ENSG00000108557	RAI1	17	17681473	17811453	protein_coding	152,26	0
ENSG00000102218	RP2	X	46836940	46882358	protein_coding	152,25	0
ENSG00000104518	GSDMD	8	143553207	143563062	protein_coding	152,09	0
ENSG00000162496	DHRS3	1	12567910	12617731	protein_coding	152,08	0
ENSG00000134030	CTIF	18	48539046	48863217	protein_coding	152,05	0
ENSG00000127054	CPSF3L	1	1311585	1324691	protein_coding	151,97	0
ENSG00000155970	MICU3	8	17027238	17122644	protein_coding	151,70	0
ENSG00000185973	TMLHE	X	155490115	155669944	protein_coding	151,50	0
ENSG00000088543	C3orf18	3	50558025	50571027	protein_coding	151,10	0
ENSG00000179344	HLA-DQB1	6	32659467	32668383	protein_coding	151,00	0
ENSG00000111276	CDKN1B	12	12715058	12722371	protein_coding	150,87	0
ENSG00000180667	YOD1	1	207043849	207052980	protein_coding	150,85	0
ENSG00000079150	FKBP7	2	178463664	178478600	protein_coding	150,82	0
ENSG00000087088	BAX	19	48954815	48961798	protein_coding	150,81	0
ENSG00000179604	CDC42EP4	17	73283624	73312175	protein_coding	150,73	0
ENSG00000162777	DENND2D	1	111187174	111204535	protein_coding	150,20	0
ENSG00000124006	OBSL1	2	219550729	219571859	protein_coding	150,18	0
ENSG00000103168	TAF1C	16	84177847	84187070	protein_coding	150,16	0
ENSG00000120049	KCNIP2	10	101825974	101843920	protein_coding	149,90	0
ENSG00000172123	SLFN12	17	35411060	35433283	protein_coding	149,80	0
ENSG00000269190	FBXO17	19	38941401	38975910	protein_coding	149,71	0
ENSG00000125503	PPP1R12C	19	55090913	55117559	protein_coding	149,66	0
ENSG00000117586	TNFSF4	1	173183734	173207313	protein_coding	149,64	0
ENSG00000166275	BORCS7	10	102854223	102864961	protein_coding	149,61	0
ENSG00000170540	ARL6IP1	16	18791667	18801678	protein_coding	149,59	0
ENSG00000183833	MAATS1	3	119703022	119767102	protein_coding	149,58	0
ENSG00000100577	GSTZ1	14	77320884	77331597	protein_coding	149,54	0
ENSG00000151914	DST	6	56457987	56954628	protein_coding	149,53	0
ENSG00000171295	ZNF440	19	11814284	11835201	protein_coding	149,50	0
ENSG00000110876	SELPLG	12	108622277	108633959	protein_coding	149,50	0
ENSG00000181577	C6orf223	6	44000580	44005958	protein_coding	149,20	0
ENSG00000129932	DOHH	19	3490821	3500940	protein_coding	149,17	0
ENSG00000157259	GATAD1	7	92447453	92458836	protein_coding	149,09	0
ENSG00000112658	SRF	6	43171299	43181507	protein_coding	148,79	0
ENSG00000174428	GTF2IRD2B	7	75092573	75149817	protein_coding	148,50	0
ENSG00000139679	LPAR6	13	48389567	48444704	protein_coding	148,45	0
ENSG00000100368	CSF2RB	22	36913628	36940449	protein_coding	148,40	0
ENSG00000171522	PTGER4	5	40679498	40693735	protein_coding	148,40	0
ENSG00000162065	TBC1D24	16	2475118	2505734	protein_coding	148,31	0
ENSG00000226763	SRRM5	19	43596617	43614497	protein_coding	148,26	0
ENSG00000181652	ATG9B	7	151012209	151024499	protein_coding	148,18	0
ENSG00000183323	CCDC125	5	69280175	69332809	protein_coding	148,15	0
ENSG00000111224	PARP11	12	3791047	3873448	protein_coding	148,05	0
ENSG00000109743	BST1	4	15702950	15738313	protein_coding	148,00	0
ENSG00000174099	MSRB3	12	65278643	65488244	protein_coding	147,48	0
ENSG00000089280	FUS	16	31180110	31194871	protein_coding	147,46	0
ENSG00000139044	B4GALNT3	12	460364	563509	protein_coding	147,26	0
ENSG00000136816	TOR1B	9	129803153	129811281	protein_coding	147,14	0
ENSG00000100345	MYH9	22	36281281	36388018	protein_coding	147,12	0
ENSG00000021355	SERPINB1	6	2832332	2842006	protein_coding	147,02	0
ENSG00000179409	GEMIN4	17	744414	753999	protein_coding	146,97	0
ENSG00000181284	TMEM102	17	7435443	7437679	protein_coding	146,60	0
ENSG00000167641	PPP1R14A	19	38251237	38256591	protein_coding	146,50	0
ENSG00000188404	SELL	1	169690667	169711698	protein_coding	146,40	0

ENSG00000160868	CYP3A4	7	99756960	99784265	protein_coding	146,30	0
ENSG00000139726	DENR	12	122752774	122771064	protein_coding	146,26	0
ENSG00000082074	FYB	5	39105236	39274528	protein_coding	146,10	0
ENSG00000119820	YIPF4	2	32277910	32316594	protein_coding	145,90	0
ENSG00000153234	NR4A2	2	156324432	156342348	protein_coding	145,87	0
ENSG00000135821	GLUL	1	182381704	182392206	protein_coding	145,46	0
ENSG00000116833	NR5A2	1	200027602	200177424	protein_coding	145,39	0
ENSG00000125962	ARMCX5	X	102599168	102604159	protein_coding	145,30	0
ENSG00000172164	SNTB1	8	120535745	120813273	protein_coding	145,26	0
ENSG00000126247	CAPNS1	19	36139575	36150353	protein_coding	145,19	0
ENSG00000185504	FAAP100	17	81539885	81553961	protein_coding	145,13	0
ENSG00000197780	TAF13	1	109062486	109076002	protein_coding	144,83	0
ENSG00000184515	BEX5	X	102153708	102156057	protein_coding	144,80	0
ENSG00000197746	PSAP	10	71816298	71851375	protein_coding	144,60	0
ENSG00000140675	SLC5A2	16	31483002	31490860	protein_coding	143,52	0
ENSG00000108590	MED31	17	6643315	6651634	protein_coding	143,24	0
ENSG00000163110	PDLIM5	4	94451857	94668227	protein_coding	143,22	0
ENSG00000005206	SPPL2B	19	2328615	2354806	protein_coding	143,09	0
ENSG00000166997	CNPY4	7	100119613	100125511	protein_coding	143,00	0
ENSG00000112964	GHR	5	42423777	42721878	protein_coding	142,60	0
ENSG00000188730	VWC2	7	49773661	49921950	protein_coding	142,40	0
ENSG00000164099	PRSS12	4	118280038	118353003	protein_coding	142,40	0
ENSG00000078808	SDF4	1	1216908	1232031	protein_coding	142,35	0
ENSG00000153976	HS3ST3A1	17	13495689	13601927	protein_coding	142,30	0
ENSG00000182534	MXRA7	17	76672551	76711016	protein_coding	142,19	0
ENSG00000130005	GAMT	19	1397084	1401570	protein_coding	142,18	0
ENSG00000196235	SUPT5H	19	39436156	39476670	protein_coding	142,16	0
ENSG00000197728	RPS26	12	56041853	56044675	protein_coding	141,75	0
ENSG00000185024	BRF1	14	105209286	105315589	protein_coding	141,70	0
ENSG00000228672	PROB1	5	139390592	139395713	protein_coding	141,70	0
ENSG00000132635	PCED1A	20	2835314	2841190	protein_coding	141,68	0
ENSG00000062716	VMP1	17	59707192	59842255	protein_coding	141,55	0
ENSG00000135002	RFK	9	76385517	76394517	protein_coding	141,50	0
ENSG00000132522	GPS2	17	7311324	7315564	protein_coding	141,49	0
ENSG00000157637	SLC38A10	17	81245000	81295547	protein_coding	141,48	0
ENSG00000168301	KCTD6	3	58492114	58502360	protein_coding	141,43	0
ENSG00000139508	SLC46A3	13	28700064	28718970	protein_coding	141,40	0
ENSG00000198794	SCAMP5	15	74957219	75021496	protein_coding	141,20	0
ENSG00000107147	KCNT1	9	135702185	135795508	protein_coding	141,14	0
ENSG00000166925	TSC22D4	7	100463359	100479279	protein_coding	140,83	0
ENSG00000185246	PRPF39	14	45084099	45116282	protein_coding	140,60	0
ENSG00000102996	MMP15	16	58025566	58046901	protein_coding	140,33	0
ENSG00000072274	TFRC	3	196027183	196082189	protein_coding	140,15	0
ENSG00000143398	PIP5K1A	1	151197949	151249536	protein_coding	140,15	0
ENSG00000129625	REEP5	5	112876379	112922539	protein_coding	140,04	0
ENSG00000114573	ATP6V1A	3	113747019	113812056	protein_coding	139,84	0
ENSG00000168116	KIAA1586	6	57046532	57055239	protein_coding	139,73	0
ENSG00000150977	RILPL2	12	123410683	123436717	protein_coding	139,54	0
ENSG00000148377	IDI2	10	1018907	1025859	protein_coding	139,31	0
ENSG00000157881	PANK4	1	2508533	2526628	protein_coding	139,16	0
ENSG00000139190	VAMP1	12	6462237	6470987	protein_coding	139,10	0
ENSG00000170370	EMX2	10	117542444	117549546	protein_coding	138,93	0
ENSG00000177706	FAM20C	7	192969	260745	protein_coding	138,60	0
ENSG00000181904	C5orf24	5	134845680	134859737	protein_coding	138,53	0
ENSG00000149927	DOC2A	16	30005509	30023270	protein_coding	138,45	0
ENSG00000018280	SLC11A1	2	218382029	218396894	protein_coding	138,40	0
ENSG00000148297	MED22	9	133338323	133348131	protein_coding	138,37	0
ENSG00000131080	EDA2R	X	66595637	66639298	protein_coding	138,00	0

ENSG00000130309	COLGALT1	19	17555594	17583162	protein_coding	138,00	0
ENSG00000042493	CAPG	2	85394748	85418432	protein_coding	137,94	0
ENSG00000169031	COL4A3	2	227164565	227314792	protein_coding	137,80	0
ENSG00000113273	ARSB	5	78777209	78986087	protein_coding	137,62	0
ENSG00000215218	UBE2QL1	5	6448623	6494909	protein_coding	137,50	0
ENSG00000109066	TMEM104	17	74776483	74839779	protein_coding	137,32	0
ENSG00000114993	RTKN	2	74425836	74442422	protein_coding	137,28	0
ENSG00000072135	PTPN18	2	130356007	130375409	protein_coding	137,08	0
ENSG00000162032	SPSB3	16	1776712	1793700	protein_coding	136,96	0
ENSG00000143119	CD53	1	110873154	110899928	protein_coding	136,92	0
ENSG00000168874	ATOH8	2	85751344	85788066	protein_coding	136,80	0
ENSG00000000938	FGR	1	27612064	27635277	protein_coding	136,70	0
ENSG00000132932	ATP8A2	13	25372071	26025851	protein_coding	136,50	0
ENSG00000120832	MTERF2	12	106977291	106987166	protein_coding	136,39	0
ENSG00000189420	ZFP92	X	153418322	153426481	protein_coding	136,30	0
ENSG00000091831	ESR1	6	151656691	152129619	protein_coding	136,00	0
ENSG00000138623	SEMA7A	15	74409289	74434467	protein_coding	135,78	0
ENSG00000197208	SLC22A4	5	132294443	132344206	protein_coding	135,70	0
ENSG00000198598	MMP17	12	131828393	131851783	protein_coding	135,50	0
ENSG00000125730	C3	19	6677704	6730562	protein_coding	135,36	0
ENSG00000163930	BAP1	3	52401013	52410350	protein_coding	135,23	0
ENSG00000130592	LSP1	11	1852970	1892267	protein_coding	135,20	0
ENSG00000135604	STX11	6	144150526	144188370	protein_coding	135,17	0
ENSG00000196141	SPATS2L	2	200305881	200482263	protein_coding	135,04	0
ENSG00000164038	SLC9B2	4	103019868	103085829	protein_coding	134,93	0
ENSG00000113805	CNTN3	3	74262568	74521140	protein_coding	134,90	0
ENSG00000157601	MX1	21	41420304	41459214	protein_coding	134,61	0
ENSG00000060749	QSER1	11	32893178	32993316	protein_coding	134,51	0
ENSG00000131042	LILRB2	19	54238904	54281184	protein_coding	134,50	0
ENSG00000227500	SCAMP4	19	1905214	1926013	protein_coding	134,35	0
ENSG00000173581	CCDC106	19	55641062	55653161	protein_coding	134,19	0
ENSG00000166501	PRKCB	16	23836001	24220611	protein_coding	134,10	0
ENSG00000143631	FLG	1	152302175	152325203	protein_coding	133,91	0
ENSG00000113263	ITK	5	157142933	157255191	protein_coding	133,90	0
ENSG00000166166	TRMT61A	14	103529184	103537073	protein_coding	133,73	0
ENSG00000240053	LY6G5B	6	31670167	31673776	protein_coding	133,60	0
ENSG00000054965	FAM168A	11	73400487	73598189	protein_coding	133,36	0
ENSG00000121413	ZSCAN18	19	58083838	58118427	protein_coding	133,35	0
ENSG00000173327	MAP3K11	11	65597755	65615382	protein_coding	133,33	0
ENSG00000196465	MYL6B	12	56152256	56159647	protein_coding	133,24	0
ENSG00000007384	RHBDF1	16	58059	76355	protein_coding	133,20	0
ENSG00000123131	PRDX4	X	23664262	23686399	protein_coding	133,17	0
ENSG00000081041	CXCL2	4	74097035	74099293	protein_coding	133,10	0
ENSG00000095303	PTGS1	9	122370530	122395703	protein_coding	133,06	0
ENSG00000107562	CXCL12	10	44370165	44386493	protein_coding	133,00	0
ENSG00000153048	CARHSP1	16	8852942	8869012	protein_coding	132,99	0
ENSG00000051128	HOMER3	19	18929201	18941261	protein_coding	132,93	0
ENSG00000103253	HAGHL	16	726936	735525	protein_coding	132,82	0
ENSG00000100644	HIF1A	14	61695513	61748259	protein_coding	132,49	0
ENSG00000149480	MTA2	11	62593214	62601840	protein_coding	132,20	0
ENSG00000116604	MEF2D	1	156463727	156500828	protein_coding	132,13	0
ENSG00000113758	DBN1	5	177456608	177474401	protein_coding	131,98	0
ENSG00000100731	PCNX	14	70907405	71115382	protein_coding	131,82	0
ENSG00000106638	TBL2	7	73568932	73578791	protein_coding	131,66	0
ENSG00000179943	FIZ1	19	55591371	55601970	protein_coding	131,36	0
ENSG00000176871	WSB2	12	118032694	118062430	protein_coding	131,34	0
ENSG00000130829	DUSP9	X	153642492	153651326	protein_coding	131,20	0
ENSG00000163463	KRTCAP2	1	155169408	155173475	protein_coding	131,10	0

ENSG00000168970	JMJD7-PLA2	15	41828095	41848155	protein_coding	131,00	0
ENSG00000146409	SLC18B1	6	132769370	132798553	protein_coding	130,90	0
ENSG00000270647	TAF15	17	35713791	35864615	protein_coding	130,88	0
ENSG00000171045	TSNARE1	8	142212080	142403240	protein_coding	130,80	0
ENSG00000130803	ZNF317	19	9140380	9163424	protein_coding	130,75	0
ENSG00000136444	RSAD1	17	50478800	50485975	protein_coding	130,75	0
ENSG00000125505	MBOAT7	19	54173412	54189882	protein_coding	130,71	0
ENSG00000169660	HEXDC	17	82418318	82442645	protein_coding	130,65	0
ENSG00000057657	PRDM1	6	106086320	106109939	protein_coding	130,58	0
ENSG00000111271	ACAD10	12	111686056	111757107	protein_coding	130,42	0
ENSG00000107897	ACBD5	10	27195214	27242130	protein_coding	130,19	0
ENSG00000147180	ZNF711	X	85244032	85273362	protein_coding	130,17	0
ENSG00000106258	CYP3A5	7	99648194	99679998	protein_coding	130,00	0
ENSG00000182809	CRIP2	14	105472962	105480170	protein_coding	129,90	0
ENSG00000185262	UBALD2	17	76265202	76271299	protein_coding	129,58	0
ENSG00000110046	ATG2A	11	64894546	64917248	protein_coding	129,35	0
ENSG00000157823	AP3S2	15	89830599	89894638	protein_coding	129,26	0
ENSG00000186318	BACE1	11	117285207	117316259	protein_coding	129,24	0
ENSG00000122257	RBBP6	16	24537693	24572863	protein_coding	128,99	0
ENSG00000083838	ZNF446	19	58474017	58481230	protein_coding	128,96	0
ENSG00000123360	PDE1B	12	54549350	54579239	protein_coding	128,90	0
ENSG00000170962	PDGFD	11	103907186	104164379	protein_coding	128,81	0
ENSG00000213928	IRF9	14	24161053	24166565	protein_coding	128,80	0
ENSG00000125755	SYMPK	19	45815410	45863290	protein_coding	128,79	0
ENSG00000157326	DHRS4	14	23953586	23969279	protein_coding	128,58	0
ENSG00000213221	DNLZ	9	136359480	136363789	protein_coding	128,20	0
ENSG00000119777	TMEM214	2	27032910	27041695	protein_coding	128,10	0
ENSG00000146094	DOK3	5	177501907	177511274	protein_coding	127,77	0
ENSG00000130299	GTPBP3	19	17334920	17342735	protein_coding	127,70	0
ENSG00000125744	RTN2	19	45485289	45497061	protein_coding	127,58	0
ENSG00000172977	KAT5	11	65711996	65719604	protein_coding	127,49	0
ENSG00000270170	NCBP2-AS2	3	196942623	196943540	protein_coding	127,40	0
ENSG00000150594	ADRA2A	10	111077163	111080907	protein_coding	127,30	0
ENSG00000160226	C21orf2	21	44328944	44339402	protein_coding	127,27	0
ENSG00000115604	IL18R1	2	102311529	102398775	protein_coding	127,14	0
ENSG00000089127	OAS1	12	112906777	112933222	protein_coding	126,90	0
ENSG00000060718	COL11A1	1	102876467	103108496	protein_coding	126,88	0
ENSG00000167114	SLC27A4	9	128340646	128361470	protein_coding	126,76	0
ENSG00000049540	ELN	7	74027789	74069907	protein_coding	126,58	0
ENSG00000163823	CCR1	3	46201709	46208396	protein_coding	126,55	0
ENSG00000178202	KDELC2	11	108472105	108498432	protein_coding	126,49	0
ENSG00000180071	ANKRD18A	9	38571358	38620660	protein_coding	126,40	0
ENSG00000119737	GPR75	2	53852913	53859989	protein_coding	126,29	0
ENSG00000182108	DEXI	16	10928891	10942460	protein_coding	126,26	0
ENSG00000003400	CASP10	2	201182881	201229406	protein_coding	126,23	0
ENSG00000196998	WDR45	X	49071470	49101170	protein_coding	125,97	0
ENSG00000102879	CORO1A	16	30182827	30189076	protein_coding	125,80	0
ENSG00000115041	KCNIP3	2	95297304	95386083	protein_coding	125,80	0
ENSG00000197818	SLC9A8	20	49812713	49892242	protein_coding	125,50	0
ENSG00000265681	RPL17	18	49488453	49492523	protein_coding	125,50	0
ENSG00000198814	GK	X	30653359	30731456	protein_coding	125,49	0
ENSG00000003096	KLHL13	X	117897813	118117340	protein_coding	125,45	0
ENSG00000198796	ALPK2	18	58481247	58628957	protein_coding	125,20	0
ENSG00000142207	URB1	21	32311018	32393026	protein_coding	124,95	0
ENSG00000074855	ANO8	19	17323223	17334829	protein_coding	124,94	0
ENSG00000169241	SLC50A1	1	155135344	155138857	protein_coding	124,65	0
ENSG00000175130	MARCKSL1	1	32333832	32336379	protein_coding	124,65	0
ENSG00000106565	TMEM176B	7	150791285	150801360	protein_coding	124,36	0

ENSG00000135740	SLC9A5	16	67237683	67272190	protein_coding	124,31	0
ENSG00000166183	ASPG	14	104085679	104115581	protein_coding	124,30	0
ENSG00000091622	PITPNM3	17	6451264	6556494	protein_coding	124,19	0
ENSG00000104946	TBC1D17	19	49877425	49888749	protein_coding	124,18	0
ENSG00000132024	CC2D1A	19	13906201	13930879	protein_coding	124,06	0
ENSG00000160877	NACC1	19	13118103	13141141	protein_coding	124,05	0
ENSG00000132170	PPARG	3	12287368	12434356	protein_coding	124,04	0
ENSG00000100292	HMOX1	22	35380361	35394214	protein_coding	124,03	0
ENSG00000162627	SNX7	1	98661701	98760500	protein_coding	123,98	0
ENSG00000279119	ENSG00000	17	38727833	38728198	protein_coding	123,71	0
ENSG00000128284	APOL3	22	36140330	36166177	protein_coding	123,70	0
ENSG00000166924	NYAP1	7	100483927	100494799	protein_coding	123,70	0
ENSG00000170275	CRTAP	3	33113979	33147773	protein_coding	123,43	0
ENSG00000204954	C12orf73	12	103950202	103965708	protein_coding	123,29	0
ENSG00000115459	ELMOD3	2	85354394	85391752	protein_coding	123,18	0
ENSG00000183783	KCTD8	4	44173909	44448807	protein_coding	123,10	0
ENSG00000131446	MGAT1	5	180784782	180815652	protein_coding	122,81	0
ENSG00000204248	COL11A2	6	33162681	33192499	protein_coding	122,80	0
ENSG00000164776	PHKG1	7	56080283	56092996	protein_coding	122,79	0
ENSG00000115902	SLC1A4	2	64988477	65023865	protein_coding	122,77	0
ENSG00000166927	MS4A7	11	60378482	60395951	protein_coding	122,70	0
ENSG00000150093	ITGB1	10	32900319	33005792	protein_coding	122,66	0
ENSG00000167972	ABCA3	16	2275881	2340746	protein_coding	122,59	0
ENSG00000130313	PGLS	19	17511629	17521288	protein_coding	122,56	0
ENSG00000136383	ALPK3	15	84816680	84873482	protein_coding	122,37	0
ENSG00000139597	N4BP2L1	13	32400723	32428311	protein_coding	122,30	0
ENSG00000197530	MIB2	1	1615415	1630610	protein_coding	122,28	0
ENSG00000115738	ID2	2	8678845	8684453	protein_coding	122,26	0
ENSG00000167378	IRGQ	19	43584369	43596135	protein_coding	122,24	0
ENSG00000120217	CD274	9	5450503	5470566	protein_coding	122,10	0
ENSG00000169105	CHST14	15	40470998	40474571	protein_coding	121,86	0
ENSG00000196639	HRH1	3	11137093	11263557	protein_coding	121,83	0
ENSG00000151692	RNF144A	2	6917392	7068286	protein_coding	121,82	0
ENSG00000161847	RAVER1	19	10316212	10333640	protein_coding	121,77	0
ENSG00000145029	NICN1	3	49422946	49429326	protein_coding	121,63	0
ENSG00000160323	ADAMTS13	9	133414358	133459402	protein_coding	121,60	0
ENSG00000166987	MBD6	12	57520710	57530148	protein_coding	121,59	0
ENSG00000006015	C19orf60	19	18588685	18592336	protein_coding	121,52	0
ENSG00000136160	EDNRB	13	77895481	77919768	protein_coding	121,50	0
ENSG00000166311	SMPD1	11	6390431	6394998	protein_coding	121,49	0
ENSG00000052802	MSMO1	4	165327623	165343160	protein_coding	121,35	0
ENSG00000243244	STON1	2	48529383	48598513	protein_coding	120,79	0
ENSG00000134970	TMED7	5	115613508	115632992	protein_coding	120,61	0
ENSG00000101986	ABCD1	X	153724868	153744762	protein_coding	120,60	0
ENSG00000137404	NRM	6	30688047	30691420	protein_coding	120,54	0
ENSG00000119630	PGF	14	74941834	74955784	protein_coding	120,47	0
ENSG00000157557	ETS2	21	38805307	38824955	protein_coding	120,25	0
ENSG00000162300	ZFPL1	11	65084223	65088400	protein_coding	120,04	0
ENSG00000158019	BRE	2	27889941	28338901	protein_coding	120,00	0
ENSG00000169902	TPST1	7	66205199	66420543	protein_coding	119,76	0
ENSG00000164129	NPY5R	4	163343939	163351934	protein_coding	119,70	0
ENSG00000107874	CUEDC2	10	102423245	102432661	protein_coding	119,46	0
ENSG00000215251	FASTKD5	20	3146519	3159897	protein_coding	119,39	0
ENSG00000167874	TMEM88	17	7855065	7856099	protein_coding	119,33	0
ENSG00000101421	CHMP4B	20	33811304	33854366	protein_coding	119,05	0
ENSG00000245848	CEBPA	19	33299934	33302564	protein_coding	119,00	0
ENSG00000159640	ACE	17	63477061	63498380	protein_coding	118,90	0
ENSG00000107537	PHYH	10	13277796	13302412	protein_coding	118,60	0

ENSG00000055813	CCDC85A	2	56184123	56386173	protein_coding	118,54	0
ENSG00000102743	SLC25A15	13	40789412	40810111	protein_coding	118,52	0
ENSG00000109680	TBC1D19	4	26576437	26755351	protein_coding	118,44	0
ENSG00000165171	WBSCR27	7	73834590	73842535	protein_coding	118,40	0
ENSG00000198837	DENND4B	1	153929501	153946696	protein_coding	118,32	0
ENSG00000139344	AMDHD1	12	95943293	95968716	protein_coding	118,08	0
ENSG00000095015	MAP3K1	5	56815574	56896152	protein_coding	118,08	0
ENSG00000099910	KLHL22	22	20441519	20495883	protein_coding	118,07	0
ENSG00000062370	ZNF112	19	44326555	44367217	protein_coding	118,00	0
ENSG00000136068	FLNB	3	58008400	58172251	protein_coding	117,93	0
ENSG00000153936	HS2ST1	1	86914648	87109998	protein_coding	117,71	0
ENSG00000275342	ENSG00000	8	8317736	8386498	protein_coding	117,60	0
ENSG00000160789	LMNA	1	156082573	156140089	protein_coding	117,52	0
ENSG00000183260	ABHD16B	20	63861498	63862988	protein_coding	117,50	0
ENSG00000269335	IKBKG	X	154541199	154565046	protein_coding	117,19	0
ENSG00000178026	LRRC75B	22	24585620	24593208	protein_coding	117,00	0
ENSG00000100802	C14orf93	14	22985908	23010166	protein_coding	116,82	0
ENSG00000162407	PLPP3	1	56494747	56645301	protein_coding	116,79	0
ENSG00000161395	PGAP3	17	39671122	39696797	protein_coding	116,61	0
ENSG00000135108	FBXO21	12	117141988	117190531	protein_coding	116,50	0
ENSG00000145536	ADAMTS16	5	5140330	5320304	protein_coding	116,40	0
ENSG00000117266	CDK18	1	205504595	205532793	protein_coding	116,16	0
ENSG00000137040	RANBP6	9	6011043	6015625	protein_coding	116,13	0
ENSG00000120318	ARAP3	5	141653401	141682221	protein_coding	116,00	0
ENSG00000143162	CREG1	1	167529677	167553767	protein_coding	115,74	0
ENSG00000138080	EMILIN1	2	27078567	27086408	protein_coding	115,64	0
ENSG00000149798	CDC42EP2	11	65314818	65322429	protein_coding	115,23	0
ENSG00000116001	TIA1	2	70209444	70248660	protein_coding	115,16	0
ENSG00000178761	FAM219B	15	74899987	74907121	protein_coding	115,16	0
ENSG00000163909	HEYL	1	39624153	39639945	protein_coding	115,08	0
ENSG00000183077	AFMID	17	78187317	78207701	protein_coding	114,85	0
ENSG00000100055	CYTH4	22	37282027	37315345	protein_coding	114,80	0
ENSG00000186998	EMID1	22	29205851	29259597	protein_coding	114,79	0
ENSG00000135678	CPM	12	68842197	68971570	protein_coding	114,76	0
ENSG00000169403	PTAFR	1	28147166	28193936	protein_coding	114,70	0
ENSG00000160973	FOXH1	8	144473412	144476335	protein_coding	114,68	0
ENSG00000090530	P3H2	3	189956728	190122437	protein_coding	114,68	0
ENSG00000161671	EMC10	19	50476400	50490870	protein_coding	114,68	0
ENSG00000132329	RAMP1	2	237858893	237912114	protein_coding	114,53	0
ENSG00000157800	SLC37A3	7	140293693	140404433	protein_coding	114,48	0
ENSG00000087085	ACHE	7	100889994	100896974	protein_coding	114,29	0
ENSG00000100379	KCTD17	22	37051736	37063390	protein_coding	114,29	0
ENSG00000174282	ZBTB4	17	7459366	7484263	protein_coding	114,21	0
ENSG00000198315	ZKSCAN8	6	28141910	28159472	protein_coding	114,19	0
ENSG00000108797	CNTNAP1	17	42682613	42699814	protein_coding	114,10	0
ENSG00000159788	RGS12	4	3293028	3439913	protein_coding	113,80	0
ENSG00000173930	SLCO4C1	5	102233986	102296549	protein_coding	113,80	0
ENSG00000168040	FADD	11	70203163	70207390	protein_coding	113,66	0
ENSG00000137700	SLC37A4	11	119024114	119030906	protein_coding	113,58	0
ENSG00000185522	LMNTD2	11	554855	560779	protein_coding	113,42	0
ENSG00000171033	PKIA	8	78516139	78605267	protein_coding	113,36	0
ENSG00000235961	PNMA6A	X	153072482	153075018	protein_coding	113,20	0
ENSG00000172086	KRCC1	2	88027205	88055729	protein_coding	113,09	0
ENSG00000134955	SLC37A2	11	125063067	125090312	protein_coding	112,90	0
ENSG00000188747	NOXA1	9	137423350	137434406	protein_coding	112,77	0
ENSG00000125458	NT5C	17	75130225	75131795	protein_coding	112,76	0
ENSG00000179583	CIITA	16	10877198	10932281	protein_coding	112,65	0
ENSG00000144792	ZNF660	3	44578223	44599694	protein_coding	112,58	0

ENSG00000167536	DHRS13	17	28897781	28903071	protein_coding	112,50	0
ENSG00000075223	SEMA3C	7	80742538	80922359	protein_coding	112,36	0
ENSG00000149761	NUDT22	11	64225941	64230686	protein_coding	112,34	0
ENSG00000175970	UNC119B	12	120710435	120723640	protein_coding	112,23	0
ENSG00000067992	PDK3	X	24465221	24539837	protein_coding	112,21	0
ENSG00000165259	HDX	X	84317874	84502479	protein_coding	112,20	0
ENSG00000133138	TBC1D8B	X	106802680	106876145	protein_coding	112,19	0
ENSG00000164488	DACT2	6	168292830	168319754	protein_coding	112,10	0
ENSG00000219607	PPP1R3G	6	5084581	5089487	protein_coding	112,10	0
ENSG00000205213	LGR4	11	27365961	27472775	protein_coding	111,80	0
ENSG00000071282	LMCD1	3	8501707	8574673	protein_coding	111,78	0
ENSG00000143401	ANP32E	1	150218417	150236156	protein_coding	111,70	0
ENSG00000099785	02-mars	19	8413270	8439017	protein_coding	111,66	0
ENSG00000124731	TREM1	6	41267926	41286719	protein_coding	111,50	0
ENSG00000187608	ISG15	1	1001138	1014541	protein_coding	111,36	0
ENSG00000106266	SNX8	7	2251770	2354318	protein_coding	111,33	0
ENSG00000154065	ANKRD29	18	23598926	23662885	protein_coding	111,27	0
ENSG00000187066	TMEM262	11	65084979	65089375	protein_coding	111,20	0
ENSG00000106261	ZKSCAN1	7	100015572	100041689	protein_coding	111,15	0
ENSG00000160305	DIP2A	21	46458899	46569852	protein_coding	111,10	0
ENSG00000185634	SHC4	15	48823735	48963444	protein_coding	111,03	0
ENSG00000165655	ZNF503	10	75397830	75401906	protein_coding	110,64	0
ENSG00000102096	PIM2	X	48913182	48919024	protein_coding	110,45	0
ENSG00000104866	PPP1R37	19	45091396	45148077	protein_coding	110,03	0
ENSG00000149179	C11orf49	11	46936689	47164385	protein_coding	110,00	0
ENSG00000124588	NQO2	6	2987987	3028869	protein_coding	109,93	0
ENSG00000181619	GPR135	14	59429022	59465342	protein_coding	109,88	0
ENSG00000125753	VASP	19	45506579	45526983	protein_coding	109,80	0
ENSG00000159314	ARHGAP27	17	45393902	45434421	protein_coding	109,80	0
ENSG00000204469	PRRC2A	6	31620720	31637771	protein_coding	109,79	0
ENSG00000204316	MRPL38	17	75898643	75905413	protein_coding	109,60	0
ENSG00000164574	GALNT10	5	154190730	154420984	protein_coding	109,58	0
ENSG00000168615	ADAM9	8	38996869	39105144	protein_coding	109,57	0
ENSG00000157625	TAB3	X	30827442	30975084	protein_coding	109,56	0
ENSG00000063180	CA11	19	48637942	48646312	protein_coding	109,30	0
ENSG00000023892	DEF6	6	35297852	35321771	protein_coding	109,20	0
ENSG00000117411	B4GALT2	1	43978943	43991170	protein_coding	108,98	0
ENSG00000174080	CTSF	11	66563463	66568841	protein_coding	108,96	0
ENSG00000213523	SRA1	5	140537340	140558310	protein_coding	108,87	0
ENSG00000092841	MYL6	12	56158161	56163496	protein_coding	108,78	0
ENSG00000112320	SOBP	6	107489958	107660167	protein_coding	108,61	0
ENSG00000117394	SLC2A1	1	42925375	42959173	protein_coding	108,47	0
ENSG00000234224	TMEM229A	7	124030916	124033023	protein_coding	108,40	0
ENSG00000205356	TECPR1	7	98214624	98252251	protein_coding	108,14	0
ENSG00000164512	ANKRD55	5	56099678	56233359	protein_coding	108,10	0
ENSG00000164124	TMEM144	4	158201604	158255411	protein_coding	108,00	0
ENSG00000172476	RAB40A	X	103499750	103519489	protein_coding	108,00	0
ENSG00000179820	MYADM	19	53866223	53876437	protein_coding	107,79	0
ENSG00000104835	SARS2	19	38915266	38930896	protein_coding	107,70	0
ENSG00000197114	ZGPAT	20	63707465	63736142	protein_coding	107,28	0
ENSG00000105967	TFEC	7	115935148	116159896	protein_coding	107,10	0
ENSG00000165029	ABCA1	9	104781002	104928237	protein_coding	106,89	0
ENSG00000088756	ARHGAP28	18	6729718	6915716	protein_coding	106,50	0
ENSG00000184937	WT1	11	32387775	32435630	protein_coding	106,30	0
ENSG00000080819	CPOX	3	98521132	98593723	protein_coding	106,24	0
ENSG00000146063	TRIM41	5	181222499	181235809	protein_coding	106,11	0
ENSG00000183762	KREMEN1	22	29073078	29168333	protein_coding	105,95	0
ENSG00000174669	SLC29A2	11	66362521	66372214	protein_coding	105,95	0

ENSG00000162066	AMDHD2	16	2520357	2531422	protein_coding	105,94	0
ENSG00000130158	DOCK6	19	11199295	11262481	protein_coding	105,78	0
ENSG00000151917	BEND6	6	56955126	57027342	protein_coding	105,59	0
ENSG00000187266	EPOR	19	11377205	11384342	protein_coding	105,54	0
ENSG00000056998	GYG2	X	2828788	2882818	protein_coding	105,51	0
ENSG00000120327	PCDHB14	5	141222932	141227759	protein_coding	105,50	0
ENSG00000066294	CD84	1	160541095	160579516	protein_coding	105,40	0
ENSG00000166165	CKB	14	103519659	103523111	protein_coding	105,30	0
ENSG00000185274	WBSCR17	7	71132169	71713600	protein_coding	105,10	0
ENSG00000135318	NT5E	6	85449584	85495791	protein_coding	105,00	0
ENSG00000165895	ARHGAP42	11	100687653	100991937	protein_coding	104,92	0
ENSG00000197779	ZNF81	X	47836902	48002561	protein_coding	104,92	0
ENSG00000136859	ANGPTL2	9	127087332	127122883	protein_coding	104,90	0
ENSG00000137760	ALKBH8	11	107502726	107565746	protein_coding	104,86	0
ENSG00000198185	ZNF334	20	46499630	46513559	protein_coding	104,78	0
ENSG00000165025	SYK	9	90801787	90898549	protein_coding	104,38	0
ENSG00000169926	KLF13	15	31326855	31435665	protein_coding	104,20	0
ENSG00000141506	PIK3R5	17	8878911	8965712	protein_coding	104,18	0
ENSG00000083844	ZNF264	19	57191500	57222846	protein_coding	104,03	0
ENSG00000253250	C8orf88	8	90958637	90985257	protein_coding	103,90	0
ENSG00000262246	CORO7	16	4354542	4425705	protein_coding	103,73	0
ENSG00000065268	WDR18	19	984271	998438	protein_coding	103,55	0
ENSG00000204356	NELFE	6	31952087	31959110	protein_coding	103,36	0
ENSG00000167524	ENSG0000001	17	28607964	28614200	protein_coding	103,18	0
ENSG00000112559	MDFI	6	41636882	41654246	protein_coding	102,90	0
ENSG00000171408	PDE7B	6	135851696	136195574	protein_coding	102,89	0
ENSG00000105968	H2AFV	7	44826791	44848083	protein_coding	102,85	0
ENSG00000254122	PCDHGB7	5	141417645	141512979	protein_coding	102,80	0
ENSG00000141744	PNMT	17	39667981	39670475	protein_coding	102,77	0
ENSG00000108691	CCL2	17	34255218	34257203	protein_coding	102,60	0
ENSG00000174327	SLC16A13	17	7036075	7040121	protein_coding	102,55	0
ENSG00000179526	SHARPIN	8	144098633	144108124	protein_coding	102,32	0
ENSG00000143546	S100A8	1	153390032	153391188	protein_coding	102,10	0
ENSG00000131015	ULBP2	6	149942000	149949235	protein_coding	101,90	0
ENSG00000167302	ENTHD2	17	81228277	81239091	protein_coding	101,88	0
ENSG00000069424	KCNAB2	1	5991466	6101193	protein_coding	101,84	0
ENSG00000025770	NCAPH2	22	50508216	50523472	protein_coding	101,55	0
ENSG00000180011	ZADH2	18	75195108	75209348	protein_coding	101,51	0
ENSG00000011021	CLCN6	1	11806096	11843144	protein_coding	101,51	0
ENSG00000126368	NR1D1	17	40092787	40100725	protein_coding	101,16	0
ENSG00000213689	TREX1	3	48465811	48467645	protein_coding	101,10	0
ENSG00000164161	HHIP	4	144646021	144745271	protein_coding	101,00	0
ENSG00000156675	RAB11FIP1	8	37858618	37899467	protein_coding	100,88	0
ENSG00000227057	WDR46	6	33279108	33289527	protein_coding	100,88	0
ENSG00000197696	NMB	15	84655129	84658563	protein_coding	100,64	0
ENSG00000116406	EDEM3	1	184690231	184754913	protein_coding	100,26	0
ENSG00000267534	S1PR2	19	10221435	10231272	protein_coding	100,17	0
ENSG00000141543	EIF4A3	17	80135214	80147183	protein_coding	100,11	0
ENSG00000175573	C11orf68	11	65916808	65919117	protein_coding	100,07	0
ENSG00000242028	HYPK	15	43796142	43803043	protein_coding	100,00	0
ENSG00000135829	DHX9	1	182839369	182887751	protein_coding	99,75	0
ENSG00000076604	TRAF4	17	28743984	28750958	protein_coding	99,74	0
ENSG00000101935	AMMECR1	X	110194186	110440233	protein_coding	99,58	0
ENSG00000089692	LAG3	12	6772512	6778455	protein_coding	99,50	0
ENSG00000181873	IBA57	1	228165815	228182257	protein_coding	99,43	0
ENSG00000256574	OR13A1	10	45302654	45315608	protein_coding	99,20	0
ENSG00000176853	FAM91A1	8	123768456	123815452	protein_coding	99,16	0
ENSG00000173546	CSPG4	15	75674322	75712848	protein_coding	99,00	0

ENSG00000213903	LTB4R	14	24311450	24318036	protein_coding	98,91	0
ENSG00000145217	SLC26A1	4	979073	993440	protein_coding	98,91	0
ENSG00000168268	NT5DC2	3	52524385	52535054	protein_coding	98,89	0
ENSG00000154269	ENPP3	6	131628442	131747418	protein_coding	98,75	0
ENSG00000188153	COL4A5	X	108439844	108697545	protein_coding	98,70	0
ENSG00000160293	VAV2	9	133761894	133992604	protein_coding	98,64	0
ENSG00000159784	FAM131B	7	143353400	143362770	protein_coding	98,60	0
ENSG00000157600	TMEM164	X	110002631	110182734	protein_coding	98,47	0
ENSG00000147853	AK3	9	4709559	4742043	protein_coding	98,46	0
ENSG00000131899	LLGL1	17	18225587	18244875	protein_coding	98,44	0
ENSG00000205517	RGL3	19	11384341	11419342	protein_coding	98,43	0
ENSG00000085514	PILRA	7	100367530	100400099	protein_coding	98,40	0
ENSG00000128311	TST	22	37010859	37020183	protein_coding	98,28	0
ENSG00000205220	PSMB10	16	67934502	67937087	protein_coding	98,07	0
ENSG00000180035	ZNF48	16	30378106	30400108	protein_coding	97,97	0
ENSG00000079335	CDC14A	1	100345025	100520277	protein_coding	97,55	0
ENSG00000188419	CHM	X	85861180	86047562	protein_coding	97,41	0
ENSG00000157399	ARSE	X	2934632	2968310	protein_coding	97,40	0
ENSG00000100226	GTPBP1	22	38705723	38738299	protein_coding	97,32	0
ENSG00000132639	SNAP25	20	10218830	10307418	protein_coding	97,30	0
ENSG00000204219	TCEA3	1	23381061	23424740	protein_coding	97,27	0
ENSG00000171860	C3AR1	12	8058302	8066471	protein_coding	97,20	0
ENSG00000185338	SOCS1	16	11254405	11256179	protein_coding	97,12	0
ENSG00000184840	TMED9	5	177592158	177596124	protein_coding	97,06	0
ENSG00000131116	ZNF428	19	43607219	43619874	protein_coding	97,03	0
ENSG00000167074	TEF	22	41367333	41399326	protein_coding	96,94	0
ENSG00000172985	SH3RF3	2	109129348	109504632	protein_coding	96,90	0
ENSG00000196275	GTF2IRD2	7	74796144	74851551	protein_coding	96,82	0
ENSG00000212123	PRR22	19	5782960	5784765	protein_coding	96,70	0
ENSG00000106665	CLIP2	7	74289475	74405943	protein_coding	96,70	0
ENSG00000100219	XBP1	22	28794555	28800597	protein_coding	96,58	0
ENSG00000154640	BTG3	21	17593653	17612947	protein_coding	96,45	0
ENSG00000179403	VWA1	1	1434861	1442882	protein_coding	96,40	0
ENSG00000159840	ZYX	7	143381080	143391111	protein_coding	96,31	0
ENSG00000186407	CD300E	17	74609887	74623738	protein_coding	96,30	0
ENSG00000246705	H2AFJ	12	14774383	14778002	protein_coding	96,29	0
ENSG00000269858	EGLN2	19	40798996	40808433	protein_coding	96,27	0
ENSG00000162783	IER5	1	181088712	181092899	protein_coding	96,25	0
ENSG00000121316	PLBD1	12	14503661	14568349	protein_coding	96,07	0
ENSG00000148843	PDCD11	10	103396648	103446292	protein_coding	96,06	0
ENSG00000273604	C17orf96	17	38671703	38675421	protein_coding	96,04	0
ENSG00000106348	IMPDH1	7	128392277	128410252	protein_coding	95,91	0
ENSG00000221843	C2orf16	2	27576522	27582720	protein_coding	95,91	0
ENSG00000114650	SCAP	3	47413694	47477126	protein_coding	95,88	0
ENSG00000154511	FAM69A	1	92832737	92961522	protein_coding	95,88	0
ENSG00000135596	MICAL1	6	109444062	109465968	protein_coding	95,77	0
ENSG00000103569	AQP9	15	58138169	58185911	protein_coding	95,75	0
ENSG00000115956	PLEK	2	68365173	68397453	protein_coding	95,75	0
ENSG00000114988	LMAN2L	2	96705929	96740064	protein_coding	95,69	0
ENSG00000123104	ITPR2	12	26336515	26833198	protein_coding	95,68	0
ENSG00000137098	SPAG8	9	35808045	35812272	protein_coding	95,60	0
ENSG00000143416	SELENBP1	1	151364302	151372733	protein_coding	95,50	0
ENSG00000126456	IRF3	19	49659569	49665875	protein_coding	95,20	0
ENSG00000179813	FAM216B	13	42781550	42791549	protein_coding	95,20	0
ENSG00000175040	CHST2	3	143119331	143124014	protein_coding	95,10	0
ENSG00000162341	TPCN2	11	69048897	69090604	protein_coding	95,09	0
ENSG00000175505	CLCF1	11	67364168	67374177	protein_coding	95,05	0
ENSG00000198189	HSD17B11	4	87336610	87391386	protein_coding	94,98	0

ENSG00000154358	OBSCN	1	228208130	228378876	protein_coding	94,88	0
ENSG00000224051	CPTP	1	1324756	1328897	protein_coding	94,76	0
ENSG00000103966	EHD4	15	41895939	41972578	protein_coding	94,72	0
ENSG00000185862	EVI2B	17	31303766	31314112	protein_coding	94,70	0
ENSG00000170145	SIK2	11	111602391	111730853	protein_coding	94,66	0
ENSG00000203896	LIME1	20	63736283	63739103	protein_coding	94,30	0
ENSG00000162426	SLC45A1	1	8317826	8344167	protein_coding	94,09	0
ENSG00000196371	FUT4	11	94543840	94549898	protein_coding	94,02	0
ENSG00000117385	P3H1	1	42746335	42767084	protein_coding	93,91	0
ENSG00000173210	ABLIM3	5	149141483	149260542	protein_coding	93,90	0
ENSG00000135144	DTX1	12	113056709	113098028	protein_coding	93,90	0
ENSG00000165966	PDZRN4	12	41188448	41574590	protein_coding	93,90	0
ENSG00000124608	AARS2	6	44299654	44313326	protein_coding	93,90	0
ENSG00000213676	ATF6B	6	32098176	32128253	protein_coding	93,84	0
ENSG00000160593	AMICA1	11	118193740	118225094	protein_coding	93,80	0
ENSG00000104852	SNRNP70	19	49085419	49108605	protein_coding	93,66	0
ENSG00000155265	GOLGA7B	10	97850239	97871580	protein_coding	93,62	0
ENSG00000182919	C11orf54	11	93741591	93764749	protein_coding	93,60	0
ENSG00000104938	CLEC4M	19	7763149	7769605	protein_coding	93,50	0
ENSG00000125967	NECAB3	20	33657087	33674463	protein_coding	93,48	0
ENSG00000095587	TLL2	10	96364606	96513918	protein_coding	93,40	0
ENSG00000135587	SMPD2	6	109440763	109443919	protein_coding	93,33	0
ENSG00000106780	MEGF9	9	120600813	120714470	protein_coding	93,33	0
ENSG00000168026	TTC21A	3	39107704	39138903	protein_coding	93,20	0
ENSG00000168306	ACOX2	3	58505136	58537319	protein_coding	93,10	0
ENSG00000079257	LXN	3	158645822	158672693	protein_coding	93,03	0
ENSG00000161544	CYGB	17	76527356	76551175	protein_coding	93,00	0
ENSG00000213782	DDX47	12	12813316	12829981	protein_coding	92,90	0
ENSG00000185238	PRMT3	11	20387530	20509294	protein_coding	92,76	0
ENSG00000137275	RIPK1	6	3063991	3115187	protein_coding	92,70	0
ENSG00000164307	ERAP1	5	96760810	96808100	protein_coding	92,62	0
ENSG00000149451	ADAM33	20	3667965	3682246	protein_coding	92,60	0
ENSG00000158122	AAED1	9	96639577	96655303	protein_coding	92,52	0
ENSG00000134762	DSC3	18	30990008	31042815	protein_coding	92,50	0
ENSG00000246922	UBAP1L	15	65092770	65115197	protein_coding	92,43	0
ENSG00000145198	VWA5B2	3	184230429	184242329	protein_coding	92,40	0
ENSG00000148386	LCN9	9	135663322	135666422	protein_coding	92,29	0
ENSG00000169372	CRADD	12	93677375	93894840	protein_coding	92,26	0
ENSG00000185359	HGS	17	81683326	81703138	protein_coding	92,25	0
ENSG00000143067	ZNF697	1	119619422	119647773	protein_coding	92,24	0
ENSG00000198026	ZNF335	20	45948653	45972172	protein_coding	92,21	0
ENSG00000196547	MAN2A2	15	90902218	90922584	protein_coding	92,03	0
ENSG00000244165	P2RY11	19	10111538	10115372	protein_coding	91,93	0
ENSG00000159216	RUNX1	21	34787801	36004667	protein_coding	91,83	0
ENSG00000117139	KDM5B	1	202724491	202809470	protein_coding	91,81	0
ENSG00000171051	FPR1	19	51745172	51804110	protein_coding	91,80	0
ENSG00000039650	PNKP	19	49861204	49878351	protein_coding	91,80	0
ENSG00000162688	AGL	1	99850084	99924023	protein_coding	91,75	0
ENSG00000160055	TMEM234	1	32214472	32222359	protein_coding	91,74	0
ENSG00000138744	NAAA	4	75913657	75941051	protein_coding	91,70	0
ENSG00000241852	C8orf58	8	22599601	22604150	protein_coding	91,57	0
ENSG00000100867	DHRS2	14	23630115	23645639	protein_coding	91,50	0
ENSG00000171298	GAA	17	80101556	80119879	protein_coding	91,47	0
ENSG00000126705	AHDC1	1	27534035	27604431	protein_coding	91,45	0
ENSG00000099365	STX1B	16	30989256	31010628	protein_coding	91,30	0
ENSG00000196189	SEMA4A	1	156147366	156177752	protein_coding	91,20	0
ENSG00000110921	MVK	12	109573255	109598117	protein_coding	91,01	0
ENSG00000135722	FBXL8	16	67159931	67164570	protein_coding	91,00	0

ENSG00000166794	PPIB	15	64155812	64163205	protein_coding	90,89	0
ENSG00000204227	RING1	6	33208495	33212722	protein_coding	90,85	0
ENSG00000102802	MEDAG	13	30906191	30925572	protein_coding	90,70	0
ENSG00000132155	RAF1	3	12583601	12664226	protein_coding	90,54	0
ENSG00000198075	SULT1C4	2	108377911	108388057	protein_coding	90,30	0
ENSG00000122971	ACADS	12	120725735	120740008	protein_coding	90,26	0
ENSG00000188916	FAM196A	10	127135426	127196158	protein_coding	90,24	0
ENSG00000100221	JOSD1	22	38685543	38701556	protein_coding	90,23	0
ENSG00000135447	PPP1R1A	12	54575387	54588659	protein_coding	90,20	0
ENSG00000184378	ACTRT3	3	169766921	169769895	protein_coding	90,20	0
ENSG00000101336	HCK	20	32052188	32101856	protein_coding	90,18	0
ENSG00000121064	SCPEP1	17	56978105	57006768	protein_coding	90,14	0
ENSG00000189007	ADAT2	6	143422832	143450673	protein_coding	90,08	0
ENSG00000010361	FUZ	19	49806869	49817376	protein_coding	90,04	0
ENSG00000066336	SPI1	11	47354860	47378576	protein_coding	90,00	0
ENSG00000112234	FBXL4	6	98868538	98948006	protein_coding	89,91	0
ENSG00000170525	PFKFB3	10	6144934	6235545	protein_coding	89,91	0
ENSG00000140057	AK7	14	96392111	96489427	protein_coding	89,90	0
ENSG00000223501	VPS52	6	33250272	33272047	protein_coding	89,78	0
ENSG00000174343	CHRNA9	4	40335329	40355217	protein_coding	89,70	0
ENSG00000162630	B3GALT2	1	193179045	193186654	protein_coding	89,70	0
ENSG00000225663	FAM195B	17	81822361	81833302	protein_coding	89,57	0
ENSG00000166073	GPR176	15	39799023	39920892	protein_coding	89,52	0
ENSG00000204152	TIMM23B	10	49942033	49974850	protein_coding	89,40	0
ENSG00000204304	PBX2	6	32184741	32190186	protein_coding	89,35	0
ENSG00000105223	PLD3	19	40348456	40380439	protein_coding	89,32	0
ENSG00000162512	SDC3	1	30869467	30908761	protein_coding	89,28	0
ENSG00000143570	SLC39A1	1	153959099	153968184	protein_coding	89,14	0
ENSG00000100985	MMP9	20	46008908	46016561	protein_coding	89,10	0
ENSG00000154222	CC2D1B	1	52345723	52366193	protein_coding	89,05	0
ENSG00000153406	NMRAL1	16	4461680	4495763	protein_coding	89,03	0
ENSG00000166979	EVA1C	21	32412006	32515397	protein_coding	89,00	0
ENSG00000196730	DAPK1	9	87497228	87708633	protein_coding	88,97	0
ENSG00000115419	GLS	2	190880827	190965552	protein_coding	88,93	0
ENSG00000124541	RRP36	6	43021645	43034156	protein_coding	88,91	0
ENSG00000175274	TP53I11	11	44885903	44951306	protein_coding	88,91	0
ENSG00000001036	FUCA2	6	143494811	143511690	protein_coding	88,88	0
ENSG00000151929	BAG3	10	119651370	119677819	protein_coding	88,85	0
ENSG00000182685	BRICD5	16	2209253	2211950	protein_coding	88,81	0
ENSG00000149150	SLC43A1	11	57484534	57515786	protein_coding	88,80	0
ENSG00000104695	PPP2CB	8	30774457	30814314	protein_coding	88,77	0
ENSG00000182979	MTA1	14	105419820	105470729	protein_coding	88,67	0
ENSG00000167671	UBXN6	19	4444999	4457822	protein_coding	88,64	0
ENSG00000119431	HDHD3	9	113373419	113376999	protein_coding	88,63	0
ENSG00000148671	ADIRF	10	86968192	86983934	protein_coding	88,60	0
ENSG00000105519	CAPS	19	5911707	5916208	protein_coding	88,57	0
ENSG00000169508	GPR183	13	99294530	99307405	protein_coding	88,55	0
ENSG00000068028	RASSF1	3	50329782	50340980	protein_coding	88,43	0
ENSG00000198353	HOXC4	12	54016931	54056030	protein_coding	88,40	0
ENSG00000114439	BBX	3	107522936	107811324	protein_coding	88,37	0
ENSG00000095906	NUBP2	16	1782901	1789191	protein_coding	88,36	0
ENSG00000019485	PRDM11	11	45095806	45235124	protein_coding	88,33	0
ENSG00000131711	MAP1B	5	72107234	72209570	protein_coding	88,28	0
ENSG00000111052	LIN7A	12	80792520	80937925	protein_coding	88,26	0
ENSG00000101365	IDH3B	20	2658395	2664219	protein_coding	88,22	0
ENSG00000173918	C1QTNF1	17	79022814	79049788	protein_coding	88,12	0
ENSG00000180730	SHISA2	13	26044597	26051031	protein_coding	88,10	0
ENSG00000159228	CBR1	21	36069941	36073166	protein_coding	88,07	0

ENSG00000177885	GRB2	17	75318076	75405709	protein_coding	87,95	0
ENSG00000169314	C22orf15	22	23763021	23765861	protein_coding	87,90	0
ENSG00000171291	ZNF439	19	11848726	11883750	protein_coding	87,79	0
ENSG00000205923	CEMP1	16	2530035	2531417	protein_coding	87,78	0
ENSG00000146216	TTBK1	6	43243680	43288259	protein_coding	87,70	0
ENSG00000136689	IL1RN	2	113107214	113134016	protein_coding	87,70	0
ENSG00000104880	ARHGEF18	19	7395113	7472477	protein_coding	87,60	0
ENSG00000104689	TNFRSF10A	8	23190452	23225126	protein_coding	87,50	0
ENSG00000132881	RSG1	1	16231700	16237162	protein_coding	87,50	0
ENSG00000174996	KLC2	11	66257294	66267860	protein_coding	87,49	0
ENSG00000148288	GBGT1	9	133152948	133163945	protein_coding	87,30	0
ENSG00000075826	SEC31B	10	100486642	100519864	protein_coding	87,29	0
ENSG00000105880	DLX5	7	97020392	97025097	protein_coding	87,28	0
ENSG00000149489	ROM1	11	62611722	62615120	protein_coding	87,17	0
ENSG00000177051	FBXO46	19	45710629	45730904	protein_coding	87,05	0
ENSG00000147231	CXorf57	X	106611930	106679442	protein_coding	86,82	0
ENSG00000133028	SCO1	17	10672474	10698375	protein_coding	86,82	0
ENSG00000173991	TCAP	17	39664187	39666555	protein_coding	86,80	0
ENSG00000164062	APEH	3	49674002	49683963	protein_coding	86,47	0
ENSG00000116032	GRIN3B	19	1000419	1009732	protein_coding	86,33	0
ENSG00000167613	LAIR1	19	54351384	54370558	protein_coding	86,30	0
ENSG00000129474	AJUBA	14	22971174	22982642	protein_coding	86,30	0
ENSG00000163501	IHH	2	219054420	219060467	protein_coding	86,30	0
ENSG00000169258	GPRIN1	5	176595802	176610133	protein_coding	86,20	0
ENSG00000189221	MAOA	X	43654907	43746824	protein_coding	86,04	0
ENSG00000064547	LPAR2	19	19623668	19628930	protein_coding	86,00	0
ENSG00000179922	ZNF784	19	55620742	55624601	protein_coding	85,84	0
ENSG00000204682	CASC10	10	21492658	21497262	protein_coding	85,80	0
ENSG00000272916	ENSG00000	10	73796514	73811651	protein_coding	85,77	0
ENSG00000133019	CHRM3	1	239386565	239915452	protein_coding	85,73	0
ENSG00000163513	TGFBR2	3	30606502	30694142	protein_coding	85,60	0
ENSG00000161328	LRRC56	11	537527	554916	protein_coding	85,50	0
ENSG00000182700	IGIP	5	140125935	140129392	protein_coding	85,42	0
ENSG00000088386	SLC15A1	13	98683801	98752654	protein_coding	85,37	0
ENSG00000064687	ABCA7	19	1040101	1065572	protein_coding	85,32	0
ENSG00000162636	FAM102B	1	108560089	108644900	protein_coding	85,31	0
ENSG00000116132	PRRX1	1	170662728	170739419	protein_coding	85,30	0
ENSG00000188070	C11orf95	11	63759892	63768775	protein_coding	85,24	0
ENSG00000105497	ZNF175	19	51571298	51592508	protein_coding	85,23	0
ENSG00000125741	OPA3	19	45527427	45602212	protein_coding	85,14	0
ENSG00000139428	MMAB	12	109553737	109573874	protein_coding	85,07	0
ENSG00000099330	OCEL1	19	17226204	17229219	protein_coding	85,00	0
ENSG00000204370	SDHD	11	112086773	112120013	protein_coding	85,00	0
ENSG00000128815	WDFY4	10	48684876	48982956	protein_coding	85,00	0
ENSG00000171790	SLFNL1	1	41015597	41023237	protein_coding	84,80	0
ENSG00000128346	C22orf23	22	37943050	37953669	protein_coding	84,65	0
ENSG00000099840	IZUMO4	19	2096429	2099593	protein_coding	84,64	0
ENSG00000243811	APOBEC3D	22	39014363	39033276	protein_coding	84,60	0
ENSG00000178343	SHISA3	4	42397839	42402487	protein_coding	84,60	0
ENSG00000080293	SCTR	2	119439843	119525301	protein_coding	84,60	0
ENSG00000173262	SLC2A14	12	7812512	7891148	protein_coding	84,43	0
ENSG00000002834	LASP1	17	38869859	38921770	protein_coding	84,36	0
ENSG00000119559	C19orf25	19	1461143	1479556	protein_coding	84,25	0
ENSG00000075234	TTC38	22	46267961	46294008	protein_coding	84,20	0
ENSG00000221994	ZNF630	X	47983356	48071658	protein_coding	84,10	0
ENSG00000174156	GSTA3	6	52847910	52909685	protein_coding	84,00	0
ENSG00000249992	TMEM158	3	45224466	45226278	protein_coding	84,00	0
ENSG00000215910	C1orf167	1	11761787	11789585	protein_coding	83,84	0

ENSG00000253626	EIF5AL1	10	79512601	79516440	protein_coding	83,79	0
ENSG00000149782	PLCB3	11	64251523	64269150	protein_coding	83,64	0
ENSG00000128294	TPST2	22	26521983	26596717	protein_coding	83,57	0
ENSG00000136830	FAM129B	9	127505339	127578989	protein_coding	83,51	0
ENSG00000117407	ARTN	1	43933320	43937241	protein_coding	83,50	0
ENSG00000111885	MAN1A1	6	119177209	119349761	protein_coding	83,50	0
ENSG00000205138	SDHAF1	19	35995199	35996315	protein_coding	83,46	0
ENSG00000146109	ABT1	6	26596952	26600744	protein_coding	83,31	0
ENSG00000168675	LDLRAD4	18	13217498	13652755	protein_coding	83,30	0
ENSG00000151006	PRSS53	16	31083425	31089628	protein_coding	83,27	0
ENSG00000101463	SYNDIG1	20	24469199	24666616	protein_coding	83,21	0
ENSG00000121769	FABP3	1	31365625	31376850	protein_coding	83,13	0
ENSG00000115263	GCG	2	162142873	162152404	protein_coding	83,10	0
ENSG00000108375	RNF43	17	58352500	58417595	protein_coding	83,00	0
ENSG00000160161	CILP2	19	19538248	19546659	protein_coding	82,80	0
ENSG00000163734	CXCL3	4	74036589	74038807	protein_coding	82,80	0
ENSG00000165271	NOL6	9	33461441	33473930	protein_coding	82,78	0
ENSG00000102908	NFAT5	16	69565094	69704666	protein_coding	82,75	0
ENSG00000113595	TRIM23	5	65589680	65625975	protein_coding	82,75	0
ENSG00000107736	CDH23	10	71396934	71815947	protein_coding	82,70	0
ENSG00000006327	TNFRSF12A	16	3018445	3022383	protein_coding	82,70	0
ENSG00000183010	PYCR1	17	81932384	81942412	protein_coding	82,67	0
ENSG00000157734	SNX22	15	64151715	64157481	protein_coding	82,66	0
ENSG00000173535	TNFRSF10C	8	23084355	23117437	protein_coding	82,64	0
ENSG00000158869	FCER1G	1	161215234	161220699	protein_coding	82,60	0
ENSG00000008311	AASS	7	122075647	122144280	protein_coding	82,54	0
ENSG00000184060	ADAP2	17	30906344	30959322	protein_coding	82,40	0
ENSG00000160883	HK3	5	176880869	176899332	protein_coding	82,40	0
ENSG00000003137	CYP26B1	2	72129238	72148038	protein_coding	82,30	0
ENSG00000143434	SEMA6C	1	151131685	151146664	protein_coding	82,30	0
ENSG00000182307	C8orf33	8	145052378	145056030	protein_coding	82,12	0
ENSG00000011600	TYROBP	19	35904401	35908295	protein_coding	82,10	0
ENSG00000185453	C19orf68	19	48170692	48197620	protein_coding	82,00	0
ENSG00000006062	MAP3K14	17	45263121	45317040	protein_coding	82,00	0
ENSG00000171302	CANT1	17	78991717	79009867	protein_coding	81,99	0
ENSG00000184281	TSSC4	11	2400488	2403878	protein_coding	81,95	0
ENSG00000151135	TMEM263	12	106955719	106978778	protein_coding	81,89	0
ENSG00000118507	AKAP7	6	131135666	131283535	protein_coding	81,88	0
ENSG00000168002	POLR2G	11	62761544	62766710	protein_coding	81,83	0
ENSG00000161179	YDJC	22	21628089	21630064	protein_coding	81,75	0
ENSG00000129128	SPCS3	4	176319964	176332245	protein_coding	81,72	0
ENSG00000106976	DNM1	9	128203352	128255248	protein_coding	81,70	0
ENSG00000142910	TINAGL1	1	31576485	31587686	protein_coding	81,70	0
ENSG00000102349	KLF8	X	56232421	56287889	protein_coding	81,70	0
ENSG00000196502	SULT1A1	16	28605196	28623625	protein_coding	81,60	0
ENSG00000147100	SLC16A2	X	74421461	74533917	protein_coding	81,60	0
ENSG00000184634	MED12	X	71118556	71142454	protein_coding	81,52	0
ENSG00000172340	SUCLG2	3	67360460	67654614	protein_coding	81,50	0
ENSG00000127419	TMEM175	4	932387	958656	protein_coding	81,47	0
ENSG00000137714	FDX1	11	110429883	110464881	protein_coding	81,45	0
ENSG00000164318	EGFLAM	5	38258409	38465021	protein_coding	81,40	0
ENSG00000111653	ING4	12	6650280	6663148	protein_coding	81,28	0
ENSG00000111335	OAS2	12	112978395	113011723	protein_coding	81,18	0
ENSG00000123374	CDK2	12	55966769	55972784	protein_coding	81,16	0
ENSG00000164056	SPRY1	4	123396795	123403760	protein_coding	81,14	0
ENSG00000115657	ABCB6	2	219209768	219218990	protein_coding	81,10	0
ENSG00000143502	SUSD4	1	223220819	223364202	protein_coding	81,07	0
ENSG00000181513	ACBD4	17	45132600	45144181	protein_coding	81,00	0

ENSG00000172269	DPAGT1	11	119096503	119108331	protein_coding	80,95	0
ENSG00000065802	ASB1	2	238426742	238452250	protein_coding	80,89	0
ENSG00000204228	HSD17B8	6	33204642	33206831	protein_coding	80,86	0
ENSG00000071967	CYBRD1	2	171522247	171558133	protein_coding	80,85	0
ENSG00000130024	PHF10	6	169703905	169725566	protein_coding	80,85	0
ENSG00000107984	DKK1	10	52314296	52318042	protein_coding	80,70	0
ENSG00000182230	FAM153B	5	176060689	176132258	protein_coding	80,63	0
ENSG00000196267	ZNF836	19	52153864	52171643	protein_coding	80,61	0
ENSG00000135709	KIAA0513	16	85027751	85094230	protein_coding	80,57	0
ENSG00000164211	STARD4	5	111496033	111512590	protein_coding	80,53	0
ENSG00000166257	SCN3B	11	123629187	123655244	protein_coding	80,50	0
ENSG00000107331	ABCA2	9	137007227	137028922	protein_coding	80,49	0
ENSG00000013563	DNASE1L1	X	154401238	154412112	protein_coding	80,43	0
ENSG00000143641	GALNT2	1	230057990	230282124	protein_coding	80,38	0
ENSG00000137462	TLR2	4	153701500	153705699	protein_coding	80,30	0
ENSG00000054219	LY75	2	159803355	159904749	protein_coding	80,30	0
ENSG00000141086	CTRL	16	67927640	67932414	protein_coding	80,26	0
ENSG00000142544	CTU1	19	51097606	51108370	protein_coding	79,90	0
ENSG00000167657	DAPK3	19	3958453	3971123	protein_coding	79,87	0
ENSG00000135077	HAVCR2	5	157085832	157142869	protein_coding	79,80	0
ENSG00000275395	FCGBP	19	39863323	39934626	protein_coding	79,79	0
ENSG00000152556	PFKM	12	48105139	48146404	protein_coding	79,50	0
ENSG00000088367	EPB41L1	20	36091504	36232799	protein_coding	79,46	0
ENSG00000154864	PIEZO2	18	10666483	11148762	protein_coding	79,40	0
ENSG00000136463	TACO1	17	63600872	63608365	protein_coding	79,40	0
ENSG00000169756	LIMS1	2	108534355	108687246	protein_coding	79,33	0
ENSG00000163331	DAPL1	2	158795317	158862958	protein_coding	79,30	0
ENSG00000140326	CDAN1	15	42723559	42737126	protein_coding	79,24	0
ENSG00000275183	LENG9	19	54461796	54463711	protein_coding	79,21	0
ENSG00000176971	FIBIN	11	26994184	26996121	protein_coding	79,20	0
ENSG00000163866	SMIM12	1	34712737	34859816	protein_coding	79,16	0
ENSG00000007202	KIAA0100	17	28614440	28645454	protein_coding	78,95	0
ENSG00000176387	HSD11B2	16	67430652	67437553	protein_coding	78,93	0
ENSG00000080573	COL5A3	19	9959561	10010471	protein_coding	78,90	0
ENSG00000111640	GAPDH	12	6533927	6538374	protein_coding	78,85	0
ENSG00000104267	CA2	8	85463852	85481493	protein_coding	78,70	0
ENSG00000176014	TUBB6	18	12307669	12344320	protein_coding	78,66	0
ENSG00000136869	TLR4	9	117704332	117716871	protein_coding	78,57	0
ENSG00000103490	PYCARD	16	31201485	31203450	protein_coding	78,50	0
ENSG00000112659	CUL9	6	43182175	43224587	protein_coding	78,47	0
ENSG00000071246	VASH1	14	76762189	76783015	protein_coding	78,45	0
ENSG00000092931	MFSD11	17	76735865	76781449	protein_coding	78,23	0
ENSG00000256269	HMBS	11	119084866	119093549	protein_coding	78,16	0
ENSG00000130940	CASZ1	1	10636604	10796650	protein_coding	78,05	0
ENSG00000120875	DUSP4	8	29333064	29350668	protein_coding	78,00	0
ENSG00000226887	ERVMER34-1	4	52722618	52751640	protein_coding	78,00	0
ENSG00000076258	FMO4	1	171314208	171342084	protein_coding	78,00	0
ENSG00000182463	TSHZ2	20	52972407	53495330	protein_coding	77,99	0
ENSG00000167614	TTYH1	19	54415219	54436900	protein_coding	77,90	0
ENSG00000066735	KIF26A	14	104138723	104180894	protein_coding	77,83	0
ENSG00000127837	AAMP	2	218264123	218270257	protein_coding	77,75	0
ENSG00000141741	MIEN1	17	39728496	39730787	protein_coding	77,65	0
ENSG00000198324	FAM109A	12	111360651	111369121	protein_coding	77,61	0
ENSG00000102890	ELMO3	16	67199111	67204029	protein_coding	77,60	0
ENSG00000197013	ZNF429	19	21496682	21556270	protein_coding	77,37	0
ENSG00000197863	ZNF790	19	36817428	36850787	protein_coding	77,35	0
ENSG00000010803	SCMH1	1	41027200	41242154	protein_coding	77,32	0
ENSG00000197757	HOXC6	12	53990624	54030823	protein_coding	77,30	0

ENSG00000151208	DLG5	10	77790791	77926526	protein_coding	77,24	0
ENSG00000131831	RAI2	X	17800049	17861337	protein_coding	77,21	0
ENSG00000167965	MLST8	16	2204248	2209416	protein_coding	77,12	0
ENSG00000184470	TXNRD2	22	19875517	19941992	protein_coding	77,10	0
ENSG00000118263	KLF7	2	207074137	207167267	protein_coding	77,06	0
ENSG00000130758	MAP3K10	19	40191744	40215575	protein_coding	77,01	0
ENSG00000221926	TRIM16	17	15627960	15684311	protein_coding	76,93	0
ENSG00000139687	RB1	13	48303751	48481986	protein_coding	76,88	0
ENSG00000144366	GULP1	2	188291669	188595931	protein_coding	76,60	0
ENSG00000189410	SH2D5	1	20719732	20732837	protein_coding	76,60	0
ENSG00000197852	FAM212B	1	111680630	111755824	protein_coding	76,58	0
ENSG00000249471	ZNF324B	19	58451604	58457833	protein_coding	76,40	0
ENSG00000145824	CXCL14	5	135570679	135579279	protein_coding	76,40	0
ENSG00000132768	DPH2	1	43970000	43973369	protein_coding	76,39	0
ENSG00000183751	TBL3	16	1972037	1982933	protein_coding	76,38	0
ENSG00000131165	CHMP1A	16	89644431	89657845	protein_coding	76,37	0
ENSG00000198003	CCDC151	19	11420604	11435782	protein_coding	76,33	0
ENSG00000172830	SSH3	11	67303448	67312607	protein_coding	76,27	0
ENSG00000161013	MGAT4B	5	179797597	179806952	protein_coding	76,27	0
ENSG00000153132	CLGN	4	140388455	140427968	protein_coding	76,25	0
ENSG00000132694	ARHGEF11	1	156934840	157045370	protein_coding	76,20	0
ENSG00000181649	PHLDA2	11	2928273	2929455	protein_coding	76,20	0
ENSG00000271425	NBPF10	1	146064699	146144942	protein_coding	76,14	0
ENSG00000104164	BLOC1S6	15	45587123	45615999	protein_coding	76,09	0
ENSG00000170421	KRT8	12	52897187	52949954	protein_coding	75,96	0
ENSG00000109917	ZPR1	11	116773799	116788050	protein_coding	75,91	0
ENSG00000126464	PRR12	19	49591643	49626439	protein_coding	75,85	0
ENSG00000172716	SLFN11	17	35350305	35373701	protein_coding	75,80	0
ENSG00000145901	TNIP1	5	151029945	151093577	protein_coding	75,74	0
ENSG00000135502	SLC26A10	12	57619527	57626151	protein_coding	75,70	0
ENSG00000142173	COL6A2	21	46098097	46132849	protein_coding	75,63	0
ENSG00000102878	HSF4	16	67164681	67169945	protein_coding	75,61	0
ENSG00000095564	BTAF1	10	91923769	92030325	protein_coding	75,52	0
ENSG00000117054	ACADM	1	75724347	75787575	protein_coding	75,43	0
ENSG00000105401	CDC37	19	10391134	10420121	protein_coding	75,39	0
ENSG00000158290	CUL4B	X	120524609	120575794	protein_coding	75,25	0
ENSG00000177963	RIC8A	11	207511	215113	protein_coding	75,25	0
ENSG00000213983	AP1G2	14	23559565	23568070	protein_coding	75,21	0
ENSG00000147642	SYBU	8	109573978	109691791	protein_coding	75,19	0
ENSG00000184465	WDR27	6	169457212	169702048	protein_coding	75,04	0
ENSG00000118804	STBD1	4	76306026	76311599	protein_coding	74,90	0
ENSG00000160679	CHTOP	1	153633982	153646306	protein_coding	74,87	0
ENSG00000278129	ZNF8	19	58278951	58302805	protein_coding	74,72	0
ENSG00000206432	TMEM200C	18	5882072	5895955	protein_coding	74,70	0
ENSG00000156860	FBRS	16	30658431	30670814	protein_coding	74,70	0
ENSG00000168077	SCARA3	8	27633868	27676776	protein_coding	74,58	0
ENSG00000171443	ZNF524	19	55600022	55603138	protein_coding	74,54	0
ENSG00000188976	NOC2L	1	944204	959309	protein_coding	74,49	0
ENSG00000028310	BRD9	5	850291	892824	protein_coding	74,40	0
ENSG00000257218	GATC	12	120446438	120463749	protein_coding	74,36	0
ENSG00000100842	EFS	14	23356402	23365752	protein_coding	74,30	0
ENSG00000137959	IFI44L	1	78619922	78646145	protein_coding	74,20	0
ENSG00000158714	SLAMF8	1	159826750	159837249	protein_coding	74,20	0
ENSG00000113361	CDH6	5	31193750	31329146	protein_coding	74,15	0
ENSG00000078618	NRD1	1	51789191	51878937	protein_coding	74,11	0
ENSG00000184508	HDDC3	15	90929964	90932569	protein_coding	74,07	0
ENSG00000140285	FGF7	15	49423096	49488775	protein_coding	74,04	0
ENSG00000100599	RIN3	14	92513774	92688994	protein_coding	74,00	0

ENSG00000169967	MAP3K2	2	127298730	127388465	protein_coding	73,99	0
ENSG00000175066	GK5	3	142157527	142225607	protein_coding	73,96	0
ENSG00000197044	ZNF441	19	11767000	11784078	protein_coding	73,86	0
ENSG00000234465	PINLYP	19	43576800	43583964	protein_coding	73,80	0
ENSG00000261408	TEN1-CDK3	17	75979231	76005999	protein_coding	73,70	0
ENSG00000140398	NEIL1	15	75346955	75357114	protein_coding	73,63	0
ENSG00000184785	SMIM10	X	134990938	134992473	protein_coding	73,60	0
ENSG00000157343	ARMC12	6	35737032	35749079	protein_coding	73,54	0
ENSG00000104812	GYS1	19	48968125	48993310	protein_coding	73,47	0
ENSG00000213906	LTB4R2	14	24305734	24312053	protein_coding	73,40	0
ENSG00000148488	ST8SIA6	10	17318383	17454330	protein_coding	73,40	0
ENSG00000081692	JMJD4	1	227730425	227735411	protein_coding	73,37	0
ENSG00000146859	TMEM140	7	135148072	135166215	protein_coding	73,32	0
ENSG00000173542	MOB1B	4	70902326	71022449	protein_coding	73,24	0
ENSG00000255398	HCAR3	12	122714756	122716892	protein_coding	73,20	0
ENSG00000242498	ARPIN	15	89895006	89912956	protein_coding	73,15	0
ENSG00000134463	ECHDC3	10	11742366	11764070	protein_coding	73,14	0
ENSG00000185811	IKZF1	7	50304124	50405101	protein_coding	73,10	0
ENSG00000064545	TMEM161A	19	19119169	19138513	protein_coding	73,08	0
ENSG00000162817	C1orf115	1	220689845	220699157	protein_coding	73,04	0
ENSG00000165644	COMTD1	10	75233969	75236030	protein_coding	73,00	0
ENSG00000112303	VNN2	6	132743870	132763459	protein_coding	73,00	0
ENSG00000135414	GDF11	12	55743280	55757278	protein_coding	72,94	0
ENSG00000136270	TBRG4	7	45100100	45112047	protein_coding	72,74	0
ENSG00000112378	PERP	6	138088505	138107511	protein_coding	72,73	0
ENSG00000135506	OS9	12	57693955	57721557	protein_coding	72,69	0
ENSG00000137834	SMAD6	15	66702228	66782848	protein_coding	72,61	0
ENSG00000265972	TXNIP	1	145992435	145996600	protein_coding	72,58	0
ENSG00000168802	CHTF8	16	69118010	69132584	protein_coding	72,58	0
ENSG00000003756	RBM5	3	50088908	50119021	protein_coding	72,58	0
ENSG00000116678	LEPR	1	65420652	65641559	protein_coding	72,58	0
ENSG00000243317	C7orf73	7	135662496	135693418	protein_coding	72,52	0
ENSG00000180638	SLC47A2	17	19678288	19718979	protein_coding	72,50	0
ENSG00000006756	ARSD	X	2903970	2929351	protein_coding	72,37	0
ENSG00000196782	MAML3	4	139716753	140154184	protein_coding	72,34	0
ENSG00000160972	PPP1R16A	8	144477969	144502121	protein_coding	72,32	0
ENSG00000179954	SSC5D	19	55488404	55519098	protein_coding	72,32	0
ENSG00000278619	MRM1	17	36601572	36608971	protein_coding	72,20	0
ENSG00000188536	HBA2	16	172847	173710	protein_coding	72,20	0
ENSG00000160862	AZGP1	7	99966720	99976157	protein_coding	72,20	0
ENSG00000122122	SASH3	X	129779979	129795201	protein_coding	72,20	0
ENSG00000131943	C19orf12	19	29698886	29715789	protein_coding	72,19	0
ENSG00000074696	HACD3	15	65530418	65578352	protein_coding	72,15	0
ENSG00000183340	JRKL	11	96389989	96507574	protein_coding	72,13	0
ENSG00000138829	FBN2	5	128257909	128659185	protein_coding	72,10	0
ENSG00000165280	VCP	9	35056064	35073249	protein_coding	72,09	0
ENSG00000143630	HCN3	1	155277583	155289848	protein_coding	72,08	0
ENSG00000102125	TAZ	X	154411518	154421726	protein_coding	72,06	0
ENSG00000203879	GDI1	X	154436913	154443467	protein_coding	72,04	0
ENSG00000187954	CYHR1	8	144449582	144465677	protein_coding	72,01	0
ENSG00000073910	FRY	13	32031300	32299122	protein_coding	72,00	0
ENSG00000197558	SSPO	7	149776042	149833979	protein_coding	72,00	0
ENSG00000197324	LRP10	14	22871613	22881580	protein_coding	71,74	0
ENSG00000198771	RCSD1	1	167630093	167706249	protein_coding	71,70	0
ENSG00000127989	MTERF1	7	91692008	91880720	protein_coding	71,62	0
ENSG00000124134	KCNS1	20	45092310	45101112	protein_coding	71,58	0
ENSG00000086730	LAT2	7	74199652	74229834	protein_coding	71,50	0
ENSG00000108828	VAT1	17	43014605	43025123	protein_coding	71,46	0

ENSG00000178531	CTXN1	19	7924485	7926166	protein_coding	71,46	0
ENSG00000111907	TPD52L1	6	125119049	125264407	protein_coding	71,40	0
ENSG00000074266	EED	11	86244544	86278813	protein_coding	71,35	0
ENSG00000151233	GXYLT1	12	42081845	42144879	protein_coding	71,30	0
ENSG00000009830	POMT2	14	77274956	77320884	protein_coding	71,24	0
ENSG00000239789	MRPS17	7	55951819	55956501	protein_coding	71,23	0
ENSG00000102312	PORCN	X	48508962	48520814	protein_coding	71,23	0
ENSG00000206530	CFAP44	3	113286930	113441610	protein_coding	71,19	0
ENSG00000244242	IFITM10	11	1732410	1750591	protein_coding	71,10	0
ENSG00000119638	NEK9	14	75079353	75127344	protein_coding	71,07	0
ENSG00000105197	TIMM50	19	39480412	39493785	protein_coding	70,93	0
ENSG00000279152	ENSG00000	17	8188933	8189688	protein_coding	70,90	0
ENSG00000100307	CBX7	22	39120167	39152674	protein_coding	70,84	0
ENSG00000137103	TMEM8B	9	35814451	35854847	protein_coding	70,78	0
ENSG00000047648	ARHGAP6	X	11137543	11665701	protein_coding	70,75	0
ENSG00000213213	CCDC183	9	136796350	136807741	protein_coding	70,70	0
ENSG00000087266	SH3BP2	4	2793023	2841098	protein_coding	70,64	0
ENSG00000158109	TPRG1L	1	3625002	3630127	protein_coding	70,63	0
ENSG00000126261	UBA2	19	34428352	34471251	protein_coding	70,63	0
ENSG00000075624	ACTB	7	5527151	5563784	protein_coding	70,62	0
ENSG00000176058	TPRN	9	137191617	137204193	protein_coding	70,50	0
ENSG00000113119	TMCO6	5	140639427	140645408	protein_coding	70,47	0
ENSG00000172888	ZNF621	3	40524878	40574685	protein_coding	70,46	0
ENSG00000196391	ZNF774	15	90352245	90369146	protein_coding	70,45	0
ENSG00000161202	DVL3	3	184155388	184173610	protein_coding	70,39	0
ENSG00000137841	PLCB2	15	40278176	40307935	protein_coding	70,38	0
ENSG00000188766	SPRED3	19	38388421	38399587	protein_coding	70,37	0
ENSG00000135333	EPHA7	6	93240020	93419547	protein_coding	70,20	0
ENSG00000168621	GDNF	5	37812677	37839686	protein_coding	70,20	0
ENSG00000165102	HGSNAT	8	43140455	43202855	protein_coding	70,12	0
ENSG00000079739	PGM1	1	63593276	63660245	protein_coding	70,10	0
ENSG00000256591	ENSG00000	11	61429220	61485822	protein_coding	70,00	0
ENSG00000155099	TMEM55A	8	90993796	91041064	protein_coding	70,00	0
ENSG00000169217	CD2BP2	16	30350766	30355361	protein_coding	69,90	0
ENSG00000129562	DAD1	14	22564905	22589269	protein_coding	69,88	0
ENSG00000244038	DDOST	1	20651767	20661544	protein_coding	69,86	0
ENSG00000167118	URM1	9	128371319	128392016	protein_coding	69,81	0
ENSG00000163528	CHCHD4	3	14112077	14124870	protein_coding	69,78	0
ENSG00000159189	C1QC	1	22643630	22648110	protein_coding	69,70	0
ENSG00000138660	AP1AR	4	112231737	112270047	protein_coding	69,60	0
ENSG00000130748	TMEM160	19	47045907	47048630	protein_coding	69,57	0
ENSG00000162576	MXRA8	1	1352689	1361777	protein_coding	69,56	0
ENSG00000142606	MMEL1	1	2590639	2632990	protein_coding	69,53	0
ENSG00000102886	GDPD3	16	30104810	30113856	protein_coding	69,50	0
ENSG00000102003	SYP	X	49187804	49200259	protein_coding	69,50	0
ENSG00000205593	DENND6B	22	50309030	50327060	protein_coding	69,47	0
ENSG00000170820	FSHR	2	48962157	49154537	protein_coding	69,43	0
ENSG00000089723	OTUB2	14	94026329	94048930	protein_coding	69,42	0
ENSG00000147813	NAPRT	8	143574785	143578649	protein_coding	69,40	0
ENSG00000197417	SHPK	17	3608262	3636322	protein_coding	69,20	0
ENSG00000140995	DEF8	16	89947925	89968060	protein_coding	69,18	0
ENSG00000197483	ZNF628	19	55476332	55484487	protein_coding	69,17	0
ENSG00000176024	ZNF613	19	51879872	51948759	protein_coding	69,11	0
ENSG00000102531	FNDC3A	13	48975912	49209779	protein_coding	69,11	0
ENSG00000172046	USP19	3	49108046	49120938	protein_coding	69,08	0
ENSG00000186765	FSCN2	17	81528396	81537130	protein_coding	69,00	0
ENSG00000140481	CCDC33	15	74236289	74336472	protein_coding	69,00	0
ENSG00000172243	CLEC7A	12	10116777	10130258	protein_coding	69,00	0

ENSG00000184788	SATL1	X	85092287	85109048	protein_coding	69,00	0
ENSG00000198198	SZT2	1	43389882	43452650	protein_coding	68,82	0
ENSG00000141012	GALNS	16	88813734	88856970	protein_coding	68,81	0
ENSG00000196735	HLA-DQA1	6	32628179	32647062	protein_coding	68,80	0
ENSG00000154639	CXADR	21	17512382	17593579	protein_coding	68,74	0
ENSG00000196154	S100A4	1	153543613	153550136	protein_coding	68,70	0
ENSG00000182272	B4GALNT4	11	369796	382116	protein_coding	68,69	0
ENSG00000213563	C8orf82	8	144525733	144529132	protein_coding	68,66	0
ENSG00000166734	CASC4	15	44288729	44415758	protein_coding	68,62	0
ENSG00000149743	TRPT1	11	64223799	64226254	protein_coding	68,60	0
ENSG00000119922	IFIT2	10	89301955	89309276	protein_coding	68,60	0
ENSG00000179564	LSMEM2	3	50279027	50288114	protein_coding	68,60	0
ENSG00000115523	GNLY	2	85685175	85698854	protein_coding	68,60	0
ENSG00000153246	PLA2R1	2	159932006	160062610	protein_coding	68,57	0
ENSG00000166173	LARP6	15	70829130	70854159	protein_coding	68,56	0
ENSG00000005249	PRKAR2B	7	107044649	107161811	protein_coding	68,52	0
ENSG00000163785	RYK	3	134065303	134250744	protein_coding	68,51	0
ENSG00000159882	ZNF230	19	44002948	44013926	protein_coding	68,50	0
ENSG00000174791	RIN1	11	66330242	66336840	protein_coding	68,50	0
ENSG00000177669	MBOAT4	8	30131824	30144686	protein_coding	68,50	0
ENSG00000106346	USP42	7	6104884	6161564	protein_coding	68,47	0
ENSG00000143819	EPHX1	1	225810092	225845563	protein_coding	68,46	0
ENSG00000100029	PES1	22	30576625	30607083	protein_coding	68,45	0
ENSG00000184967	NOC4L	12	132144448	132152473	protein_coding	68,41	0
ENSG00000136738	STAM	10	17644125	17715914	protein_coding	68,34	0
ENSG00000139291	TMEM19	12	71686087	71705046	protein_coding	68,29	0
ENSG00000071553	ATP6AP1	X	154428632	154436516	protein_coding	68,25	0
ENSG00000177303	CASKIN2	17	75500261	75515583	protein_coding	68,18	0
ENSG00000112062	MAPK14	6	36027677	36111236	protein_coding	68,17	0
ENSG00000079805	DNM2	19	10718079	10833488	protein_coding	68,09	0
ENSG00000187994	RINL	19	38867834	38878279	protein_coding	68,07	0
ENSG00000108819	PPP1R9B	17	50133735	50150630	protein_coding	68,02	0
ENSG00000177082	WDR73	15	84639281	84654343	protein_coding	68,02	0
ENSG00000139549	DHH	12	49089421	49094819	protein_coding	68,00	0
ENSG00000175203	DCTN2	12	57530102	57547331	protein_coding	68,00	0
ENSG00000185361	TNFAIP8L1	19	4639518	4655568	protein_coding	67,94	0
ENSG00000069122	ADGRF5	6	46852512	46954943	protein_coding	67,90	0
ENSG00000165055	METTL2B	7	128476729	128506602	protein_coding	67,86	0
ENSG00000163931	TKT	3	53224707	53256052	protein_coding	67,79	0
ENSG00000219200	RNASEK	17	7012417	7014532	protein_coding	67,73	0
ENSG00000161798	AQP5	12	49961870	49965681	protein_coding	67,73	0
ENSG00000139112	GABARAPL1	12	10212458	10223130	protein_coding	67,65	0
ENSG00000100300	TSPO	22	43151514	43163242	protein_coding	67,64	0
ENSG00000219626	FAM228B	2	24076526	24169640	protein_coding	67,60	0
ENSG00000163590	PPM1L	3	160755602	161078907	protein_coding	67,56	0
ENSG00000176715	ACSF3	16	89088375	89155846	protein_coding	67,55	0
ENSG00000043143	JADE2	5	134524312	134583230	protein_coding	67,49	0
ENSG00000107521	HPS1	10	98416198	98446947	protein_coding	67,48	0
ENSG00000176087	SLC35A4	5	140564456	140569103	protein_coding	67,47	0
ENSG00000167695	FAM57A	17	732412	742972	protein_coding	67,45	0
ENSG00000160961	ZNF333	19	14689801	14733746	protein_coding	67,44	0
ENSG00000101413	RPRD1B	20	38033546	38127780	protein_coding	67,42	0
ENSG00000133250	ZNF414	19	8509678	8514164	protein_coding	67,40	0
ENSG00000250510	GPR162	12	6821545	6829972	protein_coding	67,40	0
ENSG00000133135	RNF128	X	106693794	106796993	protein_coding	67,40	0
ENSG00000164403	SHROOM1	5	132822141	132830898	protein_coding	67,39	0
ENSG00000163069	SGCB	4	52020706	52038482	protein_coding	67,39	0
ENSG00000184205	TSPYL2	X	53082367	53088540	protein_coding	67,36	0

ENSG00000171204	TMEM126B	11	85628573	85636539	protein_coding	67,35	0
ENSG00000077454	LRCH4	7	100574011	100586153	protein_coding	67,32	0
ENSG00000114646	CSPG5	3	47562239	47580792	protein_coding	67,30	0
ENSG00000079385	CEACAM1	19	42507304	42561234	protein_coding	67,20	0
ENSG00000069998	CECR5	22	17137511	17165287	protein_coding	67,15	0
ENSG00000162819	BROX	1	222712553	222735196	protein_coding	67,13	0
ENSG00000264324	ENSG00000	2	74211604	74363377	protein_coding	67,08	0
ENSG00000204576	PRR3	6	30556886	30563723	protein_coding	67,04	0
ENSG00000104687	GSR	8	30678061	30727926	protein_coding	66,97	0
ENSG00000162694	EXTL2	1	100872372	100895998	protein_coding	66,95	0
ENSG00000109089	CDR2L	17	74987632	75005800	protein_coding	66,94	0
ENSG00000204252	HLA-DOA	6	33004178	33009612	protein_coding	66,90	0
ENSG00000101460	MAP1LC3A	20	34546854	34560345	protein_coding	66,87	0
ENSG00000188706	ZDHHC9	X	129803288	129843909	protein_coding	66,86	0
ENSG00000141873	SLC39A3	19	2732204	2740152	protein_coding	66,83	0
ENSG00000173227	SYT12	11	67006778	67050863	protein_coding	66,70	0
ENSG00000110811	P3H3	12	6828410	6839851	protein_coding	66,60	0
ENSG00000147437	GNRH1	8	25419260	25424654	protein_coding	66,60	0
ENSG00000162944	RFTN2	2	197568224	197676045	protein_coding	66,60	0
ENSG00000185627	PSMD13	11	236546	252984	protein_coding	66,58	0
ENSG00000154822	PLCL2	3	16802651	17090594	protein_coding	66,57	0
ENSG00000187630	DHRS4L2	14	23969874	24006408	protein_coding	66,50	0
ENSG00000133111	RFXAP	13	36819224	36829104	protein_coding	66,50	0
ENSG00000130638	ATXN10	22	45671798	45845307	protein_coding	66,45	0
ENSG00000256537	SMIM10L1	12	11171222	11176016	protein_coding	66,45	0
ENSG00000152229	PSTPIP2	18	45983536	46072272	protein_coding	66,43	0
ENSG00000167323	STIM1	11	3854527	4093210	protein_coding	66,42	0
ENSG00000162373	BEND5	1	48727523	48776969	protein_coding	66,40	0
ENSG00000142459	EVI5L	19	7830233	7864976	protein_coding	66,38	0
ENSG00000167792	NDUFV1	11	67606852	67612535	protein_coding	66,35	0
ENSG00000074964	ARHGEF10L	1	17539835	17697874	protein_coding	66,33	0
ENSG00000158352	SHROOM4	X	50591647	50814302	protein_coding	66,33	0
ENSG00000090104	RGS1	1	192575727	192580031	protein_coding	66,30	0
ENSG00000145623	OSMR	5	38845858	38945596	protein_coding	66,26	0
ENSG00000111481	COPZ1	12	54301202	54351849	protein_coding	66,21	0
ENSG00000198429	ZNF69	19	11887784	11914329	protein_coding	66,20	0
ENSG00000198431	TXNRD1	12	104215779	104350305	protein_coding	66,18	0
ENSG00000068305	MEF2A	15	99565417	99716466	protein_coding	66,08	0
ENSG00000197150	ABCB8	7	151028422	151047782	protein_coding	66,04	0
ENSG00000155729	KCTD18	2	200488952	200519784	protein_coding	65,91	0
ENSG00000147459	DOCK5	8	25184723	25418082	protein_coding	65,91	0
ENSG00000164548	TRA2A	7	23504780	23532041	protein_coding	65,89	0
ENSG00000197965	MPZL1	1	167721192	167791919	protein_coding	65,81	0
ENSG00000124766	SOX4	6	21592769	21598619	protein_coding	65,80	0
ENSG00000165300	SLITRK5	13	87672615	87696272	protein_coding	65,80	0
ENSG00000168071	CCDC88B	11	64340223	64357534	protein_coding	65,80	0
ENSG00000178896	EXOSC4	8	144078626	144080647	protein_coding	65,80	0
ENSG00000117592	PRDX6	1	173477266	173488807	protein_coding	65,79	0
ENSG00000173465	SSSCA1	11	65570430	65573942	protein_coding	65,67	0
ENSG00000187713	TMEM203	9	137204082	137205638	protein_coding	65,62	0
ENSG00000172322	CLEC12A	12	9951316	9995694	protein_coding	65,60	0
ENSG00000205085	FAM71F2	7	128672288	128687872	protein_coding	65,60	0
ENSG00000240694	PNMA2	8	26504686	26514092	protein_coding	65,58	0
ENSG00000168288	MMADHC	2	149569634	149587816	protein_coding	65,57	0
ENSG00000117480	FAAH	1	46394265	46413848	protein_coding	65,53	0
ENSG00000258947	TUBB3	16	89921392	89938761	protein_coding	65,50	0
ENSG00000075407	ZNF37A	10	38094336	38125544	protein_coding	65,39	0
ENSG00000164691	TAGAP	6	159034468	159045152	protein_coding	65,36	0

ENSG00000149218	ENDOD1	11	95089810	95132645	protein_coding	65,33	0
ENSG00000167748	KLK1	19	50819148	50823787	protein_coding	65,30	0
ENSG00000105705	SUGP1	19	19276018	19320844	protein_coding	65,30	0
ENSG00000127526	SLC35E1	19	16549831	16572382	protein_coding	65,29	0
ENSG00000172469	MANEA	6	95577543	95609457	protein_coding	65,23	0
ENSG00000276293	PIP4K2B	17	38765689	38800126	protein_coding	65,21	0
ENSG00000155629	PIK3AP1	10	96593312	96720514	protein_coding	65,19	0
ENSG00000181409	AATK	17	81117295	81166077	protein_coding	65,06	0
ENSG00000101972	STAG2	X	123960212	124422664	protein_coding	65,03	0
ENSG00000180626	ZNF594	17	5179536	5191883	protein_coding	64,97	0
ENSG00000175414	ARL10	5	176365468	176401865	protein_coding	64,86	0
ENSG00000198715	GLMP	1	156290089	156295689	protein_coding	64,79	0
ENSG00000150627	WDR17	4	176065834	176182818	protein_coding	64,73	0
ENSG00000179841	AKAP5	14	64465499	64474503	protein_coding	64,70	0
ENSG00000160999	SH2B2	7	102285091	102321711	protein_coding	64,60	0
ENSG00000178498	DTX3	12	57604622	57609804	protein_coding	64,59	0
ENSG00000182957	SPATA13	13	23979805	24307074	protein_coding	64,58	0
ENSG00000148057	IDNK	9	83623049	83644130	protein_coding	64,54	0
ENSG00000127554	GFER	16	1984207	1987749	protein_coding	64,41	0
ENSG00000067177	PHKA1	X	72578814	72714319	protein_coding	64,40	0
ENSG00000133101	CCNA1	13	36431520	36442882	protein_coding	64,35	0
ENSG00000196296	ATP2A1	16	28878405	28904509	protein_coding	64,29	0
ENSG00000261221	ZNF865	19	55605405	55617269	protein_coding	64,28	0
ENSG00000111786	SRSF9	12	120461668	120469793	protein_coding	64,26	0
ENSG00000110077	MS4A6A	11	60172014	60184666	protein_coding	64,20	0
ENSG00000130844	ZNF331	19	53520981	53580269	protein_coding	64,17	0
ENSG00000213214	ARHGEF35	7	144186083	144195655	protein_coding	64,00	0
ENSG00000188163	FAM166A	9	137243584	137247770	protein_coding	63,78	0
ENSG00000100147	CCDC134	22	41800679	41826299	protein_coding	63,77	0
ENSG00000221886	ZBED8	5	160393148	160400097	protein_coding	63,70	0
ENSG00000172986	GXYLT2	3	72888073	72998138	protein_coding	63,66	0
ENSG00000128891	C15orf57	15	40528683	40565057	protein_coding	63,62	0
ENSG00000143125	PROK1	1	110451200	110457354	protein_coding	63,50	0
ENSG00000188636	LDOC1L	22	44492572	44498298	protein_coding	63,44	0
ENSG00000100422	CERK	22	46684411	46738261	protein_coding	63,41	0
ENSG00000156510	HKDC1	10	69220303	69267559	protein_coding	63,40	0
ENSG00000142627	EPHA2	1	16124337	16156087	protein_coding	63,36	0
ENSG00000159588	CCDC17	1	45620044	45624057	protein_coding	63,30	0
ENSG00000107614	TRDMT1	10	17142254	17202054	protein_coding	63,26	0
ENSG00000226784	PGAM4	X	77968874	77969638	protein_coding	63,20	0
ENSG00000175984	DENND2C	1	114582848	114670422	protein_coding	63,18	0
ENSG00000137522	RNF121	11	71928701	71997597	protein_coding	63,14	0
ENSG00000122515	ZMIZ2	7	44748581	44769881	protein_coding	63,07	0
ENSG00000141569	TRIM65	17	75880335	75897003	protein_coding	63,00	0
ENSG00000150782	IL18	11	112143251	112164117	protein_coding	63,00	0
ENSG00000135378	PRRG4	11	32829943	32858123	protein_coding	62,94	0
ENSG00000152475	ZNF837	19	58367618	58381060	protein_coding	62,80	0
ENSG00000130830	MPP1	X	154778684	154821007	protein_coding	62,77	0
ENSG00000124786	SLC35B3	6	8413068	8435483	protein_coding	62,74	0
ENSG00000186687	LYRM7	5	131170810	131205426	protein_coding	62,73	0
ENSG00000263528	IKBKE	1	206470476	206496889	protein_coding	62,73	0
ENSG00000172071	EIF2AK3	2	88556741	88627576	protein_coding	62,71	0
ENSG00000178878	APOLD1	12	12725917	12829975	protein_coding	62,70	0
ENSG00000116774	OLFML3	1	113979391	114035572	protein_coding	62,70	0
ENSG00000150961	SEC24D	4	118722823	118838683	protein_coding	62,67	0
ENSG00000132640	BTBD3	20	11890723	11926609	protein_coding	62,65	0
ENSG00000106546	AHR	7	17298622	17346152	protein_coding	62,64	0
ENSG00000168502	MTCL1	18	8705661	8832778	protein_coding	62,61	0

ENSG00000178573	MAF	16	79585843	79600714	protein_coding	62,58	0
ENSG00000162004	CCDC78	16	722582	726954	protein_coding	62,50	0
ENSG00000065615	CYB5R4	6	83859643	83967424	protein_coding	62,48	0
ENSG00000008300	CELSR3	3	48636469	48662915	protein_coding	62,46	0
ENSG00000134627	PIWIL4	11	94543840	94621421	protein_coding	62,40	0
ENSG00000160867	FGFR4	5	177086886	177098144	protein_coding	62,27	0
ENSG00000088451	TGDS	13	94574051	94596257	protein_coding	62,14	0
ENSG00000198393	ZNF26	12	132986365	133032952	protein_coding	62,14	0
ENSG00000105662	CRTC1	19	18683677	18782333	protein_coding	62,13	0
ENSG00000197102	DYNC1H1	14	101964528	102050792	protein_coding	62,13	0
ENSG00000137672	TRPC6	11	101451564	101872562	protein_coding	62,07	0
ENSG00000110057	UNC93B1	11	67991104	68004982	protein_coding	61,97	0
ENSG00000114268	PFKFB4	3	48517684	48562015	protein_coding	61,92	0
ENSG00000148175	STOM	9	121338988	121370304	protein_coding	61,91	0
ENSG00000119718	EIF2B2	14	75002911	75012366	protein_coding	61,83	0
ENSG00000007314	SCN4A	17	63938554	63972918	protein_coding	61,80	0
ENSG00000187607	ZNF286A	17	15699577	15720787	protein_coding	61,80	0
ENSG00000133895	MEN1	11	64803510	64811294	protein_coding	61,80	0
ENSG00000196498	NCOR2	12	124324415	124567589	protein_coding	61,79	0
ENSG00000156966	B3GNT7	2	231395543	231401164	protein_coding	61,75	0
ENSG00000167434	CA4	17	60149936	60170899	protein_coding	61,70	0
ENSG00000158691	ZSCAN12	6	28378955	28399734	protein_coding	61,64	0
ENSG00000155366	RHOC	1	112701106	112707434	protein_coding	61,56	0
ENSG00000100902	PSMA6	14	35278633	35317493	protein_coding	61,55	0
ENSG00000141644	MBD1	18	50266882	50281774	protein_coding	61,53	0
ENSG00000181617	FDCSP	4	70226071	70235252	protein_coding	61,50	0
ENSG00000092969	TGFB2	1	218346235	218444619	protein_coding	61,50	0
ENSG00000166436	TRIM66	11	8612037	8671866	protein_coding	61,45	0
ENSG00000134245	WNT2B	1	112466541	112530165	protein_coding	61,42	0
ENSG00000174125	TLR1	4	38790677	38856817	protein_coding	61,40	0
ENSG00000189337	KAZN	1	14598704	15118043	protein_coding	61,40	0
ENSG00000123384	LRP1	12	57128493	57213351	protein_coding	61,37	0
ENSG00000134363	FST	5	53480409	53487134	protein_coding	61,34	0
ENSG00000166716	ZNF592	15	84748635	84806432	protein_coding	61,33	0
ENSG00000135919	SERPINE2	2	223975112	224039319	protein_coding	61,31	0
ENSG00000133433	GSTT2B	22	23957414	23961186	protein_coding	61,20	0
ENSG00000137285	TUBB2B	6	3224261	3231730	protein_coding	61,20	0
ENSG00000147383	NSDHL	X	152830967	152869729	protein_coding	61,11	0
ENSG00000197935	ZNF311	6	28994785	29005316	protein_coding	61,10	0
ENSG00000099864	PALM	19	708939	748329	protein_coding	61,09	0
ENSG00000117906	RCN2	15	76931619	76954392	protein_coding	61,05	0
ENSG00000126218	F10	13	113122814	113149529	protein_coding	61,00	0
ENSG00000129255	MPDU1	17	7583529	7592789	protein_coding	60,98	0
ENSG00000186767	SPIN4	X	63347228	63351344	protein_coding	60,70	0
ENSG00000108826	MRPL27	17	50367857	50373214	protein_coding	60,67	0
ENSG00000114626	ABTB1	3	127672935	127680920	protein_coding	60,65	0
ENSG00000087074	PPP1R15A	19	48872392	48876057	protein_coding	60,58	0
ENSG00000127922	SHFM1	7	96481626	96709891	protein_coding	60,52	0
ENSG00000175206	NPPA	1	11845709	11848345	protein_coding	60,50	0
ENSG00000113248	PCDHB15	5	141245349	141249365	protein_coding	60,40	0
ENSG00000172819	RARG	12	53210567	53232980	protein_coding	60,10	0
ENSG00000118257	NRP2	2	205681990	205798133	protein_coding	60,06	0
ENSG00000144589	STK11IP	2	219597860	219616451	protein_coding	59,95	0
ENSG00000142694	EVA1B	1	36322031	36324154	protein_coding	59,92	0
ENSG00000144120	TMEM177	2	119679167	119686507	protein_coding	59,90	0
ENSG00000110442	COMMD9	11	36269284	36289449	protein_coding	59,89	0
ENSG00000103202	NME4	16	396725	410367	protein_coding	59,85	0
ENSG00000104892	KLC3	19	45333434	45351520	protein_coding	59,79	0

ENSG00000133561	GIMAP6	7	150625375	150632648	protein_coding	59,70	0
ENSG00000104885	DOT1L	19	2164149	2232578	protein_coding	59,65	0
ENSG00000145337	PYURF	4	88520985	88523813	protein_coding	59,63	0
ENSG00000135624	CCT7	2	73233420	73253021	protein_coding	59,62	0
ENSG00000164976	KIAA1161	9	34366670	34376853	protein_coding	59,62	0
ENSG00000174989	FBXW8	12	116910956	117031148	protein_coding	59,58	0
ENSG00000111358	GTF2H3	12	123633739	123662606	protein_coding	59,57	0
ENSG00000204366	ZBTB12	6	31899607	31901992	protein_coding	59,53	0
ENSG00000099957	P2RX6	22	21009808	21028830	protein_coding	59,50	0
ENSG00000100979	PLTP	20	45898621	45912155	protein_coding	59,46	0
ENSG00000168734	PKIG	20	44531785	44624247	protein_coding	59,42	0
ENSG00000178950	GAK	4	849276	932373	protein_coding	59,34	0
ENSG00000160678	S100A1	1	153627926	153632039	protein_coding	59,30	0
ENSG00000164708	PGAM2	7	44062727	44065587	protein_coding	59,25	0
ENSG00000198933	TBKBP1	17	47694081	47712050	protein_coding	59,24	0
ENSG00000160953	MUM1	19	1285893	1378431	protein_coding	59,21	0
ENSG00000181523	SGSH	17	80206716	80220923	protein_coding	59,09	0
ENSG00000100068	LRP5L	22	25351418	25405377	protein_coding	59,00	0
ENSG00000127081	ZNF484	9	92845031	92878038	protein_coding	58,96	0
ENSG00000136156	ITM2B	13	48233158	48270357	protein_coding	58,83	0
ENSG00000116521	SCAMP3	1	155255979	155262430	protein_coding	58,82	0
ENSG00000137825	ITPKA	15	41493393	41503551	protein_coding	58,80	0
ENSG00000183020	AP2A2	11	924894	1012245	protein_coding	58,61	0
ENSG00000011638	TMEM159	16	21158377	21180616	protein_coding	58,61	0
ENSG00000175224	ATG13	11	46617527	46674818	protein_coding	58,60	0
ENSG00000131459	GFPT2	5	180300690	180353387	protein_coding	58,51	0
ENSG00000085998	POMGNT1	1	46188682	46220305	protein_coding	58,50	0
ENSG00000112308	C6orf62	6	24704861	24720836	protein_coding	58,48	0
ENSG00000122779	TRIM24	7	138460334	138589993	protein_coding	58,45	0
ENSG00000106263	EIF3B	7	2354086	2380745	protein_coding	58,45	0
ENSG00000163162	RNF149	2	101271219	101308701	protein_coding	58,45	0
ENSG00000155158	TTC39B	9	15163622	15307360	protein_coding	58,41	0
ENSG00000235568	NFAM1	22	42380410	42432395	protein_coding	58,33	0
ENSG00000160111	CPAMD8	19	16892947	17026815	protein_coding	58,30	0
ENSG00000239382	ALKBH6	19	36009120	36014239	protein_coding	58,30	0
ENSG00000095380	NANS	9	98056739	98083075	protein_coding	58,30	0
ENSG00000168237	GLYCTK	3	52287089	52293476	protein_coding	58,26	0
ENSG00000160209	PDXK	21	43719094	43762307	protein_coding	58,17	0
ENSG00000121297	TSHZ3	19	31274945	31349547	protein_coding	58,11	0
ENSG00000105472	CLEC11A	19	50723329	50725718	protein_coding	58,10	0
ENSG00000137207	YIPF3	6	43511827	43516990	protein_coding	58,09	0
ENSG00000186417	GLDN	15	51341629	51408013	protein_coding	58,07	0
ENSG00000175556	LONRF3	X	118974614	119018355	protein_coding	58,00	0
ENSG00000125148	MT2A	16	56608199	56609497	protein_coding	57,93	0
ENSG00000068885	IFT80	3	160256986	160399880	protein_coding	57,90	0
ENSG00000253305	PCDHGB6	5	141408021	141512979	protein_coding	57,90	0
ENSG00000189319	FAM53B	10	124619292	124744269	protein_coding	57,89	0
ENSG00000069493	CLEC2D	12	9664969	9699555	protein_coding	57,86	0
ENSG00000106333	PCOLCE	7	100602177	100608175	protein_coding	57,80	0
ENSG00000138100	TRIM54	2	27282392	27307439	protein_coding	57,80	0
ENSG00000106823	ECM2	9	92493554	92536655	protein_coding	57,77	0
ENSG00000105088	OLFM2	19	9853718	9936552	protein_coding	57,71	0
ENSG00000104907	TRMT1	19	13104902	13117567	protein_coding	57,71	0
ENSG00000131389	SLC6A6	3	14402576	14489349	protein_coding	57,71	0
ENSG00000182890	GLUD2	X	121047588	121050080	protein_coding	57,67	0
ENSG00000173599	PC	11	66848233	66958376	protein_coding	57,61	0
ENSG00000132507	EIF5A	17	7306999	7312463	protein_coding	57,61	0
ENSG00000113790	EHHADH	3	185190624	185281990	protein_coding	57,60	0

ENSG00000123570	RAB9B	X	103822322	103832228	protein_coding	57,60	0
ENSG00000064225	ST3GAL6	3	98732236	98821201	protein_coding	57,57	0
ENSG00000008441	NFIX	19	12995608	13098796	protein_coding	57,52	0
ENSG00000130724	CHMP2A	19	58551566	58555124	protein_coding	57,51	0
ENSG00000171097	CCBL1	9	128832942	128882494	protein_coding	57,42	0
ENSG00000165905	GYLTL1B	11	45921621	45929096	protein_coding	57,40	0
ENSG00000215193	PEX26	22	18077920	18131138	protein_coding	57,34	0
ENSG00000143494	VASH2	1	212950520	212992037	protein_coding	57,33	0
ENSG00000174130	TLR6	4	38823715	38856817	protein_coding	57,30	0
ENSG00000171189	GRIK1	21	29536933	29940033	protein_coding	57,18	0
ENSG00000151617	EDNRA	4	147480917	147544954	protein_coding	57,08	0
ENSG00000101333	PLCB4	20	9068763	9481242	protein_coding	56,91	0
ENSG00000164530	PI16	6	36948263	36964837	protein_coding	56,90	0
ENSG00000109758	HGFAC	4	3441887	3449495	protein_coding	56,90	0
ENSG00000154229	PRKCA	17	66302636	66810743	protein_coding	56,89	0
ENSG00000041988	THAP3	1	6624866	6635586	protein_coding	56,88	0
ENSG00000107262	BAG1	9	33247820	33264761	protein_coding	56,87	0
ENSG00000213420	GPC2	7	100169606	100177372	protein_coding	56,86	0
ENSG00000126882	FAM78A	9	131258076	131276547	protein_coding	56,76	0
ENSG00000050130	JKAMP	14	59484443	59505410	protein_coding	56,75	0
ENSG00000099889	ARVCF	22	19969896	20016808	protein_coding	56,71	0
ENSG00000125826	RBCK1	20	407498	430966	protein_coding	56,65	0
ENSG00000072840	EVC	4	5711197	5814305	protein_coding	56,64	0
ENSG00000104142	VPS18	15	40894430	40903975	protein_coding	56,64	0
ENSG00000010030	ETV7	6	36354091	36387800	protein_coding	56,60	0
ENSG00000123240	OPTN	10	13099449	13138308	protein_coding	56,55	0
ENSG00000162194	LBHD1	11	62662817	62672255	protein_coding	56,51	0
ENSG00000117036	ETV3	1	157121191	157138474	protein_coding	56,50	0
ENSG00000107317	PTGDS	9	136977505	136985435	protein_coding	56,50	0
ENSG00000155666	KDM8	16	27203495	27221768	protein_coding	56,44	0
ENSG00000162148	PPP1R32	11	61481120	61490931	protein_coding	56,40	0
ENSG00000135679	MDM2	12	68808172	68850686	protein_coding	56,36	0
ENSG00000176428	VPS37D	7	73667825	73672112	protein_coding	56,30	0
ENSG00000213638	ADAT3	19	1905378	1913447	protein_coding	56,20	0
ENSG00000135454	B4GALNT1	12	57623410	57633355	protein_coding	56,20	0
ENSG00000163832	ELP6	3	47495640	47513761	protein_coding	56,17	0
ENSG00000185745	IFIT1	10	89392546	89406486	protein_coding	56,10	0
ENSG00000115598	IL1RL2	2	102186973	102240002	protein_coding	56,00	0
ENSG00000181004	BBS12	4	122732702	122744943	protein_coding	55,98	0
ENSG00000185730	ZNF696	8	143289676	143298061	protein_coding	55,92	0
ENSG00000123572	NRK	X	105822543	105958610	protein_coding	55,81	0
ENSG00000027869	SH2D2A	1	156806243	156816862	protein_coding	55,70	0
ENSG00000138823	MTTP	4	99563761	99623999	protein_coding	55,68	0
ENSG00000173992	CCS	11	66592821	66606019	protein_coding	55,66	0
ENSG00000139318	DUSP6	12	89347232	89353271	protein_coding	55,60	0
ENSG00000119537	KDSR	18	63327726	63367510	protein_coding	55,57	0
ENSG00000141428	C18orf21	18	35972083	35979286	protein_coding	55,51	0
ENSG00000112992	NNT	5	43602692	43707405	protein_coding	55,49	0
ENSG00000247595	SPTY2D1-AS	11	18599787	18610255	protein_coding	55,41	0
ENSG00000099985	OSM	22	30262829	30266840	protein_coding	55,40	0
ENSG00000167895	TMC8	17	78130770	78142968	protein_coding	55,40	0
ENSG00000162711	NLRP3	1	247416156	247449108	protein_coding	55,40	0
ENSG00000089820	ARHGAP4	X	153907367	153934999	protein_coding	55,39	0
ENSG00000125459	MSTO1	1	155610205	155614967	protein_coding	55,38	0
ENSG00000004660	CAMKK1	17	3860315	3894891	protein_coding	55,21	0
ENSG00000144504	ANKMY1	2	240479422	240569209	protein_coding	55,17	0
ENSG00000116863	ADPRHL2	1	36088875	36093932	protein_coding	55,13	0
ENSG00000170092	SPDYE5	7	75493625	75504304	protein_coding	55,10	0

ENSG00000140718	FTO	16	53703963	54121941	protein_coding	55,08	0
ENSG00000197562	RAB40C	16	589357	629272	protein_coding	55,01	0
ENSG00000008283	CYB561	17	63432304	63446378	protein_coding	55,00	0
ENSG00000074416	MGLL	3	127689062	127823250	protein_coding	55,00	0
ENSG00000162909	CAPN2	1	223701593	223776018	protein_coding	54,99	0
ENSG00000101608	MYL12A	18	3247481	3256236	protein_coding	54,97	0
ENSG00000125827	TMX4	20	7977348	8019829	protein_coding	54,96	0
ENSG00000141527	CARD14	17	80169992	80209331	protein_coding	54,95	0
ENSG00000144136	SLC20A1	2	112645857	112663827	protein_coding	54,95	0
ENSG00000198205	ZXDA	X	57906708	57910820	protein_coding	54,90	0
ENSG00000161999	JMJD8	16	681671	684528	protein_coding	54,78	0
ENSG00000174903	RAB1B	11	66268533	66277492	protein_coding	54,74	0
ENSG00000074660	SCARF1	17	1633858	1645747	protein_coding	54,73	0
ENSG00000172020	GAP43	3	115623324	115721490	protein_coding	54,70	0
ENSG00000068831	RASGRP2	11	64726911	64745456	protein_coding	54,69	0
ENSG00000073150	PANX2	22	50170731	50180294	protein_coding	54,50	0
ENSG00000132581	SDF2	17	28648356	28662189	protein_coding	54,50	0
ENSG00000141750	STAC2	17	39210536	39225872	protein_coding	54,50	0
ENSG00000019169	MARCO	2	118942166	118994660	protein_coding	54,50	0
ENSG00000138185	ENTPD1	10	95711779	95869695	protein_coding	54,49	0
ENSG00000103197	TSC2	16	2047465	2088720	protein_coding	54,46	0
ENSG00000078795	PKD2L2	5	137887968	137942747	protein_coding	54,38	0
ENSG00000275023	MLLT6	17	38705542	38729803	protein_coding	54,37	0
ENSG00000126012	KDM5C	X	53191321	53225422	protein_coding	54,37	0
ENSG00000239306	RBM14	11	66616582	66627347	protein_coding	54,35	0
ENSG00000172824	CES4A	16	66988589	67009758	protein_coding	54,34	0
ENSG00000127578	WFIKKN1	16	629239	634116	protein_coding	54,30	0
ENSG00000158863	FAM160B2	8	22089159	22104898	protein_coding	54,30	0
ENSG00000277363	SRCIN1	17	38530016	38605930	protein_coding	54,29	0
ENSG00000120215	MLANA	9	5890802	5910606	protein_coding	54,20	0
ENSG00000162398	LEXM	1	54806063	54842252	protein_coding	54,20	0
ENSG00000168884	TNIP2	4	2741648	2756376	protein_coding	54,12	0
ENSG00000204472	AIF1	6	31615184	31617021	protein_coding	54,10	0
ENSG00000124370	MCEE	2	71109684	71130239	protein_coding	54,08	0
ENSG00000173852	DPY19L1	7	34928876	35038271	protein_coding	54,03	0
ENSG00000090776	EFNB1	X	68828997	68842147	protein_coding	54,01	0
ENSG00000008838	MED24	17	40019097	40061215	protein_coding	54,00	0
ENSG00000204172	AGAP9	10	47501854	47523638	protein_coding	54,00	0
ENSG00000253846	PCDHGA10	5	141412987	141512979	protein_coding	54,00	0
ENSG00000177700	POLR2L	11	837356	842545	protein_coding	53,99	0
ENSG00000127334	DYRK2	12	67648338	67665406	protein_coding	53,99	0
ENSG00000108439	PNPO	17	47941506	47948288	protein_coding	53,98	0
ENSG00000122026	RPL21	13	27251309	27256691	protein_coding	53,96	0
ENSG00000181085	MAPK15	8	143716259	143722458	protein_coding	53,90	0
ENSG00000139624	CERS5	12	50129306	50167533	protein_coding	53,84	0
ENSG00000196456	ZNF775	7	150368790	150410597	protein_coding	53,84	0
ENSG00000184208	C22orf46	22	41688939	41698136	protein_coding	53,83	0
ENSG00000112425	EPM2A	6	145501583	145736024	protein_coding	53,81	0
ENSG00000277632	CCL3	17	36088256	36090169	protein_coding	53,80	0
ENSG00000100522	GNPNAT1	14	52775194	52791668	protein_coding	53,78	0
ENSG00000086758	HUWE1	X	53532096	53686729	protein_coding	53,77	0
ENSG00000133872	SARAF	8	30063012	30083208	protein_coding	53,76	0
ENSG00000188112	C6orf132	6	42101118	42142619	protein_coding	53,76	0
ENSG00000090661	CERS4	19	8206736	8262421	protein_coding	53,74	0
ENSG00000198133	TMEM229B	14	67447084	67533739	protein_coding	53,71	0
ENSG00000242114	MTFP1	22	30425530	30429053	protein_coding	53,70	0
ENSG00000174840	PDE12	3	57556276	57566844	protein_coding	53,68	0
ENSG00000015479	MATR3	5	139293648	139331359	protein_coding	53,59	0

ENSG00000070669	ASNS	7	97852118	97872542	protein_coding	53,58	0
ENSG00000160352	ZNF714	19	21082159	21125270	protein_coding	53,55	0
ENSG00000153815	CMIP	16	81445170	81711762	protein_coding	53,52	0
ENSG00000161958	FGF11	17	7438273	7444937	protein_coding	53,50	0
ENSG00000188315	C3orf62	3	49268602	49277909	protein_coding	53,41	0
ENSG00000168159	RNF187	1	228487061	228495766	protein_coding	53,40	0
ENSG00000175489	LRRC25	19	18391144	18397617	protein_coding	53,40	0
ENSG00000242689	CNTF	11	58622673	58625733	protein_coding	53,40	0
ENSG00000168679	SLC16A4	1	110362848	110391082	protein_coding	53,40	0
ENSG00000161204	ABCF3	3	184186023	184194012	protein_coding	53,39	0
ENSG00000141391	PRELID3A	18	12407896	12432238	protein_coding	53,31	0
ENSG00000213654	GPSM3	6	32190766	32195523	protein_coding	53,30	0
ENSG00000214300	SPDYE3	7	100307702	100322196	protein_coding	53,27	0
ENSG00000006007	GDE1	16	19501689	19522145	protein_coding	53,26	0
ENSG00000142185	TRPM2	21	44350163	44443081	protein_coding	53,19	0
ENSG00000221890	NPTXR	22	38818452	38843982	protein_coding	53,15	0
ENSG00000167280	ENGASE	17	79074939	79088599	protein_coding	53,13	0
ENSG00000109103	UNC119	17	28546707	28552668	protein_coding	53,11	0
ENSG00000166743	ACSM1	16	20623237	20698890	protein_coding	53,10	0
ENSG00000148795	CYP17A1	10	102830531	102837533	protein_coding	53,10	0
ENSG00000173269	MMRN2	10	86935540	86969481	protein_coding	53,10	0
ENSG00000010626	LRRC23	12	6873569	6914243	protein_coding	53,03	0
ENSG00000139725	RHOF	12	121777754	121803403	protein_coding	53,03	0
ENSG00000117682	DHDDS	1	26432282	26471294	protein_coding	53,01	0
ENSG00000155307	SAMSN1	21	14485228	14583402	protein_coding	53,00	0
ENSG00000029364	SLC39A9	14	69398015	69462388	protein_coding	52,93	0
ENSG00000213339	QTRT1	19	10701430	10713437	protein_coding	52,91	0
ENSG00000023228	NDUFS1	2	206114817	206159603	protein_coding	52,89	0
ENSG00000184925	LCN12	9	136949551	136955497	protein_coding	52,80	0
ENSG00000164107	HAND2	4	173524969	173530229	protein_coding	52,80	0
ENSG00000168032	ENTPD3	3	40387156	40428619	protein_coding	52,80	0
ENSG00000159210	SNF8	17	48929316	48945117	protein_coding	52,76	0
ENSG00000118514	ALDH8A1	6	134917390	134950122	protein_coding	52,70	0
ENSG00000171163	ZNF692	1	248850006	248859144	protein_coding	52,67	0
ENSG00000126217	MCF2L	13	112894378	113099739	protein_coding	52,67	0
ENSG00000087494	PTHLH	12	27958084	27972705	protein_coding	52,66	0
ENSG00000138378	STAT4	2	191029576	191151596	protein_coding	52,60	0
ENSG00000188004	C1orf204	1	159834474	159855347	protein_coding	52,60	0
ENSG00000197568	HHLA3	1	70354805	70385339	protein_coding	52,54	0
ENSG00000183914	DNAH2	17	7717354	7833744	protein_coding	52,33	0
ENSG00000102804	TSC22D1	13	44432143	44577147	protein_coding	52,22	0
ENSG00000111341	MGP	12	14881181	14885926	protein_coding	52,20	0
ENSG00000196968	FUT11	10	73772291	73780251	protein_coding	52,20	0
ENSG00000104447	TRPS1	8	115408496	115809673	protein_coding	52,20	0
ENSG00000277972	CISD3	17	38730235	38735044	protein_coding	52,15	0
ENSG00000128268	MGAT3	22	39457344	39492194	protein_coding	52,10	0
ENSG00000120645	IQSEC3	12	66765	178460	protein_coding	52,10	0
ENSG00000146826	C7orf43	7	100154420	100158715	protein_coding	52,08	0
ENSG00000065361	ERBB3	12	56079857	56103505	protein_coding	52,06	0
ENSG00000153291	SLC25A27	6	46652915	46678193	protein_coding	52,00	0
ENSG00000241404	EGFL8	6	32164583	32168281	protein_coding	52,00	0
ENSG00000178623	GPR35	2	240605431	240631259	protein_coding	52,00	0
ENSG00000197879	MYO1C	17	1464098	1492812	protein_coding	51,97	0
ENSG00000169410	PTPN9	15	75463251	75579289	protein_coding	51,96	0
ENSG00000127241	MASP1	3	187217285	187292022	protein_coding	51,91	0
ENSG00000196754	S100A2	1	153561108	153567890	protein_coding	51,90	0
ENSG00000160685	ZBTB7B	1	155002630	155018522	protein_coding	51,80	0
ENSG00000004776	HSPB6	19	35754568	35758079	protein_coding	51,80	0

ENSG00000108604	SMARCD2	17	63832081	63843065	protein_coding	51,80	0
ENSG00000148834	GSTO1	10	104235356	104267459	protein_coding	51,79	0
ENSG00000063322	MED29	19	39391303	39400637	protein_coding	51,77	0
ENSG00000067225	PKM	15	72199029	72231822	protein_coding	51,74	0
ENSG00000173171	MTX1	1	155208699	155213824	protein_coding	51,71	0
ENSG00000035403	VCL	10	73995193	74121363	protein_coding	51,71	0
ENSG00000168140	VASN	16	4371848	4383528	protein_coding	51,66	0
ENSG00000160741	CRTC2	1	153947669	153958625	protein_coding	51,63	0
ENSG00000106560	GIMAP2	7	150685697	150693641	protein_coding	51,60	0
ENSG00000172831	CES2	16	66934444	66945096	protein_coding	51,54	0
ENSG00000137727	ARHGAP20	11	110577042	110713189	protein_coding	51,47	0
ENSG00000135269	TES	7	116210493	116258783	protein_coding	51,42	0
ENSG00000147883	CDKN2B	9	22002903	22009363	protein_coding	51,36	0
ENSG00000178904	DPY19L3	19	32405543	32485895	protein_coding	51,30	0
ENSG00000084207	GSTP1	11	67583595	67586660	protein_coding	51,28	0
ENSG00000133116	KL	13	33016433	33066145	protein_coding	51,26	0
ENSG00000179698	WDR97	8	144107726	144118315	protein_coding	51,21	0
ENSG00000061455	PRDM6	5	123089121	123194266	protein_coding	51,20	0
ENSG00000187944	C2orf66	2	196805002	196810276	protein_coding	51,20	0
ENSG00000204822	MRPL53	2	74471958	74473322	protein_coding	51,20	0
ENSG00000138448	ITGAV	2	186590065	186680901	protein_coding	51,13	0
ENSG00000118513	MYB	6	135181315	135219173	protein_coding	51,10	0
ENSG00000037897	METTL1	12	57768471	57772793	protein_coding	51,09	0
ENSG00000139651	ZNF740	12	53180700	53195141	protein_coding	51,09	0
ENSG00000143437	ARNT	1	150809705	150876768	protein_coding	51,07	0
ENSG00000174744	BRMS1	11	66337333	66345125	protein_coding	51,05	0
ENSG00000059769	DNAJC25	9	111631352	111654351	protein_coding	50,98	0
ENSG00000244486	SCARF2	22	20424585	20437859	protein_coding	50,90	0
ENSG00000111325	OGFOD2	12	122974580	122980043	protein_coding	50,90	0
ENSG00000181019	NQO1	16	69706996	69726951	protein_coding	50,80	0
ENSG00000114013	CD86	3	122055366	122121139	protein_coding	50,80	0
ENSG00000160613	PCSK7	11	117204337	117232525	protein_coding	50,69	0
ENSG00000089351	GRAMD1A	19	34994784	35026471	protein_coding	50,57	0
ENSG00000079999	KEAP1	19	10486120	10503741	protein_coding	50,52	0
ENSG00000164096	C4orf3	4	119296419	119304445	protein_coding	50,51	0
ENSG00000120705	ETF1	5	138506095	138543300	protein_coding	50,50	0
ENSG00000075461	CACNG4	17	66964910	67033398	protein_coding	50,40	0
ENSG00000152484	USP12	13	27066142	27171896	protein_coding	50,40	0
ENSG00000166887	VPS39	15	42158701	42208316	protein_coding	50,33	0
ENSG00000133104	SPG20	13	36301638	36370180	protein_coding	50,31	0
ENSG00000228594	C1orf233	1	1598012	1600096	protein_coding	50,30	0
ENSG00000037965	HOXC8	12	54009106	54012362	protein_coding	50,30	0
ENSG00000077420	APBB1IP	10	26438203	26567803	protein_coding	50,30	0
ENSG00000186510	CLCNKA	1	16018875	16034050	protein_coding	50,30	0
ENSG00000149091	DGKZ	11	46332905	46380554	protein_coding	50,27	0
ENSG00000142634	EFHD2	1	15409895	15430343	protein_coding	50,21	0
ENSG00000148444	COMMD3	10	22315974	22320308	protein_coding	50,20	0
ENSG00000052749	RRP12	10	97356358	97426076	protein_coding	50,16	0
ENSG00000077238	IL4R	16	27313668	27364778	protein_coding	50,14	0
ENSG00000204257	HLA-DMA	6	32948613	32969094	protein_coding	50,14	0
ENSG00000175376	EIF1AD	11	65996545	66002176	protein_coding	50,08	0
ENSG00000145147	SLIT2	4	20253260	20620561	protein_coding	50,06	0
ENSG00000185513	L3MBTL1	20	43507680	43550950	protein_coding	50,05	0
ENSG00000089327	FXYD5	19	35154730	35169883	protein_coding	50,00	0
ENSG00000114631	PODXL2	3	127629181	127672809	protein_coding	50,00	0
ENSG00000161533	ACOX1	17	75941507	75979363	protein_coding	49,95	0
ENSG00000049239	H6PD	1	9234775	9271337	protein_coding	49,92	0
ENSG00000240583	AQP1	7	30911855	30925516	protein_coding	49,90	0

ENSG00000160014	CALM3	19	46601074	46610793	protein_coding	49,84	0
ENSG00000188707	ZBED6CL	7	150329789	150332721	protein_coding	49,82	0
ENSG00000042832	TG	8	132866958	133134903	protein_coding	49,82	0
ENSG00000101049	SGK2	20	43558968	43588237	protein_coding	49,80	0
ENSG00000185482	STAC3	12	57243453	57251193	protein_coding	49,80	0
ENSG00000150681	RGS18	1	192158457	192185815	protein_coding	49,80	0
ENSG00000197782	ZNF780A	19	40069152	40090938	protein_coding	49,79	0
ENSG00000145191	EIF2B5	3	184135038	184684758	protein_coding	49,75	0
ENSG00000259207	ITGB3	17	47253846	47311816	protein_coding	49,70	0
ENSG00000167566	NCKAP5L	12	49791146	49828750	protein_coding	49,65	0
ENSG00000130052	STARD8	X	68647666	68725842	protein_coding	49,50	0
ENSG00000101638	ST8SIA5	18	46667821	46759257	protein_coding	49,48	0
ENSG00000112640	PPP2R5D	6	42984499	43012342	protein_coding	49,46	0
ENSG00000197442	MAP3K5	6	136557047	136792518	protein_coding	49,44	0
ENSG00000139193	CD27	12	6444867	6451718	protein_coding	49,40	0
ENSG00000266173	STRADA	17	63702832	63741970	protein_coding	49,37	0
ENSG00000176788	BASP1	5	17065598	17276843	protein_coding	49,33	0
ENSG00000204237	OXLD1	17	81665036	81666635	protein_coding	49,33	0
ENSG00000109111	SUPT6H	17	28662091	28702684	protein_coding	49,31	0
ENSG00000186908	ZDHHC17	12	76763588	76853696	protein_coding	49,26	0
ENSG00000129187	DCTD	4	182890060	182917936	protein_coding	49,25	0
ENSG00000143320	CRABP2	1	156699606	156705816	protein_coding	49,21	0
ENSG00000100201	DDX17	22	38483440	38507660	protein_coding	49,17	0
ENSG00000116285	ERRFI1	1	8004404	8026308	protein_coding	49,05	0
ENSG00000170144	HNRNPA3	2	177212563	177223958	protein_coding	48,96	0
ENSG00000178078	STAP2	19	4324043	4342786	protein_coding	48,91	0
ENSG00000185347	C14orf80	14	105489855	105499575	protein_coding	48,91	0
ENSG00000128016	ZFP36	19	39406813	39409412	protein_coding	48,90	0
ENSG00000172197	MBOAT1	6	20102145	20212399	protein_coding	48,90	0
ENSG00000254535	PABPC4L	4	134196333	134201748	protein_coding	48,90	0
ENSG00000103356	EARS2	16	23522014	23557731	protein_coding	48,87	0
ENSG00000169228	RAB24	5	177301198	177303744	protein_coding	48,83	0
ENSG00000115590	IL1R2	2	101991844	102028544	protein_coding	48,80	0
ENSG00000186376	ZNF75D	X	135248920	135344087	protein_coding	48,80	0
ENSG00000123096	SSPN	12	26121991	26299290	protein_coding	48,78	0
ENSG00000259431	THTPA	14	23555988	23560271	protein_coding	48,77	0
ENSG00000169246	NPIPB3	16	21402237	21448567	protein_coding	48,75	0
ENSG00000159921	GNE	9	36214441	36277056	protein_coding	48,74	0
ENSG00000198832	ENSG00000	22	31104772	31120069	protein_coding	48,71	0
ENSG00000197632	SERPINB2	18	63871692	63903890	protein_coding	48,70	0
ENSG00000120896	SORBS3	8	22544986	22575788	protein_coding	48,66	0
ENSG00000143178	TBX19	1	168281040	168314426	protein_coding	48,64	0
ENSG00000104972	LILRB1	19	54617158	54637528	protein_coding	48,60	0
ENSG00000135605	TEC	4	48135783	48269864	protein_coding	48,60	0
ENSG00000174516	PELI3	11	66466327	66477337	protein_coding	48,57	0
ENSG00000136826	KLF4	9	107484852	107490482	protein_coding	48,57	0
ENSG00000109065	NAT9	17	74770547	74776367	protein_coding	48,56	0
ENSG00000170677	SOCS6	18	70288901	70330200	protein_coding	48,54	0
ENSG00000183496	MEX3B	15	82041778	82046141	protein_coding	48,50	0
ENSG00000156097	GPR61	1	109539872	109548406	protein_coding	48,50	0
ENSG00000162704	ARPC5	1	183620846	183635757	protein_coding	48,49	0
ENSG00000164309	CMYA5	5	79689877	79800240	protein_coding	48,32	0
ENSG00000174599	TRAM1L1	4	117083554	117085576	protein_coding	48,20	0
ENSG00000167766	ZNF83	19	52594060	52690496	protein_coding	48,20	0
ENSG00000136870	ZNF189	9	101398873	101410660	protein_coding	48,16	0
ENSG00000165389	SPTSSA	14	34432789	34462356	protein_coding	48,11	0
ENSG00000111886	GABRR2	6	89257208	89315299	protein_coding	48,10	0
ENSG00000205730	ITPRIPL2	16	19113932	19121629	protein_coding	48,00	0

ENSG00000134321	RSAD2	2	6865806	6898239	protein_coding	48,00	0
ENSG00000178927	C17orf62	17	82442589	82450829	protein_coding	47,98	0
ENSG00000100243	CYB5R3	22	42617840	42649568	protein_coding	47,97	0
ENSG00000137270	GCM1	6	53126964	53148829	protein_coding	47,90	0
ENSG00000179364	PACS2	14	105300563	105398147	protein_coding	47,83	0
ENSG00000146858	ZC3HAV1L	7	139025706	139036029	protein_coding	47,81	0
ENSG00000062582	MRPS24	7	43866558	43869893	protein_coding	47,80	0
ENSG00000095319	NUP188	9	128947699	129007096	protein_coding	47,73	0
ENSG00000061918	GUCY1B3	4	155758992	155807591	protein_coding	47,70	0
ENSG00000160695	VPS11	11	119067692	119081978	protein_coding	47,66	0
ENSG00000258227	CLEC5A	7	141927357	141947007	protein_coding	47,60	0
ENSG00000106089	STX1A	7	73699206	73719672	protein_coding	47,50	0
ENSG00000242574	HLA-DMB	6	32934629	32941070	protein_coding	47,50	0
ENSG00000173372	C1QA	1	22636506	22639608	protein_coding	47,50	0
ENSG00000103266	STUB1	16	680224	682870	protein_coding	47,49	0
ENSG00000265808	SEC22B	1	120150898	120176515	protein_coding	47,44	0
ENSG00000187840	EIF4EBP1	8	38030341	38060365	protein_coding	47,43	0
ENSG00000162231	NXF1	11	62792123	62806302	protein_coding	47,42	0
ENSG00000118407	FILIP1	6	75291859	75493738	protein_coding	47,42	0
ENSG00000266967	AARSD1	17	42950526	42964498	protein_coding	47,40	0
ENSG00000151632	AKR1C2	10	4922564	5135226	protein_coding	47,40	0
ENSG00000197070	ARRDC1	9	137605654	137615360	protein_coding	47,36	0
ENSG00000102524	TNFSF13B	13	108251240	108308484	protein_coding	47,30	0
ENSG00000241043	GVQW1	9	32566789	32568621	protein_coding	47,30	0
ENSG00000142784	WDTC1	1	27234516	27308633	protein_coding	47,30	0
ENSG00000197321	SVIL	10	29457338	29736781	protein_coding	47,30	0
ENSG00000173064	HECTD4	12	112160188	112382439	protein_coding	47,29	0
ENSG00000106628	POLD2	7	44114681	44124358	protein_coding	47,26	0
ENSG00000084674	APOB	2	21001429	21044073	protein_coding	47,25	0
ENSG00000141622	RNF165	18	46326809	46463140	protein_coding	47,24	0
ENSG00000187634	SAMD11	1	924880	944581	protein_coding	47,14	0
ENSG00000170558	CDH2	18	27950966	28177446	protein_coding	47,06	0
ENSG00000020129	NCDN	1	35557473	35567274	protein_coding	47,05	0
ENSG00000105220	GPI	19	34359480	34402156	protein_coding	47,05	0
ENSG00000164323	CFAP97	4	185159665	185209504	protein_coding	47,00	0
ENSG00000214753	HNRNPUL2	11	62712630	62727349	protein_coding	46,98	0
ENSG00000139625	MAP3K12	12	53479669	53500063	protein_coding	46,93	0
ENSG00000167470	MIDN	19	1248553	1259140	protein_coding	46,86	0
ENSG00000114030	KPNA1	3	122421949	122514945	protein_coding	46,86	0
ENSG00000124193	SRSF6	20	43457928	43464247	protein_coding	46,84	0
ENSG00000149599	DUSP15	20	31847637	31870747	protein_coding	46,80	0
ENSG00000243232	PCDHAC2	5	140966235	141012344	protein_coding	46,80	0
ENSG00000176720	BOK	2	241558721	241574138	protein_coding	46,77	0
ENSG00000111912	NCOA7	6	125781161	125932030	protein_coding	46,76	0
ENSG00000168209	DDIT4	10	72273920	72276036	protein_coding	46,72	0
ENSG00000146555	SDK1	7	3301448	4269000	protein_coding	46,67	0
ENSG00000108179	PPIF	10	79347469	79355337	protein_coding	46,63	0
ENSG00000105695	MAG	19	35292125	35313804	protein_coding	46,60	0
ENSG00000136286	MYO1G	7	44962662	44979098	protein_coding	46,50	0
ENSG00000155115	GTF3C6	6	110958560	110967890	protein_coding	46,42	0
ENSG00000177383	MAGEF1	3	184710367	184712002	protein_coding	46,41	0
ENSG00000101940	WDR13	X	48590042	48608867	protein_coding	46,40	0
ENSG00000100197	CYP2D6	22	42126499	42130906	protein_coding	46,40	0
ENSG00000101152	DNAJC5	20	63895182	63936031	protein_coding	46,31	0
ENSG00000053501	USE1	19	17215346	17219829	protein_coding	46,27	0
ENSG00000187824	TMEM220	17	10699015	10730316	protein_coding	46,23	0
ENSG00000204149	AGAP6	10	49982190	50010499	protein_coding	46,19	0
ENSG00000060491	OGFR	20	62804835	62814000	protein_coding	46,18	0

ENSG00000180871	CXCR2	2	218125289	218137253	protein_coding	46,09	0
ENSG00000166272	WBP1L	10	102743970	102816267	protein_coding	46,05	0
ENSG00000077157	PPP1R12B	1	202348699	202592706	protein_coding	46,02	0
ENSG00000050628	PTGER3	1	70852353	71047808	protein_coding	46,00	0
ENSG00000136699	SMPD4	2	130151392	130182750	protein_coding	45,99	0
ENSG00000173482	PTPRM	18	7566782	8406861	protein_coding	45,98	0
ENSG00000105321	CCDC9	19	47255980	47271953	protein_coding	45,94	0
ENSG00000154217	PITPNC1	17	67377281	67697261	protein_coding	45,93	0
ENSG00000182885	ADGRG3	16	57668187	57689378	protein_coding	45,80	0
ENSG00000140030	GPR65	14	88005124	88014811	protein_coding	45,80	0
ENSG00000127418	FGFRL1	4	1009936	1026897	protein_coding	45,76	0
ENSG00000111530	CAND1	12	67269281	67319951	protein_coding	45,66	0
ENSG00000063244	U2AF2	19	55654146	55674715	protein_coding	45,64	0
ENSG00000185129	PURA	5	140107777	140125619	protein_coding	45,63	0
ENSG00000138107	ACTR1A	10	102479229	102502711	protein_coding	45,62	0
ENSG00000105483	CARD8	19	48180770	48255946	protein_coding	45,61	0
ENSG00000135929	CYP27A1	2	218781749	218815293	protein_coding	45,60	0
ENSG00000142661	MYOM3	1	24056035	24112175	protein_coding	45,60	0
ENSG00000104853	CLPTM1	19	44954585	44993341	protein_coding	45,58	0
ENSG00000172296	SPTLC3	20	13008979	13169103	protein_coding	45,56	0
ENSG00000108846	ABCC3	17	50634777	50692252	protein_coding	45,54	0
ENSG00000101997	CCDC22	X	49235467	49250526	protein_coding	45,54	0
ENSG00000144230	GPR17	2	127645864	127652639	protein_coding	45,50	0
ENSG00000015532	XYLT2	17	50346092	50363138	protein_coding	45,43	0
ENSG00000149577	SIDT2	11	117178733	117197445	protein_coding	45,36	0
ENSG00000087460	GNAS	20	58839718	58911192	protein_coding	45,36	0
ENSG00000116871	MAP7D1	1	36155579	36180849	protein_coding	45,35	0
ENSG00000141968	VAV1	19	6772714	6857366	protein_coding	45,30	0
ENSG00000258315	C17orf49	17	7014495	7017525	protein_coding	45,30	0
ENSG00000143110	C1orf162	1	111473792	111478512	protein_coding	45,30	0
ENSG00000089472	HEPH	X	66162549	66268867	protein_coding	45,30	0
ENSG00000103742	IGDCC4	15	65381464	65423072	protein_coding	45,26	0
ENSG00000141971	MVB12A	19	17405722	17433724	protein_coding	45,25	0
ENSG00000167535	CACNB3	12	48813794	48828941	protein_coding	45,21	0
ENSG00000005243	COPZ2	17	48026167	48038030	protein_coding	45,20	0
ENSG00000106123	EPHB6	7	142855061	142871094	protein_coding	45,20	0
ENSG00000242802	AP5Z1	7	4775622	4794312	protein_coding	45,15	0
ENSG00000168610	STAT3	17	42313324	42388568	protein_coding	45,12	0
ENSG00000254996	ANKHD1-EIF	5	140401908	140549569	protein_coding	45,10	0
ENSG00000168906	MAT2A	2	85539165	85545280	protein_coding	45,04	0
ENSG00000118162	KPTN	19	47475144	47484268	protein_coding	45,04	0
ENSG00000089041	P2RX7	12	121132819	121188032	protein_coding	45,04	0
ENSG00000130810	PPAN	19	10106289	10111634	protein_coding	45,00	0
ENSG00000135643	KCNMB4	12	70366276	70434292	protein_coding	45,00	0
ENSG00000073067	CYP2W1	7	983199	989640	protein_coding	45,00	0
ENSG00000184117	NIPSNAP1	22	29554808	29581337	protein_coding	44,98	0
ENSG00000204859	ZBTB48	1	6580001	6589280	protein_coding	44,98	0
ENSG00000240682	ISY1	3	129127415	129161293	protein_coding	44,94	0
ENSG00000176401	EID2B	19	39530990	39532854	protein_coding	44,90	0
ENSG00000150281	CTF1	16	30896607	30903560	protein_coding	44,90	0
ENSG00000126767	ELK1	X	47635521	47650604	protein_coding	44,90	0
ENSG00000157353	FUK	16	70454421	70480274	protein_coding	44,89	0
ENSG00000183098	GPC6	13	93226842	94407401	protein_coding	44,81	0
ENSG00000012822	CALCOCO1	12	53708517	53727745	protein_coding	44,80	0
ENSG00000102760	RGCC	13	41457559	41470882	protein_coding	44,79	0
ENSG00000081320	STK17B	2	196133566	196176503	protein_coding	44,77	0
ENSG00000159733	ZFYVE28	4	2269582	2418663	protein_coding	44,76	0
ENSG00000008516	MMP25	16	3046681	3060726	protein_coding	44,71	0

ENSG00000160791	CCR5	3	46370854	46376206	protein_coding	44,70	0
ENSG00000126878	AIF1L	9	131096476	131123152	protein_coding	44,64	0
ENSG00000087269	NOP14	4	2937933	2963385	protein_coding	44,55	0
ENSG00000117640	MTFR1L	1	25818640	25832942	protein_coding	44,55	0
ENSG00000204291	COL15A1	9	98943179	99070792	protein_coding	44,42	0
ENSG00000100258	LMF2	22	50502949	50507691	protein_coding	44,37	0
ENSG00000173641	HSPB7	1	16014028	16019594	protein_coding	44,30	0
ENSG00000161091	MFSD12	19	3538261	3574290	protein_coding	44,22	0
ENSG00000100296	THOC5	22	29505879	29555216	protein_coding	44,20	0
ENSG00000174871	CNIH2	11	66278190	66285301	protein_coding	44,20	0
ENSG00000113048	MRPS27	5	72219409	72320646	protein_coding	44,17	0
ENSG00000086015	MAST2	1	45786987	46036124	protein_coding	44,16	0
ENSG00000105397	TYK2	19	10350529	10380676	protein_coding	44,14	0
ENSG00000206172	HBA1	16	176680	177522	protein_coding	44,10	0
ENSG00000146830	GIGYF1	7	100679507	100689448	protein_coding	44,09	0
ENSG00000115977	AAK1	2	69457997	69674349	protein_coding	44,01	0
ENSG00000103522	IL21R	16	27402162	27452042	protein_coding	44,00	0
ENSG00000134256	CD101	1	117001750	117036476	protein_coding	44,00	0
ENSG00000125734	GPR108	19	6729914	6737603	protein_coding	43,99	0
ENSG00000135069	PSAT1	9	78297143	78330093	protein_coding	43,97	0
ENSG00000129646	QRICH2	17	76274049	76307680	protein_coding	43,96	0
ENSG00000222046	DCDC2B	1	32209094	32216196	protein_coding	43,94	0
ENSG00000011422	PLAUR	19	43646095	43670547	protein_coding	43,92	0
ENSG00000215717	TMEM167B	1	109089803	109096934	protein_coding	43,90	0
ENSG00000135842	FAM129A	1	184790724	184974550	protein_coding	43,88	0
ENSG00000112081	SRSF3	6	36594353	36605600	protein_coding	43,86	0
ENSG00000110660	SLC35F2	11	107790991	107928293	protein_coding	43,83	0
ENSG00000162738	VANGL2	1	160400586	160428678	protein_coding	43,76	0
ENSG00000142453	CARM1	19	10871513	10923070	protein_coding	43,75	0
ENSG00000163960	UBXN7	3	196347662	196432474	protein_coding	43,75	0
ENSG00000152076	CCDC74B	2	130139287	130145134	protein_coding	43,70	0
ENSG00000142875	PRKACB	1	84078062	84238498	protein_coding	43,66	0
ENSG00000180354	MTURN	7	30134810	30162762	protein_coding	43,65	0
ENSG00000107175	CREB3	9	35732320	35737007	protein_coding	43,63	0
ENSG00000158805	ZNF276	16	89720400	89740903	protein_coding	43,54	0
ENSG00000144357	UBR3	2	169827458	170084131	protein_coding	43,53	0
ENSG00000133985	TTC9	14	70641787	70675360	protein_coding	43,51	0
ENSG00000140044	JDP2	14	75427716	75474111	protein_coding	43,48	0
ENSG00000163626	COX18	4	73052362	73069755	protein_coding	43,38	0
ENSG00000033011	ALG1	16	5033960	5087379	protein_coding	43,35	0
ENSG00000009724	MASP2	1	11026523	11047233	protein_coding	43,30	0
ENSG00000110079	MS4A4A	11	60280541	60308972	protein_coding	43,20	0
ENSG00000154096	THY1	11	119417378	119424985	protein_coding	43,20	0
ENSG00000181789	COPG1	3	129249606	129277773	protein_coding	43,17	0
ENSG00000030419	IKZF2	2	212999691	213152427	protein_coding	43,16	0
ENSG00000126603	GLIS2	16	4314761	4339597	protein_coding	43,12	0
ENSG00000137343	ATAT1	6	30626842	30646823	protein_coding	43,11	0
ENSG00000139637	C12orf10	12	53299686	53307177	protein_coding	43,11	0
ENSG00000186010	NDUFA13	19	19515736	19529054	protein_coding	43,10	0
ENSG00000152582	SPEF2	5	35617844	35814611	protein_coding	43,10	0
ENSG00000196172	ZNF681	19	23739195	23758891	protein_coding	43,05	0
ENSG00000179855	GIPC3	19	3585553	3593541	protein_coding	43,00	0
ENSG00000072210	ALDH3A2	17	19648136	19677598	protein_coding	43,00	0
ENSG00000174938	SEZ6L2	16	29871159	29899547	protein_coding	42,99	0
ENSG00000158716	DUSP23	1	159780932	159782543	protein_coding	42,96	0
ENSG00000124574	ABCC10	6	43427366	43450430	protein_coding	42,94	0
ENSG00000244187	TMEM141	9	136791355	136793257	protein_coding	42,94	0
ENSG00000099937	SERPIND1	22	20773879	20787720	protein_coding	42,90	0

ENSG00000139209	SLC38A4	12	46764761	46832408	protein_coding	42,90	0
ENSG00000147036	LANCL3	X	37571569	37684463	protein_coding	42,82	0
ENSG00000118046	STK11	19	1177558	1228435	protein_coding	42,81	0
ENSG00000116761	CTH	1	70411218	70439851	protein_coding	42,80	0
ENSG00000103269	RHBDL1	16	675666	678268	protein_coding	42,80	0
ENSG00000110931	CAMKK2	12	121237691	121298308	protein_coding	42,80	0
ENSG00000140391	TSPAN3	15	77041404	77083984	protein_coding	42,79	0
ENSG00000067167	TRAM1	8	70573442	70608387	protein_coding	42,77	0
ENSG00000110900	TSPAN11	12	30926428	30996599	protein_coding	42,77	0
ENSG00000126070	AGO3	1	35930718	36072500	protein_coding	42,71	0
ENSG00000188559	RALGAPA2	20	20389552	20712488	protein_coding	42,70	0
ENSG00000103671	TRIP4	15	64387748	64455303	protein_coding	42,70	0
ENSG00000188848	BEND4	4	42110938	42152878	protein_coding	42,70	0
ENSG00000164733	CTSB	8	11842524	11869448	protein_coding	42,67	0
ENSG00000122481	RWDD3	1	95234155	95247225	protein_coding	42,62	0
ENSG00000100280	AP1B1	22	29327680	29423179	protein_coding	42,60	0
ENSG00000100288	CHKB	22	50578949	50601455	protein_coding	42,60	0
ENSG00000171219	CDC42BPG	11	64823387	64844569	protein_coding	42,60	0
ENSG00000120697	ALG5	13	36949775	37000261	protein_coding	42,59	0
ENSG00000123739	PLA2G12A	4	109709989	109730077	protein_coding	42,58	0
ENSG00000169045	HNRNPH1	5	179614178	179634784	protein_coding	42,57	0
ENSG00000123992	DNPEP	2	219373546	219400022	protein_coding	42,55	0
ENSG00000120686	UFM1	13	38349849	38363619	protein_coding	42,50	0
ENSG00000073803	MAP3K13	3	185282941	185489097	protein_coding	42,46	0
ENSG00000108947	EFNB3	17	7705202	7711378	protein_coding	42,43	0
ENSG00000171049	FPR2	19	51752026	51770526	protein_coding	42,40	0
ENSG00000213139	CRYGS	3	186538441	186546702	protein_coding	42,40	0
ENSG00000102032	RENBP	X	153935263	153944691	protein_coding	42,40	0
ENSG00000139517	LNX2	13	27545911	27620404	protein_coding	42,40	0
ENSG00000071894	CPSF1	8	144393229	144409349	protein_coding	42,39	0
ENSG00000122566	HNRNPA2B1	7	26189927	26201529	protein_coding	42,31	0
ENSG00000105771	SMG9	19	43727992	43754990	protein_coding	42,27	0
ENSG00000176658	MYO1D	17	32492522	32877177	protein_coding	42,26	0
ENSG00000175768	TOMM5	9	37582646	37592642	protein_coding	42,25	0
ENSG00000189184	PCDH18	4	137518918	137532494	protein_coding	42,20	0
ENSG00000174004	NRROS	3	196639686	196662004	protein_coding	42,20	0
ENSG00000067840	PDZD4	X	153802166	153830565	protein_coding	42,20	0
ENSG00000107021	TBC1D13	9	128787204	128810432	protein_coding	42,15	0
ENSG00000146250	PRSS35	6	83512538	83525704	protein_coding	42,14	0
ENSG00000145945	FAM50B	6	3849386	3851317	protein_coding	42,13	0
ENSG00000166793	YPEL4	11	57645087	57649944	protein_coding	42,10	0
ENSG00000175463	TBC1D10C	11	67403915	67410089	protein_coding	42,10	0
ENSG00000172738	TMEM217	6	37212180	37258155	protein_coding	42,00	0
ENSG00000162144	CYB561A3	11	61348745	61362299	protein_coding	41,99	0
ENSG00000131503	ANKHD1	5	140401814	140539856	protein_coding	41,92	0
ENSG00000241839	PLEKHO2	15	64841883	64868007	protein_coding	41,91	0
ENSG00000137218	FRS3	6	41770176	41786542	protein_coding	41,91	0
ENSG00000091106	NLRC4	2	32224453	32265854	protein_coding	41,90	0
ENSG00000155761	SPAG17	1	117953861	118185223	protein_coding	41,90	0
ENSG00000181396	OGFOD3	17	82389223	82418637	protein_coding	41,88	0
ENSG00000170961	HAS2	8	121612116	121641390	protein_coding	41,88	0
ENSG00000005448	WDR54	2	74421678	74425755	protein_coding	41,83	0
ENSG00000196458	ZNF605	12	132918308	132956306	protein_coding	41,79	0
ENSG00000177076	ACER2	9	19409059	19452020	protein_coding	41,76	0
ENSG00000152952	PLOD2	3	146069440	146163653	protein_coding	41,76	0
ENSG00000099203	TMED1	19	10832438	10836318	protein_coding	41,73	0
ENSG00000105447	GRWD1	19	48445773	48457022	protein_coding	41,65	0
ENSG00000103326	CAPN15	16	527717	554636	protein_coding	41,61	0

ENSG00000181016	LSMEM1	7	112480853	112491062	protein_coding	41,60	0
ENSG00000273802	HIST1H2BG	6	26215159	26216692	protein_coding	41,60	0
ENSG00000168066	SF1	11	64764606	64778786	protein_coding	41,59	0
ENSG00000104859	CLASRP	19	45039040	45070956	protein_coding	41,59	0
ENSG00000108654	DDX5	17	64499616	64508199	protein_coding	41,58	0
ENSG00000153485	TMEM251	14	93184951	93187089	protein_coding	41,58	0
ENSG00000162545	CAMK2N1	1	20482391	20486220	protein_coding	41,57	0
ENSG00000149930	TAOK2	16	29973641	29992261	protein_coding	41,53	0
ENSG00000008394	MGST1	12	16347142	16609259	protein_coding	41,52	0
ENSG00000180089	TMEM86B	19	55226639	55229264	protein_coding	41,50	0
ENSG00000164932	CTHRC1	8	103371515	103382997	protein_coding	41,50	0
ENSG00000134201	GSTM5	1	109712255	109775428	protein_coding	41,50	0
ENSG00000007516	BAIAP3	16	1333601	1349441	protein_coding	41,47	0
ENSG00000167004	PDIA3	15	43746392	43773279	protein_coding	41,42	0
ENSG00000087995	METTL2A	17	62423867	62450822	protein_coding	41,39	0
ENSG00000100926	TM9SF1	14	24189143	24195687	protein_coding	41,37	0
ENSG00000106397	PLOD3	7	101205977	101218420	protein_coding	41,36	0
ENSG00000104856	RELB	19	45001430	45038198	protein_coding	41,35	0
ENSG00000164877	MICALL2	7	1428465	1459502	protein_coding	41,30	0
ENSG00000187372	PCDHB13	5	141213919	141218979	protein_coding	41,21	0
ENSG00000186326	RGS9BP	19	32675407	32678300	protein_coding	41,21	0
ENSG00000066382	MPPED2	11	30384493	30586872	protein_coding	41,20	0
ENSG00000198585	NUDT16	3	131381671	131388830	protein_coding	41,17	0
ENSG00000140262	TCF12	15	56918623	57299281	protein_coding	41,11	0
ENSG00000154258	ABCA9	17	68974488	69061064	protein_coding	41,03	0
ENSG00000160200	CBS	21	43053191	43076943	protein_coding	41,00	0
ENSG00000182240	BACE2	21	41167801	41282518	protein_coding	40,99	0
ENSG00000134569	LRP4	11	46856868	46918642	protein_coding	40,94	0
ENSG00000071626	DAZAP1	19	1407569	1435687	protein_coding	40,93	0
ENSG00000183186	C2CD4C	19	405438	409170	protein_coding	40,90	0
ENSG00000127870	RNF6	13	26132115	26222493	protein_coding	40,90	0
ENSG00000027697	IFNGR1	6	137197484	137219449	protein_coding	40,86	0
ENSG00000144036	EXOC6B	2	72175984	72826041	protein_coding	40,86	0
ENSG00000205133	TRIQK	8	92883530	93017673	protein_coding	40,76	0
ENSG00000187624	C17orf97	17	410327	431062	protein_coding	40,75	0
ENSG00000127580	WDR24	16	684622	690444	protein_coding	40,73	0
ENSG00000126262	FFAR2	19	35443907	35451767	protein_coding	40,70	0
ENSG00000042445	RETSAT	2	85342088	85354620	protein_coding	40,67	0
ENSG00000145861	C1QTNF2	5	160347751	160370641	protein_coding	40,67	0
ENSG00000204444	APOM	6	31652416	31658210	protein_coding	40,66	0
ENSG00000182154	MRPL41	9	137551199	137552555	protein_coding	40,64	0
ENSG00000158552	ZFAND2B	2	219195237	219209651	protein_coding	40,63	0
ENSG00000143952	VPS54	2	63892146	64019072	protein_coding	40,60	0
ENSG00000164867	NOS3	7	150990995	151014588	protein_coding	40,58	0
ENSG00000152784	PRDM8	4	80183879	80204329	protein_coding	40,57	0
ENSG00000137478	FCHSD2	11	72836745	73142261	protein_coding	40,56	0
ENSG00000178718	RPP25	15	74954416	74957464	protein_coding	40,50	0
ENSG00000113209	PCDHB5	5	141135218	141138625	protein_coding	40,50	0
ENSG00000173369	C1QB	1	22652762	22661538	protein_coding	40,50	0
ENSG00000114423	CBLB	3	105655461	105869552	protein_coding	40,49	0
ENSG00000185386	MAPK11	22	50263713	50270767	protein_coding	40,40	0
ENSG00000080493	SLC4A4	4	71187286	71572087	protein_coding	40,40	0
ENSG00000161021	MAML1	5	179732850	179796511	protein_coding	40,38	0
ENSG00000146733	PSPH	7	56011051	56051604	protein_coding	40,38	0
ENSG00000112739	PRPF4B	6	4021267	4064983	protein_coding	40,36	0
ENSG00000141956	PRDM15	21	41798225	41879482	protein_coding	40,35	0
ENSG00000148343	FAM73B	9	129036621	129072082	protein_coding	40,35	0
ENSG00000186567	CEACAM19	19	44662278	44684359	protein_coding	40,30	0

ENSG00000169418	NPR1	1	153678637	153693992	protein_coding	40,30	0
ENSG00000129250	KIF1C	17	4997948	5028401	protein_coding	40,29	0
ENSG00000144746	ARL6IP5	3	69084944	69106066	protein_coding	40,27	0
ENSG00000138336	TET1	10	68560656	68694482	protein_coding	40,25	0
ENSG00000139631	CSAD	12	53157663	53180909	protein_coding	40,23	0
ENSG00000162585	FAAP20	1	2184461	2212720	protein_coding	40,22	0
ENSG00000203485	INF2	14	104689606	104722535	protein_coding	40,19	0
ENSG00000132481	TRIM47	17	75874161	75878575	protein_coding	40,12	0
ENSG00000167967	E4F1	16	2223566	2235742	protein_coding	40,11	0
ENSG00000149564	ESAM	11	124752583	124762290	protein_coding	40,10	0
ENSG00000134287	ARF3	12	48935723	48957551	protein_coding	40,05	0
ENSG00000023902	PLEKHO1	1	150149183	150164720	protein_coding	39,97	0
ENSG00000125818	PSMF1	20	1113263	1189415	protein_coding	39,90	0
ENSG00000187116	LILRA5	19	54307070	54313139	protein_coding	39,90	0
ENSG00000151062	CACNA2D4	12	1791957	1918836	protein_coding	39,90	0
ENSG00000188931	CFAP126	1	161364731	161367874	protein_coding	39,89	0
ENSG00000093144	ECHDC1	6	127288710	127343609	protein_coding	39,87	0
ENSG00000124181	PLCG1	20	41136960	41196801	protein_coding	39,83	0
ENSG00000139908	TSSK4	14	24205697	24208362	protein_coding	39,80	0
ENSG00000186642	PDE2A	11	72576141	72674591	protein_coding	39,80	0
ENSG00000142959	BEST4	1	44783585	44787705	protein_coding	39,80	0
ENSG00000010278	CD9	12	6199715	6238271	protein_coding	39,78	0
ENSG00000125846	ZNF133	20	18288283	18316996	protein_coding	39,74	0
ENSG00000141551	CSNK1D	17	82239023	82273731	protein_coding	39,74	0
ENSG00000156671	SAMD8	10	75099586	75182123	protein_coding	39,71	0
ENSG00000021461	CYP3A43	7	99828013	99866102	protein_coding	39,70	0
ENSG00000125912	NCLN	19	3185563	3209575	protein_coding	39,65	0
ENSG00000124257	NEURL2	20	45888625	45891287	protein_coding	39,60	0
ENSG00000188305	C19orf35	19	2274622	2282176	protein_coding	39,60	0
ENSG00000171161	ZNF672	1	248838210	248849517	protein_coding	39,58	0
ENSG00000141582	CBX4	17	79833156	79839429	protein_coding	39,54	0
ENSG00000100365	NCF4	22	36860988	36878015	protein_coding	39,50	0
ENSG00000177000	MTHFR	1	11785723	11806920	protein_coding	39,49	0
ENSG00000068383	INPP5A	10	132537820	132783480	protein_coding	39,44	0
ENSG00000250565	ATP6V1E2	2	46490750	46542557	protein_coding	39,44	0
ENSG00000130811	EIF3G	19	10115017	10119918	protein_coding	39,42	0
ENSG00000100100	PIK3IP1	22	31281593	31292534	protein_coding	39,39	0
ENSG00000167705	RILP	17	1646145	1650077	protein_coding	39,38	0
ENSG00000141084	RANBP10	16	67723066	67806652	protein_coding	39,38	0
ENSG00000101346	POFUT1	20	32207880	32238667	protein_coding	39,38	0
ENSG00000165898	ISCA2	14	74493720	74497106	protein_coding	39,37	0
ENSG00000168397	ATG4B	2	241637213	241673857	protein_coding	39,31	0
ENSG00000184730	APOBR	16	28494649	28498970	protein_coding	39,30	0
ENSG00000130725	UBE2M	19	58555712	58558960	protein_coding	39,26	0
ENSG00000002330	BAD	11	64269830	64284704	protein_coding	39,24	0
ENSG00000156150	ALX3	1	110059994	110070700	protein_coding	39,21	0
ENSG00000177989	ODF3B	22	50529710	50532580	protein_coding	39,20	0
ENSG00000105609	LILRB5	19	54249431	54257301	protein_coding	39,20	0
ENSG00000197599	CCDC154	16	1434383	1444556	protein_coding	39,20	0
ENSG00000184304	PRKD1	14	29576479	30191898	protein_coding	39,20	0
ENSG00000126733	DACH2	X	86148458	86832604	protein_coding	39,20	0
ENSG00000100916	BRMS1L	14	35826318	35932325	protein_coding	39,19	0
ENSG00000169740	ZNF32	10	43643859	43648856	protein_coding	39,14	0
ENSG00000117419	ERI3	1	44221070	44355260	protein_coding	39,13	0
ENSG00000136718	IMP4	2	130342225	130347810	protein_coding	39,13	0
ENSG00000184898	RBM43	2	151247940	151261879	protein_coding	39,07	0
ENSG00000107882	SUFU	10	102503987	102633535	protein_coding	39,02	0
ENSG00000110852	CLEC2B	12	9852984	9870136	protein_coding	39,00	0

ENSG00000198722	UNC13B	9	35161992	35405338	protein_coding	38,97	0
ENSG00000119866	BCL11A	2	60451167	60553567	protein_coding	38,94	0
ENSG00000169682	SPNS1	16	28974221	28984548	protein_coding	38,90	0
ENSG00000111110	PPM1H	12	62643982	62935037	protein_coding	38,84	0
ENSG00000174586	ZNF497	19	58354357	58362848	protein_coding	38,84	0
ENSG00000280071	ENSG00000	21	5079294	5128425	protein_coding	38,83	0
ENSG00000254827	SLC22A18AS	11	2887780	2903740	protein_coding	38,80	0
ENSG00000105248	CCDC94	19	4247079	4269090	protein_coding	38,80	0
ENSG00000110400	PVRL1	11	119623408	119729084	protein_coding	38,79	0
ENSG00000119318	RAD23B	9	107283137	107332194	protein_coding	38,78	0
ENSG00000188566	NDOR1	9	137205670	137217009	protein_coding	38,73	0
ENSG00000168612	ZSWIM1	20	45881227	45885266	protein_coding	38,73	0
ENSG00000057294	PKP2	12	32790745	32896840	protein_coding	38,72	0
ENSG00000123684	LPGAT1	1	211743457	211830772	protein_coding	38,72	0
ENSG00000101098	RIMS4	20	44751808	44810338	protein_coding	38,72	0
ENSG00000165152	TMEM246	9	101473171	101533537	protein_coding	38,69	0
ENSG00000198841	KTI12	1	52032103	52033816	protein_coding	38,66	0
ENSG00000197785	ATAD3A	1	1512151	1534687	protein_coding	38,65	0
ENSG00000120256	LRP11	6	149818798	149864026	protein_coding	38,64	0
ENSG00000160179	ABCG1	21	42199689	42297244	protein_coding	38,60	0
ENSG00000099622	CIRBP	19	1259384	1274880	protein_coding	38,49	0
ENSG00000148300	REXO4	9	133406059	133418096	protein_coding	38,48	0
ENSG00000082898	XPO1	2	61477849	61538626	protein_coding	38,42	0
ENSG00000144867	SRPRB	3	133784033	133825772	protein_coding	38,42	0
ENSG00000176533	GNG7	19	2511219	2702709	protein_coding	38,42	0
ENSG00000205765	C5orf51	5	41904188	41921636	protein_coding	38,38	0
ENSG00000065970	FOXJ2	12	8032703	8055503	protein_coding	38,37	0
ENSG00000242715	CCDC169	13	36222008	36297840	protein_coding	38,36	0
ENSG00000123636	BAZ2B	2	159318979	159616692	protein_coding	38,30	0
ENSG00000090539	CHRD	3	184380073	184390736	protein_coding	38,30	0
ENSG00000144488	ESPNL	2	238100157	238133287	protein_coding	38,30	0
ENSG00000170802	FOXN2	2	48314637	48379294	protein_coding	38,29	0
ENSG00000196365	LONP1	19	5691834	5720572	protein_coding	38,27	0
ENSG00000184786	TCTE3	6	169740114	169751587	protein_coding	38,23	0
ENSG00000165092	ALDH1A1	9	72900662	73080442	protein_coding	38,20	0
ENSG00000154451	GBP5	1	89258950	89272804	protein_coding	38,20	0
ENSG00000112697	TMEM30A	6	75252924	75284968	protein_coding	38,17	0
ENSG00000127663	KDM4B	19	4969113	5153595	protein_coding	38,14	0
ENSG00000137133	HINT2	9	35812960	35815354	protein_coding	38,12	0
ENSG00000105245	NUMBL	19	40665905	40690972	protein_coding	38,10	0
ENSG00000206538	VGLL3	3	86937969	86991119	protein_coding	38,10	0
ENSG00000072682	P4HA2	5	132191838	132295315	protein_coding	38,09	0
ENSG00000151414	NEK7	1	198156963	198322420	protein_coding	38,08	0
ENSG00000127483	HP1BP3	1	20742661	20787323	protein_coding	38,06	0
ENSG00000161281	COX7A1	19	36150922	36152869	protein_coding	38,00	0
ENSG00000161664	ASB16	17	44170447	44179083	protein_coding	38,00	0
ENSG00000137752	CASP1	11	105025443	105035250	protein_coding	38,00	0
ENSG00000188573	FBLL1	5	168529116	168530634	protein_coding	38,00	0
ENSG00000144908	ALDH1L1	3	126103562	126197994	protein_coding	38,00	0
ENSG00000244482	LILRA6	19	54236592	54242791	protein_coding	37,90	0
ENSG00000214530	STARD10	11	72754729	72794168	protein_coding	37,87	0
ENSG00000145949	MYLK4	6	2663629	2750966	protein_coding	37,83	0
ENSG00000015285	WAS	X	48676596	48691427	protein_coding	37,80	0
ENSG00000123562	MORF4L2	X	103675496	103688158	protein_coding	37,80	0
ENSG00000167658	EEF2	19	3976056	3985469	protein_coding	37,75	0
ENSG00000103496	STX4	16	31032889	31042975	protein_coding	37,74	0
ENSG00000112796	ENPP5	6	46159187	46170971	protein_coding	37,71	0
ENSG00000165887	ANKRD2	10	97572499	97583884	protein_coding	37,70	0

ENSG00000204344	STK19	6	31971091	31982821	protein_coding	37,69	0
ENSG00000186431	FCAR	19	54874248	54890472	protein_coding	37,60	0
ENSG00000152270	PDE3B	11	14643723	14872044	protein_coding	37,60	0
ENSG00000128617	OPN1SW	7	128772491	128775790	protein_coding	37,60	0
ENSG00000204386	NEU1	6	31857659	31862906	protein_coding	37,59	0
ENSG00000120254	MTHFD1L	6	150865549	151101887	protein_coding	37,57	0
ENSG00000135632	SMYD5	2	73214222	73227237	protein_coding	37,51	0
ENSG00000028203	VEZT	12	95217746	95302790	protein_coding	37,51	0
ENSG00000105202	FBL	19	39834458	39846414	protein_coding	37,50	0
ENSG00000196911	KPNA5	6	116681187	116741866	protein_coding	37,50	0
ENSG00000110880	CORO1C	12	108645109	108731596	protein_coding	37,50	0
ENSG00000169733	RFNG	17	82047902	82051831	protein_coding	37,49	0
ENSG00000277258	PCGF2	17	38733897	38749817	protein_coding	37,47	0
ENSG00000214253	FIS1	7	101239458	101252316	protein_coding	37,43	0
ENSG00000114120	SLC25A36	3	140941830	140979933	protein_coding	37,41	0
ENSG00000253873	PCDHGA11	5	141421047	141512979	protein_coding	37,40	0
ENSG00000204851	PNMAL2	19	46486906	46496498	protein_coding	37,36	0
ENSG00000112319	EYA4	6	133240598	133532120	protein_coding	37,35	0
ENSG00000101558	VAPA	18	9914002	9960021	protein_coding	37,34	0
ENSG00000174483	BBS1	11	66510606	66533627	protein_coding	37,31	0
ENSG00000163814	CDCP1	3	45082278	45146422	protein_coding	37,29	0
ENSG00000163431	LMOD1	1	201896452	201946588	protein_coding	37,20	0
ENSG00000171222	SCAND1	20	35953617	35959472	protein_coding	37,11	0
ENSG00000198910	L1CAM	X	153861514	153909223	protein_coding	37,10	0
ENSG00000081377	CDC14B	9	96490241	96619830	protein_coding	37,08	0
ENSG00000257315	ZBED6	1	203796309	203800558	protein_coding	37,03	0
ENSG00000197363	ZNF517	8	144798876	144811169	protein_coding	37,01	0
ENSG00000146192	FGD2	6	37005646	37029070	protein_coding	37,00	0
ENSG00000182324	KCNJ14	19	48455509	48466980	protein_coding	36,96	0
ENSG00000186889	TMEM17	2	62500221	62511894	protein_coding	36,93	0
ENSG00000105679	GAPDHS	19	35533412	35545316	protein_coding	36,90	0
ENSG00000136807	CDK9	9	127785679	127790787	protein_coding	36,84	0
ENSG00000181444	ZNF467	7	149764182	149773479	protein_coding	36,82	0
ENSG00000111906	HDDC2	6	125219962	125302078	protein_coding	36,82	0
ENSG00000128272	ATF4	22	39519695	39522685	protein_coding	36,79	0
ENSG00000080189	SLC35C2	20	46345980	46364458	protein_coding	36,77	0
ENSG00000142186	SCYL1	11	65525077	65538704	protein_coding	36,75	0
ENSG00000179912	R3HDM2	12	57253762	57431005	protein_coding	36,72	0
ENSG00000197608	ZNF841	19	52064466	52095765	protein_coding	36,70	0
ENSG00000113621	TXNDC15	5	134873803	134901525	protein_coding	36,67	0
ENSG00000038002	AGA	4	177430770	177442503	protein_coding	36,63	0
ENSG00000105559	PLEKHA4	19	48837097	48868632	protein_coding	36,62	0
ENSG00000087076	HSD17B14	19	48813017	48836678	protein_coding	36,61	0
ENSG00000170835	CEL	9	133061978	133087355	protein_coding	36,60	0
ENSG00000165061	ZMAT4	8	40530590	40897833	protein_coding	36,60	0
ENSG00000027847	B4GALT7	5	177600100	177610347	protein_coding	36,56	0
ENSG00000163738	MTHFD2L	4	74114174	74303099	protein_coding	36,55	0
ENSG00000152467	ZSCAN1	19	58034032	58054631	protein_coding	36,55	0
ENSG00000119616	FCF1	14	74713144	74738620	protein_coding	36,54	0
ENSG00000111087	GLI1	12	57460135	57472262	protein_coding	36,50	0
ENSG00000133574	GIMAP4	7	150567277	150573955	protein_coding	36,50	0
ENSG00000101158	NELFCD	20	58981208	58995133	protein_coding	36,49	0
ENSG00000136631	VPS45	1	150067293	150145327	protein_coding	36,47	0
ENSG00000138380	CARF	2	202912214	202987063	protein_coding	36,45	0
ENSG00000077463	SIRT6	19	4174109	4182604	protein_coding	36,44	0
ENSG00000158669	GPAT4	8	41577187	41625001	protein_coding	36,42	0
ENSG00000184716	SERINC4	15	43794162	43800221	protein_coding	36,37	0
ENSG00000127990	SGCE	7	94585230	94656209	protein_coding	36,36	0

ENSG00000244617	ASPRV1	2	69960089	69962265	protein_coding	36,30	0
ENSG00000077684	JADE1	4	128809623	128875224	protein_coding	36,29	0
ENSG00000160117	ANKLE1	19	17281645	17287646	protein_coding	36,27	0
ENSG00000135924	DNAJB2	2	219279267	219286900	protein_coding	36,25	0
ENSG00000145362	ANK2	4	112818109	113383740	protein_coding	36,23	0
ENSG00000108774	RAB5C	17	42124976	42155044	protein_coding	36,22	0
ENSG00000007080	CCDC124	19	17933016	17943991	protein_coding	36,15	0
ENSG00000184887	BTBD6	14	105248490	105251093	protein_coding	36,14	0
ENSG00000113532	ST8SIA4	5	100806935	100903266	protein_coding	36,12	0
ENSG00000101363	MANBAL	20	37289638	37317260	protein_coding	36,12	0
ENSG00000177380	PPFIA3	19	49119389	49151026	protein_coding	36,09	0
ENSG00000136371	MTHFS	15	79833585	79897379	protein_coding	36,09	0
ENSG00000004142	POLDIP2	17	28347177	28357522	protein_coding	36,09	0
ENSG00000167595	PROSER3	19	35758143	35771028	protein_coding	36,07	0
ENSG00000143412	ANXA9	1	150982017	150995634	protein_coding	36,00	0
ENSG00000170425	ADORA2B	17	15944917	15975746	protein_coding	35,96	0
ENSG00000166444	ST5	11	8693351	8910951	protein_coding	35,95	0
ENSG00000157764	BRAF	7	140719327	140924764	protein_coding	35,91	0
ENSG00000133321	RARRES3	11	63536809	63546462	protein_coding	35,91	0
ENSG00000117091	CD48	1	160678746	160711851	protein_coding	35,90	0
ENSG00000105325	FZR1	19	3506273	3538330	protein_coding	35,90	0
ENSG00000113070	HBEGF	5	140332843	140346631	protein_coding	35,86	0
ENSG00000164053	ATRIP	3	48446710	48465716	protein_coding	35,85	0
ENSG00000169230	PRELID1	5	177303774	177306959	protein_coding	35,80	0
ENSG00000173068	BNC2	9	16409503	16870843	protein_coding	35,76	0
ENSG00000064995	TAF11	6	34877778	34888089	protein_coding	35,72	0
ENSG00000105976	MET	7	116672390	116798386	protein_coding	35,71	0
ENSG00000178741	COX5A	15	74919791	74938168	protein_coding	35,71	0
ENSG00000125845	BMP2	20	6767664	6780280	protein_coding	35,70	0
ENSG00000198829	SUCNR1	3	151873643	151884619	protein_coding	35,70	0
ENSG00000181555	SETD2	3	47016429	47163967	protein_coding	35,69	0
ENSG00000167994	RAB3IL1	11	61897301	61920269	protein_coding	35,68	0
ENSG00000112624	GLTSCR1L	6	42746958	42868560	protein_coding	35,68	0
ENSG00000171604	CXXC5	5	139647299	139683882	protein_coding	35,66	0
ENSG00000035862	TIMP2	17	78852977	78925387	protein_coding	35,64	0
ENSG00000178922	HYI	1	43451003	43453989	protein_coding	35,63	0
ENSG00000198917	C9orf114	9	128819651	128829821	protein_coding	35,60	0
ENSG00000177453	NIM1K	5	43192071	43280850	protein_coding	35,60	0
ENSG00000091879	ANGPT2	8	6499651	6563409	protein_coding	35,56	0
ENSG00000105538	RASIP1	19	48720587	48740721	protein_coding	35,56	0
ENSG00000159399	HK2	2	74833981	74893359	protein_coding	35,55	0
ENSG00000146904	EPHA1	7	143390289	143408892	protein_coding	35,50	0
ENSG00000148362	C9orf142	9	136992418	136993984	protein_coding	35,45	0
ENSG00000163251	FZD5	2	207762586	207769563	protein_coding	35,45	0
ENSG00000100372	SLC25A17	22	40769630	40819399	protein_coding	35,42	0
ENSG00000205710	C17orf107	17	4899418	4902932	protein_coding	35,40	0
ENSG00000064601	CTSA	20	45890144	45898820	protein_coding	35,36	0
ENSG00000133313	CNDP2	18	74495816	74523454	protein_coding	35,35	0
ENSG00000117500	TMED5	1	93149742	93180728	protein_coding	35,33	0
ENSG00000140379	BCL2A1	15	79960889	79971446	protein_coding	35,30	0
ENSG00000167363	FN3K	17	82735575	82751197	protein_coding	35,30	0
ENSG00000149016	TUT1	11	62575045	62592177	protein_coding	35,29	0
ENSG00000001561	ENPP4	6	46129993	46146699	protein_coding	35,29	0
ENSG00000087253	LPCAT2	16	55508998	55586670	protein_coding	35,26	0
ENSG00000149294	NCAM1	11	112961247	113278436	protein_coding	35,21	0
ENSG00000162461	SLC25A34	1	15736405	15741396	protein_coding	35,20	0
ENSG00000137817	PARP6	15	72241181	72272999	protein_coding	35,20	0
ENSG00000243566	UPK3B	7	76510428	76516521	protein_coding	35,20	0

ENSG00000146281	PM20D2	6	89146050	89165565	protein_coding	35,16	0
ENSG00000172081	MOB3A	19	2071038	2096673	protein_coding	35,15	0
ENSG00000119669	IRF2BPL	14	77024543	77028699	protein_coding	35,14	0
ENSG00000278828	HIST1H3H	6	27810064	27811300	protein_coding	35,10	0
ENSG00000105443	CYTH2	19	48469032	48482314	protein_coding	35,08	0
ENSG00000139719	VPS33A	12	122229564	122266521	protein_coding	35,08	0
ENSG00000161791	FMNL3	12	49636499	49708165	protein_coding	35,07	0
ENSG00000108465	CDK5RAP3	17	47967810	47981774	protein_coding	35,05	0
ENSG00000165006	UBAP1	9	34179005	34252523	protein_coding	35,01	0
ENSG00000196136	SERPINA3	14	94612384	94624055	protein_coding	35,00	0
ENSG00000122965	RBM19	12	113816738	113966371	protein_coding	34,99	0
ENSG00000011132	APBA3	19	3750819	3761699	protein_coding	34,98	0
ENSG00000104331	IMPAD1	8	56957933	56993844	protein_coding	34,98	0
ENSG00000174276	ZNHIT2	11	65116403	65117708	protein_coding	34,93	0
ENSG00000172780	RAB43	3	129087569	129122801	protein_coding	34,91	0
ENSG00000142347	MYO1F	19	8520790	8577577	protein_coding	34,84	0
ENSG00000204084	INPP5B	1	37860697	37947057	protein_coding	34,75	0
ENSG00000242265	PEG10	7	94656325	94669695	protein_coding	34,75	0
ENSG00000140749	IGSF6	16	21639537	21652660	protein_coding	34,71	0
ENSG00000134061	CD180	5	67179613	67196799	protein_coding	34,70	0
ENSG00000109046	WSB1	17	27294076	27315926	protein_coding	34,66	0
ENSG00000070831	CDC42	1	22052627	22092946	protein_coding	34,64	0
ENSG00000100442	FKBP3	14	45115600	45135319	protein_coding	34,62	0
ENSG00000167685	ZNF444	19	56132599	56160893	protein_coding	34,62	0
ENSG00000130173	C19orf80	19	11237450	11241943	protein_coding	34,60	0
ENSG00000144597	EAF1	3	15427355	15450635	protein_coding	34,56	0
ENSG00000135956	TMEM127	2	96248516	96265994	protein_coding	34,55	0
ENSG00000007376	RPUSD1	16	784974	788397	protein_coding	34,52	0
ENSG00000196187	TMEM63A	1	225845536	225882369	protein_coding	34,51	0
ENSG00000254505	CHMP4A	14	24209583	24213869	protein_coding	34,50	0
ENSG00000243660	ZNF487	10	43436841	43483179	protein_coding	34,48	0
ENSG00000178971	CTC1	17	8224821	8248044	protein_coding	34,46	0
ENSG00000115257	PCSK4	19	1481428	1490752	protein_coding	34,46	0
ENSG00000112561	TFEB	6	41683978	41736259	protein_coding	34,46	0
ENSG00000136861	CDK5RAP2	9	120388869	120580170	protein_coding	34,44	0
ENSG00000163703	CRELD1	3	9933822	9945413	protein_coding	34,44	0
ENSG00000132109	TRIM21	11	4384897	4393696	protein_coding	34,42	0
ENSG00000164463	CREBRF	5	173056352	173139284	protein_coding	34,41	0
ENSG00000167968	DNASE1L2	16	2235816	2238711	protein_coding	34,40	0
ENSG00000084754	HADHA	2	26190635	26244726	protein_coding	34,36	0
ENSG00000166313	APBB1	11	6395124	6419414	protein_coding	34,35	0
ENSG00000182768	NGRN	15	90265659	90278141	protein_coding	34,33	0
ENSG00000069702	TGFBR3	1	91680343	91906335	protein_coding	34,31	0
ENSG00000141522	ARHGDIA	17	81867721	81871406	protein_coding	34,26	0
ENSG00000171262	FAM98B	15	38454127	38487710	protein_coding	34,26	0
ENSG00000182872	RBM10	X	47144869	47186813	protein_coding	34,23	0
ENSG00000095059	DHPS	19	12675717	12681902	protein_coding	34,23	0
ENSG00000279508	ENSG000002	14	19300177	19302924	protein_coding	34,18	0
ENSG00000114473	IQCG	3	197889075	197960142	protein_coding	34,16	0
ENSG00000091436	ENSG000000	2	173075435	173268010	protein_coding	34,13	0
ENSG00000197816	CCDC180	9	97307304	97378524	protein_coding	34,13	0
ENSG00000124104	SNX21	20	45833810	45843275	protein_coding	34,13	0
ENSG00000181418	DDN	12	48995149	48999309	protein_coding	34,10	0
ENSG00000166343	MSS51	10	73423579	73433561	protein_coding	34,10	0
ENSG00000112406	HECA	6	139135112	139180802	protein_coding	34,07	0
ENSG00000205323	SARNP	12	55752463	55817756	protein_coding	34,06	0
ENSG00000005884	ITGA3	17	50055968	50090481	protein_coding	34,06	0
ENSG00000179218	CALR	19	12938578	12944489	protein_coding	34,05	0

ENSG00000165806	CASP7	10	113679162	113730907	protein_coding	34,01	0
ENSG00000122861	PLAU	10	73909177	73917497	protein_coding	34,00	0
ENSG00000166510	CCDC68	18	54901509	54959508	protein_coding	34,00	0
ENSG00000002745	WNT16	7	121325367	121341104	protein_coding	34,00	0
ENSG00000173376	NDNF	4	121035613	121073021	protein_coding	34,00	0
ENSG00000135917	SLC19A3	2	227685210	227718012	protein_coding	34,00	0
ENSG00000189164	ZNF527	19	37371061	37393066	protein_coding	33,98	0
ENSG00000145012	LPP	3	188153284	188890671	protein_coding	33,95	0
ENSG00000130164	LDLR	19	11089362	11133816	protein_coding	33,89	0
ENSG00000154127	UBASH3B	11	122655675	122814473	protein_coding	33,88	0
ENSG00000100284	TOM1	22	35299275	35347994	protein_coding	33,83	0
ENSG00000159263	SIM2	21	36699133	36749917	protein_coding	33,82	0
ENSG00000203734	ECT2L	6	138795926	138904070	protein_coding	33,82	0
ENSG00000087095	NLK	17	28041737	28196381	protein_coding	33,75	0
ENSG00000257727	CNPY2	12	56309842	56316336	protein_coding	33,74	0
ENSG00000109220	CHIC2	4	54009789	54064690	protein_coding	33,70	0
ENSG00000049883	PTCD2	5	72320367	72368395	protein_coding	33,69	0
ENSG00000188277	C15orf62	15	40770080	40772449	protein_coding	33,64	0
ENSG00000101596	SMCHD1	18	2655738	2805017	protein_coding	33,60	0
ENSG00000165985	C1QL3	10	16513743	16522005	protein_coding	33,60	0
ENSG00000043591	ADRB1	10	114044056	114046908	protein_coding	33,58	0
ENSG00000077549	CAPZB	1	19338776	19485539	protein_coding	33,57	0
ENSG00000005889	ZFX	X	24149173	24216255	protein_coding	33,54	0
ENSG00000161203	AP2M1	3	184174689	184184091	protein_coding	33,52	0
ENSG00000180098	TRNAU1AP	1	28553085	28578545	protein_coding	33,49	0
ENSG00000102103	PQBP1	X	48897912	48903143	protein_coding	33,48	0
ENSG00000163082	SGPP2	2	222424517	222560948	protein_coding	33,48	0
ENSG00000175197	DDIT3	12	57516588	57520517	protein_coding	33,47	0
ENSG00000173818	ENDOV	17	80415165	80438086	protein_coding	33,47	0
ENSG00000196588	MKL1	22	40410281	40636702	protein_coding	33,44	0
ENSG00000130304	SLC27A1	19	17468769	17506168	protein_coding	33,44	0
ENSG00000198576	ARC	8	142611044	142614472	protein_coding	33,40	0
ENSG00000116299	KIAA1324	1	109113679	109206781	protein_coding	33,40	0
ENSG00000204071	TCEAL6	X	102140476	102142970	protein_coding	33,40	0
ENSG00000177728	TMEM94	17	75441159	75500090	protein_coding	33,38	0
ENSG00000138674	SEC31A	4	82818661	82901166	protein_coding	33,36	0
ENSG00000133316	WDR74	11	62832342	62841809	protein_coding	33,36	0
ENSG00000069667	RORA	15	60488284	61229319	protein_coding	33,34	0
ENSG00000065911	MTHFD2	2	74198562	74217565	protein_coding	33,34	0
ENSG00000141759	TXNL4A	18	79970811	80033949	protein_coding	33,33	0
ENSG00000116194	ANGPTL1	1	178849705	178871052	protein_coding	33,31	0
ENSG00000054277	OPN3	1	241590102	241677376	protein_coding	33,31	0
ENSG00000144591	GMPPA	2	219498867	219506989	protein_coding	33,28	0
ENSG00000107404	DVL1	1	1335276	1349350	protein_coding	33,27	0
ENSG00000125388	GRK4	4	2963608	3040747	protein_coding	33,26	0
ENSG00000068438	FTSJ1	X	48476021	48486364	protein_coding	33,25	0
ENSG00000204577	LILRB3	19	54216278	54223506	protein_coding	33,25	0
ENSG00000173480	ZNF417	19	57900296	57916610	protein_coding	33,23	0
ENSG00000204179	PTPN20	10	46911396	47002488	protein_coding	33,23	0
ENSG00000198113	TOR4A	9	137277749	137282641	protein_coding	33,20	0
ENSG00000130208	APOC1	19	44914247	44919349	protein_coding	33,17	0
ENSG00000084070	SMAP2	1	40344850	40423326	protein_coding	33,16	0
ENSG00000255423	EBLN2	3	73061659	73063337	protein_coding	33,13	0
ENSG00000160767	FAM189B	1	155247205	155255483	protein_coding	33,10	0
ENSG00000185633	NDUFA4L2	12	57234903	57240715	protein_coding	33,10	0
ENSG00000172733	PURG	8	30995802	31033715	protein_coding	33,10	0
ENSG00000154342	WNT3A	1	228007051	228061260	protein_coding	33,10	0
ENSG00000123472	ATPAF1	1	46632737	46673867	protein_coding	33,10	0

ENSG00000215305	VPS16	20	2840703	2866732	protein_coding	33,09	0
ENSG00000186501	TMEM222	1	27322145	27336400	protein_coding	33,07	0
ENSG00000221823	PPP3R1	2	68178857	68256237	protein_coding	33,07	0
ENSG00000023445	BIRC3	11	102317450	102339403	protein_coding	33,06	0
ENSG00000100075	SLC25A1	22	19175575	19178830	protein_coding	33,04	0
ENSG00000141497	ZMYND15	17	4740015	4746119	protein_coding	33,00	0
ENSG00000204323	SMIM5	17	75633434	75641404	protein_coding	33,00	0
ENSG00000087245	MMP2	16	55389700	55506691	protein_coding	33,00	0
ENSG00000181722	ZBTB20	3	114338094	115147271	protein_coding	33,00	0
ENSG00000124155	PIGT	20	45416077	45426244	protein_coding	33,00	0
ENSG00000169221	TBC1D10B	16	30357102	30370264	protein_coding	32,98	0
ENSG00000141560	FN3KRP	17	82716683	82730328	protein_coding	32,96	0
ENSG00000110721	CHKA	11	68052859	68121444	protein_coding	32,95	0
ENSG00000135930	EIF4E2	2	232550052	232583644	protein_coding	32,92	0
ENSG00000143420	ENSA	1	150600851	150629612	protein_coding	32,90	0
ENSG00000172183	ISG20	15	88636153	88656483	protein_coding	32,90	0
ENSG00000183060	LYSMD4	15	99715697	99733561	protein_coding	32,90	0
ENSG00000069431	ABCC9	12	21797401	21941402	protein_coding	32,90	0
ENSG00000143545	RAB13	1	153981617	153986358	protein_coding	32,90	0
ENSG00000136449	MYCBPAP	17	50508384	50531501	protein_coding	32,87	0
ENSG00000242372	EIF6	20	35278907	35284985	protein_coding	32,86	0
ENSG00000136908	DPM2	9	127935099	127938484	protein_coding	32,84	0
ENSG00000099331	MYO9B	19	17075781	17214537	protein_coding	32,81	0
ENSG00000167851	CD300A	17	74466416	74484796	protein_coding	32,80	0
ENSG00000173597	SULT1B1	4	69721162	69787961	protein_coding	32,80	0
ENSG00000121807	CCR2	3	46353734	46360928	protein_coding	32,80	0
ENSG00000188582	PAQR9	3	142949164	142963682	protein_coding	32,80	0
ENSG00000186395	KRT10	17	40818117	40822595	protein_coding	32,79	0
ENSG00000184792	OSBP2	22	30693782	30907824	protein_coding	32,78	0
ENSG00000160051	IQCC	1	32205661	32208687	protein_coding	32,75	0
ENSG00000063176	SPHK2	19	48619291	48630717	protein_coding	32,72	0
ENSG00000101542	CDH20	18	61333582	61555773	protein_coding	32,70	0
ENSG00000169413	RNASE6	14	20781051	20782467	protein_coding	32,70	0
ENSG00000164695	CHMP4C	8	81732434	81759515	protein_coding	32,70	0
ENSG00000196972	SMIM10L2B	X	135095028	135098634	protein_coding	32,70	0
ENSG00000221955	SLC12A8	3	125082636	125212864	protein_coding	32,67	0
ENSG00000134987	WDR36	5	111091716	111130502	protein_coding	32,67	0
ENSG00000196367	TRRAP	7	98877933	99013243	protein_coding	32,66	0
ENSG00000241370	RPP21	6	30345131	30346884	protein_coding	32,64	0
ENSG00000058091	CDK14	7	90466424	91210590	protein_coding	32,61	0
ENSG00000122218	COPA	1	160289273	160343400	protein_coding	32,61	0
ENSG00000106003	LFNG	7	2512529	2529177	protein_coding	32,60	0
ENSG00000178996	SNX18	5	54517759	54546585	protein_coding	32,57	0
ENSG00000165119	HNRNPK	9	83968083	83980616	protein_coding	32,57	0
ENSG00000148335	NTMT1	9	129608884	129636131	protein_coding	32,56	0
ENSG00000101307	SIRPB1	20	1563521	1620061	protein_coding	32,55	0
ENSG00000256771	ZNF253	19	19865886	19894674	protein_coding	32,55	0
ENSG00000089693	MLF2	12	6747996	6767475	protein_coding	32,52	0
ENSG00000151806	GUF1	4	44678427	44700926	protein_coding	32,51	0
ENSG00000153560	UBP1	3	33388336	33441371	protein_coding	32,48	0
ENSG00000087111	PIGS	17	28553383	28571872	protein_coding	32,46	0
ENSG00000172009	THOP1	19	2785460	2815807	protein_coding	32,46	0
ENSG00000116199	FAM20B	1	179025804	179076562	protein_coding	32,44	0
ENSG00000115085	ZAP70	2	97713560	97739862	protein_coding	32,40	0
ENSG00000136026	CKAP4	12	106237877	106304279	protein_coding	32,39	0
ENSG00000154654	NCAM2	21	20998315	21543329	protein_coding	32,33	0
ENSG00000175309	PHYKPL	5	178208497	178232791	protein_coding	32,32	0
ENSG00000151726	ACSL1	4	184755595	184826818	protein_coding	32,32	0

ENSG00000186166	CCDC84	11	118998142	119015791	protein_coding	32,31	0
ENSG00000089775	ZBTB25	14	64449106	64505213	protein_coding	32,31	0
ENSG00000069869	NEDD4	15	55826922	55993746	protein_coding	32,30	0
ENSG00000101605	MYOM1	18	3066807	3220108	protein_coding	32,30	0
ENSG00000121310	ECHDC2	1	52895910	52927212	protein_coding	32,27	0
ENSG00000159423	ALDH4A1	1	18871430	18902781	protein_coding	32,27	0
ENSG00000103051	COG4	16	70480568	70523565	protein_coding	32,25	0
ENSG00000114767	RRP9	3	51933430	51941941	protein_coding	32,24	0
ENSG00000148204	CRB2	9	123356170	123380324	protein_coding	32,24	0
ENSG00000113648	H2AFY	5	135333900	135399914	protein_coding	32,23	0
ENSG00000168060	NAALADL1	11	65044818	65058549	protein_coding	32,20	0
ENSG00000147689	FAM83A	8	123178960	123210079	protein_coding	32,20	0
ENSG00000158481	CD1C	1	158289786	158293630	protein_coding	32,20	0
ENSG00000163482	STK36	2	218672026	218702716	protein_coding	32,19	0
ENSG00000132005	RFX1	19	13961538	14007039	protein_coding	32,16	0
ENSG00000184545	DUSP8	11	1554044	1571920	protein_coding	32,16	0
ENSG00000198736	MSRB1	16	1938210	1943326	protein_coding	32,14	0
ENSG00000178149	DALRD3	3	49015488	49022293	protein_coding	32,11	0
ENSG00000085465	OVGP1	1	111414314	111427777	protein_coding	32,10	0
ENSG00000279457	ENSG000002	1	184923	200322	protein_coding	32,10	0
ENSG00000164924	YWHAZ	8	100916525	100953388	protein_coding	32,07	0
ENSG00000076924	XAB2	19	7619525	7629565	protein_coding	32,06	0
ENSG00000197157	SND1	7	127652180	128092609	protein_coding	32,02	0
ENSG00000156711	MAPK13	6	36127809	36144524	protein_coding	32,02	0
ENSG00000111424	VDR	12	47841537	47943048	protein_coding	32,00	0
ENSG00000230453	ANKRD18B	9	33524394	33573009	protein_coding	32,00	0
ENSG00000070193	FGF10	5	44303544	44389706	protein_coding	32,00	0
ENSG00000179630	LACC1	13	43879284	43893932	protein_coding	31,99	0
ENSG00000074370	ATP2A3	17	3923870	3964464	protein_coding	31,98	0
ENSG00000071054	MAP4K4	2	101696850	101894689	protein_coding	31,97	0
ENSG00000136273	HUS1	7	47695730	47979581	protein_coding	31,96	0
ENSG00000131398	KCNC3	19	50311937	50333515	protein_coding	31,95	0
ENSG00000155304	HSPA13	21	14371115	14383484	protein_coding	31,92	0
ENSG00000103067	ESRP2	16	68229111	68238102	protein_coding	31,91	0
ENSG00000065559	MAP2K4	17	12020824	12143830	protein_coding	31,91	0
ENSG00000129204	USP6	17	5116438	5175034	protein_coding	31,89	0
ENSG00000158828	PINK1	1	20633455	20651511	protein_coding	31,84	0
ENSG00000206560	ANKRD28	3	15667236	15859771	protein_coding	31,84	0
ENSG00000159307	SCUBE1	22	43197283	43343388	protein_coding	31,83	0
ENSG00000258472	ENSG000002	17	28455752	28614197	protein_coding	31,80	0
ENSG00000166762	CATSPER2	15	43628503	43668118	protein_coding	31,80	0
ENSG00000125991	ERGIC3	20	35542021	35557634	protein_coding	31,78	0
ENSG00000160991	ORAI2	7	102433106	102456821	protein_coding	31,77	0
ENSG00000006715	VPS41	7	38722963	38932394	protein_coding	31,72	0
ENSG00000177189	RPS6KA3	X	20149911	20267100	protein_coding	31,66	0
ENSG00000129450	SIGLEC9	19	51124908	51136651	protein_coding	31,60	0
ENSG00000274750	HIST1H3E	6	26224199	26227473	protein_coding	31,60	0
ENSG00000144040	SFXN5	2	72942036	73075619	protein_coding	31,60	0
ENSG00000108829	LRRC59	17	50375059	50397553	protein_coding	31,59	0
ENSG00000100216	TOMM22	22	38681948	38685421	protein_coding	31,56	0
ENSG00000096717	SIRT1	10	67884669	67918390	protein_coding	31,54	0
ENSG00000156973	PDE6D	2	231732425	231786272	protein_coding	31,54	0
ENSG00000253731	PCDHGA6	5	141373914	141512979	protein_coding	31,50	0
ENSG00000259956	RBM15B	3	51391268	51397908	protein_coding	31,48	0
ENSG00000173905	GOLIM4	3	168008677	168095975	protein_coding	31,43	0
ENSG00000173875	ZNF791	19	12610918	12633840	protein_coding	31,40	0
ENSG00000212127	TAS2R14	12	10937406	11171573	protein_coding	31,40	0
ENSG00000108039	XPNPEP1	10	109864766	109923553	protein_coding	31,39	0

ENSG00000132589	FLOT2	17	28879335	28897679	protein_coding	31,35	0
ENSG00000068120	COASY	17	42561467	42566277	protein_coding	31,34	0
ENSG00000110422	HIPK3	11	33256672	33357023	protein_coding	31,30	0
ENSG00000145708	CRHBP	5	76952713	76981158	protein_coding	31,24	0
ENSG00000135097	MSI1	12	120341330	120369180	protein_coding	31,21	0
ENSG00000148719	DNAJB12	10	72332830	72355230	protein_coding	31,21	0
ENSG00000139154	AEBP2	12	19404045	19720801	protein_coding	31,20	0
ENSG00000176293	ZNF135	19	58059239	58086310	protein_coding	31,18	0
ENSG00000155903	RASA2	3	141487047	141615342	protein_coding	31,18	0
ENSG00000186222	BLOC1S4	4	6716055	6717671	protein_coding	31,18	0
ENSG00000123728	RAP2C	X	132203024	132219480	protein_coding	31,17	0
ENSG00000182944	EWSR1	22	29268009	29300525	protein_coding	31,16	0
ENSG00000164535	DAGLB	7	6409126	6484190	protein_coding	31,08	0
ENSG00000119574	ZBTB45	19	58513530	58538911	protein_coding	31,08	0
ENSG00000175662	TOM1L2	17	17843511	17972422	protein_coding	31,06	0
ENSG00000262814	MRPL12	17	81703357	81707526	protein_coding	31,04	0
ENSG00000099341	PSMD8	19	38374536	38383824	protein_coding	31,02	0
ENSG00000188026	RILPL1	12	123470054	123533718	protein_coding	31,02	0
ENSG00000134109	EDEM1	3	5187646	5219957	protein_coding	31,00	0
ENSG00000148672	GLUD1	10	87050486	87094866	protein_coding	30,98	0
ENSG00000170579	DLGAP1	18	3496032	4455335	protein_coding	30,97	0
ENSG00000128045	RASL11B	4	52862290	52866835	protein_coding	30,96	0
ENSG00000171700	RGS19	20	64073181	64079988	protein_coding	30,96	0
ENSG00000170647	TMEM133	11	100991989	100993941	protein_coding	30,95	0
ENSG00000088812	ATRN	20	3471040	3651122	protein_coding	30,93	0
ENSG00000163958	ZDHHC19	3	196197449	196211437	protein_coding	30,92	0
ENSG00000162551	ALPL	1	21509372	21578412	protein_coding	30,90	0
ENSG00000175591	P2RY2	11	73218298	73236352	protein_coding	30,90	0
ENSG00000120324	PCDHB10	5	141182560	141195642	protein_coding	30,90	0
ENSG00000135776	ABCB10	1	229516582	229558695	protein_coding	30,88	0
ENSG00000150756	FAM173B	5	10226330	10249897	protein_coding	30,88	0
ENSG00000123144	C19orf43	19	12730640	12734775	protein_coding	30,87	0
ENSG00000151881	C5orf28	5	43444252	43483893	protein_coding	30,86	0
ENSG00000163155	LYSMD1	1	151159748	151165948	protein_coding	30,83	0
ENSG00000167680	SEMA6B	19	4542593	4559808	protein_coding	30,80	0
ENSG00000182196	ARL6IP4	12	122980060	122982913	protein_coding	30,80	0
ENSG00000110025	SNX15	11	65027408	65040572	protein_coding	30,80	0
ENSG00000101294	HM13	20	31514428	31577923	protein_coding	30,79	0
ENSG00000171160	MORN4	10	97614553	97633500	protein_coding	30,78	0
ENSG00000100412	ACO2	22	41469125	41528989	protein_coding	30,75	0
ENSG00000142233	NTN5	19	48661407	48673081	protein_coding	30,70	0
ENSG00000197498	RPF2	6	110982015	111028263	protein_coding	30,68	0
ENSG00000161835	GRASP	12	52006940	52015889	protein_coding	30,66	0
ENSG00000171724	VAT1L	16	77788530	77980107	protein_coding	30,64	0
ENSG00000140795	MYLK3	16	46703369	46790407	protein_coding	30,60	0
ENSG00000160870	CYP3A7	7	99705037	99735196	protein_coding	30,60	0
ENSG00000044524	EPHA3	3	89107524	89482134	protein_coding	30,60	0
ENSG00000143318	CASQ1	1	160190556	160201886	protein_coding	30,60	0
ENSG00000123415	SMUG1	12	54121277	54189008	protein_coding	30,57	0
ENSG00000179950	PUF60	8	143816344	143829859	protein_coding	30,56	0
ENSG00000156162	DPY19L4	8	94719703	94793836	protein_coding	30,55	0
ENSG00000169962	TAS1R3	1	1331314	1335306	protein_coding	30,54	0
ENSG00000177951	BET1L	11	167784	207428	protein_coding	30,52	0
ENSG00000205177	C11orf91	11	33698261	33700801	protein_coding	30,50	0
ENSG00000181638	ZFP41	8	143246821	143262705	protein_coding	30,50	0
ENSG00000174740	PABPC5	X	91434595	91438584	protein_coding	30,50	0
ENSG00000162599	NFIA	1	60865259	61462793	protein_coding	30,48	0
ENSG00000180574	ENSG00000[illegible]	12	10505602	10523135	protein_coding	30,47	0

ENSG00000166068	SPRED1	15	38252326	38357249	protein_coding	30,46	0
ENSG00000140464	PML	15	73994673	74047812	protein_coding	30,41	0
ENSG00000170881	RNF139	8	124474738	124487914	protein_coding	30,41	0
ENSG00000076201	PTPN23	3	47381011	47413441	protein_coding	30,40	0
ENSG00000269313	MAGIX	X	49162564	49168483	protein_coding	30,40	0
ENSG00000177666	PNPLA2	11	818902	825573	protein_coding	30,39	0
ENSG00000100836	PABPN1	14	23321289	23326185	protein_coding	30,30	0
ENSG00000175564	UCP3	11	74000281	74009435	protein_coding	30,30	0
ENSG00000137161	CNPY3	6	42929192	42939287	protein_coding	30,28	0
ENSG00000115073	ACTR1B	2	97655963	97664107	protein_coding	30,28	0
ENSG00000161654	LSM12	17	44034635	44067619	protein_coding	30,26	0
ENSG00000025434	NR1H3	11	47248300	47269032	protein_coding	30,26	0
ENSG00000118985	ELL2	5	95885098	95962071	protein_coding	30,23	0
ENSG00000105438	KDELR1	19	48382570	48391553	protein_coding	30,22	0
ENSG00000144852	NR1I2	3	119780484	119818485	protein_coding	30,20	0
ENSG00000099260	PALMD	1	99645943	99694541	protein_coding	30,18	0
ENSG00000165527	ARF6	14	49893092	49897054	protein_coding	30,16	0
ENSG00000136271	DDX56	7	44565417	44575051	protein_coding	30,15	0
ENSG00000148468	FAM171A1	10	15211643	15371062	protein_coding	30,14	0
ENSG00000116679	IVNS1ABP	1	185296388	185317329	protein_coding	30,14	0
ENSG00000104897	SF3A2	19	2236504	2248679	protein_coding	30,11	0
ENSG00000175832	ETV4	17	43527844	43579620	protein_coding	30,10	0
ENSG00000072195	SPEG	2	219434846	219498287	protein_coding	30,10	0
ENSG00000197915	HRNR	1	152212082	152224193	protein_coding	30,10	0
ENSG00000126746	ZNF384	12	6666477	6689572	protein_coding	30,08	0
ENSG00000115844	DLX2	2	172099439	172102900	protein_coding	30,08	0
ENSG00000101353	MROH8	20	37101226	37179588	protein_coding	30,00	0
ENSG00000197858	GPAA1	8	144082590	144086216	protein_coding	30,00	0
ENSG00000163913	IFT122	3	129440036	129520507	protein_coding	29,99	0
ENSG00000100105	PATZ1	22	31325804	31346232	protein_coding	29,99	0
ENSG00000110799	VWF	12	5948874	6124770	protein_coding	29,96	0
ENSG00000139233	LLPH	12	66116555	66130768	protein_coding	29,95	0
ENSG00000162878	PKDCC	2	42048020	42058528	protein_coding	29,93	0
ENSG00000101210	EEF1A2	20	63488013	63499315	protein_coding	29,92	0
ENSG00000023839	ABCC2	10	99782732	99852192	protein_coding	29,92	0
ENSG00000253729	PRKDC	8	47773108	47960183	protein_coding	29,91	0
ENSG00000100311	PDGFB	22	39223359	39244751	protein_coding	29,90	0
ENSG00000092850	TEKT2	1	36084075	36088275	protein_coding	29,90	0
ENSG00000157349	DDX19B	16	70289663	70335283	protein_coding	29,88	0
ENSG00000141349	G6PC3	17	44070735	44076344	protein_coding	29,87	0
ENSG00000198853	RUSC2	9	35490127	35561898	protein_coding	29,84	0
ENSG00000111726	CMAS	12	22046174	22065674	protein_coding	29,83	0
ENSG00000166321	NUDT13	10	73110375	73131828	protein_coding	29,82	0
ENSG00000163681	SLMAP	3	57755450	57929168	protein_coding	29,82	0
ENSG00000164136	IL15	4	141636599	141733987	protein_coding	29,80	0
ENSG00000096070	BRPF3	6	36196744	36232790	protein_coding	29,79	0
ENSG00000112245	PTP4A1	6	63521761	63583587	protein_coding	29,79	0
ENSG00000225190	PLEKHM1	17	45435900	45490749	protein_coding	29,75	0
ENSG00000186815	TPCN1	12	113221050	113298585	protein_coding	29,75	0
ENSG00000168994	PXDC1	6	3722614	3752026	protein_coding	29,73	0
ENSG00000143643	TTC13	1	230906243	230978875	protein_coding	29,71	0
ENSG00000204131	NHSL2	X	71910818	72161750	protein_coding	29,70	0
ENSG00000074219	TEAD2	19	49340595	49362457	protein_coding	29,69	0
ENSG00000178562	CD28	2	203706475	203738912	protein_coding	29,67	0
ENSG00000174917	C19orf70	19	5678421	5680896	protein_coding	29,64	0
ENSG00000145020	AMT	3	49416778	49422753	protein_coding	29,60	0
ENSG00000258311	ENSG00000[illegible]	12	55716036	55724703	protein_coding	29,60	0
ENSG00000184343	SRPK3	X	153776412	153785732	protein_coding	29,60	0

ENSG00000170892	TSEN34	19	54189938	54194536	protein_coding	29,60	0
ENSG00000176396	EID2	19	39538250	39540230	protein_coding	29,58	0
ENSG00000072818	ACAP1	17	7336529	7351478	protein_coding	29,53	0
ENSG00000170855	TRIAP1	12	120443961	120446412	protein_coding	29,53	0
ENSG00000053438	NNAT	20	37521215	37523693	protein_coding	29,50	0
ENSG00000164953	TMEM67	8	93754844	93819234	protein_coding	29,50	0
ENSG00000129596	CDO1	5	115804733	115816954	protein_coding	29,50	0
ENSG00000183684	ALYREF	17	81887844	81891586	protein_coding	29,48	0
ENSG00000120889	TNFRSF10B	8	23020133	23069179	protein_coding	29,47	0
ENSG00000106367	AP1S1	7	101154397	101161596	protein_coding	29,47	0
ENSG00000108950	FAM20A	17	68535113	68601389	protein_coding	29,46	0
ENSG00000159761	C16orf86	16	67666816	67668758	protein_coding	29,45	0
ENSG00000149485	FADS1	11	61799625	61829318	protein_coding	29,44	0
ENSG00000105058	FAM32A	19	16185380	16192046	protein_coding	29,42	0
ENSG00000101417	PXMP4	20	33702754	33720319	protein_coding	29,42	0
ENSG00000132570	PCBD2	5	134904906	135007959	protein_coding	29,41	0
ENSG00000198046	ZNF667	19	56439325	56478065	protein_coding	29,36	0
ENSG00000181588	MEX3D	19	1554669	1568058	protein_coding	29,33	0
ENSG00000006704	GTF2IRD1	7	74453790	74602604	protein_coding	29,30	0
ENSG00000204922	UQCC3	11	62670273	62673687	protein_coding	29,28	0
ENSG00000104728	ARHGEF10	8	1823976	1958641	protein_coding	29,27	0
ENSG00000136280	CCM2	7	44999475	45076469	protein_coding	29,24	0
ENSG00000169439	SDC2	8	96493351	96611780	protein_coding	29,22	0
ENSG00000156968	MPV17L	16	15395754	15413268	protein_coding	29,20	0
ENSG00000173391	OLR1	12	10158301	10172138	protein_coding	29,20	0
ENSG00000173020	ADRBK1	11	67266410	67286556	protein_coding	29,19	0
ENSG00000140280	LYSMD2	15	51723011	51751585	protein_coding	29,19	0
ENSG00000206418	RAB12	18	8609445	8639381	protein_coding	29,18	0
ENSG00000204052	LRRC73	6	43506969	43510686	protein_coding	29,18	0
ENSG00000132394	EEFSEC	3	128153454	128408646	protein_coding	29,16	0
ENSG00000142327	RNPEPL1	2	240565804	240581372	protein_coding	29,14	0
ENSG00000258366	RTEL1	20	63657810	63696253	protein_coding	29,13	0
ENSG00000175470	PPP2R2D	10	131900644	131959834	protein_coding	29,12	0
ENSG00000186517	ARHGAP30	1	161046946	161069970	protein_coding	29,12	0
ENSG00000184489	PTP4A3	8	141391993	141432454	protein_coding	29,08	0
ENSG00000110917	MLEC	12	120686869	120701864	protein_coding	29,07	0
ENSG00000163156	SCNM1	1	151156664	151170297	protein_coding	29,07	0
ENSG00000160447	PKN3	9	128702523	128720918	protein_coding	29,04	0
ENSG00000104549	SQLE	8	124998497	125022283	protein_coding	29,03	0
ENSG00000134905	CARS2	13	110641412	110713603	protein_coding	29,00	0
ENSG00000180644	PRF1	10	70597348	70602775	protein_coding	29,00	0
ENSG00000102385	DRP2	X	101219769	101264497	protein_coding	29,00	0
ENSG00000103502	CDIPT	16	29858357	29863736	protein_coding	29,00	0
ENSG00000137171	KLC4	6	43040777	43075099	protein_coding	28,97	0
ENSG00000004478	FKBP4	12	2794953	2805423	protein_coding	28,96	0
ENSG00000142166	IFNAR1	21	33324477	33359862	protein_coding	28,95	0
ENSG00000100399	CHADL	22	41229510	41240934	protein_coding	28,94	0
ENSG00000117408	IPO13	1	43946939	43968022	protein_coding	28,92	0
ENSG00000198756	COLGALT2	1	183929854	184037729	protein_coding	28,90	0
ENSG00000172890	NADSYN1	11	71453109	71524107	protein_coding	28,89	0
ENSG00000122783	C7orf49	7	135092363	135170795	protein_coding	28,85	0
ENSG00000188690	UROS	10	125784980	125823248	protein_coding	28,85	0
ENSG00000067064	IDI1	10	1039908	1049170	protein_coding	28,83	0
ENSG00000070214	SLC44A1	9	105244622	105439171	protein_coding	28,81	0
ENSG00000263465	SRSF8	11	95067197	95071224	protein_coding	28,81	0
ENSG00000108094	CUL2	10	35008551	35090642	protein_coding	28,75	0
ENSG00000100997	ABHD12	20	25294743	25390983	protein_coding	28,75	0
ENSG00000185164	NOMO2	16	18417325	18562211	protein_coding	28,75	0

ENSG00000134851	TMEM165	4	55395957	55453397	protein_coding	28,74	0
ENSG00000160087	UBE2J2	1	1253909	1273885	protein_coding	28,73	0
ENSG00000140105	WARS	14	100333788	100376805	protein_coding	28,71	0
ENSG00000138231	DBR1	3	138161012	138174949	protein_coding	28,70	0
ENSG00000108479	GALK1	17	75751594	75765711	protein_coding	28,67	0
ENSG00000250486	FAM218A	4	164956948	164959122	protein_coding	28,67	0
ENSG00000107263	RAPGEF1	9	131576770	131740074	protein_coding	28,66	0
ENSG00000100483	VCPKMT	14	50108632	50116600	protein_coding	28,63	0
ENSG00000147168	IL2RG	X	71107404	71112108	protein_coding	28,60	0
ENSG00000114315	HES1	3	194136145	194138732	protein_coding	28,57	0
ENSG00000162735	PEX19	1	160276812	160286348	protein_coding	28,52	0
ENSG00000139910	NOVA1	14	26443093	26597754	protein_coding	28,50	0
ENSG00000150051	MKX	10	27672875	27746060	protein_coding	28,50	0
ENSG00000185823	NPAP1	15	24675868	24683393	protein_coding	28,47	0
ENSG00000113739	STC2	5	173314713	173329503	protein_coding	28,46	0
ENSG00000141574	SECTM1	17	82321024	82334074	protein_coding	28,40	0
ENSG00000156463	SH3RF2	5	145936579	146081791	protein_coding	28,40	0
ENSG00000164120	HPGD	4	174490177	174523154	protein_coding	28,40	0
ENSG00000157064	NMNAT2	1	183248237	183418602	protein_coding	28,40	0
ENSG00000099800	TIMM13	19	2425624	2427894	protein_coding	28,39	0
ENSG00000111331	OAS3	12	112938352	112973249	protein_coding	28,39	0
ENSG00000141002	TCF25	16	89873586	89911384	protein_coding	28,39	0
ENSG00000145681	HAPLN1	5	83637805	83721613	protein_coding	28,38	0
ENSG00000170485	NPAS2	2	100820152	100996829	protein_coding	28,34	0
ENSG00000117984	CTSD	11	1752752	1763992	protein_coding	28,33	0
ENSG00000257335	MGAM	7	141907813	142106747	protein_coding	28,33	0
ENSG00000121775	TMEM39B	1	32072031	32102866	protein_coding	28,33	0
ENSG00000184900	SUMO3	21	44805617	44818779	protein_coding	28,31	0
ENSG00000204677	FAM153C	5	178006405	178055559	protein_coding	28,30	0
ENSG00000105700	KXD1	19	18557762	18569387	protein_coding	28,29	0
ENSG00000118200	CAMSAP2	1	200739558	200860704	protein_coding	28,28	0
ENSG00000110934	BIN2	12	51281038	51324668	protein_coding	28,28	0
ENSG00000117862	TXNDC12	1	52020131	52056171	protein_coding	28,28	0
ENSG00000176542	USF3	3	113648385	113696646	protein_coding	28,26	0
ENSG00000145740	SLC30A5	5	69093646	69131069	protein_coding	28,26	0
ENSG00000110107	PRPF19	11	60890730	60906588	protein_coding	28,26	0
ENSG00000154813	DPH3	3	16257978	16264972	protein_coding	28,25	0
ENSG00000132613	MTSS1L	16	70661204	70686066	protein_coding	28,23	0
ENSG00000104219	ZDHHC2	8	17156029	17224799	protein_coding	28,20	0
ENSG00000161551	ZNF577	19	51855802	51890950	protein_coding	28,20	0
ENSG00000092295	TGM1	14	24249114	24264432	protein_coding	28,20	0
ENSG00000187091	PLCD1	3	38007496	38029762	protein_coding	28,20	0
ENSG00000153885	KCTD15	19	33795933	33815763	protein_coding	28,19	0
ENSG00000111639	MRPL51	12	6491886	6493841	protein_coding	28,19	0
ENSG00000056558	TRAF1	9	120902393	120929173	protein_coding	28,16	0
ENSG00000163170	BOLA3	2	74135398	74147994	protein_coding	28,15	0
ENSG00000149571	KIRREL3	11	126423359	127003460	protein_coding	28,14	0
ENSG00000075073	TACR2	10	69403903	69416867	protein_coding	28,10	0
ENSG00000176410	DNAJC30	7	73680969	73683453	protein_coding	28,09	0
ENSG00000118960	HS1BP3	2	20560448	20651089	protein_coding	28,07	0
ENSG00000117009	KMO	1	241532134	241595642	protein_coding	28,06	0
ENSG00000148841	ITPRIP	10	104309698	104338404	protein_coding	28,06	0
ENSG00000183386	FHL3	1	37996770	38005606	protein_coding	28,05	0
ENSG00000163820	FYCO1	3	45917899	45995824	protein_coding	28,01	0
ENSG00000031698	SARS	1	109213918	109238169	protein_coding	28,01	0
ENSG00000235194	PPP1R3E	14	23295643	23302848	protein_coding	28,00	0
ENSG00000177359	ENSG00000	12	31111652	31206154	protein_coding	28,00	0
ENSG00000132612	VPS4A	16	69311356	69326939	protein_coding	27,99	0

ENSG00000178700	DHFRL1	3	94047836	94063389	protein_coding	27,99	0
ENSG00000168763	CNNM3	2	96816245	96833911	protein_coding	27,99	0
ENSG00000243716	NPIPB5	16	22479121	22536521	protein_coding	27,96	0
ENSG00000010322	NISCH	3	52455118	52493071	protein_coding	27,95	0
ENSG00000115825	PRKD3	2	37250502	37324808	protein_coding	27,93	0
ENSG00000103365	GGA2	16	23463542	23521995	protein_coding	27,92	0
ENSG00000164631	ZNF12	7	6688433	6706923	protein_coding	27,92	0
ENSG00000206203	TSSK2	22	19130808	19132623	protein_coding	27,90	0
ENSG00000167799	NUDT8	11	67627938	67629930	protein_coding	27,90	0
ENSG00000164591	MYOZ3	5	150660874	150679365	protein_coding	27,90	0
ENSG00000241489	ENSG00000	X	149482543	149533935	protein_coding	27,90	0
ENSG00000139117	CPNE8	12	38646822	38907430	protein_coding	27,89	0
ENSG00000164066	INTU	4	127623271	127726737	protein_coding	27,88	0
ENSG00000131043	AAR2	20	36236459	36270918	protein_coding	27,82	0
ENSG00000023191	RNH1	11	494512	507300	protein_coding	27,82	0
ENSG00000110104	CCDC86	11	60842071	60851081	protein_coding	27,81	0
ENSG00000150593	PDCD4	10	110871795	110900006	protein_coding	27,80	0
ENSG00000163945	UVSSA	4	1347266	1388049	protein_coding	27,79	0
ENSG00000122729	ACO1	9	32384603	32454769	protein_coding	27,79	0
ENSG00000196693	ZNF33B	10	42574185	42638568	protein_coding	27,79	0
ENSG00000179632	MAF1	8	144104499	144107611	protein_coding	27,78	0
ENSG00000215790	SLC35E2	1	1724838	1745992	protein_coding	27,75	0
ENSG00000105953	OGDH	7	44606572	44709066	protein_coding	27,74	0
ENSG00000144857	BOC	3	113211003	113287459	protein_coding	27,73	0
ENSG00000135637	CCDC142	2	74471986	74483408	protein_coding	27,72	0
ENSG00000270629	NBPF14	1	148531385	148679751	protein_coding	27,71	0
ENSG00000158864	NDUFS2	1	161197104	161214395	protein_coding	27,70	0
ENSG00000105656	ELL	19	18442663	18522127	protein_coding	27,67	0
ENSG00000103227	LMF1	16	853634	981318	protein_coding	27,66	0
ENSG00000129355	CDKN2D	19	10566462	10569059	protein_coding	27,65	0
ENSG00000164176	EDIL3	5	83940554	84384793	protein_coding	27,65	0
ENSG00000051009	FAM160A2	11	6211335	6234711	protein_coding	27,64	0
ENSG00000224982	TMEM233	12	119593459	119643066	protein_coding	27,64	0
ENSG00000128309	MPST	22	37019635	37029822	protein_coding	27,61	0
ENSG00000215277	RNF212B	14	23185316	23273477	protein_coding	27,60	0
ENSG00000135437	RDH5	12	55720367	55724705	protein_coding	27,60	0
ENSG00000137441	FGFBP2	4	15960243	15969309	protein_coding	27,60	0
ENSG00000006194	ZNF263	16	3263800	3301401	protein_coding	27,58	0
ENSG00000170634	ACYP2	2	53970838	54305300	protein_coding	27,56	0
ENSG00000119699	TGFB3	14	75958099	75982991	protein_coding	27,53	0
ENSG00000170500	LONRF2	2	100273291	100322733	protein_coding	27,53	0
ENSG00000120820	GLT8D2	12	103988984	104064183	protein_coding	27,50	0
ENSG00000100038	TOP3B	22	21957025	21982816	protein_coding	27,48	0
ENSG00000145743	FBXL17	5	107859035	108382098	protein_coding	27,44	0
ENSG00000205221	VIT	2	36696690	36814792	protein_coding	27,42	0
ENSG00000117425	PTCH2	1	44819844	44843063	protein_coding	27,41	0
ENSG00000100353	EIF3D	22	36510850	36529436	protein_coding	27,40	0
ENSG00000124251	TP53TG5	20	45372563	45407889	protein_coding	27,40	0
ENSG00000203667	COX20	1	244835322	244845057	protein_coding	27,39	0
ENSG00000143889	HNRNPLL	2	38561978	38603586	protein_coding	27,31	0
ENSG00000164850	GPER1	7	1082208	1093815	protein_coding	27,30	0
ENSG00000122692	SMU1	9	33041764	33076659	protein_coding	27,29	0
ENSG00000145685	LHFPL2	5	78485215	78770021	protein_coding	27,25	0
ENSG00000104974	LILRA1	19	54573879	54602090	protein_coding	27,20	0
ENSG00000270885	RASL10B	17	35731649	35743521	protein_coding	27,20	0
ENSG00000170075	GPR37L1	1	202122858	202133592	protein_coding	27,20	0
ENSG00000135766	EGLN1	1	231363751	231425044	protein_coding	27,19	0
ENSG00000136104	RNASEH2B	13	50909678	50973745	protein_coding	27,19	0

ENSG00000105875	WDR91	7	135183839	135211534	protein_coding	27,19	0
ENSG00000170175	CHRNB1	17	7445061	7457707	protein_coding	27,17	0
ENSG00000144031	ANKRD53	2	70978380	70985499	protein_coding	27,14	0
ENSG00000182771	GRID1	10	85599555	86366493	protein_coding	27,10	0
ENSG00000215045	GRID2IP	7	6497462	6551436	protein_coding	27,10	0
ENSG00000037280	FLT4	5	180601506	180649624	protein_coding	27,10	0
ENSG00000169435	RASSF6	4	73571550	73620631	protein_coding	27,10	0
ENSG00000185436	IFNLR1	1	24154157	24187959	protein_coding	27,10	0
ENSG00000116017	ARID3A	19	925781	975934	protein_coding	27,07	0
ENSG00000167216	KATNAL2	18	46917492	47102243	protein_coding	27,07	0
ENSG00000122482	ZNF644	1	90915298	91022272	protein_coding	27,03	0
ENSG00000164342	TLR3	4	186069152	186088069	protein_coding	27,02	0
ENSG00000102230	PCYT1B	X	24558087	24672677	protein_coding	27,01	0
ENSG00000151773	CCDC122	13	43823909	43879727	protein_coding	27,00	0
ENSG00000232112	TMA7	3	48440257	48444208	protein_coding	26,96	0
ENSG00000184857	TMEM186	16	8780384	8797648	protein_coding	26,95	0
ENSG00000139826	ABHD13	13	108218379	108234255	protein_coding	26,94	0
ENSG00000101474	APMAP	20	24962925	24992979	protein_coding	26,92	0
ENSG00000188818	ZDHHC11	5	795606	850986	protein_coding	26,92	0
ENSG00000143373	ZNF687	1	151281618	151292180	protein_coding	26,90	0
ENSG00000126749	EMG1	12	6970893	6979941	protein_coding	26,86	0
ENSG00000197265	GTF2E2	8	30578318	30658251	protein_coding	26,84	0
ENSG00000134072	CAMK1	3	9757342	9769992	protein_coding	26,81	0
ENSG00000072121	ZFYVE26	14	67727374	67816590	protein_coding	26,79	0
ENSG00000221829	FANCG	9	35073835	35080016	protein_coding	26,78	0
ENSG00000243279	PRAF2	X	49071156	49074071	protein_coding	26,77	0
ENSG00000141905	NFIC	19	3359563	3469217	protein_coding	26,76	0
ENSG00000162714	ZNF496	1	247297412	247331846	protein_coding	26,75	0
ENSG00000152291	TGOLN2	2	85318020	85328425	protein_coding	26,74	0
ENSG00000169972	PUSL1	1	1308567	1311677	protein_coding	26,73	0
ENSG00000100934	SEC23A	14	39031919	39109646	protein_coding	26,71	0
ENSG00000171126	KCNG3	2	42442017	42494097	protein_coding	26,71	0
ENSG00000187566	NHLRC1	6	18121419	18122606	protein_coding	26,70	0
ENSG00000105402	NAPA	19	47487637	47515240	protein_coding	26,68	0
ENSG00000091409	ITGA6	2	172427354	172506282	protein_coding	26,65	0
ENSG00000276547	PCDHGB5	5	141397987	141512979	protein_coding	26,63	0
ENSG00000111897	SERINC1	6	122443354	122471822	protein_coding	26,61	0
ENSG00000134242	PTPN22	1	113813811	113871759	protein_coding	26,60	0
ENSG00000186787	SPIN2B	X	57118551	57121547	protein_coding	26,60	0
ENSG00000168653	NDUFS5	1	39026318	39034636	protein_coding	26,56	0
ENSG00000081791	KIAA0141	5	141923808	141942047	protein_coding	26,55	0
ENSG00000105926	MPP6	7	24573268	24694193	protein_coding	26,54	0
ENSG00000152465	NMT2	10	15102584	15168693	protein_coding	26,53	0
ENSG00000067057	PFKP	10	3066333	3137712	protein_coding	26,52	0
ENSG00000221869	CEBPD	8	47736909	47739086	protein_coding	26,52	0
ENSG00000050555	LAMC3	9	131009082	131094473	protein_coding	26,52	0
ENSG00000059915	PSD	10	102402617	102421539	protein_coding	26,51	0
ENSG00000135341	MAP3K7	6	90513573	90587045	protein_coding	26,51	0
ENSG00000204516	MICB	6	31494881	31511124	protein_coding	26,50	0
ENSG00000163660	CCNL1	3	157146508	157160760	protein_coding	26,42	0
ENSG00000104967	NOVA2	19	45933734	45973546	protein_coding	26,40	0
ENSG00000129749	CHRNA10	11	3665587	3671384	protein_coding	26,40	0
ENSG00000161888	SPC24	19	11131520	11155808	protein_coding	26,39	0
ENSG00000196843	ARID5A	2	96536743	96552638	protein_coding	26,38	0
ENSG00000134717	BTF3L4	1	52056125	52090716	protein_coding	26,37	0
ENSG00000167123	CERCAM	9	128411751	128437351	protein_coding	26,36	0
ENSG00000165732	DDX21	10	68956128	68985073	protein_coding	26,35	0
ENSG00000125510	OPRL1	20	64080173	64100643	protein_coding	26,33	0

ENSG00000060558	GNA15	19	3136193	3163769	protein_coding	26,30	0
ENSG00000091513	TF	3	133745956	133779006	protein_coding	26,30	0
ENSG00000183354	KIAA2026	9	5881596	6007901	protein_coding	26,24	0
ENSG00000234719	ENSG00000	16	11927373	11976643	protein_coding	26,23	0
ENSG00000157551	KCNJ15	21	38157034	38307357	protein_coding	26,20	0
ENSG00000090659	CD209	19	7739994	7747564	protein_coding	26,20	0
ENSG00000105671	DDX49	19	18919675	18928633	protein_coding	26,20	0
ENSG00000103512	NOMO1	16	14833681	14896160	protein_coding	26,18	0
ENSG00000203668	CHML	1	241628853	241635930	protein_coding	26,17	0
ENSG00000241258	CRCP	7	66114604	66154568	protein_coding	26,16	0
ENSG00000104915	STX10	19	13144058	13150383	protein_coding	26,16	0
ENSG00000105642	KCNN1	19	17951293	18000080	protein_coding	26,16	0
ENSG00000159131	GART	21	33503931	33543491	protein_coding	26,15	0
ENSG00000099783	HNRNPM	19	8444767	8489114	protein_coding	26,15	0
ENSG00000243335	KCTD7	7	66628881	66811187	protein_coding	26,14	0
ENSG00000274897	PANO1	11	797511	799190	protein_coding	26,10	0
ENSG00000162267	ITIH3	3	52794768	52809009	protein_coding	26,10	0
ENSG00000010165	METTL13	1	171781664	171814023	protein_coding	26,10	0
ENSG00000008323	PLEKHG6	12	6310436	6328506	protein_coding	26,09	0
ENSG00000122068	FYTTD1	3	197737179	197787596	protein_coding	26,06	0
ENSG00000167604	NFKBID	19	35887653	35902303	protein_coding	26,05	0
ENSG00000197343	ZNF655	7	99558406	99576453	protein_coding	26,05	0
ENSG00000184984	CHRM5	15	33968720	34067457	protein_coding	26,00	0
ENSG00000257594	GALNT4	12	89519408	89524806	protein_coding	26,00	0
ENSG00000152558	TMEM123	11	102396332	102470384	protein_coding	26,00	0
ENSG00000185670	ZBTB3	11	62748319	62754188	protein_coding	25,95	0
ENSG00000144891	AGTR1	3	148697784	148743008	protein_coding	25,92	0
ENSG00000180525	PRR26	10	649948	669581	protein_coding	25,90	0
ENSG00000113504	SLC12A7	5	1050376	1112035	protein_coding	25,89	0
ENSG00000103423	DNAJA3	16	4425805	4456775	protein_coding	25,89	0
ENSG00000084444	FAM234B	12	13044284	13142521	protein_coding	25,86	0
ENSG00000187650	VMAC	19	5904858	5910853	protein_coding	25,86	0
ENSG00000214309	MBLAC1	7	100126694	100128498	protein_coding	25,86	0
ENSG00000185803	SLC52A2	8	144354135	144361272	protein_coding	25,85	0
ENSG00000162664	ZNF326	1	89995112	90035531	protein_coding	25,83	0
ENSG00000185236	RAB11B	19	8389981	8404434	protein_coding	25,81	0
ENSG00000081853	PCDHGA2	5	141338760	141512979	protein_coding	25,80	0
ENSG00000179528	LBX2	2	74497517	74503316	protein_coding	25,80	0
ENSG00000198758	EPS8L3	1	109750080	109764027	protein_coding	25,80	0
ENSG00000145782	ATG12	5	115828200	115841858	protein_coding	25,77	0
ENSG00000102302	FGD1	X	54445454	54496166	protein_coding	25,77	0
ENSG00000269713	NBPF9	1	149054027	149103561	protein_coding	25,76	0
ENSG00000117597	DIEXF	1	209828007	209857565	protein_coding	25,76	0
ENSG00000187446	CHP1	15	41230839	41281890	protein_coding	25,74	0
ENSG00000177045	SIX5	19	45764785	45769226	protein_coding	25,73	0
ENSG00000114021	NIT2	3	100334701	100361635	protein_coding	25,72	0
ENSG00000134759	ELP2	18	36129444	36180556	protein_coding	25,72	0
ENSG00000108010	GLRX3	10	130136399	130184521	protein_coding	25,72	0
ENSG00000161929	SCIMP	17	5208961	5234860	protein_coding	25,71	0
ENSG00000100030	MAPK1	22	21754500	21867680	protein_coding	25,68	0
ENSG00000171766	GATM	15	45361124	45402327	protein_coding	25,67	0
ENSG00000169018	FEM1B	15	68277803	68295865	protein_coding	25,67	0
ENSG00000228253	MT-ATP8	X	8366	8572	protein_coding	25,67	0
ENSG00000053702	NRIP2	12	2825348	2835544	protein_coding	25,64	0
ENSG00000166780	C16orf45	16	15434295	15625028	protein_coding	25,63	0
ENSG00000170345	FOS	14	75278774	75282230	protein_coding	25,61	0
ENSG00000181029	TRAPPC5	19	7680843	7687703	protein_coding	25,60	0
ENSG00000067113	PLPP1	5	55424854	55535050	protein_coding	25,58	0

ENSG00000145014	TMEM44	3	194587673	194633689	protein_coding	25,55	0
ENSG00000203710	CR1	1	207496147	207640647	protein_coding	25,53	0
ENSG00000250571	GLI4	8	143267433	143276931	protein_coding	25,51	0
ENSG00000271303	SRXN1	20	646615	653370	protein_coding	25,50	0
ENSG00000168329	CX3CR1	3	39263494	39281735	protein_coding	25,50	0
ENSG00000102317	RBM3	X	48574449	48579066	protein_coding	25,47	0
ENSG00000130985	UBA1	X	47190861	47215128	protein_coding	25,47	0
ENSG00000141699	FAM134C	17	42579513	42610623	protein_coding	25,46	0
ENSG00000146966	DENND2A	7	140518420	140673993	protein_coding	25,45	0
ENSG00000130723	PRRC2B	9	131394093	131500197	protein_coding	25,40	0
ENSG00000239998	LILRA2	19	54572920	54590287	protein_coding	25,40	0
ENSG00000136457	CHAD	17	50464496	50468966	protein_coding	25,40	0
ENSG00000049192	ADAMTS6	5	65148736	65481920	protein_coding	25,40	0
ENSG00000048405	ZNF800	7	127346790	127431924	protein_coding	25,38	0
ENSG00000100983	GSS	20	34928430	34955817	protein_coding	25,36	0
ENSG00000198589	LRBA	4	150264531	151015727	protein_coding	25,36	0
ENSG00000134744	ZCCHC11	1	52408282	52553487	protein_coding	25,36	0
ENSG00000104825	NFKBIB	19	38899700	38908893	protein_coding	25,34	0
ENSG00000134153	EMC7	15	34084017	34101948	protein_coding	25,34	0
ENSG00000187678	SPRY4	5	142310427	142326455	protein_coding	25,32	0
ENSG00000164031	DNAJB14	4	99896248	99946726	protein_coding	25,30	0
ENSG00000166803	KIAA0101	15	64364311	64387687	protein_coding	25,30	0
ENSG00000181929	PRKAG1	12	49002274	49019197	protein_coding	25,29	0
ENSG00000261150	EPPK1	8	143857324	143878464	protein_coding	25,29	0
ENSG00000105819	PMPCB	7	103297422	103329511	protein_coding	25,28	0
ENSG00000203705	TATDN3	1	212791828	212816626	protein_coding	25,26	0
ENSG00000022267	FHL1	X	136146702	136211359	protein_coding	25,26	0
ENSG00000179886	TIGD5	8	143597835	143603224	protein_coding	25,24	0
ENSG00000101546	RBFA	18	80034358	80046397	protein_coding	25,24	0
ENSG00000236287	ZBED5	11	10812074	10858796	protein_coding	25,22	0
ENSG00000185722	ANKFY1	17	4163907	4263977	protein_coding	25,20	0
ENSG00000184602	SNN	16	11668414	11679159	protein_coding	25,17	0
ENSG00000248098	BCKDHA	19	41397460	41431345	protein_coding	25,16	0
ENSG00000106086	PLEKHA8	7	30027404	30130483	protein_coding	25,14	0
ENSG00000204220	PFDN6	6	33289302	33298401	protein_coding	25,14	0
ENSG00000197386	HTT	4	3074681	3243959	protein_coding	25,13	0
ENSG00000167468	GPX4	19	1103926	1106791	protein_coding	25,12	0
ENSG00000144130	NT5DC4	2	112721486	112742879	protein_coding	25,11	0
ENSG00000105607	GCDH	19	12891026	12914207	protein_coding	25,11	0
ENSG00000197448	GSTK1	7	143244093	143270854	protein_coding	25,08	0
ENSG00000143486	EIF2D	1	206571292	206612463	protein_coding	25,04	0
ENSG00000150401	DCUN1D2	13	113455819	113490952	protein_coding	25,03	0
ENSG00000102547	CAB39L	13	49308650	49444126	protein_coding	25,01	0
ENSG00000196209	SIRPB2	20	1470741	1491587	protein_coding	25,00	0
ENSG00000126266	FFAR1	19	35351552	35353862	protein_coding	25,00	0
ENSG00000276085	CCL3L3	17	36194869	36196758	protein_coding	25,00	0
ENSG00000259529	ENSG00000	14	24151218	24167402	protein_coding	25,00	0
ENSG00000160213	CSTB	21	43772512	43776445	protein_coding	24,98	0
ENSG00000184787	UBE2G2	21	44768580	44802019	protein_coding	24,98	0
ENSG00000121653	MAPK8IP1	11	45885651	45906465	protein_coding	24,94	0
ENSG00000188997	KCTD21	11	78171249	78188822	protein_coding	24,94	0
ENSG00000137710	RDX	11	110174880	110296722	protein_coding	24,93	0
ENSG00000103196	CRISPLD2	16	84819984	84920768	protein_coding	24,91	0
ENSG00000173825	TIGD3	11	65354767	65357613	protein_coding	24,90	0
ENSG00000228300	C19orf24	19	1275438	1279249	protein_coding	24,89	0
ENSG00000166845	C18orf54	18	54357917	54385218	protein_coding	24,88	0
ENSG00000161681	SHANK1	19	50661827	50719450	protein_coding	24,85	0
ENSG00000242259	C22orf39	22	19351368	19448232	protein_coding	24,82	0

ENSG00000168490	PHYHIP	8	22219704	22232341	protein_coding	24,80	0
ENSG00000167131	CCDC103	17	44899142	44905390	protein_coding	24,79	0
ENSG00000105792	CFAP69	7	90245174	90311063	protein_coding	24,78	0
ENSG00000018236	CNTN1	12	40692442	41072418	protein_coding	24,78	0
ENSG00000053254	FOXN3	14	89124871	89619149	protein_coding	24,77	0
ENSG00000127603	MACF1	1	39081316	39487177	protein_coding	24,72	0
ENSG00000162769	FLVCR1	1	212858255	212899363	protein_coding	24,71	0
ENSG00000019144	PHLDB1	11	118606440	118658038	protein_coding	24,71	0
ENSG00000173715	C11orf80	11	66744451	66843328	protein_coding	24,70	0
ENSG00000189298	ZKSCAN3	6	28349914	28369177	protein_coding	24,70	0
ENSG00000250506	CDK3	17	76000906	76005999	protein_coding	24,70	0
ENSG00000197409	HIST1H3D	6	26196840	26197250	protein_coding	24,70	0
ENSG00000198342	ZNF442	19	12345949	12365905	protein_coding	24,69	0
ENSG00000103335	PIEZO1	16	88715343	88785211	protein_coding	24,67	0
ENSG00000013392	RWDD2A	6	83193379	83198932	protein_coding	24,67	0
ENSG00000168924	LETM1	4	1811479	1856247	protein_coding	24,66	0
ENSG00000213760	ATP6V1G2	6	31544462	31548427	protein_coding	24,65	0
ENSG00000184481	FOXO4	X	71096197	71103535	protein_coding	24,63	0
ENSG00000188542	DUSP28	2	240560054	240564014	protein_coding	24,62	0
ENSG00000151778	SERP2	13	44373665	44397714	protein_coding	24,61	0
ENSG00000177830	CHID1	11	867860	915058	protein_coding	24,60	0
ENSG00000100429	HDAC10	22	50245183	50251405	protein_coding	24,60	0
ENSG00000154760	SLFN13	17	35435096	35448837	protein_coding	24,60	0
ENSG00000182575	NXPH3	17	49575858	49583827	protein_coding	24,60	0
ENSG00000275302	CCL4	17	36103590	36105621	protein_coding	24,60	0
ENSG00000139540	SLC39A5	12	56230049	56237846	protein_coding	24,60	0
ENSG00000011009	LYPLA2	1	23790970	23795539	protein_coding	24,59	0
ENSG00000091536	MYO15A	17	18108706	18179802	protein_coding	24,59	0
ENSG00000221978	CCNL2	1	1385711	1399328	protein_coding	24,58	0
ENSG00000186818	LILRB4	19	54643889	54670359	protein_coding	24,58	0
ENSG00000112852	PCDHB2	5	141094578	141098703	protein_coding	24,58	0
ENSG00000109929	SC5D	11	121292453	121308694	protein_coding	24,57	0
ENSG00000145022	TCTA	3	49412206	49416475	protein_coding	24,56	0
ENSG00000136237	RAPGEF5	7	22118238	22357144	protein_coding	24,55	0
ENSG00000130734	ATG4D	19	10543895	10553418	protein_coding	24,53	0
ENSG00000171150	SOCS5	2	46698952	46763129	protein_coding	24,52	0
ENSG00000152082	MZT2B	2	130181737	130190729	protein_coding	24,52	0
ENSG00000119684	MLH3	14	75013764	75051532	protein_coding	24,51	0
ENSG00000112183	RBM24	6	17281346	17293875	protein_coding	24,50	0
ENSG00000253953	PCDHGB4	5	141387698	141512979	protein_coding	24,50	0
ENSG00000197217	ENTPD4	8	23385783	23457695	protein_coding	24,48	0
ENSG00000185009	AP3M1	10	74120255	74151063	protein_coding	24,48	0
ENSG00000131236	CAP1	1	40040233	40072649	protein_coding	24,48	0
ENSG00000160214	RRP1	21	43789513	43805293	protein_coding	24,46	0
ENSG00000133687	TMTC1	12	29500840	29784759	protein_coding	24,44	0
ENSG00000177300	CLDN22	4	183318194	183320774	protein_coding	24,44	0
ENSG00000126107	HECTD3	1	45002540	45011329	protein_coding	24,42	0
ENSG00000183426	NPIPA1	16	14750813	14952060	protein_coding	24,40	0
ENSG00000036828	CASR	3	122183683	122286503	protein_coding	24,40	0
ENSG00000205090	TMEM240	1	1535174	1540453	protein_coding	24,40	0
ENSG00000181404	WASH1	9	14521	29739	protein_coding	24,38	0
ENSG00000012660	ELOVL5	6	53267398	53349179	protein_coding	24,38	0
ENSG00000174456	C12orf76	12	110027028	110073686	protein_coding	24,37	0
ENSG00000007923	DNAJC11	1	6634168	6701924	protein_coding	24,35	0
ENSG00000167720	SRR	17	2303383	2325260	protein_coding	24,35	0
ENSG00000143537	ADAM15	1	155050566	155062775	protein_coding	24,34	0
ENSG00000079246	XRCC5	2	216107464	216206303	protein_coding	24,34	0
ENSG00000141295	SCRN2	17	47837692	47841333	protein_coding	24,33	0

ENSG00000204590	GNL1	6	30541377	30557174	protein_coding	24,32	0
ENSG00000162889	MAPKAPK2	1	206684944	206734283	protein_coding	24,32	0
ENSG00000198369	SPRED2	2	65310851	65432637	protein_coding	24,31	0
ENSG00000137814	HAUS2	15	42548810	42569994	protein_coding	24,31	0
ENSG00000110871	COQ5	12	120503274	120534434	protein_coding	24,31	0
ENSG00000227877	MRLN	10	59736692	59756041	protein_coding	24,30	0
ENSG00000261934	PCDHGA9	5	141402932	141512979	protein_coding	24,30	0
ENSG00000171385	KCND3	1	111770662	111989155	protein_coding	24,30	0
ENSG00000167930	FAM234A	16	234546	268971	protein_coding	24,29	0
ENSG00000114982	KANSL3	2	96593170	96642787	protein_coding	24,26	0
ENSG00000147789	ZNF7	8	144827464	144847509	protein_coding	24,26	0
ENSG00000096093	EFHC1	6	52420308	52495785	protein_coding	24,26	0
ENSG00000179044	EXOC3L1	16	67184366	67190204	protein_coding	24,24	0
ENSG00000133703	KRAS	12	25204789	25250936	protein_coding	24,23	0
ENSG00000198843	ENSG00000	3	150602875	150630445	protein_coding	24,23	0
ENSG00000100314	CABP7	22	29720084	29731839	protein_coding	24,23	0
ENSG00000143157	POGK	1	166839447	166856344	protein_coding	24,22	0
ENSG00000213780	GTF2H4	6	30908184	30914106	protein_coding	24,20	0
ENSG00000162882	HAAO	2	42767089	42792593	protein_coding	24,20	0
ENSG00000215704	CELA2B	1	15465909	15491400	protein_coding	24,20	0
ENSG00000172339	ALG14	1	94974407	95072945	protein_coding	24,20	0
ENSG00000114554	PLXNA1	3	126988594	127037392	protein_coding	24,19	0
ENSG00000179348	GATA2	3	128479427	128493185	protein_coding	24,18	0
ENSG00000150712	MTMR12	5	32226994	32313009	protein_coding	24,17	0
ENSG00000105726	ATP13A1	19	19645198	19663693	protein_coding	24,17	0
ENSG00000197948	FCHSD1	5	141639302	141651419	protein_coding	24,16	0
ENSG00000138738	PRDM5	4	120684919	120922870	protein_coding	24,12	0
ENSG00000171357	LURAP1	1	46203334	46221261	protein_coding	24,11	0
ENSG00000011485	PPP5C	19	46346994	46392981	protein_coding	24,10	0
ENSG00000121410	A1BG	19	58345178	58353499	protein_coding	24,10	0
ENSG00000171903	CYP4F11	19	15912367	15934867	protein_coding	24,10	0
ENSG00000187987	ZSCAN23	6	28431930	28443502	protein_coding	24,10	0
ENSG00000165868	HSPA12A	10	116671192	116742574	protein_coding	24,09	0
ENSG00000187796	CARD9	9	136361903	136373681	protein_coding	24,08	0
ENSG00000180938	ZNF572	8	124973298	124979389	protein_coding	24,08	0
ENSG00000092964	DPYSL2	8	26514275	26658178	protein_coding	24,08	0
ENSG00000177200	CHD9	16	53055033	53329150	protein_coding	24,07	0
ENSG00000136813	KIAA0368	9	111360692	111484745	protein_coding	24,05	0
ENSG00000065427	KARS	16	75627474	75648643	protein_coding	24,04	0
ENSG00000181045	SLC26A11	17	80219699	80253500	protein_coding	24,02	0
ENSG00000076344	RGS11	16	268301	275980	protein_coding	24,00	0
ENSG00000185585	OLFML2A	9	124777158	124814885	protein_coding	24,00	0
ENSG00000080224	EPHA6	3	96814581	97752460	protein_coding	24,00	0
ENSG00000187583	PLEKHN1	1	966497	975865	protein_coding	24,00	0
ENSG00000011304	PTBP1	19	797075	812327	protein_coding	24,00	0
ENSG00000080031	PTPRH	19	55181248	55209506	protein_coding	23,98	0
ENSG00000117868	ESYT2	7	158730995	158830253	protein_coding	23,97	0
ENSG00000141449	GREB1L	18	21242242	21525417	protein_coding	23,96	0
ENSG00000122694	GLIPR2	9	36136536	36163913	protein_coding	23,96	0
ENSG00000119812	FAM98A	2	33583658	33599382	protein_coding	23,94	0
ENSG00000178401	DNAJC22	12	49346917	49357546	protein_coding	23,92	0
ENSG00000186496	ZNF396	18	35366697	35377337	protein_coding	23,91	0
ENSG00000168454	TXNDC2	18	9885726	9889275	protein_coding	23,90	0
ENSG00000187800	PEAR1	1	156893698	156916434	protein_coding	23,90	0
ENSG00000122376	FAM35A	10	87094161	87191468	protein_coding	23,89	0
ENSG00000264364	DYNLL2	17	58083415	58095536	protein_coding	23,87	0
ENSG00000204427	ABHD16A	6	31686949	31703444	protein_coding	23,86	0
ENSG00000169718	DUS1L	17	82057506	82065887	protein_coding	23,83	0

ENSG00000102158	MAGT1	X	77826364	77895593	protein_coding	23,81	0
ENSG00000100304	TTLL12	22	43166622	43187133	protein_coding	23,81	0
ENSG00000145632	PLK2	5	58453982	58460260	protein_coding	23,80	0
ENSG00000188257	PLA2G2A	1	19975431	19980416	protein_coding	23,80	0
ENSG00000269113	TRABD2B	1	47760528	47996895	protein_coding	23,80	0
ENSG00000170027	YWHAG	7	76326794	76359031	protein_coding	23,79	0
ENSG00000162458	FBLIM1	1	15756607	15786594	protein_coding	23,79	0
ENSG00000185716	C16orf52	16	22007638	22087534	protein_coding	23,79	0
ENSG00000176490	DIRAS1	19	2714567	2721418	protein_coding	23,77	0
ENSG00000108830	RND2	17	43025241	43032036	protein_coding	23,73	0
ENSG00000214087	ARL16	17	81681174	81683924	protein_coding	23,73	0
ENSG00000166484	MAPK7	17	19377721	19383544	protein_coding	23,73	0
ENSG00000159176	CSRP1	1	201483530	201509456	protein_coding	23,73	0
ENSG00000113555	PCDH12	5	141943585	141969741	protein_coding	23,71	0
ENSG00000169905	TOR1AIP2	1	179839967	179877803	protein_coding	23,70	0
ENSG00000143624	INTS3	1	153728067	153774808	protein_coding	23,70	0
ENSG00000161526	SAP30BP	17	75667116	75708062	protein_coding	23,70	0
ENSG00000138073	PREB	2	27130756	27134675	protein_coding	23,68	0
ENSG00000123159	GIPC1	19	14477760	14496149	protein_coding	23,68	0
ENSG00000100401	RANGAP1	22	41245611	41286251	protein_coding	23,66	0
ENSG00000124615	MOCS1	6	39899578	39934551	protein_coding	23,65	0
ENSG00000182934	SRPR	11	126262919	126269144	protein_coding	23,65	0
ENSG00000165417	GTF2A1	14	81175452	81221377	protein_coding	23,64	0
ENSG00000084693	AGBL5	2	27042364	27070622	protein_coding	23,63	0
ENSG00000100319	ZMAT5	22	29730956	29767011	protein_coding	23,62	0
ENSG00000114757	PEX5L	3	179794958	180037053	protein_coding	23,62	0
ENSG00000184908	CLCNKB	1	16043736	16057308	protein_coding	23,60	0
ENSG00000187079	TEAD1	11	12674591	12944483	protein_coding	23,58	0
ENSG00000134910	STT3A	11	125591712	125625215	protein_coding	23,57	0
ENSG00000198890	PRMT6	1	107056679	107059294	protein_coding	23,56	0
ENSG00000138095	LRPPRC	2	43886508	43996005	protein_coding	23,54	0
ENSG00000152443	ZNF776	19	57746796	57758159	protein_coding	23,52	0
ENSG00000205560	CPT1B	22	50568861	50578465	protein_coding	23,50	0
ENSG00000151458	ANKRD50	4	124664052	124712732	protein_coding	23,46	0
ENSG00000185000	DGAT1	8	144314584	144326910	protein_coding	23,43	0
ENSG00000177302	TOP3A	17	18271428	18315007	protein_coding	23,43	0
ENSG00000104884	ERCC2	19	45349837	45370918	protein_coding	23,42	0
ENSG00000167395	ZNF646	16	31074422	31084196	protein_coding	23,40	0
ENSG00000176171	BNIP3	10	131966455	131982013	protein_coding	23,39	0
ENSG00000151117	TMEM86A	11	18693122	18704785	protein_coding	23,39	0
ENSG00000159692	CTBP1	4	1211448	1249953	protein_coding	23,39	0
ENSG00000108883	EFTUD2	17	44849943	44899662	protein_coding	23,39	0
ENSG00000165684	SNAPC4	9	136375577	136398797	protein_coding	23,37	0
ENSG00000167700	MFSD3	8	144509074	144511213	protein_coding	23,37	0
ENSG00000188677	PARVB	22	43999211	44172949	protein_coding	23,36	0
ENSG00000027075	PRKCH	14	61187559	61550976	protein_coding	23,35	0
ENSG00000203950	FAM127B	X	135050932	135052196	protein_coding	23,34	0
ENSG00000213930	GALT	9	34638133	34651035	protein_coding	23,31	0
ENSG00000143321	HDGF	1	156742107	156766925	protein_coding	23,30	0
ENSG00000119711	ALDH6A1	14	74056850	74084493	protein_coding	23,29	0
ENSG00000165410	CFL2	14	34706769	34714823	protein_coding	23,27	0
ENSG00000165752	STK32C	10	132207492	132331847	protein_coding	23,27	0
ENSG00000172273	HINFP	11	119121587	119136044	protein_coding	23,26	0
ENSG00000140632	GLYR1	16	4803203	4847342	protein_coding	23,24	0
ENSG00000172671	ZFAND4	10	45615501	45672780	protein_coding	23,24	0
ENSG00000112667	DNPH1	6	43225629	43229484	protein_coding	23,21	0
ENSG00000173926	03-mars	5	126867714	127030808	protein_coding	23,21	0
ENSG00000033627	ATP6V0A1	17	42458844	42522611	protein_coding	23,21	0

ENSG00000178789	CD300LB	17	74521174	74531474	protein_coding	23,20	0
ENSG00000112299	VNN1	6	132681590	132714049	protein_coding	23,20	0
ENSG00000120328	PCDHB12	5	141208697	141212571	protein_coding	23,20	0
ENSG00000100418	DESI1	22	41598028	41621096	protein_coding	23,20	0
ENSG00000138303	ASCC1	10	72096032	72217134	protein_coding	23,19	0
ENSG00000054983	GALC	14	87837820	87993665	protein_coding	23,19	0
ENSG00000014919	COX15	10	99711844	99732100	protein_coding	23,16	0
ENSG00000160201	U2AF1	21	43092956	43107587	protein_coding	23,16	0
ENSG00000092531	SNAP23	15	42491233	42545356	protein_coding	23,14	0
ENSG00000168393	DTYMK	2	241675742	241686991	protein_coding	23,12	0
ENSG00000197837	HIST4H4	12	14767999	14771131	protein_coding	23,10	0
ENSG00000214290	COLCA2	11	111298546	111308735	protein_coding	23,09	0
ENSG00000138029	HADHB	2	26243170	26290468	protein_coding	23,09	0
ENSG00000166676	TVP23A	16	10760919	10818794	protein_coding	23,08	0
ENSG00000183628	DGCR6	22	18906028	18914238	protein_coding	23,07	0
ENSG00000108528	SLC25A11	17	4937130	4940251	protein_coding	23,04	0
ENSG00000166333	ILK	11	6603708	6610874	protein_coding	23,04	0
ENSG00000185624	P4HB	17	81843159	81860694	protein_coding	23,04	0
ENSG00000149084	HSD17B12	11	43556436	43856617	protein_coding	23,03	0
ENSG00000205609	EIF3CL	16	28379579	28403879	protein_coding	23,00	0
ENSG00000117308	GALE	1	23795599	23800804	protein_coding	23,00	0
ENSG00000281741	ENSG00000	1	120913151	120914238	protein_coding	23,00	0
ENSG00000100104	SRRD	22	26483877	26494658	protein_coding	22,99	0
ENSG00000115204	MPV17	2	27309492	27325680	protein_coding	22,99	0
ENSG00000007171	NOS2	17	27756766	27800499	protein_coding	22,96	0
ENSG00000076053	RBM7	11	114400030	114414203	protein_coding	22,94	0
ENSG00000102870	ZNF629	16	30778449	30787202	protein_coding	22,91	0
ENSG00000273259	ENSG00000	14	94592058	94624646	protein_coding	22,90	0
ENSG00000165457	FOLR2	11	72216601	72221950	protein_coding	22,90	0
ENSG00000168334	XIRP1	3	39183210	39192596	protein_coding	22,90	0
ENSG00000160325	CACFD1	9	133459965	133470848	protein_coding	22,89	0
ENSG00000178952	TUFM	16	28842411	28846408	protein_coding	22,87	0
ENSG00000143845	ETNK2	1	204131062	204152003	protein_coding	22,84	0
ENSG00000144381	HSPD1	2	197486581	197516737	protein_coding	22,80	0
ENSG00000114867	EIF4G1	3	184314495	184335358	protein_coding	22,80	0
ENSG00000177576	C18orf32	18	49477250	49487252	protein_coding	22,80	0
ENSG00000183682	BMP8A	1	39491646	39525935	protein_coding	22,80	0
ENSG00000100632	ERH	14	69380123	69398627	protein_coding	22,80	0
ENSG00000171853	TRAPPC12	2	3379675	3485094	protein_coding	22,79	0
ENSG00000114480	GBE1	3	81489699	81762161	protein_coding	22,75	0
ENSG00000115307	AUP1	2	74526645	74529939	protein_coding	22,75	0
ENSG00000165312	OTUD1	10	23439458	23442390	protein_coding	22,71	0
ENSG00000138696	BMPR1B	4	94757968	95158448	protein_coding	22,71	0
ENSG00000176435	CLEC14A	14	38254103	38256369	protein_coding	22,70	0
ENSG00000001617	SEMA3F	3	50155045	50189075	protein_coding	22,70	0
ENSG00000177873	ZNF619	3	40477113	40490236	protein_coding	22,68	0
ENSG00000139163	ETNK1	12	22625075	22690665	protein_coding	22,68	0
ENSG00000204463	BAG6	6	31639028	31652705	protein_coding	22,67	0
ENSG00000175938	ORAI3	16	30949066	30956461	protein_coding	22,66	0
ENSG00000105576	TNPO2	19	12699194	12724011	protein_coding	22,63	0
ENSG00000074755	ZZEF1	17	4004445	4143020	protein_coding	22,62	0
ENSG00000173264	GPR137	11	64270062	64289500	protein_coding	22,61	0
ENSG00000116641	DOCK7	1	62454726	62688368	protein_coding	22,60	0
ENSG00000185515	BRCC3	X	155071420	155123074	protein_coding	22,59	0
ENSG00000275700	AATF	17	36948875	37056871	protein_coding	22,58	0
ENSG00000173786	CNP	17	41966741	41977731	protein_coding	22,57	0
ENSG00000010671	BTK	X	101349447	101390796	protein_coding	22,56	0
ENSG00000068024	HDAC4	2	239048168	239401654	protein_coding	22,56	0

ENSG00000204308	RNF5	6	32178354	32180793	protein_coding	22,52	0
ENSG00000264058	ENSG000002	17	40628797	40665141	protein_coding	22,50	0
ENSG00000173267	SNCG	10	86958618	86963260	protein_coding	22,50	0
ENSG00000155749	ALS2CR12	2	201288271	201357398	protein_coding	22,50	0
ENSG00000152595	MEPE	4	87821411	87846817	protein_coding	22,45	0
ENSG00000174243	DDX23	12	48829764	48852842	protein_coding	22,43	0
ENSG00000101945	SUV39H1	X	48695554	48709012	protein_coding	22,42	0
ENSG00000235863	B3GALT4	6	33277132	33284832	protein_coding	22,41	0
ENSG00000137877	SPTBN5	15	41848144	41894077	protein_coding	22,41	0
ENSG00000137409	MTCH1	6	36968141	36986298	protein_coding	22,41	0
ENSG00000186866	POFUT2	21	45263928	45287898	protein_coding	22,40	0
ENSG00000244274	DBNDD2	20	45406057	45410610	protein_coding	22,40	0
ENSG00000102935	ZNF423	16	49487524	49857919	protein_coding	22,40	0
ENSG00000100344	PNPLA3	22	43923739	43964488	protein_coding	22,39	0
ENSG00000175467	SART1	11	65961689	65979828	protein_coding	22,37	0
ENSG00000011243	AKAP8L	19	15380048	15419141	protein_coding	22,36	0
ENSG00000167548	KMT2D	12	49018975	49059774	protein_coding	22,34	0
ENSG00000154856	APCDD1	18	10454628	10489948	protein_coding	22,33	0
ENSG00000126461	SCAF1	19	49642125	49658642	protein_coding	22,33	0
ENSG00000127952	STYXL1	7	75996338	76048004	protein_coding	22,30	0
ENSG00000073008	PVR	19	44643798	44663583	protein_coding	22,30	0
ENSG00000136758	YME1L1	10	27110112	27155266	protein_coding	22,30	0
ENSG00000188133	TMEM215	9	32783499	32787399	protein_coding	22,30	0
ENSG00000204315	FKBPL	6	32128707	32130291	protein_coding	22,29	0
ENSG00000278540	ACACA	17	37084988	37406818	protein_coding	22,27	0
ENSG00000106459	NRF1	7	129611714	129757082	protein_coding	22,27	0
ENSG00000189091	SF3B3	16	70523788	70577670	protein_coding	22,27	0
ENSG00000143226	FCGR2A	1	161505430	161524013	protein_coding	22,25	0
ENSG00000112096	SOD2	6	159669057	159762529	protein_coding	22,22	0
ENSG00000255302	EID1	15	48877886	48880183	protein_coding	22,21	0
ENSG00000184209	SNRNP35	12	123457641	123473154	protein_coding	22,21	0
ENSG00000176723	ZNF843	16	31432593	31443160	protein_coding	22,20	0
ENSG00000135898	GPR55	2	230907318	230961066	protein_coding	22,20	0
ENSG00000197451	HNRNPAB	5	178204507	178211180	protein_coding	22,19	0
ENSG00000116981	NT5C1A	1	39659121	39672038	protein_coding	22,17	0
ENSG00000131779	PEX11B	1	145911350	145918837	protein_coding	22,16	0
ENSG00000137216	TMEM63B	6	44126914	44155519	protein_coding	22,16	0
ENSG00000165782	TMEM55B	14	20457719	20461612	protein_coding	22,14	0
ENSG00000125170	DOK4	16	57471922	57487327	protein_coding	22,14	0
ENSG00000100124	ANKRD54	22	37830855	37849327	protein_coding	22,13	0
ENSG00000188229	TUBB4B	9	137241213	137243707	protein_coding	22,12	0
ENSG00000145990	GFOD1	6	13357830	13487662	protein_coding	22,11	0
ENSG00000116903	EXOC8	1	231332753	231337852	protein_coding	22,10	0
ENSG00000108370	RGS9	17	65137431	65227703	protein_coding	22,10	0
ENSG00000196866	HIST1H2AD	6	26198851	26199243	protein_coding	22,10	0
ENSG00000158473	CD1D	1	158179947	158184896	protein_coding	22,10	0
ENSG00000136731	UGGT1	2	128091200	128195677	protein_coding	22,09	0
ENSG00000196408	NOXO1	16	1978917	1984192	protein_coding	22,09	0
ENSG00000080561	MID2	X	107825755	107927193	protein_coding	22,09	0
ENSG00000185567	AHNAK2	14	104937244	104978357	protein_coding	22,08	0
ENSG00000003989	SLC7A2	8	17497088	17570573	protein_coding	22,08	0
ENSG00000174231	PRPF8	17	1650629	1684882	protein_coding	22,06	0
ENSG00000153187	HNRNPU	1	244851166	244864542	protein_coding	22,05	0
ENSG00000170734	POLH	6	43576150	43615660	protein_coding	22,05	0
ENSG00000122707	RECK	9	36036433	36124451	protein_coding	22,01	0
ENSG00000100350	FOXRED2	22	36487190	36507101	protein_coding	22,01	0
ENSG00000099204	ABLIM1	10	114431113	114685003	protein_coding	22,01	0
ENSG00000178127	NDUFV2	18	9102630	9134345	protein_coding	22,00	0

ENSG00000241935	HOGA1	10	97584323	97612802	protein_coding	22,00	0
ENSG00000060138	YBX3	12	10699089	10723312	protein_coding	21,99	0
ENSG00000108061	SHOC2	10	110919547	111013667	protein_coding	21,99	0
ENSG00000066926	FECH	18	57548283	57586772	protein_coding	21,99	0
ENSG00000077264	PAK3	X	110944285	111227361	protein_coding	21,98	0
ENSG00000133619	KRBA1	7	149714781	149734575	protein_coding	21,98	0
ENSG00000111667	USP5	12	6852128	6866632	protein_coding	21,98	0
ENSG00000172375	C2CD2L	11	119102198	119118544	protein_coding	21,97	0
ENSG00000100714	MTHFD1	14	64388031	64463457	protein_coding	21,97	0
ENSG00000196407	THEM5	1	151847263	151853697	protein_coding	21,97	0
ENSG00000090861	AARS	16	70252295	70289543	protein_coding	21,95	0
ENSG00000110047	EHD1	11	64851642	64888296	protein_coding	21,95	0
ENSG00000148400	NOTCH1	9	136494444	136545862	protein_coding	21,95	0
ENSG00000078814	MYH7B	20	34975403	35002437	protein_coding	21,94	0
ENSG00000163638	ADAMTS9	3	64515654	64688000	protein_coding	21,93	0
ENSG00000111058	ACSS3	12	80936414	81261205	protein_coding	21,93	0
ENSG00000189060	H1F0	22	37805093	37807436	protein_coding	21,92	0
ENSG00000017427	IGF1	12	102395867	102480645	protein_coding	21,90	0
ENSG00000120727	PAIP2	5	139341587	139369720	protein_coding	21,88	0
ENSG00000115556	PLCD4	2	218607765	218637184	protein_coding	21,88	0
ENSG00000149182	ARFGAP2	11	47164299	47177125	protein_coding	21,87	0
ENSG00000179168	GGN	19	38384265	38388082	protein_coding	21,85	0
ENSG00000272674	PCDHB16	5	141181399	141186399	protein_coding	21,85	0
ENSG00000143811	PYCR2	1	225919877	225924340	protein_coding	21,84	0
ENSG00000197119	SLC25A29	14	100291111	100306547	protein_coding	21,82	0
ENSG00000171159	C9orf16	9	128160260	128163928	protein_coding	21,81	0
ENSG00000010256	UQCRC1	3	48599002	48610976	protein_coding	21,80	0
ENSG00000169592	INO80E	16	29995294	30005793	protein_coding	21,80	0
ENSG00000092529	CAPN3	15	42359500	42412318	protein_coding	21,80	0
ENSG00000185345	PARK2	6	161347420	162727771	protein_coding	21,80	0
ENSG00000122741	DCAF10	9	37800502	37867666	protein_coding	21,80	0
ENSG00000175115	PACS1	11	66070363	66244747	protein_coding	21,80	0
ENSG00000131759	RARA	17	40309192	40357643	protein_coding	21,78	0
ENSG00000069275	NUCKS1	1	205712819	205750276	protein_coding	21,78	0
ENSG00000160211	G6PD	X	154531391	154547572	protein_coding	21,75	0
ENSG00000153214	TMEM87B	2	112055223	112119318	protein_coding	21,73	0
ENSG00000172543	CTSW	11	65879809	65883741	protein_coding	21,70	0
ENSG00000143340	FAM163A	1	179743163	179816198	protein_coding	21,70	0
ENSG00000138760	SCARB2	4	76158737	76213893	protein_coding	21,69	0
ENSG00000213347	MXD3	5	177301461	177312757	protein_coding	21,65	0
ENSG00000165678	GHITM	10	84139440	84153555	protein_coding	21,64	0
ENSG00000100519	PSMC6	14	52707172	52728587	protein_coding	21,64	0
ENSG00000184307	ZDHHC23	3	113947901	113965401	protein_coding	21,63	0
ENSG00000108960	MMD	17	55392613	55421992	protein_coding	21,62	0
ENSG00000018189	RUFY3	4	70704204	70807315	protein_coding	21,62	0
ENSG00000142002	DPP9	19	4675224	4724673	protein_coding	21,61	0
ENSG00000178093	TSSK6	19	19512418	19515685	protein_coding	21,60	0
ENSG00000169689	STRA13	17	82018702	82024107	protein_coding	21,60	0
ENSG00000180884	ZNF792	19	34956354	34964049	protein_coding	21,59	0
ENSG00000254004	ZNF260	19	36510695	36528660	protein_coding	21,59	0
ENSG00000167676	PLIN4	19	4502180	4518465	protein_coding	21,58	0
ENSG00000185829	ARL17A	17	46516702	46579722	protein_coding	21,58	0
ENSG00000053918	KCNQ1	11	2444684	2849109	protein_coding	21,58	0
ENSG00000114395	CYB561D2	3	50350695	50358460	protein_coding	21,57	0
ENSG00000203727	SAMD5	6	147508927	147737547	protein_coding	21,56	0
ENSG00000138002	IFT172	2	27444371	27489789	protein_coding	21,56	0
ENSG00000135631	RAB11FIP5	2	73073382	73156721	protein_coding	21,56	0
ENSG00000198420	TCAF1	7	143851375	143902198	protein_coding	21,55	0

ENSG00000167476	JSRP1	19	2252252	2269759	protein_coding	21,54	0
ENSG00000170832	USP32	17	60179094	60422470	protein_coding	21,54	0
ENSG00000198001	IRAK4	12	43758944	43789543	protein_coding	21,53	0
ENSG00000164684	ZNF704	8	80628451	80874781	protein_coding	21,52	0
ENSG00000150779	TIMM8B	11	112084800	112086798	protein_coding	21,50	0
ENSG00000188986	NELFB	9	137255173	137273547	protein_coding	21,50	0
ENSG00000128383	APOBEC3A	22	38952741	38992778	protein_coding	21,50	0
ENSG00000020181	ADGRA2	8	37784191	37844896	protein_coding	21,47	0
ENSG00000163382	APOA1BP	1	156591762	156594299	protein_coding	21,47	0
ENSG00000198551	ZNF627	19	11559374	11619135	protein_coding	21,45	0
ENSG00000167207	NOD2	16	50693603	50733077	protein_coding	21,44	0
ENSG00000068878	PSME4	2	53864067	53970840	protein_coding	21,44	0
ENSG00000107036	RIC1	9	5629025	5776557	protein_coding	21,43	0
ENSG00000142082	SIRT3	11	215458	236931	protein_coding	21,42	0
ENSG00000142733	MAP3K6	1	27355184	27366892	protein_coding	21,41	0
ENSG00000168453	HR	8	22114415	22133384	protein_coding	21,40	0
ENSG00000174500	GCSAM	3	112120841	112133305	protein_coding	21,40	0
ENSG00000223658	C1GALT1C1L	2	43675151	43676322	protein_coding	21,40	0
ENSG00000101246	ARFRP1	20	63698642	63708025	protein_coding	21,39	0
ENSG00000162086	ZNF75A	16	3305406	3318852	protein_coding	21,39	0
ENSG00000197121	PGAP1	2	196833004	196927796	protein_coding	21,38	0
ENSG00000128791	TWSG1	18	9334767	9402420	protein_coding	21,37	0
ENSG00000127947	PTPN12	7	77537275	77640071	protein_coding	21,37	0
ENSG00000057704	TMCC3	12	94567124	94650562	protein_coding	21,37	0
ENSG00000111816	FRK	6	115931149	116060758	protein_coding	21,36	0
ENSG00000143771	CNIH4	1	224356850	224379459	protein_coding	21,35	0
ENSG00000169919	GUSB	7	65960684	65982314	protein_coding	21,33	0
ENSG00000187051	RPS19BP1	22	39529093	39532855	protein_coding	21,33	0
ENSG00000104969	SGTA	19	2754714	2783371	protein_coding	21,32	0
ENSG00000155367	PPM1J	1	112709994	112715477	protein_coding	21,30	0
ENSG00000204713	TRIM27	6	28903002	28923989	protein_coding	21,28	0
ENSG00000113240	CLK4	5	178602664	178630615	protein_coding	21,28	0
ENSG00000142409	ZNF787	19	56087366	56121280	protein_coding	21,28	0
ENSG00000136450	SRSF1	17	58003360	58007346	protein_coding	21,25	0
ENSG00000163481	RNF25	2	218663864	218672411	protein_coding	21,24	0
ENSG00000142303	ADAMTS10	19	8580242	8610735	protein_coding	21,24	0
ENSG00000168264	IRF2BP2	1	234604269	234609525	protein_coding	21,19	0
ENSG00000074054	CLASP1	2	121337776	121649587	protein_coding	21,16	0
ENSG00000164022	AIMP1	4	106315544	106349226	protein_coding	21,12	0
ENSG00000108448	TRIM16L	17	18697998	18736118	protein_coding	21,10	0
ENSG00000253910	PCDHGB2	5	141360042	141512979	protein_coding	21,10	0
ENSG00000147166	ITGB1BP2	X	71301734	71305371	protein_coding	21,10	0
ENSG00000167191	GPRC5B	16	19856691	19886167	protein_coding	21,07	0
ENSG00000153250	RBMS1	2	160272151	160493794	protein_coding	21,06	0
ENSG00000090372	STRN4	19	46719507	46746994	protein_coding	21,06	0
ENSG00000005187	ACSM3	16	20610243	20797581	protein_coding	21,05	0
ENSG00000141337	ARSG	17	68259182	68422731	protein_coding	21,05	0
ENSG00000173933	RBM4	11	66638617	66666682	protein_coding	21,04	0
ENSG00000065526	SPEN	1	15847864	15940460	protein_coding	21,03	0
ENSG00000132718	SYT11	1	155859509	155885199	protein_coding	21,02	0
ENSG00000121858	TNFSF10	3	172505508	172523507	protein_coding	21,00	0
ENSG00000235169	SMIM1	1	3772788	3775982	protein_coding	20,96	0
ENSG00000110375	UPK2	11	118925164	118958559	protein_coding	20,95	0
ENSG00000188554	NBR1	17	43170481	43211689	protein_coding	20,94	0
ENSG00000119408	NEK6	9	124257606	124353307	protein_coding	20,93	0
ENSG00000204618	RNF39	6	30070266	30075887	protein_coding	20,91	0
ENSG00000177455	CD19	16	28931939	28939346	protein_coding	20,90	0
ENSG00000170681	MURC	9	100578079	100587906	protein_coding	20,90	0

ENSG00000162552	WNT4	1	22117305	22143969	protein_coding	20,90	0
ENSG00000102053	ZC3H12B	X	65366638	65507887	protein_coding	20,90	0
ENSG00000180900	SCRIB	8	143790920	143815379	protein_coding	20,87	0
ENSG00000183475	ASB7	15	100602534	100651705	protein_coding	20,85	0
ENSG00000163577	EIF5A2	3	170888415	170908693	protein_coding	20,85	0
ENSG00000180694	TMEM64	8	90621995	90791632	protein_coding	20,85	0
ENSG00000087884	AAMDC	11	77821109	77918432	protein_coding	20,81	0
ENSG00000101019	UQCC1	20	35302566	35412141	protein_coding	20,81	0
ENSG00000123395	ATG101	12	52069246	52077494	protein_coding	20,80	0
ENSG00000180596	HIST1H2BC	6	26114873	26123926	protein_coding	20,80	0
ENSG00000203797	DDO	6	110391771	110415562	protein_coding	20,80	0
ENSG00000172366	FAM195A	16	636817	648474	protein_coding	20,77	0
ENSG00000126561	STAT5A	17	42287547	42311943	protein_coding	20,77	0
ENSG00000132792	CTNNBL1	20	37693955	37872129	protein_coding	20,74	0
ENSG00000167701	GPT	8	144502973	144507174	protein_coding	20,73	0
ENSG00000117676	RPS6KA1	1	26529761	26575030	protein_coding	20,72	0
ENSG00000106617	PRKAG2	7	151556111	151877125	protein_coding	20,70	0
ENSG00000154059	IMPACT	18	24426616	24453535	protein_coding	20,69	0
ENSG00000114529	C3orf52	3	112086335	112131004	protein_coding	20,69	0
ENSG00000037749	MFAP3	5	154038906	154220478	protein_coding	20,68	0
ENSG00000146223	RPL7L1	6	42879618	42889925	protein_coding	20,67	0
ENSG00000175575	PAAF1	11	73876699	73931124	protein_coding	20,66	0
ENSG00000197081	IGF2R	6	159969099	160113507	protein_coding	20,66	0
ENSG00000149716	ORAOV1	11	69653076	69675416	protein_coding	20,64	0
ENSG00000119471	HSDL2	9	112379937	112472410	protein_coding	20,64	0
ENSG00000115274	INO80B	2	74455023	74457960	protein_coding	20,64	0
ENSG00000132196	HSD17B7	1	162790702	162812817	protein_coding	20,61	0
ENSG00000112078	KCTD20	6	36442767	36491143	protein_coding	20,61	0
ENSG00000151490	PTPRO	12	15322397	15597399	protein_coding	20,60	0
ENSG00000183784	C9orf66	9	212824	215741	protein_coding	20,60	0
ENSG00000198408	MGEA5	10	101784443	101818465	protein_coding	20,59	0
ENSG00000170921	TANC2	17	63009556	63427699	protein_coding	20,57	0
ENSG00000131100	ATP6V1E1	22	17592136	17628818	protein_coding	20,55	0
ENSG00000176597	B3GNT5	3	183253244	183298504	protein_coding	20,54	0
ENSG00000122678	POLM	7	44072247	44082540	protein_coding	20,53	0
ENSG00000132680	KIAA0907	1	155913043	155934400	protein_coding	20,53	0
ENSG00000149328	GLB1L2	11	134331874	134378341	protein_coding	20,51	0
ENSG00000177888	ZBTB41	1	197153680	197200542	protein_coding	20,50	0
ENSG00000150768	DLAT	11	112024814	112064390	protein_coding	20,44	0
ENSG00000196110	ZNF699	19	9294275	9309838	protein_coding	20,40	0
ENSG00000175857	GAPT	5	58491435	58497090	protein_coding	20,40	0
ENSG00000114378	HYAL1	3	50299889	50312381	protein_coding	20,40	0
ENSG00000163933	RFT1	3	53088483	53130462	protein_coding	20,38	0
ENSG00000124116	WFDC3	20	45747944	45791932	protein_coding	20,36	0
ENSG00000172992	DCAKD	17	45023340	45061109	protein_coding	20,36	0
ENSG00000100938	GMPR2	14	24232422	24239242	protein_coding	20,35	0
ENSG00000104872	PIH1D1	19	49446298	49453497	protein_coding	20,34	0
ENSG00000065150	IPO5	13	97953658	98024297	protein_coding	20,34	0
ENSG00000134986	NREP	5	111662621	111997464	protein_coding	20,33	0
ENSG00000167863	ATP5H	17	75038863	75046985	protein_coding	20,33	0
ENSG00000136603	SKIL	3	170357678	170396835	protein_coding	20,32	0
ENSG00000183048	SLC25A10	17	81712236	81721016	protein_coding	20,30	0
ENSG00000181038	METTL23	17	76726830	76733936	protein_coding	20,29	0
ENSG00000181827	RFX7	15	56087280	56243266	protein_coding	20,29	0
ENSG00000155229	MMS19	10	97458324	97498794	protein_coding	20,28	0
ENSG00000185090	MANEAL	1	37793802	37801137	protein_coding	20,28	0
ENSG00000078114	NEBL	10	20779973	21174187	protein_coding	20,28	0
ENSG00000166575	TMEM135	11	87037844	87323758	protein_coding	20,25	0

ENSG00000178694	NSUN3	3	94062916	94128545	protein_coding	20,24	0
ENSG00000187189	TSPYL4	6	116249961	116254140	protein_coding	20,23	0
ENSG00000129245	FXR2	17	7591230	7614871	protein_coding	20,22	0
ENSG00000204397	CARD16	11	105041326	105101431	protein_coding	20,20	0
ENSG00000121797	CCRL2	3	46407163	46412997	protein_coding	20,20	0
ENSG00000168591	TMUB2	17	44186970	44191731	protein_coding	20,19	0
ENSG00000171793	CTPS1	1	40979335	41012565	protein_coding	20,19	0
ENSG00000166801	FAM111A	11	59142748	59155039	protein_coding	20,18	0
ENSG00000179295	PTPN11	12	112418351	112509913	protein_coding	20,17	0
ENSG00000103507	BCKDK	16	31106107	31112791	protein_coding	20,16	0
ENSG00000184110	EIF3C	16	28688558	28735730	protein_coding	20,15	0
ENSG00000131507	NDFIP1	5	142108505	142154443	protein_coding	20,15	0
ENSG00000138074	SLC5A6	2	27199587	27212958	protein_coding	20,13	0
ENSG00000151502	VPS26B	11	134224645	134247792	protein_coding	20,12	0
ENSG00000260230	FRRS1L	9	109130293	109167291	protein_coding	20,11	0
ENSG00000205339	IPO7	11	9384622	9448126	protein_coding	20,10	0
ENSG00000132000	PODNL1	19	13931187	13953392	protein_coding	20,10	0
ENSG00000165071	TMEM71	8	132685007	132760712	protein_coding	20,10	0
ENSG00000173705	SUSD5	3	33150042	33219215	protein_coding	20,09	0
ENSG00000146070	PLA2G7	6	46704201	46735693	protein_coding	20,07	0
ENSG00000112773	FAM46A	6	81491439	81752774	protein_coding	20,07	0
ENSG00000120725	SIL1	5	138946720	139293557	protein_coding	20,06	0
ENSG00000166685	COG1	17	73192632	73208507	protein_coding	20,05	0
ENSG00000131791	PRKAB2	1	147155106	147172550	protein_coding	20,05	0
ENSG00000143753	DEGS1	1	224175756	224193441	protein_coding	20,04	0
ENSG00000129480	DTD2	14	31446036	31457510	protein_coding	20,04	0
ENSG00000173273	TNKS	8	9555914	9782346	protein_coding	20,03	0
ENSG00000179387	ELMOD2	4	140524158	140553770	protein_coding	20,01	0
ENSG00000106635	BCL7B	7	73536356	73558002	protein_coding	20,00	0
ENSG00000139410	SDSL	12	113422237	113438276	protein_coding	20,00	0
ENSG00000178295	GEN1	2	17753858	17788941	protein_coding	20,00	0
ENSG00000155660	PDIA4	7	149003062	149028641	protein_coding	19,98	0
ENSG00000198642	KLHL9	9	21329671	21335380	protein_coding	19,98	0
ENSG00000148450	MSRB2	10	23095506	23122013	protein_coding	19,95	0
ENSG00000177463	NR2C2	3	14947584	15053600	protein_coding	19,94	0
ENSG00000150630	VEGFC	4	176683538	176792727	protein_coding	19,94	0
ENSG00000063761	ADCK1	14	77800083	77935012	protein_coding	19,92	0
ENSG00000120158	RCL1	9	4792869	4885917	protein_coding	19,91	0
ENSG00000230510	PPP5D1	19	46480796	46601200	protein_coding	19,90	0
ENSG00000118242	MREG	2	215942805	216034096	protein_coding	19,90	0
ENSG00000155957	TMBIM4	12	66135846	66170072	protein_coding	19,89	0
ENSG00000213064	SFT2D2	1	168225938	168253025	protein_coding	19,89	0
ENSG00000158092	NCK1	3	136862208	136949823	protein_coding	19,88	0
ENSG00000239672	NME1	17	51153536	51162428	protein_coding	19,88	0
ENSG00000109586	GALNT7	4	173168753	173323967	protein_coding	19,85	0
ENSG00000139668	WDFY2	13	51584455	51767707	protein_coding	19,84	0
ENSG00000165072	MAMDC2	9	70043581	70226970	protein_coding	19,83	0
ENSG00000178425	NT5DC1	6	116100849	116249497	protein_coding	19,82	0
ENSG00000176531	PHLDB3	19	43474954	43504935	protein_coding	19,80	0
ENSG00000035115	SH3YL1	2	217730	266398	protein_coding	19,80	0
ENSG00000142751	GPN2	1	26876133	26890297	protein_coding	19,80	0
ENSG00000119396	RAB14	9	121178137	121223014	protein_coding	19,78	0
ENSG00000103018	CYB5B	16	69424525	69466266	protein_coding	19,78	0
ENSG00000147123	NDUFB11	X	47142216	47145504	protein_coding	19,78	0
ENSG00000170265	ZNF282	7	149195485	149226248	protein_coding	19,76	0
ENSG00000165502	RPL36AL	14	49618519	49620685	protein_coding	19,75	0
ENSG00000104221	BRF2	8	37843268	37849904	protein_coding	19,75	0
ENSG00000142556	ZNF614	19	52012765	52030240	protein_coding	19,74	0

ENSG00000198739	LRRTM3	10	66926006	67099830	protein_coding	19,73	0
ENSG00000040633	PHF23	17	7235028	7239722	protein_coding	19,72	0
ENSG00000175745	NR2F1	5	93583337	93594615	protein_coding	19,71	0
ENSG00000179151	EDC3	15	74630558	74696292	protein_coding	19,71	0
ENSG00000174292	TNK1	17	7380534	7389774	protein_coding	19,70	0
ENSG00000248643	RBM14-RBM	11	66616626	66646469	protein_coding	19,70	0
ENSG00000188282	RUFY4	2	218034960	218090581	protein_coding	19,70	0
ENSG00000116819	TFAP2E	1	35573370	35595328	protein_coding	19,70	0
ENSG00000166377	ATP9B	18	79069285	79378283	protein_coding	19,68	0
ENSG00000124571	XPO5	6	43522330	43576075	protein_coding	19,65	0
ENSG00000113583	C5orf15	5	133955502	133968787	protein_coding	19,65	0
ENSG00000101004	NINL	20	25452705	25585517	protein_coding	19,64	0
ENSG00000188603	CLN3	16	28477279	28495575	protein_coding	19,64	0
ENSG00000158615	PPP1R15B	1	204403387	204411791	protein_coding	19,62	0
ENSG00000048828	FAM120A	9	93451722	93566107	protein_coding	19,61	0
ENSG00000169302	STK32A	5	147234963	147387852	protein_coding	19,60	0
ENSG00000179921	GPBAR1	2	218259496	218263859	protein_coding	19,60	0
ENSG00000014824	SLC30A9	4	41990472	42090457	protein_coding	19,60	0
ENSG00000170270	C14orf142	14	93202894	93207094	protein_coding	19,59	0
ENSG00000100395	L3MBTL2	22	41205205	41231271	protein_coding	19,58	0
ENSG00000090006	LTBP4	19	40592883	40629818	protein_coding	19,57	0
ENSG00000183111	ARHGEF37	5	149551947	149634968	protein_coding	19,56	0
ENSG00000138613	APH1B	15	63276018	63309126	protein_coding	19,53	0
ENSG00000198356	ASNA1	19	12737139	12748323	protein_coding	19,53	0
ENSG00000255508	ENSG000002	11	62559603	62591531	protein_coding	19,52	0
ENSG00000198677	TTC37	5	95463895	95555007	protein_coding	19,51	0
ENSG00000156873	PHKG2	16	30748270	30761176	protein_coding	19,51	0
ENSG00000185010	F8	X	154835788	155026940	protein_coding	19,51	0
ENSG00000159251	ACTC1	15	34788096	34796139	protein_coding	19,50	0
ENSG00000102445	KIAA0226L	13	46342000	46438190	protein_coding	19,50	0
ENSG00000082497	SERTAD4	1	210232799	210246631	protein_coding	19,50	0
ENSG00000235098	ANKRD65	1	1418420	1421769	protein_coding	19,50	0
ENSG00000126062	TMEM115	3	50354749	50359610	protein_coding	19,49	0
ENSG00000168101	NUDT16L1	16	4693694	4695859	protein_coding	19,47	0
ENSG00000169371	SNUPN	15	75598083	75626469	protein_coding	19,47	0
ENSG00000132670	PTPRA	20	2864184	3039076	protein_coding	19,46	0
ENSG00000198453	ZNF568	19	36916329	36998700	protein_coding	19,45	0
ENSG00000103274	NUBP1	16	10743786	10769351	protein_coding	19,45	0
ENSG00000163558	PRKCI	3	170222365	170305981	protein_coding	19,42	0
ENSG00000101846	STS	X	7219456	7354810	protein_coding	19,38	0
ENSG00000144642	RBMS3	3	29280982	30010391	protein_coding	19,38	0
ENSG00000249459	ZNF286B	17	18658429	18682262	protein_coding	19,37	0
ENSG00000176244	ACBD7	10	15077523	15088776	protein_coding	19,36	0
ENSG00000128626	MRPS12	19	38930548	38933162	protein_coding	19,36	0
ENSG00000103549	RNF40	16	30761745	30776307	protein_coding	19,36	0
ENSG00000113971	NPHP3	3	132680609	132722442	protein_coding	19,34	0
ENSG00000187147	RNF220	1	44405194	44651724	protein_coding	19,34	0
ENSG00000185838	GNB1L	22	19783224	19854939	protein_coding	19,33	0
ENSG00000183044	ABAT	16	8674565	8784575	protein_coding	19,33	0
ENSG00000114948	ADAM23	2	206443539	206621130	protein_coding	19,32	0
ENSG00000204231	RXRB	6	33193588	33200688	protein_coding	19,30	0
ENSG00000123700	KCNJ2	17	70168673	70180048	protein_coding	19,30	0
ENSG00000074047	GLI2	2	120735623	120992653	protein_coding	19,30	0
ENSG00000180875	GREM2	1	240489573	240612149	protein_coding	19,30	0
ENSG00000215788	TNFRSF25	1	6461151	6466195	protein_coding	19,30	0
ENSG00000184381	PLA2G6	22	38111495	38205690	protein_coding	19,29	0
ENSG00000217128	FNIP1	5	131641714	131797063	protein_coding	19,27	0
ENSG00000107185	RGP1	9	35749287	35758575	protein_coding	19,27	0

ENSG00000127884	ECHS1	10	133362480	133373689	protein_coding	19,25	0
ENSG00000111203	ITFG2	12	2812622	2859791	protein_coding	19,25	0
ENSG00000198720	ANKRD13B	17	29589769	29614761	protein_coding	19,21	0
ENSG00000102967	DHODH	16	72008588	72027664	protein_coding	19,20	0
ENSG00000181392	SYNE4	19	36003307	36008793	protein_coding	19,20	0
ENSG00000197016	ZNF470	19	56567511	56588911	protein_coding	19,19	0
ENSG00000111752	PHC1	12	8913896	8941467	protein_coding	19,19	0
ENSG00000103415	HMOX2	16	4474690	4510347	protein_coding	19,17	0
ENSG00000160570	DEDD2	19	42198598	42220140	protein_coding	19,17	0
ENSG00000213865	C8orf44	8	66667615	66685564	protein_coding	19,17	0
ENSG00000273136	NBPF26	1	120805418	120841481	protein_coding	19,17	0
ENSG00000047315	POLR2B	4	56977722	57031168	protein_coding	19,16	0
ENSG00000109667	SLC2A9	4	9771153	10054936	protein_coding	19,15	0
ENSG00000166278	C2	6	31897785	31945672	protein_coding	19,13	0
ENSG00000263001	GTF2I	7	74657667	74760692	protein_coding	19,13	0
ENSG00000127324	TSPAN8	12	71125085	71441898	protein_coding	19,10	0
ENSG00000166024	R3HCC1L	10	98134624	98244897	protein_coding	19,08	0
ENSG00000107872	FBXL15	10	102419189	102423136	protein_coding	19,07	0
ENSG00000175215	CTDSP2	12	57819927	57846739	protein_coding	19,07	0
ENSG00000161914	ZNF653	19	11483427	11505923	protein_coding	19,06	0
ENSG00000155792	DEPTOR	8	119873717	120050913	protein_coding	19,05	0
ENSG00000010295	IFFO1	12	6538375	6556083	protein_coding	19,04	0
ENSG00000068745	IP6K2	3	48688003	48740353	protein_coding	19,00	0
ENSG00000273820	USP27X	X	49879948	49882565	protein_coding	18,98	0
ENSG00000084623	EIF3I	1	32221928	32231604	protein_coding	18,98	0
ENSG00000125648	SLC25A23	19	6436079	6465203	protein_coding	18,97	0
ENSG00000148848	ADAM12	10	126012381	126388455	protein_coding	18,97	0
ENSG00000079819	EPB41L2	6	130839347	131063322	protein_coding	18,96	0
ENSG00000164620	RELL2	5	141636950	141641077	protein_coding	18,96	0
ENSG00000079482	OPHN1	X	68042344	68433913	protein_coding	18,94	0
ENSG00000135517	MIP	12	56449502	56469166	protein_coding	18,94	0
ENSG00000170348	TMED10	14	75131470	75176631	protein_coding	18,94	0
ENSG00000186792	HYAL3	3	50292831	50299468	protein_coding	18,92	0
ENSG00000115216	NRBP1	2	27427790	27442259	protein_coding	18,92	0
ENSG00000167978	SRRM2	16	2752329	2772538	protein_coding	18,91	0
ENSG00000136098	NEK3	13	52132639	52159861	protein_coding	18,91	0
ENSG00000144118	RALB	2	120240064	120294713	protein_coding	18,91	0
ENSG00000104957	CCDC130	19	13731760	13763296	protein_coding	18,90	0
ENSG00000204138	PHACTR4	1	28369582	28500369	protein_coding	18,89	0
ENSG00000149557	FEZ1	11	125445745	125496317	protein_coding	18,88	0
ENSG00000165475	CRYL1	13	20403667	20525857	protein_coding	18,87	0
ENSG00000188878	FBF1	17	75909574	75941140	protein_coding	18,85	0
ENSG00000078804	TP53INP2	20	34704290	34713439	protein_coding	18,84	0
ENSG00000132376	INPP5K	17	1494571	1516888	protein_coding	18,84	0
ENSG00000197702	PARVA	11	12377185	12530801	protein_coding	18,84	0
ENSG00000086159	AQP6	12	49967194	49977139	protein_coding	18,80	0
ENSG00000253537	PCDHGA7	5	141382739	141512979	protein_coding	18,80	0
ENSG00000188981	MSANTD1	4	3244369	3271738	protein_coding	18,80	0
ENSG00000096080	MRPS18A	6	43671303	43687791	protein_coding	18,80	0
ENSG00000100650	SRSF5	14	69726900	69772005	protein_coding	18,79	0
ENSG00000170909	OSCAR	19	54094668	54102692	protein_coding	18,77	0
ENSG00000082701	GSK3B	3	119821323	120094417	protein_coding	18,77	0
ENSG00000112339	HBS1L	6	134960378	135103056	protein_coding	18,75	0
ENSG00000130741	EIF2S3	X	24054716	24077971	protein_coding	18,72	0
ENSG00000132334	PTPRE	10	127907061	128085855	protein_coding	18,72	0
ENSG00000113161	HMGCR	5	75336329	75362104	protein_coding	18,72	0
ENSG00000155744	FAM126B	2	200973718	201071671	protein_coding	18,71	0
ENSG00000129055	ANAPC13	3	134477706	134486716	protein_coding	18,71	0

ENSG00000276070	CCL4L2	17	36210924	36212878	protein_coding	18,70	0
ENSG00000279765	ENSG000002	15	92883413	92949230	protein_coding	18,70	0
ENSG00000173421	CCDC36	3	49198428	49258104	protein_coding	18,70	0
ENSG00000132254	ARFIP2	11	6474683	6481479	protein_coding	18,70	0
ENSG00000141098	GFOD2	16	67674531	67719421	protein_coding	18,70	0
ENSG00000122085	MTERF4	2	241072169	241102332	protein_coding	18,69	0
ENSG00000080603	SRCAP	16	30698209	30741409	protein_coding	18,67	0
ENSG00000129219	PLD2	17	4807096	4823434	protein_coding	18,65	0
ENSG00000132330	SCLY	2	238060889	238099413	protein_coding	18,65	0
ENSG00000197467	COL13A1	10	69801931	69964275	protein_coding	18,64	0
ENSG00000112208	BAG2	6	57172326	57189833	protein_coding	18,63	0
ENSG00000126970	ZC4H2	X	64915802	65034713	protein_coding	18,62	0
ENSG00000130755	GMFG	19	39328353	39342372	protein_coding	18,60	0
ENSG00000026751	SLAMF7	1	160739057	160754821	protein_coding	18,60	0
ENSG00000188186	LAMTOR4	7	100148907	100155944	protein_coding	18,58	0
ENSG00000117395	EBNA1BP2	1	43164175	43270936	protein_coding	18,56	0
ENSG00000108953	YWHAE	17	1344272	1400378	protein_coding	18,56	0
ENSG00000254995	STX16-NPEP	20	58651434	58715410	protein_coding	18,55	0
ENSG00000119650	IFT43	14	75902136	76084585	protein_coding	18,55	0
ENSG00000129946	SHC2	19	416583	460996	protein_coding	18,54	0
ENSG00000198870	STKLD1	9	133376367	133406096	protein_coding	18,54	0
ENSG00000160062	ZBTB8A	1	32539427	32605939	protein_coding	18,53	0
ENSG00000168016	TRANK1	3	36826820	36945057	protein_coding	18,51	0
ENSG00000077312	SNRPA	19	40750637	40765389	protein_coding	18,50	0
ENSG00000204482	LST1	6	31586124	31588909	protein_coding	18,50	0
ENSG00000240038	AMY2B	1	103553815	103579534	protein_coding	18,50	0
ENSG00000234127	TRIM26	6	30184455	30213427	protein_coding	18,48	0
ENSG00000147475	ERLIN2	8	37736599	37759101	protein_coding	18,48	0
ENSG00000116990	MYCL	1	39895426	39902256	protein_coding	18,47	0
ENSG00000170088	TMEM192	4	165070608	165208549	protein_coding	18,46	0
ENSG00000140474	ULK3	15	74836116	74843346	protein_coding	18,45	0
ENSG00000124783	SSR1	6	7268306	7347446	protein_coding	18,44	0
ENSG00000040199	PHLPP2	16	71637835	71724701	protein_coding	18,43	0
ENSG00000125656	CLPP	19	6361452	6368908	protein_coding	18,43	0
ENSG00000087842	PIR	X	15384799	15493564	protein_coding	18,42	0
ENSG00000133246	PRAM1	19	8490056	8503112	protein_coding	18,42	0
ENSG00000132952	USPL1	13	30617693	30660770	protein_coding	18,42	0
ENSG00000133067	LGR6	1	202193901	202319781	protein_coding	18,41	0
ENSG00000111229	ARPC3	12	110434825	110450422	protein_coding	18,41	0
ENSG00000074071	MRPS34	16	1771890	1773155	protein_coding	18,40	0
ENSG00000102001	CACNA1F	X	49205063	49233371	protein_coding	18,40	0
ENSG00000082641	NFE2L1	17	48048329	48061487	protein_coding	18,39	0
ENSG00000160208	RRP1B	21	43659548	43696079	protein_coding	18,39	0
ENSG00000087903	RFX2	19	5993164	6199572	protein_coding	18,38	0
ENSG00000166326	TRIM44	11	35662805	35818007	protein_coding	18,33	0
ENSG00000105373	GLTSCR2	19	47745522	47757058	protein_coding	18,32	0
ENSG00000133315	MACROD1	11	63998558	64166106	protein_coding	18,32	0
ENSG00000178096	BOLA1	1	149887890	149900798	protein_coding	18,29	0
ENSG00000177646	ACAD9	3	128879596	128916067	protein_coding	18,29	0
ENSG00000132467	UTP3	4	70688479	70690551	protein_coding	18,27	0
ENSG00000141385	AFG3L2	18	12328944	12377314	protein_coding	18,26	0
ENSG00000185019	UBOX5	20	3107573	3160196	protein_coding	18,25	0
ENSG00000087053	MTMR2	11	95832882	95925315	protein_coding	18,25	0
ENSG00000159352	PSMD4	1	151254703	151267479	protein_coding	18,24	0
ENSG00000128641	MYO1B	2	191245185	191425389	protein_coding	18,21	0
ENSG00000180720	CHRM4	11	46385098	46386608	protein_coding	18,20	0
ENSG00000186603	HPDL	1	45326905	45328533	protein_coding	18,20	0
ENSG00000163053	SLC16A14	2	230034974	230068999	protein_coding	18,19	0

ENSG00000156030	ELMSAN1	14	73715122	73790285	protein_coding	18,15	0
ENSG00000119599	DCAF4	14	72926332	72959703	protein_coding	18,15	0
ENSG00000168938	PPIC	5	123023250	123036741	protein_coding	18,14	0
ENSG00000130714	POMT1	9	131502902	131523806	protein_coding	18,13	0
ENSG00000067836	ROGDI	16	4796968	4802950	protein_coding	18,11	0
ENSG00000110367	DDX6	11	118747766	118791149	protein_coding	18,03	0
ENSG00000214827	MTCP1	X	155061622	155147937	protein_coding	18,02	0
ENSG00000058673	ZC3H11A	1	203795654	203854124	protein_coding	18,00	0
ENSG00000108932	SLC16A6	17	68267026	68291267	protein_coding	18,00	0
ENSG00000161653	NAGS	17	44004546	44009063	protein_coding	18,00	0
ENSG00000167157	PRRX2	9	129665641	129722674	protein_coding	18,00	0
ENSG00000182600	C2orf82	2	232857270	232878708	protein_coding	18,00	0
ENSG00000143575	HAX1	1	154272511	154275875	protein_coding	17,99	0
ENSG00000104731	KLHDC4	16	87696485	87765992	protein_coding	17,98	0
ENSG00000159256	MORC3	21	36320189	36386148	protein_coding	17,98	0
ENSG00000175048	ZDHHC14	6	157381133	157678146	protein_coding	17,98	0
ENSG00000140650	PMM2	16	8788823	8849331	protein_coding	17,98	0
ENSG00000182149	IST1	16	71845996	71931199	protein_coding	17,97	0
ENSG00000140406	MESDC1	15	81000944	81005788	protein_coding	17,97	0
ENSG00000110844	PRPF40B	12	49568218	49644666	protein_coding	17,96	0
ENSG00000046604	DSG2	18	31498043	31549008	protein_coding	17,95	0
ENSG00000131037	EPS8L1	19	55072020	55087923	protein_coding	17,95	0
ENSG00000135597	REPS1	6	138903493	138988261	protein_coding	17,95	0
ENSG00000151746	BICD1	12	32106835	32383633	protein_coding	17,94	0
ENSG00000182220	ATP6AP2	X	40580908	40606637	protein_coding	17,94	0
ENSG00000125485	DDX31	9	132592997	132670401	protein_coding	17,93	0
ENSG00000183718	TRIM52	5	181254417	181261139	protein_coding	17,92	0
ENSG00000055070	SZRD1	1	16352575	16398145	protein_coding	17,92	0
ENSG00000135905	DOCK10	2	224765090	225042445	protein_coding	17,91	0
ENSG00000172508	CARNS1	11	67414968	67425607	protein_coding	17,91	0
ENSG00000243943	ZNF512	2	27582969	27623215	protein_coding	17,91	0
ENSG00000011198	ABHD5	3	43690113	43734371	protein_coding	17,91	0
ENSG00000266074	BAHCC1	17	81395475	81466332	protein_coding	17,91	0
ENSG00000163328	GPR155	2	174431571	174487094	protein_coding	17,90	0
ENSG00000138772	ANXA3	4	78551519	78610451	protein_coding	17,90	0
ENSG00000213672	NCKIPSD	3	48673844	48686364	protein_coding	17,89	0
ENSG00000165861	ZFYVE1	14	72969451	73027212	protein_coding	17,87	0
ENSG00000140577	CRTC3	15	90529925	90645345	protein_coding	17,86	0
ENSG00000178028	DMAP1	1	44213455	44220681	protein_coding	17,81	0
ENSG00000177169	ULK1	12	131894651	131923167	protein_coding	17,81	0
ENSG00000068654	POLR1A	2	86020216	86106155	protein_coding	17,80	0
ENSG00000267216	ENSG000002	19	58278966	58315197	protein_coding	17,80	0
ENSG00000171757	LRRC34	3	169793428	169812986	protein_coding	17,80	0
ENSG00000243696	ENSG000002	3	52813282	52835729	protein_coding	17,80	0
ENSG00000022277	RTFDC1	20	56468585	56519449	protein_coding	17,80	0
ENSG00000087087	SRRT	7	100875111	100888664	protein_coding	17,79	0
ENSG00000104472	CHRAC1	8	140511298	140517137	protein_coding	17,78	0
ENSG00000178567	EPM2AIP1	3	36985043	36993168	protein_coding	17,77	0
ENSG00000198690	FAN1	15	30903852	30943108	protein_coding	17,76	0
ENSG00000110492	MDK	11	46380756	46383837	protein_coding	17,75	0
ENSG00000001084	GCLC	6	53497341	53616970	protein_coding	17,75	0
ENSG00000107937	GTPBP4	10	988019	1019936	protein_coding	17,74	0
ENSG00000150403	TMCO3	13	113490995	113554590	protein_coding	17,73	0
ENSG00000103023	PRSS54	16	58279997	58295047	protein_coding	17,73	0
ENSG00000143315	PIGM	1	160027673	160031991	protein_coding	17,72	0
ENSG00000005844	ITGAL	16	30472658	30523185	protein_coding	17,70	0
ENSG00000179071	CCDC89	11	85684866	85686277	protein_coding	17,70	0
ENSG00000214562	NUTM2D	10	87357668	87370695	protein_coding	17,70	0

ENSG00000122733	PHF24	9	34957608	34982544	protein_coding	17,70	0
ENSG00000178038	ALS2CL	3	46668997	46693704	protein_coding	17,70	0
ENSG00000107282	APBA1	9	69427530	69672306	protein_coding	17,70	0
ENSG00000119844	AFTPH	2	64524305	64593005	protein_coding	17,69	0
ENSG00000155962	CLIC2	X	155276211	155334657	protein_coding	17,69	0
ENSG00000133794	ARNTL	11	13276652	13387266	protein_coding	17,69	0
ENSG00000150764	DIXDC1	11	111927144	112022584	protein_coding	17,69	0
ENSG00000114279	FGF12	3	192139395	192767764	protein_coding	17,68	0
ENSG00000198556	ZNF789	7	99472841	99503650	protein_coding	17,68	0
ENSG00000132846	ZBED3	5	77072072	77087323	protein_coding	17,66	0
ENSG00000005882	PDK2	17	50094737	50112152	protein_coding	17,66	0
ENSG00000167371	PRRT2	16	29811382	29815892	protein_coding	17,64	0
ENSG00000175548	ALG10B	12	38316578	38329728	protein_coding	17,64	0
ENSG00000083720	OXCT1	5	41730065	41870519	protein_coding	17,61	0
ENSG00000108798	ABI3	17	49210227	49223225	protein_coding	17,60	0
ENSG00000176125	UFSP1	7	100888723	100889718	protein_coding	17,60	0
ENSG00000142677	IL22RA1	1	24119771	24143121	protein_coding	17,60	0
ENSG00000175820	CCDC168	13	102729369	102759070	protein_coding	17,58	0
ENSG00000103363	TCEB2	16	2771414	2777297	protein_coding	17,58	0
ENSG00000141480	ARRB2	17	4710489	4721499	protein_coding	17,56	0
ENSG00000121579	NAA50	3	113716460	113746300	protein_coding	17,55	0
ENSG00000139182	CLSTN3	12	7129698	7158945	protein_coding	17,55	0
ENSG00000176946	THAP4	2	241584405	241637449	protein_coding	17,54	0
ENSG00000138468	SENP7	3	101324205	101513241	protein_coding	17,54	0
ENSG00000140443	IGF1R	15	98648971	98964530	protein_coding	17,52	0
ENSG00000132286	TIMM10B	11	6481447	6484679	protein_coding	17,52	0
ENSG00000115457	IGFBP2	2	216632828	216664436	protein_coding	17,50	0
ENSG00000179083	FAM133A	X	93674013	93712274	protein_coding	17,50	0
ENSG00000126432	PRDX5	11	64318088	64321811	protein_coding	17,49	0
ENSG00000172915	NBEA	13	34942287	35673022	protein_coding	17,49	0
ENSG00000198951	NAGA	22	42058354	42070842	protein_coding	17,48	0
ENSG00000146540	C7orf50	7	996986	1138260	protein_coding	17,47	0
ENSG00000106608	URGCP	7	43875894	43926411	protein_coding	17,46	0
ENSG00000173137	ADCK5	8	144373101	144393242	protein_coding	17,45	0
ENSG00000108799	EZH1	17	42700275	42745049	protein_coding	17,45	0
ENSG00000085563	ABCB1	7	87503633	87713323	protein_coding	17,45	0
ENSG00000170445	HARS	5	140673173	140692024	protein_coding	17,43	0
ENSG00000167264	DUS2	16	67987746	68079320	protein_coding	17,42	0
ENSG00000234616	JRK	8	142657460	142681968	protein_coding	17,41	0
ENSG00000107099	DOCK8	9	214854	465259	protein_coding	17,41	0
ENSG00000187535	IFT140	16	1510427	1612110	protein_coding	17,40	0
ENSG00000168421	RHOH	4	40191053	40246967	protein_coding	17,40	0
ENSG00000133943	C14orf159	14	91060333	91225632	protein_coding	17,40	0
ENSG00000172594	SMPDL3A	6	122789049	122809720	protein_coding	17,39	0
ENSG00000124249	KCNK15	20	44745780	44752313	protein_coding	17,39	0
ENSG00000166986	MARS	12	57475445	57517569	protein_coding	17,39	0
ENSG00000101400	SNTA1	20	33407955	33443892	protein_coding	17,37	0
ENSG00000182208	MOB2	11	1469457	1501247	protein_coding	17,36	0
ENSG00000179057	IGSF22	11	18704305	18726230	protein_coding	17,36	0
ENSG00000204410	MSH5	6	31739948	31762834	protein_coding	17,36	0
ENSG00000120137	PANK3	5	168548495	168579600	protein_coding	17,34	0
ENSG00000184220	CMSS1	3	99817834	100178603	protein_coding	17,33	0
ENSG00000099904	ZDHHC8	22	20129456	20148007	protein_coding	17,33	0
ENSG00000005469	CROT	7	87345681	87399795	protein_coding	17,32	0
ENSG00000152601	MBNL1	3	152243828	152465780	protein_coding	17,32	0
ENSG00000240230	COX19	7	898778	975599	protein_coding	17,32	0
ENSG00000173141	MRPL57	13	21176645	21179084	protein_coding	17,29	0
ENSG00000241553	ARPC4	3	9792495	9807726	protein_coding	17,28	0

ENSG00000213015	ZNF580	19	55635016	55643469	protein_coding	17,27	0
ENSG00000165028	NIPSNAP3B	9	104764157	104777457	protein_coding	17,25	0
ENSG00000145194	ECE2	3	184249650	184293031	protein_coding	17,23	0
ENSG00000157240	FZD1	7	91264364	91271326	protein_coding	17,23	0
ENSG00000162390	ACOT11	1	54542257	54639192	protein_coding	17,23	0
ENSG00000160321	ZNF208	19	21932958	22010949	protein_coding	17,21	0
ENSG00000204311	DFNB59	2	178451436	178461390	protein_coding	17,21	0
ENSG00000204396	VWA7	6	31765590	31777294	protein_coding	17,20	0
ENSG00000204956	PCDHGA1	5	141330571	141512981	protein_coding	17,20	0
ENSG00000077044	DGKD	2	233354507	233472104	protein_coding	17,20	0
ENSG00000168395	ING5	2	241702035	241729478	protein_coding	17,18	0
ENSG00000132471	WBP2	17	75845699	75856507	protein_coding	17,18	0
ENSG00000173039	RELA	11	65653596	65663094	protein_coding	17,17	0
ENSG00000146701	MDH2	7	76048051	76067508	protein_coding	17,17	0
ENSG00000160094	ZNF362	1	33256545	33300719	protein_coding	17,14	0
ENSG00000125650	PSPN	19	6375148	6379058	protein_coding	17,13	0
ENSG00000106400	ZNHIT1	7	101217668	101224190	protein_coding	17,12	0
ENSG00000185163	DDX51	12	132136594	132144335	protein_coding	17,11	0
ENSG00000176142	TMEM39A	3	119429500	119468830	protein_coding	17,11	0
ENSG00000267680	ZNF224	19	44094339	44109886	protein_coding	17,11	0
ENSG00000258429	PDF	16	69328621	69330595	protein_coding	17,11	0
ENSG00000105270	CLIP3	19	36014660	36033343	protein_coding	17,11	0
ENSG00000075945	KIFAP3	1	169921326	170085208	protein_coding	17,10	0
ENSG00000100427	MLC1	22	50059391	50085902	protein_coding	17,10	0
ENSG00000068078	FGFR3	4	1793307	1808872	protein_coding	17,10	0
ENSG00000106688	SLC1A1	9	4490444	4587469	protein_coding	17,09	0
ENSG00000050438	SLC4A8	12	51391317	51515763	protein_coding	17,09	0
ENSG00000197381	ADARB1	21	45073853	45226560	protein_coding	17,07	0
ENSG00000100416	TRMU	22	46330875	46357340	protein_coding	17,07	0
ENSG00000110092	CCND1	11	69641087	69654474	protein_coding	17,07	0
ENSG00000174106	LEMD3	12	65169571	65248327	protein_coding	17,06	0
ENSG00000228474	OST4	2	27070472	27071773	protein_coding	17,05	0
ENSG00000253304	TMEM200B	1	29119428	29123935	protein_coding	17,05	0
ENSG00000075790	BCAP29	7	107579977	107629170	protein_coding	17,05	0
ENSG00000115211	EIF2B4	2	27364352	27370486	protein_coding	17,02	0
ENSG00000100626	GALNT16	14	69259277	69357033	protein_coding	17,00	0
ENSG00000184363	PKP3	11	392614	404908	protein_coding	17,00	0
ENSG00000179133	C10orf67	10	23267195	23344845	protein_coding	17,00	0
ENSG00000171388	APLN	X	129645259	129654937	protein_coding	17,00	0
ENSG00000172270	BSG	19	571277	583493	protein_coding	17,00	0
ENSG00000122140	MRPS2	9	135499984	135504673	protein_coding	16,99	0
ENSG00000146731	CCT6A	7	56051630	56063989	protein_coding	16,96	0
ENSG00000230667	SETSIP	1	92074533	92075441	protein_coding	16,94	0
ENSG00000160058	BSDC1	1	32365103	32394731	protein_coding	16,93	0
ENSG00000058262	SEC61A1	3	128051641	128071683	protein_coding	16,93	0
ENSG00000169442	CD52	1	26317957	26320523	protein_coding	16,92	0
ENSG00000028528	SNX1	15	64094123	64146090	protein_coding	16,91	0
ENSG00000008294	SPAG9	17	50962174	51120865	protein_coding	16,90	0
ENSG00000130713	EXOSC2	9	130693721	130704894	protein_coding	16,89	0
ENSG00000175213	ZNF408	11	46700818	46705912	protein_coding	16,88	0
ENSG00000076826	CAMSAP3	19	7595902	7618304	protein_coding	16,88	0
ENSG00000151348	EXT2	11	44095549	44245429	protein_coding	16,88	0
ENSG00000176438	SYNE3	14	95407266	95475836	protein_coding	16,88	0
ENSG00000116809	ZBTB17	1	15941869	15976132	protein_coding	16,88	0
ENSG00000145908	ZNF300	5	150894392	150904983	protein_coding	16,88	0
ENSG00000152154	TMEM178A	2	39664982	39717963	protein_coding	16,86	0
ENSG00000100764	PSMC1	14	90256495	90275429	protein_coding	16,85	0
ENSG00000166963	MAP1A	15	43510958	43531620	protein_coding	16,84	0

ENSG00000078142	PIK3C3	18	41955206	42087830	protein_coding	16,84	0
ENSG00000160007	ARHGAP35	19	46918676	47005077	protein_coding	16,82	0
ENSG00000160783	PMF1	1	156212993	156240042	protein_coding	16,81	0
ENSG00000180340	FZD2	17	44557459	44559570	protein_coding	16,80	0
ENSG00000278882	ENSG00000	1	120723923	120793877	protein_coding	16,80	0
ENSG00000163349	HIPK1	1	113929192	113977869	protein_coding	16,79	0
ENSG00000224420	ADM5	19	49688664	49690575	protein_coding	16,79	0
ENSG00000134202	GSTM3	1	109733932	109741038	protein_coding	16,76	0
ENSG00000172667	ZMAT3	3	179017223	179072279	protein_coding	16,75	0
ENSG00000267127	ENSG00000	18	80034346	80097088	protein_coding	16,75	0
ENSG00000250312	ZNF718	4	124480	163989	protein_coding	16,74	0
ENSG00000138430	OLA1	2	174072447	174248698	protein_coding	16,74	0
ENSG00000112031	MTRF1L	6	152987362	153002685	protein_coding	16,73	0
ENSG00000196981	WDR5B	3	122412332	122416051	protein_coding	16,73	0
ENSG00000164983	TMEM65	8	124306189	124372692	protein_coding	16,73	0
ENSG00000206053	HN1L	16	1678256	1702280	protein_coding	16,73	0
ENSG00000135114	OASL	12	121019111	121039242	protein_coding	16,70	0
ENSG00000139626	ITGB7	12	53191318	53207307	protein_coding	16,70	0
ENSG00000138759	FRAS1	4	78057570	78544269	protein_coding	16,70	0
ENSG00000166707	ZCCHC18	X	104112131	104115846	protein_coding	16,70	0
ENSG00000151553	FAM160B1	10	114821744	114899832	protein_coding	16,70	0
ENSG00000167461	RAB8A	19	16111629	16134234	protein_coding	16,70	0
ENSG00000177599	ZNF491	19	11797667	11809622	protein_coding	16,69	0
ENSG00000115091	ACTR3	2	113889960	113962596	protein_coding	16,69	0
ENSG00000112855	HARS2	5	140691426	140699291	protein_coding	16,68	0
ENSG00000204348	DXO	6	31969810	31972292	protein_coding	16,67	0
ENSG00000164305	CASP3	4	184627696	184649509	protein_coding	16,67	0
ENSG00000165118	C9orf64	9	83938311	83956986	protein_coding	16,66	0
ENSG00000188921	HACD4	9	20999515	21031636	protein_coding	16,66	0
ENSG00000168273	SMIM4	3	52534013	52579237	protein_coding	16,64	0
ENSG00000139405	RITA1	12	113185526	113192368	protein_coding	16,64	0
ENSG00000133835	HSD17B4	5	119452443	119637199	protein_coding	16,64	0
ENSG00000108591	DRG2	17	18087886	18107971	protein_coding	16,63	0
ENSG00000173821	RNF213	17	80260866	80398786	protein_coding	16,61	0
ENSG00000105723	GSK3A	19	42230186	42242625	protein_coding	16,60	0
ENSG00000183153	GJD3	17	40362931	40363815	protein_coding	16,60	0
ENSG00000158258	CLSTN2	3	139935185	140577397	protein_coding	16,57	0
ENSG00000167113	COQ4	9	128322536	128334072	protein_coding	16,56	0
ENSG00000100239	PPP6R2	22	50343304	50445090	protein_coding	16,55	0
ENSG00000196557	CACNA1H	16	1153241	1221771	protein_coding	16,55	0
ENSG00000134013	LOXL2	8	23297189	23425328	protein_coding	16,54	0
ENSG00000160256	FAM207A	21	44940010	44976989	protein_coding	16,54	0
ENSG00000142794	NBPF3	1	21440128	21485005	protein_coding	16,54	0
ENSG00000180138	CSNK1A1L	13	37103259	37105664	protein_coding	16,53	0
ENSG00000181381	DDX60L	4	168356735	168537786	protein_coding	16,53	0
ENSG00000168000	BSCL2	11	62690275	62709845	protein_coding	16,53	0
ENSG00000105778	AVL9	7	32495426	32588721	protein_coding	16,53	0
ENSG00000072849	DERL2	17	5471251	5486811	protein_coding	16,51	0
ENSG00000100991	TRPC4AP	20	35002404	35092871	protein_coding	16,51	0
ENSG00000125657	TNFSF9	19	6530999	6535928	protein_coding	16,50	0
ENSG00000174236	REP15	12	27696519	27697596	protein_coding	16,50	0
ENSG00000112782	CLIC5	6	45898451	46080395	protein_coding	16,50	0
ENSG00000165943	MOAP1	14	93182196	93184928	protein_coding	16,50	0
ENSG00000130119	GNL3L	X	54530211	54561071	protein_coding	16,49	0
ENSG00000109133	TMEM33	4	41935120	41960572	protein_coding	16,48	0
ENSG00000188428	BLOC1S5	6	8013567	8064414	protein_coding	16,48	0
ENSG00000275464	ENSG00000	21	5130871	5154734	protein_coding	16,48	0
ENSG00000048028	USP28	11	113797874	113875570	protein_coding	16,47	0

ENSG00000106028	SSBP1	7	141738321	141787922	protein_coding	16,47	0
ENSG00000169814	BTD	3	15601341	15645822	protein_coding	16,47	0
ENSG00000121680	PEX16	11	45909669	45918812	protein_coding	16,46	0
ENSG00000111605	CPSF6	12	69239537	69274358	protein_coding	16,43	0
ENSG00000066117	SMARCD1	12	50084972	50100712	protein_coding	16,43	0
ENSG00000214063	TSPAN4	11	842808	867116	protein_coding	16,42	0
ENSG00000169169	CPT1C	19	49690898	49713731	protein_coding	16,40	0
ENSG00000241360	PDXP	22	37658727	37666934	protein_coding	16,40	0
ENSG00000003056	M6PR	12	8940363	8949955	protein_coding	16,38	0
ENSG00000241685	ARPC1A	7	99325898	99388164	protein_coding	16,38	0
ENSG00000182218	HHIPL1	14	99645110	99680569	protein_coding	16,38	0
ENSG00000148180	GSN	9	121207794	121332843	protein_coding	16,37	0
ENSG00000132716	DCAF8	1	160215715	160262531	protein_coding	16,37	0
ENSG00000166439	RNF169	11	74748868	74842413	protein_coding	16,37	0
ENSG00000150637	CD226	18	69831158	69961803	protein_coding	16,35	0
ENSG00000160124	CCDC58	3	122359591	122383231	protein_coding	16,35	0
ENSG00000175110	MRPS22	3	139005806	139357223	protein_coding	16,34	0
ENSG00000135824	RGS8	1	182641816	182684576	protein_coding	16,33	0
ENSG00000162825	NBPF20	1	145289900	145405778	protein_coding	16,33	0
ENSG00000095485	CWF19L1	10	100232298	100267680	protein_coding	16,32	0
ENSG00000174547	MRPL11	11	66435075	66466738	protein_coding	16,32	0
ENSG00000142949	PTPRF	1	43525187	43623666	protein_coding	16,32	0
ENSG00000108639	SYNGR2	17	78168558	78173527	protein_coding	16,30	0
ENSG00000134574	DDB2	11	47214465	47239240	protein_coding	16,30	0
ENSG00000126860	EVI2A	17	31317560	31321884	protein_coding	16,30	0
ENSG00000261272	MUC22	6	31010474	31035402	protein_coding	16,30	0
ENSG00000172061	LRRC15	3	194355247	194369743	protein_coding	16,30	0
ENSG00000132681	ATP1A4	1	160151570	160186977	protein_coding	16,30	0
ENSG00000240563	L1TD1	1	62194831	62212328	protein_coding	16,29	0
ENSG00000113448	PDE4D	5	58969038	60522120	protein_coding	16,23	0
ENSG00000134086	VHL	3	10141008	10152220	protein_coding	16,23	0
ENSG00000100034	PPM1F	22	21919420	21952837	protein_coding	16,22	0
ENSG00000117707	PROX1	1	213983181	214041502	protein_coding	16,22	0
ENSG00000092847	AGO1	1	35869808	35930528	protein_coding	16,21	0
ENSG00000099994	SUSD2	22	24181259	24189110	protein_coding	16,20	0
ENSG00000074966	TXK	4	48066393	48134256	protein_coding	16,20	0
ENSG00000162669	HFM1	1	91260766	91404869	protein_coding	16,20	0
ENSG00000125249	RAP2A	13	97434222	97469128	protein_coding	16,19	0
ENSG00000068971	PPP2R5B	11	64917553	64934473	protein_coding	16,18	0
ENSG00000198723	C19orf45	19	7492976	7508450	protein_coding	16,18	0
ENSG00000124422	USP22	17	20999593	21043760	protein_coding	16,18	0
ENSG00000162650	ATXN7L2	1	109483479	109492804	protein_coding	16,16	0
ENSG00000100461	RBM23	14	22893206	22919184	protein_coding	16,14	0
ENSG00000171792	RHNO1	12	2876258	2889523	protein_coding	16,13	0
ENSG00000129167	TPH1	11	18017564	18042426	protein_coding	16,13	0
ENSG00000099308	MAST3	19	18097793	18151692	protein_coding	16,12	0
ENSG00000243147	MRPL33	2	27771717	27988087	protein_coding	16,12	0
ENSG00000102144	PGK1	X	78065188	78129296	protein_coding	16,12	0
ENSG00000163545	NUAK2	1	205302059	205321791	protein_coding	16,12	0
ENSG00000107902	LHPP	10	124461834	124617888	protein_coding	16,11	0
ENSG00000132518	GUCY2D	17	8002594	8020339	protein_coding	16,10	0
ENSG00000158517	NCF1	7	74773962	74789315	protein_coding	16,10	0
ENSG00000173757	STAT5B	17	42199168	42276707	protein_coding	16,10	0
ENSG00000115414	FN1	2	215360440	215436172	protein_coding	16,09	0
ENSG00000114745	GORASP1	3	39096659	39108363	protein_coding	16,09	0
ENSG00000160401	CFAP157	9	127706989	127716002	protein_coding	16,07	0
ENSG00000172932	ANKRD13D	11	67288547	67302485	protein_coding	16,06	0
ENSG00000115808	STRN	2	36843640	36966472	protein_coding	16,05	0

ENSG00000168779	SHOX2	3	158095954	158106503	protein_coding	16,04	0
ENSG00000041802	LSG1	3	194640788	194672477	protein_coding	16,04	0
ENSG00000117450	PRDX1	1	45511036	45523047	protein_coding	16,04	0
ENSG00000119689	DLST	14	74881891	74903745	protein_coding	16,01	0
ENSG00000082781	ITGB5	3	124761948	124901418	protein_coding	16,01	0
ENSG00000182749	PAQR7	1	25861210	25871253	protein_coding	16,01	0
ENSG00000184009	ACTG1	17	81509971	81523847	protein_coding	16,00	0
ENSG00000149474	CSRP2BP	20	18138118	18188387	protein_coding	16,00	0
ENSG00000012174	MBTPS2	X	21839636	21885424	protein_coding	16,00	0
ENSG00000001167	NFYA	6	41072945	41099976	protein_coding	15,99	0
ENSG00000179091	CYC1	8	144095027	144097525	protein_coding	15,98	0
ENSG00000166949	SMAD3	15	67063763	67195195	protein_coding	15,98	0
ENSG00000100811	YY1	14	100238298	100282792	protein_coding	15,97	0
ENSG00000155324	GRAMD3	5	126360132	126496494	protein_coding	15,95	0
ENSG00000064607	SUGP2	19	18990888	19034023	protein_coding	15,94	0
ENSG00000110042	DTX4	11	59171430	59208587	protein_coding	15,93	0
ENSG00000123091	RNF11	1	51236271	51273455	protein_coding	15,93	0
ENSG00000196632	WNK3	X	54192823	54358642	protein_coding	15,91	0
ENSG00000172878	METAP1D	2	171999583	172082430	protein_coding	15,91	0
ENSG00000145244	CORIN	4	47593998	47838106	protein_coding	15,90	0
ENSG00000159461	AMFR	16	56361452	56425538	protein_coding	15,90	0
ENSG00000066027	PPP2R5A	1	212285537	212361863	protein_coding	15,90	0
ENSG00000156239	N6AMT1	21	28872191	28885371	protein_coding	15,89	0
ENSG00000049769	PPP1R3F	X	49269843	49301461	protein_coding	15,89	0
ENSG00000105379	ETFB	19	51345169	51366418	protein_coding	15,89	0
ENSG00000124713	GNMT	6	42960758	42963880	protein_coding	15,88	0
ENSG00000006125	AP2B1	17	35578046	35726409	protein_coding	15,86	0
ENSG00000196757	ZNF700	19	11925068	11950773	protein_coding	15,84	0
ENSG00000121940	CLCC1	1	108929508	108963457	protein_coding	15,83	0
ENSG00000172725	CORO1B	11	67435510	67443821	protein_coding	15,83	0
ENSG00000198157	HMGN5	X	81113701	81201942	protein_coding	15,81	0
ENSG00000176681	LRRC37A	17	46292733	46337794	protein_coding	15,80	0
ENSG00000146005	PSD2	5	139795821	139844466	protein_coding	15,80	0
ENSG00000145287	PLAC8	4	83090048	83137075	protein_coding	15,80	0
ENSG00000132688	NES	1	156668763	156677397	protein_coding	15,79	0
ENSG00000138670	RASGEF1B	4	81426393	82044244	protein_coding	15,79	0
ENSG00000108588	CCDC47	17	63745250	63776351	protein_coding	15,78	0
ENSG00000100490	CDKL1	14	50329404	50416461	protein_coding	15,77	0
ENSG00000132773	TOE1	1	45339670	45343975	protein_coding	15,77	0
ENSG00000090863	GLG1	16	74447427	74607144	protein_coding	15,77	0
ENSG00000130529	TRPM4	19	49157741	49211836	protein_coding	15,77	0
ENSG00000101162	TUBB1	20	59019254	59026654	protein_coding	15,76	0
ENSG00000138668	HNRNPD	4	82352498	82374503	protein_coding	15,76	0
ENSG00000104368	PLAT	8	42175233	42207724	protein_coding	15,74	0
ENSG00000175782	SLC35E3	12	68746106	68793964	protein_coding	15,73	0
ENSG00000075391	RASAL2	1	178094141	178484147	protein_coding	15,72	0
ENSG00000154263	ABCA10	17	69147214	69244846	protein_coding	15,71	0
ENSG00000130772	MED18	1	28329002	28335967	protein_coding	15,70	0
ENSG00000188092	GPR89B	1	147928393	147993521	protein_coding	15,70	0
ENSG00000125531	C20orf195	20	63547891	63556708	protein_coding	15,70	0
ENSG00000197471	SPN	16	29662979	29670876	protein_coding	15,70	0
ENSG00000169885	CALML6	1	1914827	1917296	protein_coding	15,70	0
ENSG00000038532	CLEC16A	16	10944488	11182189	protein_coding	15,69	0
ENSG00000100593	ISM2	14	77474394	77498850	protein_coding	15,67	0
ENSG00000147121	KRBOX4	X	46446857	46497422	protein_coding	15,67	0
ENSG00000064115	TM7SF3	12	26973195	27014434	protein_coding	15,66	0
ENSG00000145331	TRMT10A	4	99546709	99564032	protein_coding	15,64	0
ENSG00000129911	KLF16	19	1852399	1863568	protein_coding	15,63	0

ENSG00000160345	C9orf116	9	135495181	135501734	protein_coding		15,63	0
ENSG00000123136	DDX39A	19	14408819	14419383	protein_coding		15,62	0
ENSG00000258643	BCL2L2-PAB	14	23306835	23325369	protein_coding		15,60	0
ENSG00000174807	CD248	11	66314487	66317044	protein_coding		15,60	0
ENSG00000196932	TMEM26	10	61406643	61453450	protein_coding		15,60	0
ENSG00000068323	TFE3	X	49028726	49043486	protein_coding		15,59	0
ENSG00000136574	GATA4	8	11676959	11760002	protein_coding		15,59	0
ENSG00000101000	PROCR	20	35172073	35216240	protein_coding		15,59	0
ENSG00000184640	09/01/2009	17	77280569	77500596	protein_coding		15,59	0
ENSG00000127022	CANX	5	179678628	179730925	protein_coding		15,58	0
ENSG00000174373	RALGAPA1	14	35538352	35809304	protein_coding		15,57	0
ENSG00000163399	ATP1A1	1	116372668	116410261	protein_coding		15,57	0
ENSG00000170903	MSANTD4	11	105995623	106022403	protein_coding		15,55	0
ENSG00000150753	CCT5	5	10249921	10266412	protein_coding		15,55	0
ENSG00000132781	MUTYH	1	45329163	45340470	protein_coding		15,52	0
ENSG00000139880	CDH24	14	23047062	23057538	protein_coding		15,52	0
ENSG00000177752	YIPF7	4	44622065	44678556	protein_coding		15,50	0
ENSG00000173110	HSPA6	1	161524540	161526910	protein_coding		15,50	0
ENSG00000166902	MRPL16	11	59806135	59810872	protein_coding		15,49	0
ENSG00000105707	HPN	19	35040506	35066571	protein_coding		15,46	0
ENSG00000175137	SH3BP5L	1	248810446	248826633	protein_coding		15,46	0
ENSG00000111641	NOP2	12	6556863	6568691	protein_coding		15,46	0
ENSG00000171121	KCNMB3	3	179236691	179267002	protein_coding		15,45	0
ENSG00000167775	CD320	19	8302127	8308356	protein_coding		15,45	0
ENSG00000187513	GJA4	1	34792998	34795747	protein_coding		15,44	0
ENSG00000007968	E2F2	1	23506430	23531220	protein_coding		15,43	0
ENSG00000187957	DNER	2	229357629	229714558	protein_coding		15,42	0
ENSG00000162946	DISC1	1	231626815	232041272	protein_coding		15,41	0
ENSG00000167419	LPO	17	58218548	58268518	protein_coding		15,40	0
ENSG00000124772	CPNE5	6	36740775	36840002	protein_coding		15,40	0
ENSG00000122176	FMOD	1	203340628	203351489	protein_coding		15,40	0
ENSG00000185127	C6orf120	6	169702190	169704856	protein_coding		15,39	0
ENSG00000135736	CCDC102A	16	57512178	57536599	protein_coding		15,39	0
ENSG00000002016	RAD52	12	912077	990053	protein_coding		15,38	0
ENSG00000142675	CNKSR1	1	26177403	26189886	protein_coding		15,38	0
ENSG00000070269	TMEM260	14	56488354	56650606	protein_coding		15,37	0
ENSG00000116151	MORN1	1	2321253	2391707	protein_coding		15,37	0
ENSG00000135436	FAM186B	12	49582885	49605639	protein_coding		15,36	0
ENSG00000163472	TMEM79	1	156282935	156293185	protein_coding		15,35	0
ENSG00000132510	KDM6B	17	7839904	7854796	protein_coding		15,34	0
ENSG00000173166	RAPH1	2	203394345	203535410	protein_coding		15,32	0
ENSG00000198964	SGMS1	10	50305586	50625163	protein_coding		15,32	0
ENSG00000128039	SRD5A3	4	55346109	55373096	protein_coding		15,32	0
ENSG00000150459	SAP18	13	21140514	21149084	protein_coding		15,32	0
ENSG00000173456	RNF26	11	119334527	119337313	protein_coding		15,32	0
ENSG00000089248	ERP29	12	112013316	112023451	protein_coding		15,32	0
ENSG00000160949	TONSL	8	144428775	144444444	protein_coding		15,32	0
ENSG00000163655	GMPS	3	155870536	155944026	protein_coding		15,31	0
ENSG00000197405	C5AR1	19	47290023	47322066	protein_coding		15,31	0
ENSG00000168439	STIP1	11	64185272	64204543	protein_coding		15,31	0
ENSG00000165555	NOXRED1	14	77394021	77423517	protein_coding		15,30	0
ENSG00000162631	NTNG1	1	107140007	107483458	protein_coding		15,30	0
ENSG00000108523	RNF167	17	4940008	4945222	protein_coding		15,30	0
ENSG00000281079	ENSG00000[illegible]	17	42993098	42993781	protein_coding		15,27	0
ENSG00000167733	HSD11B1L	19	5680604	5688523	protein_coding		15,27	0
ENSG00000189241	TSPYL1	6	116276578	116279903	protein_coding		15,26	0
ENSG00000119760	SUPT7L	2	27650812	27663840	protein_coding		15,23	0
ENSG00000180543	TSPYL5	8	97273474	97277964	protein_coding		15,22	0

ENSG00000167528	ZNF641	12	48337180	48351414	protein_coding	15,22	0
ENSG00000172404	DNAJB7	22	40859549	40862126	protein_coding	15,22	0
ENSG00000080845	DLGAP4	20	36306336	36528637	protein_coding	15,21	0
ENSG00000112893	MAN2A1	5	109689366	109869625	protein_coding	15,21	0
ENSG00000158955	WNT9B	17	46833201	46886730	protein_coding	15,20	0
ENSG00000262576	PCDHGA4	5	141355025	141512979	protein_coding	15,20	0
ENSG00000243989	ACY1	3	51983340	51989197	protein_coding	15,20	0
ENSG00000100109	TFIP11	22	26491225	26512505	protein_coding	15,20	0
ENSG00000148308	GTF3C5	9	133030675	133058503	protein_coding	15,16	0
ENSG00000176894	PXMP2	12	132687606	132704991	protein_coding	15,15	0
ENSG00000239697	TNFSF12	17	7548891	7557890	protein_coding	15,15	0
ENSG00000196182	STK40	1	36339624	36385896	protein_coding	15,15	0
ENSG00000163126	ANKRD23	2	96824526	96857934	protein_coding	15,14	0
ENSG00000164647	STEAP1	7	90154375	90164829	protein_coding	15,13	0
ENSG00000121671	CRY2	11	45847118	45883248	protein_coding	15,13	0
ENSG00000108561	C1QBP	17	5432777	5448830	protein_coding	15,12	0
ENSG00000179832	MROH1	8	144148016	144261940	protein_coding	15,11	0
ENSG00000131355	ADGRE3	19	14619117	14690027	protein_coding	15,10	0
ENSG00000170482	SLC23A1	5	139367196	139384553	protein_coding	15,10	0
ENSG00000280649	ENSG000002	1	148458814	148459871	protein_coding	15,10	0
ENSG00000165185	KIAA1958	9	112486847	112669397	protein_coding	15,10	0
ENSG00000183977	PP2D1	3	19979961	20012330	protein_coding	15,09	0
ENSG00000128928	IVD	15	40405795	40435947	protein_coding	15,09	0
ENSG00000188859	FAM78B	1	166057426	166166969	protein_coding	15,09	0
ENSG00000173726	TOMM20	1	235109336	235128936	protein_coding	15,08	0
ENSG00000172803	SNX32	11	65833641	65856896	protein_coding	15,05	0
ENSG00000049618	ARID1B	6	156777374	157210779	protein_coding	15,02	0
ENSG00000085063	CD59	11	33698261	33736445	protein_coding	15,01	0
ENSG00000183307	CECR6	22	17116299	17121367	protein_coding	15,00	0
ENSG00000179761	PIPOX	17	28950513	29057220	protein_coding	15,00	0
ENSG00000095970	TREM2	6	41158506	41163186	protein_coding	15,00	0
ENSG00000254221	PCDHGB1	5	141350102	141512979	protein_coding	15,00	0
ENSG00000181322	NME9	3	138261437	138329886	protein_coding	15,00	0
ENSG00000185112	FAM43A	3	194686544	194689037	protein_coding	15,00	0
ENSG00000020633	RUNX3	1	24899511	24965121	protein_coding	15,00	0
ENSG00000013375	PGM3	6	83161150	83193936	protein_coding	14,99	0
ENSG00000044446	PHKA2	X	18892300	18984598	protein_coding	14,99	0
ENSG00000073711	PPP2R3A	3	135965673	136147891	protein_coding	14,98	0
ENSG00000166783	KIAA0430	16	15594386	15643166	protein_coding	14,98	0
ENSG00000177374	HIC1	17	2054154	2063241	protein_coding	14,96	0
ENSG00000251192	ZNF674	X	46497727	46545457	protein_coding	14,96	0
ENSG00000168036	CTNNB1	3	41194837	41260096	protein_coding	14,93	0
ENSG00000151116	UEVLD	11	18529609	18588747	protein_coding	14,93	0
ENSG00000100263	RHBDD3	22	29259852	29268209	protein_coding	14,92	0
ENSG00000165233	CARD19	9	93096218	93113283	protein_coding	14,92	0
ENSG00000011295	TTC19	17	15999380	16045015	protein_coding	14,91	0
ENSG00000213658	LAT	16	28984826	28990783	protein_coding	14,90	0
ENSG00000079689	SCGN	6	25652201	25701783	protein_coding	14,90	0
ENSG00000117593	DARS2	1	173824503	173858546	protein_coding	14,89	0
ENSG00000156253	RWDD2B	21	29004384	29019378	protein_coding	14,89	0
ENSG00000197892	KIF13B	8	29067279	29263124	protein_coding	14,89	0
ENSG00000049768	FOXP3	X	49250436	49264826	protein_coding	14,89	0
ENSG00000110717	NDUFS8	11	68030617	68036644	protein_coding	14,88	0
ENSG00000115677	HDLBP	2	241227264	241317061	protein_coding	14,87	0
ENSG00000133247	SUV420H2	19	55339853	55348120	protein_coding	14,87	0
ENSG00000196591	HDAC2	6	113933028	114011308	protein_coding	14,87	0
ENSG00000100796	PPP4R3A	14	91457611	91510554	protein_coding	14,87	0
ENSG00000067221	STOML1	15	73978923	73994622	protein_coding	14,86	0

ENSG00000071794	HLTF	3	149030127	149086554	protein_coding	14,84	0
ENSG00000241399	CD302	2	159768630	159798255	protein_coding	14,82	0
ENSG00000183207	RUVBL2	19	48993448	49015995	protein_coding	14,81	0
ENSG00000128271	ADORA2A	22	24417879	24442360	protein_coding	14,80	0
ENSG00000177721	ANXA2R	5	43039081	43043170	protein_coding	14,80	0
ENSG00000167380	ZNF226	19	44165073	44178381	protein_coding	14,79	0
ENSG00000160584	SIK3	11	116843402	117098437	protein_coding	14,79	0
ENSG00000007168	PAFAH1B1	17	2593210	2685615	protein_coding	14,78	0
ENSG00000118894	EEF2KMT	16	5084304	5097808	protein_coding	14,77	0
ENSG00000070061	IKBKAP	9	108867517	108934116	protein_coding	14,76	0
ENSG00000167815	PRDX2	19	12796820	12801859	protein_coding	14,76	0
ENSG00000166747	AP1G1	16	71729000	71809201	protein_coding	14,75	0
ENSG00000273398	ENSG00000	2	68131238	68261230	protein_coding	14,74	0
ENSG00000006638	TBXA2R	19	3594506	3606840	protein_coding	14,73	0
ENSG00000131871	VIMP	15	101270817	101277500	protein_coding	14,73	0
ENSG00000141499	WRAP53	17	7686071	7703502	protein_coding	14,72	0
ENSG00000002919	SNX11	17	48103357	48123074	protein_coding	14,70	0
ENSG00000129214	SHBG	17	7613946	7633383	protein_coding	14,70	0
ENSG00000185905	C16orf54	16	29742463	29746006	protein_coding	14,70	0
ENSG00000137177	KIF13A	6	17759183	17987623	protein_coding	14,70	0
ENSG00000196396	PTPN1	20	50510321	50585241	protein_coding	14,70	0
ENSG00000179010	MRFAP1	4	6640091	6642745	protein_coding	14,68	0
ENSG00000124299	PEPD	19	33386950	33521794	protein_coding	14,68	0
ENSG00000139324	TMTC3	12	88142296	88199887	protein_coding	14,68	0
ENSG00000137074	APTX	9	32972606	33025168	protein_coding	14,68	0
ENSG00000164961	KIAA0196	8	125024260	125091840	protein_coding	14,67	0
ENSG00000125089	SH3TC1	4	8182072	8241803	protein_coding	14,67	0
ENSG00000125844	RRBP1	20	17613678	17682295	protein_coding	14,64	0
ENSG00000119547	ONECUT2	18	57435685	57491297	protein_coding	14,63	0
ENSG00000106460	TMEM106B	7	12211241	12243367	protein_coding	14,63	0
ENSG00000197536	C5orf56	5	132410636	132476044	protein_coding	14,62	0
ENSG00000132485	ZRANB2	1	71063291	71081297	protein_coding	14,61	0
ENSG00000156500	FAM122C	X	134796789	134854610	protein_coding	14,59	0
ENSG00000157916	RER1	1	2391775	2405444	protein_coding	14,57	0
ENSG00000166532	RIMKLB	12	8681600	8783095	protein_coding	14,57	0
ENSG00000172458	IL17D	13	20702127	20723098	protein_coding	14,56	0
ENSG00000139173	TMEM117	12	43835967	44389762	protein_coding	14,56	0
ENSG00000197982	C1orf122	1	37806979	37809454	protein_coding	14,56	0
ENSG00000128604	IRF5	7	128937612	128950035	protein_coding	14,55	0
ENSG00000108515	ENO3	17	4948092	4957131	protein_coding	14,55	0
ENSG00000105393	BABAM1	19	17267350	17281249	protein_coding	14,53	0
ENSG00000183665	TRMT12	8	124450820	124462150	protein_coding	14,51	0
ENSG00000204946	ZNF783	7	149262171	149297302	protein_coding	14,50	0
ENSG00000196405	EVL	14	99971449	100144236	protein_coding	14,49	0
ENSG00000166557	TMED3	15	79311062	79427432	protein_coding	14,49	0
ENSG00000025796	SEC63	6	107867756	107958189	protein_coding	14,49	0
ENSG00000109381	ELF2	4	139028112	139177218	protein_coding	14,49	0
ENSG00000169085	C8orf46	8	66460003	66518524	protein_coding	14,48	0
ENSG00000034152	MAP2K3	17	21284672	21315240	protein_coding	14,48	0
ENSG00000062524	LTK	15	41503638	41513887	protein_coding	14,48	0
ENSG00000144231	POLR2D	2	127843551	127858157	protein_coding	14,46	0
ENSG00000196628	TCF4	18	55222331	55664787	protein_coding	14,46	0
ENSG00000083807	SLC27A5	19	58479512	58512413	protein_coding	14,46	0
ENSG00000163468	CCT3	1	156308968	156367873	protein_coding	14,45	0
ENSG00000120693	SMAD9	13	36844831	36920765	protein_coding	14,42	0
ENSG00000176986	SEC24C	10	73744384	73772161	protein_coding	14,42	0
ENSG00000186074	CD300LF	17	74694311	74712978	protein_coding	14,40	0
ENSG00000124602	UNC5CL	6	41026911	41039217	protein_coding	14,40	0

ENSG00000268279	ENSG00000[illegible]	3	14135217	14148252	protein_coding	14,40	0
ENSG00000186891	TNFRSF18	1	1203508	1206691	protein_coding	14,40	0
ENSG00000109606	DHX15	4	24517441	24584550	protein_coding	14,40	0
ENSG00000263002	ZNF234	19	44141557	44160309	protein_coding	14,39	0
ENSG00000093217	XYLB	3	38346760	38421348	protein_coding	14,39	0
ENSG00000168575	SLC20A2	8	42416475	42541926	protein_coding	14,39	0
ENSG00000138698	RAP1GDS1	4	98261384	98443861	protein_coding	14,38	0
ENSG00000133243	BTBD2	19	1985438	2034881	protein_coding	14,37	0
ENSG00000166025	AMOTL1	11	94706431	94876753	protein_coding	14,37	0
ENSG00000129991	TNNI3	19	55151767	55157773	protein_coding	14,35	0
ENSG00000169398	PTK2	8	140657900	141002216	protein_coding	14,33	0
ENSG00000154099	DNAAF1	16	84145287	84178767	protein_coding	14,33	0
ENSG00000239388	ASB14	3	57268347	57292682	protein_coding	14,31	0
ENSG00000166847	DCTN5	16	23641392	23677455	protein_coding	14,31	0
ENSG00000267060	PTGES3L	17	42968088	42980433	protein_coding	14,30	0
ENSG00000214872	SMTNL1	11	57542641	57550274	protein_coding	14,30	0
ENSG00000278868	ENSG00000[illegible]	1	27850574	27850744	protein_coding	14,30	0
ENSG00000156639	ZFAND3	6	37819499	38154624	protein_coding	14,29	0
ENSG00000133812	SBF2	11	9778667	10294207	protein_coding	14,29	0
ENSG00000174437	ATP2A2	12	110280756	110351093	protein_coding	14,27	0
ENSG00000179041	RRS1	8	66429028	66430733	protein_coding	14,27	0
ENSG00000006016	CRLF1	19	18572220	18607741	protein_coding	14,26	0
ENSG00000143224	PPOX	1	161166410	161178013	protein_coding	14,26	0
ENSG00000132824	SERINC3	20	44496221	44522109	protein_coding	14,26	0
ENSG00000068400	GRIPAP1	X	48973720	49002264	protein_coding	14,25	0
ENSG00000177731	FLII	17	18244836	18258916	protein_coding	14,25	0
ENSG00000115904	SOS1	2	38981396	39124345	protein_coding	14,24	0
ENSG00000031823	RANBP3	19	5916139	5978142	protein_coding	14,23	0
ENSG00000039123	SKIV2L2	5	55307760	55425581	protein_coding	14,23	0
ENSG00000184402	SS18L1	20	62143795	62182484	protein_coding	14,23	0
ENSG00000102226	USP11	X	47232690	47248328	protein_coding	14,23	0
ENSG00000059122	FLYWCH1	16	2911937	2951208	protein_coding	14,22	0
ENSG00000204314	PRRT1	6	32148359	32154373	protein_coding	14,22	0
ENSG00000198546	ZNF511	10	133308475	133313162	protein_coding	14,22	0
ENSG00000130177	CDC16	13	114234887	114272723	protein_coding	14,21	0
ENSG00000108599	AKAP10	17	19904302	19978343	protein_coding	14,21	0
ENSG00000152818	UTRN	6	144285701	144853034	protein_coding	14,21	0
ENSG00000105383	CD33	19	51225064	51243860	protein_coding	14,20	0
ENSG00000165775	FUNDC2	X	155025980	155060303	protein_coding	14,19	0
ENSG00000203791	METTL10	10	124748149	124791870	protein_coding	14,19	0
ENSG00000196517	SLC6A9	1	43991500	44031467	protein_coding	14,17	0
ENSG00000197860	SGTB	5	65665928	65723035	protein_coding	14,17	0
ENSG00000065060	UHRF1BP1	6	34792015	34883138	protein_coding	14,14	0
ENSG00000112599	GUCA1B	6	42184401	42194916	protein_coding	14,12	0
ENSG00000148296	SURF6	9	133328774	133336398	protein_coding	14,11	0
ENSG00000175003	SLC22A1	6	160121789	160158718	protein_coding	14,10	0
ENSG00000154252	GAL3ST2	2	241776825	241804208	protein_coding	14,10	0
ENSG00000180616	SSTR2	17	73165012	73176633	protein_coding	14,10	0
ENSG00000197415	VEPH1	3	157259742	157533619	protein_coding	14,08	0
ENSG00000167136	ENDOG	9	128818474	128822677	protein_coding	14,08	0
ENSG00000157911	PEX10	1	2403964	2413797	protein_coding	14,06	0
ENSG00000115841	RMDN2	2	37923187	38067142	protein_coding	14,05	0
ENSG00000142687	KIAA0319L	1	35433490	35557950	protein_coding	14,05	0
ENSG00000173744	AGFG1	2	227472152	227561214	protein_coding	14,03	0
ENSG00000197724	PHF2	9	93576407	93679587	protein_coding	14,03	0
ENSG00000100564	PIGH	14	67581955	67600287	protein_coding	14,02	0
ENSG00000176783	RUFY1	5	179550558	179610026	protein_coding	14,01	0
ENSG00000118855	MFSD1	3	158732198	158829719	protein_coding	14,00	0

ENSG00000129244	ATP1B2	17	7646627	7657768	protein_coding	14,00	0
ENSG00000122367	LDB3	10	86668449	86736068	protein_coding	14,00	0
ENSG00000124564	SLC17A3	6	25833066	25882286	protein_coding	14,00	0
ENSG00000007062	PROM1	4	15963076	16084378	protein_coding	14,00	0
ENSG00000163735	CXCL5	4	73995642	73998779	protein_coding	14,00	0
ENSG00000132031	MATN3	2	19992111	20012694	protein_coding	14,00	0
ENSG00000184599	FAM19A3	1	112720419	112727235	protein_coding	14,00	0
ENSG00000266028	SRGAP2	1	206203345	206464443	protein_coding	14,00	0
ENSG00000163444	TMEM183A	1	203007386	203024848	protein_coding	14,00	0
ENSG00000165912	PACSIN3	11	47177525	47186443	protein_coding	13,98	0
ENSG00000162642	C1orf52	1	85249953	85259672	protein_coding	13,98	0
ENSG00000131876	SNRPA1	15	101281510	101295282	protein_coding	13,96	0
ENSG00000165671	NSD1	5	177133025	177300215	protein_coding	13,96	0
ENSG00000180817	PPA1	10	70202830	70233911	protein_coding	13,95	0
ENSG00000104228	TRIM35	8	27284887	27311319	protein_coding	13,95	0
ENSG00000139946	PELI2	14	56117814	56301526	protein_coding	13,93	0
ENSG00000173486	FKBP2	11	64241003	64244132	protein_coding	13,92	0
ENSG00000187741	FANCA	16	89737549	89816657	protein_coding	13,91	0
ENSG00000167483	FAM129C	19	17523301	17553839	protein_coding	13,90	0
ENSG00000260300	ENSG00000	16	83908132	83951445	protein_coding	13,90	0
ENSG00000243708	PLA2G4B	15	41837775	41848147	protein_coding	13,90	0
ENSG00000103222	ABCC1	16	15949577	16143074	protein_coding	13,90	0
ENSG00000089847	ANKRD24	19	4183354	4224814	protein_coding	13,88	0
ENSG00000149357	LAMTOR1	11	72085895	72103387	protein_coding	13,88	0
ENSG00000205060	SLC35B4	7	134289332	134317051	protein_coding	13,87	0
ENSG00000204991	SPIRE2	16	89818179	89871319	protein_coding	13,86	0
ENSG00000103707	MTFMT	15	65001512	65029639	protein_coding	13,86	0
ENSG00000217930	PAM16	16	4331549	4355607	protein_coding	13,85	0
ENSG00000108443	RPS6KB1	17	59893046	59950564	protein_coding	13,85	0
ENSG00000107165	TYRP1	9	12685439	12710290	protein_coding	13,84	0
ENSG00000149428	HYOU1	11	119044189	119057202	protein_coding	13,84	0
ENSG00000134323	MYCN	2	15940564	15947007	protein_coding	13,83	0
ENSG00000155850	SLC26A2	5	149960737	149993455	protein_coding	13,83	0
ENSG00000188191	PRKAR1B	7	549197	727650	protein_coding	13,83	0
ENSG00000062725	APPBP2	17	60443149	60526219	protein_coding	13,81	0
ENSG00000197020	ZNF100	19	21722766	21767628	protein_coding	13,81	0
ENSG00000277075	HIST1H2AE	6	26216975	26217483	protein_coding	13,80	0
ENSG00000170469	SPATA24	5	139396563	139404088	protein_coding	13,80	0
ENSG00000174348	PODN	1	53062052	53085502	protein_coding	13,80	0
ENSG00000115233	PSMD14	2	161308038	161411717	protein_coding	13,80	0
ENSG00000146963	LUC7L2	7	139340359	139423457	protein_coding	13,79	0
ENSG00000164180	TMEM161B	5	88189633	88269476	protein_coding	13,77	0
ENSG00000158882	TOMM40L	1	161225939	161230744	protein_coding	13,76	0
ENSG00000173889	PHC3	3	170086732	170181749	protein_coding	13,76	0
ENSG00000067829	IDH3G	X	153785766	153794523	protein_coding	13,76	0
ENSG00000170291	ELP5	17	7251416	7259940	protein_coding	13,75	0
ENSG00000179833	SERTAD2	2	64631621	64751005	protein_coding	13,74	0
ENSG00000171368	TPPP	5	659862	693395	protein_coding	13,74	0
ENSG00000126106	TMEM53	1	44635238	44674555	protein_coding	13,74	0
ENSG00000130363	RSPH3	6	158972871	159000187	protein_coding	13,74	0
ENSG00000100813	ACIN1	14	23058564	23095614	protein_coding	13,74	0
ENSG00000166548	TK2	16	66508003	66552544	protein_coding	13,73	0
ENSG00000137337	MDC1	6	30699807	30717889	protein_coding	13,73	0
ENSG00000164054	SHISA5	3	48467798	48504826	protein_coding	13,72	0
ENSG00000089505	CMTM1	16	66566393	66579137	protein_coding	13,70	0
ENSG00000146530	VWDE	7	12330885	12403941	protein_coding	13,70	0
ENSG00000172757	CFL1	11	65823022	65862026	protein_coding	13,68	0
ENSG00000074621	SLC24A1	15	65611366	65660995	protein_coding	13,68	0

ENSG00000198663	C6orf89	6	36871870	36928964	protein_coding	13,67	0
ENSG00000147394	ZNF185	X	152914442	152973480	protein_coding	13,65	0
ENSG00000146729	GBAS	7	55951793	56000181	protein_coding	13,64	0
ENSG00000165983	PTER	10	16436943	16513745	protein_coding	13,64	0
ENSG00000110066	SUV420H1	11	68154863	68213828	protein_coding	13,64	0
ENSG00000111785	RIC8B	12	106774595	106889316	protein_coding	13,63	0
ENSG00000122477	LRRC39	1	100148449	100178273	protein_coding	13,63	0
ENSG00000122034	GTF3A	13	27424544	27435823	protein_coding	13,63	0
ENSG00000168710	AHCYL1	1	109984686	110023741	protein_coding	13,62	0
ENSG00000167103	PIP5KL1	9	127920879	127930797	protein_coding	13,62	0
ENSG00000178980	SEPW1	19	47778572	47784686	protein_coding	13,62	0
ENSG00000110013	SIAE	11	124633113	124695707	protein_coding	13,60	0
ENSG00000152137	HSPB8	12	119178642	119221131	protein_coding	13,60	0
ENSG00000152779	SLC16A12	10	89430299	89556641	protein_coding	13,60	0
ENSG00000142765	SYTL1	1	27342020	27353937	protein_coding	13,60	0
ENSG00000114126	TFDP2	3	141944428	142149544	protein_coding	13,60	0
ENSG00000047188	YTHDC2	5	113513683	113595285	protein_coding	13,60	0
ENSG00000185104	FAF1	1	50437028	50960263	protein_coding	13,59	0
ENSG00000025156	HSF2	6	122399546	122433119	protein_coding	13,58	0
ENSG00000114491	UMPS	3	124730366	124749273	protein_coding	13,57	0
ENSG00000174839	DENND6A	3	57625457	57693089	protein_coding	13,57	0
ENSG00000128274	A4GALT	22	42692121	42721298	protein_coding	13,57	0
ENSG00000103381	CPPED1	16	12659799	12804017	protein_coding	13,56	0
ENSG00000169297	NR0B1	X	30304206	30309598	protein_coding	13,55	0
ENSG00000105655	ISYNA1	19	18434388	18438301	protein_coding	13,54	0
ENSG00000274180	NATD1	17	21238870	21253410	protein_coding	13,53	0
ENSG00000259494	MRPL46	15	88459476	88467419	protein_coding	13,53	0
ENSG00000133706	LARS	5	146113038	146182660	protein_coding	13,52	0
ENSG00000185250	PPIL6	6	109390215	109441171	protein_coding	13,52	0
ENSG00000071991	CDH19	18	66501083	66604138	protein_coding	13,50	0
ENSG00000006210	CX3CL1	16	57372458	57385048	protein_coding	13,50	0
ENSG00000175538	KCNE3	11	74454841	74467729	protein_coding	13,50	0
ENSG00000143199	ADCY10	1	167809388	167914215	protein_coding	13,50	0
ENSG00000129933	MAU2	19	19320681	19358755	protein_coding	13,50	0
ENSG00000166446	CDYL2	16	80597906	80804329	protein_coding	13,49	0
ENSG00000136381	IREB2	15	78437431	78501456	protein_coding	13,48	0
ENSG00000167971	CASKIN1	16	2177180	2196525	protein_coding	13,48	0
ENSG00000105835	NAMPT	7	106248285	106286326	protein_coding	13,43	0
ENSG00000139718	SETD1B	12	121804180	121832584	protein_coding	13,43	0
ENSG00000088876	ZNF343	20	2481817	2524702	protein_coding	13,42	0
ENSG00000139194	RBP5	12	7123684	7128942	protein_coding	13,41	0
ENSG00000158528	PPP1R9A	7	94907202	95296415	protein_coding	13,40	0
ENSG00000175893	ZDHHC21	9	14611071	14693471	protein_coding	13,39	0
ENSG00000141556	TBCD	17	82752064	82945922	protein_coding	13,36	0
ENSG00000128591	FLNC	7	128830377	128859274	protein_coding	13,36	0
ENSG00000100220	RTCB	22	32387582	32412255	protein_coding	13,34	0
ENSG00000186660	ZFP91	11	58579111	58621042	protein_coding	13,32	0
ENSG00000132434	LANCL2	7	55365448	55433742	protein_coding	13,31	0
ENSG00000145016	RUBCN	3	197671393	197749727	protein_coding	13,30	0
ENSG00000107745	MICU1	10	72367327	72626191	protein_coding	13,30	0
ENSG00000096968	JAK2	9	4985033	5128183	protein_coding	13,30	0
ENSG00000137936	BCAR3	1	93561786	93847150	protein_coding	13,30	0
ENSG00000236279	CLEC2L	7	139523856	139544984	protein_coding	13,30	0
ENSG00000173200	PARP15	3	122577602	122639047	protein_coding	13,30	0
ENSG00000182986	ZNF320	19	52863790	52897693	protein_coding	13,30	0
ENSG00000100865	CINP	14	102341102	102362916	protein_coding	13,28	0
ENSG00000074582	BCS1L	2	218658764	218663443	protein_coding	13,28	0
ENSG00000132763	MMACHC	1	45500053	45513382	protein_coding	13,28	0

ENSG00000174652	ZNF266	19	9412598	9435578	protein_coding	13,27	0
ENSG00000131374	TBC1D5	3	17157162	18444817	protein_coding	13,27	0
ENSG00000107077	KDM4C	9	6757641	7175648	protein_coding	13,26	0
ENSG00000112200	ZNF451	6	57086844	57170307	protein_coding	13,26	0
ENSG00000184939	ZFP90	16	68530090	68576072	protein_coding	13,26	0
ENSG00000102393	GLA	X	101397803	101407925	protein_coding	13,25	0
ENSG00000136802	LRRC8A	9	128882112	128918039	protein_coding	13,24	0
ENSG00000100532	CGRRF1	14	54509812	54539309	protein_coding	13,23	0
ENSG00000143252	SDHC	1	161314257	161375340	protein_coding	13,22	0
ENSG00000128185	DGCR6L	22	20314276	20320080	protein_coding	13,22	0
ENSG00000115415	STAT1	2	190964358	191020960	protein_coding	13,22	0
ENSG00000077232	DNAJC10	2	182716041	182794464	protein_coding	13,21	0
ENSG00000111445	RFC5	12	118013588	118033130	protein_coding	13,21	0
ENSG00000111181	SLC6A12	12	190077	214570	protein_coding	13,20	0
ENSG00000110169	HPX	11	6431049	6442617	protein_coding	13,20	0
ENSG00000110665	C11orf21	11	2295645	2303049	protein_coding	13,20	0
ENSG00000222047	C10orf55	10	73909969	73922777	protein_coding	13,20	0
ENSG00000244067	GSTA2	6	52750089	52763569	protein_coding	13,20	0
ENSG00000146054	TRIM7	5	181193924	181205293	protein_coding	13,20	0
ENSG00000280789	PAGR1	16	29815952	29820117	protein_coding	13,18	0
ENSG00000166263	STXBP4	17	54968727	55173632	protein_coding	13,17	0
ENSG00000150471	ADGRL3	4	61201258	62078335	protein_coding	13,16	0
ENSG00000162227	TAF6L	11	62771303	62787342	protein_coding	13,16	0
ENSG00000111328	CDK2AP1	12	123260971	123272334	protein_coding	13,16	0
ENSG00000186063	AIDA	1	222668013	222713210	protein_coding	13,15	0
ENSG00000133612	AGAP3	7	151085831	151144436	protein_coding	13,15	0
ENSG00000175221	MED16	19	867630	893218	protein_coding	13,15	0
ENSG00000105426	PTPRS	19	5158495	5340803	protein_coding	13,14	0
ENSG00000072786	STK10	5	172042073	172188386	protein_coding	13,12	0
ENSG00000101017	CD40	20	46118272	46129863	protein_coding	13,12	0
ENSG00000166507	NDST2	10	73801911	73811798	protein_coding	13,10	0
ENSG00000137500	CCDC90B	11	83259097	83286407	protein_coding	13,10	0
ENSG00000163093	BBS5	2	169479178	169506655	protein_coding	13,08	0
ENSG00000185800	DMWD	19	45782947	45792802	protein_coding	13,07	0
ENSG00000168090	COPS6	7	100088954	100092200	protein_coding	13,06	0
ENSG00000198732	SMOC1	14	69854131	70032366	protein_coding	13,06	0
ENSG00000112146	FBXO9	6	53051991	53100873	protein_coding	13,06	0
ENSG00000175787	ZNF169	9	94259311	94301454	protein_coding	13,06	0
ENSG00000110395	CBL	11	119206276	119308149	protein_coding	13,05	0
ENSG00000155755	TMEM237	2	201620184	201643570	protein_coding	13,04	0
ENSG00000170502	NUDT9	4	87422582	87459454	protein_coding	13,02	0
ENSG00000146463	ZMYM4	1	35268967	35422058	protein_coding	13,00	0
ENSG00000139572	GPR84	12	54362445	54364487	protein_coding	13,00	0
ENSG00000178199	ZC3H12D	6	149446795	149485061	protein_coding	13,00	0
ENSG00000213397	HAUS7	X	153447666	153495516	protein_coding	13,00	0
ENSG00000148737	TCF7L2	10	112950250	113167678	protein_coding	12,99	0
ENSG00000197375	SLC22A5	5	132369752	132395614	protein_coding	12,99	0
ENSG00000177192	PUS1	12	131929200	131945896	protein_coding	12,97	0
ENSG00000168003	SLC3A2	11	62856102	62888875	protein_coding	12,95	0
ENSG00000111300	NAA25	12	112026689	112109022	protein_coding	12,94	0
ENSG00000125779	PANK2	20	3888839	3929882	protein_coding	12,94	0
ENSG00000129353	SLC44A2	19	10602457	10644559	protein_coding	12,94	0
ENSG00000119965	C10orf88	10	122930903	122954403	protein_coding	12,94	0
ENSG00000162804	SNED1	2	240998838	241095568	protein_coding	12,93	0
ENSG00000105323	HNRNPUL1	19	41262496	41307598	protein_coding	12,92	0
ENSG00000104983	CCDC61	19	45995461	46021318	protein_coding	12,92	0
ENSG00000142444	C19orf52	19	10928733	10933535	protein_coding	12,91	0
ENSG00000138018	EPT1	2	26308547	26395891	protein_coding	12,90	0

ENSG00000145428	RNF175	4	153710125	153760235	protein_coding	12,90	0
ENSG00000178104	PDE4DIP	1	148808181	149048286	protein_coding	12,88	0
ENSG00000113732	ATP6V0E1	5	172983757	173035445	protein_coding	12,86	0
ENSG00000188021	UBQLN2	X	56563639	56567868	protein_coding	12,86	0
ENSG00000147535	PLPP5	8	38263130	38269243	protein_coding	12,85	0
ENSG00000134780	DAGLA	11	61680433	61747001	protein_coding	12,84	0
ENSG00000160410	SHKBP1	19	40576851	40591399	protein_coding	12,83	0
ENSG00000171206	TRIM8	10	102644496	102658407	protein_coding	12,83	0
ENSG00000100031	GGT1	22	24583750	24629005	protein_coding	12,82	0
ENSG00000172500	FIBP	11	65883741	65888539	protein_coding	12,82	0
ENSG00000170185	USP38	4	143184917	143223830	protein_coding	12,81	0
ENSG00000187024	PTRH1	9	127692978	127724873	protein_coding	12,80	0
ENSG00000090612	ZNF268	12	133181409	133214831	protein_coding	12,80	0
ENSG00000186523	FAM86B1	8	12182096	12194133	protein_coding	12,80	0
ENSG00000015676	NUDCD3	7	44379121	44490880	protein_coding	12,80	0
ENSG00000086589	RBM22	5	150690794	150701107	protein_coding	12,78	0
ENSG00000159214	CCDC24	1	43991359	43996528	protein_coding	12,78	0
ENSG00000196655	TRAPPC4	11	119018432	119025454	protein_coding	12,78	0
ENSG00000196776	CD47	3	108043298	108091025	protein_coding	12,77	0
ENSG00000125775	SDCBP2	20	1309909	1329239	protein_coding	12,76	0
ENSG00000110429	FBXO3	11	33740939	33774543	protein_coding	12,76	0
ENSG00000116478	HDAC1	1	32292086	32333635	protein_coding	12,76	0
ENSG00000183978	COA3	17	42795147	42798704	protein_coding	12,74	0
ENSG00000187144	SPATA21	1	16387117	16437424	protein_coding	12,72	0
ENSG00000141367	CLTC	17	59619689	59696956	protein_coding	12,71	0
ENSG00000104903	LYL1	19	13099033	13103161	protein_coding	12,70	0
ENSG00000168356	SCN11A	3	38845769	38950561	protein_coding	12,70	0
ENSG00000148290	SURF1	9	133351755	133356676	protein_coding	12,70	0
ENSG00000148541	FAM13C	10	59246129	59363181	protein_coding	12,69	0
ENSG00000183864	TOB2	22	41433492	41447023	protein_coding	12,69	0
ENSG00000140350	ANP32A	15	68778535	68820897	protein_coding	12,68	0
ENSG00000100767	PAPLN	14	73237497	73274640	protein_coding	12,68	0
ENSG00000206052	DOK6	18	69401055	69849087	protein_coding	12,67	0
ENSG00000186193	SAPCD2	9	137062124	137070588	protein_coding	12,67	0
ENSG00000137265	IRF4	6	391739	411447	protein_coding	12,67	0
ENSG00000167100	SAMD14	17	50110040	50129882	protein_coding	12,66	0
ENSG00000146232	NFKBIE	6	44258166	44265788	protein_coding	12,65	0
ENSG00000115053	NCL	2	231453531	231483641	protein_coding	12,65	0
ENSG00000167257	RNF214	11	117232625	117286445	protein_coding	12,64	0
ENSG00000197859	ADAMTSL2	9	133532164	133575519	protein_coding	12,64	0
ENSG00000196236	XPNPEP3	22	40857077	40932815	protein_coding	12,64	0
ENSG00000164830	OXR1	8	106270178	106752694	protein_coding	12,63	0
ENSG00000127445	PIN1	19	9835257	9849682	protein_coding	12,63	0
ENSG00000130165	ELOF1	19	11551147	11559236	protein_coding	12,62	0
ENSG00000172531	PPP1CA	11	67398183	67421183	protein_coding	12,61	0
ENSG00000125877	ITPA	20	3208868	3223870	protein_coding	12,60	0
ENSG00000105063	PPP6R1	19	55229780	55258995	protein_coding	12,60	0
ENSG00000161405	IKZF3	17	39757715	39864188	protein_coding	12,60	0
ENSG00000103043	VAC14	16	70687439	70801161	protein_coding	12,60	0
ENSG00000043462	LCP2	5	170246237	170298227	protein_coding	12,59	0
ENSG00000138434	SSFA2	2	181891833	181930738	protein_coding	12,58	0
ENSG00000174238	PITPNA	17	1517718	1562816	protein_coding	12,58	0
ENSG00000172346	CSDC2	22	41560763	41577741	protein_coding	12,58	0
ENSG00000116141	MARK1	1	220528183	220664461	protein_coding	12,58	0
ENSG00000104960	PTOV1	19	49850735	49860744	protein_coding	12,57	0
ENSG00000171345	KRT19	17	41523617	41528308	protein_coding	12,57	0
ENSG00000088826	SMOX	20	4120980	4187747	protein_coding	12,56	0
ENSG00000145113	MUC4	3	195746765	195812277	protein_coding	12,56	0

ENSG00000159239	C2orf81	2	74414176	74421591	protein_coding	12,55	0
ENSG00000135749	PCNXL2	1	232983435	233295713	protein_coding	12,54	0
ENSG00000197619	ZNF615	19	51991332	52008230	protein_coding	12,53	0
ENSG00000141378	PTRH2	17	59674636	59707626	protein_coding	12,52	0
ENSG00000170638	TRABD	22	50185915	50199598	protein_coding	12,51	0
ENSG00000182584	ACTL10	20	33666498	33668525	protein_coding	12,50	0
ENSG00000161682	FAM171A2	17	44353215	44363875	protein_coding	12,50	0
ENSG00000039537	C6	5	41142234	41261438	protein_coding	12,50	0
ENSG00000163606	CD200R1	3	112921209	112975122	protein_coding	12,50	0
ENSG00000181036	FCRL6	1	159800511	159816251	protein_coding	12,50	0
ENSG00000076242	MLH1	3	36993332	37050918	protein_coding	12,50	0
ENSG00000162337	LRP5	11	68312609	68449275	protein_coding	12,50	0
ENSG00000120549	KIAA1217	10	23694746	24547848	protein_coding	12,49	0
ENSG00000275993	ENSG00000275993	21	6111134	6123739	protein_coding	12,49	0
ENSG00000167721	TSR1	17	2322503	2337507	protein_coding	12,48	0
ENSG00000115446	UNC50	2	98608579	98618515	protein_coding	12,47	0
ENSG00000134440	NARS	18	57600656	57622213	protein_coding	12,46	0
ENSG00000144840	RABL3	3	120686681	120742993	protein_coding	12,46	0
ENSG00000196605	ZNF846	19	9751993	9793180	protein_coding	12,46	0
ENSG00000067596	DHX8	17	43483865	43544463	protein_coding	12,46	0
ENSG00000047578	KIAA0556	16	27550133	27780369	protein_coding	12,45	0
ENSG00000136425	CIB2	15	78104606	78131544	protein_coding	12,44	0
ENSG00000159086	PAXBP1	21	32733899	32771858	protein_coding	12,43	0
ENSG00000100485	SOS2	14	50117120	50231558	protein_coding	12,42	0
ENSG00000007047	MARK4	19	45079288	45305283	protein_coding	12,42	0
ENSG00000169136	ATF5	19	49928702	49933935	protein_coding	12,42	0
ENSG00000204619	PPP1R11	6	30066709	30070333	protein_coding	12,41	0
ENSG00000103852	TTC23	15	99136323	99251223	protein_coding	12,41	0
ENSG00000149634	SPATA25	20	45886489	45887635	protein_coding	12,40	0
ENSG00000167759	KLK13	19	51056206	51065114	protein_coding	12,40	0
ENSG00000250120	PCDHA10	5	140855883	141012344	protein_coding	12,40	0
ENSG00000081051	AFP	4	73431138	73456174	protein_coding	12,40	0
ENSG00000072694	FCGR2B	1	161663147	161678654	protein_coding	12,40	0
ENSG00000173714	WFIKKN2	17	50834650	50842348	protein_coding	12,40	0
ENSG00000141519	CCDC40	17	80036632	80100613	protein_coding	12,39	0
ENSG00000163682	RPL9	4	39454124	39458948	protein_coding	12,39	0
ENSG00000101126	ADNP	20	50888919	50931240	protein_coding	12,39	0
ENSG00000171155	C1GALT1C1	X	120625793	120630150	protein_coding	12,38	0
ENSG00000188283	ZNF383	19	37217926	37248738	protein_coding	12,38	0
ENSG00000179979	CRIPAK	4	1391552	1395989	protein_coding	12,37	0
ENSG00000167723	TRPV3	17	3510502	3557995	protein_coding	12,37	0
ENSG00000157992	KRTCAP3	2	27442366	27446481	protein_coding	12,37	0
ENSG00000124733	MEA1	6	43012094	43013968	protein_coding	12,36	0
ENSG00000160688	FLAD1	1	154983338	154993111	protein_coding	12,34	0
ENSG00000188820	FAM26F	6	116461370	116463779	protein_coding	12,33	0
ENSG00000115486	GGCX	2	85544723	85561547	protein_coding	12,33	0
ENSG00000168743	NPNT	4	105894775	106004027	protein_coding	12,33	0
ENSG00000156304	SCAF4	21	31671033	31732075	protein_coding	12,32	0
ENSG00000176148	TCP11L1	11	33039417	33105943	protein_coding	12,31	0
ENSG00000253309	SERPINE3	13	51335773	51364735	protein_coding	12,31	0
ENSG00000044574	HSPA5	9	125234853	125241330	protein_coding	12,30	0
ENSG00000124743	KLHL31	6	53647901	53665708	protein_coding	12,30	0
ENSG00000134326	CMPK2	2	6840570	6866635	protein_coding	12,30	0
ENSG00000271092	TMEM56-RV	1	95117923	95247225	protein_coding	12,30	0
ENSG00000125675	GRIA3	X	123184153	123490915	protein_coding	12,30	0
ENSG00000111215	PRR4	12	10845849	11171613	protein_coding	12,29	0
ENSG00000106853	PTGR1	9	111549722	111599855	protein_coding	12,29	0
ENSG00000135778	NTPCR	1	232950605	232983882	protein_coding	12,28	0

ENSG00000108861	DUSP3	17	43766121	43778988	protein_coding	12,27	0
ENSG00000097021	ACOT7	1	6264269	6394391	protein_coding	12,27	0
ENSG00000042429	MED17	11	93784227	93814695	protein_coding	12,27	0
ENSG00000167861	HID1	17	74950743	74973166	protein_coding	12,26	0
ENSG00000145780	FEM1C	5	115520908	115544894	protein_coding	12,26	0
ENSG00000067208	EVI5	1	92508696	92792404	protein_coding	12,24	0
ENSG00000149792	MRPL49	11	65122183	65127371	protein_coding	12,24	0
ENSG00000114670	NEK11	3	131026850	131350465	protein_coding	12,23	0
ENSG00000112851	ERBB2IP	5	65926475	66082549	protein_coding	12,22	0
ENSG00000256087	ZNF432	19	52031379	52095738	protein_coding	12,21	0
ENSG00000166337	TAF10	11	6606296	6612667	protein_coding	12,21	0
ENSG00000165837	ERICH6B	13	45534522	45615739	protein_coding	12,20	0
ENSG00000185305	ARL15	5	53883945	54310582	protein_coding	12,20	0
ENSG00000133488	SEC14L4	22	30488913	30505711	protein_coding	12,20	0
ENSG00000121552	CSTA	3	122325244	122341972	protein_coding	12,20	0
ENSG00000169951	ZNF764	16	30553764	30558498	protein_coding	12,19	0
ENSG00000138757	G3BP2	4	75642782	75724525	protein_coding	12,19	0
ENSG00000180979	LRRC57	15	42537820	42548802	protein_coding	12,19	0
ENSG00000155868	MED7	5	157137412	157159019	protein_coding	12,18	0
ENSG00000130477	UNC13A	19	17601328	17688365	protein_coding	12,17	0
ENSG00000182287	AP1S2	X	15825806	15855014	protein_coding	12,17	0
ENSG00000132912	DCTN4	5	150708440	150759109	protein_coding	12,16	0
ENSG00000164024	METAP1	4	98995620	99062813	protein_coding	12,16	0
ENSG00000198466	ZNF587	19	57849857	57865112	protein_coding	12,15	0
ENSG00000226479	TMEM185B	2	120221278	120223408	protein_coding	12,12	0
ENSG00000083520	DIS3	13	72752169	72782096	protein_coding	12,12	0
ENSG00000136144	RCBTB1	13	49531946	49585583	protein_coding	12,11	0
ENSG00000073921	PICALM	11	85957684	86069882	protein_coding	12,11	0
ENSG00000166869	CHP2	16	23754627	23758951	protein_coding	12,10	0
ENSG00000123643	SLC36A1	5	151437046	151492381	protein_coding	12,09	0
ENSG00000117305	HMGCL	1	23801885	23838620	protein_coding	12,09	0
ENSG00000156398	SFXN2	10	102714538	102743492	protein_coding	12,08	0
ENSG00000107372	ZFAND5	9	72351425	72365235	protein_coding	12,08	0
ENSG00000106246	PTCD1	7	99416739	99466163	protein_coding	12,07	0
ENSG00000204371	EHMT2	6	31879759	31897687	protein_coding	12,07	0
ENSG00000107815	C10orf2	10	100987367	100994401	protein_coding	12,06	0
ENSG00000157107	FCHO2	5	72955981	73090522	protein_coding	12,06	0
ENSG00000039319	ZFYVE16	5	80408013	80479350	protein_coding	12,05	0
ENSG00000157181	C1orf27	1	186375838	186421378	protein_coding	12,04	0
ENSG00000153487	ING1	13	110712736	110723339	protein_coding	12,02	0
ENSG00000013523	ANGEL1	14	76786178	76826246	protein_coding	12,01	0
ENSG00000172115	CYCS	7	25120091	25125361	protein_coding	12,00	0
ENSG00000268182	SMIM17	19	56643145	56655766	protein_coding	12,00	0
ENSG00000059377	TBXAS1	7	139777051	140020325	protein_coding	12,00	0
ENSG00000015171	ZMYND11	10	134465	254637	protein_coding	12,00	0
ENSG00000162520	SYNC	1	32680360	32703596	protein_coding	11,99	0
ENSG00000167645	YIF1B	19	38305104	38317273	protein_coding	11,99	0
ENSG00000154832	CXXC1	18	50282343	50288304	protein_coding	11,98	0
ENSG00000136235	GPNMB	7	23235967	23275108	protein_coding	11,98	0
ENSG00000117115	PADI2	1	17066761	17119435	protein_coding	11,97	0
ENSG00000087191	PSMC5	17	63827152	63832026	protein_coding	11,96	0
ENSG00000116251	RPL22	1	6181269	6209389	protein_coding	11,96	0
ENSG00000135723	FHOD1	16	67229387	67247658	protein_coding	11,96	0
ENSG00000163320	CGGBP1	3	88051944	88149885	protein_coding	11,95	0
ENSG00000106144	CASP2	7	143288215	143307696	protein_coding	11,95	0
ENSG00000140521	POLG	15	89316305	89334861	protein_coding	11,95	0
ENSG00000155961	RAB39B	X	155258241	155264589	protein_coding	11,94	0
ENSG00000117448	AKR1A1	1	45550543	45570049	protein_coding	11,94	0

ENSG00000119862	LGALSL	2	64453969	64461381	protein_coding	11,93	0
ENSG00000075643	MOCOS	18	36187519	36272157	protein_coding	11,93	0
ENSG00000166140	ZFYVE19	15	40807086	40815084	protein_coding	11,93	0
ENSG00000276644	DACH1	13	71437966	71867192	protein_coding	11,93	0
ENSG00000104679	R3HCC1	8	23270120	23296279	protein_coding	11,93	0
ENSG00000125731	SH2D3A	19	6752160	6767588	protein_coding	11,92	0
ENSG00000204519	ZNF551	19	57681969	57717301	protein_coding	11,92	0
ENSG00000169432	SCN9A	2	166195185	166375993	protein_coding	11,91	0
ENSG00000116560	SFPQ	1	35176378	35193148	protein_coding	11,91	0
ENSG00000170382	LRRN2	1	204617170	204685733	protein_coding	11,90	0
ENSG00000004777	ARHGAP33	19	35774532	35788822	protein_coding	11,90	0
ENSG00000117533	VAMP4	1	171700160	171742247	protein_coding	11,88	0
ENSG00000159915	ZNF233	19	44259880	44275317	protein_coding	11,88	0
ENSG00000164040	PGRMC2	4	128269237	128288829	protein_coding	11,87	0
ENSG00000110172	CHORDC1	11	90201160	90223364	protein_coding	11,86	0
ENSG00000137198	GMPR	6	16238580	16295549	protein_coding	11,85	0
ENSG00000109919	MTCH2	11	47617315	47642623	protein_coding	11,84	0
ENSG00000144668	ITGA9	3	37452115	37823514	protein_coding	11,84	0
ENSG00000149823	VPS51	11	65089324	65111860	protein_coding	11,84	0
ENSG00000175536	LIPT2	11	74491712	74493733	protein_coding	11,81	0
ENSG00000213809	KLRK1	12	10372353	10391874	protein_coding	11,80	0
ENSG00000143954	REG3G	2	79025686	79028505	protein_coding	11,80	0
ENSG00000116882	HAO2	1	119368779	119394130	protein_coding	11,80	0
ENSG00000162433	AK4	1	65147549	65232145	protein_coding	11,80	0
ENSG00000091129	NRCAM	7	108147623	108456717	protein_coding	11,79	0
ENSG00000187045	TMPRSS6	22	37065436	37109563	protein_coding	11,73	0
ENSG00000170248	PDCD6IP	3	33798352	33869707	protein_coding	11,71	0
ENSG00000155621	C9orf85	9	71911510	71986054	protein_coding	11,71	0
ENSG00000160285	LSS	21	46188141	46228824	protein_coding	11,70	0
ENSG00000204923	FBXO48	2	68459419	68467258	protein_coding	11,69	0
ENSG00000141503	MINK1	17	4833388	4898061	protein_coding	11,69	0
ENSG00000147874	HAUS6	9	19053143	19103119	protein_coding	11,68	0
ENSG00000110955	ATP5B	12	56638175	56646068	protein_coding	11,68	0
ENSG00000108582	CPD	17	30378905	30469989	protein_coding	11,68	0
ENSG00000108839	ALOX12	17	6996065	7010736	protein_coding	11,67	0
ENSG00000176697	BDNF	11	27654893	27722058	protein_coding	11,66	0
ENSG00000198171	DDRGK1	20	3190350	3204685	protein_coding	11,66	0
ENSG00000146433	TMEM181	6	158536436	158635428	protein_coding	11,66	0
ENSG00000124459	ZNF45	19	43912629	43935278	protein_coding	11,65	0
ENSG00000144724	PTPRG	3	61561569	62297613	protein_coding	11,64	0
ENSG00000104951	IL4I1	19	49889654	49929539	protein_coding	11,64	0
ENSG00000159267	HLCS	21	36750888	36990236	protein_coding	11,64	0
ENSG00000076356	PLXNA2	1	208022242	208244320	protein_coding	11,64	0
ENSG00000198961	PJA2	5	109334709	109409994	protein_coding	11,63	0
ENSG00000196659	TTC30B	2	177548998	177553014	protein_coding	11,63	0
ENSG00000159202	UBE2Z	17	48908369	48929056	protein_coding	11,63	0
ENSG00000131873	CHSY1	15	101175723	101251932	protein_coding	11,63	0
ENSG00000095539	SEMA4G	10	100969518	100985871	protein_coding	11,63	0
ENSG00000164296	TIGD6	5	149993118	150001167	protein_coding	11,62	0
ENSG00000186111	PIP5K1C	19	3630183	3700479	protein_coding	11,61	0
ENSG00000121207	LRAT	4	154626945	154753118	protein_coding	11,61	0
ENSG00000143674	ENSG00000[illegible]	1	233327768	233385148	protein_coding	11,60	0
ENSG00000182853	VMO1	17	4785285	4786433	protein_coding	11,60	0
ENSG00000253485	PCDHGA5	5	141364232	141512979	protein_coding	11,60	0
ENSG00000159884	CCDC107	9	35658290	35661511	protein_coding	11,59	0
ENSG00000168275	COA6	1	234373456	234384049	protein_coding	11,58	0
ENSG00000141252	VPS53	17	508668	721717	protein_coding	11,58	0
ENSG00000198860	TSEN15	1	184051677	184074212	protein_coding	11,58	0

ENSG00000185187	SIGIRR	11	405716	417455	protein_coding	11,58	0
ENSG00000108509	CAMTA2	17	4967992	4987652	protein_coding	11,58	0
ENSG00000115946	PNO1	2	68157844	68176238	protein_coding	11,57	0
ENSG00000150967	ABCB9	12	122920951	122981649	protein_coding	11,56	0
ENSG00000256294	ZNF225	19	44112181	44134816	protein_coding	11,56	0
ENSG00000198517	MAFK	7	1530714	1543043	protein_coding	11,56	0
ENSG00000143379	SETDB1	1	150926263	150964744	protein_coding	11,55	0
ENSG00000141564	RPTOR	17	80544819	80966371	protein_coding	11,55	0
ENSG00000100196	KDELR3	22	38468062	38483447	protein_coding	11,55	0
ENSG00000198911	SREBF2	22	41833079	41907308	protein_coding	11,55	0
ENSG00000204310	AGPAT1	6	32168212	32178096	protein_coding	11,54	0
ENSG00000128739	SNRPN	15	24823637	24978723	protein_coding	11,53	0
ENSG00000168118	RAB4A	1	229271062	229305894	protein_coding	11,53	0
ENSG00000152700	SAR1B	5	134601144	134649271	protein_coding	11,52	0
ENSG00000254893	ENSG000002	5	76170093	76174331	protein_coding	11,51	0
ENSG00000237190	CDKN2AIPNI	5	134402087	134411898	protein_coding	11,49	0
ENSG00000103591	AAGAB	15	67201033	67255195	protein_coding	11,49	0
ENSG00000115649	CNPPD1	2	219171897	219178106	protein_coding	11,49	0
ENSG00000109756	RAPGEF2	4	159104178	159360169	protein_coding	11,48	0
ENSG00000163867	ZMYM6	1	34986165	35031968	protein_coding	11,48	0
ENSG00000204843	DCTN1	2	74361154	74392087	protein_coding	11,47	0
ENSG00000109475	RPL34	4	108620566	108630412	protein_coding	11,46	0
ENSG00000241973	PI4KA	22	20707691	20859417	protein_coding	11,46	0
ENSG00000271605	MILR1	17	64449037	64468643	protein_coding	11,45	0
ENSG00000166200	COPS2	15	49106068	49155661	protein_coding	11,45	0
ENSG00000100056	DGCR14	22	19130279	19144684	protein_coding	11,45	0
ENSG00000049541	RFC2	7	74231499	74254458	protein_coding	11,45	0
ENSG00000132677	RHBG	1	156369212	156385219	protein_coding	11,44	0
ENSG00000105568	PPP2R1A	19	52190039	52229533	protein_coding	11,43	0
ENSG00000111644	ACRBP	12	6638075	6647460	protein_coding	11,43	0
ENSG00000165698	C9orf9	9	132878027	132890201	protein_coding	11,42	0
ENSG00000167674	ENSG000001	19	4472287	4502211	protein_coding	11,41	0
ENSG00000198743	SLC5A3	21	34073570	34106260	protein_coding	11,41	0
ENSG00000082196	C1QTNF3	5	34019448	34043832	protein_coding	11,40	0
ENSG00000126005	MMP24-AS1	20	35201745	35278131	protein_coding	11,40	0
ENSG00000103064	SLC7A6	16	68264516	68301823	protein_coding	11,38	0
ENSG00000149260	CAPN5	11	77066932	77126155	protein_coding	11,38	0
ENSG00000148331	ASB6	9	129634604	129642169	protein_coding	11,37	0
ENSG00000173960	UBXN2A	2	23927285	24004909	protein_coding	11,36	0
ENSG00000134046	MBD2	18	54151601	54224788	protein_coding	11,36	0
ENSG00000133597	ADCK2	7	140673153	140696261	protein_coding	11,36	0
ENSG00000105889	STEAP1B	7	22419444	22632925	protein_coding	11,36	0
ENSG00000116459	ATP5F1	1	111448864	111462773	protein_coding	11,36	0
ENSG00000165819	METTL3	14	21498133	21511375	protein_coding	11,35	0
ENSG00000100209	HSCB	22	28742031	28757515	protein_coding	11,35	0
ENSG00000065978	YBX1	1	42682427	42702349	protein_coding	11,34	0
ENSG00000115661	STK16	2	219245455	219250337	protein_coding	11,34	0
ENSG00000132003	ZSWIM4	19	13795460	13832230	protein_coding	11,34	0
ENSG00000105808	RASA4	7	102573807	102616757	protein_coding	11,33	0
ENSG00000008018	PSMB1	6	170535117	170553341	protein_coding	11,33	0
ENSG00000065518	NDUFB4	3	120596309	120602500	protein_coding	11,32	0
ENSG00000178665	ZNF713	7	55887475	55942225	protein_coding	11,32	0
ENSG00000143569	UBAP2L	1	154220179	154271510	protein_coding	11,32	0
ENSG00000076662	ICAM3	19	10333776	10339823	protein_coding	11,30	0
ENSG00000160888	IER2	19	13150415	13154908	protein_coding	11,30	0
ENSG00000234409	CCDC188	22	20148427	20151065	protein_coding	11,30	0
ENSG00000197191	CYSRT1	9	137224635	137226311	protein_coding	11,30	0
ENSG00000243896	OR2A7	7	144258607	144259722	protein_coding	11,30	0

ENSG00000113212	PCDHB7	5	141172619	141176383	protein_coding	11,30	0
ENSG00000212122	TSSK1B	5	113432554	113435031	protein_coding	11,30	0
ENSG00000109320	NFKB1	4	102501329	102617302	protein_coding	11,30	0
ENSG00000125107	CNOT1	16	58519951	58629886	protein_coding	11,29	0
ENSG00000093167	LRRFIP2	3	37052656	37183689	protein_coding	11,24	0
ENSG00000162736	NCSTN	1	160343272	160358952	protein_coding	11,24	0
ENSG00000178163	ZNF518B	4	10439874	10457410	protein_coding	11,24	0
ENSG00000115282	TTC31	2	74483073	74494886	protein_coding	11,23	0
ENSG00000125247	TMTC4	13	100603927	100675093	protein_coding	11,23	0
ENSG00000180530	NRIP1	21	14961235	15065000	protein_coding	11,23	0
ENSG00000076944	STXBP2	19	7636881	7647873	protein_coding	11,22	0
ENSG00000176055	MBLAC2	5	90458203	90474768	protein_coding	11,22	0
ENSG00000135835	KIAA1614	1	180913154	180951614	protein_coding	11,22	0
ENSG00000134461	ANKRD16	10	5861617	5889906	protein_coding	11,22	0
ENSG00000149262	INTS4	11	77878720	77994678	protein_coding	11,21	0
ENSG00000157315	TMED6	16	69343248	69351809	protein_coding	11,20	0
ENSG00000183484	GPR132	14	105049389	105065445	protein_coding	11,20	0
ENSG00000123500	COL10A1	6	116118923	116158747	protein_coding	11,20	0
ENSG00000197506	SLC28A3	9	84275457	84340683	protein_coding	11,20	0
ENSG00000072803	FBXW11	5	171861549	172006873	protein_coding	11,19	0
ENSG00000166839	ANKDD1A	15	64911902	64958700	protein_coding	11,18	0
ENSG00000162971	TYW5	2	199929975	199955736	protein_coding	11,17	0
ENSG00000108406	DHX40	17	59565525	59608345	protein_coding	11,16	0
ENSG00000138639	ARHGAP24	4	85475114	86002670	protein_coding	11,16	0
ENSG00000206562	METTL6	3	15381275	15440566	protein_coding	11,15	0
ENSG00000185864	NPIPB4	16	21834569	21880827	protein_coding	11,15	0
ENSG00000108344	PSMD3	17	39980797	39997960	protein_coding	11,15	0
ENSG00000103653	CSK	15	74782057	74803198	protein_coding	11,15	0
ENSG00000155545	MIER3	5	56919602	56971675	protein_coding	11,15	0
ENSG00000134001	EIF2S1	14	67359997	67386516	protein_coding	11,14	0
ENSG00000175582	RAB6A	11	73675638	73761137	protein_coding	11,14	0
ENSG00000175756	AURKAIP1	1	1373730	1375495	protein_coding	11,14	0
ENSG00000072364	AFF4	5	132875379	132963634	protein_coding	11,13	0
ENSG00000168495	POLR3D	8	22245104	22254600	protein_coding	11,13	0
ENSG00000238227	C9orf69	9	136114581	136118863	protein_coding	11,12	0
ENSG00000036257	CUL3	2	224470150	224585397	protein_coding	11,12	0
ENSG00000146828	SLC12A9	7	100826820	100867009	protein_coding	11,12	0
ENSG00000092201	SUPT16H	14	21351472	21384266	protein_coding	11,11	0
ENSG00000138085	ATRAID	2	27212027	27217178	protein_coding	11,11	0
ENSG00000134809	TIMM10	11	57528463	57530803	protein_coding	11,11	0
ENSG00000206344	HCG27	6	31197760	31203968	protein_coding	11,10	0
ENSG00000169855	ROBO1	3	78597240	79767815	protein_coding	11,09	0
ENSG00000136100	VPS36	13	52412602	52450628	protein_coding	11,09	0
ENSG00000213967	ZNF726	19	23914876	23945159	protein_coding	11,08	0
ENSG00000163827	LRRC2	3	46515423	46580099	protein_coding	11,08	0
ENSG00000113594	LIFR	5	38474963	38608354	protein_coding	11,08	0
ENSG00000100065	CARD10	22	37490362	37519542	protein_coding	11,08	0
ENSG00000115525	ST3GAL5	2	85839144	85889014	protein_coding	11,07	0
ENSG00000152147	GEMIN6	2	38751534	38785000	protein_coding	11,07	0
ENSG00000058668	ATP2B4	1	203626561	203744081	protein_coding	11,06	0
ENSG00000122565	CBX3	7	26201162	26213356	protein_coding	11,06	0
ENSG00000140839	CLEC18B	16	74408270	74421953	protein_coding	11,05	0
ENSG00000105516	DBP	19	48630030	48637438	protein_coding	11,05	0
ENSG00000062822	POLD1	19	50384204	50418018	protein_coding	11,04	0
ENSG00000010219	DYRK4	12	4562204	4615302	protein_coding	11,04	0
ENSG00000147408	CSGALNACT	8	19404161	19758029	protein_coding	11,04	0
ENSG00000130813	C19orf66	19	10086122	10093252	protein_coding	11,02	0
ENSG00000247626	03/01/2002	2	197705369	197708387	protein_coding	11,02	0

ENSG00000162522	KIAA1522	1	32741885	32774970	protein_coding	11,00	0
ENSG00000123146	ADGRE5	19	14380501	14408725	protein_coding	11,00	0
ENSG00000160229	ZNF66	19	20776304	20807322	protein_coding	11,00	0
ENSG00000119725	ZNF410	14	73886617	73932511	protein_coding	11,00	0
ENSG00000232810	TNF	6	31575567	31578336	protein_coding	11,00	0
ENSG00000160716	CHRNB2	1	154567781	154580026	protein_coding	11,00	0
ENSG00000196338	NLGN3	X	71144831	71171201	protein_coding	11,00	0
ENSG00000155100	OTUD6B	8	91070196	91087095	protein_coding	10,99	0
ENSG00000185761	ADAMTSL5	19	1505018	1513604	protein_coding	10,99	0
ENSG00000136527	TRA2B	3	185915906	185938136	protein_coding	10,99	0
ENSG00000147400	CETN2	X	152826973	152830777	protein_coding	10,98	0
ENSG00000164077	MON1A	3	49908862	49930173	protein_coding	10,98	0
ENSG00000106245	BUD31	7	99408641	99419616	protein_coding	10,98	0
ENSG00000198225	FKBP1C	6	63211446	63213024	protein_coding	10,98	0
ENSG00000068697	LAPTM4A	2	20032650	20052028	protein_coding	10,97	0
ENSG00000168282	MGAT2	14	49620795	49623481	protein_coding	10,96	0
ENSG00000112237	CCNC	6	99542380	99568973	protein_coding	10,96	0
ENSG00000180104	EXOC3	5	443158	471937	protein_coding	10,94	0
ENSG00000007341	ST7L	1	112523518	112620825	protein_coding	10,94	0
ENSG00000101654	RNMT	18	13726660	13764558	protein_coding	10,94	0
ENSG00000131748	STARD3	17	39637065	39663484	protein_coding	10,93	0
ENSG00000089094	KDM2B	12	121429097	121581015	protein_coding	10,92	0
ENSG00000168807	SNTB2	16	69187129	69309052	protein_coding	10,92	0
ENSG00000168061	SAC3D1	11	65040901	65044828	protein_coding	10,91	0
ENSG00000169071	ROR2	9	91563091	91950162	protein_coding	10,91	0
ENSG00000153208	MERTK	2	111898479	112029561	protein_coding	10,91	0
ENSG00000182257	PRR34	22	46049478	46054144	protein_coding	10,90	0
ENSG00000064201	TSPAN32	11	2301997	2318200	protein_coding	10,90	0
ENSG00000113924	HGD	3	120628173	120682571	protein_coding	10,90	0
ENSG00000196455	PIK3R4	3	130678935	130746829	protein_coding	10,89	0
ENSG00000130640	TUBGCP2	10	133278630	133312337	protein_coding	10,88	0
ENSG00000024526	DEPDC1	1	68474152	68497221	protein_coding	10,88	0
ENSG00000140471	LINS1	15	100559369	100603230	protein_coding	10,88	0
ENSG00000113812	ACTR8	3	53867066	53882202	protein_coding	10,87	0
ENSG00000144228	SPOPL	2	138501801	138573547	protein_coding	10,86	0
ENSG00000167130	DOLPP1	9	129081100	129090438	protein_coding	10,86	0
ENSG00000038358	EDC4	16	67873023	67884503	protein_coding	10,86	0
ENSG00000107186	MPDZ	9	13105704	13279590	protein_coding	10,85	0
ENSG00000279644	ENSG00000	2	64552771	64592133	protein_coding	10,85	0
ENSG00000151067	CACNA1C	12	1970786	2697950	protein_coding	10,85	0
ENSG00000166106	ADAMTS15	11	130448974	130476641	protein_coding	10,84	0
ENSG00000152332	UHMK1	1	162497251	162529629	protein_coding	10,84	0
ENSG00000124788	ATXN1	6	16299112	16761491	protein_coding	10,82	0
ENSG00000181026	AEN	15	88621296	88632282	protein_coding	10,81	0
ENSG00000182405	PGBD4	15	34102073	34108684	protein_coding	10,81	0
ENSG00000164466	SFXN1	5	175477062	175529742	protein_coding	10,81	0
ENSG00000147364	FBXO25	8	406428	477967	protein_coding	10,80	0
ENSG00000185896	LAMP1	13	113297241	113323672	protein_coding	10,80	0
ENSG00000168398	BDKRB2	14	96204679	96244166	protein_coding	10,80	0
ENSG00000111319	SCNN1A	12	6346843	6377730	protein_coding	10,80	0
ENSG00000155893	PXYLP1	3	141228726	141294906	protein_coding	10,80	0
ENSG00000047849	MAP4	3	47850690	48089272	protein_coding	10,80	0
ENSG00000171988	JMJD1C	10	63167221	63521850	protein_coding	10,78	0
ENSG00000100150	DEPDC5	22	31753951	31907034	protein_coding	10,78	0
ENSG00000164091	WDR82	3	52254421	52288020	protein_coding	10,77	0
ENSG00000205423	CNEP1R1	16	50024410	50037088	protein_coding	10,76	0
ENSG00000179750	APOBEC3B	22	38982347	38992804	protein_coding	10,75	0
ENSG00000012983	MAP4K5	14	50418501	50561126	protein_coding	10,75	0

ENSG00000164576	SAP30L	5	154445957	154461054	protein_coding	10,74	0
ENSG00000165091	TMC1	9	72521801	72836351	protein_coding	10,73	0
ENSG00000086062	B4GALT1	9	33104082	33167356	protein_coding	10,73	0
ENSG00000144028	SNRNP200	2	96274336	96305515	protein_coding	10,73	0
ENSG00000104883	PEX11G	19	7476875	7497449	protein_coding	10,73	0
ENSG00000112514	CUTA	6	33416442	33418317	protein_coding	10,72	0
ENSG00000111266	DUSP16	12	12474210	12562383	protein_coding	10,72	0
ENSG00000006712	PAF1	19	39385852	39391195	protein_coding	10,71	0
ENSG00000126950	TMEM35	X	101078720	101096364	protein_coding	10,71	0
ENSG00000131381	RBSN	3	15070073	15099163	protein_coding	10,71	0
ENSG00000139842	CUL4A	13	113208193	113267108	protein_coding	10,70	0
ENSG00000177733	HNRNPA0	5	137745651	137754376	protein_coding	10,70	0
ENSG00000176919	C8G	9	136945246	136946974	protein_coding	10,70	0
ENSG00000157483	MYO1E	15	59132434	59372900	protein_coding	10,69	0
ENSG00000258388	PPT2-EGFL8	6	32153845	32171978	protein_coding	10,69	0
ENSG00000108518	PFN1	17	4945652	4949061	protein_coding	10,68	0
ENSG00000176915	ANKLE2	12	132725503	132761888	protein_coding	10,68	0
ENSG00000142892	PIGK	1	77088990	77219430	protein_coding	10,68	0
ENSG00000170260	ZNF212	7	149239651	149255609	protein_coding	10,65	0
ENSG00000103710	RASL12	15	65053337	65076690	protein_coding	10,65	0
ENSG00000100294	MCAT	22	43132206	43143394	protein_coding	10,65	0
ENSG00000139890	REM2	14	22883165	22887686	protein_coding	10,64	0
ENSG00000166946	CCNDBP1	15	43185118	43197176	protein_coding	10,63	0
ENSG00000121022	COPS5	8	67043079	67083783	protein_coding	10,63	0
ENSG00000150787	PTS	11	112226365	112269955	protein_coding	10,62	0
ENSG00000155096	AZIN1	8	102826357	102893864	protein_coding	10,62	0
ENSG00000141441	GAREM	18	32124877	32470484	protein_coding	10,61	0
ENSG00000119383	PPP2R4	9	129110950	129148946	protein_coding	10,60	0
ENSG00000164241	C5orf63	5	127042558	127073492	protein_coding	10,60	0
ENSG00000167850	CD300C	17	74541108	74546143	protein_coding	10,60	0
ENSG00000137571	SLCO5A1	8	69667047	69835064	protein_coding	10,60	0
ENSG00000147434	CHRNA6	8	42752620	42796392	protein_coding	10,60	0
ENSG00000179639	FCER1A	1	159289714	159308224	protein_coding	10,60	0
ENSG00000189306	RRP7A	22	42509968	42519802	protein_coding	10,60	0
ENSG00000134324	LPIN1	2	11677595	11827409	protein_coding	10,59	0
ENSG00000198885	ITPRIPL1	2	96325331	96330517	protein_coding	10,58	0
ENSG00000167637	ZNF283	19	43827292	43852017	protein_coding	10,58	0
ENSG00000188352	FOCAD	9	20658309	20995955	protein_coding	10,57	0
ENSG00000160753	RUSC1	1	155320896	155331114	protein_coding	10,57	0
ENSG00000164638	SLC29A4	7	5274369	5306870	protein_coding	10,57	0
ENSG00000180423	HARBI1	11	46602861	46617909	protein_coding	10,56	0
ENSG00000162434	JAK1	1	64833229	64966504	protein_coding	10,56	0
ENSG00000118518	RNF146	6	127266610	127288567	protein_coding	10,55	0
ENSG00000164896	FASTK	7	151076593	151080866	protein_coding	10,55	0
ENSG00000162191	UBXN1	11	62676498	62679117	protein_coding	10,54	0
ENSG00000008130	NADK	1	1751232	1780457	protein_coding	10,54	0
ENSG00000203778	FAM229B	6	112087599	112102790	protein_coding	10,54	0
ENSG00000179262	RAD23A	19	12945855	12953642	protein_coding	10,52	0
ENSG00000134444	KIAA1468	18	62187258	62307829	protein_coding	10,52	0
ENSG00000160049	DFFA	1	10456522	10472526	protein_coding	10,52	0
ENSG00000156931	VPS8	3	184812143	185052614	protein_coding	10,52	0
ENSG00000150938	CRIM1	2	36355926	36551135	protein_coding	10,51	0
ENSG00000117616	RSRP1	1	25242237	25338213	protein_coding	10,51	0
ENSG00000140832	MARVELD3	16	71626161	71642114	protein_coding	10,50	0
ENSG00000166523	CLEC4E	12	8533305	8540963	protein_coding	10,50	0
ENSG00000153944	MSI2	17	57255851	57684685	protein_coding	10,50	0
ENSG00000215114	UBXN2B	8	58411264	58451501	protein_coding	10,50	0
ENSG00000136933	RABEPK	9	125200542	125234158	protein_coding	10,50	0

ENSG00000119977	TCTN3	10	95663396	95694143	protein_coding	10,50	0
ENSG00000248487	ABHD14A	3	51971426	51981199	protein_coding	10,49	0
ENSG00000081052	COL4A4	2	227002711	227164113	protein_coding	10,49	0
ENSG00000281656	ENSG000002	7	116248600	116249422	protein_coding	10,48	0
ENSG00000070444	MNT	17	2384060	2401118	protein_coding	10,48	0
ENSG00000112578	BYSL	6	41921188	41933046	protein_coding	10,48	0
ENSG00000174165	ZDHHC24	11	66520637	66546238	protein_coding	10,48	0
ENSG00000122008	POLK	5	75511756	75601144	protein_coding	10,47	0
ENSG00000108405	P2RX1	17	3896592	3916500	protein_coding	10,46	0
ENSG00000169727	GPS1	17	82050691	82057470	protein_coding	10,46	0
ENSG00000104133	SPG11	15	44562696	44663678	protein_coding	10,45	0
ENSG00000173120	KDM2A	11	67119269	67258087	protein_coding	10,45	0
ENSG00000159905	ZNF221	19	43951223	43967709	protein_coding	10,44	0
ENSG00000127328	RAB3IP	12	69738681	69823204	protein_coding	10,44	0
ENSG00000196381	ZNF781	19	37667751	37692322	protein_coding	10,44	0
ENSG00000073849	ST6GAL1	3	186930485	187078553	protein_coding	10,44	0
ENSG00000174851	YIF1A	11	66284580	66289170	protein_coding	10,43	0
ENSG00000141646	SMAD4	18	51028394	51085045	protein_coding	10,42	0
ENSG00000215375	MYL5	4	673580	682033	protein_coding	10,42	0
ENSG00000125971	DYNLRB1	20	34516409	34540958	protein_coding	10,42	0
ENSG00000177311	ZBTB38	3	141324213	141449792	protein_coding	10,42	0
ENSG00000165275	TRMT10B	9	37753803	37778972	protein_coding	10,40	0
ENSG00000164808	SPIDR	8	47260575	47736306	protein_coding	10,40	0
ENSG00000070413	DGCR2	22	19036282	19122454	protein_coding	10,40	0
ENSG00000142599	RERE	1	8352397	8817643	protein_coding	10,39	0
ENSG00000140299	BNIP2	15	59659146	59689534	protein_coding	10,39	0
ENSG00000142655	PEX14	1	10472288	10630758	protein_coding	10,39	0
ENSG00000073598	FNDC8	17	35121579	35130732	protein_coding	10,39	0
ENSG00000153982	GDPD1	17	59220467	59275967	protein_coding	10,38	0
ENSG00000031003	FAM13B	5	137937960	138051961	protein_coding	10,37	0
ENSG00000154114	TBCEL	11	121024072	121090775	protein_coding	10,37	0
ENSG00000143314	MRPL24	1	156737303	156741590	protein_coding	10,37	0
ENSG00000089154	GCN1	12	120127203	120194710	protein_coding	10,36	0
ENSG00000120800	UTP20	12	101280109	101386616	protein_coding	10,36	0
ENSG00000069849	ATP1B3	3	141876124	141926514	protein_coding	10,33	0
ENSG00000131778	CHD1L	1	147242641	147295766	protein_coding	10,33	0
ENSG00000169189	NSMCE1	16	27224991	27268794	protein_coding	10,32	0
ENSG00000198939	ZFP2	5	178895894	178933212	protein_coding	10,31	0
ENSG00000175550	DRAP1	11	65919257	65921561	protein_coding	10,30	0
ENSG00000129467	ADCY4	14	24318349	24335093	protein_coding	10,30	0
ENSG00000120451	SNX19	11	130875436	130916509	protein_coding	10,30	0
ENSG00000184014	DENND5A	11	9138825	9265390	protein_coding	10,29	0
ENSG00000014641	MDH1	2	63588609	63607197	protein_coding	10,29	0
ENSG00000162409	PRKAA2	1	56645322	56715335	protein_coding	10,29	0
ENSG00000120733	KDM3B	5	138352596	138437028	protein_coding	10,29	0
ENSG00000196700	ZNF512B	20	63956702	64048758	protein_coding	10,28	0
ENSG00000138439	FAM117B	2	202635188	202769757	protein_coding	10,28	0
ENSG00000074842	MYDGF	19	4641374	4670370	protein_coding	10,27	0
ENSG00000106244	PDAP1	7	99392048	99408829	protein_coding	10,27	0
ENSG00000132359	RAP1GAP2	17	2777056	3037739	protein_coding	10,27	0
ENSG00000135392	DNAJC14	12	55820960	55830824	protein_coding	10,26	0
ENSG00000198252	STYX	14	52730180	52774998	protein_coding	10,26	0
ENSG00000182670	TTC3	21	37073226	37203112	protein_coding	10,26	0
ENSG00000150722	PPP1R1C	2	181954241	182131398	protein_coding	10,25	0
ENSG00000160097	FNDC5	1	32862268	32872482	protein_coding	10,23	0
ENSG00000151229	SLC2A13	12	39755021	40106089	protein_coding	10,23	0
ENSG00000101290	CDS2	20	5126786	5197887	protein_coding	10,23	0
ENSG00000116688	MFN2	1	11980181	12013514	protein_coding	10,22	0

ENSG00000075539	FRYL	4	48497361	48780322	protein_coding	10,22	0
ENSG00000122884	P4HA1	10	73007217	73096974	protein_coding	10,22	0
ENSG00000147586	MRPS28	8	79918717	80030289	protein_coding	10,22	0
ENSG00000145860	RNF145	5	159157409	159210053	protein_coding	10,22	0
ENSG00000007372	PAX6	11	31784792	31817961	protein_coding	10,21	0
ENSG00000136770	DNAJC1	10	21756537	22003769	protein_coding	10,21	0
ENSG00000035687	ADSS	1	244408494	244452134	protein_coding	10,21	0
ENSG00000139433	GLTP	12	109850943	109880488	protein_coding	10,21	0
ENSG00000079332	SAR1A	10	70147289	70170523	protein_coding	10,20	0
ENSG00000146477	SLC22A3	6	160348268	160452581	protein_coding	10,20	0
ENSG00000115008	IL1A	2	112773915	112784590	protein_coding	10,20	0
ENSG00000116191	RALGPS2	1	178725147	178921841	protein_coding	10,20	0
ENSG00000218739	CEBPZOS	2	37196488	37216193	protein_coding	10,19	0
ENSG00000121892	PDS5A	4	39822863	39977956	protein_coding	10,19	0
ENSG00000119242	CCDC92	12	123918660	123972831	protein_coding	10,19	0
ENSG00000105829	BET1	7	93962762	94004382	protein_coding	10,19	0
ENSG00000165886	UBTD1	10	97498868	97571209	protein_coding	10,19	0
ENSG00000124523	SIRT5	6	13574529	13615158	protein_coding	10,18	0
ENSG00000078668	VDAC3	8	42391624	42405897	protein_coding	10,18	0
ENSG00000198087	CD2AP	6	47477789	47627263	protein_coding	10,17	0
ENSG00000009790	TRAF3IP3	1	209756032	209782320	protein_coding	10,17	0
ENSG00000004455	AK2	1	33007940	33080996	protein_coding	10,16	0
ENSG00000135297	MTO1	6	73461578	73509236	protein_coding	10,15	0
ENSG00000146700	SSC4D	7	76389334	76409695	protein_coding	10,15	0
ENSG00000146476	ARMT1	6	151452258	151470101	protein_coding	10,14	0
ENSG00000196705	ZNF431	19	21142024	21196053	protein_coding	10,14	0
ENSG00000101974	ATP11C	X	139726346	139945276	protein_coding	10,14	0
ENSG00000126215	XRCC3	14	103697609	103715504	protein_coding	10,14	0
ENSG00000164970	FAM219A	9	34398184	34458570	protein_coding	10,13	0
ENSG00000137764	MAP2K5	15	67542709	67807123	protein_coding	10,13	0
ENSG00000004534	RBM6	3	49940007	50100045	protein_coding	10,13	0
ENSG00000103091	WDR59	16	74871367	75000173	protein_coding	10,13	0
ENSG00000124782	RREB1	6	7107597	7251980	protein_coding	10,12	0
ENSG00000185963	BICD2	9	92711363	92764812	protein_coding	10,11	0
ENSG00000166012	TAF1D	11	93729948	93784391	protein_coding	10,11	0
ENSG00000130307	USHBP1	19	17249176	17282786	protein_coding	10,10	0
ENSG00000121903	ZSCAN20	1	33472645	33496507	protein_coding	10,09	0
ENSG00000143450	OAZ3	1	151762899	151771334	protein_coding	10,09	0
ENSG00000140943	MBTPS1	16	84053761	84116906	protein_coding	10,09	0
ENSG00000181350	LRRC75A	17	16441577	16492153	protein_coding	10,08	0
ENSG00000103495	MAZ	16	29806106	29811164	protein_coding	10,08	0
ENSG00000177854	TMEM187	X	153972327	153983195	protein_coding	10,08	0
ENSG00000243725	TTC4	1	54715822	54742657	protein_coding	10,08	0
ENSG00000181830	SLC35C1	11	45804072	45813015	protein_coding	10,08	0
ENSG00000223496	EXOSC6	16	70246778	70251930	protein_coding	10,08	0
ENSG00000117632	STMN1	1	25884181	25906991	protein_coding	10,07	0
ENSG00000183773	AIFM3	22	20965108	20981360	protein_coding	10,07	0
ENSG00000055163	CYFIP2	5	157266079	157395595	protein_coding	10,06	0
ENSG00000251247	ZNF345	19	36850361	36913029	protein_coding	10,06	0
ENSG00000120029	C10orf76	10	101845599	102056193	protein_coding	10,04	0
ENSG00000134056	MRPS36	5	69217760	69230129	protein_coding	10,04	0
ENSG00000168286	THAP11	16	67842082	67844195	protein_coding	10,04	0
ENSG00000174606	ANGEL2	1	212992182	213015826	protein_coding	10,03	0
ENSG00000137691	C11orf70	11	102047443	102084560	protein_coding	10,03	0
ENSG00000068097	HEATR6	17	60043194	60078931	protein_coding	10,03	0
ENSG00000132463	GRSF1	4	70815782	70839945	protein_coding	10,02	0
ENSG00000223547	ZNF844	19	12064699	12081565	protein_coding	10,02	0
ENSG00000173915	USMG5	10	103389041	103396466	protein_coding	10,02	0

ENSG00000136854	STXBP1	9	127611760	127692936	protein_coding	10,02	0
ENSG00000166598	HSP90B1	12	103930107	103953645	protein_coding	10,01	0
ENSG00000095932	SMIM24	19	3473986	3480542	protein_coding	10,00	0
ENSG00000188868	ZNF563	19	12317477	12333720	protein_coding	10,00	0
ENSG00000158406	HIST1H4H	6	26277609	26285638	protein_coding	10,00	0
ENSG00000146083	RNF44	5	176526697	176538025	protein_coding	10,00	0
ENSG00000143061	IGSF3	1	116574399	116667755	protein_coding	9,98	0
ENSG00000168096	ANKS3	16	4696510	4734378	protein_coding	9,95	0
ENSG00000127481	UBR4	1	19074506	19210276	protein_coding	9,94	0
ENSG00000141965	FEM1A	19	4791681	4801273	protein_coding	9,94	0
ENSG00000065833	ME1	6	83210389	83431071	protein_coding	9,92	0
ENSG00000132128	LRRC41	1	46261196	46303608	protein_coding	9,91	0
ENSG00000169683	LRRC45	17	82023302	82031151	protein_coding	9,91	0
ENSG00000155066	PROM2	2	95274453	95291308	protein_coding	9,91	0
ENSG00000182866	LCK	1	32251239	32286165	protein_coding	9,91	0
ENSG00000144214	LYG1	2	99284238	99304742	protein_coding	9,90	0
ENSG00000013725	CD6	11	60971680	61020377	protein_coding	9,90	0
ENSG00000204161	C10orf128	10	49154725	49188585	protein_coding	9,90	0
ENSG00000121594	CD80	3	119524293	119559602	protein_coding	9,90	0
ENSG00000163701	IL17RE	3	9902612	9916402	protein_coding	9,90	0
ENSG00000150995	ITPR1	3	4493348	4847840	protein_coding	9,90	0
ENSG00000264343	NOTCH2NL	1	146146203	146229026	protein_coding	9,89	0
ENSG00000100413	POLR3H	22	41525804	41544606	protein_coding	9,89	0
ENSG00000170871	KIAA0232	4	6781375	6884170	protein_coding	9,89	0
ENSG00000131386	GALNT15	3	16174649	16231992	protein_coding	9,89	0
ENSG00000115694	STK25	2	241492674	241509730	protein_coding	9,88	0
ENSG00000116337	AMPD2	1	109616104	109632051	protein_coding	9,88	0
ENSG00000170085	SIMC1	5	176238367	176345991	protein_coding	9,87	0
ENSG00000185721	DRG1	22	31399523	31528740	protein_coding	9,86	0
ENSG00000162645	GBP2	1	89106132	89150456	protein_coding	9,86	0
ENSG00000141510	TP53	17	7661779	7687550	protein_coding	9,85	0
ENSG00000132823	OSER1	20	44196496	44210791	protein_coding	9,85	0
ENSG00000137573	SULF1	8	69466624	69660915	protein_coding	9,85	0
ENSG00000169692	AGPAT2	9	136673143	136687423	protein_coding	9,84	0
ENSG00000125166	GOT2	16	58707131	58734357	protein_coding	9,83	0
ENSG00000163749	CCDC158	4	76312997	76421868	protein_coding	9,83	0
ENSG00000164715	LMTK2	7	98106885	98209633	protein_coding	9,81	0
ENSG00000160633	SAFB	19	5623035	5668478	protein_coding	9,81	0
ENSG00000132664	POLR3F	20	18467127	18484643	protein_coding	9,81	0
ENSG00000101197	BIRC7	20	63235883	63240507	protein_coding	9,80	0
ENSG00000255587	RAB44	6	36697851	36733183	protein_coding	9,80	0
ENSG00000277877	ENSG00000	1	244845167	244855497	protein_coding	9,80	0
ENSG00000182318	ZSCAN22	19	58327019	58342332	protein_coding	9,80	0
ENSG00000135953	MFSD9	2	102715840	102736888	protein_coding	9,80	0
ENSG00000118965	WDR35	2	19910260	19990131	protein_coding	9,80	0
ENSG00000115183	TANC1	2	158968634	159232659	protein_coding	9,78	0
ENSG00000172748	ZNF596	8	232137	247342	protein_coding	9,78	0
ENSG00000146909	NOM1	7	156949723	156973182	protein_coding	9,77	0
ENSG00000101391	CDK5RAP1	20	33358839	33401561	protein_coding	9,77	0
ENSG00000187210	GCNT1	9	76419850	76507416	protein_coding	9,75	0
ENSG00000162444	RBP7	1	9997206	10016020	protein_coding	9,75	0
ENSG00000198440	ZNF583	19	56397966	56436035	protein_coding	9,74	0
ENSG00000100092	SH3BP1	22	37634654	37666932	protein_coding	9,74	0
ENSG00000167377	ZNF23	16	71447597	71463095	protein_coding	9,73	0
ENSG00000115970	THADA	2	43230836	43596046	protein_coding	9,73	0
ENSG00000106692	FKTN	9	105558130	105641118	protein_coding	9,72	0
ENSG00000198721	ECI2	6	4115689	4135597	protein_coding	9,72	0
ENSG00000144791	LIMD1	3	45555394	45686338	protein_coding	9,71	0

ENSG00000165923	AGBL2	11	47659591	47715389	protein_coding	9,71	0
ENSG00000104324	CPQ	8	96645227	97149654	protein_coding	9,70	0
ENSG00000213380	COG8	16	69320140	69339667	protein_coding	9,70	0
ENSG00000131634	TMEM204	16	1528688	1555580	protein_coding	9,70	0
ENSG00000273173	SNURF	15	24954986	24977850	protein_coding	9,70	0
ENSG00000157999	ANKRD61	7	6031376	6036386	protein_coding	9,70	0
ENSG00000072858	SIDT1	3	113532296	113629578	protein_coding	9,70	0
ENSG00000160050	CCDC28B	1	32200386	32205387	protein_coding	9,70	0
ENSG00000163633	C4orf36	4	86876205	86936202	protein_coding	9,68	0
ENSG00000188343	FAM92A1	8	93698561	93731527	protein_coding	9,68	0
ENSG00000144567	FAM134A	2	219176225	219185479	protein_coding	9,67	0
ENSG00000011143	MKS1	17	58205437	58219605	protein_coding	9,66	0
ENSG00000132356	PRKAA1	5	40759379	40798374	protein_coding	9,66	0
ENSG00000104365	IKBKB	8	42271302	42332653	protein_coding	9,66	0
ENSG00000126934	MAP2K2	19	4090321	4124129	protein_coding	9,66	0
ENSG00000053770	AP5M1	14	57268909	57298742	protein_coding	9,65	0
ENSG00000168255	POLR2J3	7	102537918	102572656	protein_coding	9,65	0
ENSG00000130751	NPAS1	19	47019820	47045775	protein_coding	9,64	0
ENSG00000186951	PPARA	22	46150521	46243756	protein_coding	9,64	0
ENSG00000273749	CYFIP1	15	22867052	22981063	protein_coding	9,64	0
ENSG00000125449	ARMC7	17	75109952	75130265	protein_coding	9,64	0
ENSG00000168887	C2orf68	2	85606654	85612066	protein_coding	9,63	0
ENSG00000162188	GNG3	11	62707658	62709201	protein_coding	9,63	0
ENSG00000152939	MARVELD2	5	69415112	69444330	protein_coding	9,63	0
ENSG00000135119	RNFT2	12	116738178	116853631	protein_coding	9,63	0
ENSG00000169964	TMEM42	3	44861869	44865670	protein_coding	9,62	0
ENSG00000068650	ATP11A	13	112690329	112887168	protein_coding	9,62	0
ENSG00000117013	KCNQ4	1	40784012	40840452	protein_coding	9,62	0
ENSG00000182551	ADI1	2	3497361	3519736	protein_coding	9,62	0
ENSG00000128250	RFPL1	22	29438583	29442455	protein_coding	9,61	0
ENSG00000279530	ENSG00000	12	70321677	70324274	protein_coding	9,60	0
ENSG00000154589	LY96	8	73991352	74029087	protein_coding	9,60	0
ENSG00000099991	CABIN1	22	24011192	24178628	protein_coding	9,60	0
ENSG00000070371	CLTCL1	22	19179473	19291716	protein_coding	9,60	0
ENSG00000068354	TBC1D25	X	48539457	48562609	protein_coding	9,59	0
ENSG00000172590	MRPL52	14	22829879	22835037	protein_coding	9,59	0
ENSG00000198105	ZNF248	10	37776526	37858106	protein_coding	9,59	0
ENSG00000117262	GPR89A	1	145607990	145670648	protein_coding	9,57	0
ENSG00000060237	WNK1	12	752593	911452	protein_coding	9,57	0
ENSG00000129535	NRL	14	24080107	24115014	protein_coding	9,56	0
ENSG00000144649	FAM198A	3	42979267	43060211	protein_coding	9,56	0
ENSG00000119720	NRDE2	14	90267856	90332137	protein_coding	9,55	0
ENSG00000116171	SCP2	1	52927229	53051703	protein_coding	9,55	0
ENSG00000125355	TMEM255A	X	120258650	120311556	protein_coding	9,55	0
ENSG00000154803	FLCN	17	17212212	17237188	protein_coding	9,54	0
ENSG00000197776	KLHDC1	14	49693105	49753152	protein_coding	9,53	0
ENSG00000124164	VAPB	20	58389122	58451101	protein_coding	9,51	0
ENSG00000239704	CDRT4	17	15436015	15503608	protein_coding	9,50	0
ENSG00000205669	ACOT6	14	73610945	73619888	protein_coding	9,50	0
ENSG00000125246	CLYBL	13	99606669	99897134	protein_coding	9,48	0
ENSG00000148341	SH3GLB2	9	129007036	129028303	protein_coding	9,48	0
ENSG00000085788	DDHD2	8	38225218	38275558	protein_coding	9,48	0
ENSG00000108469	RECQL5	17	75626845	75667189	protein_coding	9,48	0
ENSG00000155959	VBP1	X	155197007	155239817	protein_coding	9,47	0
ENSG00000155189	AGPAT5	8	6708357	6759666	protein_coding	9,47	0
ENSG00000133131	MORC4	X	106813871	107000244	protein_coding	9,47	0
ENSG00000133816	MICAL2	11	12094008	12263789	protein_coding	9,47	0
ENSG00000106554	CHCHD3	7	132784868	133082088	protein_coding	9,46	0

ENSG00000158050	DUSP2	2	96143166	96145440	protein_coding	9,46	0
ENSG00000140043	PTGR2	14	73851844	73886827	protein_coding	9,45	0
ENSG00000160075	SSU72	1	1541673	1574869	protein_coding	9,45	0
ENSG00000141380	SS18	18	26016253	26091217	protein_coding	9,45	0
ENSG00000117505	DR1	1	93345888	93369498	protein_coding	9,45	0
ENSG00000070495	JMJD6	17	76712832	76726799	protein_coding	9,45	0
ENSG00000141562	NARF	17	82458180	82490537	protein_coding	9,44	0
ENSG00000165637	VDAC2	10	75210154	75231448	protein_coding	9,43	0
ENSG00000204217	BMPR2	2	202376936	202567751	protein_coding	9,43	0
ENSG00000181982	CCDC149	4	24806117	24980204	protein_coding	9,43	0
ENSG00000102898	NUTF2	16	67846732	67872567	protein_coding	9,43	0
ENSG00000025772	TOMM34	20	44942130	44960486	protein_coding	9,42	0
ENSG00000138071	ACTR2	2	65227753	65271253	protein_coding	9,41	0
ENSG00000143257	NR1I3	1	161229666	161238302	protein_coding	9,41	0
ENSG00000186575	NF2	22	29603556	29698598	protein_coding	9,41	0
ENSG00000084072	PPIE	1	39692182	39763914	protein_coding	9,40	0
ENSG00000122126	OCRL	X	129539849	129592561	protein_coding	9,40	0
ENSG00000064886	CHI3L2	1	111200771	111243440	protein_coding	9,40	0
ENSG00000163467	TSACC	1	156337314	156346995	protein_coding	9,40	0
ENSG00000148158	SNX30	9	112750838	112881671	protein_coding	9,40	0
ENSG00000064961	HMG20B	19	3572777	3579088	protein_coding	9,39	0
ENSG00000109534	GAR1	4	109815510	109824740	protein_coding	9,38	0
ENSG00000054116	TRAPPC3	1	36136570	36156053	protein_coding	9,37	0
ENSG00000162813	BPNT1	1	220057482	220090462	protein_coding	9,37	0
ENSG00000187860	CCDC157	22	30356635	30378658	protein_coding	9,37	0
ENSG00000077097	TOP2B	3	25597905	25664907	protein_coding	9,37	0
ENSG00000139684	ESD	13	46771256	46797232	protein_coding	9,37	0
ENSG00000203872	C6orf163	6	87344849	87365463	protein_coding	9,36	0
ENSG00000183172	SMDT1	22	42079691	42084284	protein_coding	9,36	0
ENSG00000094880	CDC23	5	138187648	138213343	protein_coding	9,35	0
ENSG00000111057	KRT18	12	52948871	52952901	protein_coding	9,34	0
ENSG00000167208	SNX20	16	50666300	50681353	protein_coding	9,33	0
ENSG00000151491	EPS8	12	15620158	15882329	protein_coding	9,33	0
ENSG00000141542	RAB40B	17	82654973	82698728	protein_coding	9,33	0
ENSG00000103510	KAT8	16	31115754	31131393	protein_coding	9,32	0
ENSG00000135407	AVIL	12	57797376	57818704	protein_coding	9,32	0
ENSG00000198945	L3MBTL3	6	130013699	130141451	protein_coding	9,32	0
ENSG00000160691	SHC1	1	154962298	154974395	protein_coding	9,31	0
ENSG00000116898	MRPS15	1	36455718	36464437	protein_coding	9,31	0
ENSG00000196865	NHLRC2	10	113854661	113917194	protein_coding	9,31	0
ENSG00000112305	SMAP1	6	70667776	70862015	protein_coding	9,30	0
ENSG00000165288	BRWD3	X	80670854	80809688	protein_coding	9,30	0
ENSG00000104804	TULP2	19	48880965	48898733	protein_coding	9,30	0
ENSG00000082293	COL19A1	6	69866571	70209976	protein_coding	9,30	0
ENSG00000186007	LEMD1	1	205381378	205457091	protein_coding	9,30	0
ENSG00000117899	MESDC2	15	80946289	80989878	protein_coding	9,28	0
ENSG00000113742	CPEB4	5	173888280	173961976	protein_coding	9,28	0
ENSG00000278845	MRPL45	17	38297023	38323218	protein_coding	9,28	0
ENSG00000163803	PLB1	2	28457145	28643788	protein_coding	9,27	0
ENSG00000182612	TSPAN10	17	81637171	81648749	protein_coding	9,27	0
ENSG00000161217	PCYT1A	3	196214222	196287957	protein_coding	9,27	0
ENSG00000072518	MARK2	11	63838928	63911019	protein_coding	9,26	0
ENSG00000158555	GDPD5	11	75434640	75525903	protein_coding	9,25	0
ENSG00000111196	MAGOHB	12	10604190	10613623	protein_coding	9,25	0
ENSG00000106952	TNFSF8	9	114893343	114930595	protein_coding	9,25	0
ENSG00000178607	ERN1	17	64039142	64130819	protein_coding	9,25	0
ENSG00000103932	RPAP1	15	41517176	41544269	protein_coding	9,24	0
ENSG00000136143	SUCLA2	13	47936491	48001354	protein_coding	9,24	0

ENSG00000113194	FAF2	5	176447628	176510074	protein_coding	9,24	0
ENSG00000147162	OGT	X	71533083	71575897	protein_coding	9,24	0
ENSG00000183778	B3GALT5	21	39556442	39673137	protein_coding	9,23	0
ENSG00000009335	UBE3C	7	157138913	157269372	protein_coding	9,21	0
ENSG00000118058	KMT2A	11	118436490	118526832	protein_coding	9,21	0
ENSG00000109062	SLC9A3R1	17	74748652	74769353	protein_coding	9,20	0
ENSG00000070388	FGF22	19	639879	644371	protein_coding	9,20	0
ENSG00000196684	HSH2D	19	16134028	16158575	protein_coding	9,20	0
ENSG00000182580	EPHB3	3	184561784	184582409	protein_coding	9,20	0
ENSG00000119509	INVS	9	100099256	100301000	protein_coding	9,20	0
ENSG00000056586	RC3H2	9	122844556	122905341	protein_coding	9,18	0
ENSG00000206190	ATP10A	15	25677273	25865172	protein_coding	9,18	0
ENSG00000198019	FCGR1B	1	121087345	121096310	protein_coding	9,18	0
ENSG00000078699	CBFA2T2	20	33490075	33650036	protein_coding	9,18	0
ENSG00000161980	POLR3K	16	46407	53628	protein_coding	9,18	0
ENSG00000115963	RND3	2	150468195	150539011	protein_coding	9,17	0
ENSG00000163704	PRRT3	3	9945542	9952394	protein_coding	9,17	0
ENSG00000051620	HEBP2	6	138403531	138422197	protein_coding	9,17	0
ENSG00000099797	TECR	19	14517085	14565980	protein_coding	9,17	0
ENSG00000182831	C16orf72	16	9091648	9121640	protein_coding	9,17	0
ENSG00000099381	SETD1A	16	30957294	30985116	protein_coding	9,16	0
ENSG00000153820	SPHKAP	2	227979950	228181645	protein_coding	9,15	0
ENSG00000137996	RTCA	1	100266207	100292769	protein_coding	9,15	0
ENSG00000143514	TP53BP2	1	223779899	223845972	protein_coding	9,15	0
ENSG00000158161	EYA3	1	27970344	28088696	protein_coding	9,14	0
ENSG00000110881	ASIC1	12	50057548	50083611	protein_coding	9,14	0
ENSG00000117174	ZNHIT6	1	85649423	85708433	protein_coding	9,14	0
ENSG00000131067	GGT7	20	34844720	34872860	protein_coding	9,14	0
ENSG00000161677	JOSD2	19	50505998	50511353	protein_coding	9,13	0
ENSG00000139146	FAM60A	12	31280584	31327058	protein_coding	9,13	0
ENSG00000153113	CAST	5	96525267	96779595	protein_coding	9,13	0
ENSG00000110218	PANX1	11	94128928	94181972	protein_coding	9,13	0
ENSG00000164291	ARSK	5	95555074	95605064	protein_coding	9,13	0
ENSG00000104765	BNIP3L	8	26382898	26505636	protein_coding	9,12	0
ENSG00000171444	MCC	5	113022099	113488830	protein_coding	9,11	0
ENSG00000161647	MPP3	17	43800799	43833170	protein_coding	9,11	0
ENSG00000151461	UPF2	10	11920022	12043170	protein_coding	9,10	0
ENSG00000174951	FUT1	19	48748011	48755390	protein_coding	9,10	0
ENSG00000121068	TBX2	17	61399896	61409466	protein_coding	9,10	0
ENSG00000069764	PLA2G10	16	14672545	14694669	protein_coding	9,10	0
ENSG00000099834	CDHR5	11	616565	626078	protein_coding	9,10	0
ENSG00000108176	DNAJC12	10	67796665	67838166	protein_coding	9,10	0
ENSG00000225932	CTAGE4	7	144183466	144186053	protein_coding	9,10	0
ENSG00000113211	PCDHB6	5	141150022	141153287	protein_coding	9,10	0
ENSG00000253710	ALG11	13	52012398	52029664	protein_coding	9,09	0
ENSG00000079337	RAPGEF3	12	47734367	47771040	protein_coding	9,09	0
ENSG00000145087	STXBP5L	3	120908072	121424761	protein_coding	9,09	0
ENSG00000142252	GEMIN7	19	45079195	45091524	protein_coding	9,09	0
ENSG00000197937	ZNF347	19	53124072	53159075	protein_coding	9,09	0
ENSG00000136895	GARNL3	9	127224265	127393660	protein_coding	9,08	0
ENSG00000115286	NDUFS7	19	1383527	1395589	protein_coding	9,08	0
ENSG00000205476	CCDC85C	14	99500180	99604026	protein_coding	9,07	0
ENSG00000154380	ENAH	1	225486835	225653142	protein_coding	9,07	0
ENSG00000130227	XPO7	8	21919671	22006585	protein_coding	9,07	0
ENSG00000134243	SORT1	1	109309568	109397951	protein_coding	9,07	0
ENSG00000171067	C11orf24	11	68261335	68272001	protein_coding	9,06	0
ENSG00000160799	CCDC12	3	46921726	46982010	protein_coding	9,06	0
ENSG00000059758	CDK17	12	96278261	96400560	protein_coding	9,06	0

ENSG00000079134	THOC1	18	214520	268050	protein_coding	9,06	0
ENSG00000266338	NBPF15	1	144421386	144461674	protein_coding	9,05	0
ENSG00000084073	ZMPSTE24	1	40258107	40294184	protein_coding	9,05	0
ENSG00000102796	DHRS12	13	51767993	51804157	protein_coding	9,05	0
ENSG00000104081	BMF	15	40087890	40108892	protein_coding	9,05	0
ENSG00000161267	BDH1	3	197509783	197573323	protein_coding	9,05	0
ENSG00000112769	LAMA4	6	112108760	112254939	protein_coding	9,04	0
ENSG00000125875	TBC1D20	20	435480	462553	protein_coding	9,04	0
ENSG00000133606	MKRN1	7	140453040	140479569	protein_coding	9,03	0
ENSG00000054611	TBC1D22A	22	46762617	47175699	protein_coding	9,03	0
ENSG00000133265	HSPBP1	19	55262231	55280381	protein_coding	9,01	0
ENSG00000124767	GLO1	6	38675925	38703141	protein_coding	9,01	0
ENSG00000183401	CCDC159	19	11344684	11354944	protein_coding	9,01	0
ENSG00000125812	GZF1	20	23362182	23373063	protein_coding	9,00	0
ENSG00000140323	DISP2	15	40358235	40378639	protein_coding	9,00	0
ENSG00000115084	SLC35F5	2	113705011	113756823	protein_coding	9,00	0
ENSG00000134198	TSPAN2	1	115048011	115089500	protein_coding	9,00	0
ENSG00000092439	TRPM7	15	50552473	50686815	protein_coding	9,00	0
ENSG00000128609	NDUFA5	7	123536997	123558255	protein_coding	9,00	0
ENSG00000105676	ARMC6	19	19033575	19060311	protein_coding	8,99	0
ENSG00000130733	YIPF2	19	10922185	10928681	protein_coding	8,98	0
ENSG00000197223	C1D	2	68041130	68110948	protein_coding	8,97	0
ENSG00000123810	B9D2	19	41354421	41364173	protein_coding	8,96	0
ENSG00000065485	PDIA5	3	123067062	123225227	protein_coding	8,95	0
ENSG00000232859	LYRM9	17	27878314	27894752	protein_coding	8,94	0
ENSG00000240857	RDH14	2	18554723	18560680	protein_coding	8,94	0
ENSG00000143036	SLC44A3	1	94820342	94895246	protein_coding	8,94	0
ENSG00000084636	COL16A1	1	31652247	31704319	protein_coding	8,94	0
ENSG00000228696	ARL17B	17	46274784	46361797	protein_coding	8,93	0
ENSG00000182858	ALG12	22	49900229	49918458	protein_coding	8,93	0
ENSG00000037637	FBXO42	1	16246839	16352454	protein_coding	8,92	0
ENSG00000125445	MRPS7	17	75261674	75266373	protein_coding	8,91	0
ENSG00000205269	TMEM170B	6	11538278	11583524	protein_coding	8,90	0
ENSG00000102854	MSLN	16	643262	768865	protein_coding	8,90	0
ENSG00000102962	CCL22	16	57358772	57366190	protein_coding	8,90	0
ENSG00000164694	FNDC1	6	159169397	159272109	protein_coding	8,90	0
ENSG00000172901	LVRN	5	115962454	116027619	protein_coding	8,90	0
ENSG00000123416	TUBA1B	12	49127782	49131397	protein_coding	8,89	0
ENSG00000184056	VPS33B	15	90998416	91022603	protein_coding	8,89	0
ENSG00000100347	SAMM50	22	43955421	44010531	protein_coding	8,89	0
ENSG00000168291	PDHB	3	58427630	58433857	protein_coding	8,88	0
ENSG00000151689	INPP1	2	190343470	190371665	protein_coding	8,88	0
ENSG00000129197	RPAIN	17	5419641	5432876	protein_coding	8,87	0
ENSG00000136044	APPL2	12	105173296	105236238	protein_coding	8,87	0
ENSG00000139083	ETV6	12	11649854	11895402	protein_coding	8,87	0
ENSG00000108424	KPNB1	17	47649476	47685505	protein_coding	8,87	0
ENSG00000177663	IL17RA	22	17084954	17115694	protein_coding	8,85	0
ENSG00000043093	DCUN1D1	3	182938074	182985953	protein_coding	8,85	0
ENSG00000106526	ACTR3C	7	150243916	150323725	protein_coding	8,85	0
ENSG00000013306	SLC25A39	17	44319625	44324870	protein_coding	8,85	0
ENSG00000099817	POLR2E	19	1086579	1095380	protein_coding	8,85	0
ENSG00000102904	TSNAXIP1	16	67806765	67832148	protein_coding	8,84	0
ENSG00000168701	TMEM208	16	67227103	67229278	protein_coding	8,84	0
ENSG00000138190	EXOC6	10	92834713	93059493	protein_coding	8,84	0
ENSG00000118495	PLAGL1	6	143940300	144064599	protein_coding	8,84	0
ENSG00000102172	SMS	X	21940573	21994835	protein_coding	8,83	0
ENSG00000213995	CARKD	13	110615460	110639993	protein_coding	8,82	0
ENSG00000147127	RAB41	X	70282093	70285002	protein_coding	8,82	0

ENSG00000196305	IARS	9	92210207	92293756	protein_coding	8,82	0
ENSG00000173214	KIAA1919	6	111259348	111271167	protein_coding	8,81	0
ENSG00000103111	MON1B	16	77190835	77202405	protein_coding	8,80	0
ENSG00000146242	TPBG	6	82363206	82370828	protein_coding	8,80	0
ENSG00000132010	ZNF20	19	12092843	12140407	protein_coding	8,80	0
ENSG00000126353	CCR7	17	40553769	40565472	protein_coding	8,80	0
ENSG00000093134	VNN3	6	132722787	132734765	protein_coding	8,80	0
ENSG00000262209	PCDHGB3	5	141370264	141512979	protein_coding	8,80	0
ENSG00000109193	SULT1E1	4	69810780	69860152	protein_coding	8,80	0
ENSG00000120280	CXorf21	X	30558824	30577844	protein_coding	8,80	0
ENSG00000198798	MAGEB3	X	30230436	30237492	protein_coding	8,80	0
ENSG00000122359	ANXA11	10	80150889	80205572	protein_coding	8,80	0
ENSG00000155034	FBXL18	7	5431335	5513798	protein_coding	8,79	0
ENSG00000232838	PET117	20	18137873	18143169	protein_coding	8,79	0
ENSG00000008513	ST3GAL1	8	133454848	133571940	protein_coding	8,78	0
ENSG00000122545	09/01/2007	7	35800932	35907105	protein_coding	8,78	0
ENSG00000008256	CYTH3	7	6161776	6272644	protein_coding	8,78	0
ENSG00000105355	PLIN3	19	4838341	4867768	protein_coding	8,77	0
ENSG00000183826	BTBD9	6	38168451	38640148	protein_coding	8,76	0
ENSG00000160298	C21orf58	21	46300181	46323875	protein_coding	8,76	0
ENSG00000090674	MCOLN1	19	7522626	7534009	protein_coding	8,74	0
ENSG00000205832	C16orf96	16	4556490	4600714	protein_coding	8,73	0
ENSG00000166401	SERPINB8	18	63969925	64005667	protein_coding	8,72	0
ENSG00000170364	SETMAR	3	4303304	4317567	protein_coding	8,72	0
ENSG00000158158	CNNM4	2	96760902	96811891	protein_coding	8,72	0
ENSG00000178177	LCORL	4	17841199	18021876	protein_coding	8,72	0
ENSG00000125447	GGA3	17	75236599	75262363	protein_coding	8,71	0
ENSG00000124496	TRERF1	6	42224931	42452051	protein_coding	8,71	0
ENSG00000135686	KLHL36	16	84648525	84667686	protein_coding	8,71	0
ENSG00000110925	CSRNP2	12	51061205	51083664	protein_coding	8,70	0
ENSG00000198597	ZNF536	19	30228290	30713538	protein_coding	8,70	0
ENSG00000180316	PNPLA1	6	36243203	36308595	protein_coding	8,70	0
ENSG00000180891	CUEDC1	17	57861243	57955323	protein_coding	8,69	0
ENSG00000166912	MTMR10	15	30938941	30991607	protein_coding	8,69	0
ENSG00000141040	ZNF287	17	16551387	16569206	protein_coding	8,69	0
ENSG00000242485	MRPL20	1	1401908	1407313	protein_coding	8,68	0
ENSG00000120314	WDR55	5	140664676	140674124	protein_coding	8,68	0
ENSG00000168434	COG7	16	23388493	23453180	protein_coding	8,67	0
ENSG00000084774	CAD	2	27217390	27243943	protein_coding	8,67	0
ENSG00000086598	TMED2	12	123584531	123598577	protein_coding	8,66	0
ENSG00000122484	RPAP2	1	92298965	92402056	protein_coding	8,65	0
ENSG00000165060	FXN	9	69035259	69100178	protein_coding	8,65	0
ENSG00000127914	AKAP9	7	91940867	92110673	protein_coding	8,65	0
ENSG00000188234	AGAP4	10	45825594	45853875	protein_coding	8,65	0
ENSG00000204628	GNB2L1	5	181236909	181248096	protein_coding	8,63	0
ENSG00000137106	GRHPR	9	37422666	37436990	protein_coding	8,63	0
ENSG00000105186	ANKRD27	19	32597007	32676597	protein_coding	8,63	0
ENSG00000138678	GPAT3	4	83535914	83605875	protein_coding	8,62	0
ENSG00000256229	ZNF486	19	20167228	20200490	protein_coding	8,61	0
ENSG00000165325	CCDC67	11	93329971	93438487	protein_coding	8,60	0
ENSG00000158683	PKD1L1	7	47774652	47948491	protein_coding	8,60	0
ENSG00000163221	S100A12	1	153373706	153375649	protein_coding	8,60	0
ENSG00000111269	CREBL2	12	12611827	12645108	protein_coding	8,60	0
ENSG00000148337	CIZ1	9	128166064	128204383	protein_coding	8,60	0
ENSG00000073792	IGF2BP2	3	185643739	185825056	protein_coding	8,59	0
ENSG00000086102	NFX1	9	33290511	33371157	protein_coding	8,59	0
ENSG00000261236	BOP1	8	144262102	144291370	protein_coding	8,59	0
ENSG00000196975	ANXA4	2	69644425	69827100	protein_coding	8,58	0

ENSG00000053524	MCF2L2	3	183178043	183428778	protein_coding	8,58	0
ENSG00000112144	ICK	6	53001279	53061802	protein_coding	8,57	0
ENSG00000134490	TMEM241	18	23197144	23437961	protein_coding	8,57	0
ENSG00000103034	NDRG4	16	58462846	58513628	protein_coding	8,57	0
ENSG00000167740	CYB5D2	17	4143168	4187310	protein_coding	8,56	0
ENSG00000147224	PRPS1	X	107628424	107651026	protein_coding	8,56	0
ENSG00000138495	COX17	3	119654513	119677454	protein_coding	8,56	0
ENSG00000163219	ARHGAP25	2	68679601	68826833	protein_coding	8,56	0
ENSG00000065613	SLK	10	103967201	104029233	protein_coding	8,56	0
ENSG00000198934	MAGEE1	X	76427724	76431353	protein_coding	8,55	0
ENSG00000260916	CCPG1	15	55340032	55408510	protein_coding	8,55	0
ENSG00000176994	SMCR8	17	18315310	18328055	protein_coding	8,55	0
ENSG00000204536	CCHCR1	6	31142439	31158238	protein_coding	8,54	0
ENSG00000104131	EIF3J	15	44537057	44563029	protein_coding	8,54	0
ENSG00000129195	FAM64A	17	6444415	6451469	protein_coding	8,54	0
ENSG00000135070	ISCA1	9	86264546	86282538	protein_coding	8,54	0
ENSG00000018510	AGPS	2	177392644	177543836	protein_coding	8,53	0
ENSG00000058056	USP13	3	179652755	179789401	protein_coding	8,53	0
ENSG00000114353	GNAI2	3	50226292	50259355	protein_coding	8,53	0
ENSG00000135862	LAMC1	1	183023460	183145592	protein_coding	8,53	0
ENSG00000131051	RBM39	20	35703609	35742312	protein_coding	8,52	0
ENSG00000149932	TMEM219	16	29940885	29973052	protein_coding	8,52	0
ENSG00000113013	HSPA9	5	138554882	138575444	protein_coding	8,50	0
ENSG00000077984	CST7	20	24949230	24959928	protein_coding	8,50	0
ENSG00000092051	JPH4	14	23568035	23578800	protein_coding	8,50	0
ENSG00000119714	GPR68	14	91232532	91253925	protein_coding	8,50	0
ENSG00000203666	EFCAB2	1	244969705	245127164	protein_coding	8,50	0
ENSG00000167632	TRAPPC9	8	139730343	140458579	protein_coding	8,48	0
ENSG00000122203	KIAA1191	5	176346061	176361968	protein_coding	8,48	0
ENSG00000204611	ZNF616	19	52113091	52139922	protein_coding	8,47	0
ENSG00000105127	AKAP8	19	15353385	15379798	protein_coding	8,47	0
ENSG00000161714	PLCD3	17	45108967	45133354	protein_coding	8,46	0
ENSG00000155380	SLC16A1	1	112911847	112957013	protein_coding	8,46	0
ENSG00000104976	SNAPC2	19	7920316	7923250	protein_coding	8,46	0
ENSG00000104886	PLEKHJ1	19	2230084	2237704	protein_coding	8,46	0
ENSG00000181135	ZNF707	8	143684452	143713898	protein_coding	8,46	0
ENSG00000070526	ST6GALNAC1	17	76624761	76643838	protein_coding	8,45	0
ENSG00000189180	ZNF33A	10	38010650	38065088	protein_coding	8,45	0
ENSG00000165731	RET	10	43077027	43130351	protein_coding	8,45	0
ENSG00000144566	RAB5A	3	19947079	19985175	protein_coding	8,45	0
ENSG00000080815	PSEN1	14	73136418	73223691	protein_coding	8,45	0
ENSG00000009413	REV3L	6	111299028	111483715	protein_coding	8,44	0
ENSG00000141577	CEP131	17	81189593	81222999	protein_coding	8,44	0
ENSG00000183808	RBM12B	8	93729356	93741017	protein_coding	8,44	0
ENSG00000166947	EPB42	15	43106225	43221283	protein_coding	8,43	0
ENSG00000172939	OXSR1	3	38165089	38255488	protein_coding	8,43	0
ENSG00000144029	MRPS5	2	95087207	95149434	protein_coding	8,43	0
ENSG00000126785	RHOJ	14	63204114	63293219	protein_coding	8,42	0
ENSG00000118454	ANKRD13C	1	70260588	70354734	protein_coding	8,41	0
ENSG00000140022	STON2	14	81260656	81436465	protein_coding	8,41	0
ENSG00000125821	DTD1	20	18567453	18763917	protein_coding	8,41	0
ENSG00000175220	ARHGAP1	11	46677080	46700615	protein_coding	8,41	0
ENSG00000171236	LRG1	19	4536409	4540474	protein_coding	8,41	0
ENSG00000002549	LAP3	4	17577192	17607972	protein_coding	8,41	0
ENSG00000126214	KLC1	14	103561896	103714249	protein_coding	8,40	0
ENSG00000105855	ITGB8	7	20330702	20415754	protein_coding	8,40	0
ENSG00000171861	RNMTL1	17	782273	792509	protein_coding	8,40	0
ENSG00000133275	CSNK1G2	19	1941149	1981338	protein_coding	8,40	0

ENSG00000010310	GIPR	19	45668244	45683724	protein_coding	8,40	0
ENSG00000213203	GIMAP1	7	150716557	150724284	protein_coding	8,40	0
ENSG00000074276	CDHR2	5	176542511	176595974	protein_coding	8,40	0
ENSG00000145687	SSBP2	5	81413021	81751797	protein_coding	8,39	0
ENSG00000120071	KANSL1	17	46029916	46225374	protein_coding	8,39	0
ENSG00000106351	AGFG2	7	100539211	100568219	protein_coding	8,39	0
ENSG00000279767	ENSG000002	1	153772371	153774079	protein_coding	8,38	0
ENSG00000186716	BCR	22	23179704	23318037	protein_coding	8,38	0
ENSG00000075415	SLC25A3	12	98593591	98606379	protein_coding	8,37	0
ENSG00000109189	USP46	4	52590972	52659335	protein_coding	8,37	0
ENSG00000198830	HMGN2	1	26472450	26475972	protein_coding	8,36	0
ENSG00000253958	CLDN23	8	8701938	8704106	protein_coding	8,36	0
ENSG00000119878	CRIPT	2	46616416	46625742	protein_coding	8,35	0
ENSG00000130669	PAK4	19	39125770	39182816	protein_coding	8,35	0
ENSG00000132300	PTCD3	2	86106182	86142157	protein_coding	8,35	0
ENSG00000185728	YTHDF3	8	63168553	63212786	protein_coding	8,35	0
ENSG00000121350	PYROXD1	12	21437615	21471252	protein_coding	8,34	0
ENSG00000165675	ENOX2	X	130623369	130903317	protein_coding	8,32	0
ENSG00000135677	GNS	12	64713445	64759447	protein_coding	8,30	0
ENSG00000177191	B3GNT8	19	41425359	41428730	protein_coding	8,30	0
ENSG00000136514	RTP4	3	187368332	187372076	protein_coding	8,30	0
ENSG00000156990	RPUSD3	3	9837849	9844602	protein_coding	8,29	0
ENSG00000162368	CMPK1	1	47333797	47378839	protein_coding	8,28	0
ENSG00000196510	ANAPC7	12	110372900	110403730	protein_coding	8,28	0
ENSG00000163013	FBXO41	2	73254682	73284431	protein_coding	8,28	0
ENSG00000144824	PHLDB2	3	111732497	111976517	protein_coding	8,28	0
ENSG00000148832	PAOX	10	133379234	133391694	protein_coding	8,28	0
ENSG00000022567	SLC45A4	8	141207166	141308305	protein_coding	8,27	0
ENSG00000151014	NOCT	4	139015789	139045939	protein_coding	8,27	0
ENSG00000167969	ECI1	16	2239395	2252300	protein_coding	8,27	0
ENSG00000119402	FBXW2	9	120751978	120793412	protein_coding	8,25	0
ENSG00000197111	PCBP2	12	53452102	53481162	protein_coding	8,25	0
ENSG00000144021	CIAO1	2	96266132	96273349	protein_coding	8,24	0
ENSG00000083067	TRPM3	9	70529063	71446904	protein_coding	8,24	0
ENSG00000106785	TRIM14	9	98069275	98119212	protein_coding	8,23	0
ENSG00000179115	FARSA	19	12922479	12934037	protein_coding	8,23	0
ENSG00000149476	TKFC	11	61333210	61353295	protein_coding	8,22	0
ENSG00000143786	CNIH3	1	224434660	224740549	protein_coding	8,22	0
ENSG00000180357	ZNF609	15	64460742	64686068	protein_coding	8,22	0
ENSG00000188529	SRSF10	1	23964804	23980927	protein_coding	8,22	0
ENSG00000177084	POLE	12	132623753	132687365	protein_coding	8,21	0
ENSG00000170653	ATF7	12	53507856	53626410	protein_coding	8,21	0
ENSG00000147592	LACTB2	8	70635318	70669174	protein_coding	8,21	0
ENSG00000127533	F2RL3	19	16888860	16892606	protein_coding	8,20	0
ENSG00000111540	RAB5B	12	55973913	55996683	protein_coding	8,20	0
ENSG00000114999	TTL	2	112482154	112541739	protein_coding	8,20	0
ENSG00000102893	PHKB	16	47461123	47701523	protein_coding	8,20	0
ENSG00000163956	LRPAP1	4	3506376	3532559	protein_coding	8,20	0
ENSG00000198795	ZNF521	18	25061926	25352190	protein_coding	8,19	0
ENSG00000123352	SPATS2	12	49366584	49527424	protein_coding	8,19	0
ENSG00000120992	LYPLA1	8	54046367	54102017	protein_coding	8,18	0
ENSG00000033327	GAB2	11	78215297	78418348	protein_coding	8,18	0
ENSG00000116539	ASH1L	1	155335268	155562807	protein_coding	8,17	0
ENSG00000102595	UGGT2	13	95801580	96053482	protein_coding	8,17	0
ENSG00000125484	GTF3C4	9	132670035	132694955	protein_coding	8,17	0
ENSG00000164010	ERMAP	1	42817124	42844989	protein_coding	8,16	0
ENSG00000168374	ARF4	3	57571363	57598220	protein_coding	8,16	0
ENSG00000133065	SLC41A1	1	205789093	205813748	protein_coding	8,16	0

ENSG00000100852	ARHGAP5	14	32076114	32159728	protein_coding	8,15	0
ENSG00000105176	URI1	19	29923644	30016608	protein_coding	8,15	0
ENSG00000005893	LAMP2	X	120427827	120469365	protein_coding	8,15	0
ENSG00000107164	FUBP3	9	130578965	130638352	protein_coding	8,15	0
ENSG00000131061	ZNF341	20	33731657	33792269	protein_coding	8,14	0
ENSG00000109016	DHRS7B	17	21123364	21193265	protein_coding	8,14	0
ENSG00000118292	C1orf54	1	150268200	150280916	protein_coding	8,13	0
ENSG00000166123	GPT2	16	46884378	46931297	protein_coding	8,13	0
ENSG00000143183	TMCO1	1	165724293	165827755	protein_coding	8,13	0
ENSG00000243646	IL10RB	21	33266358	33297234	protein_coding	8,11	0
ENSG00000125780	TGM3	20	2296001	2341078	protein_coding	8,10	0
ENSG00000173868	PHOSPHO1	17	49223362	49230766	protein_coding	8,10	0
ENSG00000198851	CD3E	11	118304545	118316175	protein_coding	8,10	0
ENSG00000152763	WDR78	1	66812885	66924887	protein_coding	8,10	0
ENSG00000148680	HTR7	10	90740823	90857698	protein_coding	8,09	0
ENSG00000196867	ZFP28	19	56538948	56556810	protein_coding	8,09	0
ENSG00000167232	ZNF91	19	23304991	23395560	protein_coding	8,09	0
ENSG00000089053	ANAPC5	12	121308245	121399896	protein_coding	8,09	0
ENSG00000130635	COL5A1	9	134641774	134844843	protein_coding	8,09	0
ENSG00000083857	FAT1	4	186587783	186726722	protein_coding	8,08	0
ENSG00000168259	DNAJC7	17	41976433	42021376	protein_coding	8,08	0
ENSG00000099995	SF3A1	22	30331988	30356947	protein_coding	8,08	0
ENSG00000107560	RAB11FIP2	10	118004916	118046603	protein_coding	8,07	0
ENSG00000100060	MFNG	22	37469063	37486401	protein_coding	8,07	0
ENSG00000130489	SCO2	22	50523568	50526439	protein_coding	8,07	0
ENSG00000184083	FAM120C	X	54068324	54183281	protein_coding	8,06	0
ENSG00000164338	UTP15	5	73565443	73583377	protein_coding	8,06	0
ENSG00000150760	DOCK1	10	126905409	127452517	protein_coding	8,06	0
ENSG00000105650	PDE4C	19	18207961	18255419	protein_coding	8,06	0
ENSG00000055211	GINM1	6	149566294	149591748	protein_coding	8,06	0
ENSG00000133195	SLC39A11	17	72645949	73092712	protein_coding	8,05	0
ENSG00000101298	SNPH	20	1266316	1309328	protein_coding	8,05	0
ENSG00000178917	ZNF852	3	44498970	44510640	protein_coding	8,05	0
ENSG00000050820	BCAR1	16	75228187	75268053	protein_coding	8,05	0
ENSG00000078687	TNRC6C	17	78004168	78108835	protein_coding	8,05	0
ENSG00000129083	COPB1	11	14443440	14500027	protein_coding	8,04	0
ENSG00000023041	ZDHHC6	10	112424428	112446917	protein_coding	8,04	0
ENSG00000135049	AGTPBP1	9	85546539	85742029	protein_coding	8,03	0
ENSG00000168137	SETD5	3	9397615	9479240	protein_coding	8,03	0
ENSG00000152904	GGPS1	1	235327350	235344532	protein_coding	8,03	0
ENSG00000125966	MMP24	20	35226654	35277000	protein_coding	8,02	0
ENSG00000198131	ZNF544	19	58228594	58277495	protein_coding	8,02	0
ENSG00000115234	SNX17	2	27370496	27377533	protein_coding	8,02	0
ENSG00000214756	METTL12	11	62665309	62668496	protein_coding	8,02	0
ENSG00000154642	C21orf91	21	17788967	17819386	protein_coding	8,02	0
ENSG00000198482	ZNF808	19	52527652	52564464	protein_coding	8,02	0
ENSG00000170471	RALGAPB	20	38472816	38578861	protein_coding	8,01	0
ENSG00000148459	PDSS1	10	26697659	26746798	protein_coding	8,01	0
ENSG00000158710	TAGLN2	1	159918107	159925732	protein_coding	8,01	0
ENSG00000185344	ATP6V0A2	12	123712318	123761755	protein_coding	8,00	0
ENSG00000122224	LY9	1	160796074	160828261	protein_coding	8,00	0
ENSG00000133048	CHI3L1	1	203178931	203186749	protein_coding	8,00	0
ENSG00000136630	HLX	1	220879400	220885059	protein_coding	8,00	0
ENSG00000198879	SFMBT2	10	7158624	7411486	protein_coding	8,00	0
ENSG00000092330	TINF2	14	24239643	24242674	protein_coding	7,99	0
ENSG00000077150	NFKB2	10	102394110	102402529	protein_coding	7,99	0
ENSG00000197822	OCLN	5	69492292	69558104	protein_coding	7,98	0
ENSG00000072506	HSD17B10	X	53431258	53434373	protein_coding	7,98	0

ENSG00000128050	PAICS	4	56435741	56464579	protein_coding	7,97	0
ENSG00000185664	PMEL	12	55954105	55973317	protein_coding	7,97	0
ENSG00000171606	ZNF274	19	58183029	58213562	protein_coding	7,97	0
ENSG00000183255	PTTG1IP	21	44849585	44873903	protein_coding	7,97	0
ENSG00000108100	CCNY	10	35247025	35572669	protein_coding	7,97	0
ENSG00000175106	TVP23C	17	15502264	15563595	protein_coding	7,96	0
ENSG00000102897	LYRM1	16	20899868	20925006	protein_coding	7,96	0
ENSG00000138382	METTL5	2	169810081	169824931	protein_coding	7,96	0
ENSG00000150477	KIAA1328	18	36829106	37232172	protein_coding	7,96	0
ENSG00000137193	PIM1	6	37170203	37175426	protein_coding	7,96	0
ENSG00000130414	NDUFA10	2	239892450	240025402	protein_coding	7,96	0
ENSG00000125970	RALY	20	33993646	34108308	protein_coding	7,95	0
ENSG00000108256	NUFIP2	17	29255836	29294118	protein_coding	7,95	0
ENSG00000067606	PRKCZ	1	2050470	2185395	protein_coding	7,95	0
ENSG00000125122	LRRC29	16	67207139	67227048	protein_coding	7,94	0
ENSG00000157014	TATDN2	3	10248023	10281218	protein_coding	7,94	0
ENSG00000162595	DIRAS3	1	68045962	68051631	protein_coding	7,94	0
ENSG00000180628	PCGF5	10	91163012	91284331	protein_coding	7,93	0
ENSG00000184408	KCND2	7	120273668	120750331	protein_coding	7,93	0
ENSG00000164951	PDP1	8	93857807	93926066	protein_coding	7,92	0
ENSG00000169976	SF3B5	6	144094881	144095573	protein_coding	7,92	0
ENSG00000254999	BRK1	3	10115592	10127190	protein_coding	7,92	0
ENSG00000090520	DNAJB11	3	186567403	186597203	protein_coding	7,92	0
ENSG00000198538	ZNF28	19	52797409	52857600	protein_coding	7,91	0
ENSG00000130402	ACTN4	19	38647649	38731583	protein_coding	7,91	0
ENSG00000080371	RAB21	12	71754874	71800285	protein_coding	7,91	0
ENSG00000113643	RARS	5	168486445	168519299	protein_coding	7,91	0
ENSG00000255561	FDXACB1	11	111874056	111881243	protein_coding	7,91	0
ENSG00000172354	GNB2	7	100673531	100679174	protein_coding	7,90	0
ENSG00000169181	GSG1L	16	27787535	28063509	protein_coding	7,90	0
ENSG00000156127	BATF	14	75522425	75547015	protein_coding	7,90	0
ENSG00000255112	CHMP1B	18	11851396	11854449	protein_coding	7,90	0
ENSG00000073331	ALPK1	4	112285509	112442620	protein_coding	7,88	0
ENSG00000184182	UBE2F	2	237966827	238042782	protein_coding	7,88	0
ENSG00000080839	RBL1	20	36996349	37095995	protein_coding	7,87	0
ENSG00000040531	CTNS	17	3636468	3661542	protein_coding	7,87	0
ENSG00000196642	RABL6	9	136807943	136957733	protein_coding	7,86	0
ENSG00000108433	GOSR2	17	46923117	46967019	protein_coding	7,86	0
ENSG00000153395	LPCAT1	5	1456480	1523977	protein_coding	7,85	0
ENSG00000141429	GALNT1	18	35581117	35711834	protein_coding	7,85	0
ENSG00000164199	ADGRV1	5	90529344	91164221	protein_coding	7,85	0
ENSG00000103047	TANGO6	16	68843604	69085180	protein_coding	7,83	0
ENSG00000123124	WWP1	8	86342738	86478420	protein_coding	7,83	0
ENSG00000146416	AIG1	6	143060496	143340304	protein_coding	7,83	0
ENSG00000196352	CD55	1	207321508	207386804	protein_coding	7,82	0
ENSG00000163479	SSR2	1	156009048	156020959	protein_coding	7,82	0
ENSG00000091527	CDV3	3	133573730	133590261	protein_coding	7,82	0
ENSG00000166135	HIF1AN	10	100529072	100559998	protein_coding	7,81	0
ENSG00000075785	RAB7A	3	128726122	128814796	protein_coding	7,80	0
ENSG00000104998	IL27RA	19	14031748	14053216	protein_coding	7,80	0
ENSG00000144406	UNC80	2	209771993	209999300	protein_coding	7,80	0
ENSG00000167771	RCOR2	11	63911221	63916844	protein_coding	7,79	0
ENSG00000143436	MRPL9	1	151759643	151763564	protein_coding	7,78	0
ENSG00000180758	GPR157	1	9100305	9129170	protein_coding	7,78	0
ENSG00000196419	XRCC6	22	41621119	41664048	protein_coding	7,78	0
ENSG00000008869	HEATR5B	2	36968383	37084342	protein_coding	7,78	0
ENSG00000152795	HNRNPDL	4	82422564	82430408	protein_coding	7,77	0
ENSG00000117751	PPP1R8	1	27830778	27851676	protein_coding	7,77	0

ENSG00000167173	C15orf39	15	75195643	75212169	protein_coding	7,77	0
ENSG00000077348	EXOSC5	19	41386374	41397479	protein_coding	7,77	0
ENSG00000127511	SIN3B	19	16829400	16880353	protein_coding	7,74	0
ENSG00000144043	TEX261	2	70985938	70994945	protein_coding	7,74	0
ENSG00000148396	SEC16A	9	136440096	136483759	protein_coding	7,74	0
ENSG00000122591	FAM126A	7	22889371	23014130	protein_coding	7,74	0
ENSG00000138050	THUMPD2	2	39736060	39779267	protein_coding	7,73	0
ENSG00000151445	VIPAS39	14	77426675	77457952	protein_coding	7,73	0
ENSG00000144034	TPRKB	2	73729104	73737400	protein_coding	7,71	0
ENSG00000185085	INTS5	11	62646848	62653302	protein_coding	7,71	0
ENSG00000160948	VPS28	8	144423601	144428563	protein_coding	7,71	0
ENSG00000096996	IL12RB1	19	18058995	18098944	protein_coding	7,70	0
ENSG00000015520	NPC1L1	7	44512535	44541315	protein_coding	7,70	0
ENSG00000091138	SLC26A3	7	107765467	107803225	protein_coding	7,70	0
ENSG00000135622	SEMA4F	2	74654228	74683853	protein_coding	7,70	0
ENSG00000084234	APLP2	11	130069837	130144811	protein_coding	7,70	0
ENSG00000130508	PXDN	2	1631887	1744852	protein_coding	7,69	0
ENSG00000164081	TEX264	3	51662693	51704323	protein_coding	7,69	0
ENSG00000136490	LIMD2	17	63695902	63701172	protein_coding	7,68	0
ENSG00000198355	PIM3	22	49960513	49964080	protein_coding	7,68	0
ENSG00000110841	PPFIBP1	12	27523431	27695564	protein_coding	7,67	0
ENSG00000082512	TRAF5	1	211326615	211374946	protein_coding	7,66	0
ENSG00000171552	BCL2L1	20	31664452	31723989	protein_coding	7,66	0
ENSG00000176102	CSTF3	11	33077188	33162371	protein_coding	7,65	0
ENSG00000119661	DNAL1	14	73644875	73703732	protein_coding	7,65	0
ENSG00000087299	L2HGDH	14	50237563	50312548	protein_coding	7,65	0
ENSG00000102383	ZDHHC15	X	75368427	75523502	protein_coding	7,65	0
ENSG00000149100	EIF3M	11	32583798	32606262	protein_coding	7,64	0
ENSG00000159720	ATP6V0D1	16	67438014	67481237	protein_coding	7,64	0
ENSG00000123094	RASSF8	12	25959029	26079892	protein_coding	7,64	0
ENSG00000163810	TGM4	3	44874608	44914990	protein_coding	7,63	0
ENSG00000186834	HEXIM1	17	45148502	45152101	protein_coding	7,63	0
ENSG00000169696	ASPSCR1	17	81976807	82017406	protein_coding	7,63	0
ENSG00000176809	LRRC37A3	17	64854312	64919480	protein_coding	7,62	0
ENSG00000135926	TMBIM1	2	218274192	218292586	protein_coding	7,61	0
ENSG00000057252	SOAT1	1	179293714	179358680	protein_coding	7,61	0
ENSG00000071537	SEL1L	14	81471549	81533861	protein_coding	7,60	0
ENSG00000180389	ATP5EP2	13	27945206	27945590	protein_coding	7,60	0
ENSG00000129317	PUS7L	12	43718993	43758817	protein_coding	7,59	0
ENSG00000104529	EEF1D	8	143579697	143599541	protein_coding	7,59	0
ENSG00000089818	NECAP1	12	8082211	8097771	protein_coding	7,59	0
ENSG00000130244	FAM98C	19	38403135	38409088	protein_coding	7,58	0
ENSG00000198873	GRK5	10	119207589	119459742	protein_coding	7,58	0
ENSG00000116954	RRAGC	1	38838198	38859823	protein_coding	7,58	0
ENSG00000130684	ZNF337	20	25674215	25696841	protein_coding	7,58	0
ENSG00000114796	KLHL24	3	183635568	183684477	protein_coding	7,58	0
ENSG00000182389	CACNB4	2	151832776	152099079	protein_coding	7,57	0
ENSG00000130159	ECSIT	19	11505916	11529174	protein_coding	7,57	0
ENSG00000149177	PTPRJ	11	47980558	48170841	protein_coding	7,56	0
ENSG00000101966	XIAP	X	123859724	123913979	protein_coding	7,56	0
ENSG00000162129	CLPB	11	72292425	72434680	protein_coding	7,56	0
ENSG00000171466	ZNF562	19	9641808	9675086	protein_coding	7,56	0
ENSG00000196363	WDR5	9	134135365	134159968	protein_coding	7,55	0
ENSG00000274070	GATSL2	7	74964818	75024798	protein_coding	7,55	0
ENSG00000092929	UNC13D	17	75827225	75844717	protein_coding	7,55	0
ENSG00000135482	ZC3H10	12	56118159	56127514	protein_coding	7,55	0
ENSG00000070366	SMG6	17	2059839	2303771	protein_coding	7,54	0
ENSG00000164073	MFSD8	4	127917805	127965995	protein_coding	7,54	0

ENSG00000163527	STT3B	3	31532638	31637622	protein_coding	7,53	0
ENSG00000144827	ABHD10	3	111979010	111993363	protein_coding	7,52	0
ENSG00000129636	ITFG1	16	47154387	47464149	protein_coding	7,52	0
ENSG00000112210	RAB23	6	57186992	57222314	protein_coding	7,51	0
ENSG00000186141	POLR3C	1	145824088	145842505	protein_coding	7,51	0
ENSG00000144635	DYNC1LI1	3	32525971	32570874	protein_coding	7,50	0
ENSG00000188549	C15orf52	15	40331452	40340967	protein_coding	7,50	0
ENSG00000137747	TMPRSS13	11	117900643	117929459	protein_coding	7,50	0
ENSG00000145777	TSLP	5	111070062	111078024	protein_coding	7,50	0
ENSG00000144227	NXPH2	2	138670772	138780348	protein_coding	7,50	0
ENSG00000164916	FOXK1	7	4682309	4771443	protein_coding	7,49	0
ENSG00000106080	FKBP14	7	30010587	30026684	protein_coding	7,49	0
ENSG00000167333	TRIM68	11	4598672	4608259	protein_coding	7,48	0
ENSG00000109576	AADAT	4	170060222	170091699	protein_coding	7,47	0
ENSG00000144026	ZNF514	2	95147330	95165413	protein_coding	7,47	0
ENSG00000168175	MAPK1IP1L	14	55051631	55070192	protein_coding	7,47	0
ENSG00000197646	PDCD1LG2	9	5510570	5571254	protein_coding	7,46	0
ENSG00000158458	NRG2	5	139846779	140043299	protein_coding	7,45	0
ENSG00000164292	RHOBTB3	5	95713522	95824383	protein_coding	7,45	0
ENSG00000169057	MECP2	X	154021573	154137103	protein_coding	7,45	0
ENSG00000188725	SMIM15	5	61157709	61162474	protein_coding	7,44	0
ENSG00000147996	CBWD5	9	65668805	65734041	protein_coding	7,43	0
ENSG00000198700	IPO9	1	201829141	201884294	protein_coding	7,42	0
ENSG00000113716	HMGXB3	5	150000046	150053142	protein_coding	7,42	0
ENSG00000162517	PEF1	1	31629862	31644896	protein_coding	7,41	0
ENSG00000124160	NCOA5	20	46060985	46089952	protein_coding	7,40	0
ENSG00000198793	MTOR	1	11106535	11262507	protein_coding	7,40	0
ENSG00000117528	ABCD3	1	94418455	94518666	protein_coding	7,40	0
ENSG00000178226	PRSS36	16	31138925	31150094	protein_coding	7,40	0
ENSG00000260729	ENSG00000[illegible]	15	72284727	72375981	protein_coding	7,40	0
ENSG00000004838	ZMYND10	3	50341110	50346852	protein_coding	7,40	0
ENSG00000213085	CFAP45	1	159872364	159900163	protein_coding	7,40	0
ENSG00000122121	XPNPEP2	X	129738974	129769538	protein_coding	7,40	0
ENSG00000111962	UST	6	148747328	149076990	protein_coding	7,39	0
ENSG00000196453	ZNF777	7	149431363	149461123	protein_coding	7,38	0
ENSG00000077235	GTF3C1	16	27459555	27549913	protein_coding	7,38	0
ENSG00000160714	UBE2Q1	1	154548577	154559028	protein_coding	7,38	0
ENSG00000119682	AREL1	14	74653437	74713115	protein_coding	7,37	0
ENSG00000163291	PAQR3	4	78887127	78939438	protein_coding	7,37	0
ENSG00000136636	KCTD3	1	215567392	215621807	protein_coding	7,36	0
ENSG00000169180	XPO6	16	28097979	28211920	protein_coding	7,36	0
ENSG00000076067	RBMS2	12	56521929	56596196	protein_coding	7,35	0
ENSG00000180440	SERTM1	13	36673912	36697839	protein_coding	7,34	0
ENSG00000187676	B3GLCT	13	31199936	31332276	protein_coding	7,33	0
ENSG00000161940	BCL6B	17	7023020	7030290	protein_coding	7,33	0
ENSG00000179241	LDLRAD3	11	35943981	36232136	protein_coding	7,33	0
ENSG00000167635	ZNF146	19	36214602	36238774	protein_coding	7,33	0
ENSG00000204209	DAXX	6	33318558	33329269	protein_coding	7,32	0
ENSG00000242247	ARFGAP3	22	42796502	42858106	protein_coding	7,32	0
ENSG00000116977	LGALS8	1	236518000	236552981	protein_coding	7,32	0
ENSG00000058799	YIPF1	1	53851719	53889834	protein_coding	7,32	0
ENSG00000138443	ABI2	2	203328219	203447723	protein_coding	7,31	0
ENSG00000254598	CSNK2A3	11	11351942	11353357	protein_coding	7,31	0
ENSG00000124380	SNRNP27	2	69893560	69905575	protein_coding	7,31	0
ENSG00000179941	BBS10	12	76344474	76348442	protein_coding	7,31	0
ENSG00000099284	H2AFY2	10	70052796	70112280	protein_coding	7,31	0
ENSG00000163683	SMIM14	4	39546330	39639090	protein_coding	7,30	0
ENSG00000075240	GRAMD4	22	46576012	46679790	protein_coding	7,30	0

ENSG00000270149	ENSG00000[illegible]	1	160997957	161038962	protein_coding	7,30	0
ENSG00000185825	BCAP31	X	153700497	153724697	protein_coding	7,30	0
ENSG00000128191	DGCR8	22	20080232	20111877	protein_coding	7,30	0
ENSG00000054118	THRAP3	1	36224416	36305357	protein_coding	7,29	0
ENSG00000004700	RECQL	12	21468911	21501669	protein_coding	7,28	0
ENSG00000104375	STK3	8	98401403	98942827	protein_coding	7,28	0
ENSG00000141013	GAS8	16	90019629	90044975	protein_coding	7,28	0
ENSG00000197056	ZMYM1	1	35059786	35115859	protein_coding	7,28	0
ENSG00000167920	TMEM99	17	40819106	40836274	protein_coding	7,27	0
ENSG00000132002	DNAJB1	19	14514770	14529770	protein_coding	7,27	0
ENSG00000106125	FAM188B	7	30771417	30892387	protein_coding	7,27	0
ENSG00000138646	HERC5	4	88457117	88506163	protein_coding	7,27	0
ENSG00000148358	GPR107	9	130053426	130140169	protein_coding	7,27	0
ENSG00000119041	GTF3C3	2	196763032	196799725	protein_coding	7,26	0
ENSG00000129116	PALLD	4	168497066	168928457	protein_coding	7,26	0
ENSG00000145246	ATP10D	4	47485288	47593486	protein_coding	7,24	0
ENSG00000116786	PLEKHM2	1	15684332	15734769	protein_coding	7,24	0
ENSG00000113068	PFDN1	5	140245039	140303121	protein_coding	7,24	0
ENSG00000125434	SLC25A35	17	8287763	8295343	protein_coding	7,24	0
ENSG00000185619	PCGF3	4	705748	770640	protein_coding	7,24	0
ENSG00000088833	NSFL1C	20	1442162	1473842	protein_coding	7,23	0
ENSG00000147862	NFIB	9	14081843	14398983	protein_coding	7,23	0
ENSG00000174915	PTDSS2	11	448268	491399	protein_coding	7,23	0
ENSG00000251322	SHANK3	22	50674415	50733298	protein_coding	7,22	0
ENSG00000105982	RNF32	7	156640281	156677130	protein_coding	7,21	0
ENSG00000108506	INTS2	17	61865367	61928016	protein_coding	7,20	0
ENSG00000183160	TMEM119	12	108589846	108598320	protein_coding	7,20	0
ENSG00000184923	NUTM2A	10	87225448	87236908	protein_coding	7,20	0
ENSG00000227507	LTB	6	31580525	31582522	protein_coding	7,20	0
ENSG00000170837	GPR27	3	71754050	71756496	protein_coding	7,20	0
ENSG00000133639	BTG1	12	92140278	92145897	protein_coding	7,19	0
ENSG00000072071	ADGRL1	19	14147743	14206187	protein_coding	7,19	0
ENSG00000178297	TMPRSS9	19	2389771	2426239	protein_coding	7,18	0
ENSG00000066697	MSANTD3	9	100427156	100451711	protein_coding	7,17	0
ENSG00000065809	FAM107B	10	14518557	14774897	protein_coding	7,17	0
ENSG00000170537	TMC7	16	18983934	19063942	protein_coding	7,17	0
ENSG00000158470	B4GALT5	20	49632945	49713878	protein_coding	7,17	0
ENSG00000160194	NDUFV3	21	42879644	42913304	protein_coding	7,17	0
ENSG00000077721	UBE2A	X	119574467	119591083	protein_coding	7,17	0
ENSG00000163818	LZTFL1	3	45823316	45916042	protein_coding	7,16	0
ENSG00000128731	HERC2	15	28111040	28322152	protein_coding	7,15	0
ENSG00000127804	METTL16	17	2405562	2511891	protein_coding	7,15	0
ENSG00000171016	PYGO1	15	55538890	55588947	protein_coding	7,15	0
ENSG00000166816	LDHD	16	75111860	75116771	protein_coding	7,15	0
ENSG00000133422	MORC2	22	30925130	30968298	protein_coding	7,14	0
ENSG00000148429	USP6NL	10	11453946	11611754	protein_coding	7,14	0
ENSG00000204580	DDR1	6	30876421	30900156	protein_coding	7,14	0
ENSG00000144306	SCRN3	2	174395730	174429575	protein_coding	7,14	0
ENSG00000180917	CMTR2	16	71281389	71289715	protein_coding	7,14	0
ENSG00000184677	ZBTB40	1	22451851	22531157	protein_coding	7,13	0
ENSG00000153904	DDAH1	1	85318481	85578363	protein_coding	7,12	0
ENSG00000163904	SENP2	3	185582496	185633551	protein_coding	7,12	0
ENSG00000040487	PQLC2	1	19312326	19329300	protein_coding	7,12	0
ENSG00000104093	DMXL2	15	51447711	51622833	protein_coding	7,12	0
ENSG00000152782	PANK1	10	89579497	89645572	protein_coding	7,11	0
ENSG00000118849	RARRES1	3	158696892	158732696	protein_coding	7,10	0
ENSG00000204130	RUFY2	10	68341107	68407294	protein_coding	7,10	0
ENSG00000253598	SLC10A5	8	81693607	81696174	protein_coding	7,10	0

ENSG00000113205	PCDHB3	5	141100473	141103827	protein_coding	7,10	0
ENSG00000153563	CD8A	2	86784610	86808396	protein_coding	7,10	0
ENSG00000262180	OCLM	1	186400572	186401455	protein_coding	7,10	0
ENSG00000143870	PDIA6	2	10783391	10837977	protein_coding	7,10	0
ENSG00000145354	CISD2	4	102868978	102889242	protein_coding	7,10	0
ENSG00000100325	ASCC2	22	29788608	29838304	protein_coding	7,09	0
ENSG00000060688	SNRNP40	1	31259568	31296782	protein_coding	7,09	0
ENSG00000161048	NAPEPLD	7	103099776	103149560	protein_coding	7,09	0
ENSG00000155542	SETD9	5	56909260	56925532	protein_coding	7,08	0
ENSG00000122390	NAA60	16	3443611	3486963	protein_coding	7,08	0
ENSG00000198792	TMEM184B	22	38219291	38273034	protein_coding	7,07	0
ENSG00000122378	FAM213A	10	80407829	80437115	protein_coding	7,06	0
ENSG00000070047	PHRF1	11	576486	612222	protein_coding	7,06	0
ENSG00000034533	ASTE1	3	131013875	131027649	protein_coding	7,06	0
ENSG00000230124	ACBD6	1	180269653	180502954	protein_coding	7,06	0
ENSG00000134644	PUM1	1	30931506	31065991	protein_coding	7,06	0
ENSG00000197978	GOLGA6L9	15	82430018	82439153	protein_coding	7,06	0
ENSG00000172007	RAB33B	4	139453232	139476609	protein_coding	7,06	0
ENSG00000054967	RELT	11	73376264	73397474	protein_coding	7,05	0
ENSG00000105699	LSR	19	35248330	35267964	protein_coding	7,05	0
ENSG00000129351	ILF3	19	10654261	10692417	protein_coding	7,05	0
ENSG00000105887	MTPN	7	135926761	135977353	protein_coding	7,04	0
ENSG00000274349	ZNF658	9	66856426	66932141	protein_coding	7,04	0
ENSG00000111737	RAB35	12	120095095	120117502	protein_coding	7,03	0
ENSG00000163214	DHX57	2	38797729	38875934	protein_coding	7,03	0
ENSG00000174485	DENND4A	15	65658046	65792293	protein_coding	7,03	0
ENSG00000130254	SAFB2	19	5586999	5624046	protein_coding	7,03	0
ENSG00000109618	SEPSECS	4	25120014	25160442	protein_coding	7,02	0
ENSG00000148248	SURF4	9	133361449	133376166	protein_coding	7,01	0
ENSG00000103248	MTHFSD	16	86530176	86555235	protein_coding	7,00	0
ENSG00000128886	ELL3	15	43772600	43777543	protein_coding	7,00	0
ENSG00000139714	MORN3	12	121648742	121672631	protein_coding	7,00	0
ENSG00000146233	CYP39A1	6	46549580	46652830	protein_coding	7,00	0
ENSG00000224389	C4B	6	32014762	32035418	protein_coding	7,00	0
ENSG00000058600	POLR3E	16	22297375	22335103	protein_coding	7,00	0
ENSG00000140950	TLDC1	16	84476421	84554033	protein_coding	7,00	0
ENSG00000140750	ARHGAP17	16	24919385	25015666	protein_coding	7,00	0
ENSG00000015475	BID	22	17734138	17774770	protein_coding	6,99	0
ENSG00000137547	MRPL15	8	54135210	54147901	protein_coding	6,98	0
ENSG00000149212	SESN3	11	95165513	95232541	protein_coding	6,98	0
ENSG00000131653	TRAF7	16	2155698	2178129	protein_coding	6,98	0
ENSG00000131475	VPS25	17	42773436	42779599	protein_coding	6,97	0
ENSG00000135124	P2RX4	12	121209857	121234106	protein_coding	6,96	0
ENSG00000126945	HNRNPH2	X	101408295	101414133	protein_coding	6,96	0
ENSG00000001629	ANKIB1	7	92246234	92401384	protein_coding	6,96	0
ENSG00000169955	ZNF747	16	30530367	30535347	protein_coding	6,96	0
ENSG00000110497	AMBRA1	11	46396414	46594125	protein_coding	6,96	0
ENSG00000134897	BIVM	13	102799049	102841535	protein_coding	6,96	0
ENSG00000135457	TFCP2	12	51093663	51173134	protein_coding	6,95	0
ENSG00000134255	CEPT1	1	111139627	111185102	protein_coding	6,95	0
ENSG00000112309	B3GAT2	6	70856679	70957038	protein_coding	6,95	0
ENSG00000080298	RFX3	9	3218297	3526004	protein_coding	6,95	0
ENSG00000162613	FUBP1	1	77944055	77979110	protein_coding	6,94	0
ENSG00000119703	ZC2HC1C	14	75064170	75079987	protein_coding	6,94	0
ENSG00000135365	PHF21A	11	45929323	46121178	protein_coding	6,94	0
ENSG00000132541	HRSP12	8	98102344	98117241	protein_coding	6,92	0
ENSG00000186230	ZNF749	19	57435329	57445485	protein_coding	6,92	0
ENSG00000163719	MTMR14	3	9649433	9702394	protein_coding	6,91	0

ENSG00000128989	ARPP19	15	52547045	52569883	protein_coding	6,91	0
ENSG00000114859	CLCN2	3	184346185	184361651	protein_coding	6,91	0
ENSG00000151240	DIP2C	10	274190	689668	protein_coding	6,91	0
ENSG00000176261	ZBTB8OS	1	32600172	32650903	protein_coding	6,91	0
ENSG00000109083	IFT20	17	28328325	28335489	protein_coding	6,90	0
ENSG00000117069	ST6GALNAC!	1	76867441	77065711	protein_coding	6,90	0
ENSG00000118971	CCND2	12	4273772	4305350	protein_coding	6,90	0
ENSG00000182040	USH1G	17	74916084	74923256	protein_coding	6,90	0
ENSG00000243789	JMJD7	15	41828085	41837581	protein_coding	6,90	0
ENSG00000120500	ARR3	X	70268305	70281840	protein_coding	6,90	0
ENSG00000188269	OR7A5	19	14792490	14835376	protein_coding	6,89	0
ENSG00000235376	RPEL1	10	103245887	103248016	protein_coding	6,89	0
ENSG00000132004	FBXW9	19	12688053	12696643	protein_coding	6,89	0
ENSG00000196449	YRDC	1	37802944	37808185	protein_coding	6,88	0
ENSG00000092445	TYRO3	15	41557675	41583586	protein_coding	6,88	0
ENSG00000166822	TMEM170A	16	75443054	75465497	protein_coding	6,88	0
ENSG00000198746	GPATCH3	1	26890488	26900466	protein_coding	6,87	0
ENSG00000197024	ZNF398	7	149126416	149182802	protein_coding	6,86	0
ENSG00000134152	KATNBL1	15	34140674	34210096	protein_coding	6,86	0
ENSG00000099219	ERMP1	9	5765076	5833117	protein_coding	6,86	0
ENSG00000198521	ZNF43	19	21804949	21852125	protein_coding	6,86	0
ENSG00000163257	DCAF16	4	17800655	17810758	protein_coding	6,86	0
ENSG00000106683	LIMK1	7	74082933	74122525	protein_coding	6,86	0
ENSG00000100129	EIF3L	22	37848868	37889407	protein_coding	6,85	0
ENSG00000184432	COPB2	3	139355600	139389732	protein_coding	6,85	0
ENSG00000179029	TMEM107	17	8173237	8176399	protein_coding	6,84	0
ENSG00000158711	ELK4	1	205597556	205631962	protein_coding	6,84	0
ENSG00000167842	MIS12	17	5486285	5490814	protein_coding	6,84	0
ENSG00000165650	PDZD8	10	117277274	117375467	protein_coding	6,84	0
ENSG00000171456	ASXL1	20	32358344	32439319	protein_coding	6,83	0
ENSG00000122188	LAX1	1	203765176	203776233	protein_coding	6,83	0
ENSG00000186130	ZBTB6	9	122908056	122913330	protein_coding	6,83	0
ENSG00000172840	PDP2	16	66878589	66895754	protein_coding	6,83	0
ENSG00000049323	LTBP1	2	32946972	33399509	protein_coding	6,83	0
ENSG00000152749	GPR180	13	94601903	94634645	protein_coding	6,82	0
ENSG00000091656	ZFHX4	8	76681219	76867285	protein_coding	6,82	0
ENSG00000281527	ENSG00000２	8	116849884	116851073	protein_coding	6,82	0
ENSG00000108773	KAT2A	17	42113108	42121358	protein_coding	6,81	0
ENSG00000107140	TESK1	9	35605305	35610041	protein_coding	6,81	0
ENSG00000122912	SLC25A16	10	68477999	68527474	protein_coding	6,81	0
ENSG00000125686	MED1	17	39404285	39451286	protein_coding	6,81	0
ENSG00000181264	TMEM136	11	120325129	120333682	protein_coding	6,81	0
ENSG00000070540	WIPI1	17	68420948	68457513	protein_coding	6,80	0
ENSG00000078747	ITCH	20	34363235	34511393	protein_coding	6,80	0
ENSG00000125895	TMEM74B	20	1180561	1185415	protein_coding	6,80	0
ENSG00000166428	PLD4	14	104924816	104937790	protein_coding	6,80	0
ENSG00000276966	HIST1H4E	6	26204552	26206038	protein_coding	6,80	0
ENSG00000253159	PCDHGA12	5	141430589	141512979	protein_coding	6,80	0
ENSG00000254245	PCDHGA3	5	141343829	141512979	protein_coding	6,80	0
ENSG00000144063	MALL	2	110083870	110116566	protein_coding	6,80	0
ENSG00000136634	IL10	1	206767602	206772494	protein_coding	6,80	0
ENSG00000163154	TNFAIP8L2	1	151156629	151159749	protein_coding	6,80	0
ENSG00000281571	ENSG00000２	1	145214851	145215938	protein_coding	6,80	0
ENSG00000154945	ANKRD40	17	50693190	50707924	protein_coding	6,80	0
ENSG00000078269	SYNJ2	6	157981887	158099176	protein_coding	6,80	0
ENSG00000101745	ANKRD12	18	9136228	9285985	protein_coding	6,80	0
ENSG00000187792	ZNF70	22	23738678	23751092	protein_coding	6,80	0
ENSG00000133678	TMEM254	10	80078646	80092557	protein_coding	6,79	0

ENSG00000055917	PUM2	2	20248691	20352234	protein_coding	6,79	0
ENSG00000085832	EPS15	1	51354263	51519328	protein_coding	6,79	0
ENSG00000124593	PRICKLE4	6	41780349	41790141	protein_coding	6,79	0
ENSG00000100883	SRP54	14	34981957	35029567	protein_coding	6,79	0
ENSG00000175711	B3GNTL1	17	82942155	83051810	protein_coding	6,79	0
ENSG00000197694	SPTAN1	9	128552558	128633662	protein_coding	6,77	0
ENSG00000130338	TULP4	6	158232236	158511828	protein_coding	6,77	0
ENSG00000243477	NAT6	3	50296402	50299421	protein_coding	6,77	0
ENSG00000087008	ACOX3	4	8366282	8440723	protein_coding	6,77	0
ENSG00000128973	CLN6	15	68206992	68257211	protein_coding	6,76	0
ENSG00000127948	POR	7	75899200	75986855	protein_coding	6,76	0
ENSG00000116698	SMG7	1	183472216	183598246	protein_coding	6,76	0
ENSG00000131263	RLIM	X	74585217	74614617	protein_coding	6,75	0
ENSG00000108587	GOSR1	17	30477362	30527592	protein_coding	6,75	0
ENSG00000116922	C1orf109	1	37681570	37692249	protein_coding	6,75	0
ENSG00000179119	SPTY2D1	11	18606401	18634791	protein_coding	6,75	0
ENSG00000188493	C19orf54	19	40740856	40751553	protein_coding	6,74	0
ENSG00000160131	VMA21	X	151396515	151409364	protein_coding	6,74	0
ENSG00000214944	ARHGEF28	5	73626158	73941993	protein_coding	6,74	0
ENSG00000159842	ABR	17	1003518	1229021	protein_coding	6,74	0
ENSG00000241127	YAE1D1	7	39566376	39610320	protein_coding	6,74	0
ENSG00000099953	MMP11	22	23768226	23784316	protein_coding	6,73	0
ENSG00000179299	NSUN7	4	40749897	40809985	protein_coding	6,73	0
ENSG00000073536	NLE1	17	35128753	35142315	protein_coding	6,73	0
ENSG00000196850	PPTC7	12	110533245	110583320	protein_coding	6,73	0
ENSG00000188710	QRFP	9	130892702	130896812	protein_coding	6,73	0
ENSG00000134508	CABLES1	18	23134564	23260467	protein_coding	6,73	0
ENSG00000159140	SON	21	33542618	33577481	protein_coding	6,72	0
ENSG00000092199	HNRNPC	14	21209136	21269494	protein_coding	6,72	0
ENSG00000115241	PPM1G	2	27381194	27409687	protein_coding	6,72	0
ENSG00000154889	MPPE1	18	11882622	11909223	protein_coding	6,71	0
ENSG00000133030	MPRIP	17	17042545	17217679	protein_coding	6,71	0
ENSG00000112249	ASCC3	6	100508194	100881372	protein_coding	6,70	0
ENSG00000105398	SULT2A1	19	47870466	47886397	protein_coding	6,70	0
ENSG00000244731	C4A	6	31982024	32002681	protein_coding	6,70	0
ENSG00000117133	RPF1	1	84479259	84497790	protein_coding	6,70	0
ENSG00000182983	ZNF662	3	42905731	42917641	protein_coding	6,69	0
ENSG00000157827	FMNL2	2	152335237	152649834	protein_coding	6,68	0
ENSG00000145348	TBCK	4	106041599	106321495	protein_coding	6,68	0
ENSG00000166128	RAB8B	15	63189469	63267782	protein_coding	6,68	0
ENSG00000140403	DNAJA4	15	78264086	78282196	protein_coding	6,68	0
ENSG00000167220	HDHD2	18	47107403	47150520	protein_coding	6,68	0
ENSG00000158042	MRPL17	11	6680782	6683401	protein_coding	6,68	0
ENSG00000125772	GPCPD1	20	5544404	5611026	protein_coding	6,68	0
ENSG00000198718	FAM179B	14	44962208	45074431	protein_coding	6,67	0
ENSG00000280550	ENSG00000	11	33073925	33074214	protein_coding	6,67	0
ENSG00000189159	HN1	17	75135248	75168281	protein_coding	6,66	0
ENSG00000107863	ARHGAP21	10	24583609	24723668	protein_coding	6,66	0
ENSG00000163872	YEATS2	3	183697818	183812625	protein_coding	6,66	0
ENSG00000185689	C6orf201	6	4079209	4130951	protein_coding	6,66	0
ENSG00000125124	BBS2	16	56466836	56520283	protein_coding	6,65	0
ENSG00000011258	MBTD1	17	51177425	51260163	protein_coding	6,65	0
ENSG00000108946	PRKAR1A	17	68511780	68551319	protein_coding	6,65	0
ENSG00000100949	RABGGTA	14	24265538	24271739	protein_coding	6,64	0
ENSG00000156599	ZDHHC5	11	57667747	57701187	protein_coding	6,64	0
ENSG00000143924	EML4	2	42169350	42332548	protein_coding	6,64	0
ENSG00000165972	CCDC38	12	95867048	95942974	protein_coding	6,64	0
ENSG00000074201	CLNS1A	11	77514936	77637805	protein_coding	6,64	0

ENSG00000170271	FAXDC2	5	154818491	154859252	protein_coding	6,63	0
ENSG00000135974	C2orf49	2	105337359	105349211	protein_coding	6,62	0
ENSG00000155363	MOV10	1	112673141	112700746	protein_coding	6,62	0
ENSG00000110911	SLC11A2	12	50979401	51028566	protein_coding	6,62	0
ENSG00000152223	EPG5	18	45847609	45967274	protein_coding	6,61	0
ENSG00000167986	DDB1	11	61299451	61342596	protein_coding	6,61	0
ENSG00000148225	WDR31	9	113313222	113340298	protein_coding	6,61	0
ENSG00000100079	LGALS2	22	37570246	37582616	protein_coding	6,60	0
ENSG00000136999	NOV	8	119416306	119424353	protein_coding	6,60	0
ENSG00000124098	FAM210B	20	56358915	56368663	protein_coding	6,60	0
ENSG00000117151	CTBS	1	84549606	84574480	protein_coding	6,58	0
ENSG00000110011	DNAJC4	11	64230278	64234286	protein_coding	6,58	0
ENSG00000121864	ZNF639	3	179322991	179338583	protein_coding	6,58	0
ENSG00000205531	NAP1L4	11	2944431	2992377	protein_coding	6,58	0
ENSG00000197905	TEAD4	12	2959330	3040673	protein_coding	6,58	0
ENSG00000132382	MYBBP1A	17	4538897	4555631	protein_coding	6,57	0
ENSG00000129347	KRI1	19	10553078	10566037	protein_coding	6,57	0
ENSG00000120519	SLC10A7	4	146253975	146521964	protein_coding	6,57	0
ENSG00000114779	ABHD14B	3	51968510	51983409	protein_coding	6,57	0
ENSG00000122958	VPS26A	10	69123512	69172861	protein_coding	6,57	0
ENSG00000272325	NUDT3	6	34279679	34392674	protein_coding	6,56	0
ENSG00000123213	NLN	5	65722196	65871725	protein_coding	6,56	0
ENSG00000118620	ZNF430	19	21020620	21060050	protein_coding	6,56	0
ENSG00000196792	STRN3	14	30893799	31026401	protein_coding	6,55	0
ENSG00000081019	RSBN1	1	113761832	113812476	protein_coding	6,54	0
ENSG00000221944	TIGD1	2	232547968	232550592	protein_coding	6,54	0
ENSG00000072954	TMEM38A	19	16661127	16690029	protein_coding	6,54	0
ENSG00000151729	SLC25A4	4	185143241	185150382	protein_coding	6,53	0
ENSG00000086189	DIMT1	5	62387254	62403939	protein_coding	6,53	0
ENSG00000064393	HIPK2	7	139561570	139777778	protein_coding	6,53	0
ENSG00000069345	DNAJA2	16	46955362	46973788	protein_coding	6,53	0
ENSG00000070476	ZXDC	3	126437601	126475919	protein_coding	6,52	0
ENSG00000076685	NT5C2	10	103088017	103193306	protein_coding	6,52	0
ENSG00000164172	MOCS2	5	53095679	53110063	protein_coding	6,51	0
ENSG00000132963	POMP	13	28659104	28678925	protein_coding	6,51	0
ENSG00000146834	MEPCE	7	100428790	100434126	protein_coding	6,51	0
ENSG00000166086	JAM3	11	134068925	134152001	protein_coding	6,51	0
ENSG00000100804	PSMB5	14	23016543	23035230	protein_coding	6,51	0
ENSG00000185942	NKAIN3	8	62248591	62999652	protein_coding	6,50	0
ENSG00000260097	SPDYE6	7	102347206	102356444	protein_coding	6,50	0
ENSG00000085733	CTTN	11	70398404	70436584	protein_coding	6,49	0
ENSG00000134830	C5AR2	19	47332147	47347327	protein_coding	6,48	0
ENSG00000137642	SORL1	11	121452203	121633693	protein_coding	6,47	0
ENSG00000167562	ZNF701	19	52555822	52587174	protein_coding	6,47	0
ENSG00000158467	AHCYL2	7	129225023	129430211	protein_coding	6,47	0
ENSG00000047597	XK	X	37685759	37732130	protein_coding	6,46	0
ENSG00000105948	TTC26	7	139133744	139191986	protein_coding	6,46	0
ENSG00000088179	PTPN4	2	119759631	119983818	protein_coding	6,45	0
ENSG00000105738	SIPA1L3	19	37907228	38208372	protein_coding	6,44	0
ENSG00000161036	LRWD1	7	102464929	102473168	protein_coding	6,44	0
ENSG00000074527	NTN4	12	95657807	95791152	protein_coding	6,44	0
ENSG00000166974	MAPRE2	18	34976928	35143470	protein_coding	6,44	0
ENSG00000185591	SP1	12	53380176	53416446	protein_coding	6,43	0
ENSG00000269693	ENSG000002	19	12525720	12580975	protein_coding	6,42	0
ENSG00000161981	SNRNP25	16	53010	57669	protein_coding	6,42	0
ENSG00000114861	FOXP1	3	70954693	71583989	protein_coding	6,40	0
ENSG00000175581	MRPL48	11	73787316	73865133	protein_coding	6,40	0
ENSG00000170522	ELOVL6	4	110045846	110199199	protein_coding	6,39	0

ENSG00000186448	ZNF197	3	44584888	44648471	protein_coding	6,39	0
ENSG00000135336	ORC3	6	87590067	87667453	protein_coding	6,39	0
ENSG00000142546	NOSIP	19	49555711	49590262	protein_coding	6,39	0
ENSG00000119231	SENP5	3	196867856	196934714	protein_coding	6,39	0
ENSG00000102901	CENPT	16	67828157	67847811	protein_coding	6,39	0
ENSG00000053371	AKR7A2	1	19303965	19312146	protein_coding	6,38	0
ENSG00000130935	NOL11	17	67717833	67744531	protein_coding	6,38	0
ENSG00000110651	CD81	11	2376177	2397419	protein_coding	6,38	0
ENSG00000266412	NCOA4	10	46005088	46030714	protein_coding	6,37	0
ENSG00000180488	FAM73A	1	77779624	77879539	protein_coding	6,37	0
ENSG00000151835	SACS	13	23328823	23433728	protein_coding	6,37	0
ENSG00000151292	CSNK1G3	5	123512099	123617045	protein_coding	6,37	0
ENSG00000163576	EFHB	3	19879472	19947025	protein_coding	6,36	0
ENSG00000189308	LIN54	4	82909973	83012926	protein_coding	6,36	0
ENSG00000204070	SYS1	20	45361937	45376798	protein_coding	6,36	0
ENSG00000171823	FBXL14	12	1565993	1594165	protein_coding	6,36	0
ENSG00000164048	ZNF589	3	48241100	48299253	protein_coding	6,36	0
ENSG00000154485	MMP21	10	125766453	125775821	protein_coding	6,36	0
ENSG00000137693	YAP1	11	102110461	102233423	protein_coding	6,36	0
ENSG00000114125	RNF7	3	141738204	141747560	protein_coding	6,36	0
ENSG00000118961	LDAH	2	20684014	20823130	protein_coding	6,35	0
ENSG00000028137	TNFRSF1B	1	12167003	12209228	protein_coding	6,35	0
ENSG00000159147	DONSON	21	33559542	33588708	protein_coding	6,35	0
ENSG00000171847	FAM90A1	12	8221260	8227618	protein_coding	6,35	0
ENSG00000139505	MTMR6	13	25246201	25288009	protein_coding	6,35	0
ENSG00000050426	LETMD1	12	51047962	51060424	protein_coding	6,35	0
ENSG00000109814	UGDH	4	39498755	39528311	protein_coding	6,34	0
ENSG00000094916	CBX5	12	54230940	54280133	protein_coding	6,33	0
ENSG00000165533	TTC8	14	88824153	88881078	protein_coding	6,32	0
ENSG00000142864	SERBP1	1	67407810	67430415	protein_coding	6,32	0
ENSG00000105619	TFPT	19	54107013	54115675	protein_coding	6,32	0
ENSG00000077254	USP33	1	77695987	77759852	protein_coding	6,32	0
ENSG00000188501	LCTL	15	66547179	66565979	protein_coding	6,32	0
ENSG00000213593	TMX2	11	57712600	57740973	protein_coding	6,32	0
ENSG00000101442	ACTR5	20	38748442	38772520	protein_coding	6,31	0
ENSG00000116489	CAPZA1	1	112619173	112671619	protein_coding	6,31	0
ENSG00000160216	AGPAT3	21	43865186	43986536	protein_coding	6,31	0
ENSG00000131725	WDR44	X	118346073	118449961	protein_coding	6,31	0
ENSG00000127995	CASD1	7	94509219	94557019	protein_coding	6,30	0
ENSG00000154328	NEIL2	8	11769639	11787346	protein_coding	6,30	0
ENSG00000175229	GAL3ST3	11	66041952	66049180	protein_coding	6,30	0
ENSG00000187486	KCNJ11	11	17385859	17389331	protein_coding	6,30	0
ENSG00000204967	PCDHA4	5	140806929	141012344	protein_coding	6,30	0
ENSG00000147548	WHSC1L1	8	38269697	38382272	protein_coding	6,30	0
ENSG00000162430	SEPN1	1	25800176	25818224	protein_coding	6,30	0
ENSG00000197170	PSMD12	17	67337916	67366627	protein_coding	6,30	0
ENSG00000189067	LITAF	16	11547722	11636381	protein_coding	6,30	0
ENSG00000175182	FAM131A	3	184335926	184346275	protein_coding	6,30	0
ENSG00000068796	KIF2A	5	62306162	62537249	protein_coding	6,28	0
ENSG00000184979	USP18	22	18149899	18177397	protein_coding	6,28	0
ENSG00000168538	TRAPPC11	4	183659267	183713594	protein_coding	6,27	0
ENSG00000185101	ANO9	11	417933	442011	protein_coding	6,27	0
ENSG00000169895	SYAP1	X	16719632	16765336	protein_coding	6,27	0
ENSG00000021762	OSBPL5	11	3087116	3166739	protein_coding	6,27	0
ENSG00000159363	ATP13A2	1	16985958	17011928	protein_coding	6,27	0
ENSG00000160785	SLC25A44	1	156193932	156212796	protein_coding	6,26	0
ENSG00000128595	CALU	7	128739292	128771807	protein_coding	6,26	0
ENSG00000185842	DNAH14	1	224896262	225399292	protein_coding	6,25	0

ENSG00000196268	ZNF493	19	21397119	21427573	protein_coding	6,25	0
ENSG00000104177	MYEF2	15	48134631	48178517	protein_coding	6,25	0
ENSG00000205352	PRR13	12	53441605	53446645	protein_coding	6,25	0
ENSG00000115317	HTRA2	2	74529377	74533348	protein_coding	6,25	0
ENSG00000157593	SLC35B2	6	44254096	44257890	protein_coding	6,24	0
ENSG00000138629	UBL7	15	74445977	74461182	protein_coding	6,24	0
ENSG00000087470	DNM1L	12	32679200	32745650	protein_coding	6,24	0
ENSG00000123080	CDKN2C	1	50960745	50974633	protein_coding	6,24	0
ENSG00000198276	UCKL1	20	63939829	63956415	protein_coding	6,24	0
ENSG00000080822	CLDND1	3	98497912	98523066	protein_coding	6,23	0
ENSG00000135720	DYNC1LI2	16	66720893	66751798	protein_coding	6,23	0
ENSG00000139644	TMBIM6	12	49707725	49764934	protein_coding	6,23	0
ENSG00000174444	RPL4	15	66498015	66524532	protein_coding	6,23	0
ENSG00000151276	MAGI1	3	65353525	66038834	protein_coding	6,23	0
ENSG00000125520	SLC2A4RG	20	63739861	63743505	protein_coding	6,22	0
ENSG00000113384	GOLPH3	5	32124704	32174350	protein_coding	6,22	0
ENSG00000106344	RBM28	7	128297685	128343908	protein_coding	6,22	0
ENSG00000117143	UAP1	1	162561506	162599842	protein_coding	6,22	0
ENSG00000213918	DNASE1	16	3611728	3680143	protein_coding	6,21	0
ENSG00000196378	ZNF34	8	144773114	144787345	protein_coding	6,21	0
ENSG00000157540	DYRK1A	21	37365790	37517450	protein_coding	6,21	0
ENSG00000131558	EXOC4	7	133253073	134066589	protein_coding	6,21	0
ENSG00000174804	FZD4	11	86945679	86955391	protein_coding	6,21	0
ENSG00000129009	ISLR	15	74173671	74176872	protein_coding	6,20	0
ENSG00000186399	GOLGA8R	15	30403740	30414162	protein_coding	6,20	0
ENSG00000215712	TMEM242	6	157289386	157323601	protein_coding	6,19	0
ENSG00000213626	LBH	2	30231531	30323730	protein_coding	6,19	0
ENSG00000134900	TPP2	13	102597003	102679958	protein_coding	6,19	0
ENSG00000160193	WDR4	21	42843094	42879568	protein_coding	6,19	0
ENSG00000165240	ATP7A	X	77910656	78050395	protein_coding	6,19	0
ENSG00000166411	IDH3A	15	78131498	78171949	protein_coding	6,18	0
ENSG00000188158	NHS	X	17375420	17735994	protein_coding	6,18	0
ENSG00000137871	ZNF280D	15	56630181	56918571	protein_coding	6,18	0
ENSG00000102858	MGRN1	16	4616493	4690974	protein_coding	6,18	0
ENSG00000015153	YAF2	12	42157104	42238349	protein_coding	6,18	0
ENSG00000159592	GPBP1L1	1	45627304	45688113	protein_coding	6,18	0
ENSG00000161057	PSMC2	7	103344254	103369395	protein_coding	6,17	0
ENSG00000145725	PPIP5K2	5	103120149	103212799	protein_coding	6,17	0
ENSG00000182362	YBEY	21	46286337	46297751	protein_coding	6,17	0
ENSG00000178057	NDUFAF3	3	49020459	49023495	protein_coding	6,17	0
ENSG00000137414	FAM8A1	6	17600355	17611719	protein_coding	6,17	0
ENSG00000185869	ZNF829	19	36888124	36916291	protein_coding	6,17	0
ENSG00000107798	LIPA	10	89213569	89414557	protein_coding	6,17	0
ENSG00000161298	ZNF382	19	36604817	36634113	protein_coding	6,17	0
ENSG00000188227	ZNF793	19	37506939	37548762	protein_coding	6,16	0
ENSG00000117569	PTBP2	1	96721665	96823738	protein_coding	6,16	0
ENSG00000088832	FKBP1A	20	1368978	1393172	protein_coding	6,16	0
ENSG00000135845	PIGC	1	172370189	172444086	protein_coding	6,15	0
ENSG00000188295	ZNF669	1	247099962	247104372	protein_coding	6,15	0
ENSG00000089916	GPATCH2L	14	76151916	76254342	protein_coding	6,15	0
ENSG00000097096	SYDE2	1	85156873	85201046	protein_coding	6,15	0
ENSG00000128908	INO80	15	40978880	41116354	protein_coding	6,15	0
ENSG00000121644	DESI2	1	244652935	244709033	protein_coding	6,15	0
ENSG00000119596	YLPM1	14	74763366	74859435	protein_coding	6,14	0
ENSG00000137776	SLTM	15	58879045	58933653	protein_coding	6,14	0
ENSG00000137274	BPHL	6	3118374	3153578	protein_coding	6,14	0
ENSG00000129696	TTI2	8	33473386	33513601	protein_coding	6,14	0
ENSG00000115310	RTN4	2	54972187	55112621	protein_coding	6,14	0

ENSG00000185278	ZBTB37	1	173868082	173903549	protein_coding	6,14	0
ENSG00000187118	CMC1	3	28241584	28325142	protein_coding	6,13	0
ENSG00000258289	CHURC1	14	64914361	64944591	protein_coding	6,12	0
ENSG00000187554	TLR5	1	223109406	223143282	protein_coding	6,12	0
ENSG00000198707	CEP290	12	88049014	88142216	protein_coding	6,12	0
ENSG00000196793	ZNF239	10	43556344	43574618	protein_coding	6,11	0
ENSG00000176095	IP6K1	3	49724294	49786542	protein_coding	6,10	0
ENSG00000160294	MCM3AP	21	46235126	46286297	protein_coding	6,10	0
ENSG00000152213	ARL11	13	49628299	49633872	protein_coding	6,10	0
ENSG00000213204	ENSG000002	6	87408012	87511634	protein_coding	6,10	0
ENSG00000163993	S100P	4	6693069	6697170	protein_coding	6,10	0
ENSG00000125944	HNRNPR	1	23303771	23344336	protein_coding	6,10	0
ENSG00000115993	TRAK2	2	201377207	201451611	protein_coding	6,09	0
ENSG00000151366	NDUFC2	11	78068304	78080219	protein_coding	6,08	0
ENSG00000092010	PSME1	14	24136158	24138967	protein_coding	6,08	0
ENSG00000132676	DAP3	1	155687960	155739010	protein_coding	6,08	0
ENSG00000088682	COQ9	16	57447425	57461275	protein_coding	6,08	0
ENSG00000173517	PEAK1	15	77100656	77420144	protein_coding	6,08	0
ENSG00000198728	LDB1	10	102107560	102120453	protein_coding	6,07	0
ENSG00000108389	MTMR4	17	58489529	58517905	protein_coding	6,06	0
ENSG00000162384	C1orf123	1	53214099	53220617	protein_coding	6,06	0
ENSG00000128829	EIF2AK4	15	39934146	40035591	protein_coding	6,06	0
ENSG00000133401	PDZD2	5	31639410	32110931	protein_coding	6,05	0
ENSG00000054523	KIF1B	1	10210805	10381603	protein_coding	6,05	0
ENSG00000177932	ZNF354C	5	179060415	179083537	protein_coding	6,05	0
ENSG00000138768	USO1	4	75724593	75814286	protein_coding	6,05	0
ENSG00000091164	TXNL1	18	56597208	56651600	protein_coding	6,05	0
ENSG00000197563	PIGN	18	62043567	62187118	protein_coding	6,04	0
ENSG00000101189	MRGBP	20	62796453	62801738	protein_coding	6,02	0
ENSG00000198586	TLK1	2	170990823	171231314	protein_coding	6,01	0
ENSG00000135999	EPC2	2	148644440	148787568	protein_coding	6,01	0
ENSG00000186260	MKL2	16	14071321	14266773	protein_coding	6,01	0
ENSG00000107789	MINPP1	10	87504875	87553460	protein_coding	6,01	0
ENSG00000141279	NPEPPS	17	47522942	47623276	protein_coding	6,00	0
ENSG00000130943	PKDREJ	22	46255663	46263355	protein_coding	6,00	0
ENSG00000180881	CAPS2	12	75275979	75390928	protein_coding	6,00	0
ENSG00000143740	SNAP47	1	227728539	227781231	protein_coding	6,00	0
ENSG00000139926	FRMD6	14	51489100	51730727	protein_coding	5,99	0
ENSG00000171448	ZBTB26	9	122915566	122931500	protein_coding	5,99	0
ENSG00000162377	COA7	1	52684451	52698366	protein_coding	5,99	0
ENSG00000102931	ARL2BP	16	57245098	57253635	protein_coding	5,99	0
ENSG00000114316	USP4	3	49277831	49340712	protein_coding	5,97	0
ENSG00000105221	AKT2	19	40230317	40285536	protein_coding	5,97	0
ENSG00000063978	RNF4	4	2462220	2625320	protein_coding	5,97	0
ENSG00000084731	KIF3C	2	25926596	25982749	protein_coding	5,96	0
ENSG00000157036	EXOG	3	38496127	38542161	protein_coding	5,96	0
ENSG00000139219	COL2A1	12	47972965	48004486	protein_coding	5,95	0
ENSG00000126777	KTN1	14	55559072	55701526	protein_coding	5,95	0
ENSG00000126088	UROD	1	45012147	45015575	protein_coding	5,95	0
ENSG00000254726	MEX3A	1	156072013	156081998	protein_coding	5,95	0
ENSG00000164061	BSN	3	49554489	49671545	protein_coding	5,95	0
ENSG00000204560	DHX16	6	30653119	30673037	protein_coding	5,94	0
ENSG00000265241	RBM8A	1	145917714	145927678	protein_coding	5,94	0
ENSG00000249115	HAUS5	19	35612744	35625349	protein_coding	5,93	0
ENSG00000184436	THAP7	22	20999104	21002196	protein_coding	5,93	0
ENSG00000011260	UTP18	17	51260528	51297936	protein_coding	5,92	0
ENSG00000169288	MRPL1	4	77862520	77952790	protein_coding	5,92	0
ENSG00000154978	VOPP1	7	55436056	55572988	protein_coding	5,92	0

ENSG00000148634	HERC4	10	67921899	68075348	protein_coding	5,91	0
ENSG00000137845	ADAM10	15	58588807	58749978	protein_coding	5,91	0
ENSG00000134371	CDC73	1	193122017	193253901	protein_coding	5,91	0
ENSG00000173402	DAG1	3	49468703	49535618	protein_coding	5,91	0
ENSG00000186994	KANK3	19	8322584	8343262	protein_coding	5,91	0
ENSG00000181027	FKRP	19	46746046	46776988	protein_coding	5,91	0
ENSG00000141456	PELP1	17	4669774	4704337	protein_coding	5,91	0
ENSG00000101349	PAK7	20	9537389	9839041	protein_coding	5,90	0
ENSG00000261582	ENSG000002	20	35267885	35280043	protein_coding	5,90	0
ENSG00000172954	LCLAT1	2	30447226	30644225	protein_coding	5,90	0
ENSG00000188177	ZC3H6	2	112275594	112340063	protein_coding	5,90	0
ENSG00000177565	TBL1XR1	3	177019355	177197396	protein_coding	5,90	0
ENSG00000163807	KIAA1143	3	44737661	44761662	protein_coding	5,89	0
ENSG00000198382	UVRAG	11	75815167	76143195	protein_coding	5,89	0
ENSG00000178234	GALNT11	7	152025674	152122347	protein_coding	5,89	0
ENSG00000131473	ACLY	17	41866908	41930542	protein_coding	5,89	0
ENSG00000147274	RBMX	X	136848004	136880764	protein_coding	5,89	0
ENSG00000145916	RMND5B	5	178130996	178150565	protein_coding	5,89	0
ENSG00000166169	POLL	10	101578882	101588270	protein_coding	5,89	0
ENSG00000058063	ATP11B	3	182793500	182921635	protein_coding	5,88	0
ENSG00000138386	NAB1	2	190646746	190692766	protein_coding	5,87	0
ENSG00000170417	TMEM182	2	102736908	102843893	protein_coding	5,87	0
ENSG00000256683	ZNF350	19	51964343	51986856	protein_coding	5,87	0
ENSG00000073578	SDHA	5	218241	256700	protein_coding	5,87	0
ENSG00000116514	RNF19B	1	32936445	32964685	protein_coding	5,87	0
ENSG00000181220	ZNF746	7	149472794	149497817	protein_coding	5,85	0
ENSG00000048991	R3HDM1	2	135531455	135725270	protein_coding	5,85	0
ENSG00000188167	TMPPE	3	33090421	33096801	protein_coding	5,85	0
ENSG00000149380	P4HA3	11	74235801	74311657	protein_coding	5,84	0
ENSG00000185414	MRPL30	2	99181079	99197626	protein_coding	5,84	0
ENSG00000132182	NUP210	3	13316235	13420309	protein_coding	5,84	0
ENSG00000213516	RBMXL1	1	88979456	88992960	protein_coding	5,84	0
ENSG00000170191	NANP	20	25612935	25624175	protein_coding	5,84	0
ENSG00000175764	TTLL11	9	121821928	122093606	protein_coding	5,83	0
ENSG00000157510	AFAP1L1	5	149271871	149341802	protein_coding	5,83	0
ENSG00000144848	ATG3	3	112532509	112562046	protein_coding	5,83	0
ENSG00000099246	RAB18	10	27504174	27542237	protein_coding	5,83	0
ENSG00000115685	PPP1R7	2	241149576	241183652	protein_coding	5,83	0
ENSG00000090432	MUL1	1	20499448	20508161	protein_coding	5,83	0
ENSG00000092203	TOX4	14	21476597	21499175	protein_coding	5,82	0
ENSG00000159346	ADIPOR1	1	202940823	202958572	protein_coding	5,82	0
ENSG00000138119	MYOF	10	93306429	93482317	protein_coding	5,82	0
ENSG00000083896	YTHDC1	4	68310387	68350089	protein_coding	5,82	0
ENSG00000130726	TRIM28	19	58544091	58550722	protein_coding	5,82	0
ENSG00000139921	TMX1	14	51240162	51257546	protein_coding	5,81	0
ENSG00000100316	RPL3	22	39312882	39320389	protein_coding	5,81	0
ENSG00000146411	SLC2A12	6	133988697	134052636	protein_coding	5,81	0
ENSG00000158321	AUTS2	7	69598919	70793068	protein_coding	5,81	0
ENSG00000135316	SYNCRIP	6	85607785	85643792	protein_coding	5,81	0
ENSG00000048471	SNX29	16	11976737	12574289	protein_coding	5,81	0
ENSG00000156508	EEF1A1	6	73515750	73523797	protein_coding	5,81	0
ENSG00000150907	FOXO1	13	40555667	40666597	protein_coding	5,80	0
ENSG00000100078	PLA2G3	22	31134809	31140607	protein_coding	5,80	0
ENSG00000269533	ENSG000002	19	57389884	57419994	protein_coding	5,80	0
ENSG00000271503	CCL5	17	35871491	35880793	protein_coding	5,80	0
ENSG00000186583	SPATC1	8	144012414	144047085	protein_coding	5,80	0
ENSG00000114812	VIPR1	3	42489299	42537573	protein_coding	5,80	0
ENSG00000173436	MINOS1	1	19596977	19629821	protein_coding	5,80	0

ENSG00000111237	VPS29	12	110491097	110502117	protein_coding	5,80	0
ENSG00000121289	CEP89	19	32875925	32971991	protein_coding	5,79	0
ENSG00000164975	SNAPC3	9	15422704	15465953	protein_coding	5,79	0
ENSG00000092140	G2E3	14	30559123	30620063	protein_coding	5,78	0
ENSG00000206535	LNP1	3	100401193	100456319	protein_coding	5,78	0
ENSG00000164037	SLC9B1	4	102885048	103019739	protein_coding	5,78	0
ENSG00000120053	GOT1	10	99396870	99430624	protein_coding	5,78	0
ENSG00000006831	ADIPOR2	12	1688574	1788678	protein_coding	5,78	0
ENSG00000159445	THEM4	1	151873584	151909808	protein_coding	5,78	0
ENSG00000182400	TRAPPC6B	14	39147811	39170532	protein_coding	5,77	0
ENSG00000143756	FBXO28	1	224114087	224162047	protein_coding	5,77	0
ENSG00000163714	U2SURP	3	142964497	143060546	protein_coding	5,77	0
ENSG00000065135	GNAI3	1	109548611	109618321	protein_coding	5,76	0
ENSG00000171574	ZNF584	19	58401504	58418327	protein_coding	5,76	0
ENSG00000183087	GAS6	13	113820549	113864067	protein_coding	5,76	0
ENSG00000183309	ZNF623	8	143636013	143656418	protein_coding	5,76	0
ENSG00000171877	FRMD5	15	43870761	44195252	protein_coding	5,76	0
ENSG00000155506	LARP1	5	154712902	154817607	protein_coding	5,75	0
ENSG00000167555	ZNF528	19	52397849	52418412	protein_coding	5,75	0
ENSG00000102189	EEA1	12	92770637	92929331	protein_coding	5,75	0
ENSG00000137497	NUMA1	11	72002864	72080693	protein_coding	5,74	0
ENSG00000090686	USP48	1	21678298	21783606	protein_coding	5,73	0
ENSG00000158793	NIT1	1	161118086	161125445	protein_coding	5,73	0
ENSG00000126953	TIMM8A	X	101345661	101349196	protein_coding	5,73	0
ENSG00000110274	CEP164	11	117314557	117413268	protein_coding	5,73	0
ENSG00000119411	BSPRY	9	113349541	113371233	protein_coding	5,73	0
ENSG00000143549	TPM3	1	154155304	154194648	protein_coding	5,73	0
ENSG00000119335	SET	9	128683424	128696400	protein_coding	5,72	0
ENSG00000183287	CCBE1	18	59430940	59697380	protein_coding	5,71	0
ENSG00000144535	DIS3L2	2	231961245	232344350	protein_coding	5,71	0
ENSG00000171608	PIK3CD	1	9651732	9729114	protein_coding	5,71	0
ENSG00000182247	UBE2E2	3	23203020	23591793	protein_coding	5,71	0
ENSG00000148814	LRRC27	10	132332154	132379918	protein_coding	5,71	0
ENSG00000104723	TUSC3	8	15417215	15766649	protein_coding	5,71	0
ENSG00000106733	NMRK1	9	75060573	75088217	protein_coding	5,70	0
ENSG00000168883	USP39	2	85602856	85649282	protein_coding	5,70	0
ENSG00000162521	RBBP4	1	32651142	32686211	protein_coding	5,70	0
ENSG00000155097	ATP6V1C1	8	103021063	103073051	protein_coding	5,70	0
ENSG00000127838	PNKD	2	218270392	218346793	protein_coding	5,69	0
ENSG00000117016	RIMS3	1	40620679	40665657	protein_coding	5,69	0
ENSG00000135655	USP15	12	62260338	62417431	protein_coding	5,69	0
ENSG00000170954	ZNF415	19	53107879	53133077	protein_coding	5,69	0
ENSG00000109184	DCUN1D4	4	51843000	51916837	protein_coding	5,69	0
ENSG00000123607	TTC21B	2	165857475	165953843	protein_coding	5,69	0
ENSG00000169504	CLIC4	1	24745357	24844324	protein_coding	5,68	0
ENSG00000157741	UBN2	7	139230356	139308236	protein_coding	5,68	0
ENSG00000145907	G3BP1	5	151771045	151812785	protein_coding	5,68	0
ENSG00000172845	SP3	2	173906459	173965702	protein_coding	5,67	0
ENSG00000167315	ACAA2	18	49782167	49813960	protein_coding	5,67	0
ENSG00000110031	LPXN	11	58526871	58578220	protein_coding	5,67	0
ENSG00000116005	PCYOX1	2	70257386	70281191	protein_coding	5,66	0
ENSG00000101407	TTI1	20	37983007	38033468	protein_coding	5,66	0
ENSG00000143479	DYRK3	1	206635536	206684419	protein_coding	5,66	0
ENSG00000198162	MAN1A2	1	117367449	117528872	protein_coding	5,66	0
ENSG00000121774	KHDRBS1	1	32013829	32060850	protein_coding	5,66	0
ENSG00000137474	MYO7A	11	77128264	77215239	protein_coding	5,66	0
ENSG00000153339	TRAPPC8	18	31829173	31953136	protein_coding	5,65	0
ENSG00000170142	UBE2E1	3	23805903	23891316	protein_coding	5,65	0

ENSG00000163625	WDFY3	4	84669610	84966391	protein_coding	5,65	0
ENSG00000029363	BCLAF1	6	136256863	136289851	protein_coding	5,64	0
ENSG00000162391	FAM151A	1	54609182	54623556	protein_coding	5,64	0
ENSG00000151065	DCP1B	12	1946054	2004535	protein_coding	5,63	0
ENSG00000083312	TNPO1	5	72816312	72916733	protein_coding	5,63	0
ENSG00000095066	HOOK2	19	12763003	12872740	protein_coding	5,63	0
ENSG00000180370	PAK2	3	196739857	196832647	protein_coding	5,63	0
ENSG00000108669	CYTH1	17	78674048	78782297	protein_coding	5,62	0
ENSG00000088247	KHSRP	19	6413348	6424794	protein_coding	5,61	0
ENSG00000155561	NUP205	7	135557919	135648757	protein_coding	5,61	0
ENSG00000275835	TUBGCP5	15	22983192	23039673	protein_coding	5,61	0
ENSG00000144445	KANSL1L	2	210021423	210171383	protein_coding	5,61	0
ENSG00000166260	COX11	17	54951902	54968785	protein_coding	5,60	0
ENSG00000005189	ENSG00000(	16	20806429	20849668	protein_coding	5,60	0
ENSG00000137878	GCOM1	15	57591908	57714745	protein_coding	5,60	0
ENSG00000075089	ACTR6	12	100199122	100241865	protein_coding	5,60	0
ENSG00000125910	S1PR4	19	3172346	3180332	protein_coding	5,60	0
ENSG00000129465	RIPK3	14	24336021	24340045	protein_coding	5,60	0
ENSG00000101473	ACOT8	20	45841721	45857406	protein_coding	5,60	0
ENSG00000130479	MAP1S	19	17719242	17734516	protein_coding	5,59	0
ENSG00000101191	DIDO1	20	62877738	62937952	protein_coding	5,59	0
ENSG00000143322	ABL2	1	179099327	179229684	protein_coding	5,58	0
ENSG00000128011	LRFN1	19	39306568	39315336	protein_coding	5,58	0
ENSG00000141219	C17orf80	17	73232233	73248947	protein_coding	5,57	0
ENSG00000172380	GNG12	1	67701466	67833467	protein_coding	5,57	0
ENSG00000101216	GMEB2	20	63587602	63627041	protein_coding	5,57	0
ENSG00000197601	FAR1	11	13668670	13732346	protein_coding	5,53	0
ENSG00000175054	ATR	3	142449235	142578826	protein_coding	5,53	0
ENSG00000171798	KNDC1	10	133160447	133226412	protein_coding	5,52	0
ENSG00000116205	TCEANC2	1	54053587	54112519	protein_coding	5,52	0
ENSG00000176974	SHMT1	17	18327860	18363563	protein_coding	5,52	0
ENSG00000164484	TMEM200A	6	130365734	130443063	protein_coding	5,51	0
ENSG00000136868	SLC31A1	9	113221562	113264492	protein_coding	5,51	0
ENSG00000170889	RPS9	19	54200742	54249003	protein_coding	5,50	0
ENSG00000100033	PRODH	22	18912777	18936553	protein_coding	5,50	0
ENSG00000274322	ENSG000002	20	1317571	1393096	protein_coding	5,50	0
ENSG00000182327	GLTPD2	17	4788959	4790390	protein_coding	5,50	0
ENSG00000186354	C9orf47	9	88990863	88996140	protein_coding	5,50	0
ENSG00000173320	STOX2	4	183853431	184023526	protein_coding	5,50	0
ENSG00000213614	HEXA	15	72340919	72376476	protein_coding	5,50	0
ENSG00000169764	UGP2	2	63840940	63891562	protein_coding	5,50	0
ENSG00000186666	BCDIN3D	12	49836039	49843129	protein_coding	5,49	0
ENSG00000164168	TMEM184C	4	147617383	147672044	protein_coding	5,49	0
ENSG00000180096	09/01/2001	16	30378133	30395991	protein_coding	5,49	0
ENSG00000137312	FLOT1	6	30727709	30742733	protein_coding	5,49	0
ENSG00000121764	HCRTR1	1	31617686	31632518	protein_coding	5,49	0
ENSG00000279018	ENSG000002	16	53207941	53226385	protein_coding	5,48	0
ENSG00000078967	UBE2D4	7	43926438	43956136	protein_coding	5,47	0
ENSG00000136866	ZFP37	9	113038380	113056759	protein_coding	5,47	0
ENSG00000077147	TM9SF3	10	96518109	96587452	protein_coding	5,46	0
ENSG00000135932	CAB39	2	230712845	230821075	protein_coding	5,45	0
ENSG00000101849	TBL1X	X	9463295	9719743	protein_coding	5,44	0
ENSG00000100526	CDKN3	14	54396849	54420218	protein_coding	5,44	0
ENSG00000157796	WDR19	4	39182404	39285810	protein_coding	5,44	0
ENSG00000197622	CDC42SE1	1	151050971	151070325	protein_coding	5,43	0
ENSG00000112218	GPR63	6	96794126	96837463	protein_coding	5,41	0
ENSG00000118705	RPN2	20	37178410	37241623	protein_coding	5,41	0
ENSG00000196569	LAMA2	6	128883141	129516569	protein_coding	5,41	0

ENSG00000065883	CDK13	7	39950037	40097134	protein_coding	5,41	0
ENSG00000079785	DDX1	2	15591178	15631111	protein_coding	5,41	0
ENSG00000205208	C4orf46	4	158666679	158672255	protein_coding	5,41	0
ENSG00000132275	RRP8	11	6595075	6603620	protein_coding	5,41	0
ENSG00000163812	ZDHHC3	3	44915257	44976185	protein_coding	5,41	0
ENSG00000155906	RMND1	6	151404763	151452181	protein_coding	5,40	0
ENSG00000184428	TOP1MT	8	143304384	143359979	protein_coding	5,40	0
ENSG00000147454	SLC25A37	8	23528805	23575463	protein_coding	5,40	0
ENSG00000114850	SSR3	3	156540140	156555184	protein_coding	5,40	0
ENSG00000154080	CHST9	18	26906481	27185317	protein_coding	5,40	0
ENSG00000106714	CNTNAP3	9	39072767	39288315	protein_coding	5,40	0
ENSG00000196747	HIST1H2AI	6	27808199	27808701	protein_coding	5,40	0
ENSG00000211452	DIO1	1	53891239	53911086	protein_coding	5,40	0
ENSG00000172113	NME6	3	48293264	48301685	protein_coding	5,40	0
ENSG00000133424	LARGE	22	33162226	33922841	protein_coding	5,39	0
ENSG00000159753	RLTPR	16	67644919	67657569	protein_coding	5,39	0
ENSG00000143499	SMYD2	1	214281102	214337131	protein_coding	5,39	0
ENSG00000116663	FBXO6	1	11664124	11674354	protein_coding	5,39	0
ENSG00000168522	FNTA	8	43034194	43085788	protein_coding	5,39	0
ENSG00000161594	KLHL10	17	41835685	41848384	protein_coding	5,38	0
ENSG00000117481	NSUN4	1	46340177	46365152	protein_coding	5,37	0
ENSG00000147852	VLDLR	9	2621834	2660053	protein_coding	5,37	0
ENSG00000270011	ZNF559-ZNF	19	9324174	9382617	protein_coding	5,36	0
ENSG00000048707	VPS13D	1	12230067	12512047	protein_coding	5,36	0
ENSG00000141524	TMC6	17	78110458	78132407	protein_coding	5,36	0
ENSG00000182952	HMGN4	6	26538405	26546254	protein_coding	5,36	0
ENSG00000110693	SOX6	11	15966449	16739591	protein_coding	5,36	0
ENSG00000156052	GNAQ	9	77716087	78031458	protein_coding	5,36	0
ENSG00000132600	PRMT7	16	68310974	68358563	protein_coding	5,36	0
ENSG00000113645	WWC1	5	168291651	168472303	protein_coding	5,35	0
ENSG00000163041	H3F3A	1	226061851	226072001	protein_coding	5,35	0
ENSG00000022976	ZNF839	14	102317377	102342702	protein_coding	5,35	0
ENSG00000131724	IL13RA1	X	118727572	118794539	protein_coding	5,35	0
ENSG00000130958	SLC35D2	9	96320706	96383710	protein_coding	5,35	0
ENSG00000196177	ACADSB	10	123008979	123058311	protein_coding	5,35	0
ENSG00000187699	C2orf88	2	189879609	190203484	protein_coding	5,34	0
ENSG00000134291	TMEM106C	12	47963569	47968878	protein_coding	5,34	0
ENSG00000161277	THAP8	19	36034985	36054762	protein_coding	5,33	0
ENSG00000211584	SLC48A1	12	47753916	47782753	protein_coding	5,33	0
ENSG00000110723	EXPH5	11	108505431	108593738	protein_coding	5,33	0
ENSG00000196821	C6orf106	6	34587288	34696859	protein_coding	5,33	0
ENSG00000129472	RAB2B	14	21459020	21476973	protein_coding	5,33	0
ENSG00000136710	CCDC115	2	130338241	130342349	protein_coding	5,33	0
ENSG00000107581	EIF3A	10	119033670	119080823	protein_coding	5,33	0
ENSG00000111880	RNGTT	6	88610272	88963721	protein_coding	5,32	0
ENSG00000113407	TARS	5	33440696	33469539	protein_coding	5,32	0
ENSG00000177479	ARIH2	3	48918821	48986382	protein_coding	5,32	0
ENSG00000101843	PSMD10	X	108084207	108091618	protein_coding	5,31	0
ENSG00000128607	KLHDC10	7	130070510	130135720	protein_coding	5,31	0
ENSG00000152127	MGAT5	2	134119983	134454621	protein_coding	5,31	0
ENSG00000145882	PCYOX1L	5	149358007	149369653	protein_coding	5,31	0
ENSG00000181666	HKR1	19	37312837	37369365	protein_coding	5,30	0
ENSG00000196562	SULF2	20	47656348	47786616	protein_coding	5,30	0
ENSG00000110777	POU2AF1	11	111352252	111455630	protein_coding	5,30	0
ENSG00000205238	SPDYE2	7	102551232	102562308	protein_coding	5,30	0
ENSG00000151967	SCHIP1	3	159839861	159897360	protein_coding	5,30	0
ENSG00000051382	PIK3CB	3	138652699	138834938	protein_coding	5,30	0
ENSG00000188938	FAM120AOS	9	93446494	93453592	protein_coding	5,30	0

ENSG00000163728	TTC14	3	180602130	180617828	protein_coding	5,30	0
ENSG00000118922	KLF12	13	73686089	74133905	protein_coding	5,29	0
ENSG00000068394	GPKOW	X	49113389	49123801	protein_coding	5,29	0
ENSG00000126804	ZBTB1	14	64503712	64533690	protein_coding	5,29	0
ENSG00000103199	ZNF500	16	4748239	4767624	protein_coding	5,29	0
ENSG00000229676	ZNF492	19	22634324	22667670	protein_coding	5,29	0
ENSG00000261884	ENSG000002	16	67929614	67936017	protein_coding	5,29	0
ENSG00000162601	MYSM1	1	58654739	58700092	protein_coding	5,27	0
ENSG00000198018	ENTPD7	10	99659506	99706240	protein_coding	5,27	0
ENSG00000165406	08-mars	10	45454585	45594906	protein_coding	5,27	0
ENSG00000112511	PHF1	6	33410399	33416453	protein_coding	5,27	0
ENSG00000130340	SNX9	6	157700387	157945077	protein_coding	5,26	0
ENSG00000187260	WDR86	7	151375909	151410727	protein_coding	5,26	0
ENSG00000113441	LNPEP	5	96935394	97037515	protein_coding	5,26	0
ENSG00000115207	GTF3C2	2	27325849	27357034	protein_coding	5,26	0
ENSG00000078328	RBFOX1	16	6019094	7713338	protein_coding	5,26	0
ENSG00000163517	HDAC11	3	13479724	13506424	protein_coding	5,25	0
ENSG00000145495	06-mars	5	10353703	10440388	protein_coding	5,25	0
ENSG00000166295	ANAPC16	10	72216000	72235860	protein_coding	5,25	0
ENSG00000071127	WDR1	4	10074339	10116949	protein_coding	5,25	0
ENSG00000108592	FTSJ3	17	63819433	63830012	protein_coding	5,24	0
ENSG00000072062	PRKACA	19	14091688	14118084	protein_coding	5,24	0
ENSG00000109079	TNFAIP1	17	28335602	28347009	protein_coding	5,24	0
ENSG00000155463	OXA1L	14	22766522	22773041	protein_coding	5,23	0
ENSG00000119004	CYP20A1	2	203238449	203305840	protein_coding	5,23	0
ENSG00000198363	ASPH	8	61500556	61714640	protein_coding	5,23	0
ENSG00000094841	UPRT	X	75274085	75304600	protein_coding	5,22	0
ENSG00000149657	LSM14B	20	62122461	62135378	protein_coding	5,21	0
ENSG00000081177	EXD2	14	69191511	69244020	protein_coding	5,21	0
ENSG00000117620	SLC35A3	1	99969789	100026979	protein_coding	5,21	0
ENSG00000113712	CSNK1A1	5	149492197	149551552	protein_coding	5,21	0
ENSG00000182568	SATB1	3	18345387	18445588	protein_coding	5,21	0
ENSG00000122042	UBL3	13	29764371	29850684	protein_coding	5,20	0
ENSG00000183889	ENSG000001	16	16317444	16350590	protein_coding	5,20	0
ENSG00000198848	CES1	16	55802851	55833337	protein_coding	5,20	0
ENSG00000280273	ENSG000002	8	11283500	11285068	protein_coding	5,20	0
ENSG00000262621	ENSG000002	16	3365099	3479550	protein_coding	5,20	0
ENSG00000151779	NBAS	2	15166909	15561330	protein_coding	5,20	0
ENSG00000130147	SH3BP4	2	234951973	235055714	protein_coding	5,20	0
ENSG00000169184	MN1	22	27748277	27801498	protein_coding	5,20	0
ENSG00000078902	TOLLIP	11	1274371	1309654	protein_coding	5,20	0
ENSG00000166224	SGPL1	10	70815961	70881173	protein_coding	5,19	0
ENSG00000123268	ATF1	12	50763710	50821122	protein_coding	5,18	0
ENSG00000156958	GALK2	15	49155656	49367869	protein_coding	5,18	0
ENSG00000204186	ZDBF2	2	206274663	206314428	protein_coding	5,17	0
ENSG00000137200	CMTR1	6	37433219	37482827	protein_coding	5,17	0
ENSG00000138069	RAB1A	2	65070701	65130106	protein_coding	5,17	0
ENSG00000250317	SMIM20	4	25861830	25929874	protein_coding	5,17	0
ENSG00000264522	OTUD7B	1	149937812	150010676	protein_coding	5,17	0
ENSG00000183579	ZNRF3	22	28883592	29057487	protein_coding	5,16	0
ENSG00000178988	MRFAP1L1	4	6707701	6709880	protein_coding	5,16	0
ENSG00000115816	CEBPZ	2	37201612	37231713	protein_coding	5,16	0
ENSG00000120742	SERP1	3	150541994	150603228	protein_coding	5,16	0
ENSG00000103647	CORO2B	15	68578969	68727806	protein_coding	5,16	0
ENSG00000083223	ZCCHC6	9	86287733	86354454	protein_coding	5,16	0
ENSG00000065308	TRAM2	6	52497402	52576915	protein_coding	5,16	0
ENSG00000184517	ZFP1	16	75148492	75172236	protein_coding	5,15	0
ENSG00000167770	OTUB1	11	63985853	64001811	protein_coding	5,15	0

ENSG00000107554	DNMBP	10	99875577	100009919	protein_coding	5,15	0
ENSG00000197372	ZNF675	19	23525631	23687220	protein_coding	5,15	0
ENSG00000008853	RHOBTB2	8	22987417	23020199	protein_coding	5,14	0
ENSG00000127311	HELB	12	66302545	66347645	protein_coding	5,14	0
ENSG00000109501	WFS1	4	6269849	6303265	protein_coding	5,14	0
ENSG00000166359	WDR88	19	33132090	33175795	protein_coding	5,14	0
ENSG00000159433	STARD9	15	42575659	42720981	protein_coding	5,14	0
ENSG00000078124	ACER3	11	76860867	77026797	protein_coding	5,14	0
ENSG00000131508	UBE2D2	5	139526431	139628433	protein_coding	5,13	0
ENSG00000090060	PAPOLA	14	96501433	96567111	protein_coding	5,13	0
ENSG00000165914	TTC7B	14	90524564	90816479	protein_coding	5,13	0
ENSG00000164051	CCDC51	3	48432164	48440456	protein_coding	5,12	0
ENSG00000090054	SPTLC1	9	92031999	92115384	protein_coding	5,11	0
ENSG00000166471	TMEM41B	11	9280654	9314780	protein_coding	5,10	0
ENSG00000171631	P2RY6	11	73264505	73298617	protein_coding	5,10	0
ENSG00000214688	C10orf105	10	71711701	71737824	protein_coding	5,10	0
ENSG00000146802	TMEM168	7	112762382	112790592	protein_coding	5,10	0
ENSG00000119906	SLF2	10	100912569	100965136	protein_coding	5,09	0
ENSG00000037474	NSUN2	5	6599239	6633291	protein_coding	5,09	0
ENSG00000129315	CCNT1	12	48688458	48716998	protein_coding	5,09	0
ENSG00000106524	ANKMY2	7	16599776	16645817	protein_coding	5,09	0
ENSG00000150457	LATS2	13	20973032	21061547	protein_coding	5,08	0
ENSG00000101350	KIF3B	20	32277664	32335011	protein_coding	5,08	0
ENSG00000108187	PBLD	10	68282660	68333049	protein_coding	5,08	0
ENSG00000001497	LAS1L	X	65512582	65534775	protein_coding	5,08	0
ENSG00000204604	ZNF468	19	52838008	52857649	protein_coding	5,07	0
ENSG00000040933	INPP4A	2	98444854	98594390	protein_coding	5,07	0
ENSG00000137364	TPMT	6	18128311	18155074	protein_coding	5,07	0
ENSG00000134748	PRPF38A	1	52404564	52420839	protein_coding	5,07	0
ENSG00000088038	CNOT3	19	54137728	54155681	protein_coding	5,06	0
ENSG00000107960	OBFC1	10	103882542	103918205	protein_coding	5,05	0
ENSG00000140740	UQCRC2	16	21952660	21983660	protein_coding	5,04	0
ENSG00000182180	MRPS16	10	73248843	73252693	protein_coding	5,04	0
ENSG00000132256	TRIM5	11	5663557	5938619	protein_coding	5,04	0
ENSG00000112033	PPARD	6	35342558	35428191	protein_coding	5,04	0
ENSG00000005436	GCFC2	2	75652000	75710989	protein_coding	5,04	0
ENSG00000147130	ZMYM3	X	71239624	71255146	protein_coding	5,03	0
ENSG00000156642	NPTN	15	73560014	73634134	protein_coding	5,03	0
ENSG00000136436	CALCOCO2	17	48830988	48866522	protein_coding	5,02	0
ENSG00000204406	MBD5	2	148021011	148516971	protein_coding	5,02	0
ENSG00000138081	FBXO11	2	47789316	47905793	protein_coding	5,02	0
ENSG00000187609	EXD3	9	137306896	137423262	protein_coding	5,02	0
ENSG00000144580	RQCD1	2	218568580	218597080	protein_coding	5,01	0
ENSG00000153147	SMARCA5	4	143513463	143557486	protein_coding	5,01	0
ENSG00000101166	PRELID3B	20	59033145	59042909	protein_coding	5,01	0
ENSG00000137145	DENND4C	9	19230435	19373545	protein_coding	5,01	0
ENSG00000135372	NAT10	11	34105602	34147670	protein_coding	5,01	0
ENSG00000084112	SSH1	12	108778192	108857590	protein_coding	5,00	0
ENSG00000068308	OTUD5	X	48922028	48958386	protein_coding	5,00	0
ENSG00000158941	CCAR2	8	22604632	22621514	protein_coding	5,00	0
ENSG00000008735	MAPK8IP2	22	50600685	50613981	protein_coding	5,00	0
ENSG00000196689	TRPV1	17	3565444	3609411	protein_coding	5,00	0
ENSG00000160539	PLPP7	9	131289694	131309262	protein_coding	5,00	0
ENSG00000204385	SLC44A4	6	31863192	31879046	protein_coding	5,00	0
ENSG00000280987	ENSG000002	5	139273752	139331677	protein_coding	5,00	0
ENSG00000162543	UBXN10	1	20186085	20196048	protein_coding	5,00	0
ENSG00000188895	MSL1	17	40122298	40136916	protein_coding	5,00	0
ENSG00000104660	LEPROTL1	8	30095398	30177208	protein_coding	4,99	0

ENSG00000114770	ABCC5	3	183919934	184018015	protein_coding	4,99	0
ENSG00000119203	CPSF3	2	9423568	9473101	protein_coding	4,99	0
ENSG00000111725	PRKAB1	12	119667753	119681630	protein_coding	4,99	0
ENSG00000154845	PPP4R1	18	9546791	9615240	protein_coding	4,98	0
ENSG00000095370	SH2D3C	9	127738317	127778741	protein_coding	4,98	0
ENSG00000275052	PPP4R3B	2	55547292	55618880	protein_coding	4,96	0
ENSG00000106682	EIF4H	7	74174245	74197101	protein_coding	4,96	0
ENSG00000196743	GM2A	5	151212150	151270440	protein_coding	4,95	0
ENSG00000073417	PDE8A	15	84980440	85139145	protein_coding	4,95	0
ENSG00000168813	ZNF507	19	32345594	32387667	protein_coding	4,95	0
ENSG00000204634	TBC1D8	2	101007617	101252866	protein_coding	4,95	0
ENSG00000167261	DPEP2	16	67987394	68000586	protein_coding	4,94	0
ENSG00000090316	MAEA	4	1289851	1340147	protein_coding	4,94	0
ENSG00000131788	PIAS3	1	145848522	145859836	protein_coding	4,94	0
ENSG00000276045	ORAI1	12	121626550	121642677	protein_coding	4,93	0
ENSG00000036549	ZZZ3	1	77562416	77683419	protein_coding	4,93	0
ENSG00000168488	ATXN2L	16	28823035	28837237	protein_coding	4,93	0
ENSG00000100380	ST13	22	40824535	40857022	protein_coding	4,93	0
ENSG00000171490	RSL1D1	16	11833850	11851585	protein_coding	4,93	0
ENSG00000197362	ZNF786	7	149069643	149090782	protein_coding	4,93	0
ENSG00000180198	RCC1	1	28505943	28539300	protein_coding	4,93	0
ENSG00000136932	TRMO	9	97904489	97922570	protein_coding	4,93	0
ENSG00000269743	SLC25A53	X	104099214	104157027	protein_coding	4,92	0
ENSG00000204856	FAM216A	12	110468364	110490385	protein_coding	4,92	0
ENSG00000091009	RBM27	5	146203600	146289132	protein_coding	4,92	0
ENSG00000137819	PAQR5	15	69298947	69407780	protein_coding	4,91	0
ENSG00000122882	ECD	10	73130155	73169055	protein_coding	4,90	0
ENSG00000189362	NEMP2	2	190504342	190534722	protein_coding	4,90	0
ENSG00000140987	ZSCAN32	16	3382081	3401065	protein_coding	4,90	0
ENSG00000003249	DBNDD1	16	90004865	90020128	protein_coding	4,90	0
ENSG00000161542	PRPSAP1	17	76309486	76384521	protein_coding	4,90	0
ENSG00000141642	ELAC1	18	50967991	50988121	protein_coding	4,89	0
ENSG00000180008	SOCS4	14	55027230	55049488	protein_coding	4,89	0
ENSG00000183520	UTP11L	1	38009258	38024824	protein_coding	4,89	0
ENSG00000157500	APPL1	3	57227737	57273468	protein_coding	4,89	0
ENSG00000138688	KIAA1109	4	122152333	122362758	protein_coding	4,88	0
ENSG00000135245	HILPDA	7	128455849	128458418	protein_coding	4,88	0
ENSG00000171462	DLK2	6	43450352	43456632	protein_coding	4,88	0
ENSG00000134539	KLRD1	12	10226058	10329600	protein_coding	4,88	0
ENSG00000143155	TIPRL	1	168178933	168202114	protein_coding	4,88	0
ENSG00000172766	NAA16	13	41311205	41377030	protein_coding	4,87	0
ENSG00000109854	HTATIP2	11	20363685	20383783	protein_coding	4,87	0
ENSG00000107854	TNKS2	10	91798312	91865276	protein_coding	4,87	0
ENSG00000126351	THRA	17	40058290	40093867	protein_coding	4,87	0
ENSG00000170473	PYM1	12	55901413	55932618	protein_coding	4,87	0
ENSG00000151327	FAM177A1	14	35044907	35113130	protein_coding	4,86	0
ENSG00000183291	ENSG000001	1	86862445	86914424	protein_coding	4,86	0
ENSG00000067369	TP53BP1	15	43403061	43510728	protein_coding	4,86	0
ENSG00000152242	C18orf25	18	46173553	46266991	protein_coding	4,86	0
ENSG00000115145	STAM2	2	152116801	152175992	protein_coding	4,86	0
ENSG00000123600	METTL8	2	171317405	171434802	protein_coding	4,85	0
ENSG00000154079	SDHAF4	6	70566917	70589569	protein_coding	4,84	0
ENSG00000163393	SLC22A15	1	115976498	116070054	protein_coding	4,84	0
ENSG00000144134	RABL2A	2	113627229	113643396	protein_coding	4,84	0
ENSG00000137575	SDCBP	8	58552924	58582860	protein_coding	4,84	0
ENSG00000127666	TICAM1	19	4815932	4831704	protein_coding	4,84	0
ENSG00000148339	SLC25A25	9	128068201	128109245	protein_coding	4,84	0
ENSG00000145975	FAM217A	6	4049434	4087344	protein_coding	4,83	0

ENSG00000129292	PHF20L1	8	132775358	132848807	protein_coding	4,83	0
ENSG00000177917	ARL6IP6	2	152717893	152761253	protein_coding	4,83	0
ENSG00000206199	ANKUB1	3	149761105	149968385	protein_coding	4,83	0
ENSG00000112981	NME5	5	138115172	138139443	protein_coding	4,83	0
ENSG00000083544	TDRD3	13	60396457	60573878	protein_coding	4,83	0
ENSG00000178035	IMPDH2	3	49024325	49029408	protein_coding	4,82	0
ENSG00000140932	CMTM2	16	66579448	66588275	protein_coding	4,82	0
ENSG00000181894	ZNF329	19	58126252	58155110	protein_coding	4,81	0
ENSG00000149269	PAK1	11	77321707	77474635	protein_coding	4,81	0
ENSG00000140829	DHX38	16	72093562	72112912	protein_coding	4,81	0
ENSG00000112701	SENP6	6	75601509	75718278	protein_coding	4,80	0
ENSG00000129003	VPS13C	15	61852389	62060473	protein_coding	4,80	0
ENSG00000080854	IGSF9B	11	133908564	133956985	protein_coding	4,80	0
ENSG00000106701	FSD1L	9	105447796	105552433	protein_coding	4,80	0
ENSG00000101574	METTL4	18	2537525	2571509	protein_coding	4,80	0
ENSG00000134247	PTGFRN	1	116910057	116990358	protein_coding	4,79	0
ENSG00000178913	TAF7	5	141260225	141320821	protein_coding	4,79	0
ENSG00000090061	CCNK	14	99481169	99535044	protein_coding	4,79	0
ENSG00000171824	EXOSC10	1	11066618	11099881	protein_coding	4,78	0
ENSG00000135469	COQ10A	12	56266858	56270966	protein_coding	4,78	0
ENSG00000198815	FOXJ3	1	42176539	42335877	protein_coding	4,77	0
ENSG00000169139	UBE2V2	8	48008400	48064708	protein_coding	4,77	0
ENSG00000146247	PHIP	6	78935867	79078236	protein_coding	4,77	0
ENSG00000162869	PPP1R21	2	48440598	48515391	protein_coding	4,77	0
ENSG00000171469	ZNF561	19	9604680	9621399	protein_coding	4,77	0
ENSG00000105479	CCDC114	19	48296457	48321894	protein_coding	4,76	0
ENSG00000134686	PHC2	1	33323623	33431052	protein_coding	4,76	0
ENSG00000065000	AP3D1	19	2100988	2164465	protein_coding	4,75	0
ENSG00000087274	ADD1	4	2843857	2930076	protein_coding	4,75	0
ENSG00000225921	NOL7	6	13615327	13632739	protein_coding	4,75	0
ENSG00000120694	HSPH1	13	31134974	31162388	protein_coding	4,75	0
ENSG00000105341	ATP5SL	19	41431318	41440717	protein_coding	4,75	0
ENSG00000061676	NCKAP1	2	182909115	183038858	protein_coding	4,75	0
ENSG00000156170	NDUFAF6	8	94895767	95116455	protein_coding	4,75	0
ENSG00000136754	ABI1	10	26746593	26861087	protein_coding	4,75	0
ENSG00000179134	SAMD4B	19	39342396	39385710	protein_coding	4,75	0
ENSG00000116266	STXBP3	1	108746674	108809526	protein_coding	4,75	0
ENSG00000052841	TTC17	11	43358932	43494933	protein_coding	4,74	0
ENSG00000198887	SMC5	9	70258962	70354888	protein_coding	4,74	0
ENSG00000198160	MIER1	1	66924895	66988619	protein_coding	4,74	0
ENSG00000132423	COQ3	6	99369400	99394204	protein_coding	4,74	0
ENSG00000103740	ACSBG1	15	78167468	78245688	protein_coding	4,73	0
ENSG00000083168	KAT6A	8	41929479	42051990	protein_coding	4,73	0
ENSG00000198799	LRIG2	1	113073209	113132260	protein_coding	4,73	0
ENSG00000162591	MEGF6	1	3489920	3611495	protein_coding	4,72	0
ENSG00000162408	NOL9	1	6521347	6554535	protein_coding	4,72	0
ENSG00000168806	LCMT2	15	43323649	43330605	protein_coding	4,72	0
ENSG00000136643	RPS6KC1	1	213051233	213274773	protein_coding	4,72	0
ENSG00000099814	CEP170B	14	104865280	104896770	protein_coding	4,71	0
ENSG00000184007	PTP4A2	1	31906421	31944856	protein_coding	4,71	0
ENSG00000095574	IKZF5	10	122990806	123008817	protein_coding	4,71	0
ENSG00000129518	EAPP	14	34515929	34539711	protein_coding	4,71	0
ENSG00000172466	ZNF24	18	35332212	35345482	protein_coding	4,71	0
ENSG00000176953	NFATC2IP	16	28950807	28967097	protein_coding	4,70	0
ENSG00000038382	TRIO	5	14143702	14532128	protein_coding	4,70	0
ENSG00000165669	FAM204A	10	118297930	118342328	protein_coding	4,70	0
ENSG00000136682	CBWD2	2	113437691	113496189	protein_coding	4,69	0
ENSG00000162994	CLHC1	2	55174791	55232563	protein_coding	4,69	0

ENSG00000063046	EIF4B	12	53006158	53042209	protein_coding	4,69	0
ENSG00000110318	CEP126	11	101915015	102001058	protein_coding	4,69	0
ENSG00000063241	ISOC2	19	55452985	55462343	protein_coding	4,69	0
ENSG00000204852	TCTN1	12	110614027	110649430	protein_coding	4,68	0
ENSG00000099810	MTAP	9	21802543	21937651	protein_coding	4,68	0
ENSG00000169629	RGPD8	2	112370092	112434488	protein_coding	4,68	0
ENSG00000079387	SENP1	12	48042898	48106308	protein_coding	4,68	0
ENSG00000101266	CSNK2A1	20	473591	543821	protein_coding	4,67	0
ENSG00000185630	PBX1	1	164555584	164899296	protein_coding	4,67	0
ENSG00000170464	DNAJC18	5	139408588	139444491	protein_coding	4,67	0
ENSG00000138942	RNF185	22	31160183	31207019	protein_coding	4,67	0
ENSG00000142039	CCDC97	19	41310189	41324883	protein_coding	4,67	0
ENSG00000183688	FAM101B	17	439978	445939	protein_coding	4,66	0
ENSG00000130764	LRRC47	1	3778558	3796504	protein_coding	4,66	0
ENSG00000188917	TRMT2B	X	101009346	101052116	protein_coding	4,66	0
ENSG00000135047	CTSL	9	87725519	87731393	protein_coding	4,65	0
ENSG00000121964	GTDC1	2	143938068	144332568	protein_coding	4,65	0
ENSG00000067365	METTL22	16	8621683	8649654	protein_coding	4,65	0
ENSG00000160299	PCNT	21	46324122	46445769	protein_coding	4,64	0
ENSG00000110583	NAA40	11	63938959	63957328	protein_coding	4,64	0
ENSG00000221923	ZNF880	19	52369917	52385795	protein_coding	4,64	0
ENSG00000173614	NMNAT1	1	9943428	9985501	protein_coding	4,64	0
ENSG00000089737	DDX24	14	94048291	94081245	protein_coding	4,64	0
ENSG00000274523	WBSCR16	7	75027122	75074228	protein_coding	4,63	0
ENSG00000065491	TBC1D22B	6	37257772	37332970	protein_coding	4,63	0
ENSG00000116701	NCF2	1	183555563	183590876	protein_coding	4,62	0
ENSG00000149136	SSRP1	11	57325985	57335877	protein_coding	4,62	0
ENSG00000166225	FRS2	12	69470349	69579789	protein_coding	4,61	0
ENSG00000171940	ZNF217	20	53567065	53609907	protein_coding	4,61	0
ENSG00000173451	THAP2	12	71663009	71680639	protein_coding	4,61	0
ENSG00000179988	PSTK	10	122954381	122997513	protein_coding	4,61	0
ENSG00000055332	EIF2AK2	2	37099210	37157065	protein_coding	4,61	0
ENSG00000179195	ZNF664	12	123971845	124015439	protein_coding	4,60	0
ENSG00000159958	TNFRSF13C	22	41922023	41926818	protein_coding	4,60	0
ENSG00000277224	HIST1H2BF	6	26199520	26200715	protein_coding	4,60	0
ENSG00000279386	ENSG000002	4	37866561	37867091	protein_coding	4,60	0
ENSG00000123843	C4BPB	1	207088842	207099993	protein_coding	4,60	0
ENSG00000197548	ATG7	3	11272309	11557665	protein_coding	4,60	0
ENSG00000010404	IDS	X	149476990	149521096	protein_coding	4,59	0
ENSG00000154645	CHODL	21	17901263	18267373	protein_coding	4,59	0
ENSG00000221883	ARIH2OS	3	48917788	48919385	protein_coding	4,59	0
ENSG00000184226	PCDH9	13	66302834	67230445	protein_coding	4,58	0
ENSG00000081307	UBA5	3	132654446	132678097	protein_coding	4,58	0
ENSG00000157426	AASDH	4	56338287	56387508	protein_coding	4,58	0
ENSG00000047056	WDR37	10	1049538	1132297	protein_coding	4,57	0
ENSG00000125037	EMC3	3	9962537	10011116	protein_coding	4,57	0
ENSG00000109466	KLHL2	4	165207618	165323156	protein_coding	4,56	0
ENSG00000247746	USP51	X	55484616	55489202	protein_coding	4,56	0
ENSG00000079974	RABL2B	22	50767501	50783663	protein_coding	4,56	0
ENSG00000171148	TADA3	3	9779860	9793011	protein_coding	4,56	0
ENSG00000182628	SKA2	17	59109951	59155269	protein_coding	4,55	0
ENSG00000280178	ENSG000002	22	23782283	23783958	protein_coding	4,55	0
ENSG00000215301	DDX3X	X	41333348	41364472	protein_coding	4,54	0
ENSG00000176834	VSIG10	12	118063593	118136026	protein_coding	4,54	0
ENSG00000074695	LMAN1	18	59327823	59359962	protein_coding	4,54	0
ENSG00000011451	WIZ	19	15419980	15449951	protein_coding	4,54	0
ENSG00000115484	CCT4	2	61868089	61888804	protein_coding	4,54	0
ENSG00000143653	SCCPDH	1	246724047	246768137	protein_coding	4,53	0

ENSG00000103342	GSPT1	16	11868128	11916082	protein_coding	4,53	0
ENSG00000197302	ZNF720	16	31713229	31794869	protein_coding	4,53	0
ENSG00000184988	TMEM106A	17	43211835	43220041	protein_coding	4,52	0
ENSG00000164659	KIAA1324L	7	86876906	87059699	protein_coding	4,52	0
ENSG00000146842	TMEM209	7	130164715	130207770	protein_coding	4,52	0
ENSG00000107758	PPP3CB	10	73436428	73496024	protein_coding	4,52	0
ENSG00000119685	TTLL5	14	75633625	75955078	protein_coding	4,51	0
ENSG00000144747	TMF1	3	69019827	69052303	protein_coding	4,51	0
ENSG00000164938	TP53INP1	8	94925972	94949411	protein_coding	4,51	0
ENSG00000151413	NUBPL	14	31489956	31861224	protein_coding	4,51	0
ENSG00000104231	ZFAND1	8	81701334	81732903	protein_coding	4,51	0
ENSG00000136930	PSMB7	9	124353466	124415444	protein_coding	4,51	0
ENSG00000174799	CEP135	4	55948871	56033363	protein_coding	4,50	0
ENSG00000131023	LATS1	6	149658153	149718256	protein_coding	4,50	0
ENSG00000111412	C12orf49	12	116710185	116738070	protein_coding	4,50	0
ENSG00000117472	TSPAN1	1	46175073	46185958	protein_coding	4,50	0
ENSG00000158623	COPG2	7	130506238	130668748	protein_coding	4,49	0
ENSG00000145911	N4BP3	5	178113443	178126087	protein_coding	4,49	0
ENSG00000066427	ATXN3	14	92038652	92106621	protein_coding	4,49	0
ENSG00000161011	SQSTM1	5	179806398	179838078	protein_coding	4,49	0
ENSG00000122970	IFT81	12	110124335	110218797	protein_coding	4,48	0
ENSG00000013016	EHD3	2	31234337	31269447	protein_coding	4,48	0
ENSG00000131368	MRPS25	3	15042460	15065335	protein_coding	4,48	0
ENSG00000182985	CADM1	11	115169218	115504957	protein_coding	4,48	0
ENSG00000140006	WDR89	14	63597039	63641861	protein_coding	4,48	0
ENSG00000124787	RPP40	6	4994732	5004063	protein_coding	4,48	0
ENSG00000109572	CLCN3	4	169612633	169723673	protein_coding	4,47	0
ENSG00000106799	TGFBR1	9	99104038	99154192	protein_coding	4,47	0
ENSG00000073584	SMARCE1	17	40624962	40648508	protein_coding	4,47	0
ENSG00000147324	MFHAS1	8	8783354	8893645	protein_coding	4,46	0
ENSG00000164366	CCDC127	5	196871	218215	protein_coding	4,46	0
ENSG00000166938	DIS3L	15	66293217	66333898	protein_coding	4,45	0
ENSG00000169490	TM2D2	8	38988808	38996824	protein_coding	4,45	0
ENSG00000090273	NUDC	1	26900238	26946862	protein_coding	4,45	0
ENSG00000082258	CCNT2	2	134918235	134959342	protein_coding	4,45	0
ENSG00000138764	CCNG2	4	77157151	77433388	protein_coding	4,45	0
ENSG00000107290	SETX	9	132261356	132354985	protein_coding	4,45	0
ENSG00000146282	RARS2	6	87514378	87590003	protein_coding	4,45	0
ENSG00000108219	TSPAN14	10	80454166	80533123	protein_coding	4,44	0
ENSG00000175283	DOLK	9	128945530	128947619	protein_coding	4,44	0
ENSG00000168067	MAP4K2	11	64784914	64803241	protein_coding	4,44	0
ENSG00000166508	MCM7	7	100092728	100101940	protein_coding	4,44	0
ENSG00000125863	MKKS	20	10401009	10434222	protein_coding	4,44	0
ENSG00000165997	ARL5B	10	18659405	18681639	protein_coding	4,44	0
ENSG00000140848	CPNE2	16	57092537	57148367	protein_coding	4,44	0
ENSG00000215126	CBWD7	9	41131306	41199261	protein_coding	4,43	0
ENSG00000150337	FCGR1A	1	149782671	149792518	protein_coding	4,43	0
ENSG00000112290	WASF1	6	110099819	110180004	protein_coding	4,42	0
ENSG00000133466	C1QTNF6	22	37180167	37199385	protein_coding	4,41	0
ENSG00000064490	RFXANK	19	19192229	19201869	protein_coding	4,41	0
ENSG00000163635	ATXN7	3	63864557	64003462	protein_coding	4,41	0
ENSG00000120915	EPHX2	8	27490779	27545564	protein_coding	4,41	0
ENSG00000119121	TRPM6	9	74722495	74888094	protein_coding	4,40	0
ENSG00000198785	GRIN3A	9	101569353	101738580	protein_coding	4,40	0
ENSG00000198523	PLN	6	118548298	118560730	protein_coding	4,40	0
ENSG00000183513	COA5	2	98599310	98608515	protein_coding	4,40	0
ENSG00000102786	INTS6	13	51354077	51454264	protein_coding	4,40	0
ENSG00000168646	AXIN2	17	65528563	65561647	protein_coding	4,39	0

ENSG00000185252	ZNF74	22	20394115	20408461	protein_coding	4,39	0
ENSG00000133103	COG6	13	39655627	39791665	protein_coding	4,39	0
ENSG00000170385	SLC30A1	1	211571568	211578742	protein_coding	4,39	0
ENSG00000124635	HIST1H2BJ	6	27125897	27132750	protein_coding	4,39	0
ENSG00000187068	C3orf70	3	185078050	185153014	protein_coding	4,38	0
ENSG00000143337	TOR1AIP1	1	179882042	179925000	protein_coding	4,38	0
ENSG00000131127	ZNF141	4	337814	384864	protein_coding	4,38	0
ENSG00000164011	ZNF691	1	42846573	42852477	protein_coding	4,37	0
ENSG00000171488	LRRC8C	1	89633072	89769903	protein_coding	4,37	0
ENSG00000140157	NIPA2	15	22838641	22868384	protein_coding	4,36	0
ENSG00000102362	SYTL4	X	100674491	100732123	protein_coding	4,36	0
ENSG00000141076	CIRH1A	16	69131291	69231130	protein_coding	4,35	0
ENSG00000010244	ZNF207	17	32350117	32381886	protein_coding	4,35	0
ENSG00000213639	PPP1CB	2	28751640	28802940	protein_coding	4,35	0
ENSG00000104419	NDRG1	8	133237171	133302022	protein_coding	4,35	0
ENSG00000238083	LRRC37A2	17	46511511	46555650	protein_coding	4,34	0
ENSG00000125814	NAPB	20	23374519	23421519	protein_coding	4,34	0
ENSG00000213625	LEPROT	1	65420587	65436007	protein_coding	4,34	0
ENSG00000138802	SEC24B	4	109433772	109540896	protein_coding	4,33	0
ENSG00000134716	CYP2J2	1	59893308	59926790	protein_coding	4,33	0
ENSG00000095139	ARCN1	11	118572390	118603033	protein_coding	4,33	0
ENSG00000143164	DCAF6	1	167935783	168075843	protein_coding	4,33	0
ENSG00000198218	QRICH1	3	49029707	49094363	protein_coding	4,32	0
ENSG00000173674	EIF1AX	X	20124518	20141844	protein_coding	4,32	0
ENSG00000136367	ZFHX2	14	23520855	23556192	protein_coding	4,31	0
ENSG00000113391	FAM172A	5	93618069	94111699	protein_coding	4,31	0
ENSG00000124532	MRS2	6	24402908	24426194	protein_coding	4,30	0
ENSG00000164604	GPR85	7	113078331	113087778	protein_coding	4,30	0
ENSG00000198752	CDC42BPB	14	102932379	103057462	protein_coding	4,30	0
ENSG00000102221	JADE3	X	46912276	47061242	protein_coding	4,30	0
ENSG00000151846	PABPC3	13	25095868	25099254	protein_coding	4,30	0
ENSG00000124120	TTPAL	20	44475886	44494603	protein_coding	4,30	0
ENSG00000116096	SPR	2	72887360	72892158	protein_coding	4,29	0
ENSG00000109436	TBC1D9	4	140620765	140756120	protein_coding	4,29	0
ENSG00000177425	PAWR	12	79574979	79691097	protein_coding	4,29	0
ENSG00000163072	NOSTRIN	2	168786539	168865514	protein_coding	4,28	0
ENSG00000004866	ST7	7	116953238	117230103	protein_coding	4,28	0
ENSG00000176018	LYSMD3	5	90515611	90529584	protein_coding	4,28	0
ENSG00000136937	NCBP1	9	97633626	97673748	protein_coding	4,28	0
ENSG00000100014	SPECC1L	22	24270817	24417740	protein_coding	4,28	0
ENSG00000162402	USP24	1	55066359	55215113	protein_coding	4,28	0
ENSG00000100982	PCIF1	20	45934628	45948023	protein_coding	4,28	0
ENSG00000111911	HINT3	6	125956781	125980244	protein_coding	4,27	0
ENSG00000172534	HCFC1	X	153947553	153971807	protein_coding	4,27	0
ENSG00000090989	EXOC1	4	55853616	55905034	protein_coding	4,27	0
ENSG00000222011	FAM185A	7	102748971	102809225	protein_coding	4,27	0
ENSG00000087365	SF3B2	11	66050729	66069308	protein_coding	4,27	0
ENSG00000138777	PPA2	4	105369077	105474081	protein_coding	4,26	0
ENSG00000029153	ARNTL2	12	27332854	27425289	protein_coding	4,26	0
ENSG00000066777	ARFGEF1	8	67173511	67343677	protein_coding	4,25	0
ENSG00000166503	ENSG00000	15	83112738	83208018	protein_coding	4,25	0
ENSG00000113580	NR3C1	5	143277931	143435512	protein_coding	4,25	0
ENSG00000136206	SPDYE1	7	44000889	44010122	protein_coding	4,25	0
ENSG00000158457	TSPAN33	7	129144892	129169697	protein_coding	4,25	0
ENSG00000072609	CHFR	12	132822187	132956304	protein_coding	4,24	0
ENSG00000088298	EDEM2	20	35115357	35147364	protein_coding	4,24	0
ENSG00000170542	SERPINB9	6	2887266	2903280	protein_coding	4,24	0
ENSG00000124201	ZNFX1	20	49237946	49278426	protein_coding	4,24	0

ENSG00000092148	HECTD1	14	31100112	31207804	protein_coding	4,24	0
ENSG00000070950	RAD18	3	8775402	8963773	protein_coding	4,24	0
ENSG00000081386	ZNF510	9	96755865	96778129	protein_coding	4,24	0
ENSG00000120662	MTRF1	13	41216369	41263577	protein_coding	4,23	0
ENSG00000145868	FBXO38	5	148383935	148442836	protein_coding	4,23	0
ENSG00000165626	BEND7	10	13438484	13528974	protein_coding	4,23	0
ENSG00000057608	GDI2	10	5765223	5842132	protein_coding	4,23	0
ENSG00000132141	CCT6B	17	34927859	34981078	protein_coding	4,23	0
ENSG00000197037	ZSCAN25	7	99616946	99632407	protein_coding	4,22	0
ENSG00000117543	DPH5	1	100989623	101026088	protein_coding	4,22	0
ENSG00000185684	EP400NL	12	132084283	132131639	protein_coding	4,21	0
ENSG00000149311	ATM	11	108222484	108369102	protein_coding	4,21	0
ENSG00000214022	REPIN1	7	150368189	150374044	protein_coding	4,20	0
ENSG00000101052	IFT52	20	43590931	43647296	protein_coding	4,20	0
ENSG00000181192	DHTKD1	10	12068972	12123225	protein_coding	4,20	0
ENSG00000224470	ATXN1L	16	71845991	71885268	protein_coding	4,20	0
ENSG00000186522	09/01/2010	2	109542982	109614206	protein_coding	4,20	0
ENSG00000166197	NOLC1	10	102152176	102163871	protein_coding	4,20	0
ENSG00000085978	ATG16L1	2	233210051	233295674	protein_coding	4,18	0
ENSG00000122728	TAF1L	9	32629454	32635669	protein_coding	4,18	0
ENSG00000071242	RPS6KA2	6	166409364	166906451	protein_coding	4,18	0
ENSG00000116874	WARS2	1	119031216	119140671	protein_coding	4,17	0
ENSG00000086200	IPO11	5	62403972	62628582	protein_coding	4,17	0
ENSG00000112282	MED23	6	131573966	131628229	protein_coding	4,17	0
ENSG00000113163	COL4A3BP	5	75368486	75512138	protein_coding	4,16	0
ENSG00000221909	FAM200A	7	99546308	99558536	protein_coding	4,16	0
ENSG00000174010	KLHL15	X	23983720	24027186	protein_coding	4,15	0
ENSG00000196417	ZNF765	19	53389793	53430413	protein_coding	4,15	0
ENSG00000164187	LMBRD2	5	36098412	36151961	protein_coding	4,15	0
ENSG00000011105	TSPAN9	12	3077355	3286564	protein_coding	4,15	0
ENSG00000167085	PHB	17	49404049	49414905	protein_coding	4,15	0
ENSG00000143578	CREB3L4	1	153967534	153974363	protein_coding	4,15	0
ENSG00000075151	EIF4G3	1	20806292	21176888	protein_coding	4,15	0
ENSG00000177853	ZNF518A	10	96129715	96205288	protein_coding	4,14	0
ENSG00000138363	ATIC	2	215311817	215349773	protein_coding	4,14	0
ENSG00000171962	DRC3	17	17972813	18016889	protein_coding	4,14	0
ENSG00000167491	GATAD2A	19	19385826	19508931	protein_coding	4,14	0
ENSG00000103404	USP31	16	23061406	23149270	protein_coding	4,14	0
ENSG00000133026	MYH10	17	8474205	8630761	protein_coding	4,14	0
ENSG00000116704	SLC35D1	1	66999332	67054099	protein_coding	4,13	0
ENSG00000070756	PABPC1	8	100685816	100722809	protein_coding	4,13	0
ENSG00000099956	SMARCB1	22	23786963	23834516	protein_coding	4,13	0
ENSG00000204977	TRIM13	13	49995888	50020481	protein_coding	4,12	0
ENSG00000137166	FOXP4	6	41546426	41602384	protein_coding	4,12	0
ENSG00000118579	MED28	4	17614631	17634105	protein_coding	4,12	0
ENSG00000185379	RAD51D	17	35092208	35121522	protein_coding	4,12	0
ENSG00000198876	DCAF12	9	34086387	34127399	protein_coding	4,12	0
ENSG00000196150	ZNF250	8	144876497	144902168	protein_coding	4,12	0
ENSG00000078140	UBE2K	4	39698044	39782792	protein_coding	4,11	0
ENSG00000170037	CNTROB	17	7932101	7949918	protein_coding	4,11	0
ENSG00000114388	NPRL2	3	50347330	50351091	protein_coding	4,11	0
ENSG00000128266	GNAZ	22	23070361	23125037	protein_coding	4,10	0
ENSG00000182704	TSKU	11	76782251	76798154	protein_coding	4,10	0
ENSG00000104522	TSTA3	8	143612618	143618048	protein_coding	4,10	0
ENSG00000134602	STK26	X	132023265	132075943	protein_coding	4,10	0
ENSG00000196535	MYO18A	17	29073517	29180412	protein_coding	4,10	0
ENSG00000133858	ZFC3H1	12	71609472	71667725	protein_coding	4,09	0
ENSG00000175161	CADM2	3	84958981	86074429	protein_coding	4,09	0

ENSG00000249715	FER1L5	2	96642737	96704887	protein_coding	4,09	0
ENSG00000160710	ADAR	1	154582062	154627999	protein_coding	4,09	0
ENSG00000183955	SETD8	12	123383773	123409358	protein_coding	4,08	0
ENSG00000120438	TCP1	6	159778498	159789749	protein_coding	4,08	0
ENSG00000116128	BCL9	1	147541412	147626216	protein_coding	4,08	0
ENSG00000163104	SMARCAD1	4	94207611	94291292	protein_coding	4,08	0
ENSG00000173598	NUDT4	12	93377883	93408146	protein_coding	4,08	0
ENSG00000138814	PPP3CA	4	101023409	101348278	protein_coding	4,08	0
ENSG00000003509	NDUFAF7	2	37231631	37253403	protein_coding	4,08	0
ENSG00000075303	SLC25A40	7	87833568	87876357	protein_coding	4,06	0
ENSG00000147601	TERF1	8	73008864	73048122	protein_coding	4,05	0
ENSG00000171603	CLSTN1	1	9729026	9824526	protein_coding	4,05	0
ENSG00000166349	RAG1	11	36510709	36593156	protein_coding	4,05	0
ENSG00000138641	HERC3	4	88521048	88708542	protein_coding	4,05	0
ENSG00000129158	SERGEF	11	17788048	18013162	protein_coding	4,04	0
ENSG00000132478	UNK	17	75784771	75825799	protein_coding	4,04	0
ENSG00000100154	TTC28	22	27978014	28679865	protein_coding	4,04	0
ENSG00000075292	ZNF638	2	71276561	71435069	protein_coding	4,04	0
ENSG00000152104	PTPN14	1	214348696	214552449	protein_coding	4,04	0
ENSG00000102934	PLLP	16	57248547	57284687	protein_coding	4,04	0
ENSG00000109118	PHF12	17	28905250	28951771	protein_coding	4,04	0
ENSG00000163602	RYBP	3	72371825	72446918	protein_coding	4,03	0
ENSG00000101150	TPD52L2	20	63865228	63891545	protein_coding	4,03	0
ENSG00000164327	RICTOR	5	38937919	39074408	protein_coding	4,03	0
ENSG00000055208	TAB2	6	149218641	149411613	protein_coding	4,02	0
ENSG00000198246	SLC29A3	10	71319258	71363385	protein_coding	4,02	0
ENSG00000134278	SPIRE1	18	12446512	12658134	protein_coding	4,02	0
ENSG00000148606	POLR3A	10	77969251	78029545	protein_coding	4,02	0
ENSG00000105983	LMBR1	7	156668946	156893230	protein_coding	4,02	0
ENSG00000164346	NSA2	5	74766991	74780113	protein_coding	4,02	0
ENSG00000114416	FXR1	3	180868141	180982753	protein_coding	4,01	0
ENSG00000129657	SEC14L1	17	77086716	77217101	protein_coding	4,01	0
ENSG00000017260	ATP2C1	3	130850595	131016712	protein_coding	4,00	0
ENSG00000129673	AANAT	17	76453351	76470117	protein_coding	4,00	0
ENSG00000171989	LDHAL6B	15	59206823	59208515	protein_coding	4,00	0
ENSG00000150455	TIRAP	11	126283065	126298845	protein_coding	4,00	0
ENSG00000186051	TAL2	9	105662457	105663112	protein_coding	4,00	0
ENSG00000188152	NUTM2G	9	96928310	96940253	protein_coding	4,00	0
ENSG00000275713	HIST1H2BH	6	26251651	26253710	protein_coding	4,00	0
ENSG00000163032	VSNL1	2	17539126	17657018	protein_coding	4,00	0
ENSG00000187808	SOWAHD	X	119758613	119760164	protein_coding	4,00	0
ENSG00000107104	KANK1	9	470291	746106	protein_coding	4,00	0
ENSG00000101452	DHX35	20	38962299	39039723	protein_coding	3,99	0
ENSG00000163029	SMC6	2	17663812	17800242	protein_coding	3,99	0
ENSG00000122557	HERPUD2	7	35632659	35695571	protein_coding	3,99	0
ENSG00000128833	MYO5C	15	52192322	52295798	protein_coding	3,99	0
ENSG00000104408	EIF3E	8	108201216	108435333	protein_coding	3,98	0
ENSG00000152683	SLC30A6	2	32165841	32224379	protein_coding	3,98	0
ENSG00000153790	C7orf31	7	25134697	25180356	protein_coding	3,98	0
ENSG00000180425	C11orf71	11	114391443	114400550	protein_coding	3,97	0
ENSG00000112159	MDN1	6	89642499	89819723	protein_coding	3,97	0
ENSG00000047621	C12orf4	12	4487728	4538508	protein_coding	3,97	0
ENSG00000124356	STAMBP	2	73828916	73873659	protein_coding	3,96	0
ENSG00000004864	SLC25A13	7	96120220	96322147	protein_coding	3,96	0
ENSG00000119392	GLE1	9	128504700	128542288	protein_coding	3,96	0
ENSG00000054793	ATP9A	20	51596514	51768634	protein_coding	3,95	0
ENSG00000126870	WDR60	7	158856578	158956747	protein_coding	3,95	0
ENSG00000087301	TXNDC16	14	52430590	52552522	protein_coding	3,95	0

ENSG00000134375	TIMM17A	1	201955491	201970661	protein_coding	3,95	0
ENSG00000164181	ELOVL7	5	60751791	60844389	protein_coding	3,94	0
ENSG00000112584	FAM120B	6	170290703	170407065	protein_coding	3,93	0
ENSG00000120910	PPP3CC	8	22440819	22541142	protein_coding	3,93	0
ENSG00000120370	GORAB	1	170532129	170553446	protein_coding	3,93	0
ENSG00000158985	CDC42SE2	5	131245493	131398447	protein_coding	3,92	0
ENSG00000163636	PSMD6	3	64010549	64024010	protein_coding	3,92	0
ENSG00000154447	SH3RF1	4	169094256	169271105	protein_coding	3,92	0
ENSG00000127314	RAP1B	12	68610839	68671901	protein_coding	3,91	0
ENSG00000148411	NACC2	9	136006537	136095285	protein_coding	3,91	0
ENSG00000145723	GIN1	5	103086000	103120151	protein_coding	3,91	0
ENSG00000110696	C11orf58	11	16613132	16756881	protein_coding	3,91	0
ENSG00000164654	MIOS	7	7566872	7608929	protein_coding	3,90	0
ENSG00000239389	PCDHA13	5	140882208	141012344	protein_coding	3,90	0
ENSG00000171943	SRGAP2C	1	121184810	121392822	protein_coding	3,89	0
ENSG00000143612	C1orf43	1	154206706	154220628	protein_coding	3,89	0
ENSG00000121749	TBC1D15	12	71839707	71927248	protein_coding	3,89	0
ENSG00000131697	NPHP4	1	5862811	5992473	protein_coding	3,88	0
ENSG00000100227	POLDIP3	22	42583721	42614962	protein_coding	3,88	0
ENSG00000107929	LARP4B	10	806914	931705	protein_coding	3,88	0
ENSG00000162894	FCMR	1	206904386	206923247	protein_coding	3,88	0
ENSG00000143303	RRNAD1	1	156728442	156736960	protein_coding	3,87	0
ENSG00000143367	TUFT1	1	151540305	151583583	protein_coding	3,87	0
ENSG00000123444	KBTBD4	11	47572197	47579015	protein_coding	3,87	0
ENSG00000126457	PRMT1	19	49675786	49689029	protein_coding	3,87	0
ENSG00000116062	MSH6	2	47695530	47810101	protein_coding	3,87	0
ENSG00000171862	PTEN	10	87863113	87971930	protein_coding	3,86	0
ENSG00000172053	QARS	3	49095932	49105135	protein_coding	3,86	0
ENSG00000135387	CAPRIN1	11	34051683	34101156	protein_coding	3,86	0
ENSG00000244005	NFS1	20	35668055	35699359	protein_coding	3,86	0
ENSG00000156795	WDYHV1	8	123416725	123467230	protein_coding	3,86	0
ENSG00000136560	TANK	2	161136908	161236221	protein_coding	3,85	0
ENSG00000006607	FARP2	2	241356243	241494841	protein_coding	3,85	0
ENSG00000131238	PPT1	1	40072705	40097703	protein_coding	3,85	0
ENSG00000170100	ZNF778	16	89217703	89237071	protein_coding	3,85	0
ENSG00000150991	UBC	12	124911604	124917368	protein_coding	3,85	0
ENSG00000243710	CFAP57	1	43172149	43254358	protein_coding	3,85	0
ENSG00000165699	TSC1	9	132891348	132944633	protein_coding	3,85	0
ENSG00000182150	ERCC6L2	9	95875701	96014571	protein_coding	3,84	0
ENSG00000197714	ZNF460	19	57280051	57293569	protein_coding	3,84	0
ENSG00000161955	TNFSF13	17	7558292	7561608	protein_coding	3,84	0
ENSG00000140526	ABHD2	15	89087459	89202360	protein_coding	3,83	0
ENSG00000060566	CREB3L3	19	4153601	4173054	protein_coding	3,83	0
ENSG00000131089	ARHGEF9	X	63634967	63809274	protein_coding	3,83	0
ENSG00000234444	ZNF736	7	64307459	64354860	protein_coding	3,83	0
ENSG00000072401	UBE2D1	10	58334975	58370753	protein_coding	3,83	0
ENSG00000162600	OMA1	1	58415384	58546802	protein_coding	3,83	0
ENSG00000152208	GRID2	4	92303622	93774556	protein_coding	3,83	0
ENSG00000172661	FAM21C	10	45727200	45792961	protein_coding	3,82	0
ENSG00000134014	ELP3	8	28089673	28191156	protein_coding	3,82	0
ENSG00000135537	LACE1	6	108294894	108526796	protein_coding	3,82	0
ENSG00000174405	LIG4	13	108207439	108218368	protein_coding	3,82	0
ENSG00000137310	TCF19	6	31158542	31167159	protein_coding	3,82	0
ENSG00000145041	VPRBP	3	51395867	51500002	protein_coding	3,81	0
ENSG00000102978	POLR2C	16	57462387	57472010	protein_coding	3,81	0
ENSG00000174032	SLC25A30	13	45393316	45418455	protein_coding	3,81	0
ENSG00000116138	DNAJC16	1	15526813	15592379	protein_coding	3,81	0
ENSG00000197969	VPS13A	9	77177353	77421541	protein_coding	3,80	0

ENSG00000114062	UBE3A	15	25333728	25439056	protein_coding	3,80	0
ENSG00000205929	C21orf62	21	32790674	32813743	protein_coding	3,80	0
ENSG00000204963	PCDHA7	5	140834248	141012344	protein_coding	3,80	0
ENSG00000205572	SERF1B	5	70025247	70043113	protein_coding	3,80	0
ENSG00000118640	VAMP8	2	85561562	85582031	protein_coding	3,80	0
ENSG00000203685	C1orf95	1	226548800	226609214	protein_coding	3,80	0
ENSG00000122644	ARL4A	7	12686856	12690934	protein_coding	3,80	0
ENSG00000065243	PKN2	1	88684222	88836255	protein_coding	3,80	0
ENSG00000118900	UBN1	16	4846665	4882360	protein_coding	3,80	0
ENSG00000081760	AACS	12	125065379	125143333	protein_coding	3,80	0
ENSG00000253719	ATXN7L3B	12	74537827	74545430	protein_coding	3,79	0
ENSG00000144647	POMGNT2	3	43079232	43106076	protein_coding	3,79	0
ENSG00000104980	TIMM44	19	7926718	7943920	protein_coding	3,79	0
ENSG00000124613	ZNF391	6	27374615	27403904	protein_coding	3,79	0
ENSG00000089335	ZNF302	19	34677639	34686397	protein_coding	3,78	0
ENSG00000075413	MARK3	14	103385392	103503831	protein_coding	3,78	0
ENSG00000196787	HIST1H2AG	6	27133042	27135291	protein_coding	3,78	0
ENSG00000125868	DSTN	20	17569863	17609919	protein_coding	3,78	0
ENSG00000181852	RNF41	12	56202175	56221933	protein_coding	3,78	0
ENSG00000099901	RANBP1	22	20115938	20127357	protein_coding	3,78	0
ENSG00000062485	CS	12	56271699	56300392	protein_coding	3,77	0
ENSG00000196323	ZBTB44	11	130226677	130314686	protein_coding	3,77	0
ENSG00000170759	KIF5B	10	32009010	32056431	protein_coding	3,77	0
ENSG00000130348	QRSL1	6	106629578	106668417	protein_coding	3,77	0
ENSG00000144233	AMMECR1L	2	127861630	127885922	protein_coding	3,77	0
ENSG00000120457	KCNJ5	11	128891356	128921035	protein_coding	3,76	0
ENSG00000128585	MKLN1	7	131110096	131496636	protein_coding	3,76	0
ENSG00000177034	MTX3	5	79976731	79991262	protein_coding	3,76	0
ENSG00000172292	CERS6	2	168455862	168775137	protein_coding	3,76	0
ENSG00000102081	FMR1	X	147911951	147951125	protein_coding	3,75	0
ENSG00000196312	HIATL2	9	96898066	97013580	protein_coding	3,75	0
ENSG00000127616	SMARCA4	19	10961001	11065395	protein_coding	3,75	0
ENSG00000198498	TMA16	4	163494442	163520539	protein_coding	3,75	0
ENSG00000004961	HCCS	X	11111301	11123078	protein_coding	3,74	0
ENSG00000144283	PKP4	2	158456964	158682879	protein_coding	3,74	0
ENSG00000103126	AXIN1	16	287440	352673	protein_coding	3,74	0
ENSG00000103160	HSDL1	16	84122146	84145192	protein_coding	3,74	0
ENSG00000174370	C11orf45	11	128899565	128906035	protein_coding	3,74	0
ENSG00000070010	UFD1L	22	19449910	19479215	protein_coding	3,74	0
ENSG00000136152	COG3	13	45464898	45536630	protein_coding	3,74	0
ENSG00000164930	FZD6	8	103298433	103332866	protein_coding	3,74	0
ENSG00000184743	ATL3	11	63624087	63671921	protein_coding	3,73	0
ENSG00000112685	EXOC2	6	485133	693111	protein_coding	3,73	0
ENSG00000156050	FAM161B	14	73931501	73950414	protein_coding	3,73	0
ENSG00000137073	UBAP2	9	33921693	34048949	protein_coding	3,73	0
ENSG00000143727	ACP1	2	264140	278283	protein_coding	3,73	0
ENSG00000178074	C2orf69	2	199911256	199955935	protein_coding	3,73	0
ENSG00000165832	TRUB1	10	114938193	114977676	protein_coding	3,72	0
ENSG00000104356	POP1	8	98117297	98159834	protein_coding	3,72	0
ENSG00000177119	ANO6	12	45215987	45440404	protein_coding	3,72	0
ENSG00000103061	SLC7A6OS	16	68284503	68310951	protein_coding	3,72	0
ENSG00000168234	TTC39C	18	23992773	24135610	protein_coding	3,72	0
ENSG00000176390	CRLF3	17	30769388	30824776	protein_coding	3,72	0
ENSG00000155657	TTN	2	178525989	178830802	protein_coding	3,72	0
ENSG00000164663	USP49	6	41789896	41895361	protein_coding	3,71	0
ENSG00000091483	FH	1	241497603	241519761	protein_coding	3,71	0
ENSG00000168004	HRASLS5	11	63461404	63491194	protein_coding	3,71	0
ENSG00000113838	TBCCD1	3	186546073	186570543	protein_coding	3,71	0

ENSG00000204256	BRD2	6	32968660	32981505	protein_coding	3,70	0
ENSG00000170606	HSPA4	5	133051962	133106449	protein_coding	3,70	0
ENSG00000132692	BCAN	1	156641390	156659532	protein_coding	3,70	0
ENSG00000196152	ZNF79	9	127424374	127445372	protein_coding	3,70	0
ENSG00000213020	ZNF611	19	52702813	52735073	protein_coding	3,70	0
ENSG00000151148	UBE3B	12	109477402	109536705	protein_coding	3,70	0
ENSG00000086848	ALG9	11	111782195	111871581	protein_coding	3,69	0
ENSG00000163161	ERCC3	2	127257290	127294176	protein_coding	3,69	0
ENSG00000174842	GLMN	1	92246402	92298987	protein_coding	3,69	0
ENSG00000138594	TMOD3	15	51829628	51947295	protein_coding	3,69	0
ENSG00000137770	CTDSPL2	15	44427234	44529038	protein_coding	3,68	0
ENSG00000179456	ZBTB18	1	244048939	244057476	protein_coding	3,68	0
ENSG00000151718	WWC2	4	183099293	183320777	protein_coding	3,68	0
ENSG00000168172	HOOK3	8	42896932	43030539	protein_coding	3,68	0
ENSG00000135164	DMTF1	7	87152361	87196337	protein_coding	3,68	0
ENSG00000183671	GPR1	2	206175316	206218047	protein_coding	3,68	0
ENSG00000180787	ZFP3	17	5078248	5096374	protein_coding	3,67	0
ENSG00000214013	GANC	15	42273233	42356935	protein_coding	3,67	0
ENSG00000010292	NCAPD2	12	6493356	6531955	protein_coding	3,67	0
ENSG00000150076	CCDC7	10	32567723	32882874	protein_coding	3,67	0
ENSG00000173041	ZNF680	7	64519884	64563106	protein_coding	3,66	0
ENSG00000160602	NEK8	17	28725897	28743455	protein_coding	3,66	0
ENSG00000100266	PACSIN2	22	42835412	43015145	protein_coding	3,66	0
ENSG00000244754	N4BP2L2	13	32432417	32538885	protein_coding	3,66	0
ENSG00000184047	DIABLO	12	122207662	122227534	protein_coding	3,65	0
ENSG00000158079	PTPDC1	9	94030794	94109856	protein_coding	3,65	0
ENSG00000128881	TTBK2	15	42738734	42920809	protein_coding	3,65	0
ENSG00000159459	UBR1	15	42942897	43106113	protein_coding	3,65	0
ENSG00000143951	WDPCP	2	63121383	63827843	protein_coding	3,65	0
ENSG00000236104	ZBTB22	6	33314406	33317942	protein_coding	3,64	0
ENSG00000081154	PCNP	3	101574095	101594437	protein_coding	3,64	0
ENSG00000142731	PLK4	4	127880861	127899195	protein_coding	3,63	0
ENSG00000101347	SAMHD1	20	36890229	36951843	protein_coding	3,63	0
ENSG00000144711	IQSEC1	3	12897220	13073117	protein_coding	3,63	0
ENSG00000148229	POLE3	9	113407235	113410672	protein_coding	3,63	0
ENSG00000005007	UPF1	19	18831938	18868236	protein_coding	3,63	0
ENSG00000198081	ZBTB14	18	5289019	5297053	protein_coding	3,63	0
ENSG00000175395	ZNF25	10	37949572	37976633	protein_coding	3,63	0
ENSG00000132561	MATN2	8	97868840	98036716	protein_coding	3,63	0
ENSG00000170624	SGCD	5	155870344	156767788	protein_coding	3,62	0
ENSG00000135253	KCP	7	128862451	128910719	protein_coding	3,62	0
ENSG00000135250	SRPK2	7	105110704	105399308	protein_coding	3,61	0
ENSG00000151532	VTI1A	10	112446998	112818744	protein_coding	3,61	0
ENSG00000258405	ZNF578	19	52453576	52512154	protein_coding	3,61	0
ENSG00000005483	KMT2E	7	105014179	105114361	protein_coding	3,61	0
ENSG00000196914	ARHGEF12	11	120336914	120489936	protein_coding	3,60	0
ENSG00000213585	VDAC1	5	133971915	134005133	protein_coding	3,60	0
ENSG00000170776	AKAP13	15	85380571	85749358	protein_coding	3,60	0
ENSG00000162367	TAL1	1	47216290	47232220	protein_coding	3,60	0
ENSG00000198754	OXCT2	1	39769523	39771348	protein_coding	3,60	0
ENSG00000116667	C1orf21	1	184387058	184629020	protein_coding	3,60	0
ENSG00000183283	DAZAP2	12	51238292	51271362	protein_coding	3,59	0
ENSG00000102805	CLN5	13	76990660	77002517	protein_coding	3,59	0
ENSG00000121988	ZRANB3	2	135136916	135531236	protein_coding	3,59	0
ENSG00000168769	TET2	4	105145875	105279816	protein_coding	3,58	0
ENSG00000110514	MADD	11	47269161	47330031	protein_coding	3,58	0
ENSG00000171840	NINJ2	12	564296	663779	protein_coding	3,58	0
ENSG00000104756	KCTD9	8	25427847	25458476	protein_coding	3,58	0

ENSG00000141425	RPRD1A	18	35984387	36067576	protein_coding	3,57	0
ENSG00000158195	WASF2	1	27404226	27490158	protein_coding	3,57	0
ENSG00000180257	ZNF816	19	52949379	52962911	protein_coding	3,57	0
ENSG00000101109	STK4	20	44966474	45079959	protein_coding	3,57	0
ENSG00000006744	ELAC2	17	12992391	13018187	protein_coding	3,57	0
ENSG00000154146	NRGN	11	124739846	124747210	protein_coding	3,57	0
ENSG00000173567	ADGRF3	2	26308173	26346817	protein_coding	3,57	0
ENSG00000133657	ATP13A3	3	194402672	194498364	protein_coding	3,57	0
ENSG00000133256	PDE6B	4	625584	670782	protein_coding	3,56	0
ENSG00000106049	HIBADH	7	27525442	27662995	protein_coding	3,56	0
ENSG00000162695	SLC30A7	1	100896076	100981753	protein_coding	3,56	0
ENSG00000114742	WDR48	3	39051998	39096671	protein_coding	3,56	0
ENSG00000159111	MRPL10	17	47823272	47831534	protein_coding	3,56	0
ENSG00000114904	NEK4	3	52708449	52770949	protein_coding	3,55	0
ENSG00000172167	MTBP	8	120445400	120542133	protein_coding	3,55	0
ENSG00000070785	EIF2B3	1	44850522	44986722	protein_coding	3,54	0
ENSG00000203880	PCMTD2	20	64255695	64287821	protein_coding	3,54	0
ENSG00000011201	ANOS1	X	8528874	8732187	protein_coding	3,54	0
ENSG00000013374	NUB1	7	151341699	151378449	protein_coding	3,54	0
ENSG00000272305	ENSG00000	3	52969119	53099453	protein_coding	3,53	0
ENSG00000185418	TARSL2	15	101653598	101724604	protein_coding	3,53	0
ENSG00000172062	SMN1	5	70925030	70953942	protein_coding	3,53	0
ENSG00000076513	ANKRD13A	12	109999186	110039763	protein_coding	3,53	0
ENSG00000251287	ALG1L2	3	130081831	130113227	protein_coding	3,52	0
ENSG00000121879	PIK3CA	3	179148114	179240093	protein_coding	3,52	0
ENSG00000160908	ZNF394	7	99486519	99500324	protein_coding	3,52	0
ENSG00000162616	DNAJB4	1	77979175	78017964	protein_coding	3,52	0
ENSG00000244462	RBM12	20	35648925	35664956	protein_coding	3,52	0
ENSG00000114520	SNX4	3	125446644	125520197	protein_coding	3,52	0
ENSG00000163050	ADCK3	1	226897536	226987545	protein_coding	3,52	0
ENSG00000188467	SLC24A5	15	48120972	48142672	protein_coding	3,51	0
ENSG00000105053	VRK3	19	49976467	50025946	protein_coding	3,51	0
ENSG00000101752	MIB1	18	21704957	21870957	protein_coding	3,51	0
ENSG00000196937	FAM3C	7	121348851	121396364	protein_coding	3,51	0
ENSG00000134313	KIDINS220	2	8725278	8837630	protein_coding	3,51	0
ENSG00000167984	NLRC3	16	3539033	3577400	protein_coding	3,51	0
ENSG00000177692	DNAJC28	21	33485530	33491720	protein_coding	3,50	0
ENSG00000249428	CFAP99	4	2426476	2462926	protein_coding	3,50	0
ENSG00000163568	AIM2	1	159062484	159147096	protein_coding	3,50	0
ENSG00000180957	PITPNB	22	27851669	27920134	protein_coding	3,50	0
ENSG00000136404	TM6SF1	15	83107407	83144854	protein_coding	3,50	0
ENSG00000131781	FMO5	1	147175351	147243050	protein_coding	3,49	0
ENSG00000134265	NAPG	18	10525905	10552761	protein_coding	3,49	0
ENSG00000066084	DIP2B	12	50504985	50748667	protein_coding	3,49	0
ENSG00000163781	TOPBP1	3	133598175	133661893	protein_coding	3,48	0
ENSG00000064999	ANKS1A	6	34889265	35091413	protein_coding	3,48	0
ENSG00000147099	HDAC8	X	72329516	72573103	protein_coding	3,48	0
ENSG00000149231	CCDC82	11	96352769	96389923	protein_coding	3,48	0
ENSG00000137713	PPP2R1B	11	111726908	111766427	protein_coding	3,48	0
ENSG00000101193	GID8	20	62938119	62948475	protein_coding	3,47	0
ENSG00000214517	PPME1	11	74171099	74254703	protein_coding	3,47	0
ENSG00000197603	C5orf42	5	37106228	37249428	protein_coding	3,47	0
ENSG00000108064	TFAM	10	58385022	58399221	protein_coding	3,47	0
ENSG00000089022	MAPKAPK5	12	111841978	111902238	protein_coding	3,47	0
ENSG00000104067	TJP1	15	29699367	29968865	protein_coding	3,47	0
ENSG00000091039	OSBPL8	12	76351797	76559809	protein_coding	3,46	0
ENSG00000125304	TM9SF2	13	99501417	99564006	protein_coding	3,46	0
ENSG00000108578	BLMH	17	30248195	30292056	protein_coding	3,46	0

ENSG00000169641	LUZP1	1	23084023	23177808	protein_coding	3,45	0
ENSG00000107821	KAZALD1	10	101061841	101068131	protein_coding	3,45	0
ENSG00000174780	SRP72	4	56466915	56503680	protein_coding	3,45	0
ENSG00000183576	SETD3	14	99397746	99480889	protein_coding	3,45	0
ENSG00000011114	BTBD7	14	93237550	93333092	protein_coding	3,45	0
ENSG00000213281	NRAS	1	114704469	114716894	protein_coding	3,44	0
ENSG00000090447	TFAP4	16	4257186	4273075	protein_coding	3,43	0
ENSG00000108312	UBTF	17	44205033	44221626	protein_coding	3,43	0
ENSG00000116750	UCHL5	1	193012250	193060080	protein_coding	3,43	0
ENSG00000079156	OSBPL6	2	178194481	178402891	protein_coding	3,43	0
ENSG00000196724	ZNF418	19	57921884	57935393	protein_coding	3,42	0
ENSG00000167384	ZNF180	19	44474428	44500524	protein_coding	3,42	0
ENSG00000064651	SLC12A2	5	128083766	128189688	protein_coding	3,42	0
ENSG00000166130	IKBIP	12	98613405	98645113	protein_coding	3,42	0
ENSG00000128513	POT1	7	124822386	124929983	protein_coding	3,42	0
ENSG00000168827	GFM1	3	158644278	158692575	protein_coding	3,42	0
ENSG00000163689	C3orf67	3	58717365	59050084	protein_coding	3,41	0
ENSG00000056097	ZFR	5	32354350	32444761	protein_coding	3,41	0
ENSG00000164074	ABHD18	4	127965306	128039711	protein_coding	3,41	0
ENSG00000278053	DDX52	17	37609739	37643464	protein_coding	3,41	0
ENSG00000268043	NBPF12	1	146938744	146996202	protein_coding	3,41	0
ENSG00000067900	ROCK1	18	20946906	21111851	protein_coding	3,41	0
ENSG00000144455	SUMF1	3	3700814	4467281	protein_coding	3,41	0
ENSG00000196369	SRGAP2B	1	144887265	145095528	protein_coding	3,40	0
ENSG00000145103	ILDR1	3	121987323	122022204	protein_coding	3,40	0
ENSG00000158604	TMED4	7	44577894	44582287	protein_coding	3,40	0
ENSG00000160310	PRMT2	21	46635167	46665124	protein_coding	3,40	0
ENSG00000139218	SCAF11	12	45919131	45992120	protein_coding	3,40	0
ENSG00000160199	PKNOX1	21	42974510	43033931	protein_coding	3,39	0
ENSG00000197557	TTC30A	2	177612992	177618966	protein_coding	3,39	0
ENSG00000102974	CTCF	16	67562407	67639183	protein_coding	3,38	0
ENSG00000097033	SH3GLB1	1	86704570	86748184	protein_coding	3,38	0
ENSG00000153317	ASAP1	8	130052104	130443660	protein_coding	3,38	0
ENSG00000140694	PARN	16	14435701	14632728	protein_coding	3,38	0
ENSG00000070087	PFN2	3	149964904	150050788	protein_coding	3,38	0
ENSG00000196715	VKORC1L1	7	65873267	65959563	protein_coding	3,38	0
ENSG00000145545	SRD5A1	5	6633343	6674386	protein_coding	3,37	0
ENSG00000122696	SLC25A51	9	37879400	37904353	protein_coding	3,37	0
ENSG00000111790	FGFR1OP2	12	26938383	26966650	protein_coding	3,37	0
ENSG00000156232	WHAMM	15	82809628	82836108	protein_coding	3,37	0
ENSG00000167881	SRP68	17	76038775	76072653	protein_coding	3,36	0
ENSG00000164506	STXBP5	6	147204425	147390476	protein_coding	3,36	0
ENSG00000147649	MTDH	8	97644179	97728770	protein_coding	3,35	0
ENSG00000159023	EPB41	1	28887091	29120046	protein_coding	3,35	0
ENSG00000081087	OSTM1	6	108041409	108165854	protein_coding	3,35	0
ENSG00000066739	ATG2B	14	96279202	96363870	protein_coding	3,34	0
ENSG00000136891	TEX10	9	100302077	100352939	protein_coding	3,34	0
ENSG00000104450	SPAG1	8	100157906	100259278	protein_coding	3,34	0
ENSG00000153956	CACNA2D1	7	81946444	82443798	protein_coding	3,33	0
ENSG00000179152	TCAIM	3	44338119	44409451	protein_coding	3,33	0
ENSG00000085185	BCORL1	X	129981107	130058083	protein_coding	3,33	0
ENSG00000197566	ZNF624	17	16620737	16653856	protein_coding	3,33	0
ENSG00000138593	SECISBP2L	15	48988476	49046563	protein_coding	3,32	0
ENSG00000175792	RUVBL1	3	128064778	128153914	protein_coding	3,32	0
ENSG00000141298	SSH2	17	29625938	29930276	protein_coding	3,32	0
ENSG00000156531	PHF6	X	134373253	134428791	protein_coding	3,32	0
ENSG00000112977	DAP	5	10679230	10761272	protein_coding	3,31	0
ENSG00000168385	09/01/2002	2	241315100	241354027	protein_coding	3,31	0

ENSG00000133119	RFC3	13	33818049	33966558	protein_coding	3,31	0
ENSG00000008952	SEC62	3	169966635	169998373	protein_coding	3,31	0
ENSG00000197951	ZNF71	19	56595264	56626481	protein_coding	3,31	0
ENSG00000133059	DSTYK	1	205142505	205211566	protein_coding	3,31	0
ENSG00000083123	BCKDHB	6	80106647	80346270	protein_coding	3,30	0
ENSG00000062282	DGAT2	11	75759512	75801535	protein_coding	3,30	0
ENSG00000140199	SLC12A6	15	34229996	34338060	protein_coding	3,30	0
ENSG00000115365	LANCL1	2	210431249	210477652	protein_coding	3,30	0
ENSG00000104894	CD37	19	49335171	49343335	protein_coding	3,29	0
ENSG00000179562	GCC1	7	127580618	127593611	protein_coding	3,29	0
ENSG00000147251	DOCK11	X	118495898	118686163	protein_coding	3,28	0
ENSG00000100335	MIEF1	22	39499432	39518132	protein_coding	3,28	0
ENSG00000116747	TROVE2	1	193059422	193091777	protein_coding	3,28	0
ENSG00000052723	SIKE1	1	114769479	114780685	protein_coding	3,28	0
ENSG00000156802	ATAD2	8	123319850	123416350	protein_coding	3,27	0
ENSG00000111696	NT5DC3	12	103770453	103841197	protein_coding	3,27	0
ENSG00000138411	HECW2	2	196194370	196593692	protein_coding	3,27	0
ENSG00000106771	TMEM245	9	109015152	109119945	protein_coding	3,27	0
ENSG00000254986	DPP3	11	66480013	66509657	protein_coding	3,27	0
ENSG00000144736	SHQ1	3	72749277	72861914	protein_coding	3,26	0
ENSG00000111670	GNPTAB	12	101745497	101830938	protein_coding	3,26	0
ENSG00000103769	RAB11A	15	65726054	65891991	protein_coding	3,26	0
ENSG00000171777	RASGRP4	19	38409051	38426305	protein_coding	3,25	0
ENSG00000122335	SERAC1	6	158109515	158168270	protein_coding	3,25	0
ENSG00000136451	VEZF1	17	57971547	57988259	protein_coding	3,25	0
ENSG00000106070	GRB10	7	50590063	50793462	protein_coding	3,25	0
ENSG00000143850	PLEKHA6	1	204218851	204377665	protein_coding	3,25	0
ENSG00000103888	CEMIP	15	80779343	80951776	protein_coding	3,25	0
ENSG00000152492	CCDC50	3	191329077	191398670	protein_coding	3,25	0
ENSG00000114544	SLC41A3	3	126006355	126101561	protein_coding	3,25	0
ENSG00000078369	GNB1	1	1785285	1891117	protein_coding	3,24	0
ENSG00000143374	TARS2	1	150487364	150507609	protein_coding	3,24	0
ENSG00000113851	CRBN	3	3148992	3179710	protein_coding	3,23	0
ENSG00000214367	HAUS3	4	2227464	2242164	protein_coding	3,23	0
ENSG00000181467	RAP2B	3	153162270	153167173	protein_coding	3,23	0
ENSG00000135541	AHI1	6	135283532	135497776	protein_coding	3,23	0
ENSG00000149313	AASDHPPT	11	106075501	106098710	protein_coding	3,23	0
ENSG00000154265	ABCA5	17	69244311	69327244	protein_coding	3,22	0
ENSG00000203814	HIST2H2BF	1	149782689	149812373	protein_coding	3,22	0
ENSG00000108262	GIT1	17	29573469	29594054	protein_coding	3,21	0
ENSG00000146350	TBC1D32	6	121079494	121334745	protein_coding	3,21	0
ENSG00000183605	SFXN4	10	119140767	119165667	protein_coding	3,20	0
ENSG00000172399	MYOZ2	4	119135784	119187789	protein_coding	3,20	0
ENSG00000143742	SRP9	1	225777813	225790466	protein_coding	3,18	0
ENSG00000115840	SLC25A12	2	171784370	171999859	protein_coding	3,18	0
ENSG00000140575	IQGAP1	15	90388218	90502243	protein_coding	3,17	0
ENSG00000198373	WWP2	16	69762306	69941741	protein_coding	3,17	0
ENSG00000117475	BLZF1	1	169367970	169396540	protein_coding	3,17	0
ENSG00000011523	CEP68	2	65056366	65087004	protein_coding	3,17	0
ENSG00000091592	NLRP1	17	5499427	5619424	protein_coding	3,17	0
ENSG00000137992	DBT	1	100186919	100249834	protein_coding	3,16	0
ENSG00000152102	FAM168B	2	131047876	131093460	protein_coding	3,16	0
ENSG00000237765	FAM200B	4	15681662	15705565	protein_coding	3,16	0
ENSG00000138381	ASNSD1	2	189661385	189670831	protein_coding	3,16	0
ENSG00000167767	KRT80	12	52168996	52192000	protein_coding	3,16	0
ENSG00000143970	ASXL2	2	25733753	25878516	protein_coding	3,15	0
ENSG00000160746	ANO10	3	43354859	43691594	protein_coding	3,15	0
ENSG00000121481	RNF2	1	185045364	185102608	protein_coding	3,14	0

ENSG00000112996	MRPS30	5	44808925	44820428	protein_coding	3,14	0
ENSG00000145414	NAF1	4	163110073	163166921	protein_coding	3,14	0
ENSG00000100364	KIAA0930	22	45190338	45240769	protein_coding	3,14	0
ENSG00000188827	SLX4	16	3581181	3611598	protein_coding	3,14	0
ENSG00000114978	MOB1A	2	74152528	74178898	protein_coding	3,13	0
ENSG00000124575	HIST1H1D	6	26234268	26234933	protein_coding	3,13	0
ENSG00000143458	GABPB2	1	151070578	151125542	protein_coding	3,13	0
ENSG00000142208	AKT1	14	104769349	104795751	protein_coding	3,13	0
ENSG00000164944	KIAA1429	8	94487693	94553529	protein_coding	3,12	0
ENSG00000139174	PRICKLE1	12	42456754	42590355	protein_coding	3,12	0
ENSG00000104643	MTMR9	8	11284416	11328146	protein_coding	3,12	0
ENSG00000051825	MPHOSPH9	12	123152320	123244014	protein_coding	3,11	0
ENSG00000115266	APC2	19	1446302	1473244	protein_coding	3,11	0
ENSG00000188994	ZNF292	6	87152833	87264196	protein_coding	3,11	0
ENSG00000168795	ZBTB5	9	37438114	37465399	protein_coding	3,11	0
ENSG00000198863	RUNDC1	17	42980565	42993690	protein_coding	3,11	0
ENSG00000166479	TMX3	18	68673688	68715298	protein_coding	3,10	0
ENSG00000163285	GABRG1	4	46035769	46124081	protein_coding	3,10	0
ENSG00000186073	C15orf41	15	36579611	36810248	protein_coding	3,09	0
ENSG00000134186	PRPF38B	1	108692323	108701803	protein_coding	3,09	0
ENSG00000174953	DHX36	3	154272546	154324497	protein_coding	3,08	0
ENSG00000138756	BMP2K	4	78776342	78916372	protein_coding	3,08	0
ENSG00000166454	ATMIN	16	81035847	81047358	protein_coding	3,08	0
ENSG00000120063	GNA13	17	65010715	65056839	protein_coding	3,07	0
ENSG00000182134	TDRKH	1	151770107	151791416	protein_coding	3,07	0
ENSG00000169989	TIGD4	4	152769354	152779764	protein_coding	3,06	0
ENSG00000102763	VWA8	13	41566837	41961120	protein_coding	3,06	0
ENSG00000166889	PATL1	11	59636716	59668980	protein_coding	3,06	0
ENSG00000163939	PBRM1	3	52545352	52685917	protein_coding	3,05	0
ENSG00000064199	SPA17	11	124673798	124697518	protein_coding	3,05	0
ENSG00000129515	SNX6	14	34561094	34630183	protein_coding	3,05	0
ENSG00000110075	PPP6R3	11	68460731	68615334	protein_coding	3,04	0
ENSG00000116685	KIAA2013	1	11919591	11926428	protein_coding	3,04	0
ENSG00000140548	ZNF710	15	90001392	90082206	protein_coding	3,04	0
ENSG00000119636	BBOF1	14	74019353	74082863	protein_coding	3,03	0
ENSG00000131504	DIAPH1	5	141515016	141619055	protein_coding	3,02	0
ENSG00000121440	PDZRN3	3	73382433	73624940	protein_coding	3,02	0
ENSG00000136021	SCYL2	12	100267140	100341724	protein_coding	3,02	0
ENSG00000119321	FKBP15	9	113161006	113221361	protein_coding	3,01	0
ENSG00000115568	ZNF142	2	218637916	218659655	protein_coding	3,01	0
ENSG00000019995	ZRANB1	10	124942123	124988189	protein_coding	3,00	0
ENSG00000196350	ZNF729	19	22286408	22317176	protein_coding	3,00	0
ENSG00000175426	PCSK1	5	96390415	96434143	protein_coding	3,00	0
ENSG00000138685	FGF2	4	122826708	122898236	protein_coding	3,00	0
ENSG00000178385	PLEKHM3	2	207828303	208025560	protein_coding	2,99	0
ENSG00000163697	APBB2	4	40810027	41216714	protein_coding	2,99	0
ENSG00000115998	C2orf42	2	70149880	70248615	protein_coding	2,98	0
ENSG00000118217	ATF6	1	161766294	161964070	protein_coding	2,98	0
ENSG00000005175	RPAP3	12	47661249	47706061	protein_coding	2,96	0
ENSG00000100697	DICER1	14	95086228	95158010	protein_coding	2,96	0
ENSG00000133114	GPALPP1	13	44989529	45037669	protein_coding	2,96	0
ENSG00000152457	DCLRE1C	10	14897359	14954432	protein_coding	2,94	0
ENSG00000103479	RBL2	16	53433977	53491649	protein_coding	2,94	0
ENSG00000029725	RABEP1	17	5282265	5385812	protein_coding	2,93	0
ENSG00000100462	PRMT5	14	22920511	22929585	protein_coding	2,93	0
ENSG00000136040	PLXNC1	12	94148723	94307675	protein_coding	2,93	0
ENSG00000084733	RAB10	2	26034107	26137454	protein_coding	2,93	0
ENSG00000151376	ME3	11	86441108	86672636	protein_coding	2,93	0

ENSG00000145390	USP53	4	119212587	119295517	protein_coding	2,92	0
ENSG00000198053	SIRPA	20	1894167	1940592	protein_coding	2,92	0
ENSG00000069966	GNB5	15	52115105	52191369	protein_coding	2,92	0
ENSG00000160218	TRAPPC10	21	44012319	44106552	protein_coding	2,92	0
ENSG00000124198	ARFGEF2	20	48921890	49036693	protein_coding	2,91	0
ENSG00000136169	SETDB2	13	49444374	49495003	protein_coding	2,91	0
ENSG00000103811	CTSH	15	78921058	78949574	protein_coding	2,90	0
ENSG00000119927	GPAM	10	112149864	112215377	protein_coding	2,90	0
ENSG00000187325	TAF9B	X	78129748	78139706	protein_coding	2,90	0
ENSG00000105514	RAB3D	19	11322046	11346270	protein_coding	2,90	0
ENSG00000127946	HIP1	7	75533300	75738962	protein_coding	2,90	0
ENSG00000148110	HIATL1	9	94374551	94461042	protein_coding	2,90	0
ENSG00000147164	SNX12	X	71056332	71073426	protein_coding	2,89	0
ENSG00000081665	ZNF506	19	19785839	19821751	protein_coding	2,89	0
ENSG00000083642	PDS5B	13	32586427	32778019	protein_coding	2,89	0
ENSG00000121390	PSPC1	13	19674752	19783019	protein_coding	2,89	0
ENSG00000032742	IFT88	13	20567069	20691437	protein_coding	2,89	0
ENSG00000161813	LARP4	12	50392383	50480004	protein_coding	2,88	0
ENSG00000132294	EFR3A	8	131904088	132013642	protein_coding	2,88	0
ENSG00000061987	MON2	12	62466817	62600479	protein_coding	2,87	0
ENSG00000145675	PIK3R1	5	68215720	68301821	protein_coding	2,87	0
ENSG00000130939	UBE4B	1	10032832	10181239	protein_coding	2,87	0
ENSG00000197050	ZNF420	19	37007857	37130314	protein_coding	2,87	0
ENSG00000124486	USP9X	X	41085635	41236579	protein_coding	2,85	0
ENSG00000245680	ZNF585B	19	37181579	37218153	protein_coding	2,84	0
ENSG00000115524	SF3B1	2	197389784	197435091	protein_coding	2,84	0
ENSG00000168778	TCTN2	12	123671113	123708403	protein_coding	2,83	0
ENSG00000120333	MRPS14	1	175010789	175023425	protein_coding	2,83	0
ENSG00000254685	FPGT	1	74198212	74234086	protein_coding	2,82	0
ENSG00000115942	ORC2	2	200908973	200963680	protein_coding	2,81	0
ENSG00000248919	ATP5J2-PTCI	7	99419749	99466197	protein_coding	2,81	0
ENSG00000136867	SLC31A2	9	113150942	113164137	protein_coding	2,81	0
ENSG00000157106	SMG1	16	18804853	18926454	protein_coding	2,80	0
ENSG00000126773	PCNXL4	14	60091911	60169133	protein_coding	2,79	0
ENSG00000082146	STRADB	2	201387858	201480846	protein_coding	2,79	0
ENSG00000075568	TMEM131	2	97756333	97995891	protein_coding	2,79	0
ENSG00000136536	07-mars	2	159712457	159771027	protein_coding	2,78	0
ENSG00000156521	TYSND1	10	70137981	70146676	protein_coding	2,77	0
ENSG00000146414	SHPRH	6	145864245	145964423	protein_coding	2,77	0
ENSG00000064313	TAF2	8	119730775	119832863	protein_coding	2,77	0
ENSG00000150867	PIP4K2A	10	22534849	22714555	protein_coding	2,77	0
ENSG00000084093	REST	4	56907876	56935844	protein_coding	2,77	0
ENSG00000198146	ZNF770	15	34978341	34988287	protein_coding	2,76	0
ENSG00000085982	USP40	2	233475520	233566782	protein_coding	2,76	0
ENSG00000114331	ACAP2	3	195274736	195443078	protein_coding	2,75	0
ENSG00000253797	UTP14C	13	52024691	52033600	protein_coding	2,75	0
ENSG00000023287	RB1CC1	8	52622456	52745843	protein_coding	2,74	0
ENSG00000144426	NBEAL1	2	203014879	203226378	protein_coding	2,73	0
ENSG00000160551	TAOK1	17	29390464	29551904	protein_coding	2,73	0
ENSG00000152240	HAUS1	18	46104332	46128333	protein_coding	2,73	0
ENSG00000212916	MAP10	1	232804892	232808407	protein_coding	2,73	0
ENSG00000101337	TM9SF4	20	32109506	32167258	protein_coding	2,72	0
ENSG00000171723	GPHN	14	66507407	67181803	protein_coding	2,72	0
ENSG00000100393	EP300	22	41091786	41180079	protein_coding	2,71	0
ENSG00000131149	GSE1	16	85611409	85676204	protein_coding	2,70	0
ENSG00000221910	OR2F2	7	143935166	143936279	protein_coding	2,70	0
ENSG00000170190	SLC16A5	17	75087727	75106162	protein_coding	2,69	0
ENSG00000088387	DOCK9	13	98793487	99086625	protein_coding	2,69	0

ENSG00000168936	TMEM129	4	1715952	1721358	protein_coding	2,69	0
ENSG00000109171	SLAIN2	4	48341322	48426212	protein_coding	2,68	0
ENSG00000123066	MED13L	12	115953872	116277338	protein_coding	2,67	0
ENSG00000034677	RNF19A	8	100257059	100410015	protein_coding	2,67	0
ENSG00000107518	ATRNL1	10	115093365	115948992	protein_coding	2,66	0
ENSG00000124789	NUP153	6	17615035	17706834	protein_coding	2,66	0
ENSG00000169891	REPS2	X	16946691	17153280	protein_coding	2,66	0
ENSG00000197008	ZNF138	7	64794388	64833681	protein_coding	2,66	0
ENSG00000258102	MAP1LC3B2	12	116548105	116576448	protein_coding	2,65	0
ENSG00000115839	RAB3GAP1	2	135052265	135176394	protein_coding	2,64	0
ENSG00000147133	TAF1	X	71366239	71532374	protein_coding	2,63	0
ENSG00000251369	ZNF550	19	57535257	57559863	protein_coding	2,62	0
ENSG00000070778	PTPN21	14	88465778	88554733	protein_coding	2,62	0
ENSG00000145715	RASA1	5	87267888	87391931	protein_coding	2,61	0
ENSG00000145555	MYO10	5	16661914	16936276	protein_coding	2,61	0
ENSG00000113296	THBS4	5	79991311	80083287	protein_coding	2,60	0
ENSG00000162927	PUS10	2	60940222	61018259	protein_coding	2,60	0
ENSG00000187164	SHTN1	10	116881482	117126586	protein_coding	2,60	0
ENSG00000178338	ZNF354B	5	178859953	178888122	protein_coding	2,60	0
ENSG00000126883	NUP214	9	131125561	131234670	protein_coding	2,60	0
ENSG00000078070	MCCC1	3	183015218	183116075	protein_coding	2,59	0
ENSG00000134954	ETS1	11	128458761	128587558	protein_coding	2,59	0
ENSG00000253251	ENSG00000[illegible]	5	65624765	65630891	protein_coding	2,59	0
ENSG00000269556	TMEM185A	X	149596556	149631912	protein_coding	2,58	0
ENSG00000163322	FAM175A	4	83459517	83523348	protein_coding	2,58	0
ENSG00000164163	ABCE1	4	145097932	145129179	protein_coding	2,58	0
ENSG00000061337	LZTS1	8	20246165	20303963	protein_coding	2,58	0
ENSG00000103994	ZNF106	15	42412823	42491123	protein_coding	2,57	0
ENSG00000144712	CAND2	3	12796472	12871916	protein_coding	2,57	0
ENSG00000198919	DZIP3	3	108589682	108694846	protein_coding	2,57	0
ENSG00000101901	ALG13	X	111665811	111760649	protein_coding	2,56	0
ENSG00000050748	MAPK9	5	180233143	180292099	protein_coding	2,56	0
ENSG00000227124	ZNF717	3	75709643	75785583	protein_coding	2,56	0
ENSG00000028116	VRK2	2	57907651	58159920	protein_coding	2,56	0
ENSG00000136051	KIAA1033	12	105107324	105169134	protein_coding	2,56	0
ENSG00000159873	CCDC117	22	28772674	28789301	protein_coding	2,56	0
ENSG00000135018	UBQLN1	9	83659963	83708203	protein_coding	2,55	0
ENSG00000154305	MIA3	1	222618086	222668012	protein_coding	2,55	0
ENSG00000070367	EXOC5	14	57200507	57269008	protein_coding	2,54	0
ENSG00000178537	SLC25A20	3	48856931	48898993	protein_coding	2,53	0
ENSG00000100503	NIN	14	50719763	50831121	protein_coding	2,53	0
ENSG00000121957	GPSM2	1	108875350	108934545	protein_coding	2,53	0
ENSG00000089159	PXN	12	120210439	120265771	protein_coding	2,53	0
ENSG00000172493	AFF1	4	86935002	87141054	protein_coding	2,52	0
ENSG00000092820	EZR	6	158765741	158819412	protein_coding	2,51	0
ENSG00000158186	MRAS	3	138347648	138405534	protein_coding	2,51	0
ENSG00000143442	POGZ	1	151402724	151459465	protein_coding	2,51	0
ENSG00000174796	THAP6	4	75513946	75550473	protein_coding	2,50	0
ENSG00000125945	ZNF436	1	23359448	23369442	protein_coding	2,50	0
ENSG00000197535	MYO5A	15	52307283	52529050	protein_coding	2,50	0
ENSG00000115306	SPTBN1	2	54456285	54671445	protein_coding	2,49	0
ENSG00000164494	PDSS2	6	107152557	107459564	protein_coding	2,49	0
ENSG00000139197	PEX5	12	7188685	7218574	protein_coding	2,48	0
ENSG00000085365	SCAMP1	5	78360583	78479071	protein_coding	2,48	0
ENSG00000176222	ZNF404	19	43872363	43901385	protein_coding	2,47	0
ENSG00000263956	NBPF11	1	148102046	148152322	protein_coding	2,47	0
ENSG00000048649	RSF1	11	77659996	77821017	protein_coding	2,47	0
ENSG00000169519	METTL15	11	28108248	28527041	protein_coding	2,46	0

ENSG00000198399	ITSN2	2	24202864	24360714	protein_coding	2,46	0
ENSG00000117625	RCOR3	1	211258377	211316385	protein_coding	2,46	0
ENSG00000198093	ZNF649	19	51889224	51905040	protein_coding	2,45	0
ENSG00000175029	CTBP2	10	124984317	125161170	protein_coding	2,45	0
ENSG00000115966	ATF2	2	175072250	175168382	protein_coding	2,44	0
ENSG00000110768	GTF2H1	11	18322295	18367044	protein_coding	2,44	0
ENSG00000146950	SHROOM2	X	9786456	9949443	protein_coding	2,43	0
ENSG00000003393	ALS2	2	201700554	201781189	protein_coding	2,43	0
ENSG00000100426	ZBED4	22	49853842	49890078	protein_coding	2,43	0
ENSG00000119401	TRIM32	9	116687302	116701300	protein_coding	2,43	0
ENSG00000170456	DENND5B	12	31382223	31591097	protein_coding	2,43	0
ENSG00000196670	ZFP62	5	180847611	180861285	protein_coding	2,43	0
ENSG00000163959	SLC51A	3	196211487	196243178	protein_coding	2,42	0
ENSG00000169826	CSGALNACT	10	43138486	43185308	protein_coding	2,41	0
ENSG00000127980	PEX1	7	92487020	92528531	protein_coding	2,41	0
ENSG00000138796	HADH	4	107989714	108035175	protein_coding	2,41	0
ENSG00000154721	JAM2	21	25639272	25717562	protein_coding	2,41	0
ENSG00000004487	KDM1A	1	23019448	23083689	protein_coding	2,40	0
ENSG00000094975	SUCO	1	172532349	172611833	protein_coding	2,39	0
ENSG00000143207	RFWD2	1	175944831	176207493	protein_coding	2,39	0
ENSG00000115760	BIRC6	2	32357028	32618899	protein_coding	2,37	0
ENSG00000154174	TOMM70A	3	100363431	100401398	protein_coding	2,37	0
ENSG00000196712	NF1	17	31094927	31382116	protein_coding	2,36	0
ENSG00000072501	SMC1A	X	53374149	53422728	protein_coding	2,34	0
ENSG00000085224	ATRX	X	77504878	77786269	protein_coding	2,31	0
ENSG00000153827	TRIP12	2	229763838	229923239	protein_coding	2,31	0
ENSG00000116675	DNAJC6	1	65248219	65415869	protein_coding	2,31	0
ENSG00000118260	CREB1	2	207529737	207603431	protein_coding	2,29	0
ENSG00000138138	ATAD1	10	87751512	87841343	protein_coding	2,28	0
ENSG00000133739	LRRCC1	8	85107147	85146076	protein_coding	2,28	0
ENSG00000143493	INTS7	1	211940399	212035542	protein_coding	2,28	0
ENSG00000112367	FIG4	6	109691312	109825428	protein_coding	2,28	0
ENSG00000175455	CCDC14	3	123897305	123961408	protein_coding	2,27	0
ENSG00000163964	PIGX	3	196639775	196736007	protein_coding	2,24	0
ENSG00000213079	SCAF8	6	154733325	154834244	protein_coding	2,23	0
ENSG00000116984	MTR	1	236795281	236903981	protein_coding	2,22	0
ENSG00000120868	APAF1	12	98645141	98735433	protein_coding	2,21	0
ENSG00000135837	CEP350	1	179954738	180114880	protein_coding	2,21	0
ENSG00000132953	XPO4	13	20777329	20903048	protein_coding	2,21	0
ENSG00000113658	SMAD5	5	136132845	136188747	protein_coding	2,21	0
ENSG00000099290	FAM21A	10	50067888	50133506	protein_coding	2,20	0
ENSG00000101310	SEC23B	20	18507493	18561415	protein_coding	2,20	0
ENSG00000180815	MAP3K15	X	19360056	19515261	protein_coding	2,20	0
ENSG00000124207	CSE1L	20	49046246	49096960	protein_coding	2,20	0
ENSG00000155313	USP25	21	15730025	15880069	protein_coding	2,18	0
ENSG00000196505	GDAP2	1	117863485	117929630	protein_coding	2,17	0
ENSG00000143776	CDC42BPA	1	226989865	227318474	protein_coding	2,17	0
ENSG00000171928	TVP23B	17	18780995	18806714	protein_coding	2,17	0
ENSG00000113569	NUP155	5	37288137	37371181	protein_coding	2,12	0
ENSG00000183495	EP400	12	131949920	132081102	protein_coding	2,12	0
ENSG00000171681	ATF7IP	12	14365632	14502935	protein_coding	2,11	0
ENSG00000103657	HERC1	15	63608618	63833942	protein_coding	2,10	0
ENSG00000154727	GABPA	21	25734570	25772460	protein_coding	2,10	0
ENSG00000153832	FBXO36	2	229922302	230013109	protein_coding	2,09	0
ENSG00000105866	SP4	7	21428034	21514822	protein_coding	2,08	0
ENSG00000115355	CCDC88A	2	55287842	55419921	protein_coding	2,08	0
ENSG00000174718	KIAA1551	12	31959370	31993107	protein_coding	2,03	0
ENSG00000170949	ZNF160	19	53066606	53103436	protein_coding	2,02	0

Supplementary Table S3: List of the 2,021 lncRNAs upregulated in MII oocytes

Gene ID	Gene Name	Chromo	Start_position	End_position	Gene_biotype	Fold Change	q-value(%)
ENSG00000261812	TUBB8P7	16	90093154	90096354	transcribed_unproces	71259,46	0
ENSG00000214788	OOSP1	11	59938455	59995845	unitary_pseudogene	43555,92	0
ENSG00000188831	DPPA3P2	14	36371165	36372383	transcribed_unproces	24554,10	0
ENSG00000214324	C3orf56	3	127193131	127198185	lincRNA	19086,80	0
ENSG00000173213	TUBB8P12	18	47390	49557	transcribed_unproces	18506,17	0
ENSG00000251297	TUBB7P	4	189982523	189984871	unprocessed_pseudog	15447,50	0
ENSG00000159247	TUBBP5	9	138150075	138177433	transcribed_unproces	15149,50	0
ENSG00000262117	BCAR4	16	11819829	11828845	lincRNA	14488,17	0
ENSG00000184617	ZNF840P	20	46484461	46492640	unprocessed_pseudog	9756,20	0
ENSG00000253585	ENSG00000253585	8	94097764	94104322	lincRNA	5380,60	0
ENSG00000250447	ENSG00000250447	5	53776644	53819686	lincRNA	4265,50	0
ENSG00000227154	ENSG00000227154	6	35443044	35449866	unprocessed_pseudog	4016,35	0
ENSG00000256340	ABCC6P1	16	18571162	18598328	transcribed_unproces	3521,40	0
ENSG00000222005	LINC01118	2	46816668	46822657	lincRNA	3359,92	0
ENSG00000229251	HNRNPA1P8	7	84983556	84984506	processed_pseudoger	3235,67	0
ENSG00000250788	TUBB8P4	12	34164773	34166954	unprocessed_pseudog	2762,20	0
ENSG00000262983	ENSG00000262983	16	21194847	21205977	antisense	2681,30	0
ENSG00000257275	ENSG00000257275	14	95711747	95757656	antisense	2037,80	0
ENSG00000231332	OOEP-AS1	6	73369704	73387717	antisense	1764,50	0
ENSG00000213113	TUBB8P8	3	198119721	198120778	unprocessed_pseudog	1720,90	0
ENSG00000228650	ENSG00000228650	5	56770799	56772303	lincRNA	1633,70	0
ENSG00000280639	ENSG00000280639	15	70570958	70586606	lincRNA	1617,15	0
ENSG00000258977	LINC01467	14	81605347	81623061	lincRNA	1487,40	0
ENSG00000256039	ENSG00000256039	12	10553363	10558049	lincRNA	1377,73	0
ENSG00000248571	ENSG00000248571	4	152666368	152670107	antisense	1176,70	0
ENSG00000184608	FAM167A-AS1	8	11368402	11438658	processed_transcript	1017,09	0
ENSG00000229414	KCNQ1-AS1	11	2840135	2861568	antisense	982,73	0
ENSG00000251218	ENSG00000251218	8	134849935	134881899	lincRNA	910,40	0
ENSG00000218357	ENSG00000218357	22	47461299	47487111	lincRNA	897,70	0
ENSG00000277738	ENSG00000277738	12	91634887	91635644	lincRNA	881,80	0
ENSG00000228561	ENSG00000228561	3	177683627	177691250	lincRNA	865,60	0
ENSG00000234711	TUBB8P11	1	873292	874349	unprocessed_pseudog	796,75	0
ENSG00000228775	WEE2-AS1	7	141704338	141738346	antisense	720,47	0
ENSG00000261713	SSTR5-AS1	16	1064093	1078731	processed_transcript	698,60	0
ENSG00000262921	ENSG00000262921	17	4565752	4571760	lincRNA	669,00	0
ENSG00000237250	ENSG00000237250	1	237862175	237928321	antisense	666,00	0
ENSG00000260734	ENSG00000260734	16	71430262	71432282	transcribed_unproces	627,20	0
ENSG00000250544	ENSG00000250544	5	86746818	86749772	lincRNA	619,18	0
ENSG00000215237	ENSG00000215237	9	14993312	15019729	unprocessed_pseudog	611,86	0
ENSG00000254951	ENSG00000254951	11	7754393	7905955	sense_overlapping	547,30	0
ENSG00000213005	PTTG3P	8	66767400	66768005	processed_pseudoger	519,78	0,7582127
ENSG00000218561	ENSG00000218561	6	86729708	86730108	processed_pseudoger	518,80	0
ENSG00000254993	TRIM77BP	11	49117019	49124570	unprocessed_pseudog	500,60	0
ENSG00000247134	ENSG00000247134	8	32996178	33044855	lincRNA	485,08	0
ENSG00000246228	CASC8	8	127289817	127482139	antisense	479,70	0
ENSG00000246214	ENSG00000246214	5	16615926	16629969	antisense	444,06	0
ENSG00000237469	TUBB8P10	1	227493029	227495142	unprocessed_pseudog	442,40	0
ENSG00000258754	LINC01579	15	93718542	94070820	lincRNA	441,20	0
ENSG00000255393	ENSG00000255393	11	60024908	60031077	lincRNA	428,77	0
ENSG00000214433	GOLGA2P8	15	90291962	90295683	transcribed_unproces	426,75	6,23E-03
ENSG00000225660	ENSG00000225660	X	146955075	146955209	processed_pseudoger	416,40	0
ENSG00000263146	ENSG00000263146	18	78976555	78979074	lincRNA	392,30	0
ENSG00000278060	ENSG00000278060	2	72932974	72934355	antisense	384,46	0
ENSG00000229664	ENSG00000229664	10	6025978	6036427	antisense	384,40	0
ENSG00000228708	ENSG00000228708	21	18477358	18486599	sense_overlapping	379,20	0
ENSG00000227681	ENSG00000227681	6	147660703	147953937	lincRNA	364,10	0

ENSG00000224356	ENSG00000224356	13	100535741	100587146	sense_intronic	359,30	2,95E-02
ENSG00000233878	ENSG00000233878	7	156437789	156445588	lincRNA	338,14	0
ENSG00000248202	ENSG00000248202	5	97881420	97922695	lincRNA	336,00	0
ENSG00000247213	LINC01498	12	108434130	108492585	antisense	331,85	0
ENSG00000254266	PKIA-AS1	8	78426103	78558503	lincRNA	327,20	0
ENSG00000261458	ENSG00000261458	16	74842587	74843883	unprocessed_pseudog	308,60	0
ENSG00000254919	ENSG00000254919	11	35915051	35918739	antisense	307,10	0
ENSG00000198491	ENSG00000198491	3	187197090	187207629	antisense	297,70	6,23E-03
ENSG00000226397	C12orf77	12	24993424	24997519	lincRNA	293,80	0
ENSG00000266489	ENSG00000266489	18	24516883	24518006	lincRNA	292,50	0
ENSG00000233508	ENSG00000233508	20	43190101	43202599	lincRNA	287,39	0
ENSG00000234354	RPS26P47	13	100539901	100540248	processed_pseudogen	286,35	0
ENSG00000268942	CKS1BP3	5	62512246	62512482	processed_pseudogen	282,25	0
ENSG00000189238	LINC00943	12	126726270	126746252	lincRNA	278,60	0
ENSG00000226792	LINC00371	13	51082119	51172388	lincRNA	278,27	0
ENSG00000257859	CASC18	12	105704203	105744062	lincRNA	278,10	0
ENSG00000230356	NCAPD2P1	7	34750253	34752053	processed_pseudogen	276,99	0
ENSG00000263041	ENSG00000263041	10	117825903	117830518	lincRNA	273,00	0
ENSG00000279924	ENSG00000279924	13	18174010	18178465	unprocessed_pseudog	270,55	4,86E-02
ENSG00000259156	CHEK2P2	15	20282744	20291586	transcribed_unproces	266,60	0
ENSG00000227902	ENSG00000227902	2	146879909	146898218	lincRNA	259,30	0
ENSG00000227835	CARM1P1	9	2907073	3053408	transcribed_unproces	249,40	0
ENSG00000227517	LINC01483	17	69577251	69903000	lincRNA	236,27	0
ENSG00000227042	ENSG00000227042	X	10242339	10365323	lincRNA	230,10	0
ENSG00000246316	ENSG00000246316	5	114488997	114668410	antisense	224,47	0
ENSG00000254968	ENSG00000254968	11	112393118	112621729	lincRNA	222,40	0
ENSG00000226191	CLK3P2	1	247936061	247937513	processed_pseudogen	218,71	6,23E-03
ENSG00000251391	ENSG00000251391	5	66298468	66322944	lincRNA	218,70	6,23E-03
ENSG00000250400	LINC00977	8	129216467	129241250	lincRNA	216,40	0
ENSG00000250366	TUNAR	14	95876392	95925571	lincRNA	214,70	0
ENSG00000223611	SUPT20HL2	X	24310907	24313315	processed_pseudogen	202,10	6,23E-03
ENSG00000248118	LINC01019	5	3417152	3536094	lincRNA	187,00	0
ENSG00000255039	ENSG00000255039	11	97222644	97259987	lincRNA	184,30	0
ENSG00000253576	ENSG00000253576	8	91975908	91977418	antisense	181,90	0,1217903
ENSG00000224625	TUBB8P6	1	242057085	242060242	unprocessed_pseudog	181,70	0
ENSG00000167355	ENSG00000167355	11	5304976	5505652	processed_transcript	177,50	0
ENSG00000237838	ENSG00000237838	3	24687919	25174305	lincRNA	175,80	0
ENSG00000250584	LINC01511	5	1363582	1380067	lincRNA	170,70	0
ENSG00000253877	LINC01608	8	110937690	111027433	lincRNA	166,20	0
ENSG00000253642	ENSG00000253642	8	33604856	34039008	lincRNA	160,30	0
ENSG00000236345	ENSG00000236345	6	63806836	63822642	antisense	159,93	0
ENSG00000228791	THRB-AS1	3	24494087	24681711	processed_transcript	159,50	0
ENSG00000243144	ENSG00000243144	7	91311368	91515409	lincRNA	158,40	0
ENSG00000254369	HOXA-AS3	7	27129977	27155928	antisense	156,32	0
ENSG00000237534	LINC00383	13	69222346	69292154	lincRNA	151,80	0
ENSG00000276012	ENSG00000276012	13	101717564	101723106	antisense	150,60	0
ENSG00000236377	ENSG00000236377	17	32905662	32906584	lincRNA	150,53	0
ENSG00000266181	EIF4A3P1	18	24522273	24522919	processed_pseudogen	149,90	6,23E-03
ENSG00000258879	ENSG00000258879	12	25011415	25020893	lincRNA	149,40	2,95E-02
ENSG00000259343	TMC3-AS1	15	81324377	81362037	antisense	145,56	0
ENSG00000232524	ENSG00000232524	7	123456629	123459856	antisense	145,50	0
ENSG00000228358	ENSG00000228358	4	117360625	117372852	lincRNA	140,70	0
ENSG00000235827	TUBB8P9	1	227506182	227508292	unprocessed_pseudog	137,70	0
ENSG00000214009	PCNAP3	X	46058751	46059528	processed_pseudogen	136,75	1,6174211
ENSG00000235008	ENSG00000235008	6	166701094	166702839	processed_pseudogen	136,40	6,23E-03
ENSG00000223742	ENSG00000223742	X	28571532	28586395	lincRNA	135,10	0
ENSG00000218052	ADAMTS7P4	15	85255369	85330334	transcribed_unproces	133,41	0
ENSG00000261722	ENSG00000261722	16	79212711	79229453	lincRNA	133,38	0
ENSG00000249365	ENSG00000249365	5	125108204	125149929	lincRNA	132,70	0

ENSG00000223838	ENSG00000223838	7	19353534	19578606	lincRNA	131,00	0
ENSG00000272163	ENSG00000272163	8	24912165	24914717	antisense	130,77	0,4332376
ENSG00000226484	ENSG00000226484	X	36365626	36440292	antisense	129,20	0
ENSG00000267291	ENSG00000267291	19	38596346	38601694	antisense	127,21	6,23E-03
ENSG00000273920	ENSG00000273920	15	81335577	81336119	antisense	124,80	0
ENSG00000234715	ENSG00000234715	7	103445207	103514007	antisense	124,20	0
ENSG00000267429	ENSG00000267429	19	56347081	56348183	sense_intronic	122,69	0
ENSG00000236453	ENSG00000236453	7	94022833	94066661	lincRNA	122,00	0,1217903
ENSG00000214237	ENSG00000214237	3	150871045	150891060	transcribed_unproces	121,90	6,23E-03
ENSG00000261780	ENSG00000261780	18	73324941	73349878	processed_transcript	120,90	0
ENSG00000243797	ENSG00000243797	7	106372251	106770207	sense_overlapping	120,40	0
ENSG00000280169	ENSG00000280169	13	100537365	100539567	antisense	118,67	0,3565078
ENSG00000261206	LINC00561	13	75932395	75935132	lincRNA	117,90	6,23E-03
ENSG00000255311	ENSG00000255311	11	83643602	83725390	antisense	117,83	0
ENSG00000233491	ENSG00000233491	7	81576386	81626216	lincRNA	117,33	0
ENSG00000257474	ENSG00000257474	12	79540203	79550535	lincRNA	116,60	0
ENSG00000253100	ENSG00000253100	8	25685798	25687524	lincRNA	116,40	6,23E-03
ENSG00000267109	ENSG00000267109	17	70312831	70368916	lincRNA	114,70	0
ENSG00000255457	ENSG00000255457	8	13695574	13705506	processed_pseudoger	114,69	0
ENSG00000275612	ENSG00000275612	11	7698951	7699393	sense_intronic	114,40	0
ENSG00000237571	ENSG00000237571	2	215274992	215277947	lincRNA	113,70	0
ENSG00000253288	ENSG00000253288	8	137809444	138083570	lincRNA	112,20	0
ENSG00000224746	ENSG00000224746	7	135926455	135932881	antisense	110,50	0
ENSG00000254811	ENSG00000254811	11	106250019	106264905	transcribed_unproces	110,30	0
ENSG00000245719	ENSG00000245719	15	67834310	67838879	lincRNA	109,54	0
ENSG00000268696	ZNF723P	19	22832201	22859422	unprocessed_pseudog	109,20	0
ENSG00000259293	ENSG00000259293	15	58434890	58498735	antisense	108,00	0
ENSG00000255099	ENSG00000255099	8	109777912	109782288	processed_pseudoger	105,60	0
ENSG00000267134	ENSG00000267134	18	64104091	64423601	lincRNA	104,90	0
ENSG00000256124	LINC01152	17	72030291	72041297	lincRNA	104,09	0
ENSG00000217455	ENSG00000217455	7	3140234	3174654	lincRNA	103,82	0
ENSG00000250874	ENSG00000250874	5	85663232	85664684	lincRNA	103,50	0
ENSG00000259392	ENSG00000259392	15	32836983	32837326	transcribed_processe	102,70	6,23E-03
ENSG00000232642	ENSG00000232642	2	24165884	24175005	antisense	100,57	2,21E-02
ENSG00000226320	ENSG00000226320	3	34159334	34562877	lincRNA	99,30	0
ENSG00000261720	ENSG00000261720	16	1065240	1066502	lincRNA	99,10	2,95E-02
ENSG00000256717	ENSG00000256717	11	115659658	115843146	lincRNA	98,80	0
ENSG00000231873	ENSG00000231873	3	40766207	40862626	lincRNA	98,30	0
ENSG00000230448	LINC00276	2	13710531	14400963	lincRNA	96,10	0
ENSG00000238009	ENSG00000238009	1	89295	133723	lincRNA	95,91	6,23E-03
ENSG00000254031	ENSG00000254031	8	71155457	71204223	antisense	95,80	0
ENSG00000197984	OR51A8P	11	4832133	4833072	unprocessed_pseudog	95,10	0
ENSG00000278130	ENSG00000278130	9	138177426	138179774	unprocessed_pseudog	93,50	0
ENSG00000232461	ENSG00000232461	3	176643941	176867838	lincRNA	92,80	0
ENSG00000255027	ENSG00000255027	11	125903247	125938916	antisense	92,00	0,0358681
ENSG00000226674	TEX41	2	144667967	145262988	lincRNA	91,95	0
ENSG00000223715	LINC01208	3	176604143	176635532	lincRNA	90,08	0
ENSG00000223930	ENSG00000223930	3	178202050	178385417	lincRNA	89,90	0
ENSG00000267337	LINC01478	18	44323436	44531697	lincRNA	89,50	0
ENSG00000234787	LINC00458	13	54115783	54132866	lincRNA	89,00	6,23E-03
ENSG00000241668	RPL19P11	5	31053565	31054153	processed_pseudoger	88,18	0
ENSG00000228711	ENSG00000228711	7	82657035	82657411	processed_pseudoger	87,70	2,95E-02
ENSG00000260896	ENSG00000260896	16	80828735	80892595	lincRNA	87,37	0
ENSG00000224643	ENSG00000224643	2	183083405	183108519	antisense	87,09	0
ENSG00000231421	ENSG00000231421	17	30573471	30577000	processed_transcript	86,85	6,23E-03
ENSG00000243273	ENSG00000243273	3	150890636	151038818	antisense	85,60	0
ENSG00000255425	OR8G3P	11	124214767	124215687	unprocessed_pseudog	85,00	0
ENSG00000250891	ENSG00000250891	5	118468156	118562117	lincRNA	84,77	0
ENSG00000275078	ENSG00000275078	8	139508701	139508971	unprocessed_pseudog	84,30	0,1217903

ENSG00000245750	DRAIC	15	69463026	69571440	lincRNA	83,58	4,86E-02
ENSG00000220575	HTR5A-AS1	7	155067067	155071557	antisense	83,00	0,1217903
ENSG00000236452	ENSG00000236452	3	34203244	34268811	lincRNA	82,50	0,1217903
ENSG00000242850	RPL23AP68	12	87169985	87170429	processed_pseudoger	79,60	0
ENSG00000175841	FAM172BP	3	101521891	101522979	processed_pseudoger	79,50	2,95E-02
ENSG00000237301	ENSG00000237301	1	15586136	15603626	antisense	78,41	6,23E-03
ENSG00000250411	ENSG00000250411	5	125036831	125042239	lincRNA	78,20	0
ENSG00000234921	ENSG00000234921	9	124031624	124032524	antisense	77,80	0,1217903
ENSG00000253535	ENSG00000253535	8	24295814	24912073	antisense	77,00	6,23E-03
ENSG00000253972	ENSG00000253972	8	119215111	119246848	lincRNA	77,00	0
ENSG00000267555	ENSG00000267555	19	32831295	32833168	antisense	76,91	0
ENSG00000273628	ENSG00000273628	13	24933006	24936796	unprocessed_pseudog	75,27	0
ENSG00000185847	LINC01405	12	110936585	110958208	lincRNA	75,20	0
ENSG00000238150	ENSG00000238150	19	53775599	53775882	processed_pseudoger	74,96	0
ENSG00000254480	ENSG00000254480	11	4187140	4202655	lincRNA	74,50	0
ENSG00000244018	RPL35P6	8	18774547	18774918	processed_pseudoger	73,96	0
ENSG00000253799	LINC01030	8	90592804	90606065	lincRNA	73,40	6,23E-03
ENSG00000251259	ENSG00000251259	4	105137280	105140619	lincRNA	72,65	3,27E-03
ENSG00000267193	ENSG00000267193	18	45669367	45747215	antisense	72,50	0
ENSG00000237605	ENSG00000237605	1	206634649	206635622	antisense	72,18	6,23E-03
ENSG00000248311	ENSG00000248311	5	4775476	4805769	lincRNA	72,10	0
ENSG00000261555	ENSG00000261555	16	17825252	17826906	lincRNA	71,10	0
ENSG00000261848	ENSG00000261848	17	3134969	3177031	lincRNA	70,90	6,23E-03
ENSG00000248486	ENSG00000248486	5	15192139	15266541	lincRNA	70,80	0
ENSG00000231459	LINC00032	9	27245684	27282793	lincRNA	70,54	4,86E-02
ENSG00000256879	ENSG00000256879	12	20361732	20370262	antisense	70,45	0
ENSG00000229259	LRRC37A12P	1	32423214	32426789	unprocessed_pseudog	69,60	2,95E-02
ENSG00000272027	ENSG00000272027	2	34692290	34703606	lincRNA	69,20	0
ENSG00000254180	ENSG00000254180	8	90534611	90620070	lincRNA	68,64	6,23E-03
ENSG00000244053	RPL13AP2	14	47200575	47201104	processed_pseudoger	68,19	0
ENSG00000235974	VN2R19P	19	58012589	58025926	unitary_pseudogene	67,85	0,4850164
ENSG00000249396	ENSG00000249396	5	10493527	10502728	lincRNA	67,50	6,23E-03
ENSG00000267568	ENSG00000267568	17	76950317	76969156	processed_transcript	67,27	0
ENSG00000203585	ENSG00000203585	12	67519829	67567126	lincRNA	66,17	0,2746192
ENSG00000243499	RPS6P21	12	131667492	131668246	processed_pseudoger	66,10	0
ENSG00000253619	ENSG00000253619	8	120913065	121119754	lincRNA	65,36	0
ENSG00000177112	MRVI1-AS1	11	10541272	10599932	antisense	64,88	0
ENSG00000250420	AACSP1	5	178764861	178818435	transcribed_unproces	64,00	0
ENSG00000254235	ENSG00000254235	8	9249417	9413714	lincRNA	63,50	0,1217903
ENSG00000253539	ENSG00000253539	8	99796615	99799187	antisense	63,40	0
ENSG00000230131	ENSG00000230131	10	123356450	123517708	lincRNA	62,10	0
ENSG00000255046	ENSG00000255046	8	11797928	11802568	lincRNA	61,05	0,6309753
ENSG00000272525	ENSG00000272525	5	73497550	73498293	lincRNA	60,34	1,40E-02
ENSG00000251138	ENSG00000251138	12	74133166	74402535	lincRNA	60,30	0
ENSG00000239219	ENSG00000239219	3	169939353	169966734	antisense	60,27	0,0358681
ENSG00000237813	ENSG00000237813	7	116238260	116499465	antisense	60,10	6,23E-03
ENSG00000260586	ENSG00000260586	15	71342324	71348471	sense_intronic	59,30	6,23E-03
ENSG00000258700	LINC00871	14	45940943	46501823	lincRNA	59,20	0
ENSG00000228624	ENSG00000228624	6	113969701	114471705	antisense	58,38	0
ENSG00000257364	VENTXP3	12	74292324	74293096	processed_pseudoger	58,30	0
ENSG00000253591	ENSG00000253591	5	170639158	170681437	antisense	58,10	0
ENSG00000258803	ENSG00000258803	14	56514331	56551309	lincRNA	58,06	0
ENSG00000258637	ENSG00000258637	14	79661666	79791263	antisense	57,60	6,23E-03
ENSG00000230552	ENSG00000230552	2	176724268	176819310	lincRNA	57,50	0
ENSG00000227579	ENSG00000227579	1	177393287	177597709	lincRNA	57,30	0
ENSG00000243069	ARHGEF26-AS1	3	154024401	154121332	processed_transcript	56,50	0
ENSG00000258220	ENSG00000258220	12	78326680	78359746	lincRNA	56,00	0
ENSG00000271989	ENSG00000271989	1	10429881	10430677	antisense	55,17	0,0358681
ENSG00000224517	HTR2A-AS1	13	46852143	46856299	antisense	55,00	0

ENSG00000249484	LINC01470	5	152618965	153223543	lincRNA		54,90	0
ENSG00000249464	LINC01091	4	123650267	123930406	lincRNA		54,54	0
ENSG00000258454	ENSG00000258454	14	76235817	76263474	sense_overlapping		54,41	1,40E-02
ENSG00000256995	ENSG00000256995	12	22699859	23174125	lincRNA		54,20	0
ENSG00000257986	ENSG00000257986	14	25835858	26143305	lincRNA		53,00	6,23E-03
ENSG00000228221	LINC00578	3	177441921	177752305	lincRNA		53,00	0
ENSG00000237647	ERICH1-AS1	8	737651	1137777	antisense		52,50	0
ENSG00000237433	RPSAP11	3	32190747	32191627	processed_pseudogen		51,80	2,95E-02
ENSG00000254194	ENSG00000254194	8	33973701	34009595	lincRNA		51,50	0
ENSG00000276668	ENSG00000276668	11	7699562	7699988	sense_intronic		51,30	0
ENSG00000261584	ENSG00000261584	6	26686241	26687964	lincRNA		51,30	0
ENSG00000232046	ENSG00000232046	2	66574030	66730157	lincRNA		50,90	0
ENSG00000269009	SLC6A21P	19	49255472	49256102	processed_pseudogen		50,30	2,95E-02
ENSG00000250682	LINC00491	5	102609156	102671559	lincRNA		50,30	6,23E-03
ENSG00000227948	ENSG00000227948	7	111411319	111411883	processed_pseudogen		50,00	0
ENSG00000232599	ENSG00000232599	X	125203805	125204338	processed_pseudogen		49,92	0,3565078
ENSG00000249388	ENSG00000249388	12	54082118	54102693	lincRNA		49,90	0
ENSG00000231826	ENSG00000231826	2	43027853	43039547	lincRNA		49,80	6,23E-03
ENSG00000257817	ENSG00000257817	12	114713811	114767957	lincRNA		49,75	0
ENSG00000228824	MIR4500HG	13	87427214	87671259	lincRNA		49,60	0
ENSG00000258725	PRC1-AS1	15	90966345	90988624	antisense		49,51	0
ENSG00000254560	BBOX1-AS1	11	27047186	27220113	antisense		49,10	2,95E-02
ENSG00000224506	ENSG00000224506	6	125674353	125720218	lincRNA		48,64	0
ENSG00000233338	TLR8-AS1	X	12902817	12908333	antisense		48,60	6,23E-03
ENSG00000267761	ENSG00000267761	18	47285725	47594550	lincRNA		48,30	0
ENSG00000253438	PCAT1	8	126847055	127021014	lincRNA		48,20	0
ENSG00000224932	ENSG00000224932	4	117314597	117360639	lincRNA		48,20	0
ENSG00000257636	ENSG00000257636	14	30437992	30573629	antisense		47,80	2,95E-02
ENSG00000232692	ENSG00000232692	21	26378552	26471698	antisense		47,76	1,40E-02
ENSG00000262898	ENSG00000262898	17	83144131	83177607	lincRNA		47,73	0,0358681
ENSG00000232310	ENSG00000232310	6	134525314	134659836	lincRNA		47,26	6,23E-03
ENSG00000226083	SLC39A12-AS1	10	18001786	18010562	antisense		47,20	6,23E-03
ENSG00000256663	ENSG00000256663	12	20551590	20553012	processed_pseudogen		47,12	0,8926569
ENSG00000261405	ENSG00000261405	16	33562649	33570935	transcribed_unproces		47,10	2,95E-02
ENSG00000277851	ENSG00000277851	12	92247756	92363832	lincRNA		47,10	0
ENSG00000250685	ENSG00000250685	16	84192558	84197053	antisense		47,00	4,86E-02
ENSG00000257219	ENSG00000257219	12	76259839	76305131	lincRNA		47,00	0
ENSG00000166408	OR5P1P	11	7772890	7773814	unprocessed_pseudog		46,80	0
ENSG00000234509	ENSG00000234509	21	31653593	31659500	lincRNA		46,60	9,18E-02
ENSG00000250237	ENSG00000250237	5	69038518	69043821	lincRNA		46,59	7,06E-02
ENSG00000197085	NPSR1-AS1	7	34346512	34871582	processed_transcript		46,50	0
ENSG00000233996	ENSG00000233996	2	189486480	189487992	processed_pseudogen		45,38	6,23E-03
ENSG00000248588	ENSG00000248588	5	92826255	92844004	lincRNA		45,30	6,23E-03
ENSG00000258066	ENSG00000258066	12	77775783	77783576	lincRNA		45,20	0
ENSG00000277215	SPANXA2-OT1	X	141502849	141649927	lincRNA		45,19	4,86E-02
ENSG00000232537	ENSG00000232537	1	209147267	209155101	lincRNA		44,90	0
ENSG00000251577	ENSG00000251577	4	103548745	103624534	antisense		44,10	6,23E-03
ENSG00000248491	ENSG00000248491	4	127096891	127470569	lincRNA		44,08	0
ENSG00000276842	ENSG00000276842	12	25103124	25103869	antisense		44,06	0
ENSG00000231107	LINC01508	9	90300902	90433505	lincRNA		44,00	6,23E-03
ENSG00000230489	VAV3-AS1	1	107964443	107994607	antisense		43,20	2,95E-02
ENSG00000254695	ENSG00000254695	11	15643885	15705368	lincRNA		42,90	0
ENSG00000249041	ENSG00000249041	4	154754756	154781873	antisense		42,60	0,1217903
ENSG00000267287	ENSG00000267287	18	79576460	79589010	processed_transcript		42,58	0,512233
ENSG00000248834	MARK2P5	5	15384220	15384853	processed_pseudogen		42,50	2,95E-02
ENSG00000280206	ENSG00000280206	16	15701237	15702118	lincRNA		42,44	0
ENSG00000224367	OACYLP	18	58996734	59069338	unitary_pseudogene		42,20	0
ENSG00000263655	ENSG00000263655	18	73914405	74034161	lincRNA		42,20	6,23E-03
ENSG00000231557	ENSG00000231557	2	198882573	199071791	lincRNA		42,00	0

ENSG00000235111	ENSG00000235111	22	49612657	49615716	sense_intronic	41,70	6,23E-03
ENSG00000251165	F11-AS1	4	186286094	186500997	antisense	41,70	6,23E-03
ENSG00000266984	POLR3GP2	18	48478561	48479228	processed_pseudoger	41,58	0,8926569
ENSG00000267174	ENSG00000267174	19	11300777	11324441	3prime_overlapping_	41,30	2,95E-02
ENSG00000250131	ENSG00000250131	4	177444979	177677126	antisense	40,74	3,27E-03
ENSG00000263427	ENSG00000263427	17	8056225	8057621	lincRNA	40,50	0
ENSG00000237720	ENSG00000237720	2	2834264	2838391	lincRNA	40,50	2,95E-02
ENSG00000236922	LINC01378	4	117406166	117691188	lincRNA	40,10	0,1217903
ENSG00000243988	RPS24P17	16	56906534	56906956	processed_pseudoger	40,00	1,6498059
ENSG00000274204	ENSG00000274204	13	106506046	106506713	sense_intronic	39,23	0
ENSG00000253868	FER1L6-AS2	8	124046073	124171522	antisense	39,20	0
ENSG00000249797	ENSG00000249797	5	117925008	118266035	lincRNA	39,20	0
ENSG00000267052	ENSG00000267052	7	106570947	106598908	lincRNA	38,40	0
ENSG00000268295	POLR3GP1	14	91603173	91603433	transcribed_processe	38,30	0
ENSG00000231134	TCF7L1-IT1	2	85186409	85187253	sense_intronic	37,73	6,23E-03
ENSG00000257222	ENSG00000257222	12	102063355	102074820	antisense	37,65	6,23E-03
ENSG00000186232	SSU72P3	11	4329865	4330446	processed_pseudoger	37,40	2,95E-02
ENSG00000278239	ENSG00000278239	19	41833686	41835950	lincRNA	37,30	0
ENSG00000232715	LINC01022	7	158590629	158591120	antisense	37,30	6,23E-03
ENSG00000247732	ENSG00000247732	5	7290823	7296345	unprocessed_pseudog	37,10	2,95E-02
ENSG00000248112	ENSG00000248112	5	82919376	82921119	lincRNA	36,90	0
ENSG00000261305	ENSG00000261305	7	150341771	150342607	sense_overlapping	36,40	0
ENSG00000254813	ENSG00000254813	8	12765849	12811478	lincRNA	36,20	0
ENSG00000255028	ENSG00000255028	11	109355085	109583907	antisense	36,10	2,95E-02
ENSG00000236885	ENSG00000236885	2	151117955	151186209	lincRNA	36,00	0
ENSG00000261742	LINC00922	16	65284499	65576300	lincRNA	35,73	0
ENSG00000231747	ENSG00000231747	2	112621809	112622167	processed_pseudoger	35,72	0,6526668
ENSG00000261418	ENSG00000261418	15	23430839	23435212	unprocessed_pseudog	35,50	6,23E-03
ENSG00000229336	ENSG00000229336	21	19739709	19740150	processed_pseudoger	35,36	6,23E-03
ENSG00000233735	ENSG00000233735	1	240177839	240179644	antisense	35,20	0
ENSG00000258535	ENSG00000258535	14	51765276	51825422	sense_overlapping	35,00	0
ENSG00000228463	ENSG00000228463	1	257864	297502	lincRNA	34,92	0
ENSG00000276121	ENSG00000276121	15	44778196	44778721	lincRNA	34,67	0
ENSG00000232021	LEF1-AS1	4	108167525	108256836	processed_transcript	34,52	3,27E-03
ENSG00000261600	ENSG00000261600	2	91580336	91580863	lincRNA	33,88	0
ENSG00000248019	FAM13A-AS1	4	88709789	88730103	antisense	33,76	0,8468482
ENSG00000261645	DISC1FP1	11	90251232	90915052	processed_transcript	33,75	1,40E-02
ENSG00000224099	ENSG00000224099	2	195448532	195478925	lincRNA	33,60	0,5865398
ENSG00000234511	C5orf58	5	170232447	170252575	processed_transcript	33,53	1,95E-02
ENSG00000203523	TAS2R2P	7	12491095	12492004	processed_pseudoger	33,50	2,95E-02
ENSG00000248538	ENSG00000248538	8	9189011	9202854	lincRNA	33,38	0
ENSG00000274979	ENSG00000274979	12	69326574	69331882	lincRNA	33,27	0
ENSG00000241956	ENSG00000241956	5	164296696	165171643	antisense	33,10	0
ENSG00000224184	MIR3681HG	2	11848622	12578348	lincRNA	33,08	0
ENSG00000228714	ENSG00000228714	9	115739663	115744239	lincRNA	33,00	0
ENSG00000260798	ENSG00000260798	16	68814330	68823526	antisense	32,80	0,2746192
ENSG00000254024	ENSG00000254024	8	101461177	101492499	antisense	32,80	2,95E-02
ENSG00000255583	ENSG00000255583	12	63682523	63724935	transcribed_unproces	32,70	6,23E-03
ENSG00000274719	ENSG00000274719	15	57990217	57990636	sense_intronic	32,62	0
ENSG00000258616	ENSG00000258616	14	45706250	45715952	lincRNA	32,40	2,95E-02
ENSG00000267665	ENSG00000267665	17	77526998	77536592	lincRNA	32,20	6,23E-03
ENSG00000260658	ENSG00000260658	16	63314264	63618046	lincRNA	32,10	6,23E-03
ENSG00000253407	ENSG00000253407	8	131130388	131144881	lincRNA	32,10	2,95E-02
ENSG00000225398	PGM5P4	2	113541937	113563298	unprocessed_pseudog	32,10	0
ENSG00000230002	ALMS1-IT1	2	73456764	73459484	sense_intronic	32,03	0
ENSG00000264693	ENSG00000264693	18	77622552	77624515	lincRNA	31,90	2,95E-02
ENSG00000258342	ENSG00000258342	14	35897982	36063743	sense_overlapping	31,80	6,23E-03
ENSG00000228898	MTCO1P43	2	117026537	117027024	unprocessed_pseudog	31,80	0
ENSG00000224655	ENSG00000224655	2	121790444	121796702	lincRNA	31,40	6,23E-03

ENSG00000260459	FTLP14	16	68822587	68823070	processed_pseudogen	31,20	0
ENSG00000256734	ENSG00000256734	11	18450112	18450868	processed_pseudogen	31,00	0,1217903
ENSG00000249856	ENSG00000249856	5	74917726	75023928	lincRNA	30,75	0,1187106
ENSG00000167117	LINC00483	17	50761029	50767557	processed_transcript	30,50	6,23E-03
ENSG00000237053	FUCA1P1	2	176804987	176806337	processed_pseudogen	30,50	0
ENSG00000255660	RERG-AS1	12	15151923	15155283	lincRNA	30,10	2,95E-02
ENSG00000230525	ENSG00000230525	2	66904436	66971462	lincRNA	30,10	6,23E-03
ENSG00000261298	ENSG00000261298	2	234222838	234224514	lincRNA	30,08	6,23E-03
ENSG00000237137	ENSG00000237137	9	14531916	14532042	processed_pseudogen	30,00	0
ENSG00000229618	ENSG00000229618	7	13101391	13704149	lincRNA	30,00	0
ENSG00000257826	ENSG00000257826	14	36061026	36067190	lincRNA	29,80	6,23E-03
ENSG00000254303	ENSG00000254303	8	121954640	122127184	lincRNA	29,80	2,95E-02
ENSG00000230649	ENSG00000230649	7	136025761	136084668	lincRNA	29,70	0
ENSG00000260706	ENSG00000260706	16	80065836	80167577	lincRNA	29,50	6,23E-03
ENSG00000255124	ENSG00000255124	11	125939212	125943702	lincRNA	29,40	2,95E-02
ENSG00000266844	ENSG00000266844	18	76974690	76984162	antisense	29,35	0
ENSG00000248307	LINC00616	4	138027422	138130709	lincRNA	29,30	2,95E-02
ENSG00000249988	ENSG00000249988	4	14164455	14242813	lincRNA	29,10	0
ENSG00000270816	LINC00221	14	106482439	106521073	lincRNA	29,03	6,23E-03
ENSG00000245384	ENSG00000245384	4	104490965	104697592	antisense	29,00	6,23E-03
ENSG00000205054	LINC01121	2	45164870	45323295	processed_transcript	28,91	4,86E-02
ENSG00000249618	ENSG00000249618	4	129771659	129955368	lincRNA	28,90	6,23E-03
ENSG00000260792	ENSG00000260792	14	104661120	104665558	lincRNA	28,50	0
ENSG00000254095	ENSG00000254095	8	31339197	31346479	processed_transcript	28,50	0
ENSG00000229703	ENSG00000229703	1	248692191	248699110	lincRNA	28,30	6,23E-03
ENSG00000260577	ENSG00000260577	16	68644248	68646168	antisense	28,21	0
ENSG00000227718	ENSG00000227718	2	13723048	13758152	lincRNA	28,20	2,95E-02
ENSG00000231121	ENSG00000231121	12	77324641	77572275	lincRNA	28,15	0
ENSG00000280241	ENSG00000280241	4	154142122	154298819	antisense	28,00	6,23E-03
ENSG00000206066	IGLL3P	22	25318256	25320080	unprocessed_pseudog	27,96	0
ENSG00000213876	RPL7AP64	17	7140930	7141592	processed_pseudogen	27,93	6,23E-03
ENSG00000281508	CDR1-AS	X	140783176	140784660	antisense	27,91	0,5865398
ENSG00000267746	ENSG00000267746	18	34222965	34224761	antisense	27,90	0,1217903
ENSG00000255704	ENSG00000255704	12	131596857	131597115	processed_pseudogen	27,90	6,23E-03
ENSG00000205622	ENSG00000205622	21	38863676	38956467	lincRNA	27,86	6,23E-03
ENSG00000250940	ENSG00000250940	4	4086638	4086814	processed_pseudogen	27,60	0
ENSG00000228966	HOMER2P1	14	106460092	106461055	processed_pseudogen	27,59	0,1187106
ENSG00000179136	LINC00670	17	12549782	12642854	lincRNA	27,50	0
ENSG00000231476	ENSG00000231476	7	1620654	1621405	lincRNA	27,36	0,4909563
ENSG00000248927	ENSG00000248927	5	120781218	120790778	lincRNA	27,36	0,0358681
ENSG00000236318	ENSG00000236318	7	17374867	17466664	lincRNA	27,10	6,23E-03
ENSG00000228033	ENSG00000228033	2	52722677	52910020	lincRNA	27,10	0,1217903
ENSG00000254101	ENSG00000254101	8	136530798	136984935	lincRNA	27,09	0
ENSG00000246130	ENSG00000246130	8	23068229	23083619	antisense	27,07	0
ENSG00000230234	ENSG00000230234	6	160272617	160276130	lincRNA	27,00	6,23E-03
ENSG00000247157	LINC01252	12	11548030	11590369	lincRNA	26,86	0
ENSG00000227375	DLG1-AS1	3	197298252	197303747	antisense	26,54	1,40E-02
ENSG00000237400	ENSG00000237400	7	36463023	36504513	antisense	26,50	6,23E-03
ENSG00000223586	LINC01312	6	133821147	133851800	lincRNA	26,33	0,0358681
ENSG00000259457	ENSG00000259457	15	69564724	69565790	lincRNA	26,30	2,95E-02
ENSG00000267496	FAM215A	17	43917194	43917985	lincRNA	26,20	0,1217903
ENSG00000233009	NALCN-AS1	13	100708325	101059286	antisense	26,08	0
ENSG00000234626	ENSG00000234626	22	32327171	32343105	antisense	25,70	6,23E-03
ENSG00000251418	ENSG00000251418	4	148526493	148527139	processed_pseudogen	25,62	6,23E-03
ENSG00000256288	ENSG00000256288	12	10332861	10338292	lincRNA	25,60	6,23E-03
ENSG00000231806	PCAT7	9	94555069	94568127	antisense	25,50	0,1217903
ENSG00000174977	ENSG00000174977	17	18650195	18651542	processed_pseudogen	25,45	0
ENSG00000226337	ENSG00000226337	9	68541036	68644442	lincRNA	25,30	6,23E-03
ENSG00000275773	ENSG00000275773	6	60723148	60723898	lincRNA	25,30	0

ENSG00000251027	ENSG00000251027	5	106815197	107011014	lincRNA		25,20	0
ENSG00000259039	ENSG00000259039	14	57578632	57581320	antisense		25,08	0
ENSG00000271133	ENSG00000271133	7	20328299	20331747	antisense		25,05	1,40E-02
ENSG00000233825	ENSG00000233825	10	32346499	32347179	antisense		24,86	0,6898868
ENSG00000258784	ENSG00000258784	14	55748208	55773203	lincRNA		24,70	6,23E-03
ENSG00000233577	ENSG00000233577	22	25279529	25282674	lincRNA		24,60	0
ENSG00000261175	ENSG00000261175	16	86722091	86741059	lincRNA		24,60	0,4036702
ENSG00000225542	ZNF385D-AS1	3	21542816	21579959	antisense		24,60	0
ENSG00000178556	CKS1BP6	X	30617454	30617693	processed_pseudogen		24,44	0,6526668
ENSG00000254610	ENSG00000254610	11	67934563	67955802	lincRNA		24,33	6,23E-03
ENSG00000254238	ENSG00000254238	8	75026428	75029460	antisense		24,32	3,27E-03
ENSG00000232006	ENSG00000232006	7	42954135	43113931	lincRNA		24,30	3,27E-03
ENSG00000261617	ENSG00000261617	16	9355588	9408093	lincRNA		24,24	0,1217903
ENSG00000254192	ENSG00000254192	5	168654513	168667761	antisense		24,20	4,86E-02
ENSG00000213885	RPL13AP7	21	25361821	25362431	processed_pseudogen		24,05	6,23E-03
ENSG00000234899	SOX9-AS1	17	72034107	72237203	processed_transcript		24,04	0
ENSG00000237057	ENSG00000237057	17	15806241	15817742	lincRNA		24,00	6,23E-03
ENSG00000234828	ENSG00000234828	4	149352017	149896233	lincRNA		24,00	0
ENSG00000251129	ENSG00000251129	4	31997397	32155406	lincRNA		24,00	2,95E-02
ENSG00000256306	ENSG00000256306	12	13526854	13547582	processed_transcript		23,80	6,23E-03
ENSG00000253819	LINC01151	8	122670385	122694106	lincRNA		23,70	6,23E-03
ENSG00000258842	ENSG00000258842	14	62069518	62081154	lincRNA		23,61	0,4332376
ENSG00000267586	LINC00907	18	42159283	42691422	lincRNA		23,55	0
ENSG00000259473	ENSG00000259473	15	70503907	70505928	lincRNA		23,50	0
ENSG00000264151	ENSG00000264151	18	27336379	27595164	lincRNA		23,40	6,23E-03
ENSG00000224968	ENSG00000224968	1	177351586	177366272	lincRNA		23,40	6,23E-03
ENSG00000224271	ENSG00000224271	22	47631674	47855600	lincRNA		23,20	0,1217903
ENSG00000233125	ACTBP12	1	92229018	92229435	processed_pseudogen		23,10	0,1217903
ENSG00000255042	ENSG00000255042	11	50287661	50295334	transcribed_unproces		22,97	0
ENSG00000267573	KRT8P5	18	44320800	44322257	processed_pseudogen		22,80	0,1217903
ENSG00000270540	ENSG00000270540	2	236367560	236369102	antisense		22,76	0,2746192
ENSG00000225564	LINC01492	9	103140523	103325034	lincRNA		22,70	6,23E-03
ENSG00000258245	ENSG00000258245	12	75688794	75689096	processed_pseudogen		22,68	3,27E-03
ENSG00000248428	ENSG00000248428	5	110970951	111008899	lincRNA		22,60	6,23E-03
ENSG00000229271	ENSG00000229271	3	16687986	16697479	lincRNA		22,50	6,23E-03
ENSG00000250920	ENSG00000250920	4	103550927	103559127	lincRNA		22,40	6,23E-03
ENSG00000226453	ENSG00000226453	6	81845185	81933480	lincRNA		22,26	0
ENSG00000258903	ENSG00000258903	14	62103378	62117175	lincRNA		22,16	0,1187106
ENSG00000259941	ENSG00000259941	15	56394321	56396785	lincRNA		22,14	2,95E-02
ENSG00000250921	ENSG00000250921	5	4135682	4143648	lincRNA		22,00	0,1217903
ENSG00000247400	DNAJC3-AS1	13	95648733	95676925	lincRNA		21,94	0,0358681
ENSG00000253968	ENSG00000253968	5	173414035	173451165	lincRNA		21,90	0,1217903
ENSG00000145063	ENSG00000145063	2	11105317	11132821	lincRNA		21,90	2,95E-02
ENSG00000234235	BOK-AS1	2	241544403	241558977	antisense		21,84	0
ENSG00000244342	LINC00698	3	62950430	63125062	lincRNA		21,82	0
ENSG00000232271	ENSG00000232271	20	7069614	7146656	lincRNA		21,70	6,23E-03
ENSG00000258231	ENSG00000258231	12	58544124	58813060	lincRNA		21,70	0
ENSG00000230013	ENSG00000230013	9	104990796	104991781	lincRNA		21,70	6,23E-03
ENSG00000227087	RBMX2P5	5	80331573	80332541	processed_pseudogen		21,62	3,27E-03
ENSG00000246225	ENSG00000246225	11	22829380	22945393	antisense		21,61	2,95E-02
ENSG00000225243	ENSG00000225243	1	171199244	171227788	antisense		21,50	0,4036702
ENSG00000230227	SIAH1P1	X	35626142	35627064	processed_pseudogen		21,50	0,5865398
ENSG00000250786	SNHG18	5	9546200	9550609	lincRNA		21,49	0
ENSG00000236714	ENSG00000236714	5	142745600	142760998	lincRNA		21,20	2,95E-02
ENSG00000273341	ENSG00000273341	7	77416673	77425443	lincRNA		21,15	6,23E-03
ENSG00000267251	ENSG00000267251	18	80183680	80202992	antisense		21,12	0
ENSG00000230368	FAM41C	1	868071	876903	lincRNA		21,10	0
ENSG00000260923	ENSG00000260923	16	90185997	90222678	lincRNA		21,03	0
ENSG00000271109	ENSG00000271109	19	34849278	34860576	lincRNA		21,00	6,23E-03

ENSG00000254902	ANO1-AS1	11	70187788	70188509	antisense	21,00	0
ENSG00000224475	ENSG00000224475	7	97200708	97201483	processed_pseudoger	21,00	2,95E-02
ENSG00000237031	ENSG00000237031	2	80572681	80618777	antisense	20,84	3,27E-03
ENSG00000225532	ENSG00000225532	6	170736173	170737777	lincRNA	20,70	6,23E-03
ENSG00000255308	ENSG00000255308	11	19196775	19281426	antisense	20,69	0
ENSG00000272595	OR10AH1P	7	5117169	5118087	unprocessed_pseudog	20,60	0,1217903
ENSG00000265489	ENSG00000265489	17	12760140	12790242	antisense	20,56	4,86E-02
ENSG00000258770	ENSG00000258770	14	87634379	87655294	lincRNA	20,52	0
ENSG00000248801	C8orf34-AS1	8	68303468	68331491	antisense	20,50	0,5865398
ENSG00000214998	CCNB2P1	7	7909300	7910449	processed_pseudoger	20,46	0,6526668
ENSG00000258979	ENSG00000258979	14	96741191	96790613	lincRNA	20,40	0
ENSG00000260661	ENSG00000260661	15	92148752	92331037	antisense	20,39	4,86E-02
ENSG00000120055	C10orf95	10	102449837	102451543	lincRNA	20,20	3,27E-03
ENSG00000267669	ENSG00000267669	18	59821040	59825261	transcribed_processe	20,20	6,23E-03
ENSG00000224885	TSSC1-IT1	2	3298341	3301465	sense_intronic	20,20	0
ENSG00000254331	CKS1BP7	8	80644939	80645173	processed_pseudoger	20,11	0
ENSG00000282199	ENSG00000282199	17	43914433	43923001	processed_transcript	20,10	2,95E-02
ENSG00000258631	ENSG00000258631	15	93589867	93760799	lincRNA	20,10	2,95E-02
ENSG00000232591	ENSG00000232591	10	7445558	7471942	lincRNA	20,10	2,95E-02
ENSG00000248901	ENSG00000248901	5	98086260	98161193	lincRNA	20,10	0
ENSG00000225356	ENSG00000225356	4	181522666	181523001	processed_pseudoger	20,10	0,8926569
ENSG00000250102	ENSG00000250102	4	131380013	131541397	lincRNA	20,10	6,23E-03
ENSG00000248150	ENSG00000248150	5	16373361	16440081	lincRNA	20,09	4,86E-02
ENSG00000276166	ENSG00000276166	16	57759358	57760024	antisense	19,99	0,3565078
ENSG00000260205	ENSG00000260205	10	22218074	22221168	lincRNA	19,80	0,1217903
ENSG00000258791	LINC00520	14	55781135	55796688	lincRNA	19,75	1,8851183
ENSG00000159712	ANKRD18CP	9	97156570	97221235	unprocessed_pseudog	19,75	0,2746192
ENSG00000246662	LINC00535	8	93213302	93700433	antisense	19,75	9,18E-02
ENSG00000223813	ENSG00000223813	7	29514225	29563670	antisense	19,75	0
ENSG00000254367	ENSG00000254367	8	8723693	8782479	lincRNA	19,64	4,86E-02
ENSG00000224652	LINC00885	3	196142636	196160890	lincRNA	19,60	0,1217903
ENSG00000261596	ENSG00000261596	16	21626742	21627569	sense_intronic	19,50	0,1217903
ENSG00000261303	ENSG00000261303	15	78660644	78735495	transcribed_unproces	19,50	2,95E-02
ENSG00000228918	LINC01344	1	182129310	182314061	lincRNA	19,50	0,8926569
ENSG00000249345	ENSG00000249345	12	126915203	127060401	lincRNA	19,41	0
ENSG00000255129	ENSG00000255129	11	113278437	113314437	antisense	19,30	6,23E-03
ENSG00000254575	ENSG00000254575	8	14161297	14165359	antisense	19,30	1,8851183
ENSG00000214295	FOXO1B	5	181099140	181100666	processed_pseudoger	19,30	2,95E-02
ENSG00000249203	ENSG00000249203	5	44495618	44510282	lincRNA	19,30	0,8926569
ENSG00000203386	LINC01317	2	33706886	34297753	lincRNA	19,30	2,95E-02
ENSG00000237251	ENSG00000237251	7	39816549	39868140	unprocessed_pseudog	19,20	6,23E-03
ENSG00000251574	ENSG00000251574	5	104383298	105392970	lincRNA	19,20	6,23E-03
ENSG00000274961	ENSG00000274961	20	59123404	59147026	lincRNA	19,10	6,23E-03
ENSG00000255740	ENSG00000255740	12	16786762	16787845	lincRNA	18,90	0
ENSG00000230836	LINC01293	2	74940258	74942670	lincRNA	18,90	2,95E-02
ENSG00000226522	ENSG00000226522	7	18892096	18899433	antisense	18,80	2,95E-02
ENSG00000270087	ENSG00000270087	10	75279726	75401246	lincRNA	18,60	0,1217903
ENSG00000223795	ENSG00000223795	9	134505472	134521442	lincRNA	18,50	1,40E-02
ENSG00000272851	ENSG00000272851	2	207753872	207754435	antisense	18,40	2,21E-02
ENSG00000231842	ENSG00000231842	6	126177193	126202239	lincRNA	18,38	0,3565078
ENSG00000267374	ENSG00000267374	18	39206924	39800318	lincRNA	18,36	0
ENSG00000224404	ENSG00000224404	22	34208141	34209262	lincRNA	18,30	2,95E-02
ENSG00000249738	ENSG00000249738	5	159310933	159451999	lincRNA	18,30	0,1217903
ENSG00000230214	FTLP18	1	36630335	36630857	processed_pseudoger	18,30	6,23E-03
ENSG00000266304	ENSG00000266304	18	70335929	70337310	lincRNA	18,20	0,4036702
ENSG00000254146	HMGB1P46	8	107173200	107173809	processed_pseudoger	18,20	2,95E-02
ENSG00000256304	CCDC150P1	12	122322122	122324336	processed_pseudoger	18,16	0,8926569
ENSG00000269303	ENSG00000269303	19	34998233	35000170	antisense	18,10	6,23E-03
ENSG00000206120	EGFEM1P	3	168249522	168830599	unitary_pseudogene	18,00	2,95E-02

ENSG00000280580	ENSG00000280580	13	68878380	68893573	lincRNA	17,90	0,1217903
ENSG00000249645	ENSG00000249645	4	23105502	23123487	lincRNA	17,90	0
ENSG00000243744	ENSG00000243744	5	13637919	13638381	processed_pseudoger	17,85	0,2746192
ENSG00000264964	ENSG00000264964	18	9315194	9334441	lincRNA	17,82	0
ENSG00000267710	ENSG00000267710	19	56272769	56310450	processed_transcript	17,78	0,1217903
ENSG00000276688	ENSG00000276688	20	5407564	5407876	lincRNA	17,70	2,95E-02
ENSG00000237268	ENSG00000237268	7	56421857	56448375	transcribed_unproces	17,64	0,1836819
ENSG00000242440	ENSG00000242440	3	148280891	148399956	lincRNA	17,60	2,95E-02
ENSG00000264775	PPIAP14	18	5002759	5003254	processed_pseudoger	17,45	0,1836819
ENSG00000233891	ENSG00000233891	2	59238714	59733396	lincRNA	17,40	0,1217903
ENSG00000205918	PDPK2P	16	2616121	2643296	transcribed_unproces	17,39	0,4036702
ENSG00000276403	ENSG00000276403	18	58476191	58477484	lincRNA	17,36	0,0358681
ENSG00000258751	ENSG00000258751	14	48396508	48491767	lincRNA	17,30	0,1217903
ENSG00000205035	ENSG00000205035	11	49558546	49810419	transcribed_unproces	17,30	0,4036702
ENSG00000216471	RPSAP43	6	114084168	114084792	processed_pseudoger	17,20	2,95E-02
ENSG00000258998	ENSG00000258998	14	44763157	44782829	lincRNA	17,10	6,23E-03
ENSG00000258445	ENSG00000258445	14	60779978	60780595	processed_pseudoger	17,05	0
ENSG00000258807	ENSG00000258807	14	87710419	87872291	lincRNA	16,98	6,23E-03
ENSG00000258969	ENSG00000258969	14	43995781	44384545	lincRNA	16,90	2,95E-02
ENSG00000250342	SNRPCP16	4	9051842	9052051	processed_pseudoger	16,88	0,5502547
ENSG00000188511	C22orf34	22	49414524	49657542	lincRNA	16,73	0
ENSG00000265992	ESRG	3	54632122	54639857	sense_intronic	16,70	0,1217903
ENSG00000238105	GOLGA2P5	12	100156357	100173659	transcribed_unproces	16,53	6,23E-03
ENSG00000250198	ENSG00000250198	5	8839732	8881525	lincRNA	16,50	0,1217903
ENSG00000232893	IQCA1-AS1	2	236391074	236392388	antisense	16,43	0,5682268
ENSG00000261127	ENSG00000261127	16	32289547	32310555	transcribed_processed	16,40	0
ENSG00000178412	ENSG00000178412	18	79638928	79679745	lincRNA	16,28	3,27E-03
ENSG00000204929	ENSG00000204929	2	65436711	66084639	lincRNA	16,27	0,6526668
ENSG00000213194	ENSG00000213194	2	157210847	157211539	processed_pseudoger	16,26	1,95E-02
ENSG00000259380	ENSG00000259380	15	38139595	38226887	lincRNA	16,10	0,5865398
ENSG00000276476	LINC00540	13	22040975	22276524	lincRNA	16,10	0,1217903
ENSG00000270405	ENSG00000270405	7	152366763	152367015	processed_pseudoger	16,05	0,1392851
ENSG00000256278	ENSG00000256278	15	84611689	84614969	antisense	15,90	0
ENSG00000227863	ENSG00000227863	7	89443974	89494262	lincRNA	15,90	0
ENSG00000241048	ENSG00000241048	3	150202174	150225190	lincRNA	15,90	0,1217903
ENSG00000215533	LINC00189	21	29193480	29288205	sense_overlapping	15,80	0
ENSG00000226401	ENSG00000226401	7	63632608	63636617	unprocessed_pseudog	15,80	0,4036702
ENSG00000237039	ENSG00000237039	2	231256097	231256306	processed_pseudoger	15,76	0,4332376
ENSG00000243836	WDR86-AS1	7	151409161	151413354	processed_transcript	15,75	6,23E-03
ENSG00000257543	ENSG00000257543	12	101408372	101409060	lincRNA	15,74	0
ENSG00000234948	LINC01524	20	52210645	52650431	lincRNA	15,70	2,95E-02
ENSG00000258449	ENSG00000258449	12	25096868	25100980	antisense	15,69	0
ENSG00000225706	PTPRD-AS1	9	8858130	8862255	lincRNA	15,63	3,27E-03
ENSG00000253622	ENSG00000253622	8	117128455	117130299	sense_intronic	15,60	0
ENSG00000228361	ENSG00000228361	6	168229732	168240715	lincRNA	15,60	0,1217903
ENSG00000249307	LINC01088	4	78971748	79308798	antisense	15,57	0,3565078
ENSG00000250313	ENSG00000250313	5	66507544	66511604	lincRNA	15,50	0,4036702
ENSG00000273891	ENSG00000273891	10	121965764	121967700	antisense	15,46	0
ENSG00000261837	ENSG00000261837	16	78534374	78535648	sense_intronic	15,40	2,95E-02
ENSG00000276742	ENSG00000276742	10	121956782	121957098	antisense	15,37	0,4036702
ENSG00000243453	COX7BP1	22	35194699	35194942	processed_pseudoger	15,30	2,95E-02
ENSG00000235531	MSC-AS1	8	71828167	72118393	antisense	15,26	2,95E-02
ENSG00000230115	TPRG1-AS2	3	189238686	189240594	antisense	15,22	0
ENSG00000281460	ENSG00000281460	20	48476999	48477553	lincRNA	15,20	6,23E-03
ENSG00000257737	ENSG00000257737	12	103654780	103657995	antisense	15,20	2,95E-02
ENSG00000281641	SAMD12-AS1	8	118621001	118858218	lincRNA	15,10	0
ENSG00000231200	ENSG00000231200	2	21933336	22531105	lincRNA	15,10	2,95E-02
ENSG00000253932	ENSG00000253932	8	13338574	13342754	antisense	15,00	2,95E-02
ENSG00000214106	PAXIP1-AS2	7	154928498	154949908	antisense	14,88	0

ENSG00000251331	ENSG00000251331	4	81602797	82044244	lincRNA	14,80	2,95E-02
ENSG00000218690	HIST1H2APS4	6	26272021	26272701	transcribed_unproces	14,75	0,2746192
ENSG00000253773	C8orf37-AS1	8	95204456	95810136	lincRNA	14,74	0
ENSG00000237919	ENSG00000237919	1	70013982	70031222	antisense	14,70	2,95E-02
ENSG00000224189	HAGLR	2	176173195	176188958	antisense	14,69	0
ENSG00000254731	ENSG00000254731	11	86703099	86714092	lincRNA	14,67	0,3565078
ENSG00000204241	ENSG00000204241	11	134032272	134046849	lincRNA	14,63	0,8926569
ENSG00000236231	ENSG00000236231	2	176524879	176531683	lincRNA	14,60	6,23E-03
ENSG00000226798	ENSG00000226798	9	80869758	80871295	lincRNA	14,50	0,4036702
ENSG00000250384	UBE2CP3	4	57072683	57073132	processed_pseudoger	14,50	0,4036702
ENSG00000224227	OR2L1P	1	247990267	247991204	unprocessed_pseudog	14,50	0,4036702
ENSG00000234523	NDUFB1P2	1	222945725	222945900	processed_pseudoger	14,45	6,23E-03
ENSG00000259664	ENSG00000259664	15	97370371	97521811	lincRNA	14,40	2,95E-02
ENSG00000255354	ENSG00000255354	8	11558466	11560020	antisense	14,38	0
ENSG00000267593	ENSG00000267593	18	58813880	58834364	lincRNA	14,30	0,4036702
ENSG00000214281	HMGN2P39	13	59010332	59010604	processed_pseudoger	14,30	2,95E-02
ENSG00000282221	ENSG00000282221	1	201399633	201401190	antisense	14,30	0,1217903
ENSG00000266357	ENSG00000266357	17	74043452	74154212	lincRNA	14,10	0,8926569
ENSG00000225249	LINC00378	13	60685199	60694528	lincRNA	14,10	2,95E-02
ENSG00000271860	ENSG00000271860	6	97283303	98399872	lincRNA	14,10	0,1217903
ENSG00000251611	ENSG00000251611	4	151407551	151408835	lincRNA	14,10	2,95E-02
ENSG00000228569	ENSG00000228569	7	156432523	156435833	lincRNA	14,08	0,2746192
ENSG00000243004	ENSG00000243004	7	19918981	20140453	sense_overlapping	14,05	6,23E-03
ENSG00000267783	ENSG00000267783	19	14119106	14119537	antisense	14,00	0,0358681
ENSG00000261801	LOXL1-AS1	15	73908071	73928248	antisense	14,00	1,40E-02
ENSG00000248457	ENSG00000248457	5	12914068	13032886	lincRNA	14,00	0,4036702
ENSG00000251003	ZFPM2-AS1	8	105780246	106060524	processed_transcript	13,85	4,86E-02
ENSG00000226608	FTLP3	20	4023917	4024444	processed_pseudoger	13,84	0
ENSG00000233052	ENSG00000233052	10	1361001	1361637	sense_intronic	13,80	6,23E-03
ENSG00000250546	ENSG00000250546	4	83796436	84293412	processed_transcript	13,80	2,95E-02
ENSG00000248459	ENSG00000248459	3	129998531	129998936	processed_pseudoger	13,80	0,5865398
ENSG00000259961	ENSG00000259961	1	13513220	13516270	antisense	13,79	0,1217903
ENSG00000231312	ENSG00000231312	2	39436637	39665343	antisense	13,76	0
ENSG00000269667	ENSG00000269667	16	85981750	85984723	lincRNA	13,70	6,23E-03
ENSG00000227463	ENSG00000227463	9	84063443	84094577	lincRNA	13,70	6,23E-03
ENSG00000226272	ARHGAP26-AS1	5	142859604	142868922	antisense	13,60	6,23E-03
ENSG00000236264	RPL26P30	11	2335132	2335776	transcribed_processe	13,57	0
ENSG00000259734	ENSG00000259734	15	86630266	86631136	antisense	13,50	0
ENSG00000254076	ENSG00000254076	8	137696724	137698467	processed_pseudoger	13,50	0,1217903
ENSG00000259929	ENSG00000259929	16	17933189	18151595	lincRNA	13,45	0
ENSG00000261276	ENSG00000261276	11	69004394	69005100	antisense	13,42	6,23E-03
ENSG00000248714	ENSG00000248714	17	49361165	49369998	lincRNA	13,36	0,2746192
ENSG00000227303	ENSG00000227303	X	143284977	143516803	antisense	13,33	0,0358681
ENSG00000250149	ENSG00000250149	4	125676718	125752793	lincRNA	13,30	6,23E-03
ENSG00000236056	GAPDHP14	21	29222321	29223257	processed_pseudoger	13,29	0,3565078
ENSG00000267098	ENSG00000267098	18	60630167	60902726	lincRNA	13,20	0,4036702
ENSG00000226709	FGF12-AS3	3	192516831	192521398	antisense	13,20	2,95E-02
ENSG00000225843	NIPA2P1	7	91320128	91321103	processed_pseudoger	13,10	2,95E-02
ENSG00000248425	ENSG00000248425	4	14390439	14393992	lincRNA	13,10	0
ENSG00000231714	ENSG00000231714	1	194350943	194352426	lincRNA	13,10	0,1217903
ENSG00000280013	ENSG00000280013	21	6008604	6008810	processed_pseudoger	13,00	6,23E-03
ENSG00000260516	ENSG00000260516	16	32356981	32363695	unprocessed_pseudog	13,00	2,95E-02
ENSG00000250654	ENSG00000250654	12	54076838	54081903	transcribed_unproces	13,00	2,21E-02
ENSG00000249951	ENSG00000249951	4	94675245	94702570	lincRNA	13,00	0,4036702
ENSG00000231758	ENSG00000231758	2	143640756	143656179	antisense	12,91	6,23E-03
ENSG00000234723	ENSG00000234723	3	22670201	22670711	unprocessed_pseudog	12,90	0,8926569
ENSG00000231007	CDC20P1	9	87011652	87013151	processed_pseudoger	12,85	0
ENSG00000269072	ENSG00000269072	19	51152923	51181966	antisense	12,82	0
ENSG00000256199	ENSG00000256199	12	64507166	64533638	lincRNA	12,80	2,95E-02

ENSG00000260183	ENSG00000260183	16	80547842	80552514	antisense	12,64	0,7582127
ENSG00000258732	ENSG00000258732	15	22278971	22282872	unprocessed_pseudog	12,63	0
ENSG00000259033	ENSG00000259033	14	70275296	70291859	lincRNA	12,60	0,1217903
ENSG00000227564	LINC00376	13	63183101	63328094	lincRNA	12,60	0,4036702
ENSG00000234763	ENSG00000234763	6	8341937	8343021	lincRNA	12,60	6,23E-03
ENSG00000266579	ENSG00000266579	6	57493855	57497691	lincRNA	12,60	0,1217903
ENSG00000248431	ENSG00000248431	4	162740668	162742048	lincRNA	12,60	0,4036702
ENSG00000235729	ENSG00000235729	X	143806391	143806733	processed_pseudoger	12,50	2,95E-02
ENSG00000249867	ENSG00000249867	11	28702615	29063821	lincRNA	12,46	2,21E-02
ENSG00000228559	ENSG00000228559	6	35544632	35545669	lincRNA	12,44	6,23E-03
ENSG00000258168	ENSG00000258168	12	70468080	70543040	antisense	12,43	0
ENSG00000257035	ENSG00000257035	12	127624153	127626267	unprocessed_pseudog	12,40	0,5865398
ENSG00000224091	ENSG00000224091	11	5205041	5207308	antisense	12,40	6,23E-03
ENSG00000264272	ENSG00000264272	17	74256896	74262020	antisense	12,32	1,40E-02
ENSG00000258343	ENSG00000258343	12	95795345	95858839	antisense	12,20	0,4036702
ENSG00000248424	OR51K1P	11	5430653	5431602	unitary_pseudogene	12,20	2,95E-02
ENSG00000229559	ENSG00000229559	6	37543553	37547280	transcribed_processe	12,20	0
ENSG00000254486	ENSG00000254486	11	12030875	12061785	lincRNA	12,14	0,2746192
ENSG00000237224	ENSG00000237224	10	129693722	129702117	antisense	12,10	2,95E-02
ENSG00000272343	ENSG00000272343	8	56222688	56223173	lincRNA	12,08	0,3565078
ENSG00000241388	HNF1A-AS1	12	120941728	120980965	processed_transcript	12,00	0,4036702
ENSG00000274797	ENSG00000274797	12	50953924	50954356	antisense	12,00	0,4036702
ENSG00000226598	ENSG00000226598	7	17463445	17558909	lincRNA	12,00	6,23E-03
ENSG00000226919	ENSG00000226919	1	240765557	240768680	antisense	11,92	0,6526668
ENSG00000248973	ENSG00000248973	5	4451930	4866221	lincRNA	11,90	0
ENSG00000250327	RPSAP70	4	186157896	186158843	processed_pseudoger	11,89	0,2746192
ENSG00000233005	ENSG00000233005	2	21221175	21970959	lincRNA	11,82	6,23E-03
ENSG00000228639	ENSG00000228639	17	72021851	72034092	lincRNA	11,80	6,23E-03
ENSG00000236389	ENSG00000236389	6	134706060	134707349	lincRNA	11,80	1,3038915
ENSG00000227165	WDR11-AS1	10	120761812	120851345	antisense	11,62	4,86E-02
ENSG00000257345	ENSG00000257345	12	92999218	93019820	lincRNA	11,60	0,5865398
ENSG00000253901	ENSG00000253901	8	91542924	91907619	lincRNA	11,60	0
ENSG00000256968	SNRPEP2	9	6748703	6748981	processed_pseudoger	11,58	0,1187106
ENSG00000232335	ENSG00000232335	1	35739389	35743576	antisense	11,56	0,5682268
ENSG00000260569	ENSG00000260569	18	74211391	74240555	lincRNA	11,50	0,4036702
ENSG00000237654	ENSG00000237654	11	131502759	131540867	antisense	11,50	0,8926569
ENSG00000197462	ENSG00000197462	7	125917871	125933832	lincRNA	11,50	2,95E-02
ENSG00000235640	ENSG00000235640	2	123695302	123697396	processed_pseudoger	11,50	0,1217903
ENSG00000261707	ENSG00000261707	16	77741468	77743000	antisense	11,43	1,95E-02
ENSG00000233860	ENSG00000233860	4	76756960	76758474	antisense	11,43	0,3318209
ENSG00000230666	CEACAM22P	19	44537058	44620821	transcribed_unproces	11,42	0,4909563
ENSG00000258038	ENSG00000258038	14	28830244	28968400	lincRNA	11,40	2,95E-02
ENSG00000274614	ENSG00000274614	13	111182111	111182692	unprocessed_pseudog	11,40	0,5865398
ENSG00000281131	SCHLAP1	2	180692104	180916939	lincRNA	11,40	0,4036702
ENSG00000258776	ENSG00000258776	14	56817570	56893710	lincRNA	11,30	0,8926569
ENSG00000236695	HNRNPA1P47	2	194187379	194188309	processed_pseudoger	11,27	0,3565078
ENSG00000259700	ENSG00000259700	15	48810701	48811909	processed_transcript	11,24	0,2746192
ENSG00000258131	ENSG00000258131	12	96985656	97185609	lincRNA	11,20	0,1217903
ENSG00000249848	ENSG00000249848	8	10336782	10337677	processed_pseudoger	11,20	2,95E-02
ENSG00000224016	ENSG00000224016	7	149891191	149909704	unprocessed_pseudog	11,20	0,1217903
ENSG00000248837	ENSG00000248837	4	22997551	23056862	lincRNA	11,20	2,95E-02
ENSG00000259450	ENSG00000259450	15	39188438	39194309	lincRNA	11,14	0,4850164
ENSG00000189212	DPY19L2P1	7	35079989	35186041	transcribed_unproces	11,13	3,27E-03
ENSG00000229017	LINC01277	6	142966421	143038077	lincRNA	11,10	0,6309753
ENSG00000226088	ENSG00000226088	1	70359562	70360437	antisense	11,10	1,5943798
ENSG00000260592	ENSG00000260592	16	19476916	19487899	antisense	11,09	0,1836819
ENSG00000230650	ENSG00000230650	2	110360608	110363737	processed_pseudoger	11,05	0,1392851
ENSG00000225976	ENSG00000225976	10	29697935	29698174	processed_pseudoger	11,00	2,95E-02
ENSG00000248400	ST13P12	5	82968888	82977153	processed_pseudoger	11,00	2,95E-02

ENSG00000240521	ENSG00000240521	3	148850933	148960112	antisense	11,00	6,23E-03
ENSG00000231040	ENSG00000231040	2	146837749	146850310	lincRNA	11,00	0,4036702
ENSG00000236240	GPC5-IT1	13	92484606	92510094	sense_intronic	10,90	0,5865398
ENSG00000258084	ENSG00000258084	12	78352519	78483019	lincRNA	10,90	6,23E-03
ENSG00000235688	ENSG00000235688	2	949627	950274	antisense	10,89	2,95E-02
ENSG00000272620	AFAP1-AS1	4	7754090	7778928	antisense	10,88	0
ENSG00000257501	ENSG00000257501	12	97713412	97761859	transcribed_processe	10,87	0,4036702
ENSG00000268686	ENSG00000268686	19	49368705	49388081	antisense	10,85	6,23E-03
ENSG00000257322	ENSG00000257322	12	93003415	93215679	antisense	10,83	0,2746192
ENSG00000251230	MIR3945HG	4	184844585	184855751	lincRNA	10,81	1,95E-02
ENSG00000267354	ENSG00000267354	18	45507202	45550183	sense_intronic	10,80	0,5865398
ENSG00000260198	ENSG00000260198	16	56300616	56302904	sense_intronic	10,80	0,4036702
ENSG00000258672	ENSG00000258672	14	99691445	99693648	lincRNA	10,80	6,23E-03
ENSG00000253400	ENSG00000253400	9	26801733	26805862	lincRNA	10,80	0,4036702
ENSG00000244998	ENSG00000244998	8	141389939	141392574	antisense	10,80	0,4036702
ENSG00000253693	ENSG00000253693	5	165349030	165778689	lincRNA	10,80	2,95E-02
ENSG00000281920	ENSG00000281920	2	65623272	65628424	lincRNA	10,80	0,5865398
ENSG00000271901	ENSG00000271901	13	34545929	34671182	lincRNA	10,70	0,1217903
ENSG00000170688	OR5E1P	11	7848700	7849626	unprocessed_pseudog	10,70	0,4036702
ENSG00000251363	ENSG00000251363	14	40954898	40975877	lincRNA	10,69	0
ENSG00000254526	ENSG00000254526	11	29159956	29266734	lincRNA	10,69	0
ENSG00000238755	ENSG00000238755	3	153384934	153980186	processed_transcript	10,67	4,86E-02
ENSG00000235269	ENSG00000235269	14	53768942	53850882	lincRNA	10,60	0,1217903
ENSG00000255532	ENSG00000255532	11	49305714	49379669	transcribed_unproces	10,60	2,95E-02
ENSG00000254339	ENSG00000254339	8	114282067	114295839	lincRNA	10,60	2,95E-02
ENSG00000233270	SNRPEP4	19	5576660	5576938	processed_pseudoger	10,58	0,3565078
ENSG00000260936	FTO-IT1	16	54039393	54040726	sense_intronic	10,52	2,95E-02
ENSG00000266988	ENSG00000266988	18	45483345	45529855	sense_intronic	10,50	2,95E-02
ENSG00000273569	ENSG00000273569	5	163855140	163856642	processed_pseudoger	10,50	0,1217903
ENSG00000227308	ENSG00000227308	2	219685381	219737937	lincRNA	10,49	1,6739638
ENSG00000275120	ENSG00000275120	15	84599434	84606463	antisense	10,47	0
ENSG00000262358	ENSG00000262358	17	3729361	3730493	antisense	10,46	6,23E-03
ENSG00000254740	ENSG00000254740	11	119372706	119381613	antisense	10,46	0,6526668
ENSG00000266368	ENSG00000266368	17	11953889	11977594	antisense	10,40	2,95E-02
ENSG00000282757	DUXB	16	75694434	75700152	unprocessed_pseudog	10,40	0,1217903
ENSG00000253397	ENSG00000253397	8	27904481	27910310	antisense	10,40	0
ENSG00000248125	ENSG00000248125	5	145429868	145451099	lincRNA	10,40	0,8926569
ENSG00000274840	ENSG00000274840	3	18013226	18041603	lincRNA	10,40	2,95E-02
ENSG00000236230	ENSG00000236230	1	222089169	222387434	lincRNA	10,40	0,1217903
ENSG00000241358	ENSG00000241358	3	146391363	146416603	transcribed_processe	10,36	4,86E-02
ENSG00000227719	ENSG00000227719	7	8114025	8116561	antisense	10,36	0
ENSG00000263711	ENSG00000263711	18	73151241	73264480	processed_transcript	10,33	0
ENSG00000228802	ENSG00000228802	2	223965701	223967706	antisense	10,33	1,95E-02
ENSG00000234008	PPP1R2P2	21	35887195	35887807	processed_pseudoger	10,30	0,4036702
ENSG00000235526	ENSG00000235526	1	110177643	110178719	antisense	10,30	0,1217903
ENSG00000250033	SLC7A11-AS1	4	138057464	138178177	processed_transcript	10,28	0,2746192
ENSG00000233067	PTCHD1-AS	X	22259797	23293146	lincRNA	10,27	0
ENSG00000223963	PRKRIRP8	1	240769420	240771534	processed_pseudoger	10,23	0,6898868
ENSG00000214043	ENSG00000214043	12	126442481	126472790	lincRNA	10,20	0,1217903
ENSG00000253627	ENSG00000253627	8	134211865	134320447	lincRNA	10,20	0,1217903
ENSG00000266466	ENSG00000266466	17	21532974	21542786	antisense	10,18	2,21E-02
ENSG00000274253	ENSG00000274253	15	22757857	22778741	sense_overlapping	10,12	0,2161218
ENSG00000256969	ENSG00000256969	12	4020977	4026658	lincRNA	10,10	2,95E-02
ENSG00000249815	ENSG00000249815	4	111826881	112072698	lincRNA	10,10	0,4036702
ENSG00000273063	ENSG00000273063	2	58241349	58241686	antisense	10,10	2,95E-02
ENSG00000241042	ENSG00000241042	1	59054397	59056049	lincRNA	10,10	0,4036702
ENSG00000273919	ENSG00000273919	13	53345211	53410880	lincRNA	10,00	0,1217903
ENSG00000253111	ENSG00000253111	8	125466939	125541373	lincRNA	10,00	0,2746192
ENSG00000229688	ISPD-AS1	7	16210488	16270604	antisense	10,00	1,95E-02

ENSG00000205791	LOH12CR2	12	12355406	12357067	lincRNA	9,97	0
ENSG00000213089	PDCL3P5	6	150827663	150828384	processed_pseudogen	9,96	9,18E-02
ENSG00000248245	ENSG00000248245	5	133160438	133224413	antisense	9,95	6,23E-03
ENSG00000170089	ENSG00000170089	5	177809407	177950732	transcribed_unproces	9,95	0
ENSG00000213735	ANAPC10P1	1	52253621	52254176	processed_pseudogen	9,94	0,4036702
ENSG00000267500	ZNF887P	19	11648364	11652877	transcribed_unproces	9,92	4,86E-02
ENSG00000215325	ASS1P10	5	145228811	145230054	processed_pseudogen	9,87	0,1392851
ENSG00000223563	ENSG00000223563	21	26889376	26939742	antisense	9,80	0,1217903
ENSG00000250105	ENSG00000250105	11	66558866	66560384	lincRNA	9,80	1,3038915
ENSG00000234248	ENSG00000234248	10	6891457	6920472	lincRNA	9,80	0,4036702
ENSG00000227482	ENSG00000227482	9	113650956	113682855	lincRNA	9,80	0,1217903
ENSG00000236719	OVAAL	1	180558976	180566518	lincRNA	9,80	0,5865398
ENSG00000237464	ENSG00000237464	20	45487613	45490248	antisense	9,70	0,4036702
ENSG00000258050	ENSG00000258050	14	55196727	55199013	lincRNA	9,70	0,5865398
ENSG00000254277	ENSG00000254277	8	71675300	71702786	lincRNA	9,70	2,95E-02
ENSG00000230404	ENSG00000230404	1	234565298	234570088	lincRNA	9,70	0,1217903
ENSG00000225918	RPL7P59	7	145039999	145040941	processed_pseudogen	9,69	0,3318209
ENSG00000223820	CFL1P1	10	87817928	87845612	transcribed_unproces	9,65	0
ENSG00000281365	ENSG00000281365	20	48471308	48472414	lincRNA	9,60	0,5865398
ENSG00000260357	DNM1P34	15	75299953	75302965	transcribed_unproces	9,60	0,1217903
ENSG00000256971	LINC00508	12	127940595	127950160	lincRNA	9,60	0
ENSG00000245832	MIR4300HG	11	81879851	82718082	lincRNA	9,60	0,1217903
ENSG00000230033	ENSG00000230033	7	156944721	156945645	lincRNA	9,60	0,4036702
ENSG00000231519	ENSG00000231519	7	30069658	30075115	antisense	9,58	0,8926569
ENSG00000260372	AQP4-AS1	18	26655742	27190698	antisense	9,56	3,27E-03
ENSG00000244502	HDAC11-AS1	3	13476982	13480053	antisense	9,55	0,2161218
ENSG00000248356	ENSG00000248356	4	145642336	145650624	antisense	9,51	0
ENSG00000228235	ENSG00000228235	21	46052596	46053105	lincRNA	9,50	0,4036702
ENSG00000259995	ENSG00000259995	16	76736342	76928169	lincRNA	9,50	0,4036702
ENSG00000261540	ENSG00000261540	16	78014683	78059454	antisense	9,50	0,1217903
ENSG00000256923	ENSG00000256923	12	22742582	22743091	lincRNA	9,50	2,95E-02
ENSG00000234710	ENSG00000234710	7	9737322	9769513	lincRNA	9,50	2,95E-02
ENSG00000224478	ENSG00000224478	6	159107791	159116053	lincRNA	9,50	0,4036702
ENSG00000241767	LINC01324	3	164714095	164831480	lincRNA	9,50	0,4036702
ENSG00000249599	BMPR1B-AS1	4	94743800	94757533	lincRNA	9,49	1,1047191
ENSG00000233838	DPH3P1	20	62845664	62845912	processed_pseudogen	9,48	0,512233
ENSG00000225077	LINC00337	1	6236240	6239444	lincRNA	9,47	0,5682268
ENSG00000259259	NPM1P42	15	72899399	72900260	processed_pseudogen	9,44	0,1217903
ENSG00000251031	ENSG00000251031	5	145222610	145228982	lincRNA	9,44	0,1187106
ENSG00000278737	ENSG00000278737	15	65083042	65083663	lincRNA	9,36	0,1217903
ENSG00000279072	ENSG00000279072	7	56809214	56848800	lincRNA	9,33	1,4448456
ENSG00000255178	ENSG00000255178	11	80751200	80762826	lincRNA	9,30	6,23E-03
ENSG00000261030	ENSG00000261030	X	13093660	13094573	lincRNA	9,30	0,1217903
ENSG00000232564	ENSG00000232564	22	40521800	40526707	antisense	9,23	0
ENSG00000232518	ENSG00000232518	2	38668202	38671421	antisense	9,20	2,95E-02
ENSG00000176659	C20orf197	20	60055925	60072953	lincRNA	9,18	0,0358681
ENSG00000227192	ENSG00000227192	6	143039425	143042324	lincRNA	9,18	0,2161218
ENSG00000251209	LINC00923	15	97572185	97874550	lincRNA	9,10	0,4036702
ENSG00000261402	ENSG00000261402	9	19926094	19929937	lincRNA	9,08	0,8108966
ENSG00000258733	ENSG00000258733	14	85934710	86129778	lincRNA	9,04	0,1217903
ENSG00000227920	ENSG00000227920	6	37545145	37550860	lincRNA	9,00	2,95E-02
ENSG00000251303	CAB39P1	5	60630514	60630973	processed_pseudogen	9,00	0,1217903
ENSG00000237013	ENSG00000237013	2	67796054	67825562	lincRNA	9,00	2,95E-02
ENSG00000271787	ENSG00000271787	2	10054421	10054866	antisense	8,96	3,27E-03
ENSG00000259712	ENSG00000259712	15	52056675	52100523	antisense	8,93	0,6309753
ENSG00000214803	ENSG00000214803	8	124192671	124247398	lincRNA	8,92	0,5682268
ENSG00000258871	ENSG00000258871	14	72552580	72595125	lincRNA	8,91	6,23E-03
ENSG00000259203	ENSG00000259203	15	52801614	52804942	lincRNA	8,90	0,1217903
ENSG00000254381	TUBB8P5	12	38201566	38203792	unprocessed_pseudog	8,90	2,95E-02

ENSG00000248131	LINC01194	5	12574857	12804363	lincRNA	8,90	0,1217903
ENSG00000253357	ENSG00000253357	5	167721363	167729124	sense_intronic	8,90	2,95E-02
ENSG00000261863	ENSG00000261863	17	4610449	4617650	lincRNA	8,80	0,1217903
ENSG00000152931	PART1	5	60487713	60547657	lincRNA	8,80	6,23E-03
ENSG00000232000	CLCN3P1	9	14987302	15146401	transcribed_unproces	8,78	0,1217903
ENSG00000258077	ENSG00000258077	12	75563202	75984015	lincRNA	8,77	1,95E-02
ENSG00000255239	TREHP1	11	118688039	118690600	unprocessed_pseudog	8,73	0,0358681
ENSG00000241556	ENSG00000241556	12	95467397	95467861	processed_pseudoger	8,71	2,007219
ENSG00000257400	ENSG00000257400	12	94491546	94496442	lincRNA	8,70	1,3038915
ENSG00000225548	ENSG00000225548	3	27802762	27891301	lincRNA	8,70	1,3038915
ENSG00000261758	ENSG00000261758	3	136752630	136755780	antisense	8,70	0,4036702
ENSG00000274849	ENSG00000274849	18	36189824	36190272	sense_intronic	8,68	2,95E-02
ENSG00000257060	ENSG00000257060	15	93312557	93569483	lincRNA	8,64	0,1836819
ENSG00000276399	ENSG00000276399	17	22406019	22413744	lincRNA	8,60	0,1217903
ENSG00000254319	ENSG00000254319	8	2666079	2728451	lincRNA	8,60	0,1217903
ENSG00000273272	ENSG00000273272	22	50542650	50543011	lincRNA	8,55	0,512233
ENSG00000254018	ENSG00000254018	8	121884724	121904273	lincRNA	8,55	0,0358681
ENSG00000272666	ENSG00000272666	22	50542305	50542906	lincRNA	8,54	0,2746192
ENSG00000228923	ENSG00000228923	22	24516508	24518386	antisense	8,50	0,4036702
ENSG00000230867	ENSG00000230867	9	32727069	32727522	processed_pseudoger	8,50	0,4036702
ENSG00000234040	ENSG00000234040	12	68626870	68627187	processed_pseudoger	8,43	1,6174211
ENSG00000229151	ENSG00000229151	X	51356944	51396462	antisense	8,42	0,8926569
ENSG00000261274	TP53TG3GP	16	35207903	35208236	processed_pseudoger	8,40	1,3038915
ENSG00000254587	ENSG00000254587	11	96590317	96713822	lincRNA	8,40	2,95E-02
ENSG00000243969	ENSG00000243969	3	154827016	154861017	lincRNA	8,40	0,5865398
ENSG00000224195	ENSG00000224195	10	73813518	73814737	antisense	8,39	0,5865398
ENSG00000269813	ENSG00000269813	19	7959123	7960012	antisense	8,34	3,27E-03
ENSG00000261239	ANKRD26P1	16	46469341	46569097	transcribed_unproces	8,33	0,5502547
ENSG00000231092	ENSG00000231092	2	216259595	216266130	antisense	8,31	0,1836819
ENSG00000258566	ENSG00000258566	14	76690139	76690742	processed_pseudoger	8,30	0,4036702
ENSG00000258626	COX7A2P1	14	67652300	67652614	processed_pseudoger	8,29	1,40E-02
ENSG00000258789	ENSG00000258789	14	88551597	88552493	antisense	8,29	0,8468482
ENSG00000265750	ENSG00000265750	18	24159509	24162211	lincRNA	8,29	0
ENSG00000267139	ENSG00000267139	19	3118665	3119304	antisense	8,28	0,8926569
ENSG00000259240	ENSG00000259240	15	51037488	51293912	lincRNA	8,25	6,23E-03
ENSG00000230863	ENSG00000230863	1	75641346	75642087	processed_pseudoger	8,21	1,6174211
ENSG00000259670	ENSG00000259670	15	48783190	48784121	antisense	8,20	0,8926569
ENSG00000226476	ENSG00000226476	1	60515716	60640491	lincRNA	8,18	1,1964007
ENSG00000280739	EIF1B-AS1	3	40173145	40309698	processed_transcript	8,12	0
ENSG00000257624	ENSG00000257624	12	112000739	112000985	processed_pseudoger	8,12	1,95E-02
ENSG00000249816	LINC00964	8	124848737	124954328	lincRNA	8,12	0,5502547
ENSG00000255388	ENSG00000255388	11	41518895	41714448	lincRNA	8,10	0,5865398
ENSG00000230196	DDX43P3	7	81610884	81611326	processed_pseudoger	8,10	0,4036702
ENSG00000233085	ENSG00000233085	6	169286534	169289216	lincRNA	8,10	1,3038915
ENSG00000261786	ENSG00000261786	3	44117299	44122365	lincRNA	8,10	0,1217903
ENSG00000233620	ENSG00000233620	1	216072465	216086917	antisense	8,10	0,5865398
ENSG00000279463	ENSG00000279463	13	73844683	73845130	sense_intronic	8,08	0,512233
ENSG00000259098	ENSG00000259098	15	22258138	22258848	processed_pseudoger	8,07	1,5943798
ENSG00000250027	ENSG00000250027	4	163108785	163119965	antisense	8,06	0,1187106
ENSG00000249846	ENSG00000249846	3	130112550	130120579	lincRNA	8,03	0,1187106
ENSG00000213609	ENSG00000213609	10	63902451	63903245	processed_pseudoger	8,00	0
ENSG00000273797	ENSG00000273797	14	69617122	69617648	sense_intronic	8,00	0,8926569
ENSG00000254757	ENSG00000254757	11	3469319	3531328	lincRNA	8,00	1,40E-02
ENSG00000235335	ENSG00000235335	2	167814774	167941144	antisense	8,00	1,1217155
ENSG00000235215	ENSG00000235215	1	59055999	59078116	lincRNA	8,00	0,5865398
ENSG00000258940	ENSG00000258940	14	39265703	39267061	antisense	7,98	3,27E-03
ENSG00000230562	FAM133DP	2	157379724	157380414	processed_pseudoger	7,97	1,40E-02
ENSG00000228317	ENSG00000228317	9	105554035	105558029	antisense	7,92	1,5215308
ENSG00000258558	ENSG00000258558	14	30734926	30822702	antisense	7,91	1,1217155

ENSG00000251436	NUP58P1	4	125681177	125683668	processed_pseudogen	7,90	0,4036702
ENSG00000228262	LINC01320	2	34677555	34738231	lincRNA	7,90	0,1217903
ENSG00000237667	LINC01115	2	779840	868426	lincRNA	7,82	0,2746192
ENSG00000250290	NCAPGP1	8	120635558	120638204	processed_pseudogen	7,81	1,2188867
ENSG00000254233	ENSG00000254233	4	184584093	184625030	lincRNA	7,81	0,5682268
ENSG00000255353	ENSG00000255353	11	107176286	107177530	processed_pseudogen	7,81	0,1187106
ENSG00000233080	LINC01399	22	35119824	35231056	lincRNA	7,80	0,1217903
ENSG00000260123	ENSG00000260123	15	89041223	89082819	lincRNA	7,70	0,5865398
ENSG00000255194	SSU72P2	11	4242062	4242641	processed_pseudogen	7,70	0,4036702
ENSG00000233752	ENSG00000233752	2	36299388	36299830	processed_pseudogen	7,70	0,8926569
ENSG00000237843	ENSG00000237843	2	204475356	204507672	lincRNA	7,70	0,4036702
ENSG00000237742	ENSG00000237742	6	125578558	125749190	antisense	7,69	3,27E-03
ENSG00000250619	ENSG00000250619	5	9001774	9045940	antisense	7,63	6,23E-03
ENSG00000231731	ENSG00000231731	2	127455394	127514623	antisense	7,61	0,8926569
ENSG00000261177	ENSG00000261177	16	86081409	86089526	lincRNA	7,60	6,23E-03
ENSG00000270038	ENSG00000270038	14	95644508	95645232	lincRNA	7,60	0,8926569
ENSG00000245857	ENSG00000245857	8	6835554	6885276	lincRNA	7,60	0,5865398
ENSG00000219653	GCNT1P4	6	63857441	63857769	processed_pseudogen	7,60	2,95E-02
ENSG00000250331	LINC01340	5	97504696	97671046	lincRNA	7,60	0,1217903
ENSG00000251412	ENSG00000251412	4	14383123	14409078	lincRNA	7,60	0
ENSG00000224288	MTCYBP11	2	143093056	143094164	processed_pseudogen	7,60	0,4036702
ENSG00000214748	ENSG00000214748	X	137324972	137325049	processed_pseudogen	7,57	0,5682268
ENSG00000232445	ENSG00000232445	7	101308346	101310985	antisense	7,55	0,4332376
ENSG00000242951	ENSG00000242951	14	75595805	75596206	processed_pseudogen	7,51	1,8851183
ENSG00000255270	NAV2-IT1	11	19380484	19385014	sense_intronic	7,50	4,86E-02
ENSG00000255314	ENSG00000255314	11	46123573	46177106	lincRNA	7,50	1,3038915
ENSG00000254302	ENSG00000254302	8	34174886	34184895	lincRNA	7,50	0,1217903
ENSG00000261568	ENSG00000261568	6	22220781	22222395	lincRNA	7,50	0,1187106
ENSG00000247345	ENSG00000247345	5	59039761	59063503	antisense	7,50	0,4036702
ENSG00000214018	RRM2P3	X	44309314	44310476	processed_pseudogen	7,49	1,95E-02
ENSG00000250030	ENSG00000250030	4	67446267	67446574	processed_pseudogen	7,47	0,4332376
ENSG00000261200	ENSG00000261200	16	33541842	33545812	lincRNA	7,46	0,512233
ENSG00000237872	POU5F1P4	1	155433178	155434262	processed_pseudogen	7,46	0,3565078
ENSG00000002079	MYH16	7	99238794	99311130	unitary_pseudogene	7,45	0,1836819
ENSG00000266826	ENSG00000266826	17	58660580	58661599	processed_pseudogen	7,45	6,23E-03
ENSG00000213777	ENSG00000213777	19	53503392	53512687	transcribed_processed	7,42	1,6498059
ENSG00000215771	LRRC37A14P	22	41189727	41190188	processed_pseudogen	7,40	0,4036702
ENSG00000224777	OR4F2P	11	86649	87586	unprocessed_pseudog	7,40	0,1217903
ENSG00000251637	ENSG00000251637	11	67886477	67906350	lincRNA	7,40	2,21E-02
ENSG00000250354	ENSG00000250354	4	148146471	148208880	antisense	7,40	6,23E-03
ENSG00000225258	ENSG00000225258	2	180571712	180692454	lincRNA	7,40	0,4036702
ENSG00000231386	ENSG00000231386	2	70810798	70811474	processed_pseudogen	7,38	1,3038915
ENSG00000231822	ENSG00000231822	2	99102018	99102752	processed_pseudogen	7,36	6,23E-03
ENSG00000235489	DBF4P1	10	64168959	64170850	processed_pseudogen	7,32	0,8926569
ENSG00000269906	ENSG00000269906	14	50662511	50663178	sense_intronic	7,31	0,3565078
ENSG00000258232	ENSG00000258232	12	49265156	49273306	antisense	7,30	6,23E-03
ENSG00000223608	DSCR4-IT1	21	38006544	38010618	sense_intronic	7,30	0,8926569
ENSG00000257119	EEF1B2P4	12	106901283	106901984	processed_pseudogen	7,30	2,95E-02
ENSG00000231812	SNRPCP9	7	97885868	97886074	processed_pseudogen	7,20	1,3038915
ENSG00000248747	ENSG00000248747	4	141240739	141278789	lincRNA	7,20	0,1217903
ENSG00000251513	ENSG00000251513	5	97089075	97437217	lincRNA	7,13	0
ENSG00000253500	ENSG00000253500	8	87540835	87755718	antisense	7,10	0,1217903
ENSG00000253880	ENSG00000253880	8	6044353	6257537	lincRNA	7,10	2,95E-02
ENSG00000250947	TRPC7-AS2	5	136303757	136316100	antisense	7,10	0,1217903
ENSG00000260518	BMS1P8	16	33687025	33698504	transcribed_unproces	7,07	0,3318209
ENSG00000250910	ENSG00000250910	4	155173716	155381694	antisense	7,07	0,512233
ENSG00000251639	ENSG00000251639	4	1100016	1101558	processed_pseudogen	7,05	0,2746192
ENSG00000231907	GAPDHP37	22	40673484	40674451	processed_pseudogen	7,00	0,5865398
ENSG00000269930	ENSG00000269930	15	30616958	30617749	lincRNA	7,00	6,23E-03

ENSG00000218261	ENSG00000218261	6	53378503	53378861	processed_pseudoger	7,00	1,6739638
ENSG00000225744	ENSG00000225744	2	111607841	111612518	lincRNA	7,00	0,1217903
ENSG00000233538	ENSG00000233538	2	231452195	231453153	lincRNA	7,00	4,86E-02
ENSG00000253204	ENSG00000253204	8	30921060	30921335	processed_pseudoger	6,92	0,1187106
ENSG00000244119	PDCL3P4	3	101712472	101713191	processed_pseudoger	6,91	0,1217903
ENSG00000266977	ENSG00000266977	19	27849730	27889222	lincRNA	6,90	0,5865398
ENSG00000228933	ENSG00000228933	X	27174920	27398997	lincRNA	6,90	0,8926569
ENSG00000269416	LINC01224	19	23399233	23416075	lincRNA	6,86	0,5502547
ENSG00000272817	ENSG00000272817	10	91908131	91909348	antisense	6,85	0,2746192
ENSG00000273816	ENSG00000273816	17	9244666	9244897	lincRNA	6,83	9,18E-02
ENSG00000258933	ENSG00000258933	14	94960277	94962976	lincRNA	6,82	0,512233
ENSG00000266885	ENSG00000266885	17	22435390	22439033	transcribed_processe	6,80	0,1217903
ENSG00000250974	ENSG00000250974	5	6933670	7190812	lincRNA	6,80	2,95E-02
ENSG00000275945	EIF3FP1	21	10330732	10331537	processed_pseudoger	6,75	1,2188867
ENSG00000272205	ENSG00000272205	1	167219831	167220512	lincRNA	6,75	0,5502547
ENSG00000259673	IQCH-AS1	15	67403619	67521844	lincRNA	6,71	6,23E-03
ENSG00000239426	OR8F1P	11	124207183	124208112	unprocessed_pseudog	6,70	0,8926569
ENSG00000253584	ENSG00000253584	5	104917492	105246364	lincRNA	6,70	0,4036702
ENSG00000250038	ENSG00000250038	4	28362279	28402864	lincRNA	6,70	0,5865398
ENSG00000237844	ENSG00000237844	2	163749573	164352223	lincRNA	6,70	0,4036702
ENSG00000231345	BEND3P1	10	50655967	50660472	transcribed_processe	6,70	4,86E-02
ENSG00000251405	ENSG00000251405	5	157362615	157460078	sense_overlapping	6,67	0,7367804
ENSG00000275874	LINC00162	21	44999208	45004727	lincRNA	6,64	0,4909563
ENSG00000265656	ENSG00000265656	18	21673628	21682561	antisense	6,64	0,1836819
ENSG00000274093	ENSG00000274093	16	69632141	69632571	sense_intronic	6,64	1,1964007
ENSG00000228888	LINC01428	20	7146467	7254202	lincRNA	6,60	0,1217903
ENSG00000235160	ENSG00000235160	15	26395072	26446774	lincRNA	6,60	0,4036702
ENSG00000235529	AGAP1-IT1	2	235505751	235507566	sense_intronic	6,60	0,1217903
ENSG00000235036	ENSG00000235036	20	10612861	10614229	processed_pseudoger	6,57	2,95E-02
ENSG00000205871	RPS3AP47	15	43115702	43116493	processed_pseudoger	6,57	1,1217155
ENSG00000245059	ENSG00000245059	16	81077319	81078861	lincRNA	6,54	0,1392851
ENSG00000259410	ENSG00000259410	15	67832725	67873866	processed_transcript	6,50	2,95E-02
ENSG00000260128	ULK4P2	15	30572738	30600647	transcribed_unproces	6,50	0,1217903
ENSG00000260172	LINC01413	15	57319138	57323908	lincRNA	6,50	1,3038915
ENSG00000228566	ENSG00000228566	10	63664664	63990568	lincRNA	6,50	0,5865398
ENSG00000248185	ENSG00000248185	5	63301523	63302056	processed_pseudoger	6,50	0,1217903
ENSG00000251687	ENSG00000251687	4	145833118	145839580	antisense	6,50	0,4036702
ENSG00000280634	THRIL	12	125025434	125027410	antisense	6,49	0
ENSG00000248810	ENSG00000248810	4	141319450	141332618	lincRNA	6,46	0,3565078
ENSG00000260790	ENSG00000260790	16	22374859	22378180	antisense	6,46	0,2746192
ENSG00000273082	ENSG00000273082	22	33922422	33922766	antisense	6,45	0,1836819
ENSG00000230058	ENSG00000230058	13	40343957	40350303	lincRNA	6,43	0,3318209
ENSG00000261462	ENSG00000261462	7	87109539	87111282	lincRNA	6,42	0,5502547
ENSG00000227011	C17orf112	17	52985520	52987652	lincRNA	6,40	0,1217903
ENSG00000233928	ENSG00000233928	X	33726508	33942280	lincRNA	6,40	2,95E-02
ENSG00000273796	ENSG00000273796	21	45403809	45404369	lincRNA	6,39	0,1187106
ENSG00000260922	ENSG00000260922	16	77234877	77290934	antisense	6,38	3,27E-03
ENSG00000265334	ENSG00000265334	17	30834325	30863028	antisense	6,33	1,1047191
ENSG00000203799	CCDC162P	6	109285485	109355063	unitary_pseudogene	6,32	1,40E-02
ENSG00000149656	LINC00266-1	20	64290385	64313132	lincRNA	6,30	1,8851183
ENSG00000255133	ENSG00000255133	11	33814877	33822095	lincRNA	6,30	0,1217903
ENSG00000213067	ENSG00000213067	7	56597379	56598342	processed_pseudoger	6,30	1,3038915
ENSG00000273011	ENSG00000273011	7	149890739	149891416	lincRNA	6,30	0,1217903
ENSG00000250328	ENSG00000250328	5	122436497	122479087	antisense	6,30	2,95E-02
ENSG00000240992	RPS23P4	4	145269810	145270558	processed_pseudoger	6,30	0,8926569
ENSG00000183444	OR7E38P	7	97966090	97967074	unprocessed_pseudog	6,28	6,23E-03
ENSG00000267688	ENSG00000267688	19	3121116	3122128	antisense	6,20	1,2188867
ENSG00000261776	ENSG00000261776	16	78055824	78056927	processed_pseudoger	6,20	0,1217903
ENSG00000258742	ENSG00000258742	14	92886352	92893506	lincRNA	6,20	2,95E-02

ENSG00000235497	ENSG00000235497	2	23375229	23381299	lincRNA	6,20	2,95E-02
ENSG00000271955	ENSG00000271955	2	59218680	60100200	lincRNA	6,20	0,5865398
ENSG00000173954	SNURFL	X	139362040	139362382	processed_pseudoger	6,20	0,1217903
ENSG00000254042	ENSG00000254042	5	168706567	168720884	antisense	6,19	0,6526668
ENSG00000236484	RRM2P2	1	161378707	161379358	processed_pseudoger	6,19	2,0633527
ENSG00000261898	ENSG00000261898	17	4731756	4732371	antisense	6,17	6,23E-03
ENSG00000232027	ENSG00000232027	1	51372270	51373224	processed_pseudoger	6,15	0,2161218
ENSG00000266258	ENSG00000266258	18	70335439	70352459	lincRNA	6,14	1,3038915
ENSG00000278981	ENSG00000278981	4	154235980	154237598	antisense	6,13	0,512233
ENSG00000280356	ENSG00000280356	22	34641179	34651862	lincRNA	6,10	0,4036702
ENSG00000260364	ENSG00000260364	16	65233056	65432820	lincRNA	6,10	0,1217903
ENSG00000239227	ENSG00000239227	3	166861944	166862110	processed_pseudoger	6,10	0,1217903
ENSG00000233540	DNM3-IT1	1	171864187	171864687	sense_intronic	6,10	1,8851183
ENSG00000255655	ENSG00000255655	12	109445410	109447497	antisense	6,10	0,1217903
ENSG00000279159	ENSG00000279159	22	29978950	30028236	antisense	6,07	0
ENSG00000231652	ENSG00000231652	6	73693903	73696131	antisense	6,06	0,4036702
ENSG00000258561	ENSG00000258561	14	66212810	66509394	lincRNA	6,06	0,4850164
ENSG00000274993	ENSG00000274993	7	100963828	100968124	antisense	6,04	6,23E-03
ENSG00000233766	ENSG00000233766	2	191846539	192044525	antisense	6,02	0,1836819
ENSG00000261517	LINC00558	13	53815419	53876119	lincRNA	6,00	2,95E-02
ENSG00000238001	SNRPCP1	9	32676063	32676520	processed_pseudoger	6,00	0,1217903
ENSG00000181211	HECW1-IT1	7	43117896	43163187	sense_intronic	6,00	2,95E-02
ENSG00000232627	ENSG00000232627	7	23365618	23365976	processed_pseudoger	6,00	0,8926569
ENSG00000219404	ENSG00000219404	6	22213306	22214237	processed_pseudoger	6,00	0,512233
ENSG00000231690	LINC00574	6	169790321	169802873	lincRNA	6,00	0,7367804
ENSG00000241369	LINC01192	3	163127923	163361563	lincRNA	6,00	0,4036702
ENSG00000234754	ENSG00000234754	1	221330080	221336296	lincRNA	6,00	0,5865398
ENSG00000237845	ENSG00000237845	1	235942553	235943805	lincRNA	6,00	0,1836819
ENSG00000238205	MPC1L	X	40623566	40623860	processed_pseudoger	6,00	0,5865398
ENSG00000260107	ENSG00000260107	16	1997654	1998374	lincRNA	5,98	4,86E-02
ENSG00000238081	ENSG00000238081	1	89289676	89290337	processed_pseudoger	5,97	1,6174211
ENSG00000267992	ENSG00000267992	19	39030436	39031323	antisense	5,97	2,0846889
ENSG00000226432	ENSG00000226432	5	24170370	24171246	processed_pseudoger	5,96	3,27E-03
ENSG00000260808	ENSG00000260808	11	68612899	68616711	lincRNA	5,94	3,27E-03
ENSG00000251364	ENSG00000251364	11	7427266	7512515	antisense	5,93	1,5943798
ENSG00000259180	ENSG00000259180	15	55680385	55681463	antisense	5,92	0,6526668
ENSG00000186369	LINC00643	14	62114353	62130962	lincRNA	5,92	0,8926569
ENSG00000253945	ENSG00000253945	8	95403802	95403894	processed_pseudoger	5,91	0,2746192
ENSG00000225431	ENSG00000225431	21	42599280	42615058	lincRNA	5,90	0,5865398
ENSG00000257587	ENSG00000257587	12	73906940	73919337	lincRNA	5,90	0,8926569
ENSG00000248701	ENSG00000248701	5	86797685	86800280	lincRNA	5,90	1,3038915
ENSG00000234125	EEF1GP8	4	129903010	129904163	processed_pseudoger	5,90	0,4036702
ENSG00000249098	ENSG00000249098	3	130918226	130919120	processed_pseudoger	5,87	0,2746192
ENSG00000225606	ENSG00000225606	7	12570125	12571392	antisense	5,87	0,4036702
ENSG00000255970	ENSG00000255970	12	67709047	67729475	lincRNA	5,86	0,512233
ENSG00000230711	CTAGE13P	6	168286905	168289350	processed_pseudoger	5,86	3,27E-03
ENSG00000275450	ENSG00000275450	9	41276280	41282846	lincRNA	5,83	1,40E-02
ENSG00000249532	ENSG00000249532	4	112646476	112650051	antisense	5,82	0,3565078
ENSG00000255836	ENSG00000255836	12	7438780	7439990	processed_pseudoger	5,82	1,0789438
ENSG00000233922	ENSG00000233922	21	45593654	45603056	lincRNA	5,81	7,06E-02
ENSG00000242445	RPL7AP11	3	121494110	121494908	processed_pseudoger	5,80	1,40E-02
ENSG00000234479	AP1B1P1	22	32121977	32133469	transcribed_unproces	5,80	0,1217903
ENSG00000259076	ENSG00000259076	14	64552694	64596536	antisense	5,80	1,8851183
ENSG00000234551	LINC01309	13	103425200	103427685	lincRNA	5,80	0,4036702
ENSG00000253887	ENSG00000253887	8	9255349	9260295	lincRNA	5,80	0,5865398
ENSG00000226965	ENSG00000226965	7	110432239	110534754	lincRNA	5,80	0,1217903
ENSG00000237870	ENSG00000237870	7	116275606	116286734	processed_transcript	5,80	0,4036702
ENSG00000232234	ENSG00000232234	6	8329655	8342455	lincRNA	5,80	2,95E-02
ENSG00000249128	ENSG00000249128	5	118596188	118628092	lincRNA	5,80	1,3038915

ENSG00000234371	RPSAP31	3	183884924	183888449	transcribed_processe	5,80	6,23E-03
ENSG00000232688	ENSG00000232688	2	66921510	66922457	lincRNA	5,80	0,4036702
ENSG00000271550	BNIP3P11	7	64678954	64687393	processed_pseudoger	5,78	0,7367804
ENSG00000223694	ADH5P3	1	240170155	240171291	processed_pseudoger	5,75	0,512233
ENSG00000232677	LINC00665	19	36313067	36331718	lincRNA	5,74	0
ENSG00000249849	ENSG00000249849	5	177682294	177713969	antisense	5,73	0,3565078
ENSG00000248544	ENSG00000248544	5	157375741	157384950	antisense	5,72	0,5682268
ENSG00000264672	SEPT4-AS1	17	58519837	58556977	antisense	5,71	0,7367804
ENSG00000223646	ENSG00000223646	7	112622378	112708080	lincRNA	5,70	0,5865398
ENSG00000280776	LINC01202	5	161910252	162001196	lincRNA	5,70	0,4036702
ENSG00000231532	LINC01249	2	4628222	4656215	lincRNA	5,70	0,8926569
ENSG00000244310	ENSG00000244310	2	9115197	9116884	lincRNA	5,70	0,8926569
ENSG00000232514	ENSG00000232514	1	48497263	48497736	processed_pseudoger	5,70	0,4036702
ENSG00000279414	ENSG00000279414	21	9026821	9027329	processed_pseudoger	5,66	0,2746192
ENSG00000245522	ENSG00000245522	11	9754770	9759533	lincRNA	5,63	0,6309753
ENSG00000266801	ENSG00000266801	16	68933820	68937725	sense_intronic	5,62	0,4036702
ENSG00000176054	RPL23P2	21	28997613	28998033	processed_pseudoger	5,60	1,5215308
ENSG00000258496	ENSG00000258496	14	87251103	87251679	lincRNA	5,60	0,5865398
ENSG00000250049	ENSG00000250049	5	92082597	92479426	lincRNA	5,60	0,5865398
ENSG00000251613	ENSG00000251613	5	72687112	72762727	lincRNA	5,60	0,4036702
ENSG00000248210	ENSG00000248210	4	149154295	149278123	lincRNA	5,60	1,3038915
ENSG00000243491	ENSG00000243491	2	9757496	9770341	lincRNA	5,60	0,8926569
ENSG00000236643	ENSG00000236643	9	124770123	124772927	antisense	5,58	0,2161218
ENSG00000229436	ENSG00000229436	7	80662331	80662585	processed_pseudoger	5,58	0,512233
ENSG00000229928	LINC00400	13	43158631	43159466	lincRNA	5,57	0,5865398
ENSG00000214975	PPIAP29	6	24976419	24976982	processed_pseudoger	5,56	0,7095968
ENSG00000258416	ENSG00000258416	14	79893080	79974169	lincRNA	5,55	1,6739638
ENSG00000273989	ENSG00000273989	12	28236227	28236828	sense_intronic	5,55	0,2746192
ENSG00000250551	ENSG00000250551	5	96050115	96215519	lincRNA	5,54	2,95E-02
ENSG00000249898	MCPH1-AS1	8	6618475	6708209	antisense	5,53	2,21E-02
ENSG00000273987	ENSG00000273987	12	75333798	75334486	sense_intronic	5,52	0,3318209
ENSG00000214198	TTC41P	12	103843749	103930211	unitary_pseudogene	5,52	2,95E-02
ENSG00000261327	ENSG00000261327	16	88177298	88178941	lincRNA	5,50	0,5865398
ENSG00000258927	ENSG00000258927	14	95620914	95643285	lincRNA	5,50	0,8926569
ENSG00000255523	ENSG00000255523	11	58917015	58928689	antisense	5,50	0,4036702
ENSG00000204832	ST8SIA6-AS1	10	17386936	17413503	antisense	5,50	0
ENSG00000280366	ENSG00000280366	9	79975023	79990064	lincRNA	5,50	0,1217903
ENSG00000250407	ENSG00000250407	5	146563226	146617004	antisense	5,50	0,1217903
ENSG00000251321	PCAT4	4	79827471	79877770	lincRNA	5,50	0,4036702
ENSG00000231482	ENSG00000231482	2	1572554	1580311	lincRNA	5,50	0,512233
ENSG00000236605	ENSG00000236605	2	67324627	67325304	lincRNA	5,50	0
ENSG00000253269	ENSG00000253269	5	169772966	169779365	antisense	5,45	1,6739638
ENSG00000232517	ENSG00000232517	5	54808210	54872551	unitary_pseudogene	5,43	1,0789438
ENSG00000232093	ENSG00000232093	1	155045191	155046118	antisense	5,42	0,6526668
ENSG00000259904	ACTG1P15	15	34373541	34374603	processed_pseudoger	5,40	0,512233
ENSG00000258444	ENSG00000258444	14	23415339	23415686	antisense	5,40	0,8926569
ENSG00000255079	ENSG00000255079	11	44694863	44696301	lincRNA	5,40	0,8926569
ENSG00000227531	ENSG00000227531	9	111139246	111284836	lincRNA	5,40	0,5865398
ENSG00000253567	ENSG00000253567	8	28447264	28455902	antisense	5,40	0,4036702
ENSG00000225416	ENSG00000225416	7	152602213	152602892	processed_pseudoger	5,40	1,3038915
ENSG00000248373	ENSG00000248373	4	104907357	105120000	lincRNA	5,40	0,8926569
ENSG00000232455	LARS2-AS1	3	45483974	45509545	antisense	5,40	0,1217903
ENSG00000234796	ENSG00000234796	2	910926	921124	antisense	5,40	1,3038915
ENSG00000229119	ENSG00000229119	5	166382305	166382599	processed_pseudoger	5,39	0,2746192
ENSG00000234840	LINC01239	9	22646200	22824213	lincRNA	5,33	4,86E-02
ENSG00000225405	RPS15AP17	4	62105660	62106049	processed_pseudoger	5,33	0,4909563
ENSG00000227694	RPL23AP74	17	61069871	61070341	processed_pseudoger	5,33	0,5865398
ENSG00000258628	ENSG00000258628	15	20228620	20277503	unprocessed_pseudog	5,31	0,5682268
ENSG00000229715	EEF1DP3	13	31846841	31953472	transcribed_unproces	5,31	0,9880903

ENSG00000262402	ENSG00000262402	17	2127430	2127904	processed_pseudoger		5,31	0,8468482
ENSG00000259627	ENSG00000259627	15	63070025	63071911	antisense		5,30	0,7582127
ENSG00000259447	ENSG00000259447	15	39300418	39310782	lincRNA		5,30	1,3038915
ENSG00000248478	ENSG00000248478	8	121679126	121707631	lincRNA		5,30	0,1217903
ENSG00000228400	ENSG00000228400	2	124016858	124025173	antisense		5,30	0,4036702
ENSG00000260509	ENSG00000260509	13	25300124	25301438	lincRNA		5,28	1,0510989
ENSG00000275322	ENSG00000275322	15	96342953	96345651	lincRNA		5,26	0,4909563
ENSG00000237404	ENSG00000237404	6	19689825	19753113	lincRNA		5,26	2,95E-02
ENSG00000253978	ENSG00000253978	5	168229583	168232357	antisense		5,25	0,2746192
ENSG00000274317	ENSG00000274317	13	37934565	38048169	lincRNA		5,24	0,8468482
ENSG00000263470	ENSG00000263470	17	65100812	65111058	lincRNA		5,23	9,18E-02
ENSG00000256843	ENSG00000256843	12	31748100	31748240	processed_pseudoger		5,22	0,2161218
ENSG00000260785	CASC17	17	71097775	71202177	lincRNA		5,20	0,1217903
ENSG00000259459	ENSG00000259459	15	63390136	63438320	lincRNA		5,20	0,1217903
ENSG00000237937	ENSG00000237937	11	116639422	116658252	lincRNA		5,20	0,8926569
ENSG00000251381	LINC00958	11	12961842	12989548	lincRNA		5,20	0,5865398
ENSG00000236712	ENSG00000236712	7	7293508	7293880	processed_pseudoger		5,20	0,4036702
ENSG00000272701	MESTIT1	7	130486042	130491033	antisense		5,20	0,7582127
ENSG00000254365	ENSG00000254365	5	167287320	167294273	antisense		5,20	0,4036702
ENSG00000272218	ENSG00000272218	4	186892552	186892983	lincRNA		5,20	0,4036702
ENSG00000231915	SALL4P5	3	22989823	22991582	unprocessed_pseudog		5,20	0,4036702
ENSG00000227061	ENSG00000227061	2	197569	202605	antisense		5,20	0,5865398
ENSG00000233045	ENSG00000233045	2	138278574	138279210	processed_pseudoger		5,20	4,86E-02
ENSG00000234810	ENSG00000234810	1	55581037	55868248	lincRNA		5,20	0,7367804
ENSG00000164616	FBXL21	5	135930317	135951591	transcribed_unitary_p		5,19	4,86E-02
ENSG00000264956	ENSG00000264956	17	22266425	22288133	lincRNA		5,18	1,1964007
ENSG00000226747	ENSG00000226747	2	185719874	185740479	antisense		5,18	0,2746192
ENSG00000261286	ENSG00000261286	16	84459259	84467361	antisense		5,17	2,21E-02
ENSG00000271283	ENSG00000271283	19	19699203	19699409	processed_pseudoger		5,17	0,5682268
ENSG00000231409	ENSG00000231409	15	34943080	34943394	processed_pseudoger		5,17	1,1217155
ENSG00000260093	ENSG00000260093	8	10050485	10054254	antisense		5,17	1,3038915
ENSG00000231908	IDH1-AS1	2	208255247	208256181	antisense		5,17	0,3565078
ENSG00000231638	ENSG00000231638	7	43508728	43522542	antisense		5,16	0,4909563
ENSG00000273254	ENSG00000273254	21	29024255	29024890	antisense		5,11	1,8851183
ENSG00000274605	ENSG00000274605	13	100086031	100088848	lincRNA		5,11	0,4036702
ENSG00000269043	ENSG00000269043	19	20432552	20528615	lincRNA		5,10	0,5865398
ENSG00000267579	ENSG00000267579	18	58557070	58566677	antisense		5,10	0,5865398
ENSG00000261122	ENSG00000261122	16	35743268	35756515	lincRNA		5,10	0,8926569
ENSG00000242775	ENSG00000242775	3	55360443	55361829	lincRNA		5,10	0,8926569
ENSG00000238042	ENSG00000238042	1	221880981	221978523	lincRNA		5,10	0,5865398
ENSG00000159904	ZNF890P	7	5121239	5144546	transcribed_unproces		5,09	0,512233
ENSG00000245248	USP2-AS1	11	119381778	119526664	antisense		5,09	4,86E-02
ENSG00000258153	HSPE1P4	12	101873316	101873623	processed_pseudoger		5,09	0,5682268
ENSG00000258088	ENSG00000258088	12	75694010	75698816	lincRNA		5,07	0,1217903
ENSG00000272155	ENSG00000272155	8	65714334	65714778	antisense		5,04	1,547231
ENSG00000254420	ENSG00000254420	11	78324758	78444049	antisense		5,03	3,27E-03
ENSG00000234577	SYNE1-AS1	6	152380546	152381564	antisense		5,02	1,0177863
ENSG00000260958	ENSG00000260958	16	35207937	35284146	lincRNA		5,00	0,8926569
ENSG00000259342	ENSG00000259342	15	45430652	45441808	antisense		5,00	0,4036702
ENSG00000253633	ENSG00000253633	8	102528740	102538668	lincRNA		5,00	0,4036702
ENSG00000235538	ENSG00000235538	6	163703904	163759841	lincRNA		5,00	0,1217903
ENSG00000214108	TPT1P5	5	31908361	31909186	processed_pseudoger		5,00	0,4332376
ENSG00000213519	ENSG00000213519	3	197580344	197580629	processed_pseudoger		5,00	0,3318209
ENSG00000230773	ENSG00000230773	2	47941696	48240983	lincRNA		5,00	1,6739638
ENSG00000224445	ENSG00000224445	1	99472332	99600995	lincRNA		5,00	0,8926569
ENSG00000229007	EXOSC3P1	21	32496812	32497311	processed_pseudoger		5,00	0,1217903
ENSG00000253480	ENSG00000253480	5	180085717	180087038	processed_pseudoger		4,96	9,18E-02
ENSG00000271329	ENSG00000271329	1	44187943	44189049	processed_pseudoger		4,94	1,3038915
ENSG00000269086	ENSG00000269086	19	34837889	34855304	lincRNA		4,90	0,1217903

ENSG00000260834	ENSG00000260834	16	65190973	65234914	lincRNA	4,90	0,1217903
ENSG00000257109	OR4F28P	15	101875964	101876901	unprocessed_pseudog	4,90	0,1217903
ENSG00000228115	ENSG00000228115	9	487774	495610	sense_intronic	4,90	0,8926569
ENSG00000272243	ENSG00000272243	6	74530248	74734279	lincRNA	4,90	0,5865398
ENSG00000230126	FGF12-AS2	3	192515022	192516573	antisense	4,90	0,4036702
ENSG00000263884	ENSG00000263884	18	268148	270278	lincRNA	4,86	1,0789438
ENSG00000255847	ENSG00000255847	11	73963657	73970287	antisense	4,84	0,1392851
ENSG00000278931	ENSG00000278931	21	8857260	8880976	unprocessed_pseudog	4,84	0,4036702
ENSG00000257698	ENSG00000257698	12	57931528	57936175	lincRNA	4,83	2,21E-02
ENSG00000227107	ENSG00000227107	2	237612977	237626525	lincRNA	4,83	0,8468482
ENSG00000224361	ENSG00000224361	2	23507043	23524344	antisense	4,82	0,7582127
ENSG00000229989	MIR181A1HG	1	198807493	198937429	lincRNA	4,82	0,2161218
ENSG00000253392	ENSG00000253392	19	2915146	2926807	antisense	4,81	0,1392851
ENSG00000241357	ENSG00000241357	7	100435257	100436510	antisense	4,80	3,27E-03
ENSG00000239674	ENSG00000239674	22	32141267	32143272	lincRNA	4,80	0,1217903
ENSG00000279851	ENSG00000279851	21	10122273	10129029	lincRNA	4,80	0,5865398
ENSG00000176840	MIR7-3HG	19	4769140	4772533	lincRNA	4,80	0,5865398
ENSG00000267466	ENSG00000267466	17	77563368	77568695	lincRNA	4,80	0,8926569
ENSG00000227624	SNRPEP3	16	20238972	20239247	processed_pseudoger	4,80	1,3038915
ENSG00000214344	OR4F13P	15	101842119	101850324	transcribed_unproces	4,80	0,5865398
ENSG00000241582	RPL23AP8	14	34694170	34694644	processed_pseudoger	4,80	0,4036702
ENSG00000232849	LINC00363	13	93056657	93057926	lincRNA	4,80	1,3038915
ENSG00000256209	ENSG00000256209	12	131469311	131492951	lincRNA	4,80	1,8851183
ENSG00000234506	LINC01506	9	68543541	68546589	lincRNA	4,80	0,8926569
ENSG00000269957	ENSG00000269957	9	24545952	24591993	lincRNA	4,80	0,4036702
ENSG00000229140	CCDC26	8	129351691	129680239	lincRNA	4,80	1,3038915
ENSG00000255364	ENSG00000255364	8	122414332	122428551	lincRNA	4,80	0,4036702
ENSG00000224865	ENSG00000224865	7	131897641	131948953	lincRNA	4,80	0,8926569
ENSG00000239614	HMGN1P7	3	93988634	93988946	processed_pseudoger	4,80	0,1217903
ENSG00000213981	ENSG00000213981	2	170640374	170695374	antisense	4,80	0,5865398
ENSG00000271286	ENSG00000271286	X	21939461	21940160	processed_pseudoger	4,80	0,5865398
ENSG00000213070	HMGB3P6	1	164356767	164357364	processed_pseudoger	4,78	0,7095968
ENSG00000249492	ENSG00000249492	5	43483959	43509356	processed_transcript	4,75	0,6526668
ENSG00000232682	ENSG00000232682	10	60050668	60060743	antisense	4,71	1,0789438
ENSG00000267070	ENSG00000267070	16	5098739	5142595	lincRNA	4,70	0,4036702
ENSG00000271395	ENSG00000271395	13	89551233	89552389	lincRNA	4,70	1,3038915
ENSG00000273819	ENPP7P7	11	67812557	67873367	unprocessed_pseudog	4,70	1,8851183
ENSG00000235743	ENSG00000235743	6	23337711	23346560	lincRNA	4,70	0,8926569
ENSG00000273118	ENSG00000273118	2	212581357	213021545	sense_overlapping	4,70	1,3038915
ENSG00000273800	ENSG00000273800	X	12583415	12583526	unprocessed_pseudog	4,70	2,95E-02
ENSG00000270402	ENSG00000270402	19	19708965	19710465	processed_pseudoger	4,69	1,2188867
ENSG00000278390	ENSG00000278390	13	41132939	41236686	antisense	4,68	1,2188867
ENSG00000228794	LINC01128	1	825138	859446	processed_transcript	4,66	0
ENSG00000267123	ENSG00000267123	17	78617389	78632057	lincRNA	4,64	0,7582127
ENSG00000229191	ENSG00000229191	1	201023949	201028792	antisense	4,64	1,2188867
ENSG00000279062	ENSG00000279062	21	10136419	10137004	unprocessed_pseudog	4,60	0,8926569
ENSG00000278531	ENSG00000278531	10	45187292	45198323	unprocessed_pseudog	4,60	0,4036702
ENSG00000254181	SLC25A51P3	8	80594815	80595703	processed_pseudoger	4,60	0,5865398
ENSG00000228700	ENSG00000228700	7	128433422	128433713	processed_pseudoger	4,60	1,8851183
ENSG00000232756	ENSG00000232756	7	77990384	77995171	antisense	4,60	0,4036702
ENSG00000233108	ENSG00000233108	7	7958183	7969903	antisense	4,60	0,5682268
ENSG00000248551	ENSG00000248551	4	175346358	175403110	lincRNA	4,60	2,95E-02
ENSG00000223783	ENSG00000223783	3	195836193	195860404	lincRNA	4,60	0,8926569
ENSG00000228308	LINC01209	3	176814155	176817001	lincRNA	4,60	1,3038915
ENSG00000237750	ENSG00000237750	2	162768936	162797972	antisense	4,60	0,5865398
ENSG00000259153	ENSG00000259153	14	70810205	70815403	lincRNA	4,58	2,0846889
ENSG00000229044	ENSG00000229044	1	31333067	31346799	antisense	4,58	0,8468482
ENSG00000268894	PLCE1-AS1	10	94279277	94287478	antisense	4,57	2,007219
ENSG00000254777	ENSG00000254777	8	60910053	60966557	lincRNA	4,56	0,4909563

ENSG00000196204	RNF216P1	7	4973988	5040675	transcribed_unproces	4,55	0
ENSG00000240280	TCAM1P	17	63849292	63864379	unitary_pseudogene	4,55	0,4036702
ENSG00000277301	ENSG00000277301	20	32509959	32520285	antisense	4,54	0,8108966
ENSG00000233457	ENSG00000233457	9	78150532	78151193	processed_pseudoger	4,54	1,4697798
ENSG00000178458	H3F3AP6	4	139698144	139698554	processed_pseudoger	4,53	0,3565078
ENSG00000268050	ENSG00000268050	9	129175807	129177575	antisense	4,53	0,7582127
ENSG00000235776	ENSG00000235776	22	19792294	19793094	processed_pseudoger	4,50	0
ENSG00000230352	ENSG00000230352	20	59360927	59364773	lincRNA	4,50	0,1217903
ENSG00000235621	LINC00494	20	48359950	48370629	lincRNA	4,50	0,4036702
ENSG00000260162	ENSG00000260162	16	88177289	88178646	lincRNA	4,50	1,1964007
ENSG00000259636	ENSG00000259636	15	86932880	86946331	sense_intronic	4,50	0,5865398
ENSG00000254140	ENSG00000254140	14	105601472	105605357	lincRNA	4,50	0,4036702
ENSG00000233379	ENSG00000233379	13	77939162	77944874	lincRNA	4,50	0,8926569
ENSG00000275598	ENSG00000275598	11	63469376	63470944	processed_pseudoger	4,50	0,8926569
ENSG00000228280	ENSG00000228280	10	75742740	75743755	sense_intronic	4,50	0,4036702
ENSG00000236748	ENSG00000236748	7	8938787	8939369	processed_pseudoger	4,50	0,5865398
ENSG00000250461	ENSG00000250461	5	61375112	61375855	processed_pseudoger	4,50	1,6174211
ENSG00000239265	CLRN1-AS1	3	150852484	151080726	antisense	4,50	0,7367804
ENSG00000222030	ENSG00000222030	2	59217708	59279400	lincRNA	4,50	2,95E-02
ENSG00000236720	ENSG00000236720	1	177700524	177710330	lincRNA	4,50	0,1217903
ENSG00000180385	EMC3-AS1	3	9986893	10006990	transcribed_unproces	4,49	2,21E-02
ENSG00000248636	ENSG00000248636	12	119387987	119668079	lincRNA	4,48	0,512233
ENSG00000232134	RPS15AP12	1	220143964	220144351	processed_pseudoger	4,48	0,8108966
ENSG00000223803	RPS20P14	3	186900198	186900557	processed_pseudoger	4,47	0,4332376
ENSG00000227028	SLC8A1-AS1	2	39786453	40255209	antisense	4,45	4,86E-02
ENSG00000279047	ENSG00000279047	5	141046260	141096402	antisense	4,44	0,5682268
ENSG00000206567	ENSG00000206567	3	10006418	10011209	lincRNA	4,43	3,27E-03
ENSG00000226047	ENSG00000226047	9	107117459	107121705	lincRNA	4,42	0,8468482
ENSG00000262943	ALOX12P2	17	6853861	6954107	transcribed_unproces	4,42	1,40E-02
ENSG00000251211	ENSG00000251211	5	178165702	178166562	processed_pseudoger	4,41	1,2188867
ENSG00000257883	ENSG00000257883	12	116661582	116698065	lincRNA	4,40	0,1217903
ENSG00000256195	ENSG00000256195	11	114360635	114380045	antisense	4,40	1,3038915
ENSG00000226140	ENSG00000226140	10	16278701	16295858	lincRNA	4,40	0,4036702
ENSG00000253470	ENSG00000253470	8	125749055	125750241	lincRNA	4,40	0,8926569
ENSG00000253659	ENSG00000253659	8	79297717	79314471	lincRNA	4,40	1,3038915
ENSG00000231539	ENSG00000231539	7	42829672	42844438	lincRNA	4,40	2,95E-02
ENSG00000249462	MLLT10P2	4	189973599	189973847	processed_pseudoger	4,40	0,8926569
ENSG00000228952	ENSG00000228952	3	187448845	187449450	lincRNA	4,40	0,4036702
ENSG00000227824	ENSG00000227824	2	215869923	215870518	lincRNA	4,40	0,8926569
ENSG00000269974	ENSG00000269974	15	30648797	30649529	lincRNA	4,39	1,2188867
ENSG00000261377	PDCD6IPP2	15	28789664	28859007	transcribed_unproces	4,37	0,512233
ENSG00000255450	ENSG00000255450	11	29275655	29276565	processed_pseudoger	4,36	0,7367804
ENSG00000267194	ENSG00000267194	17	69551358	69553861	lincRNA	4,35	0,2746192
ENSG00000267356	ENSG00000267356	18	14104542	14105226	antisense	4,35	0,4850164
ENSG00000226435	ANKRD18DP	3	198053522	198080720	transcribed_unproces	4,33	1,3038915
ENSG00000259083	ENSG00000259083	14	39174885	39175880	antisense	4,32	1,1217155
ENSG00000253741	ENSG00000253741	8	142702252	142726973	antisense	4,32	0,4332376
ENSG00000231295	ENSG00000231295	7	120746738	120752514	antisense	4,31	0,6898868
ENSG00000239780	RPLP0P11	18	50457887	50458795	processed_pseudoger	4,30	0,8926569
ENSG00000262670	ENSG00000262670	17	3278775	3386339	sense_overlapping	4,30	0,5865398
ENSG00000250514	ENSG00000250514	16	76634998	76658478	lincRNA	4,30	0,4036702
ENSG00000259441	MRPL15P1	15	89752296	89753493	processed_pseudoger	4,30	0,4036702
ENSG00000231829	ENSG00000231829	10	95141925	95168425	lincRNA	4,30	0,4036702
ENSG00000243531	ENSG00000243531	7	6900424	6900623	processed_pseudoger	4,30	1,3038915
ENSG00000248994	ENSG00000248994	5	1933863	1959176	lincRNA	4,30	0,4036702
ENSG00000229221	HNRNPA1P66	2	63751697	63752630	processed_pseudoger	4,30	0,1217903
ENSG00000231290	APCDD1L-AS1	20	58515379	58619888	processed_transcript	4,30	0,6526668
ENSG00000247240	UBL7-AS1	15	74461265	74481302	antisense	4,29	1,0177863
ENSG00000267226	ENSG00000267226	18	58670009	58671877	antisense	4,28	0,4909563

ENSG00000179818	PCBP1-AS1	2	69962263	70103220	processed_transcript	4,28	0
ENSG00000227359	ENSG00000227359	2	113677702	113704078	lincRNA	4,28	0,512233
ENSG00000228397	ENSG00000228397	1	22023990	22026048	lincRNA	4,27	1,1217155
ENSG00000258759	ENSG00000258759	14	67799004	67799609	processed_pseudoger	4,27	0,3565078
ENSG00000242083	RPL7AP31	4	56356135	56356932	processed_pseudoger	4,26	2,21E-02
ENSG00000225265	TAF1A-AS1	1	222589825	222593032	antisense	4,26	0,4850164
ENSG00000181800	CELF2-AS1	10	11316834	11319884	antisense	4,25	0,4036702
ENSG00000198155	ZNF876P	4	212610	255985	transcribed_unproces	4,24	0,1187106
ENSG00000243083	LINC00870	3	72151257	72174332	lincRNA	4,21	1,6498059
ENSG00000227540	ENSG00000227540	10	73252791	73254349	antisense	4,21	0,6898868
ENSG00000277112	ENSG00000277112	20	30681825	30723932	transcribed_unproces	4,20	0,4036702
ENSG00000261815	ENSG00000261815	16	49170552	49171786	lincRNA	4,20	0,1217903
ENSG00000259611	LINC01582	15	98085627	98103712	lincRNA	4,20	0,8926569
ENSG00000259675	ENSG00000259675	15	61639349	61715171	lincRNA	4,20	0,4036702
ENSG00000236339	POM121L13P	13	28778558	28778937	processed_pseudoger	4,20	0,8926569
ENSG00000254906	ENSG00000254906	11	20670425	20671297	antisense	4,20	0,4036702
ENSG00000253944	ENSG00000253944	8	17801345	17861069	antisense	4,20	0,4036702
ENSG00000254222	ENSG00000254222	8	61264624	61292039	antisense	4,20	0,5865398
ENSG00000225365	ENSG00000225365	7	158537495	158539879	antisense	4,20	0,1217903
ENSG00000196634	LUADT1	6	147158925	147180992	lincRNA	4,20	1,3038915
ENSG00000224039	ENSG00000224039	3	16692745	16694202	unprocessed_pseudog	4,20	1,3038915
ENSG00000241359	SYNPR-AS1	3	63423596	63550051	antisense	4,20	0,8926569
ENSG00000230183	CNOT6LP1	15	56005715	56007176	processed_pseudoger	4,19	0,8926569
ENSG00000254693	ENSG00000254693	11	44604508	44605337	antisense	4,19	2,0846889
ENSG00000261054	ENSG00000261054	15	99128832	99131806	antisense	4,19	0,8926569
ENSG00000267243	ENSG00000267243	19	28435388	28727777	lincRNA	4,18	0,7367804
ENSG00000267507	ENSG00000267507	19	55119250	55120383	processed_pseudoger	4,18	0,512233
ENSG00000275234	ENSG00000275234	19	6469465	6470152	antisense	4,18	0,6309753
ENSG00000231294	ENSG00000231294	2	210171518	210230383	antisense	4,18	0,512233
ENSG00000231066	NPM1P9	X	14834476	14835319	processed_pseudoger	4,18	2,0846889
ENSG00000248278	SUMO2P17	17	48874860	48908983	transcribed_processe	4,17	0,6898868
ENSG00000231851	ENSG00000231851	2	105097052	105102944	antisense	4,17	3,27E-03
ENSG00000234393	ENSG00000234393	10	113710681	113719332	antisense	4,15	0,3318209
ENSG00000230483	ENSG00000230483	11	2404515	2407908	antisense	4,13	0,512233
ENSG00000198153	ZNF849P	19	22685167	22686732	unprocessed_pseudog	4,12	0,5682268
ENSG00000204745	ENSG00000204745	2	87125390	87196647	unprocessed_pseudog	4,12	0,1187106
ENSG00000230333	ENSG00000230333	7	11180902	11520175	processed_transcript	4,11	9,18E-02
ENSG00000180042	OR1R1P	17	3385930	3386833	unprocessed_pseudog	4,10	0,1217903
ENSG00000264067	ENSG00000264067	17	10320392	10341458	antisense	4,10	0,4036702
ENSG00000261161	ENSG00000261161	16	86646301	86668923	lincRNA	4,10	0,4036702
ENSG00000231518	ENSG00000231518	9	35930012	35937151	antisense	4,10	2,95E-02
ENSG00000249734	ENSG00000249734	5	6795880	6826728	lincRNA	4,10	0,4036702
ENSG00000254239	ENSG00000254239	5	135897637	135900250	lincRNA	4,10	1,3038915
ENSG00000214210	ENSG00000214210	3	164001606	164002465	processed_pseudoger	4,10	1,3038915
ENSG00000271413	ENSG00000271413	3	176069107	176069252	processed_pseudoger	4,10	0,8926569
ENSG00000237220	ENSG00000237220	2	150566134	150568080	lincRNA	4,10	0,4036702
ENSG00000226436	ENSG00000226436	X	28659065	28659469	processed_pseudoger	4,10	2,95E-02
ENSG00000253893	FAM85B	8	8167819	8226614	antisense	4,10	0,5682268
ENSG00000254802	ENSG00000254802	8	60965802	60967775	lincRNA	4,10	0,5682268
ENSG00000248568	KRT8P48	5	146706381	146707600	processed_pseudoger	4,09	1,6498059
ENSG00000270207	ENSG00000270207	3	7606890	7607897	antisense	4,09	1,2188867
ENSG00000229291	ENSG00000229291	1	235957879	235971825	lincRNA	4,09	0,1836819
ENSG00000214432	ENSG00000214432	15	91022619	91036611	antisense	4,08	0,2746192
ENSG00000261222	ENSG00000261222	17	74599840	74607229	lincRNA	4,08	0,512233
ENSG00000270750	ENSG00000270750	4	152320544	152321044	lincRNA	4,07	1,6174211
ENSG00000271711	ENSG00000271711	3	150611262	150611877	processed_pseudoger	4,06	1,1047191
ENSG00000236438	FAM157A	3	198153287	198222513	transcribed_unproces	4,05	0,4909563
ENSG00000227751	ENSG00000227751	1	17406760	17407382	antisense	4,03	0,0358681
ENSG00000279184	ENSG00000279184	22	35160947	35215025	antisense	4,02	0,1187106

ENSG00000215086	NPM1P24	10	72917641	72918534	processed_pseudoger	4,01	0,5682268
ENSG00000204666	ENSG00000204666	19	50050589	50066793	sense_overlapping	4,00	0,8926569
ENSG00000263818	ENSG00000263818	17	39057019	39113190	transcribed_processe	4,00	1,1047191
ENSG00000258611	ENSG00000258611	15	93065586	93066606	transcribed_processe	4,00	0,4036702
ENSG00000258065	ENSG00000258065	14	28857384	28858701	processed_pseudoger	4,00	0,8926569
ENSG00000259048	ENSG00000259048	14	38034287	38194281	antisense	4,00	0,8926569
ENSG00000256172	ENSG00000256172	12	67440998	67442559	lincRNA	4,00	1,3038915
ENSG00000257835	ENSG00000257835	12	77379820	77390864	lincRNA	4,00	0,5865398
ENSG00000254792	ENSG00000254792	11	67840942	67841049	processed_pseudoger	4,00	0,1217903
ENSG00000249600	ENSG00000249600	5	121671373	121671872	processed_pseudoger	4,00	0,5865398
ENSG00000250266	LINC01612	4	170273919	170283079	lincRNA	4,00	0,1217903
ENSG00000250753	ENSG00000250753	4	49579833	49580189	processed_pseudoger	4,00	0,5682268
ENSG00000259855	ENSG00000259855	2	216995906	216996490	lincRNA	4,00	0,5865398
ENSG00000270039	ENSG00000270039	12	57803838	57804415	lincRNA	3,99	0,3565078
ENSG00000124097	HMGB1P1	20	57488392	57489027	processed_pseudoger	3,94	0,6309753
ENSG00000269332	GOLGA2P9	19	22596257	22603550	transcribed_unproces	3,93	1,0789438
ENSG00000262668	ENSG00000262668	16	3188212	3224779	antisense	3,93	1,4697798
ENSG00000250261	CCDC74BP1	22	20587496	20592218	unprocessed_pseudog	3,93	0,7095968
ENSG00000241220	ENSG00000241220	3	153357107	153374186	lincRNA	3,92	0,6526668
ENSG00000254568	ENSG00000254568	11	124883691	124887789	antisense	3,92	0,7582127
ENSG00000272746	ENSG00000272746	18	13526078	13526688	sense_intronic	3,92	1,4697798
ENSG00000258738	ENSG00000258738	14	34874343	34876459	antisense	3,91	0,8926569
ENSG00000238186	ENSG00000238186	1	40515754	40517174	antisense	3,90	0,1392851
ENSG00000267284	ENSG00000267284	18	55721063	55788761	lincRNA	3,90	0,4036702
ENSG00000255945	ENSG00000255945	12	127598168	127599715	lincRNA	3,90	1,8851183
ENSG00000257265	ENSG00000257265	12	71007773	71032083	lincRNA	3,90	1,8851183
ENSG00000274373	ENSG00000274373	12	132424504	132425208	lincRNA	3,90	1,8851183
ENSG00000248935	ENSG00000248935	5	60021249	60033020	antisense	3,90	1,8851183
ENSG00000233461	ENSG00000233461	1	231522388	231528556	antisense	3,90	0,1217903
ENSG00000242375	ENSG00000242375	9	97195351	97197687	lincRNA	3,88	0,8468482
ENSG00000233544	EIF3KP2	3	24555834	24556459	processed_pseudoger	3,86	0,1217903
ENSG00000235574	ENSG00000235574	7	27491682	27492765	processed_pseudoger	3,86	0,7095968
ENSG00000232034	ENSG00000232034	2	100822661	100847220	sense_intronic	3,84	1,6739638
ENSG00000253744	ENSG00000253744	5	66144156	66144795	antisense	3,83	0,8926569
ENSG00000251018	HMMR-AS1	5	163483065	163494058	antisense	3,82	1,547231
ENSG00000175509	ENSG00000175509	2	113667478	113668621	processed_pseudoger	3,82	1,1217155
ENSG00000227008	ENSG00000227008	X	133670971	133671386	processed_pseudoger	3,81	1,2188867
ENSG00000245768	ENSG00000245768	16	58733912	59108974	lincRNA	3,81	1,95E-02
ENSG00000240207	ENSG00000240207	3	158732263	158784070	antisense	3,81	0,6309753
ENSG00000239213	NCK1-AS1	3	136841726	136862054	antisense	3,80	1,5943798
ENSG00000225785	ENSG00000225785	20	52451464	52455619	lincRNA	3,80	0,4036702
ENSG00000255775	ENSG00000255775	12	6155035	6160719	lincRNA	3,80	0,8926569
ENSG00000258026	ENSG00000258026	12	81094375	81125845	antisense	3,80	1,3038915
ENSG00000275427	ENSG00000275427	8	1296034	1302607	lincRNA	3,80	0,8926569
ENSG00000224374	ENSG00000224374	6	135259996	135260933	antisense	3,80	0,8926569
ENSG00000250509	ENSG00000250509	5	180441559	180443238	lincRNA	3,80	0,4036702
ENSG00000248432	ENSG00000248432	4	111804418	111809694	lincRNA	3,80	0,8926569
ENSG00000249998	ENSG00000249998	4	16973275	17073903	lincRNA	3,80	0,8926569
ENSG00000271964	ENSG00000271964	3	16314439	16314987	antisense	3,80	2,0846889
ENSG00000231083	ENSG00000231083	2	8600892	8622942	lincRNA	3,80	0,8926569
ENSG00000234898	CHEK2P3	2	91957436	91963610	unprocessed_pseudog	3,80	0,5865398
ENSG00000259792	ENSG00000259792	15	77993405	77995289	antisense	3,79	9,18E-02
ENSG00000243175	RPSAP36	4	143424753	143426013	transcribed_processe	3,77	1,2188867
ENSG00000231940	RPS7P3	1	233288868	233289447	processed_pseudoger	3,77	2,0846889
ENSG00000229981	LINC01435	10	107694973	108197849	lincRNA	3,75	9,18E-02
ENSG00000233633	ENSG00000233633	2	558204	578145	lincRNA	3,75	1,6739638
ENSG00000225912	ENSG00000225912	X	92676519	92676954	processed_pseudoger	3,73	0,1187106
ENSG00000253524	ENSG00000253524	8	36004316	36095046	lincRNA	3,73	1,1964007
ENSG00000232746	ENSG00000232746	3	12877522	12885211	lincRNA	3,73	1,2188867

ENSG00000254760	ENSG00000254760	19	51414298	51414965	antisense	3,72	0,8468482
ENSG00000231650	RFESDP1	13	22850101	22850561	processed_pseudoger	3,70	1,6739638
ENSG00000231620	ENSG00000231620	21	19301613	19303739	lincRNA	3,70	0,4036702
ENSG00000214318	ATP5G1P6	18	63496989	63497400	processed_pseudoger	3,70	0,5865398
ENSG00000258474	ENSG00000258474	14	31944853	31950382	lincRNA	3,70	1,8851183
ENSG00000254399	GLYATL1P4	11	59042737	59050971	unprocessed_pseudog	3,70	1,3038915
ENSG00000254991	ENSG00000254991	11	12066929	12073014	lincRNA	3,70	0,5865398
ENSG00000271897	ENSG00000271897	6	11607552	11607981	lincRNA	3,70	0,8926569
ENSG00000281608	ENSG00000281608	5	125325350	125367734	lincRNA	3,70	1,3038915
ENSG00000248242	ENSG00000248242	4	104653874	104966793	lincRNA	3,70	0,4036702
ENSG00000249519	LINC01438	4	110794403	110797344	lincRNA	3,70	0,8926569
ENSG00000250064	ENSG00000250064	4	28435449	28600275	lincRNA	3,70	0,5865398
ENSG00000232498	ENSG00000232498	1	192517639	192567217	antisense	3,70	0,8926569
ENSG00000240785	RPL36AP21	5	18049550	18049872	processed_pseudoger	3,69	1,6739638
ENSG00000247193	ENSG00000247193	4	36244116	36274220	antisense	3,68	0,6526668
ENSG00000218890	NUFIP1P	6	66093431	66094909	processed_pseudoger	3,67	1,8851183
ENSG00000249364	ENSG00000249364	5	67379378	67805238	lincRNA	3,67	0,4850164
ENSG00000227091	ENSG00000227091	1	110166186	110172489	antisense	3,65	1,6174211
ENSG00000225764	P3H2-AS1	3	190120964	190144846	antisense	3,64	0,3318209
ENSG00000213557	ENSG00000213557	9	108040213	108040592	processed_pseudoger	3,64	1,1217155
ENSG00000233478	ENSG00000233478	1	25644544	25659111	antisense	3,64	1,1217155
ENSG00000254271	ENSG00000254271	10	60734342	60741828	lincRNA	3,63	1,6739638
ENSG00000272874	ENSG00000272874	20	267186	268857	lincRNA	3,60	0,8926569
ENSG00000269365	ENSG00000269365	18	47878295	47882444	antisense	3,60	0,1392851
ENSG00000257391	MIR3180-4	16	15154903	15157020	lincRNA	3,60	1,3038915
ENSG00000268754	ENSG00000268754	16	86225580	86275745	lincRNA	3,60	0,5865398
ENSG00000269186	LINC01082	16	86196181	86199720	lincRNA	3,60	0,5865398
ENSG00000259434	ENSG00000259434	15	37365027	37490808	lincRNA	3,60	0,5865398
ENSG00000242293	ENSG00000242293	14	69023150	69023308	processed_pseudoger	3,60	1,3038915
ENSG00000259106	ENSG00000259106	14	79611418	79633311	antisense	3,60	0,5865398
ENSG00000213049	HNRNPA1P34	12	9027532	9029089	processed_pseudoger	3,60	1,3038915
ENSG00000254334	ENSG00000254334	8	21298206	21309449	lincRNA	3,60	0,5865398
ENSG00000230372	ENSG00000230372	6	25061625	25063507	lincRNA	3,60	1,3038915
ENSG00000240241	ENSG00000240241	3	78266940	78294731	lincRNA	3,60	0,1217903
ENSG00000241168	ENSG00000241168	3	163026396	163232149	lincRNA	3,60	0,5865398
ENSG00000223530	ENSG00000223530	2	23330664	23332044	lincRNA	3,60	1,8851183
ENSG00000231918	ENSG00000231918	2	51032601	52407917	lincRNA	3,60	1,3038915
ENSG00000233934	RPL21P38	2	171587093	171587259	processed_pseudoger	3,60	0,5865398
ENSG00000226530	ENSG00000226530	X	51396511	51465661	lincRNA	3,60	0,5865398
ENSG00000282317	ENSG00000282317	1	244731024	244731586	lincRNA	3,59	0,7582127
ENSG00000227615	ENSG00000227615	11	74745716	74746114	processed_pseudoger	3,58	1,1217155
ENSG00000250751	ENSG00000250751	17	49708334	49720060	antisense	3,55	0,512233
ENSG00000260430	ENSG00000260430	16	19086856	19098110	lincRNA	3,55	0,1836819
ENSG00000235071	ENSG00000235071	X	16474342	16475340	processed_pseudoger	3,55	2,007219
ENSG00000246263	UBR5-AS1	8	102239394	102253333	antisense	3,54	1,2188867
ENSG00000253851	ENSG00000253851	8	103228425	103229314	antisense	3,52	1,4448456
ENSG00000259001	ENSG00000259001	14	20343048	20343685	antisense	3,52	0,8468482
ENSG00000225739	NPM1P18	7	36818959	36819690	processed_pseudoger	3,51	0,512233
ENSG00000229876	CASC20	20	6446723	6528459	lincRNA	3,50	0,8926569
ENSG00000277453	ENSG00000277453	19	40426115	40426702	lincRNA	3,50	1,3038915
ENSG00000263004	ENSG00000263004	17	57078298	57085024	lincRNA	3,50	0,4850164
ENSG00000259560	ENSG00000259560	15	87432058	87703852	lincRNA	3,50	0,5865398
ENSG00000259616	ENSG00000259616	15	61298791	61635449	lincRNA	3,50	0,8926569
ENSG00000226695	ANKRD20A10P	13	24750657	24752171	processed_pseudoger	3,50	0,8926569
ENSG00000255258	ENSG00000255258	11	133783671	133810376	lincRNA	3,50	0,5865398
ENSG00000260049	ENSG00000260049	6	22260424	22317798	antisense	3,50	0,5865398
ENSG00000229855	ENSG00000229855	5	121199704	121357398	lincRNA	3,50	0,1217903
ENSG00000249945	ENSG00000249945	4	175458289	175466697	lincRNA	3,50	0,8926569
ENSG00000240405	LINC01212	3	69999577	70015318	lincRNA	3,50	0,8926569

ENSG00000250433	CLSTN2-AS1	3	140505611	140508789	antisense	3,50	0,4036702
ENSG00000234932	ENSG00000234932	2	152850653	152850824	processed_pseudoger	3,50	0,8926569
ENSG00000271597	ENSG00000271597	2	69251818	69252364	processed_pseudoger	3,50	0,4036702
ENSG00000224260	ENSG00000224260	1	209528455	209567673	lincRNA	3,50	0,8926569
ENSG00000214881	TMEM14DP	10	68544489	68544833	processed_pseudoger	3,48	0,0358681
ENSG00000251056	ANKRD20A17P	4	49502145	49506022	unprocessed_pseudog	3,47	0,8468482
ENSG00000268119	ENSG00000268119	19	21444241	21463908	processed_transcript	3,47	0
ENSG00000255442	ENSG00000255442	11	50170156	50190441	unprocessed_pseudog	3,46	1,4697798
ENSG00000224965	KCNC4-AS1	1	110208834	110209987	antisense	3,46	0,6526668
ENSG00000233971	RPS20P10	2	71984182	71984434	processed_pseudoger	3,45	1,8851183
ENSG00000281501	SEPSECS-AS1	4	25160641	25201440	antisense	3,44	1,0510989
ENSG00000251533	LINC00605	14	103187221	103189028	lincRNA	3,43	0,8926569
ENSG00000231185	ENSG00000231185	5	142325293	142672001	antisense	3,43	1,95E-02
ENSG00000232998	VPS13A-AS1	9	77176756	77177994	antisense	3,42	1,0177863
ENSG00000276385	ENSG00000276385	20	8934984	8935481	processed_pseudoger	3,40	0,8926569
ENSG00000260026	ENSG00000260026	16	86720688	86721954	lincRNA	3,40	1,3038915
ENSG00000177350	RPL13AP3	14	55766177	55767717	transcribed_processe	3,40	0,5682268
ENSG00000230157	ATP5G1P1	14	105536861	105537239	processed_pseudoger	3,40	0,4036702
ENSG00000198671	ENSG00000198671	12	65017468	65054124	antisense	3,40	0,5865398
ENSG00000241825	RPL7P42	12	68684595	68686308	processed_pseudoger	3,40	0,1217903
ENSG00000175728	C11orf44	11	130672956	130717352	lincRNA	3,40	0,4036702
ENSG00000254561	ENSG00000254561	11	119608423	119659284	antisense	3,40	0,1217903
ENSG00000254768	ENSG00000254768	11	22283730	22338245	antisense	3,40	0,8926569
ENSG00000236842	ENSG00000236842	10	75430571	75431588	antisense	3,40	0,4036702
ENSG00000253929	CASC21	8	127339274	127392631	lincRNA	3,40	1,3038915
ENSG00000271654	ENSG00000271654	8	18387108	18387777	processed_pseudoger	3,40	0,8926569
ENSG00000236978	ENSG00000236978	7	70837738	70838013	processed_pseudoger	3,40	1,3038915
ENSG00000235994	ENSG00000235994	6	167975924	167979776	lincRNA	3,40	1,8851183
ENSG00000248752	ENSG00000248752	5	126076800	126279801	lincRNA	3,40	1,3038915
ENSG00000250167	ENSG00000250167	5	135559577	135634874	antisense	3,40	0,5865398
ENSG00000271235	ENSG00000271235	3	22811390	22812047	processed_pseudoger	3,40	1,3038915
ENSG00000229568	SMC4P1	2	136544712	136545542	unprocessed_pseudog	3,40	0,8926569
ENSG00000225366	TDGF1P3	X	110520312	110523021	transcribed_processe	3,39	0,8926569
ENSG00000255511	ENSG00000255511	11	18870989	18873462	processed_pseudoger	3,38	0,4036702
ENSG00000262636	ENSG00000262636	16	11380859	11381118	processed_pseudoger	3,38	0,512233
ENSG00000228929	RPS13P2	1	52772194	52772648	processed_pseudoger	3,35	0,8926569
ENSG00000238178	ENSG00000238178	X	16152941	16170869	antisense	3,35	1,2188867
ENSG00000230629	RPS23P8	X	70962964	70963293	processed_pseudoger	3,35	1,1217155
ENSG00000255909	PDCD5P1	12	19413153	19413461	processed_pseudoger	3,34	1,6739638
ENSG00000272910	ENSG00000272910	3	179583262	179583762	antisense	3,34	0,4036702
ENSG00000229534	HNRNPA1P53	11	5571247	5572192	processed_pseudoger	3,33	0,512233
ENSG00000227852	ENSG00000227852	9	35592786	35592951	processed_pseudoger	3,33	0,2746192
ENSG00000255202	ENSG00000255202	11	33665220	33696701	antisense	3,32	7,06E-02
ENSG00000265055	ENSG00000265055	17	68096046	68101474	lincRNA	3,31	1,2188867
ENSG00000259354	ENSG00000259354	15	45448427	45513767	antisense	3,31	0,6526668
ENSG00000228962	HCG23	6	32390510	32393686	antisense	3,30	0,5865398
ENSG00000234964	FABP5P7	11	59781318	59781722	processed_pseudoger	3,30	0,4036702
ENSG00000260701	ENSG00000260701	16	77433382	77444336	antisense	3,30	0,4036702
ENSG00000261436	ENSG00000261436	16	60359842	60442245	lincRNA	3,30	1,3038915
ENSG00000273553	ENSG00000273553	16	27687182	27687522	sense_intronic	3,30	0,5865398
ENSG00000187812	ENSG00000187812	15	75775553	75786898	transcribed_unproces	3,30	1,8851183
ENSG00000227862	HNRNPA1P31	13	80619098	80620067	processed_pseudoger	3,30	1,3038915
ENSG00000249926	ENSG00000249926	12	131662596	131664704	lincRNA	3,30	1,3038915
ENSG00000258159	IMMP1LP2	12	113015607	113016098	processed_pseudoger	3,30	1,3038915
ENSG00000243316	GUCY2GP	10	112308181	112356574	unitary_pseudogene	3,30	0,5865398
ENSG00000260863	CYP2C60P	10	95009674	95009812	unprocessed_pseudog	3,30	0,5865398
ENSG00000234675	ENSG00000234675	6	148017422	148020974	lincRNA	3,30	1,8851183
ENSG00000226994	ENSG00000226994	2	34799850	35185689	lincRNA	3,30	1,3038915
ENSG00000204380	ENSG00000204380	2	158658337	158735002	antisense	3,30	3,27E-03

ENSG00000234026	ENSG00000234026	10	95228243	95231144	antisense	3,29	0,4850164
ENSG00000235944	ZNF815P	7	5823160	5854435	transcribed_unproces	3,29	0,2746192
ENSG00000225778	PROSER2-AS1	10	11849608	11894700	antisense	3,29	0,8468482
ENSG00000272973	ENSG00000272973	22	23856427	23857039	antisense	3,29	1,1217155
ENSG00000273693	C2orf27AP1	X	11085476	11086061	processed_pseudogen	3,28	1,3038915
ENSG00000241741	RPL7AP30	4	112788083	112788878	processed_pseudogen	3,28	0,1187106
ENSG00000255794	RMST	12	97431653	97565035	processed_transcript	3,27	1,1964007
ENSG00000182383	RPL27AP5	5	74990189	74990637	processed_pseudogen	3,27	1,1217155
ENSG00000213881	NPM1P6	8	61202350	61203220	processed_pseudogen	3,27	9,18E-02
ENSG00000258913	ENSG00000258913	14	104223584	104288069	lincRNA	3,26	1,5215308
ENSG00000240975	ENSG00000240975	11	39161453	39161967	processed_pseudogen	3,24	0,4332376
ENSG00000258136	ENSG00000258136	12	107736555	107759968	antisense	3,24	0,7582127
ENSG00000262160	ENSG00000262160	16	68225969	68229145	antisense	3,24	2,95E-02
ENSG00000267184	ENSG00000267184	17	69707193	69707373	processed_pseudogen	3,23	1,1217155
ENSG00000249502	ENSG00000249502	4	17587467	17614571	antisense	3,23	1,2188867
ENSG00000232936	ENSG00000232936	10	89645282	89650667	antisense	3,23	0,8108966
ENSG00000143429	ENSG00000143429	2	91617160	91659972	transcribed_unproces	3,23	0,6898868
ENSG00000230501	ANKRD30BP3	10	45156775	45176925	unprocessed_pseudog	3,22	0,4850164
ENSG00000204792	LINC01291	2	74918148	74938418	lincRNA	3,22	0,6526668
ENSG00000132832	LINC01260	20	44656451	44696096	antisense	3,20	0,7367804
ENSG00000261156	ENSG00000261156	17	34169372	34195937	lincRNA	3,20	0,8926569
ENSG00000276707	ENSG00000276707	17	36940049	36943456	antisense	3,20	0,4036702
ENSG00000227468	ENSG00000227468	14	105597691	105600209	lincRNA	3,20	1,8851183
ENSG00000241941	RPL32P26	12	62658823	62659225	processed_pseudogen	3,20	0,8926569
ENSG00000256192	ENSG00000256192	12	64266413	64266766	processed_pseudogen	3,20	1,8851183
ENSG00000257165	ENSG00000257165	12	78448995	78540675	lincRNA	3,20	0,8926569
ENSG00000237565	SSU72P5	11	4233286	4233869	processed_pseudogen	3,20	0,8926569
ENSG00000255171	LINC01499	11	41714568	41836442	lincRNA	3,20	0,4036702
ENSG00000253711	ENSG00000253711	8	61300164	61301069	antisense	3,20	0,8926569
ENSG00000253872	ENSG00000253872	8	95505406	95527524	lincRNA	3,20	1,8851183
ENSG00000232821	ENSG00000232821	7	19112474	19114271	antisense	3,20	0,8926569
ENSG00000243648	ENSG00000243648	5	134422838	134423335	processed_pseudogen	3,20	0,8926569
ENSG00000249667	LINC01259	4	38509767	38518056	lincRNA	3,20	1,3038915
ENSG00000250573	ENSG00000250573	4	10259445	10260138	processed_pseudogen	3,20	1,3038915
ENSG00000236665	ENSG00000236665	2	1158310	1160424	antisense	3,20	1,3038915
ENSG00000237556	KCND3-AS1	1	111909336	111910931	antisense	3,20	0,8926569
ENSG00000259849	VENTXP1	X	26558337	26561052	lincRNA	3,20	0,8926569
ENSG00000262519	TXNP4	17	4572206	4572515	processed_pseudogen	3,19	0,5682268
ENSG00000254744	ENSG00000254744	15	99970215	99974010	antisense	3,19	0,3565078
ENSG00000239835	ENSG00000239835	3	120028740	120029868	processed_pseudogen	3,19	1,8851183
ENSG00000249825	ENSG00000249825	5	80052374	80083654	antisense	3,19	2,95E-02
ENSG00000231234	SKP1P1	7	66423405	66423893	processed_pseudogen	3,19	1,0510989
ENSG00000249635	ENSG00000249635	4	106003317	106022478	antisense	3,18	0,4036702
ENSG00000272345	ENSG00000272345	6	24700907	24701793	antisense	3,18	0,4909563
ENSG00000261008	LINC01572	16	72283301	72665009	lincRNA	3,18	0,4332376
ENSG00000188985	DHFRP1	18	26170726	26171284	processed_pseudogen	3,17	0,5682268
ENSG00000236924	ENSG00000236924	9	6645956	6670635	lincRNA	3,17	0,512233
ENSG00000261266	ENSG00000261266	16	23687049	23687689	antisense	3,15	1,547231
ENSG00000273394	ENSG00000273394	3	113947005	113947570	antisense	3,12	1,6174211
ENSG00000243779	ENSG00000243779	18	13919603	13919923	processed_pseudogen	3,10	1,3038915
ENSG00000263553	ENSG00000263553	18	24673163	24674348	lincRNA	3,10	0,8926569
ENSG00000267506	ENSG00000267506	17	77546940	77563243	lincRNA	3,10	1,3038915
ENSG00000260177	ENSG00000260177	16	87780134	87806476	lincRNA	3,10	0,8926569
ENSG00000261259	ENSG00000261259	16	32951993	33028037	transcribed_processed	3,10	1,3038915
ENSG00000268505	ENSG00000268505	16	86192793	86198913	lincRNA	3,10	0,4036702
ENSG00000254529	ENSG00000254529	11	18863558	18864509	unprocessed_pseudog	3,10	0,8926569
ENSG00000254546	ENSG00000254546	11	18196657	18197337	processed_pseudogen	3,10	0,8926569
ENSG00000255045	ENSG00000255045	11	124744634	124746337	antisense	3,10	0,8926569
ENSG00000253634	ENSG00000253634	8	92565443	92655494	lincRNA	3,10	0,8926569

ENSG00000231533	ENSG00000231533	6	79420297	79421305	lincRNA	3,10	1,8851183
ENSG00000249937	ENSG00000249937	5	17684584	17955857	lincRNA	3,10	0,1217903
ENSG00000242936	RPL30P6	4	95644952	95645293	processed_pseudoger	3,10	1,3038915
ENSG00000230299	ENSG00000230299	2	85262144	85262267	processed_pseudoger	3,10	0,8926569
ENSG00000226715	ENSG00000226715	1	101639548	101787572	lincRNA	3,10	1,8851183
ENSG00000261642	ENSG00000261642	1	191151510	191154634	lincRNA	3,10	0,5865398
ENSG00000227528	DIAPH3-AS1	13	60012751	60044357	antisense	3,10	1,6739638
ENSG00000213318	ENSG00000213318	16	75192465	75193247	processed_pseudoger	3,09	1,6739638
ENSG00000259251	ENSG00000259251	15	62060503	62062434	antisense	3,09	1,6739638
ENSG00000239523	MYLK-AS1	3	123585542	123644568	antisense	3,09	0,8468482
ENSG00000267607	ENSG00000267607	19	10285801	10289019	antisense	3,09	9,18E-02
ENSG00000266916	ZNF793-AS1	19	37497159	37507046	antisense	3,09	0,3565078
ENSG00000234335	RPS4XP11	10	32102522	32103293	processed_pseudoger	3,07	1,4697798
ENSG00000248608	ENSG00000248608	4	25504997	25506675	processed_pseudoger	3,05	1,2188867
ENSG00000213862	ENSG00000213862	15	47730144	47730935	processed_pseudoger	3,05	0,1217903
ENSG00000223561	ENSG00000223561	7	25593351	25750994	lincRNA	3,04	1,0789438
ENSG00000271119	ENSG00000271119	5	1594626	1611467	lincRNA	3,03	1,8851183
ENSG00000270020	ENSG00000270020	16	86520383	86523897	lincRNA	3,02	1,547231
ENSG00000269210	ENSG00000269210	2	38959287	38960342	antisense	3,02	0,6309753
ENSG00000183298	RPSAP19	1	101786340	101787219	processed_pseudoger	3,02	9,18E-02
ENSG00000213959	ENSG00000213959	20	38614913	38615476	processed_pseudoger	3,00	1,1217155
ENSG00000245598	DACT3-AS1	19	46660364	46677447	antisense	3,00	0,7582127
ENSG00000248257	PSG10P	19	42839368	42855718	transcribed_unproces	3,00	0,8926569
ENSG00000259445	ENSG00000259445	15	81953303	81995666	lincRNA	3,00	1,6498059
ENSG00000257124	ENSG00000257124	12	83661009	83661719	lincRNA	3,00	1,8851183
ENSG00000229205	LINC00200	10	1159768	1164672	lincRNA	3,00	1,6498059
ENSG00000229019	ENSG00000229019	9	68426843	68429369	sense_intronic	3,00	0,5865398
ENSG00000253916	ENSG00000253916	8	133755845	133756646	processed_pseudoger	3,00	1,3038915
ENSG00000224999	VTA1P1	6	169296471	169297959	processed_pseudoger	3,00	0,4036702
ENSG00000248477	ENSG00000248477	5	69607099	69624049	transcribed_unproces	3,00	1,3038915
ENSG00000249746	ENSG00000249746	5	96213346	96215075	lincRNA	3,00	0,8926569
ENSG00000250600	ROPN1L-AS1	5	10441290	10441792	antisense	3,00	0,8926569
ENSG00000260066	ENSG00000260066	5	1725149	1728172	lincRNA	3,00	1,0177863
ENSG00000249755	ENSG00000249755	4	88527160	88528170	processed_pseudoger	3,00	0,4036702
ENSG00000224287	MSL3P1	2	233865437	233868444	transcribed_processe	3,00	1,6174211
ENSG00000231173	ENSG00000231173	2	741977	749856	lincRNA	3,00	1,3038915
ENSG00000235721	ENSG00000235721	2	110007675	110010783	processed_pseudoger	3,00	0,4036702
ENSG00000230937	MIR205HG	1	209428820	209432838	processed_transcript	3,00	0,8926569
ENSG00000204904	LINC01545	X	46887417	46899703	lincRNA	3,00	1,5943798
ENSG00000235834	ENSG00000235834	X	17528435	17587160	sense_intronic	3,00	0,512233
ENSG00000228196	PTPN2P1	1	178746683	178747754	processed_pseudoger	2,99	0,4909563
ENSG00000237943	PRKCQ-AS1	10	6580419	6616452	processed_transcript	2,98	0,1836819
ENSG00000239557	ENSG00000239557	3	52373652	52374882	processed_pseudoger	2,96	0,5682268
ENSG00000267255	ENSG00000267255	19	4429689	4430934	antisense	2,96	1,0789438
ENSG00000257599	OVCH1-AS1	12	29389294	29487488	antisense	2,95	1,2188867
ENSG00000261312	ENSG00000261312	16	19706351	19715383	antisense	2,95	1,3038915
ENSG00000225555	ENSG00000225555	21	34370802	34375348	antisense	2,95	0,512233
ENSG00000270945	HSPE1P7	16	74612596	74613221	processed_pseudoger	2,95	2,0846889
ENSG00000249772	ENSG00000249772	5	81113385	81114852	antisense	2,94	1,3038915
ENSG00000265401	ENSG00000265401	17	16382152	16382669	antisense	2,94	1,40E-02
ENSG00000248360	LINC00504	4	14470465	14888169	lincRNA	2,93	0,7582127
ENSG00000233296	ENSG00000233296	2	677186	697371	antisense	2,93	2,007219
ENSG00000239467	ENSG00000239467	2	170771113	170778148	lincRNA	2,92	0,6898868
ENSG00000226967	HAUS4P1	1	170369223	170370367	processed_pseudoger	2,92	0,7095968
ENSG00000236404	VLDLR-AS1	9	2422702	2643359	antisense	2,92	0,6898868
ENSG00000178464	ENSG00000178464	19	12643275	12643919	processed_pseudoger	2,91	4,86E-02
ENSG00000258609	LINC-ROR	18	57054559	57072119	lincRNA	2,91	0,7582127
ENSG00000255572	ENSG00000255572	12	7166674	7189069	antisense	2,91	1,1217155
ENSG00000249395	CASC9	8	75223404	75324741	lincRNA	2,91	1,6739638

ENSG00000254620	ENSG00000254620	20	58594417	58603973	lincRNA	2,90	0,8926569
ENSG00000267606	ENSG00000267606	19	56354493	56368053	lincRNA	2,90	0,8926569
ENSG00000257156	ENSG00000257156	12	89048187	89309553	lincRNA	2,90	1,3038915
ENSG00000236671	PRKG1-AS1	10	52230742	52314128	antisense	2,90	0,4036702
ENSG00000253281	ENSG00000253281	8	58255771	58272119	processed_transcript	2,90	0,8926569
ENSG00000282692	ENSG00000282692	8	1018757	1019704	lincRNA	2,90	0,8926569
ENSG00000233437	ENSG00000233437	7	56875385	56882146	transcribed_unproces	2,90	0,4036702
ENSG00000234826	ENSG00000234826	7	117998858	118004045	lincRNA	2,90	0,8926569
ENSG00000240790	ENSG00000240790	7	127644685	127652012	antisense	2,90	0,8926569
ENSG00000216966	ENSG00000216966	6	167208631	167208897	processed_pseudogen	2,90	2,95E-02
ENSG00000238254	ENSG00000238254	5	79849552	79849846	processed_pseudogen	2,90	1,3038915
ENSG00000248965	ENSG00000248965	5	168993000	168995677	antisense	2,90	0,4036702
ENSG00000226239	ENSG00000226239	20	32027753	32031575	antisense	2,88	1,8851183
ENSG00000236532	ENSG00000236532	21	28116094	28228667	lincRNA	2,87	0,7582127
ENSG00000268066	FMR1-AS1	X	147909431	147911817	processed_transcript	2,86	0,4909563
ENSG00000267009	ENSG00000267009	17	68413623	68524949	processed_transcript	2,85	7,06E-02
ENSG00000258882	ENSG00000258882	14	61811974	61970319	lincRNA	2,85	0,3318209
ENSG00000227879	PSPC1P1	13	19201637	19227432	unprocessed_pseudog	2,85	0,6526668
ENSG00000214283	RAD51AP1P1	3	136899076	136900076	processed_pseudogen	2,85	2,0846889
ENSG00000228541	ENSG00000228541	2	62463127	62464070	lincRNA	2,85	1,4697798
ENSG00000270194	ENSG00000270194	3	37241789	37244177	antisense	2,85	2,0846889
ENSG00000282418	ENSG00000282418	1	225465021	225473837	lincRNA	2,83	0,5682268
ENSG00000270823	ENSG00000270823	7	130495794	130498427	antisense	2,83	1,2188867
ENSG00000250195	ENSG00000250195	4	138819954	139012646	antisense	2,82	1,547231
ENSG00000255441	ENSG00000255441	19	51415724	51417425	antisense	2,82	1,6739638
ENSG00000248703	ENSG00000248703	12	131296110	131297972	lincRNA	2,82	0,2746192
ENSG00000276853	ENSG00000276853	12	65171262	65171917	sense_intronic	2,82	1,2188867
ENSG00000184414	ENSG00000184414	7	100570131	100571136	processed_pseudogen	2,81	0,7367804
ENSG00000188314	OR7D1P	19	9235144	9236076	unprocessed_pseudog	2,80	0,5865398
ENSG00000267175	ENSG00000267175	18	61592375	61748832	lincRNA	2,80	1,8851183
ENSG00000260575	ENSG00000260575	16	32250620	32254422	antisense	2,80	0,6526668
ENSG00000261218	ENSG00000261218	16	81738248	81767868	lincRNA	2,80	0,5865398
ENSG00000229775	ENSG00000229775	10	106140264	106188722	lincRNA	2,80	0,8926569
ENSG00000248222	ENSG00000248222	5	169013227	169037998	antisense	2,80	0,8926569
ENSG00000249601	LINC01187	5	170191579	170199141	lincRNA	2,80	0,8926569
ENSG00000250155	ENSG00000250155	5	36666214	36725195	antisense	2,80	0,8926569
ENSG00000240354	ENSG00000240354	3	161816909	161821908	lincRNA	2,80	0,8926569
ENSG00000277241	ENSG00000277241	3	177700346	177701072	processed_pseudogen	2,80	0,8926569
ENSG00000224626	ENSG00000224626	2	18784807	18787752	lincRNA	2,80	1,3038915
ENSG00000225446	ENSG00000225446	1	90851759	90855253	lincRNA	2,80	0,8926569
ENSG00000227538	HNRNPFP1	1	42040597	42041729	processed_pseudogen	2,80	0,5865398
ENSG00000271917	ENSG00000271917	1	164828436	164829952	sense_intronic	2,80	0,4036702
ENSG00000237171	ENSG00000237171	X	26223903	26224658	unprocessed_pseudog	2,80	0,8926569
ENSG00000260683	ENSG00000260683	X	127660631	127662530	lincRNA	2,80	0,4036702
ENSG00000254449	SF3A3P2	11	123597946	123599452	processed_pseudogen	2,79	1,547231
ENSG00000229388	ENSG00000229388	1	28643228	28648581	lincRNA	2,79	0,5682268
ENSG00000249631	ENSG00000249631	4	11625714	11813958	lincRNA	2,77	0,5865398
ENSG00000242071	RPL7AP6	14	69885340	69886140	processed_pseudogen	2,76	4,86E-02
ENSG00000267605	ENSG00000267605	19	37265939	37271518	lincRNA	2,75	1,6498059
ENSG00000178081	ULK4P3	15	30103720	30131757	transcribed_unproces	2,75	0,5682268
ENSG00000244619	ENSG00000244619	1	145892847	145893483	antisense	2,75	0,2746192
ENSG00000278740	ENSG00000278740	17	68188547	68189165	lincRNA	2,74	2,0633527
ENSG00000277299	ENSG00000277299	12	109948389	109949029	antisense	2,73	0,5865398
ENSG00000264859	DSG2-AS1	18	31542146	31556911	antisense	2,73	0,4909563
ENSG00000235010	ENSG00000235010	10	132181225	132185962	antisense	2,73	0,5682268
ENSG00000235749	ENSG00000235749	1	247639749	247747062	antisense	2,73	1,1964007
ENSG00000270269	IMMP1LP1	11	63632233	63632720	processed_pseudogen	2,72	1,5215308
ENSG00000236008	ENSG00000236008	2	8559833	8583792	lincRNA	2,71	1,6498059
ENSG00000236773	ENSG00000236773	1	224175476	224175706	processed_pseudogen	2,70	0,6526668

ENSG00000269564	ENSG00000269564	19	53788782	53789168	lincRNA	2,70	1,3038915
ENSG00000266840	ENSG00000266840	18	69704978	69724975	antisense	2,70	0,8926569
ENSG00000266952	LINC01538	18	64213082	64260055	lincRNA	2,70	1,8851183
ENSG00000260338	LINC01570	16	5601169	5616196	antisense	2,70	0,4036702
ENSG00000261727	ENSG00000261727	16	32008438	32084785	transcribed_processed	2,70	1,3038915
ENSG00000227659	CLYBL-AS2	13	99690081	99690971	antisense	2,70	0,8926569
ENSG00000228074	UBBP5	13	87219004	87219269	processed_pseudogen	2,70	0,8926569
ENSG00000237361	TUSC8	13	44400250	44405984	lincRNA	2,70	0,8926569
ENSG00000257004	ENSG00000257004	12	12668982	12685075	lincRNA	2,70	0,8926569
ENSG00000257292	ENSG00000257292	12	38205098	38206009	unprocessed_pseudog	2,70	0,8926569
ENSG00000276667	ENSG00000276667	9	19895800	19895973	unprocessed_pseudog	2,70	0,8926569
ENSG00000254286	ENSG00000254286	8	126557875	126713415	antisense	2,70	0,5865398
ENSG00000231382	NBPF21P	3	36616006	36637457	unprocessed_pseudog	2,70	0,5865398
ENSG00000231254	PCED1CP	2	212832161	212832945	processed_pseudogen	2,70	1,8851183
ENSG00000203307	ENSG00000203307	1	166081183	166087483	antisense	2,70	1,3038915
ENSG00000250321	ENSG00000250321	4	342821	342962	processed_pseudogen	2,70	1,5943798
ENSG00000277077	ENSG00000277077	13	21287634	21287828	unprocessed_pseudog	2,69	0,8108966
ENSG00000214533	KRT18P33	2	65666695	65667737	processed_pseudogen	2,69	1,4697798
ENSG00000230176	LINC01433	20	4193065	4195943	antisense	2,69	0,8468482
ENSG00000272143	FGF14-AS2	13	102394630	102395703	lincRNA	2,67	0,5682268
ENSG00000235590	GNAS-AS1	20	58818919	58850903	antisense	2,67	1,547231
ENSG00000261599	HERC2P8	16	33094204	33129090	transcribed_unproces	2,67	0,8926569
ENSG00000226051	ZNF503-AS1	10	75269819	75373500	lincRNA	2,67	1,6498059
ENSG00000258608	DNAJC19P9	14	45290036	45290386	processed_pseudogen	2,66	0,4332376
ENSG00000248632	ENSG00000248632	4	164968587	164970002	processed_pseudogen	2,66	0,8468482
ENSG00000267160	ENSG00000267160	17	44673689	44676257	antisense	2,66	1,1964007
ENSG00000272678	ENSG00000272678	3	123283593	123283983	antisense	2,64	0,6526668
ENSG00000259907	ENSG00000259907	14	71181456	71182106	lincRNA	2,64	1,1217155
ENSG00000258895	ENSG00000258895	15	94033658	94034177	processed_pseudogen	2,64	0,1217903
ENSG00000257285	ENSG00000257285	14	22929609	22955562	antisense	2,63	0,8926569
ENSG00000233485	ENSG00000233485	1	15326680	15343876	antisense	2,62	1,5943798
ENSG00000259498	ENSG00000259498	15	63046034	63049387	antisense	2,61	0,5865398
ENSG00000237036	ZEB1-AS1	10	31206278	31320447	antisense	2,61	0,4036702
ENSG00000186162	CIDECP	3	10014238	10026365	transcribed_unproces	2,61	0,3565078
ENSG00000224973	LARGE-AS1	22	33725014	33750843	processed_transcript	2,60	0,5865398
ENSG00000235102	ENSG00000235102	20	41331123	41331660	processed_pseudogen	2,60	1,3038915
ENSG00000266573	ENSG00000266573	18	24725781	24929076	lincRNA	2,60	1,3038915
ENSG00000273388	ENSG00000273388	17	10291820	10317926	antisense	2,60	0,8926569
ENSG00000243122	ENSG00000243122	15	37490691	37491103	processed_pseudogen	2,60	0,5865398
ENSG00000260139	CSPG4P13	15	77894684	77904674	transcribed_unproces	2,60	1,8851183
ENSG00000258804	ENSG00000258804	14	86905778	86922755	lincRNA	2,60	0,5865398
ENSG00000257512	ENSG00000257512	12	93314809	93315941	transcribed_processed	2,60	0,8926569
ENSG00000231039	ENSG00000231039	10	5234358	5263408	lincRNA	2,60	0,8926569
ENSG00000249917	LINC00536	8	115950511	116325059	lincRNA	2,60	0,8926569
ENSG00000253432	ENSG00000253432	8	139096305	139102830	lincRNA	2,60	0,5865398
ENSG00000254349	MIR2052HG	8	74599775	74823313	lincRNA	2,60	1,8851183
ENSG00000239311	ENSG00000239311	3	111466313	111497095	lincRNA	2,60	1,3038915
ENSG00000203897	SPATA42	1	108857217	108858524	antisense	2,60	1,8851183
ENSG00000231512	ENSG00000231512	1	243029512	243052252	lincRNA	2,60	1,3038915
ENSG00000236846	ENSG00000236846	1	223144049	223144954	lincRNA	2,60	1,3038915
ENSG00000215162	LINC00269	X	69179557	69209924	lincRNA	2,60	1,3038915
ENSG00000230265	ENSG00000230265	X	26557675	26558330	processed_pseudogen	2,60	1,8851183
ENSG00000256594	ENSG00000256594	12	9617284	9658412	transcribed_unproces	2,59	0,4036702
ENSG00000261033	ENSG00000261033	17	20008051	20009234	antisense	2,59	2,0846889
ENSG00000259746	HSPE1P3	15	90634725	90635033	processed_pseudogen	2,58	1,6174211
ENSG00000238072	ENSG00000238072	7	129410113	129410370	processed_pseudogen	2,58	0,7367804
ENSG00000236333	TRHDE-AS1	12	72253508	72274907	processed_transcript	2,58	0,5682268
ENSG00000249679	ENSG00000249679	4	185471516	185472263	antisense	2,56	1,6174211
ENSG00000246889	ENSG00000246889	11	70372246	70398488	antisense	2,55	1,1217155

ENSG00000266921	ENSG00000266921	19	43996896	44002836	antisense	2,55	0,2161218
ENSG00000258736	ENSG00000258736	14	104653548	104655787	lincRNA	2,55	0,5502547
ENSG00000184523	PTGER4P2	9	62843919	62844170	processed_pseudogen	2,55	0,512233
ENSG00000251087	ALG1L3P	4	9703754	9710812	unprocessed_pseudog	2,55	1,6739638
ENSG00000259726	CSPG4P11	15	84186752	84197384	unprocessed_pseudog	2,54	1,6739638
ENSG00000106133	NSUN5P2	7	72947581	72954790	transcribed_unproces	2,54	0,1187106
ENSG00000231120	BTF3P10	6	149977922	149978416	processed_pseudogen	2,53	1,8851183
ENSG00000273585	ENSG00000273585	15	101613676	101614339	lincRNA	2,53	0,512233
ENSG00000259867	ENSG00000259867	16	80155053	80563135	antisense	2,53	0,8926569
ENSG00000163364	LINC01116	2	176629589	176637931	lincRNA	2,53	0,7095968
ENSG00000255539	ENSG00000255539	11	70324871	70327209	antisense	2,52	0,8926569
ENSG00000237121	PIEZO1P2	20	58740532	58783821	unprocessed_pseudog	2,50	0,4036702
ENSG00000268536	ENSG00000268536	19	4785120	4791207	lincRNA	2,50	0,8926569
ENSG00000262339	ENSG00000262339	17	82978525	82981738	antisense	2,50	1,3038915
ENSG00000261404	ENSG00000261404	16	74054151	74296762	lincRNA	2,50	0,7582127
ENSG00000257325	ENSG00000257325	12	100852331	100859262	antisense	2,50	0,8926569
ENSG00000254532	ENSG00000254532	11	30044058	30084343	lincRNA	2,50	0,8926569
ENSG00000254619	ENSG00000254619	11	33810145	33811178	lincRNA	2,50	0,5865398
ENSG00000227683	ENSG00000227683	10	45164228	45181427	lincRNA	2,50	1,3038915
ENSG00000230945	LINC01507	9	80030579	80034555	lincRNA	2,50	0,8926569
ENSG00000253290	ENSG00000253290	8	27903229	27903495	unprocessed_pseudog	2,50	1,3038915
ENSG00000254007	ENSG00000254007	8	5659679	5670077	lincRNA	2,50	0,5865398
ENSG00000254178	ENSG00000254178	8	25834129	25840135	lincRNA	2,50	1,8851183
ENSG00000228680	ENSG00000228680	7	43239066	43249268	antisense	2,50	0,8926569
ENSG00000249647	C5orf66-AS2	5	135236234	135248179	lincRNA	2,50	1,8851183
ENSG00000251309	ENSG00000251309	4	101976894	102036903	antisense	2,50	1,8851183
ENSG00000231054	ENSG00000231054	2	45168583	45169414	lincRNA	2,50	1,6174211
ENSG00000238098	ABCA17P	16	2339150	2426699	unitary_pseudogene	2,50	0,3565078
ENSG00000240591	ENSG00000240591	22	31454251	31464204	antisense	2,50	0,8926569
ENSG00000267058	ENSG00000267058	19	43891804	43901805	lincRNA	2,49	0,1392851
ENSG00000234297	ENSG00000234297	9	15055057	15056052	processed_pseudogen	2,48	1,1964007
ENSG00000249353	NPM1P27	5	93682838	93683667	processed_pseudogen	2,48	0,4909563
ENSG00000132967	HMGB1P5	3	22381819	22382929	transcribed_processe	2,48	1,0789438
ENSG00000184100	BRD7P2	3	160100850	160102793	processed_pseudogen	2,47	0,6898868
ENSG00000223756	TSSC2	11	3380961	3408978	transcribed_unproces	2,47	1,6174211
ENSG00000213140	ELK2AP	14	105672308	105673314	processed_pseudogen	2,45	1,2188867
ENSG00000251603	ENSG00000251603	4	151667224	151670502	antisense	2,45	0,7582127
ENSG00000246560	ENSG00000246560	4	102828055	102844075	antisense	2,45	0,512233
ENSG00000228063	LYPLAL1-AS1	1	219086602	219173961	antisense	2,44	1,0510989
ENSG00000259248	USP3-AS1	15	63544247	63601589	antisense	2,43	4,86E-02
ENSG00000228407	ENSG00000228407	1	52160261	52160600	processed_pseudogen	2,43	0,512233
ENSG00000236439	ENSG00000236439	1	202471864	202472117	processed_pseudogen	2,43	0,6526668
ENSG00000276026	ENSG00000276026	20	1117847	1118450	antisense	2,43	0,8108966
ENSG00000225308	ASS1P11	7	21220213	21221428	processed_pseudogen	2,42	1,0510989
ENSG00000248787	ENSG00000248787	3	125908005	125910272	lincRNA	2,42	0,3565078
ENSG00000273702	ENSG00000273702	17	59618553	59619714	lincRNA	2,41	1,8851183
ENSG00000267811	ENSG00000267811	11	62771120	62771606	antisense	2,41	1,6174211
ENSG00000233213	KCNJ6-AS1	21	37717102	37719569	sense_intronic	2,40	1,3038915
ENSG00000225069	ENSG00000225069	20	25099105	25099928	processed_pseudogen	2,40	1,3038915
ENSG00000225831	RPS18P1	20	5532878	5533335	processed_pseudogen	2,40	0,8926569
ENSG00000264212	ENSG00000264212	18	76256891	76258337	lincRNA	2,40	1,8851183
ENSG00000263571	ENSG00000263571	17	34161737	34164251	lincRNA	2,40	1,8851183
ENSG00000261466	ENSG00000261466	16	33548297	33554408	lincRNA	2,40	1,8851183
ENSG00000274367	ENSG00000274367	16	2981175	2981591	lincRNA	2,40	0,5865398
ENSG00000258785	LINC01580	15	93900701	93984150	lincRNA	2,40	0,5865398
ENSG00000259527	LINC00052	15	87576929	87579866	lincRNA	2,40	1,3038915
ENSG00000281721	LINC01080	13	80011077	80028283	lincRNA	2,40	1,3038915
ENSG00000255946	ENSG00000255946	12	120740470	120761592	antisense	2,40	1,8851183
ENSG00000234170	ENSG00000234170	10	2446253	2447352	lincRNA	2,40	1,3038915

ENSG00000212994	RPS26P6	8	100895771	100896118	processed_pseudoger	2,40	1,3038915
ENSG00000251563	IARS2P1	8	87598462	87601634	processed_pseudoger	2,40	0,5865398
ENSG00000253394	LINC00534	8	90221488	90569318	lincRNA	2,40	1,8851183
ENSG00000281657	LINC00976	8	128904773	128966001	lincRNA	2,40	0,8926569
ENSG00000224683	RPL36AP29	7	16208945	16209265	processed_pseudoger	2,40	0,5865398
ENSG00000230190	ENSG00000230190	7	147671711	147673143	sense_intronic	2,40	1,8851183
ENSG00000232053	ENSG00000232053	7	136092925	136437426	lincRNA	2,40	0,8926569
ENSG00000234352	ENSG00000234352	7	136685559	137182107	antisense	2,40	0,8926569
ENSG00000204661	C5orf60	5	179641544	179645046	processed_transcript	2,40	1,8851183
ENSG00000250634	LINC01182	4	13655179	13977075	lincRNA	2,40	0,5865398
ENSG00000250997	ENSG00000250997	4	160521829	160522311	processed_pseudoger	2,40	1,8851183
ENSG00000260519	ENSG00000260519	4	42657496	42657928	antisense	2,40	1,8851183
ENSG00000214691	ENSG00000214691	2	41877074	41894046	lincRNA	2,40	0,5865398
ENSG00000223929	MIR4432HG	2	60359216	60391375	lincRNA	2,40	0,8926569
ENSG00000224638	ENSG00000224638	2	173880865	173899428	lincRNA	2,40	1,3038915
ENSG00000236348	PSMC1P10	2	17385091	17386417	processed_pseudoger	2,40	0,8926569
ENSG00000216866	RPS2P55	X	40934982	40935864	processed_pseudoger	2,40	1,6739638
ENSG00000259370	ENSG00000259370	15	62827675	62884034	antisense	2,39	1,3038915
ENSG00000267112	ENSG00000267112	18	54885866	54898083	antisense	2,39	1,4448456
ENSG00000250983	ENSG00000250983	3	133546071	133546272	processed_pseudoger	2,38	1,5215308
ENSG00000234477	ENSG00000234477	17	40921430	40975926	antisense	2,38	1,547231
ENSG00000242759	LINC00882	3	106836811	107240641	lincRNA	2,38	1,5215308
ENSG00000175886	RPL7AP66	18	39334872	39335672	processed_pseudoger	2,37	0,4332376
ENSG00000254789	ENSG00000254789	11	15572023	15622391	lincRNA	2,36	1,6739638
ENSG00000225721	ENSG00000225721	1	44759037	44775810	antisense	2,36	1,6498059
ENSG00000257452	ENSG00000257452	12	112907628	113017751	antisense	2,36	0,7582127
ENSG00000242258	LINC00996	7	150433654	150448140	lincRNA	2,36	1,0510989
ENSG00000240057	ENSG00000240057	3	113019532	113183301	antisense	2,36	2,0633527
ENSG00000225868	ENSG00000225868	19	37823722	37855215	lincRNA	2,35	0,7367804
ENSG00000228620	ENSG00000228620	22	38424288	38427336	antisense	2,33	0,8926569
ENSG00000254946	ENSG00000254946	11	15552855	15591098	lincRNA	2,33	1,4448456
ENSG00000179101	ENSG00000179101	X	123514473	123514872	processed_pseudoger	2,33	1,5215308
ENSG00000275765	ENSG00000275765	5	151770242	151771508	lincRNA	2,32	0,6898868
ENSG00000254485	ENSG00000254485	3	9292588	9363303	antisense	2,32	1,2188867
ENSG00000242391	ENSG00000242391	1	52881216	52881730	antisense	2,31	0,6309753
ENSG00000245748	ENSG00000245748	4	7030554	7046231	antisense	2,30	0,1187106
ENSG00000225083	GRTP1-AS1	13	113351673	113361868	antisense	2,30	1,6174211
ENSG00000224141	MIR548XHG	21	18561265	18760003	lincRNA	2,30	0,8926569
ENSG00000229005	HNF4A-AS1	20	44372746	44395706	antisense	2,30	1,8851183
ENSG00000278816	ENSG00000278816	20	5061037	5061340	lincRNA	2,30	1,8851183
ENSG00000259200	ENSG00000259200	15	45705078	45931069	lincRNA	2,30	0,5865398
ENSG00000259672	ENSG00000259672	15	63098870	63110403	lincRNA	2,30	1,3038915
ENSG00000232881	RPS10P21	13	71913704	71914178	processed_pseudoger	2,30	1,3038915
ENSG00000254456	ENSG00000254456	11	25734770	25781758	lincRNA	2,30	0,5865398
ENSG00000254037	ENSG00000254037	8	119480279	119480775	processed_pseudoger	2,30	1,3038915
ENSG00000261437	ENSG00000261437	8	94637285	94639467	antisense	2,30	1,8851183
ENSG00000225329	LHFPL3-AS2	7	104894628	104926645	processed_transcript	2,30	0,5502547
ENSG00000248430	HMGB3P16	5	112452703	112452989	processed_pseudoger	2,30	1,3038915
ENSG00000250250	ENSG00000250250	5	15602189	15607348	antisense	2,30	1,8851183
ENSG00000251206	ENSG00000251206	5	67268022	67270182	lincRNA	2,30	0,8926569
ENSG00000230292	NAALADL2-AS3	3	175079307	175115242	processed_transcript	2,30	1,8851183
ENSG00000225982	ENSG00000225982	1	182086551	182090112	lincRNA	2,30	1,3038915
ENSG00000259623	ENSG00000259623	17	41848518	41851447	sense_overlapping	2,30	1,6739638
ENSG00000249684	ENSG00000249684	5	177950335	177963960	antisense	2,29	0,7582127
ENSG00000267440	ENSG00000267440	17	43680273	43705884	lincRNA	2,27	1,547231
ENSG00000205740	ENSG00000205740	10	971146	988341	processed_transcript	2,26	2,007219
ENSG00000239473	RPL7P38	12	80028893	80029631	processed_pseudoger	2,25	2,0846889
ENSG00000215004	MESTP4	3	29087396	29088599	processed_pseudoger	2,25	2,0846889
ENSG00000261872	ENSG00000261872	17	47169826	47171049	antisense	2,24	2,007219

ENSG00000271127	ENSG00000271127	22	15796959	15798346	sense_intronic	2,24	0,6309753
ENSG00000277498	ENSG00000277498	2	61858137	61860298	unprocessed_pseudog	2,23	0,6526668
ENSG00000173811	CCDC13-AS1	3	42732575	42746768	antisense	2,22	0,6898868
ENSG00000247708	STX18-AS1	4	4542131	4710938	antisense	2,21	0,4909563
ENSG00000233271	ENSG00000233271	1	54980950	54992274	antisense	2,21	1,6739638
ENSG00000212802	RPL15P3	6	12514110	12514724	processed_pseudogen	2,21	0,5865398
ENSG00000276354	UPF3BP1	X	119831616	119831759	unprocessed_pseudog	2,21	0,8108966
ENSG00000267083	KRT18P61	17	60810165	60811462	processed_pseudogen	2,20	1,3038915
ENSG00000267667	ENSG00000267667	17	61130704	61135964	antisense	2,20	1,3038915
ENSG00000260979	ENSG00000260979	16	8962706	8966990	lincRNA	2,20	1,3038915
ENSG00000261439	ENSG00000261439	16	56192614	56194518	antisense	2,20	0,8926569
ENSG00000258831	ENSG00000258831	15	93882082	93886743	lincRNA	2,20	1,3038915
ENSG00000226903	LINC00354	13	111899985	111901176	lincRNA	2,20	0,8926569
ENSG00000257191	ENSG00000257191	12	78960258	79045644	antisense	2,20	1,3038915
ENSG00000255411	ENSG00000255411	11	13785390	13848002	lincRNA	2,20	0,8926569
ENSG00000189275	LINC01164	10	131772398	131790199	lincRNA	2,20	1,8851183
ENSG00000226762	ENSG00000226762	10	3240371	3257210	lincRNA	2,20	1,3038915
ENSG00000233569	ENSG00000233569	9	117648606	117657027	lincRNA	2,20	0,5865398
ENSG00000253684	BUD31P1	8	33641581	33641939	processed_pseudogen	2,20	1,8851183
ENSG00000254100	ENSG00000254100	8	38552248	38559020	lincRNA	2,20	1,3038915
ENSG00000224970	ENSG00000224970	7	142875836	142892743	antisense	2,20	1,8851183
ENSG00000253183	ENSG00000253183	7	139502453	139503812	processed_pseudogen	2,20	0,8926569
ENSG00000223786	ENSG00000223786	6	74069451	74690727	lincRNA	2,20	0,8926569
ENSG00000233358	ENSG00000233358	6	22744395	23031780	lincRNA	2,20	1,3038915
ENSG00000251026	ENSG00000251026	5	104079911	104105403	lincRNA	2,20	1,8851183
ENSG00000249534	LINC01258	4	38420662	38523180	lincRNA	2,20	1,3038915
ENSG00000205837	LINC00487	2	6728177	6770311	lincRNA	2,20	1,8851183
ENSG00000228222	ENSG00000228222	2	167293171	167558333	lincRNA	2,20	1,3038915
ENSG00000235495	ENSG00000235495	2	67565604	67684077	lincRNA	2,20	0,8926569
ENSG00000233558	ENSG00000233558	6	116258493	116259115	processed_pseudogen	2,20	0,7095968
ENSG00000234996	ENSG00000234996	1	202861754	202875241	transcribed_processed	2,19	0,7095968
ENSG00000262728	ENSG00000262728	15	32586105	32615158	antisense	2,18	1,1964007
ENSG00000266614	ENSG00000266614	18	76259166	76260114	lincRNA	2,18	1,6498059
ENSG00000234323	ENSG00000234323	9	106278392	106604795	lincRNA	2,18	1,1217155
ENSG00000250994	ENSG00000250994	5	134436711	134492519	lincRNA	2,18	1,6498059
ENSG00000267345	ENSG00000267345	19	37075113	37078605	antisense	2,18	2,007219
ENSG00000272002	ENSG00000272002	2	7260871	7261504	lincRNA	2,18	1,3038915
ENSG00000223911	ENSG00000223911	2	147899401	147902956	antisense	2,18	1,4697798
ENSG00000272844	ENSG00000272844	3	112990447	112991153	antisense	2,17	1,0177863
ENSG00000213104	NPM1P46	2	197379701	197380892	processed_pseudogen	2,17	0,8926569
ENSG00000223576	ENSG00000223576	13	19841827	19843672	antisense	2,17	1,6739638
ENSG00000232917	HSPE1P6	1	203903723	203904013	processed_pseudogen	2,16	1,4448456
ENSG00000226478	UPF3AP1	17	16745636	16746761	processed_pseudogen	2,15	1,0789438
ENSG00000203876	ADD3-AS1	10	109940104	110008381	processed_transcript	2,14	1,3038915
ENSG00000227477	STK4-AS1	20	44963794	44966402	lincRNA	2,14	0,9880903
ENSG00000278090	ENSG00000278090	15	99028538	99031053	lincRNA	2,13	1,1047191
ENSG00000261762	ENSG00000261762	15	78589123	78591276	antisense	2,13	0,8468482
ENSG00000272168	CASC15	6	21664772	22214505	lincRNA	2,13	0,512233
ENSG00000204894	ENSG00000204894	7	152367171	152367260	processed_pseudogen	2,11	0,4909563
ENSG00000237481	ENSG00000237481	1	229319403	229323087	antisense	2,10	1,1964007
ENSG00000230107	ENSG00000230107	22	42438023	42446195	lincRNA	2,10	1,3038915
ENSG00000278292	RFPL4AP6	22	29432944	29435005	unprocessed_pseudog	2,10	1,3038915
ENSG00000222042	ENSG00000222042	21	25169431	25333825	lincRNA	2,10	1,3038915
ENSG00000241054	ENSG00000241054	20	20094401	20095684	antisense	2,10	1,3038915
ENSG00000265975	ENSG00000265975	17	8967523	8976995	lincRNA	2,10	0,7582127
ENSG00000258455	ENSG00000258455	14	55325834	55339501	antisense	2,10	0,8926569
ENSG00000258958	ENSG00000258958	14	87323753	87332390	lincRNA	2,10	1,8851183
ENSG00000225760	LINC00431	13	110983307	110990564	lincRNA	2,10	1,3038915
ENSG00000256699	ENSG00000256699	12	129208601	129212662	lincRNA	2,10	0,8926569

ENSG00000225208	ENSG00000225208	10	101311018	101311505	lincRNA		2,10	0,8926569
ENSG00000270727	ENSG00000270727	10	20547171	20547422	processed_pseudogen		2,10	1,3038915
ENSG00000253983	ENSG00000253983	8	74199396	74208441	antisense		2,10	1,8851183
ENSG00000254202	ENSG00000254202	8	83912713	84140283	lincRNA		2,10	0,5865398
ENSG00000254269	ENSG00000254269	8	1104882	1262580	processed_transcript		2,10	0,8926569
ENSG00000280511	ENSG00000280511	6	76561328	76562590	lincRNA		2,10	0,8926569
ENSG00000227762	GUSBP8	5	99532628	99534611	unprocessed_pseudog		2,10	1,3038915
ENSG00000249400	HMGB3P17	5	123468781	123469429	processed_pseudogen		2,10	1,3038915
ENSG00000251296	ENSG00000251296	4	10167159	10167765	processed_pseudogen		2,10	1,3038915
ENSG00000273238	ENSG00000273238	4	573880	574412	lincRNA		2,10	1,3038915
ENSG00000186543	CROCCP5	1	21434318	21437558	unprocessed_pseudog		2,10	0,8926569
ENSG00000230817	LINC01362	1	82903183	83166815	lincRNA		2,10	1,8851183
ENSG00000229335	DANT1	X	115840964	115843050	antisense		2,10	1,8851183
ENSG00000261723	ENSG00000261723	16	23605841	23624107	antisense		2,09	1,5215308
ENSG00000267677	ENSG00000267677	18	59360700	59386749	lincRNA		2,09	1,2188867
ENSG00000260862	ENSG00000260862	16	82773319	82829638	sense_intronic		2,09	0,8108966
ENSG00000229893	ENSG00000229893	7	27733064	27740395	antisense		2,08	1,547231
ENSG00000236801	RPL24P8	9	70217195	70217668	processed_pseudogen		2,08	0,7095968
ENSG00000268412	TRMT112P6	2	26028208	26028612	processed_pseudogen		2,07	0,3565078
ENSG00000235381	ENSG00000235381	6	155253139	155256724	antisense		2,07	0,9880903
ENSG00000229887	HNRNPA1P6	1	58047889	58049335	transcribed_unproces		2,07	1,6739638
ENSG00000231607	DLEU2	13	49982552	50125720	antisense		2,07	1,3038915
ENSG00000236266	ENSG00000236266	1	7810242	7827342	antisense		2,07	0,8468482
ENSG00000255090	MIR100HG	11	122155422	122422871	lincRNA		2,06	0,7582127
ENSG00000262468	LINC01569	16	4245825	4253789	lincRNA		2,06	1,5943798
ENSG00000265688	MAFG-AS1	17	81927829	81930753	antisense		2,06	1,5215308
ENSG00000204758	ENSG00000204758	5	172954786	172959392	antisense		2,05	1,2188867
ENSG00000263563	UBBP4	17	22202995	22205154	transcribed_unproces		2,05	0,4036702
ENSG00000253516	HMGB1P41	8	80812142	80812581	processed_pseudogen		2,04	1,6739638
ENSG00000213261	EEF1B2P6	7	131661952	131662624	processed_pseudogen		2,04	1,4697798
ENSG00000232709	MARK2P9	10	92418667	92420875	processed_pseudogen		2,03	1,8851183
ENSG00000227620	ALG1L8P	11	67785273	67792335	unprocessed_pseudog		2,03	1,6174211
ENSG00000254418	ENSG00000254418	11	14262846	14273691	antisense		2,03	1,0789438
ENSG00000219682	ENSG00000219682	6	25140003	25141403	processed_pseudogen		2,02	0,4909563
ENSG00000250405	ENSG00000250405	5	130994253	130996164	processed_pseudogen		2,01	1,0789438
ENSG00000235241	ENSG00000235241	1	16889095	16889602	processed_pseudogen		2,01	1,4448456
ENSG00000249858	SNX5P1	4	76344550	76345590	processed_pseudogen		2,00	0,512233
ENSG00000233393	ENSG00000233393	21	36104881	36109690	lincRNA		2,00	0,8926569
ENSG00000269316	ENSG00000269316	19	22609606	22610601	processed_pseudogen		2,00	0,8926569
ENSG00000265101	ENSG00000265101	18	78925064	78927441	lincRNA		2,00	1,3038915
ENSG00000277310	ENSG00000277310	18	50256036	50256461	sense_intronic		2,00	1,4448456
ENSG00000266126	ENSG00000266126	17	19929372	19929737	sense_intronic		2,00	1,1217155
ENSG00000257180	ENSG00000257180	16	6056975	6092954	sense_intronic		2,00	1,8851183
ENSG00000260643	ENSG00000260643	16	81053665	81087651	processed_transcript		2,00	1,6174211
ENSG00000276822	ENSG00000276822	16	74393076	74393183	unprocessed_pseudog		2,00	0,5865398
ENSG00000278716	ENSG00000278716	16	85489813	85490831	lincRNA		2,00	1,3038915
ENSG00000259485	ENSG00000259485	15	97295881	97432094	lincRNA		2,00	1,3038915
ENSG00000259671	MTCYBP23	15	58154960	58156061	processed_pseudogen		2,00	1,2188867
ENSG00000259692	ENSG00000259692	15	81660482	81871125	lincRNA		2,00	0,8468482
ENSG00000259035	ENSG00000259035	14	81741002	82030349	lincRNA		2,00	0,8926569
ENSG00000224419	KRT18P27	13	90230384	90231682	processed_pseudogen		2,00	1,3038915
ENSG00000226317	LINC00351	13	85363601	85544570	lincRNA		2,00	1,8851183
ENSG00000277227	ENSG00000277227	13	28778224	28778448	unprocessed_pseudog		2,00	1,3038915
ENSG00000256442	ENSG00000256442	12	9647014	9648009	antisense		2,00	0,5865398
ENSG00000258279	LINC00592	12	52210930	52223804	lincRNA		2,00	0,8926569
ENSG00000198217	OR51H2P	11	4876557	4877462	unprocessed_pseudog		2,00	1,8851183
ENSG00000205494	OR52A4P	11	5120621	5124513	transcribed_unproces		2,00	1,8851183
ENSG00000223581	ENSG00000223581	10	7097152	7097675	lincRNA		2,00	0,8926569
ENSG00000225768	ENSG00000225768	10	104474939	104480274	lincRNA		2,00	1,3038915

ENSG00000225639	PGAM1P2	9	35943210	35943934	processed_pseudoger	2,00	1,8851183
ENSG00000237548	TTLL11-IT1	9	121884636	121963719	lincRNA	2,00	1,3038915
ENSG00000253728	ENSG00000253728	8	142834138	142834940	antisense	2,00	1,8851183
ENSG00000228334	ENSG00000228334	7	3083252	3086421	lincRNA	2,00	0,8926569
ENSG00000220212	OR4F1P	6	105919	106856	unprocessed_pseudog	2,00	0,8926569
ENSG00000227131	ENSG00000227131	6	40271566	40276237	lincRNA	2,00	0,8926569
ENSG00000236336	ENSG00000236336	6	4774526	4775408	antisense	2,00	1,8851183
ENSG00000213433	RPLP1P6	5	151765859	151766378	transcribed_processe	2,00	1,6174211
ENSG00000272139	ENSG00000272139	5	122311297	122311673	antisense	2,00	0,5865398
ENSG00000173966	ENSG00000173966	4	53575713	53576387	processed_pseudoger	2,00	1,8851183
ENSG00000228919	ENSG00000228919	4	7939001	7940296	lincRNA	2,00	1,4697798
ENSG00000246095	LINC01096	4	13546075	13547801	lincRNA	2,00	1,8851183
ENSG00000248749	ENSG00000248749	4	84371393	84380189	lincRNA	2,00	1,3038915
ENSG00000249752	ENSG00000249752	4	149027445	149062522	lincRNA	2,00	1,8851183
ENSG00000249993	BFSP2-AS1	3	133429269	133455776	antisense	2,00	0,5865398
ENSG00000271893	ENSG00000271893	2	195451778	195515699	lincRNA	2,00	1,3038915
ENSG00000260021	ENSG00000260021	1	202810238	202810829	antisense	2,00	1,6739638
ENSG00000261060	ENSG00000261060	1	179590372	179591305	sense_overlapping	2,00	1,3038915

Supplementary Table S4: List of the 6,236 lncRNAs upregulated in cumulus granulosa cells

Gene ID	Gene Name	Chromo	Start_position	End_position	Gene_biotype	Fold Change	q-value(%)
ENSG00000245532	NEAT1	11	65422774	65445540	lincRNA	18882,70	0
ENSG00000242396	ENSG00000242396	1	54886875	54888001	antisense	13618,82	0
ENSG00000231991	ANXA2P2	9	33624274	33625293	processed_pseudogene	12551,54	0
ENSG00000229807	XIST	X	73820651	73852753	lincRNA	9253,80	0
ENSG00000270641	TSIX	X	73792205	73829231	lincRNA	6409,10	0
ENSG00000229124	VIM-AS1	10	17214239	17229985	antisense	6248,93	0
ENSG00000214548	MEG3	14	100779410	100861031	lincRNA	5217,70	0
ENSG00000130600	H19	11	1995163	2001470	processed_transcript	4333,60	0
ENSG00000260032	LINC00657	20	36045622	36050960	lincRNA	3387,08	0
ENSG00000272540	ENSG00000272540	6	30723105	30723877	antisense	3200,00	0
ENSG00000261821	ENSG00000261821	15	74365435	74371211	antisense	2756,90	0
ENSG00000266962	ENSG00000266962	17	42552436	42554748	antisense	2612,70	0
ENSG00000223749	MIR503HG	X	134543337	134546632	lincRNA	2429,80	0
ENSG00000226549	SCDP1	17	20784645	20785725	processed_pseudogene	2114,10	0
ENSG00000213846	ENSG00000213846	3	27632976	27635427	transcribed_processed_pseudogene	2022,70	0
ENSG00000244953	ENSG00000244953	11	43943787	43947206	lincRNA	1982,30	0
ENSG00000214455	RCN1P2	13	45390353	45391267	processed_pseudogene	1977,10	0
ENSG00000227766	HCG4P5	6	29942075	29943067	unprocessed_pseudogene	1912,30	0
ENSG00000249835	VCAN-AS1	5	83531352	83581320	antisense	1847,28	0
ENSG00000227018	IL6STP1	17	15783288	15784307	processed_pseudogene	1786,40	0
ENSG00000254332	ENSG00000254332	8	69129598	69130345	processed_pseudogene	1772,50	0
ENSG00000262769	ENSG00000262769	17	19560111	19597922	antisense	1767,90	0
ENSG00000245067	IGFBP7-AS1	4	57109762	57205510	antisense	1755,30	0
ENSG00000254667	ENSG00000254667	11	123430737	123435710	transcribed_unprocessed_pseudogene	1689,40	0
ENSG00000256040	PAPPA-AS1	9	116398157	116400606	antisense	1641,45	0
ENSG00000234961	ENSG00000234961	10	17233325	17234833	antisense	1617,14	0
ENSG00000266283	ENSG00000266283	18	22200619	22205229	antisense	1541,40	0
ENSG00000248429	ENSG00000248429	4	158170752	158202877	antisense	1497,70	0
ENSG00000204949	FAM83A-AS1	8	123201172	123202743	antisense	1250,64	0
ENSG00000256013	ENSG00000256013	16	10529440	10532082	antisense	1104,40	0
ENSG00000225855	RUSC1-AS1	1	155316863	155324176	antisense	1076,98	0
ENSG00000214266	STARP1	13	65309855	65310953	processed_pseudogene	1056,60	0
ENSG00000259172	ENSG00000259172	15	101295419	101305737	antisense	1034,91	0
ENSG00000249035	ENSG00000249035	5	151676945	151724782	antisense	1007,30	0
ENSG00000267259	ENSG00000267259	17	28232590	28235281	lincRNA	1002,50	0
ENSG00000266010	GATA6-AS1	18	22166898	22168968	lincRNA	987,10	0
ENSG00000226243	RPL37AP1	20	44466564	44466842	processed_pseudogene	983,70	0,2161218
ENSG00000259884	ENSG00000259884	12	52058459	52059503	lincRNA	937,91	0
ENSG00000274177	ENSG00000274177	19	1010221	1010907	antisense	928,10	0
ENSG00000257453	ENSG00000257453	12	76030494	76031378	antisense	922,38	0
ENSG00000267469	ENSG00000267469	19	3052910	3053724	antisense	900,45	0
ENSG00000260290	ENSG00000260290	16	69756536	69757937	processed_pseudogene	844,46	0
ENSG00000238258	ENSG00000238258	10	33211277	33213804	antisense	812,30	0
ENSG00000259279	ENSG00000259279	15	39593580	39594231	antisense	792,10	0
ENSG00000232814	COL4A2-AS1	13	110502575	110508179	antisense	783,70	0
ENSG00000232320	ENSG00000232320	2	161340816	161341326	processed_pseudogene	774,64	0
ENSG00000127589	TUBBP1	8	30351873	30353518	transcribed_processed_pseudogene	768,00	0
ENSG00000233588	CYP51A1P2	13	28722386	28724021	processed_pseudogene	765,00	0
ENSG00000278621	ENSG00000278621	15	39588357	39588882	antisense	759,70	0
ENSG00000261026	ENSG00000261026	8	22679013	22684009	sense_overlapping	744,60	0
ENSG00000271581	ENSG00000271581	6	31356647	31357637	unprocessed_pseudogene	741,40	0
ENSG00000265168	ENSG00000265168	17	28573117	28574243	antisense	740,30	0
ENSG00000206417	H1FX-AS1	3	129315392	129326225	antisense	711,30	0

ENSG00000248187	ENSG00000248187	4	128567972	128570531	lincRNA	710,00	0
ENSG00000243701	DUBR	3	107240692	107326964	lincRNA	666,00	0
ENSG00000185275	CD24P4	X	18992467	18992709	processed_pseudogene	653,00	0
ENSG00000251562	MALAT1	11	65497762	65506516	lincRNA	651,73	0
ENSG00000258727	ENSG00000258727	14	23561097	23568073	antisense	634,62	0
ENSG00000251593	MSNP1	5	25909503	25911234	processed_pseudogene	617,50	0
ENSG00000236654	ENSG00000236654	7	23480753	23480959	processed_pseudogene	595,22	0
ENSG00000213684	LDHBP2	X	76334841	76335845	processed_pseudogene	583,30	0
ENSG00000262211	ENSG00000262211	5	55936143	55941727	antisense	577,97	0
ENSG00000213406	ANXA2P1	4	153307792	153308717	processed_pseudogene	575,50	0
ENSG00000133519	ZDHHC8P1	22	23390606	23402726	transcribed_unprocessed_pseudogene	575,20	0
ENSG00000180139	ACTA2-AS1	10	88932390	88940820	antisense	574,65	0
ENSG00000223949	ROR1-AS1	1	64094442	64171297	antisense	564,20	0
ENSG00000213763	ACTBP2	5	77784881	77786003	processed_pseudogene	561,75	0
ENSG00000259479	SORD2P	15	44825747	44884694	transcribed_unprocessed_pseudogene	555,80	0
ENSG00000232133	IMPDH1P10	2	201137516	201140027	processed_pseudogene	555,80	0
ENSG00000214297	ALDOAP2	10	125666875	125667950	processed_pseudogene	552,30	0
ENSG00000235847	LDHAP7	2	84777259	84778223	processed_pseudogene	536,09	0
ENSG00000255252	ENSG00000255252	11	32097143	32105091	antisense	535,00	0
ENSG00000259917	HNRNPLP2	15	34489002	34490571	unprocessed_pseudogene	511,92	0
ENSG00000272234	ENSG00000272234	5	42806394	42806997	antisense	508,80	0
ENSG00000259583	ENSG00000259583	15	100892859	100919283	antisense	505,50	0
ENSG00000204261	PSMB8-AS1	6	32844086	32846495	lincRNA	504,50	0
ENSG00000250508	ENSG00000250508	11	68870664	68874542	lincRNA	498,60	0
ENSG00000257378	ENSG00000257378	12	49954639	49956125	antisense	493,20	0
ENSG00000236480	PKMP1	1	114535995	114537840	processed_pseudogene	488,64	0
ENSG00000259352	ENSG00000259352	15	45073492	45074048	antisense	487,70	0
ENSG00000234645	YWHAEP5	2	138288029	138288793	processed_pseudogene	487,58	0
ENSG00000236136	ADORA2BP1	1	209744373	209745214	processed_pseudogene	482,40	0
ENSG00000244161	FLNB-AS1	3	58162547	58170636	antisense	476,88	0
ENSG00000232220	ENSG00000232220	19	53874626	53876049	antisense	473,79	0
ENSG00000260412	ENSG00000260412	9	27937617	27944497	sense_overlapping	447,59	0
ENSG00000259205	PRKXP1	15	100553529	100558954	processed_pseudogene	443,00	0
ENSG00000203858	HSD3BP2	1	119439001	119446086	unprocessed_pseudogene	440,20	0
ENSG00000263424	ENSG00000263424	18	67506589	67514030	antisense	438,30	0
ENSG00000233355	CHRM3-AS2	1	239703381	239730465	antisense	435,70	0
ENSG00000274415	ENSG00000274415	1	147757185	147758434	antisense	432,91	0
ENSG00000231313	CLIC1P1	12	120914400	120915123	processed_pseudogene	431,09	0
ENSG00000220785	MTMR9LP	1	32231658	32241620	transcribed_unprocessed_pseudogene	428,10	0
ENSG00000256364	ENSG00000256364	12	120697124	120699541	antisense	415,15	0
ENSG00000230149	ENSG00000230149	22	38734730	38738990	antisense	408,00	0
ENSG00000254635	WAC-AS1	10	28522652	28532743	antisense	404,80	0
ENSG00000225450	ENSG00000225450	22	38739003	38749041	antisense	404,00	0
ENSG00000241095	CYP51A1P1	3	82806515	82808021	processed_pseudogene	397,69	0
ENSG00000248445	SEMA6A-AS1	5	116447547	116508276	antisense	389,10	0
ENSG00000213942	ENSG00000213942	12	6409637	6410013	processed_pseudogene	387,64	0
ENSG00000232187	FTH1P7	13	22696023	22696574	processed_pseudogene	379,48	0
ENSG00000225462	FDPSP1	1	187563061	187564131	processed_pseudogene	377,60	0
ENSG00000272273	ENSG00000272273	6	30742929	30743592	antisense	375,06	0
ENSG00000206341	HLA-H	6	29887752	29890482	unprocessed_pseudogene	371,90	0
ENSG00000244151	ENSG00000244151	7	151074742	151076530	antisense	358,67	0
ENSG00000254545	ENSG00000254545	1	31789130	31791322	antisense	356,82	0
ENSG00000247095	MIR210HG	11	565660	568457	lincRNA	353,10	0
ENSG00000215861	ENSG00000215861	1	149197992	149321732	unprocessed_pseudogene	352,20	0

ENSG00000235910	APOA1-AS	11	116836117	116855729	antisense	351,57	0
ENSG00000224721	ENSG00000224721	14	75574888	75579588	antisense	347,80	0
ENSG00000271216	LINC01050	13	42810366	42812562	lincRNA	344,90	0
ENSG00000231625	ENSG00000231625	17	19615789	19633825	transcribed_unprocessed_pseudogene	343,70	0
ENSG00000213574	LDHAP5	10	118932674	118933295	processed_pseudogene	340,17	0
ENSG00000259049	ENSG00000259049	14	52775237	52777740	antisense	338,08	0
ENSG00000235191	NUCB1-AS1	19	48910930	48918891	antisense	335,00	0
ENSG00000256913	ENSG00000256913	12	6477714	6480207	processed_pseudogene	333,70	0
ENSG00000205885	C1RL-AS1	12	7108052	7122501	antisense	333,30	0
ENSG00000250859	HNRNPKP1	5	127511464	127512842	processed_pseudogene	325,80	0
ENSG00000260565	ENSG00000260565	16	2660349	2673444	processed_transcript	320,10	0
ENSG00000233111	RAB1C	9	37636688	37637293	processed_pseudogene	319,80	0
ENSG00000224063	ENSG00000224063	2	187003220	187554663	antisense	317,17	0
ENSG00000216285	ENSG00000216285	12	104030779	104031543	processed_pseudogene	311,29	0
ENSG00000255737	AGAP2-AS1	12	57726271	57728356	antisense	309,30	0
ENSG00000213222	ENSG00000213222	2	131723253	131724279	processed_pseudogene	308,20	0
ENSG00000261461	UBE2MP1	16	35169692	35170241	processed_pseudogene	302,64	0
ENSG00000272734	ADIRF-AS1	10	86965345	86971311	processed_transcript	300,80	0
ENSG00000268220	ENSG00000268220	3	170087810	170089590	antisense	297,90	0
ENSG00000271122	ENSG00000271122	7	35695214	35699413	antisense	297,50	0
ENSG00000253125	ENSG00000253125	8	22690150	22798616	processed_transcript	297,30	0
ENSG00000254416	ENSG00000254416	11	110355130	110406400	lincRNA	293,70	0
ENSG00000258663	ENSG00000258663	14	100834432	100861026	antisense	293,40	0
ENSG00000204054	LINC00963	9	129483451	129513683	processed_transcript	292,27	0
ENSG00000268189	ENSG00000268189	19	15379076	15381194	processed_transcript	291,33	0
ENSG00000215039	CD27-AS1	12	6439001	6451567	processed_transcript	287,40	0
ENSG00000224821	COL4A2-AS2	13	110456396	110463287	antisense	286,10	0
ENSG00000224596	ZMIZ1-AS1	10	78943328	79067895	antisense	284,60	0
ENSG00000268069	ENSG00000268069	12	47784923	47786002	antisense	283,30	0
ENSG00000230581	ACTG1P14	9	6834456	6835577	processed_pseudogene	283,30	0
ENSG00000255478	ENSG00000255478	11	65367438	65375299	antisense	281,50	0
ENSG00000230097	ME2P1	9	29824742	29826711	processed_pseudogene	281,10	0
ENSG00000256482	ENSG00000256482	12	24830421	24836558	antisense	280,80	0
ENSG00000235016	SEMA3F-AS1	3	50116022	50156085	antisense	280,09	0
ENSG00000214110	LDHAP4	9	14921337	14922334	processed_pseudogene	278,60	0
ENSG00000250746	ENSG00000250746	4	164188311	164190367	processed_pseudogene	278,30	0
ENSG00000267519	ENSG00000267519	19	13834516	13836289	lincRNA	276,60	0
ENSG00000270362	HMGN3-AS1	6	79233718	79236797	antisense	275,60	0
ENSG00000225511	LINC00475	9	92141298	92160114	lincRNA	274,20	0
ENSG00000233668	ENSG00000233668	9	34223984	34224628	processed_pseudogene	272,80	1,40E-02
ENSG00000259976	ENSG00000259976	3	114314501	114316179	lincRNA	271,70	0
ENSG00000255182	ENSG00000255182	8	144495458	144505444	processed_transcript	270,85	0
ENSG00000276550	HERC2P2	15	22495570	22590815	transcribed_unprocessed_pseudogene	270,18	0
ENSG00000248626	GAPDHP40	5	159950493	159951498	processed_pseudogene	269,98	0
ENSG00000274627	ENSG00000274627	16	89516797	89522217	sense_intronic	269,70	0
ENSG00000275678	ENSG00000275678	1	67121605	67123956	sense_intronic	269,70	0
ENSG00000228544	CCDC183-AS1	9	136803927	136808848	antisense	265,73	0
ENSG00000228275	ARMCX3-AS1	X	101622983	101624164	antisense	265,60	0
ENSG00000236570	RAD23BP1	3	18538673	18539899	processed_pseudogene	262,91	0
ENSG00000124224	PPP4R1L	20	58228940	58309439	transcribed_unprocessed_pseudogene	260,79	0
ENSG00000271857	ENSG00000271857	6	45421079	45422005	antisense	256,60	0
ENSG00000226415	TPI1P1	1	76699789	76700538	processed_pseudogene	254,74	0
ENSG00000261716	ENSG00000261716	1	149844498	149849024	sense_overlapping	251,00	0
ENSG00000228612	HK2P1	X	80571871	80574607	processed_pseudogene	249,40	0
ENSG00000260466	ENSG00000260466	16	87836532	87837663	antisense	249,20	0
ENSG00000243547	HNRNPKP4	3	96349554	96350939	processed_pseudogene	248,31	0
ENSG00000267282	ENSG00000267282	19	44882027	44890876	antisense	242,50	0
ENSG00000233110	ENSG00000233110	4	185587909	185594003	antisense	241,00	0

ENSG00000230508	RPL19P21	X	150993501	150994091	processed_pseudogene	240,00	7,06E-02
ENSG00000234420	ZNF37BP	10	42513510	42552822	transcribed_processed_pseudogene	238,44	0
ENSG00000234975	FTH1P2	1	228687415	228687826	processed_pseudogene	238,00	0
ENSG00000257653	ENSG00000257653	12	48766194	48767323	antisense	236,50	0
ENSG00000232774	ENSG00000232774	14	61570540	61658696	lincRNA	234,33	0
ENSG00000261549	ENSG00000261549	15	88989519	88990245	processed_pseudogene	233,70	0
ENSG00000273151	ENSG00000273151	7	879790	886547	antisense	233,56	0
ENSG00000260196	ENSG00000260196	11	17380649	17383531	antisense	233,20	0
ENSG00000205562	ENSG00000205562	14	85524432	85529988	lincRNA	231,90	0
ENSG00000256007	ARAP1-AS1	11	72685075	72693808	antisense	231,73	0
ENSG00000262533	ENSG00000262533	17	2042900	2043425	antisense	231,56	0
ENSG00000232499	ENSG00000232499	1	113449700	113450728	processed_pseudogene	230,60	0
ENSG00000223768	LINC00205	21	45293285	45297354	lincRNA	230,20	0
ENSG00000258745	ENSG00000258745	14	50912226	50913358	antisense	229,90	0
ENSG00000173295	FAM86B3P	8	8228595	8244865	transcribed_unprocessed_pseudogene	225,60	0
ENSG00000233033	CASK-AS1	X	41520036	41522336	antisense	219,80	0
ENSG00000215478	CES5AP1	22	23359603	23387731	transcribed_unprocessed_pseudogene	218,80	0
ENSG00000265069	ENSG00000265069	18	6954677	6957419	antisense	218,50	0
ENSG00000178146	ENSG00000178146	X	101778969	101780538	processed_pseudogene	218,22	0
ENSG00000257790	EIF4A1P4	12	53153272	53154484	processed_pseudogene	217,27	0,1392851
ENSG00000264112	ENSG00000264112	17	57989039	57994850	lincRNA	216,90	0
ENSG00000225420	ENSG00000225420	2	88538720	88575610	antisense	216,88	0
ENSG00000246627	CACNA1C-AS1	12	2676001	2691200	antisense	216,55	0
ENSG00000230521	HCG4P7	6	29887294	29888268	unprocessed_pseudogene	215,50	0
ENSG00000267395	ENSG00000267395	19	45767796	45772504	antisense	215,20	0
ENSG00000260852	FBXL19-AS1	16	30919319	30923269	antisense	215,10	0
ENSG00000261604	ENSG00000261604	5	43287601	43290839	antisense	214,46	0
ENSG00000263345	ENSG00000263345	17	2375061	2379306	antisense	212,70	0
ENSG00000248854	HNRNPH1P3	5	55838801	55840025	processed_pseudogene	212,50	0
ENSG00000230551	ENSG00000230551	5	149494314	149504670	processed_transcript	211,18	0
ENSG00000237417	XRCC6P1	10	93206857	93209627	processed_pseudogene	210,00	0
ENSG00000231298	LINC00704	10	4650185	4678154	lincRNA	206,90	0
ENSG00000274904	ENSG00000274904	16	30064306	30064825	antisense	206,14	0
ENSG00000228804	ENSG00000228804	3	187702313	187733849	antisense	205,82	0
ENSG00000259939	ENSG00000259939	16	8853312	8854347	antisense	205,63	0
ENSG00000254307	ENSG00000254307	8	94791643	94793106	antisense	203,23	0
ENSG00000238000	ENSG00000238000	5	98213402	98214121	processed_pseudogene	203,00	0
ENSG00000226312	CFLAR-AS1	2	201140278	201157823	antisense	203,00	0
ENSG00000245322	ENSG00000245322	4	99950006	100195099	antisense	202,27	0
ENSG00000281490	CICP14	7	128655962	128658791	processed_pseudogene	201,64	0
ENSG00000266066	POLRMTP1	17	62136972	62140639	processed_pseudogene	200,93	0
ENSG00000259006	ENSG00000259006	16	89919827	89922662	antisense	200,60	0
ENSG00000272114	ENSG00000272114	6	43770429	43770616	antisense	200,50	0
ENSG00000280780	JAKMIP2-AS1	5	147559994	147662009	antisense	199,30	0
ENSG00000227051	C14orf132	14	96039324	96094080	lincRNA	198,32	0
ENSG00000250742	ENSG00000250742	12	54126098	54142493	lincRNA	198,00	0
ENSG00000267633	ENSG00000267633	19	13772118	13774118	antisense	197,20	0
ENSG00000248208	ENSG00000248208	4	153658572	153659653	processed_pseudogene	197,00	0
ENSG00000228002	DHX9P1	13	76984868	76987943	processed_pseudogene	196,93	0
ENSG00000271984	ENSG00000271984	20	45892694	45893419	antisense	196,87	0
ENSG00000203804	ADAMTSL4-AS1	1	150560202	150574552	processed_transcript	196,07	0
ENSG00000257588	ENSG00000257588	12	49951512	49962924	antisense	195,70	0
ENSG00000258086	ENSG00000258086	12	54353792	54466985	antisense	194,40	0
ENSG00000254528	ENSG00000254528	11	117833719	117838942	antisense	193,70	0
ENSG00000240882	ENSG00000240882	3	120306726	120307952	processed_pseudogene	193,60	1,40E-02
ENSG00000257181	ENSG00000257181	12	68841288	68843237	antisense	193,35	0
ENSG00000259953	ENSG00000259953	9	112032555	112037730	sense_overlapping	193,09	0

ENSG00000236060	HSPB1P1	9	72007881	72008136	processed_pseudogene	192,84	0,4850164
ENSG00000273729	ENSG00000273729	14	77028086	77031572	antisense	192,50	0
ENSG00000259595	ENSG00000259595	15	44402685	44427144	antisense	192,19	0
ENSG00000248092	NNT-AS1	5	43571594	43603230	antisense	189,90	0
ENSG00000267512	ENSG00000267512	19	13139617	13141147	antisense	189,67	0
ENSG00000116883	ENSG00000116883	1	36323734	36329221	antisense	188,80	0
ENSG00000253645	ENSG00000253645	8	38970360	38973011	antisense	187,30	0
ENSG00000262112	ENSG00000262112	17	56888880	56891841	antisense	185,40	0
ENSG00000245156	ENSG00000245156	11	66269832	66278525	lincRNA	185,09	0
ENSG00000213383	ENSG00000213383	3	17871729	17872598	processed_pseudogene	184,55	0
ENSG00000213650	ENSG00000213650	7	56567906	56568858	processed_pseudogene	184,46	0
ENSG00000258012	ENSG00000258012	12	92858079	92859273	processed_pseudogene	183,83	0
ENSG00000226721	EEF1DP2	9	92836826	92837668	processed_pseudogene	183,00	0
ENSG00000237632	S100A11P1	7	103262000	103262311	processed_pseudogene	182,40	0
ENSG00000178248	ENSG00000178248	22	23462086	23486980	lincRNA	181,46	0
ENSG00000227543	SPAG5-AS1	17	28598790	28617377	processed_transcript	181,10	0
ENSG00000230807	ENSG00000230807	3	27486247	27486617	processed_pseudogene	181,10	0,2161218
ENSG00000265678	ENSG00000265678	17	82153430	82154815	antisense	180,10	0
ENSG00000232267	ACTR3P2	X	68771322	68772578	processed_pseudogene	179,20	0
ENSG00000262528	ENSG00000262528	16	654611	656194	antisense	177,60	0
ENSG00000224280	ENSG00000224280	7	16583782	16586757	processed_pseudogene	175,90	0
ENSG00000235852	ENSG00000235852	2	190880797	190882059	antisense	175,73	0
ENSG00000254211	LINC01485	5	173786790	173809039	lincRNA	175,20	0
ENSG00000275665	ENSG00000275665	17	38715328	38720272	antisense	174,90	0
ENSG00000233117	LINC00702	10	4201141	4243912	lincRNA	173,50	0
ENSG00000261578	ENSG00000261578	11	76800364	76804555	sense_overlapping	172,70	0
ENSG00000268191	ENSG00000268191	19	6494320	6495025	antisense	172,00	0
ENSG00000152117	ENSG00000152117	2	131492813	131521573	transcribed_processed_pseudogene	171,10	0
ENSG00000226137	BAIAP2-AS1	17	81029130	81034881	lincRNA	168,92	0
ENSG00000166917	MIR202HG	10	133246478	133247891	antisense	168,50	0
ENSG00000260304	ENSG00000260304	16	31182511	31183285	antisense	168,39	0
ENSG00000263218	ENSG00000263218	17	80999509	81000130	antisense	168,30	0
ENSG00000226564	FTH1P20	2	180872867	180873414	processed_pseudogene	167,43	0
ENSG00000218803	GSTM2P1	6	111046868	111047521	processed_pseudogene	167,40	0
ENSG00000259081	ENSG00000259081	14	76774284	76781518	antisense	167,35	0
ENSG00000216624	GAPDHP72	6	166064291	166065300	processed_pseudogene	167,22	0
ENSG00000261888	ENSG00000261888	17	83104255	83106910	lincRNA	166,30	0
ENSG00000255390	ENSG00000255390	11	6621451	6622322	antisense	165,77	0
ENSG00000273270	ENSG00000273270	7	128524016	128531069	lincRNA	165,62	0
ENSG00000224668	IPO8P1	1	210859177	210862285	processed_pseudogene	163,78	0
ENSG00000244491	ENSG00000244491	22	38734725	38738765	antisense	163,27	0
ENSG00000231466	ENSG00000231466	22	25349543	25350322	processed_pseudogene	162,80	0
ENSG00000271430	ENSG00000271430	X	74200229	74242148	sense_intronic	162,50	0
ENSG00000275131	ENSG00000275131	1	120489625	120579190	unprocessed_pseudogene	162,12	0
ENSG00000258501	EIF3LP1	14	81916231	81917888	processed_pseudogene	161,23	3,30E-03
ENSG00000227741	ENSG00000227741	1	160202199	160208869	antisense	161,09	0
ENSG00000232748	ENSG00000232748	16	31056460	31062803	lincRNA	160,46	0
ENSG00000276101	ENSG00000276101	17	81251194	81251803	antisense	159,00	0
ENSG00000245937	LINC01184	5	127940426	128083172	lincRNA	157,43	0
ENSG00000213073	ENSG00000213073	6	160093082	160096212	transcribed_processed_pseudogene	156,10	0
ENSG00000224645	ENSG00000224645	1	151340648	151341966	antisense	155,80	0
ENSG00000263142	LRRC37A17P	17	46978481	47054569	transcribed_unprocessed_pseudogene	155,60	0
ENSG00000251596	HADHAP1	4	165404067	165406350	processed_pseudogene	155,10	0
ENSG00000226806	ENSG00000226806	2	135820191	135823087	antisense	154,30	0
ENSG00000183458	ENSG00000183458	16	14911551	14935708	transcribed_unprocessed_pseudogene	154,10	0
ENSG00000226925	ENSG00000226925	2	102172621	102182108	antisense	153,90	0

ENSG00000235587	GAPDHP65	X	46439709	46440714	processed_pseudogene	153,53	0
ENSG00000186301	MST1P2	1	16645622	16650289	unprocessed_pseudogene	153,40	0
ENSG00000256448	ENSG00000256448	11	73405297	73410682	antisense	153,35	0
ENSG00000232229	LINC00865	10	89829510	89840861	lincRNA	153,30	0
ENSG00000234882	EIF3EP1	6	73291962	73293277	processed_pseudogene	152,23	0
ENSG00000277476	ENSG00000277476	17	68133201	68135935	lincRNA	151,40	0
ENSG00000227198	C6orf47-AS1	6	31658329	31660721	antisense	151,00	0
ENSG00000236901	MIR600HG	9	123109494	123115477	sense_intronic	150,89	0
ENSG00000233503	HNRNPLP1	6	7481094	7482594	processed_pseudogene	149,82	0
ENSG00000258469	CHMP4BP1	14	55298644	55299231	processed_pseudogene	149,10	0
ENSG00000270706	PRMT1P1	5	177265580	177266588	processed_pseudogene	148,00	0
ENSG00000229835	KHSRPP1	9	21695176	21696943	processed_pseudogene	147,28	0
ENSG00000215835	ENSG00000215835	1	166275629	166277597	processed_pseudogene	146,81	0
ENSG00000181741	FDX1P1	20	34475924	34476474	processed_pseudogene	146,40	0
ENSG00000256569	ENSG00000256569	12	120721507	120723639	antisense	145,69	0
ENSG00000268205	ENSG00000268205	19	57304305	57308562	lincRNA	145,65	0
ENSG00000237493	ENSG00000237493	12	55980432	55981035	processed_pseudogene	145,38	0
ENSG00000230364	RPL4P3	1	171683128	171684438	processed_pseudogene	145,00	0
ENSG00000260267	ENSG00000260267	16	31456711	31459736	antisense	144,90	0
ENSG00000254815	ENSG00000254815	11	557595	560107	antisense	144,72	0
ENSG00000236942	ENSG00000236942	1	205625483	205626153	processed_pseudogene	144,36	0
ENSG00000241627	UBQLN4P1	3	148985868	148987668	processed_pseudogene	144,28	0
ENSG00000277534	ENSG00000277534	18	26542971	26545791	sense_intronic	144,20	0
ENSG00000281207	SLFNL1-AS1	1	41014590	41043890	antisense	143,53	0
ENSG00000273419	ENSG00000273419	7	149858400	149862492	antisense	143,50	0
ENSG00000269439	ENSG00000269439	19	17488990	17511889	lincRNA	143,46	0
ENSG00000261614	ENSG00000261614	16	31568386	31569475	processed_pseudogene	143,35	0
ENSG00000278175	GLIDR	9	39807361	39810063	lincRNA	142,50	0
ENSG00000232623	ENSG00000232623	21	32306464	32308737	antisense	142,40	0
ENSG00000228983	SLC47A1P1	17	19579943	19596058	transcribed_unprocessed_pseudogene	142,20	0
ENSG00000249786	EAF1-AS1	3	15436171	15455940	antisense	142,13	0
ENSG00000248593	DSTNP2	12	6884682	6885786	transcribed_unprocessed_pseudogene	142,00	0
ENSG00000258384	ENSG00000258384	15	90952239	90955225	antisense	141,92	0
ENSG00000227942	FRMD8P1	X	65550898	65552421	processed_pseudogene	141,40	0
ENSG00000259211	ENSG00000259211	15	40464193	40466726	antisense	141,20	0
ENSG00000269987	ENSG00000269987	22	30976515	30978848	lincRNA	141,06	0
ENSG00000262791	ENSG00000262791	17	1725748	1738585	antisense	140,90	0
ENSG00000230584	CCT5P2	13	78908919	78910542	processed_pseudogene	139,08	0
ENSG00000231955	ENSG00000231955	2	206115547	206122323	antisense	138,90	0
ENSG00000268858	ENSG00000268858	20	63861212	63864293	antisense	138,15	0
ENSG00000267533	ENSG00000267533	18	12067173	12068417	processed_pseudogene	137,60	0
ENSG00000224975	INE1	X	47204921	47205865	sense_intronic	137,20	0
ENSG00000124549	BTN2A3P	6	26421391	26432383	transcribed_unprocessed_pseudogene	136,80	0
ENSG00000223599	ENSG00000223599	1	153852106	153853414	processed_pseudogene	136,43	0
ENSG00000278713	ENSG00000278713	16	29862760	29863417	antisense	135,90	0
ENSG00000275764	ENSG00000275764	12	27037100	27038960	lincRNA	135,70	0
ENSG00000235027	ENSG00000235027	11	1760348	1762486	antisense	134,53	0
ENSG00000225568	ENSG00000225568	1	87045875	87046700	processed_pseudogene	134,45	0
ENSG00000277449	CEBPB-AS1	20	50184598	50191498	antisense	133,82	0
ENSG00000256072	ENSG00000256072	12	66251745	66257434	antisense	133,70	0
ENSG00000260111	ENSG00000260111	16	70379457	70399502	antisense	133,40	0
ENSG00000254681	PKD1P5	16	18374521	18401940	transcribed_unprocessed_pseudogene	133,10	0
ENSG00000261641	ENSG00000261641	16	1445343	1446519	antisense	132,97	0
ENSG00000229056	ENSG00000229056	2	196260024	196264204	antisense	132,70	0
ENSG00000278133	ENSG00000278133	16	31122235	31124064	sense_intronic	132,40	0
ENSG00000229180	ENSG00000229180	7	66526088	66542624	lincRNA	132,30	0

ENSG00000280832	ST3GAL4-AS1	11	126340889	126355587	antisense	132,30	0
ENSG00000251095	ENSG00000251095	4	89551356	89726752	antisense	132,10	0
ENSG00000204429	ENSG00000204429	9	88180673	88181447	processed_pseudogene	132,00	0
ENSG00000281371	INE2	X	15785716	15787589	antisense	132,00	0
ENSG00000264538	SUZ12P1	17	30709299	30790908	transcribed_unprocessed_pseudogene	131,90	0
ENSG00000257607	ENSG00000257607	7	1055360	1059261	antisense	131,90	0
ENSG00000177822	ENSG00000177822	4	181874438	182145249	antisense	131,90	0
ENSG00000188681	TEKT4P2	21	9068361	9129752	transcribed_unprocessed_pseudogene	131,70	0
ENSG00000249207	ENSG00000249207	4	39112677	39126818	antisense	131,38	0
ENSG00000265096	C1QTNF1-AS1	17	79019209	79027655	antisense	131,20	0
ENSG00000240132	ETF1P2	7	151501878	151503463	processed_pseudogene	130,93	0
ENSG00000228986	ENSG00000228986	X	155292169	155293432	processed_pseudogene	130,54	0
ENSG00000237575	PYY2	17	28226563	28228065	transcribed_unprocessed_pseudogene	130,50	0
ENSG00000213003	BTF3P12	8	70273369	70273837	processed_pseudogene	130,10	0,2161218
ENSG00000261490	ENSG00000261490	4	10068089	10073019	sense_overlapping	130,05	0
ENSG00000259187	ENSG00000259187	15	44826540	44827094	lincRNA	130,00	0
ENSG00000227347	HNRNPKP2	2	136199114	136200503	processed_pseudogene	129,83	0
ENSG00000133624	ZNF767P	7	149547154	149624752	transcribed_unprocessed_pseudogene	129,43	0
ENSG00000206195	ENSG00000206195	22	15784959	15829984	processed_transcript	129,11	0
ENSG00000223722	ENSG00000223722	12	31754720	31755121	processed_pseudogene	129,10	0
ENSG00000255387	ENSG00000255387	11	4210354	4212091	processed_pseudogene	129,05	0
ENSG00000235512	TAB3-AS2	X	30854321	30854707	antisense	128,89	0
ENSG00000225536	STIP1P3	X	86084716	86086339	processed_pseudogene	128,78	0
ENSG00000205940	HSP90AB2P	4	13333414	13338657	transcribed_processed_pseudogene	128,43	0,4036702
ENSG00000218502	H2AFZP3	13	99215372	99215758	processed_pseudogene	128,33	0
ENSG00000225648	SBDSP1	7	72829425	72836701	transcribed_unprocessed_pseudogene	128,10	0
ENSG00000261457	ENSG00000261457	16	31802947	31807973	transcribed_processed_pseudogene	128,00	0
ENSG00000233459	ENSG00000233459	2	203634577	203636016	processed_pseudogene	127,20	0
ENSG00000225131	PSME2P2	13	48771128	48771827	processed_pseudogene	126,90	0
ENSG00000278772	LINC00548	13	40194509	40220502	lincRNA	126,70	0
ENSG00000187984	ANKRD19P	9	92809388	92888693	transcribed_unprocessed_pseudogene	126,29	0
ENSG00000219410	ENSG00000219410	12	6663260	6672069	antisense	126,12	0
ENSG00000269371	ENSG00000269371	19	7519916	7520460	antisense	126,00	0
ENSG00000257243	ENSG00000257243	12	49595148	49595688	processed_pseudogene	125,70	1,40E-02
ENSG00000260005	ENSG00000260005	17	81228707	81233983	antisense	125,47	0
ENSG00000253606	AFG3L2P1	8	43270198	43271960	processed_pseudogene	125,35	0
ENSG00000263232	ATP5A1P3	16	72005037	72006543	processed_pseudogene	124,97	0
ENSG00000232098	ENSG00000232098	19	58404238	58408484	lincRNA	124,90	0
ENSG00000214279	ENSG00000214279	10	133453928	133523558	unitary_pseudogene	123,69	0
ENSG00000254665	ENSG00000254665	11	8693357	8696607	antisense	123,53	0
ENSG00000218582	GAPDHP63	6	79953005	79954011	processed_pseudogene	123,30	0
ENSG00000273137	ENSG00000273137	22	50208461	50209542	antisense	123,20	0
ENSG00000257809	ENSG00000257809	12	56150796	56158220	antisense	122,47	0
ENSG00000230432	ENSG00000230432	2	219299002	219304130	antisense	121,80	0
ENSG00000265646	TUFMP1	17	27082690	27084036	processed_pseudogene	120,87	1,95E-02
ENSG00000245148	ARAP1-AS2	11	72700474	72705607	antisense	120,80	0
ENSG00000225674	IPO7P2	13	24122065	24123814	processed_pseudogene	120,68	0
ENSG00000251235	SNRPCP2	5	76376675	76377149	processed_pseudogene	120,50	0
ENSG00000269292	ENSG00000269292	19	46609277	46610779	antisense	120,49	0
ENSG00000256393	RPL41P5	12	93083598	93083675	processed_pseudogene	120,40	0
ENSG00000270015	ENSG00000270015	15	30926514	30928407	sense_intronic	120,20	0
ENSG00000267858	MZF1-AS1	19	58559129	58574797	antisense	119,86	0
ENSG00000262580	ENSG00000262580	17	80200673	80205949	antisense	119,61	0

ENSG00000269480	ENSG00000269480	19	17207138	17208010	antisense	119,31	0
ENSG00000228203	RNF144A-AS1	2	6912277	6918709	processed_transcript	118,90	0
ENSG00000225439	BOLA3-AS1	2	74148009	74150061	antisense	118,33	0
ENSG00000181260	MTHFD2P7	3	179464346	179465328	processed_pseudogene	117,90	0
ENSG00000229320	KRT8P12	3	160565447	160569248	transcribed_processed_pseudogene	117,80	0
ENSG00000254618	TMED10P1	8	144994886	144995540	processed_pseudogene	117,09	0
ENSG00000267122	ENSG00000267122	19	2212029	2215565	antisense	116,78	0
ENSG00000279010	ENSG00000279010	22	37948352	38002889	sense_overlapping	116,67	0
ENSG00000255680	ENSG00000255680	11	6618790	6619764	antisense	116,67	0
ENSG00000258021	ENSG00000258021	12	51900565	51901336	processed_pseudogene	116,30	0
ENSG00000213290	PGK1P2	19	12559571	12561105	processed_pseudogene	116,05	0
ENSG00000204959	ARHGEF34P	7	144272445	144286966	unprocessed_pseudogene	116,00	0
ENSG00000244171	PBX2P1	3	143176327	143177617	processed_pseudogene	115,87	0
ENSG00000241313	WWTR1-AS1	3	149657020	149661364	antisense	115,80	0
ENSG00000273038	ENSG00000273038	10	32887255	32889311	lincRNA	115,68	0
ENSG00000254859	ENSG00000254859	8	143541973	143549729	antisense	115,24	0
ENSG00000257151	PWAR6	15	25031873	25036490	lincRNA	114,79	0
ENSG00000228223	HCG11	6	26523450	26526579	lincRNA	114,60	0
ENSG00000248734	ENSG00000248734	5	96784777	96785999	antisense	114,50	0
ENSG00000224713	ENSG00000224713	12	57612118	57619638	antisense	114,30	0
ENSG00000099725	PRKY	X	7273972	7381548	transcribed_unprocessed_pseudogene	114,00	1,40E-02
ENSG00000233347	ERP29P1	20	52110344	52111049	processed_pseudogene	113,22	0
ENSG00000261799	ENSG00000261799	12	273954	277123	sense_overlapping	112,65	0
ENSG00000232656	IDI2-AS1	10	1022666	1044201	antisense	112,55	0
ENSG00000237438	CECR7	22	17036570	17060825	lincRNA	112,45	0
ENSG00000277801	ENSG00000277801	14	89156743	89157574	lincRNA	112,10	0
ENSG00000260766	ENSG00000260766	1	160024953	160026794	sense_overlapping	112,00	0
ENSG00000170919	TPT1-AS1	13	45341345	45393413	antisense	111,96	0
ENSG00000269976	ENSG00000269976	2	20586248	20586686	lincRNA	111,80	0
ENSG00000238057	ZEB2-AS1	2	144518097	144521477	antisense	111,50	0
ENSG00000188242	ENSG00000188242	5	466124	473098	antisense	111,48	0
ENSG00000230185	C9orf147	9	112433816	112487204	antisense	111,37	0
ENSG00000239791	ENSG00000239791	16	30572250	30583860	antisense	111,30	0
ENSG00000228492	RAB11FIP1P1	X	74202834	74204595	processed_pseudogene	111,10	0
ENSG00000219565	ZNF259P1	6	108782126	108787053	transcribed_processed_pseudogene	111,00	0
ENSG00000236496	GPS2P1	9	2875442	2876427	processed_pseudogene	110,73	0
ENSG00000261691	ENSG00000261691	16	547185	553847	antisense	110,57	0
ENSG00000258581	ENSG00000258581	14	100366628	100366920	processed_pseudogene	110,50	0,2161218
ENSG00000261559	FSCN1P1	15	34494420	34495900	processed_pseudogene	109,70	0
ENSG00000271646	ENSG00000271646	4	184474802	184477304	lincRNA	109,70	0
ENSG00000248180	GAPDHP60	4	87207092	87208086	processed_pseudogene	109,12	0
ENSG00000227827	ENSG00000227827	16	16356224	16377507	unprocessed_pseudogene	108,80	0
ENSG00000235371	ENSG00000235371	1	236110061	236112370	processed_pseudogene	108,45	0
ENSG00000270100	ENSG00000270100	2	20678254	20678932	lincRNA	108,40	0
ENSG00000268854	ENSG00000268854	19	50480119	50483351	antisense	107,30	0
ENSG00000265148	BZRAP1-AS1	17	58325450	58415766	antisense	107,17	0
ENSG00000251411	ENSG00000251411	4	86913266	86914817	processed_pseudogene	107,14	0
ENSG00000175730	BAK1P1	20	32690180	32690815	processed_pseudogene	107,10	0
ENSG00000273747	ENSG00000273747	15	83020115	83020802	antisense	107,10	0
ENSG00000257043	ENSG00000257043	11	18665050	18665548	processed_pseudogene	106,97	0
ENSG00000261822	ENSG00000261822	15	42567031	42569994	antisense	106,50	0
ENSG00000186676	EEF1GP1	7	125033453	125035301	processed_pseudogene	106,23	0
ENSG00000228232	GAPDHP1	X	39787132	39788136	processed_pseudogene	106,17	0
ENSG00000273066	ENSG00000273066	9	136799223	136810042	processed_transcript	106,07	0
ENSG00000268509	ENSG00000268509	3	5187172	5188298	antisense	106,00	0
ENSG00000260246	ENSG00000260246	1	109693117	109693742	antisense	106,00	0

ENSG00000257342	ENSG00000257342	12	57694132	57721510	antisense	105,91	0
ENSG00000269972	ENSG00000269972	22	30977516	30977858	lincRNA	105,85	0
ENSG00000243302	ENSG00000243302	7	128651185	128652334	processed_pseudogene	105,77	0
ENSG00000257764	ENSG00000257764	12	69353493	69354225	antisense	105,00	0
ENSG00000269487	ENSG00000269487	19	46728603	46732700	antisense	104,55	0
ENSG00000228218	ATF4P3	17	76225751	76226806	processed_pseudogene	104,44	0
ENSG00000260822	ENSG00000260822	X	24545516	24550466	sense_overlapping	104,43	0
ENSG00000267755	ENSG00000267755	19	1376773	1377520	antisense	104,26	0
ENSG00000223414	LINC00473	6	165908802	165988048	lincRNA	104,20	0
ENSG00000228739	ENSG00000228739	9	6047360	6066714	lincRNA	104,10	0
ENSG00000259144	RANBP20P	14	20720742	20723127	processed_pseudogene	103,90	0
ENSG00000228343	ENSG00000228343	X	48579774	48581157	antisense	103,40	0
ENSG00000214810	CYCSP55	6	34219439	34220066	processed_pseudogene	103,31	0
ENSG00000236276	NDP-AS1	X	43949732	43969620	antisense	103,30	0
ENSG00000232295	ENSG00000232295	6	71221457	71328228	processed_transcript	102,80	0
ENSG00000251017	ENSG00000251017	4	74085995	74086672	processed_pseudogene	102,50	0
ENSG00000274471	ENSG00000274471	15	23309607	23313276	unprocessed_pseudogene	102,20	0
ENSG00000225733	FGD5-AS1	3	14920347	14948424	antisense	102,07	0
ENSG00000261335	ENSG00000261335	17	76671942	76673658	antisense	101,70	0
ENSG00000230006	ANKRD36BP2	2	88765807	88806612	transcribed_unprocessed_pseudogene	101,15	0
ENSG00000277147	LINC00869	1	149606334	149679523	lincRNA	101,10	0
ENSG00000238193	ENSG00000238193	X	5305714	5307128	processed_pseudogene	101,08	0
ENSG00000229152	ANKRD10-IT1	13	110894639	110899172	sense_intronic	100,90	0
ENSG00000264769	ENSG00000264769	17	81922899	81924511	antisense	100,82	0
ENSG00000227973	PIN4P1	15	43875849	43876244	processed_pseudogene	100,73	0
ENSG00000261373	VPS9D1-AS1	16	89711856	89718165	antisense	100,49	0
ENSG00000245864	ENSG00000245864	5	88676218	88722831	antisense	100,20	0
ENSG00000203914	HSP90B3P	1	91642516	91644082	processed_pseudogene	99,98	0
ENSG00000261532	ENSG00000261532	16	2211997	2212863	lincRNA	99,60	0
ENSG00000267317	ENSG00000267317	19	1457670	1458580	antisense	99,50	0
ENSG00000231948	HS1BP3-IT1	2	20590775	20592548	sense_intronic	98,70	0
ENSG00000255310	ENSG00000255310	8	11107788	11109726	sense_intronic	98,67	0
ENSG00000259607	ENSG00000259607	8	29067279	29068454	antisense	98,55	0
ENSG00000257851	HNRNPA3P10	12	51712469	51713606	processed_pseudogene	98,55	0
ENSG00000241749	RPSAP52	12	65758020	65826997	transcribed_processed_pseudogene	98,50	0
ENSG00000237686	ENSG00000237686	6	43995723	44074652	antisense	98,40	0
ENSG00000249859	PVT1	8	127794533	128101253	lincRNA	97,80	0
ENSG00000223361	FTH1P10	5	17353695	17354624	transcribed_processed_pseudogene	97,67	0
ENSG00000231542	TAB3-AS1	X	30834623	30835300	antisense	97,40	0
ENSG00000228264	PSMD8P1	1	154414369	154415137	processed_pseudogene	97,10	0
ENSG00000237781	ENSG00000237781	1	150548562	150557724	antisense	96,80	0
ENSG00000266469	ENSG00000266469	17	39401793	39406233	antisense	96,32	0
ENSG00000248648	ENSG00000248648	5	133003119	133003365	processed_pseudogene	96,30	7,06E-02
ENSG00000269652	ENSG00000269652	19	41221426	41222051	antisense	96,10	0
ENSG00000253200	ENSG00000253200	8	22613908	22616657	antisense	96,01	0
ENSG00000229372	SZT2-AS1	1	43447776	43448644	antisense	96,00	0
ENSG00000218305	CDC14C	7	48919765	48926013	processed_pseudogene	95,80	0
ENSG00000205746	ENSG00000205746	16	18334400	18352476	transcribed_unprocessed_pseudogene	95,40	0
ENSG00000254721	ENSG00000254721	11	70206291	70207390	antisense	95,04	0
ENSG00000236509	RPL21P133	X	134607157	134607632	processed_pseudogene	94,40	0
ENSG00000225282	ENSG00000225282	22	23926900	23929574	processed_pseudogene	94,30	7,06E-02
ENSG00000254708	ENSG00000254708	11	34335118	34336003	processed_pseudogene	94,20	0
ENSG00000235674	LDHAP2	1	235738005	235738989	processed_pseudogene	93,96	0
ENSG00000250802	ZBED3-AS1	5	77086740	77166909	antisense	93,90	0
ENSG00000255949	ENSG00000255949	11	67431367	67435399	antisense	93,88	0

ENSG00000106610	STAG3L4	7	67302621	67321526	transcribed_unprocessed_pseudogene	93,86	0
ENSG00000264739	ENSG00000264739	17	16414524	16416689	antisense	93,80	0
ENSG00000233467	ENSG00000233467	X	148198014	148198878	processed_pseudogene	93,40	0
ENSG00000238132	CASC4P1	13	19563589	19564900	processed_pseudogene	93,36	0
ENSG00000279080	ENSG00000279080	22	38130216	38150612	antisense	93,20	0
ENSG00000260804	ENSG00000260804	2	216217045	216220192	lincRNA	93,08	0
ENSG00000246451	ENSG00000246451	14	103682362	103684015	antisense	93,00	0
ENSG00000255967	ENSG00000255967	12	8634367	8637608	processed_pseudogene	93,00	0
ENSG00000267034	ENSG00000267034	2	222317242	222318653	lincRNA	92,90	0
ENSG00000232593	KANTR	X	53094145	53167014	lincRNA	92,67	0
ENSG00000269044	ENSG00000269044	19	16633797	16635269	sense_intronic	92,40	0
ENSG00000235217	TSPY26P	20	32186477	32190527	transcribed_processed_pseudogene	92,20	0
ENSG00000233554	B4GALT1-AS1	9	33166975	33179983	antisense	91,90	0
ENSG00000219507	FTH1P8	X	148052233	148052746	processed_pseudogene	91,80	0
ENSG00000232951	IPO7P1	X	51921864	51922499	processed_pseudogene	91,80	0
ENSG00000265511	ENSG00000265511	17	17507351	17508308	antisense	91,20	0
ENSG00000243974	VTI1BP1	3	100225374	100226073	processed_pseudogene	91,20	0
ENSG00000271474	ENSG00000271474	4	95549129	95552457	antisense	90,80	0
ENSG00000263171	ENSG00000263171	17	7352687	7354944	antisense	90,73	0
ENSG00000224080	UBE2FP1	3	37143512	37143958	processed_pseudogene	90,30	0
ENSG00000260083	MIR762HG	16	30875222	30895220	antisense	90,23	0
ENSG00000244578	LINC01391	3	138935189	138944020	lincRNA	90,20	0
ENSG00000272335	ENSG00000272335	5	44826076	44828592	lincRNA	90,00	0
ENSG00000260404	ENSG00000260404	4	118591773	118633729	transcribed_unprocessed_pseudogene	90,00	0
ENSG00000244026	FAM86DP	3	75421552	75435110	transcribed_unprocessed_pseudogene	89,85	0
ENSG00000253540	FAM86HP	3	130099092	130111472	transcribed_unprocessed_pseudogene	89,60	0
ENSG00000235813	ENSG00000235813	X	134599457	134606254	lincRNA	89,40	0
ENSG00000258945	ENSG00000258945	14	85528599	85529386	lincRNA	89,20	0
ENSG00000186076	ENSG00000186076	12	93640822	93641586	processed_pseudogene	88,81	3,30E-03
ENSG00000248839	ENSG00000248839	3	98522570	98525334	antisense	88,80	0
ENSG00000278206	ENSG00000278206	6	143484979	143507327	processed_transcript	88,67	0
ENSG00000260941	LINC00622	1	119597702	119599271	sense_overlapping	88,60	0
ENSG00000259536	ENSG00000259536	15	40488041	40558019	antisense	88,41	0
ENSG00000255108	ENSG00000255108	11	823634	832883	antisense	88,11	0
ENSG00000235351	ENSG00000235351	2	241724615	241725693	antisense	87,80	0
ENSG00000235872	ENSG00000235872	12	67658991	67670919	antisense	87,75	0
ENSG00000261662	ENSG00000261662	1	119909255	119910613	sense_overlapping	87,70	0
ENSG00000197182	MIRLET7BHG	22	46053869	46113928	lincRNA	87,36	0
ENSG00000232878	DPYD-AS1	1	97095923	97322955	antisense	86,50	0
ENSG00000266642	ENSG00000266642	17	28897738	28899402	antisense	86,30	0
ENSG00000261295	ENSG00000261295	X	100673330	100673981	antisense	86,20	0
ENSG00000244560	ENSG00000244560	7	149285281	149297312	transcribed_unprocessed_pseudogene	86,00	0
ENSG00000250538	ENSG00000250538	4	155206529	155209027	antisense	86,00	0
ENSG00000244586	WNT5A-AS1	3	55487699	55488308	antisense	86,00	0
ENSG00000266993	ENSG00000266993	1	51793934	51799154	antisense	86,00	0
ENSG00000267352	SH3GL1P3	17	68134675	68135604	processed_pseudogene	85,90	0
ENSG00000247699	ENSG00000247699	5	160195744	160204826	antisense	85,80	0
ENSG00000236090	LDHAP3	2	41819747	41820754	processed_pseudogene	85,78	0
ENSG00000234292	ENSG00000234292	5	91280097	91281142	lincRNA	85,70	0
ENSG00000213753	CENPBD1P1	19	58573503	58599801	transcribed_processed_pseudogene	85,62	0
ENSG00000268575	ENSG00000268575	1	1702736	1737688	processed_transcript	85,60	0
ENSG00000267735	ENSG00000267735	19	12880969	12884088	antisense	85,36	0
ENSG00000235884	LINC00941	12	30795681	30802711	lincRNA	84,90	0
ENSG00000276855	ENSG00000276855	17	15789016	15789705	lincRNA	84,60	0

ENSG00000259945	ENSG00000259945	16	67542123	67542963	antisense	84,60	0
ENSG00000272558	ENSG00000272558	6	25983812	25999167	antisense	84,20	0
ENSG00000226235	LEMD1-AS1	1	205373252	205387440	antisense	84,20	0
ENSG00000232358	ENSG00000232358	20	50999370	51010019	antisense	84,10	0
ENSG00000261687	ENSG00000261687	15	43184079	43185141	antisense	83,80	0
ENSG00000269386	RAB11B-AS1	19	8374373	8390685	antisense	83,60	0
ENSG00000238164	ENSG00000238164	1	2549920	2557031	antisense	83,40	0
ENSG00000235609	ENSG00000235609	21	14818843	15014430	lincRNA	83,20	0
ENSG00000234203	ENSG00000234203	17	4972851	4974681	antisense	83,20	0
ENSG00000235448	LURAP1L-AS1	9	12700100	12814345	antisense	83,20	0
ENSG00000253981	ALG1L13P	8	8236003	8244667	transcribed_unprocessed_pseudogene	83,00	0
ENSG00000173727	ENSG00000173727	11	65455258	65466720	lincRNA	82,96	0
ENSG00000205790	DPP9-AS1	19	4679282	4685948	antisense	82,92	0
ENSG00000231013	ENSG00000231013	2	119476448	119487346	antisense	82,70	0
ENSG00000259096	FAM35CP	14	87952527	87955203	processed_pseudogene	82,60	0
ENSG00000254479	SLC25A1P1	11	85934737	85935663	processed_pseudogene	82,60	0,2161218
ENSG00000260276	ENSG00000260276	16	8848105	8860456	antisense	82,36	0
ENSG00000260266	ENSG00000260266	15	74350768	74364620	transcribed_processed_pseudogene	82,20	0
ENSG00000100058	CRYBB2P1	22	25448105	25520854	transcribed_unprocessed_pseudogene	82,06	0
ENSG00000230715	ENSG00000230715	7	128652841	128653243	processed_pseudogene	81,90	0
ENSG00000257616	ENSG00000257616	12	52730425	52732865	processed_pseudogene	81,83	0
ENSG00000246067	RAB30-AS1	11	83072066	83106719	lincRNA	81,83	0
ENSG00000228887	EEF1DP1	19	14070342	14071237	processed_pseudogene	81,65	0
ENSG00000254607	ENSG00000254607	11	126652852	126682104	antisense	81,60	0
ENSG00000182109	ENSG00000182109	1	39522280	39546187	antisense	81,50	0
ENSG00000261455	LINC01003	7	152463786	152465549	lincRNA	81,10	0
ENSG00000254285	KRT8P3	8	61578220	61579668	processed_pseudogene	80,91	0
ENSG00000236540	ENSG00000236540	22	20058030	20070569	antisense	80,70	0
ENSG00000249193	HSPD1P5	4	144845625	144847344	processed_pseudogene	80,70	0
ENSG00000233833	ETF1P3	X	65794345	65795835	processed_pseudogene	80,70	0
ENSG00000260894	ENSG00000260894	16	67542304	67542572	antisense	80,60	0
ENSG00000273036	FAM95C	9	38540567	38545372	lincRNA	80,60	0
ENSG00000269378	ITGB1P1	19	14621634	14622242	processed_pseudogene	80,36	0
ENSG00000280007	ENSG00000280007	22	18110759	18131154	antisense	80,30	0
ENSG00000245958	ENSG00000245958	4	119454791	119552025	transcribed_unprocessed_pseudogene	80,20	0
ENSG00000269968	ENSG00000269968	12	6537794	6538370	antisense	79,94	0
ENSG00000230918	ENSG00000230918	2	162073256	162075169	antisense	79,50	0
ENSG00000240809	ENSG00000240809	3	76434018	76435428	processed_pseudogene	79,31	0
ENSG00000249986	YWHAQP6	3	141600276	141600579	processed_pseudogene	79,30	0,2161218
ENSG00000215049	PRDX2P1	13	30296147	30296735	processed_pseudogene	79,18	2,95E-02
ENSG00000237264	FTH1P11	8	81521682	81522232	processed_pseudogene	79,10	0
ENSG00000215447	ENSG00000215447	21	45288052	45291738	processed_transcript	78,46	0
ENSG00000235298	ENSG00000235298	9	83972233	83975777	antisense	78,45	0
ENSG00000245571	ENSG00000245571	11	59130133	59143015	lincRNA	78,40	0
ENSG00000255394	C8orf49	8	11761256	11763223	lincRNA	78,40	0
ENSG00000242140	ENSG00000242140	3	181146233	181146463	processed_pseudogene	78,36	0
ENSG00000243679	ENSG00000243679	7	128653969	128654722	processed_pseudogene	78,21	0
ENSG00000236349	SUCLG2P2	12	94548241	94549536	processed_pseudogene	78,14	0
ENSG00000213569	ENSG00000213569	X	107131659	107132269	processed_pseudogene	78,05	0
ENSG00000261759	ENSG00000261759	16	19119976	19121629	antisense	78,00	0
ENSG00000237499	ENSG00000237499	6	137823673	137868233	antisense	78,00	0
ENSG00000254400	ENSG00000254400	11	6603642	6604420	antisense	77,96	0
ENSG00000250548	ENSG00000250548	14	61556313	61570653	lincRNA	77,80	0
ENSG00000206337	HCP5	6	31400702	31477506	sense_overlapping	77,80	0
ENSG00000213409	C1QBPP2	11	66761575	66762399	processed_pseudogene	77,60	0
ENSG00000258660	ENSG00000258660	14	73698103	73700351	antisense	77,27	0
ENSG00000232931	LINC00342	2	95807118	95816215	lincRNA	77,00	0

ENSG00000231700	ENSG00000231700	1	157709086	157709643	processed_pseudogene	76,80	0
ENSG00000229036	VDAC1P8	6	143490424	143506406	transcribed_processed_pseudogene	76,60	0
ENSG00000251615	ENSG00000251615	4	8355090	8358338	lincRNA	76,50	0
ENSG00000272030	ENSG00000272030	1	153631438	153634397	antisense	76,50	0
ENSG00000179611	DGKZP1	13	43968424	43971199	processed_pseudogene	76,43	0
ENSG00000275888	ENSG00000275888	17	82244770	82245591	antisense	76,38	0
ENSG00000260349	ENSG00000260349	16	9105834	9107174	antisense	76,33	0
ENSG00000269929	ENSG00000269929	9	94176458	94177892	lincRNA	76,30	0
ENSG00000236686	BZW1P1	3	172425850	172427089	processed_pseudogene	76,23	0
ENSG00000235912	ENSG00000235912	1	27649419	27649610	processed_pseudogene	76,23	0
ENSG00000254615	ENSG00000254615	8	106270144	106272899	lincRNA	76,09	0
ENSG00000198658	ABHD17AP1	1	148146395	148149566	unprocessed_pseudogene	75,70	0
ENSG00000268292	ENSG00000268292	22	20064552	20065705	antisense	75,67	0
ENSG00000203999	LINC01270	20	50292720	50314922	lincRNA	75,50	0
ENSG00000234629	WDR82P1	3	94937086	94938024	processed_pseudogene	75,33	0
ENSG00000226200	SGMS1-AS1	10	50624951	50641451	antisense	75,23	0
ENSG00000240477	ENSG00000240477	3	150050729	150051456	processed_pseudogene	75,17	0
ENSG00000259051	HNRNPUP1	14	43300263	43300595	processed_pseudogene	74,78	0
ENSG00000268636	ENSG00000268636	19	49625994	49626439	antisense	74,40	0
ENSG00000220494	YAP1P1	6	147406889	147408281	processed_pseudogene	74,20	1,40E-02
ENSG00000268555	ENSG00000268555	19	21570822	21587322	lincRNA	74,00	0
ENSG00000269886	ENSG00000269886	3	9812762	9813097	antisense	74,00	0
ENSG00000258920	FOXN3-AS1	14	89417354	89419793	antisense	73,70	0
ENSG00000272462	ENSG00000272462	6	25992662	26001775	lincRNA	73,70	0
ENSG00000217241	CBX3P9	6	116453014	116453565	processed_pseudogene	73,65	0
ENSG00000266208	ENSG00000266208	17	40360655	40364693	antisense	73,60	0
ENSG00000263219	RYKP1	17	4222091	4223828	processed_pseudogene	73,53	0
ENSG00000248930	ENSG00000248930	5	73020700	73021279	processed_pseudogene	73,46	0
ENSG00000215908	CROCCP2	1	16618969	16644683	transcribed_unprocessed_pseudogene	73,40	0
ENSG00000256940	ENSG00000256940	11	64245964	64248217	antisense	73,20	0
ENSG00000248866	USP46-AS1	4	52659406	52661668	lincRNA	73,20	0
ENSG00000275807	ENSG00000275807	16	28822431	28823969	antisense	73,15	0
ENSG00000272661	ENSG00000272661	7	151240399	151240972	antisense	73,10	0
ENSG00000260549	MT1L	16	56617476	56618818	unitary_pseudogene	73,00	0
ENSG00000258441	LINC00641	14	21200079	21206900	processed_transcript	72,86	0
ENSG00000267244	ENSG00000267244	19	1822089	1824542	processed_transcript	72,80	0
ENSG00000240854	ENSG00000240854	3	132175402	132176711	processed_pseudogene	72,79	0
ENSG00000277938	ENSG00000277938	20	25229150	25231933	lincRNA	72,71	0
ENSG00000277749	ENSG00000277749	15	74311516	74319688	antisense	72,60	1,40E-02
ENSG00000231340	ACTG1P10	X	53142832	53143913	processed_pseudogene	72,60	7,06E-02
ENSG00000255959	ENSG00000255959	11	60835996	60842965	antisense	72,57	0
ENSG00000230844	ZNF674-AS1	X	46545493	46548408	lincRNA	72,52	0
ENSG00000228318	ENSG00000228318	21	41441056	41445708	antisense	72,40	0
ENSG00000205485	ENSG00000205485	7	76549360	76627982	transcribed_unprocessed_pseudogene	72,31	0
ENSG00000226786	ENSG00000226786	6	19534944	19839080	antisense	72,30	0
ENSG00000183929	DUSP5P1	1	228650241	228651379	processed_pseudogene	72,00	0
ENSG00000188856	RPSAP47	8	80558870	80559757	processed_pseudogene	71,89	0
ENSG00000196696	ENSG00000196696	16	69976297	70065948	processed_transcript	71,60	0
ENSG00000262712	ENSG00000262712	16	4335870	4337818	sense_intronic	71,50	0
ENSG00000229273	ENSG00000229273	9	39809562	39810019	processed_pseudogene	71,40	0
ENSG00000234925	ATP5HP4	12	68642519	68642993	processed_pseudogene	71,14	0
ENSG00000253173	ENSG00000253173	8	70103798	70104179	processed_pseudogene	71,09	0
ENSG00000272341	ENSG00000272341	6	16764346	16766883	lincRNA	71,08	0
ENSG00000280852	ENSG00000280852	17	60101759	60102919	transcribed_processed_pseudogene	71,00	0
ENSG00000236680	ENSG00000236680	9	19026892	19028129	processed_pseudogene	71,00	0,1217903
ENSG00000271533	ENSG00000271533	X	74209976	74213660	sense_intronic	71,00	0

ENSG00000256167	ATF4P4	11	113789231	113791366	transcribed_processed_pseudogene	70,98	0
ENSG00000217950	ENSG00000217950	2	131442644	131444587	processed_pseudogene	70,89	0
ENSG00000213131	YWHAZP4	6	127355756	127356789	processed_pseudogene	70,68	0
ENSG00000230710	LINC00332	13	40181809	40189028	lincRNA	70,60	0
ENSG00000254023	PKMP4	8	75376709	75377014	processed_pseudogene	70,60	0
ENSG00000226752	PSMD5-AS1	9	120824828	120854385	antisense	70,59	0
ENSG00000213212	NCLP1	9	136812788	136815536	processed_pseudogene	70,55	3,30E-03
ENSG00000243613	ENSG00000243613	1	153746851	153751227	antisense	70,45	0
ENSG00000245149	RNF139-AS1	8	124462485	124474576	lincRNA	70,20	0
ENSG00000234793	ENSG00000234793	2	241754793	241755740	antisense	70,20	0
ENSG00000258301	ENSG00000258301	14	76781733	76786724	lincRNA	70,12	0
ENSG00000215769	ENSG00000215769	17	64749663	64781707	processed_transcript	70,10	0
ENSG00000272758	ENSG00000272758	3	122416207	122443180	antisense	70,00	0
ENSG00000229867	STEAP3-AS1	2	119244422	119249071	antisense	69,90	0
ENSG00000261534	ENSG00000261534	9	121815674	121819452	sense_overlapping	69,80	0
ENSG00000224908	TIMM8BP2	X	134166682	134166932	processed_pseudogene	69,80	0
ENSG00000218537	MIF-AS1	22	23894426	23898930	antisense	69,58	0
ENSG00000255443	ENSG00000255443	11	35210343	35214985	antisense	69,50	0
ENSG00000270670	ENSG00000270670	10	89837612	89839334	processed_pseudogene	69,50	0
ENSG00000253570	RNF5P1	8	38600661	38601200	processed_pseudogene	69,37	0
ENSG00000239223	RPL34P31	17	27274107	27274477	processed_pseudogene	69,36	0
ENSG00000253683	ENSG00000253683	5	172656522	172656713	processed_pseudogene	69,32	0
ENSG00000269834	ZNF528-AS1	19	52388842	52397766	processed_transcript	69,13	0
ENSG00000236565	HNRNPA3P5	13	65787932	65788764	processed_pseudogene	68,96	0
ENSG00000266933	ENSG00000266933	19	490046	507833	antisense	68,80	0
ENSG00000231468	PRDX3P2	1	28526318	28527227	processed_pseudogene	68,60	0,2161218
ENSG00000217130	ENSG00000217130	6	34744176	34744673	processed_pseudogene	68,53	0
ENSG00000262879	ENSG00000262879	17	46984045	47100323	processed_transcript	68,40	0
ENSG00000272669	ENSG00000272669	22	38742625	38743115	antisense	68,30	0
ENSG00000187481	HSD3BP1	1	119467221	119473803	unprocessed_pseudogene	68,20	0
ENSG00000241318	WDR82P2	1	91534666	91535593	processed_pseudogene	68,20	0
ENSG00000170161	ENSG00000170161	9	62897449	62900104	lincRNA	68,08	0
ENSG00000234661	CHL1-AS1	3	363370	385795	antisense	68,00	0
ENSG00000269837	IPO5P1	19	23255053	23257939	transcribed_processed_pseudogene	67,86	0
ENSG00000244479	OR2A1-AS1	7	144251264	144356181	antisense	67,40	0
ENSG00000260441	ENSG00000260441	16	68256162	68260443	antisense	67,30	0
ENSG00000228360	ENSG00000228360	7	139227537	139227828	processed_pseudogene	67,30	7,06E-02
ENSG00000227183	HDGFP1	X	131646639	131646890	processed_pseudogene	67,30	0
ENSG00000263050	ENSG00000263050	17	2043475	2044968	lincRNA	67,27	0
ENSG00000257354	ENSG00000257354	12	62602752	62622213	lincRNA	67,27	0
ENSG00000272941	ENSG00000272941	7	135168403	135169547	antisense	67,17	0
ENSG00000236526	ENSG00000236526	20	11909404	11918677	antisense	67,14	0
ENSG00000259540	ENSG00000259540	15	100558677	100559798	antisense	67,10	7,06E-02
ENSG00000186715	MST1L	1	16754910	16770237	transcribed_unprocessed_pseudogene	67,10	0
ENSG00000267397	ENSG00000267397	18	35716370	35717978	lincRNA	67,00	0
ENSG00000260934	ENSG00000260934	16	19501689	19502286	antisense	67,00	0
ENSG00000203387	ENSG00000203387	2	228352120	228353215	processed_pseudogene	67,00	0
ENSG00000274985	PTCHD3P1	10	29421476	29422012	unprocessed_pseudogene	66,92	0
ENSG00000277142	LINC00235	16	525155	527407	lincRNA	66,90	0
ENSG00000260742	ENSG00000260742	2	181887851	181891663	antisense	66,90	0
ENSG00000233695	GAS6-AS1	13	113815630	113845744	antisense	66,88	0
ENSG00000238273	ENSG00000238273	2	105363038	105378839	antisense	66,82	0
ENSG00000230262	MIRLET7DHG	9	94176602	94204568	lincRNA	66,80	0
ENSG00000272994	ENSG00000272994	2	105334027	105337475	lincRNA	66,70	0
ENSG00000254461	ENSG00000254461	11	66259567	66261834	antisense	66,40	0
ENSG00000204791	ENSG00000204791	8	144049129	144051522	antisense	66,40	0

ENSG00000247516	MIR4458HG	5	8450743	8463095	lincRNA	66,31	0
ENSG00000264475	ENSG00000264475	18	7076817	7080123	antisense	66,20	0
ENSG00000257086	ENSG00000257086	11	64246939	64249494	lincRNA	66,20	0
ENSG00000204049	ENSG00000204049	10	77866875	77869610	antisense	66,18	0
ENSG00000236054	ENSG00000236054	22	32583300	32584204	antisense	66,10	0
ENSG00000241684	ADAMTS9-AS2	3	64684909	65011468	antisense	65,92	0
ENSG00000232004	CAP1P2	10	43604843	43606251	processed_pseudogene	65,87	0
ENSG00000219891	ZSCAN12P1	6	28091154	28093664	transcribed_unprocessed_pseudogene	65,85	0
ENSG00000234585	CCT6P3	7	65038354	65074713	transcribed_unprocessed_pseudogene	65,80	0
ENSG00000234912	SNHG20	17	77086716	77094990	processed_transcript	65,50	0
ENSG00000259933	ENSG00000259933	16	2091436	2095433	sense_overlapping	65,50	0
ENSG00000217643	PTGES3P2	2	25822469	25822950	processed_pseudogene	65,50	1,40E-02
ENSG00000232727	YWHAEP1	7	64433830	64434592	processed_pseudogene	65,38	0
ENSG00000259516	ANP32AP1	15	35181799	35238197	transcribed_processed_pseudogene	65,37	0
ENSG00000242516	LINC00960	3	75672391	75679303	lincRNA	65,32	0
ENSG00000213480	ENSG00000213480	4	121369433	121370345	processed_pseudogene	65,27	0
ENSG00000229132	EIF4A1P10	X	92113246	92114461	processed_pseudogene	65,22	0
ENSG00000227825	SLC9A7P1	12	98453835	98457145	transcribed_processed_pseudogene	65,10	0
ENSG00000235251	ENSG00000235251	1	87044935	87045871	processed_pseudogene	65,10	1,40E-02
ENSG00000269069	ENSG00000269069	19	40023384	40025502	processed_pseudogene	65,09	0
ENSG00000247982	LINC00926	15	57300365	57307769	lincRNA	65,00	0
ENSG00000273247	ENSG00000273247	4	139411927	139454034	antisense	64,83	0
ENSG00000237975	FLG-AS1	1	152168125	152445456	antisense	64,75	0
ENSG00000247679	ENSG00000247679	5	177611253	177619754	antisense	64,67	0
ENSG00000225756	DBH-AS1	9	133654586	133657313	antisense	64,60	0
ENSG00000108785	HSD17B1P1	17	42546764	42548706	unprocessed_pseudogene	64,30	0
ENSG00000230140	ENSG00000230140	2	100972648	100977161	antisense	64,19	0
ENSG00000259408	ENSG00000259408	15	33851785	33856809	antisense	64,18	0
ENSG00000248546	ANP32C	4	164197007	164197711	processed_pseudogene	64,10	0
ENSG00000197927	C2orf27A	2	131722375	131767404	transcribed_unprocessed_pseudogene	64,05	0
ENSG00000216657	GLRX3P2	6	3978062	3979099	processed_pseudogene	63,96	0
ENSG00000265519	ENSG00000265519	17	15787787	15788205	lincRNA	63,80	0
ENSG00000224081	LINC01057	1	94613814	94855426	transcribed_processed_pseudogene	63,74	0
ENSG00000233441	CYP2AB1P	3	183895900	183910936	transcribed_unprocessed_pseudogene	63,70	0
ENSG00000262222	ENSG00000262222	16	10940719	10943021	antisense	63,60	0
ENSG00000249474	ENSG00000249474	3	101823793	101824998	antisense	63,60	0
ENSG00000223459	TCAF1P1	7	143598040	143604839	unprocessed_pseudogene	63,60	0
ENSG00000223401	ENSG00000223401	3	187743686	187745420	lincRNA	63,40	0
ENSG00000263072	ZNF213-AS1	16	3110460	3134882	antisense	63,35	0
ENSG00000229268	PES1P2	9	13986175	13987909	processed_pseudogene	63,18	0
ENSG00000223901	ENSG00000223901	21	46220269	46225364	antisense	63,10	0
ENSG00000249593	ENSG00000249593	5	139012647	139051203	antisense	62,94	0
ENSG00000228328	ENSG00000228328	X	68070520	68071767	processed_pseudogene	62,85	0
ENSG00000185485	SDHAP1	3	195959748	195990318	transcribed_unprocessed_pseudogene	62,24	0
ENSG00000258056	ENSG00000258056	12	55729104	55730852	antisense	62,20	0
ENSG00000235236	ENSG00000235236	3	48979918	48983985	antisense	62,15	0
ENSG00000258509	ENSG00000258509	14	44887434	44887741	processed_pseudogene	62,00	7,06E-02
ENSG00000223612	ENSG00000223612	1	145233001	145233519	processed_pseudogene	61,92	0
ENSG00000226067	LINC00623	1	120913275	121009291	lincRNA	61,90	0
ENSG00000234614	ENSG00000234614	1	151841877	151850385	antisense	61,90	0
ENSG00000255725	TDGP1	12	25803298	25804526	processed_pseudogene	61,90	0

ENSG00000230424	ENSG00000230424	1	19210501	19240704	antisense	61,87	0
ENSG00000260051	ENSG00000260051	16	1451760	1452653	antisense	61,80	0
ENSG00000236886	ENSG00000236886	2	216694464	216994079	antisense	61,78	0
ENSG00000184844	CYCSP45	X	153841251	153841565	processed_pseudogene	61,64	3,30E-03
ENSG00000255173	ENSG00000255173	11	65117157	65117458	antisense	61,60	0
ENSG00000196566	ENSG00000196566	10	87610163	87660003	antisense	61,50	0
ENSG00000248323	LUCAT1	5	91303029	91314402	lincRNA	61,40	0
ENSG00000272100	ENSG00000272100	1	52353487	52353877	antisense	61,12	0
ENSG00000213866	YBX1P10	9	35971344	35972318	processed_pseudogene	61,05	0
ENSG00000220378	KRT8P42	6	134296997	134298695	processed_pseudogene	60,90	0
ENSG00000279833	ENSG00000279833	22	39133090	39136760	antisense	60,77	0
ENSG00000237931	CLIC4P3	X	41076999	41077750	processed_pseudogene	60,70	0
ENSG00000259952	ENSG00000259952	16	29806496	29807732	antisense	60,66	0
ENSG00000263069	ENSG00000263069	17	80351828	80415168	antisense	60,63	0
ENSG00000228192	ENSG00000228192	1	42834681	42846422	antisense	60,62	0
ENSG00000260912	ENSG00000260912	9	19453209	19455173	sense_overlapping	60,59	0
ENSG00000266036	ENSG00000266036	17	74747319	74748912	antisense	60,50	0
ENSG00000259948	ENSG00000259948	15	89201091	89201768	processed_pseudogene	60,47	0
ENSG00000236456	ENSG00000236456	20	34245953	34246537	processed_pseudogene	60,36	0
ENSG00000237732	ENSG00000237732	2	222318275	222352989	transcribed_unprocessed_pseudogene	60,30	0
ENSG00000278212	MAFIP	X	11153858	11159013	unprocessed_pseudogene	60,30	0
ENSG00000225465	RFPL1S	22	29436534	29478175	antisense	60,25	0
ENSG00000259460	ENSG00000259460	15	37099339	37100173	antisense	60,20	0
ENSG00000274021	ENSG00000274021	12	89351015	89353271	lincRNA	59,96	0
ENSG00000250444	CCT5P1	5	115512077	115513694	processed_pseudogene	59,88	0
ENSG00000213774	ENSG00000213774	2	7324748	7325062	processed_pseudogene	59,80	0
ENSG00000268001	CARD8-AS1	19	48255675	48258199	antisense	59,73	0
ENSG00000263301	ENSG00000263301	17	7439159	7443327	antisense	59,70	0
ENSG00000256293	ENSG00000256293	12	64338178	64338797	processed_pseudogene	59,69	0
ENSG00000260917	ENSG00000260917	10	112823490	112827726	sense_overlapping	59,55	0
ENSG00000260367	ENSG00000260367	16	28973962	28978824	antisense	59,54	0
ENSG00000214820	MPRIPP1	3	44579938	44581026	processed_pseudogene	59,14	0
ENSG00000234684	SDCBP2-AS1	20	1325405	1378734	antisense	59,05	0
ENSG00000255921	ENSG00000255921	12	24949163	24960158	antisense	59,00	0
ENSG00000222032	ENSG00000222032	2	237428920	237434822	lincRNA	59,00	0
ENSG00000215146	ENSG00000215146	10	42331866	42367974	transcribed_unprocessed_pseudogene	58,80	0
ENSG00000271525	ARF4P1	9	134078904	134079444	processed_pseudogene	58,79	0
ENSG00000242479	ENSG00000242479	3	142450102	142452149	processed_pseudogene	58,78	0
ENSG00000268896	ENSG00000268896	2	219547211	219547658	antisense	58,70	0
ENSG00000231344	ENSG00000231344	1	27739091	27739439	processed_pseudogene	58,60	0,2161218
ENSG00000240731	ENSG00000240731	1	1317581	1318689	sense_intronic	58,55	0
ENSG00000251348	HSPD1P11	5	95768999	95770700	processed_pseudogene	58,30	0
ENSG00000232380	ZDHHC20P4	13	68985058	68986064	processed_pseudogene	58,20	0
ENSG00000224897	POT1-AS1	7	124929873	125179315	antisense	58,19	0
ENSG00000272993	ENSG00000272993	1	149845816	149846486	lincRNA	58,10	0
ENSG00000213236	YWHAZP2	2	126557435	126558162	processed_pseudogene	57,92	0
ENSG00000246250	ENSG00000246250	11	43829709	43880726	antisense	57,88	0
ENSG00000229932	YWHAZP3	10	23136924	23137661	processed_pseudogene	57,87	0
ENSG00000269680	ENSG00000269680	19	6748293	6751467	antisense	57,82	0
ENSG00000219329	ENSG00000219329	6	110923566	110924107	processed_pseudogene	57,80	7,06E-02
ENSG00000240602	AADACP1	3	151770428	151784894	transcribed_unprocessed_pseudogene	57,70	0
ENSG00000235501	ENSG00000235501	1	94927566	94963270	antisense	57,58	0
ENSG00000274272	ENSG00000274272	7	100572232	100578700	processed_transcript	57,30	0
ENSG00000218226	TATDN2P2	6	158609706	158621636	processed_pseudogene	57,30	0
ENSG00000257702	LBX2-AS1	2	74502595	74504678	antisense	57,30	0
ENSG00000226419	SLC16A1-AS1	1	112956415	113047055	antisense	57,22	0
ENSG00000260521	ENSG00000260521	15	94855474	94856914	processed_pseudogene	57,21	0

ENSG00000278600	ENSG00000278600	15	79920195	79922455	sense_intronic	57,20	0
ENSG00000232952	ENSG00000232952	1	105891739	105893517	processed_pseudogene	57,12	0
ENSG00000269825	ENSG00000269825	19	52650437	52653284	sense_intronic	57,08	0
ENSG00000223343	ENSG00000223343	3	48985049	48989988	antisense	57,07	0
ENSG00000274602	PI4KAP1	22	18533646	18577968	transcribed_unprocessed_pseudogene	57,06	0
ENSG00000255734	HNRNPABP1	12	10111738	10112471	processed_pseudogene	57,00	7,06E-02
ENSG00000272767	JMJD1C-AS1	10	63465229	63466563	antisense	57,00	0
ENSG00000182796	TMEM198B	12	55829745	55836246	unitary_pseudogene	56,89	0
ENSG00000229647	ENSG00000229647	2	207239650	207245887	lincRNA	56,65	0
ENSG00000247049	ENSG00000247049	5	181224646	181230685	antisense	56,41	0
ENSG00000182873	ENSG00000182873	1	2181794	2184389	antisense	56,30	0
ENSG00000230956	ENSG00000230956	X	88096393	88097225	processed_pseudogene	56,26	0
ENSG00000227934	ENSG00000227934	1	231021611	231022183	processed_pseudogene	56,17	0
ENSG00000253445	ENSG00000253445	5	172690454	172697720	antisense	56,13	0
ENSG00000254270	ERHP1	7	139534240	139534549	processed_pseudogene	56,07	0
ENSG00000258734	ENSG00000258734	14	58827679	58828176	processed_pseudogene	56,07	0
ENSG00000261324	ENSG00000261324	12	14762504	14767931	sense_overlapping	55,70	0
ENSG00000224057	EGFR-AS1	7	55179750	55188934	antisense	55,70	0
ENSG00000268530	ENSG00000268530	19	48465593	48469693	antisense	55,69	0
ENSG00000160766	GBAP1	1	155213821	155227422	transcribed_unprocessed_pseudogene	55,60	0
ENSG00000272768	ENSG00000272768	7	44884953	44886393	antisense	55,50	0
ENSG00000204622	HLA-J	6	30005971	30009956	transcribed_unprocessed_pseudogene	55,30	0
ENSG00000278831	ENSG00000278831	10	124623353	124624079	antisense	55,13	0
ENSG00000262877	ENSG00000262877	17	81388126	81390256	lincRNA	55,10	0
ENSG00000272078	ENSG00000272078	1	10639241	10654333	antisense	55,00	0
ENSG00000177337	DLGAP1-AS1	18	3593732	3598352	antisense	54,90	0
ENSG00000254964	ENSG00000254964	11	62606161	62606405	antisense	54,90	0
ENSG00000230079	STK24P1	X	136295690	136300298	processed_pseudogene	54,86	3,30E-03
ENSG00000203497	PDCD4-AS1	10	110869868	110872233	antisense	54,70	0
ENSG00000267769	ENSG00000267769	19	4454014	4455286	antisense	54,42	0
ENSG00000248774	ENSG00000248774	4	173322206	173329694	antisense	54,36	0
ENSG00000271147	ARMCX5-GPRASP2	X	102599512	102714671	processed_transcript	54,30	0
ENSG00000269148	ENSG00000269148	19	45830164	45831108	antisense	54,19	0
ENSG00000231329	ENSG00000231329	6	139144204	139239653	antisense	54,18	0
ENSG00000206149	HERC2P9	15	28589492	28685264	transcribed_unprocessed_pseudogene	54,13	0
ENSG00000229894	ENSG00000229894	4	165277984	165279645	processed_pseudogene	54,10	0
ENSG00000257576	ENSG00000257576	12	56511002	56512703	processed_pseudogene	54,09	0
ENSG00000267169	ENSG00000267169	19	14137179	14171267	antisense	54,04	0
ENSG00000131797	CLUHP3	16	31700590	31711986	transcribed_unprocessed_pseudogene	54,00	0
ENSG00000244926	ALKBH3-AS1	11	43909292	43920944	antisense	54,00	0
ENSG00000235651	ENSG00000235651	2	135510546	135511943	processed_pseudogene	53,94	0
ENSG00000225871	ENSG00000225871	1	148435105	148436926	processed_pseudogene	53,92	0
ENSG00000261396	ENSG00000261396	16	67538557	67540106	antisense	53,90	0
ENSG00000177738	ENSG00000177738	5	43014414	43067419	sense_overlapping	53,90	0
ENSG00000268282	ENSG00000268282	19	53004040	53005150	processed_pseudogene	53,55	0
ENSG00000183935	HTR7P1	12	13000420	13004830	transcribed_processed_pseudogene	53,40	0
ENSG00000226666	HSPA9P1	2	221961737	221963765	processed_pseudogene	53,34	0
ENSG00000269374	ENSG00000269374	19	42485076	42485714	processed_pseudogene	53,26	0
ENSG00000267387	ENSG00000267387	19	10089032	10090377	antisense	53,24	0
ENSG00000123870	ZNF137P	19	52588505	52597345	transcribed_unprocessed_pseudogene	53,20	0
ENSG00000236393	ENSG00000236393	X	40262917	40287720	lincRNA	53,20	0
ENSG00000198857	HSD3BP5	1	119601340	119609250	transcribed_unprocessed_pseudogene	53,10	0
ENSG00000260285	ENSG00000260285	14	103525010	103529072	antisense	53,00	0

ENSG00000280798	LINC00294	11	33076149	33079454	lincRNA	52,95	0
ENSG00000273679	ENSG00000273679	15	22371736	22373570	processed_pseudogene	52,86	0
ENSG00000232940	HCG25	6	33249534	33254989	antisense	52,81	0
ENSG00000179523	EIF3J-AS1	15	44527257	44536923	lincRNA	52,76	0
ENSG00000271980	ENSG00000271980	5	10264597	10267146	antisense	52,73	0
ENSG00000265254	ENSG00000265254	17	28405240	28406796	antisense	52,60	0
ENSG00000242349	NPPA-AS1	1	11841017	11848079	antisense	52,60	0
ENSG00000233098	CCDC144NL-AS1	17	20868433	21002276	antisense	52,54	0
ENSG00000236472	ENSG00000236472	17	50135586	50146176	antisense	52,50	0
ENSG00000214783	POLR2J4	7	43940895	44019175	processed_transcript	52,43	0
ENSG00000238123	MID1IP1-AS1	X	38801568	38803883	antisense	52,30	0
ENSG00000258646	ENSG00000258646	14	75004719	75008481	antisense	52,27	0
ENSG00000248223	ENSG00000248223	5	17353910	17354899	lincRNA	52,08	0
ENSG00000262454	MIR193BHG	16	14301389	14326744	lincRNA	52,00	0
ENSG00000223500	ENSG00000223500	7	135128444	135129685	processed_pseudogene	52,00	0
ENSG00000225603	ENSG00000225603	1	147050817	147052481	processed_pseudogene	51,93	0
ENSG00000222022	ENSG00000222022	2	237421420	237425276	lincRNA	51,91	0
ENSG00000253517	XRCC6P4	8	62855068	62857134	processed_pseudogene	51,88	0
ENSG00000267580	ENSG00000267580	19	33299934	33301168	antisense	51,70	0
ENSG00000261635	ENSG00000261635	15	36613474	36614198	processed_pseudogene	51,62	0
ENSG00000240370	ENSG00000240370	12	6873389	6884741	transcribed_processed_pseudogene	51,54	0
ENSG00000270189	ENSG00000270189	X	53169097	53170914	lincRNA	51,50	0
ENSG00000241170	ENSG00000241170	11	74876286	74920022	transcribed_processed_pseudogene	51,40	0
ENSG00000229897	SEPT7P7	9	98606833	98608133	processed_pseudogene	51,28	0
ENSG00000180867	PDIA3P1	1	147178113	147179622	processed_pseudogene	51,23	0
ENSG00000267312	ENSG00000267312	17	35459481	35460550	processed_pseudogene	51,14	0
ENSG00000248409	ENSG00000248409	22	23392771	23393839	unprocessed_pseudogene	51,10	0
ENSG00000234160	ENSG00000234160	9	37509150	37510299	antisense	51,10	0
ENSG00000272600	ENSG00000272600	22	20889206	20891214	antisense	51,06	0
ENSG00000259314	ENSG00000259314	15	90604225	90614558	antisense	50,91	0
ENSG00000267125	ENSG00000267125	19	1852382	1853622	antisense	50,91	0
ENSG00000255320	ENSG00000255320	11	66244840	66246239	antisense	50,80	0
ENSG00000239653	PSMD6-AS2	3	64004022	64012148	antisense	50,69	0
ENSG00000275516	ENSG00000275516	17	79952663	79952992	antisense	50,60	0
ENSG00000280623	PCAT14	22	23536881	23547797	lincRNA	50,50	0
ENSG00000267493	CIRBP-AS1	19	1267814	1270241	antisense	50,50	0
ENSG00000277688	ENSG00000277688	17	37609739	37613841	antisense	50,46	0
ENSG00000254343	ENSG00000254343	8	120052180	120056201	lincRNA	50,40	0
ENSG00000237861	ENSG00000237861	1	197222222	197223255	processed_pseudogene	50,40	0
ENSG00000215866	LINC01356	1	112820170	112850643	lincRNA	50,30	0
ENSG00000176700	SCAND2P	15	84631451	84647478	transcribed_unprocessed_pseudogene	50,26	0
ENSG00000263970	ENSG00000263970	18	8406761	8406953	antisense	50,23	0
ENSG00000267023	LRRC37A16P	17	68125777	68152468	transcribed_unprocessed_pseudogene	50,13	0
ENSG00000248923	MTND5P11	5	134924648	134926459	processed_pseudogene	50,08	0,0358681
ENSG00000203761	MSTO2P	1	155745829	155750137	unprocessed_pseudogene	49,92	0
ENSG00000224687	RASAL2-AS1	1	178091508	178093984	lincRNA	49,83	0
ENSG00000267615	ENSG00000267615	17	75943832	75945142	antisense	49,78	0
ENSG00000254340	ENSG00000254340	8	9141424	9145435	antisense	49,75	0
ENSG00000258741	ENSG00000258741	15	92715710	92734195	transcribed_processed_pseudogene	49,68	0
ENSG00000231588	ENSG00000231588	10	50629532	50631075	processed_pseudogene	49,62	0
ENSG00000270012	ENSG00000270012	X	49273054	49275768	lincRNA	49,50	0
ENSG00000225335	ENSG00000225335	22	18076527	18078884	antisense	49,38	0
ENSG00000267321	ENSG00000267321	17	35568120	35574792	lincRNA	49,30	0
ENSG00000232533	ENSG00000232533	7	143379692	143380495	antisense	49,21	0

ENSG00000215386	MIR99AHG	21	16070522	16627397	lincRNA	49,12	0
ENSG00000227262	HCG4B	6	29925983	29926973	unprocessed_pseudogene	49,10	0
ENSG00000260630	SNAI3-AS1	16	88663298	88687186	antisense	49,07	0
ENSG00000231831	MTHFD1P1	X	57392646	57395409	processed_pseudogene	49,05	0
ENSG00000176593	ENSG00000176593	19	58002061	58011232	processed_transcript	48,96	0
ENSG00000189223	PAX8-AS1	2	113211522	113276581	processed_transcript	48,95	0
ENSG00000234130	ENSG00000234130	X	93222220	93225015	processed_pseudogene	48,89	0
ENSG00000264044	ENSG00000264044	17	28633206	28635950	antisense	48,75	0
ENSG00000267594	CYP4F24P	19	15760241	15779909	unprocessed_pseudogene	48,70	1,40E-02
ENSG00000261189	ENSG00000261189	6	7540451	7541338	antisense	48,70	0
ENSG00000231160	KLF3-AS1	4	38612701	38664883	processed_transcript	48,70	0
ENSG00000260879	ENSG00000260879	1	108199926	108201491	antisense	48,70	0
ENSG00000267422	ENSG00000267422	19	37779686	37792865	transcribed_processed_pseudogene	48,67	0
ENSG00000235138	ENSG00000235138	9	134054290	134058805	antisense	48,60	0
ENSG00000237972	TUBG1P	7	43918697	43920054	processed_pseudogene	48,60	1,40E-02
ENSG00000230733	ENSG00000230733	7	5475804	5479811	lincRNA	48,60	0
ENSG00000233138	ENSG00000233138	6	142748443	142753759	antisense	48,50	0
ENSG00000224680	PLA2G12AP1	1	52368677	52369244	processed_pseudogene	48,46	0
ENSG00000183022	TPM3P8	2	230573167	230573809	processed_pseudogene	48,36	0
ENSG00000254680	ENSG00000254680	11	12261426	12263173	antisense	48,33	0
ENSG00000225099	ATP6V1E1P1	1	42903232	42903912	processed_pseudogene	48,31	0
ENSG00000270069	MIR222HG	X	45745211	45770274	lincRNA	48,10	0
ENSG00000228285	LYPLA2P1	6	33365548	33366243	processed_pseudogene	48,05	0
ENSG00000255050	ENSG00000255050	8	143573490	143577397	antisense	47,92	0
ENSG00000275964	ENSG00000275964	13	19863858	19865048	lincRNA	47,90	0
ENSG00000273295	ENSG00000273295	22	23901432	23907068	lincRNA	47,80	0
ENSG00000276952	ENSG00000276952	20	25284915	25285588	antisense	47,80	0
ENSG00000240787	ENSG00000240787	3	111570638	111571054	processed_pseudogene	47,80	0
ENSG00000231064	ENSG00000231064	1	155195004	155205495	antisense	47,76	0
ENSG00000245261	ENSG00000245261	6	43213801	43223860	antisense	47,75	0
ENSG00000268895	A1BG-AS1	19	58347751	58355183	antisense	47,65	0
ENSG00000226247	SUPT4H1P1	2	75651288	75651627	processed_pseudogene	47,64	0
ENSG00000232780	GAPDHP74	1	119434166	119435080	transcribed_processed_pseudogene	47,60	0
ENSG00000232630	PRPS1P2	9	125150653	125151589	processed_pseudogene	47,58	0
ENSG00000243055	GK-AS1	X	30699998	30724174	antisense	47,50	0
ENSG00000234537	ENSG00000234537	9	92132398	92138629	unprocessed_pseudogene	47,40	0
ENSG00000273230	ENSG00000273230	7	1464497	1467522	lincRNA	47,36	0
ENSG00000262585	ENSG00000262585	17	79915252	79926725	lincRNA	47,30	0
ENSG00000274653	ENSG00000274653	16	30359825	30360336	antisense	47,25	0
ENSG00000229880	IMMTP1	21	44675868	44678086	processed_pseudogene	47,11	7,06E-02
ENSG00000236467	KCNMA1-AS1	10	76888044	76980624	antisense	47,11	0
ENSG00000164845	FAM86FP	12	8232512	8242948	transcribed_unprocessed_pseudogene	47,10	0
ENSG00000248610	HSPA8P4	5	130140031	130141950	processed_pseudogene	47,10	0
ENSG00000227199	ST7-AS1	7	116952446	116954334	antisense	46,96	0
ENSG00000231025	ENSG00000231025	10	96992450	96995959	antisense	46,85	0
ENSG00000267691	SHC1P2	17	44228760	44230228	processed_pseudogene	46,80	7,06E-02
ENSG00000275719	ENSG00000275719	19	46787815	46789043	antisense	46,73	0
ENSG00000184441	ENSG00000184441	21	44331234	44335851	antisense	46,72	0
ENSG00000223764	ENSG00000223764	1	916865	921016	lincRNA	46,70	0
ENSG00000272711	ENSG00000272711	2	74832655	74833987	lincRNA	46,67	0
ENSG00000255389	ENSG00000255389	6	111599875	111602295	antisense	46,60	0
ENSG00000226872	ENSG00000226872	22	21031357	21043969	antisense	46,50	0
ENSG00000261326	LINC01355	1	23281309	23286752	lincRNA	46,40	0
ENSG00000233077	LINC01271	20	50310711	50321342	lincRNA	46,30	0
ENSG00000254762	ENSG00000254762	11	66267635	66268129	antisense	46,10	0

ENSG00000213613	ENSG00000213613	10	87945502	87946024	processed_pseudogene	46,10	0
ENSG00000235823	OLMALINC	10	100373615	100383368	lincRNA	45,90	0
ENSG00000259891	ENSG00000259891	8	140505813	140508043	antisense	45,90	0
ENSG00000263033	ENSG00000263033	16	11196177	11224969	lincRNA	45,60	0
ENSG00000230955	ENSG00000230955	1	37860697	37861580	antisense	45,54	0
ENSG00000231154	MORF4L2-AS1	X	103687284	103691772	antisense	45,54	0
ENSG00000244535	ENSG00000244535	11	32758268	32758614	processed_pseudogene	45,48	0
ENSG00000231686	ENSG00000231686	X	146619597	146620584	processed_pseudogene	45,41	0
ENSG00000272599	ENSG00000272599	10	73124573	73125532	antisense	45,40	0
ENSG00000259994	ENSG00000259994	10	30302826	30306066	sense_overlapping	45,30	0
ENSG00000229413	ENSG00000229413	7	128653690	128654019	processed_pseudogene	45,30	0
ENSG00000272077	ENSG00000272077	3	44667412	44669364	lincRNA	45,27	0
ENSG00000258634	ENSG00000258634	1	110058340	110062555	antisense	45,23	0
ENSG00000242125	SNHG3	1	28505980	28510892	sense_intronic	45,15	0
ENSG00000237286	ENSG00000237286	7	2944035	2947091	antisense	45,10	0
ENSG00000267439	ENSG00000267439	19	35747057	35753415	antisense	45,00	0
ENSG00000204685	STARD7-AS1	2	96208416	96242621	processed_transcript	44,98	0
ENSG00000182310	SPACA6P	19	51693340	51712387	processed_transcript	44,90	0
ENSG00000268471	ENSG00000268471	4	152536264	152539263	lincRNA	44,88	0
ENSG00000261061	ENSG00000261061	16	81030770	81031485	sense_intronic	44,80	0
ENSG00000235316	DUSP8P5	10	73731824	73733638	processed_pseudogene	44,80	0
ENSG00000224167	ENSG00000224167	1	112850028	112877871	lincRNA	44,80	0
ENSG00000228404	ENSG00000228404	21	46185079	46188941	antisense	44,77	0
ENSG00000255435	ENSG00000255435	11	118511911	118531094	antisense	44,73	0
ENSG00000213971	ENSG00000213971	19	23259906	23274251	processed_transcript	44,64	0
ENSG00000215347	SLC25A5P1	22	42001069	42001966	processed_pseudogene	44,60	7,06E-02
ENSG00000239415	ENSG00000239415	21	46251549	46254133	antisense	44,60	0
ENSG00000238184	CD81-AS1	11	2328749	2377992	antisense	44,59	0
ENSG00000238247	ENSG00000238247	X	27517884	27519759	processed_pseudogene	44,59	0
ENSG00000219102	HNRNPA3P12	1	53974969	53976031	processed_pseudogene	44,55	0
ENSG00000257202	ENSG00000257202	12	101923410	101924719	antisense	44,50	0
ENSG00000233230	ENSG00000233230	2	47905678	47907810	antisense	44,30	0
ENSG00000184068	ENSG00000184068	22	41831215	41834665	antisense	44,21	0
ENSG00000217624	ENSG00000217624	X	41675760	41676494	processed_pseudogene	44,15	0
ENSG00000162840	MT2P1	4	68376323	68376505	processed_pseudogene	44,14	2,21E-02
ENSG00000218980	FTH1P15	6	57004520	57004799	processed_pseudogene	44,10	0
ENSG00000239528	RPS14P8	5	116562562	116562930	processed_pseudogene	43,90	0,2161218
ENSG00000233251	ENSG00000233251	2	56173534	56185770	antisense	43,90	0
ENSG00000225377	NRSN2-AS1	20	320313	348224	antisense	43,73	0
ENSG00000224904	ENSG00000224904	1	11877770	11880406	processed_pseudogene	43,70	0
ENSG00000235703	LINC00894	X	149938628	150224580	antisense	43,67	0
ENSG00000234290	ENSG00000234290	5	132468890	132473043	antisense	43,50	0
ENSG00000174171	ENSG00000174171	15	41892793	41898575	antisense	43,46	0
ENSG00000267257	ENSG00000267257	18	58535415	58538552	antisense	43,45	0
ENSG00000248115	ENSG00000248115	4	52945649	52958087	antisense	43,40	0
ENSG00000272770	ENSG00000272770	17	2683305	2685088	antisense	43,39	0
ENSG00000267106	ZNF561-AS1	19	9621291	9645896	processed_transcript	43,33	0
ENSG00000236675	MTX1P1	1	155230975	155234325	unprocessed_pseudogene	43,30	0
ENSG00000272419	ENSG00000272419	1	145164099	145216058	processed_transcript	43,30	0
ENSG00000218350	LYPLA1P3	6	71165076	71165770	processed_pseudogene	43,24	0
ENSG00000267980	ENSG00000267980	19	4363789	4364640	antisense	43,23	0
ENSG00000259456	ADNP-AS1	20	50930984	50945134	antisense	43,20	0
ENSG00000204177	BMS1P1	10	46786674	46811989	transcribed_unprocessed_pseudogene	43,20	0
ENSG00000223403	MEG9	14	101069911	101072937	lincRNA	43,10	0
ENSG00000263624	ENSG00000263624	17	17167946	17185554	antisense	42,96	0
ENSG00000280927	CTBP1-AS	4	1210120	1218591	antisense	42,96	0
ENSG00000227473	TSSK5P	8	144141214	144143664	unitary_pseudogene	42,90	0
ENSG00000267283	ENSG00000267283	19	1989401	1990370	processed_transcript	42,80	0
ENSG00000277283	ENSG00000277283	12	120116907	120119000	antisense	42,80	0

ENSG00000272720	ENSG00000272720	22	38090127	38091559	lincRNA	42,70	0
ENSG00000269867	ENSG00000269867	19	57867038	57868172	sense_intronic	42,64	0
ENSG00000188013	MEIS3P2	17	20589293	20590367	processed_pseudogene	42,64	0
ENSG00000267801	ENSG00000267801	17	75876372	75879546	antisense	42,50	0
ENSG00000257732	ENSG00000257732	12	104262314	104280722	antisense	42,50	0
ENSG00000240449	ENSG00000240449	7	150363777	150372590	antisense	42,50	0
ENSG00000242294	STAG3L5P	7	100336079	100351900	transcribed_unprocessed_pseudogene	42,49	0
ENSG00000228409	CCT6P1	7	65751142	65763354	transcribed_unprocessed_pseudogene	42,47	0
ENSG00000266904	ENSG00000266904	19	19757366	19776423	lincRNA	42,40	0
ENSG00000250893	ENSG00000250893	4	40426119	40427585	antisense	42,40	0
ENSG00000214900	LINC01588	14	49927571	50092643	lincRNA	42,38	0
ENSG00000274259	ENSG00000274259	6	33437363	33454453	antisense	42,37	0
ENSG00000251571	DDX3P3	4	103572089	103574082	processed_pseudogene	42,35	0
ENSG00000233830	EIF4HP1	7	27458163	27458849	processed_pseudogene	42,31	0
ENSG00000234166	ARHGEF19-AS1	1	16197854	16198357	antisense	42,30	0
ENSG00000272742	ENSG00000272742	5	139364677	139369717	antisense	42,11	0
ENSG00000196421	LINC00176	20	64034344	64039962	lincRNA	42,10	0
ENSG00000197989	SNHG12	1	28578538	28582983	antisense	42,03	0
ENSG00000269194	ENSG00000269194	19	49808933	49809738	antisense	42,00	0
ENSG00000273389	ENSG00000273389	4	86000327	86000766	antisense	42,00	0
ENSG00000217060	ENSG00000217060	6	85286076	85290849	unprocessed_pseudogene	41,90	0
ENSG00000248415	GAPDHP61	15	64528667	64529671	processed_pseudogene	41,90	0,2161218
ENSG00000253607	ENSG00000253607	8	123002560	123030751	antisense	41,90	0
ENSG00000269688	ENSG00000269688	19	38844729	38845499	sense_intronic	41,80	0
ENSG00000261971	MMP25-AS1	16	3051096	3059370	antisense	41,79	0
ENSG00000263585	ENSG00000263585	17	81932398	81933058	antisense	41,79	0
ENSG00000227678	ENSG00000227678	6	130133410	130146179	antisense	41,77	0
ENSG00000224597	SVIL-AS1	10	29409402	29487745	antisense	41,76	0
ENSG00000213842	SUGT1P2	3	32752910	32753901	processed_pseudogene	41,70	7,06E-02
ENSG00000223427	ENSG00000223427	2	80162428	80163135	processed_pseudogene	41,62	0
ENSG00000185596	WASH3P	15	101961603	101976543	transcribed_unprocessed_pseudogene	41,59	0
ENSG00000223745	ENSG00000223745	1	93262186	93346025	processed_transcript	41,51	0
ENSG00000225345	SNX18P3	9	38566260	38568211	transcribed_unprocessed_pseudogene	41,50	0
ENSG00000270983	ENSG00000270983	6	142062717	142063053	processed_pseudogene	41,46	0
ENSG00000231205	ZNF826P	19	20340269	20424969	transcribed_unprocessed_pseudogene	41,42	0
ENSG00000235246	ENSG00000235246	22	38231320	38232248	antisense	41,36	0
ENSG00000281183	NPTN-IT1	15	73567012	73569294	sense_intronic	41,33	0
ENSG00000223825	DAZAP2P1	2	202201384	202201886	processed_pseudogene	41,29	0
ENSG00000267475	ENSG00000267475	19	32687089	32691750	lincRNA	41,28	0
ENSG00000232959	ENSG00000232959	1	170024077	170024683	antisense	41,20	0
ENSG00000205763	RP9P	7	32916815	32943176	transcribed_unprocessed_pseudogene	41,14	0
ENSG00000224407	ENSG00000224407	1	230280312	230281893	antisense	41,13	0
ENSG00000268030	ENSG00000268030	19	18557775	18561560	antisense	41,00	0
ENSG00000253352	TUG1	22	30970677	30979395	antisense	40,97	0
ENSG00000236319	ENSG00000236319	17	55526964	55561577	transcribed_processed_pseudogene	40,92	0
ENSG00000260563	ENSG00000260563	17	82293716	82294910	lincRNA	40,90	0
ENSG00000261240	ENSG00000261240	16	2112335	2113342	antisense	40,90	0
ENSG00000260219	ENSG00000260219	16	30355441	30357104	lincRNA	40,87	0
ENSG00000218416	ENSG00000218416	2	240449315	240456714	processed_transcript	40,80	0
ENSG00000278730	ENSG00000278730	17	68126666	68129586	lincRNA	40,72	0
ENSG00000269896	ENSG00000269896	1	2350414	2352820	transcribed_processed_pseudogene	40,70	0
ENSG00000272654	ENSG00000272654	1	153977743	153979160	lincRNA	40,70	0

ENSG00000227671	ENSG00000227671	1	247189851	247210856	transcribed_unprocessed_pseudogene	40,65	0
ENSG00000230067	HSPD1P6	3	36767117	36784167	transcribed_processed_pseudogene	40,64	0
ENSG00000271198	VDAC3P1	14	99964529	99965378	processed_pseudogene	40,62	0,2746192
ENSG00000259321	ENSG00000259321	14	24139445	24140444	lincRNA	40,60	0
ENSG00000262049	ENSG00000262049	17	81701324	81703300	antisense	40,57	0
ENSG00000263388	ENSG00000263388	17	10680734	10683988	antisense	40,50	0
ENSG00000275741	ENSG00000275741	13	109785831	109808252	antisense	40,50	0
ENSG00000248890	HHIP-AS1	4	144642922	144661357	antisense	40,50	0
ENSG00000272455	ENSG00000272455	1	1409096	1410618	lincRNA	40,50	0
ENSG00000230626	ENSG00000230626	7	129126518	129130793	transcribed_processed_pseudogene	40,44	0
ENSG00000099251	HSD17B7P2	10	38356380	38378505	transcribed_unprocessed_pseudogene	40,40	0
ENSG00000280594	ENSG00000280594	21	17611744	17633199	processed_transcript	40,40	0
ENSG00000250031	ENSG00000250031	8	58424588	58426685	processed_pseudogene	40,40	0
ENSG00000270210	ENSG00000270210	2	28425945	28426719	lincRNA	40,40	0
ENSG00000250539	KRT8P33	5	123400922	123402344	processed_pseudogene	40,36	0
ENSG00000242261	ENSG00000242261	7	128306649	128307678	processed_pseudogene	40,27	0
ENSG00000204620	ENSG00000204620	X	48568014	48574860	antisense	40,13	0
ENSG00000261087	ENSG00000261087	8	101166805	101169629	lincRNA	40,10	0
ENSG00000215692	ENSG00000215692	2	241734715	241735498	antisense	40,10	0
ENSG00000233871	DLG5-AS1	10	77927372	77929824	processed_transcript	40,00	0
ENSG00000270082	ENSG00000270082	16	87326987	87327584	antisense	39,90	0
ENSG00000230373	GOLGA6L5P	15	84507885	84516814	transcribed_unprocessed_pseudogene	39,90	0
ENSG00000146556	WASH2P	2	113588550	113599043	transcribed_unprocessed_pseudogene	39,82	0
ENSG00000226986	ENSG00000226986	1	211207239	211207897	processed_pseudogene	39,82	0
ENSG00000269176	ENSG00000269176	11	62786023	62786785	antisense	39,77	0
ENSG00000269926	ENSG00000269926	10	72274915	72275980	antisense	39,76	0
ENSG00000256799	ENSG00000256799	12	4635349	4636140	processed_pseudogene	39,70	0
ENSG00000261546	ENSG00000261546	16	89113175	89115279	antisense	39,69	0
ENSG00000224870	ENSG00000224870	1	1399522	1402046	processed_transcript	39,64	0
ENSG00000214176	PLEKHM1P	17	64779259	64837154	transcribed_unprocessed_pseudogene	39,62	0
ENSG00000197083	ZNF300P1	5	150930645	150946289	transcribed_unprocessed_pseudogene	39,58	0
ENSG00000232742	RHOQP2	2	131460999	131461577	processed_pseudogene	39,58	0
ENSG00000203709	C1orf132	1	207801518	207869150	lincRNA	39,48	0
ENSG00000180229	HERC2P3	15	20379495	20506180	transcribed_unprocessed_pseudogene	39,28	0
ENSG00000258559	ENSG00000258559	14	74289127	74294425	sense_overlapping	39,25	0
ENSG00000254614	ENSG00000254614	11	65177606	65181834	antisense	39,20	0
ENSG00000272655	ENSG00000272655	7	44013562	44019170	transcribed_unprocessed_pseudogene	39,10	0
ENSG00000274818	ENSG00000274818	14	70906657	70907111	lincRNA	39,04	0
ENSG00000230532	ENSG00000230532	17	48931791	48937100	antisense	39,02	0
ENSG00000226009	KCNIP2-AS1	10	101819078	101828779	antisense	39,00	0
ENSG00000265287	ENSG00000265287	17	28607963	28609730	antisense	38,90	0
ENSG00000225864	HCG4P11	6	29722981	29723971	unprocessed_pseudogene	38,90	0
ENSG00000214194	LINC00998	7	113116718	113118613	lincRNA	38,82	0
ENSG00000274020	LINC01138	1	148362995	148460150	lincRNA	38,80	0
ENSG00000235554	ENSG00000235554	17	16616848	16617881	processed_pseudogene	38,77	0
ENSG00000228748	ENSG00000228748	10	77782866	77793176	antisense	38,73	0
ENSG00000270574	ENSG00000270574	2	178578790	178580906	antisense	38,73	0
ENSG00000276728	ENSG00000276728	17	45146730	45148470	lincRNA	38,70	0
ENSG00000234405	ENSG00000234405	X	103497523	103500317	antisense	38,67	0
ENSG00000261790	ENSG00000261790	16	1984877	1990357	lincRNA	38,64	0

ENSG00000213513	ENSG00000213513	10	77780337	77781876	processed_pseudogene	38,60	0
ENSG00000248802	ENSG00000248802	4	128582999	128601407	lincRNA	38,60	0
ENSG00000236528	ENSG00000236528	1	25859613	25863420	antisense	38,59	0
ENSG00000261659	ENSG00000261659	16	689001	692554	processed_transcript	38,55	0
ENSG00000257800	FNBP1P1	2	74120680	74123218	processed_pseudogene	38,55	0
ENSG00000270333	ENSG00000270333	10	109661814	109662050	processed_pseudogene	38,50	0
ENSG00000213197	ENSG00000213197	2	152389937	152390630	processed_pseudogene	38,50	0
ENSG00000213963	ENSG00000213963	2	177283508	177392691	sense_overlapping	38,50	0
ENSG00000267315	ENSG00000267315	17	35377416	35377678	processed_pseudogene	38,43	0
ENSG00000272909	ENSG00000272909	14	64440369	64442238	antisense	38,43	0
ENSG00000238113	LINC01410	9	62801461	62813486	lincRNA	38,40	0
ENSG00000228838	ENSG00000228838	1	53288024	53289706	antisense	38,40	0
ENSG00000256249	ENSG00000256249	12	122687125	122715979	lincRNA	38,20	0
ENSG00000226081	USP12PX	X	90112650	90113706	processed_pseudogene	38,18	0
ENSG00000187952	HS6ST1P1	1	21428303	21429536	processed_pseudogene	38,14	0
ENSG00000277089	ENSG00000277089	17	36072866	36090134	antisense	38,10	0
ENSG00000272604	ENSG00000272604	7	105571083	105573660	antisense	38,10	0
ENSG00000259125	LRP1-AS	12	57144620	57147619	antisense	38,00	0
ENSG00000254230	ENSG00000254230	8	22565997	22567171	antisense	38,00	0
ENSG00000261167	ENSG00000261167	3	131455126	131458598	sense_overlapping	38,00	0
ENSG00000231970	ENSG00000231970	10	97401115	97419524	antisense	37,93	0
ENSG00000180747	SMG1P3	16	21446683	21520444	transcribed_unprocessed_pseudogene	37,91	0
ENSG00000237188	ENSG00000237188	1	147172771	147211568	antisense	37,91	0
ENSG00000237432	RPS7P12	X	134650830	134651411	processed_pseudogene	37,90	0
ENSG00000227544	ENSG00000227544	7	35715034	35734887	lincRNA	37,83	0
ENSG00000182165	TP53TG1	7	87325225	87345515	lincRNA	37,80	0
ENSG00000238084	ENSG00000238084	1	25398721	25399198	processed_pseudogene	37,73	0
ENSG00000236829	ENSG00000236829	16	382097	392960	transcribed_processed_pseudogene	37,70	0
ENSG00000269940	ENSG00000269940	14	103694560	103695170	sense_intronic	37,61	0
ENSG00000225269	LINC00705	10	4655427	4662419	lincRNA	37,60	0
ENSG00000272899	ENSG00000272899	7	128866579	128872044	lincRNA	37,60	0
ENSG00000229659	ENSG00000229659	10	73422259	73422696	processed_pseudogene	37,57	0,2161218
ENSG00000272056	ENSG00000272056	2	27053618	27054276	antisense	37,55	0
ENSG00000227115	ENSG00000227115	18	51346249	51643939	lincRNA	37,51	0
ENSG00000235546	ENSG00000235546	17	20612667	20614008	processed_pseudogene	37,50	0
ENSG00000260174	ENSG00000260174	15	30044455	30045296	processed_pseudogene	37,50	0
ENSG00000267049	ENSG00000267049	19	35769144	35771028	antisense	37,40	0
ENSG00000260317	ENSG00000260317	8	80541300	80543104	lincRNA	37,40	0
ENSG00000240616	RPS6P25	19	12894133	12894880	processed_pseudogene	37,39	0
ENSG00000259706	HSP90B2P	15	99257632	99260015	processed_pseudogene	37,37	0
ENSG00000268309	ENSG00000268309	19	16551773	16552328	antisense	37,36	0
ENSG00000226698	ENSG00000226698	1	26876133	26878245	antisense	37,30	0
ENSG00000276141	WHAMMP3	15	22664359	22686213	transcribed_unprocessed_pseudogene	37,27	0
ENSG00000271538	ENSG00000271538	4	184506460	184537554	lincRNA	37,22	0
ENSG00000272149	ENSG00000272149	3	33144104	33147721	antisense	37,20	0
ENSG00000254459	ENSG00000254459	11	77829654	77872262	antisense	37,11	0
ENSG00000227492	ENSG00000227492	10	100229667	100234000	antisense	37,08	0
ENSG00000234764	E2F6P1	22	18833694	18834438	processed_pseudogene	37,00	0
ENSG00000249249	ENSG00000249249	5	115602057	115620659	antisense	36,95	0
ENSG00000225507	ENSG00000225507	7	47956793	47957318	processed_pseudogene	36,90	0
ENSG00000180764	PIPSL	10	93958191	93961540	transcribed_processed_pseudogene	36,87	0
ENSG00000268812	ENSG00000268812	22	30246205	30246998	antisense	36,70	0
ENSG00000239345	HNRNPA1P26	X	100855288	100856379	processed_pseudogene	36,70	0
ENSG00000257605	ENSG00000257605	12	53298655	53300314	antisense	36,68	0
ENSG00000232160	RAP2C-AS1	X	132217147	132432862	antisense	36,63	0
ENSG00000230291	ENSG00000230291	12	80102899	80103333	processed_pseudogene	36,61	0
ENSG00000231964	ENSG00000231964	10	45444570	45453121	antisense	36,60	0

ENSG00000269737	ENSG00000269737	1	1671990	1673411	antisense	36,60	0
ENSG00000232956	SNHG15	7	44983023	44986961	lincRNA	36,59	0
ENSG00000178503	NECAP1P1	7	37585500	37586329	processed_pseudogene	36,58	0
ENSG00000246596	ENSG00000246596	5	177619059	177672209	transcribed_unprocessed_pseudogene	36,53	0
ENSG00000266296	ARIH2P1	18	28651732	28653208	processed_pseudogene	36,52	0
ENSG00000226328	NUP50-AS1	22	45133020	45163781	lincRNA	36,43	0
ENSG00000234742	ENSG00000234742	3	197850400	197850954	processed_pseudogene	36,43	0,0358681
ENSG00000249026	ENSG00000249026	5	115389643	115392370	processed_pseudogene	36,42	0
ENSG00000228703	ENSG00000228703	1	109628417	109630305	antisense	36,34	0
ENSG00000253138	LINC00967	8	66192093	66197315	lincRNA	36,30	0
ENSG00000276529	ENSG00000276529	21	44978832	44979274	lincRNA	36,27	0
ENSG00000270987	ENSG00000270987	6	100889603	100890338	processed_pseudogene	36,24	0
ENSG00000223534	HLA-DQB1-AS1	6	32659880	32660729	antisense	36,20	0
ENSG00000228989	ENSG00000228989	2	241690414	241694289	antisense	36,20	0
ENSG00000272256	ENSG00000272256	8	30082758	30083467	antisense	36,20	0
ENSG00000224628	ENSG00000224628	20	31285317	31286835	processed_pseudogene	36,15	0
ENSG00000267419	ENSG00000267419	19	19776602	19836073	transcribed_unprocessed_pseudogene	36,10	0
ENSG00000230870	FBXW11P1	21	31627127	31628600	processed_pseudogene	36,09	0
ENSG00000231544	RSL24D1P11	18	57838476	57838966	processed_pseudogene	36,00	0,2161218
ENSG00000255284	ENSG00000255284	11	777578	784297	lincRNA	36,00	0
ENSG00000255958	ENSG00000255958	12	10214161	10214761	antisense	35,90	0
ENSG00000230454	ENSG00000230454	3	50260303	50263358	lincRNA	35,80	0
ENSG00000226963	ENSG00000226963	2	172427774	172428603	antisense	35,77	0
ENSG00000188206	HNRNPU-AS1	1	244840638	244846903	antisense	35,73	0
ENSG00000278709	NKILA	20	57710183	57712780	antisense	35,70	0
ENSG00000259775	ENSG00000259775	14	103331674	103332367	antisense	35,70	0
ENSG00000229519	ENSG00000229519	1	6547905	6548619	processed_pseudogene	35,70	1,40E-02
ENSG00000259216	ENSG00000259216	15	48725338	48725827	processed_pseudogene	35,69	0
ENSG00000226696	LENG8-AS1	19	54444813	54449045	antisense	35,60	0
ENSG00000256282	ENSG00000256282	11	18595459	18599683	transcribed_processed_pseudogene	35,60	1,40E-02
ENSG00000247925	ENSG00000247925	6	11173452	11259099	antisense	35,60	0
ENSG00000254855	ENSG00000254855	11	66264777	66265666	antisense	35,58	0
ENSG00000269973	ENSG00000269973	2	9936360	9939590	lincRNA	35,52	0
ENSG00000263859	ENSG00000263859	17	81878667	81879557	lincRNA	35,50	0
ENSG00000233846	ENSG00000233846	9	6278667	6279153	processed_pseudogene	35,40	0
ENSG00000269918	ENSG00000269918	8	11104691	11106704	sense_intronic	35,37	0
ENSG00000262001	DLGAP1-AS2	18	3603000	3608336	antisense	35,30	0
ENSG00000261338	ENSG00000261338	2	218255319	218257366	sense_overlapping	35,30	0
ENSG00000230849	GOT2P2	1	173141100	173142350	processed_pseudogene	35,30	0
ENSG00000268199	ENSG00000268199	19	18441419	18443597	antisense	35,29	0
ENSG00000260231	JHDM1D-AS1	7	140177261	140179640	antisense	35,28	0
ENSG00000229023	ENSG00000229023	2	190992639	190993567	processed_pseudogene	35,23	0
ENSG00000219790	OSTCP6	6	56975606	56976062	processed_pseudogene	35,20	0,2161218
ENSG00000236013	ENSG00000236013	6	140067435	140093721	lincRNA	35,20	0
ENSG00000258959	ENSG00000258959	14	102036315	102066228	antisense	35,16	0
ENSG00000232149	FERP1	X	123603139	123603931	processed_pseudogene	35,16	0
ENSG00000270504	ENSG00000270504	6	3751111	3753871	antisense	35,10	0
ENSG00000248694	ENSG00000248694	4	182772565	182773931	antisense	35,10	0
ENSG00000225138	ENSG00000225138	5	473236	480884	processed_transcript	35,07	0
ENSG00000241158	ADAMTS9-AS1	3	64561322	64592757	antisense	35,06	0
ENSG00000224334	ENSG00000224334	22	24686923	24688087	processed_pseudogene	35,00	0
ENSG00000235665	LINC00298	2	7922425	8278084	lincRNA	34,96	0
ENSG00000232788	ENSG00000232788	2	172464262	172466022	antisense	34,94	0
ENSG00000232176	ENSG00000232176	9	19200335	19201046	processed_pseudogene	34,91	0,1836819
ENSG00000257878	ENSG00000257878	12	95996521	96011489	antisense	34,90	0
ENSG00000277728	ENSG00000277728	17	73202968	73203431	antisense	34,90	0
ENSG00000227487	NCAM1-AS1	11	113269532	113273901	antisense	34,69	0
ENSG00000271707	ATP1B3P1	2	60734895	60735715	processed_pseudogene	34,58	0

ENSG00000270959	LPP-AS2	3	188151206	188154057	antisense	34,53	0
ENSG00000259865	ENSG00000259865	1	247187281	247188526	lincRNA	34,50	0
ENSG00000238278	ALG1L6P	3	75415070	75422143	unprocessed_pseudogene	34,45	0
ENSG00000260621	ENSG00000260621	16	56409320	56411683	antisense	34,43	0
ENSG00000251556	ENSG00000251556	5	146099406	146120412	antisense	34,41	0
ENSG00000256612	CYP2B7P	19	40924219	40950660	transcribed_unprocessed_pseudogene	34,40	0
ENSG00000260855	ENSG00000260855	1	246772301	246775772	antisense	34,40	0
ENSG00000254414	ENSG00000254414	15	84631898	84633987	antisense	34,36	0
ENSG00000272831	ENSG00000272831	7	66739829	66740385	antisense	34,36	0
ENSG00000232794	HNRNPDP1	X	64044305	64045058	processed_pseudogene	34,31	0
ENSG00000274561	ENSG00000274561	17	68131462	68131907	lincRNA	34,30	0
ENSG00000225357	RPF2P1	20	35752814	35753719	processed_pseudogene	34,26	0
ENSG00000268006	PTOV1-AS1	19	49838639	49851676	antisense	34,26	0
ENSG00000205898	ENSG00000205898	7	125159974	125160547	processed_pseudogene	34,20	0
ENSG00000271270	TMCC1-AS1	3	129893871	129918575	antisense	34,18	0
ENSG00000257621	PSMA3-AS1	14	58265365	58298134	antisense	34,15	0
ENSG00000273576	ENSG00000273576	17	39566915	39567559	lincRNA	34,10	0
ENSG00000224172	ENSG00000224172	7	64735933	64736743	processed_pseudogene	34,10	0
ENSG00000225449	RAB6C-AS1	2	129966592	129980466	transcribed_unprocessed_pseudogene	34,09	6,27E-03
ENSG00000232043	ENSG00000232043	20	50570975	50578041	antisense	34,08	0
ENSG00000235001	EIF4A1P2	X	5337745	5338964	processed_pseudogene	34,06	0
ENSG00000204860	FAM201A	9	38620474	38624990	antisense	34,00	0
ENSG00000270223	ENSG00000270223	X	74009389	74011468	sense_intronic	34,00	0
ENSG00000272631	ENSG00000272631	10	86749754	86756298	antisense	33,88	0
ENSG00000272004	ENSG00000272004	1	1659325	1662602	antisense	33,81	0
ENSG00000236754	ENSG00000236754	22	17580157	17589192	antisense	33,80	0
ENSG00000271032	ENSG00000271032	19	35014961	35025335	antisense	33,80	0
ENSG00000233622	CYP2T1P	19	40808525	40811390	unitary_pseudogene	33,75	0
ENSG00000261278	ENSG00000261278	16	60024578	60025141	processed_pseudogene	33,70	7,06E-02
ENSG00000223741	PSMD4P1	21	36485983	36487411	processed_pseudogene	33,60	0,2161218
ENSG00000276278	ENSG00000276278	15	84622015	84623237	antisense	33,60	0
ENSG00000227220	ENSG00000227220	6	131950946	132077393	antisense	33,60	0
ENSG00000251435	C1GALT1P2	5	56192530	56193633	processed_pseudogene	33,60	0
ENSG00000271774	ENSG00000271774	20	53255979	53487274	antisense	33,56	0
ENSG00000266980	ENSG00000266980	17	75818815	75820055	antisense	33,46	0
ENSG00000246100	LINC00900	11	115754248	115760627	lincRNA	33,40	0
ENSG00000260805	ENSG00000260805	1	212557833	212559731	lincRNA	33,40	0
ENSG00000272356	ENSG00000272356	6	111309203	111313517	antisense	33,36	0
ENSG00000227201	CNN2P1	22	30046276	30047193	processed_pseudogene	33,30	0
ENSG00000231162	COX11P1	6	28446973	28447807	processed_pseudogene	33,30	0
ENSG00000265399	ENSG00000265399	18	3190397	3247277	antisense	33,29	0
ENSG00000259856	RAB43P1	16	46626404	46626992	processed_pseudogene	33,13	0
ENSG00000262772	ENSG00000262772	17	79823452	79827704	lincRNA	33,10	0
ENSG00000272324	ENSG00000272324	5	10761065	10770294	antisense	33,10	0
ENSG00000228305	ENSG00000228305	2	63622178	63622831	processed_pseudogene	33,08	0
ENSG00000226210	ENSG00000226210	12	14522	32015	unprocessed_pseudogene	33,03	0
ENSG00000174365	SNHG11	20	38446578	38450921	processed_transcript	33,00	0
ENSG00000223973	ENSG00000223973	2	111383752	111384992	transcribed_processed_pseudogene	33,00	0
ENSG00000165511	C10orf25	10	44997698	45000888	antisense	32,94	0
ENSG00000264701	ENSG00000264701	17	47980398	47996196	antisense	32,92	0
ENSG00000257307	ENSG00000257307	14	35881001	35881709	processed_pseudogene	32,90	0
ENSG00000235314	LINC00957	7	44039171	44042306	lincRNA	32,90	0
ENSG00000267328	ENSG00000267328	19	35754566	35755490	antisense	32,89	0
ENSG00000229190	ENSG00000229190	10	17695709	17700232	antisense	32,80	0
ENSG00000244009	B3GAT3P1	3	160452598	160453403	processed_pseudogene	32,80	0
ENSG00000108958	ENSG00000108958	17	1858039	1858446	processed_pseudogene	32,78	0

ENSG00000230163	ENSG00000230163	1	34850694	34851555	antisense	32,70	0
ENSG00000213904	LIPE-AS1	19	42397128	42652355	antisense	32,61	0
ENSG00000250115	AK3P2	8	143057060	143057816	processed_pseudogene	32,60	0
ENSG00000251441	RTEL1P1	4	112356135	112359819	processed_pseudogene	32,60	0
ENSG00000260572	ENSG00000260572	3	160753428	160755142	antisense	32,55	0
ENSG00000260465	ENSG00000260465	16	66720897	66731785	antisense	32,52	0
ENSG00000235472	EIF4A1P7	13	28598833	28600052	processed_pseudogene	32,52	0
ENSG00000268047	ENSG00000268047	19	49852887	49854967	antisense	32,51	0
ENSG00000176826	FKBP9P1	7	55681074	55713252	transcribed_unprocessed_pseudogene	32,50	0
ENSG00000255468	ENSG00000255468	11	66347950	66364804	antisense	32,42	0
ENSG00000257359	ENSG00000257359	12	113932569	113937255	antisense	32,36	0
ENSG00000235437	LINC01278	X	63343227	63561071	processed_transcript	32,25	0
ENSG00000277558	ENSG00000277558	20	34476205	34476787	sense_intronic	32,20	0
ENSG00000233222	ENSG00000233222	1	153750983	153752176	antisense	32,20	0
ENSG00000213839	TMX2P1	9	37885683	37886390	processed_pseudogene	32,13	0
ENSG00000258515	ENSG00000258515	14	20451305	20451918	antisense	32,10	0
ENSG00000278784	ENSG00000278784	14	24201612	24202811	sense_intronic	32,10	0
ENSG00000240471	PHBP8	3	119791829	119792553	processed_pseudogene	32,08	7,06E-02
ENSG00000254559	ENSG00000254559	11	203623	205470	antisense	32,07	0
ENSG00000223482	NUTM2A-AS1	10	87203875	87342612	antisense	32,04	0
ENSG00000233929	MT1XP1	1	16241213	16241398	processed_pseudogene	32,02	0,4850164
ENSG00000268379	ENSG00000268379	19	57175233	57177921	processed_pseudogene	32,00	0
ENSG00000259032	ENSAP2	14	82692704	82693055	processed_pseudogene	31,94	0
ENSG00000273203	ENSG00000273203	22	17067821	17070675	lincRNA	31,90	0
ENSG00000215241	ENSG00000215241	12	8235415	8242564	lincRNA	31,90	0
ENSG00000274964	ENSG00000274964	12	32339368	32340724	sense_intronic	31,82	0
ENSG00000245025	ENSG00000245025	8	22984596	23019335	antisense	31,82	0
ENSG00000272574	ENSG00000272574	1	162593103	162593754	sense_intronic	31,80	0
ENSG00000254860	TMEM9B-AS1	11	8964675	8977527	antisense	31,73	0
ENSG00000279738	ENSG00000279738	22	37876148	37895563	sense_overlapping	31,70	0
ENSG00000235706	DICER1-AS1	14	95157645	95179933	antisense	31,70	0
ENSG00000229644	NAMPTP1	10	36521721	36524234	processed_pseudogene	31,69	0
ENSG00000246985	SOCS2-AS1	12	93542463	93571768	processed_transcript	31,63	0
ENSG00000264714	ENSG00000264714	18	10724619	10728539	transcribed_processed_pseudogene	31,60	0
ENSG00000250332	ENSG00000250332	5	23303565	23305143	processed_pseudogene	31,60	0
ENSG00000256469	ENSG00000256469	11	94874052	94925521	antisense	31,57	0
ENSG00000236779	ENSG00000236779	1	204528845	204529692	processed_pseudogene	31,50	0
ENSG00000264235	ENSG00000264235	18	3255436	3261850	antisense	31,49	0
ENSG00000248881	ENSG00000248881	5	75598482	75599380	antisense	31,48	0
ENSG00000232882	PHKA1P1	1	90892992	90893612	processed_pseudogene	31,47	0
ENSG00000233621	LINC01137	1	37454879	37474411	antisense	31,45	0
ENSG00000268729	ENSG00000268729	19	55312029	55312495	antisense	31,44	0
ENSG00000273058	ENSG00000273058	1	236536162	236536704	antisense	31,40	0
ENSG00000257270	ENSG00000257270	14	105467793	105470617	antisense	31,31	0
ENSG00000267481	ENSG00000267481	19	19788755	19790531	sense_intronic	31,31	0
ENSG00000270392	PFN1P2	1	120432204	120434052	transcribed_processed_pseudogene	31,30	0
ENSG00000227063	RPL41P1	20	21755270	21755350	processed_pseudogene	31,24	0
ENSG00000253676	TAGLN2P1	8	106697427	106698013	processed_pseudogene	31,24	0
ENSG00000251239	ENSG00000251239	17	50627032	50631940	antisense	31,20	0
ENSG00000244625	MIATNB	22	26672767	26780207	lincRNA	31,10	0
ENSG00000261101	ENSG00000261101	X	101627868	101628523	sense_overlapping	31,10	0
ENSG00000248049	UBA6-AS1	4	67701280	68080952	antisense	31,02	0
ENSG00000259780	ENSG00000259780	16	2235689	2236913	lincRNA	31,00	0
ENSG00000224097	ENSG00000224097	4	39480255	39481905	transcribed_processed_pseudogene	31,00	0
ENSG00000274414	ENSG00000274414	20	25239007	25245229	lincRNA	30,90	0
ENSG00000235879	FAR1P1	13	90170866	90171799	processed_pseudogene	30,90	0
ENSG00000250271	ENSG00000250271	3	151797484	151808249	lincRNA	30,90	0

ENSG00000239455	ENSG00000239455	3	107104911	107107000	processed_pseudogene	30,82	0
ENSG00000161643	SIGLEC16	19	49969673	49975814	transcribed_unprocessed_pseudogene	30,80	0
ENSG00000265474	ENSG00000265474	17	28745569	28747652	antisense	30,80	0
ENSG00000215493	ENSG00000215493	22	20450122	20451824	processed_pseudogene	30,73	0
ENSG00000261188	ENSG00000261188	22	26512537	26514568	antisense	30,70	0
ENSG00000224138	ENSG00000224138	7	127350128	127351523	antisense	30,70	0
ENSG00000272760	ENSG00000272760	7	154951226	154952188	antisense	30,70	0
ENSG00000213493	ACTN4P1	4	116598161	116599611	processed_pseudogene	30,68	0
ENSG00000234055	ENSG00000234055	9	129097854	129100266	antisense	30,60	0
ENSG00000225969	ABHD11-AS1	7	73735038	73736054	antisense	30,60	0
ENSG00000258092	ENSG00000258092	12	2797136	2803938	antisense	30,60	0
ENSG00000257376	ENSG00000257376	12	42431665	42433357	processed_pseudogene	30,54	0
ENSG00000267254	ZNF790-AS1	19	36797518	36828115	antisense	30,54	0
ENSG00000264019	ENSG00000264019	17	47946802	47948275	antisense	30,54	0
ENSG00000253982	ENSG00000253982	8	1761990	1764502	antisense	30,53	0
ENSG00000080947	CROCCP3	1	16467436	16499257	transcribed_unprocessed_pseudogene	30,52	0
ENSG00000261526	ENSG00000261526	19	1874871	1876169	lincRNA	30,50	0
ENSG00000260105	AOC4P	17	42865922	42874369	transcribed_unprocessed_pseudogene	30,50	0
ENSG00000260911	ENSG00000260911	16	31043150	31049868	lincRNA	30,50	0
ENSG00000268234	FKBP4P6	9	42384805	42386136	processed_pseudogene	30,47	0
ENSG00000255503	ENSG00000255503	11	83072402	83097196	lincRNA	30,44	0
ENSG00000237719	ENSG00000237719	6	36091991	36092646	processed_pseudogene	30,40	0
ENSG00000237798	ENSG00000237798	2	174575227	174587726	antisense	30,40	0
ENSG00000197301	ENSG00000197301	12	65851340	65882167	antisense	30,30	0
ENSG00000185986	SDHAP3	5	1572222	1594620	transcribed_unprocessed_pseudogene	30,29	0
ENSG00000234936	ENSG00000234936	2	43229573	43233394	antisense	30,23	0
ENSG00000183506	PI4KAP2	22	21473000	21517533	transcribed_unprocessed_pseudogene	30,21	0
ENSG00000235958	UBOX5-AS1	20	3106913	3150867	antisense	30,20	0
ENSG00000236581	STARD13-AS	13	33180401	33281584	processed_transcript	30,20	0
ENSG00000276517	ENSG00000276517	2	32526504	32529507	sense_intronic	30,13	0
ENSG00000255422	ENSG00000255422	11	118704607	118750263	antisense	30,12	0
ENSG00000236567	TCF3P1	9	5110913	5112849	processed_pseudogene	30,10	0
ENSG00000272142	ENSG00000272142	6	5029972	5043449	lincRNA	30,10	0
ENSG00000273485	ENSG00000273485	10	103450196	103450852	antisense	30,00	0
ENSG00000088340	FER1L4	20	35558737	35607562	unitary_pseudogene	29,97	0
ENSG00000233030	ENSG00000233030	1	149785659	149793020	antisense	29,90	0
ENSG00000229851	ARSD-AS1	X	2904904	2906081	antisense	29,87	0
ENSG00000245904	ENSG00000245904	12	92145573	92189660	antisense	29,82	0
ENSG00000229758	DYNLT3P2	2	128199901	128200195	processed_pseudogene	29,80	0
ENSG00000244567	ENSG00000244567	2	207821290	207822769	lincRNA	29,76	0
ENSG00000255455	ENSG00000255455	11	130866254	130870247	lincRNA	29,76	0
ENSG00000240291	ENSG00000240291	10	18513115	18545651	antisense	29,75	0
ENSG00000262691	ENSG00000262691	16	67261108	67263784	processed_transcript	29,70	0
ENSG00000249779	ENSG00000249779	5	43206709	43207811	processed_pseudogene	29,70	0,2161218
ENSG00000214465	SMARCE1P6	2	26149204	26150731	processed_pseudogene	29,67	0,1392851
ENSG00000251667	BRCC3P1	5	176308063	176309013	processed_pseudogene	29,61	0
ENSG00000231871	IPO9-AS1	1	201688259	201829559	antisense	29,55	0
ENSG00000187953	PMS2CL	7	6710128	6753862	transcribed_unprocessed_pseudogene	29,54	0
ENSG00000172460	PRSS30P	16	2839568	2842744	unitary_pseudogene	29,50	0
ENSG00000281332	LINC00997	7	32760279	32762924	lincRNA	29,50	0
ENSG00000238197	PAXBP1-AS1	21	32728115	32743122	antisense	29,49	0
ENSG00000255993	PSMC1P9	12	18692762	18694096	processed_pseudogene	29,45	4,86E-02
ENSG00000263326	ENSG00000263326	16	25071490	25072727	unprocessed_pseudogene	29,40	0
ENSG00000257808	ENSG00000257808	12	53159586	53161000	lincRNA	29,38	0

ENSG00000273271	ENSG00000273271	21	31666728	31667247	antisense	29,30	0
ENSG00000267571	ENSG00000267571	19	5911578	5913899	antisense	29,20	0
ENSG00000223509	ENSG00000223509	15	32519848	32536926	transcribed_processed_pseudogene	29,20	0
ENSG00000067601	PMS2P4	7	67295608	67302907	transcribed_unprocessed_pseudogene	29,17	0
ENSG00000274383	ENSG00000274383	15	64950916	64951435	antisense	29,10	0
ENSG00000235902	ENSG00000235902	4	185665885	185675417	antisense	29,10	0
ENSG00000273179	ENSG00000273179	4	1167778	1168174	antisense	29,10	0
ENSG00000236138	DUX4L26	3	75668931	75670185	processed_pseudogene	29,10	7,06E-02
ENSG00000234449	ENSG00000234449	X	3853010	3882317	lincRNA	29,08	0
ENSG00000241111	ENSG00000241111	3	64067964	64103131	antisense	29,08	0
ENSG00000233351	ENSG00000233351	6	129479615	129481410	antisense	29,05	0
ENSG00000228830	ENSG00000228830	1	234607008	234609483	antisense	29,03	0
ENSG00000256116	ENSG00000256116	11	64229214	64234352	antisense	29,01	0
ENSG00000248027	ENSG00000248027	11	100684162	100687955	antisense	29,00	0
ENSG00000214961	ENSG00000214961	X	134272287	134273658	processed_pseudogene	29,00	0
ENSG00000117242	PINK1-AS	1	20642657	20652193	antisense	28,98	0
ENSG00000188185	LINC00265	7	39733632	39793092	lincRNA	28,95	0
ENSG00000237842	ENSG00000237842	1	157059232	157060762	processed_pseudogene	28,93	0
ENSG00000228393	LINC01004	7	104950315	105013044	antisense	28,90	0
ENSG00000243150	ENSG00000243150	3	158545220	158571066	antisense	28,88	0
ENSG00000258900	HNRNPCP1	14	58521223	58521988	processed_pseudogene	28,84	6,27E-03
ENSG00000272578	ENSG00000272578	22	23658094	23717356	transcribed_unprocessed_pseudogene	28,82	0
ENSG00000261616	ENSG00000261616	15	99139317	99145370	antisense	28,80	0
ENSG00000270050	ENSG00000270050	X	102599657	102659712	antisense	28,70	0
ENSG00000272841	ENSG00000272841	6	160990318	160992342	antisense	28,67	0
ENSG00000240375	VPS26AP1	3	113919222	113920200	processed_pseudogene	28,67	9,18E-02
ENSG00000253882	ENSG00000253882	7	143761790	143836933	transcribed_unprocessed_pseudogene	28,63	0
ENSG00000224936	SUCLA2P1	6	30468882	30470251	processed_pseudogene	28,63	0
ENSG00000214019	ENSG00000214019	X	44029332	44030921	processed_pseudogene	28,58	0
ENSG00000215817	ZC3H11B	1	219609627	219612062	processed_pseudogene	28,57	0
ENSG00000237714	P4HA2-AS1	5	132184876	132192808	antisense	28,53	0
ENSG00000267390	ENSG00000267390	18	63367328	63381629	antisense	28,50	0
ENSG00000262624	ENSG00000262624	17	7436557	7437523	antisense	28,50	0
ENSG00000272129	ENSG00000272129	6	80355424	80356859	lincRNA	28,50	0
ENSG00000260000	ENSG00000260000	6	100881471	100882987	antisense	28,47	0
ENSG00000258300	NUTF2P2	14	35819837	35820220	processed_pseudogene	28,43	0
ENSG00000276710	CSPG4P10	15	82459472	82477258	transcribed_unprocessed_pseudogene	28,41	0
ENSG00000259250	ENSG00000259250	15	58587507	58591676	antisense	28,40	0
ENSG00000273226	ENSG00000273226	9	19375451	19375996	antisense	28,40	0
ENSG00000213300	HNRNPA3P6	3	75214631	75215636	processed_pseudogene	28,37	0
ENSG00000276570	ENSG00000276570	19	55227219	55230279	processed_transcript	28,30	0
ENSG00000259424	ENSG00000259424	15	98646951	98647371	antisense	28,30	0
ENSG00000215571	GRK6P1	13	21319156	21320866	processed_pseudogene	28,30	0
ENSG00000243323	PTPRVP	1	202168051	202189455	unitary_pseudogene	28,30	0
ENSG00000235245	ENSG00000235245	10	133295187	133295977	antisense	28,24	0
ENSG00000241537	ENSG00000241537	3	72772539	72773471	processed_pseudogene	28,24	0
ENSG00000247828	TMEM161B-AS1	5	88268895	88436685	antisense	28,23	0
ENSG00000261602	ENSG00000261602	16	69709874	69710583	antisense	28,20	0
ENSG00000274227	ENSG00000274227	12	112018804	112019430	antisense	28,20	0
ENSG00000253669	ENSG00000253669	8	102805517	102809971	lincRNA	28,20	0
ENSG00000236015	ENSG00000236015	7	39569376	39570198	processed_pseudogene	28,20	0
ENSG00000249379	ENSG00000249379	6	53503185	53506919	antisense	28,20	0
ENSG00000276248	ENSG00000276248	13	113527260	113530621	sense_intronic	28,18	0
ENSG00000241014	ENSG00000241014	1	34974356	34985313	processed_transcript	28,17	0
ENSG00000269910	ENSG00000269910	14	103694516	103695050	antisense	28,12	0
ENSG00000249142	ENSG00000249142	5	32522758	32523865	processed_pseudogene	28,10	0

ENSG00000217165	ANKRD18EP	6	39110321	39112952	processed_pseudogene	28,07	0
ENSG00000272789	ENSG00000272789	2	127625997	127626848	antisense	28,07	0,1392851
ENSG00000264247	LINC00909	18	74591774	74598508	lincRNA	28,06	0
ENSG00000271970	ENSG00000271970	6	43561872	43562419	antisense	28,05	0
ENSG00000272688	ENSG00000272688	18	3246401	3247086	lincRNA	28,00	0
ENSG00000264920	ENSG00000264920	17	47891255	47895812	processed_transcript	28,00	0
ENSG00000259165	DDX18P1	14	69083398	69085389	processed_pseudogene	28,00	0
ENSG00000267940	ENSG00000267940	11	6201901	6203253	lincRNA	28,00	0
ENSG00000244968	LIFR-AS1	5	38556786	38671216	antisense	28,00	0
ENSG00000272667	ENSG00000272667	2	127886556	127887185	lincRNA	27,90	0
ENSG00000176318	FOXN3P1	X	102546734	102548213	processed_pseudogene	27,90	0
ENSG00000264666	ENSG00000264666	17	17591428	17610485	antisense	27,85	0
ENSG00000254913	ENSG00000254913	1	149006309	149009527	antisense	27,80	0
ENSG00000253330	ENSG00000253330	8	47511034	47512141	processed_pseudogene	27,75	0
ENSG00000258554	ENSG00000258554	12	55966838	55967474	antisense	27,74	0
ENSG00000213530	MTHFD2P5	7	82589848	82590691	processed_pseudogene	27,70	1,40E-02
ENSG00000272223	ENSG00000272223	6	43033897	43034405	antisense	27,70	0
ENSG00000229020	AKR7A2P1	1	112923423	112924337	processed_pseudogene	27,70	0
ENSG00000270380	ENSG00000270380	1	110456505	110457354	antisense	27,70	0
ENSG00000270075	ENSG00000270075	10	104312141	104313881	antisense	27,67	0
ENSG00000269958	ENSG00000269958	14	103696353	103697163	sense_intronic	27,64	0
ENSG00000213434	VTI1BP2	4	183483490	183484185	processed_pseudogene	27,62	0,1836819
ENSG00000270598	ENSG00000270598	1	226127178	226127346	processed_pseudogene	27,60	0
ENSG00000251022	THAP9-AS1	4	82893009	82900960	antisense	27,59	0
ENSG00000260185	ENSG00000260185	16	71655027	71664212	antisense	27,58	0
ENSG00000250397	ENSG00000250397	11	856880	859795	antisense	27,50	0
ENSG00000214135	ENSG00000214135	3	197578213	197627906	transcribed_unprocessed_pseudogene	27,40	0
ENSG00000274213	ENSG00000274213	17	56914186	56914533	lincRNA	27,40	0
ENSG00000269765	ENSG00000269765	19	15515342	15516836	unprocessed_pseudogene	27,36	0
ENSG00000251474	RPL32P3	3	129382922	129399655	transcribed_unprocessed_pseudogene	27,27	0
ENSG00000265273	PGDP1	18	31962178	31963618	processed_pseudogene	27,25	0
ENSG00000273373	ENSG00000273373	1	110370154	110373003	antisense	27,25	0
ENSG00000230565	ZNF32-AS2	10	43645942	43648019	antisense	27,23	0
ENSG00000273237	ENSG00000273237	7	26173533	26174945	antisense	27,20	0
ENSG00000250526	CCT6P2	5	14639426	14641018	processed_pseudogene	27,20	0
ENSG00000234558	API5P1	X	116106921	116108430	processed_pseudogene	27,19	0
ENSG00000213500	LAP3P2	6	36673817	36675270	processed_pseudogene	27,14	0
ENSG00000278291	ENSG00000278291	13	20699307	20703718	antisense	27,10	0
ENSG00000235865	GSN-AS1	9	121280768	121285530	antisense	27,07	0
ENSG00000261359	PYCARD-AS1	16	31201885	31203452	antisense	27,00	0
ENSG00000226380	ENSG00000226380	7	130876809	130913310	lincRNA	26,93	0
ENSG00000281691	RBM5-AS1	3	50099603	50100988	antisense	26,92	0
ENSG00000214076	CPSF1P1	22	32269381	32273110	processed_pseudogene	26,90	0
ENSG00000250286	ENSG00000250286	17	50537294	50538754	processed_transcript	26,90	0
ENSG00000282728	ENSG00000282728	11	101129077	101209591	processed_transcript	26,90	0
ENSG00000234534	CSNK1G2P1	9	5040945	5041940	processed_pseudogene	26,90	0
ENSG00000223797	ENTPD3-AS1	3	40313802	40453329	antisense	26,90	0
ENSG00000256271	CACNA1C-AS2	12	2668500	2672220	antisense	26,84	0
ENSG00000224837	GCSHP5	1	168055901	168056422	processed_pseudogene	26,83	0
ENSG00000237788	ENSG00000237788	20	49829125	49831085	processed_pseudogene	26,80	0
ENSG00000242797	GLYCTK-AS1	3	52288580	52299067	processed_transcript	26,80	0
ENSG00000236811	GAPDHP2	20	13392677	13393674	processed_pseudogene	26,70	0,2161218
ENSG00000237118	CYP2F2P	19	40818721	40826772	unitary_pseudogene	26,70	0
ENSG00000261094	ENSG00000261094	9	122937623	122940333	sense_overlapping	26,70	0
ENSG00000219294	PIP5K1P1	6	7986537	7988192	processed_pseudogene	26,70	0
ENSG00000231203	KRT8P10	2	183071040	183072486	processed_pseudogene	26,70	0
ENSG00000242282	ENSG00000242282	2	3531813	3536873	lincRNA	26,70	0
ENSG00000262420	ENSG00000262420	16	11741910	11744506	antisense	26,64	0

ENSG00000267370	ENSG00000267370	19	9756152	9756863	unprocessed_pseudogene	26,60	0
ENSG00000266101	ENSG00000266101	17	39173290	39177503	antisense	26,60	0
ENSG00000235423	ENSG00000235423	12	123252030	123261483	antisense	26,60	0
ENSG00000255410	ENSG00000255410	11	6630603	6635208	antisense	26,60	0
ENSG00000259488	ENSG00000259488	15	48312353	48331856	antisense	26,58	0
ENSG00000223947	ENSG00000223947	2	100993676	101002244	antisense	26,56	0
ENSG00000198406	BZW1P2	3	116645902	116646727	processed_pseudogene	26,54	0
ENSG00000228638	FCF1P2	3	48290793	48291375	processed_pseudogene	26,52	0
ENSG00000261098	ENSG00000261098	11	107312132	107316271	lincRNA	26,50	0
ENSG00000248565	TECRP2	4	124462863	124463790	processed_pseudogene	26,50	0,2161218
ENSG00000227742	CALR4P	1	51561866	51594897	unitary_pseudogene	26,50	0
ENSG00000237897	ENSG00000237897	1	105890693	105891430	processed_pseudogene	26,50	7,06E-02
ENSG00000250071	ENSG00000250071	5	74798994	74800100	processed_pseudogene	26,47	0
ENSG00000254428	ENSG00000254428	11	119003742	119004893	antisense	26,40	0
ENSG00000232303	DFFBP1	9	32566148	32567126	processed_pseudogene	26,40	0
ENSG00000240950	ENSG00000240950	3	142827096	142827728	processed_pseudogene	26,38	3,30E-03
ENSG00000268983	ENSG00000268983	19	18568506	18569375	antisense	26,37	0
ENSG00000229848	ENSG00000229848	17	81514047	81527776	antisense	26,36	0
ENSG00000229692	SOS1-IT1	2	38992279	38993857	sense_intronic	26,33	0
ENSG00000228314	CYP4F29P	21	13843133	13848364	transcribed_unprocessed_pseudogene	26,30	0
ENSG00000274370	ENSG00000274370	17	83098377	83098987	lincRNA	26,30	0
ENSG00000232104	RFX3-AS1	9	3526723	3691814	lincRNA	26,30	0
ENSG00000246145	RRS1-AS1	8	66419589	66428977	lincRNA	26,30	0
ENSG00000273356	ENSG00000273356	3	50669989	50672048	lincRNA	26,30	0
ENSG00000255500	ENSG00000255500	11	50268161	50269273	processed_pseudogene	26,29	0
ENSG00000232909	ENSG00000232909	6	35733867	35736947	antisense	26,25	0
ENSG00000223358	EHHADH-AS1	3	185162871	185191955	antisense	26,20	0
ENSG00000214424	FAM149B1P1	15	96756987	96758596	processed_pseudogene	26,17	0
ENSG00000273348	ENSG00000273348	18	22347846	22348252	lincRNA	26,10	0
ENSG00000225423	TNPO1P1	10	84390908	84392045	processed_pseudogene	26,10	0
ENSG00000249167	ENSG00000249167	5	116574482	116591398	lincRNA	26,10	0
ENSG00000172799	ZBTB8OSP2	2	231433923	231434426	processed_pseudogene	26,10	0
ENSG00000281195	ZNF638-IT1	2	71373938	71376320	processed_transcript	26,10	0
ENSG00000278058	ENSG00000278058	16	79605802	79606605	lincRNA	26,00	0
ENSG00000257524	ENSG00000257524	9	127867864	127886787	processed_transcript	26,00	0
ENSG00000270228	ENSG00000270228	4	67718996	67724262	processed_pseudogene	26,00	0
ENSG00000273275	ENSG00000273275	2	68179833	68180532	antisense	25,98	0
ENSG00000236975	ENSG00000236975	1	229092815	229094905	lincRNA	25,93	0
ENSG00000271971	ENSG00000271971	8	93715378	93716113	antisense	25,90	0
ENSG00000219755	ENSG00000219755	6	99575712	99576456	processed_pseudogene	25,90	0
ENSG00000275880	ENSG00000275880	13	110613082	110616353	antisense	25,89	0
ENSG00000242352	FAM91A3P	1	143766540	143769083	transcribed_processed_pseudogene	25,86	0
ENSG00000205181	LINC00654	20	5496067	5504613	lincRNA	25,84	0
ENSG00000213713	PIGCP1	11	33075566	33076460	processed_pseudogene	25,83	0
ENSG00000217801	ENSG00000217801	1	1059734	1069355	transcribed_unprocessed_pseudogene	25,83	2,21E-02
ENSG00000267080	ASB16-AS1	17	44175973	44186717	antisense	25,80	0
ENSG00000237522	NONOP2	2	60936819	60938049	processed_pseudogene	25,80	0
ENSG00000267523	ENSG00000267523	19	55661901	55674715	antisense	25,75	0
ENSG00000235081	ENSG00000235081	19	54229036	54230213	processed_pseudogene	25,74	0
ENSG00000276107	ENSG00000276107	15	39586561	39587293	sense_intronic	25,73	0
ENSG00000243829	ENSG00000243829	19	49719498	49720081	processed_pseudogene	25,72	0
ENSG00000266709	ENSG00000266709	17	14303854	14305505	lincRNA	25,70	0
ENSG00000233205	ENSG00000233205	2	87972656	87973082	processed_pseudogene	25,70	0
ENSG00000250519	ENSG00000250519	11	94238150	94279206	lincRNA	25,60	0
ENSG00000229391	HLA-DRB6	6	32552713	32560022	transcribed_unprocessed_pseudogene	25,60	0
ENSG00000237753	ENSG00000237753	2	112641832	112645690	lincRNA	25,60	0

ENSG00000251485	ENSG00000251485	2	232343116	232343903	processed_pseudogene	25,60	0
ENSG00000223891	OSER1-AS1	20	44210960	44226027	lincRNA	25,56	0
ENSG00000230795	HLA-K	6	29926459	29929232	unprocessed_pseudoge ne	25,50	0
ENSG00000267510	ENSG00000267510	19	9291515	9294482	antisense	25,45	0
ENSG00000228125	AKIRIN1P2	X	68132868	68133437	processed_pseudogene	25,44	0
ENSG00000224888	ENSG00000224888	16	88731180	88741425	antisense	25,43	0
ENSG00000272918	ENSG00000272918	7	105102838	105105483	antisense	25,40	0
ENSG00000273478	ENSG00000273478	1	201995696	201996352	antisense	25,40	0
ENSG00000177803	ENSG00000177803	4	163932526	163933182	processed_pseudogene	25,36	0
ENSG00000226783	TLK1P1	9	122685933	122688372	processed_pseudogene	25,30	0
ENSG00000259150	LINC00929	15	26115813	26133037	lincRNA	25,30	7,06E-02
ENSG00000251356	RAB5CP2	5	91476382	91477018	processed_pseudogene	25,30	0
ENSG00000237276	ANO7P1	1	16215909	16228027	transcribed_unprocess ed_pseudogene	25,30	0
ENSG00000271643	ENSG00000271643	3	33795688	33796950	lincRNA	25,24	0
ENSG00000278727	ENSG00000278727	13	76887551	76891135	lincRNA	25,18	0
ENSG00000235426	ENSG00000235426	10	79382328	79409274	antisense	25,18	6,27E-03
ENSG00000248256	OCIAD1-AS1	4	48852008	48860203	antisense	25,18	0
ENSG00000255220	DDX18P5	11	130295641	130296946	processed_pseudogene	25,17	0
ENSG00000258048	ENSG00000258048	12	79690144	79778451	lincRNA	25,08	0
ENSG00000254452	ENSG00000254452	11	66276779	66277492	antisense	25,08	0
ENSG00000259022	DNAJC8P1	14	35430260	35430631	processed_pseudogene	25,07	0
ENSG00000227227	ENSG00000227227	2	186641339	186695287	antisense	25,07	0
ENSG00000248113	ENSG00000248113	4	82580117	82581384	processed_pseudogene	25,06	0
ENSG00000228766	RPL7L1P8	3	182610674	182611414	processed_pseudogene	24,92	3,30E-03
ENSG00000281896	ENSG00000281896	7	128617865	128625905	transcribed_unprocess ed_pseudogene	24,91	0
ENSG00000270344	ENSG00000270344	12	89525654	89548005	antisense	24,90	0
ENSG00000225217	HSPA7	1	161606291	161608217	unprocessed_pseudoge ne	24,90	0
ENSG00000249673	NOP14-AS1	4	2934899	2961738	antisense	24,85	0
ENSG00000258128	MKRN9P	12	87782163	87784568	transcribed_processed_ pseudogene	24,81	0
ENSG00000233739	ENSG00000233739	22	38057180	38073940	antisense	24,80	0
ENSG00000250604	ENSG00000250604	4	158199105	158200442	antisense	24,80	0
ENSG00000258824	ENSG00000258824	14	64422935	64448557	antisense	24,79	0
ENSG00000226396	ENSG00000226396	1	19608114	19608568	processed_pseudogene	24,78	0,4332376
ENSG00000225408	ENSG00000225408	9	5719021	5720244	antisense	24,75	0
ENSG00000236035	ENSG00000236035	1	203656969	203657760	processed_pseudogene	24,72	0
ENSG00000224086	ENSG00000224086	22	21938293	21977632	antisense	24,71	0
ENSG00000234493	RHOXF1P1	X	120010718	120015544	transcribed_unprocess ed_pseudogene	24,70	0
ENSG00000227339	THRAP3P1	3	31452832	31455894	processed_pseudogene	24,69	0
ENSG00000177447	CBX3P1	11	27806443	27806996	processed_pseudogene	24,69	0
ENSG00000243431	RPL5P30	11	118560690	118561580	processed_pseudogene	24,67	0,3318209
ENSG00000269001	ZNF818P	19	53212988	53213396	processed_pseudogene	24,60	0
ENSG00000259959	ENSG00000259959	4	47840122	47844339	lincRNA	24,56	0
ENSG00000250462	LRRC37BP1	17	30629680	30637466	transcribed_unprocess ed_pseudogene	24,54	0
ENSG00000214812	ENSG00000214812	1	28120449	28121321	processed_pseudogene	24,54	0
ENSG00000261512	ENSG00000261512	16	46622861	46624451	lincRNA	24,50	0
ENSG00000271554	ENSG00000271554	1	35992109	36013630	antisense	24,50	0
ENSG00000235078	ENSG00000235078	2	3519275	3523197	antisense	24,44	0
ENSG00000263731	ENSG00000263731	17	81878425	81881106	lincRNA	24,44	0
ENSG00000269821	KCNQ1OT1	11	2608328	2699994	antisense	24,42	0
ENSG00000256690	ENSG00000256690	11	62832234	62834043	antisense	24,41	0
ENSG00000225470	JPX	X	73944324	74070408	lincRNA	24,41	0
ENSG00000276144	ENSG00000276144	12	7115736	7116486	lincRNA	24,40	0
ENSG00000259515	ENSG00000259515	14	102933574	102937177	antisense	24,39	0
ENSG00000239268	ENSG00000239268	3	117672154	117997592	lincRNA	24,38	0

ENSG00000258938	ENSG00000258938	14	35819224	35826765	antisense	24,37	0
ENSG00000176868	ENSG00000176868	9	131497479	131500191	antisense	24,35	0
ENSG00000270091	ENSG00000270091	17	19896590	19897287	lincRNA	24,30	0
ENSG00000271344	ENSG00000271344	7	128690451	128691717	lincRNA	24,30	0
ENSG00000274265	ENSG00000274265	1	149176022	149251013	lincRNA	24,30	0
ENSG00000225313	ENSG00000225313	1	33307348	33349245	antisense	24,29	0
ENSG00000257337	ENSG00000257337	12	53014596	53054438	antisense	24,27	0
ENSG00000267601	ENSG00000267601	17	78855478	78855844	antisense	24,26	0
ENSG00000235013	COX20P2	2	230957593	230957950	processed_pseudogene	24,22	0
ENSG00000272630	ENSG00000272630	10	73098044	73101297	lincRNA	24,20	0
ENSG00000238279	ENSG00000238279	1	153533603	153535115	antisense	24,20	0
ENSG00000259924	ENSG00000259924	15	65771176	65772139	processed_pseudogene	24,19	0
ENSG00000259407	ENSG00000259407	15	85744109	85750281	antisense	24,17	0
ENSG00000232060	SLC4A1APP1	9	30558880	30559481	processed_pseudogene	24,15	0
ENSG00000262681	ENSG00000262681	17	19719059	19722428	lincRNA	24,10	7,06E-02
ENSG00000271670	ENSG00000271670	10	120879256	120880667	antisense	24,10	0
ENSG00000253716	MINCR	8	143280161	143281690	antisense	24,10	0
ENSG00000225234	TRAPPC12-AS1	2	3481242	3482409	antisense	24,10	0
ENSG00000224468	ENSG00000224468	1	183138402	183141282	antisense	24,10	0
ENSG00000273218	ENSG00000273218	19	15346068	15348417	lincRNA	24,00	0
ENSG00000257941	ENSG00000257941	12	76057538	76058115	processed_pseudogene	24,00	0
ENSG00000254429	ENSG00000254429	11	75260127	75261025	antisense	24,00	0
ENSG00000230091	TMEM254-AS1	10	80046860	80078912	antisense	24,00	0
ENSG00000218586	ENSG00000218586	7	54933699	54938184	processed_pseudogene	24,00	0
ENSG00000221971	TTC4P1	7	45999621	46000778	processed_pseudogene	24,00	0
ENSG00000220412	ENSG00000220412	6	137705423	137707201	processed_pseudogene	24,00	0
ENSG00000237125	HAND2-AS1	4	173527270	173591324	antisense	24,00	0
ENSG00000243495	GMFBP1	3	100665050	100665476	processed_pseudogene	24,00	0
ENSG00000226688	ENTPD1-AS1	10	95753206	96090238	antisense	23,92	0
ENSG00000236824	BCYRN1	2	47331060	47344517	lincRNA	23,90	0
ENSG00000239332	LINC01119	2	46816697	46859007	lincRNA	23,90	0
ENSG00000214331	ENSG00000214331	16	74332402	74368240	transcribed_unprocessed_pseudogene	23,86	0
ENSG00000274925	ENSG00000274925	16	25257952	25261066	lincRNA	23,82	0
ENSG00000267934	ENSG00000267934	19	23323968	23329101	antisense	23,80	0
ENSG00000281026	N4BP2L2-IT2	13	32504506	32509395	sense_intronic	23,80	0
ENSG00000254702	ENSG00000254702	11	107736009	107738718	processed_pseudogene	23,80	0
ENSG00000267436	ENSG00000267436	19	3544199	3557569	antisense	23,78	0
ENSG00000259366	ENSG00000259366	8	29055935	29056685	sense_intronic	23,78	0
ENSG00000237892	KLF7-IT1	2	207120884	207122044	sense_intronic	23,77	0
ENSG00000214174	AMZ2P1	17	64966550	64975576	transcribed_unprocessed_pseudogene	23,74	0
ENSG00000275367	ENSG00000275367	12	8217758	8221115	lincRNA	23,70	0
ENSG00000260686	ENSG00000260686	5	122832356	122834533	sense_overlapping	23,70	0
ENSG00000239880	ENSG00000239880	3	180323107	180325059	processed_pseudogene	23,70	0,2161218
ENSG00000228384	ENSG00000228384	2	70994510	71002754	antisense	23,70	0
ENSG00000231105	ENSG00000231105	1	21293290	21299774	antisense	23,70	0
ENSG00000270017	ENSG00000270017	15	94855586	94857011	lincRNA	23,69	0,3565078
ENSG00000180015	ENSG00000180015	4	188738373	188739494	processed_pseudogene	23,66	0
ENSG00000258651	ENSG00000258651	14	39103140	39103812	lincRNA	23,64	0
ENSG00000231880	ENSG00000231880	11	47577725	47578277	antisense	23,63	0
ENSG00000236152	MRPS36P1	3	6773037	6773343	processed_pseudogene	23,60	0
ENSG00000273387	ENSG00000273387	22	31082156	31083565	antisense	23,60	0
ENSG00000266777	SH3GL1P1	17	32039974	32042828	transcribed_processed_pseudogene	23,60	0
ENSG00000267002	ENSG00000267002	17	43148368	43171037	lincRNA	23,60	0
ENSG00000248441	LINC01197	15	95209099	95327129	lincRNA	23,60	0
ENSG00000253477	ENSG00000253477	8	103483398	103501676	antisense	23,60	0
ENSG00000251553	ENSG00000251553	5	98679402	98681367	processed_pseudogene	23,58	0
ENSG00000227105	PARP1P1	13	110936759	110939784	processed_pseudogene	23,56	0
ENSG00000267904	ENSG00000267904	19	16639967	16640668	sense_intronic	23,50	0

ENSG00000263753	LINC00667	18	5237826	5246508	lincRNA	23,50	0
ENSG00000264012	ENSG00000264012	18	22175465	22176662	antisense	23,50	0
ENSG00000258701	LINC00638	14	104821201	104823718	lincRNA	23,50	0
ENSG00000255224	ENSG00000255224	8	144078002	144079265	antisense	23,41	0
ENSG00000261270	ENSG00000261270	16	56940278	56941342	sense_intronic	23,40	0
ENSG00000270028	ENSG00000270028	12	123925461	123926083	antisense	23,40	0
ENSG00000229593	SUCLA2P3	2	25079901	25081689	processed_pseudogene	23,40	0
ENSG00000256813	ENSG00000256813	11	60841806	60851081	antisense	23,36	0
ENSG00000276663	ENSG00000276663	16	57245832	57246396	antisense	23,32	0
ENSG00000241218	ENSG00000241218	3	107327830	107329197	processed_pseudogene	23,30	0
ENSG00000235862	ENSG00000235862	1	212624284	212626771	antisense	23,30	0
ENSG00000270231	NBPF8	1	120436353	120467739	unprocessed_pseudogene	23,28	0
ENSG00000226245	ZNF32-AS1	10	43643872	43645047	antisense	23,26	0
ENSG00000257769	ENSG00000257769	16	15608474	15610563	antisense	23,25	0
ENSG00000254578	ENSG00000254578	8	144463817	144465101	antisense	23,20	0
ENSG00000177173	NAP1L4P1	1	116532936	116534092	processed_pseudogene	23,09	0
ENSG00000267078	ENSG00000267078	17	76569792	76571240	antisense	23,00	0
ENSG00000240338	ENSG00000240338	16	75226074	75228197	transcribed_unprocessed_pseudogene	23,00	1,40E-02
ENSG00000259954	IL21R-AS1	16	27447669	27453393	antisense	23,00	0
ENSG00000256661	A2ML1-AS1	12	8776219	8830947	antisense	23,00	0
ENSG00000261428	ENSG00000261428	2	222566899	222569719	antisense	23,00	0
ENSG00000259605	ENSG00000259605	19	45764785	45769806	processed_transcript	22,93	0
ENSG00000258988	ENSG00000258988	14	67199062	67200327	processed_pseudogene	22,92	0
ENSG00000260657	ENSG00000260657	15	68267792	68277994	lincRNA	22,90	0
ENSG00000230438	SERPINB9P1	6	2854657	2881407	lincRNA	22,90	0
ENSG00000227799	ENSG00000227799	2	55224280	55225908	processed_pseudogene	22,90	0
ENSG00000243762	ENSG00000243762	22	20110821	20111875	antisense	22,83	0
ENSG00000265393	ENSG00000265393	8	144512567	144513672	antisense	22,82	0
ENSG00000258101	ENSG00000258101	12	49232790	49264756	antisense	22,80	0
ENSG00000278266	ENSG00000278266	12	127147149	127150081	lincRNA	22,77	0
ENSG00000267218	ENSG00000267218	19	15902300	15903238	processed_pseudogene	22,75	0
ENSG00000273311	DGCR11	22	19046162	19048375	sense_intronic	22,70	0
ENSG00000183250	LINC01547	21	44932814	44939913	lincRNA	22,69	0
ENSG00000234171	RNASEH1-AS1	2	3558492	3561745	antisense	22,69	0
ENSG00000229562	ZFYVE9P1	X	137660519	137662242	processed_pseudogene	22,69	0
ENSG00000180581	SRP9P1	10	91807179	91807439	processed_pseudogene	22,64	0
ENSG00000266261	ENSG00000266261	17	15651590	15654489	antisense	22,60	0
ENSG00000243236	GSTA9P	6	52939726	52957521	transcribed_unprocessed_pseudogene	22,60	0
ENSG00000281832	LINC00602	6	165987551	165989615	lincRNA	22,60	0
ENSG00000175749	EIF3KP1	5	103032376	103033031	processed_pseudogene	22,60	7,06E-02
ENSG00000255031	ENSG00000255031	11	68050740	68053762	antisense	22,56	0
ENSG00000275197	ENSG00000275197	12	25225103	25225665	antisense	22,56	0
ENSG00000205663	ENSG00000205663	X	3891438	3920746	lincRNA	22,55	3,30E-03
ENSG00000224328	MDC1-AS1	6	30703067	30713184	antisense	22,50	0
ENSG00000256667	KLRAP1	12	10588063	10599669	transcribed_unprocessed_pseudogene	22,47	0
ENSG00000259940	ENSG00000259940	16	27213308	27214993	antisense	22,40	0
ENSG00000234685	NUS1P2	13	22915651	22916523	processed_pseudogene	22,40	0
ENSG00000270689	BUD13P1	11	95143637	95145125	processed_pseudogene	22,40	0
ENSG00000237437	ASS1P12	9	32945996	32947222	processed_pseudogene	22,40	0
ENSG00000272505	ENSG00000272505	8	10486807	10489666	lincRNA	22,40	0
ENSG00000248664	ENSG00000248664	5	69113112	69136394	antisense	22,39	0
ENSG00000278769	ENSG00000278769	15	42724102	42724922	antisense	22,38	0
ENSG00000255449	ENSG00000255449	11	77866412	77870091	antisense	22,36	0
ENSG00000279210	ENSG00000279210	1	178479247	178482365	antisense	22,31	0
ENSG00000257896	ENSG00000257896	12	43736628	43741067	transcribed_unprocessed_pseudogene	22,30	0
ENSG00000273329	ENSG00000273329	7	129604548	129611630	lincRNA	22,29	0

ENSG00000239689	RPL17P46	18	50823577	50824120	processed_pseudogene	22,20	0
ENSG00000267233	HNRNPA3P16	18	50814392	50815730	processed_pseudogene	22,20	0
ENSG00000259750	ENSG00000259750	15	59401221	59402004	processed_pseudogene	22,20	0,2161218
ENSG00000278126	ENSG00000278126	12	51201684	51202581	sense_intronic	22,20	0
ENSG00000269153	LYPLA2P2	19	7879445	7880120	processed_pseudogene	22,16	0
ENSG00000278530	CHMP1B2P	X	80228489	80335364	unitary_pseudogene	22,15	0
ENSG00000239801	DENND6A-AS1	3	57628810	57654918	antisense	22,13	0
ENSG00000242659	ENSG00000242659	3	113746872	113747408	lincRNA	22,10	0
ENSG00000224072	ENSG00000224072	X	46454097	46457199	processed_pseudogene	22,10	1,40E-02
ENSG00000259079	ENSG00000259079	14	71330342	71330738	processed_pseudogene	22,08	0
ENSG00000273262	ENSG00000273262	10	102483039	102483559	antisense	22,03	0
ENSG00000235095	ENSG00000235095	7	57147986	57150347	unprocessed_pseudogene	22,01	0
ENSG00000235374	SSR4P1	21	45070952	45074165	transcribed_processed_pseudogene	22,00	0
ENSG00000204850	ENSG00000204850	19	46494508	46496502	antisense	22,00	0
ENSG00000268573	ENSG00000268573	18	35280867	35290201	lincRNA	22,00	0
ENSG00000230325	ENSG00000230325	1	236540094	236550280	antisense	22,00	0
ENSG00000225610	ENSG00000225610	2	205989585	206000858	antisense	21,94	0
ENSG00000272836	ENSG00000272836	22	50205585	50206062	antisense	21,90	0
ENSG00000272849	ENSG00000272849	12	121797511	121801972	lincRNA	21,90	0
ENSG00000238109	ENSG00000238109	7	98998538	98999930	processed_pseudogene	21,90	1,40E-02
ENSG00000271862	ENSG00000271862	5	83049376	83050964	lincRNA	21,90	0
ENSG00000229939	ENSG00000229939	X	111618808	111619094	processed_pseudogene	21,90	0
ENSG00000174353	STAG3L3	7	72969696	73005922	transcribed_unprocessed_pseudogene	21,89	0
ENSG00000226803	ENSG00000226803	6	57114894	57174236	antisense	21,87	0
ENSG00000235354	ENSG00000235354	7	103348601	103348771	processed_pseudogene	21,87	3,30E-03
ENSG00000267156	TPMTP1	18	47630112	47630848	processed_pseudogene	21,87	0
ENSG00000259367	ENSG00000259367	15	85619623	85670948	antisense	21,80	0
ENSG00000171889	MIR31HG	9	21455642	21559669	sense_overlapping	21,80	0
ENSG00000271913	ENSG00000271913	6	158988178	159064925	antisense	21,80	0
ENSG00000271895	ENSG00000271895	1	11029659	11030528	antisense	21,80	0
ENSG00000269019	ENSG00000269019	19	18940322	18946831	antisense	21,70	0
ENSG00000221844	DPP3P2	9	73474378	73476073	processed_pseudogene	21,70	0,2161218
ENSG00000215270	ENSG00000215270	22	15854195	15855243	processed_pseudogene	21,60	0
ENSG00000260425	ENSG00000260425	16	1358900	1361405	antisense	21,60	0
ENSG00000259327	ENSG00000259327	15	52116574	52122131	lincRNA	21,60	0
ENSG00000204188	GGNBP1	6	33540046	33589026	unitary_pseudogene	21,60	0
ENSG00000240194	CYMP	1	110480752	110491277	unitary_pseudogene	21,60	7,06E-02
ENSG00000241720	ENSG00000241720	1	109725820	109775252	antisense	21,59	0
ENSG00000223390	ENSG00000223390	1	52033391	52044279	antisense	21,59	0
ENSG00000251204	ENSG00000251204	5	106415576	106417241	processed_pseudogene	21,58	0
ENSG00000238041	ENSG00000238041	11	56690465	56690723	processed_pseudogene	21,57	0
ENSG00000245694	CRNDE	16	54918863	54929189	lincRNA	21,52	0
ENSG00000268093	ENSG00000268093	19	48619272	48624132	antisense	21,50	0
ENSG00000236539	HNRNPA1P54	1	179447602	179457315	processed_pseudogene	21,50	0,2161218
ENSG00000271182	ENSG00000271182	19	21634833	21635175	processed_pseudogene	21,46	0
ENSG00000213090	ENSG00000213090	2	201410544	201413308	processed_pseudogene	21,46	0
ENSG00000213260	YWHAZP5	10	105686322	105687051	processed_pseudogene	21,45	0
ENSG00000215067	ALOX12-AS1	17	6876635	7012349	antisense	21,44	0
ENSG00000246851	ENSG00000246851	9	131189910	131194205	antisense	21,44	0
ENSG00000229766	ENSG00000229766	20	8019223	8043512	antisense	21,40	0
ENSG00000258457	ENSG00000258457	14	22982698	22999078	antisense	21,40	0
ENSG00000258682	ENSG00000258682	14	58264662	58269681	sense_overlapping	21,40	0
ENSG00000245498	ENSG00000245498	11	124800450	124834487	antisense	21,38	0
ENSG00000261069	ENSG00000261069	15	25087661	25088896	lincRNA	21,37	0
ENSG00000223697	ENSG00000223697	8	132838117	132844298	antisense	21,34	0
ENSG00000213962	API5P2	2	177997273	177998770	processed_pseudogene	21,33	0
ENSG00000263812	LINC00908	18	76528655	76610968	lincRNA	21,30	0
ENSG00000261924	ENSG00000261924	17	80966239	80971213	antisense	21,27	6,27E-03

ENSG00000197815	ENSG00000197815	17	17858227	17860041	antisense	21,20	0
ENSG00000271105	SCML2P2	16	25069570	25070109	processed_pseudogene	21,20	0
ENSG00000234432	ENSG00000234432	7	5426277	5428927	lincRNA	21,20	0
ENSG00000212694	LINC01089	12	121795267	121803906	lincRNA	21,19	0
ENSG00000260401	ENSG00000260401	11	73238975	73242335	sense_overlapping	21,17	0
ENSG00000275854	ENSG00000275854	12	32736930	32737660	sense_intronic	21,13	0
ENSG00000177335	C8orf31	8	143039209	143059942	processed_transcript	21,13	0
ENSG00000256806	C17orf100	17	6651718	6693202	lincRNA	21,12	0
ENSG00000277496	ENSG00000277496	20	62648961	62650767	antisense	21,10	0
ENSG00000242615	ENSG00000242615	19	12141806	12143322	transcribed_processed_pseudogene	21,10	0
ENSG00000244198	ENSG00000244198	7	144194858	144280547	antisense	21,10	0
ENSG00000224318	CHL1-AS2	3	195758	197341	processed_transcript	21,10	0
ENSG00000228925	ENSG00000228925	2	46899275	46908678	antisense	21,07	0
ENSG00000268056	ENSG00000268056	19	17152588	17168051	antisense	21,05	0
ENSG00000252690	SCARNA15	15	82752884	82757208	processed_transcript	21,00	0
ENSG00000259696	ENSG00000259696	15	31453201	31454139	processed_pseudogene	21,00	0
ENSG00000230953	ENSG00000230953	1	52920422	52920596	processed_pseudogene	21,00	0
ENSG00000233937	ENSG00000233937	5	181246523	181272167	processed_transcript	20,95	0
ENSG00000260114	ENSG00000260114	16	29919634	29921905	sense_intronic	20,93	0
ENSG00000265784	ENSG00000265784	17	38918801	38921769	antisense	20,91	0
ENSG00000265487	ENSG00000265487	18	5956101	5960785	antisense	20,90	0
ENSG00000261542	ENSG00000261542	8	63215981	63218034	lincRNA	20,83	0
ENSG00000213430	HSPD1P1	5	21882585	21884310	processed_pseudogene	20,83	0
ENSG00000230590	FTX	X	73963955	74293574	lincRNA	20,82	0
ENSG00000266501	ENSG00000266501	17	73067870	73068288	processed_pseudogene	20,81	0
ENSG00000234567	ENSG00000234567	6	133452857	133456605	antisense	20,80	0
ENSG00000267919	ENSG00000267919	2	219645090	219645631	lincRNA	20,80	0
ENSG00000226711	FAM66C	12	8180209	8216151	antisense	20,77	0
ENSG00000242622	ENSG00000242622	3	120094895	120136783	lincRNA	20,73	0
ENSG00000255198	SNHG9	16	1964959	1965509	lincRNA	20,71	3,30E-03
ENSG00000273300	ENSG00000273300	22	19454179	19454605	antisense	20,70	0
ENSG00000269890	ENSG00000269890	1	228270443	228274397	antisense	20,70	0
ENSG00000229358	DPY19L1P1	7	32580949	32761787	unprocessed_pseudogene	20,70	0
ENSG00000247570	SDCBPP2	8	69942948	69944648	processed_pseudogene	20,67	0
ENSG00000228819	ENSG00000228819	X	132130091	132131352	processed_pseudogene	20,67	0,3318209
ENSG00000224413	ENSG00000224413	21	46037052	46039807	lincRNA	20,60	0
ENSG00000267727	ENSG00000267727	19	33301279	33301940	antisense	20,60	0
ENSG00000272240	ENSG00000272240	8	234347	234887	antisense	20,60	0
ENSG00000233221	ENSG00000233221	2	130830978	130836994	antisense	20,60	0
ENSG00000237206	IMPDH1P4	X	43278125	43279658	processed_pseudogene	20,60	0
ENSG00000227176	ENSG00000227176	2	170077224	170078294	processed_pseudogene	20,56	0
ENSG00000253213	ENSG00000253213	5	171251866	171252982	processed_pseudogene	20,50	0
ENSG00000244932	ENSG00000244932	3	129381298	129394149	unprocessed_pseudogene	20,50	0
ENSG00000228335	ENSG00000228335	7	99442890	99443496	processed_pseudogene	20,49	0
ENSG00000228717	ENSG00000228717	X	151226229	151227715	processed_pseudogene	20,43	0
ENSG00000239665	ENSG00000239665	10	13631143	13668445	processed_transcript	20,42	0
ENSG00000271020	ENSG00000271020	3	33797149	33797681	lincRNA	20,42	0
ENSG00000227252	ENSG00000227252	2	237059434	237085817	antisense	20,40	0
ENSG00000230415	ENSG00000230415	1	1275223	1280420	lincRNA	20,40	0
ENSG00000256073	URB1-AS1	21	32393130	32393960	lincRNA	20,39	0
ENSG00000231549	USMG5P1	X	74173890	74174066	processed_pseudogene	20,39	0
ENSG00000225791	TRAM2-AS1	6	52577307	52583993	lincRNA	20,36	0
ENSG00000260236	ENSG00000260236	3	47379089	47380999	antisense	20,36	0
ENSG00000281205	LINC00950	9	35858738	35865518	lincRNA	20,31	0
ENSG00000269051	ENSG00000269051	19	53197111	53211015	lincRNA	20,30	0
ENSG00000261208	ENSG00000261208	14	52286797	52289268	processed_pseudogene	20,30	7,06E-02
ENSG00000254595	ENSG00000254595	11	6488186	6489377	processed_pseudogene	20,30	0

ENSG00000227775	ENSG00000227775	1	1724512	1737251	transcribed_processed_pseudogene	20,22	0
ENSG00000261438	ENSG00000261438	10	89015836	89017059	sense_overlapping	20,20	0
ENSG00000250846	EPHA5-AS1	4	65669961	65698029	lincRNA	20,20	0
ENSG00000224884	ENSG00000224884	3	8573726	8593124	lincRNA	20,20	0
ENSG00000259683	ENSG00000259683	15	84389729	84395903	unprocessed_pseudogene	20,15	0
ENSG00000253785	ENSG00000253785	5	172975511	172976374	processed_pseudogene	20,12	0
ENSG00000272733	ENSG00000272733	22	23580880	23583859	lincRNA	20,10	0
ENSG00000240970	RPL23AP64	11	119003012	119003446	processed_pseudogene	20,10	0
ENSG00000247416	ENSG00000247416	11	112959279	112963460	antisense	20,10	0
ENSG00000261051	ENSG00000261051	3	146059585	146061679	sense_overlapping	20,10	0
ENSG00000227295	ELL2P1	1	158175850	158177755	processed_pseudogene	20,03	0
ENSG00000268203	ENSG00000268203	19	6494320	6494805	antisense	20,00	0
ENSG00000181227	ENSG00000181227	1	75743423	75744776	processed_pseudogene	19,96	0
ENSG00000188512	ENSG00000188512	8	33969567	33969879	processed_pseudogene	19,93	0
ENSG00000213985	ENSG00000213985	19	20257720	20259418	processed_pseudogene	19,92	0
ENSG00000270060	ENSG00000270060	11	47168281	47169563	sense_intronic	19,90	0
ENSG00000250569	NTAN1P2	8	86481754	86483002	processed_pseudogene	19,90	0
ENSG00000258365	ENSG00000258365	12	94277758	94282844	antisense	19,82	0
ENSG00000259562	ENSG00000259562	15	78290527	78291221	transcribed_processed_pseudogene	19,80	0
ENSG00000225333	ENSG00000225333	1	39718028	39718595	processed_pseudogene	19,80	0,2161218
ENSG00000276136	ENSG00000276136	12	32000375	32001222	lincRNA	19,70	0
ENSG00000270441	ENSG00000270441	3	49140086	49160851	unprocessed_pseudogene	19,69	0
ENSG00000258359	PCNPP1	12	111669852	111670362	processed_pseudogene	19,69	0,1392851
ENSG00000211451	GNRHR2	1	145919013	145925341	unitary_pseudogene	19,67	0
ENSG00000257497	ENSG00000257497	12	75483454	75489820	antisense	19,64	0
ENSG00000236216	PPP1R11P1	1	21397987	21398362	processed_pseudogene	19,64	0
ENSG00000267342	ENSG00000267342	17	75897060	75900148	antisense	19,61	0
ENSG00000249740	OSMR-AS1	5	38736055	38845829	lincRNA	19,60	0
ENSG00000224728	ENSG00000224728	3	15878047	15879571	processed_pseudogene	19,60	0
ENSG00000231365	ENSG00000231365	1	119140396	119275973	antisense	19,55	0
ENSG00000257674	ENSG00000257674	12	42286911	42288560	processed_pseudogene	19,54	0
ENSG00000247317	ENSG00000247317	8	142981738	143018437	lincRNA	19,53	0
ENSG00000214425	LRRC37A4P	17	45506741	45550335	transcribed_unprocessed_pseudogene	19,50	0
ENSG00000260381	ENSG00000260381	16	50100339	50121943	antisense	19,50	0
ENSG00000259052	ENSG00000259052	14	100279959	100291456	sense_overlapping	19,50	0
ENSG00000256075	ENSG00000256075	12	68473741	68474902	processed_pseudogene	19,50	0
ENSG00000250959	GLUD1P3	10	73730562	73737311	transcribed_unprocessed_pseudogene	19,50	0
ENSG00000224025	ENSG00000224025	9	68609125	68611984	processed_pseudogene	19,50	0
ENSG00000280543	ASAP1-IT2	8	130082738	130084768	sense_intronic	19,50	0
ENSG00000234123	RHBDF1P1	3	14572852	14574792	processed_pseudogene	19,50	1,40E-02
ENSG00000121089	NACA3P	4	164943290	164943937	processed_pseudogene	19,49	0
ENSG00000229091	HSPA8P8	7	10451311	10453252	processed_pseudogene	19,48	0
ENSG00000230756	RHOQP3	2	130212870	130213490	processed_pseudogene	19,47	0
ENSG00000250909	ENSG00000250909	5	176347941	176353584	antisense	19,46	0
ENSG00000234500	ENSG00000234500	7	66511556	66545066	unprocessed_pseudogene	19,45	0
ENSG00000234084	ENSG00000234084	6	135301568	135307158	antisense	19,45	0
ENSG00000251032	CUL1P1	5	127784618	127787042	processed_pseudogene	19,43	0
ENSG00000213344	PCNPP3	12	65645992	65646462	processed_pseudogene	19,40	7,06E-02
ENSG00000272871	ENSG00000272871	9	14588797	14590065	lincRNA	19,40	0
ENSG00000214846	ENSG00000214846	4	15730962	15731627	processed_pseudogene	19,40	7,06E-02
ENSG00000240524	ENSG00000240524	1	229270927	229271599	transcribed_processed_pseudogene	19,38	0
ENSG00000260617	ENSG00000260617	16	88741631	88742367	antisense	19,33	0
ENSG00000251867	ENSG00000251867	8	80484561	80485619	antisense	19,32	0

ENSG00000229689	ENSG00000229689	2	95525345	95532405	transcribed_unprocessed_pseudogene	19,31	0
ENSG00000234892	ENSG00000234892	22	43232141	43239010	antisense	19,30	0
ENSG00000269399	ENSG00000269399	19	16542746	16544814	lincRNA	19,30	0
ENSG00000273447	ENSG00000273447	4	109692004	109692703	antisense	19,30	0
ENSG00000231327	ENSG00000231327	2	70124036	70125317	lincRNA	19,25	0
ENSG00000224186	C5orf66	5	135033280	135358219	antisense	19,25	0
ENSG00000265840	ENSG00000265840	17	28749731	28750079	antisense	19,23	0
ENSG00000265417	ENSG00000265417	18	1362445	1363873	processed_pseudogene	19,22	0
ENSG00000262413	ENSG00000262413	17	81867721	81868552	antisense	19,21	0
ENSG00000256433	ENSG00000256433	12	6393905	6396148	lincRNA	19,20	0
ENSG00000233237	LINC00472	6	71344344	71420769	lincRNA	19,20	0
ENSG00000274877	ENSG00000274877	2	113237595	113240825	antisense	19,20	0
ENSG00000180284	ENSG00000180284	X	103651735	103652106	processed_pseudogene	19,20	0
ENSG00000259661	ENSG00000259661	15	90920218	90921186	antisense	19,13	0
ENSG00000233087	ENSG00000233087	2	131362956	131363720	transcribed_processed_pseudogene	19,11	0
ENSG00000258891	ENSG00000258891	14	73896164	73938114	antisense	19,11	0
ENSG00000272072	ENSG00000272072	7	107192559	107193300	antisense	19,10	0
ENSG00000251414	ENSG00000251414	5	176354206	176356168	antisense	19,10	0
ENSG00000224272	ENSG00000224272	2	241808312	241812016	antisense	19,10	0
ENSG00000236364	ENSG00000236364	1	165890795	165900683	antisense	19,06	0
ENSG00000205771	CATSPER2P1	15	43726918	43747094	transcribed_unprocessed_pseudogene	19,04	0
ENSG00000269481	ENSG00000269481	19	17414257	17422324	antisense	19,00	0
ENSG00000226890	ENSG00000226890	16	1625628	1626160	antisense	19,00	0
ENSG00000271815	ENSG00000271815	5	75363760	75364242	lincRNA	19,00	0
ENSG00000250325	IGBP1P4	4	82401578	82402456	processed_pseudogene	19,00	1,40E-02
ENSG00000237940	ENSG00000237940	2	241970683	241977276	lincRNA	19,00	0
ENSG00000230896	ENSG00000230896	1	45694684	45697075	sense_intronic	19,00	0
ENSG00000234072	ENSG00000234072	2	27356246	27367622	antisense	18,98	0
ENSG00000272267	ENSG00000272267	8	9555144	9556520	antisense	18,96	0
ENSG00000223984	HNRNPRP1	10	21624592	21626286	processed_pseudogene	18,95	0
ENSG00000225355	ARL6IP1P2	10	36995023	36995585	processed_pseudogene	18,92	0
ENSG00000217414	ENSG00000217414	6	18363417	18365423	processed_pseudogene	18,91	0
ENSG00000226143	ENSG00000226143	20	43549389	43550949	antisense	18,90	0
ENSG00000256164	CCND2-AS1	12	4248765	4276184	antisense	18,90	0
ENSG00000256673	ENSG00000256673	12	9398355	9414851	unprocessed_pseudogene	18,90	0
ENSG00000231655	ENSG00000231655	2	26140263	26141264	processed_pseudogene	18,90	1,40E-02
ENSG00000185031	SLC2A3P2	1	64984608	64986087	processed_pseudogene	18,90	0
ENSG00000227496	ENSG00000227496	1	225700604	225752243	antisense	18,90	0
ENSG00000270127	ENSG00000270127	15	100547765	100550153	lincRNA	18,82	0
ENSG00000259668	ENSG00000259668	15	51457286	51460582	antisense	18,81	0
ENSG00000256704	SDCCAG3P1	18	60009994	60011143	processed_pseudogene	18,80	0,2161218
ENSG00000261067	ENSG00000261067	16	28974804	28990783	processed_transcript	18,80	0
ENSG00000236548	RNF217-AS1	6	124909093	124963039	antisense	18,80	0
ENSG00000258451	ENSG00000258451	14	20693480	20707120	antisense	18,79	0
ENSG00000267253	ENSG00000267253	17	43718314	43720436	processed_pseudogene	18,79	0
ENSG00000254422	ENSG00000254422	11	102229851	102230922	antisense	18,78	0
ENSG00000270055	ENSG00000270055	15	30487963	30490313	sense_intronic	18,77	0
ENSG00000255035	SDHCP4	11	17435672	17436181	processed_pseudogene	18,76	0
ENSG00000246203	ENSG00000246203	1	155614726	155660245	unprocessed_pseudogene	18,75	6,27E-03
ENSG00000228265	RALY-AS1	20	33983052	33994357	lincRNA	18,70	0
ENSG00000267102	ENSG00000267102	17	35409602	35410228	transcribed_processed_pseudogene	18,70	0
ENSG00000258926	ENSG00000258926	14	61537508	61545287	antisense	18,70	0
ENSG00000273448	ENSG00000273448	7	67333047	67334383	lincRNA	18,70	0
ENSG00000223804	ENSG00000223804	1	120267334	120341871	transcribed_unprocessed_pseudogene	18,70	0

ENSG00000215070	XRCC6P5	X	99719364	99721158	processed_pseudogene	18,70	7,06E-02
ENSG00000256238	SUPT16HP1	12	16399274	16402406	processed_pseudogene	18,69	0
ENSG00000206573	THUMPD3-AS1	3	9349689	9398579	antisense	18,66	0
ENSG00000278811	LINC00624	1	147258885	147517875	antisense	18,65	0
ENSG00000251143	ENSG00000251143	11	72014291	72020910	antisense	18,64	0
ENSG00000236739	CLIC4P1	9	22747700	22748234	processed_pseudogene	18,64	0
ENSG00000271676	ENSG00000271676	4	77112495	77113458	processed_pseudogene	18,63	0
ENSG00000263990	ENSG00000263990	17	31873926	31886666	lincRNA	18,60	0
ENSG00000263126	ENSG00000263126	16	67882461	67886367	lincRNA	18,60	0
ENSG00000258667	HIF1A-AS2	14	61715558	61751097	lincRNA	18,60	0
ENSG00000230982	DSTNP1	21	46653558	46654022	processed_pseudogene	18,50	0
ENSG00000259826	ENSG00000259826	7	39947522	39949755	antisense	18,50	0
ENSG00000267272	LINC01140	1	87129765	87169198	lincRNA	18,50	0
ENSG00000230409	TCEA1P2	3	37275693	37276598	processed_pseudogene	18,49	0
ENSG00000228507	DAP3P2	2	171491422	171491931	processed_pseudogene	18,47	0
ENSG00000248008	NRAV	12	120490328	120495940	antisense	18,41	0
ENSG00000224397	LINC01272	20	50267486	50279795	lincRNA	18,40	0
ENSG00000230555	ENSG00000230555	10	43420738	43422100	lincRNA	18,40	0
ENSG00000253854	ENSG00000253854	8	93719574	93721167	antisense	18,40	0
ENSG00000225264	ZNRF2P2	7	29598795	29685255	transcribed_processed_pseudogene	18,40	0
ENSG00000235407	ENSG00000235407	1	110487680	110490258	lincRNA	18,40	7,06E-02
ENSG00000197358	BNIP3P1	14	28264390	28265974	transcribed_processed_pseudogene	18,39	0
ENSG00000228677	TTC3-AS1	21	37187666	37193926	antisense	18,36	0
ENSG00000260773	ENSG00000260773	15	66314914	66331703	antisense	18,36	0
ENSG00000246982	ENSG00000246982	6	36146698	36197205	antisense	18,36	0
ENSG00000224831	ENSG00000224831	3	149982181	149983308	processed_pseudogene	18,32	0
ENSG00000265413	ENSG00000265413	18	8402847	8405161	sense_intronic	18,30	0
ENSG00000261485	PAN3-AS1	13	28136843	28138193	antisense	18,30	0
ENSG00000229106	BTBD6P1	1	23901471	23902737	processed_pseudogene	18,30	0
ENSG00000241886	ENSG00000241886	X	30698207	30721932	sense_intronic	18,30	0
ENSG00000247033	ENSG00000247033	16	75108601	75110712	antisense	18,26	0
ENSG00000246174	KCTD21-AS1	11	78139771	78175323	antisense	18,26	0
ENSG00000185839	ENSG00000185839	1	58630841	58631530	processed_pseudogene	18,23	0
ENSG00000213144	ENSG00000213144	12	119194850	119195394	processed_pseudogene	18,23	0
ENSG00000267603	LINC01028	17	70051277	70068095	lincRNA	18,20	0
ENSG00000259118	ENSG00000259118	14	65082034	65094212	antisense	18,17	0
ENSG00000223705	NSUN5P1	7	75410322	75416787	transcribed_unprocessed_pseudogene	18,16	0
ENSG00000270236	ENSG00000270236	9	69107926	69108217	processed_pseudogene	18,10	0
ENSG00000258666	ENSG00000258666	14	100333790	100354061	antisense	18,06	0
ENSG00000177197	PCNPP5	13	48328084	48328564	processed_pseudogene	18,01	0
ENSG00000275371	ENSG00000275371	16	30110895	30111955	antisense	18,00	0
ENSG00000260898	ADPGK-AS1	15	72782835	72798199	antisense	18,00	0
ENSG00000230408	ENSG00000230408	2	200780495	200812170	antisense	17,99	0
ENSG00000236256	DIAPH2-AS1	X	97431286	97642589	antisense	17,93	0
ENSG00000186019	ENSG00000186019	19	44105463	44113145	antisense	17,91	0
ENSG00000267121	ENSG00000267121	17	45190931	45222222	antisense	17,90	0
ENSG00000273084	ENSG00000273084	7	5428731	5429672	lincRNA	17,90	0
ENSG00000270890	ENSG00000270890	6	143858062	143858689	processed_pseudogene	17,90	0
ENSG00000232633	ENSG00000232633	5	113323028	113437174	antisense	17,90	0
ENSG00000227709	ENSG00000227709	3	156175852	156176911	processed_pseudogene	17,88	0
ENSG00000197180	ENSG00000197180	X	154424380	154428479	lincRNA	17,85	0
ENSG00000225053	PRPF38AP1	10	8161260	8162192	processed_pseudogene	17,83	6,27E-03
ENSG00000263823	ENSG00000263823	18	31942575	31944156	antisense	17,80	0
ENSG00000261220	ENSG00000261220	8	133573183	133573861	lincRNA	17,80	0
ENSG00000225705	ENSG00000225705	7	48846426	48852898	processed_pseudogene	17,80	0
ENSG00000232665	PHBP10	X	104647216	104647999	processed_pseudogene	17,80	0
ENSG00000241478	HSPA8P9	3	137880295	137882237	processed_pseudogene	17,80	0
ENSG00000266708	ENSG00000266708	18	8635179	8636347	antisense	17,76	0

ENSG00000253102	ENSG00000253102	17	50396438	50397888	antisense	17,74	0
ENSG00000230267	HERC2P4	16	32103245	32188107	transcribed_unprocessed_pseudogene	17,72	0
ENSG00000159860	TCAF2P1	7	143800732	143817973	unprocessed_pseudogene	17,67	0
ENSG00000227513	ENSG00000227513	2	106344566	106345433	processed_pseudogene	17,67	0,3318209
ENSG00000179899	PHC1P1	12	55411727	55414787	processed_pseudogene	17,62	0
ENSG00000181101	SDCCAG3P2	1	175044626	175045648	processed_pseudogene	17,61	0
ENSG00000227401	RPL37P1	20	35588324	35588607	processed_pseudogene	17,60	0,2161218
ENSG00000240211	ENSG00000240211	7	100436204	100438504	antisense	17,60	2,21E-02
ENSG00000162997	PRORSD1P	2	55282319	55284522	unitary_pseudogene	17,60	0
ENSG00000269068	ENSG00000269068	2	219559083	219559626	antisense	17,60	0
ENSG00000273033	ENSG00000273033	3	122886941	122892416	lincRNA	17,60	0
ENSG00000253106	ENSG00000253106	8	124488510	124491643	antisense	17,58	0
ENSG00000227495	ENSG00000227495	17	5019214	5020093	antisense	17,57	0
ENSG00000270177	ENSG00000270177	5	134226410	134227827	lincRNA	17,56	0
ENSG00000226098	SEC11B	8	54522799	54523297	processed_pseudogene	17,54	0
ENSG00000242756	RHOT1P3	13	18837768	18838098	processed_pseudogene	17,52	0
ENSG00000263235	ENSG00000263235	16	3650636	3651703	sense_overlapping	17,50	0
ENSG00000270304	ENSG00000270304	15	37253879	37254952	processed_pseudogene	17,50	0
ENSG00000238271	IFNWP19	9	21455484	21456049	unprocessed_pseudogene	17,50	0
ENSG00000267598	ENSG00000267598	19	13153071	13154193	antisense	17,48	0
ENSG00000265205	ENSG00000265205	17	28670054	28672804	antisense	17,40	0
ENSG00000240399	ENSG00000240399	12	48054813	48055591	transcribed_processed_pseudogene	17,40	1,40E-02
ENSG00000231351	ENSG00000231351	2	86930250	86930754	processed_pseudogene	17,40	0
ENSG00000273306	ENSG00000273306	2	99405218	99405843	antisense	17,40	0
ENSG00000223353	ENSG00000223353	1	245123471	245124450	processed_pseudogene	17,40	0
ENSG00000224217	ENSG00000224217	X	116748530	116748877	processed_pseudogene	17,40	0
ENSG00000281450	PANDAR	6	36673621	36675126	lincRNA	17,37	0
ENSG00000229419	RALGAPA1P	9	105520128	105526359	processed_pseudogene	17,32	0
ENSG00000250483	PPM1AP1	8	15806149	15807283	processed_pseudogene	17,30	0
ENSG00000243155	ENSG00000243155	1	180944042	180976482	antisense	17,30	0
ENSG00000261063	ENSG00000261063	16	77201474	77249957	antisense	17,30	0
ENSG00000227939	RPL3P2	6	31280317	31281519	processed_pseudogene	17,28	0
ENSG00000267565	ENSG00000267565	19	19892433	19895847	antisense	17,27	0
ENSG00000254129	ENSG00000254129	8	29110573	29140729	antisense	17,27	0
ENSG00000249348	UGDH-AS1	4	39528019	39594707	antisense	17,27	0
ENSG00000249087	ZNF436-AS1	1	23368997	23371839	antisense	17,22	0
ENSG00000267787	ENSG00000267787	18	57639455	57738044	antisense	17,20	0
ENSG00000273018	ENSG00000273018	17	18511221	18551705	processed_transcript	17,20	0
ENSG00000257346	ENSG00000257346	12	49090208	49093312	antisense	17,20	0
ENSG00000257410	ENSG00000257410	12	71793855	71799627	lincRNA	17,20	1,40E-02
ENSG00000228863	ENSG00000228863	1	160670778	160699761	antisense	17,20	0
ENSG00000196295	ENSG00000196295	7	30516309	30594809	processed_transcript	17,20	0
ENSG00000261552	ENSG00000261552	16	28989140	28990778	antisense	17,18	0,1392851
ENSG00000247373	ENSG00000247373	12	123575891	123585115	lincRNA	17,18	0
ENSG00000244380	ENSG00000244380	3	48440352	48446656	antisense	17,17	0
ENSG00000267336	EIF4A2P1	18	12912844	12913906	processed_pseudogene	17,15	4,86E-02
ENSG00000231563	ENSG00000231563	1	228407381	228409694	antisense	17,14	0
ENSG00000251432	ENSG00000251432	4	128292751	128519394	lincRNA	17,14	0
ENSG00000225031	EIF4BP7	X	111619677	111621489	processed_pseudogene	17,14	0
ENSG00000234337	ENSG00000234337	16	52655307	52656045	processed_pseudogene	17,12	0
ENSG00000264578	ENSG00000264578	8	41609692	41621502	antisense	17,11	0
ENSG00000274092	ENSG00000274092	16	27313387	27314101	antisense	17,10	0
ENSG00000271072	PHBP20	15	72450657	72451474	processed_pseudogene	17,10	0,2161218
ENSG00000215483	LINC00598	13	40450934	40535807	lincRNA	17,10	0
ENSG00000255197	ENSG00000255197	11	47383148	47409190	antisense	17,10	0
ENSG00000242861	ENSG00000242861	1	225840883	225846522	antisense	17,09	0
ENSG00000234648	ENSG00000234648	14	98973314	98973471	processed_pseudogene	17,03	0

ENSG00000223723	ENSG00000223723	X	36962196	36963425	processed_pseudogene	17,02	0
ENSG00000267232	ENSG00000267232	19	1875016	1875992	lincRNA	17,00	0
ENSG00000230113	ENSG00000230113	17	30956280	30956961	antisense	17,00	0
ENSG00000257283	ENSG00000257283	12	93894965	93943603	antisense	17,00	0
ENSG00000234825	XRCC6P2	X	150231075	150232896	processed_pseudogene	16,99	0
ENSG00000214765	SEPT7P2	7	45723780	45768985	transcribed_unprocessed_pseudogene	16,99	0
ENSG00000188693	CYP51A1-AS1	7	92134604	92180725	antisense	16,94	0
ENSG00000203469	ENSG00000203469	1	10458555	10459338	antisense	16,94	0
ENSG00000237350	CDC42P6	4	22727375	22727950	processed_pseudogene	16,94	0
ENSG00000256616	ENSG00000256616	18	12073232	12076654	transcribed_processed_pseudogene	16,91	0
ENSG00000226314	ZNF192P1	6	28161781	28169594	transcribed_unprocessed_pseudogene	16,91	0
ENSG00000269843	ENSG00000269843	19	40831221	40837210	lincRNA	16,90	0
ENSG00000231084	ENSG00000231084	1	185171335	185171710	processed_pseudogene	16,82	0
ENSG00000267632	ENSG00000267632	17	42509784	42511519	sense_intronic	16,80	0
ENSG00000272170	ENSG00000272170	6	43074331	43074739	antisense	16,80	0
ENSG00000225769	CROCCP1	X	135432960	135438634	processed_pseudogene	16,80	1,40E-02
ENSG00000234213	FHP1	13	42255873	42256575	processed_pseudogene	16,74	0
ENSG00000188599	NPIPP1	16	15104312	15123498	transcribed_unprocessed_pseudogene	16,74	0
ENSG00000232855	ENSG00000232855	21	28439346	28674848	lincRNA	16,70	0
ENSG00000268970	ENSG00000268970	19	52597699	52598887	sense_intronic	16,70	0
ENSG00000221949	LINC01465	12	62601751	62603690	lincRNA	16,70	0
ENSG00000256377	ENSG00000256377	12	27696388	27710770	antisense	16,70	0
ENSG00000227344	HAUS6P1	7	53187388	53188938	processed_pseudogene	16,64	0
ENSG00000261093	ENSG00000261093	16	2597881	2599718	antisense	16,63	0
ENSG00000225486	ENSG00000225486	1	233836080	233836432	processed_pseudogene	16,63	0
ENSG00000228089	PNKDP1	X	131803312	131803741	processed_pseudogene	16,62	0
ENSG00000278869	ENSG00000278869	22	49933198	49934074	lincRNA	16,60	0
ENSG00000268049	ENSG00000268049	19	58357999	58359603	antisense	16,60	0
ENSG00000224825	RORB-AS1	9	74485551	74499127	antisense	16,60	0
ENSG00000236018	ENSG00000236018	7	157197600	157198511	processed_pseudogene	16,60	0
ENSG00000279467	ENSG00000279467	22	23865248	23873277	antisense	16,58	0
ENSG00000219201	ENSG00000219201	1	77810861	77811781	processed_pseudogene	16,57	0
ENSG00000254294	IMPDH1P6	8	123400582	123402095	processed_pseudogene	16,56	0
ENSG00000233175	ENSG00000233175	17	45238028	45241734	antisense	16,53	0
ENSG00000232973	CYP1B1-AS1	2	38073447	38231651	antisense	16,51	0
ENSG00000224950	ENSG00000224950	1	116493016	116499212	lincRNA	16,50	0
ENSG00000263781	ENSG00000263781	17	29021325	29021640	processed_pseudogene	16,48	0
ENSG00000272906	ENSG00000272906	1	179881607	179882595	lincRNA	16,46	0
ENSG00000268230	ENSG00000268230	19	58346854	58362751	processed_transcript	16,42	0
ENSG00000249868	ENSG00000249868	8	311133	331026	lincRNA	16,42	0,1836819
ENSG00000234703	ENSG00000234703	21	35136638	35139222	lincRNA	16,40	0
ENSG00000263271	ENSG00000263271	17	81461013	81461937	antisense	16,40	0
ENSG00000245105	A2M-AS1	12	9065177	9068060	antisense	16,40	0
ENSG00000250770	ENSG00000250770	12	3296202	3300224	lincRNA	16,40	1,40E-02
ENSG00000256030	CBX3P4	12	2786931	2794295	transcribed_processed_pseudogene	16,40	7,06E-02
ENSG00000276115	ENSG00000276115	12	32352349	32354144	sense_intronic	16,40	0
ENSG00000270067	ENSG00000270067	5	112893333	112894001	antisense	16,40	0
ENSG00000249685	ENSG00000249685	4	39133913	39135608	lincRNA	16,40	0
ENSG00000250318	ENSG00000250318	22	30653877	30654814	unprocessed_pseudogene	16,38	0
ENSG00000228366	ENSG00000228366	9	105710290	105710781	processed_pseudogene	16,35	0
ENSG00000254907	ENSG00000254907	11	43328748	43359296	antisense	16,31	0
ENSG00000259943	ENSG00000259943	1	40256427	40257967	antisense	16,31	0
ENSG00000254909	ENSG00000254909	11	119005727	119005934	processed_pseudogene	16,30	0
ENSG00000273521	ENSG00000273521	10	131980240	131981337	antisense	16,30	0
ENSG00000228653	HNRNPCP7	7	64500825	64501729	processed_pseudogene	16,30	0

ENSG00000272221	ENSG00000272221	6	31394289	31395495	lincRNA	16,30	0
ENSG00000260118	ENSG00000260118	X	68013470	68014901	lincRNA	16,30	0
ENSG00000183171	ENSG00000183171	X	150114378	150116520	processed_pseudogene	16,29	0
ENSG00000256385	UBE2NP1	14	21611924	21612381	processed_pseudogene	16,27	0
ENSG00000224066	ENSG00000224066	1	32204769	32206814	antisense	16,27	0
ENSG00000267984	ENSG00000267984	19	51345169	51353293	antisense	16,27	0
ENSG00000255883	ENSG00000255883	5	73508685	73509254	processed_pseudogene	16,26	0
ENSG00000224003	YES1P1	22	25647261	25649232	processed_pseudogene	16,26	0
ENSG00000241007	SEPT7P6	7	152367519	152368661	processed_pseudogene	16,22	0
ENSG00000225498	ENSG00000225498	7	90312496	90322592	lincRNA	16,20	0
ENSG00000242960	FTH1P23	3	72929044	72929593	processed_pseudogene	16,20	0
ENSG00000243742	RPLP0P2	11	61615036	61639449	transcribed_processed_pseudogene	16,19	2,95E-02
ENSG00000248254	ENSG00000248254	4	47556731	47560259	antisense	16,18	0
ENSG00000260121	ENSG00000260121	16	88718615	88720459	antisense	16,17	0
ENSG00000234636	MED14OS	X	40735400	40738701	antisense	16,17	0
ENSG00000260920	ENSG00000260920	1	40464319	40466767	sense_overlapping	16,12	0
ENSG00000224505	ENSG00000224505	17	45150400	45161510	antisense	16,12	0
ENSG00000268081	ENSG00000268081	19	21554640	21569237	antisense	16,10	0
ENSG00000228510	ENSG00000228510	9	128444587	128446628	processed_pseudogene	16,10	0
ENSG00000243406	MRPS31P5	13	52167709	52194465	transcribed_unprocessed_pseudogene	16,09	0
ENSG00000247498	ENSG00000247498	12	12927726	12984645	antisense	16,09	0
ENSG00000240254	B4GALT4-AS1	3	119226486	119290666	antisense	16,02	0
ENSG00000232559	ENSG00000232559	7	66554588	66576923	unprocessed_pseudogene	16,01	0
ENSG00000244558	KCNK15-AS1	20	44694892	44746021	antisense	16,00	0
ENSG00000264278	ENSG00000264278	18	76794732	76822295	lincRNA	16,00	0
ENSG00000249176	ENSG00000249176	17	49939122	49939819	processed_pseudogene	16,00	0
ENSG00000259969	ENSG00000259969	14	57993545	57994525	sense_overlapping	16,00	0
ENSG00000234814	SVILP1	10	30671566	30717266	transcribed_unprocessed_pseudogene	16,00	0
ENSG00000127957	PMS2P3	7	75502930	75528148	transcribed_unprocessed_pseudogene	16,00	0
ENSG00000215190	LINC00680	6	57946074	57961501	lincRNA	16,00	0
ENSG00000225216	ENSG00000225216	2	205756469	205764006	antisense	16,00	0
ENSG00000233223	ENSG00000233223	17	7581964	7584072	antisense	15,97	0
ENSG00000240914	RPL15P2	14	94800849	94801463	processed_pseudogene	15,94	0,1836819
ENSG00000225210	DUXAP9	14	19062316	19115270	transcribed_processed_pseudogene	15,93	0
ENSG00000273148	ENSG00000273148	20	18794529	18796067	lincRNA	15,90	0
ENSG00000215572	ESRRAP1	13	19560013	19561269	processed_pseudogene	15,90	0
ENSG00000223542	ENSG00000223542	6	133435077	133439464	antisense	15,90	7,06E-02
ENSG00000271551	ENSG00000271551	6	156776360	156778422	antisense	15,90	0
ENSG00000281398	SNHG4	5	139274102	139283244	processed_transcript	15,90	0
ENSG00000237973	MTCO1P12	1	631074	632616	unprocessed_pseudogene	15,88	0
ENSG00000244879	GABPB1-AS1	15	50354959	50372202	antisense	15,87	0
ENSG00000245685	ENSG00000245685	4	189780336	189940271	lincRNA	15,85	2,21E-02
ENSG00000248690	HAS2-AS1	8	121639293	121644693	antisense	15,84	0
ENSG00000237080	EHMT2-AS1	6	31883761	31884204	antisense	15,81	0
ENSG00000224806	ARL5AP4	22	24686168	24686679	processed_pseudogene	15,80	0,2161218
ENSG00000275645	ENSG00000275645	15	75346744	75347161	antisense	15,80	0
ENSG00000262619	LINC00621	13	22894656	22916369	lincRNA	15,80	0
ENSG00000272986	ENSG00000272986	4	70703747	70704491	antisense	15,80	1,40E-02
ENSG00000254454	RCC2P6	11	62371146	62373168	processed_pseudogene	15,77	0
ENSG00000183604	SMG1P5	16	30267553	30335374	transcribed_unprocessed_pseudogene	15,77	0
ENSG00000259113	ENSG00000259113	14	50448807	50456742	lincRNA	15,75	0
ENSG00000261207	ENSG00000261207	16	1751559	1752262	antisense	15,73	0
ENSG00000273032	DGCR9	22	19017834	19020248	lincRNA	15,70	1,40E-02

ENSG00000214401	KANSL1-AS1	17	46193576	46196723	antisense	15,70	0
ENSG00000266648	SETP3	17	58476138	58476950	processed_pseudogene	15,70	0
ENSG00000180422	LINC00304	16	89159146	89164245	lincRNA	15,70	1,40E-02
ENSG00000272463	ENSG00000272463	6	708592	711405	lincRNA	15,70	0
ENSG00000224459	ENSG00000224459	1	15740051	15749896	antisense	15,70	0
ENSG00000237976	ENSG00000237976	1	151346967	151348027	antisense	15,70	0
ENSG00000203865	ATP1A1-AS1	1	116392247	116418622	processed_transcript	15,69	0
ENSG00000226367	ST7-AS2	7	117072072	117146480	antisense	15,69	0
ENSG00000254481	PTP4A2P2	11	134123828	134124329	processed_pseudogene	15,67	1,95E-02
ENSG00000224424	PRKAR2A-AS1	3	48847572	48851981	antisense	15,65	0
ENSG00000269904	MAP2K4P1	X	73524275	73563085	transcribed_processed_pseudogene	15,63	0
ENSG00000276259	ENSG00000276259	16	58522970	58523842	antisense	15,62	0
ENSG00000270093	ENSG00000270093	21	16643529	16645065	lincRNA	15,60	0
ENSG00000268521	VN1R83P	19	21289554	21289998	unprocessed_pseudogene	15,60	0
ENSG00000269486	ENSG00000269486	19	38935297	38938632	lincRNA	15,60	0
ENSG00000260751	ENSG00000260751	16	23568673	23569696	sense_intronic	15,60	0
ENSG00000259539	ENSG00000259539	15	45152664	45167526	3prime_overlapping_ncrna	15,60	0
ENSG00000215199	YWHAZP6	9	34922184	34922921	processed_pseudogene	15,60	0,2161218
ENSG00000273145	ENSG00000273145	22	46013606	46015498	lincRNA	15,58	0
ENSG00000260777	ENSG00000260777	17	28263634	28266369	lincRNA	15,58	0
ENSG00000272008	ENSG00000272008	6	87151159	87155285	antisense	15,55	0
ENSG00000253356	ENSG00000253356	8	38148741	38163772	antisense	15,51	0
ENSG00000268087	ENSG00000268087	19	16610411	16636531	lincRNA	15,50	0
ENSG00000230177	ENSG00000230177	6	111277932	111278742	antisense	15,50	0
ENSG00000231802	ENSG00000231802	2	219684030	219684795	processed_pseudogene	15,50	1,40E-02
ENSG00000226648	PLCG1-AS1	20	41098329	41138003	antisense	15,49	0
ENSG00000227850	SEPT2P1	1	105698039	105698218	processed_pseudogene	15,49	0
ENSG00000235370	DNM1P51	15	84398316	84411701	transcribed_unprocessed_pseudogene	15,46	0
ENSG00000178977	LINC00324	17	8220642	8224043	lincRNA	15,45	0
ENSG00000241570	PAQR9-AS1	3	142960650	143001559	antisense	15,45	0
ENSG00000254065	ENSG00000254065	8	47592147	47592609	processed_pseudogene	15,43	0
ENSG00000216775	ENSG00000216775	6	52665148	52669155	transcribed_unprocessed_pseudogene	15,41	0
ENSG00000223704	LINC01422	22	26858634	26865786	lincRNA	15,40	0
ENSG00000230613	HM13-AS1	20	31567707	31573263	antisense	15,40	0
ENSG00000231113	ENSG00000231113	6	42940364	42948360	antisense	15,40	0
ENSG00000260760	PWRN3	15	24441127	24447967	lincRNA	15,36	0
ENSG00000277013	ENSG00000277013	19	33906352	33908391	lincRNA	15,30	0
ENSG00000260545	ENSG00000260545	16	58116885	58119353	antisense	15,30	0
ENSG00000198580	ENSG00000198580	7	6673494	6676366	unprocessed_pseudogene	15,30	0
ENSG00000272320	ENSG00000272320	6	3311662	3313650	antisense	15,30	0
ENSG00000254298	ENSG00000254298	5	151158106	151158462	lincRNA	15,30	0
ENSG00000239828	ENSG00000239828	3	107272611	107421304	lincRNA	15,30	0
ENSG00000258317	ENSG00000258317	12	56120033	56129619	antisense	15,29	0
ENSG00000179031	ENSG00000179031	X	100910712	100911489	processed_pseudogene	15,29	9,18E-02
ENSG00000213252	ENSG00000213252	11	106826392	106827890	processed_pseudogene	15,25	0
ENSG00000227036	LINC00511	17	72323123	72640472	lincRNA	15,25	6,27E-03
ENSG00000224635	ENSG00000224635	20	38406011	38416797	lincRNA	15,20	1,40E-02
ENSG00000270871	ENSG00000270871	17	35816717	35830293	antisense	15,20	0
ENSG00000257647	ENSG00000257647	15	25027736	25032047	lincRNA	15,20	0
ENSG00000272518	ENSG00000272518	8	79956465	79957381	sense_intronic	15,20	0
ENSG00000178162	FAR2P2	2	130416755	130431363	transcribed_unprocessed_pseudogene	15,20	0
ENSG00000227081	ENSG00000227081	12	3211663	3211917	processed_pseudogene	15,16	0
ENSG00000232801	SDCBPP3	X	40890647	40891537	processed_pseudogene	15,14	0
ENSG00000260233	SSSCA1-AS1	11	65568482	65570423	processed_transcript	15,14	0

ENSG00000175061	LRRC75A-AS1	17	16438822	16478678	processed_transcript	15,13	0
ENSG00000236105	PRELID3BP10	7	127295620	127296203	processed_pseudogene	15,12	0
ENSG00000183242	WT1-AS	11	32435518	32458769	antisense	15,10	0
ENSG00000273402	ENSG00000273402	8	237045	237669	antisense	15,10	0
ENSG00000272040	ENSG00000272040	5	75608817	75609983	lincRNA	15,10	0
ENSG00000269243	ENSG00000269243	19	16123661	16139892	antisense	15,06	0
ENSG00000256849	ENSG00000256849	12	19941521	19943018	processed_pseudogene	15,05	0
ENSG00000234665	ENSG00000234665	9	62856999	62898087	lincRNA	15,04	0
ENSG00000280758	ENSG00000280758	9	128630328	128631685	antisense	15,04	0
ENSG00000203644	ENSG00000203644	3	129847048	129847957	sense_intronic	15,02	0
ENSG00000260160	ENSG00000260160	19	52058490	52063703	sense_overlapping	15,00	0
ENSG00000263724	DLGAP1-AS3	18	3878180	3897069	antisense	15,00	2,95E-02
ENSG00000274487	ENSG00000274487	17	38195740	38257152	unprocessed_pseudogene	15,00	0
ENSG00000254872	ENSG00000254872	11	1049880	1055749	lincRNA	15,00	7,06E-02
ENSG00000272277	ENSG00000272277	6	3068045	3068894	antisense	15,00	0
ENSG00000253955	ENSG00000253955	5	173579643	173585068	antisense	15,00	0
ENSG00000261488	ENSG00000261488	3	112133423	112135359	antisense	15,00	0
ENSG00000256210	ENSG00000256210	19	14792318	14792533	processed_pseudogene	14,99	0
ENSG00000237938	ENSG00000237938	1	15720312	15736896	antisense	14,96	0
ENSG00000246695	RASSF8-AS1	12	25939329	25959765	antisense	14,96	0
ENSG00000272017	ENSG00000272017	6	99568097	99569096	antisense	14,94	0
ENSG00000235400	ENSG00000235400	1	78749073	78750659	processed_pseudogene	14,91	7,06E-02
ENSG00000213790	OLA1P1	22	42107765	42108953	processed_pseudogene	14,90	0
ENSG00000269892	ENSG00000269892	12	6742985	6743641	lincRNA	14,90	0
ENSG00000270175	ENSG00000270175	12	53500162	53500936	antisense	14,90	0
ENSG00000233038	ENSG00000233038	7	157854529	157866092	antisense	14,90	0
ENSG00000273361	ENSG00000273361	2	218398743	218399219	antisense	14,90	0
ENSG00000229820	ENSG00000229820	1	28453541	28453934	processed_pseudogene	14,90	7,06E-02
ENSG00000236263	ENSG00000236263	1	155211151	155213819	antisense	14,90	0
ENSG00000246273	SBF2-AS1	11	9758292	9811319	antisense	14,88	0
ENSG00000255517	ENSG00000255517	11	66473490	66480233	antisense	14,88	0
ENSG00000238116	ENSG00000238116	X	100801188	100803060	processed_pseudogene	14,82	0
ENSG00000264311	CCDC58P1	18	10623876	10624305	processed_pseudogene	14,82	0
ENSG00000276975	HYDIN2	1	146547367	146914294	transcribed_unprocessed_pseudogene	14,82	0
ENSG00000261342	ENSG00000261342	19	2727743	2729327	sense_intronic	14,80	0
ENSG00000229368	ENSG00000229368	11	3854612	3855399	sense_overlapping	14,80	0
ENSG00000227006	ENSG00000227006	1	230258694	230268483	antisense	14,80	0
ENSG00000232442	ENSG00000232442	20	63627227	63628824	antisense	14,79	1,95E-02
ENSG00000275202	ENSG00000275202	13	48974967	48976867	antisense	14,76	0
ENSG00000254471	ENSG00000254471	11	79987513	79989630	processed_pseudogene	14,73	0
ENSG00000267062	ENSG00000267062	19	12796823	12801849	antisense	14,72	0
ENSG00000279182	ENSG00000279182	22	50316035	50317025	antisense	14,71	0
ENSG00000230077	MTAPP2	3	189969030	189969861	processed_pseudogene	14,71	0
ENSG00000242159	ABCF2P1	3	88317156	88319022	processed_pseudogene	14,70	0
ENSG00000268810	ENSG00000268810	19	46382492	46383169	antisense	14,70	0
ENSG00000260240	APOOP5	16	59709993	59755026	transcribed_processed_pseudogene	14,70	0
ENSG00000259319	ENSG00000259319	14	75423683	75427741	antisense	14,70	0
ENSG00000273108	ENSG00000273108	10	103608619	103610050	antisense	14,70	7,06E-02
ENSG00000281186	LINC00706	10	6774307	6779180	lincRNA	14,70	7,06E-02
ENSG00000239831	RNF7P1	3	57519669	57520010	processed_pseudogene	14,69	0
ENSG00000215424	MCM3AP-AS1	21	46229217	46259390	antisense	14,65	0
ENSG00000248980	ENSG00000248980	4	176308268	176320458	antisense	14,64	0
ENSG00000218027	ENSG00000218027	6	1513698	1515289	processed_pseudogene	14,64	0
ENSG00000226526	ENSG00000226526	1	16978926	17005091	antisense	14,61	0
ENSG00000260036	ENSG00000260036	15	55442635	55443357	processed_pseudogene	14,60	0
ENSG00000237234	ENSG00000237234	6	112154765	112166476	antisense	14,60	0
ENSG00000215795	ENSG00000215795	1	247183813	247185482	processed_pseudogene	14,60	1,40E-02
ENSG00000213025	COX20P1	10	68632371	68632727	processed_pseudogene	14,59	0

ENSG00000278341	ENSG00000278341	16	88708956	88710437	antisense	14,59	0
ENSG00000272374	ENSG00000272374	6	35220370	35224630	lincRNA	14,58	3,30E-03
ENSG00000265574	WDR45BP1	17	32111562	32112590	processed_pseudogene	14,58	0
ENSG00000223642	ENSG00000223642	2	159386367	159404636	antisense	14,55	0
ENSG00000236326	ENSG00000236326	6	116244187	116244728	antisense	14,54	0
ENSG00000260260	SNHG19	16	2154797	2155358	lincRNA	14,54	3,30E-03
ENSG00000273117	ENSG00000273117	7	155295918	155297541	lincRNA	14,53	0
ENSG00000245648	ENSG00000245648	12	10363769	10398506	antisense	14,51	0
ENSG00000213801	ZNF321P	19	52927135	52942601	transcribed_processed_pseudogene	14,50	0
ENSG00000232871	SEC1P	19	48638071	48682245	unitary_pseudogene	14,50	0
ENSG00000266975	FARSA-AS1	19	12930522	12933296	antisense	14,50	0
ENSG00000267030	ENSG00000267030	19	4447304	4448217	antisense	14,50	0
ENSG00000278722	ENSG00000278722	13	57632759	57633575	antisense	14,50	0
ENSG00000256804	ENSG00000256804	12	132126461	132126764	processed_pseudogene	14,50	0
ENSG00000234956	ENSG00000234956	6	137730170	137738983	lincRNA	14,50	1,40E-02
ENSG00000249417	ENSG00000249417	3	141267353	141367137	antisense	14,50	0
ENSG00000260261	ENSG00000260261	3	195949188	195952695	lincRNA	14,50	0
ENSG00000273211	ENSG00000273211	3	48985485	48985963	lincRNA	14,50	7,06E-02
ENSG00000272807	ENSG00000272807	2	210028417	210029156	antisense	14,50	0
ENSG00000232450	ENSG00000232450	1	113698884	113699631	transcribed_processed_pseudogene	14,50	0
ENSG00000179277	MEIS3P1	17	15786618	15787575	processed_pseudogene	14,48	0
ENSG00000237248	LINC00987	12	9240003	9243052	lincRNA	14,47	0
ENSG00000236199	ENSG00000236199	9	2041900	2046023	antisense	14,46	0
ENSG00000253677	UBE2HP1	8	81254870	81255229	processed_pseudogene	14,44	0
ENSG00000258757	ENSG00000258757	14	52640839	52641566	antisense	14,42	0
ENSG00000254835	RNF185-AS1	22	31205264	31205616	antisense	14,42	0
ENSG00000268677	ENSG00000268677	19	49688853	49690573	antisense	14,41	0
ENSG00000268947	ENSG00000268947	19	35262846	35264804	sense_intronic	14,40	0
ENSG00000275527	ENSG00000275527	15	74598919	74599397	lincRNA	14,40	0
ENSG00000259090	SEPT7P1	14	35157904	35159099	processed_pseudogene	14,40	7,06E-02
ENSG00000253476	ENSG00000253476	8	25425521	25426580	lincRNA	14,40	0
ENSG00000235444	PSMB3P2	2	216610342	216610959	processed_pseudogene	14,40	0,2161218
ENSG00000231551	ENSG00000231551	1	148402516	148432545	lincRNA	14,40	0
ENSG00000260948	ENSG00000260948	1	111431046	111433068	sense_overlapping	14,40	0
ENSG00000261254	ENSG00000261254	1	100030566	100035637	sense_overlapping	14,40	0
ENSG00000272084	ENSG00000272084	1	19072110	19075511	3prime_overlapping_ncrna	14,40	0
ENSG00000262903	ENSG00000262903	17	3655621	3658092	antisense	14,38	0
ENSG00000260037	ENSG00000260037	15	71818396	71823384	antisense	14,37	0
ENSG00000235065	RPL24P2	20	21114723	21115197	processed_pseudogene	14,34	6,27E-03
ENSG00000237682	ENSG00000237682	X	101537485	101539276	processed_pseudogene	14,32	0
ENSG00000261126	ENSG00000261126	18	80046900	80095482	lincRNA	14,32	0
ENSG00000240494	RPS12P28	17	28655557	28655983	processed_pseudogene	14,30	0
ENSG00000262333	HNRNPA1P16	17	2306761	2307715	processed_pseudogene	14,30	0
ENSG00000263400	TMEM220-AS1	17	10729777	10815164	antisense	14,30	0
ENSG00000256552	ENSG00000256552	12	8320381	8369555	transcribed_processed_pseudogene	14,30	0
ENSG00000260597	ENSG00000260597	12	54019910	54022589	lincRNA	14,30	0
ENSG00000248996	ENSG00000248996	5	177494995	177503647	antisense	14,30	0
ENSG00000231147	ARHGAP42P2	2	130006200	130009123	unprocessed_pseudogene	14,30	0
ENSG00000179406	LINC00174	7	66376044	66401338	lincRNA	14,28	0
ENSG00000215256	DHRS4-AS1	14	23938731	23988839	antisense	14,28	0
ENSG00000232729	ENSG00000232729	7	74688939	74729001	processed_transcript	14,25	0
ENSG00000122432	SPATA1	1	84506291	84566194	transcribed_unprocessed_pseudogene	14,25	0
ENSG00000239246	ENSG00000239246	17	62516321	62516767	processed_pseudogene	14,22	0
ENSG00000272447	ENSG00000272447	10	79825902	79827602	lincRNA	14,22	0
ENSG00000272814	ENSG00000272814	2	46956615	46956888	antisense	14,21	0

ENSG00000266053	NDUFV2-AS1	18	9121265	9136645	antisense	14,20	0
ENSG00000188933	USP32P1	17	16786489	16804455	transcribed_unprocessed_pseudogene	14,20	0
ENSG00000263503	ENSG00000263503	17	45600869	45602340	processed_pseudogene	14,20	0
ENSG00000257271	KIRREL3-AS1	11	126543947	126610948	antisense	14,20	7,06E-02
ENSG00000232470	ENSG00000232470	10	110869743	110871594	lincRNA	14,20	0
ENSG00000261643	ENSG00000261643	4	3503597	3504457	sense_overlapping	14,20	0
ENSG00000214870	ENSG00000214870	7	26398593	26494256	lincRNA	14,18	3,30E-03
ENSG00000261423	ENSG00000261423	15	72407778	72475168	lincRNA	14,16	0
ENSG00000272501	ENSG00000272501	6	31195200	31198037	antisense	14,13	0
ENSG00000214077	GNAQP1	2	131423801	131424867	processed_pseudogene	14,13	0
ENSG00000248492	ZFAT-AS1	8	134598071	134600689	antisense	14,11	0
ENSG00000274460	ENSG00000274460	16	21950218	21951708	processed_transcript	14,10	7,06E-02
ENSG00000256268	ENSG00000256268	12	65602869	65612997	lincRNA	14,10	0
ENSG00000196167	COLCA1	11	111290787	111305045	antisense	14,10	0
ENSG00000226576	ENSG00000226576	10	48984564	49018897	antisense	14,10	0
ENSG00000250899	ENSG00000250899	12	3041437	3044950	lincRNA	14,08	0
ENSG00000257663	ENSG00000257663	12	52076841	52082084	antisense	14,06	0
ENSG00000274425	ENSG00000274425	19	10333436	10336248	antisense	14,05	0
ENSG00000236144	TMEM147-AS1	19	35540738	35546029	antisense	14,03	0
ENSG00000214185	XPOTP1	20	34213495	34215892	processed_pseudogene	14,02	0
ENSG00000267372	ENSG00000267372	19	1321225	1322846	lincRNA	14,00	0
ENSG00000263179	HNRNPCP4	16	11242653	11243552	processed_pseudogene	14,00	0
ENSG00000257350	ENSG00000257350	12	55122668	55124120	processed_pseudogene	14,00	0
ENSG00000234604	ENSG00000234604	1	169474060	169474159	processed_pseudogene	14,00	0
ENSG00000157021	FAM92A1P1	15	41163162	41164374	unprocessed_pseudogene	13,95	0
ENSG00000263272	ENSG00000263272	17	5425139	5432876	antisense	13,93	0
ENSG00000247092	SNHG10	14	95532297	95534872	antisense	13,93	0
ENSG00000232389	ENSG00000232389	6	70608234	70609334	processed_pseudogene	13,92	0
ENSG00000254510	ENSG00000254510	11	66409158	66417137	processed_transcript	13,92	4,86E-02
ENSG00000256034	ENSG00000256034	11	73760563	73761070	antisense	13,91	0
ENSG00000225792	ENSG00000225792	7	26372144	26376701	antisense	13,91	0
ENSG00000266896	ENSG00000266896	6	169770413	169772042	sense_intronic	13,91	0
ENSG00000226450	CYP2D8P	22	42149886	42155001	unprocessed_pseudogene	13,90	0
ENSG00000261474	ENSG00000261474	16	31449535	31453493	lincRNA	13,90	0
ENSG00000261644	ENSG00000261644	16	50735713	50740716	antisense	13,90	0
ENSG00000258376	ENSG00000258376	14	73242651	73245979	antisense	13,90	0
ENSG00000234043	ENSG00000234043	10	91152605	91153349	processed_pseudogene	13,90	0
ENSG00000273597	ENSG00000273597	9	35001342	35002994	processed_pseudogene	13,90	1,40E-02
ENSG00000239254	ENSG00000239254	7	139172516	139174266	processed_pseudogene	13,90	0,2161218
ENSG00000226445	ENSG00000226445	6	169213254	169239565	antisense	13,90	0
ENSG00000243592	RPL17P22	5	56136979	56137531	processed_pseudogene	13,90	0
ENSG00000248213	CICP16	4	118635970	118638782	processed_pseudogene	13,90	0
ENSG00000198590	C3orf35	3	37386269	37435497	lincRNA	13,90	0
ENSG00000279873	LINC01126	2	43227210	43228855	lincRNA	13,90	0
ENSG00000177788	ENSG00000177788	1	229258281	229271028	lincRNA	13,90	0
ENSG00000266527	ENSG00000266527	17	27874645	27881237	antisense	13,88	0
ENSG00000259137	ENSG00000259137	14	44120210	44120761	processed_pseudogene	13,87	0
ENSG00000187534	PRR13P5	19	39943239	39943680	processed_pseudogene	13,84	0
ENSG00000262456	ENSG00000262456	17	2384847	2386664	antisense	13,84	0
ENSG00000230022	FNTAP2	13	21530690	21531323	processed_pseudogene	13,83	0,0358681
ENSG00000267458	ENSG00000267458	19	12944118	12944487	antisense	13,83	0
ENSG00000242779	ZNF702P	19	52968251	53037898	transcribed_processed_pseudogene	13,83	0
ENSG00000241769	LINC00893	X	149527591	149540959	antisense	13,82	0
ENSG00000258199	ENSG00000258199	12	56162359	56190284	sense_overlapping	13,82	0
ENSG00000272677	ENSG00000272677	4	82374301	82384027	antisense	13,82	0
ENSG00000273893	ENSG00000273893	20	46681676	46682375	sense_intronic	13,80	0
ENSG00000268516	ENSG00000268516	19	58257270	58278808	antisense	13,80	0

ENSG00000281005	LINC00921	16	3263743	3267567	lincRNA	13,80	0
ENSG00000243193	ENSG00000243193	7	107066591	107133733	processed_transcript	13,80	0
ENSG00000249244	ENSG00000249244	4	119391831	119395335	unprocessed_pseudogene	13,80	0
ENSG00000261533	ENSG00000261533	3	100179567	100181732	sense_overlapping	13,80	0
ENSG00000232800	SLC7A15P	2	20386386	20396588	unitary_pseudogene	13,80	0
ENSG00000237514	PTP4A1P7	1	176616273	176616786	processed_pseudogene	13,80	0
ENSG00000232725	ENSG00000232725	X	153735626	153766478	antisense	13,80	0
ENSG00000245849	RAD51-AS1	15	40686724	40695107	processed_transcript	13,80	0
ENSG00000263311	ENSG00000263311	16	72004239	72004803	unprocessed_pseudogene	13,77	1,40E-02
ENSG00000261065	ENSG00000261065	1	204131062	204131966	antisense	13,73	0
ENSG00000269106	ENSG00000269106	19	58475355	58475763	antisense	13,73	0
ENSG00000235079	ZRANB2-AS1	1	71048855	71067184	antisense	13,70	0
ENSG00000267355	RPL9P29	17	78845953	78846868	processed_pseudogene	13,70	0
ENSG00000213149	CNN2P9	6	110858239	110859147	processed_pseudogene	13,70	1,40E-02
ENSG00000234789	ENSG00000234789	9	129640476	129641282	antisense	13,68	0
ENSG00000268061	NAPA-AS1	19	47484282	47501597	antisense	13,68	0
ENSG00000237596	ENSG00000237596	6	135991936	136225751	antisense	13,67	0
ENSG00000241889	ENSG00000241889	3	113885298	113886031	processed_pseudogene	13,67	0
ENSG00000213642	ENSG00000213642	7	64569428	64570437	processed_pseudogene	13,67	0
ENSG00000183199	HSP90AB3P	4	87891843	87894015	processed_pseudogene	13,66	0
ENSG00000279571	ENSG00000279571	9	127690098	127690840	lincRNA	13,64	0
ENSG00000261777	ENSG00000261777	16	70315640	70346747	processed_transcript	13,61	0
ENSG00000281404	LINC01176	7	30390885	30412375	lincRNA	13,60	7,06E-02
ENSG00000227212	PFN1P6	1	144442606	144443004	processed_pseudogene	13,60	0
ENSG00000227279	ENSG00000227279	18	27954519	27963687	antisense	13,59	0
ENSG00000260689	HNRNPA3P11	15	57246960	57247893	processed_pseudogene	13,55	0
ENSG00000260892	ENSG00000260892	15	75676227	75677162	antisense	13,50	0
ENSG00000254554	ENSG00000254554	11	10302657	10303704	antisense	13,50	0
ENSG00000226206	ENSG00000226206	9	89799695	89821753	lincRNA	13,50	0
ENSG00000234946	SDHCP3	2	23943846	23944351	processed_pseudogene	13,50	0
ENSG00000174028	FAM3C2	X	23075758	23076767	processed_pseudogene	13,46	0
ENSG00000261770	ENSG00000261770	19	27757184	27760849	lincRNA	13,45	0,0358681
ENSG00000232818	RPS2P32	7	23490473	23491364	processed_pseudogene	13,45	0
ENSG00000241288	ENSG00000241288	3	125827238	125916384	processed_transcript	13,45	0
ENSG00000232888	RPS11P5	12	132825701	132826184	processed_pseudogene	13,42	0
ENSG00000224078	SNHG14	15	24978583	25419462	processed_transcript	13,40	0
ENSG00000269549	ENSG00000269549	20	45445431	45448580	lincRNA	13,40	0
ENSG00000263874	LINC00672	17	38925168	38928057	lincRNA	13,40	0
ENSG00000261336	EIF4BP5	16	47565090	47566884	processed_pseudogene	13,40	1,40E-02
ENSG00000238244	GABARAPL3	15	90348844	90349197	processed_pseudogene	13,40	0
ENSG00000263873	ENSG00000263873	11	119417951	119419114	sense_intronic	13,40	7,06E-02
ENSG00000249863	ENSG00000249863	4	37868292	37869978	processed_pseudogene	13,40	0
ENSG00000265298	ENSG00000265298	17	64750420	64751311	unprocessed_pseudogene	13,36	0
ENSG00000273466	ENSG00000273466	2	218633256	218634014	antisense	13,35	0
ENSG00000204282	TNRC6C-AS1	17	78107398	78111799	processed_transcript	13,33	0
ENSG00000183562	ENSG00000183562	11	2989863	2991344	antisense	13,33	0
ENSG00000256745	ENSG00000256745	11	94188449	94188997	processed_pseudogene	13,31	0
ENSG00000267655	ENSG00000267655	18	79117207	79117920	sense_intronic	13,30	0
ENSG00000227685	ENSG00000227685	17	20612912	20633234	lincRNA	13,30	0
ENSG00000259244	ENSG00000259244	15	84513241	84526949	processed_transcript	13,30	0
ENSG00000254162	ENSG00000254162	8	80535006	80539135	lincRNA	13,30	0
ENSG00000243243	ENSG00000243243	7	116237929	116327896	antisense	13,30	0
ENSG00000238221	ENSG00000238221	6	7276031	7298872	antisense	13,29	0
ENSG00000257530	ENSG00000257530	12	32104117	32107528	antisense	13,27	0
ENSG00000271976	ENSG00000271976	3	53858994	53861576	antisense	13,27	0
ENSG00000256712	ENSG00000256712	12	11166090	11171353	sense_intronic	13,23	0
ENSG00000268034	ENSG00000268034	19	41506152	41506898	processed_pseudogene	13,23	0

ENSG00000265018	CTGLF12P	10	48009873	48031640	transcribed_unprocessed_pseudogene	13,20	0
ENSG00000246582	ENSG00000246582	8	23224471	23230926	processed_transcript	13,20	0
ENSG00000271367	ENSG00000271367	6	53350158	53350705	lincRNA	13,20	0
ENSG00000271978	ENSG00000271978	6	5031756	5054423	lincRNA	13,20	0
ENSG00000250942	ENPP7P11	4	9677308	9677934	processed_pseudogene	13,20	7,06E-02
ENSG00000232721	ENSG00000232721	1	143736066	143739506	lincRNA	13,20	1,40E-02
ENSG00000232927	USP12PY	X	3682805	3683868	processed_pseudogene	13,20	0
ENSG00000230154	ENSG00000230154	2	11308025	11308941	processed_pseudogene	13,18	0
ENSG00000232842	ENSG00000232842	X	95973328	95974315	processed_pseudogene	13,17	0
ENSG00000266341	ENSG00000266341	17	48060383	48060669	antisense	13,16	0
ENSG00000257779	ENSG00000257779	12	63002469	63002795	processed_pseudogene	13,15	0
ENSG00000260326	PHBP21	16	52934813	52935635	processed_pseudogene	13,15	0
ENSG00000176857	GJA1P1	5	109051315	109052457	processed_pseudogene	13,15	0,3565078
ENSG00000264985	ENSG00000264985	17	73513685	73518770	antisense	13,13	4,86E-02
ENSG00000260966	ENSG00000260966	11	103050687	103055799	sense_overlapping	13,12	0
ENSG00000231341	VDAC1P6	X	5207215	5208069	processed_pseudogene	13,11	0
ENSG00000239736	CEACAMP3	19	41599735	41605984	transcribed_unprocessed_pseudogene	13,10	0
ENSG00000272140	ENSG00000272140	10	73703735	73713581	lincRNA	13,10	0
ENSG00000275160	ENSG00000275160	9	41479960	41480548	processed_pseudogene	13,10	0
ENSG00000248958	ZSWIM5P3	4	129135926	129139740	processed_pseudogene	13,10	0
ENSG00000224520	KRT8P45	1	157073257	157074703	processed_pseudogene	13,10	0
ENSG00000251141	ENSG00000251141	5	44744900	44808777	antisense	13,09	0
ENSG00000261684	ENSG00000261684	15	42531867	42532840	antisense	13,09	0
ENSG00000268583	ENSG00000268583	19	48204083	48213154	antisense	13,07	0
ENSG00000229931	ENSG00000229931	6	16761138	16762652	antisense	13,05	0
ENSG00000233885	YEATS2-AS1	3	183806457	183810783	antisense	13,04	0
ENSG00000229944	EIF4EP2	17	49424269	49424922	processed_pseudogene	13,02	0
ENSG00000266049	ENSG00000266049	18	2688564	2833065	antisense	13,00	0
ENSG00000268873	ENSG00000268873	18	34737795	34767663	antisense	13,00	0
ENSG00000260279	ENSG00000260279	16	89297508	89298317	antisense	13,00	0
ENSG00000260350	ENSG00000260350	16	8847650	8848724	antisense	13,00	0
ENSG00000276931	ENSG00000276931	16	968375	969012	sense_intronic	13,00	0
ENSG00000203392	ENSG00000203392	15	75678548	75680752	antisense	13,00	1,40E-02
ENSG00000255874	LINC00346	13	110863987	110870251	lincRNA	13,00	0
ENSG00000224899	ENSG00000224899	7	125151326	125153611	lincRNA	13,00	0
ENSG00000215068	ENSG00000215068	5	43041575	43045390	antisense	13,00	0
ENSG00000245112	SMARCA5-AS1	4	143513472	143514635	antisense	13,00	0
ENSG00000225554	ENSG00000225554	1	241424292	241433492	lincRNA	13,00	7,06E-02
ENSG00000250536	ABHD17AP3	1	214605470	214608840	unprocessed_pseudogene	13,00	0
ENSG00000274629	ENSG00000274629	2	224464682	224474500	antisense	12,98	0
ENSG00000225057	ENSG00000225057	2	238231684	238255633	processed_transcript	12,98	0
ENSG00000257279	ENSG00000257279	12	116977442	116987337	antisense	12,96	0
ENSG00000262155	ENSG00000262155	16	25066937	25068943	lincRNA	12,96	0
ENSG00000258461	ENSG00000258461	15	42348103	42412317	processed_transcript	12,95	0
ENSG00000233706	ENSG00000233706	1	226992140	226993206	antisense	12,94	0
ENSG00000227953	LINC01341	1	246776013	246792385	processed_transcript	12,91	6,27E-03
ENSG00000232530	ENSG00000232530	22	30239194	30240538	antisense	12,90	1,40E-02
ENSG00000258302	ENSG00000258302	12	89561129	89594878	antisense	12,90	0
ENSG00000260400	ENSG00000260400	10	68698500	68700794	sense_overlapping	12,90	0
ENSG00000271361	HTATSF1P2	6	3023142	3023772	processed_pseudogene	12,90	0
ENSG00000242539	ENSG00000242539	3	179396961	179399191	antisense	12,89	0
ENSG00000224237	MINOS1P3	3	27214816	27215018	processed_pseudogene	12,89	7,06E-02
ENSG00000263931	ENSG00000263931	17	62935402	62966810	lincRNA	12,87	0
ENSG00000228057	SEC63P1	1	97545701	97546958	processed_pseudogene	12,85	0
ENSG00000248334	WHAMMP2	15	28737583	28759204	transcribed_unprocessed_pseudogene	12,84	0
ENSG00000264932	ENSG00000264932	17	19649373	19649935	antisense	12,80	0
ENSG00000250790	ENSG00000250790	12	132911470	132914732	lincRNA	12,80	0

ENSG00000254851	ENSG00000254851	11	117135528	117138582	unprocessed_pseudogene	12,80	1,40E-02
ENSG00000271141	ENSG00000271141	2	178616581	178617123	lincRNA	12,80	0
ENSG00000281162	LINC01127	2	101962056	101987167	lincRNA	12,80	0
ENSG00000269925	ENSG00000269925	1	7776383	7776775	sense_intronic	12,80	0
ENSG00000263756	ENSG00000263756	6	32972065	32972853	antisense	12,79	0
ENSG00000253919	PRKRIRP7	8	78760142	78762431	processed_pseudogene	12,79	0
ENSG00000230221	ENSG00000230221	9	95292338	95294810	processed_pseudogene	12,75	0
ENSG00000267791	ENSG00000267791	19	12688922	12689238	antisense	12,72	0
ENSG00000268565	ENSG00000268565	19	4654964	4655524	antisense	12,71	0
ENSG00000273350	ENSG00000273350	22	30420512	30420912	sense_intronic	12,70	0
ENSG00000262188	ENSG00000262188	17	79919357	79925462	lincRNA	12,70	7,06E-02
ENSG00000273680	ENSG00000273680	12	29332733	29333383	antisense	12,70	0
ENSG00000254963	ENSG00000254963	11	75264289	75265170	antisense	12,70	0
ENSG00000232387	SKA2P1	9	122761844	122762233	processed_pseudogene	12,70	0
ENSG00000204789	ZNF204P	6	27357825	27360221	processed_pseudogene	12,70	0
ENSG00000240050	ENSG00000240050	6	116280199	116370273	lincRNA	12,70	0
ENSG00000237786	GFOD1-AS1	6	13486294	13486852	antisense	12,64	0
ENSG00000229348	HYI-AS1	1	43453927	43456995	antisense	12,64	3,30E-03
ENSG00000235636	NUS1P1	X	47512602	47513483	processed_pseudogene	12,62	0
ENSG00000244158	ENSG00000244158	6	116460739	116463692	antisense	12,62	0
ENSG00000225783	MIAT	22	26646428	26676475	lincRNA	12,60	0
ENSG00000231713	ENSG00000231713	21	39727755	39730680	lincRNA	12,60	0
ENSG00000256176	ENSG00000256176	12	31311617	31312029	processed_pseudogene	12,60	0,2161218
ENSG00000255135	ENSG00000255135	11	76441338	76444656	lincRNA	12,60	0
ENSG00000232815	DUX4L50	9	63817748	63818462	unprocessed_pseudogene	12,60	0
ENSG00000244219	ENSG00000244219	7	99598066	99610813	lincRNA	12,60	7,06E-02
ENSG00000229727	ENSG00000229727	2	7421261	7450254	processed_transcript	12,60	1,40E-02
ENSG00000272644	ENSG00000272644	2	219069354	219069809	lincRNA	12,60	0
ENSG00000213754	ENSG00000213754	X	64305047	64306733	processed_pseudogene	12,60	0,2161218
ENSG00000237126	ENSG00000237126	2	232580948	232611971	antisense	12,56	0
ENSG00000228847	ATP5G2P4	X	42098754	42099173	processed_pseudogene	12,54	6,27E-03
ENSG00000234327	ENSG00000234327	17	5111468	5115004	processed_transcript	12,54	1,40E-02
ENSG00000172974	ENSG00000172974	2	65205108	65205988	processed_pseudogene	12,53	0
ENSG00000233521	ENSG00000233521	22	27221349	27224727	lincRNA	12,50	7,06E-02
ENSG00000270001	ENSG00000270001	20	23631826	23632316	lincRNA	12,50	0
ENSG00000273958	ENSG00000273958	15	60593027	60593460	lincRNA	12,50	0
ENSG00000258407	ENSG00000258407	14	88010787	88015611	antisense	12,50	0
ENSG00000276698	ENSG00000276698	14	24198433	24199090	lincRNA	12,50	0
ENSG00000255091	ENSG00000255091	11	45651529	45652691	antisense	12,50	0
ENSG00000270589	ENSG00000270589	10	110910596	110912244	antisense	12,50	0
ENSG00000250320	ENSG00000250320	5	84384427	84490765	antisense	12,50	0
ENSG00000253167	KIAA0196-AS1	8	125040684	125044989	antisense	12,49	0
ENSG00000225026	ENSG00000225026	3	12328003	12328274	processed_pseudogene	12,46	0
ENSG00000213888	LINC01521	22	31346777	31348719	lincRNA	12,45	0
ENSG00000270681	ENSG00000270681	4	151139991	151141103	antisense	12,44	0
ENSG00000267205	ENSG00000267205	19	3753840	3756517	antisense	12,44	0
ENSG00000249700	SRD5A3-AS1	4	55363971	55395847	processed_transcript	12,42	0
ENSG00000274859	ENSG00000274859	12	118066398	118066725	antisense	12,42	0
ENSG00000281538	ENSG00000281538	22	42138060	42139726	lincRNA	12,40	0
ENSG00000256802	ENSG00000256802	15	29674990	29680957	antisense	12,40	0
ENSG00000271882	ENSG00000271882	8	101208148	101208558	lincRNA	12,40	0
ENSG00000273014	ENSG00000273014	7	32758882	32759353	lincRNA	12,40	0
ENSG00000223522	ENSG00000223522	2	28307691	28310459	antisense	12,40	0
ENSG00000246575	ENSG00000246575	2	85315041	85316529	unprocessed_pseudogene	12,40	0
ENSG00000243970	PPIEL	1	39531838	39558707	unprocessed_pseudogene	12,40	0
ENSG00000278834	ENSG00000278834	17	40648300	40649718	antisense	12,39	0
ENSG00000231167	YBX1P2	7	105582258	105583256	processed_pseudogene	12,32	0

ENSG00000226284	ARPC3P1	20	49134480	49135012	processed_pseudogene	12,31	0
ENSG00000260924	LINC01311	22	19171395	19172839	antisense	12,30	1,40E-02
ENSG00000236914	ENSG00000236914	15	38069481	38072959	lincRNA	12,30	0
ENSG00000282100	HSP90AB4P	15	58691106	58693125	processed_pseudogene	12,30	0,2161218
ENSG00000258985	ENSG00000258985	14	53036755	53038251	antisense	12,30	0
ENSG00000213365	ENSG00000213365	11	72280151	72281178	processed_pseudogene	12,30	7,06E-02
ENSG00000242474	ENSG00000242474	7	135853	149466	lincRNA	12,30	0
ENSG00000223774	ENSG00000223774	1	201893842	201899978	antisense	12,30	0
ENSG00000272405	ENSG00000272405	1	156641666	156644887	antisense	12,30	0
ENSG00000274422	ENSG00000274422	22	22283928	22287220	lincRNA	12,29	0
ENSG00000238251	ENSG00000238251	9	96407284	96407797	processed_pseudogene	12,28	0
ENSG00000261663	ENSG00000261663	16	2240487	2241818	antisense	12,27	3,30E-03
ENSG00000229127	ENSG00000229127	2	210030733	210064356	antisense	12,27	0
ENSG00000256737	RBBP4P5	14	21563819	21565084	processed_pseudogene	12,27	0
ENSG00000217239	ENSG00000217239	6	5788346	5788699	processed_pseudogene	12,23	3,30E-03
ENSG00000230630	DNM3OS	1	172136531	172144794	antisense	12,23	0
ENSG00000226706	ENSG00000226706	9	135503273	135506447	antisense	12,23	0
ENSG00000275494	ENSG00000275494	16	25106569	25107102	sense_intronic	12,20	0
ENSG00000258260	ENSG00000258260	12	56267793	56270104	antisense	12,20	0
ENSG00000275318	ENSG00000275318	9	92840955	92841688	unprocessed_pseudogene	12,20	0
ENSG00000259820	ENSG00000259820	8	134792020	134798272	lincRNA	12,20	0
ENSG00000272656	ENSG00000272656	3	139349024	139349371	antisense	12,20	0
ENSG00000280374	ENSG00000280374	2	177317715	177318471	sense_intronic	12,20	0
ENSG00000233184	ENSG00000233184	1	101025878	101087268	antisense	12,19	0
ENSG00000227230	ENSG00000227230	1	243135898	243140588	antisense	12,18	0
ENSG00000278921	EPB41L4A-AS2	5	112419583	112420978	lincRNA	12,18	0
ENSG00000246334	PRR7-AS1	5	177438503	177447699	antisense	12,17	0
ENSG00000244153	WWP1P1	3	98657802	98660570	processed_pseudogene	12,17	0
ENSG00000182574	ENSG00000182574	X	100956593	100957538	processed_pseudogene	12,17	0
ENSG00000278768	BACE1-AS	11	117290874	117293571	antisense	12,15	0
ENSG00000246859	STARD4-AS1	5	111512226	111739726	antisense	12,15	0
ENSG00000225194	LINC00092	9	96019732	96027965	lincRNA	12,15	0
ENSG00000228878	SEPT7-AS1	7	35751856	35800616	lincRNA	12,15	0
ENSG00000214561	RBBP4P4	6	58119741	58121029	processed_pseudogene	12,14	0
ENSG00000223725	ENSG00000223725	2	207239864	207529795	antisense	12,13	0
ENSG00000267040	ENSG00000267040	18	57630302	57669296	antisense	12,12	0
ENSG00000259581	TYRO3P	15	76258986	76261690	processed_pseudogene	12,12	0
ENSG00000233868	ENSG00000233868	2	221944223	221944612	processed_pseudogene	12,11	0
ENSG00000230928	ENSG00000230928	10	98252023	98256575	antisense	12,10	0
ENSG00000254363	ENSG00000254363	5	140157319	140173051	processed_transcript	12,10	0
ENSG00000270426	ENSG00000270426	4	184471924	184472609	sense_intronic	12,10	1,40E-02
ENSG00000244300	GATA2-AS1	3	128489212	128502348	antisense	12,10	0
ENSG00000237641	ENSG00000237641	2	231789481	231789887	processed_pseudogene	12,10	0
ENSG00000234115	ENSG00000234115	6	7516600	7517840	processed_pseudogene	12,08	0
ENSG00000234945	GTF3C2-AS1	2	27335535	27342599	antisense	12,08	0
ENSG00000272106	ENSG00000272106	1	1613758	1615795	antisense	12,06	0
ENSG00000248697	TOX4P1	4	112455924	112457785	processed_pseudogene	12,05	0
ENSG00000269388	ENSG00000269388	19	51594561	51606150	transcribed_processed_pseudogene	12,00	7,06E-02
ENSG00000262089	ENSG00000262089	17	6994642	6995189	lincRNA	12,00	6,27E-03
ENSG00000226180	ENSG00000226180	16	87693537	87696147	lincRNA	12,00	0
ENSG00000275149	ENSG00000275149	13	40079106	40273509	lincRNA	12,00	1,40E-02
ENSG00000257802	ENSG00000257802	12	71849228	71850428	processed_pseudogene	12,00	1,40E-02
ENSG00000242798	ENSG00000242798	7	100115214	100127139	antisense	12,00	0
ENSG00000223821	ENSG00000223821	6	73492025	73492742	antisense	12,00	0
ENSG00000273345	ENSG00000273345	5	134205614	134371044	processed_transcript	12,00	0
ENSG00000184139	RPL7AP28	4	131857343	131857913	processed_pseudogene	12,00	0
ENSG00000242428	C3orf67-AS1	3	58824437	59019093	antisense	12,00	0
ENSG00000271452	ENSG00000271452	2	75669989	75670454	sense_intronic	12,00	0

ENSG00000224775	BRAFP1	X	75582676	75585506	unprocessed_pseudogene	12,00	0
ENSG00000236756	DNAJC9-AS1	10	73247360	73276984	antisense	11,99	0
ENSG00000269352	PTOV1-AS2	19	49856970	49859289	antisense	11,99	0
ENSG00000280213	UCKL1-AS1	20	63953384	63956985	antisense	11,91	0
ENSG00000237187	NR2F1-AS1	5	93409359	93585648	antisense	11,91	0
ENSG00000184809	B3GALT5-AS1	21	39597147	39612821	antisense	11,91	0
ENSG00000272908	ENSG00000272908	7	38326070	38329643	lincRNA	11,91	0
ENSG00000255929	ENSG00000255929	11	94545330	94740355	antisense	11,90	0
ENSG00000267344	ENSG00000267344	17	45396932	45397477	antisense	11,90	0
ENSG00000225746	SNHG23	14	100937401	100960199	lincRNA	11,90	0
ENSG00000272092	ENSG00000272092	8	38382364	38383461	lincRNA	11,90	0
ENSG00000213057	C1orf220	1	178542752	178548889	lincRNA	11,90	0
ENSG00000226862	ENSG00000226862	1	202604268	202605293	antisense	11,90	7,06E-02
ENSG00000234546	ENSG00000234546	1	9182004	9196284	lincRNA	11,90	3,30E-03
ENSG00000215414	PSMA6P1	X	13286638	13287378	processed_pseudogene	11,90	0
ENSG00000224914	LINC00863	10	87342736	87357882	lincRNA	11,89	0
ENSG00000260807	ENSG00000260807	16	975761	981596	lincRNA	11,87	7,06E-02
ENSG00000227372	TP73-AS1	1	3735601	3747336	antisense	11,86	0
ENSG00000272913	ENSG00000272913	2	95525109	95526702	lincRNA	11,86	0
ENSG00000229334	ENSG00000229334	3	194632923	194645401	antisense	11,86	0
ENSG00000254473	ENSG00000254473	9	83707594	83713378	antisense	11,86	0
ENSG00000268996	MAN1B1-AS1	9	137084946	137086817	antisense	11,83	0
ENSG00000255438	ENSG00000255438	20	47677901	47686297	antisense	11,83	0
ENSG00000223923	ENSG00000223923	2	217978707	217992615	antisense	11,82	0
ENSG00000272872	ENSG00000272872	22	15823197	15823890	sense_intronic	11,80	0
ENSG00000275902	ENSG00000275902	17	81697025	81697714	antisense	11,80	0
ENSG00000239763	ENSG00000239763	16	74312609	74313390	processed_pseudogene	11,80	0
ENSG00000277999	ENSG00000277999	16	29272220	29272772	lincRNA	11,80	0
ENSG00000258399	MEG8	14	100894770	100935999	lincRNA	11,80	0
ENSG00000274956	ENSG00000274956	8	62977861	62984900	lincRNA	11,80	0
ENSG00000272693	ENSG00000272693	7	65647010	65770810	lincRNA	11,80	0
ENSG00000231668	PPP2R2DP1	3	38051759	38052395	processed_pseudogene	11,80	0,2161218
ENSG00000273455	ENSG00000273455	3	136087475	136087913	antisense	11,80	0
ENSG00000227817	ARHGAP42P1	2	131328176	131330857	unprocessed_pseudogene	11,80	0
ENSG00000223356	ENSG00000223356	1	156712212	156713174	antisense	11,80	7,06E-02
ENSG00000227082	ENSG00000227082	1	121396754	121463129	lincRNA	11,80	0
ENSG00000273129	PACERR	1	186680622	186681446	lincRNA	11,80	0
ENSG00000267278	MAP3K14-AS1	17	45247925	45268630	antisense	11,77	0
ENSG00000231259	ENSG00000231259	2	87031815	87053069	unprocessed_pseudogene	11,76	0
ENSG00000213742	ZNF337-AS1	20	25624045	25678074	antisense	11,75	0
ENSG00000223519	KIF28P	1	246771837	246830059	unitary_pseudogene	11,74	0
ENSG00000255498	ENSG00000255498	11	45905941	45906461	antisense	11,73	0
ENSG00000262313	ENSG00000262313	17	80940418	80942033	antisense	11,73	0
ENSG00000278616	BEND3P3	10	79682997	79685436	processed_pseudogene	11,71	0
ENSG00000269982	ENSG00000269982	3	9958717	9962539	antisense	11,71	0
ENSG00000189423	USP32P3	17	20415547	20431008	transcribed_unprocessed_pseudogene	11,70	0
ENSG00000263644	ENSG00000263644	17	63391191	63431089	antisense	11,70	0
ENSG00000266651	ENSG00000266651	17	16440479	16440952	lincRNA	11,70	0
ENSG00000273723	ENSG00000273723	13	52651305	52652279	lincRNA	11,70	0
ENSG00000254966	ENSG00000254966	11	18706537	18740568	antisense	11,70	0
ENSG00000227462	ENSG00000227462	10	22225698	22226024	processed_pseudogene	11,70	0
ENSG00000272777	ENSG00000272777	4	99067256	99068125	lincRNA	11,70	1,40E-02
ENSG00000233589	ENSG00000233589	1	68479129	68483539	antisense	11,70	0
ENSG00000251359	WWC2-AS2	4	183097017	183099199	lincRNA	11,69	0
ENSG00000272054	ENSG00000272054	2	37208875	37212677	sense_intronic	11,68	0
ENSG00000078319	PMS2P1	7	100320992	100341908	unprocessed_pseudogene	11,68	0

ENSG00000263165	ENSG00000263165	17	4163910	4164713	antisense	11,64	0
ENSG00000240571	ENSG00000240571	7	130173718	130205361	antisense	11,63	0
ENSG00000228941	UBE3AP2	21	31060600	31063168	processed_pseudogene	11,62	0,3565078
ENSG00000272476	ENSG00000272476	6	107957413	107959986	antisense	11,62	0
ENSG00000268670	ENSG00000268670	19	4061615	4062749	sense_intronic	11,60	0
ENSG00000254676	ENSG00000254676	11	83180144	83184520	sense_overlapping	11,60	0
ENSG00000218772	FAM8A6P	6	134603564	134604698	processed_pseudogene	11,60	0
ENSG00000268592	ENSG00000268592	6	149863494	149919507	antisense	11,60	0
ENSG00000235927	NEXN-AS1	1	77881348	77889539	antisense	11,60	0
ENSG00000267620	ENSG00000267620	18	60196555	60198172	processed_pseudogene	11,59	0
ENSG00000214855	APOC1P1	19	44926804	44931386	transcribed_unprocessed_pseudogene	11,58	0,0358681
ENSG00000274245	ENSG00000274245	1	207372559	207373252	lincRNA	11,58	0,0358681
ENSG00000205794	ENSG00000205794	4	40042917	40057199	transcribed_processed_pseudogene	11,58	0
ENSG00000266680	ENSG00000266680	6	63571005	63572408	antisense	11,57	0
ENSG00000262766	ENSG00000262766	16	31118078	31118747	sense_intronic	11,56	0
ENSG00000245213	ENSG00000245213	4	173131928	173169652	antisense	11,56	0
ENSG00000267705	ENSG00000267705	18	58752179	58753898	lincRNA	11,53	0
ENSG00000230897	RPS18P12	17	14705076	14705534	processed_pseudogene	11,51	0
ENSG00000245385	ENSG00000245385	11	119336249	119337309	antisense	11,51	0
ENSG00000272977	ENSG00000272977	22	25476218	25479971	sense_intronic	11,50	0
ENSG00000230865	TSEN15P1	17	17456427	17457259	processed_pseudogene	11,50	0
ENSG00000265136	ENSG00000265136	17	82576616	82577391	processed_pseudogene	11,50	1,40E-02
ENSG00000266998	ENSG00000266998	17	77373818	77377236	antisense	11,50	0
ENSG00000242902	ENSG00000242902	7	128850162	128862626	antisense	11,50	0
ENSG00000228643	ENSG00000228643	2	286419	301515	processed_transcript	11,50	7,06E-02
ENSG00000215833	QRSL1P1	1	168449672	168451211	processed_pseudogene	11,50	0,2161218
ENSG00000251661	ENSG00000251661	11	318640	325631	antisense	11,46	0
ENSG00000248827	ENSG00000248827	5	107724961	107727117	processed_pseudogene	11,46	0
ENSG00000251429	ENSG00000251429	4	158270378	158278676	transcribed_processed_pseudogene	11,45	0
ENSG00000273472	ENSG00000273472	4	140756528	140757921	lincRNA	11,45	0
ENSG00000250116	ENSG00000250116	2	46568256	46580238	antisense	11,45	0
ENSG00000238222	MKRN4P	X	40834485	40836005	processed_pseudogene	11,45	0
ENSG00000240875	LINC00886	3	156747346	156817062	lincRNA	11,43	0
ENSG00000255085	ENSG00000255085	8	144700353	144708517	transcribed_unprocessed_pseudogene	11,41	0
ENSG00000266405	CBX3P2	18	2652170	2655395	transcribed_processed_pseudogene	11,40	0
ENSG00000278000	ENSG00000278000	18	80161752	80162413	sense_intronic	11,40	7,06E-02
ENSG00000230662	TNPO1P2	17	18460391	18463055	processed_pseudogene	11,40	0
ENSG00000259493	ENSG00000259493	15	79559256	79560304	processed_pseudogene	11,40	0
ENSG00000260669	ENSG00000260669	14	24209646	24215987	processed_transcript	11,40	0
ENSG00000253667	ENSG00000253667	8	53971231	53974210	processed_pseudogene	11,40	7,06E-02
ENSG00000233028	ENSG00000233028	7	56175634	56176412	processed_pseudogene	11,40	0,2161218
ENSG00000248676	ENSG00000248676	4	99594799	99625913	antisense	11,40	0
ENSG00000243422	RPL23AP49	3	75624686	75625153	processed_pseudogene	11,40	0
ENSG00000235954	TTC28-AS1	22	27919376	28008581	processed_transcript	11,37	0
ENSG00000232860	SMG7-AS1	1	183460874	183472265	processed_transcript	11,37	0
ENSG00000213326	RPS7P11	17	46721582	46722167	processed_pseudogene	11,35	6,27E-03
ENSG00000262831	ENSG00000262831	17	81843165	81843958	antisense	11,35	0
ENSG00000232400	RAD17P1	7	16864267	16866308	processed_pseudogene	11,33	6,27E-03
ENSG00000231841	ENSG00000231841	4	152927446	152928207	processed_pseudogene	11,33	0
ENSG00000279730	SETD8P1	13	18297554	18298733	processed_pseudogene	11,32	0
ENSG00000267649	ENSG00000267649	19	55216660	55221616	lincRNA	11,30	0
ENSG00000271347	ENSG00000271347	15	24991486	24991753	sense_intronic	11,30	0
ENSG00000257954	ENSG00000257954	12	49389516	49390162	processed_pseudogene	11,30	7,06E-02
ENSG00000271327	ENSG00000271327	12	89367807	89369301	lincRNA	11,30	7,06E-02
ENSG00000253608	ENSG00000253608	8	48551567	48698510	antisense	11,30	0

ENSG00000244480	ENSG00000244480	7	30523143	30524535	transcribed_processed_pseudogene	11,30	1,40E-02
ENSG00000272217	ENSG00000272217	6	33246075	33246856	lincRNA	11,30	1,40E-02
ENSG00000272588	ENSG00000272588	4	757022	757740	antisense	11,30	0
ENSG00000234617	SNRK-AS1	3	43349644	43351962	antisense	11,30	0
ENSG00000230212	ENSG00000230212	21	36069642	36126640	sense_intronic	11,27	0
ENSG00000179141	MTUS2-AS1	13	29476515	29490105	antisense	11,26	0,1217903
ENSG00000259185	ENSG00000259185	15	51908902	51909642	antisense	11,26	0
ENSG00000226377	ENSG00000226377	17	32876759	32878637	antisense	11,25	0
ENSG00000237008	LAPTM4BP1	1	153379821	153380799	processed_pseudogene	11,24	1,40E-02
ENSG00000266402	SNHG25	17	64145970	64146476	lincRNA	11,20	0,1392851
ENSG00000275630	ENSG00000275630	14	70822004	70823984	lincRNA	11,20	7,06E-02
ENSG00000214776	ENSG00000214776	12	9467552	9576275	transcribed_unprocessed_pseudogene	11,20	0
ENSG00000271335	ENSG00000271335	10	35314552	35336401	antisense	11,20	0
ENSG00000272983	ENSG00000272983	10	38137337	38144399	lincRNA	11,20	0
ENSG00000253320	AZIN1-AS1	8	102864300	102977876	antisense	11,20	0
ENSG00000250889	LINC01336	5	75047719	75052843	lincRNA	11,20	0
ENSG00000243629	LINC00880	3	157081667	157123004	lincRNA	11,20	0
ENSG00000264443	ENSG00000264443	1	24538802	24556024	lincRNA	11,20	0
ENSG00000215030	RPL13P12	17	17383377	17384012	processed_pseudogene	11,20	0
ENSG00000264982	ENSG00000264982	18	32287437	32290340	antisense	11,19	0
ENSG00000258377	ENSG00000258377	14	49620815	49623480	antisense	11,18	0
ENSG00000274554	ENSG00000274554	12	116948738	116951422	antisense	11,18	0
ENSG00000249996	ENSG00000249996	5	123036271	123054667	antisense	11,18	0
ENSG00000267924	ENSG00000267924	19	23817599	23874701	lincRNA	11,16	0,2161218
ENSG00000230289	ENSG00000230289	9	131516558	131522229	antisense	11,14	0
ENSG00000260755	ENSG00000260755	16	66509437	66510048	lincRNA	11,13	0
ENSG00000233585	ENSG00000233585	X	48775993	48776970	processed_pseudogene	11,11	0
ENSG00000228606	ENSG00000228606	1	160261744	160262778	antisense	11,11	0
ENSG00000272316	ENSG00000272316	6	57908560	57913911	lincRNA	11,11	0
ENSG00000276071	ENSG00000276071	19	36668102	36669404	lincRNA	11,10	0
ENSG00000260578	ENSG00000260578	18	67481791	67484966	lincRNA	11,10	1,40E-02
ENSG00000261416	ENSG00000261416	16	30183505	30184957	antisense	11,10	0
ENSG00000270195	ENSG00000270195	4	1712821	1713622	lincRNA	11,10	0
ENSG00000273369	ENSG00000273369	4	44693946	44694386	antisense	11,10	0
ENSG00000234329	ENSG00000234329	1	45651039	45651826	processed_pseudogene	11,10	0
ENSG00000237278	RLIMP2	1	113125321	113126883	processed_pseudogene	11,10	0
ENSG00000267379	ENSG00000267379	19	14402717	14408723	antisense	11,10	0
ENSG00000273132	ENSG00000273132	6	149852462	149853192	antisense	11,09	0
ENSG00000225798	ENSG00000225798	15	58889967	58894082	antisense	11,08	0
ENSG00000249245	ENSG00000249245	4	145624159	145626035	processed_pseudogene	11,07	0
ENSG00000253327	RAD21-AS1	8	116874424	116876868	antisense	11,06	0
ENSG00000236636	ENSG00000236636	1	227264776	227265207	processed_pseudogene	11,06	1,40E-02
ENSG00000262470	TVP23CP2	16	14200357	14200979	processed_pseudogene	11,05	1,40E-02
ENSG00000277459	ENSG00000277459	11	102109827	102110457	lincRNA	11,05	0
ENSG00000247903	ENSG00000247903	12	26971586	26979582	antisense	11,03	0
ENSG00000267612	ENSG00000267612	19	9730853	9731943	lincRNA	11,00	7,06E-02
ENSG00000261560	ENSG00000261560	16	11881075	11882569	sense_intronic	11,00	0
ENSG00000236426	ENSG00000236426	10	119330233	119336182	antisense	11,00	0
ENSG00000260193	ENSG00000260193	9	136107808	136109424	lincRNA	11,00	0
ENSG00000214544	GTF2IRD2P1	7	73242751	73280119	transcribed_unprocessed_pseudogene	11,00	0
ENSG00000270558	ENSG00000270558	5	37286449	37286977	processed_pseudogene	11,00	1,40E-02
ENSG00000249661	TNRC18P1	4	140641840	140645489	processed_pseudogene	11,00	0
ENSG00000242545	ENSG00000242545	3	59464330	59510678	lincRNA	11,00	0
ENSG00000213226	ENSG00000213226	1	147319110	147320224	processed_pseudogene	11,00	0
ENSG00000214111	ENSG00000214111	X	25029545	25030235	processed_pseudogene	11,00	7,06E-02
ENSG00000258777	HIF1A-AS1	14	61681041	61695823	lincRNA	10,94	0
ENSG00000226544	RPL7P22	5	72725419	72726151	processed_pseudogene	10,94	0

ENSG00000233325	MIPEPP3	13	21298139	21306373	transcribed_unprocessed_pseudogene	10,92	0
ENSG00000229589	ACVR2B-AS1	3	38451027	38454820	antisense	10,91	0
ENSG00000215559	ANKRD20A11P	21	13909574	13980437	transcribed_unprocessed_pseudogene	10,90	0
ENSG00000228798	ENSG00000228798	21	16630827	16640683	lincRNA	10,90	1,40E-02
ENSG00000275413	ENSG00000275413	17	16023323	16023653	antisense	10,90	0
ENSG00000259448	ENSG00000259448	15	31216020	31224445	lincRNA	10,90	0
ENSG00000269951	ENSG00000269951	15	77067654	77068325	sense_intronic	10,90	0
ENSG00000243433	ENSG00000243433	7	151028781	151029754	antisense	10,90	0
ENSG00000271553	ENSG00000271553	7	128667043	128668156	lincRNA	10,90	0
ENSG00000270419	CAHM	6	163413065	163413960	lincRNA	10,90	1,40E-02
ENSG00000229220	ENSG00000229220	1	200147531	200148279	processed_pseudogene	10,90	0
ENSG00000219186	FTH1P19	X	37492021	37492548	unprocessed_pseudogene	10,90	0
ENSG00000251186	ENSG00000251186	4	8453410	8454942	antisense	10,89	0
ENSG00000234118	RPL13AP6	10	110936622	110937233	processed_pseudogene	10,86	0,1836819
ENSG00000273888	FRMD6-AS1	14	51649516	51651744	antisense	10,85	0
ENSG00000204253	HNRNPCP2	2	189923336	189924216	processed_pseudogene	10,84	0
ENSG00000268635	ENSG00000268635	11	77473371	77477030	antisense	10,83	0
ENSG00000256747	ENSG00000256747	12	27779821	27781067	antisense	10,83	0
ENSG00000182397	DNM1P46	15	99790156	99806927	transcribed_unprocessed_pseudogene	10,81	0
ENSG00000259363	ENSG00000259363	15	99807023	99877148	lincRNA	10,81	0
ENSG00000258325	ENSG00000258325	12	2796877	2812902	antisense	10,81	0
ENSG00000226723	ENSG00000226723	1	192246708	192247487	processed_pseudogene	10,81	0
ENSG00000232907	DLGAP4-AS1	20	36507702	36573391	antisense	10,81	0
ENSG00000237015	ENSG00000237015	22	29260889	29262037	antisense	10,80	0
ENSG00000241717	VWFP1	22	16690103	16704477	transcribed_unprocessed_pseudogene	10,80	0
ENSG00000270083	ENSG00000270083	22	42089630	42090028	sense_intronic	10,80	0
ENSG00000233930	KRTAP5-AS1	11	1571353	1599184	antisense	10,80	0
ENSG00000255200	ENSG00000255200	11	65174117	65176470	transcribed_processed_pseudogene	10,80	0
ENSG00000251191	LINC00589	8	29673922	29748109	lincRNA	10,80	0
ENSG00000229153	EPHA1-AS1	7	143407813	143523449	antisense	10,80	1,40E-02
ENSG00000213608	SLC25A14P1	4	83477524	83478424	processed_pseudogene	10,80	0,2161218
ENSG00000229915	ENSG00000229915	2	238427077	238427729	antisense	10,80	0
ENSG00000272211	ENSG00000272211	2	196151263	196154881	antisense	10,80	0
ENSG00000233040	FAM204BP	1	197746751	197747451	processed_pseudogene	10,80	0
ENSG00000204706	MAMDC2-AS1	9	70033921	70175888	antisense	10,79	3,30E-03
ENSG00000225093	RPL3P7	6	108004357	108005568	processed_pseudogene	10,79	0
ENSG00000251458	ENSG00000251458	5	176124210	176131461	lincRNA	10,79	0
ENSG00000268218	ENSG00000268218	16	89268104	89273044	antisense	10,73	0
ENSG00000225544	ENSG00000225544	22	21885282	21885673	processed_pseudogene	10,73	0
ENSG00000227376	FTH1P16	11	77734475	77735026	processed_pseudogene	10,73	0,1392851
ENSG00000226342	NMD3P1	7	63908966	63910453	processed_pseudogene	10,72	0
ENSG00000236229	VEZF1P1	3	193155456	193157208	processed_pseudogene	10,72	0
ENSG00000232828	ENSG00000232828	X	48698963	48737163	antisense	10,72	0
ENSG00000266373	ENSG00000266373	18	3580169	3580754	processed_pseudogene	10,70	0
ENSG00000257331	ENSG00000257331	12	45063473	45065351	processed_pseudogene	10,70	0,2161218
ENSG00000270578	ENSG00000270578	11	95145437	95146004	processed_pseudogene	10,70	0
ENSG00000277290	ENSG00000277290	11	243099	243483	processed_pseudogene	10,70	1,40E-02
ENSG00000172965	MIR4435-2HG	2	111196350	111495100	lincRNA	10,70	0
ENSG00000253406	ENSG00000253406	5	149216523	149276805	antisense	10,69	0
ENSG00000179859	ENSG00000179859	17	7913324	7915953	antisense	10,66	0
ENSG00000226017	PRICKLE2-AS3	3	64187544	64200965	antisense	10,64	0
ENSG00000224165	DNAJC27-AS1	2	24971390	25039694	antisense	10,64	0
ENSG00000258944	ENSG00000258944	14	73272182	73274081	antisense	10,63	0
ENSG00000233360	ENSG00000233360	22	37641832	37658377	antisense	10,61	0
ENSG00000214708	ENSG00000214708	17	32141226	32143135	antisense	10,61	0

ENSG00000213976	ENSG00000213976	19	21382865	21387177	unprocessed_pseudogene	10,61	0
ENSG00000234880	LINC00163	21	44989864	44994086	lincRNA	10,60	7,06E-02
ENSG00000266743	ENSG00000266743	18	76173304	76177738	lincRNA	10,60	0,2161218
ENSG00000270580	ENSG00000270580	16	15104723	15131601	processed_transcript	10,60	0
ENSG00000224116	INHBA-AS1	7	41693916	41779388	antisense	10,60	0
ENSG00000243410	ENSG00000243410	3	64011964	64016246	antisense	10,60	0
ENSG00000273174	ENSG00000273174	3	129123439	129124003	antisense	10,60	0
ENSG00000274642	ENSG00000274642	1	120197085	120319680	unprocessed_pseudogene	10,60	0
ENSG00000214973	CHCHD3P3	1	27200834	27201473	processed_pseudogene	10,58	0
ENSG00000261879	ENSG00000261879	17	5192084	5248069	antisense	10,57	0
ENSG00000259299	ENSG00000259299	15	29233673	29235137	processed_pseudogene	10,52	0
ENSG00000257550	ENSG00000257550	12	53513984	53517608	antisense	10,52	0
ENSG00000223573	TINCR	19	5558167	5568034	lincRNA	10,50	6,27E-03
ENSG00000277806	ENSG00000277806	19	43976815	43977448	sense_intronic	10,50	0
ENSG00000259843	ENSG00000259843	16	50046429	50066224	antisense	10,50	0
ENSG00000240163	ENSG00000240163	15	60390371	60390682	processed_pseudogene	10,50	0
ENSG00000260645	ENSG00000260645	6	80466958	80469080	lincRNA	10,50	0
ENSG00000227486	ENSG00000227486	X	55908123	56015173	lincRNA	10,50	0
ENSG00000271933	ENSG00000271933	10	84138420	84140582	antisense	10,50	0
ENSG00000228395	ENSG00000228395	9	128528901	128552410	antisense	10,49	0
ENSG00000248508	SRP14-AS1	15	40039311	40067290	lincRNA	10,49	0
ENSG00000253341	ENSG00000253341	8	74603244	74604244	processed_pseudogene	10,48	0
ENSG00000205583	STAG3L1	7	75359194	75395383	transcribed_unprocessed_pseudogene	10,46	0
ENSG00000268912	ENSG00000268912	19	58428632	58431148	lincRNA	10,45	0
ENSG00000215105	TTC3P1	X	75740831	75746911	processed_pseudogene	10,45	0
ENSG00000272293	ENSG00000272293	8	450714	451343	antisense	10,44	0
ENSG00000259238	ENSG00000259238	15	59688517	59689418	antisense	10,41	0
ENSG00000235890	TSPEAR-AS1	21	44506807	44516575	antisense	10,40	0
ENSG00000237807	ENSG00000237807	8	53515171	53523931	lincRNA	10,40	0
ENSG00000232713	ENSG00000232713	2	60938204	60938604	processed_pseudogene	10,40	0
ENSG00000231228	ENSG00000231228	5	180982273	180982611	processed_pseudogene	10,36	0
ENSG00000229759	MRPS18AP1	3	48256350	48256938	processed_pseudogene	10,36	0
ENSG00000259905	PWRN1	15	24493137	24652130	lincRNA	10,35	0
ENSG00000177770	CDKN2AIPNLP1	1	226493188	226493489	processed_pseudogene	10,32	0
ENSG00000251417	ENSG00000251417	16	28802743	28817828	lincRNA	10,30	0
ENSG00000177340	ENSG00000177340	12	31324316	31325829	antisense	10,30	0
ENSG00000236908	ENSG00000236908	12	3318718	3325343	lincRNA	10,30	7,06E-02
ENSG00000275180	ENSG00000275180	12	62603909	62604399	lincRNA	10,30	1,40E-02
ENSG00000230224	PHBP9	10	100248271	100249095	processed_pseudogene	10,30	0
ENSG00000235475	LINC01372	7	67335976	67340024	lincRNA	10,30	7,06E-02
ENSG00000236708	ENSG00000236708	7	3264032	3302452	antisense	10,29	0
ENSG00000213225	ENSG00000213225	2	130229379	130232106	transcribed_processed_pseudogene	10,28	0
ENSG00000269604	ENSG00000269604	19	4791745	4795559	antisense	10,27	0
ENSG00000226352	ENSG00000226352	13	19674624	19675884	antisense	10,27	0
ENSG00000230280	HNRNPA1P59	1	202911812	202912729	processed_pseudogene	10,27	0
ENSG00000233728	ENSG00000233728	1	37799720	37800879	antisense	10,27	0
ENSG00000222043	ENSG00000222043	2	177264359	177265515	antisense	10,23	0
ENSG00000273055	ENSG00000273055	7	107942116	107942740	antisense	10,23	0
ENSG00000241990	PRR34-AS1	22	46053705	46057210	antisense	10,20	0
ENSG00000276073	ENSG00000276073	20	33985617	33988989	lincRNA	10,20	0
ENSG00000268743	ENSG00000268743	19	16844025	16846473	sense_intronic	10,20	1,40E-02
ENSG00000281468	ENSG00000281468	19	27802838	27803472	sense_intronic	10,20	0
ENSG00000270246	ENSG00000270246	15	25041918	25042428	sense_intronic	10,20	0
ENSG00000275552	ENSG00000275552	14	22595808	22598946	antisense	10,20	0
ENSG00000240859	ENSG00000240859	7	149597	155465	lincRNA	10,20	0
ENSG00000243230	ENSG00000243230	7	129209775	129213545	antisense	10,20	1,40E-02
ENSG00000196951	SCOC-AS1	4	140283724	140373381	antisense	10,20	0

ENSG00000273654	ENSG00000273654	19	18022403	18032099	transcribed_processed_pseudogene	10,19	0
ENSG00000272689	ENSG00000272689	22	30421206	30421536	sense_intronic	10,18	0
ENSG00000227409	ZMYM4-AS1	1	35358822	35366077	antisense	10,18	0
ENSG00000230550	ENSG00000230550	1	204141408	204143009	antisense	10,18	0
ENSG00000240135	PSMD12P	3	72876737	72878102	unprocessed_pseudogene	10,18	0
ENSG00000236257	EI24P2	1	158454198	158455273	processed_pseudogene	10,18	6,27E-03
ENSG00000235174	RPL39P3	6	73373108	73373263	processed_pseudogene	10,17	0
ENSG00000223473	ENSG00000223473	7	66491049	66493566	lincRNA	10,17	0
ENSG00000236432	ENSG00000236432	2	227221052	227325201	antisense	10,17	0
ENSG00000226054	MEMO1P1	21	36130489	36131376	processed_pseudogene	10,17	0
ENSG00000241015	TPM3P9	19	53431984	53444670	transcribed_processed_pseudogene	10,16	0
ENSG00000259330	INAFM2	15	40325216	40326715	antisense	10,13	0
ENSG00000270975	MAGOH3P	14	68861762	68862525	unprocessed_pseudogene	10,13	2,95E-02
ENSG00000198106	ENSG00000198106	16	29291220	29372110	lincRNA	10,10	0
ENSG00000263013	ENSG00000263013	16	10938886	10940044	sense_intronic	10,10	0
ENSG00000277559	ENSG00000277559	16	54290965	54292422	lincRNA	10,10	0
ENSG00000237181	ENSG00000237181	7	603185	608482	antisense	10,10	0
ENSG00000146001	PCDHB18P	5	141234333	141237529	transcribed_unprocessed_pseudogene	10,10	0
ENSG00000249911	LINC01265	5	38710367	38720273	lincRNA	10,10	1,40E-02
ENSG00000255769	GOLGA2P10	15	82472993	82513950	transcribed_unprocessed_pseudogene	10,09	0
ENSG00000227484	ENSG00000227484	22	50314631	50316008	antisense	10,08	0
ENSG00000237773	ENSG00000237773	7	17279834	17299357	antisense	10,07	0
ENSG00000271851	ENSG00000271851	17	9553323	9555696	sense_intronic	10,06	0
ENSG00000269696	ENSG00000269696	19	56545566	56567411	antisense	10,06	0
ENSG00000233874	ENSG00000233874	X	109568478	109570289	processed_pseudogene	10,03	0
ENSG00000231794	ENSG00000231794	7	135198401	135209837	antisense	10,03	0
ENSG00000259877	ENSG00000259877	16	89215211	89217653	antisense	10,03	0
ENSG00000229107	ABHD17AP4	22	20667836	20670984	processed_pseudogene	10,00	0,2161218
ENSG00000279278	ENSG00000279278	22	22264601	22273020	lincRNA	10,00	0,2161218
ENSG00000237259	ENSG00000237259	20	18313765	18315111	antisense	10,00	0
ENSG00000276449	ENSG00000276449	19	57449689	57453011	antisense	10,00	0
ENSG00000264608	ENSG00000264608	17	28601827	28602284	sense_intronic	10,00	1,40E-02
ENSG00000260884	ENSG00000260884	16	74313337	74315634	lincRNA	10,00	0
ENSG00000259523	ENSG00000259523	15	29822631	29824081	antisense	10,00	0
ENSG00000271417	SNHG24	14	100967652	100987280	lincRNA	10,00	7,06E-02
ENSG00000277156	ENSG00000277156	14	19267115	19268164	processed_pseudogene	10,00	0
ENSG00000236583	ENSG00000236583	8	99233048	99234459	processed_pseudogene	10,00	0,2161218
ENSG00000232415	ENSG00000232415	7	74059576	74062284	antisense	10,00	0
ENSG00000272087	ENSG00000272087	3	158693120	158693768	lincRNA	10,00	0
ENSG00000236255	ENSG00000236255	2	117833937	117841658	lincRNA	10,00	0
ENSG00000236682	ENSG00000236682	2	127389130	127400580	lincRNA	10,00	0
ENSG00000225492	GBP1P1	1	89407679	89426243	transcribed_unprocessed_pseudogene	10,00	0
ENSG00000229956	ZRANB2-AS2	1	71081324	71489976	processed_transcript	10,00	0
ENSG00000259773	ENSG00000259773	15	50746709	50749829	antisense	9,97	0
ENSG00000255437	ENSG00000255437	8	14022687	14023422	processed_pseudogene	9,95	0
ENSG00000260793	ENSG00000260793	17	44221401	44223710	antisense	9,92	3,30E-03
ENSG00000270964	ENSG00000270964	15	67541072	67542604	lincRNA	9,91	0
ENSG00000227407	ENSG00000227407	19	54430654	54434698	antisense	9,90	1,40E-02
ENSG00000255547	RPA2P3	11	100336856	100337625	processed_pseudogene	9,90	0
ENSG00000260368	ENSG00000260368	8	100913247	100914388	sense_overlapping	9,90	0
ENSG00000237669	HCG4P3	6	30006121	30007116	unprocessed_pseudogene	9,90	0
ENSG00000225986	UBXN10-AS1	1	20184242	20186486	antisense	9,90	0
ENSG00000272523	LINC01023	5	108727825	108728260	lincRNA	9,87	0

ENSG00000266145	RHOT1P1	18	14222008	14222331	processed_pseudogene	9,84	4,86E-02
ENSG00000259099	ENSG00000259099	12	56380361	56382711	processed_pseudogene	9,83	1,95E-02
ENSG00000263535	AK4P1	17	31345521	31346187	processed_pseudogene	9,83	0
ENSG00000258017	ENSG00000258017	12	49127782	49147869	antisense	9,83	0
ENSG00000230707	ENSG00000230707	X	140091743	140092561	processed_pseudogene	9,83	0
ENSG00000249215	ENSG00000249215	5	139494256	139496116	processed_pseudogene	9,82	0
ENSG00000260075	NSFP1	17	46372855	46487141	unprocessed_pseudogene	9,82	0
ENSG00000250073	ENSG00000250073	11	124759129	124765936	antisense	9,80	1,40E-02
ENSG00000224934	ENSG00000224934	10	99431191	99438117	lincRNA	9,80	1,40E-02
ENSG00000228010	ENSG00000228010	7	6663974	6708901	antisense	9,80	0
ENSG00000218713	ENSG00000218713	6	53206598	53208697	processed_pseudogene	9,80	1,40E-02
ENSG00000227214	HCG15	6	28986203	28987484	antisense	9,80	0
ENSG00000250579	ENSG00000250579	5	5132780	5140054	antisense	9,80	7,06E-02
ENSG00000225808	DNAJC19P5	2	177229191	177229506	processed_pseudogene	9,80	0
ENSG00000225953	SATB2-AS1	2	199457700	199476935	antisense	9,80	0
ENSG00000230207	RPL4P5	9	7477045	7478320	processed_pseudogene	9,78	0
ENSG00000281357	ARRDC3-AS1	5	91380349	91439085	lincRNA	9,77	0
ENSG00000213036	ENSG00000213036	1	214482813	214483387	processed_pseudogene	9,76	0
ENSG00000261243	ENSG00000261243	16	84495599	84497495	antisense	9,76	0
ENSG00000259955	ENSG00000259955	16	25238318	25239287	antisense	9,73	0
ENSG00000218857	ENSG00000218857	6	131184325	131185486	processed_pseudogene	9,72	0
ENSG00000256967	ENSG00000256967	12	7129079	7131198	antisense	9,70	0
ENSG00000230736	ENSG00000230736	22	32376664	32384343	lincRNA	9,70	0
ENSG00000231711	LINC00899	22	46039907	46044853	processed_transcript	9,70	0
ENSG00000268734	ENSG00000268734	19	54890673	54891420	lincRNA	9,70	0
ENSG00000269546	CLIC4P2	19	6562123	6563848	processed_pseudogene	9,70	0
ENSG00000267745	ENSG00000267745	17	35406684	35409768	processed_transcript	9,70	0
ENSG00000250616	ENSG00000250616	16	30096430	30104116	antisense	9,70	0
ENSG00000236242	MYO16-AS1	13	109163902	109201483	antisense	9,70	7,06E-02
ENSG00000257824	ENSG00000257824	12	54543111	54544105	antisense	9,70	1,40E-02
ENSG00000273008	ENSG00000273008	10	43136824	43138334	lincRNA	9,70	0
ENSG00000253930	ENSG00000253930	8	23189279	23190675	antisense	9,70	0
ENSG00000245205	EEF1A1P4	12	19456244	19457648	processed_pseudogene	9,69	9,18E-02
ENSG00000233058	LINC00884	3	194487454	194518279	antisense	9,69	0
ENSG00000235257	ITGA9-AS1	3	37745432	37861780	processed_transcript	9,69	0
ENSG00000172250	SERHL	22	42500579	42512560	processed_transcript	9,69	0
ENSG00000238082	ENSG00000238082	2	178454716	178455428	processed_pseudogene	9,68	0
ENSG00000232260	BTF3L4P1	21	17518526	17518993	processed_pseudogene	9,67	0
ENSG00000250615	ENSG00000250615	5	77073881	77074520	antisense	9,66	0
ENSG00000276791	ENSG00000276791	16	2777319	2780568	lincRNA	9,65	0
ENSG00000240429	LRRFIP1P1	3	179259028	179261273	processed_pseudogene	9,62	0
ENSG00000241506	PSMC1P1	3	68635705	68637024	processed_pseudogene	9,62	0
ENSG00000256458	ENSG00000256458	2	203328459	203329226	antisense	9,62	0
ENSG00000213303	ENSG00000213303	19	11523436	11523831	processed_pseudogene	9,60	0,2161218
ENSG00000269535	ENSG00000269535	19	52300693	52345229	antisense	9,60	1,40E-02
ENSG00000282458	WASH5P	19	60951	71626	transcribed_processed_pseudogene	9,60	0
ENSG00000262663	ENSG00000262663	17	82918282	82918785	lincRNA	9,60	0
ENSG00000229670	PKP4P1	2	209178966	209181123	unprocessed_pseudogene	9,59	0
ENSG00000225205	ENSG00000225205	2	172480840	172556596	antisense	9,59	0
ENSG00000277959	ENSG00000277959	10	131971202	131971533	antisense	9,58	0
ENSG00000257303	ENSG00000257303	12	56300142	56314808	antisense	9,58	0
ENSG00000259163	ENSG00000259163	14	90642638	90648894	antisense	9,56	0
ENSG00000261976	ENSG00000261976	17	50840057	50841626	antisense	9,54	0
ENSG00000223528	ENSG00000223528	10	126988095	127026507	antisense	9,53	0
ENSG00000272153	ENSG00000272153	1	3785008	3785538	antisense	9,53	0
ENSG00000233578	EIF4EP1	20	5548840	5549481	processed_pseudogene	9,50	1,40E-02
ENSG00000229980	TOB1-AS1	17	50866679	50909737	processed_transcript	9,50	0
ENSG00000261868	MFSD1P1	17	4301372	4302698	processed_pseudogene	9,50	0,2161218

ENSG00000262905	ENSG00000262905	17	552566	553417	antisense	9,50	0
ENSG00000255885	ENSG00000255885	12	7870852	7871370	processed_pseudogene	9,50	0
ENSG00000264204	AGAP7P	10	46109621	46131358	unprocessed_pseudogene	9,50	0
ENSG00000237339	LINC01502	9	135574935	135587112	lincRNA	9,50	0
ENSG00000281189	GHET1	7	148987527	148989432	lincRNA	9,50	0
ENSG00000224666	ENSG00000224666	6	36386831	36393462	antisense	9,50	7,06E-02
ENSG00000232352	SEMA3B-AS1	3	50266641	50267371	antisense	9,50	0
ENSG00000270096	ENSG00000270096	3	169794962	169796213	antisense	9,50	0
ENSG00000226553	ENSG00000226553	2	187001876	187003377	processed_pseudogene	9,50	0
ENSG00000259684	ENSG00000259684	15	50557601	50560500	antisense	9,46	0
ENSG00000258794	DUX4L27	12	34208415	34209675	processed_pseudogene	9,45	3,30E-03
ENSG00000233203	ENSG00000233203	1	54887563	54888850	antisense	9,45	0
ENSG00000233029	ENSG00000233029	1	121090289	121097655	antisense	9,42	0
ENSG00000198416	ZNF658B	9	39443815	39508885	transcribed_unprocessed_pseudogene	9,41	0
ENSG00000229083	PSMA6P2	X	12825840	12826833	processed_pseudogene	9,40	1,40E-02
ENSG00000269901	ENSG00000269901	16	87362536	87367476	antisense	9,40	0
ENSG00000257596	ENSG00000257596	12	54262615	54279063	antisense	9,40	1,40E-02
ENSG00000254941	ENSG00000254941	11	124807822	124808269	processed_pseudogene	9,40	0
ENSG00000228339	AMD1P1	10	20350049	20351100	processed_pseudogene	9,40	7,06E-02
ENSG00000253688	ENSG00000253688	8	48552015	48556441	lincRNA	9,40	0
ENSG00000146722	ENSG00000146722	7	75391955	75395434	transcribed_unprocessed_pseudogene	9,40	0
ENSG00000260091	ENSG00000260091	4	119409333	119410233	lincRNA	9,40	0
ENSG00000272927	ENSG00000272927	4	661209	661945	antisense	9,40	0
ENSG00000272967	ENSG00000272967	3	119579212	119579650	antisense	9,40	0
ENSG00000236155	ENSG00000236155	1	26209741	26229840	transcribed_unprocessed_pseudogene	9,40	0
ENSG00000232611	ENSG00000232611	X	136840931	136847797	lincRNA	9,40	0
ENSG00000225259	ST13P6	8	133408006	133409107	processed_pseudogene	9,39	0
ENSG00000237970	TMEM161BP1	10	37337415	37338871	processed_pseudogene	9,38	0
ENSG00000238280	ENSG00000238280	10	62793562	62805887	antisense	9,38	0
ENSG00000249855	EEF1A1P19	5	43495073	43496454	processed_pseudogene	9,35	0
ENSG00000267698	ENSG00000267698	19	36014508	36045972	antisense	9,33	0
ENSG00000272744	ENSG00000272744	4	183516894	183517527	lincRNA	9,33	2,95E-02
ENSG00000259918	NDUFA5P11	16	47598654	47598996	processed_pseudogene	9,31	0
ENSG00000267100	ILF3-AS1	19	10651862	10653844	lincRNA	9,30	0
ENSG00000276727	ENSG00000276727	12	57229498	57230198	antisense	9,30	0
ENSG00000268038	ENSG00000268038	19	22615557	22623971	lincRNA	9,30	0
ENSG00000205890	ENSG00000205890	16	3032481	3039133	antisense	9,30	7,06E-02
ENSG00000223518	CSNK1A1P1	15	36798596	36818459	transcribed_processed_pseudogene	9,30	1,40E-02
ENSG00000259732	ENSG00000259732	15	59121034	59133250	sense_overlapping	9,30	0
ENSG00000255142	ENSG00000255142	11	781645	782105	lincRNA	9,30	0
ENSG00000255158	ENSG00000255158	11	665910	678391	antisense	9,30	0
ENSG00000220739	ENSG00000220739	6	144708106	144708497	processed_pseudogene	9,30	1,40E-02
ENSG00000249363	ENSG00000249363	5	145728360	145729349	processed_pseudogene	9,30	0
ENSG00000272156	ENSG00000272156	2	54082554	54085066	lincRNA	9,30	0
ENSG00000174912	METTL15P1	3	156713884	156714928	processed_pseudogene	9,30	0
ENSG00000232640	ENSG00000232640	6	169725091	169725854	antisense	9,25	0
ENSG00000275223	ENSG00000275223	20	33655701	33656423	lincRNA	9,25	0
ENSG00000266473	ENSG00000266473	17	67244837	67245806	antisense	9,25	0
ENSG00000205534	SMG1P2	16	29527568	29594966	unprocessed_pseudogene	9,25	0
ENSG00000249014	HMGN2P4	5	76242024	76242404	processed_pseudogene	9,24	0
ENSG00000262899	ENSG00000262899	16	3307573	3308393	antisense	9,23	0
ENSG00000275055	ENSG00000275055	19	52049007	52049754	antisense	9,21	0
ENSG00000254419	ENSG00000254419	20	58710795	58711633	sense_intronic	9,20	0
ENSG00000269938	ENSG00000269938	12	123968023	123968579	sense_intronic	9,20	0
ENSG00000253358	ENSG00000253358	8	91016588	91018655	antisense	9,20	0

ENSG00000228960	OR2A9P	7	144294480	144300934	transcribed_unprocessed_pseudogene	9,20	0
ENSG00000271789	ENSG00000271789	6	111297126	111298510	lincRNA	9,20	1,40E-02
ENSG00000242858	ENSG00000242858	5	83746388	83746989	processed_pseudogene	9,20	0
ENSG00000249637	ENSG00000249637	5	140370891	140401367	antisense	9,20	0
ENSG00000230069	LRRC37A15P	4	102727274	102730721	processed_pseudogene	9,20	0
ENSG00000240137	ERICH6-AS1	3	150703564	150720146	antisense	9,20	0
ENSG00000271992	ENSG00000271992	1	70445071	70445536	lincRNA	9,20	7,06E-02
ENSG00000273443	ENSG00000273443	1	1062208	1063288	lincRNA	9,20	7,06E-02
ENSG00000228906	ENSG00000228906	X	73948973	73949558	lincRNA	9,20	0
ENSG00000233895	ENSG00000233895	20	19757708	19809675	lincRNA	9,19	0
ENSG00000255351	ENSG00000255351	11	11352426	11353307	antisense	9,16	0
ENSG00000186056	MATN1-AS1	1	30718504	30726827	antisense	9,14	0
ENSG00000241472	PTPRG-AS1	3	62260865	62369330	processed_transcript	9,13	0
ENSG00000235313	HM13-IT1	20	31563166	31564076	sense_intronic	9,10	0
ENSG00000260510	ENSG00000260510	16	20743663	20766620	antisense	9,10	0
ENSG00000258768	ENSG00000258768	14	20260480	20264308	lincRNA	9,10	0
ENSG00000256185	ENSG00000256185	12	26335864	26336950	3prime_overlapping_ncrna	9,10	0
ENSG00000232600	TONSL-AS1	8	144437675	144439971	antisense	9,10	0
ENSG00000242607	RPS3AP34	8	12570350	12571130	processed_pseudogene	9,10	0
ENSG00000278275	ENSG00000278275	8	128010188	128010428	processed_pseudogene	9,10	0
ENSG00000250934	ENSG00000250934	3	126266796	126291279	lincRNA	9,10	0
ENSG00000281406	BLACAT1	1	205434886	205437879	lincRNA	9,10	0
ENSG00000236337	FMR1-IT1	X	147946941	147947583	sense_intronic	9,10	0
ENSG00000178082	TWF1P1	17	29203426	29204474	processed_pseudogene	9,10	0
ENSG00000260774	ENSG00000260774	5	213898	217279	processed_transcript	9,09	0
ENSG00000232934	ENSG00000232934	10	112395813	112425589	antisense	9,06	0
ENSG00000253541	SEPT10P1	8	56476826	56477987	processed_pseudogene	9,05	0
ENSG00000235411	ENSG00000235411	2	20239892	20241046	processed_pseudogene	9,03	0
ENSG00000267092	ENSG00000267092	19	1508375	1508963	antisense	9,00	3,30E-03
ENSG00000267898	ENSG00000267898	19	48963975	48965158	lincRNA	9,00	1,40E-02
ENSG00000269792	ENSG00000269792	19	39532412	39534422	antisense	9,00	0
ENSG00000267404	ENSG00000267404	18	36273574	36274599	processed_pseudogene	9,00	0
ENSG00000261499	ENSG00000261499	17	36274191	36320547	unprocessed_pseudogene	9,00	0
ENSG00000232224	LINC00202-1	10	26931206	26942001	lincRNA	9,00	0
ENSG00000119440	LCN1P1	9	133224905	133228591	unprocessed_pseudogene	9,00	7,06E-02
ENSG00000215022	ENSG00000215022	6	13264861	13295586	antisense	9,00	0
ENSG00000220635	KRASP1	6	54770583	54771134	processed_pseudogene	9,00	0
ENSG00000260296	ENSG00000260296	4	40166675	40167831	sense_overlapping	9,00	0,3318209
ENSG00000239799	ITIH4-AS1	3	52823935	52825314	antisense	9,00	0
ENSG00000243849	CFAP44-AS1	3	113403991	113433992	antisense	9,00	0
ENSG00000227033	ENSG00000227033	2	231978488	232015720	antisense	9,00	0
ENSG00000271947	ENSG00000271947	2	6905724	6906301	lincRNA	9,00	1,40E-02
ENSG00000274010	ENSG00000274010	2	127840606	127841763	processed_pseudogene	9,00	0,2161218
ENSG00000226085	UQCRFS1P1	22	39875289	39876108	processed_pseudogene	8,97	0
ENSG00000228051	ENSG00000228051	X	80560146	80562054	processed_pseudogene	8,96	0,1392851
ENSG00000260853	ENSG00000260853	16	28956687	28966883	antisense	8,96	0
ENSG00000270696	ENSG00000270696	2	75660462	75662208	antisense	8,95	0
ENSG00000232549	SRD5A1P1	X	139446891	139447676	processed_pseudogene	8,93	0
ENSG00000268798	ENSG00000268798	19	1440839	1441938	lincRNA	8,91	0
ENSG00000260747	ENSG00000260747	18	68277802	68278243	processed_pseudogene	8,90	0
ENSG00000232775	BMS1P22	22	15805263	15820884	transcribed_unprocessed_pseudogene	8,90	0
ENSG00000267707	ENSG00000267707	18	37243776	37247506	antisense	8,90	7,06E-02
ENSG00000266830	ENSG00000266830	17	28246454	28248006	lincRNA	8,90	0
ENSG00000258907	ENSG00000258907	12	55654558	55655168	processed_pseudogene	8,90	7,06E-02
ENSG00000269997	ENSG00000269997	12	123966077	123966629	sense_intronic	8,90	0
ENSG00000185904	LINC00839	10	42475543	42495336	lincRNA	8,90	7,06E-02

ENSG00000272764	ENSG00000272764	10	5813985	5814441	lincRNA	8,90	0
ENSG00000254088	SLC2A3P4	8	86503591	86505061	processed_pseudogene	8,90	0
ENSG00000215492	HNRNPA1P7	18	32412214	32413176	processed_pseudogene	8,88	4,86E-02
ENSG00000258282	BTF3P2	14	28673241	28673727	processed_pseudogene	8,88	2,95E-02
ENSG00000236088	COX10-AS1	17	13756478	14069495	processed_transcript	8,87	0
ENSG00000186704	DTX2P1	7	76978617	77004308	transcribed_unprocessed_pseudogene	8,87	0
ENSG00000238018	ENSG00000238018	2	54661011	54680045	antisense	8,86	0
ENSG00000255139	ENSG00000255139	11	59616429	59639861	antisense	8,85	0
ENSG00000229586	TNPO1P3	17	20481330	20483991	processed_pseudogene	8,85	0
ENSG00000258428	ENSG00000258428	14	56633244	56648658	antisense	8,83	0
ENSG00000278367	ENSG00000278367	20	35174355	35174919	antisense	8,83	0
ENSG00000226964	RHEBP2	10	47706203	47706802	processed_pseudogene	8,82	0
ENSG00000273017	ENSG00000273017	21	29359002	29359453	sense_intronic	8,80	7,06E-02
ENSG00000264736	BDP1P	18	77435253	77437242	processed_pseudogene	8,80	1,40E-02
ENSG00000236194	ENSG00000236194	17	42270517	42272683	sense_intronic	8,80	0
ENSG00000260306	ENSG00000260306	16	21794095	21795759	lincRNA	8,80	0
ENSG00000259475	ENSG00000259475	15	98954149	99105824	antisense	8,80	0
ENSG00000259676	ENSG00000259676	15	88797413	88798734	lincRNA	8,80	0
ENSG00000243250	RPS6P16	11	112235371	112236025	processed_pseudogene	8,80	0
ENSG00000256091	MTRF1LP1	11	113711720	113712398	processed_pseudogene	8,80	0
ENSG00000264299	ENSG00000264299	11	125957900	125958652	antisense	8,80	0
ENSG00000271830	ENSG00000271830	8	103481266	103481619	lincRNA	8,80	1,40E-02
ENSG00000226824	ENSG00000226824	7	66654538	66669855	sense_intronic	8,80	0
ENSG00000213386	ENSG00000213386	5	172762521	172763258	processed_pseudogene	8,80	0
ENSG00000250725	ENSG00000250725	4	165664350	165665671	processed_pseudogene	8,80	0
ENSG00000224232	ENSG00000224232	2	241478316	241478993	processed_pseudogene	8,80	1,40E-02
ENSG00000261773	ENSG00000261773	X	154517840	154518631	sense_overlapping	8,80	7,06E-02
ENSG00000259287	ENSG00000259287	15	33858602	33864825	antisense	8,80	0
ENSG00000271828	ENSG00000271828	5	56927874	56929573	antisense	8,79	0
ENSG00000253837	ENSG00000253837	8	23336171	23366125	processed_transcript	8,79	0
ENSG00000271869	ENSG00000271869	8	30155830	30156232	lincRNA	8,77	0,2161218
ENSG00000248668	OXCT1-AS1	5	41870030	41872241	processed_transcript	8,76	0
ENSG00000234928	ENSG00000234928	22	23433564	23435071	lincRNA	8,76	0
ENSG00000233967	ENSG00000233967	6	80443344	80465927	lincRNA	8,76	0
ENSG00000272812	ENSG00000272812	8	233119	233692	sense_intronic	8,75	0
ENSG00000254502	ENSG00000254502	11	86195132	86196266	processed_pseudogene	8,74	0
ENSG00000182347	PDSS1P1	9	5084999	5086112	processed_pseudogene	8,73	0,0358681
ENSG00000236814	ENSG00000236814	8	56050038	56051207	processed_pseudogene	8,71	0
ENSG00000237977	EIF4HP2	22	30902219	30902781	processed_pseudogene	8,71	3,30E-03
ENSG00000270878	ENSG00000270878	14	63594031	63595291	processed_pseudogene	8,71	0
ENSG00000269559	ENSG00000269559	4	74156511	74158373	antisense	8,70	0
ENSG00000274717	ENSG00000274717	22	42791814	42794313	processed_pseudogene	8,70	0
ENSG00000266378	ENSG00000266378	17	14327335	14329474	lincRNA	8,70	7,06E-02
ENSG00000247809	NR2F2-AS1	15	96110040	96327361	antisense	8,70	0
ENSG00000258303	ENSG00000258303	12	93836167	93838038	sense_intronic	8,70	0,2161218
ENSG00000273568	ENSG00000273568	12	131934642	131934928	sense_intronic	8,70	0
ENSG00000228828	ENSG00000228828	10	37818606	37820982	processed_pseudogene	8,70	7,06E-02
ENSG00000253523	ENSG00000253523	8	58031334	58032682	lincRNA	8,70	0
ENSG00000234484	ENSG00000234484	6	132752675	132753951	antisense	8,70	1,40E-02
ENSG00000250986	ENSG00000250986	4	3758748	3763390	lincRNA	8,70	0
ENSG00000203855	HSD3BP4	1	119564066	119572067	unprocessed_pseudogene	8,70	7,06E-02
ENSG00000274895	ENSG00000274895	1	213983793	213986419	antisense	8,70	7,06E-02
ENSG00000261556	SMG1P7	16	70219574	70245616	transcribed_unprocessed_pseudogene	8,66	0
ENSG00000224843	LINC00240	6	26956992	27023924	lincRNA	8,64	3,30E-03
ENSG00000233432	ENSG00000233432	1	121502344	121503891	processed_pseudogene	8,64	0
ENSG00000233635	ENSG00000233635	17	63193930	63339053	antisense	8,61	0
ENSG00000273188	ENSG00000273188	22	50191724	50192402	antisense	8,60	0
ENSG00000231125	ENSG00000231125	21	29058073	29060095	sense_intronic	8,60	1,40E-02

ENSG00000259000	DOCK11P1	14	44860316	44866247	processed_pseudogene	8,60	0
ENSG00000228661	ENSG00000228661	11	3854318	3855509	antisense	8,60	0
ENSG00000255342	ENSG00000255342	11	123135923	123136376	processed_pseudogene	8,60	7,06E-02
ENSG00000164556	FAM183BP	7	38685346	38687037	processed_pseudogene	8,60	0,2161218
ENSG00000250343	ENSG00000250343	5	147180204	147234859	antisense	8,60	0
ENSG00000280029	ENSG00000280029	5	141136683	141245380	antisense	8,60	0
ENSG00000242337	TFP1	3	133661998	133754576	transcribed_unprocessed_pseudogene	8,60	0
ENSG00000240244	GAPDHP33	1	119596167	119597179	processed_pseudogene	8,60	1,40E-02
ENSG00000272668	ENSG00000272668	1	159866954	159867685	antisense	8,60	7,06E-02
ENSG00000214654	ENSG00000214654	9	120793551	120799570	transcribed_processed_pseudogene	8,59	0
ENSG00000273523	ENSG00000273523	13	52128891	52132723	antisense	8,58	6,27E-03
ENSG00000231177	LINC00852	3	10284419	10285746	antisense	8,56	0
ENSG00000260389	WBP11P1	18	32511663	32514634	transcribed_processed_pseudogene	8,56	0
ENSG00000248015	ENSG00000248015	19	1392170	1396467	antisense	8,56	0
ENSG00000225578	NCBP2-AS1	3	196939877	196942534	antisense	8,54	0
ENSG00000225484	NUTM2B-AS1	10	79663088	79826594	antisense	8,53	0
ENSG00000267096	ENSG00000267096	19	55670632	55672069	sense_intronic	8,50	0
ENSG00000277744	ENSG00000277744	19	41373971	41374419	sense_intronic	8,50	0
ENSG00000259635	ENSG00000259635	15	64701248	64719602	antisense	8,50	0
ENSG00000274270	ENSG00000274270	13	50125816	50128463	sense_intronic	8,50	7,06E-02
ENSG00000254829	ENSG00000254829	11	78015715	78016495	antisense	8,50	0
ENSG00000227603	ENSG00000227603	9	93435332	93437121	antisense	8,50	0
ENSG00000233231	HNRNPA1P49	6	166334568	166335471	processed_pseudogene	8,50	0
ENSG00000232717	TRIM51JP	2	95574899	95580764	unprocessed_pseudogene	8,50	1,40E-02
ENSG00000225671	FCF1P6	1	50405430	50406024	processed_pseudogene	8,50	0,2161218
ENSG00000240219	ENSG00000240219	1	204626775	204629712	lincRNA	8,50	1,40E-02
ENSG00000225008	ENSG00000225008	X	155290360	155291353	processed_pseudogene	8,50	0
ENSG00000269139	ENSG00000269139	19	7926001	7926810	processed_transcript	8,49	9,18E-02
ENSG00000273344	PAXIP1-AS1	7	155003448	155005703	lincRNA	8,48	0
ENSG00000275638	ENSG00000275638	15	65655620	65656085	antisense	8,47	0
ENSG00000224152	ENSG00000224152	2	159615296	159617082	antisense	8,45	0
ENSG00000225770	ENSG00000225770	3	196536532	196536702	processed_pseudogene	8,44	0
ENSG00000273015	ENSG00000273015	12	45718046	45727775	lincRNA	8,42	0
ENSG00000213856	VDAC1P2	X	49632500	49633349	processed_pseudogene	8,41	0
ENSG00000280383	ENSG00000280383	22	45657019	45680130	sense_overlapping	8,40	0
ENSG00000203616	RHOT1P2	21	13936993	13937325	processed_pseudogene	8,40	0
ENSG00000234883	MIR155HG	21	25561909	25575168	lincRNA	8,40	0
ENSG00000261123	ENSG00000261123	16	2094830	2097026	sense_intronic	8,40	1,40E-02
ENSG00000261744	ENSG00000261744	16	88512960	88531053	antisense	8,40	0
ENSG00000270184	ENSG00000270184	16	85784382	85787617	antisense	8,40	0
ENSG00000259721	ENSG00000259721	15	32717270	32719007	lincRNA	8,40	7,06E-02
ENSG00000255581	ENSG00000255581	12	8017201	8017860	processed_pseudogene	8,40	0
ENSG00000254910	ENSG00000254910	11	310139	311141	antisense	8,40	0
ENSG00000176236	C10orf111	10	15095385	15097319	antisense	8,40	4,86E-02
ENSG00000261068	ENSG00000261068	6	42092233	42094259	lincRNA	8,40	1,40E-02
ENSG00000275339	ENSG00000275339	6	122454358	122454612	processed_pseudogene	8,40	1,40E-02
ENSG00000227403	ENSG00000227403	2	161244739	161249050	lincRNA	8,40	1,40E-02
ENSG00000230385	ENSG00000230385	2	230908852	230910102	antisense	8,40	0
ENSG00000228086	ENSG00000228086	1	100462399	100485997	antisense	8,40	0
ENSG00000249741	ENSG00000249741	4	143911514	143912053	processed_pseudogene	8,38	0
ENSG00000231579	RPL7P21	5	143332605	143333239	processed_pseudogene	8,37	0
ENSG00000278177	ENSG00000278177	13	93226612	93227317	antisense	8,37	0
ENSG00000237161	ENSG00000237161	15	21293653	21295201	processed_pseudogene	8,36	0
ENSG00000189136	UBE2Q2P1	15	84526781	84571216	transcribed_unprocessed_pseudogene	8,34	0
ENSG00000234176	HSPA8P1	X	121203182	121205014	processed_pseudogene	8,34	0
ENSG00000229591	ENSG00000229591	7	152120001	152121717	antisense	8,33	0

ENSG00000222019	URAHP	16	90039761	90047773	transcribed_unprocessed_pseudogene	8,33	0
ENSG00000251224	CNOT10-AS1	3	32730635	32737454	antisense	8,32	0
ENSG00000228158	TLE1P1	X	65408084	65408672	processed_pseudogene	8,30	0
ENSG00000240522	RPL7AP10	19	21149648	21150438	processed_pseudogene	8,30	0
ENSG00000267640	ENSG00000267640	19	37817359	37826638	lincRNA	8,30	0
ENSG00000260347	MOCS1P1	16	48496990	48498601	processed_pseudogene	8,30	0
ENSG00000260442	ATP2A1-AS1	16	28878957	28879920	antisense	8,30	1,40E-02
ENSG00000259495	ENSG00000259495	15	80344853	80403575	lincRNA	8,30	0
ENSG00000274376	ADAMTS7P1	15	82298553	82334609	transcribed_unprocessed_pseudogene	8,30	0
ENSG00000176654	NANOGP1	12	7890801	7900140	transcribed_unprocessed_pseudogene	8,30	0
ENSG00000226416	MRPL23-AS1	11	1983237	1989920	antisense	8,30	7,06E-02
ENSG00000231864	ENSG00000231864	9	137063535	137064581	antisense	8,30	0
ENSG00000180712	ENSG00000180712	4	184340756	184353977	lincRNA	8,30	0
ENSG00000248375	ENSG00000248375	4	52720081	52720831	processed_pseudogene	8,30	0
ENSG00000242052	ENSG00000242052	3	119635526	119636150	processed_pseudogene	8,30	0
ENSG00000260633	ENSG00000260633	3	134347288	134349233	lincRNA	8,30	0
ENSG00000177133	LINC00982	1	3059615	3068437	antisense	8,30	0,2161218
ENSG00000220685	ENSG00000220685	6	5065795	5066982	processed_pseudogene	8,29	0
ENSG00000231305	ENSG00000231305	3	128861313	128871540	antisense	8,29	0
ENSG00000272645	GTF2IP20	1	223951394	223992594	transcribed_unprocessed_pseudogene	8,28	0
ENSG00000261824	LINC00662	19	27684580	27793940	lincRNA	8,28	0
ENSG00000265752	ENSG00000265752	18	23957754	23982556	lincRNA	8,27	0
ENSG00000241081	ENSG00000241081	14	76886377	76886750	processed_pseudogene	8,27	0
ENSG00000269145	ENSG00000269145	19	18144522	18151691	antisense	8,26	0
ENSG00000277879	ENSG00000277879	10	117267116	117268668	antisense	8,26	0
ENSG00000262477	ENSG00000262477	18	32412182	32413236	lincRNA	8,25	2,21E-02
ENSG00000214353	VAC14-AS1	16	70755098	70773251	antisense	8,24	0
ENSG00000248641	HMGA1P2	4	73098822	73099145	processed_pseudogene	8,23	1,40E-02
ENSG00000124399	ENSG00000124399	4	43898962	43899351	processed_pseudogene	8,22	0
ENSG00000254236	ENSG00000254236	8	103020187	103021428	antisense	8,21	0
ENSG00000276488	ENSG00000276488	19	55612490	55613097	antisense	8,20	0
ENSG00000262372	ENSG00000262372	17	46035313	46035770	antisense	8,20	0
ENSG00000264070	DND1P1	17	45585871	45586929	processed_pseudogene	8,20	7,06E-02
ENSG00000260953	ENSG00000260953	16	29262273	29264479	lincRNA	8,20	0
ENSG00000262721	ENSG00000262721	16	30875766	30895216	sense_overlapping	8,20	1,40E-02
ENSG00000180221	TPT1P10	10	58212541	58213036	processed_pseudogene	8,20	0,2161218
ENSG00000251396	LINC01301	8	60384588	60516795	lincRNA	8,20	0
ENSG00000251023	ENSG00000251023	5	93860669	93863825	sense_intronic	8,20	1,40E-02
ENSG00000228857	ENSG00000228857	2	113831049	113843356	lincRNA	8,20	7,06E-02
ENSG00000248821	ENSG00000248821	2	95413456	95414296	unprocessed_pseudogene	8,20	0
ENSG00000269981	ENSG00000269981	1	137682	137965	processed_pseudogene	8,20	0
ENSG00000225400	RAB28P5	X	136847384	136848034	processed_pseudogene	8,20	1,40E-02
ENSG00000213514	ENSG00000213514	10	77730766	77734769	processed_pseudogene	8,20	0
ENSG00000258357	ENSG00000258357	12	94521090	94521869	processed_pseudogene	8,19	0
ENSG00000230445	LRRC37A6P	10	27246350	27259455	transcribed_processed_pseudogene	8,19	0
ENSG00000253557	ENSG00000253557	8	19084992	19259469	lincRNA	8,18	0,1392851
ENSG00000238045	ENSG00000238045	16	29808636	29821252	antisense	8,17	0
ENSG00000271797	ENSG00000271797	5	115262505	115263448	antisense	8,17	0
ENSG00000255750	ENSG00000255750	12	26230819	26319720	antisense	8,15	0
ENSG00000271743	ENSG00000271743	8	6615604	6617198	lincRNA	8,14	6,27E-03
ENSG00000272622	ENSG00000272622	2	226800146	226811029	lincRNA	8,13	0
ENSG00000235454	HAUS6P3	7	53862233	53863339	processed_pseudogene	8,12	0
ENSG00000253833	ENSG00000253833	8	73984493	73984883	processed_pseudogene	8,11	0
ENSG00000215458	AATBC	21	43805758	43812567	antisense	8,10	0
ENSG00000270164	LINC01480	19	41535183	41536904	lincRNA	8,10	1,40E-02

ENSG00000260293	ENSG00000260293	16	2476558	2482173	sense_intronic	8,10	1,40E-02
ENSG00000261346	ENSG00000261346	16	30477180	30489353	antisense	8,10	0
ENSG00000260103	ENSG00000260103	15	74478070	74490286	transcribed_processed_pseudogene	8,10	0
ENSG00000265967	ENSG00000265967	15	64695041	64695594	antisense	8,10	0
ENSG00000255038	ENSG00000255038	11	66067277	66069619	antisense	8,10	0
ENSG00000242338	BMS1P4	10	73715843	73730469	transcribed_unprocessed_pseudogene	8,10	0
ENSG00000253636	ENSG00000253636	8	73052178	73063061	antisense	8,10	0
ENSG00000263683	ENSG00000263683	7	30550217	30551569	lincRNA	8,10	0
ENSG00000241155	ARHGAP31-AS1	3	119314293	119322760	antisense	8,10	0
ENSG00000249602	ENSG00000249602	1	151763384	151769501	antisense	8,10	0
ENSG00000226679	ENSG00000226679	X	38221391	38223667	antisense	8,10	0
ENSG00000264486	ENSG00000264486	17	28256438	28265551	lincRNA	8,09	0
ENSG00000254821	ENSG00000254821	6	4135423	4146053	antisense	8,09	0
ENSG00000257839	ENSG00000257839	12	76032658	76033897	antisense	8,08	0
ENSG00000179967	PPP1R14BP3	4	139114930	139115374	processed_pseudogene	8,04	0
ENSG00000266876	ENSG00000266876	17	30068632	30069631	transcribed_processed_pseudogene	8,03	0
ENSG00000233328	PFN1P1	1	171670517	171670939	processed_pseudogene	8,00	3,30E-03
ENSG00000246898	LINC00920	16	66408524	66412135	lincRNA	8,00	0
ENSG00000261592	ENSG00000261592	16	87396704	87400295	antisense	8,00	0
ENSG00000244050	ENSG00000244050	12	8199599	8218911	transcribed_unprocessed_pseudogene	8,00	0
ENSG00000280828	ENSG00000280828	7	128533652	128615970	unprocessed_pseudogene	8,00	0
ENSG00000235663	SAPCD1-AS1	6	31764310	31765588	antisense	8,00	0
ENSG00000229122	AGBL5-IT1	2	27061038	27061815	sense_intronic	8,00	1,40E-02
ENSG00000272702	ENSG00000272702	2	73113018	73115907	processed_transcript	8,00	7,06E-02
ENSG00000225206	MIR137HG	1	97986740	98049863	lincRNA	8,00	0
ENSG00000225620	ENSG00000225620	1	202632428	202632911	lincRNA	8,00	7,06E-02
ENSG00000260698	ENSG00000260698	1	246682108	246685075	lincRNA	8,00	0
ENSG00000219699	ENSG00000219699	6	127659690	127660579	unprocessed_pseudogene	7,97	0
ENSG00000278576	ENSG00000278576	14	88589231	88592408	sense_intronic	7,94	0
ENSG00000213205	STRADBP1	7	150512559	150513839	processed_pseudogene	7,93	0
ENSG00000227232	WASH7P	1	14404	29570	unprocessed_pseudogene	7,93	0
ENSG00000236530	ENSG00000236530	4	80079532	80081122	processed_pseudogene	7,92	9,18E-02
ENSG00000235349	ENSG00000235349	7	64566814	64567443	processed_pseudogene	7,92	2,95E-02
ENSG00000244041	LINC01011	6	2987967	2991173	lincRNA	7,91	0
ENSG00000236360	ENSG00000236360	1	52993201	52993702	processed_pseudogene	7,91	0
ENSG00000265666	RARA-AS1	17	40340867	40343136	antisense	7,91	0
ENSG00000272787	ENSG00000272787	22	23969211	23969873	lincRNA	7,90	0
ENSG00000268673	ENSG00000268673	19	15760375	15770828	lincRNA	7,90	0,2161218
ENSG00000278107	ENSG00000278107	18	76805151	76805736	lincRNA	7,90	0
ENSG00000269947	ENSG00000269947	17	8277763	8278436	lincRNA	7,90	0
ENSG00000258708	SLC25A21-AS1	14	37171888	37173811	antisense	7,90	0
ENSG00000259018	ENSG00000259018	14	23356406	23357003	antisense	7,90	0
ENSG00000239617	ENSG00000239617	12	48903418	48903813	processed_pseudogene	7,90	1,40E-02
ENSG00000270640	ENSG00000270640	2	28396815	28397110	sense_intronic	7,90	0
ENSG00000205105	COX17P1	13	46490930	46491121	processed_pseudogene	7,89	0
ENSG00000265496	ENSG00000265496	18	49487339	49491878	antisense	7,88	0
ENSG00000233330	ENSG00000233330	6	149591755	149592663	antisense	7,88	0
ENSG00000243759	ST13P15	3	143517123	143518229	processed_pseudogene	7,88	0
ENSG00000259585	RBM17P4	15	35298249	35299440	processed_pseudogene	7,88	7,06E-02
ENSG00000261315	LARP4P	15	36617089	36619265	transcribed_processed_pseudogene	7,88	0
ENSG00000073905	VDAC1P1	X	80929500	80930347	processed_pseudogene	7,86	0
ENSG00000231890	DARS-AS1	2	135985176	136022593	antisense	7,85	0
ENSG00000272182	ENSG00000272182	3	58428255	58428815	antisense	7,85	0

ENSG00000261840	ENSG00000261840	16	30697707	30699058	antisense	7,83	0
ENSG00000260589	STAM-AS1	10	17641284	17643878	antisense	7,83	0,1392851
ENSG00000260082	ENSG00000260082	16	30821068	30822110	processed_pseudogene	7,82	0,1392851
ENSG00000277151	ENSG00000277151	13	32782874	32788178	lincRNA	7,82	0
ENSG00000243349	ENSG00000243349	10	45147394	45171389	processed_transcript	7,82	0
ENSG00000237705	ENSG00000237705	5	56842016	56862164	antisense	7,81	0
ENSG00000261737	ENSG00000261737	1	86703502	86704462	antisense	7,81	0
ENSG00000258016	HIGD1AP1	12	53142053	53142327	processed_pseudogene	7,81	0
ENSG00000272799	ENSG00000272799	18	9912316	9912849	lincRNA	7,80	0
ENSG00000223878	ENSG00000223878	17	15506866	15507354	processed_pseudogene	7,80	0
ENSG00000262251	ENSG00000262251	17	7685260	7686371	sense_intronic	7,80	0
ENSG00000259116	ENSG00000259116	14	64514154	64540368	antisense	7,80	0
ENSG00000255445	ENSG00000255445	11	93152075	93152750	processed_pseudogene	7,80	0
ENSG00000272589	ZSWIM8-AS1	10	73796514	73801399	antisense	7,80	0
ENSG00000234779	ENSG00000234779	9	16726814	16727524	antisense	7,80	7,06E-02
ENSG00000176933	TOB2P1	6	28217643	28218634	processed_pseudogene	7,80	0
ENSG00000244675	ENSG00000244675	3	194496317	194501503	sense_overlapping	7,80	0
ENSG00000270178	ENSG00000270178	3	179804063	179804366	lincRNA	7,80	7,06E-02
ENSG00000231333	RPL34P6	1	200863808	200864152	processed_pseudogene	7,80	7,06E-02
ENSG00000103472	RRN3P2	16	29074976	29116718	transcribed_unprocessed_pseudogene	7,79	0
ENSG00000226823	ENSG00000226823	9	33503655	33510711	unprocessed_pseudogene	7,78	0
ENSG00000236753	MKLN1-AS	7	131309744	131328222	processed_transcript	7,77	0
ENSG00000188451	SRP72P2	6	158237336	158242797	processed_pseudogene	7,77	1,40E-02
ENSG00000234106	ENSG00000234106	11	93535468	93535816	processed_pseudogene	7,76	7,06E-02
ENSG00000277763	ENSG00000277763	14	56117316	56117990	lincRNA	7,75	0
ENSG00000224546	EIF4BP3	9	96146007	96147856	processed_pseudogene	7,75	0
ENSG00000257715	ENSG00000257715	12	96025323	96027971	antisense	7,73	0
ENSG00000105988	NHP2P1	10	92216332	92216793	processed_pseudogene	7,73	0
ENSG00000164669	INTS4P1	7	65141225	65234216	transcribed_unprocessed_pseudogene	7,73	0
ENSG00000236582	PRPF38AP2	10	17577362	17578212	processed_pseudogene	7,72	0
ENSG00000226029	ENSG00000226029	1	16460948	16468481	lincRNA	7,72	3,30E-03
ENSG00000275582	ENSG00000275582	20	3921279	3923400	antisense	7,70	0
ENSG00000232085	ENSG00000232085	1	243164638	243169445	antisense	7,70	0
ENSG00000264449	ENSG00000264449	18	5887487	5909122	antisense	7,70	0
ENSG00000263412	ENSG00000263412	17	48045141	48048073	processed_transcript	7,70	1,40E-02
ENSG00000259652	ENSG00000259652	15	77043680	77045160	lincRNA	7,70	0
ENSG00000250748	ENSG00000250748	12	65466820	65642372	lincRNA	7,70	0
ENSG00000235286	ENSG00000235286	11	111670956	111672404	processed_pseudogene	7,70	0
ENSG00000233334	FAM53B-AS1	10	124703625	124714217	antisense	7,70	0
ENSG00000235677	NPM1P26	10	96560185	96560922	processed_pseudogene	7,70	0
ENSG00000238228	OR7E7P	7	97946987	97947998	unprocessed_pseudogene	7,70	0
ENSG00000273183	ENSG00000273183	7	154956429	154957107	antisense	7,70	1,40E-02
ENSG00000230082	PRRT3-AS1	3	9947404	9954787	antisense	7,70	0
ENSG00000275956	ENSG00000275956	3	52732547	52733867	processed_pseudogene	7,70	0,2161218
ENSG00000272175	ENSG00000272175	1	51801028	51801307	antisense	7,70	7,06E-02
ENSG00000267484	ENSG00000267484	19	4838332	4838907	antisense	7,69	6,27E-03
ENSG00000236986	ENSG00000236986	9	131132852	131167505	antisense	7,69	0
ENSG00000230074	ENSG00000230074	9	34665665	34681298	antisense	7,68	0
ENSG00000270607	ENSG00000270607	11	19710934	19712619	lincRNA	7,67	0,0358681
ENSG00000254551	ENSG00000254551	11	83209431	83213379	antisense	7,66	0
ENSG00000261819	ENSG00000261819	16	14988259	14990160	processed_pseudogene	7,65	0
ENSG00000272990	ENSG00000272990	3	156523740	156524247	antisense	7,65	6,27E-03
ENSG00000251136	ENSG00000251136	8	89609409	89757727	lincRNA	7,62	0
ENSG00000270953	ENSG00000270953	7	130507660	130508282	antisense	7,60	0
ENSG00000236850	BMS1P20	22	22303224	22322847	processed_transcript	7,60	0
ENSG00000264570	SNX19P3	18	14629832	14632806	processed_pseudogene	7,60	0
ENSG00000265778	ENSG00000265778	18	76491652	76493918	antisense	7,60	0

ENSG00000231258	ZSWIM5P2	17	20583758	20591180	unprocessed_pseudogene	7,60	0
ENSG00000265702	ENSG00000265702	17	62699244	62737922	antisense	7,60	1,40E-02
ENSG00000259677	ENSG00000259677	15	89876540	89877285	processed_pseudogene	7,60	7,06E-02
ENSG00000259715	ENSG00000259715	15	50359450	50360194	lincRNA	7,60	0
ENSG00000235550	ANKRD26P2	13	38922727	38936199	unprocessed_pseudogene	7,60	0
ENSG00000276718	ENSG00000276718	12	6466537	6467135	antisense	7,60	0
ENSG00000254783	ENSG00000254783	11	86283927	86284668	processed_pseudogene	7,60	0
ENSG00000244733	ENSG00000244733	10	79660891	79677996	lincRNA	7,60	0
ENSG00000261353	ENSG00000261353	6	26527063	26527404	lincRNA	7,60	0
ENSG00000251066	ENSG00000251066	5	85549409	85549952	processed_pseudogene	7,60	2,21E-02
ENSG00000249212	ATP1B1P1	4	42029209	42030123	unprocessed_pseudogene	7,60	7,06E-02
ENSG00000236137	ENSG00000236137	1	117025482	117059490	antisense	7,60	0
ENSG00000279529	ENSG00000279529	19	16572624	16575340	antisense	7,60	7,06E-02
ENSG00000229619	MBNL1-AS1	3	152262616	152269555	antisense	7,60	0
ENSG00000235530	ENSG00000235530	17	21214331	21230035	antisense	7,58	6,27E-03
ENSG00000251188	ENSG00000251188	4	268982	269531	processed_pseudogene	7,57	1,40E-02
ENSG00000241163	LINC00877	3	72035300	72279503	lincRNA	7,57	0
ENSG00000213189	BTF3L4P2	2	159003975	159004320	processed_pseudogene	7,56	0
ENSG00000255367	ENSG00000255367	11	3481520	3581211	lincRNA	7,56	0
ENSG00000178803	ADORA2A-AS1	22	24429206	24495074	antisense	7,55	6,27E-03
ENSG00000257261	ENSG00000257261	12	46383679	46876159	lincRNA	7,55	0
ENSG00000275106	ENSG00000275106	7	128952527	128953316	lincRNA	7,55	0,1836819
ENSG00000179978	ENSG00000179978	5	70094659	70128434	unprocessed_pseudogene	7,55	0
ENSG00000243176	ENSG00000243176	3	157175223	157381265	processed_transcript	7,54	0
ENSG00000269349	ENSG00000269349	19	52511282	52512342	antisense	7,53	0
ENSG00000100181	TPTEP1	22	16601887	16698742	lincRNA	7,51	0
ENSG00000229917	RPL7P46	16	11027024	11027763	processed_pseudogene	7,51	0
ENSG00000215481	BCRP3	22	24632915	24653356	transcribed_unprocessed_pseudogene	7,50	1,40E-02
ENSG00000267938	EIF1P6	19	4347780	4349061	unprocessed_pseudogene	7,50	1,40E-02
ENSG00000266129	SRP68P1	17	16787235	16788141	processed_pseudogene	7,50	1,40E-02
ENSG00000259381	ENSG00000259381	15	100645434	100646655	processed_pseudogene	7,50	1,40E-02
ENSG00000223685	LINC00571	13	38050817	38143232	lincRNA	7,50	0
ENSG00000271579	ENSG00000271579	12	115299588	115300708	lincRNA	7,50	0,1836819
ENSG00000278112	ENSG00000278112	12	123519390	123519856	sense_intronic	7,50	7,06E-02
ENSG00000226645	ENSG00000226645	11	116773389	116774205	antisense	7,50	3,30E-03
ENSG00000253483	ENSG00000253483	8	23483016	23483534	processed_pseudogene	7,50	0,2161218
ENSG00000281706	LINC01012	6	27694035	27710222	lincRNA	7,50	0
ENSG00000260641	ENSG00000260641	4	98658904	98664550	antisense	7,50	6,27E-03
ENSG00000273302	ENSG00000273302	2	61199979	61200769	sense_intronic	7,50	0
ENSG00000203706	SERTAD4-AS1	1	210231456	210234047	antisense	7,50	1,40E-02
ENSG00000226443	GAPDHP32	1	119533749	119534746	processed_pseudogene	7,50	1,40E-02
ENSG00000235595	GAPDHP23	1	119462029	119463021	processed_pseudogene	7,50	1,40E-02
ENSG00000236751	LINC01186	X	46325924	46327645	lincRNA	7,50	0
ENSG00000273253	ENSG00000273253	22	50199090	50200837	antisense	7,50	6,27E-03
ENSG00000213117	ENSG00000213117	6	134897874	134898759	processed_pseudogene	7,49	0
ENSG00000251548	ENSG00000251548	5	10664495	10665113	processed_pseudogene	7,48	0
ENSG00000267113	ENSG00000267113	19	35957121	35957382	processed_pseudogene	7,47	0
ENSG00000213018	ENSG00000213018	X	71420083	71420514	processed_pseudogene	7,47	0
ENSG00000255201	ENSG00000255201	8	38421889	38426096	antisense	7,46	0
ENSG00000255248	ENSG00000255248	11	122028355	122116323	sense_overlapping	7,46	0
ENSG00000265790	RNASEH1P1	17	20901939	20902680	processed_pseudogene	7,45	0
ENSG00000236204	LINC01376	2	18986451	19346748	lincRNA	7,45	0
ENSG00000250471	GMPSP1	4	8174421	8174999	processed_pseudogene	7,45	0
ENSG00000248966	BCLAF1P1	5	110948029	110950780	processed_pseudogene	7,42	6,27E-03
ENSG00000214719	ENSG00000214719	17	30576464	30672789	processed_transcript	7,42	0

ENSG00000224452	RSL24D1P6	20	32170390	32170790	processed_pseudogene	7,40	1,40E-02
ENSG00000231703	ENSG00000231703	20	53397661	53412910	antisense	7,40	1,40E-02
ENSG00000268747	ENSG00000268747	19	21276023	21276332	processed_pseudogene	7,40	7,06E-02
ENSG00000274918	ENSG00000274918	18	35317396	35317848	lincRNA	7,40	1,40E-02
ENSG00000120664	SPG20-AS1	13	36346431	36369601	antisense	7,40	0
ENSG00000271155	ENSG00000271155	9	95506235	95507636	antisense	7,40	0
ENSG00000227855	DPY19L2P3	7	29650227	29742594	transcribed_unprocessed_pseudogene	7,40	0
ENSG00000234338	ENSG00000234338	7	64835280	64836882	processed_pseudogene	7,40	1,40E-02
ENSG00000234263	ENSG00000234263	6	136629172	136647999	antisense	7,40	0
ENSG00000248503	ENSG00000248503	X	77910741	77964890	transcribed_processed_pseudogene	7,40	0
ENSG00000273599	ENSG00000273599	10	124996064	125001491	antisense	7,40	0
ENSG00000235092	ID2-AS1	2	8666636	8681863	antisense	7,39	0
ENSG00000242193	ENSG00000242193	1	178006136	178037950	unitary_pseudogene	7,38	0
ENSG00000257913	ENSG00000257913	12	48998367	49019235	antisense	7,38	0
ENSG00000266282	UBL5P2	17	32227890	32228108	processed_pseudogene	7,38	2,21E-02
ENSG00000227782	ENSG00000227782	17	16040472	16041273	antisense	7,34	0
ENSG00000229873	OGFR-AS1	20	62800627	62805587	antisense	7,33	2,95E-02
ENSG00000224785	ENSG00000224785	7	23562459	23564261	processed_pseudogene	7,33	0
ENSG00000232531	ENSG00000232531	2	91747940	91748986	processed_pseudogene	7,32	0
ENSG00000203993	ARRDC1-AS1	9	137615332	137618906	antisense	7,32	0
ENSG00000230461	PROX1-AS1	1	213817751	213988508	processed_transcript	7,32	3,30E-03
ENSG00000240898	ENSG00000240898	17	3665220	3665702	processed_pseudogene	7,32	6,27E-03
ENSG00000189089	RIMKLBP1	21	36050214	36051377	processed_pseudogene	7,31	0
ENSG00000259657	PIGHP1	15	59676757	59677321	processed_pseudogene	7,30	0
ENSG00000267882	ENSG00000267882	20	47318502	47320754	antisense	7,30	0
ENSG00000277383	ENSG00000277383	19	47607873	47608454	antisense	7,30	7,06E-02
ENSG00000260740	ENSG00000260740	16	31487370	31488492	antisense	7,30	1,40E-02
ENSG00000277767	ENSG00000277767	13	110916004	110917827	lincRNA	7,30	1,40E-02
ENSG00000254602	ENSG00000254602	11	57638024	57652790	sense_overlapping	7,30	0
ENSG00000255404	ENSG00000255404	11	65795946	65797219	antisense	7,30	0,2161218
ENSG00000214188	ST7-OT4	7	116953899	117098806	sense_intronic	7,30	0
ENSG00000248971	KRT8P46	4	102728746	102730171	processed_pseudogene	7,30	0
ENSG00000251442	LINC01094	4	78645903	78682699	lincRNA	7,30	0
ENSG00000271324	ENSG00000271324	3	33793644	33794145	lincRNA	7,30	1,40E-02
ENSG00000227740	ENSG00000227740	1	175904762	175920513	lincRNA	7,30	7,06E-02
ENSG00000196205	EEF1A1P5	9	133019486	133020874	processed_pseudogene	7,29	7,06E-02
ENSG00000213264	NIP7P2	3	132401253	132401796	processed_pseudogene	7,29	0
ENSG00000244306	LINC01296	14	19284653	19337730	transcribed_processed_pseudogene	7,27	0
ENSG00000275400	ENSG00000275400	7	66553805	66554199	processed_pseudogene	7,25	0
ENSG00000219642	BMPR1APS1	6	129157523	129159120	processed_pseudogene	7,24	0
ENSG00000101898	MCTS2P	20	31547504	31548049	processed_pseudogene	7,24	0
ENSG00000229018	PMS2P7	7	73005541	73021103	unprocessed_pseudogene	7,23	0
ENSG00000177993	ZNRF3-AS1	22	29024999	29031476	antisense	7,20	0
ENSG00000270393	ENSG00000270393	22	19018043	19018916	processed_pseudogene	7,20	1,40E-02
ENSG00000279345	ENSG00000279345	22	49805452	49807208	antisense	7,20	1,40E-02
ENSG00000266932	ENSG00000266932	19	56342432	56343419	transcribed_processed_pseudogene	7,20	0,2161218
ENSG00000262692	ENSG00000262692	17	3721628	3722488	antisense	7,20	0
ENSG00000265982	ENSG00000265982	17	64778842	64778937	unprocessed_pseudogene	7,20	0
ENSG00000237425	RPSAP2	6	28732017	28732904	processed_pseudogene	7,20	0
ENSG00000248587	GDNF-AS1	5	37840438	37875799	lincRNA	7,20	1,40E-02
ENSG00000246090	ENSG00000246090	4	99088857	99301356	antisense	7,20	0
ENSG00000240541	TM4SF1-AS1	3	149377778	149386583	antisense	7,20	0
ENSG00000241544	ENSG00000241544	3	157081841	157088547	lincRNA	7,20	0,2161218
ENSG00000272181	ENSG00000272181	3	64019508	64019925	antisense	7,20	7,06E-02
ENSG00000272597	ENSG00000272597	3	107329430	107329962	lincRNA	7,20	1,40E-02

ENSG00000229447	ENSG00000229447	1	31263245	31263681	processed_pseudogene	7,20	0
ENSG00000237491	ENSG00000237491	1	778770	810060	lincRNA	7,20	0
ENSG00000269227	ENSG00000269227	1	1673275	1674397	transcribed_processed_pseudogene	7,20	0
ENSG00000250656	ST3GAL1P1	4	67716375	67717388	processed_pseudogene	7,18	0,4332376
ENSG00000262662	ENSG00000262662	17	80960766	80961713	antisense	7,17	0
ENSG00000231858	ENSG00000231858	2	191021526	191032314	processed_transcript	7,17	0
ENSG00000224155	ENSG00000224155	7	56304678	56305526	processed_pseudogene	7,16	0
ENSG00000228834	ENSG00000228834	6	108907615	108907873	processed_pseudogene	7,16	6,27E-03
ENSG00000229780	UBE2Q1-AS1	1	154553609	154555017	antisense	7,16	0
ENSG00000237846	ENSG00000237846	9	40105822	40106527	processed_pseudogene	7,15	9,18E-02
ENSG00000248795	ENSG00000248795	4	138560704	138561941	processed_pseudogene	7,15	0
ENSG00000239569	KMT2E-AS1	7	105013425	105014321	antisense	7,15	0
ENSG00000257045	ENSG00000257045	2	199325310	199329362	antisense	7,13	0
ENSG00000240048	DDX50P2	3	154352136	154354799	processed_pseudogene	7,11	0
ENSG00000235105	ENSG00000235105	1	48435967	48437223	processed_pseudogene	7,10	0
ENSG00000275540	ENSG00000275540	19	19740884	19750127	unprocessed_pseudogene	7,10	7,06E-02
ENSG00000266954	ENSG00000266954	18	13644815	13645685	antisense	7,10	0
ENSG00000264456	ENSG00000264456	17	30971652	30973312	sense_intronic	7,10	0
ENSG00000265542	ENSG00000265542	17	57771946	57834749	lincRNA	7,10	0
ENSG00000258951	KRT18P7	14	70594552	70595770	processed_pseudogene	7,10	0
ENSG00000255627	ENSG00000255627	12	22008348	22008880	processed_pseudogene	7,10	0
ENSG00000257718	ENSG00000257718	12	38906451	38909592	antisense	7,10	7,06E-02
ENSG00000205584	ENSG00000205584	7	72969814	72971727	unprocessed_pseudogene	7,10	0
ENSG00000228204	ENSG00000228204	7	50866747	51022990	antisense	7,10	7,06E-02
ENSG00000273319	ENSG00000273319	7	130936464	130939661	lincRNA	7,10	0
ENSG00000242198	ENSG00000242198	5	75374463	75374809	processed_pseudogene	7,10	0
ENSG00000235151	ENSG00000235151	2	241844380	241845036	lincRNA	7,10	1,40E-02
ENSG00000267090	ENSG00000267090	19	38385522	38386759	antisense	7,09	0,0358681
ENSG00000232545	ENSG00000232545	22	24101689	24103354	sense_intronic	7,08	0
ENSG00000276223	ENSG00000276223	20	44746642	44747201	sense_intronic	7,08	0,4332376
ENSG00000231184	FAM58DP	13	19382454	19383072	processed_pseudogene	7,08	0
ENSG00000205913	SRRM2-AS1	16	2737076	2752600	antisense	7,07	0
ENSG00000237737	DCTN1-AS1	2	74385486	74393882	antisense	7,07	0
ENSG00000249592	ENSG00000249592	4	762387	781849	antisense	7,06	0
ENSG00000215210	RBMXP2	9	30689105	30690272	processed_pseudogene	7,06	0
ENSG00000250251	PKD1P6	16	15125242	15154564	transcribed_unprocessed_pseudogene	7,05	0
ENSG00000273210	ENSG00000273210	21	37365477	37365932	antisense	7,00	1,40E-02
ENSG00000213671	OLA1P2	17	20775642	20776905	processed_pseudogene	7,00	0
ENSG00000262370	ENSG00000262370	16	3076911	3087100	lincRNA	7,00	0
ENSG00000276523	ENSG00000276523	16	75572185	75572685	antisense	7,00	0
ENSG00000259100	ENSG00000259100	14	39385404	39387102	processed_pseudogene	7,00	0
ENSG00000256262	USP30-AS1	12	109052350	109053952	antisense	7,00	0
ENSG00000236710	ENSG00000236710	11	3084393	3085443	processed_pseudogene	7,00	7,06E-02
ENSG00000269915	ENSG00000269915	11	708564	727047	antisense	7,00	1,40E-02
ENSG00000254507	ENSG00000254507	8	12003400	12004082	transcribed_processed_pseudogene	7,00	0
ENSG00000230732	ENSG00000230732	3	196912646	196914579	sense_intronic	7,00	1,40E-02
ENSG00000261159	ENSG00000261159	3	128859716	128860526	lincRNA	7,00	0
ENSG00000232676	ADH5P2	1	79521080	79522203	processed_pseudogene	7,00	0,2161218
ENSG00000226199	ENSG00000226199	X	27832175	27833204	processed_pseudogene	7,00	0
ENSG00000223959	AFG3L1P	16	89972586	90002161	unitary_pseudogene	6,99	0
ENSG00000272379	ENSG00000272379	6	13290018	13290490	lincRNA	6,98	0
ENSG00000230832	ENSG00000230832	1	147082338	147083578	processed_pseudogene	6,98	2,21E-02
ENSG00000259345	ENSG00000259345	15	38865322	39427195	lincRNA	6,96	1,40E-02
ENSG00000251260	WDFY3-AS1	4	84803258	84810391	antisense	6,96	0
ENSG00000234241	ENSG00000234241	20	6213699	6215381	processed_pseudogene	6,96	0
ENSG00000229847	EMX2OS	10	117473215	117545068	antisense	6,94	0

ENSG00000272821	ENSG00000272821	22	50523926	50524780	antisense	6,93	0
ENSG00000224426	SLC31A1P1	3	172603440	172604006	processed_pseudogene	6,93	0
ENSG00000242068	ENSG00000242068	3	180772750	180773914	processed_pseudogene	6,92	0
ENSG00000261572	ENSG00000261572	3	32236688	32238578	sense_intronic	6,92	0
ENSG00000270076	ENSG00000270076	8	11202965	11203671	lincRNA	6,91	0,1392851
ENSG00000270804	ENSG00000270804	19	57867885	57868834	transcribed_processed_pseudogene	6,91	0
ENSG00000229771	ENSG00000229771	20	40696499	40698616	lincRNA	6,90	0,2161218
ENSG00000234187	AIMP1P1	20	14127400	14128336	processed_pseudogene	6,90	0,2161218
ENSG00000228323	ENSG00000228323	19	53854581	53869107	antisense	6,90	0
ENSG00000267765	ENSG00000267765	17	42683187	42699466	antisense	6,90	0
ENSG00000227258	SMIM2-AS1	13	44110451	44240517	antisense	6,90	0
ENSG00000255867	DENND5B-AS1	12	31589923	31615666	antisense	6,90	0
ENSG00000256069	A2MP1	12	9228533	9275817	transcribed_unprocessed_pseudogene	6,90	0
ENSG00000256139	ENSG00000256139	12	109111218	109125594	sense_overlapping	6,90	0
ENSG00000275936	ENSG00000275936	12	120224744	120225421	antisense	6,90	7,06E-02
ENSG00000261671	ENSG00000261671	10	22257786	22258548	lincRNA	6,90	7,06E-02
ENSG00000227582	ADGRF5P1	9	62860873	62864957	transcribed_processed_pseudogene	6,90	7,06E-02
ENSG00000167912	ENSG00000167912	8	59119040	59121346	antisense	6,90	1,40E-02
ENSG00000248309	MEF2C-AS1	5	88883328	89466398	antisense	6,90	0
ENSG00000254138	ENSG00000254138	5	31093977	31267610	antisense	6,90	0
ENSG00000227267	ENSG00000227267	3	136055184	136055764	processed_pseudogene	6,90	0,2161218
ENSG00000222041	LINC00152	2	87455368	87606805	lincRNA	6,90	0
ENSG00000261379	ENSG00000261379	2	226804036	226805061	sense_intronic	6,90	0
ENSG00000180458	ENSG00000180458	19	37545470	37549171	antisense	6,89	0
ENSG00000179743	ENSG00000179743	1	15834474	15848147	antisense	6,88	0
ENSG00000272606	ENSG00000272606	2	55617909	55618373	antisense	6,88	0
ENSG00000228363	ENSG00000228363	2	86562070	86618766	antisense	6,87	0
ENSG00000273619	ENSG00000273619	20	62386303	62386970	antisense	6,86	4,86E-02
ENSG00000258504	ENSG00000258504	14	100291117	100294656	lincRNA	6,85	0
ENSG00000247324	ENSG00000247324	16	71462278	71465941	antisense	6,85	0
ENSG00000182366	FAM87A	8	375931	383174	lincRNA	6,85	0
ENSG00000279720	ENSG00000279720	21	9082601	9083101	processed_pseudogene	6,83	0
ENSG00000263606	ENSG00000263606	18	2945550	2946890	transcribed_processed_pseudogene	6,83	0
ENSG00000224236	MRRFP1	X	123116107	123117781	processed_pseudogene	6,83	3,30E-03
ENSG00000241316	SUCLG2-AS1	3	67654697	67947713	lincRNA	6,81	0
ENSG00000230311	TOMM20P4	X	73223124	73223558	processed_pseudogene	6,81	0
ENSG00000264635	ENSG00000264635	18	738058	739662	sense_intronic	6,80	0
ENSG00000204584	ENSG00000204584	17	49845910	49848837	antisense	6,80	1,40E-02
ENSG00000248121	SMURF2P1	17	30600796	30615980	transcribed_unprocessed_pseudogene	6,80	1,40E-02
ENSG00000265218	ENSG00000265218	17	64892729	64910180	antisense	6,80	0
ENSG00000267361	ENSG00000267361	17	69094289	69095499	processed_pseudogene	6,80	0
ENSG00000261685	ENSG00000261685	16	50645809	50649249	lincRNA	6,80	0
ENSG00000259570	ENSG00000259570	15	84394512	84395514	unprocessed_pseudogene	6,80	0
ENSG00000259687	LINC01220	14	75294404	75296638	lincRNA	6,80	0
ENSG00000270978	ENSG00000270978	5	151848886	151850791	processed_pseudogene	6,80	0,2161218
ENSG00000250326	ENSG00000250326	4	142933195	143184861	antisense	6,80	0
ENSG00000231249	ITPR1-AS1	3	4490891	4493163	antisense	6,80	1,40E-02
ENSG00000229951	ENSG00000229951	2	28384409	28394672	antisense	6,80	0
ENSG00000232702	ENSG00000232702	6	51410081	51410537	processed_pseudogene	6,79	0
ENSG00000267076	CCDC58P3	18	12211378	12211807	processed_pseudogene	6,79	0
ENSG00000269570	ENSG00000269570	11	58611119	58612642	antisense	6,79	0
ENSG00000230189	ENSG00000230189	7	66409143	66490059	transcribed_unprocessed_pseudogene	6,77	0
ENSG00000261332	ENSG00000261332	16	30498766	30499554	antisense	6,77	0,1392851

ENSG00000228782	ENSG00000228782	17	47450568	47492492	transcribed_unprocessed_pseudogene	6,76	0
ENSG00000229043	ENSG00000229043	7	1160374	1165267	antisense	6,76	0
ENSG00000278518	ENSG00000278518	10	133526259	133527513	antisense	6,73	7,06E-02
ENSG00000226471	ENSG00000226471	22	28800683	28848559	antisense	6,73	2,95E-02
ENSG00000278611	ENSG00000278611	19	9538733	9539734	lincRNA	6,73	0,1392851
ENSG00000254208	ENSG00000254208	8	85177522	85178150	antisense	6,73	0
ENSG00000273096	ENSG00000273096	22	38736610	38736792	sense_intronic	6,70	0
ENSG00000227039	ITGB2-AS1	21	44921051	44929678	antisense	6,70	0
ENSG00000215388	ACTG1P3	20	1160584	1161697	processed_pseudogene	6,70	0
ENSG00000268751	SCGB1B2P	19	34576733	34577701	lincRNA	6,70	0,2161218
ENSG00000223544	ENSG00000223544	17	15588852	15589084	unprocessed_pseudogene	6,70	0
ENSG00000277182	ENSG00000277182	17	38749360	38751457	antisense	6,70	7,06E-02
ENSG00000278829	ENSG00000278829	17	42272069	42275571	sense_intronic	6,70	0
ENSG00000260488	ENSG00000260488	16	11976851	11977850	antisense	6,70	0
ENSG00000260727	SLC7A5P1	16	29613104	29613640	unprocessed_pseudogene	6,70	0
ENSG00000259318	ENSG00000259318	14	55394940	55395233	processed_pseudogene	6,70	1,40E-02
ENSG00000277715	ENSG00000277715	12	106250759	106252786	antisense	6,70	1,40E-02
ENSG00000254409	ENSG00000254409	11	43921059	44001157	sense_overlapping	6,70	0
ENSG00000254477	ENSG00000254477	11	59753015	59754975	antisense	6,70	7,06E-02
ENSG00000229672	ENSG00000229672	10	3751067	3763226	antisense	6,70	0
ENSG00000204837	ENSG00000204837	9	39817530	39818923	unprocessed_pseudogene	6,70	0
ENSG00000260190	ENSG00000260190	9	136937169	136937988	sense_overlapping	6,70	0
ENSG00000250490	ENSG00000250490	5	6310441	6339884	lincRNA	6,70	7,06E-02
ENSG00000230606	ENSG00000230606	2	97416165	97433527	lincRNA	6,70	0
ENSG00000228526	MIR34AHG	1	9148011	9196983	lincRNA	6,70	7,06E-02
ENSG00000232912	ENSG00000232912	1	8424645	8434838	antisense	6,70	1,40E-02
ENSG00000223652	ENSG00000223652	5	123087248	123090299	antisense	6,69	3,30E-03
ENSG00000256789	ENSG00000256789	11	63637677	63658962	antisense	6,69	0
ENSG00000236772	ENSG00000236772	20	32449755	32453607	antisense	6,68	0
ENSG00000238133	MLK7-AS1	2	173166446	173282036	antisense	6,68	0
ENSG00000214027	ARPC3P5	6	15934782	15935315	processed_pseudogene	6,67	0
ENSG00000267394	ENSG00000267394	17	44198882	44216565	antisense	6,67	0
ENSG00000277687	ENSG00000277687	10	118692361	118693535	sense_intronic	6,67	0
ENSG00000217416	ISCA1P1	5	62776877	62777263	processed_pseudogene	6,66	0
ENSG00000214035	ENSG00000214035	7	145009961	145010993	processed_pseudogene	6,64	0,0358681
ENSG00000272102	ENSG00000272102	6	105273220	105273760	lincRNA	6,64	0,0358681
ENSG00000229161	TCP1P1	7	42794906	42802494	unprocessed_pseudogene	6,63	0
ENSG00000274756	ENSG00000274756	17	36574462	36575325	processed_pseudogene	6,62	0
ENSG00000231443	ENSG00000231443	3	195937243	195938739	processed_pseudogene	6,62	0
ENSG00000240890	ENSG00000240890	3	132386522	132389074	processed_pseudogene	6,61	0
ENSG00000224789	ENSG00000224789	2	120174885	120216544	antisense	6,61	0
ENSG00000240489	SETP14	3	155987304	155988176	processed_pseudogene	6,61	0
ENSG00000128254	C22orf24	22	31933521	31945518	processed_transcript	6,60	0
ENSG00000232710	ENSG00000232710	22	42136433	42139927	lincRNA	6,60	0
ENSG00000224497	RPL36P4	20	35544430	35544747	processed_pseudogene	6,60	1,40E-02
ENSG00000275894	ENSG00000275894	20	45345115	45345823	sense_intronic	6,60	0
ENSG00000274767	ENSG00000274767	17	36183235	36196471	antisense	6,60	1,40E-02
ENSG00000260259	ENSG00000260259	16	89682620	89686569	lincRNA	6,60	0
ENSG00000259772	ENSG00000259772	15	31221999	31230838	lincRNA	6,60	7,06E-02
ENSG00000229456	RLIMP1	13	40618738	40621348	processed_pseudogene	6,60	7,06E-02
ENSG00000277423	ENSG00000277423	12	120703867	120704282	lincRNA	6,60	0
ENSG00000255515	ENSG00000255515	11	93609760	93610333	processed_pseudogene	6,60	0
ENSG00000227073	SDHDP2	7	135444461	135444925	processed_pseudogene	6,60	0
ENSG00000270638	ENSG00000270638	6	145735570	145737218	lincRNA	6,60	1,40E-02
ENSG00000233006	ENSG00000233006	5	132311285	132369916	processed_transcript	6,60	0
ENSG00000278936	ENSG00000278936	5	141183401	141201396	antisense	6,60	0

ENSG00000215859	ENSG00000215859	1	147993862	148014956	lincRNA	6,60	1,40E-02
ENSG00000269934	ENSG00000269934	1	228274584	228276066	antisense	6,60	0
ENSG00000272853	ENSG00000272853	10	14877688	14878686	lincRNA	6,58	0
ENSG00000236031	ENSG00000236031	1	243545532	243548329	antisense	6,57	0
ENSG00000237758	BANF1P3	2	230725143	230725403	processed_pseudogene	6,56	0
ENSG00000228801	ENSG00000228801	8	51899325	51947173	antisense	6,56	0
ENSG00000278376	ENSG00000278376	11	118791254	118793137	lincRNA	6,56	3,30E-03
ENSG00000280434	ENSG00000280434	22	44139365	44153626	sense_overlapping	6,55	3,30E-03
ENSG00000274220	ENSG00000274220	16	75433836	75436392	lincRNA	6,55	0
ENSG00000248493	ENSG00000248493	5	145997218	145998003	processed_pseudogene	6,55	2,95E-02
ENSG00000267896	ENSG00000267896	19	49859882	49860514	antisense	6,54	2,21E-02
ENSG00000272625	ENSG00000272625	18	2920966	2921685	antisense	6,52	0
ENSG00000217716	RPS10P3	9	88016300	88016793	processed_pseudogene	6,52	0,1392851
ENSG00000220583	RPL35P2	6	34263311	34263673	processed_pseudogene	6,52	0,1836819
ENSG00000269473	ENSG00000269473	19	58440448	58445849	lincRNA	6,50	1,40E-02
ENSG00000259286	ENSG00000259286	15	68833830	68834749	processed_pseudogene	6,50	0,2161218
ENSG00000259705	ENSG00000259705	15	48645951	48652016	lincRNA	6,50	0
ENSG00000278594	ENSG00000278594	14	19131842	19132890	processed_pseudogene	6,50	0
ENSG00000235481	UBE2R2-AS1	9	33785950	33818795	antisense	6,50	0
ENSG00000254165	ENSG00000254165	8	42537529	42538304	antisense	6,50	0
ENSG00000272338	ENSG00000272338	8	33360839	33361415	lincRNA	6,50	1,40E-02
ENSG00000227986	TRIM60P18	7	64355078	64356199	unprocessed_pseudogene	6,50	4,86E-02
ENSG00000273156	ENSG00000273156	4	82344876	82345540	lincRNA	6,50	1,40E-02
ENSG00000225399	ENSG00000225399	3	49260085	49261316	lincRNA	6,50	0
ENSG00000269889	ENSG00000269889	3	169769649	169772043	antisense	6,50	0
ENSG00000273437	ENSG00000273437	3	129163606	129163940	lincRNA	6,50	0
ENSG00000234969	ENSG00000234969	X	74069276	74070832	processed_pseudogene	6,50	1,40E-02
ENSG00000224093	ENSG00000224093	1	93592199	93605573	antisense	6,48	0
ENSG00000233247	ENSG00000233247	X	14244382	14244928	processed_pseudogene	6,48	0
ENSG00000226864	ATE1-AS1	10	121928312	121951965	antisense	6,48	3,30E-03
ENSG00000215158	ENSG00000215158	5	34164698	34244796	transcribed_unprocessed_pseudogene	6,47	0
ENSG00000261766	ENSG00000261766	16	28862166	28863340	antisense	6,46	0
ENSG00000225880	LINC00115	1	826206	827522	lincRNA	6,46	4,86E-02
ENSG00000269300	ENSG00000269300	19	8821501	8824110	antisense	6,46	0
ENSG00000272894	ENSG00000272894	7	7552462	7566996	lincRNA	6,46	0
ENSG00000205041	ENSG00000205041	19	40273489	40275479	sense_intronic	6,45	3,30E-03
ENSG00000234773	ENSG00000234773	19	12195015	12237767	lincRNA	6,44	0
ENSG00000274457	ENSG00000274457	22	29437583	29437733	unprocessed_pseudogene	6,40	1,40E-02
ENSG00000269959	SPACA6P-AS	19	51685363	51693456	lincRNA	6,40	0
ENSG00000274015	ENSG00000274015	14	63642143	63642696	lincRNA	6,40	7,06E-02
ENSG00000226604	PAPPA-AS2	9	116285829	116288769	antisense	6,40	0
ENSG00000232283	ENSG00000232283	9	96246462	96250393	antisense	6,40	7,06E-02
ENSG00000254548	ENSG00000254548	8	143718246	143718891	antisense	6,40	0
ENSG00000237815	ENSG00000237815	7	77060345	77060479	processed_pseudogene	6,40	0
ENSG00000220748	ENSG00000220748	6	23971879	23972941	processed_pseudogene	6,40	0
ENSG00000260273	ENSG00000260273	6	109382795	109383666	antisense	6,40	0
ENSG00000249572	ENSG00000249572	5	33424025	33440619	antisense	6,40	0
ENSG00000247950	SEC24B-AS1	4	109347475	109433817	antisense	6,40	1,40E-02
ENSG00000232439	RPL18AP7	3	38526802	38527325	processed_pseudogene	6,40	0
ENSG00000272148	ENSG00000272148	2	27062428	27062907	antisense	6,40	0
ENSG00000272432	ENSG00000272432	1	25247837	25248321	lincRNA	6,40	0
ENSG00000239883	PARGP1	10	45854093	45972154	transcribed_unprocessed_pseudogene	6,39	0
ENSG00000226149	ENSG00000226149	6	129526626	129552587	lincRNA	6,38	0
ENSG00000175147	TMEM51-AS1	1	15111815	15152464	antisense	6,38	3,30E-03
ENSG00000272948	ENSG00000272948	21	37267784	37268497	antisense	6,38	0
ENSG00000245330	ENSG00000245330	8	119867419	119874488	lincRNA	6,38	0
ENSG00000272269	ENSG00000272269	6	17706257	17707344	antisense	6,38	0

ENSG00000248898	ENSG00000248898	5	52675193	52788026	antisense	6,37	6,27E-03
ENSG00000268225	ENSG00000268225	19	52861807	52862866	processed_pseudogene	6,36	0
ENSG00000230186	ENSG00000230186	1	143905487	143934776	antisense	6,35	0
ENSG00000267270	PARD6G-AS1	18	80147924	80178432	antisense	6,34	0
ENSG00000131401	NAPSB	19	50333796	50344767	transcribed_unprocessed_pseudogene	6,33	0
ENSG00000255986	MT1JP	16	56635739	56637086	transcribed_unprocessed_pseudogene	6,33	0,1392851
ENSG00000274943	ENSG00000274943	12	42361267	42361703	sense_intronic	6,32	0
ENSG00000262884	ENSG00000262884	17	2962248	2965895	antisense	6,31	0
ENSG00000278416	PMS2P2	7	75344015	75359550	unprocessed_pseudogene	6,31	9,18E-02
ENSG00000267082	ENSG00000267082	19	11203628	11216168	antisense	6,30	0
ENSG00000261783	ENSG00000261783	16	75379818	75381260	sense_intronic	6,30	1,40E-02
ENSG00000277050	ENSG00000277050	14	51637348	51637947	antisense	6,30	7,06E-02
ENSG00000238286	SLC35E1P1	13	20607268	20608131	processed_pseudogene	6,30	0
ENSG00000260910	LINC00565	13	113926514	113928844	lincRNA	6,30	1,40E-02
ENSG00000276916	ENSG00000276916	13	113511747	113514473	sense_intronic	6,30	0
ENSG00000257221	ENSG00000257221	12	108628687	108641318	antisense	6,30	1,40E-02
ENSG00000270130	ENSG00000270130	12	123960717	123961244	sense_intronic	6,30	0
ENSG00000272696	ENSG00000272696	9	128316337	128316909	antisense	6,30	7,06E-02
ENSG00000214243	ENSG00000214243	7	76650401	76650897	processed_pseudogene	6,30	0
ENSG00000242593	ENSG00000242593	7	124032205	124395118	antisense	6,30	1,40E-02
ENSG00000249767	ENPP7P10	4	9079023	9141608	unprocessed_pseudogene	6,30	0
ENSG00000241429	EEF1A1P25	3	138825063	138826329	processed_pseudogene	6,30	7,06E-02
ENSG00000244124	ATP1B3-AS1	3	141918252	141919021	antisense	6,30	0
ENSG00000224376	ENSG00000224376	2	231388976	231394991	processed_transcript	6,30	7,06E-02
ENSG00000282111	ENSG00000282111	1	229022773	229038274	lincRNA	6,30	0
ENSG00000237917	PARP4P1	X	26594851	26634652	unprocessed_pseudogene	6,30	7,06E-02
ENSG00000213700	RPL17P50	10	73005833	73006595	processed_pseudogene	6,29	0,0358681
ENSG00000225822	UBXN7-AS1	3	196431385	196432530	antisense	6,29	0
ENSG00000177640	CASC2	10	118046279	118210153	antisense	6,29	0
ENSG00000272579	ENSG00000272579	1	193090866	193091556	antisense	6,29	0
ENSG00000180105	ENSG00000180105	2	206410087	206410909	processed_pseudogene	6,27	0
ENSG00000196741	LINC01560	X	47483571	47484823	lincRNA	6,27	0
ENSG00000242553	ENSG00000242553	21	37221419	37237744	lincRNA	6,27	0
ENSG00000226259	GTF2H2B	5	70415352	70448015	transcribed_unprocessed_pseudogene	6,27	0
ENSG00000230701	FBXW4P1	22	23262767	23265005	processed_pseudogene	6,25	6,27E-03
ENSG00000240509	RPL34P18	8	93957338	93957691	processed_pseudogene	6,24	0
ENSG00000267309	ENSG00000267309	19	36489649	36491040	antisense	6,23	0
ENSG00000261173	ENSG00000261173	16	46973989	46978983	antisense	6,23	0
ENSG00000272750	ENSG00000272750	1	222658867	222661512	antisense	6,23	0
ENSG00000255642	PABPC1P4	12	63822021	63823895	processed_pseudogene	6,22	0
ENSG00000260448	LCMT1-AS1	16	25085592	25111555	lincRNA	6,22	0
ENSG00000228137	ENSG00000228137	21	46246890	46247682	antisense	6,20	0
ENSG00000250731	TPM3P6	19	53479350	53480091	processed_pseudogene	6,20	0
ENSG00000270614	ENSG00000270614	19	9348106	9348703	processed_pseudogene	6,20	0
ENSG00000263847	ENSG00000263847	18	9112404	9115877	antisense	6,20	0
ENSG00000215097	DUSP8P3	10	46774421	46776180	processed_pseudogene	6,20	1,40E-02
ENSG00000227388	ENSG00000227388	9	35772163	35790432	lincRNA	6,20	0
ENSG00000230295	GTF2IP23	7	66880708	66882981	unprocessed_pseudogene	6,20	0
ENSG00000243305	ENSG00000243305	3	152457759	152496813	antisense	6,20	0
ENSG00000233806	LINC01237	2	241881363	242078722	processed_transcript	6,20	0
ENSG00000272512	ENSG00000272512	1	995966	998051	lincRNA	6,20	0,2161218
ENSG00000236188	PRKX-AS1	X	3659487	3668192	antisense	6,20	0
ENSG00000250492	INTS6P1	5	39718984	39721513	processed_pseudogene	6,18	0
ENSG00000225793	ENSG00000225793	6	75285014	75297003	lincRNA	6,18	0

ENSG00000268403	ENSG00000268403	11	9459556	9460702	antisense	6,17	0
ENSG00000261079	ENSG00000261079	16	74367462	74369826	lincRNA	6,17	0
ENSG00000271966	ENSG00000271966	8	67343975	67345087	lincRNA	6,17	4,86E-02
ENSG00000253629	ENSG00000253629	8	101686547	101689093	antisense	6,16	9,18E-02
ENSG00000237868	ENSG00000237868	4	122077840	122078318	processed_pseudogene	6,16	0
ENSG00000262115	ENSG00000262115	17	81197393	81200288	antisense	6,15	0
ENSG00000261460	ENSG00000261460	15	72278867	72351794	antisense	6,15	0
ENSG00000279636	LINC00216	14	58288033	58289158	lincRNA	6,14	0
ENSG00000205861	C1QTNF9B-AS1	13	23888889	23897263	antisense	6,14	2,21E-02
ENSG00000248079	DPH6-AS1	15	35546195	35859001	lincRNA	6,14	0
ENSG00000225339	ENSG00000225339	6	34248568	34286768	processed_transcript	6,13	0
ENSG00000235413	KRT18P63	4	17911674	17912976	processed_pseudogene	6,12	0
ENSG00000267623	ENSG00000267623	19	27902595	27984743	transcribed_unprocessed_pseudogene	6,10	1,40E-02
ENSG00000259409	ENSG00000259409	15	40088832	40089386	antisense	6,10	0
ENSG00000213411	RBM22P2	13	30539597	30543461	processed_pseudogene	6,10	0,2161218
ENSG00000237672	KRR1P1	13	20070250	20072136	processed_pseudogene	6,10	0
ENSG00000255968	ENSG00000255968	12	26318953	26421462	antisense	6,10	0
ENSG00000254756	ENSG00000254756	11	66334494	66339875	antisense	6,10	1,40E-02
ENSG00000222012	ENSG00000222012	7	157614023	157618765	antisense	6,10	1,40E-02
ENSG00000226102	SEPT7P3	7	35946294	35972587	unprocessed_pseudogene	6,10	0,2161218
ENSG00000227436	FCF1P1	7	23619333	23622826	processed_pseudogene	6,10	7,06E-02
ENSG00000250474	WBP1LP2	7	107628553	107629498	processed_pseudogene	6,10	0
ENSG00000245711	NADK2-AS1	5	36221055	36221902	antisense	6,10	0
ENSG00000228852	ENSG00000228852	1	95243167	95278940	antisense	6,10	7,06E-02
ENSG00000236065	ENSG00000236065	1	32987075	33032469	antisense	6,10	0
ENSG00000260528	FAM157C	16	90102271	90186204	lincRNA	6,08	0
ENSG00000253228	NRBF2P4	8	107983116	107983965	processed_pseudogene	6,07	0
ENSG00000217791	ASS1P9	5	53859166	53860401	processed_pseudogene	6,07	0,2161218
ENSG00000242588	ENSG00000242588	7	128574751	128622694	processed_transcript	6,07	0
ENSG00000224224	HAUS1P2	X	18865771	18866554	processed_pseudogene	6,07	6,27E-03
ENSG00000277873	ENSG00000277873	12	118024817	118025518	antisense	6,06	0
ENSG00000237768	ENSG00000237768	10	73071295	73074008	antisense	6,06	0
ENSG00000228486	LINC01125	2	97664217	97703064	antisense	6,04	0
ENSG00000232024	LSM12P1	8	35525176	35525763	processed_pseudogene	6,03	0
ENSG00000257267	ZNF271P	18	35290282	35310766	unitary_pseudogene	6,03	0
ENSG00000245482	ENSG00000245482	12	34022281	34046417	antisense	6,03	0
ENSG00000227398	KIF9-AS1	3	47164497	47246601	antisense	6,02	0
ENSG00000273192	ENSG00000273192	22	49902228	49904576	lincRNA	6,01	0
ENSG00000224790	ENSG00000224790	21	36966512	36971828	lincRNA	6,00	1,40E-02
ENSG00000270112	ENSG00000270112	18	46756487	46764408	processed_transcript	6,00	0
ENSG00000264290	ENSG00000264290	17	29569580	29570519	sense_intronic	6,00	0
ENSG00000267042	ENSG00000267042	17	42679963	42682020	antisense	6,00	1,40E-02
ENSG00000277597	ENSG00000277597	17	1684726	1685151	antisense	6,00	0
ENSG00000258603	ENSG00000258603	14	73616700	73633941	antisense	6,00	0
ENSG00000259015	ENSG00000259015	14	72960595	72961993	processed_pseudogene	6,00	0
ENSG00000250133	HOXC-AS2	12	53993810	53996785	processed_transcript	6,00	0
ENSG00000256678	ENSG00000256678	12	68805011	68805479	processed_pseudogene	6,00	1,40E-02
ENSG00000277130	ENSG00000277130	12	79818784	79819465	sense_intronic	6,00	0
ENSG00000246523	ENSG00000246523	11	86955619	87000959	lincRNA	6,00	0
ENSG00000196366	C9orf163	9	136483495	136486067	lincRNA	6,00	0
ENSG00000250714	ENSG00000250714	8	23225233	23230915	antisense	6,00	0
ENSG00000253553	ENSG00000253553	8	88326836	88737134	antisense	6,00	0
ENSG00000216331	HIST1H1PS1	6	26195566	26195771	unprocessed_pseudogene	6,00	0
ENSG00000241458	RPL7P19	5	138473744	138474361	processed_pseudogene	6,00	0
ENSG00000248216	KCTD9P5	5	37505688	37506857	processed_pseudogene	6,00	0
ENSG00000232202	ENSG00000232202	2	42826322	42827617	processed_pseudogene	6,00	0
ENSG00000261117	ENSG00000261117	2	12715415	12716227	lincRNA	6,00	0
ENSG00000225171	DUTP6	1	166868748	166869209	processed_pseudogene	6,00	1,40E-02

ENSG00000230787	PSAT1P3	1	79054945	79056055	processed_pseudogene	6,00	0
ENSG00000271914	ENSG00000271914	1	35929720	35930115	lincRNA	6,00	1,40E-02
ENSG00000280486	ENSG00000280486	19	1386804	1389651	antisense	5,99	0
ENSG00000243680	RPL37P23	19	52143043	52143336	processed_pseudogene	5,98	1,40E-02
ENSG00000184669	OR7E14P	11	17013998	17053024	transcribed_unprocessed_pseudogene	5,95	6,27E-03
ENSG00000259544	ENSG00000259544	15	85726115	85727227	antisense	5,95	0
ENSG00000262601	ENSG00000262601	16	85171276	85185363	lincRNA	5,95	0
ENSG00000123009	NME2P1	12	120282303	120282716	processed_pseudogene	5,95	0,0358681
ENSG00000272779	ENSG00000272779	22	22303224	22310401	transcribed_unprocessed_pseudogene	5,95	0
ENSG00000261294	ENSG00000261294	16	1206560	1207124	antisense	5,94	0
ENSG00000259429	UBE2Q2P2	15	82355142	82420075	transcribed_unprocessed_pseudogene	5,93	0
ENSG00000263829	FAM60BP	18	26255698	26256355	processed_pseudogene	5,93	0
ENSG00000214391	TUBAP2	11	90282560	90284172	processed_pseudogene	5,92	1,40E-02
ENSG00000257042	ENSG00000257042	12	27958517	27969813	antisense	5,91	0,3318209
ENSG00000242457	RBBP4P2	3	94075912	94077175	processed_pseudogene	5,91	0
ENSG00000266903	ENSG00000266903	19	44632199	44718759	antisense	5,90	1,40E-02
ENSG00000267107	PCAT19	19	41454169	41500649	lincRNA	5,90	0
ENSG00000265533	ENSG00000265533	18	67516546	67899619	lincRNA	5,90	7,06E-02
ENSG00000267456	ENSG00000267456	18	36278735	36279119	processed_pseudogene	5,90	0
ENSG00000235085	ENSG00000235085	17	4673830	4696831	antisense	5,90	0
ENSG00000266970	ENSG00000266970	17	78360453	78373911	lincRNA	5,90	0
ENSG00000260735	ENSG00000260735	16	15094411	15109197	antisense	5,90	0
ENSG00000263276	ENSG00000263276	16	68224713	68227734	sense_overlapping	5,90	0
ENSG00000232018	ENSG00000232018	14	100960509	100967040	lincRNA	5,90	7,06E-02
ENSG00000258930	ENSG00000258930	14	71031686	71031970	transcribed_processed_pseudogene	5,90	1,40E-02
ENSG00000277662	ENSG00000277662	13	41229180	41229676	sense_intronic	5,90	0
ENSG00000249550	LINC01234	12	113679459	113773683	lincRNA	5,90	7,06E-02
ENSG00000256234	ENSG00000256234	12	26211164	26335856	antisense	5,90	0
ENSG00000257740	ENSG00000257740	12	56308868	56309449	antisense	5,90	0
ENSG00000274105	ENSG00000274105	12	32728169	32729024	sense_intronic	5,90	0
ENSG00000260398	ENSG00000260398	8	78605952	78609705	sense_overlapping	5,90	0
ENSG00000272375	ENSG00000272375	8	30197404	30198048	lincRNA	5,90	1,40E-02
ENSG00000281881	SPRY4-IT1	5	142317620	142318322	lincRNA	5,90	0
ENSG00000226423	ENSG00000226423	2	242025183	242026176	lincRNA	5,90	1,40E-02
ENSG00000273305	ENSG00000273305	2	95537969	95538469	lincRNA	5,90	0
ENSG00000268883	PNMA6B	X	153075769	153076968	unprocessed_pseudogene	5,90	0,2161218
ENSG00000270820	ENSG00000270820	2	61471188	61484130	antisense	5,88	0
ENSG00000233020	ENSG00000233020	1	70530526	70531492	processed_pseudogene	5,87	0
ENSG00000259201	ENSG00000259201	15	51887560	51901123	antisense	5,87	0
ENSG00000224773	HSPA8P7	X	114774828	114776775	processed_pseudogene	5,87	0
ENSG00000229598	PRDX3P1	22	38722743	38723505	processed_pseudogene	5,86	2,21E-02
ENSG00000267454	ZNF582-AS1	19	56393656	56399172	lincRNA	5,83	3,30E-03
ENSG00000267248	ENSG00000267248	17	60126535	60135644	processed_transcript	5,83	0,1836819
ENSG00000254694	ENSG00000254694	11	126208611	126209027	antisense	5,83	0
ENSG00000234818	ENSG00000234818	2	10589166	10604830	antisense	5,83	0
ENSG00000271855	ENSG00000271855	2	9555899	9556775	lincRNA	5,83	2,95E-02
ENSG00000271011	ENSG00000271011	2	178577103	178577622	antisense	5,83	0
ENSG00000231445	TIMM8AP1	2	162077357	162077651	processed_pseudogene	5,81	0
ENSG00000224903	ENSG00000224903	7	156654185	156657693	antisense	5,81	1,40E-02
ENSG00000273076	ENSG00000273076	22	38743495	38743910	antisense	5,80	1,40E-02
ENSG00000275632	ENSG00000275632	20	6000418	6000941	lincRNA	5,80	7,06E-02
ENSG00000268758	ADGRE4P	19	6952500	6990846	transcribed_unprocessed_pseudogene	5,80	0
ENSG00000282393	ENSG00000282393	19	248551	251571	lincRNA	5,80	0
ENSG00000264031	ABHD15-AS1	17	29560547	29707090	antisense	5,80	1,40E-02
ENSG00000280734	LINC01232	13	99486962	99499306	processed_transcript	5,80	0

ENSG00000254928	ENSG00000254928	11	74455348	74456825	antisense	5,80	0
ENSG00000224215	ENSG00000224215	10	23343957	23345181	antisense	5,80	1,40E-02
ENSG00000261366	MANEA-AS1	6	95575183	95577450	antisense	5,80	0
ENSG00000215196	ENSG00000215196	5	17130028	17217047	antisense	5,80	0
ENSG00000259863	SH3RF3-AS1	2	109127327	109128930	lincRNA	5,80	0
ENSG00000237623	ENSG00000237623	X	47273366	47274390	processed_pseudogene	5,80	0
ENSG00000229502	ENSG00000229502	6	157872571	157875210	antisense	5,79	0
ENSG00000144158	ENSG00000144158	2	113657908	113658675	processed_pseudogene	5,79	0
ENSG00000228252	COL6A4P2	3	130212823	130273806	unitary_pseudogene	5,79	6,27E-03
ENSG00000259102	SMARCE1P3	14	21162513	21163743	processed_pseudogene	5,79	1,40E-02
ENSG00000254244	PAICSP4	8	4787332	4788584	processed_pseudogene	5,78	0
ENSG00000237836	PHKA2-AS1	X	18890296	18894497	antisense	5,78	6,27E-03
ENSG00000214192	UBE2V1P2	3	175718231	175718670	processed_pseudogene	5,78	0
ENSG00000214144	ENSG00000214144	1	247229940	247231880	processed_pseudogene	5,77	7,06E-02
ENSG00000253722	ENSG00000253722	8	93733216	93734022	antisense	5,75	0
ENSG00000224431	ENSG00000224431	5	132199456	132203487	processed_pseudogene	5,75	0
ENSG00000233994	GDI2P2	1	72274552	72275159	processed_pseudogene	5,74	0
ENSG00000256250	ENSG00000256250	12	130810606	130812438	lincRNA	5,71	1,40E-02
ENSG00000225849	ENSG00000225849	20	46463902	46465124	processed_pseudogene	5,70	1,40E-02
ENSG00000267757	EML2-AS1	19	45641494	45642840	processed_transcript	5,70	0
ENSG00000264808	ENSG00000264808	17	29369717	29390777	antisense	5,70	1,40E-02
ENSG00000264943	SH3GL1P2	17	30624413	30625494	processed_pseudogene	5,70	1,40E-02
ENSG00000265664	ENSG00000265664	17	67032409	67033290	antisense	5,70	1,40E-02
ENSG00000277214	ENSG00000277214	16	79603572	79604177	lincRNA	5,70	0
ENSG00000261187	ENSG00000261187	15	72465128	72466262	lincRNA	5,70	0,2161218
ENSG00000276172	ELMO2P1	15	23083094	23121324	transcribed_unprocessed_pseudogene	5,70	7,06E-02
ENSG00000259129	LINC00648	14	47764954	47795092	lincRNA	5,70	0,2161218
ENSG00000212125	TAS2R15P	12	10964425	10965352	processed_pseudogene	5,70	1,40E-02
ENSG00000247131	ENSG00000247131	12	69713633	69738568	antisense	5,70	1,40E-02
ENSG00000229587	ENSG00000229587	9	75009828	75016036	sense_intronic	5,70	1,40E-02
ENSG00000260454	ENSG00000260454	9	90997054	91001871	lincRNA	5,70	7,06E-02
ENSG00000184616	SPDYE12P	7	74906673	74913256	unprocessed_pseudogene	5,70	0
ENSG00000248577	ENSG00000248577	5	38763158	38764289	processed_pseudogene	5,70	0,2161218
ENSG00000271993	ENSG00000271993	3	37182107	37182734	antisense	5,70	0,2161218
ENSG00000204581	ENSG00000204581	2	111098345	111115588	antisense	5,70	0
ENSG00000225938	ENSG00000225938	1	101235683	101236528	antisense	5,70	0
ENSG00000227536	SOCS5P4	X	71043214	71045470	processed_pseudogene	5,70	7,06E-02
ENSG00000245060	LINC00847	5	180830957	180835726	lincRNA	5,69	0
ENSG00000258983	ENSG00000258983	14	88499334	88515502	antisense	5,68	0
ENSG00000267265	ENSG00000267265	19	55006193	55048086	antisense	5,68	0
ENSG00000267165	ENSG00000267165	18	11851414	11852751	antisense	5,68	0
ENSG00000230615	ENSG00000230615	1	44030443	44115913	lincRNA	5,67	0
ENSG00000182625	ENSG00000182625	15	58686411	58686617	processed_pseudogene	5,67	0
ENSG00000279623	ENSG00000279623	10	21865335	21865921	sense_intronic	5,67	0,1836819
ENSG00000250988	SNHG21	15	82750564	82757206	antisense	5,65	4,86E-02
ENSG00000229638	RPL4P4	3	185417495	185418778	processed_pseudogene	5,64	0
ENSG00000180673	EXOC5P1	4	62816826	62818794	processed_pseudogene	5,61	0
ENSG00000234353	ENSG00000234353	22	23638492	23640762	transcribed_unprocessed_pseudogene	5,60	7,06E-02
ENSG00000279954	ENSG00000279954	22	45875932	45879247	antisense	5,60	0
ENSG00000267874	ENSG00000267874	19	35036334	35037060	sense_intronic	5,60	1,40E-02
ENSG00000268204	ENSG00000268204	19	7633766	7636990	antisense	5,60	1,40E-02
ENSG00000268278	ENSG00000268278	19	21637974	21656300	unprocessed_pseudogene	5,60	0
ENSG00000270325	BNIP3P9	19	19856975	19857527	processed_pseudogene	5,60	0,2161218
ENSG00000265139	ENSG00000265139	17	32328441	32329395	lincRNA	5,60	1,40E-02
ENSG00000261273	ENSG00000261273	16	88234785	88302511	lincRNA	5,60	0
ENSG00000253284	ENSG00000253284	12	19147074	19154659	sense_intronic	5,60	0
ENSG00000257496	ENSG00000257496	12	46384233	46386991	lincRNA	5,60	7,06E-02

ENSG00000274315	ENSG00000274315	12	29331434	29331936	antisense	5,60	0
ENSG00000275703	ENSG00000275703	12	6899752	6900212	lincRNA	5,60	1,40E-02
ENSG00000213234	ST13P10	11	112267290	112268371	processed_pseudogene	5,60	0
ENSG00000255176	ENSG00000255176	11	118636620	118638097	antisense	5,60	1,40E-02
ENSG00000225850	ENSG00000225850	10	97334564	97343203	antisense	5,60	0
ENSG00000236184	TCEA1P4	9	32979560	32980403	processed_pseudogene	5,60	0
ENSG00000254281	ENSG00000254281	8	102978785	103000184	lincRNA	5,60	0
ENSG00000203808	BVES-AS1	6	105136308	105169945	antisense	5,60	0
ENSG00000225407	ENSG00000225407	5	76691439	76716215	antisense	5,60	0
ENSG00000248692	ADGRL3-AS1	4	62071752	62165554	antisense	5,60	0
ENSG00000250410	ENSG00000250410	4	185370725	185390928	antisense	5,60	7,06E-02
ENSG00000229325	ACAP2-IT1	3	195280723	195282741	sense_intronic	5,60	0
ENSG00000265451	ENSG00000265451	2	121530422	121532705	antisense	5,60	0
ENSG00000226758	DISC1-IT1	1	231925834	231945233	sense_intronic	5,60	0
ENSG00000271997	ENSG00000271997	15	88501944	88505787	sense_intronic	5,59	0
ENSG00000261586	ENSG00000261586	12	51817899	51820150	lincRNA	5,59	0,3318209
ENSG00000273080	ENSG00000273080	2	86195590	86196049	antisense	5,59	7,06E-02
ENSG00000253738	OTUD6B-AS1	8	91059909	91070189	antisense	5,59	0
ENSG00000235903	CPB2-AS1	13	46052806	46113332	antisense	5,58	0
ENSG00000213080	ENSG00000213080	1	160894980	160896076	processed_pseudogene	5,55	0
ENSG00000254099	ENSG00000254099	5	141970637	141982687	antisense	5,55	0
ENSG00000241547	ACTG1P20	1	27325329	27325796	processed_pseudogene	5,54	0
ENSG00000262429	ENSG00000262429	17	4967995	4968822	antisense	5,54	0
ENSG00000215284	ENSG00000215284	X	46725164	46725818	processed_pseudogene	5,54	2,21E-02
ENSG00000236700	LINC01010	6	134437716	134504581	lincRNA	5,53	6,27E-03
ENSG00000257884	ENSG00000257884	14	19677837	19680104	transcribed_processed_pseudogene	5,52	6,27E-03
ENSG00000243663	RPS4XP14	12	1757231	1758011	processed_pseudogene	5,51	4,86E-02
ENSG00000266924	ENSG00000266924	18	79787121	79791432	lincRNA	5,50	1,40E-02
ENSG00000266957	ENSG00000266957	18	47077361	47091280	antisense	5,50	1,40E-02
ENSG00000262500	ENSG00000262500	17	46243606	46245044	processed_pseudogene	5,50	1,40E-02
ENSG00000267547	ENSG00000267547	17	35403837	35404373	lincRNA	5,50	0
ENSG00000271009	ENSG00000271009	16	73060470	73062316	lincRNA	5,50	7,06E-02
ENSG00000258982	ENSG00000258982	14	100207407	100238555	antisense	5,50	7,06E-02
ENSG00000259065	ENSG00000259065	14	73787360	73803270	antisense	5,50	0
ENSG00000259648	ENSG00000259648	14	67614929	67616626	transcribed_processed_pseudogene	5,50	7,06E-02
ENSG00000273711	ENSG00000273711	14	73885392	73885555	antisense	5,50	0,2161218
ENSG00000278743	ENSG00000278743	12	25385670	25386241	lincRNA	5,50	0
ENSG00000227896	ENSG00000227896	10	86521945	86525101	antisense	5,50	0,4036702
ENSG00000229656	ENSG00000229656	10	32958845	33082102	lincRNA	5,50	1,40E-02
ENSG00000234175	ENSG00000234175	10	13139196	13139533	processed_pseudogene	5,50	0
ENSG00000204055	ENSG00000204055	9	129176771	129210548	antisense	5,50	9,18E-02
ENSG00000224846	ENSG00000224846	6	2989722	2999134	antisense	5,50	0,1836819
ENSG00000214273	AGGF1P1	4	190041342	190043414	processed_pseudogene	5,50	7,06E-02
ENSG00000241560	ZBTB20-AS1	3	114351811	114388978	antisense	5,50	0
ENSG00000239300	ENSG00000239300	2	9505445	9512412	antisense	5,50	1,40E-02
ENSG00000281937	ENSG00000281937	1	9197143	9198906	lincRNA	5,50	1,40E-02
ENSG00000229563	LINC01204	X	45505388	45630202	lincRNA	5,50	0
ENSG00000235522	ENSG00000235522	2	105846534	105857177	antisense	5,50	0
ENSG00000168405	CMAHP	6	25081068	25166555	unitary_pseudogene	5,50	0
ENSG00000235106	LINC00094	9	134025439	134034666	antisense	5,50	0
ENSG00000244490	RWDD4P1	7	105301522	105302086	processed_pseudogene	5,49	0
ENSG00000232767	ENSG00000232767	10	116670103	116672739	antisense	5,49	1,40E-02
ENSG00000267348	ENSG00000267348	19	45076510	45090391	antisense	5,48	0
ENSG00000227954	TARID	6	133502252	133892802	antisense	5,48	9,18E-02
ENSG00000268460	ENSG00000268460	19	46203426	46214837	processed_transcript	5,47	0,4332376
ENSG00000269807	ENSG00000269807	19	4347244	4354057	antisense	5,47	0
ENSG00000182921	CCDC75P1	3	73182501	73183291	processed_pseudogene	5,47	2,21E-02
ENSG00000281021	ENSG00000281021	6	149576089	149590864	antisense	5,46	0
ENSG00000249435	ENSG00000249435	19	53426019	53426499	processed_pseudogene	5,45	0

ENSG00000269151	ENSG00000269151	19	46390515	46390852	antisense		5,45	1,40E-02
ENSG00000224729	PCOLCE-AS1	7	100589402	100604206	antisense		5,45	0
ENSG00000276863	ENSG00000276863	17	80333841	80334366	antisense		5,45	0
ENSG00000249476	ENSG00000249476	5	109237120	109326369	lincRNA		5,44	0,3318209
ENSG00000228110	ST13P19	1	210265636	210267241	processed_pseudogene		5,44	0
ENSG00000230882	ENSG00000230882	7	76071469	76074963	processed_pseudogene		5,43	0
ENSG00000223650	UHRF2P1	X	74105572	74107958	processed_pseudogene		5,42	0
ENSG00000272944	ENSG00000272944	2	221572506	221574454	antisense		5,41	3,30E-03
ENSG00000236296	GUSBP5	4	143559472	143649305	transcribed_processed_pseudogene		5,40	0
ENSG00000272367	ENSG00000272367	5	87376256	87377180	antisense		5,40	0
ENSG00000268564	ENSG00000268564	19	15124160	15126174	antisense		5,40	0
ENSG00000227158	ENSG00000227158	17	17422681	17423704	processed_pseudogene		5,40	0
ENSG00000266236	NARF-IT1	17	82482098	82483388	sense_intronic		5,40	0
ENSG00000260628	ENSG00000260628	16	31975803	32003728	transcribed_unprocessed_pseudogene		5,40	1,40E-02
ENSG00000260144	ENSG00000260144	15	72605183	72612326	unprocessed_pseudogene		5,40	1,40E-02
ENSG00000257509	ENSG00000257509	12	55761550	55762628	sense_intronic		5,40	0
ENSG00000237512	UNC5B-AS1	10	71217224	71218228	antisense		5,40	7,06E-02
ENSG00000228707	ENSG00000228707	9	116012361	116012965	lincRNA		5,40	7,06E-02
ENSG00000213435	ATP6V0CP3	6	42727234	42727700	processed_pseudogene		5,40	1,40E-02
ENSG00000219163	HMGB1P20	6	53235621	53236297	processed_pseudogene		5,40	1,40E-02
ENSG00000229950	TFAP2A-AS1	6	10409340	10416446	antisense		5,40	0,2161218
ENSG00000271888	ENSG00000271888	6	15243923	15245000	lincRNA		5,40	0,2161218
ENSG00000250403	ENSG00000250403	4	99990737	99991477	processed_pseudogene		5,40	1,40E-02
ENSG00000273449	ENSG00000273449	4	163529771	163530697	lincRNA		5,40	7,06E-02
ENSG00000234199	LINC01191	2	113979569	114007302	lincRNA		5,40	0,2161218
ENSG00000236431	ENSG00000236431	2	95536117	95536495	processed_pseudogene		5,40	0
ENSG00000236269	ENO1-IT1	1	8875788	8878007	sense_intronic		5,40	0
ENSG00000254942	ENSG00000254942	1	109539906	109543837	antisense		5,40	1,40E-02
ENSG00000242628	ENSG00000242628	2	24214381	24221516	antisense		5,40	0
ENSG00000228509	ENSG00000228509	2	190676944	190708716	antisense		5,38	0
ENSG00000204044	ENSG00000204044	20	46013500	46022073	antisense		5,38	0
ENSG00000265182	SRP72P1	18	49495147	49496896	processed_pseudogene		5,38	2,95E-02
ENSG00000261779	ENSG00000261779	15	75211301	75212167	antisense		5,37	3,30E-03
ENSG00000274922	ENSG00000274922	13	112686769	112689815	lincRNA		5,37	0
ENSG00000276317	ENSG00000276317	20	62651272	62652186	antisense		5,36	0
ENSG00000271614	LINC00936	12	89708959	89712590	lincRNA		5,36	0
ENSG00000236308	ENSG00000236308	10	100190036	100190747	antisense		5,36	0
ENSG00000214264	KCTD9P4	11	112180773	112183191	processed_pseudogene		5,36	0
ENSG00000277072	STAG3L2	7	74882163	74890610	transcribed_unprocessed_pseudogene		5,35	0
ENSG00000267892	ENSG00000267892	19	38738284	38739863	antisense		5,35	1,95E-02
ENSG00000235635	ENSG00000235635	5	56941307	56947152	antisense		5,35	0
ENSG00000213178	ENSG00000213178	3	169483671	169484080	processed_pseudogene		5,34	0
ENSG00000215630	GUSBP9	5	71197646	71208130	unprocessed_pseudogene		5,33	0
ENSG00000178631	ACTG1P1	3	139493809	139494937	processed_pseudogene		5,33	0,4850164
ENSG00000225602	MTOR-AS1	1	11143898	11149537	antisense		5,33	0
ENSG00000228170	ENSG00000228170	6	3138394	3153062	antisense		5,31	0
ENSG00000260552	ENSG00000260552	18	36179996	36187448	antisense		5,31	6,27E-03
ENSG00000254503	ENSG00000254503	19	17417031	17418288	processed_pseudogene		5,30	0
ENSG00000260060	ENSG00000260060	16	31196131	31196963	antisense		5,30	0,2161218
ENSG00000260394	ENSG00000260394	16	678504	679777	antisense		5,30	7,06E-02
ENSG00000262904	TMPOP2	16	74667506	74668706	processed_pseudogene		5,30	0,2161218
ENSG00000258571	PTTG4P	14	71085482	71085833	processed_pseudogene		5,30	0
ENSG00000235049	LINC00940	12	1929202	1936574	lincRNA		5,30	0
ENSG00000256633	ENSG00000256633	11	72584572	72587979	antisense		5,30	0
ENSG00000246366	LACTB2-AS1	8	70608577	70663279	antisense		5,30	0
ENSG00000255366	ENSG00000255366	8	47190772	47193262	lincRNA		5,30	0

ENSG00000272115	ENSG00000272115	8	144409492	144409976	antisense	5,30	0
ENSG00000236591	ENSG00000236591	6	149027700	149032573	antisense	5,30	7,06E-02
ENSG00000254333	ENSG00000254333	5	150475531	150485968	lincRNA	5,30	0
ENSG00000250238	ENSG00000250238	4	105959	107114	processed_pseudogene	5,30	0,2161218
ENSG00000271746	ENSG00000271746	1	6393555	6394391	antisense	5,30	0,2161218
ENSG00000274536	ENSG00000274536	X	66015461	66020422	antisense	5,30	0
ENSG00000250602	ENSG00000250602	5	126433505	126490371	antisense	5,29	0
ENSG00000253954	HMGN1P38	15	92711808	92712110	processed_pseudogene	5,29	0
ENSG00000213793	ZNF888	19	52915196	52923470	lincRNA	5,25	0
ENSG00000241975	TCEB1P19	1	88829102	88829419	processed_pseudogene	5,25	0
ENSG00000260077	ENSG00000260077	2	10039092	10040663	lincRNA	5,25	0
ENSG00000272888	LINC01578	15	92882707	92899701	processed_transcript	5,23	3,30E-03
ENSG00000223442	TH2LCRR	5	132630589	132664272	antisense	5,23	0
ENSG00000255893	ENSG00000255893	11	94472908	94473570	antisense	5,23	1,40E-02
ENSG00000255585	ENSG00000255585	9	137776539	137789235	transcribed_processed_pseudogene	5,21	0
ENSG00000224578	HNRNPA1P48	16	51645756	51647132	transcribed_processed_pseudogene	5,21	0
ENSG00000267396	ENSG00000267396	18	58398663	58400082	antisense	5,21	6,27E-03
ENSG00000249626	ENSG00000249626	3	197634315	197635811	processed_pseudogene	5,21	0
ENSG00000232926	ENSG00000232926	22	19887289	19887970	processed_pseudogene	5,20	0
ENSG00000225988	LAMP5-AS1	20	9505180	9514998	antisense	5,20	0
ENSG00000267838	ENSG00000267838	19	54438665	54439544	lincRNA	5,20	1,40E-02
ENSG00000269050	ENSG00000269050	19	38870159	38873763	antisense	5,20	7,06E-02
ENSG00000277511	ENSG00000277511	17	32127595	32128454	lincRNA	5,20	0,2161218
ENSG00000260467	ENSG00000260467	16	57681124	57701730	antisense	5,20	0
ENSG00000258489	ENSG00000258489	15	95463592	95493770	lincRNA	5,20	0
ENSG00000259134	LINC00924	15	95433095	95507847	lincRNA	5,20	0
ENSG00000259659	ENSG00000259659	15	44557829	44559188	sense_intronic	5,20	1,40E-02
ENSG00000247774	PCED1B-AS1	12	47205898	47216456	processed_transcript	5,20	0
ENSG00000269514	ENSG00000269514	12	48198387	48202031	lincRNA	5,20	1,40E-02
ENSG00000233690	EBAG9P1	10	99697407	99697949	processed_pseudogene	5,20	1,40E-02
ENSG00000236114	ENSG00000236114	10	43523256	43525217	unprocessed_pseudogene	5,20	7,06E-02
ENSG00000282164	PEG13	8	140094894	140099137	sense_intronic	5,20	1,40E-02
ENSG00000272370	ENSG00000272370	5	65020038	65020551	lincRNA	5,20	1,40E-02
ENSG00000235908	RHOA-IT1	3	49365145	49367006	sense_intronic	5,20	0
ENSG00000251448	ENSG00000251448	3	126288867	126294024	lincRNA	5,20	0
ENSG00000175772	LINC01106	2	110375138	110384442	lincRNA	5,20	0
ENSG00000236841	ENSG00000236841	2	162159762	162173223	antisense	5,20	0,2161218
ENSG00000234678	ENSG00000234678	1	202000094	202010353	antisense	5,20	0
ENSG00000234998	ENSG00000234998	1	121087528	121116676	antisense	5,20	0
ENSG00000237415	NRBF2P3	1	110848077	110848941	processed_pseudogene	5,20	1,40E-02
ENSG00000237741	ENSG00000237741	X	148498113	148500615	antisense	5,20	0
ENSG00000268357	VN1R81P	19	21123817	21124188	unprocessed_pseudogene	5,18	0
ENSG00000225140	ENSG00000225140	10	628638	631255	antisense	5,18	0,1392851
ENSG00000250796	ENSG00000250796	3	128869624	128870026	processed_pseudogene	5,18	0
ENSG00000227193	ENSG00000227193	1	121118126	121146826	lincRNA	5,18	0
ENSG00000259985	ENSG00000259985	18	31685655	31686823	antisense	5,17	0
ENSG00000233966	UBE2SP1	17	15704232	15704900	processed_pseudogene	5,17	0
ENSG00000228843	ENSG00000228843	9	35756712	35757940	antisense	5,17	6,27E-03
ENSG00000272486	ENSG00000272486	8	124579631	124580648	antisense	5,17	3,30E-03
ENSG00000272848	ENSG00000272848	6	169426420	169427069	lincRNA	5,17	0,1836819
ENSG00000238039	ENSG00000238039	X	149511509	149526264	lincRNA	5,17	0
ENSG00000177406	ENSG00000177406	12	630891	663706	antisense	5,17	0
ENSG00000198711	SSBP3-AS1	1	54236440	54239063	antisense	5,14	0
ENSG00000259736	CRTC3-AS1	15	90620007	90717141	antisense	5,13	0
ENSG00000241923	RPL14P3	4	140366240	140366861	processed_pseudogene	5,13	0
ENSG00000185495	ENSG00000185495	1	223992743	224010612	transcribed_unprocessed_pseudogene	5,13	0

ENSG00000230487	PSMG3-AS1	7	1570073	1589626	lincRNA	5,12	0
ENSG00000248476	BACH1-IT1	21	29351634	29361894	sense_intronic	5,10	0
ENSG00000268739	ENSG00000268739	19	51014374	51014734	antisense	5,10	0,2161218
ENSG00000269054	ENSG00000269054	19	58362585	58366591	antisense	5,10	0
ENSG00000248858	ENSG00000248858	8	92699742	92879502	processed_transcript	5,10	7,06E-02
ENSG00000214988	RPL7AP26	4	82490823	82491635	processed_pseudogene	5,10	7,06E-02
ENSG00000249341	ENSG00000249341	4	53659208	53737156	sense_intronic	5,10	1,40E-02
ENSG00000269506	ENSG00000269506	4	54059597	54061210	antisense	5,10	0
ENSG00000213244	HIST2H3DP1	1	121118195	121118610	unprocessed_pseudogene	5,10	0
ENSG00000228549	ENSG00000228549	1	16870945	16874092	lincRNA	5,10	0
ENSG00000224204	PHEX-AS1	X	22162733	22172983	antisense	5,10	7,06E-02
ENSG00000251453	HAUS1P1	5	140581504	140582335	processed_pseudogene	5,10	0
ENSG00000272123	ENSG00000272123	5	53089016	53089468	antisense	5,09	0
ENSG00000225094	SETP20	4	109553243	109554094	processed_pseudogene	5,09	0,0358681
ENSG00000238198	ENSG00000238198	1	113011687	113073105	lincRNA	5,09	0
ENSG00000215417	MIR17HG	13	91347820	91354579	processed_transcript	5,09	0,3318209
ENSG00000226438	ENSG00000226438	1	39249838	39257649	antisense	5,06	0
ENSG00000260277	ENSG00000260277	16	22083256	22092231	antisense	5,06	0
ENSG00000278022	ENSG00000278022	15	98660210	98660668	sense_intronic	5,06	0,3318209
ENSG00000273142	ENSG00000273142	7	66902857	66906297	lincRNA	5,05	0
ENSG00000276390	ENSG00000276390	12	47699401	47699917	sense_intronic	5,05	0,2161218
ENSG00000273456	ENSG00000273456	2	202374932	202375604	lincRNA	5,05	0,3565078
ENSG00000268015	ENSG00000268015	19	52222225	52231294	antisense	5,04	0
ENSG00000197258	EIF4BP6	7	104667749	104669576	processed_pseudogene	5,04	0
ENSG00000227946	ENSG00000227946	2	206084605	206086564	lincRNA	5,03	3,30E-03
ENSG00000255121	ENSG00000255121	11	118994824	118998004	lincRNA	5,02	0
ENSG00000225225	ARL2BPP10	22	18102457	18103649	processed_pseudogene	5,00	1,40E-02
ENSG00000227413	ENSG00000227413	22	40433136	40433641	processed_pseudogene	5,00	0
ENSG00000272834	ENSG00000272834	22	40415003	40415445	antisense	5,00	0
ENSG00000227256	MIS18A-AS1	21	32277863	32280988	antisense	5,00	4,86E-02
ENSG00000234750	ENSG00000234750	19	12284337	12285185	processed_pseudogene	5,00	0
ENSG00000268362	ENSG00000268362	19	24033445	24066909	lincRNA	5,00	0
ENSG00000268568	ENSG00000268568	19	56672574	56673901	lincRNA	5,00	7,06E-02
ENSG00000269289	ENSG00000269289	19	23919081	23957930	antisense	5,00	7,06E-02
ENSG00000270947	ENSG00000270947	19	22455988	22456459	lincRNA	5,00	0
ENSG00000264885	ENSG00000264885	17	18667629	18669461	sense_intronic	5,00	0
ENSG00000234769	WASH4P	16	14381	18068	unprocessed_pseudogene	5,00	0
ENSG00000254634	SMG1P6	16	29425800	29447026	unprocessed_pseudogene	5,00	0
ENSG00000277010	ENSG00000277010	16	1223639	1224143	antisense	5,00	0,2161218
ENSG00000251161	ENSG00000251161	15	40906811	40910337	lincRNA	5,00	0
ENSG00000274403	ENSG00000274403	15	42726583	42727211	antisense	5,00	0
ENSG00000274667	ENSG00000274667	15	56729932	56730611	sense_intronic	5,00	0,0358681
ENSG00000260615	RPL23AP97	13	114346167	114346637	processed_pseudogene	5,00	0,1836819
ENSG00000228753	EIF4BP2	10	21028999	21030792	processed_pseudogene	5,00	0
ENSG00000273474	ENSG00000273474	10	13675646	13675990	antisense	5,00	1,40E-02
ENSG00000215374	FAM66B	8	7301611	7355354	lincRNA	5,00	0
ENSG00000253829	ENSG00000253829	8	38844866	38846439	antisense	5,00	0
ENSG00000272502	ENSG00000272502	8	120812219	120813359	antisense	5,00	1,40E-02
ENSG00000272361	ENSG00000272361	7	16593626	16594224	lincRNA	5,00	0,2161218
ENSG00000218996	ENSG00000218996	6	150934968	150935566	processed_pseudogene	5,00	0
ENSG00000251634	ENSG00000251634	5	71102898	71128753	unprocessed_pseudogene	5,00	0
ENSG00000214184	GCC2-AS1	2	108507515	108534196	antisense	5,00	0
ENSG00000265531	FCGR1C	1	143874793	143883575	unprocessed_pseudogene	5,00	7,06E-02
ENSG00000273338	ENSG00000273338	1	78004346	78004554	antisense	5,00	0
ENSG00000240695	ENSG00000240695	3	136736500	136737229	processed_pseudogene	4,99	0
ENSG00000261938	ENSG00000261938	16	3581181	3583266	lincRNA	4,99	0

ENSG00000214322	CBX1P2	X	17099782	17100239	processed_pseudogene	4,98	0
ENSG00000249180	ENSG00000249180	5	96741079	96742698	antisense	4,96	0
ENSG00000267260	ENSG00000267260	19	36773153	36777078	lincRNA	4,96	0
ENSG00000271754	ENSG00000271754	6	43519180	43519724	antisense	4,96	0
ENSG00000228217	PNRC2P1	1	117778087	117778506	processed_pseudogene	4,95	0
ENSG00000245573	BDNF-AS	11	27506838	27698174	antisense	4,94	9,18E-02
ENSG00000270605	ENSG00000270605	1	28239509	28241453	antisense	4,94	0
ENSG00000239407	ENSG00000239407	X	102884414	102906158	lincRNA	4,93	0
ENSG00000217835	ENSG00000217835	X	65956289	65957196	processed_pseudogene	4,93	0
ENSG00000170629	DPY19L2P2	7	103175133	103280410	transcribed_unprocessed_pseudogene	4,93	0
ENSG00000213667	PGGT1BP1	10	50726947	50727969	processed_pseudogene	4,92	0
ENSG00000254064	ENSG00000254064	8	22254576	22275162	antisense	4,92	0
ENSG00000259275	ENSG00000259275	15	96354237	96403803	lincRNA	4,92	4,86E-02
ENSG00000223977	ENSG00000223977	2	83218890	83219105	processed_pseudogene	4,92	0
ENSG00000259388	ENSG00000259388	15	49794246	49796097	processed_pseudogene	4,91	0
ENSG00000217325	PRELID1P1	6	126643488	126644390	transcribed_processed_pseudogene	4,91	0
ENSG00000261557	EEF1A1P38	16	27133483	27135179	processed_pseudogene	4,91	2,95E-02
ENSG00000256001	ENSG00000256001	12	127145072	127146532	lincRNA	4,91	0
ENSG00000251455	ENSG00000251455	4	151674483	151677893	antisense	4,91	0,0358681
ENSG00000254873	ENSG00000254873	11	118397095	118401895	antisense	4,91	0
ENSG00000268560	ENSG00000268560	19	20611559	20615389	lincRNA	4,90	0
ENSG00000268654	MIMT1	19	56840902	56848556	lincRNA	4,90	0
ENSG00000261731	ENSG00000261731	16	31709113	31711984	antisense	4,90	0
ENSG00000263307	ENSG00000263307	16	11851649	11895611	antisense	4,90	0
ENSG00000188388	GOLGA6L3	15	85240472	85247170	unprocessed_pseudogene	4,90	0
ENSG00000275580	ENSG00000275580	15	49343608	49344254	lincRNA	4,90	7,06E-02
ENSG00000258875	ENSG00000258875	14	91242759	91252211	processed_transcript	4,90	7,06E-02
ENSG00000259868	ENSG00000259868	14	55896547	55962970	processed_transcript	4,90	7,06E-02
ENSG00000257242	LINC01619	12	91984976	92142914	processed_transcript	4,90	7,06E-02
ENSG00000255029	ENSG00000255029	11	29519076	29552639	lincRNA	4,90	7,06E-02
ENSG00000253641	ENSG00000253641	8	10474565	10481974	lincRNA	4,90	0
ENSG00000227038	GTF2IP7	7	76090431	76108779	transcribed_unprocessed_pseudogene	4,90	0
ENSG00000229380	ENSG00000229380	7	561958	565619	antisense	4,90	0,2161218
ENSG00000233559	ENSG00000233559	7	130853720	130928649	lincRNA	4,90	0
ENSG00000271664	ENSG00000271664	7	149275619	149277992	unprocessed_pseudogene	4,90	0
ENSG00000249960	ENSG00000249960	4	82571137	82571814	processed_pseudogene	4,90	0
ENSG00000223505	ENSG00000223505	1	203835585	203835912	processed_pseudogene	4,90	7,06E-02
ENSG00000238199	UBE2V2P3	1	11278616	11279351	processed_pseudogene	4,90	0,2161218
ENSG00000227060	LINC00629	X	134549973	134559923	lincRNA	4,90	0
ENSG00000230397	SPTLC1P1	10	31360955	31361215	transcribed_processed_pseudogene	4,89	0
ENSG00000235499	ENSG00000235499	2	73985132	73986343	lincRNA	4,88	0
ENSG00000215023	ENSG00000215023	2	241800916	241801907	antisense	4,88	0,1836819
ENSG00000267577	ENSG00000267577	19	55158939	55177540	antisense	4,88	0,4036702
ENSG00000270021	ENSG00000270021	5	135399280	135401296	antisense	4,88	1,40E-02
ENSG00000226121	ENSG00000226121	2	161500885	161507705	processed_pseudogene	4,86	0
ENSG00000214584	PGGT1BP2	10	79892148	79893175	processed_pseudogene	4,84	0
ENSG00000257286	ENSG00000257286	12	113185624	113192161	antisense	4,84	9,18E-02
ENSG00000124915	ENSG00000124915	11	61746493	61757655	lincRNA	4,84	0,1836819
ENSG00000273759	ENSG00000273759	20	62928621	62929297	lincRNA	4,83	6,27E-03
ENSG00000265188	ENSG00000265188	18	5240255	5241167	processed_pseudogene	4,83	0
ENSG00000274630	ENSG00000274630	17	41867581	41867736	antisense	4,83	0
ENSG00000267185	PTP4A2P1	17	42533532	42534009	processed_pseudogene	4,82	0
ENSG00000218512	SPTLC1P2	6	23856698	23856818	processed_pseudogene	4,82	0
ENSG00000236397	DDX11L2	2	113599036	113601261	unprocessed_pseudogene	4,81	1,40E-02

ENSG00000270728	ENSG00000270728	1	19297080	19297903	processed_pseudogene	4,81	0
ENSG00000224886	ENSG00000224886	10	79684494	79684983	transcribed_processed_pseudogene	4,80	0
ENSG00000234222	ENSG00000234222	1	145926590	145959179	antisense	4,80	0
ENSG00000227370	ENSG00000227370	22	42132543	42132998	processed_pseudogene	4,80	0,1392851
ENSG00000228817	BACH1-IT2	21	29370497	29373709	lincRNA	4,80	1,40E-02
ENSG00000267682	ENSG00000267682	19	37337236	37337743	sense_intronic	4,80	0
ENSG00000269191	ENSG00000269191	19	18532908	18536188	antisense	4,80	7,06E-02
ENSG00000269482	ENSG00000269482	16	90631	91102	antisense	4,80	0
ENSG00000259644	ENSG00000259644	15	29728186	29729531	sense_intronic	4,80	0
ENSG00000260388	LINC00562	13	47930153	47932622	lincRNA	4,80	7,06E-02
ENSG00000275963	ENSG00000275963	12	12648939	12649713	lincRNA	4,80	7,06E-02
ENSG00000276148	ENSG00000276148	12	32725248	32725660	antisense	4,80	7,06E-02
ENSG00000224812	TMEM72-AS1	10	44811024	44959689	antisense	4,80	7,06E-02
ENSG00000237631	ENSG00000237631	9	95918136	95918704	processed_pseudogene	4,80	0,2161218
ENSG00000196114	ENSG00000196114	6	34576258	34576656	processed_pseudogene	4,80	0,2161218
ENSG00000260581	ENSG00000260581	5	151652275	151655449	antisense	4,80	7,06E-02
ENSG00000271737	ENSG00000271737	5	134005147	134005562	lincRNA	4,80	0
ENSG00000251229	ENSG00000251229	4	2505081	2506279	processed_pseudogene	4,80	1,40E-02
ENSG00000240373	SEC62-AS1	3	169978536	169985715	antisense	4,80	1,40E-02
ENSG00000243795	ENSG00000243795	3	113142350	113167819	lincRNA	4,80	7,06E-02
ENSG00000241114	ENSG00000241114	2	54079974	54080280	processed_pseudogene	4,80	0
ENSG00000258545	RHOXF1-AS1	X	120036236	120146854	antisense	4,80	1,40E-02
ENSG00000260081	ENSG00000260081	X	153599340	153604353	antisense	4,80	7,06E-02
ENSG00000233262	ENSG00000233262	9	84278219	84290131	antisense	4,80	6,27E-03
ENSG00000272686	ENSG00000272686	7	123749068	123751166	antisense	4,79	0
ENSG00000269800	PLEKHA3P1	19	41521043	41521989	processed_pseudogene	4,79	0
ENSG00000268129	ENSG00000268129	3	143000907	143001467	lincRNA	4,78	2,21E-02
ENSG00000220695	ENSG00000220695	6	100530276	100531052	processed_pseudogene	4,77	0
ENSG00000231245	C1DP1	10	32511336	32511737	processed_pseudogene	4,77	0
ENSG00000277324	ENSG00000277324	18	54268346	54270028	antisense	4,76	0
ENSG00000249465	RBMXP4	4	109346326	109347459	processed_pseudogene	4,76	0
ENSG00000214262	ANKRD36BP1	1	168245565	168247343	transcribed_processed_pseudogene	4,76	0
ENSG00000276291	FRG1HP	9	40992249	41218410	transcribed_unprocessed_pseudogene	4,76	0
ENSG00000260063	ENSG00000260063	1	26692132	26694131	antisense	4,76	6,27E-03
ENSG00000244556	ODCP	7	129028889	129030527	processed_pseudogene	4,75	0,1836819
ENSG00000230561	LINC01183	5	127703390	127941634	lincRNA	4,75	0
ENSG00000205578	POM121B	7	73293497	73301161	unprocessed_pseudogene	4,74	0
ENSG00000255471	ENSG00000255471	11	86892214	86925037	antisense	4,74	0
ENSG00000244627	ENSG00000244627	22	38335762	38398929	transcribed_unprocessed_pseudogene	4,73	4,86E-02
ENSG00000215244	ENSG00000215244	10	6277687	6335982	lincRNA	4,73	0
ENSG00000214012	KRT18P38	6	19612755	19614036	processed_pseudogene	4,73	0
ENSG00000272368	ENSG00000272368	12	50112197	50165618	antisense	4,71	6,27E-03
ENSG00000203499	FAM83H-AS1	8	143734140	143746337	lincRNA	4,71	0
ENSG00000275910	ENSG00000275910	16	15015828	15016390	antisense	4,71	6,27E-03
ENSG00000235978	ENSG00000235978	3	4814294	4887293	antisense	4,70	0
ENSG00000273428	ENSG00000273428	22	30435544	30436247	lincRNA	4,70	0,2161218
ENSG00000227456	LINC00310	21	34157724	34190244	lincRNA	4,70	7,06E-02
ENSG00000272659	ENSG00000272659	21	33309491	33310181	lincRNA	4,70	0,2161218
ENSG00000267749	ENSG00000267749	19	2641838	2643853	sense_intronic	4,70	1,40E-02
ENSG00000267800	ENSG00000267800	18	47137018	47137290	processed_pseudogene	4,70	0
ENSG00000262777	ENSG00000262777	17	1424473	1426484	antisense	4,70	0
ENSG00000263107	ENSG00000263107	17	19582116	19582275	processed_pseudogene	4,70	0
ENSG00000267365	KCNJ2-AS1	17	70166961	70169402	antisense	4,70	0
ENSG00000261613	ENSG00000261613	16	2554060	2556060	antisense	4,70	7,06E-02
ENSG00000259658	ENSG00000259658	15	101737099	101745710	lincRNA	4,70	0
ENSG00000277144	ENSG00000277144	15	59115547	59116089	lincRNA	4,70	0,2161218

ENSG00000260711	ENSG00000260711	14	91752856	91759798	sense_intronic	4,70	7,06E-02
ENSG00000256973	ENSG00000256973	12	22460519	22463914	lincRNA	4,70	0
ENSG00000258181	ENSG00000258181	12	47248124	47257539	lincRNA	4,70	0
ENSG00000276505	ENSG00000276505	11	117297005	117297328	sense_intronic	4,70	7,06E-02
ENSG00000226562	CYP4F26P	9	33580695	33605293	lincRNA	4,70	0
ENSG00000234709	UPF3AP3	9	99998301	99999069	transcribed_processed_pseudogene	4,70	0
ENSG00000170356	OR2A20P	7	144250045	144252957	transcribed_unprocessed_pseudogene	4,70	1,40E-02
ENSG00000270933	ENSG00000270933	7	25948657	25949403	lincRNA	4,70	0,2161218
ENSG00000271537	ENSG00000271537	7	139198557	139199142	processed_pseudogene	4,70	1,40E-02
ENSG00000272402	ENSG00000272402	6	24706747	24707151	antisense	4,70	0
ENSG00000249942	ENSG00000249942	4	74552584	74589481	antisense	4,70	7,06E-02
ENSG00000241439	ENSG00000241439	3	125958556	125958817	processed_pseudogene	4,70	7,06E-02
ENSG00000203684	IBA57-AS1	1	228164086	228165512	lincRNA	4,70	7,06E-02
ENSG00000229635	ENSG00000229635	1	93384487	93384998	processed_pseudogene	4,70	7,06E-02
ENSG00000233337	UBE2FP3	1	111437514	111438037	processed_pseudogene	4,70	1,40E-02
ENSG00000238061	ENSG00000238061	1	185280844	185281816	processed_pseudogene	4,70	7,06E-02
ENSG00000272068	ENSG00000272068	1	156637783	156641004	lincRNA	4,70	0
ENSG00000282386	ENSG00000282386	1	153964361	153965070	antisense	4,70	0
ENSG00000229816	DDX50P1	2	32201600	32203999	processed_pseudogene	4,69	3,30E-03
ENSG00000258655	ARHGAP5-AS1	14	32074946	32076793	antisense	4,69	6,27E-03
ENSG00000262700	ENSG00000262700	16	25093387	25095255	processed_pseudogene	4,69	3,30E-03
ENSG00000248124	RRN3P1	16	21796106	21820410	transcribed_unprocessed_pseudogene	4,68	0
ENSG00000253223	ENSG00000253223	8	66984135	66986187	processed_pseudogene	4,68	0,4332376
ENSG00000182586	LINC00334	21	45234340	45258730	lincRNA	4,67	0,3565078
ENSG00000278916	CEP83-AS1	12	94460003	94462484	lincRNA	4,67	6,27E-03
ENSG00000244945	ENSG00000244945	5	179595904	179603741	antisense	4,67	0
ENSG00000230939	ENSG00000230939	6	8784178	8785445	lincRNA	4,65	1,95E-02
ENSG00000214293	APTR	7	77657660	77696265	lincRNA	4,64	0
ENSG00000230330	HMGN2P3	16	26032539	26032811	processed_pseudogene	4,64	0
ENSG00000270061	ENSG00000270061	12	123969990	123970344	sense_intronic	4,64	6,27E-03
ENSG00000230064	ENSG00000230064	9	133338990	133339465	processed_pseudogene	4,64	0
ENSG00000230257	NFE4	7	102973522	102988856	antisense	4,64	0
ENSG00000226810	ENSG00000226810	5	54857272	54858491	processed_pseudogene	4,64	0
ENSG00000260179	ENSG00000260179	1	1249777	1251334	lincRNA	4,64	0,1392851
ENSG00000214826	DDX12P	12	9418673	9448229	unprocessed_pseudogene	4,62	0
ENSG00000229047	ENSG00000229047	21	14961309	14964233	antisense	4,61	6,27E-03
ENSG00000236852	ENSG00000236852	X	62779250	62780326	processed_pseudogene	4,61	0,0358681
ENSG00000278383	ENSG00000278383	20	25680781	25681246	antisense	4,60	0
ENSG00000268756	ENSG00000268756	19	38820166	38823223	antisense	4,60	0
ENSG00000268938	ENSG00000268938	19	18531613	18532632	antisense	4,60	7,06E-02
ENSG00000266401	ENSG00000266401	18	3653410	3656282	antisense	4,60	0
ENSG00000141028	CDRT15P1	17	14024514	14025488	unprocessed_pseudogene	4,60	0
ENSG00000231595	ENSG00000231595	17	14210488	14217922	lincRNA	4,60	0
ENSG00000234494	SP2-AS1	17	47897330	47941410	antisense	4,60	0
ENSG00000262050	ENSG00000262050	17	2712309	2712833	antisense	4,60	0
ENSG00000275056	ENSG00000275056	16	4839244	4840334	sense_intronic	4,60	1,40E-02
ENSG00000254398	ENSG00000254398	15	28679405	28682252	unprocessed_pseudogene	4,60	0
ENSG00000270704	ENSG00000270704	15	24984864	24985173	sense_intronic	4,60	1,40E-02
ENSG00000258233	ARHGAP42P5	14	18623852	18626787	processed_pseudogene	4,60	0
ENSG00000258876	ENSG00000258876	14	75970924	75971587	lincRNA	4,60	0
ENSG00000213235	EEF1A1P16	12	16990670	16992083	processed_pseudogene	4,60	0
ENSG00000260470	ENSG00000260470	12	54145069	54147225	lincRNA	4,60	0,2161218
ENSG00000255306	ENSG00000255306	11	68024809	68030461	antisense	4,60	1,40E-02
ENSG00000270510	ENSG00000270510	11	87323709	87324359	processed_pseudogene	4,60	0
ENSG00000270903	HNRNPA3P9	11	32591793	32592771	processed_pseudogene	4,60	0

ENSG00000178440	LINC00843	10	49972763	49982053	lincRNA	4,60	0
ENSG00000212743	ENSG00000212743	10	6350316	6352762	lincRNA	4,60	1,40E-02
ENSG00000229261	ENSG00000229261	10	69215333	69232490	antisense	4,60	7,06E-02
ENSG00000233081	ENSG00000233081	9	91426238	91427144	lincRNA	4,60	0
ENSG00000248896	ENSG00000248896	8	10729314	10771392	antisense	4,60	0
ENSG00000224384	ENSG00000224384	6	96785137	96795821	antisense	4,60	0
ENSG00000228577	ENSG00000228577	2	206606497	206609812	antisense	4,60	7,06E-02
ENSG00000235321	ENSG00000235321	2	169100743	169101210	antisense	4,60	0
ENSG00000236209	ENSG00000236209	2	74919555	74924846	lincRNA	4,60	7,06E-02
ENSG00000273196	ENSG00000273196	2	85387074	85387146	antisense	4,60	0
ENSG00000224939	LINC00184	1	234629311	234634780	lincRNA	4,60	1,40E-02
ENSG00000227533	SLC2A1-AS1	1	42959049	42983358	lincRNA	4,60	0,2161218
ENSG00000230679	ENO1-AS1	1	8878835	8879894	antisense	4,60	0
ENSG00000232188	ENSG00000232188	1	160931739	160934380	lincRNA	4,60	0,2161218
ENSG00000223440	ENSG00000223440	9	32633454	32648685	antisense	4,59	0
ENSG00000255114	ENSG00000255114	11	119044188	119045493	antisense	4,59	4,86E-02
ENSG00000233178	ENSG00000233178	9	68393881	68406500	antisense	4,58	0
ENSG00000231310	TBL1XR1-AS1	3	177037405	177047923	antisense	4,58	0
ENSG00000273489	ENSG00000273489	7	131493964	131497694	antisense	4,58	0
ENSG00000258749	ENSG00000258749	14	99512501	99513576	antisense	4,57	0
ENSG00000272434	ENSG00000272434	3	49029316	49029706	antisense	4,56	0
ENSG00000227945	ENSG00000227945	6	128027886	128085248	antisense	4,56	0
ENSG00000255717	SNHG1	11	62851988	62855914	processed_transcript	4,56	0
ENSG00000235919	ASH1L-AS1	1	155562042	155563944	antisense	4,56	3,30E-03
ENSG00000260136	ENSG00000260136	16	23452758	23457606	lincRNA	4,56	0
ENSG00000275454	ENSG00000275454	15	75639760	75640976	antisense	4,55	0
ENSG00000268442	ENSG00000268442	19	24162370	24163425	processed_pseudogene	4,55	0,1836819
ENSG00000227725	GCOM2	4	68038544	68039647	processed_pseudogene	4,54	0
ENSG00000250132	ENSG00000250132	12	974133	991190	antisense	4,54	0
ENSG00000228288	PCAT6	1	202810954	202812156	antisense	4,54	4,86E-02
ENSG00000267277	ENSG00000267277	19	11368123	11374935	antisense	4,54	0
ENSG00000233381	AK4P3	12	31615771	31616439	processed_pseudogene	4,54	0,0358681
ENSG00000256248	ENSG00000256248	12	66891876	67069162	lincRNA	4,54	1,40E-02
ENSG00000217027	TPT1P4	6	144200447	144200965	processed_pseudogene	4,52	0,2161218
ENSG00000236778	INTS6-AS1	13	51452367	51552364	antisense	4,52	0
ENSG00000237821	ENSG00000237821	7	133169416	133170514	processed_pseudogene	4,51	0
ENSG00000227695	DNMBP-AS1	10	99927010	99958381	antisense	4,51	0
ENSG00000236810	TCEB3-AS1	1	23760382	23778287	antisense	4,51	0
ENSG00000268628	ENSG00000268628	20	19756390	19758037	lincRNA	4,50	0
ENSG00000205786	LINC01531	19	35399511	35419385	lincRNA	4,50	0,2161218
ENSG00000267424	ENSG00000267424	19	12825711	12832983	antisense	4,50	0,2161218
ENSG00000267551	ENSG00000267551	19	3141576	3155175	antisense	4,50	1,40E-02
ENSG00000269553	ENSG00000269553	19	35330843	35331920	sense_intronic	4,50	0
ENSG00000267313	ENSG00000267313	18	41465783	41632185	lincRNA	4,50	7,06E-02
ENSG00000214546	ENSG00000214546	17	39619613	39622513	lincRNA	4,50	7,06E-02
ENSG00000265845	ENSG00000265845	17	28926275	28944749	antisense	4,50	0
ENSG00000276250	ENSG00000276250	17	30803654	30804077	antisense	4,50	0
ENSG00000189149	CRYM-AS1	16	21300849	21318591	lincRNA	4,50	0
ENSG00000251393	ENSG00000251393	14	73462423	73477175	processed_transcript	4,50	0
ENSG00000274987	ENSG00000274987	12	25210652	25211233	antisense	4,50	0
ENSG00000224919	ENSG00000224919	10	47496212	47502195	antisense	4,50	0
ENSG00000242600	MBL1P	10	79904898	79950336	unitary_pseudogene	4,50	0
ENSG00000249456	ENSG00000249456	10	124917143	124942881	sense_overlapping	4,50	7,06E-02
ENSG00000271816	ENSG00000271816	10	73699151	73730487	processed_transcript	4,50	1,40E-02
ENSG00000182487	NCF1B	7	73220624	73235945	transcribed_unprocessed_pseudogene	4,50	7,06E-02
ENSG00000239911	PRKAG2-AS1	7	151877042	151879223	antisense	4,50	0
ENSG00000241269	ENSG00000241269	7	5419827	5420767	antisense	4,50	7,06E-02
ENSG00000230433	ENSG00000230433	6	524171	525581	antisense	4,50	0,2161218
ENSG00000229666	MAST4-AS1	5	67001383	67003953	antisense	4,50	1,40E-02
ENSG00000248489	ENSG00000248489	5	98929171	98995013	lincRNA	4,50	0,2161218

ENSG00000250848	ENSG00000250848	5	288833	290321	processed_pseudogene	4,50	0
ENSG00000180610	ZBTB12P1	4	39770081	39771371	processed_pseudogene	4,50	0
ENSG00000189196	LINC00994	3	64078361	64087363	lincRNA	4,50	7,06E-02
ENSG00000213450	VDAC1P7	3	77316752	77317598	processed_pseudogene	4,50	0
ENSG00000239503	MARK2P8	3	128748538	128749489	processed_pseudogene	4,50	0
ENSG00000239994	ENSG00000239994	3	119744139	119750350	antisense	4,50	0
ENSG00000269894	ENSG00000269894	3	9935706	9936258	sense_intronic	4,50	7,06E-02
ENSG00000270059	ENSG00000270059	3	65359268	65359717	sense_intronic	4,50	0
ENSG00000235369	RPL36AP15	2	47797826	47798091	processed_pseudogene	4,50	7,06E-02
ENSG00000279220	GPR1-AS	2	206203376	206266243	processed_transcript	4,50	0
ENSG00000277007	ENSG00000277007	1	219270774	219273387	lincRNA	4,50	1,40E-02
ENSG00000226107	ENSG00000226107	X	134522702	134522990	processed_pseudogene	4,50	1,40E-02
ENSG00000258114	ENSG00000258114	10	117425194	117490419	lincRNA	4,49	0
ENSG00000233369	GTF2IP4	7	73154938	73207283	transcribed_processed_pseudogene	4,48	0
ENSG00000240356	RPL23AP7	2	113610502	113627090	transcribed_processed_pseudogene	4,46	0
ENSG00000233903	ENSG00000233903	22	42276355	42277052	lincRNA	4,45	0
ENSG00000230358	SPDYE21P	7	67279778	67286230	unprocessed_pseudogene	4,45	0
ENSG00000266865	ENSG00000266865	17	31008497	31093127	transcribed_unprocessed_pseudogene	4,44	0
ENSG00000268658	LINC00664	19	21483374	21503238	lincRNA	4,43	0
ENSG00000254719	ENSG00000254719	11	10272052	10272259	processed_pseudogene	4,42	0
ENSG00000224861	YBX1P1	14	66012830	66013789	processed_pseudogene	4,42	0
ENSG00000256512	ENSG00000256512	12	27798641	27800708	antisense	4,41	0
ENSG00000217702	ENSG00000217702	2	74130583	74135395	lincRNA	4,41	0,2746192
ENSG00000234983	ENSG00000234983	7	42918741	42920084	processed_pseudogene	4,41	7,06E-02
ENSG00000214552	COPS8P2	3	68145004	68145630	processed_pseudogene	4,41	3,30E-03
ENSG00000204637	ENSG00000204637	2	100509277	100509612	processed_pseudogene	4,40	0
ENSG00000229728	ENSG00000229728	20	1361622	1362585	antisense	4,40	1,40E-02
ENSG00000237595	ENSG00000237595	20	50162765	50166102	lincRNA	4,40	0,2161218
ENSG00000268518	ENSG00000268518	19	50486810	50487638	lincRNA	4,40	7,06E-02
ENSG00000269397	ENSG00000269397	19	23927788	23929287	sense_intronic	4,40	0,2161218
ENSG00000264825	ENSG00000264825	18	22213778	22228029	lincRNA	4,40	0,2161218
ENSG00000264843	ENSG00000264843	18	10704297	10709599	antisense	4,40	0
ENSG00000270469	ENSG00000270469	18	14053951	14054761	processed_pseudogene	4,40	0,2161218
ENSG00000213373	LINC00671	17	42874670	42898704	lincRNA	4,40	0,2161218
ENSG00000265060	PPY2P	17	28247444	28248289	transcribed_unprocessed_pseudogene	4,40	0
ENSG00000267546	ENSG00000267546	17	76545668	76557683	antisense	4,40	7,06E-02
ENSG00000270993	ENSG00000270993	17	75060491	75060620	processed_pseudogene	4,40	7,06E-02
ENSG00000261527	ENSG00000261527	16	69443240	69444290	processed_pseudogene	4,40	0,2161218
ENSG00000242085	RPS20P33	14	105374618	105374971	processed_pseudogene	4,40	0
ENSG00000256894	ENSG00000256894	12	26125155	26126617	antisense	4,40	0
ENSG00000276261	ENSG00000276261	12	27700066	27700574	antisense	4,40	7,06E-02
ENSG00000271584	ENSG00000271584	11	111091932	111097357	lincRNA	4,40	0
ENSG00000233665	ENSG00000233665	10	48976554	48993046	processed_transcript	4,40	0
ENSG00000270427	NRBF2P5	10	5776071	5776858	processed_pseudogene	4,40	1,40E-02
ENSG00000270767	ENSG00000270767	10	44488335	44488445	processed_pseudogene	4,40	1,40E-02
ENSG00000272572	ENSG00000272572	10	100911103	100912739	antisense	4,40	1,40E-02
ENSG00000272914	ENSG00000272914	10	30831828	30833387	sense_intronic	4,40	1,40E-02
ENSG00000230225	MTND5P14	9	5109496	5110762	unprocessed_pseudogene	4,40	0
ENSG00000253258	ENSG00000253258	8	123181638	123182788	antisense	4,40	7,06E-02
ENSG00000272010	ENSG00000272010	8	65591850	65592472	lincRNA	4,40	7,06E-02
ENSG00000231170	ENSG00000231170	7	95596682	95613719	antisense	4,40	0
ENSG00000233025	CRYZP1	7	103088664	103089610	processed_pseudogene	4,40	0
ENSG00000261019	ENSG00000261019	7	42901272	42902639	lincRNA	4,40	0
ENSG00000216809	ENSG00000216809	6	118452469	118454992	processed_pseudogene	4,40	0
ENSG00000250056	LINC01018	5	6582136	6588499	lincRNA	4,40	0

ENSG00000253295	ENSG00000253295	5	172758226	172762556	antisense	4,40	7,06E-02
ENSG00000261360	ENSG00000261360	5	14661808	14664604	antisense	4,40	0,2161218
ENSG00000279727	ENSG00000279727	3	36819276	36822498	lincRNA	4,40	1,40E-02
ENSG00000223382	ENSG00000223382	1	30824228	30834431	lincRNA	4,40	7,06E-02
ENSG00000230699	ENSG00000230699	1	911435	914948	lincRNA	4,40	7,06E-02
ENSG00000233396	ENSG00000233396	1	146052566	146061948	lincRNA	4,40	0
ENSG00000279049	ENSG00000279049	1	54099968	54100224	pseudogene	4,40	0
ENSG00000271360	ENSG00000271360	10	11611305	11612227	antisense	4,38	0
ENSG00000250794	ALG1L12P	8	12421032	12425000	unprocessed_pseudogene	4,38	0
ENSG00000225434	LINC01504	9	72305430	72343210	lincRNA	4,38	3,30E-03
ENSG00000255234	ENSG00000255234	11	83286128	83423516	antisense	4,38	0
ENSG00000248873	SERBP1P6	5	42465400	42468868	processed_pseudogene	4,38	0
ENSG00000214249	CTAGE11P	13	75237944	75240296	processed_pseudogene	4,37	0
ENSG00000224953	SRIP3	X	6718962	6719180	processed_pseudogene	4,37	4,86E-02
ENSG00000256283	METTL8P1	12	15255213	15255823	processed_pseudogene	4,37	0
ENSG00000273138	ENSG00000273138	7	95416108	95416462	antisense	4,37	0
ENSG00000255507	ENSG00000255507	11	75803431	75815406	antisense	4,36	0
ENSG00000231028	LINC00271	6	135497801	135716055	lincRNA	4,36	0
ENSG00000215873	FEN1P1	1	91328369	91329513	processed_pseudogene	4,36	3,30E-03
ENSG00000239335	LLPH-AS1	12	66130751	66134449	antisense	4,36	0
ENSG00000266677	ENSG00000266677	17	18172625	18184753	antisense	4,35	0
ENSG00000196810	CTBP1-AS2	4	1249300	1288291	antisense	4,35	0
ENSG00000269235	ZNF350-AS1	19	51949134	51981367	antisense	4,33	6,27E-03
ENSG00000275560	ENSG00000275560	12	12718973	12719521	sense_intronic	4,33	0
ENSG00000259709	ENSG00000259709	15	52124561	52140246	antisense	4,33	0
ENSG00000228797	FAM207BP	13	18697003	18697507	processed_pseudogene	4,33	0
ENSG00000267575	ENSG00000267575	19	27793463	27918863	lincRNA	4,33	0
ENSG00000269583	ENSG00000269583	19	43574638	43575618	antisense	4,32	0
ENSG00000231770	TMEM44-AS1	3	194584011	194590260	antisense	4,32	0
ENSG00000269939	ENSG00000269939	11	83185521	83187036	lincRNA	4,32	7,06E-02
ENSG00000259834	ENSG00000259834	1	110653560	110657040	lincRNA	4,32	0
ENSG00000215302	ENSG00000215302	15	30470779	30507623	transcribed_unprocessed_pseudogene	4,31	0
ENSG00000236662	ENSG00000236662	10	100980507	100985614	antisense	4,31	0
ENSG00000242748	RPL23AP81	20	41196691	41197157	transcribed_processed_pseudogene	4,30	7,06E-02
ENSG00000263797	ENSG00000263797	18	6873399	6875024	antisense	4,30	0
ENSG00000265791	ENSG00000265791	17	30781493	30782221	sense_intronic	4,30	7,06E-02
ENSG00000237718	ENSG00000237718	16	67614381	67616146	antisense	4,30	0
ENSG00000272808	ENSG00000272808	15	100849831	100865457	processed_transcript	4,30	7,06E-02
ENSG00000232587	EEF1A1P3	13	28214513	28215897	processed_pseudogene	4,30	0,2161218
ENSG00000232977	LINC00327	13	23466571	23487464	lincRNA	4,30	0
ENSG00000226472	ENSG00000226472	12	30978308	31006010	antisense	4,30	7,06E-02
ENSG00000256897	ENSG00000256897	11	47220218	47221751	antisense	4,30	1,40E-02
ENSG00000226393	IFNA20P	9	21321300	21321827	unprocessed_pseudogene	4,30	0
ENSG00000239392	ENSG00000239392	9	34133157	34134696	processed_pseudogene	4,30	0
ENSG00000213542	ENSG00000213542	7	77115399	77116192	processed_pseudogene	4,30	0
ENSG00000260418	ENSG00000260418	6	136335714	136336087	lincRNA	4,30	7,06E-02
ENSG00000241907	RPS20P4	5	150021567	150021921	processed_pseudogene	4,30	0
ENSG00000250337	LINC01021	5	27472292	27496401	lincRNA	4,30	0
ENSG00000251131	ENSG00000251131	5	43018429	43024247	antisense	4,30	0
ENSG00000269921	ENSG00000269921	4	56387625	56388153	lincRNA	4,30	0,2161218
ENSG00000213943	KRT18P17	3	12787393	12788671	processed_pseudogene	4,30	0
ENSG00000242992	FTH1P4	3	128764466	128765018	processed_pseudogene	4,30	7,06E-02
ENSG00000228431	ARL5AP3	1	68049360	68049745	processed_pseudogene	4,30	0
ENSG00000273026	ENSG00000273026	1	153966516	153966930	antisense	4,30	7,06E-02
ENSG00000214207	KRT18P10	X	5573145	5574431	processed_pseudogene	4,30	0
ENSG00000233250	ENSG00000233250	X	53432722	53433032	antisense	4,30	7,06E-02
ENSG00000250995	ENSG00000250995	X	118769796	118770857	processed_pseudogene	4,30	0

ENSG00000214199	EEF1A1P12	2	106697331	106698676	processed_pseudogene	4,30	7,06E-02
ENSG00000277782	ENSG00000277782	15	80999593	80999981	lincRNA	4,29	0
ENSG00000270966	ENSG00000270966	8	8933721	8934006	processed_pseudogene	4,29	3,30E-03
ENSG00000203288	ENSG00000203288	1	151790804	151794402	antisense	4,29	4,86E-02
ENSG00000183666	GUSBP1	5	21341833	21589372	transcribed_unprocessed_pseudogene	4,28	0
ENSG00000185065	ENSG00000185065	22	19447893	19450105	antisense	4,28	1,40E-02
ENSG00000266907	ENSG00000266907	19	56319790	56322310	processed_pseudogene	4,28	0
ENSG00000249889	ALG1L11P	8	12178697	12182719	unprocessed_pseudogene	4,27	3,30E-03
ENSG00000224331	ENSG00000224331	2	164687287	164687596	processed_pseudogene	4,27	0,1836819
ENSG00000260329	ENSG00000260329	12	106954029	106955497	antisense	4,27	0
ENSG00000272418	ENSG00000272418	15	78280950	78282190	sense_intronic	4,26	6,27E-03
ENSG00000257038	ENSG00000257038	11	73307235	73309361	antisense	4,26	0
ENSG00000223374	ENSG00000223374	2	241351340	241353104	antisense	4,25	0
ENSG00000214380	ENSG00000214380	3	109915976	109916940	processed_pseudogene	4,25	0,1836819
ENSG00000230046	BIRC6-AS1	2	32377631	32379599	antisense	4,24	0
ENSG00000277914	DDX6P2	13	86275493	86277262	processed_pseudogene	4,24	3,30E-03
ENSG00000271806	ENSG00000271806	1	2141084	2145279	antisense	4,23	0
ENSG00000266490	ENSG00000266490	17	30792372	30792833	lincRNA	4,23	0
ENSG00000234322	ST13P18	X	93287733	93288759	processed_pseudogene	4,22	0,2161218
ENSG00000240024	LINC00888	3	183447608	183456013	transcribed_unprocessed_pseudogene	4,21	3,30E-03
ENSG00000263878	DLGAP1-AS4	18	3962353	4013943	antisense	4,20	7,06E-02
ENSG00000263657	ENSG00000263657	17	29761103	29787836	antisense	4,20	0
ENSG00000205037	ENSG00000205037	16	88088041	88100985	lincRNA	4,20	0
ENSG00000260038	ENSG00000260038	16	57247350	57248492	sense_intronic	4,20	7,06E-02
ENSG00000270165	ENSG00000270165	16	67738588	67739922	sense_intronic	4,20	0
ENSG00000275040	ENSG00000275040	16	79619469	79620110	lincRNA	4,20	0,2161218
ENSG00000275673	ENSG00000275673	16	10934258	10934887	sense_intronic	4,20	0
ENSG00000278434	ENSG00000278434	16	4532216	4533670	sense_intronic	4,20	1,40E-02
ENSG00000246548	ENSG00000246548	14	77041064	77069503	lincRNA	4,20	1,40E-02
ENSG00000276957	ENSG00000276957	13	102593338	102593873	lincRNA	4,20	0,2161218
ENSG00000233560	KRT8P39	12	68705634	68707066	processed_pseudogene	4,20	7,06E-02
ENSG00000254427	ENSG00000254427	11	45355371	45366121	lincRNA	4,20	7,06E-02
ENSG00000255227	ENSG00000255227	11	29445487	29457393	lincRNA	4,20	0,2161218
ENSG00000260475	ENSG00000260475	10	99621055	99621918	lincRNA	4,20	0
ENSG00000267834	ENSG00000267834	9	129004502	129004998	lincRNA	4,20	7,06E-02
ENSG00000276462	ENSG00000276462	9	41100794	41106229	lincRNA	4,20	7,06E-02
ENSG00000254533	ENSG00000254533	8	144698614	144699185	lincRNA	4,20	1,40E-02
ENSG00000213385	ENSG00000213385	7	102755146	102755939	processed_pseudogene	4,20	7,06E-02
ENSG00000228019	ENSG00000228019	7	67307605	67308356	unprocessed_pseudogene	4,20	1,40E-02
ENSG00000231359	ENSG00000231359	7	6490793	6491098	processed_pseudogene	4,20	0,2161218
ENSG00000236861	ENSG00000236861	7	93969442	94011113	antisense	4,20	7,06E-02
ENSG00000277053	GTF2IP1	7	75185385	75237696	transcribed_unprocessed_pseudogene	4,20	0
ENSG00000220305	HNRNPH1P1	6	159712801	159713985	processed_pseudogene	4,20	7,06E-02
ENSG00000260604	ENSG00000260604	6	3904920	3911979	lincRNA	4,20	1,40E-02
ENSG00000261211	ENSG00000261211	6	6680309	6683633	lincRNA	4,20	7,06E-02
ENSG00000248590	GLDCP1	4	56593004	56595681	transcribed_processed_pseudogene	4,20	0
ENSG00000272498	ENSG00000272498	3	16339308	16339871	antisense	4,20	0
ENSG00000235586	ENSG00000235586	2	38601598	38602178	antisense	4,20	0
ENSG00000224238	WARS2-IT1	1	119047405	119064785	antisense	4,20	0
ENSG00000229509	ENSG00000229509	1	206333327	206333438	unprocessed_pseudogene	4,20	1,40E-02
ENSG00000260281	ITFG1-AS1	16	47144323	47162747	antisense	4,19	0
ENSG00000247473	CARS-AS1	11	3029009	3041260	antisense	4,18	0
ENSG00000227627	ENSG00000227627	6	152983750	152989223	antisense	4,18	0
ENSG00000273483	ENSG00000273483	1	112517799	112518441	antisense	4,18	0,4332376

ENSG00000227621	PHBP11	1	223856579	223857387	processed_pseudogene	4,18	0
ENSG00000182057	OGFRP1	22	42269753	42275196	lincRNA	4,17	6,27E-03
ENSG00000273061	CDC37L1-AS1	9	4676600	4679502	lincRNA	4,17	9,18E-02
ENSG00000227542	ENSG00000227542	2	191229165	191246172	lincRNA	4,17	0
ENSG00000231198	ENSG00000231198	7	39833286	39833793	processed_pseudogene	4,16	0
ENSG00000213683	ENSG00000213683	22	50754675	50755434	processed_pseudogene	4,15	0
ENSG00000253384	ENSG00000253384	8	18088569	18089687	processed_pseudogene	4,13	0
ENSG00000226400	ENSG00000226400	5	28927029	28927284	processed_pseudogene	4,13	0
ENSG00000235339	GEMIN2P2	3	41977008	41978034	processed_pseudogene	4,13	0,2746192
ENSG00000230793	SMARCE1P5	13	44594858	44596082	processed_pseudogene	4,12	1,40E-02
ENSG00000258297	ENSG00000258297	11	66666036	66668374	antisense	4,12	2,95E-02
ENSG00000249485	RBBP4P1	5	14797125	14798400	processed_pseudogene	4,10	0
ENSG00000228107	ENSG00000228107	21	36360630	36362040	sense_overlapping	4,10	0
ENSG00000268355	ENSG00000268355	19	41531206	41532174	lincRNA	4,10	0
ENSG00000273733	ENSG00000273733	19	11221083	11221573	antisense	4,10	7,06E-02
ENSG00000226101	ENSG00000226101	17	72072333	72093481	lincRNA	4,10	1,40E-02
ENSG00000250107	CACNA1G-AS1	17	50556207	50562108	antisense	4,10	1,40E-02
ENSG00000264016	ENSG00000264016	17	10741267	10769016	lincRNA	4,10	0
ENSG00000275897	ENSG00000275897	17	50475819	50478391	lincRNA	4,10	7,06E-02
ENSG00000259895	ENSG00000259895	16	2464950	2468213	antisense	4,10	0,2161218
ENSG00000273615	ENSG00000273615	16	54851640	54852733	lincRNA	4,10	7,06E-02
ENSG00000230724	LINC01001	11	127204	139612	lincRNA	4,10	0
ENSG00000269038	ENSG00000269038	11	64778954	64779405	lincRNA	4,10	7,06E-02
ENSG00000271848	ENSG00000271848	10	73654039	73674719	antisense	4,10	0
ENSG00000249491	EGFLAM-AS1	5	38425036	38427376	antisense	4,10	0
ENSG00000250015	ENSG00000250015	5	116302354	116304134	lincRNA	4,10	0
ENSG00000272239	ENSG00000272239	5	147401760	147401996	antisense	4,10	1,40E-02
ENSG00000240668	KRT8P36	3	138101478	138102917	processed_pseudogene	4,10	1,40E-02
ENSG00000241634	ENSG00000241634	3	108543367	108543875	processed_pseudogene	4,10	0
ENSG00000205716	FAM183DP	2	102249857	102250255	processed_pseudogene	4,10	0,2161218
ENSG00000232377	ENSG00000232377	2	143937073	143964156	antisense	4,10	0
ENSG00000232485	ENSG00000232485	2	216479030	216498761	processed_transcript	4,10	0
ENSG00000237624	OXCT2P1	1	39514956	39516490	unprocessed_pseudogene	4,10	0
ENSG00000234036	TXNP6	4	76958860	76959169	processed_pseudogene	4,10	0,2161218
ENSG00000237263	MAPK6PS3	13	42068932	42071093	processed_pseudogene	4,10	0
ENSG00000260776	ENSG00000260776	15	77914217	77926846	transcribed_unprocessed_pseudogene	4,09	3,30E-03
ENSG00000270919	ENSG00000270919	15	80580029	80580566	antisense	4,09	6,27E-03
ENSG00000272150	NBPF25P	1	145572345	145607858	transcribed_unprocessed_pseudogene	4,09	0
ENSG00000256299	ENSG00000256299	12	130810821	130812622	lincRNA	4,08	0,0358681
ENSG00000233892	PAIP1P1	6	30186798	30188614	processed_pseudogene	4,08	6,27E-03
ENSG00000272510	ENSG00000272510	1	15565611	15565956	antisense	4,08	0
ENSG00000232344	ENSG00000232344	17	18010643	18011822	processed_pseudogene	4,08	0
ENSG00000271795	ENSG00000271795	5	151509453	151512769	antisense	4,07	0,4036702
ENSG00000259375	ENSG00000259375	15	85579046	85580178	antisense	4,05	0
ENSG00000257494	ENSG00000257494	12	112256800	112259091	antisense	4,05	0
ENSG00000274292	ENSG00000274292	12	121800797	121803403	lincRNA	4,05	7,06E-02
ENSG00000255817	ENSG00000255817	12	64108763	64120489	antisense	4,05	0
ENSG00000229852	ENSG00000229852	6	73263215	73301401	antisense	4,04	1,95E-02
ENSG00000261654	ENSG00000261654	1	110936369	110942353	sense_overlapping	4,04	0
ENSG00000237296	SMG1P1	16	22437008	22492220	transcribed_unprocessed_pseudogene	4,04	0
ENSG00000248880	ICE2P1	4	188387471	188389666	processed_pseudogene	4,04	2,21E-02
ENSG00000225422	RBMS1P1	12	66234079	66235234	processed_pseudogene	4,04	0
ENSG00000251333	RTN3P1	4	145375543	145376253	processed_pseudogene	4,04	0
ENSG00000259234	ANKRD34C-AS1	15	79191707	79283945	lincRNA	4,04	1,40E-02
ENSG00000259010	ENSG00000259010	14	64600243	64601644	processed_pseudogene	4,03	0
ENSG00000224985	ENSG00000224985	1	161153760	161159349	antisense	4,03	0
ENSG00000261317	ENSG00000261317	16	89969791	89972544	antisense	4,03	0

ENSG00000133136	GNG5P2	X	110346703	110346909	processed_pseudogene	4,03	0,1836819
ENSG00000233799	ENSG00000233799	4	764487	765074	antisense	4,02	0
ENSG00000224856	ENSG00000224856	22	43957027	43957348	processed_pseudogene	4,02	0,1836819
ENSG00000216895	ENSG00000216895	7	155611231	155645205	antisense	4,02	0
ENSG00000254898	ENSG00000254898	8	38335981	38337551	antisense	4,02	0
ENSG00000244723	ASLP1	22	23649213	23653334	unprocessed_pseudogene	4,00	7,06E-02
ENSG00000273342	ENSG00000273342	22	21640844	21641284	lincRNA	4,00	7,06E-02
ENSG00000227698	ENSG00000227698	21	41874756	41877613	sense_intronic	4,00	1,40E-02
ENSG00000232680	ENSG00000232680	19	35432957	35434642	lincRNA	4,00	0,2161218
ENSG00000269815	ENSG00000269815	19	17296196	17296695	sense_intronic	4,00	1,40E-02
ENSG00000273791	ENSG00000273791	19	19908268	19909146	unprocessed_pseudogene	4,00	0,2161218
ENSG00000263982	ENSG00000263982	18	76372717	76378275	antisense	4,00	0
ENSG00000262052	ENSG00000262052	17	55842677	55872939	lincRNA	4,00	1,40E-02
ENSG00000267203	SNRPGP4	17	69318733	69318961	processed_pseudogene	4,00	0
ENSG00000277589	ENSG00000277589	17	36998598	37000034	sense_intronic	4,00	7,06E-02
ENSG00000260750	ENSG00000260750	16	87492555	87515635	lincRNA	4,00	1,40E-02
ENSG00000260816	ENSG00000260816	16	78895361	78899644	sense_intronic	4,00	0,2161218
ENSG00000261519	ENSG00000261519	16	66549280	66551189	lincRNA	4,00	0
ENSG00000245975	ENSG00000245975	15	58768072	58770974	lincRNA	4,00	0
ENSG00000259645	ENSG00000259645	15	69391192	69392149	sense_intronic	4,00	7,06E-02
ENSG00000261229	ENSG00000261229	15	79843547	79844304	lincRNA	4,00	0
ENSG00000259158	ADAM20P1	14	70468881	70483756	lincRNA	4,00	7,06E-02
ENSG00000255882	ENSG00000255882	12	9867027	9869808	antisense	4,00	0
ENSG00000257298	ENSG00000257298	12	50185580	50191363	sense_intronic	4,00	0
ENSG00000258230	ENSG00000258230	12	101773114	101773755	transcribed_processed_pseudogene	4,00	7,06E-02
ENSG00000275389	ENSG00000275389	12	124085761	124088598	lincRNA	4,00	0,2161218
ENSG00000279243	ENSG00000279243	11	65487241	65488136	lincRNA	4,00	0,2161218
ENSG00000226659	ENSG00000226659	10	80529597	80535942	antisense	4,00	1,40E-02
ENSG00000231748	ENSG00000231748	10	69265342	69268148	antisense	4,00	7,06E-02
ENSG00000276662	ENSG00000276662	10	147820	148751	unprocessed_pseudogene	4,00	1,40E-02
ENSG00000238245	MYO5BP2	9	62857208	62858484	processed_pseudogene	4,00	1,40E-02
ENSG00000254338	MAFA-AS1	8	143417679	143419150	antisense	4,00	0,2161218
ENSG00000272172	ENSG00000272172	8	143290399	143290621	antisense	4,00	7,06E-02
ENSG00000205482	SPDYE18	7	77052785	77059440	unprocessed_pseudogene	4,00	0
ENSG00000232546	ENSG00000232546	7	66848496	66858136	unprocessed_pseudogene	4,00	0
ENSG00000243107	ENSG00000243107	7	92200014	92206857	sense_overlapping	4,00	1,40E-02
ENSG00000224460	ENSG00000224460	6	142946047	142956698	lincRNA	4,00	0
ENSG00000270604	HCG17	6	30234039	30326134	lincRNA	4,00	9,18E-02
ENSG00000250222	ENSG00000250222	5	181191924	181194429	antisense	4,00	2,95E-02
ENSG00000272308	ENSG00000272308	5	60866457	60866935	lincRNA	4,00	0,2161218
ENSG00000244459	ENSG00000244459	4	1574062	1580253	lincRNA	4,00	0,2161218
ENSG00000243224	ENSG00000243224	3	52239258	52241097	antisense	4,00	0
ENSG00000246022	ALDH1L1-AS2	3	126180065	126210169	antisense	4,00	0
ENSG00000271843	ENSG00000271843	3	64008082	64008692	lincRNA	4,00	6,27E-03
ENSG00000203327	ENSG00000203327	2	55214387	55216126	antisense	4,00	0
ENSG00000204588	LINC01123	2	109987063	109996140	lincRNA	4,00	0,2161218
ENSG00000213453	FTH1P3	2	27392784	27393367	processed_pseudogene	4,00	7,06E-02
ENSG00000272183	ENSG00000272183	2	74501717	74502365	antisense	4,00	7,06E-02
ENSG00000273113	ENSG00000273113	2	241581922	241582726	lincRNA	4,00	7,06E-02
ENSG00000215014	ENSG00000215014	1	1574975	1577075	lincRNA	4,00	0
ENSG00000215840	ENSG00000215840	1	161406068	161407082	processed_pseudogene	4,00	0,2161218
ENSG00000237436	ENSG00000237436	1	6783892	6784843	antisense	4,00	7,06E-02
ENSG00000240520	UOX	1	84365428	84397831	unitary_pseudogene	4,00	7,06E-02
ENSG00000203588	IGBP1-AS1	X	70163842	70165206	antisense	4,00	1,40E-02
ENSG00000175741	RWDD4P2	7	39854494	39855056	processed_pseudogene	3,99	0

ENSG00000281344	HELLPAR	12	102197585	102402596	macro_lncRNA	3,99	0
ENSG00000254894	NAV2-AS1	11	20119684	20120632	antisense	3,98	0
ENSG00000259468	ENSG00000259468	15	34255286	34257832	antisense	3,96	0
ENSG00000218676	BRD7P4	6	111430260	111431781	processed_pseudogene	3,96	0
ENSG00000218069	RSL24D1P1	6	27780619	27781111	processed_pseudogene	3,96	0
ENSG00000226138	ENSG00000226138	12	48085062	48085421	processed_pseudogene	3,95	0,1836819
ENSG00000258908	ENSG00000258908	14	20474789	20477089	lincRNA	3,95	2,21E-02
ENSG00000228737	ENSG00000228737	5	141618414	141626481	antisense	3,95	0
ENSG00000256928	ENSG00000256928	11	73395559	73396436	antisense	3,94	2,95E-02
ENSG00000218418	ENSG00000218418	6	80064286	80070199	processed_pseudogene	3,94	0
ENSG00000261329	ENSG00000261329	16	27678940	27718806	antisense	3,93	0
ENSG00000262678	ENSG00000262678	17	4986466	4987324	antisense	3,93	0
ENSG00000218426	ENSG00000218426	6	153282287	153282733	processed_pseudogene	3,93	0,4332376
ENSG00000269952	ENSG00000269952	10	35210416	35210750	sense_intronic	3,93	0,2161218
ENSG00000245281	ENSG00000245281	8	18084868	18096394	antisense	3,93	0
ENSG00000260017	ENSG00000260017	16	18803083	18812181	antisense	3,92	0
ENSG00000261267	ENSG00000261267	16	48559661	48587403	antisense	3,92	0
ENSG00000267323	SLC25A1P5	19	27805423	27806205	processed_pseudogene	3,92	6,27E-03
ENSG00000281649	EBLN3	9	37079857	37090507	lincRNA	3,91	0
ENSG00000269903	ENSG00000269903	12	57814494	57814926	lincRNA	3,91	0,4332376
ENSG00000233266	HMGB1P31	2	54051334	54051760	processed_pseudogene	3,91	6,27E-03
ENSG00000274828	ENSG00000274828	18	79677287	79679358	lincRNA	3,91	2,95E-02
ENSG00000273221	ENSG00000273221	1	111184415	111185061	antisense	3,90	3,30E-03
ENSG00000206028	ENSG00000206028	22	26667693	26672654	lincRNA	3,90	1,40E-02
ENSG00000230772	VN1R108P	20	25734264	25735093	unprocessed_pseudogene	3,90	0
ENSG00000275401	ENSG00000275401	20	38418483	38419202	lincRNA	3,90	1,40E-02
ENSG00000277692	ENSG00000277692	20	32355053	32355734	lincRNA	3,90	0,2161218
ENSG00000267033	ENSG00000267033	19	16186276	16189458	antisense	3,90	0
ENSG00000267650	ENSG00000267650	19	9995997	9997163	antisense	3,90	7,06E-02
ENSG00000273189	ENSG00000273189	19	49474586	49487637	processed_transcript	3,90	7,06E-02
ENSG00000267095	ENSG00000267095	17	60135762	60140081	lincRNA	3,90	0,2161218
ENSG00000275263	ENSG00000275263	16	30956872	30957199	lincRNA	3,90	1,40E-02
ENSG00000246877	DNM1P35	15	75727670	75738623	antisense	3,90	7,06E-02
ENSG00000257231	DYNLL1P4	12	113789542	113789807	processed_pseudogene	3,90	7,06E-02
ENSG00000277595	ENSG00000277595	12	110032245	110032803	sense_intronic	3,90	0,2161218
ENSG00000227972	PRKRIRP3	10	52411135	52413492	processed_pseudogene	3,90	0
ENSG00000236514	ENSG00000236514	10	37857740	37859110	antisense	3,90	1,40E-02
ENSG00000270874	ENSG00000270874	10	86854696	86856236	processed_pseudogene	3,90	7,06E-02
ENSG00000224083	ENSG00000224083	9	5096666	5098193	unprocessed_pseudogene	3,90	0
ENSG00000226877	ENSG00000226877	9	83829656	83837861	antisense	3,90	0
ENSG00000234373	SNX18P7	9	33576380	33577014	processed_pseudogene	3,90	7,06E-02
ENSG00000236024	PRRX2-AS1	9	129712896	129718635	antisense	3,90	0,2161218
ENSG00000253426	ENSG00000253426	8	9151742	9168136	lincRNA	3,90	7,06E-02
ENSG00000229862	ENSG00000229862	6	73523618	73570596	antisense	3,90	0
ENSG00000260212	ENSG00000260212	6	127659424	127660338	unprocessed_pseudogene	3,90	1,40E-02
ENSG00000272053	ENSG00000272053	6	25014952	25042170	lincRNA	3,90	6,27E-03
ENSG00000272980	ENSG00000272980	6	166999405	167139141	processed_transcript	3,90	0
ENSG00000271849	ENSG00000271849	5	109687802	109688329	lincRNA	3,90	0,2161218
ENSG00000270480	ENSG00000270480	4	82691737	82692468	processed_pseudogene	3,90	1,40E-02
ENSG00000272576	ENSG00000272576	4	51918772	51919381	lincRNA	3,90	0,2161218
ENSG00000235845	ENSG00000235845	3	44899178	44900925	antisense	3,90	1,40E-02
ENSG00000260743	ENSG00000260743	3	179340322	179341887	lincRNA	3,90	7,06E-02
ENSG00000227470	ENSG00000227470	2	108878308	108878460	processed_pseudogene	3,90	1,40E-02
ENSG00000260331	ENSG00000260331	2	18547386	18548204	lincRNA	3,90	0,2161218
ENSG00000271443	ENSG00000271443	2	38535258	38536249	transcribed_processed_pseudogene	3,90	0,2161218
ENSG00000162888	C1orf147	1	206491116	206497728	antisense	3,90	0
ENSG00000177757	FAM87B	1	817371	819837	lincRNA	3,90	0

ENSG00000203819	HIST2H2BC	1	149850193	149850772	processed_pseudogene	3,90	0
ENSG00000235790	ENSG00000235790	1	31644049	31660162	antisense	3,90	7,06E-02
ENSG00000276255	ENSG00000276255	1	228073909	228076550	lincRNA	3,90	1,40E-02
ENSG00000214653	HNRNPA3P3	X	140032344	140033480	processed_pseudogene	3,90	1,40E-02
ENSG00000271918	ENSG00000271918	5	116083807	116085416	antisense	3,89	0
ENSG00000260942	CAPN10-AS1	2	240582700	240586699	antisense	3,89	2,95E-02
ENSG00000178660	ARMC10P1	3	94506766	94507620	processed_pseudogene	3,88	0
ENSG00000228172	ENSG00000228172	1	25816749	25820797	antisense	3,87	0
ENSG00000254198	ENSG00000254198	8	43125995	43126397	processed_pseudogene	3,86	0
ENSG00000260999	ENSG00000260999	16	68927547	68948261	lincRNA	3,86	0
ENSG00000236383	LINC00854	17	43216941	43305976	processed_transcript	3,86	0
ENSG00000227963	ENSG00000227963	1	110286375	110339171	antisense	3,86	2,21E-02
ENSG00000260235	ENSG00000260235	15	75624793	75625690	antisense	3,85	0
ENSG00000236859	NIFK-AS1	2	121649650	121728481	antisense	3,84	0
ENSG00000236048	ENSG00000236048	7	12471352	12472545	processed_pseudogene	3,84	2,21E-02
ENSG00000165121	ENSG00000165121	9	85815966	85849511	unprocessed_pseudogene	3,83	0
ENSG00000273013	ENSG00000273013	3	196474801	196475394	antisense	3,83	0
ENSG00000236312	RPL34P34	19	22368599	22368952	processed_pseudogene	3,82	1,40E-02
ENSG00000264304	ENSG00000264304	17	28892469	28893342	antisense	3,82	3,30E-03
ENSG00000273618	ENSG00000273618	14	23530682	23532367	antisense	3,82	2,95E-02
ENSG00000230735	ENSG00000230735	1	89629725	89676386	sense_intronic	3,82	0
ENSG00000157306	ENSG00000157306	14	23511760	23560778	processed_transcript	3,82	3,30E-03
ENSG00000257122	RRN3P3	16	22418672	22437715	transcribed_unprocessed_pseudogene	3,81	4,86E-02
ENSG00000174680	GRIK1-AS1	21	29748175	29764002	antisense	3,80	0
ENSG00000230753	ZNF341-AS1	20	33787373	33811097	antisense	3,80	7,06E-02
ENSG00000268941	ENSG00000268941	20	58634772	58635738	lincRNA	3,80	0,2161218
ENSG00000267421	ENSG00000267421	19	56536156	56538575	lincRNA	3,80	1,40E-02
ENSG00000267651	ENSG00000267651	18	37234119	37236242	lincRNA	3,80	0
ENSG00000274184	ENSG00000274184	18	35268218	35270238	lincRNA	3,80	7,06E-02
ENSG00000266947	ENSG00000266947	17	35231450	35242963	antisense	3,80	7,06E-02
ENSG00000262140	ENSG00000262140	16	71833787	71835932	unprocessed_pseudogene	3,80	1,40E-02
ENSG00000243094	ENSG00000243094	15	34085911	34086314	processed_pseudogene	3,80	0
ENSG00000260469	C15orf59-AS1	15	73752317	73770613	lincRNA	3,80	0,2161218
ENSG00000258591	ENSG00000258591	14	39108342	39108707	processed_pseudogene	3,80	0,2161218
ENSG00000277128	ENSG00000277128	14	19127736	19175530	lincRNA	3,80	1,40E-02
ENSG00000176268	CYCSP34	13	40863599	40863902	processed_pseudogene	3,80	0
ENSG00000099869	IGF2-AS	11	2140501	2148666	antisense	3,80	7,06E-02
ENSG00000228353	ENSG00000228353	10	3767915	3768751	antisense	3,80	0
ENSG00000226669	ENSG00000226669	9	3684076	3689626	lincRNA	3,80	7,06E-02
ENSG00000254527	ENPP7P12	8	12205759	12206389	processed_pseudogene	3,80	0
ENSG00000165178	NCF1C	7	75156639	75172044	unprocessed_pseudogene	3,80	0
ENSG00000213394	RPSAP46	7	101203614	101204495	processed_pseudogene	3,80	7,06E-02
ENSG00000204338	CYP21A1P	6	32005636	32008451	transcribed_unprocessed_pseudogene	3,80	7,06E-02
ENSG00000231441	ENSG00000231441	6	56844002	56864078	antisense	3,80	0,2161218
ENSG00000250764	ENSG00000250764	5	32103445	32121941	antisense	3,80	2,21E-02
ENSG00000251314	ENSG00000251314	5	95962001	96631085	antisense	3,80	0
ENSG00000269936	ENSG00000269936	5	149430286	149432834	lincRNA	3,80	1,40E-02
ENSG00000270257	ENSG00000270257	4	67638177	67638359	processed_pseudogene	3,80	1,40E-02
ENSG00000248557	ENSG00000248557	3	126624792	126626061	processed_pseudogene	3,80	7,06E-02
ENSG00000249558	RCC2P4	3	126766434	126767475	processed_pseudogene	3,80	0
ENSG00000268603	ENSG00000268603	2	219497611	219498246	antisense	3,80	0,2161218
ENSG00000231346	LINC01160	1	111599655	111608723	lincRNA	3,80	0
ENSG00000260940	ENSG00000260940	1	101243158	101243749	sense_overlapping	3,80	0,2161218
ENSG00000271576	ENSG00000271576	1	84076331	84077931	lincRNA	3,80	0,2161218
ENSG00000275557	ENSG00000275557	1	149607765	149612402	lincRNA	3,80	0
ENSG00000231104	ENSG00000231104	10	118017487	118045810	antisense	3,80	0

ENSG00000263327	TAPT1-AS1	4	16226685	16320140	antisense		3,79	0,4332376
ENSG00000259802	ENSG00000259802	5	10352701	10353601	antisense		3,78	2,95E-02
ENSG00000232150	ST13P4	13	50172089	50173181	processed_pseudogene		3,78	4,86E-02
ENSG00000254988	ENSG00000254988	11	76955417	76978619	antisense		3,78	0
ENSG00000243926	TIPARP-AS1	3	156671862	156674378	antisense		3,78	0,1836819
ENSG00000273004	ENSG00000273004	1	185317779	185318530	lincRNA		3,77	1,40E-02
ENSG00000213630	BOLA3P3	5	126663337	126663568	processed_pseudogene		3,77	0,3318209
ENSG00000254317	ENSG00000254317	8	129832301	129844504	antisense		3,76	9,18E-02
ENSG00000274104	ENSG00000274104	19	34733298	34733837	lincRNA		3,75	7,06E-02
ENSG00000224437	PIGUP1	9	70916997	70917501	processed_pseudogene		3,75	3,30E-03
ENSG00000176812	ENSG00000176812	X	25194381	25195207	processed_pseudogene		3,75	0,1836819
ENSG00000250186	ENSG00000250186	17	49404081	49405197	antisense		3,74	3,30E-03
ENSG00000272516	ENSG00000272516	10	22332404	22332987	lincRNA		3,73	4,86E-02
ENSG00000250740	ENSG00000250740	4	105927060	105932722	antisense		3,73	3,30E-03
ENSG00000260244	ENSG00000260244	4	155734448	155737062	sense_overlapping		3,73	3,30E-03
ENSG00000203362	ENSG00000203362	6	43588230	43591362	antisense		3,72	0,0358681
ENSG00000246283	ENSG00000246283	15	42739118	42743202	lincRNA		3,72	0
ENSG00000249565	SERBP1P5	4	78180866	78182046	processed_pseudogene		3,70	0
ENSG00000224747	MTCYBP21	21	44469929	44472516	processed_pseudogene		3,70	0,2161218
ENSG00000271784	ENSG00000271784	20	46689659	46690289	lincRNA		3,70	7,06E-02
ENSG00000260500	ENSG00000260500	19	7918652	7919157	antisense		3,70	0,2161218
ENSG00000267474	ENSG00000267474	19	14458401	14459366	sense_intronic		3,70	0,2161218
ENSG00000274776	ENSG00000274776	18	46786699	46789297	lincRNA		3,70	0
ENSG00000241157	ENSG00000241157	17	60245288	60245647	processed_pseudogene		3,70	0,2161218
ENSG00000263786	ENSG00000263786	17	75145261	75146546	sense_intronic		3,70	0
ENSG00000267221	ENSG00000267221	17	42040713	42043117	sense_intronic		3,70	0
ENSG00000274758	ENSG00000274758	17	2366589	2366791	antisense		3,70	7,06E-02
ENSG00000279066	HEXDC-IT1	17	82425498	82427310	sense_intronic		3,70	1,40E-02
ENSG00000260859	ENSG00000260859	16	84828263	84829242	sense_intronic		3,70	0
ENSG00000258590	NBEAP1	15	20657638	20688408	transcribed_unprocessed_pseudogene		3,70	0
ENSG00000259738	ZNF444P1	15	58865006	58865303	processed_pseudogene		3,70	0,2161218
ENSG00000249695	ENSG00000249695	12	137411	149169	antisense		3,70	1,40E-02
ENSG00000275476	ENSG00000275476	12	29277397	29277882	antisense		3,70	0,2161218
ENSG00000255026	ENSG00000255026	11	287305	288987	antisense		3,70	7,06E-02
ENSG00000232075	MRPL35P2	10	63634317	63634827	processed_pseudogene		3,70	7,06E-02
ENSG00000188801	ZNF322P1	9	97198303	97199511	processed_pseudogene		3,70	0
ENSG00000213362	FTH1P12	9	15527133	15527683	processed_pseudogene		3,70	0,2161218
ENSG00000230188	ENSG00000230188	9	37490421	37490893	processed_pseudogene		3,70	7,06E-02
ENSG00000253161	LINC01605	8	37421341	37554183	lincRNA		3,70	0,2161218
ENSG00000271781	ENSG00000271781	5	675826	676616	antisense		3,70	1,40E-02
ENSG00000280026	ENSG00000280026	5	141350109	141350662	antisense		3,70	0
ENSG00000231574	ENSG00000231574	3	177816865	177899224	lincRNA		3,70	0
ENSG00000241278	ENPP7P4	3	125848223	125909372	unprocessed_pseudogene		3,70	6,27E-03
ENSG00000243415	ENSG00000243415	3	146064042	146105204	antisense		3,70	7,06E-02
ENSG00000223922	ASS1P2	2	38810432	38811654	processed_pseudogene		3,70	1,40E-02
ENSG00000215808	LINC01139	1	238480384	238486023	lincRNA		3,70	0,2161218
ENSG00000235545	ENSG00000235545	1	62688482	62710694	antisense		3,70	1,40E-02
ENSG00000237200	ZBTB40-IT1	1	22517474	22519708	sense_intronic		3,70	0
ENSG00000237505	PKN2-AS1	1	88537513	88685204	antisense		3,70	1,40E-02
ENSG00000278467	ENSG00000278467	1	223994262	223995196	lincRNA		3,70	0,2161218
ENSG00000233073	ENSG00000233073	7	86784226	86786670	antisense		3,70	4,86E-02
ENSG00000276805	ENSG00000276805	10	38403189	38452153	transcribed_unprocessed_pseudogene		3,69	0
ENSG00000241671	ENSG00000241671	3	153129074	153129640	processed_pseudogene		3,69	0
ENSG00000225361	PPP1R26-AS1	9	135462727	135480777	antisense		3,69	0,0358681
ENSG00000248932	ENSG00000248932	3	139389815	139583319	antisense		3,69	0
ENSG00000235042	ENSG00000235042	2	216385288	216412696	antisense		3,68	0
ENSG00000251034	ENSG00000251034	8	22540845	22545405	antisense		3,68	2,95E-02
ENSG00000219451	ENSG00000219451	7	20827326	20827753	processed_pseudogene		3,68	7,06E-02

ENSG00000256694	ENSG00000256694	12	164664	166321	antisense	3,67	0
ENSG00000234664	HMGN2P5	15	29730713	29730985	processed_pseudogene	3,66	4,86E-02
ENSG00000258034	ENSG00000258034	12	115961187	115962733	antisense	3,65	0
ENSG00000267742	FAM60CP	18	60016778	60017441	processed_pseudogene	3,64	0
ENSG00000281376	ABALON	20	31721507	31723409	antisense	3,64	0
ENSG00000203414	BTBD7P1	10	13156684	13158136	processed_pseudogene	3,64	0,4332376
ENSG00000260494	ENSG00000260494	16	30537202	30538231	unprocessed_pseudogene	3,63	0
ENSG00000234771	SLC25A25-AS1	9	128108581	128118693	antisense	3,63	0,1187106
ENSG00000279658	ENSG00000279658	3	97836986	97874691	antisense	3,63	0
ENSG00000235734	HMGN1P36	2	97827248	97827545	processed_pseudogene	3,62	0,3565078
ENSG00000276535	ENSG00000276535	9	88144851	88145850	processed_pseudogene	3,62	2,21E-02
ENSG00000223653	ENSG00000223653	1	85276715	85448124	antisense	3,62	6,27E-03
ENSG00000228436	ENSG00000228436	1	38860000	38919396	antisense	3,62	0,1187106
ENSG00000250138	ENSG00000250138	5	69631963	69636399	unprocessed_pseudogene	3,62	3,30E-03
ENSG00000255629	ENSG00000255629	12	64146388	64147857	antisense	3,61	3,30E-03
ENSG00000267064	UXT-AS1	X	47658833	47660377	antisense	3,61	4,86E-02
ENSG00000231035	RPL7L1P9	2	217760547	217761291	processed_pseudogene	3,61	3,30E-03
ENSG00000268366	ENSG00000268366	19	40443436	40444087	antisense	3,61	0
ENSG00000255062	ENSG00000255062	11	126294298	126304318	antisense	3,60	0
ENSG00000175611	LINC00476	9	95759231	95875977	processed_transcript	3,60	0
ENSG00000211683	ENSG00000211683	22	23630618	23638941	processed_transcript	3,60	1,40E-02
ENSG00000281530	DGCR12	22	19061041	19061843	sense_intronic	3,60	0
ENSG00000232118	BACH1-AS1	21	29370019	29376339	lincRNA	3,60	1,40E-02
ENSG00000231742	LINC01273	20	50172550	50176671	lincRNA	3,60	1,40E-02
ENSG00000268945	ENSG00000268945	19	12682693	12687279	lincRNA	3,60	1,40E-02
ENSG00000269578	ENSG00000269578	19	16586905	16587985	sense_intronic	3,60	1,40E-02
ENSG00000265100	ENSG00000265100	17	68246629	68247938	antisense	3,60	7,06E-02
ENSG00000182376	ENSG00000182376	16	88742767	88745748	antisense	3,60	0,2161218
ENSG00000274677	ENSG00000274677	16	84085979	84086590	sense_intronic	3,60	0
ENSG00000276075	ENSG00000276075	16	67517862	67528675	antisense	3,60	1,40E-02
ENSG00000259744	ENSG00000259744	15	70848883	70849770	sense_intronic	3,60	0,2161218
ENSG00000247970	ENSG00000247970	14	99604556	99625740	lincRNA	3,60	1,40E-02
ENSG00000168852	TPTE2P5	13	40822296	40921749	transcribed_unprocessed_pseudogene	3,60	0
ENSG00000249790	ENSG00000249790	12	8788257	8795789	lincRNA	3,60	1,40E-02
ENSG00000258285	TESC-AS1	12	117099481	117142091	lincRNA	3,60	0,2161218
ENSG00000254639	ENSG00000254639	11	46238382	46239267	lincRNA	3,60	0,2161218
ENSG00000256006	ENSG00000256006	11	18405609	18406731	sense_intronic	3,60	0
ENSG00000270030	ENSG00000270030	11	325703	326294	lincRNA	3,60	7,06E-02
ENSG00000180539	C9orf139	9	137027464	137037957	antisense	3,60	1,40E-02
ENSG00000232486	ENSG00000232486	9	105758164	105760755	processed_pseudogene	3,60	0
ENSG00000269946	ENSG00000269946	9	94166289	94200627	lincRNA	3,60	7,06E-02
ENSG00000273381	ENSG00000273381	9	91206175	91210299	lincRNA	3,60	1,40E-02
ENSG00000250563	KNOP1P5	8	126072587	126074231	processed_pseudogene	3,60	0,2161218
ENSG00000204876	ENSG00000204876	7	155962632	155966343	lincRNA	3,60	7,06E-02
ENSG00000272556	GTF2IP13	7	45769105	45815263	transcribed_unprocessed_pseudogene	3,60	7,06E-02
ENSG00000272950	ENSG00000272950	7	98322853	98323430	antisense	3,60	1,40E-02
ENSG00000230845	GSTA10P	6	52873014	52879940	unprocessed_pseudogene	3,60	1,40E-02
ENSG00000248469	ENSG00000248469	5	176049678	176062021	antisense	3,60	0
ENSG00000248925	ENSG00000248925	5	269858	271516	antisense	3,60	0
ENSG00000250787	HMGN1P17	5	56381781	56382075	processed_pseudogene	3,60	0,2161218
ENSG00000251330	ENSG00000251330	5	148430159	148430807	antisense	3,60	7,06E-02
ENSG00000249096	ENSG00000249096	4	184365183	184382306	lincRNA	3,60	1,40E-02
ENSG00000249717	ENSG00000249717	4	74955974	74970362	antisense	3,60	7,06E-02
ENSG00000226441	PLCL2-AS1	3	17042742	17044192	antisense	3,60	0
ENSG00000272902	ENSG00000272902	2	101151660	101155412	lincRNA	3,60	1,40E-02
ENSG00000176320	ENSG00000176320	1	158197922	158203877	lincRNA	3,60	1,40E-02

ENSG00000198358	ENSG00000198358	1	160932465	160949922	antisense	3,60	7,06E-02
ENSG00000224183	SDHDP6	1	25294164	25294643	processed_pseudogene	3,60	1,40E-02
ENSG00000227141	ENSG00000227141	1	179586705	179589175	processed_pseudogene	3,60	0,2161218
ENSG00000229828	PDE4DIPP1	1	148080598	148093883	unprocessed_pseudogene	3,60	1,40E-02
ENSG00000231181	ENSG00000231181	1	9576427	9576985	processed_pseudogene	3,60	7,06E-02
ENSG00000234571	ENSG00000234571	1	143877731	143885076	lincRNA	3,60	0
ENSG00000258082	ENSG00000258082	1	234979647	234980804	lincRNA	3,60	7,06E-02
ENSG00000268288	ENSG00000268288	1	151766486	151767000	antisense	3,60	1,40E-02
ENSG00000268949	MRPS17P1	1	44988705	44991834	processed_pseudogene	3,60	7,06E-02
ENSG00000272691	ENSG00000272691	1	85578500	85578742	lincRNA	3,60	7,06E-02
ENSG00000282057	ENSG00000282057	1	85482281	85578250	lincRNA	3,60	1,40E-02
ENSG00000224401	RPL7P57	X	47840963	47841702	processed_pseudogene	3,60	0
ENSG00000258800	ENSG00000258800	14	63598874	63599248	lincRNA	3,60	0
ENSG00000260947	ENSG00000260947	9	33697459	33700986	lincRNA	3,59	0
ENSG00000275437	ENSG00000275437	20	62402236	62405935	sense_intronic	3,58	0
ENSG00000234424	ENSG00000234424	9	85753306	85786575	unprocessed_pseudogene	3,57	1,40E-02
ENSG00000248530	BCL2L12P1	3	131526447	131527179	processed_pseudogene	3,56	2,95E-02
ENSG00000214223	HNRNPA1P10	19	11666069	11667030	processed_pseudogene	3,56	0,2746192
ENSG00000237298	TTN-AS1	2	178521183	178779963	antisense	3,55	0
ENSG00000264558	ENSG00000264558	17	47682417	47682683	antisense	3,54	0
ENSG00000227288	ENSG00000227288	1	81501794	81503468	processed_pseudogene	3,54	0
ENSG00000251226	ENSG00000251226	11	134735596	134763810	lincRNA	3,53	0,2161218
ENSG00000242358	RPS21P4	4	16256308	16256555	processed_pseudogene	3,52	2,21E-02
ENSG00000273199	ENSG00000273199	21	36319792	36320670	antisense	3,52	0
ENSG00000233547	ENSG00000233547	10	112950646	112951875	antisense	3,51	0
ENSG00000246477	ENSG00000246477	8	11315859	11325429	antisense	3,51	0
ENSG00000198547	C20orf203	20	32631652	32673941	lincRNA	3,50	7,06E-02
ENSG00000225872	LINC01529	19	35788876	35797885	lincRNA	3,50	0,2161218
ENSG00000269646	ENSG00000269646	19	52923382	52924075	lincRNA	3,50	6,27E-03
ENSG00000277531	ENSG00000277531	19	46424697	46425237	lincRNA	3,50	0,2161218
ENSG00000263862	LINC01543	18	26422995	26426101	lincRNA	3,50	0,2161218
ENSG00000263321	ENSG00000263321	17	82729164	82734143	lincRNA	3,50	0,3318209
ENSG00000264107	ENSG00000264107	17	31090787	31095450	antisense	3,50	0
ENSG00000271749	ENSG00000271749	17	63393355	63393586	unprocessed_pseudogene	3,50	0
ENSG00000228201	ENSG00000228201	16	648473	649200	antisense	3,50	1,40E-02
ENSG00000261669	ENSG00000261669	16	24803451	24819739	antisense	3,50	1,40E-02
ENSG00000274099	ABCB10P1	15	23179869	23182049	processed_pseudogene	3,50	0,2161218
ENSG00000258949	ENSG00000258949	14	44898908	44911863	antisense	3,50	0
ENSG00000233672	RNASEH2B-AS1	13	50862172	50910764	antisense	3,50	0
ENSG00000226413	OR8T1P	12	48442030	48442947	unprocessed_pseudogene	3,50	0
ENSG00000258099	ENSG00000258099	12	111599498	111600256	antisense	3,50	7,06E-02
ENSG00000224992	ENSG00000224992	9	132768965	132770212	sense_intronic	3,50	7,06E-02
ENSG00000230303	ENSG00000230303	9	85785722	85793480	lincRNA	3,50	0
ENSG00000237711	MTCO3P11	9	5100236	5101009	unprocessed_pseudogene	3,50	0
ENSG00000225535	LINC01393	7	115078958	115126314	lincRNA	3,50	0,3318209
ENSG00000240758	ENSG00000240758	7	128455937	128469197	processed_transcript	3,50	1,40E-02
ENSG00000273432	ENSG00000273432	7	43951910	44019151	processed_transcript	3,50	7,06E-02
ENSG00000248126	ENSG00000248126	5	1598127	1598247	unprocessed_pseudogene	3,50	0
ENSG00000248677	ENSG00000248677	5	6686325	6707711	lincRNA	3,50	0,2161218
ENSG00000261269	ENSG00000261269	5	72439903	72442387	sense_overlapping	3,50	0,2161218
ENSG00000272389	ENSG00000272389	5	112898023	112898371	antisense	3,50	0,2161218
ENSG00000272717	ENSG00000272717	4	139556799	139557643	antisense	3,50	7,06E-02
ENSG00000224962	PSAT1P4	3	166691187	166691712	processed_pseudogene	3,50	0
ENSG00000273125	ENSG00000273125	3	107430930	107463912	lincRNA	3,50	7,06E-02
ENSG00000227479	ENSG00000227479	2	240227560	240256035	lincRNA	3,50	7,06E-02

ENSG00000233786	CDC27P1	2	132262328	132263042	processed_pseudogene	3,50	2,21E-02
ENSG00000237916	ENSG00000237916	2	109552536	109552846	processed_pseudogene	3,50	0
ENSG00000228084	ENSG00000228084	1	99968383	99969864	antisense	3,50	0,2161218
ENSG00000232671	ENSG00000232671	1	151280024	151281929	antisense	3,50	2,95E-02
ENSG00000236936	ENSG00000236936	1	21266082	21267251	antisense	3,50	0
ENSG00000268869	ESPNP	1	16687339	16720157	transcribed_unprocessed_pseudogene	3,50	7,06E-02
ENSG00000237863	ENSG00000237863	X	108736011	108738903	antisense	3,50	1,40E-02
ENSG00000229111	MED4-AS1	13	48077137	48079991	antisense	3,50	0
ENSG00000225963	ENSG00000225963	2	230121370	230174223	antisense	3,50	0
ENSG00000261505	ENSG00000261505	16	1317891	1322845	antisense	3,49	3,30E-03
ENSG00000226535	ENSG00000226535	9	107169569	107171148	processed_pseudogene	3,49	0,0358681
ENSG00000237017	ENSG00000237017	19	54119511	54125343	antisense	3,49	0
ENSG00000273352	ENSG00000273352	18	9519449	9520199	sense_intronic	3,47	3,30E-03
ENSG00000261118	ENSG00000261118	16	89492017	89504460	antisense	3,47	0,1392851
ENSG00000257681	ENSG00000257681	12	103746315	103768858	antisense	3,47	0,4036702
ENSG00000241525	ENSG00000241525	17	404468	414023	antisense	3,47	1,40E-02
ENSG00000251307	ENSG00000251307	5	55233934	55295201	antisense	3,46	0
ENSG00000231840	ENSG00000231840	7	143255264	143287380	antisense	3,45	0,0358681
ENSG00000213115	ENSG00000213115	2	188227189	188228382	processed_pseudogene	3,45	0,0358681
ENSG00000259977	ENSG00000259977	X	37906147	37949405	lincRNA	3,45	3,30E-03
ENSG00000170846	ENSG00000170846	4	6673451	6676047	lincRNA	3,44	1,40E-02
ENSG00000273828	ENSG00000273828	20	46364551	46390885	antisense	3,43	0
ENSG00000271780	ENSG00000271780	14	101948347	101949425	lincRNA	3,43	0
ENSG00000238103	RPL9P7	X	23836742	23837321	processed_pseudogene	3,43	0,4036702
ENSG00000269177	ENSG00000269177	19	43553445	43555494	antisense	3,41	0
ENSG00000273492	ENSG00000273492	21	26170871	26217381	lincRNA	3,40	1,40E-02
ENSG00000236992	RPL12P12	20	19823443	19823943	processed_pseudogene	3,40	7,06E-02
ENSG00000266983	ENSG00000266983	19	5978403	6020363	antisense	3,40	0
ENSG00000263916	ENSG00000263916	18	49484536	49486149	sense_intronic	3,40	0,2161218
ENSG00000274354	ENSG00000274354	18	58511598	58512310	sense_intronic	3,40	0,2161218
ENSG00000197291	RAMP2-AS1	17	42753914	42761257	lincRNA	3,40	7,06E-02
ENSG00000262410	ENSG00000262410	17	82745068	82745709	sense_intronic	3,40	7,06E-02
ENSG00000263531	ENSG00000263531	17	30863921	30864940	antisense	3,40	0,2161218
ENSG00000264772	ENSG00000264772	17	7572826	7582024	processed_transcript	3,40	0,2161218
ENSG00000265478	ENSG00000265478	17	18809956	18810940	lincRNA	3,40	0,2161218
ENSG00000267731	ENSG00000267731	17	68189884	68192802	lincRNA	3,40	0
ENSG00000267072	ENSG00000267072	16	5010909	5043999	lincRNA	3,40	1,40E-02
ENSG00000270049	ENSG00000270049	16	67481314	67505063	antisense	3,40	0
ENSG00000275236	ENSG00000275236	16	74289593	74291052	lincRNA	3,40	0,2161218
ENSG00000261064	ENSG00000261064	15	32536047	32587613	lincRNA	3,40	1,40E-02
ENSG00000196668	LINC00173	12	116533422	116536518	processed_transcript	3,40	0,2161218
ENSG00000258345	ENSG00000258345	12	56118968	56119939	antisense	3,40	6,27E-03
ENSG00000270048	ENSG00000270048	12	123962555	123962817	sense_intronic	3,40	7,06E-02
ENSG00000255237	ENSG00000255237	11	462930	463899	antisense	3,40	0,2161218
ENSG00000256944	ENSG00000256944	11	60913166	60914052	antisense	3,40	1,40E-02
ENSG00000228352	ENSG00000228352	9	34084332	34096225	antisense	3,40	1,40E-02
ENSG00000236254	MTND4P14	9	5107937	5109290	unprocessed_pseudogene	3,40	0
ENSG00000254388	DUTP2	8	123352181	123352643	processed_pseudogene	3,40	7,06E-02
ENSG00000226045	ENSG00000226045	7	130645225	130645594	processed_pseudogene	3,40	0,2161218
ENSG00000226851	ENSG00000226851	7	112328189	112409623	antisense	3,40	1,40E-02
ENSG00000227113	ENSG00000227113	7	65075023	65078780	unprocessed_pseudogene	3,40	1,40E-02
ENSG00000229677	ENSG00000229677	7	139049456	139050006	processed_pseudogene	3,40	0
ENSG00000272843	ENSG00000272843	7	72924418	72925125	antisense	3,40	0,2161218
ENSG00000217275	ENSG00000217275	6	26202156	26202654	processed_pseudogene	3,40	0
ENSG00000220563	PKMP3	6	85659892	85660606	processed_pseudogene	3,40	7,06E-02
ENSG00000271821	ENSG00000271821	6	31200165	31201918	lincRNA	3,40	1,40E-02
ENSG00000250347	ENSG00000250347	5	142036707	142037125	processed_pseudogene	3,40	0,2161218

ENSG00000250892	ENSG00000250892	4	201409	205009	unprocessed_pseudogene	3,40	0
ENSG00000271653	ENSG00000271653	3	37216779	37217988	lincRNA	3,40	0,2161218
ENSG00000225062	CATIP-AS1	2	218366665	218367835	processed_transcript	3,40	0,2161218
ENSG00000225166	ENSG00000225166	2	215453707	215463871	lincRNA	3,40	7,06E-02
ENSG00000230638	ENSG00000230638	1	41542069	41544310	processed_pseudogene	3,40	0
ENSG00000232208	ENSG00000232208	1	8907393	8907744	processed_pseudogene	3,40	1,40E-02
ENSG00000232453	ENSG00000232453	1	58882868	58903747	lincRNA	3,40	0,2161218
ENSG00000237094	ENSG00000237094	1	365389	501617	lincRNA	3,40	0
ENSG00000240929	HIST2H2BB	1	143875171	143904650	transcribed_unprocessed_pseudogene	3,40	0
ENSG00000280195	ENSG00000280195	X	154396878	154398816	antisense	3,40	0
ENSG00000176761	ZNF285B	19	44467641	44473227	unprocessed_pseudogene	3,38	0,0358681
ENSG00000265490	ENSG00000265490	18	706523	707648	antisense	3,38	0
ENSG00000225302	ENSG00000225302	10	116828761	116850205	antisense	3,38	0
ENSG00000260616	ENSG00000260616	16	50783859	50803338	antisense	3,38	6,27E-03
ENSG00000160172	FAM86C2P	11	67791648	67805336	transcribed_unprocessed_pseudogene	3,37	0
ENSG00000273027	ENSG00000273027	21	44929653	44930112	sense_intronic	3,36	0,0358681
ENSG00000267681	ENSG00000267681	17	43144956	43145255	transcribed_processed_pseudogene	3,36	0,1392851
ENSG00000234193	ENSG00000234193	2	222917387	222919363	antisense	3,36	0,0358681
ENSG00000254604	ENSG00000254604	11	70282367	70363368	antisense	3,36	0
ENSG00000281912	LINC01144	1	45303910	45305619	lincRNA	3,36	0,2161218
ENSG00000215179	MAPK6PS4	8	46972476	46974617	processed_pseudogene	3,35	1,40E-02
ENSG00000231721	LINC-PINT	7	130941760	131110176	antisense	3,34	6,27E-03
ENSG00000184319	RPL23AP82	22	50756948	50801309	transcribed_unprocessed_pseudogene	3,34	0
ENSG00000212789	ST13P5	11	18261982	18263091	processed_pseudogene	3,34	0
ENSG00000255100	ENSG00000255100	11	76782581	76783062	antisense	3,33	0,1836819
ENSG00000273133	ENSG00000273133	4	15563698	15564253	antisense	3,33	6,27E-03
ENSG00000234390	USP27X-AS1	X	49876724	49879241	lincRNA	3,33	0
ENSG00000223345	HIST2H2BA	1	121108210	121117257	transcribed_unprocessed_pseudogene	3,33	0
ENSG00000233527	ZNF529-AS1	19	36573070	36594708	antisense	3,31	0,1217903
ENSG00000224781	EIF4A2P4	X	52832704	52833899	processed_pseudogene	3,31	0,3318209
ENSG00000253948	ENSG00000253948	8	98996763	99013044	lincRNA	3,31	0,1392851
ENSG00000273090	ENSG00000273090	2	36513255	36513732	antisense	3,31	0
ENSG00000183535	COL18A1-AS1	21	45419716	45425070	antisense	3,30	1,40E-02
ENSG00000237338	FTCD-AS1	21	46151614	46152647	antisense	3,30	1,40E-02
ENSG00000268433	ENSG00000268433	19	21677550	21678221	processed_pseudogene	3,30	0,2161218
ENSG00000267560	ENSG00000267560	18	62300036	62300998	antisense	3,30	0
ENSG00000260369	ENSG00000260369	17	80453735	80454729	lincRNA	3,30	0,2161218
ENSG00000262248	ENSG00000262248	17	3619256	3619913	processed_pseudogene	3,30	7,06E-02
ENSG00000265206	ENSG00000265206	17	58330884	58332508	antisense	3,30	0
ENSG00000267340	ENSG00000267340	17	43169880	43170077	processed_pseudogene	3,30	7,06E-02
ENSG00000260022	ENSG00000260022	16	883780	885090	antisense	3,30	0
ENSG00000260468	LINC01290	16	10514842	10528202	lincRNA	3,30	1,40E-02
ENSG00000262703	ENSG00000262703	16	11348143	11349321	antisense	3,30	0,2161218
ENSG00000260919	ENSG00000260919	15	74613194	74615596	antisense	3,30	1,40E-02
ENSG00000224743	TEX26-AS1	13	30881933	30933846	antisense	3,30	7,06E-02
ENSG00000275409	ENSG00000275409	12	118430147	118430699	lincRNA	3,30	0,2161218
ENSG00000254509	ENSG00000254509	11	124791228	124791660	processed_pseudogene	3,30	0
ENSG00000227374	ENSG00000227374	10	129768844	129769435	lincRNA	3,30	0
ENSG00000234311	ENSG00000234311	10	132433733	132441484	antisense	3,30	0,2161218
ENSG00000237840	FAM21FP	10	45706431	45727231	transcribed_unprocessed_pseudogene	3,30	0
ENSG00000224809	BEND3P2	9	92171241	92173494	processed_pseudogene	3,30	0,2161218
ENSG00000226237	ENSG00000226237	9	86948699	87002033	lincRNA	3,30	1,40E-02
ENSG00000229029	CDCA4P1	9	14593577	14594200	processed_pseudogene	3,30	7,06E-02

ENSG00000261215	ENSG00000261215	9	34661903	34666029	processed_transcript	3,30	0,2161218
ENSG00000237586	ENSG00000237586	8	124301315	124301804	processed_pseudogene	3,30	0,2161218
ENSG00000226002	GTF2IP14	7	65084103	65100232	unprocessed_pseudogene	3,30	0,2161218
ENSG00000230825	ENSG00000230825	7	7255154	7277779	processed_transcript	3,30	7,06E-02
ENSG00000239486	ENSG00000239486	7	102380465	102382737	unprocessed_pseudogene	3,30	7,06E-02
ENSG00000254035	ENSG00000254035	5	178969390	178990116	antisense	3,30	0
ENSG00000271871	ENSG00000271871	5	141952419	141953375	antisense	3,30	0,2161218
ENSG00000248161	ENSG00000248161	4	102418602	102450010	lincRNA	3,30	0,2161218
ENSG00000248479	ENSG00000248479	4	65702202	65705553	lincRNA	3,30	7,06E-02
ENSG00000228446	ENSG00000228446	2	224499387	224500100	processed_pseudogene	3,30	7,06E-02
ENSG00000230646	KLF2P2	2	130427731	130429225	processed_pseudogene	3,30	0
ENSG00000220323	HIST2H2BD	1	149843041	149843533	processed_pseudogene	3,30	1,40E-02
ENSG00000228477	ENSG00000228477	1	39962680	39963404	processed_pseudogene	3,30	1,40E-02
ENSG00000237934	ENSG00000237934	1	29223933	29224816	antisense	3,30	0
ENSG00000273175	ENSG00000273175	1	244864738	244865272	lincRNA	3,30	7,06E-02
ENSG00000237341	SYP-AS1	X	49198966	49202454	antisense	3,30	1,40E-02
ENSG00000245311	ARNTL2-AS1	12	27389789	27446625	antisense	3,29	0
ENSG00000231856	ENSG00000231856	13	51803838	51813832	antisense	3,29	0
ENSG00000258072	ENSG00000258072	12	107903270	107903948	processed_pseudogene	3,29	4,86E-02
ENSG00000224614	TNK2-AS1	3	195908076	195911257	antisense	3,29	7,06E-02
ENSG00000154874	CCDC144B	17	18537800	18625617	transcribed_unprocessed_pseudogene	3,28	0
ENSG00000271868	ENSG00000271868	2	3496956	3497428	lincRNA	3,28	0,1217903
ENSG00000183154	ENSG00000183154	8	37734761	37737426	antisense	3,28	0
ENSG00000231889	TRAF3IP2-AS1	6	111483511	111598302	antisense	3,28	6,27E-03
ENSG00000224316	GTF2IP5	7	65773620	65802067	unprocessed_pseudogene	3,27	0,1836819
ENSG00000267549	ENSG00000267549	19	56314703	56341287	antisense	3,26	1,40E-02
ENSG00000260929	ENSG00000260929	16	50740910	50742815	antisense	3,26	0
ENSG00000259295	CSPG4P12	15	85191438	85213905	transcribed_unprocessed_pseudogene	3,26	9,18E-02
ENSG00000270157	ENSG00000270157	7	141662922	141663846	sense_intronic	3,26	0
ENSG00000213304	ENSG00000213304	19	11521968	11522498	processed_pseudogene	3,25	0,0358681
ENSG00000251015	SLC25A30-AS1	13	45418162	45420371	lincRNA	3,25	0
ENSG00000260588	ENSG00000260588	8	41828165	41829934	lincRNA	3,25	0
ENSG00000263766	ENSG00000263766	17	47603860	47649420	antisense	3,25	0
ENSG00000186594	MIR22HG	17	1711493	1717174	lincRNA	3,24	4,86E-02
ENSG00000228113	ENSG00000228113	7	88219359	88304367	antisense	3,23	9,18E-02
ENSG00000267871	ENSG00000267871	19	57267220	57280334	antisense	3,23	0,2161218
ENSG00000260339	HEXA-AS1	15	72376113	72378788	antisense	3,23	0
ENSG00000234076	TPRG1-AS1	3	188941715	188947639	lincRNA	3,23	6,27E-03
ENSG00000238287	ENSG00000238287	1	40493157	40508661	antisense	3,23	0,1392851
ENSG00000257225	ENSG00000257225	12	42459366	42466128	antisense	3,23	0
ENSG00000264575	LINC00526	18	5236724	5238598	lincRNA	3,23	1,40E-02
ENSG00000227581	ENSG00000227581	X	90039213	90039834	processed_pseudogene	3,23	3,30E-03
ENSG00000214992	AKAP17BP	X	119249346	119257667	unprocessed_pseudogene	3,23	0
ENSG00000166770	ZNF667-AS1	19	56477250	56500666	lincRNA	3,22	3,30E-03
ENSG00000248785	HIGD1AP14	4	109673843	109674124	processed_pseudogene	3,22	0
ENSG00000261183	ENSG00000261183	15	40835808	40844387	antisense	3,21	0,2161218
ENSG00000250990	ENSG00000250990	7	77246340	77258123	antisense	3,21	0,4332376
ENSG00000234608	MAPKAPK5-AS1	12	111839764	111842902	lincRNA	3,20	2,21E-02
ENSG00000241954	ENSG00000241954	22	32383786	32385631	lincRNA	3,20	1,40E-02
ENSG00000277232	GTSE1-AS1	22	46295143	46296660	lincRNA	3,20	7,06E-02
ENSG00000233056	ERVH48-1	21	42916803	42925646	lincRNA	3,20	0
ENSG00000228663	PSMD10P1	20	50913731	50914411	processed_pseudogene	3,20	0,2161218
ENSG00000265933	LINC00668	18	6919496	6929966	lincRNA	3,20	0
ENSG00000237911	SRP68P3	17	20415792	20416697	unprocessed_pseudogene	3,20	1,40E-02

ENSG00000259349	ENSG00000259349	17	60526293	60550798	antisense	3,20	1,40E-02
ENSG00000274341	ENSG00000274341	17	32408013	32408309	lincRNA	3,20	7,06E-02
ENSG00000278668	ENSG00000278668	17	32410159	32410746	lincRNA	3,20	0,2161218
ENSG00000259209	ENSG00000259209	16	72805998	72809872	antisense	3,20	0
ENSG00000260932	ENSG00000260932	16	83929796	83931223	antisense	3,20	0
ENSG00000261386	ENSG00000261386	16	67549214	67563958	lincRNA	3,20	0,2161218
ENSG00000261513	ENSG00000261513	16	71723180	71724230	antisense	3,20	0
ENSG00000273551	ENSG00000273551	16	1148224	1148754	lincRNA	3,20	7,06E-02
ENSG00000277639	ENSG00000277639	16	53043066	53044216	lincRNA	3,20	0,2161218
ENSG00000244952	ENSG00000244952	15	32613733	32615111	lincRNA	3,20	0
ENSG00000273771	ENSG00000273771	15	96080713	96084584	lincRNA	3,20	7,06E-02
ENSG00000259502	ENSG00000259502	14	67610986	67613864	processed_pseudogene	3,20	1,40E-02
ENSG00000262119	ENSG00000262119	14	26592747	26594517	sense_intronic	3,20	1,40E-02
ENSG00000275481	ENSG00000275481	12	46388856	46392126	lincRNA	3,20	7,06E-02
ENSG00000255328	ENSG00000255328	11	327171	330122	lincRNA	3,20	0,2161218
ENSG00000261340	LINC01616	11	29980113	29982392	lincRNA	3,20	0,2161218
ENSG00000226318	ENSG00000226318	10	67960702	67961464	processed_pseudogene	3,20	1,40E-02
ENSG00000228651	ENSG00000228651	10	52556702	52755409	antisense	3,20	7,06E-02
ENSG00000272791	ENSG00000272791	10	73630556	73631490	lincRNA	3,20	7,06E-02
ENSG00000273012	ENSG00000273012	10	33341655	33341905	lincRNA	3,20	0,2161218
ENSG00000223621	AK4P4	9	5855781	5856426	processed_pseudogene	3,20	7,06E-02
ENSG00000227666	CYCSP24	9	94038030	94038345	processed_pseudogene	3,20	0
ENSG00000237212	ENSG00000237212	9	96095211	96101912	lincRNA	3,20	7,06E-02
ENSG00000237357	ENSG00000237357	9	42566679	42569353	lincRNA	3,20	0
ENSG00000273186	ENSG00000273186	9	128431598	128432006	sense_intronic	3,20	0,2161218
ENSG00000231881	ENSG00000231881	6	44073913	44077952	lincRNA	3,20	0,2161218
ENSG00000244349	HCG16	6	28986800	28988536	antisense	3,20	1,40E-02
ENSG00000261003	ENSG00000261003	6	170414139	170419325	sense_overlapping	3,20	7,06E-02
ENSG00000248511	ENSG00000248511	4	92297251	92304178	lincRNA	3,20	0,2161218
ENSG00000236409	NRADDP	3	47011542	47013146	unitary_pseudogene	3,20	1,40E-02
ENSG00000272263	ENSG00000272263	3	12832219	12832728	antisense	3,20	7,06E-02
ENSG00000223884	ENSG00000223884	2	7062727	7077880	antisense	3,20	0,2161218
ENSG00000226791	ENSG00000226791	2	98761938	98772920	lincRNA	3,20	7,06E-02
ENSG00000233155	HMGA1P8	2	74418122	74418442	processed_pseudogene	3,20	0
ENSG00000271228	ENSG00000271228	2	32013061	32013368	antisense	3,20	7,06E-02
ENSG00000221953	C1orf229	1	247110160	247112417	lincRNA	3,20	1,40E-02
ENSG00000224014	ENSG00000224014	1	247175256	247175525	processed_pseudogene	3,20	0
ENSG00000224751	SHMT1P1	1	43850300	43851611	processed_pseudogene	3,20	0,2161218
ENSG00000227811	FAM212B-AS1	1	111739841	111747798	antisense	3,20	1,40E-02
ENSG00000260464	ENSG00000260464	1	93847174	93848939	lincRNA	3,20	7,06E-02
ENSG00000270110	ENSG00000270110	1	228295911	228302998	antisense	3,20	7,06E-02
ENSG00000272482	ENSG00000272482	1	12618900	12619244	lincRNA	3,20	0,2161218
ENSG00000272982	ENSG00000272982	1	111181374	111181491	antisense	3,20	0
ENSG00000234230	ZFX-AS1	X	24146225	24149654	antisense	3,20	7,06E-02
ENSG00000242562	DCAF13P1	7	106125371	106126700	processed_pseudogene	3,20	0,0358681
ENSG00000205959	ENSG00000205959	4	8482270	8512610	lincRNA	3,20	7,06E-02
ENSG00000258057	BCDIN3D-AS1	12	49828542	49841143	antisense	3,20	0
ENSG00000215349	MRPL3P1	13	19304593	19305625	processed_pseudogene	3,20	6,27E-03
ENSG00000250903	GMDS-AS1	6	2245748	2482022	lincRNA	3,19	0
ENSG00000269293	ZSCAN16-AS1	6	28121795	28137293	antisense	3,19	2,21E-02
ENSG00000236411	NDUFAF4P3	3	37789921	37790445	processed_pseudogene	3,19	3,30E-03
ENSG00000278719	MCM8-AS1	20	5990943	6005821	antisense	3,18	0,0358681
ENSG00000271662	ENSG00000271662	3	195650146	195651472	processed_pseudogene	3,18	0,0358681
ENSG00000241494	ENSG00000241494	14	101677943	101678380	processed_pseudogene	3,18	0,1836819
ENSG00000232586	KIAA1614-AS1	1	180949699	180954887	antisense	3,16	1,95E-02
ENSG00000260405	ENSG00000260405	16	17096202	17096421	processed_pseudogene	3,15	7,06E-02
ENSG00000197670	ENSG00000197670	20	53552770	53575863	antisense	3,15	0
ENSG00000231095	GYG1P3	1	55222379	55223372	processed_pseudogene	3,14	7,06E-02
ENSG00000250896	RNPS1P1	4	11371975	11372892	processed_pseudogene	3,14	0
ENSG00000187988	KCTD9P3	9	37477149	37478317	processed_pseudogene	3,13	0
ENSG00000225261	ENSG00000225261	X	149548210	149549835	antisense	3,13	1,95E-02

ENSG00000277978	ENSG00000277978	16	66565620	66566001	antisense	3,11	0,1836819
ENSG00000271870	ENSG00000271870	3	3152942	3153435	antisense	3,10	6,27E-03
ENSG00000232186	ENSG00000232186	X	75326772	75327999	processed_pseudogene	3,10	0
ENSG00000229999	ENSG00000229999	22	40372664	40388290	antisense	3,10	0,2161218
ENSG00000231933	ENSG00000231933	22	25876854	25903247	antisense	3,10	7,06E-02
ENSG00000231302	RPL36P2	20	50620763	50621077	processed_pseudogene	3,10	0,2161218
ENSG00000276603	ENSG00000276603	20	37526642	37527060	sense_intronic	3,10	1,40E-02
ENSG00000231561	CEACAMP5	19	42654200	42669076	unprocessed_pseudogene	3,10	0
ENSG00000267056	ENSG00000267056	19	15910582	15911824	processed_pseudogene	3,10	0
ENSG00000267274	ENSG00000267274	19	11939959	11953381	sense_overlapping	3,10	1,40E-02
ENSG00000267498	ENSG00000267498	19	29213235	29215815	lincRNA	3,10	0
ENSG00000269053	ENSG00000269053	19	17419305	17419774	antisense	3,10	1,40E-02
ENSG00000264269	ENSG00000264269	18	49023703	49048474	antisense	3,10	1,40E-02
ENSG00000259212	ENSG00000259212	15	90595840	90596447	antisense	3,10	0
ENSG00000259358	ENSG00000259358	15	31765743	31768201	processed_pseudogene	3,10	7,06E-02
ENSG00000260211	ENSG00000260211	15	32313126	32315654	processed_pseudogene	3,10	1,40E-02
ENSG00000259138	ENSG00000259138	14	75127153	75136930	antisense	3,10	0
ENSG00000255566	ENSG00000255566	12	64442510	64443330	processed_pseudogene	3,10	1,40E-02
ENSG00000257752	ENSG00000257752	12	88057876	88058578	processed_pseudogene	3,10	7,06E-02
ENSG00000251323	ENSG00000251323	11	78423982	78429836	lincRNA	3,10	1,40E-02
ENSG00000224745	ENSG00000224745	10	87878692	87880427	sense_intronic	3,10	7,06E-02
ENSG00000227730	MTND6P5	9	5091093	5091604	unprocessed_pseudogene	3,10	7,06E-02
ENSG00000236717	ENSG00000236717	9	92150382	92157445	lincRNA	3,10	7,06E-02
ENSG00000246792	ENSG00000246792	8	90646441	90656598	antisense	3,10	0
ENSG00000189316	ENSG00000189316	7	64888527	64890100	antisense	3,10	1,40E-02
ENSG00000227014	ENSG00000227014	7	29988600	30025660	antisense	3,10	7,06E-02
ENSG00000232716	ENSG00000232716	7	130840204	130843725	processed_pseudogene	3,10	7,06E-02
ENSG00000235253	ENSG00000235253	5	132942932	132945050	processed_pseudogene	3,10	0,2161218
ENSG00000241112	RPL29P14	5	149545383	149545810	processed_pseudogene	3,10	1,40E-02
ENSG00000262096	PCDHB19P	5	141239923	141243034	transcribed_unprocessed_pseudogene	3,10	0,2161218
ENSG00000272144	ENSG00000272144	5	43061395	43062441	lincRNA	3,10	0,2161218
ENSG00000248174	ENSG00000248174	4	174094660	174220398	lincRNA	3,10	7,06E-02
ENSG00000248196	ENSG00000248196	4	87166844	87167734	processed_pseudogene	3,10	0,2161218
ENSG00000231464	ENSG00000231464	3	195996738	195998233	processed_pseudogene	3,10	1,40E-02
ENSG00000239482	ENSG00000239482	3	112302478	112332791	lincRNA	3,10	1,40E-02
ENSG00000271973	ENSG00000271973	3	48672455	48672733	lincRNA	3,10	7,06E-02
ENSG00000225991	RPL23AP34	2	28308161	28308570	processed_pseudogene	3,10	0,2161218
ENSG00000272716	ENSG00000272716	2	32165046	32165757	lincRNA	3,10	7,06E-02
ENSG00000225704	ENSG00000225704	1	171345089	171345320	processed_pseudogene	3,10	7,06E-02
ENSG00000229914	ENSG00000229914	1	158195633	158196131	processed_pseudogene	3,10	7,06E-02
ENSG00000233421	ENSG00000233421	1	16533886	16536172	lincRNA	3,10	7,06E-02
ENSG00000236546	ENSG00000236546	1	39897745	39899098	antisense	3,10	1,40E-02
ENSG00000213997	PGAM1P7	X	46646594	46647330	processed_pseudogene	3,10	1,40E-02
ENSG00000237351	ENSG00000237351	X	47156638	47158199	processed_pseudogene	3,10	7,06E-02
ENSG00000279392	ENSG00000279392	14	55229217	55229681	processed_pseudogene	3,10	3,30E-03
ENSG00000267567	ENSG00000267567	19	32718298	32719595	lincRNA	3,09	0,4332376
ENSG00000277977	ENSG00000277977	19	52392659	52392755	unprocessed_pseudogene	3,09	0,4332376
ENSG00000260213	ENSG00000260213	16	81016792	81035759	antisense	3,08	0,1836819
ENSG00000255571	MIR9-3HG	15	89361579	89398487	lincRNA	3,08	0,2161218
ENSG00000254934	LINC00678	11	27617626	27634627	lincRNA	3,08	0,1836819
ENSG00000214559	ENSG00000214559	4	98251688	98261630	antisense	3,08	0
ENSG00000242899	RPL7P16	3	132243528	132244265	processed_pseudogene	3,08	0,0358681
ENSG00000273486	ENSG00000273486	3	136837338	136839021	antisense	3,08	0
ENSG00000226086	EIF3LP3	10	38080056	38080877	processed_pseudogene	3,08	0
ENSG00000236299	ENSG00000236299	7	63888200	63900794	lincRNA	3,08	0
ENSG00000225489	ENSG00000225489	9	6716495	6727438	lincRNA	3,07	4,86E-02
ENSG00000266601	ENSG00000266601	17	47929682	47933106	antisense	3,06	1,40E-02

ENSG00000149531	FRG1BP	20	30377372	30399257	unprocessed_pseudogene	3,05	0
ENSG00000279006	ENSG00000279006	22	25048967	25051966	antisense	3,05	0
ENSG00000272146	ARF4-AS1	3	57597715	57600927	antisense	3,05	0,1836819
ENSG00000267724	ENSG00000267724	18	47105946	47108062	antisense	3,04	2,21E-02
ENSG00000234618	RPSAP9	9	76398699	76399586	processed_pseudogene	3,04	0
ENSG00000263846	CIAPIN1P	18	26599408	26600338	processed_pseudogene	3,04	3,30E-03
ENSG00000263826	ENSG00000263826	3	186781780	186784179	antisense	3,02	0
ENSG00000225137	DYNC1I2P1	10	50264978	50266815	processed_pseudogene	3,02	0
ENSG00000228348	EFCAB14P1	1	35122022	35122484	processed_pseudogene	3,02	0
ENSG00000235513	ENSG00000235513	22	41209122	41217627	antisense	3,01	0
ENSG00000111788	ENSG00000111788	12	9277235	9313241	unprocessed_pseudogene	3,01	3,30E-03
ENSG00000204934	ATP6V0E2-AS1	7	149867697	149880610	antisense	3,01	0,2161218
ENSG00000270294	ENSG00000270294	10	109680442	109681041	processed_pseudogene	3,01	0
ENSG00000245910	SNHG6	8	66921684	66926398	processed_transcript	3,00	0,1392851
ENSG00000249310	APOBEC3B-AS1	22	38991559	38998209	antisense	3,00	2,21E-02
ENSG00000273464	ENSG00000273464	21	29657406	29657831	antisense	3,00	0,2161218
ENSG00000278041	ENSG00000278041	20	21499261	21502934	lincRNA	3,00	0,2161218
ENSG00000261754	ENSG00000261754	19	34811589	34814345	lincRNA	3,00	7,06E-02
ENSG00000266941	ENSG00000266941	19	5899895	5901511	antisense	3,00	2,95E-02
ENSG00000266963	ENSG00000266963	19	38361795	38362484	antisense	3,00	7,06E-02
ENSG00000268496	ENSG00000268496	19	54434183	54434665	sense_intronic	3,00	0,2161218
ENSG00000269091	ENSG00000269091	19	50043196	50051062	antisense	3,00	0
ENSG00000271499	ENSG00000271499	19	19750618	19752544	processed_pseudogene	3,00	0
ENSG00000264339	ENSG00000264339	18	738058	739444	antisense	3,00	1,40E-02
ENSG00000260034	LCMT1-AS2	16	25140577	25149032	antisense	3,00	6,27E-03
ENSG00000261172	ENSG00000261172	16	90110574	90168225	lincRNA	3,00	0
ENSG00000276571	ENSG00000276571	16	19694035	19694428	antisense	3,00	1,40E-02
ENSG00000260814	ENSG00000260814	15	41770756	41772732	lincRNA	3,00	0,0358681
ENSG00000237356	ENSG00000237356	14	53169054	53687258	processed_transcript	3,00	0
ENSG00000258569	ENSG00000258569	14	76710658	76761388	lincRNA	3,00	7,06E-02
ENSG00000258689	LINC01269	14	70698698	70712153	lincRNA	3,00	0,2161218
ENSG00000231530	ENSG00000231530	13	42043727	42044247	antisense	3,00	7,06E-02
ENSG00000277831	ENSG00000277831	13	44369365	44369877	lincRNA	3,00	0,2161218
ENSG00000242661	RPS3AP43	12	8359054	8359834	processed_pseudogene	3,00	0,2161218
ENSG00000248265	ENSG00000248265	12	54058254	54122234	lincRNA	3,00	7,06E-02
ENSG00000257097	CLIP1-AS1	12	122395542	122400857	lincRNA	3,00	6,27E-03
ENSG00000271963	ENSG00000271963	12	133115692	133122340	antisense	3,00	0
ENSG00000229512	ENSG00000229512	11	1763009	1763749	sense_intronic	3,00	0
ENSG00000241782	ENSG00000241782	11	77868722	77869195	processed_pseudogene	3,00	0,2161218
ENSG00000254739	ENSG00000254739	11	528907	529659	antisense	3,00	0,2161218
ENSG00000233435	AGGF1P2	10	133640789	133642894	processed_pseudogene	3,00	7,06E-02
ENSG00000251079	BMS1P2	10	47538377	47551954	transcribed_unprocessed_pseudogene	3,00	7,06E-02
ENSG00000271981	ENSG00000271981	10	22332587	22332981	lincRNA	3,00	0,2161218
ENSG00000230537	ENSG00000230537	9	91104957	91107304	lincRNA	3,00	7,06E-02
ENSG00000236083	OR13E1P	9	35859062	35859983	unitary_pseudogene	3,00	0
ENSG00000275549	C9orf173-AS1	9	137250219	137253497	antisense	3,00	0,2161218
ENSG00000253549	CA3-AS1	8	85441851	85464915	antisense	3,00	0,2161218
ENSG00000253931	ENSG00000253931	8	143412749	143417054	antisense	3,00	0,2161218
ENSG00000231892	ENSG00000231892	7	3196626	3198256	lincRNA	3,00	7,06E-02
ENSG00000219470	ENSG00000219470	6	43538822	43539703	processed_pseudogene	3,00	2,21E-02
ENSG00000233902	ENSG00000233902	6	31462728	31463336	unprocessed_pseudogene	3,00	0
ENSG00000230612	ENSG00000230612	5	132688681	132723725	antisense	3,00	7,06E-02
ENSG00000277192	ENSG00000277192	5	132488382	132488702	antisense	3,00	0
ENSG00000226155	ENSG00000226155	3	195912049	195913986	antisense	3,00	0,2161218
ENSG00000240032	ENSG00000240032	3	146066344	146069185	antisense	3,00	7,06E-02
ENSG00000244515	KRT18P34	3	157162663	157163932	processed_pseudogene	3,00	7,06E-02
ENSG00000272483	ENSG00000272483	3	11611602	11612197	antisense	3,00	0,2161218

ENSG00000223935	ENSG00000223935	2	64395220	64453798	antisense	3,00	1,40E-02
ENSG00000224337	FAM8A3P	2	149320593	149321436	processed_pseudogene	3,00	0
ENSG00000266931	ENSG00000266931	2	87055658	87055800	unprocessed_pseudogene	3,00	0
ENSG00000225315	ENSG00000225315	1	24040835	24086799	antisense	3,00	0,2161218
ENSG00000225920	RIMKLBP2	1	219199914	219200567	processed_pseudogene	3,00	0
ENSG00000229739	ENSG00000229739	1	186435161	186470291	antisense	3,00	0,2161218
ENSG00000230092	ENSG00000230092	1	800879	817712	transcribed_unprocessed_pseudogene	3,00	3,30E-03
ENSG00000231979	ENSG00000231979	1	239915439	239916955	lincRNA	3,00	0,2161218
ENSG00000232879	ENSG00000232879	1	161034834	161035006	processed_pseudogene	3,00	7,06E-02
ENSG00000270585	ENSG00000270585	1	220207618	220208282	processed_pseudogene	3,00	7,06E-02
ENSG00000227329	ENSG00000227329	X	53164391	53164557	processed_pseudogene	3,00	0
ENSG00000233984	RPSAP14	X	74031462	74031820	processed_pseudogene	3,00	0
ENSG00000224660	SH3BP5-AS1	3	15254184	15264493	antisense	3,00	0,3318209
ENSG00000267776	ENSG00000267776	19	56376704	56377284	sense_intronic	2,99	0
ENSG00000236869	ENSG00000236869	3	44557357	44685653	antisense	2,98	0
ENSG00000253334	ENSG00000253334	8	81791823	81815714	antisense	2,97	6,27E-03
ENSG00000266002	ENSG00000266002	17	59400488	59403303	antisense	2,97	6,27E-03
ENSG00000259322	ENSG00000259322	15	78293286	78296049	antisense	2,97	0
ENSG00000231799	ENSG00000231799	9	89449558	89450754	processed_pseudogene	2,97	2,21E-02
ENSG00000228451	SDAD1P1	8	26379259	26382953	transcribed_processed_pseudogene	2,96	1,40E-02
ENSG00000258568	RHOQP1	14	49599994	49600572	processed_pseudogene	2,96	3,30E-03
ENSG00000233382	NKAPP1	X	120120953	120245267	transcribed_processed_pseudogene	2,96	0
ENSG00000258090	ENSG00000258090	12	76757191	76757914	processed_pseudogene	2,96	7,06E-02
ENSG00000225975	LINC01534	19	36685440	36687449	lincRNA	2,96	6,27E-03
ENSG00000259093	ENSG00000259093	14	63123033	63128214	lincRNA	2,95	6,27E-03
ENSG00000255992	ENSG00000255992	12	131924736	131929351	lincRNA	2,95	0
ENSG00000227040	ENSG00000227040	4	56760919	56761485	processed_pseudogene	2,94	9,18E-02
ENSG00000214280	ENSG00000214280	3	139582928	139583593	processed_pseudogene	2,94	3,30E-03
ENSG00000225670	CADM3-AS1	1	159194325	159207973	antisense	2,93	0,4036702
ENSG00000223396	ENSG00000223396	1	201518703	201530474	lincRNA	2,93	6,27E-03
ENSG00000220804	ENSG00000220804	2	242088633	242160153	transcribed_unprocessed_pseudogene	2,92	0
ENSG00000261143	ADAMTS7P3	15	77976042	77993057	transcribed_unprocessed_pseudogene	2,92	0,4332376
ENSG00000243024	RPS11P6	12	64222337	64397065	transcribed_processed_pseudogene	2,92	2,21E-02
ENSG00000246308	ENSG00000246308	11	10809297	10822931	antisense	2,92	0,3318209
ENSG00000250412	KLHL2P1	4	119334329	119378233	unprocessed_pseudogene	2,92	0
ENSG00000235358	ENSG00000235358	1	41242373	41284861	lincRNA	2,92	0
ENSG00000230071	RPL4P6	22	40586700	40588003	processed_pseudogene	2,92	3,30E-03
ENSG00000231749	ABCA9-AS1	17	68944531	69042784	antisense	2,91	3,30E-03
ENSG00000228839	PIK3IP1-AS1	22	31292499	31338021	antisense	2,91	0
ENSG00000263335	ENSG00000263335	16	15726674	15732993	antisense	2,91	6,27E-03
ENSG00000205662	ENSG00000205662	X	3929869	3937855	lincRNA	2,91	0,4332376
ENSG00000231458	RNASEH1P2	17	16683438	16684227	processed_pseudogene	2,91	0
ENSG00000224490	TTC21B-AS1	2	165933857	165949891	processed_transcript	2,90	0
ENSG00000232581	ENSG00000232581	7	6578565	6588974	antisense	2,90	0,3318209
ENSG00000236213	ENSG00000236213	2	37600136	37646698	antisense	2,90	4,86E-02
ENSG00000234380	LINC01426	21	34745757	34784886	antisense	2,90	0,2161218
ENSG00000267197	ENSG00000267197	19	10490339	10491000	sense_intronic	2,90	0,2161218
ENSG00000270679	ENSG00000270679	19	44290081	44290911	processed_pseudogene	2,90	0,2161218
ENSG00000264745	TTC39C-AS1	18	23994213	24015339	antisense	2,90	7,06E-02
ENSG00000267543	ENSG00000267543	17	76549951	76550826	sense_intronic	2,90	1,40E-02
ENSG00000259711	ENSG00000259711	16	54934913	54954665	lincRNA	2,90	7,06E-02
ENSG00000260495	ENSG00000260495	16	81310731	81313423	antisense	2,90	0,2161218
ENSG00000261390	MAFTRR	16	79715232	79770563	lincRNA	2,90	0,2161218

ENSG00000259656	ENSG00000259656	15	49101791	49103264	lincRNA	2,90	1,40E-02
ENSG00000258283	ENSG00000258283	12	48995150	48996334	antisense	2,90	7,06E-02
ENSG00000189014	FAM35DP	10	47689707	47730436	unprocessed_pseudogene	2,90	1,40E-02
ENSG00000226578	ENSG00000226578	10	37775371	37784131	lincRNA	2,90	1,40E-02
ENSG00000228882	CICP9	10	38452987	38455365	processed_pseudogene	2,90	0,2161218
ENSG00000229081	LINC01165	10	132520827	132522449	antisense	2,90	1,40E-02
ENSG00000273980	ENSG00000273980	10	133257144	133257551	lincRNA	2,90	7,06E-02
ENSG00000204778	ENSG00000204778	9	65287914	65323015	unprocessed_pseudogene	2,90	0
ENSG00000227638	HNRNPA1P14	9	88410529	88411463	processed_pseudogene	2,90	0
ENSG00000229065	ENSG00000229065	9	95113708	95114461	antisense	2,90	1,40E-02
ENSG00000261451	ENSG00000261451	8	10433672	10438312	sense_overlapping	2,90	7,06E-02
ENSG00000270131	ENSG00000270131	8	96140572	96140944	lincRNA	2,90	0,2161218
ENSG00000219074	SOD1P1	6	53196720	53197159	processed_pseudogene	2,90	0,2161218
ENSG00000237927	ENSG00000237927	6	159586955	159589169	lincRNA	2,90	7,06E-02
ENSG00000271734	ENSG00000271734	6	108030249	108030718	lincRNA	2,90	7,06E-02
ENSG00000271755	ENSG00000271755	6	27404010	27406964	lincRNA	2,90	7,06E-02
ENSG00000240729	ENSG00000240729	5	177264766	177265241	processed_pseudogene	2,90	0,2161218
ENSG00000249947	XBP1P1	5	112885094	112885745	processed_pseudogene	2,90	7,06E-02
ENSG00000228413	ENSG00000228413	3	196044695	196045254	processed_pseudogene	2,90	0,2161218
ENSG00000229048	DUTP1	3	125310881	125311350	processed_pseudogene	2,90	7,06E-02
ENSG00000242578	ENSG00000242578	3	170410512	170418615	lincRNA	2,90	0,2161218
ENSG00000249540	ENSG00000249540	3	141307612	141307933	processed_pseudogene	2,90	1,40E-02
ENSG00000273308	ENSG00000273308	3	197645669	197646017	lincRNA	2,90	0,2161218
ENSG00000229754	CXCR2P1	2	218059155	218065729	transcribed_unprocessed_pseudogene	2,90	0,2161218
ENSG00000230044	ENSG00000230044	2	11665369	11665969	processed_pseudogene	2,90	0,2161218
ENSG00000230799	ENSG00000230799	2	201762737	201763573	processed_pseudogene	2,90	1,40E-02
ENSG00000231610	ENSG00000231610	2	6495877	6496798	processed_pseudogene	2,90	0,2161218
ENSG00000233718	MYCNOS	2	15921037	15942249	antisense	2,90	1,40E-02
ENSG00000237979	ENSG00000237979	2	65500993	65502138	processed_pseudogene	2,90	0,2161218
ENSG00000224616	RTCA-AS1	1	100264009	100266120	antisense	2,90	0,2161218
ENSG00000281091	ENSG00000281091	X	103404777	103408299	lincRNA	2,90	1,40E-02
ENSG00000225284	ENSG00000225284	2	38861720	38863133	antisense	2,89	0
ENSG00000230337	ENSG00000230337	1	11099675	11102100	antisense	2,89	0
ENSG00000228283	KATNBL1P6	6	146802359	146803824	transcribed_processed_pseudogene	2,89	0
ENSG00000225014	KCTD9P1	17	20111824	20112990	transcribed_processed_pseudogene	2,89	0
ENSG00000213492	NT5C3AP1	4	117574512	117576174	transcribed_processed_pseudogene	2,88	3,30E-03
ENSG00000242208	RPL5P29	11	56357328	56358221	processed_pseudogene	2,88	6,27E-03
ENSG00000257773	ST13P3	12	106015897	106017006	processed_pseudogene	2,87	0,2161218
ENSG00000229785	SLC25A38P1	1	172748560	172749502	processed_pseudogene	2,87	4,86E-02
ENSG00000226971	ENSG00000226971	X	48446893	48448879	processed_pseudogene	2,87	0
ENSG00000236577	SNRPGP14	13	49488970	49489364	processed_pseudogene	2,87	6,27E-03
ENSG00000253431	SRPK2P	8	62863082	62864181	processed_pseudogene	2,87	2,21E-02
ENSG00000270587	ENSG00000270587	17	63610772	63611184	processed_pseudogene	2,86	1,40E-02
ENSG00000242477	ENSG00000242477	5	86884231	86884881	processed_pseudogene	2,86	0,3318209
ENSG00000228818	ENSG00000228818	1	240142670	240145389	processed_pseudogene	2,85	0,4036702
ENSG00000244716	ENSG00000244716	1	108992282	108992836	processed_pseudogene	2,85	0,2161218
ENSG00000231884	NDUFB1P1	3	48181547	48181726	processed_pseudogene	2,84	0,4850164
ENSG00000228439	TSTD3	6	99520693	99531918	antisense	2,84	0
ENSG00000237476	ENSG00000237476	22	20957092	20964679	lincRNA	2,83	4,86E-02
ENSG00000260796	ENSG00000260796	16	28820570	28822033	antisense	2,83	0,1836819
ENSG00000237004	ZNRF2P1	7	32728569	32729011	processed_pseudogene	2,83	1,40E-02
ENSG00000271580	ENSG00000271580	1	205091163	205091946	processed_pseudogene	2,83	0,1836819
ENSG00000233476	EEF1A1P6	7	22510616	22511999	processed_pseudogene	2,83	9,18E-02
ENSG00000263707	ENSG00000263707	17	12990149	12990610	antisense	2,83	6,27E-03
ENSG00000261071	ENSG00000261071	6	13614111	13615155	antisense	2,83	0,3318209

ENSG00000204650	CRHR1-IT1	17	45620328	45655156	processed_transcript	2,83	4,86E-02
ENSG00000205930	C21orf62-AS1	21	32772100	32893735	lincRNA	2,82	6,27E-03
ENSG00000268201	ENSG00000268201	19	58266635	58267685	sense_intronic	2,82	0,1392851
ENSG00000234149	ENSG00000234149	10	75003055	75025174	antisense	2,82	0
ENSG00000226754	ENSG00000226754	1	53238610	53242783	antisense	2,81	0,1217903
ENSG00000231646	FSIP2-AS1	2	185783691	185800151	antisense	2,81	1,95E-02
ENSG00000276564	ENSG00000276564	16	14150833	14153235	lincRNA	2,81	6,27E-03
ENSG00000259790	ANP32BP1	15	75321397	75322693	transcribed_processed_pseudogene	2,80	2,21E-02
ENSG00000258995	ENSG00000258995	14	63123007	63123201	processed_pseudogene	2,80	3,30E-03
ENSG00000180279	ENSG00000180279	19	50808874	50818880	lincRNA	2,80	0,2161218
ENSG00000235681	ENSG00000235681	19	54446830	54447047	processed_pseudogene	2,80	0,2161218
ENSG00000268297	CLEC4GP1	19	7787549	7790621	unprocessed_pseudogene	2,80	1,40E-02
ENSG00000270802	ENSG00000270802	19	15338948	15339237	processed_pseudogene	2,80	7,06E-02
ENSG00000235028	HMGN1P30	18	58018794	58019061	processed_pseudogene	2,80	1,40E-02
ENSG00000187013	C17orf82	17	61411751	61413280	lincRNA	2,80	0,2161218
ENSG00000224254	MTCO1P39	17	19605454	19605933	unprocessed_pseudogene	2,80	0,2161218
ENSG00000262652	ENSG00000262652	17	82578023	82578616	sense_intronic	2,80	1,40E-02
ENSG00000264057	ENSG00000264057	17	64927634	64934203	transcribed_unprocessed_pseudogene	2,80	0
ENSG00000265460	ENSG00000265460	17	39238466	39250818	transcribed_processed_pseudogene	2,80	0,2161218
ENSG00000267048	ENSG00000267048	16	89906157	89918233	processed_transcript	2,80	1,40E-02
ENSG00000275445	ENSG00000275445	16	22007480	22008062	antisense	2,80	7,06E-02
ENSG00000259767	ENSG00000259767	15	83022236	83024336	sense_intronic	2,80	0,2161218
ENSG00000260361	ENSG00000260361	15	92779757	92781492	sense_intronic	2,80	7,06E-02
ENSG00000276702	ENSG00000276702	15	33850538	33851178	antisense	2,80	7,06E-02
ENSG00000228444	ENSG00000228444	13	75631271	75632135	sense_intronic	2,80	7,06E-02
ENSG00000236520	GPC6-AS1	13	94154193	94187991	antisense	2,80	0,2161218
ENSG00000238025	ZDHHC4P1	13	42933858	42934892	processed_pseudogene	2,80	7,06E-02
ENSG00000275294	ENSG00000275294	13	27178263	27251288	lincRNA	2,80	7,06E-02
ENSG00000255933	ENSG00000255933	12	131756966	131758047	lincRNA	2,80	1,40E-02
ENSG00000257467	ENSG00000257467	12	81270675	81312422	lincRNA	2,80	7,06E-02
ENSG00000258172	ENSG00000258172	12	94272150	94277195	antisense	2,80	7,06E-02
ENSG00000228915	OR7E128P	11	71893410	71894433	unprocessed_pseudogene	2,80	1,40E-02
ENSG00000231233	CFAP58-AS1	10	104351591	104353575	antisense	2,80	7,06E-02
ENSG00000205879	FAM90A2P	8	12172202	12178575	unprocessed_pseudogene	2,80	0
ENSG00000254936	ENSG00000254936	8	11123381	11126064	sense_intronic	2,80	7,06E-02
ENSG00000229660	ENSG00000229660	7	157281536	157282686	lincRNA	2,80	7,06E-02
ENSG00000231255	ENSG00000231255	7	86775081	86776022	antisense	2,80	0,2161218
ENSG00000236510	ENSG00000236510	7	3642815	3643784	antisense	2,80	0,2161218
ENSG00000271064	ENSG00000271064	7	66748838	66749077	processed_pseudogene	2,80	7,06E-02
ENSG00000282572	ENSG00000282572	7	19018	35489	processed_transcript	2,80	7,06E-02
ENSG00000181126	HLA-V	6	29790954	29797811	transcribed_unprocessed_pseudogene	2,80	0,2161218
ENSG00000230910	ENSG00000230910	6	68629962	68635027	antisense	2,80	0,2161218
ENSG00000251682	ENSG00000251682	5	61347126	61347716	processed_pseudogene	2,80	1,40E-02
ENSG00000272070	ENSG00000272070	5	141326210	141329357	lincRNA	2,80	0,2161218
ENSG00000276945	ENSG00000276945	5	61145846	61146376	unprocessed_pseudogene	2,80	1,40E-02
ENSG00000248416	ENSG00000248416	4	722275	724034	antisense	2,80	1,40E-02
ENSG00000236833	ENSG00000236833	3	197660565	197665757	lincRNA	2,80	7,06E-02
ENSG00000244383	FAM3D-AS1	3	58607080	58634440	antisense	2,80	0,2161218
ENSG00000224346	BICD1P1	2	200844240	200845461	processed_pseudogene	2,80	0
ENSG00000227938	ENSG00000227938	2	28448167	28450184	lincRNA	2,80	0,2161218
ENSG00000237380	HOXD-AS2	2	176121611	176137098	antisense	2,80	7,06E-02
ENSG00000271889	ENSG00000271889	2	61151433	61162105	lincRNA	2,80	0,2161218

ENSG00000272180	ENSG00000272180	2	55952158	56181652	lincRNA	2,80	7,06E-02
ENSG00000272979	ENSG00000272979	2	190454092	190454521	antisense	2,80	0,2161218
ENSG00000224259	LINC01133	1	159961218	159984750	lincRNA	2,80	1,40E-02
ENSG00000226499	ENSG00000226499	1	44843921	44844082	processed_pseudogene	2,80	0,2161218
ENSG00000259678	ENSG00000259678	15	51414097	51498833	antisense	2,80	0
ENSG00000277998	ENSG00000277998	2	164573741	164574054	unprocessed_pseudogene	2,79	0,0358681
ENSG00000239959	ENPP7P2	3	75446769	75491202	unprocessed_pseudogene	2,79	0
ENSG00000260708	ENSG00000260708	22	46761894	46762563	antisense	2,79	0,2161218
ENSG00000223508	RPL23AP53	8	213186	232231	transcribed_processed_pseudogene	2,79	0
ENSG00000271268	ENSG00000271268	17	35861093	35861566	processed_pseudogene	2,78	0
ENSG00000271266	LAPTM4BP2	3	72884234	72884679	processed_pseudogene	2,78	0
ENSG00000267207	ENSG00000267207	17	61034636	61070122	antisense	2,78	0
ENSG00000226266	ENSG00000226266	2	159670708	159712435	lincRNA	2,78	3,30E-03
ENSG00000247934	ENSG00000247934	12	28163298	28190738	antisense	2,77	1,95E-02
ENSG00000226816	ENSG00000226816	7	23206013	23208045	lincRNA	2,77	9,18E-02
ENSG00000235237	ENSG00000235237	22	37166757	37182850	antisense	2,76	0
ENSG00000236792	ENSG00000236792	1	192247505	192247947	processed_pseudogene	2,76	0
ENSG00000226167	AP4B1-AS1	1	113856635	113901237	antisense	2,75	6,27E-03
ENSG00000235453	TOPORS-AS1	9	32551144	32567005	antisense	2,75	6,27E-03
ENSG00000226332	ENSG00000226332	20	62305432	62306325	antisense	2,75	0
ENSG00000244513	ENSG00000244513	3	69013941	69056622	antisense	2,74	0
ENSG00000255650	FAM222A-AS1	12	109734166	109773508	antisense	2,73	4,86E-02
ENSG00000244265	SIAH2-AS1	3	150761937	150762538	antisense	2,73	0
ENSG00000277954	ENSG00000277954	16	79202624	79206739	antisense	2,73	0,1187106
ENSG00000265282	ENSG00000265282	17	63430468	63432211	lincRNA	2,73	0,1836819
ENSG00000260269	ENSG00000260269	15	75527150	75601205	processed_transcript	2,73	6,27E-03
ENSG00000275611	ENSG00000275611	13	110429026	110429342	sense_intronic	2,73	0,1836819
ENSG00000256947	ENSG00000256947	11	114210616	114356571	antisense	2,73	6,27E-03
ENSG00000253704	ENSG00000253704	8	94553722	94569745	lincRNA	2,73	0,1836819
ENSG00000226599	ENSG00000226599	6	150650772	150652025	lincRNA	2,73	0,0358681
ENSG00000163915	IGF2BP2-AS1	3	185712528	185729787	antisense	2,73	0,1392851
ENSG00000260025	ENSG00000260025	2	36354749	36355114	lincRNA	2,73	0,1836819
ENSG00000255495	ENSG00000255495	8	12194467	12196280	antisense	2,72	3,30E-03
ENSG00000250218	ALDH1L1-AS1	3	126103640	126108069	antisense	2,71	0,1836819
ENSG00000247556	OIP5-AS1	15	41283990	41309737	processed_transcript	2,71	0
ENSG00000259071	ENSG00000259071	14	50326526	50327909	lincRNA	2,71	0,0358681
ENSG00000263006	ROCK1P1	18	109065	122219	transcribed_unprocessed_pseudogene	2,71	3,30E-03
ENSG00000215102	TERF1P4	X	83748825	83749939	processed_pseudogene	2,71	1,40E-02
ENSG00000230912	ENSG00000230912	22	38666508	38668750	antisense	2,70	7,06E-02
ENSG00000204556	ENSG00000204556	20	26018832	26020684	unprocessed_pseudogene	2,70	7,06E-02
ENSG00000226465	ENSG00000226465	20	25751218	25752761	antisense	2,70	0,2161218
ENSG00000278035	ENSG00000278035	20	36233851	36234297	lincRNA	2,70	7,06E-02
ENSG00000266936	ENSG00000266936	19	11010917	11016011	antisense	2,70	7,06E-02
ENSG00000268621	ENSG00000268621	19	46189166	46203083	lincRNA	2,70	0,2161218
ENSG00000269802	ENSG00000269802	19	6343761	6362149	antisense	2,70	0,2161218
ENSG00000262228	ENSG00000262228	17	763565	765319	lincRNA	2,70	0,2161218
ENSG00000265010	ENSG00000265010	17	73243093	73244706	sense_intronic	2,70	7,06E-02
ENSG00000265800	ENSG00000265800	17	75138416	75141350	sense_intronic	2,70	7,06E-02
ENSG00000265971	ENSG00000265971	17	63454993	63455817	lincRNA	2,70	0,2161218
ENSG00000267369	ENSG00000267369	17	35498218	35499175	processed_pseudogene	2,70	0,2161218
ENSG00000267644	ENSG00000267644	17	28219285	28220005	lincRNA	2,70	0,2161218
ENSG00000277382	ENSG00000277382	17	76709760	76710045	antisense	2,70	0,2161218
ENSG00000260420	ENSG00000260420	16	88194281	88195217	lincRNA	2,70	7,06E-02
ENSG00000260671	ENSG00000260671	16	87739771	87747706	sense_intronic	2,70	1,40E-02
ENSG00000260908	ENSG00000260908	16	29053732	29108108	transcribed_unprocessed_pseudogene	2,70	0

ENSG00000260927	ENSG00000260927	16	58129529	58159133	antisense	2,70	0,2161218
ENSG00000270006	ENSG00000270006	16	87317509	87318043	antisense	2,70	7,06E-02
ENSG00000243404	RPL35AP32	15	66446455	66446777	processed_pseudogene	2,70	0,2161218
ENSG00000260685	ENSG00000260685	15	75950464	75951144	processed_pseudogene	2,70	0,2161218
ENSG00000254016	ALG1L10P	12	8227734	8231764	unprocessed_pseudogene	2,70	0
ENSG00000255966	ENSG00000255966	12	6532290	6533498	antisense	2,70	7,06E-02
ENSG00000224295	OLFM5P	11	5518441	5524955	unprocessed_pseudogene	2,70	0
ENSG00000270972	ENSG00000270972	11	333192	333688	lincRNA	2,70	0,2161218
ENSG00000227076	ENSG00000227076	10	127934698	127936167	sense_intronic	2,70	7,06E-02
ENSG00000231920	NEBL-AS1	10	21174014	21175048	antisense	2,70	0,2161218
ENSG00000234256	PTCD2P2	10	88876450	88877544	processed_pseudogene	2,70	1,40E-02
ENSG00000235618	FAM21EP	10	50021182	50067803	transcribed_unprocessed_pseudogene	2,70	7,06E-02
ENSG00000237949	LINC00844	10	58999626	59001543	lincRNA	2,70	1,40E-02
ENSG00000272319	ENSG00000272319	10	42579724	42579858	unprocessed_pseudogene	2,70	1,40E-02
ENSG00000270917	ENSG00000270917	9	120812475	120812845	processed_pseudogene	2,70	1,40E-02
ENSG00000214733	ENSG00000214733	8	143788920	143790459	antisense	2,70	0,2161218
ENSG00000223893	GNL2P1	7	49942251	49944440	processed_pseudogene	2,70	0,2161218
ENSG00000249574	ENSG00000249574	7	379359	382712	lincRNA	2,70	1,40E-02
ENSG00000240003	RPL7P24	5	80500332	80501400	processed_pseudogene	2,70	0,2161218
ENSG00000248968	ENSG00000248968	5	10248325	10249915	antisense	2,70	7,06E-02
ENSG00000251139	ENSG00000251139	4	184813619	184821300	antisense	2,70	7,06E-02
ENSG00000261496	ENSG00000261496	4	86012296	86013874	sense_overlapping	2,70	0,2161218
ENSG00000223899	SEC13P1	3	33033863	33034810	processed_pseudogene	2,70	0,2161218
ENSG00000239483	RPS15AP16	3	128798841	128799201	processed_pseudogene	2,70	1,40E-02
ENSG00000243429	OR7E29P	3	125712139	125713045	unprocessed_pseudogene	2,70	0,2161218
ENSG00000244327	ENSG00000244327	3	142465315	142472337	sense_overlapping	2,70	0
ENSG00000270135	ENSG00000270135	3	169793495	169793966	antisense	2,70	1,40E-02
ENSG00000224425	ENSG00000224425	2	131352214	131353239	processed_pseudogene	2,70	1,40E-02
ENSG00000215811	BTNL10	1	228508655	228512305	unprocessed_pseudogene	2,70	7,06E-02
ENSG00000224690	UBE2D3P3	1	150800473	150801031	processed_pseudogene	2,70	0,2161218
ENSG00000229021	ENSG00000229021	1	151994531	152042774	antisense	2,70	7,06E-02
ENSG00000229901	ENSG00000229901	1	41375004	41375669	antisense	2,70	0,2161218
ENSG00000235308	ENSG00000235308	1	88923370	88923844	processed_pseudogene	2,70	0,2161218
ENSG00000239873	GAPDHP27	1	119558755	119559774	processed_pseudogene	2,70	0,2161218
ENSG00000231728	ENSG00000231728	X	103917411	103919548	antisense	2,70	0
ENSG00000237903	ENSG00000237903	X	119422917	119423547	sense_intronic	2,70	7,06E-02
ENSG00000270497	ENSG00000270497	X	53065053	53065548	processed_pseudogene	2,70	0,2161218
ENSG00000259642	ST20-AS1	15	79922771	79926993	antisense	2,70	3,30E-03
ENSG00000226329	ENSG00000226329	7	22863874	22881350	unprocessed_pseudogene	2,69	0,3318209
ENSG00000270761	ENSG00000270761	6	47477243	47477572	lincRNA	2,69	0,1836819
ENSG00000224043	CCNT2-AS1	2	134735464	134918710	antisense	2,69	6,27E-03
ENSG00000221990	EXOC3-AS1	5	441498	443160	antisense	2,69	1,40E-02
ENSG00000230750	SDAD1P2	20	10385779	10387806	processed_pseudogene	2,69	2,21E-02
ENSG00000267319	ENSG00000267319	19	37693280	37693564	processed_pseudogene	2,69	6,27E-03
ENSG00000213871	TAF9BP1	3	25754925	25755670	processed_pseudogene	2,69	0,1836819
ENSG00000251669	FAM86EP	4	3941760	3955419	transcribed_unprocessed_pseudogene	2,69	1,40E-02
ENSG00000270179	ENSG00000270179	11	113368478	113369117	sense_intronic	2,68	1,40E-02
ENSG00000225177	ENSG00000225177	6	138692548	138697288	antisense	2,68	0,2746192
ENSG00000240225	ZNF542P	19	56368099	56379828	transcribed_unprocessed_pseudogene	2,67	1,40E-02
ENSG00000139239	RPL14P1	12	62965325	62965969	processed_pseudogene	2,67	9,18E-02
ENSG00000275763	C18orf65	18	76495521	76498088	lincRNA	2,67	1,40E-02
ENSG00000261465	ENSG00000261465	16	19062499	19067691	antisense	2,67	0

ENSG00000225913	ENSG00000225913	10	87607985	87659279	lincRNA	2,67	0,4332376
ENSG00000231367	ENSG00000231367	2	38406719	38515740	lincRNA	2,67	0,3565078
ENSG00000196922	ZNF252P	8	144973589	145002895	unitary_pseudogene	2,66	0
ENSG00000237149	ZNF503-AS2	10	75401519	75408982	antisense	2,66	2,21E-02
ENSG00000267809	NDUFV2P1	19	53223973	53224879	processed_pseudogene	2,66	6,27E-03
ENSG00000235652	ENSG00000235652	6	145799409	145886585	antisense	2,66	0
ENSG00000267774	ENSG00000267774	18	59696459	59698111	antisense	2,65	0
ENSG00000163597	SNHG16	17	76557766	76565348	processed_transcript	2,65	0,0358681
ENSG00000154608	CEP170P1	4	118467590	118554204	transcribed_unprocessed_pseudogene	2,65	0
ENSG00000227617	CERS6-AS1	2	168771953	168786961	processed_transcript	2,65	0
ENSG00000235859	ENSG00000235859	7	30371508	30372741	processed_pseudogene	2,65	9,18E-02
ENSG00000228903	RASA4CP	7	44026951	44041892	transcribed_unprocessed_pseudogene	2,65	3,30E-03
ENSG00000274499	ENSG00000274499	15	20803505	20826173	transcribed_processed_pseudogene	2,64	0,2746192
ENSG00000213740	SERBP1P1	X	68783472	68785066	processed_pseudogene	2,64	0
ENSG00000271631	ENSG00000271631	9	112885158	112885767	antisense	2,64	0,1836819
ENSG00000224855	OPA1-AS1	3	193618609	193627337	antisense	2,64	0
ENSG00000276578	ENSG00000276578	19	56485923	56494280	unprocessed_pseudogene	2,63	1,95E-02
ENSG00000240445	FOXO3B	17	18670946	18672873	processed_pseudogene	2,63	0
ENSG00000259298	ENSG00000259298	15	50497195	50498744	antisense	2,63	0
ENSG00000255621	ENSG00000255621	12	13000451	13040679	lincRNA	2,63	0,2161218
ENSG00000261668	ENSG00000261668	4	127840198	127844040	antisense	2,63	0,4332376
ENSG00000235084	CHCHD2P6	1	15604597	15605043	processed_pseudogene	2,62	0,2161218
ENSG00000181819	KCTD9P2	X	105405647	105407039	processed_pseudogene	2,62	0
ENSG00000236790	LINC00299	2	8007771	8383621	lincRNA	2,62	0,0358681
ENSG00000267199	ENSG00000267199	18	12438890	12448205	antisense	2,61	2,21E-02
ENSG00000266908	ENSG00000266908	20	5499116	5502881	lincRNA	2,61	0
ENSG00000270751	ENSG00000270751	4	152337655	152338098	antisense	2,61	0
ENSG00000212664	ENSG00000212664	15	71341158	71341712	processed_pseudogene	2,61	1,95E-02
ENSG00000213209	ENSG00000213209	7	148637179	148637587	processed_pseudogene	2,60	0,1392851
ENSG00000235837	ENSG00000235837	7	16695871	16719898	antisense	2,60	2,21E-02
ENSG00000237517	DGCR5	22	18970514	19031242	antisense	2,60	0,2161218
ENSG00000273442	ENSG00000273442	22	17080701	17081456	lincRNA	2,60	7,06E-02
ENSG00000281420	ENSG00000281420	21	43748365	43748468	pseudogene	2,60	0,2161218
ENSG00000167046	ENSG00000167046	20	62640719	62643304	antisense	2,60	7,06E-02
ENSG00000242325	RPS12P31	19	32636078	32636475	processed_pseudogene	2,60	7,06E-02
ENSG00000267296	CEBPA-AS1	19	33302857	33305054	antisense	2,60	0,2161218
ENSG00000264707	L3MBTL4-AS1	18	6256747	6260934	lincRNA	2,60	7,06E-02
ENSG00000261575	ENSG00000261575	17	46267037	46268694	transcribed_unprocessed_pseudogene	2,60	0,2161218
ENSG00000262223	ENSG00000262223	17	81376001	81385360	lincRNA	2,60	0,2161218
ENSG00000261889	ENSG00000261889	16	3156736	3157483	lincRNA	2,60	0,2161218
ENSG00000249839	ENSG00000249839	15	43663654	43684339	unprocessed_pseudogene	2,60	1,40E-02
ENSG00000259368	ENSG00000259368	15	40368925	40369640	antisense	2,60	0
ENSG00000259771	ENSG00000259771	15	59266720	59271162	sense_intronic	2,60	0,2161218
ENSG00000275332	ENSG00000275332	15	64943240	64943672	antisense	2,60	0,2746192
ENSG00000277152	ENSG00000277152	15	66860303	66867023	lincRNA	2,60	0,2161218
ENSG00000234911	TEX21P	14	64346171	64374666	unitary_pseudogene	2,60	0
ENSG00000258458	ENSG00000258458	14	22702611	22766562	processed_transcript	2,60	0
ENSG00000258867	LINC01146	14	88024550	88097619	lincRNA	2,60	7,06E-02
ENSG00000270062	ENSG00000270062	14	50723777	50724272	sense_intronic	2,60	1,40E-02
ENSG00000224418	STK24-AS1	13	98577244	98578830	antisense	2,60	0,2161218
ENSG00000235280	MCF2L-AS1	13	112967484	112968824	antisense	2,60	0,2161218
ENSG00000225342	ENSG00000225342	12	40186009	40224915	antisense	2,60	0,2161218
ENSG00000256888	ENSG00000256888	12	10750234	10777444	lincRNA	2,60	0,2161218
ENSG00000258210	ENSG00000258210	12	110387463	110445548	processed_transcript	2,60	7,06E-02
ENSG00000254463	ENSG00000254463	11	43466392	43466871	processed_pseudogene	2,60	0,2161218

ENSG00000268472	ENSG00000268472	11	112260265	112261396	antisense	2,60	7,06E-02
ENSG00000212952	ENSG00000212952	9	42568865	42569315	processed_pseudogene	2,60	0,2161218
ENSG00000225411	ENSG00000225411	9	63802144	63814159	lincRNA	2,60	0,2161218
ENSG00000206014	OR7E161P	8	11928597	11929563	unprocessed_pseudogene	2,60	0,2161218
ENSG00000248159	HSPA8P11	8	30237382	30240997	processed_pseudogene	2,60	0,2161218
ENSG00000254948	OR7E158P	8	11919900	11920809	unprocessed_pseudogene	2,60	0,2161218
ENSG00000255052	FAM66D	8	12115782	12177550	antisense	2,60	0
ENSG00000258256	ENSG00000258256	8	85222446	85245717	antisense	2,60	7,06E-02
ENSG00000231951	ENSG00000231951	7	39609610	39610290	antisense	2,60	7,06E-02
ENSG00000240499	ENSG00000240499	7	122328469	122440388	antisense	2,60	0
ENSG00000272953	ENSG00000272953	7	5425770	5426401	lincRNA	2,60	0,2161218
ENSG00000273297	ENSG00000273297	7	134416290	134432453	lincRNA	2,60	7,06E-02
ENSG00000214894	LINC00243	6	30798654	30830659	lincRNA	2,60	0,2161218
ENSG00000219392	ENSG00000219392	6	28115628	28116551	unprocessed_pseudogene	2,60	7,06E-02
ENSG00000219395	HSPA8P15	6	151411259	151413945	processed_pseudogene	2,60	0,2161218
ENSG00000225914	ENSG00000225914	6	32255711	32265838	antisense	2,60	1,40E-02
ENSG00000248464	FGF10-AS1	5	44388732	44413989	antisense	2,60	1,40E-02
ENSG00000248559	ENSG00000248559	5	134399495	134401921	antisense	2,60	7,06E-02
ENSG00000249068	ENSG00000249068	5	109840128	109840692	processed_pseudogene	2,60	1,40E-02
ENSG00000250309	ENSG00000250309	5	150670658	150672390	antisense	2,60	1,40E-02
ENSG00000251000	ENSG00000251000	5	95834424	95835046	processed_pseudogene	2,60	7,06E-02
ENSG00000272382	ENSG00000272382	5	43033716	43034635	lincRNA	2,60	7,06E-02
ENSG00000248399	ENSG00000248399	4	2463797	2464124	sense_intronic	2,60	0
ENSG00000251600	ENSG00000251600	4	143912331	143982454	lincRNA	2,60	7,06E-02
ENSG00000239804	ENSG00000239804	3	125787888	125788146	processed_pseudogene	2,60	1,40E-02
ENSG00000248916	NUP210P3	3	129323046	129325426	unprocessed_pseudogene	2,60	0,2161218
ENSG00000272989	ENSG00000272989	3	197505262	197506986	lincRNA	2,60	7,06E-02
ENSG00000276925	ENSG00000276925	3	47469777	47469987	processed_pseudogene	2,60	1,40E-02
ENSG00000259056	DUXAP1	2	84750769	84751231	transcribed_unprocessed_pseudogene	2,60	0,2161218
ENSG00000272861	ENSG00000272861	2	105249404	105249794	lincRNA	2,60	7,06E-02
ENSG00000273763	ENSG00000273763	2	65203571	65203813	processed_pseudogene	2,60	7,06E-02
ENSG00000229953	ENSG00000229953	1	156646507	156661424	antisense	2,60	0,2161218
ENSG00000268062	TOP1P1	1	171345105	171345307	processed_pseudogene	2,60	7,06E-02
ENSG00000271267	ENSG00000271267	1	155566050	155566306	processed_pseudogene	2,60	0
ENSG00000214558	ENSG00000214558	6	65302522	65304957	processed_pseudogene	2,59	0,1187106
ENSG00000224722	ENSG00000224722	8	127086263	127087510	processed_pseudogene	2,59	2,95E-02
ENSG00000238165	ENSG00000238165	2	50829442	50829678	processed_pseudogene	2,57	0
ENSG00000226950	DANCR	4	52712404	52720351	processed_transcript	2,57	0,3318209
ENSG00000223799	IL10RB-AS1	21	33263873	33266260	antisense	2,57	2,21E-02
ENSG00000276182	ENSG00000276182	14	96537170	96537904	sense_intronic	2,57	0
ENSG00000225022	UBE2D3P1	20	5292388	5292831	processed_pseudogene	2,57	0
ENSG00000270108	ENSG00000270108	14	103687576	103688127	sense_intronic	2,57	0
ENSG00000261114	ENSG00000261114	16	56941028	56941726	sense_intronic	2,57	6,27E-03
ENSG00000244363	RPL7P23	5	77582376	77583122	processed_pseudogene	2,57	2,95E-02
ENSG00000231999	ENSG00000231999	1	89583241	89632894	antisense	2,56	0
ENSG00000278075	ENSG00000278075	18	79900555	79900903	lincRNA	2,55	0,0358681
ENSG00000229750	ENSG00000229750	2	175167904	175168522	antisense	2,55	6,27E-03
ENSG00000219665	ENSG00000219665	19	11987617	12046275	processed_transcript	2,55	0,2161218
ENSG00000254911	SCARNA9	11	93721513	93721865	antisense	2,55	0,4332376
ENSG00000254978	ALG1L9P	11	71800541	71804640	transcribed_unprocessed_pseudogene	2,54	6,27E-03
ENSG00000250299	MRPS31P4	13	52600119	52637446	transcribed_unprocessed_pseudogene	2,54	1,40E-02
ENSG00000254013	MAP2K1P1	8	30027713	30028900	processed_pseudogene	2,54	0,4332376
ENSG00000228506	ENSG00000228506	6	99424922	99431373	antisense	2,53	2,21E-02
ENSG00000218283	MORF4L1P1	1	220253570	220254536	processed_pseudogene	2,52	0

ENSG00000237447	CDC27P2	X	10190377	10192298	processed_pseudogene	2,52	0
ENSG00000271075	ENSG00000271075	14	52871458	52872217	processed_pseudogene	2,52	0
ENSG00000213062	ENSG00000213062	1	169486076	169486986	lincRNA	2,52	1,40E-02
ENSG00000187653	TMSB4XP8	4	90838903	90839037	processed_pseudogene	2,51	0
ENSG00000230769	ENSG00000230769	22	40788777	40789267	processed_pseudogene	2,50	0,2161218
ENSG00000235091	ENSG00000235091	22	45875999	45887748	lincRNA	2,50	1,40E-02
ENSG00000281903	ENSG00000281903	21	14819699	14918552	lincRNA	2,50	0
ENSG00000230495	ENSG00000230495	20	17599255	17599807	processed_pseudogene	2,50	0,2161218
ENSG00000272407	ENSG00000272407	20	41196647	41196938	transcribed_processed_pseudogene	2,50	0,2161218
ENSG00000278192	ENSG00000278192	20	6065966	6067897	lincRNA	2,50	0,2161218
ENSG00000267219	ENSG00000267219	19	34348356	34359412	antisense	2,50	7,06E-02
ENSG00000269102	ENSG00000269102	19	52284780	52297920	antisense	2,50	0,2161218
ENSG00000270317	ENSG00000270317	19	22501699	22501948	processed_pseudogene	2,50	7,06E-02
ENSG00000273901	ENSG00000273901	19	58500505	58501137	antisense	2,50	1,40E-02
ENSG00000267717	SRSF10P1	18	51158282	51159383	processed_pseudogene	2,50	0,2161218
ENSG00000273664	ENSG00000273664	18	59700523	59700946	lincRNA	2,50	1,40E-02
ENSG00000278330	ENSG00000278330	18	77112602	77115726	antisense	2,50	0,2161218
ENSG00000263164	ENSG00000263164	17	5240508	5241543	lincRNA	2,50	7,06E-02
ENSG00000264727	ENSG00000264727	17	11288205	11290811	lincRNA	2,50	0,2161218
ENSG00000266934	ENSG00000266934	17	61382785	61384680	sense_intronic	2,50	0,2161218
ENSG00000267648	ENSG00000267648	17	35406948	35407818	transcribed_processed_pseudogene	2,50	7,06E-02
ENSG00000260249	ENSG00000260249	16	50666211	50671639	antisense	2,50	0
ENSG00000260316	ENSG00000260316	16	898967	905224	sense_intronic	2,50	7,06E-02
ENSG00000260517	ENSG00000260517	16	29116127	29217860	lincRNA	2,50	0,2161218
ENSG00000260876	LINC01229	16	79676108	79807922	lincRNA	2,50	7,06E-02
ENSG00000166763	STRCP1	15	43699488	43718184	unprocessed_pseudogene	2,50	0,2161218
ENSG00000259592	PRELID1P4	15	35121573	35122084	processed_pseudogene	2,50	7,06E-02
ENSG00000259151	CAP2P1	14	89290205	89291594	transcribed_processed_pseudogene	2,50	1,40E-02
ENSG00000261553	ENSG00000261553	13	75549773	75807120	processed_transcript	2,50	0,3565078
ENSG00000254397	ENSG00000254397	11	9430356	9433486	antisense	2,50	0
ENSG00000255236	ENSG00000255236	11	68122053	68130518	antisense	2,50	7,06E-02
ENSG00000226842	ENSG00000226842	10	32281686	32282829	antisense	2,50	0,2161218
ENSG00000232259	ENSG00000232259	10	127929376	127934517	lincRNA	2,50	1,40E-02
ENSG00000259267	ENSG00000259267	10	70939053	70955641	lincRNA	2,50	7,06E-02
ENSG00000226334	ENSG00000226334	9	104927553	104928892	antisense	2,50	6,27E-03
ENSG00000232850	PTGES2-AS1	9	128128529	128130628	lincRNA	2,50	7,06E-02
ENSG00000188365	ENSG00000188365	7	5419846	5423122	antisense	2,50	7,06E-02
ENSG00000226874	ENSG00000226874	7	30544053	30544431	processed_pseudogene	2,50	7,06E-02
ENSG00000234185	ENSG00000234185	7	66119603	66165011	antisense	2,50	0,2161218
ENSG00000272752	STAG3L5P-PVRIG2P	7	100336104	100367831	processed_transcript	2,50	7,06E-02
ENSG00000231683	ENSG00000231683	6	53561289	53617171	lincRNA	2,50	1,40E-02
ENSG00000236475	TRIM26BP	6	30238301	30242279	unprocessed_pseudogene	2,50	0,2161218
ENSG00000248367	ENSG00000248367	5	180293245	180295253	antisense	2,50	7,06E-02
ENSG00000249619	HMGN1P13	5	111572102	111572239	processed_pseudogene	2,50	0,2161218
ENSG00000213872	ENSG00000213872	3	25749488	25749896	processed_pseudogene	2,50	0,2161218
ENSG00000261468	ENSG00000261468	3	30697661	30699576	sense_overlapping	2,50	7,06E-02
ENSG00000233255	ENSG00000233255	2	164840749	164849334	antisense	2,50	7,06E-02
ENSG00000233397	ENSG00000233397	2	162093429	162094854	lincRNA	2,50	0,2161218
ENSG00000235848	RMDN2-AS1	2	37949911	38067041	antisense	2,50	9,18E-02
ENSG00000272754	ENSG00000272754	2	32321638	32323002	lincRNA	2,50	0,2161218
ENSG00000280721	ENSG00000280721	2	87439523	87459044	lincRNA	2,50	0,2161218
ENSG00000215089	KRT18P11	X	92459670	92460952	processed_pseudogene	2,50	6,27E-03
ENSG00000225071	ENSG00000225071	X	24429573	24429920	processed_pseudogene	2,50	0,2161218
ENSG00000219023	ENSG00000219023	6	35555873	35556264	processed_pseudogene	2,50	9,18E-02
ENSG00000250068	ENSG00000250068	9	112581779	112582590	unprocessed_pseudogene	2,49	6,27E-03

ENSG00000232460	BMPR1APS2	11	121361854	121362865	processed_pseudogene	2,48	0,1836819
ENSG00000188825	LINC00910	17	43369845	43389199	lincRNA	2,48	3,30E-03
ENSG00000258427	RBM8B	14	60397757	60398281	processed_pseudogene	2,48	0
ENSG00000235560	ENSG00000235560	16	30534752	30537144	antisense	2,48	6,27E-03
ENSG00000237399	PITRM1-AS1	10	3141632	3167972	antisense	2,48	2,21E-02
ENSG00000272323	ENSG00000272323	5	34837549	34839278	antisense	2,46	2,95E-02
ENSG00000268745	ENSG00000268745	6	41791410	41791477	antisense	2,46	0,1217903
ENSG00000233593	ENSG00000233593	1	90782983	90851657	lincRNA	2,46	0,1392851
ENSG00000197099	ENSG00000197099	3	186579476	186772986	antisense	2,46	2,95E-02
ENSG00000234383	CTBP2P8	1	68161761	68163090	processed_pseudogene	2,46	0
ENSG00000259175	ENSG00000259175	15	80554609	80562944	antisense	2,45	0,1836819
ENSG00000255644	ENSG00000255644	12	21759980	21766705	antisense	2,45	1,40E-02
ENSG00000237461	ENSG00000237461	9	99585786	99819889	lincRNA	2,45	0,0358681
ENSG00000239650	GUSBP4	6	57919784	57930291	unprocessed_pseudogene	2,45	6,27E-03
ENSG00000224479	ENSG00000224479	3	63742293	63827445	transcribed_unprocessed_pseudogene	2,45	0,0358681
ENSG00000228589	SPCS2P4	1	28095742	28096422	processed_pseudogene	2,45	0
ENSG00000275441	ENSG00000275441	16	28553709	28554140	antisense	2,44	6,27E-03
ENSG00000233847	ENSG00000233847	5	60917490	60919573	antisense	2,44	6,27E-03
ENSG00000234630	ENSG00000234630	22	22293733	22294794	lincRNA	2,44	2,95E-02
ENSG00000265263	ENSG00000265263	17	19334308	19336127	antisense	2,43	2,95E-02
ENSG00000217289	ENSG00000217289	2	129992889	129994040	processed_pseudogene	2,43	2,21E-02
ENSG00000237550	RPL9P9	15	82372196	82372912	transcribed_processed_pseudogene	2,43	1,40E-02
ENSG00000276434	ENSG00000276434	13	113339450	113339958	sense_intronic	2,43	7,06E-02
ENSG00000216906	ENSG00000216906	6	149904243	149906418	processed_pseudogene	2,43	3,30E-03
ENSG00000237655	ENSG00000237655	2	177603089	177618572	antisense	2,43	6,27E-03
ENSG00000269937	ENSG00000269937	16	2561471	2565096	antisense	2,42	1,40E-02
ENSG00000230513	THAP7-AS1	22	21001886	21010342	antisense	2,42	0,4036702
ENSG00000281106	LINC00282	13	51813347	51845175	lincRNA	2,42	0,0358681
ENSG00000244055	ENSG00000244055	7	92457564	92491610	antisense	2,42	6,27E-03
ENSG00000259660	DNM1P47	15	101751032	101794302	transcribed_unprocessed_pseudogene	2,42	0
ENSG00000251532	ENSG00000251532	5	1544107	1551710	lincRNA	2,42	0,1836819
ENSG00000186235	ENSG00000186235	2	238224552	238231677	processed_transcript	2,40	0,4036702
ENSG00000240342	RPS2P5	12	118246084	118246962	processed_pseudogene	2,40	0,0358681
ENSG00000273000	ENSG00000273000	22	23652860	23717347	processed_transcript	2,40	7,06E-02
ENSG00000272825	ENSG00000272825	21	44936303	44936954	antisense	2,40	7,06E-02
ENSG00000230725	ENSG00000230725	20	25140789	25149258	lincRNA	2,40	6,27E-03
ENSG00000260536	ENSG00000260536	20	32843128	32854257	antisense	2,40	7,06E-02
ENSG00000130612	CYP2G1P	19	40890826	40900508	transcribed_unprocessed_pseudogene	2,40	0,2161218
ENSG00000268475	ENSG00000268475	19	41425359	41426237	antisense	2,40	7,06E-02
ENSG00000263627	PPP4R1-AS1	18	9615264	9619363	lincRNA	2,40	0,2161218
ENSG00000267000	ENSG00000267000	18	59459072	59465682	antisense	2,40	0,2161218
ENSG00000240270	RPL12P37	17	80976454	80976948	processed_pseudogene	2,40	7,06E-02
ENSG00000260647	ENSG00000260647	17	18268080	18268828	antisense	2,40	0,2161218
ENSG00000263015	ENSG00000263015	17	503171	511347	antisense	2,40	0,2161218
ENSG00000264569	ENSG00000264569	17	82037905	82039380	antisense	2,40	0,2161218
ENSG00000265349	ENSG00000265349	17	9638768	9645325	antisense	2,40	0,2161218
ENSG00000276790	ENSG00000276790	17	47279526	47280047	antisense	2,40	0,2161218
ENSG00000260095	ENSG00000260095	16	2452581	2452977	antisense	2,40	1,40E-02
ENSG00000261216	ENSG00000261216	16	11908208	11908916	antisense	2,40	1,40E-02
ENSG00000262686	GLIS2-AS1	16	4324667	4328340	lincRNA	2,40	0,2161218
ENSG00000258712	CXADRP2	15	21372793	21373881	processed_pseudogene	2,40	7,06E-02
ENSG00000259315	ACTG1P17	15	82725873	82738904	transcribed_processed_pseudogene	2,40	0,2161218
ENSG00000259666	LINGO1-AS1	15	77641764	77652288	antisense	2,40	0,2161218
ENSG00000259921	ENSG00000259921	15	99157231	99161140	sense_intronic	2,40	0,2161218
ENSG00000258521	ENSG00000258521	14	100339832	100340554	antisense	2,40	0,2161218

ENSG00000258904	ENSG00000258904	14	100263781	100265405	sense_intronic	2,40	1,40E-02
ENSG00000275569	ENSG00000275569	14	56305838	56306394	antisense	2,40	0,2161218
ENSG00000233613	DCUN1D2-AS	13	113468901	113476135	antisense	2,40	1,40E-02
ENSG00000219355	RPL31P52	12	120450437	120450803	processed_pseudogene	2,40	0,2161218
ENSG00000255474	ENSG00000255474	12	4700417	4720102	lincRNA	2,40	7,06E-02
ENSG00000274737	ENSG00000274737	12	47817451	47817966	sense_intronic	2,40	0
ENSG00000278095	ENSG00000278095	12	26273329	26273900	antisense	2,40	0,2161218
ENSG00000273312	ENSG00000273312	10	35606001	35608153	antisense	2,40	0,2161218
ENSG00000213612	FAM220CP	9	97121659	97122489	processed_pseudogene	2,40	0,2161218
ENSG00000225472	ENSG00000225472	9	14317085	14357908	antisense	2,40	0,2161218
ENSG00000235387	LINC00961	9	35909483	35911686	lincRNA	2,40	2,95E-02
ENSG00000253708	ENSG00000253708	8	30176554	30180888	antisense	2,40	0,2161218
ENSG00000261670	ENSG00000261670	8	103464389	103468985	lincRNA	2,40	0,2161218
ENSG00000234450	ENSG00000234450	7	156633355	156633787	processed_pseudogene	2,40	1,40E-02
ENSG00000236976	ENSG00000236976	7	64141538	64142401	processed_pseudogene	2,40	0,2161218
ENSG00000239556	ENSG00000239556	7	43984994	43990207	transcribed_unprocessed_pseudogene	2,40	0,2161218
ENSG00000214922	HLA-F-AS1	6	29726601	29749049	processed_transcript	2,40	0,2161218
ENSG00000225415	ENSG00000225415	6	138822747	138823799	processed_pseudogene	2,40	0,2161218
ENSG00000234515	PPP1R2P1	6	32879171	32879848	transcribed_processed_pseudogene	2,40	0,2161218
ENSG00000237174	ENSG00000237174	6	75357214	75399300	antisense	2,40	0,2161218
ENSG00000230929	ENSG00000230929	5	126628019	126628319	processed_pseudogene	2,40	1,40E-02
ENSG00000253628	ENSG00000253628	5	172816621	172819958	lincRNA	2,40	0,2161218
ENSG00000253686	LINC01484	5	173707614	173746211	lincRNA	2,40	1,40E-02
ENSG00000246526	ENSG00000246526	4	6687448	6690519	lincRNA	2,40	7,06E-02
ENSG00000213431	ENSG00000213431	3	100910975	100912175	processed_pseudogene	2,40	0,2161218
ENSG00000244668	SNRPCP3	3	150719997	150720836	processed_pseudogene	2,40	0,2161218
ENSG00000244681	MTHFD2P1	3	95654423	95683193	transcribed_processed_pseudogene	2,40	7,06E-02
ENSG00000271856	LINC01215	3	108125821	108138610	lincRNA	2,40	7,06E-02
ENSG00000272699	ENSG00000272699	3	179405448	179405944	antisense	2,40	0,2161218
ENSG00000234281	LANCL1-AS1	2	210324759	210469246	antisense	2,40	0,2161218
ENSG00000243389	ENSG00000243389	2	112589040	112614431	3prime_overlapping_ncrna	2,40	0,2161218
ENSG00000271825	ENSG00000271825	2	177300600	177302006	antisense	2,40	0,2161218
ENSG00000228955	ENSG00000228955	1	246632251	246632478	processed_pseudogene	2,40	7,06E-02
ENSG00000231407	ENSG00000231407	1	170460453	170532647	antisense	2,40	0,2161218
ENSG00000232022	FAAHP1	1	46432129	46482493	transcribed_unprocessed_pseudogene	2,40	0,2161218
ENSG00000238290	ENSG00000238290	1	8026738	8122702	lincRNA	2,40	7,06E-02
ENSG00000243960	ENSG00000243960	1	111438638	111441364	sense_overlapping	2,40	7,06E-02
ENSG00000271475	ENSG00000271475	1	228776312	228776865	processed_pseudogene	2,40	0,2161218
ENSG00000274386	ENSG00000274386	1	42787990	42798230	lincRNA	2,40	1,40E-02
ENSG00000233661	SPIN4-AS1	X	63349646	63352178	antisense	2,40	7,06E-02
ENSG00000234557	ENSG00000234557	X	101443707	101444103	processed_pseudogene	2,40	1,40E-02
ENSG00000232995	ENSG00000232995	1	163244505	163321894	antisense	2,39	0,1392851
ENSG00000227704	ENSG00000227704	6	169725504	169738992	antisense	2,39	0,1836819
ENSG00000233452	STXBP5-AS1	6	146841901	147204614	antisense	2,39	3,30E-03
ENSG00000255487	ENSG00000255487	8	38275888	38276680	antisense	2,39	6,27E-03
ENSG00000272529	ENSG00000272529	3	16345126	16346440	antisense	2,39	0,3565078
ENSG00000255340	ENSG00000255340	11	43378882	43385671	antisense	2,39	0
ENSG00000232332	ENSG00000232332	X	76029895	76030330	processed_pseudogene	2,38	0,0358681
ENSG00000229204	PTGES3P3	4	169791221	169791702	processed_pseudogene	2,38	2,95E-02
ENSG00000268903	ENSG00000268903	1	135141	135895	processed_pseudogene	2,38	0,1392851
ENSG00000254662	ENSG00000254662	11	57325603	57327958	antisense	2,37	2,21E-02
ENSG00000227910	ENSG00000227910	7	63924787	63926306	transcribed_processed_pseudogene	2,36	1,40E-02
ENSG00000164621	SMAD5-AS1	5	136129507	136134890	antisense	2,36	0,0358681
ENSG00000256152	ENSG00000256152	12	122865335	122867021	lincRNA	2,36	0,1836819
ENSG00000260659	ENSG00000260659	16	89226807	89228692	antisense	2,36	1,40E-02

ENSG00000227888	FAM66A	8	12362019	12388296	lincRNA	2,36	3,30E-03
ENSG00000224040	HMGN1P4	1	182942115	182942404	processed_pseudogene	2,35	1,95E-02
ENSG00000281358	RASSF1-AS1	3	50337511	50338300	antisense	2,35	0,0358681
ENSG00000268112	ENSG00000268112	19	17727840	17734513	antisense	2,35	3,30E-03
ENSG00000244676	ENSG00000244676	21	17793488	17810845	antisense	2,34	0
ENSG00000220472	ENSG00000220472	6	7338839	7339568	processed_pseudogene	2,34	6,27E-03
ENSG00000266978	ENSG00000266978	19	10252268	10285108	antisense	2,33	7,06E-02
ENSG00000231296	ENSG00000231296	1	40262672	40262984	processed_pseudogene	2,33	0,2161218
ENSG00000254433	ENSG00000254433	11	106085990	106101975	antisense	2,33	6,27E-03
ENSG00000245468	ENSG00000245468	4	7094571	7103385	lincRNA	2,32	7,06E-02
ENSG00000260101	ENSG00000260101	2	64522187	64524093	lincRNA	2,32	0,1836819
ENSG00000271936	ENSG00000271936	2	24825610	24826717	antisense	2,32	6,27E-03
ENSG00000254295	ENSG00000254295	5	172954907	172957162	lincRNA	2,31	0,1836819
ENSG00000270714	MINOS1P2	17	64747264	64747492	processed_pseudogene	2,31	9,18E-02
ENSG00000231764	DLX6-AS1	7	96955141	97014065	antisense	2,31	0,1836819
ENSG00000255541	ENSG00000255541	11	83209399	83209634	processed_pseudogene	2,31	0,2746192
ENSG00000272154	ENSG00000272154	5	141100242	141174391	antisense	2,31	0,2161218
ENSG00000262587	ENSG00000262587	16	25031744	25058109	transcribed_unprocessed_pseudogene	2,31	0,2161218
ENSG00000255125	ENSG00000255125	11	10783313	10801625	antisense	2,30	1,40E-02
ENSG00000183822	ENSG00000183822	22	36847372	36870441	antisense	2,30	7,06E-02
ENSG00000270116	ENSG00000270116	21	37100814	37101343	sense_intronic	2,30	0,2161218
ENSG00000225246	RPS2P1	20	34122470	34123290	processed_pseudogene	2,30	0,2161218
ENSG00000229222	KRT18P4	20	49956745	49958032	processed_pseudogene	2,30	0,2161218
ENSG00000272945	ENSG00000272945	20	34281632	34286466	sense_intronic	2,30	7,06E-02
ENSG00000266897	ENSG00000266897	19	13131571	13132410	lincRNA	2,30	0
ENSG00000267138	ENSG00000267138	19	3754620	3756659	sense_intronic	2,30	1,40E-02
ENSG00000268392	ENSG00000268392	19	57625032	57626950	lincRNA	2,30	7,06E-02
ENSG00000268981	ENSG00000268981	19	22665552	22716991	antisense	2,30	0,2161218
ENSG00000265962	GACAT2	18	8695856	8707621	antisense	2,30	0,2161218
ENSG00000267108	ENSG00000267108	18	12432897	12437635	lincRNA	2,30	0,2161218
ENSG00000236234	ENSG00000236234	17	45452844	45464065	antisense	2,30	1,40E-02
ENSG00000236838	ENSG00000236838	17	2213697	2214414	antisense	2,30	0,2161218
ENSG00000260011	ENSG00000260011	17	82381110	82382690	lincRNA	2,30	1,40E-02
ENSG00000262492	ENSG00000262492	17	7954868	7955622	processed_pseudogene	2,30	0,2161218
ENSG00000277450	ENSG00000277450	17	28373256	28373562	sense_intronic	2,30	0,2161218
ENSG00000260541	ENSG00000260541	16	1841020	1843547	sense_overlapping	2,30	7,06E-02
ENSG00000261131	ENSG00000261131	16	46660696	46661591	sense_intronic	2,30	7,06E-02
ENSG00000260693	ENSG00000260693	15	30540093	30545969	lincRNA	2,30	7,06E-02
ENSG00000258580	ENSG00000258580	14	32373337	32417974	antisense	2,30	0,2161218
ENSG00000273565	ENSG00000273565	14	75176929	75177418	lincRNA	2,30	0,2161218
ENSG00000233532	LINC00460	13	106376563	106378217	lincRNA	2,30	7,06E-02
ENSG00000235205	TATDN2P3	13	23243520	23247184	processed_pseudogene	2,30	0,2161218
ENSG00000236953	ZDHHC20-IT1	13	21376977	21377874	sense_intronic	2,30	7,06E-02
ENSG00000250451	HOXC-AS1	12	53999022	54000010	antisense	2,30	0,2161218
ENSG00000256325	ENSG00000256325	12	68828118	68828553	antisense	2,30	1,40E-02
ENSG00000257475	ENSG00000257475	12	53012104	53013921	sense_intronic	2,30	0,2161218
ENSG00000229719	MIR194-2HG	11	64889560	64893449	lincRNA	2,30	0,2161218
ENSG00000231793	DOC2GP	11	67612653	67616257	unitary_pseudogene	2,30	0,2161218
ENSG00000254944	ATP5F1P5	11	122960327	122961086	processed_pseudogene	2,30	0,2161218
ENSG00000256779	ENSG00000256779	11	95037482	95038456	processed_pseudogene	2,30	7,06E-02
ENSG00000257058	ENSG00000257058	11	62545999	62547699	antisense	2,30	0,2161218
ENSG00000272301	ENSG00000272301	11	76389648	76390128	lincRNA	2,30	0,2161218
ENSG00000229298	TUBB8P1	9	21811621	21812347	processed_pseudogene	2,30	0,2161218
ENSG00000231995	ENSG00000231995	9	62266319	62266919	processed_pseudogene	2,30	7,06E-02
ENSG00000221845	C7orf65	7	47655244	47661648	lincRNA	2,30	0,2161218
ENSG00000232491	SAPCD2P3	7	66556216	66557065	processed_pseudogene	2,30	0,2161218
ENSG00000236703	MYB-AS1	6	135195083	135195995	antisense	2,30	0
ENSG00000271931	ENSG00000271931	6	89080164	89080667	antisense	2,30	1,40E-02
ENSG00000230847	ENSG00000230847	5	71074225	71093193	unprocessed_pseudogene	2,30	0,2161218

ENSG00000253921	ENSG00000253921	5	151753992	151767247	antisense	2,30	0
ENSG00000272109	ENSG00000272109	5	96804353	96806105	antisense	2,30	0,2161218
ENSG00000255021	ENSG00000255021	3	14272373	14303845	lincRNA	2,30	7,06E-02
ENSG00000272334	ENSG00000272334	3	36973117	36973672	lincRNA	2,30	0,2161218
ENSG00000273682	ENSG00000273682	3	46918807	46918935	processed_pseudogene	2,30	1,40E-02
ENSG00000180152	ENSG00000180152	2	112853328	112854122	processed_pseudogene	2,30	0,1836819
ENSG00000232479	ENSG00000232479	2	202924609	202925904	processed_pseudogene	2,30	1,40E-02
ENSG00000234174	ENSG00000234174	2	113171535	113175360	antisense	2,30	0,2161218
ENSG00000235192	ENSG00000235192	2	165794851	165810010	lincRNA	2,30	0,2161218
ENSG00000271151	ENSG00000271151	2	173968351	173969418	lincRNA	2,30	0,2161218
ENSG00000273240	ENSG00000273240	2	189763859	189764456	antisense	2,30	7,06E-02
ENSG00000223583	ENSG00000223583	1	26454653	26455187	processed_pseudogene	2,30	0,2161218
ENSG00000231073	ENSG00000231073	1	150973123	150975534	antisense	2,30	7,06E-02
ENSG00000232993	ENSG00000232993	1	53069938	53085502	antisense	2,30	7,06E-02
ENSG00000237899	ENSG00000237899	1	40669089	40687588	lincRNA	2,30	7,06E-02
ENSG00000243659	ENSG00000243659	1	27311240	27311838	processed_pseudogene	2,30	7,06E-02
ENSG00000271647	KRT8P47	1	44103306	44104703	processed_pseudogene	2,30	0,2161218
ENSG00000277420	ENSG00000277420	1	146514471	146525289	lincRNA	2,30	0,2161218
ENSG00000234355	FTH1P27	X	37505310	37505860	unprocessed_pseudogene	2,30	0,2161218
ENSG00000272145	NFYC-AS1	1	40690380	40692066	antisense	2,29	0
ENSG00000272842	ENSG00000272842	9	19371386	19371945	antisense	2,29	1,40E-02
ENSG00000213526	SETP8	X	116963049	116963676	processed_pseudogene	2,29	0,1217903
ENSG00000265194	ENSG00000265194	9	21858910	21861926	antisense	2,29	6,27E-03
ENSG00000225075	ENSG00000225075	1	112693688	112696621	antisense	2,28	1,40E-02
ENSG00000226049	TLK2P1	17	34036681	34039872	processed_pseudogene	2,28	0
ENSG00000266171	ENSG00000266171	18	735746	737459	antisense	2,28	0
ENSG00000215154	ENSG00000215154	16	2603350	2630494	transcribed_unprocessed_pseudogene	2,28	9,18E-02
ENSG00000250734	ENSG00000250734	1	151612038	151613363	antisense	2,28	7,06E-02
ENSG00000224699	LAMTOR5-AS1	1	110347116	110443817	processed_transcript	2,27	2,95E-02
ENSG00000241213	ENSG00000241213	3	117678693	117690932	lincRNA	2,27	0,1836819
ENSG00000242670	ENSG00000242670	4	54221126	54221472	processed_pseudogene	2,27	0,1392851
ENSG00000264273	ENSG00000264273	17	18859354	18861466	antisense	2,26	4,86E-02
ENSG00000231663	ENSG00000231663	1	234372807	234373593	antisense	2,26	0,0358681
ENSG00000228623	ZNF883	9	112997120	113050043	lincRNA	2,25	0,0358681
ENSG00000214535	RPS15AP1	20	21166206	21166596	processed_pseudogene	2,25	0,1836819
ENSG00000270532	PEBP1P2	2	85341281	85341838	processed_pseudogene	2,24	0,0358681
ENSG00000245293	ENSG00000245293	4	107863479	107978799	antisense	2,24	2,95E-02
ENSG00000247572	CKMT2-AS1	5	81204084	81301560	antisense	2,23	0,0358681
ENSG00000163016	ALMS1P	2	73644919	73685576	transcribed_unprocessed_pseudogene	2,23	9,18E-02
ENSG00000259813	ENSG00000259813	16	88696501	88698610	antisense	2,23	0,2161218
ENSG00000237854	LINC00674	17	68101908	68115518	transcribed_unprocessed_pseudogene	2,22	0
ENSG00000240005	ENSG00000240005	4	26859806	26860599	antisense	2,21	0,2161218
ENSG00000214439	FAM185BP	7	77083886	77122116	unprocessed_pseudogene	2,21	0
ENSG00000231989	PPP1R2P3	5	156850563	156851174	processed_pseudogene	2,21	6,27E-03
ENSG00000128262	POM121L9P	22	24251828	24265525	transcribed_unprocessed_pseudogene	2,20	0,2161218
ENSG00000232218	ENSG00000232218	22	32386668	32386868	unprocessed_pseudogene	2,20	0,2161218
ENSG00000273243	ENSG00000273243	22	45155539	45156011	lincRNA	2,20	1,40E-02
ENSG00000225330	ENSG00000225330	21	39630271	39726085	sense_overlapping	2,20	1,40E-02
ENSG00000275142	ENSG00000275142	20	19871891	19872284	lincRNA	2,20	0,2161218
ENSG00000248830	ZNF807	19	34542462	34545395	unprocessed_pseudogene	2,20	0,2161218
ENSG00000260599	ENSG00000260599	19	22520995	22527949	sense_intronic	2,20	7,06E-02
ENSG00000267045	ENSG00000267045	19	45238632	45245370	lincRNA	2,20	0,2161218
ENSG00000267778	ENSG00000267778	19	1238179	1239522	lincRNA	2,20	7,06E-02

ENSG00000271366	ENSG00000271366	19	35251440	35253639	antisense		2,20	0,2161218
ENSG00000266256	LINC00683	18	76615212	76625940	lincRNA		2,20	0,2161218
ENSG00000261320	ENSG00000261320	16	67430667	67431464	lincRNA		2,20	7,06E-02
ENSG00000261442	ENSG00000261442	16	4634329	4640623	antisense		2,20	0,2161218
ENSG00000261567	ENSG00000261567	16	85357463	85359449	lincRNA		2,20	7,06E-02
ENSG00000261265	ENSG00000261265	15	57527187	57529009	lincRNA		2,20	7,06E-02
ENSG00000276524	ENSG00000276524	15	56887107	56888219	lincRNA		2,20	0,2161218
ENSG00000251602	ENSG00000251602	14	105416884	105419739	antisense		2,20	0,0358681
ENSG00000258896	SCOCP1	14	60158976	60159324	processed_pseudogene		2,20	0,2161218
ENSG00000240441	ENSG00000240441	12	107719599	107720133	processed_pseudogene		2,20	0,2161218
ENSG00000249628	LINC00942	12	1500525	1507318	lincRNA		2,20	7,06E-02
ENSG00000257735	ENSG00000257735	12	48350945	48442411	antisense		2,20	0,2161218
ENSG00000257894	ENSG00000257894	12	79341205	79503396	lincRNA		2,20	0,2161218
ENSG00000270095	ENSG00000270095	12	123971457	123971714	sense_intronic		2,20	0,2161218
ENSG00000255409	RSF1-IT1	11	77738680	77739568	sense_intronic		2,20	7,06E-02
ENSG00000255710	ENSG00000255710	11	113820856	113821885	processed_pseudogene		2,20	0,2161218
ENSG00000204365	C10orf126	10	28846408	28881898	lincRNA		2,20	7,06E-02
ENSG00000273372	SFTPD-AS1	10	79968213	79973213	antisense		2,20	0,2161218
ENSG00000225684	FAM225B	9	113103338	113111500	lincRNA		2,20	7,06E-02
ENSG00000231967	ENSG00000231967	9	5356312	5356525	processed_pseudogene		2,20	7,06E-02
ENSG00000235641	LINC00484	9	91159573	91165658	lincRNA		2,20	1,40E-02
ENSG00000236507	ENSG00000236507	9	89801727	89803061	lincRNA		2,20	0,2161218
ENSG00000237886	NALT1	9	136546212	136549893	antisense		2,20	0,2161218
ENSG00000254034	ENSG00000254034	8	28798142	28799469	antisense		2,20	1,40E-02
ENSG00000254362	ENSG00000254362	8	26440491	26442595	sense_intronic		2,20	0,2161218
ENSG00000254973	ENSG00000254973	8	143758153	143771822	lincRNA		2,20	0,2161218
ENSG00000270923	TAS2R6P	7	141787815	141788640	processed_pseudogene		2,20	0,2161218
ENSG00000215156	ENSG00000215156	5	34190056	34193653	processed_pseudogene		2,20	0,2161218
ENSG00000249899	ENSG00000249899	5	52932419	52990278	antisense		2,20	0,2161218
ENSG00000250635	ENSG00000250635	5	139648999	139649728	lincRNA		2,20	1,40E-02
ENSG00000224786	CETN4P	4	122730548	122732193	unitary_pseudogene		2,20	1,40E-02
ENSG00000249347	ENSG00000249347	4	8990455	8990806	processed_pseudogene		2,20	7,06E-02
ENSG00000239462	ENSG00000239462	3	98902424	99018562	antisense		2,20	7,06E-02
ENSG00000248773	ENSG00000248773	3	140972744	140973255	sense_intronic		2,20	7,06E-02
ENSG00000224132	ENSG00000224132	2	237257091	237257676	lincRNA		2,20	0,2161218
ENSG00000231903	ENSG00000231903	2	202178660	202179391	antisense		2,20	0,2161218
ENSG00000270019	ENSG00000270019	2	113888203	113889750	lincRNA		2,20	0
ENSG00000270659	ENSG00000270659	2	213152970	213153659	lincRNA		2,20	0,2161218
ENSG00000225762	LINC01389	1	47380928	47408477	antisense		2,20	7,06E-02
ENSG00000235121	ENSG00000235121	1	201723294	201737506	antisense		2,20	1,40E-02
ENSG00000237457	LINC01351	1	190478551	190480735	lincRNA		2,20	0,2161218
ENSG00000239670	ENSG00000239670	1	32986952	32988233	unprocessed_pseudogene		2,20	7,06E-02
ENSG00000269998	ENSG00000269998	1	97933474	97941746	lincRNA		2,20	0,2161218
ENSG00000235806	ENSG00000235806	X	38770331	38799658	lincRNA		2,20	7,06E-02
ENSG00000259419	HNRNPCP3	15	79236332	79237206	processed_pseudogene		2,20	0,0358681
ENSG00000224769	MUC20P1	3	195614947	195620233	unprocessed_pseudogene		2,20	9,18E-02
ENSG00000231043	ENSG00000231043	2	58460292	58462032	processed_pseudogene		2,20	0,1836819
ENSG00000213250	RBMS2P1	12	94423744	94424969	processed_pseudogene		2,19	0
ENSG00000238171	ENSG00000238171	2	181076051	181105968	lincRNA		2,19	0,1217903
ENSG00000248724	NPHP3-AS1	3	132721750	132874223	antisense		2,19	6,27E-03
ENSG00000221817	PPP3CB-AS1	10	73495525	73520070	processed_transcript		2,18	0
ENSG00000228889	UBAC2-AS1	13	99196377	99200710	lincRNA		2,18	0,1392851
ENSG00000239917	RPS10P16	8	117523271	117524061	unprocessed_pseudogene		2,18	0,1836819
ENSG00000249955	ENSG00000249955	4	169981747	169993806	antisense		2,18	0,1836819
ENSG00000247271	ZBED5-AS1	11	10858259	10906802	antisense		2,18	0,1392851
ENSG00000237037	NDUFA6-AS1	22	42090931	42137742	processed_transcript		2,17	0,1392851
ENSG00000214892	USP8P1	6	31275572	31278754	processed_pseudogene		2,17	0,0358681
ENSG00000269984	ENSG00000269984	3	169777192	169780334	antisense		2,17	1,40E-02

ENSG00000236165	PRADC1P1	3	36976316	36976840	processed_pseudogene	2,16	0,1187106
ENSG00000265415	ENSG00000265415	17	59202677	59203829	antisense	2,16	4,86E-02
ENSG00000271218	ENSG00000271218	6	53918974	53920788	antisense	2,16	0,4036702
ENSG00000259438	ENSG00000259438	15	52010999	52019095	lincRNA	2,15	0,2161218
ENSG00000231616	ENSG00000231616	9	83831586	83868532	antisense	2,15	3,30E-03
ENSG00000237513	ENSG00000237513	7	104941063	104962334	lincRNA	2,15	0,1836819
ENSG00000225215	SMARCE1P1	11	107403404	107404589	processed_pseudogene	2,15	1,40E-02
ENSG00000236493	EIF2S2P3	10	92668745	92669743	processed_pseudogene	2,15	0,0358681
ENSG00000229431	ENSG00000229431	1	43385113	43389155	antisense	2,14	4,86E-02
ENSG00000257386	ENSG00000257386	12	74538145	74538633	antisense	2,14	3,30E-03
ENSG00000234740	ENSG00000234740	9	13274510	13279187	sense_intronic	2,14	0,3565078
ENSG00000250877	ENSG00000250877	4	72323028	72330751	antisense	2,14	2,21E-02
ENSG00000237214	ENSG00000237214	22	41064052	41064633	processed_pseudogene	2,13	0,3565078
ENSG00000267322	SNHG22	18	49814023	49851059	antisense	2,13	2,95E-02
ENSG00000177855	CACYBPP2	2	183607442	183608119	processed_pseudogene	2,13	2,95E-02
ENSG00000253144	ENSG00000253144	5	179082680	179083194	processed_pseudogene	2,13	0,1836819
ENSG00000227253	ENSG00000227253	10	32266289	32269474	antisense	2,13	0,3318209
ENSG00000237307	SRRM1P3	X	136976417	136985705	processed_pseudogene	2,12	2,21E-02
ENSG00000260461	ENSG00000260461	10	103877374	103879761	sense_overlapping	2,12	6,27E-03
ENSG00000230673	PABPC1P3	X	74583088	74583546	processed_pseudogene	2,12	2,21E-02
ENSG00000247675	LRP4-AS1	11	46846412	46874396	antisense	2,12	7,06E-02
ENSG00000267459	ENSG00000267459	19	56377037	56377792	transcribed_processed_pseudogene	2,12	4,86E-02
ENSG00000262920	ENSG00000262920	17	321392	322453	antisense	2,11	1,40E-02
ENSG00000224324	THAP5P1	7	158623188	158623449	processed_pseudogene	2,11	3,30E-03
ENSG00000237945	LINC00649	21	33915534	33977691	antisense	2,11	0,0358681
ENSG00000161132	ENSG00000161132	22	20299760	20313216	unprocessed_pseudogene	2,10	0,2161218
ENSG00000230637	ENSG00000230637	22	25327252	25339975	unprocessed_pseudogene	2,10	1,40E-02
ENSG00000235328	ENSG00000235328	22	17093612	17093939	processed_pseudogene	2,10	0
ENSG00000261251	ENSG00000261251	22	43812456	43817394	antisense	2,10	0,2161218
ENSG00000273164	ENSG00000273164	22	19023056	19023550	sense_intronic	2,10	1,40E-02
ENSG00000273366	ENSG00000273366	22	42278188	42278846	lincRNA	2,10	0,2161218
ENSG00000231119	ENSG00000231119	20	47352561	47354633	antisense	2,10	1,40E-02
ENSG00000262497	FAM187B2P	19	35232291	35233003	unprocessed_pseudogene	2,10	0,2161218
ENSG00000269085	ENSG00000269085	19	16565551	16566330	antisense	2,10	7,06E-02
ENSG00000267758	ENSG00000267758	17	42268587	42269807	antisense	2,10	0,2161218
ENSG00000261056	ENSG00000261056	16	53298224	53299792	transcribed_processed_pseudogene	2,10	7,06E-02
ENSG00000261084	PPP1R1AP2	16	35912120	35912516	processed_pseudogene	2,10	7,06E-02
ENSG00000261469	ENSG00000261469	16	68236845	68237667	sense_intronic	2,10	0,2161218
ENSG00000262312	ENSG00000262312	16	3542739	3545785	antisense	2,10	0
ENSG00000225151	GOLGA2P7	15	84199311	84230136	transcribed_unprocessed_pseudogene	2,10	1,40E-02
ENSG00000228036	HSPD1P9	13	37993085	37994720	processed_pseudogene	2,10	0,2161218
ENSG00000235076	GAPDHP52	13	21358373	21359314	processed_pseudogene	2,10	7,06E-02
ENSG00000257886	ENSG00000257886	12	105072486	105074021	antisense	2,10	7,06E-02
ENSG00000257999	ENSG00000257999	12	105102472	105107179	antisense	2,10	0,2161218
ENSG00000258096	ENSG00000258096	12	46371463	46373778	antisense	2,10	0
ENSG00000237233	TMEM26-AS1	10	61452639	61481956	antisense	2,10	7,06E-02
ENSG00000272489	ENSG00000272489	10	79663192	79664786	sense_intronic	2,10	7,06E-02
ENSG00000272912	ENSG00000272912	10	102914585	102915404	lincRNA	2,10	0,2161218
ENSG00000264527	ENSG00000264527	9	136249971	136251205	lincRNA	2,10	7,06E-02
ENSG00000273399	ENSG00000273399	9	14586766	14587326	lincRNA	2,10	0,2161218
ENSG00000275017	ENSG00000275017	9	88391362	88391832	processed_pseudogene	2,10	1,40E-02
ENSG00000249258	ENSG00000249258	8	17131181	17149789	lincRNA	2,10	0,2161218
ENSG00000272425	ENSG00000272425	8	81275399	81277570	lincRNA	2,10	0,2161218
ENSG00000223969	ENSG00000223969	7	90590619	90597353	antisense	2,10	0,2161218

ENSG00000251451	GTF2IP9	7	66407288	66409239	unprocessed_pseudogene	2,10	7,06E-02
ENSG00000274080	ENSG00000274080	7	73609262	73611502	sense_intronic	2,10	0,2161218
ENSG00000216990	HSPD1P10	6	87298772	87300447	processed_pseudogene	2,10	0,2161218
ENSG00000227803	ENSG00000227803	6	20212087	20317739	antisense	2,10	1,40E-02
ENSG00000234206	ENSG00000234206	6	107509803	107511721	antisense	2,10	0,2161218
ENSG00000271788	ENSG00000271788	5	43006733	43007543	lincRNA	2,10	0,2161218
ENSG00000260651	ENSG00000260651	4	102500841	102501319	antisense	2,10	1,40E-02
ENSG00000272646	ENSG00000272646	4	182881777	182882203	lincRNA	2,10	0,2161218
ENSG00000272885	ENSG00000272885	4	416118	416537	lincRNA	2,10	7,06E-02
ENSG00000227245	ENSG00000227245	3	43087479	43088068	lincRNA	2,10	0,2161218
ENSG00000228350	CELSR3-AS1	3	48663776	48669174	antisense	2,10	0,2161218
ENSG00000241120	HMGN1P8	3	167694355	167694642	processed_pseudogene	2,10	0,2161218
ENSG00000271716	ENSG00000271716	3	11607184	11610748	antisense	2,10	0,2161218
ENSG00000233392	ENSG00000233392	2	240954617	240967451	processed_transcript	2,10	7,06E-02
ENSG00000233416	ENSG00000233416	2	20606280	20606654	processed_pseudogene	2,10	0,2161218
ENSG00000234842	MTCO2P16	2	201549631	201550307	unprocessed_pseudogene	2,10	7,06E-02
ENSG00000225891	ENSG00000225891	1	26462756	26467282	antisense	2,10	7,06E-02
ENSG00000226026	ENSG00000226026	1	95163219	95233982	antisense	2,10	7,06E-02
ENSG00000227712	ENSG00000227712	1	119260265	119327303	lincRNA	2,10	7,06E-02
ENSG00000231080	ENSG00000231080	1	66826942	66828558	antisense	2,10	1,40E-02
ENSG00000232862	ENSG00000232862	1	36080066	36084026	transcribed_processed_pseudogene	2,10	7,06E-02
ENSG00000234601	CHRM3-AS1	1	239898016	239899872	antisense	2,10	7,06E-02
ENSG00000254154	ENSG00000254154	1	177928788	178038007	processed_transcript	2,10	0,2161218
ENSG00000270115	ENSG00000270115	1	33261212	33261680	lincRNA	2,10	7,06E-02
ENSG00000278504	UPF3BP2	X	119822906	119823043	unprocessed_pseudogene	2,10	0,2161218
ENSG00000226632	UBE2V1P1	20	3361880	3362324	processed_pseudogene	2,10	6,27E-03
ENSG00000226361	TERF1P5	13	18680494	18681700	processed_pseudogene	2,09	0,0358681
ENSG00000181524	RPL24P4	6	42956345	42956765	processed_pseudogene	2,09	0,4332376
ENSG00000213938	SEPHS1P6	2	199659551	199660729	processed_pseudogene	2,09	2,21E-02
ENSG00000234694	ENSG00000234694	1	43354684	43358658	antisense	2,09	0,1836819
ENSG00000225695	HNRNPA1P35	2	201063089	201064051	processed_pseudogene	2,08	9,18E-02
ENSG00000205664	ENSG00000205664	X	3817528	3843857	lincRNA	2,08	6,27E-03
ENSG00000257027	ENSG00000257027	12	9658567	9662085	sense_intronic	2,07	6,27E-03
ENSG00000280604	PCBP3-OT1	21	45914296	45919483	sense_overlapping	2,07	0,2746192
ENSG00000224905	ENSG00000224905	21	14027421	14144468	antisense	2,07	0,0358681
ENSG00000282772	ENSG00000282772	10	102845761	102854513	antisense	2,07	2,21E-02
ENSG00000231504	NMD3P2	7	56502284	56503044	processed_pseudogene	2,06	7,06E-02
ENSG00000235720	ENSG00000235720	7	63893286	63894641	processed_pseudogene	2,05	3,30E-03
ENSG00000239718	HLTF-AS1	3	149086332	149102823	antisense	2,05	0
ENSG00000265749	ENSG00000265749	17	8365563	8381328	antisense	2,04	9,18E-02
ENSG00000226309	SMARCE1P2	6	84429584	84430802	processed_pseudogene	2,04	4,86E-02
ENSG00000272195	ENSG00000272195	1	244969350	244971088	antisense	2,03	4,86E-02
ENSG00000279762	ENSG00000279762	17	32423971	32446038	antisense	2,03	0,3565078
ENSG00000229808	ENSG00000229808	1	161890833	161892196	transcribed_processed_pseudogene	2,03	0,3318209
ENSG00000248596	ENSG00000248596	5	176143085	176199295	lincRNA	2,02	0,1836819
ENSG00000253618	GRPEL2-AS1	5	149348116	149357642	antisense	2,02	0,1217903
ENSG00000234478	ENSG00000234478	1	226148003	226155071	antisense	2,02	1,40E-02
ENSG00000255857	PXN-AS1	12	120201291	120213138	antisense	2,02	2,21E-02
ENSG00000278932	ENSG00000278932	21	9325013	9368775	lincRNA	2,02	0,1187106
ENSG00000230360	DDX10P2	9	82086155	82088768	processed_pseudogene	2,01	0,1187106
ENSG00000219928	ENSG00000219928	9	87525302	87525475	processed_pseudogene	2,01	4,86E-02
ENSG00000245317	ENSG00000245317	5	179859013	179861283	lincRNA	2,01	0,2161218
ENSG00000247121	ENSG00000247121	5	96814028	96935809	antisense	2,01	4,86E-02
ENSG00000263266	RPS7P1	17	28467822	28468406	processed_pseudogene	2,00	2,21E-02
ENSG00000231201	ENSG00000231201	21	14971470	14992854	antisense	2,00	0,2161218
ENSG00000236545	ENSG00000236545	21	41870633	41872054	antisense	2,00	0,2161218

ENSG00000237989	ENSG00000237989	21	43358147	43362349	lincRNA	2,00	1,40E-02
ENSG00000275784	ENSG00000275784	20	33989480	33991818	lincRNA	2,00	4,86E-02
ENSG00000267117	ENSG00000267117	19	55694118	55694558	antisense	2,00	0,2161218
ENSG00000267264	ENSG00000267264	19	27803538	27804060	processed_pseudogene	2,00	7,06E-02
ENSG00000267689	ENSG00000267689	19	55705978	55706172	processed_pseudogene	2,00	0,2161218
ENSG00000268027	ENSG00000268027	19	41545192	41555462	lincRNA	2,00	1,40E-02
ENSG00000268416	ENSG00000268416	19	20746923	20755250	lincRNA	2,00	0,2161218
ENSG00000228979	ENSG00000228979	17	63471604	63472093	processed_pseudogene	2,00	0,2161218
ENSG00000265683	ENSG00000265683	17	27351858	27352584	processed_pseudogene	2,00	0,2161218
ENSG00000206168	ENSG00000206168	16	231134	231472	processed_pseudogene	2,00	2,21E-02
ENSG00000221819	GAS8-AS1	16	90028908	90029367	antisense	2,00	7,06E-02
ENSG00000240793	UBA52P8	16	48334471	48334854	processed_pseudogene	2,00	0,3565078
ENSG00000261227	ENSG00000261227	16	73232055	73233970	lincRNA	2,00	0,2161218
ENSG00000262488	ENSG00000262488	16	10840384	10841544	processed_pseudogene	2,00	0,2161218
ENSG00000224441	ENSG00000224441	15	90932586	90935130	antisense	2,00	0,2161218
ENSG00000274294	ENSG00000274294	15	99416584	99417204	lincRNA	2,00	0,2161218
ENSG00000275601	ENSG00000275601	15	43642389	43643023	sense_intronic	2,00	0,2161218
ENSG00000180189	HMGB1P14	14	58284773	58285363	processed_pseudogene	2,00	7,06E-02
ENSG00000251002	ENSG00000251002	14	22380680	22482959	processed_transcript	2,00	7,06E-02
ENSG00000258413	ENSG00000258413	14	55262767	55272075	lincRNA	2,00	0,4332376
ENSG00000258450	ENSG00000258450	14	49629291	49629890	sense_intronic	2,00	7,06E-02
ENSG00000275198	ENSG00000275198	14	90383365	90387973	lincRNA	2,00	1,40E-02
ENSG00000272274	LINC00551	13	106617810	106631359	lincRNA	2,00	0,2161218
ENSG00000214659	KRT8P26	11	65726939	65728214	processed_pseudogene	2,00	0,1836819
ENSG00000256443	ENSG00000256443	11	61539516	61542259	lincRNA	2,00	0,2161218
ENSG00000227518	ENSG00000227518	9	27657	30891	antisense	2,00	7,06E-02
ENSG00000244757	ENSG00000244757	9	116288618	116318689	antisense	2,00	7,06E-02
ENSG00000254231	ENSG00000254231	8	86333274	86343314	processed_transcript	2,00	0,2161218
ENSG00000225173	ENSG00000225173	6	28837869	28839006	lincRNA	2,00	0,2161218
ENSG00000227885	ENSG00000227885	6	53740749	53747022	lincRNA	2,00	1,40E-02
ENSG00000261080	ENSG00000261080	6	45573346	45576770	lincRNA	2,00	7,06E-02
ENSG00000272009	ENSG00000272009	6	28078792	28081130	antisense	2,00	0,2161218
ENSG00000249129	SUDS3P1	5	177971316	177972281	processed_pseudogene	2,00	0
ENSG00000250900	ENSG00000250900	5	181195496	181203103	antisense	2,00	0,2161218
ENSG00000229585	RPL21P44	4	53986587	53987058	processed_pseudogene	2,00	0,2161218
ENSG00000249138	ENSG00000249138	4	184798296	184799046	transcribed_processed_pseudogene	2,00	0,2161218
ENSG00000251155	SEPT14P4	4	118640673	118640858	processed_pseudogene	2,00	0,2161218
ENSG00000254094	ENSG00000254094	4	1356581	1358075	sense_intronic	2,00	0,2161218
ENSG00000205955	HSP90AA5P	3	184115352	184117898	transcribed_processed_pseudogene	2,00	0,2161218
ENSG00000233912	ENSG00000233912	3	5156905	5187329	antisense	2,00	0,2161218
ENSG00000235904	RBMS3-AS3	3	29264194	29290726	antisense	2,00	0,2161218
ENSG00000240497	ENSG00000240497	3	171468084	171469633	lincRNA	2,00	0,2161218
ENSG00000241622	RARRES2P1	3	75658534	75659014	processed_pseudogene	2,00	0,2161218
ENSG00000281392	LINC00506	3	87089129	87157069	lincRNA	2,00	7,06E-02
ENSG00000234350	ENSG00000234350	2	170723086	170770768	lincRNA	2,00	0,2161218
ENSG00000235267	ENSG00000235267	2	27455156	27455862	processed_pseudogene	2,00	0,2161218
ENSG00000235724	ENSG00000235724	2	161222785	161308303	antisense	2,00	0,3318209
ENSG00000153363	LINC00467	1	211382803	211435333	lincRNA	2,00	2,21E-02
ENSG00000227066	ENSG00000227066	1	20154338	20160568	lincRNA	2,00	0,2161218
ENSG00000227135	GCSAML-AS1	1	247524679	247526752	antisense	2,00	0,2161218
ENSG00000232678	SPTLC1P4	1	152077952	152078211	processed_pseudogene	2,00	7,06E-02
ENSG00000235613	NSRP1P1	1	77847110	77848802	processed_pseudogene	2,00	0,0358681
ENSG00000235795	ENSG00000235795	1	100995473	100996260	antisense	2,00	0,2161218
ENSG00000236947	ENSG00000236947	1	156446412	156456577	lincRNA	2,00	0,2161218
ENSG00000273059	ENSG00000273059	1	148011799	148012228	lincRNA	2,00	0,2161218
ENSG00000271826	PLS3-AS1	X	115518182	115562731	antisense	2,00	0,2161218
ENSG00000274162	SNX18P1Y	X	11178306	11178816	processed_pseudogene	2,00	0,2161218

Supplementary Table S5: Lists of eQTL-linked lncRNA and mRNA pairs in CCs and MII oocytes

eQTL-linked lncRNAs and mRNA pairs in CCs		eQTL-linked lncRNAs and mRNA pairs in MII oocytes	
ENSG00000224460	ENSG00000001036	ENSG00000237813	ENSG00000001626
ENSG00000229036	ENSG00000001036	ENSG00000248360	ENSG00000004468
ENSG00000278206	ENSG00000001036	ENSG00000236453	ENSG00000004948
ENSG00000230845	ENSG00000001084	ENSG00000230333	ENSG00000005108
ENSG00000231683	ENSG00000001084	ENSG00000229618	ENSG00000006747
ENSG00000249379	ENSG00000001084	ENSG00000264067	ENSG00000006788
ENSG00000235016	ENSG00000001617	ENSG00000273388	ENSG00000006788
ENSG00000188693	ENSG00000001630	ENSG00000238098	ENSG00000007038
ENSG00000256789	ENSG00000002330	ENSG00000179136	ENSG00000007174
ENSG00000257086	ENSG00000002330	ENSG00000173811	ENSG00000008324
ENSG00000265784	ENSG00000002834	ENSG00000238098	ENSG00000010539
ENSG00000266101	ENSG00000002834	ENSG00000237400	ENSG00000011426
ENSG00000263412	ENSG00000002919	ENSG00000282199	ENSG00000012048
ENSG00000263766	ENSG00000002919	ENSG00000239219	ENSG00000013293
ENSG00000164845	ENSG00000003056	ENSG00000231294	ENSG00000021826
ENSG00000215241	ENSG00000003056	ENSG00000223586	ENSG00000028839
ENSG00000237248	ENSG00000003056	ENSG00000227531	ENSG00000030304
ENSG00000256069	ENSG00000003056	ENSG00000225548	ENSG00000033867
ENSG00000256552	ENSG00000003056	ENSG00000229017	ENSG00000034693
ENSG00000256673	ENSG00000003056	ENSG00000152931	ENSG00000035499
ENSG00000222019	ENSG00000003249	ENSG00000254319	ENSG00000036448
ENSG00000223959	ENSG00000003249	ENSG00000245248	ENSG00000036672
ENSG00000260259	ENSG00000003249	ENSG00000254740	ENSG00000036672
ENSG00000226312	ENSG00000003400	ENSG00000253968	ENSG00000037241
ENSG00000226312	ENSG00000003402	ENSG00000260798	ENSG00000039068
ENSG00000224063	ENSG00000003436	ENSG00000267112	ENSG00000041353
ENSG00000227227	ENSG00000003436	ENSG00000233009	ENSG00000043355
ENSG00000235848	ENSG00000003509	ENSG00000259712	ENSG00000047346
ENSG00000235016	ENSG00000003756	ENSG00000236318	ENSG00000048052
ENSG00000249258	ENSG00000003989	ENSG00000248994	ENSG00000049656
ENSG00000265254	ENSG00000004139	ENSG00000224367	ENSG00000049759
ENSG00000265254	ENSG00000004142	ENSG00000267593	ENSG00000049759
ENSG00000206417	ENSG00000004399	ENSG00000231512	ENSG00000054282
ENSG00000228252	ENSG00000004399	ENSG00000224260	ENSG00000054392
ENSG00000248916	ENSG00000004399	ENSG00000259076	ENSG00000054654
ENSG00000225313	ENSG00000004455	ENSG00000257191	ENSG00000058272
ENSG00000236065	ENSG00000004455	ENSG00000257474	ENSG00000058272
ENSG00000239670	ENSG00000004455	ENSG00000248901	ENSG00000058729
ENSG00000235049	ENSG00000004478	ENSG00000250331	ENSG00000058729
ENSG00000246627	ENSG00000004478	ENSG00000251513	ENSG00000058729
ENSG00000235016	ENSG00000004534	ENSG00000238098	ENSG00000059145
ENSG00000267049	ENSG00000004776	ENSG00000261713	ENSG00000059145
ENSG00000267698	ENSG00000004776	ENSG00000179818	ENSG00000059728
ENSG00000267049	ENSG00000004777	ENSG00000256209	ENSG00000060709
ENSG00000267874	ENSG00000004777	ENSG00000260798	ENSG00000062038

ENSG00000231170	ENSG00000004799
ENSG00000235016	ENSG00000004838
ENSG00000231170	ENSG00000004864
ENSG00000231764	ENSG00000004864
ENSG00000214188	ENSG00000004866
ENSG00000226367	ENSG00000004866
ENSG00000243243	ENSG00000004866
ENSG00000267419	ENSG00000005007
ENSG00000269145	ENSG00000005007
ENSG00000273654	ENSG00000005007
ENSG00000257261	ENSG00000005175
ENSG00000257735	ENSG00000005175
ENSG00000260510	ENSG00000005187
ENSG00000189149	ENSG00000005189
ENSG00000260510	ENSG00000005189
ENSG00000230074	ENSG00000005238
ENSG00000262879	ENSG00000005243
ENSG00000264701	ENSG00000005243
ENSG00000266341	ENSG00000005243
ENSG00000243193	ENSG00000005249
ENSG00000265148	ENSG00000005379
ENSG00000237737	ENSG00000005448
ENSG00000257800	ENSG00000005448
ENSG00000182165	ENSG00000005469
ENSG00000228393	ENSG00000005483
ENSG00000205485	ENSG00000005486
ENSG00000205534	ENSG00000005844
ENSG00000260494	ENSG00000005844
ENSG00000260911	ENSG00000005844
ENSG00000261346	ENSG00000005844
ENSG00000261474	ENSG00000005844
ENSG00000234230	ENSG00000005889
ENSG00000233382	ENSG00000005893
ENSG00000258545	ENSG00000005893
ENSG00000268030	ENSG00000006015
ENSG00000268199	ENSG00000006016
ENSG00000234494	ENSG00000006025
ENSG00000263412	ENSG00000006025
ENSG00000214425	ENSG00000006062
ENSG00000267121	ENSG00000006062
ENSG00000277089	ENSG00000006125
ENSG00000205890	ENSG00000006194
ENSG00000260565	ENSG00000006194
ENSG00000262370	ENSG00000006194
ENSG00000260467	ENSG00000006210
ENSG00000250107	ENSG00000006282
ENSG00000251239	ENSG00000006282
ENSG00000229980	ENSG00000006283

ENSG00000268686	ENSG00000063127
ENSG00000267606	ENSG00000063245
ENSG00000267710	ENSG00000063245
ENSG00000261286	ENSG00000064270
ENSG00000229855	ENSG00000064692
ENSG00000250328	ENSG00000064692
ENSG00000238098	ENSG00000065057
ENSG00000261713	ENSG00000065057
ENSG00000230131	ENSG00000065154
ENSG00000265975	ENSG00000065320
ENSG00000239523	ENSG00000065534
ENSG00000237943	ENSG00000065675
ENSG00000227165	ENSG00000066468
ENSG00000257165	ENSG00000067715
ENSG00000257191	ENSG00000067715
ENSG00000258084	ENSG00000067715
ENSG00000258220	ENSG00000067715
ENSG00000257165	ENSG00000067798
ENSG00000258084	ENSG00000067798
ENSG00000250167	ENSG00000069018
ENSG00000249364	ENSG00000069020
ENSG00000251391	ENSG00000069020
ENSG00000257004	ENSG00000070018
ENSG00000271119	ENSG00000071539
ENSG00000241956	ENSG00000072571
ENSG00000236333	ENSG00000072657
ENSG00000177112	ENSG00000072952
ENSG00000225764	ENSG00000073282
ENSG00000255308	ENSG00000074319
ENSG00000247213	ENSG00000075035
ENSG00000232455	ENSG00000075914
ENSG00000228791	ENSG00000077092
ENSG00000237838	ENSG00000077092
ENSG00000229291	ENSG00000077585
ENSG00000226140	ENSG00000077943
ENSG00000253634	ENSG00000079102
ENSG00000253901	ENSG00000079102
ENSG00000246662	ENSG00000079112
ENSG00000253773	ENSG00000079112
ENSG00000223786	ENSG00000080007
ENSG00000231332	ENSG00000080007
ENSG00000246316	ENSG00000080709
ENSG00000248245	ENSG00000081059
ENSG00000250994	ENSG00000081059
ENSG00000254024	ENSG00000083307
ENSG00000261848	ENSG00000083454
ENSG00000261848	ENSG00000083457
ENSG00000261898	ENSG00000083457

ENSG00000250107	ENSG00000006283
ENSG00000215154	ENSG00000006327
ENSG00000260565	ENSG00000006327
ENSG00000160172	ENSG00000006534
ENSG00000255306	ENSG00000006534
ENSG00000220804	ENSG00000006607
ENSG00000267551	ENSG00000006638
ENSG00000269486	ENSG00000006712
ENSG00000188185	ENSG00000006715
ENSG00000229851	ENSG00000006756
ENSG00000266903	ENSG00000007047
ENSG00000269815	ENSG00000007080
ENSG00000273654	ENSG00000007080
ENSG00000130612	ENSG00000007129
ENSG00000239736	ENSG00000007129
ENSG00000267107	ENSG00000007129
ENSG00000268027	ENSG00000007129
ENSG00000270164	ENSG00000007129
ENSG00000262791	ENSG00000007168
ENSG00000266527	ENSG00000007171
ENSG00000264031	ENSG00000007202
ENSG00000264808	ENSG00000007202
ENSG00000266527	ENSG00000007202
ENSG00000267259	ENSG00000007202
ENSG00000233635	ENSG00000007314
ENSG00000263644	ENSG00000007314
ENSG00000236829	ENSG00000007376
ENSG00000261691	ENSG00000007376
ENSG00000234769	ENSG00000007384
ENSG00000236829	ENSG00000007384
ENSG00000260316	ENSG00000007384
ENSG00000261659	ENSG00000007384
ENSG00000236829	ENSG00000007392
ENSG00000261659	ENSG00000007392
ENSG00000236829	ENSG00000007516
ENSG00000229106	ENSG00000007968
ENSG00000227775	ENSG00000008130
ENSG00000230092	ENSG00000008130
ENSG00000237491	ENSG00000008130
ENSG00000268575	ENSG00000008130
ENSG00000233635	ENSG00000008283
ENSG00000265702	ENSG00000008283
ENSG00000229980	ENSG00000008294
ENSG00000228350	ENSG00000008300
ENSG00000260565	ENSG00000008516
ENSG00000261971	ENSG00000008516
ENSG00000263072	ENSG00000008516
ENSG00000261971	ENSG00000008517

ENSG00000262670	ENSG00000083457
ENSG00000262921	ENSG00000083457
ENSG00000228802	ENSG00000085449
ENSG00000250195	ENSG00000085871
ENSG00000261713	ENSG00000086506
ENSG00000229291	ENSG00000086619
ENSG00000204666	ENSG00000086967
ENSG00000258535	ENSG00000087302
ENSG00000231290	ENSG00000087495
ENSG00000235590	ENSG00000087495
ENSG00000237121	ENSG00000087495
ENSG00000254620	ENSG00000087495
ENSG00000253392	ENSG00000088256
ENSG00000277301	ENSG00000088325
ENSG00000232746	ENSG00000088726
ENSG00000251165	ENSG00000088926
ENSG00000257883	ENSG00000088992
ENSG00000257452	ENSG00000089169
ENSG00000261713	ENSG00000090581
ENSG00000261239	ENSG00000091651
ENSG00000257285	ENSG00000092036
ENSG00000257285	ENSG00000092054
ENSG00000234511	ENSG00000094755
ENSG00000253591	ENSG00000094755
ENSG00000234477	ENSG00000094804
ENSG00000225243	ENSG00000094963
ENSG00000230107	ENSG00000100162
ENSG00000188511	ENSG00000100253
ENSG00000233080	ENSG00000100281
ENSG00000279184	ENSG00000100281
ENSG00000279159	ENSG00000100330
ENSG00000257636	ENSG00000100473
ENSG00000258558	ENSG00000100473
ENSG00000259039	ENSG00000100567
ENSG00000258416	ENSG00000100629
ENSG00000259035	ENSG00000100629
ENSG00000258979	ENSG00000100749
ENSG00000258342	ENSG00000100906
ENSG00000234948	ENSG00000101115
ENSG00000132832	ENSG00000101443
ENSG00000264859	ENSG00000101695
ENSG00000227042	ENSG00000101871
ENSG00000235834	ENSG00000102054
ENSG00000233009	ENSG00000102452
ENSG00000276012	ENSG00000102466
ENSG00000276476	ENSG00000102678
ENSG00000261008	ENSG00000102984
ENSG00000260798	ENSG00000103044

ENSG00000260293	ENSG00000008710
ENSG00000262370	ENSG00000008710
ENSG00000184319	ENSG00000008735
ENSG00000245025	ENSG00000008853
ENSG00000253125	ENSG00000008853
ENSG00000254064	ENSG00000008853
ENSG00000232973	ENSG00000008869
ENSG00000235848	ENSG00000008869
ENSG00000236213	ENSG00000008869
ENSG00000233038	ENSG00000009335
ENSG00000231889	ENSG00000009413
ENSG00000271789	ENSG00000009413
ENSG00000272078	ENSG00000009724
ENSG00000224666	ENSG00000010030
ENSG00000246982	ENSG00000010030
ENSG00000224424	ENSG00000010256
ENSG00000215039	ENSG00000010292
ENSG00000219410	ENSG00000010295
ENSG00000214855	ENSG00000010310
ENSG00000266903	ENSG00000010310
ENSG00000267282	ENSG00000010310
ENSG00000242797	ENSG00000010322
ENSG00000180279	ENSG00000010361
ENSG00000269352	ENSG00000010361
ENSG00000235703	ENSG00000010404
ENSG00000238039	ENSG00000010404
ENSG00000241769	ENSG00000010404
ENSG00000205885	ENSG00000010610
ENSG00000215039	ENSG00000010610
ENSG00000168405	ENSG00000010704
ENSG00000228223	ENSG00000010704
ENSG00000235358	ENSG00000010803
ENSG00000260920	ENSG00000010803
ENSG00000281207	ENSG00000010803
ENSG00000225315	ENSG00000011009
ENSG00000264443	ENSG00000011009
ENSG00000242349	ENSG00000011021
ENSG00000233635	ENSG00000011028
ENSG00000263644	ENSG00000011028
ENSG00000265702	ENSG00000011028
ENSG00000236908	ENSG00000011105
ENSG00000246627	ENSG00000011105
ENSG00000258325	ENSG00000011105
ENSG00000265148	ENSG00000011143
ENSG00000265542	ENSG00000011143
ENSG00000236869	ENSG00000011198
ENSG00000273218	ENSG00000011243
ENSG00000229980	ENSG00000011258

ENSG00000260798	ENSG00000103056
ENSG00000260338	ENSG00000103174
ENSG00000274367	ENSG00000103355
ENSG00000259929	ENSG00000103528
ENSG00000256340	ENSG00000103534
ENSG00000260430	ENSG00000103540
ENSG00000260592	ENSG00000103540
ENSG00000259673	ENSG00000103599
ENSG00000259700	ENSG00000103995
ENSG00000253944	ENSG00000104213
ENSG00000235531	ENSG00000104313
ENSG00000254031	ENSG00000104313
ENSG00000254277	ENSG00000104313
ENSG00000235531	ENSG00000104321
ENSG00000253394	ENSG00000104327
ENSG00000253799	ENSG00000104327
ENSG00000254180	ENSG00000104327
ENSG00000249395	ENSG00000104369
ENSG00000253983	ENSG00000104369
ENSG00000254349	ENSG00000104369
ENSG00000249395	ENSG00000104381
ENSG00000254349	ENSG00000104381
ENSG00000253773	ENSG00000104413
ENSG00000253893	ENSG00000104626
ENSG00000254235	ENSG00000104626
ENSG00000254367	ENSG00000104626
ENSG00000253535	ENSG00000104722
ENSG00000248257	ENSG00000105732
ENSG00000223838	ENSG00000105849
ENSG00000243004	ENSG00000105849
ENSG00000243797	ENSG00000105865
ENSG00000234352	ENSG00000105894
ENSG00000223561	ENSG00000105928
ENSG00000254369	ENSG00000105991
ENSG00000254369	ENSG00000105996
ENSG00000223813	ENSG00000106069
ENSG00000233108	ENSG00000106415
ENSG00000230333	ENSG00000106443
ENSG00000272701	ENSG00000106484
ENSG00000181211	ENSG00000106588
ENSG00000231539	ENSG00000106588
ENSG00000231638	ENSG00000106588
ENSG00000232006	ENSG00000106588
ENSG00000227482	ENSG00000106927
ENSG00000229981	ENSG00000108018
ENSG00000203876	ENSG00000108055
ENSG00000231829	ENSG00000108242
ENSG00000234477	ENSG00000108244

ENSG00000229980	ENSG00000011260
ENSG00000175061	ENSG00000011295
ENSG00000227782	ENSG00000011295
ENSG00000266933	ENSG00000011304
ENSG00000186019	ENSG00000011422
ENSG00000257242	ENSG00000011465
ENSG00000266903	ENSG00000011478
ENSG00000205786	ENSG00000011600
ENSG00000267254	ENSG00000011600
ENSG00000267698	ENSG00000011600
ENSG00000180747	ENSG00000011638
ENSG00000275445	ENSG00000011638
ENSG00000267219	ENSG00000012124
ENSG00000267698	ENSG00000012124
ENSG00000235016	ENSG00000012171
ENSG00000204620	ENSG00000012211
ENSG00000224812	ENSG00000012779
ENSG00000231964	ENSG00000012779
ENSG00000237840	ENSG00000012779
ENSG00000248265	ENSG00000012822
ENSG00000258086	ENSG00000012822
ENSG00000214900	ENSG00000012983
ENSG00000259113	ENSG00000012983
ENSG00000242578	ENSG00000013297
ENSG00000269889	ENSG00000013297
ENSG00000267121	ENSG00000013306
ENSG00000240449	ENSG00000013374
ENSG00000258301	ENSG00000013523
ENSG00000258569	ENSG00000013523
ENSG00000259081	ENSG00000013523
ENSG00000232725	ENSG00000013563
ENSG00000280195	ENSG00000013563
ENSG00000183935	ENSG00000013583
ENSG00000247498	ENSG00000013583
ENSG00000255621	ENSG00000013583
ENSG00000235703	ENSG00000013619
ENSG00000243742	ENSG00000013725
ENSG00000256813	ENSG00000013725
ENSG00000245532	ENSG00000014216
ENSG00000254614	ENSG00000014216
ENSG00000255478	ENSG00000014216
ENSG00000257086	ENSG00000014216
ENSG00000248724	ENSG00000014257
ENSG00000223935	ENSG00000014641
ENSG00000220323	ENSG00000014914
ENSG00000277147	ENSG00000014914
ENSG00000232656	ENSG00000015171
ENSG00000237341	ENSG00000015285

ENSG00000267440	ENSG00000108849
ENSG00000282199	ENSG00000108852
ENSG00000227517	ENSG00000108984
ENSG00000267009	ENSG00000108984
ENSG00000248747	ENSG00000109445
ENSG00000248810	ENSG00000109445
ENSG00000250131	ENSG00000109674
ENSG00000251230	ENSG00000109762
ENSG00000250327	ENSG00000109794
ENSG00000255027	ENSG00000109832
ENSG00000254560	ENSG00000109881
ENSG00000255425	ENSG00000110002
ENSG00000214788	ENSG00000110108
ENSG00000255202	ENSG00000110427
ENSG00000258026	ENSG00000111046
ENSG00000258131	ENSG00000111145
ENSG00000185847	ENSG00000111249
ENSG00000203585	ENSG00000111554
ENSG00000241825	ENSG00000111581
ENSG00000258168	ENSG00000111596
ENSG00000223786	ENSG00000111799
ENSG00000272243	ENSG00000111799
ENSG00000271860	ENSG00000112214
ENSG00000272168	ENSG00000112273
ENSG00000227192	ENSG00000112414
ENSG00000229017	ENSG00000112414
ENSG00000250974	ENSG00000112941
ENSG00000248994	ENSG00000113430
ENSG00000248245	ENSG00000113558
ENSG00000250994	ENSG00000113558
ENSG00000206120	ENSG00000114204
ENSG00000238755	ENSG00000114790
ENSG00000241220	ENSG00000114790
ENSG00000243069	ENSG00000114790
ENSG00000225258	ENSG00000115232
ENSG00000281131	ENSG00000115232
ENSG00000224643	ENSG00000115252
ENSG00000237750	ENSG00000115271
ENSG00000232034	ENSG00000115539
ENSG00000233633	ENSG00000115705
ENSG00000145063	ENSG00000115758
ENSG00000205054	ENSG00000116016
ENSG00000232498	ENSG00000116741
ENSG00000233478	ENSG00000117643
ENSG00000264859	ENSG00000118276
ENSG00000237548	ENSG00000119397
ENSG00000235495	ENSG00000119865
ENSG00000230773	ENSG00000119888

ENSG00000237517	ENSG00000015475		ENSG00000278390	ENSG00000120696
ENSG00000254363	ENSG00000015479		ENSG00000257322	ENSG00000120833
ENSG00000228903	ENSG00000015520		ENSG00000249395	ENSG00000121005
ENSG00000229980	ENSG00000015532		ENSG00000248278	ENSG00000121073
ENSG00000228252	ENSG00000017260		ENSG00000248714	ENSG00000121073
ENSG00000281344	ENSG00000017427		ENSG00000264672	ENSG00000121101
ENSG00000241313	ENSG00000018408		ENSG00000248278	ENSG00000121104
ENSG00000213963	ENSG00000018510		ENSG00000280241	ENSG00000121211
ENSG00000253608	ENSG00000019549		ENSG00000167355	ENSG00000121236
ENSG00000254333	ENSG00000019582		ENSG00000249867	ENSG00000121621
ENSG00000233334	ENSG00000019995		ENSG00000254560	ENSG00000121621
ENSG00000249456	ENSG00000019995		ENSG00000255202	ENSG00000121690
ENSG00000253161	ENSG00000020181		ENSG00000230131	ENSG00000121898
ENSG00000228172	ENSG00000020633		ENSG00000223911	ENSG00000121989
ENSG00000230438	ENSG00000021355		ENSG00000235944	ENSG00000122543
ENSG00000250903	ENSG00000021355		ENSG00000237400	ENSG00000122547
ENSG00000078319	ENSG00000021461		ENSG00000223838	ENSG00000122691
ENSG00000242294	ENSG00000021461		ENSG00000226522	ENSG00000122691
ENSG00000272752	ENSG00000021461		ENSG00000243004	ENSG00000122691
ENSG00000238184	ENSG00000021762		ENSG00000248636	ENSG00000122966
ENSG00000258959	ENSG00000022976		ENSG00000257452	ENSG00000123064
ENSG00000232934	ENSG00000023041		ENSG00000132832	ENSG00000124196
ENSG00000251661	ENSG00000023191		ENSG00000267440	ENSG00000125319
ENSG00000255237	ENSG00000023191		ENSG00000229876	ENSG00000125872
ENSG00000269915	ENSG00000023191		ENSG00000232677	ENSG00000126246
ENSG00000231955	ENSG00000023228		ENSG00000269086	ENSG00000126249
ENSG00000279220	ENSG00000023228		ENSG00000234477	ENSG00000126337
ENSG00000228801	ENSG00000023287		ENSG00000238098	ENSG00000127564
ENSG00000227695	ENSG00000023839		ENSG00000260107	ENSG00000127564
ENSG00000246982	ENSG00000023892		ENSG00000262668	ENSG00000127564
ENSG00000277147	ENSG00000023902		ENSG00000227308	ENSG00000127824
ENSG00000268061	ENSG00000024422		ENSG00000267606	ENSG00000127903
ENSG00000184319	ENSG00000025708		ENSG00000234626	ENSG00000128276
ENSG00000278869	ENSG00000025708		ENSG00000234715	ENSG00000128606
ENSG00000273188	ENSG00000025770		ENSG00000224189	ENSG00000128645
ENSG00000234961	ENSG00000026025		ENSG00000213981	ENSG00000128683
ENSG00000203999	ENSG00000026559		ENSG00000259240	ENSG00000128872
ENSG00000224397	ENSG00000026559		ENSG00000259712	ENSG00000128872
ENSG00000198358	ENSG00000026751		ENSG00000245719	ENSG00000129007
ENSG00000224259	ENSG00000026751		ENSG00000259410	ENSG00000129007
ENSG00000124549	ENSG00000026950		ENSG00000259673	ENSG00000129007
ENSG00000232774	ENSG00000027075		ENSG00000261801	ENSG00000129038
ENSG00000234263	ENSG00000027697		ENSG00000249993	ENSG00000129048
ENSG00000246596	ENSG00000027847		ENSG00000255308	ENSG00000129170
ENSG00000247679	ENSG00000027847		ENSG00000255511	ENSG00000129170
ENSG00000236947	ENSG00000027869		ENSG00000255308	ENSG00000129173
ENSG00000245311	ENSG00000029153		ENSG00000262943	ENSG00000129194
ENSG00000255968	ENSG00000029153		ENSG00000253944	ENSG00000129422

ENSG00000231028	ENSG00000029363
ENSG00000237596	ENSG00000029363
ENSG00000261879	ENSG00000029725
ENSG00000204188	ENSG00000030110
ENSG00000240254	ENSG00000031081
ENSG00000242622	ENSG00000031081
ENSG00000241720	ENSG00000031698
ENSG00000268191	ENSG00000031823
ENSG00000268203	ENSG00000031823
ENSG00000268758	ENSG00000031823
ENSG00000268758	ENSG00000032444
ENSG00000262686	ENSG00000033011
ENSG00000268635	ENSG00000033327
ENSG00000267002	ENSG00000033627
ENSG00000233098	ENSG00000034152
ENSG00000228252	ENSG00000034533
ENSG00000228643	ENSG00000035115
ENSG00000271816	ENSG00000035403
ENSG00000271848	ENSG00000035403
ENSG00000272140	ENSG00000035403
ENSG00000273175	ENSG00000035687
ENSG00000266970	ENSG00000035862
ENSG00000080947	ENSG00000037637
ENSG00000215908	ENSG00000037637
ENSG00000237276	ENSG00000037637
ENSG00000248265	ENSG00000037965
ENSG00000250742	ENSG00000037965
ENSG00000258086	ENSG00000037965
ENSG00000261386	ENSG00000038358
ENSG00000249835	ENSG00000038427
ENSG00000250320	ENSG00000038427
ENSG00000263033	ENSG00000038532
ENSG00000251307	ENSG00000039123
ENSG00000247572	ENSG00000039319
ENSG00000161643	ENSG00000039650
ENSG00000268006	ENSG00000039650
ENSG00000268047	ENSG00000039650
ENSG00000269352	ENSG00000039650
ENSG00000214353	ENSG00000040199
ENSG00000260185	ENSG00000040199
ENSG00000261513	ENSG00000040199
ENSG00000262903	ENSG00000040531
ENSG00000215067	ENSG00000040633
ENSG00000256806	ENSG00000040633
ENSG00000228486	ENSG00000040933
ENSG00000250519	ENSG00000042429
ENSG00000255929	ENSG00000042429
ENSG00000224186	ENSG00000043143

ENSG00000267174	ENSG00000130167
ENSG00000267174	ENSG00000130176
ENSG00000230666	ENSG00000130201
ENSG00000231806	ENSG00000130957
ENSG00000261175	ENSG00000131148
ENSG00000178412	ENSG00000131196
ENSG00000267287	ENSG00000131196
ENSG00000248245	ENSG00000131437
ENSG00000246889	ENSG00000131626
ENSG00000234477	ENSG00000131747
ENSG00000251218	ENSG00000131773
ENSG00000262670	ENSG00000132388
ENSG00000273819	ENSG00000132749
ENSG00000225721	ENSG00000132780
ENSG00000176659	ENSG00000132825
ENSG00000267337	ENSG00000132874
ENSG00000274317	ENSG00000133115
ENSG00000251381	ENSG00000133808
ENSG00000254486	ENSG00000133808
ENSG00000257275	ENSG00000133937
ENSG00000267761	ENSG00000134049
ENSG00000186162	ENSG00000134070
ENSG00000254485	ENSG00000134070
ENSG00000230489	ENSG00000134215
ENSG00000254456	ENSG00000134343
ENSG00000234248	ENSG00000134460
ENSG00000237943	ENSG00000134460
ENSG00000255660	ENSG00000134533
ENSG00000226476	ENSG00000134709
ENSG00000264859	ENSG00000134758
ENSG00000224356	ENSG00000134864
ENSG00000233009	ENSG00000134864
ENSG00000280169	ENSG00000134864
ENSG00000247400	ENSG00000134874
ENSG00000226337	ENSG00000135063
ENSG00000257452	ENSG00000135094
ENSG00000241388	ENSG00000135100
ENSG00000255946	ENSG00000135100
ENSG00000241388	ENSG00000135127
ENSG00000248636	ENSG00000135127
ENSG00000257452	ENSG00000135148
ENSG00000259867	ENSG00000135697
ENSG00000261218	ENSG00000135697
ENSG00000236719	ENSG00000135823
ENSG00000228918	ENSG00000135838
ENSG00000231851	ENSG00000135972
ENSG00000214198	ENSG00000136011
ENSG00000258343	ENSG00000136014

ENSG00000273345	ENSG00000043143
ENSG00000237686	ENSG00000044090
ENSG00000232656	ENSG00000047056
ENSG00000232633	ENSG00000047188
ENSG00000281371	ENSG00000047230
ENSG00000245067	ENSG00000047315
ENSG00000261329	ENSG00000047578
ENSG00000259977	ENSG00000047597
ENSG00000255474	ENSG00000047621
ENSG00000227398	ENSG00000047849
ENSG00000256091	ENSG00000048028
ENSG00000256947	ENSG00000048028
ENSG00000248596	ENSG00000048140
ENSG00000236105	ENSG00000048405
ENSG00000254459	ENSG00000048649
ENSG00000254829	ENSG00000048649
ENSG00000224043	ENSG00000048991
ENSG00000182109	ENSG00000049089
ENSG00000235358	ENSG00000049089
ENSG00000243970	ENSG00000049089
ENSG00000250286	ENSG00000049283
ENSG00000251239	ENSG00000049283
ENSG00000275897	ENSG00000049283
ENSG00000255252	ENSG00000049449
ENSG00000165178	ENSG00000049541
ENSG00000214544	ENSG00000049541
ENSG00000277072	ENSG00000049541
ENSG00000226971	ENSG00000049768
ENSG00000228960	ENSG00000050327
ENSG00000229153	ENSG00000050327
ENSG00000244479	ENSG00000050327
ENSG00000253882	ENSG00000050327
ENSG00000236986	ENSG00000050555
ENSG00000229956	ENSG00000050628
ENSG00000233020	ENSG00000050628
ENSG00000235079	ENSG00000050628
ENSG00000248367	ENSG00000050748
ENSG00000214331	ENSG00000050820
ENSG00000240338	ENSG00000050820
ENSG00000267940	ENSG00000051009
ENSG00000266904	ENSG00000051128
ENSG00000269019	ENSG00000051128
ENSG00000248932	ENSG00000051382
ENSG00000260630	ENSG00000051523
ENSG00000235423	ENSG00000051825
ENSG00000231970	ENSG00000052749
ENSG00000246250	ENSG00000052841
ENSG00000254409	ENSG00000052841

ENSG00000258136	ENSG00000136045
ENSG00000261206	ENSG00000136153
ENSG00000184608	ENSG00000136573
ENSG00000225564	ENSG00000136824
ENSG00000159712	ENSG00000136842
ENSG00000237548	ENSG00000136940
ENSG00000159712	ENSG00000136943
ENSG00000281641	ENSG00000136960
ENSG00000237937	ENSG00000137656
ENSG00000256717	ENSG00000137656
ENSG00000259200	ENSG00000137767
ENSG00000245750	ENSG00000137807
ENSG00000259473	ENSG00000137821
ENSG00000259240	ENSG00000137875
ENSG00000259712	ENSG00000137875
ENSG00000205054	ENSG00000138079
ENSG00000248019	ENSG00000138640
ENSG00000251577	ENSG00000138778
ENSG00000232021	ENSG00000138795
ENSG00000232021	ENSG00000138801
ENSG00000255794	ENSG00000139350
ENSG00000258131	ENSG00000139350
ENSG00000257325	ENSG00000139351
ENSG00000257325	ENSG00000139354
ENSG00000248636	ENSG00000139767
ENSG00000225083	ENSG00000139835
ENSG00000258751	ENSG00000139915
ENSG00000259240	ENSG00000140284
ENSG00000187812	ENSG00000140367
ENSG00000260357	ENSG00000140367
ENSG00000187812	ENSG00000140374
ENSG00000259370	ENSG00000140416
ENSG00000259459	ENSG00000140416
ENSG00000259672	ENSG00000140416
ENSG00000260123	ENSG00000140534
ENSG00000259560	ENSG00000140538
ENSG00000260123	ENSG00000140538
ENSG00000260123	ENSG00000140543
ENSG00000257060	ENSG00000140563
ENSG00000258631	ENSG00000140563
ENSG00000258754	ENSG00000140563
ENSG00000258785	ENSG00000140563
ENSG00000245768	ENSG00000140859
ENSG00000259995	ENSG00000140873
ENSG00000261707	ENSG00000140876
ENSG00000250685	ENSG00000140955
ENSG00000261177	ENSG00000140968
ENSG00000268505	ENSG00000140968

ENSG00000254907	ENSG00000052841	ENSG00000268754	ENSG00000140968
ENSG00000258920	ENSG00000053254	ENSG00000179136	ENSG00000141052
ENSG00000224635	ENSG00000053438	ENSG00000265489	ENSG00000141052
ENSG00000269145	ENSG00000053501	ENSG00000261848	ENSG00000141255
ENSG00000269439	ENSG00000053501	ENSG00000260372	ENSG00000141384
ENSG00000269481	ENSG00000053501	ENSG00000267193	ENSG00000141469
ENSG00000256271	ENSG00000053702	ENSG00000264272	ENSG00000141540
ENSG00000258092	ENSG00000053702	ENSG00000266357	ENSG00000141540
ENSG00000258325	ENSG00000053702	ENSG00000260569	ENSG00000141665
ENSG00000264745	ENSG00000053747	ENSG00000263655	ENSG00000141665
ENSG00000259969	ENSG00000053770	ENSG00000263818	ENSG00000141738
ENSG00000099869	ENSG00000053918	ENSG00000225721	ENSG00000142945
ENSG00000233930	ENSG00000053918	ENSG00000233271	ENSG00000143001
ENSG00000269821	ENSG00000053918	ENSG00000234810	ENSG00000143001
ENSG00000197182	ENSG00000054611	ENSG00000228918	ENSG00000143333
ENSG00000235091	ENSG00000054611	ENSG00000228063	ENSG00000143353
ENSG00000260708	ENSG00000054611	ENSG00000236230	ENSG00000143498
ENSG00000279954	ENSG00000054611	ENSG00000238042	ENSG00000143498
ENSG00000260401	ENSG00000054967	ENSG00000236230	ENSG00000143507
ENSG00000258407	ENSG00000054983	ENSG00000238042	ENSG00000143507
ENSG00000258867	ENSG00000054983	ENSG00000272851	ENSG00000144401
ENSG00000259096	ENSG00000054983	ENSG00000273118	ENSG00000144451
ENSG00000268869	ENSG00000055070	ENSG00000237571	ENSG00000144452
ENSG00000268592	ENSG00000055208	ENSG00000186162	ENSG00000144550
ENSG00000281021	ENSG00000055211	ENSG00000254485	ENSG00000144550
ENSG00000232973	ENSG00000055332	ENSG00000254485	ENSG00000144554
ENSG00000235848	ENSG00000055332	ENSG00000249993	ENSG00000144868
ENSG00000272180	ENSG00000055813	ENSG00000239265	ENSG00000144893
ENSG00000239462	ENSG00000057019	ENSG00000243273	ENSG00000144893
ENSG00000236539	ENSG00000057252	ENSG00000239265	ENSG00000144895
ENSG00000223969	ENSG00000058091	ENSG00000243273	ENSG00000144895
ENSG00000226526	ENSG00000058453	ENSG00000249815	ENSG00000145365
ENSG00000189149	ENSG00000058600	ENSG00000249464	ENSG00000145375
ENSG00000237296	ENSG00000058600	ENSG00000249396	ENSG00000145491
ENSG00000232993	ENSG00000058799	ENSG00000250155	ENSG00000145604
ENSG00000259953	ENSG00000059769	ENSG00000248901	ENSG00000145721
ENSG00000176654	ENSG00000059804	ENSG00000250551	ENSG00000145721
ENSG00000256661	ENSG00000059804	ENSG00000251513	ENSG00000145721
ENSG00000282772	ENSG00000059915	ENSG00000231185	ENSG00000145819
ENSG00000256888	ENSG00000060138	ENSG00000250167	ENSG00000145832
ENSG00000229873	ENSG00000060491	ENSG00000251405	ENSG00000145850
ENSG00000277496	ENSG00000060491	ENSG00000231533	ENSG00000146243
ENSG00000261342	ENSG00000060558	ENSG00000271860	ENSG00000146263
ENSG00000267436	ENSG00000060558	ENSG00000271860	ENSG00000146267
ENSG00000267551	ENSG00000060558	ENSG00000232053	ENSG00000146857
ENSG00000183242	ENSG00000060749	ENSG00000253972	ENSG00000147676
ENSG00000255921	ENSG00000060982	ENSG00000281641	ENSG00000147676
ENSG00000278743	ENSG00000060982	ENSG00000253288	ENSG00000147724

ENSG00000257261	ENSG00000061273
ENSG00000257735	ENSG00000061273
ENSG00000274737	ENSG00000061273
ENSG00000249996	ENSG00000061455
ENSG00000088340	ENSG00000061656
ENSG00000232907	ENSG00000061656
ENSG00000241170	ENSG00000062282
ENSG00000214783	ENSG00000062582
ENSG00000228903	ENSG00000062582
ENSG00000273432	ENSG00000062582
ENSG00000259349	ENSG00000062716
ENSG00000266002	ENSG00000062716
ENSG00000267248	ENSG00000062716
ENSG00000280852	ENSG00000062716
ENSG00000259349	ENSG00000062725
ENSG00000268006	ENSG00000062822
ENSG00000257337	ENSG00000063046
ENSG00000257475	ENSG00000063046
ENSG00000232871	ENSG00000063176
ENSG00000268093	ENSG00000063176
ENSG00000232871	ENSG00000063180
ENSG00000235191	ENSG00000063180
ENSG00000267265	ENSG00000063241
ENSG00000267577	ENSG00000063241
ENSG00000266907	ENSG00000063244
ENSG00000267549	ENSG00000063244
ENSG00000218416	ENSG00000063660
ENSG00000227479	ENSG00000063660
ENSG00000251229	ENSG00000063978
ENSG00000247903	ENSG00000064115
ENSG00000245498	ENSG00000064199
ENSG00000250073	ENSG00000064199
ENSG00000238184	ENSG00000064201
ENSG00000269821	ENSG00000064201
ENSG00000244558	ENSG00000064205
ENSG00000239462	ENSG00000064225
ENSG00000267419	ENSG00000064490
ENSG00000268030	ENSG00000064490
ENSG00000266904	ENSG00000064545
ENSG00000269191	ENSG00000064545
ENSG00000267419	ENSG00000064547
ENSG00000275540	ENSG00000064547
ENSG00000269549	ENSG00000064601
ENSG00000271984	ENSG00000064601
ENSG00000267419	ENSG00000064607
ENSG00000275540	ENSG00000064607
ENSG00000230561	ENSG00000064651
ENSG00000245937	ENSG00000064651

ENSG00000236924	ENSG00000147854
ENSG00000227463	ENSG00000148053
ENSG00000236643	ENSG00000148200
ENSG00000225778	ENSG00000148426
ENSG00000203876	ENSG00000148700
ENSG00000255411	ENSG00000148925
ENSG00000254560	ENSG00000148942
ENSG00000255171	ENSG00000148948
ENSG00000255388	ENSG00000148948
ENSG00000251364	ENSG00000149050
ENSG00000254951	ENSG00000149050
ENSG00000254951	ENSG00000149054
ENSG00000254561	ENSG00000149403
ENSG00000175728	ENSG00000149418
ENSG00000255027	ENSG00000149548
ENSG00000255027	ENSG00000149554
ENSG00000132832	ENSG00000149596
ENSG00000229005	ENSG00000149596
ENSG00000233067	ENSG00000149970
ENSG00000248551	ENSG00000150625
ENSG00000249945	ENSG00000150625
ENSG00000263655	ENSG00000150656
ENSG00000228791	ENSG00000151090
ENSG00000237838	ENSG00000151090
ENSG00000232682	ENSG00000151150
ENSG00000258474	ENSG00000151320
ENSG00000258342	ENSG00000151332
ENSG00000248491	ENSG00000151475
ENSG00000248356	ENSG00000151611
ENSG00000234828	ENSG00000151623
ENSG00000248210	ENSG00000151623
ENSG00000249752	ENSG00000151623
ENSG00000250354	ENSG00000151623
ENSG00000234248	ENSG00000151655
ENSG00000236008	ENSG00000151693
ENSG00000254233	ENSG00000151725
ENSG00000132967	ENSG00000151789
ENSG00000225542	ENSG00000151789
ENSG00000250910	ENSG00000151962
ENSG00000267586	ENSG00000152214
ENSG00000249825	ENSG00000152380
ENSG00000249825	ENSG00000152409
ENSG00000246316	ENSG00000152503
ENSG00000239265	ENSG00000152580
ENSG00000243273	ENSG00000152580
ENSG00000203386	ENSG00000152689
ENSG00000259995	ENSG00000152910
ENSG00000247708	ENSG00000152953

ENSG00000267317	ENSG00000064666
ENSG00000266933	ENSG00000064687
ENSG00000224699	ENSG00000064886
ENSG00000231346	ENSG00000064886
ENSG00000235407	ENSG00000064886
ENSG00000240194	ENSG00000064886
ENSG00000266933	ENSG00000064932
ENSG00000267551	ENSG00000064961
ENSG00000224063	ENSG00000064989
ENSG00000227227	ENSG00000064989
ENSG00000225339	ENSG00000064995
ENSG00000196114	ENSG00000064999
ENSG00000225339	ENSG00000064999
ENSG00000267122	ENSG00000065000
ENSG00000267551	ENSG00000065000
ENSG00000241720	ENSG00000065135
ENSG00000254942	ENSG00000065135
ENSG00000236137	ENSG00000065183
ENSG00000225492	ENSG00000065243
ENSG00000231999	ENSG00000065243
ENSG00000237505	ENSG00000065243
ENSG00000225791	ENSG00000065308
ENSG00000257303	ENSG00000065357
ENSG00000258056	ENSG00000065357
ENSG00000272758	ENSG00000065485
ENSG00000240882	ENSG00000065518
ENSG00000242622	ENSG00000065518
ENSG00000186715	ENSG00000065526
ENSG00000237276	ENSG00000065526
ENSG00000268869	ENSG00000065526
ENSG00000225057	ENSG00000065802
ENSG00000188185	ENSG00000065883
ENSG00000217702	ENSG00000065911
ENSG00000257800	ENSG00000065911
ENSG00000176654	ENSG00000065970
ENSG00000256661	ENSG00000065970
ENSG00000257588	ENSG00000066117
ENSG00000272368	ENSG00000066117
ENSG00000198358	ENSG00000066294
ENSG00000228192	ENSG00000066322
ENSG00000230615	ENSG00000066322
ENSG00000255197	ENSG00000066336
ENSG00000237461	ENSG00000066697
ENSG00000227051	ENSG00000066739
ENSG00000267040	ENSG00000066926
ENSG00000237399	ENSG00000067057
ENSG00000225963	ENSG00000067066
ENSG00000233117	ENSG00000067082

ENSG00000251309	ENSG00000153064
ENSG00000258168	ENSG00000153233
ENSG00000204380	ENSG00000153237
ENSG00000244342	ENSG00000153266
ENSG00000265750	ENSG00000154040
ENSG00000266573	ENSG00000154040
ENSG00000261175	ENSG00000154102
ENSG00000261175	ENSG00000154118
ENSG00000184608	ENSG00000154319
ENSG00000237481	ENSG00000154429
ENSG00000213981	ENSG00000154479
ENSG00000260372	ENSG00000154611
ENSG00000273388	ENSG00000154914
ENSG00000232021	ENSG00000155011
ENSG00000235944	ENSG00000155026
ENSG00000249484	ENSG00000155511
ENSG00000237400	ENSG00000155849
ENSG00000251405	ENSG00000155858
ENSG00000256199	ENSG00000156076
ENSG00000259343	ENSG00000156206
ENSG00000259692	ENSG00000156206
ENSG00000215533	ENSG00000156265
ENSG00000236532	ENSG00000156265
ENSG00000249307	ENSG00000156269
ENSG00000215533	ENSG00000156273
ENSG00000225768	ENSG00000156395
ENSG00000253773	ENSG00000156469
ENSG00000253872	ENSG00000156469
ENSG00000254303	ENSG00000156787
ENSG00000214803	ENSG00000156831
ENSG00000249816	ENSG00000156831
ENSG00000253111	ENSG00000156831
ENSG00000253868	ENSG00000156831
ENSG00000224445	ENSG00000156876
ENSG00000259447	ENSG00000156970
ENSG00000254485	ENSG00000156983
ENSG00000251613	ENSG00000157111
ENSG00000259293	ENSG00000157456
ENSG00000259293	ENSG00000157470
ENSG00000205622	ENSG00000157554
ENSG00000227482	ENSG00000157657
ENSG00000234352	ENSG00000157680
ENSG00000196204	ENSG00000157927
ENSG00000251364	ENSG00000158077
ENSG00000265656	ENSG00000158201
ENSG00000232034	ENSG00000158435
ENSG00000260177	ENSG00000158545
ENSG00000215162	ENSG00000158813

ENSG00000237399	ENSG00000067082
ENSG00000251307	ENSG00000067113
ENSG00000265460	ENSG00000067191
ENSG00000223745	ENSG00000067208
ENSG00000224093	ENSG00000067208
ENSG00000260037	ENSG00000067225
ENSG00000260339	ENSG00000067225
ENSG00000261423	ENSG00000067225
ENSG00000261460	ENSG00000067225
ENSG00000205771	ENSG00000067369
ENSG00000259595	ENSG00000067369
ENSG00000188825	ENSG00000067596
ENSG00000213373	ENSG00000067596
ENSG00000267002	ENSG00000067596
ENSG00000217801	ENSG00000067606
ENSG00000227775	ENSG00000067606
ENSG00000268575	ENSG00000067606
ENSG00000232725	ENSG00000067829
ENSG00000267072	ENSG00000067836
ENSG00000184844	ENSG00000067840
ENSG00000232725	ENSG00000067840
ENSG00000260822	ENSG00000067992
ENSG00000230454	ENSG00000068028
ENSG00000235016	ENSG00000068028
ENSG00000188825	ENSG00000068079
ENSG00000267002	ENSG00000068079
ENSG00000267095	ENSG00000068097
ENSG00000267248	ENSG00000068097
ENSG00000188825	ENSG00000068120
ENSG00000213373	ENSG00000068120
ENSG00000188825	ENSG00000068137
ENSG00000267002	ENSG00000068137
ENSG00000182397	ENSG00000068305
ENSG00000259475	ENSG00000068305
ENSG00000226971	ENSG00000068323
ENSG00000226971	ENSG00000068354
ENSG00000267064	ENSG00000068354
ENSG00000214279	ENSG00000068383
ENSG00000229081	ENSG00000068383
ENSG00000234311	ENSG00000068383
ENSG00000226971	ENSG00000068400
ENSG00000232828	ENSG00000068400
ENSG00000226971	ENSG00000068438
ENSG00000274922	ENSG00000068650
ENSG00000232800	ENSG00000068697
ENSG00000236204	ENSG00000068697
ENSG00000228925	ENSG00000068724
ENSG00000239332	ENSG00000068724

ENSG00000234509	ENSG00000159055
ENSG00000282221	ENSG00000159166
ENSG00000282221	ENSG00000159173
ENSG00000239523	ENSG00000160145
ENSG00000225431	ENSG00000160191
ENSG00000253741	ENSG00000160886
ENSG00000234715	ENSG00000161040
ENSG00000263818	ENSG00000161381
ENSG00000268686	ENSG00000161609
ENSG00000262668	ENSG00000162076
ENSG00000234810	ENSG00000162396
ENSG00000230489	ENSG00000162639
ENSG00000261060	ENSG00000162782
ENSG00000145063	ENSG00000162976
ENSG00000224184	ENSG00000162976
ENSG00000224643	ENSG00000162999
ENSG00000224643	ENSG00000163002
ENSG00000224361	ENSG00000163026
ENSG00000235497	ENSG00000163026
ENSG00000235335	ENSG00000163092
ENSG00000247708	ENSG00000163132
ENSG00000231731	ENSG00000163166
ENSG00000225548	ENSG00000163508
ENSG00000241359	ENSG00000163618
ENSG00000244342	ENSG00000163618
ENSG00000248749	ENSG00000163624
ENSG00000250546	ENSG00000163624
ENSG00000241359	ENSG00000163632
ENSG00000244342	ENSG00000163632
ENSG00000241359	ENSG00000163634
ENSG00000244342	ENSG00000163634
ENSG00000241358	ENSG00000163746
ENSG00000240521	ENSG00000163754
ENSG00000248491	ENSG00000164070
ENSG00000232021	ENSG00000164089
ENSG00000249041	ENSG00000164114
ENSG00000250910	ENSG00000164114
ENSG00000250910	ENSG00000164116
ENSG00000251687	ENSG00000164162
ENSG00000249396	ENSG00000164236
ENSG00000249738	ENSG00000164330
ENSG00000248245	ENSG00000164404
ENSG00000223786	ENSG00000164430
ENSG00000197085	ENSG00000164532
ENSG00000243797	ENSG00000164597
ENSG00000250167	ENSG00000164615
ENSG00000250994	ENSG00000164615
ENSG00000232053	ENSG00000164707

ENSG00000235236	ENSG00000068745	ENSG00000249917	ENSG00000164756
ENSG00000245532	ENSG00000068831	ENSG00000225329	ENSG00000164815
ENSG00000256116	ENSG00000068831	ENSG00000246263	ENSG00000164929
ENSG00000269038	ENSG00000068831	ENSG00000253773	ENSG00000164941
ENSG00000229719	ENSG00000068971	ENSG00000232000	ENSG00000164989
ENSG00000255478	ENSG00000068971	ENSG00000231107	ENSG00000165023
ENSG00000266903	ENSG00000069399	ENSG00000248801	ENSG00000165084
ENSG00000267348	ENSG00000069399	ENSG00000227463	ENSG00000165113
ENSG00000111788	ENSG00000069493	ENSG00000227531	ENSG00000165124
ENSG00000214776	ENSG00000069493	ENSG00000226484	ENSG00000165164
ENSG00000245648	ENSG00000069493	ENSG00000276476	ENSG00000165480
ENSG00000256069	ENSG00000069493	ENSG00000245832	ENSG00000165490
ENSG00000236276	ENSG00000069535	ENSG00000258776	ENSG00000165588
ENSG00000183458	ENSG00000069764	ENSG00000234248	ENSG00000165632
ENSG00000260735	ENSG00000069764	ENSG00000257275	ENSG00000165959
ENSG00000249417	ENSG00000069849	ENSG00000251364	ENSG00000166387
ENSG00000259709	ENSG00000069966	ENSG00000254951	ENSG00000166387
ENSG00000100181	ENSG00000069998	ENSG00000251364	ENSG00000166407
ENSG00000280007	ENSG00000069998	ENSG00000254951	ENSG00000166407
ENSG00000236540	ENSG00000070010	ENSG00000261303	ENSG00000166426
ENSG00000237517	ENSG00000070010	ENSG00000245059	ENSG00000166455
ENSG00000255158	ENSG00000070047	ENSG00000259867	ENSG00000166455
ENSG00000255328	ENSG00000070047	ENSG00000260643	ENSG00000166455
ENSG00000240137	ENSG00000070087	ENSG00000260706	ENSG00000166455
ENSG00000240541	ENSG00000070087	ENSG00000260896	ENSG00000166455
ENSG00000248092	ENSG00000070193	ENSG00000259240	ENSG00000166477
ENSG00000258428	ENSG00000070269	ENSG00000002079	ENSG00000166526
ENSG00000259868	ENSG00000070269	ENSG00000256594	ENSG00000166535
ENSG00000266933	ENSG00000070404	ENSG00000261742	ENSG00000166546
ENSG00000237517	ENSG00000070413	ENSG00000257452	ENSG00000166578
ENSG00000262791	ENSG00000070444	ENSG00000255308	ENSG00000166800
ENSG00000263345	ENSG00000070444	ENSG00000260123	ENSG00000166823
ENSG00000265100	ENSG00000070540	ENSG00000261723	ENSG00000166851
ENSG00000267023	ENSG00000070540	ENSG00000259200	ENSG00000166920
ENSG00000227388	ENSG00000070610	ENSG00000259342	ENSG00000166920
ENSG00000230551	ENSG00000070614	ENSG00000259354	ENSG00000166920
ENSG00000231764	ENSG00000070669	ENSG00000259200	ENSG00000167014
ENSG00000258867	ENSG00000070778	ENSG00000253535	ENSG00000167034
ENSG00000230615	ENSG00000070785	ENSG00000267193	ENSG00000167210
ENSG00000268949	ENSG00000070785	ENSG00000267761	ENSG00000167210
ENSG00000226925	ENSG00000071054	ENSG00000258232	ENSG00000167553
ENSG00000249767	ENSG00000071127	ENSG00000251637	ENSG00000167797
ENSG00000272980	ENSG00000071242	ENSG00000273819	ENSG00000167797
ENSG00000258301	ENSG00000071246	ENSG00000267506	ENSG00000167889
ENSG00000258569	ENSG00000071246	ENSG00000282199	ENSG00000167941
ENSG00000206573	ENSG00000071282	ENSG00000253397	ENSG00000168078
ENSG00000232725	ENSG00000071553	ENSG00000227835	ENSG00000168263
ENSG00000280195	ENSG00000071553	ENSG00000236404	ENSG00000168263

ENSG00000239718	ENSG00000071794	ENSG00000261584	ENSG00000168298
ENSG00000232725	ENSG00000071859	ENSG00000250685	ENSG00000168418
ENSG00000280195	ENSG00000071859	ENSG00000233766	ENSG00000168497
ENSG00000265533	ENSG00000071991	ENSG00000259867	ENSG00000168589
ENSG00000227685	ENSG00000072210	ENSG00000260643	ENSG00000168589
ENSG00000228983	ENSG00000072210	ENSG00000260706	ENSG00000168589
ENSG00000231625	ENSG00000072210	ENSG00000246228	ENSG00000168672
ENSG00000262769	ENSG00000072210	ENSG00000253438	ENSG00000168672
ENSG00000264885	ENSG00000072210	ENSG00000253929	ENSG00000168672
ENSG00000185485	ENSG00000072274	ENSG00000254286	ENSG00000168672
ENSG00000235253	ENSG00000072364	ENSG00000245384	ENSG00000168772
ENSG00000250790	ENSG00000072609	ENSG00000248242	ENSG00000168772
ENSG00000223442	ENSG00000072682	ENSG00000248373	ENSG00000168772
ENSG00000230612	ENSG00000072682	ENSG00000251577	ENSG00000168772
ENSG00000233006	ENSG00000072682	ENSG00000247708	ENSG00000168826
ENSG00000237714	ENSG00000072682	ENSG00000257986	ENSG00000168952
ENSG00000215067	ENSG00000072778	ENSG00000239523	ENSG00000169087
ENSG00000215067	ENSG00000072818	ENSG00000223742	ENSG00000169306
ENSG00000261879	ENSG00000072849	ENSG00000187812	ENSG00000169375
ENSG00000241560	ENSG00000072858	ENSG00000235944	ENSG00000169402
ENSG00000269439	ENSG00000072954	ENSG00000231386	ENSG00000169564
ENSG00000266903	ENSG00000073008	ENSG00000227359	ENSG00000169607
ENSG00000267282	ENSG00000073008	ENSG00000187812	ENSG00000169752
ENSG00000230487	ENSG00000073067	ENSG00000187812	ENSG00000169758
ENSG00000273253	ENSG00000073150	ENSG00000260357	ENSG00000169758
ENSG00000272836	ENSG00000073169	ENSG00000260139	ENSG00000169783
ENSG00000273137	ENSG00000073169	ENSG00000261303	ENSG00000169783
ENSG00000273253	ENSG00000073169	ENSG00000245384	ENSG00000169836
ENSG00000189136	ENSG00000073417	ENSG00000251577	ENSG00000169836
ENSG00000230373	ENSG00000073417	ENSG00000248131	ENSG00000169862
ENSG00000277089	ENSG00000073536	ENSG00000251003	ENSG00000169946
ENSG00000260774	ENSG00000073578	ENSG00000251687	ENSG00000170365
ENSG00000197099	ENSG00000073792	ENSG00000234828	ENSG00000170390
ENSG00000223358	ENSG00000073792	ENSG00000248210	ENSG00000170390
ENSG00000223358	ENSG00000073803	ENSG00000241956	ENSG00000170584
ENSG00000197099	ENSG00000073849	ENSG00000255946	ENSG00000170633
ENSG00000228804	ENSG00000073849	ENSG00000251364	ENSG00000170743
ENSG00000246523	ENSG00000073921	ENSG00000184608	ENSG00000171056
ENSG00000224789	ENSG00000074047	ENSG00000260093	ENSG00000171056
ENSG00000236859	ENSG00000074047	ENSG00000254777	ENSG00000171316
ENSG00000260316	ENSG00000074071	ENSG00000227308	ENSG00000171450
ENSG00000246174	ENSG00000074201	ENSG00000234477	ENSG00000171475
ENSG00000235191	ENSG00000074219	ENSG00000235974	ENSG00000171649
ENSG00000255471	ENSG00000074266	ENSG00000225868	ENSG00000171804
ENSG00000246334	ENSG00000074276	ENSG00000225868	ENSG00000171827
ENSG00000244300	ENSG00000074416	ENSG00000267605	ENSG00000171827
ENSG00000229754	ENSG00000074582	ENSG00000259370	ENSG00000171914
ENSG00000231035	ENSG00000074582	ENSG00000259616	ENSG00000171914

ENSG00000268603	ENSG00000074582		ENSG00000259675	ENSG00000171914
ENSG00000263219	ENSG00000074755		ENSG00000227463	ENSG00000172159
ENSG00000228526	ENSG00000074800		ENSG00000233358	ENSG00000172179
ENSG00000236269	ENSG00000074800		ENSG00000272168	ENSG00000172179
ENSG00000205790	ENSG00000074842		ENSG00000243797	ENSG00000172209
ENSG00000273654	ENSG00000074855		ENSG00000249203	ENSG00000172244
ENSG00000268869	ENSG00000074964		ENSG00000260177	ENSG00000172530
ENSG00000277232	ENSG00000075234		ENSG00000256663	ENSG00000172572
ENSG00000228113	ENSG00000075303		ENSG00000256879	ENSG00000172572
ENSG00000242193	ENSG00000075391		ENSG00000259380	ENSG00000172575
ENSG00000254154	ENSG00000075391		ENSG00000258232	ENSG00000172602
ENSG00000223959	ENSG00000075399		ENSG00000245748	ENSG00000173013
ENSG00000260617	ENSG00000075399		ENSG00000268894	ENSG00000173145
ENSG00000229951	ENSG00000075426		ENSG00000239523	ENSG00000173175
ENSG00000228486	ENSG00000075568		ENSG00000227531	ENSG00000173258
ENSG00000260552	ENSG00000075643		ENSG00000255308	ENSG00000173432
ENSG00000206417	ENSG00000075785		ENSG00000234477	ENSG00000173805
ENSG00000244300	ENSG00000075785		ENSG00000224652	ENSG00000174013
ENSG00000261159	ENSG00000075785		ENSG00000247193	ENSG00000174145
ENSG00000231407	ENSG00000075945		ENSG00000247213	ENSG00000174527
ENSG00000256947	ENSG00000076053		ENSG00000249532	ENSG00000174720
ENSG00000227398	ENSG00000076201		ENSG00000249815	ENSG00000174720
ENSG00000230067	ENSG00000076242		ENSG00000206120	ENSG00000174776
ENSG00000235257	ENSG00000076242		ENSG00000239227	ENSG00000174776
ENSG00000227543	ENSG00000076351		ENSG00000184617	ENSG00000175063
ENSG00000260777	ENSG00000076351		ENSG00000198491	ENSG00000175077
ENSG00000266642	ENSG00000076351		ENSG00000257219	ENSG00000175183
ENSG00000203709	ENSG00000076356		ENSG00000258077	ENSG00000175183
ENSG00000255650	ENSG00000076513		ENSG00000262983	ENSG00000175267
ENSG00000258210	ENSG00000076513		ENSG00000271989	ENSG00000175279
ENSG00000256139	ENSG00000076555		ENSG00000253773	ENSG00000175305
ENSG00000257221	ENSG00000076555		ENSG00000253872	ENSG00000175305
ENSG00000264031	ENSG00000076604		ENSG00000250551	ENSG00000175449
ENSG00000264486	ENSG00000076604		ENSG00000253392	ENSG00000175691
ENSG00000266527	ENSG00000076604		ENSG00000248538	ENSG00000175806
ENSG00000274425	ENSG00000076662		ENSG00000254235	ENSG00000175806
ENSG00000282772	ENSG00000076685		ENSG00000265688	ENSG00000175866
ENSG00000232160	ENSG00000076770		ENSG00000230552	ENSG00000175879
ENSG00000268297	ENSG00000076826		ENSG00000236231	ENSG00000175879
ENSG00000268204	ENSG00000076924		ENSG00000275874	ENSG00000175894
ENSG00000213904	ENSG00000076928		ENSG00000282199	ENSG00000175906
ENSG00000268027	ENSG00000076928		ENSG00000231607	ENSG00000176124
ENSG00000268204	ENSG00000076944		ENSG00000265334	ENSG00000176208
ENSG00000268758	ENSG00000076944		ENSG00000266258	ENSG00000176225
ENSG00000226688	ENSG00000077147		ENSG00000218052	ENSG00000176371
ENSG00000231871	ENSG00000077157		ENSG00000256278	ENSG00000176371
ENSG00000235121	ENSG00000077157		ENSG00000275120	ENSG00000176371
ENSG00000261329	ENSG00000077235		ENSG00000234840	ENSG00000176399

ENSG00000256612	ENSG00000077312
ENSG00000267107	ENSG00000077312
ENSG00000237118	ENSG00000077348
ENSG00000268027	ENSG00000077348
ENSG00000244219	ENSG00000077454
ENSG00000274272	ENSG00000077454
ENSG00000267436	ENSG00000077463
ENSG00000235371	ENSG00000077522
ENSG00000225986	ENSG00000077549
ENSG00000251432	ENSG00000077684
ENSG00000261668	ENSG00000077684
ENSG00000233382	ENSG00000077721
ENSG00000258545	ENSG00000077721
ENSG00000234281	ENSG00000078018
ENSG00000254988	ENSG00000078124
ENSG00000249348	ENSG00000078140
ENSG00000218226	ENSG00000078269
ENSG00000182873	ENSG00000078369
ENSG00000269896	ENSG00000078369
ENSG00000278530	ENSG00000078596
ENSG00000266993	ENSG00000078618
ENSG00000230753	ENSG00000078699
ENSG00000230753	ENSG00000078747
ENSG00000177757	ENSG00000078808
ENSG00000223764	ENSG00000078808
ENSG00000237491	ENSG00000078808
ENSG00000260179	ENSG00000078808
ENSG00000272512	ENSG00000078808
ENSG00000088340	ENSG00000078814
ENSG00000214783	ENSG00000078967
ENSG00000239556	ENSG00000078967
ENSG00000273432	ENSG00000078967
ENSG00000263006	ENSG00000079134
ENSG00000237298	ENSG00000079150
ENSG00000213963	ENSG00000079156
ENSG00000237298	ENSG00000079156
ENSG00000225166	ENSG00000079246
ENSG00000235042	ENSG00000079246
ENSG00000236886	ENSG00000079246
ENSG00000243150	ENSG00000079257
ENSG00000260940	ENSG00000079335
ENSG00000247774	ENSG00000079337
ENSG00000257261	ENSG00000079337
ENSG00000257735	ENSG00000079337
ENSG00000213904	ENSG00000079385
ENSG00000257735	ENSG00000079387
ENSG00000269514	ENSG00000079387
ENSG00000213904	ENSG00000079432

ENSG00000223813	ENSG00000176532
ENSG00000253500	ENSG00000176571
ENSG00000227538	ENSG00000177181
ENSG00000235749	ENSG00000177275
ENSG00000235526	ENSG00000177301
ENSG00000227107	ENSG00000177483
ENSG00000281641	ENSG00000177570
ENSG00000261848	ENSG00000177602
ENSG00000255028	ENSG00000178105
ENSG00000178412	ENSG00000178184
ENSG00000267251	ENSG00000178184
ENSG00000231039	ENSG00000178462
ENSG00000273118	ENSG00000178568
ENSG00000227463	ENSG00000178966
ENSG00000265975	ENSG00000179148
ENSG00000178464	ENSG00000179284
ENSG00000267710	ENSG00000179709
ENSG00000231918	ENSG00000179915
ENSG00000184100	ENSG00000180044
ENSG00000261848	ENSG00000180090
ENSG00000262670	ENSG00000180090
ENSG00000257400	ENSG00000180263
ENSG00000258343	ENSG00000180263
ENSG00000167355	ENSG00000180785
ENSG00000227308	ENSG00000181378
ENSG00000234828	ENSG00000181541
ENSG00000226337	ENSG00000181778
ENSG00000249534	ENSG00000181826
ENSG00000245768	ENSG00000181938
ENSG00000245832	ENSG00000182103
ENSG00000255311	ENSG00000182103
ENSG00000257060	ENSG00000182175
ENSG00000258631	ENSG00000182175
ENSG00000258754	ENSG00000182175
ENSG00000258785	ENSG00000182175
ENSG00000260661	ENSG00000182175
ENSG00000235160	ENSG00000182256
ENSG00000254951	ENSG00000182334
ENSG00000227863	ENSG00000182348
ENSG00000237654	ENSG00000182667
ENSG00000235531	ENSG00000182674
ENSG00000258871	ENSG00000182732
ENSG00000227028	ENSG00000183023
ENSG00000258342	ENSG00000183032
ENSG00000236885	ENSG00000183091
ENSG00000257004	ENSG00000183150
ENSG00000248636	ENSG00000183273
ENSG00000254951	ENSG00000183378

ENSG00000267107	ENSG00000079432		ENSG00000235974	ENSG00000183647
ENSG00000213904	ENSG00000079435		ENSG00000246214	ENSG00000183654
ENSG00000231561	ENSG00000079435		ENSG00000248150	ENSG00000183654
ENSG00000268475	ENSG00000079435		ENSG00000214018	ENSG00000183690
ENSG00000168405	ENSG00000079689		ENSG00000223838	ENSG00000183742
ENSG00000272053	ENSG00000079689		ENSG00000243004	ENSG00000183742
ENSG00000233718	ENSG00000079785		ENSG00000251364	ENSG00000183801
ENSG00000266978	ENSG00000079805		ENSG00000254951	ENSG00000183801
ENSG00000184319	ENSG00000079974		ENSG00000264956	ENSG00000184185
ENSG00000267082	ENSG00000079999		ENSG00000266466	ENSG00000184185
ENSG00000274425	ENSG00000079999		ENSG00000277215	ENSG00000184258
ENSG00000267265	ENSG00000080031		ENSG00000248222	ENSG00000184347
ENSG00000267649	ENSG00000080031		ENSG00000249601	ENSG00000184347
ENSG00000225849	ENSG00000080189		ENSG00000254042	ENSG00000184347
ENSG00000273828	ENSG00000080189		ENSG00000251027	ENSG00000184349
ENSG00000231013	ENSG00000080293		ENSG00000271860	ENSG00000184486
ENSG00000232104	ENSG00000080298		ENSG00000237844	ENSG00000184611
ENSG00000238045	ENSG00000080603		ENSG00000253535	ENSG00000184661
ENSG00000239791	ENSG00000080603		ENSG00000254202	ENSG00000184672
ENSG00000261346	ENSG00000080603		ENSG00000229855	ENSG00000184838
ENSG00000278133	ENSG00000080603		ENSG00000260643	ENSG00000184860
ENSG00000239462	ENSG00000080819		ENSG00000260862	ENSG00000184860
ENSG00000088340	ENSG00000080845		ENSG00000225329	ENSG00000185055
ENSG00000232907	ENSG00000080845		ENSG00000243797	ENSG00000185055
ENSG00000251226	ENSG00000080854		ENSG00000267052	ENSG00000185055
ENSG00000215866	ENSG00000081019		ENSG00000251209	ENSG00000185087
ENSG00000226167	ENSG00000081019		ENSG00000247400	ENSG00000185352
ENSG00000226419	ENSG00000081019		ENSG00000257060	ENSG00000185442
ENSG00000238198	ENSG00000081019		ENSG00000258754	ENSG00000185442
ENSG00000249942	ENSG00000081041		ENSG00000260661	ENSG00000185442
ENSG00000236432	ENSG00000081052		ENSG00000002079	ENSG00000185467
ENSG00000245864	ENSG00000081189		ENSG00000261127	ENSG00000185947
ENSG00000248309	ENSG00000081189		ENSG00000261727	ENSG00000185947
ENSG00000240890	ENSG00000081307		ENSG00000261540	ENSG00000186153
ENSG00000248724	ENSG00000081307		ENSG00000271860	ENSG00000186231
ENSG00000229056	ENSG00000081320		ENSG00000250407	ENSG00000186314
ENSG00000266904	ENSG00000081665		ENSG00000251031	ENSG00000186314
ENSG00000267419	ENSG00000081665		ENSG00000251165	ENSG00000186352
ENSG00000267565	ENSG00000081665		ENSG00000254233	ENSG00000186352
ENSG00000269019	ENSG00000081665		ENSG00000232642	ENSG00000186453
ENSG00000272154	ENSG00000081791		ENSG00000258535	ENSG00000186469
ENSG00000278936	ENSG00000081853		ENSG00000257452	ENSG00000186710
ENSG00000280029	ENSG00000081853		ENSG00000198155	ENSG00000186777
ENSG00000244968	ENSG00000082074		ENSG00000173811	ENSG00000187094
ENSG00000249740	ENSG00000082074		ENSG00000257219	ENSG00000187109
ENSG00000223825	ENSG00000082146		ENSG00000258077	ENSG00000187109
ENSG00000215158	ENSG00000082196		ENSG00000197085	ENSG00000187258
ENSG00000227115	ENSG00000082212		ENSG00000254485	ENSG00000187288

ENSG00000224043	ENSG00000082258
ENSG00000153363	ENSG00000082512
ENSG00000263412	ENSG00000082641
ENSG00000263766	ENSG00000082641
ENSG00000264701	ENSG00000082641
ENSG00000242622	ENSG00000082701
ENSG00000241278	ENSG00000082781
ENSG00000241288	ENSG00000082781
ENSG00000165121	ENSG00000083223
ENSG00000234424	ENSG00000083223
ENSG00000236581	ENSG00000083642
ENSG00000248668	ENSG00000083720
ENSG00000213753	ENSG00000083807
ENSG00000232098	ENSG00000083807
ENSG00000267858	ENSG00000083807
ENSG00000268230	ENSG00000083807
ENSG00000269054	ENSG00000083807
ENSG00000269106	ENSG00000083807
ENSG00000269473	ENSG00000083807
ENSG00000268516	ENSG00000083814
ENSG00000213753	ENSG00000083838
ENSG00000267858	ENSG00000083838
ENSG00000269054	ENSG00000083838
ENSG00000267871	ENSG00000083844
ENSG00000269867	ENSG00000083844
ENSG00000248049	ENSG00000083896
ENSG00000281207	ENSG00000084070
ENSG00000231296	ENSG00000084073
ENSG00000238287	ENSG00000084073
ENSG00000245067	ENSG00000084093
ENSG00000248590	ENSG00000084093
ENSG00000255650	ENSG00000084112
ENSG00000247498	ENSG00000084444
ENSG00000255621	ENSG00000084444
ENSG00000220785	ENSG00000084623
ENSG00000235790	ENSG00000084623
ENSG00000220785	ENSG00000084628
ENSG00000235790	ENSG00000084652
ENSG00000232800	ENSG00000084674
ENSG00000227938	ENSG00000084734
ENSG00000248503	ENSG00000085224
ENSG00000225361	ENSG00000085265
ENSG00000260879	ENSG00000085491
ENSG00000078319	ENSG00000085514
ENSG00000242294	ENSG00000085514
ENSG00000242798	ENSG00000085514
ENSG00000272752	ENSG00000085514
ENSG00000182165	ENSG00000085563

ENSG00000225329	ENSG00000187416
ENSG00000234715	ENSG00000187416
ENSG00000229688	ENSG00000187546
ENSG00000226530	ENSG00000187690
ENSG00000229151	ENSG00000187690
ENSG00000260586	ENSG00000187720
ENSG00000258700	ENSG00000187790
ENSG00000258969	ENSG00000187790
ENSG00000258998	ENSG00000187790
ENSG00000230131	ENSG00000187908
ENSG00000257599	ENSG00000187950
ENSG00000267123	ENSG00000187997
ENSG00000267466	ENSG00000187997
ENSG00000225258	ENSG00000188452
ENSG00000281131	ENSG00000188452
ENSG00000254695	ENSG00000188487
ENSG00000254789	ENSG00000188487
ENSG00000254946	ENSG00000188487
ENSG00000258131	ENSG00000188596
ENSG00000258343	ENSG00000188596
ENSG00000226747	ENSG00000188738
ENSG00000259410	ENSG00000188779
ENSG00000259673	ENSG00000188779
ENSG00000259343	ENSG00000188869
ENSG00000259445	ENSG00000188869
ENSG00000259692	ENSG00000188869
ENSG00000247213	ENSG00000189046
ENSG00000234715	ENSG00000189056
ENSG00000214788	ENSG00000189057
ENSG00000259410	ENSG00000189227
ENSG00000259673	ENSG00000189227
ENSG00000230649	ENSG00000189320
ENSG00000232053	ENSG00000189320
ENSG00000226191	ENSG00000196071
ENSG00000159712	ENSG00000196116
ENSG00000266357	ENSG00000196169
ENSG00000225868	ENSG00000196218
ENSG00000226530	ENSG00000196368
ENSG00000267174	ENSG00000196646
ENSG00000258279	ENSG00000196876
ENSG00000262983	ENSG00000197006
ENSG00000246662	ENSG00000197275
ENSG00000253585	ENSG00000197275
ENSG00000253773	ENSG00000197275
ENSG00000214432	ENSG00000197299
ENSG00000214433	ENSG00000197299
ENSG00000259248	ENSG00000197361
ENSG00000259370	ENSG00000197361

ENSG00000228113	ENSG00000085563
ENSG00000205890	ENSG00000085644
ENSG00000263072	ENSG00000085644
ENSG00000254231	ENSG00000085719
ENSG00000254604	ENSG00000085733
ENSG00000232022	ENSG00000085998
ENSG00000232022	ENSG00000086015
ENSG00000226562	ENSG00000086062
ENSG00000233554	ENSG00000086062
ENSG00000232303	ENSG00000086102
ENSG00000235453	ENSG00000086102
ENSG00000257378	ENSG00000086159
ENSG00000257588	ENSG00000086159
ENSG00000258057	ENSG00000086159
ENSG00000272368	ENSG00000086159
ENSG00000236829	ENSG00000086504
ENSG00000260316	ENSG00000086504
ENSG00000267107	ENSG00000086544
ENSG00000249035	ENSG00000086589
ENSG00000247373	ENSG00000086598
ENSG00000214544	ENSG00000086730
ENSG00000232593	ENSG00000086758
ENSG00000205959	ENSG00000087008
ENSG00000249767	ENSG00000087008
ENSG00000078319	ENSG00000087077
ENSG00000242294	ENSG00000087077
ENSG00000272752	ENSG00000087077
ENSG00000232871	ENSG00000087088
ENSG00000235191	ENSG00000087088
ENSG00000227543	ENSG00000087095
ENSG00000266527	ENSG00000087095
ENSG00000224505	ENSG00000087152
ENSG00000267121	ENSG00000087152
ENSG00000267394	ENSG00000087152
ENSG00000251229	ENSG00000087266
ENSG00000249673	ENSG00000087269
ENSG00000249673	ENSG00000087274
ENSG00000251229	ENSG00000087274
ENSG00000214900	ENSG00000087299
ENSG00000237356	ENSG00000087301
ENSG00000255038	ENSG00000087365
ENSG00000247903	ENSG00000087494
ENSG00000257042	ENSG00000087494
ENSG00000246174	ENSG00000087884
ENSG00000268758	ENSG00000087903
ENSG00000265702	ENSG00000087995
ENSG00000267265	ENSG00000088038
ENSG00000224789	ENSG00000088179

ENSG00000249041	ENSG00000197410
ENSG00000280241	ENSG00000197410
ENSG00000235749	ENSG00000197472
ENSG00000254369	ENSG00000197576
ENSG00000260643	ENSG00000197943
ENSG00000264693	ENSG00000197971
ENSG00000227531	ENSG00000198121
ENSG00000255027	ENSG00000198331
ENSG00000233009	ENSG00000198542
ENSG00000261008	ENSG00000198650
ENSG00000248714	ENSG00000198740
ENSG00000250751	ENSG00000198740
ENSG00000249856	ENSG00000198780
ENSG00000258342	ENSG00000198807
ENSG00000214432	ENSG00000198901
ENSG00000258725	ENSG00000198901
ENSG00000229989	ENSG00000203724
ENSG00000231842	ENSG00000203760
ENSG00000237742	ENSG00000203760
ENSG00000223786	ENSG00000203907
ENSG00000231332	ENSG00000203907
ENSG00000223786	ENSG00000203908
ENSG00000267710	ENSG00000204524
ENSG00000234477	ENSG00000204815
ENSG00000260162	ENSG00000205022
ENSG00000261327	ENSG00000205022
ENSG00000214691	ENSG00000205086
ENSG00000227028	ENSG00000205111
ENSG00000231312	ENSG00000205111
ENSG00000251165	ENSG00000205129
ENSG00000254233	ENSG00000205129
ENSG00000261127	ENSG00000205456
ENSG00000258871	ENSG00000205683
ENSG00000234626	ENSG00000205853
ENSG00000267134	ENSG00000206075
ENSG00000225548	ENSG00000206559
ENSG00000246228	ENSG00000212993
ENSG00000253438	ENSG00000212993
ENSG00000254286	ENSG00000212993
ENSG00000235994	ENSG00000213066
ENSG00000230131	ENSG00000213185
ENSG00000269043	ENSG00000213988
ENSG00000228775	ENSG00000214102
ENSG00000267174	ENSG00000214212
ENSG00000277851	ENSG00000214215
ENSG00000240207	ENSG00000214216
ENSG00000279159	ENSG00000214491
ENSG00000227028	ENSG00000214694

ENSG00000225315	ENSG00000088280
ENSG00000088340	ENSG00000088298
ENSG00000272945	ENSG00000088298
ENSG00000232907	ENSG00000088367
ENSG00000235016	ENSG00000088543
ENSG00000265933	ENSG00000088756
ENSG00000235958	ENSG00000088812
ENSG00000235958	ENSG00000088827
ENSG00000234684	ENSG00000088832
ENSG00000235958	ENSG00000088899
ENSG00000249550	ENSG00000089060
ENSG00000249550	ENSG00000089127
ENSG00000255857	ENSG00000089154
ENSG00000255857	ENSG00000089159
ENSG00000230267	ENSG00000089280
ENSG00000239791	ENSG00000089280
ENSG00000261416	ENSG00000089280
ENSG00000267049	ENSG00000089327
ENSG00000271032	ENSG00000089351
ENSG00000254454	ENSG00000089597
ENSG00000231205	ENSG00000089639
ENSG00000269019	ENSG00000089639
ENSG00000240370	ENSG00000089692
ENSG00000205885	ENSG00000089693
ENSG00000258824	ENSG00000089775
ENSG00000259116	ENSG00000089775
ENSG00000164845	ENSG00000089818
ENSG00000205885	ENSG00000089818
ENSG00000256552	ENSG00000089818
ENSG00000232725	ENSG00000089820
ENSG00000130612	ENSG00000090006
ENSG00000267107	ENSG00000090006
ENSG00000237118	ENSG00000090013
ENSG00000234537	ENSG00000090054
ENSG00000235641	ENSG00000090054
ENSG00000258504	ENSG00000090061
ENSG00000258666	ENSG00000090061
ENSG00000258982	ENSG00000090061
ENSG00000239799	ENSG00000090097
ENSG00000196810	ENSG00000090316
ENSG00000269487	ENSG00000090372
ENSG00000241749	ENSG00000090376
ENSG00000250748	ENSG00000090376
ENSG00000256248	ENSG00000090376
ENSG00000262686	ENSG00000090447
ENSG00000197099	ENSG00000090520
ENSG00000233441	ENSG00000090539
ENSG00000271963	ENSG00000090612

ENSG00000231312	ENSG00000214694
ENSG00000232518	ENSG00000214694
ENSG00000214803	ENSG00000214814
ENSG00000253868	ENSG00000214814
ENSG00000236318	ENSG00000214960
ENSG00000260569	ENSG00000215421
ENSG00000263655	ENSG00000215421
ENSG00000188511	ENSG00000219438
ENSG00000224271	ENSG00000219438
ENSG00000253100	ENSG00000221818
ENSG00000224865	ENSG00000221866
ENSG00000259342	ENSG00000229474
ENSG00000259354	ENSG00000229474
ENSG00000267606	ENSG00000231274
ENSG00000261303	ENSG00000235711
ENSG00000002079	ENSG00000239521
ENSG00000227308	ENSG00000243910
ENSG00000214198	ENSG00000255150
ENSG00000239426	ENSG00000255298
ENSG00000265101	ENSG00000256463
ENSG00000267287	ENSG00000256463
ENSG00000258913	ENSG00000258986
ENSG00000260172	ENSG00000263155
ENSG00000267710	ENSG00000269699

ENSG00000268758	ENSG00000090659
ENSG00000269386	ENSG00000090674
ENSG00000269680	ENSG00000090674
ENSG00000196696	ENSG00000090861
ENSG00000214353	ENSG00000090861
ENSG00000261556	ENSG00000090861
ENSG00000205041	ENSG00000090924
ENSG00000256249	ENSG00000090975
ENSG00000243193	ENSG00000091138
ENSG00000225205	ENSG00000091409
ENSG00000238133	ENSG00000091409
ENSG00000225205	ENSG00000091428
ENSG00000226963	ENSG00000091428
ENSG00000238133	ENSG00000091428
ENSG00000225205	ENSG00000091436
ENSG00000238133	ENSG00000091436
ENSG00000225554	ENSG00000091483
ENSG00000242337	ENSG00000091513
ENSG00000248724	ENSG00000091513
ENSG00000242337	ENSG00000091527
ENSG00000248724	ENSG00000091527
ENSG00000154874	ENSG00000091536
ENSG00000197815	ENSG00000091536
ENSG00000263624	ENSG00000091536
ENSG00000266677	ENSG00000091536
ENSG00000261879	ENSG00000091592
ENSG00000215067	ENSG00000091622
ENSG00000256806	ENSG00000091622
ENSG00000157306	ENSG00000092010
ENSG00000215256	ENSG00000092010
ENSG00000260669	ENSG00000092010
ENSG00000215256	ENSG00000092051
ENSG00000258457	ENSG00000092051
ENSG00000258458	ENSG00000092051
ENSG00000258515	ENSG00000092094
ENSG00000215256	ENSG00000092096
ENSG00000258458	ENSG00000092096
ENSG00000215256	ENSG00000092098
ENSG00000157306	ENSG00000092330
ENSG00000215256	ENSG00000092330
ENSG00000248445	ENSG00000092421
ENSG00000244879	ENSG00000092439
ENSG00000259678	ENSG00000092439
ENSG00000259715	ENSG00000092439
ENSG00000251161	ENSG00000092445
ENSG00000174171	ENSG00000092529
ENSG00000258461	ENSG00000092529
ENSG00000227712	ENSG00000092621

ENSG00000231365	ENSG00000092621
ENSG00000218226	ENSG00000092820
ENSG00000271913	ENSG00000092820
ENSG00000237493	ENSG00000092841
ENSG00000258199	ENSG00000092841
ENSG00000116883	ENSG00000092847
ENSG00000163597	ENSG00000092929
ENSG00000267078	ENSG00000092929
ENSG00000267342	ENSG00000092929
ENSG00000267546	ENSG00000092929
ENSG00000163597	ENSG00000092931
ENSG00000234912	ENSG00000092931
ENSG00000161132	ENSG00000093010
ENSG00000227220	ENSG00000093134
ENSG00000227954	ENSG00000093134
ENSG00000227945	ENSG00000093144
ENSG00000230067	ENSG00000093167
ENSG00000229589	ENSG00000093217
ENSG00000235257	ENSG00000093217
ENSG00000249593	ENSG00000094880
ENSG00000248265	ENSG00000094916
ENSG00000258086	ENSG00000094916
ENSG00000237705	ENSG00000095015
ENSG00000267062	ENSG00000095059
ENSG00000267735	ENSG00000095059
ENSG00000268945	ENSG00000095059
ENSG00000267424	ENSG00000095066
ENSG00000255422	ENSG00000095139
ENSG00000228395	ENSG00000095319
ENSG00000204054	ENSG00000095321
ENSG00000228395	ENSG00000095370
ENSG00000257524	ENSG00000095370
ENSG00000226009	ENSG00000095539
ENSG00000226688	ENSG00000095587
ENSG00000260565	ENSG00000095906
ENSG00000237686	ENSG00000096080
ENSG00000220472	ENSG00000096696
ENSG00000268056	ENSG00000096996
ENSG00000273654	ENSG00000096996
ENSG00000267272	ENSG00000097033
ENSG00000223653	ENSG00000097096
ENSG00000240520	ENSG00000097096
ENSG00000282057	ENSG00000097096
ENSG00000266978	ENSG00000099203
ENSG00000267277	ENSG00000099203
ENSG00000229656	ENSG00000099250
ENSG00000259267	ENSG00000099284
ENSG00000235618	ENSG00000099290

ENSG00000269439	ENSG00000099308
ENSG00000213753	ENSG00000099326
ENSG00000232098	ENSG00000099326
ENSG00000267858	ENSG00000099326
ENSG00000269243	ENSG00000099331
ENSG00000183604	ENSG00000099364
ENSG00000235560	ENSG00000099364
ENSG00000260911	ENSG00000099364
ENSG00000183604	ENSG00000099365
ENSG00000183604	ENSG00000099377
ENSG00000235560	ENSG00000099377
ENSG00000260083	ENSG00000099377
ENSG00000261614	ENSG00000099377
ENSG00000262721	ENSG00000099377
ENSG00000183604	ENSG00000099381
ENSG00000239791	ENSG00000099381
ENSG00000183604	ENSG00000099385
ENSG00000250616	ENSG00000099385
ENSG00000260083	ENSG00000099385
ENSG00000261346	ENSG00000099385
ENSG00000261416	ENSG00000099385
ENSG00000262721	ENSG00000099385
ENSG00000266933	ENSG00000099625
ENSG00000269300	ENSG00000099783
ENSG00000171889	ENSG00000099810
ENSG00000247095	ENSG00000099834
ENSG00000251661	ENSG00000099834
ENSG00000255158	ENSG00000099834
ENSG00000255284	ENSG00000099834
ENSG00000269915	ENSG00000099834
ENSG00000230724	ENSG00000099849
ENSG00000247095	ENSG00000099849
ENSG00000255108	ENSG00000099849
ENSG00000255158	ENSG00000099849
ENSG00000267778	ENSG00000099864
ENSG00000236540	ENSG00000099901
ENSG00000274602	ENSG00000099901
ENSG00000243762	ENSG00000099904
ENSG00000243762	ENSG00000099910
ENSG00000183506	ENSG00000099937
ENSG00000229107	ENSG00000099937
ENSG00000237476	ENSG00000099937
ENSG00000230513	ENSG00000099949
ENSG00000237476	ENSG00000099949
ENSG00000178248	ENSG00000099953
ENSG00000178803	ENSG00000099953
ENSG00000215478	ENSG00000099953
ENSG00000272578	ENSG00000099953

ENSG00000273000	ENSG00000099953
ENSG00000273295	ENSG00000099953
ENSG00000133519	ENSG00000099956
ENSG00000230513	ENSG00000099957
ENSG00000183506	ENSG00000099960
ENSG00000226872	ENSG00000099960
ENSG00000229107	ENSG00000099960
ENSG00000230513	ENSG00000099960
ENSG00000128262	ENSG00000099991
ENSG00000230701	ENSG00000099991
ENSG00000273295	ENSG00000099991
ENSG00000218537	ENSG00000099994
ENSG00000225465	ENSG00000099999
ENSG00000228839	ENSG00000099999
ENSG00000128262	ENSG00000100014
ENSG00000178248	ENSG00000100014
ENSG00000178803	ENSG00000100014
ENSG00000215478	ENSG00000100014
ENSG00000272578	ENSG00000100014
ENSG00000273000	ENSG00000100014
ENSG00000280623	ENSG00000100014
ENSG00000230513	ENSG00000100030
ENSG00000236850	ENSG00000100030
ENSG00000272779	ENSG00000100030
ENSG00000237517	ENSG00000100033
ENSG00000224086	ENSG00000100034
ENSG00000237476	ENSG00000100034
ENSG00000224086	ENSG00000100038
ENSG00000237517	ENSG00000100056
ENSG00000235237	ENSG00000100060
ENSG00000279738	ENSG00000100060
ENSG00000233739	ENSG00000100065
ENSG00000244627	ENSG00000100065
ENSG00000279010	ENSG00000100065
ENSG00000279738	ENSG00000100065
ENSG00000100058	ENSG00000100068
ENSG00000230637	ENSG00000100068
ENSG00000272977	ENSG00000100068
ENSG00000128254	ENSG00000100078
ENSG00000244627	ENSG00000100079
ENSG00000279010	ENSG00000100083
ENSG00000233360	ENSG00000100092
ENSG00000128254	ENSG00000100100
ENSG00000231933	ENSG00000100104
ENSG00000244625	ENSG00000100104
ENSG00000214076	ENSG00000100105
ENSG00000228839	ENSG00000100105
ENSG00000244625	ENSG00000100109

ENSG00000261188	ENSG00000100109
ENSG00000183822	ENSG00000100124
ENSG00000233360	ENSG00000100124
ENSG00000244627	ENSG00000100129
ENSG00000279010	ENSG00000100129
ENSG00000279080	ENSG00000100129
ENSG00000233360	ENSG00000100139
ENSG00000235237	ENSG00000100139
ENSG00000279010	ENSG00000100139
ENSG00000279738	ENSG00000100139
ENSG00000184068	ENSG00000100147
ENSG00000230736	ENSG00000100150
ENSG00000233739	ENSG00000100151
ENSG00000235237	ENSG00000100151
ENSG00000249310	ENSG00000100151
ENSG00000279010	ENSG00000100151
ENSG00000225465	ENSG00000100154
ENSG00000235954	ENSG00000100154
ENSG00000230149	ENSG00000100196
ENSG00000244491	ENSG00000100196
ENSG00000244627	ENSG00000100196
ENSG00000227370	ENSG00000100197
ENSG00000232710	ENSG00000100197
ENSG00000237037	ENSG00000100197
ENSG00000270083	ENSG00000100197
ENSG00000281538	ENSG00000100197
ENSG00000225450	ENSG00000100201
ENSG00000230149	ENSG00000100201
ENSG00000244491	ENSG00000100201
ENSG00000244627	ENSG00000100201
ENSG00000225465	ENSG00000100209
ENSG00000226471	ENSG00000100209
ENSG00000235954	ENSG00000100209
ENSG00000230149	ENSG00000100216
ENSG00000244491	ENSG00000100216
ENSG00000244627	ENSG00000100216
ENSG00000225465	ENSG00000100219
ENSG00000226471	ENSG00000100219
ENSG00000235954	ENSG00000100219
ENSG00000230736	ENSG00000100220
ENSG00000230149	ENSG00000100221
ENSG00000230912	ENSG00000100221
ENSG00000244491	ENSG00000100221
ENSG00000244627	ENSG00000100221
ENSG00000244627	ENSG00000100226
ENSG00000279010	ENSG00000100226
ENSG00000279080	ENSG00000100226
ENSG00000184319	ENSG00000100239

ENSG00000273137	ENSG00000100239
ENSG00000279182	ENSG00000100239
ENSG00000184319	ENSG00000100241
ENSG00000213683	ENSG00000100241
ENSG00000233739	ENSG00000100242
ENSG00000244627	ENSG00000100242
ENSG00000249310	ENSG00000100242
ENSG00000225465	ENSG00000100263
ENSG00000227201	ENSG00000100263
ENSG00000226450	ENSG00000100266
ENSG00000274717	ENSG00000100266
ENSG00000225465	ENSG00000100280
ENSG00000234892	ENSG00000100294
ENSG00000172250	ENSG00000100300
ENSG00000274717	ENSG00000100304
ENSG00000225450	ENSG00000100307
ENSG00000244627	ENSG00000100307
ENSG00000244627	ENSG00000100311
ENSG00000225465	ENSG00000100314
ENSG00000272689	ENSG00000100319
ENSG00000249310	ENSG00000100335
ENSG00000272834	ENSG00000100335
ENSG00000183822	ENSG00000100342
ENSG00000235237	ENSG00000100342
ENSG00000235237	ENSG00000100345
ENSG00000183822	ENSG00000100353
ENSG00000235237	ENSG00000100353
ENSG00000229999	ENSG00000100359
ENSG00000197182	ENSG00000100364
ENSG00000183822	ENSG00000100365
ENSG00000233360	ENSG00000100365
ENSG00000235237	ENSG00000100365
ENSG00000183822	ENSG00000100368
ENSG00000235513	ENSG00000100380
ENSG00000229999	ENSG00000100393
ENSG00000235513	ENSG00000100395
ENSG00000235513	ENSG00000100399
ENSG00000237037	ENSG00000100401
ENSG00000184068	ENSG00000100403
ENSG00000237037	ENSG00000100403
ENSG00000184068	ENSG00000100412
ENSG00000237037	ENSG00000100412
ENSG00000197182	ENSG00000100416
ENSG00000277232	ENSG00000100416
ENSG00000280383	ENSG00000100416
ENSG00000184068	ENSG00000100418
ENSG00000237037	ENSG00000100418
ENSG00000270083	ENSG00000100418

ENSG00000197182	ENSG00000100422
ENSG00000184319	ENSG00000100429
ENSG00000272836	ENSG00000100429
ENSG00000273192	ENSG00000100429
ENSG00000157306	ENSG00000100439
ENSG00000251002	ENSG00000100439
ENSG00000258458	ENSG00000100439
ENSG00000275552	ENSG00000100439
ENSG00000215256	ENSG00000100445
ENSG00000157306	ENSG00000100461
ENSG00000258457	ENSG00000100461
ENSG00000157306	ENSG00000100462
ENSG00000251002	ENSG00000100462
ENSG00000258457	ENSG00000100462
ENSG00000258458	ENSG00000100462
ENSG00000214900	ENSG00000100483
ENSG00000214900	ENSG00000100485
ENSG00000214900	ENSG00000100490
ENSG00000214900	ENSG00000100503
ENSG00000214900	ENSG00000100504
ENSG00000237356	ENSG00000100519
ENSG00000237356	ENSG00000100522
ENSG00000237356	ENSG00000100526
ENSG00000237356	ENSG00000100532
ENSG00000258569	ENSG00000100593
ENSG00000246548	ENSG00000100596
ENSG00000258569	ENSG00000100596
ENSG00000232774	ENSG00000100644
ENSG00000259158	ENSG00000100650
ENSG00000227051	ENSG00000100697
ENSG00000235706	ENSG00000100697
ENSG00000234911	ENSG00000100714
ENSG00000258824	ENSG00000100714
ENSG00000258376	ENSG00000100767
ENSG00000259065	ENSG00000100767
ENSG00000259163	ENSG00000100796
ENSG00000157306	ENSG00000100802
ENSG00000251002	ENSG00000100802
ENSG00000258458	ENSG00000100802
ENSG00000215256	ENSG00000100804
ENSG00000214548	ENSG00000100811
ENSG00000258504	ENSG00000100811
ENSG00000258663	ENSG00000100811
ENSG00000258982	ENSG00000100811
ENSG00000259052	ENSG00000100811
ENSG00000258458	ENSG00000100813
ENSG00000251002	ENSG00000100836
ENSG00000258457	ENSG00000100836

ENSG00000258458	ENSG00000100836
ENSG00000157306	ENSG00000100842
ENSG00000215256	ENSG00000100842
ENSG00000251002	ENSG00000100842
ENSG00000258458	ENSG00000100842
ENSG00000258580	ENSG00000100852
ENSG00000157306	ENSG00000100867
ENSG00000215256	ENSG00000100867
ENSG00000157306	ENSG00000100889
ENSG00000215256	ENSG00000100889
ENSG00000260669	ENSG00000100889
ENSG00000258938	ENSG00000100902
ENSG00000157306	ENSG00000100908
ENSG00000215256	ENSG00000100908
ENSG00000259321	ENSG00000100908
ENSG00000157306	ENSG00000100911
ENSG00000215256	ENSG00000100911
ENSG00000260669	ENSG00000100911
ENSG00000215256	ENSG00000100918
ENSG00000157306	ENSG00000100926
ENSG00000278784	ENSG00000100926
ENSG00000215256	ENSG00000100938
ENSG00000157306	ENSG00000100949
ENSG00000215256	ENSG00000100968
ENSG00000258727	ENSG00000100968
ENSG00000088340	ENSG00000100983
ENSG00000204044	ENSG00000100985
ENSG00000088340	ENSG00000100991
ENSG00000213742	ENSG00000100994
ENSG00000088340	ENSG00000101019
ENSG00000244558	ENSG00000101098
ENSG00000244558	ENSG00000101109
ENSG00000203999	ENSG00000101126
ENSG00000233077	ENSG00000101126
ENSG00000232442	ENSG00000101150
ENSG00000268858	ENSG00000101150
ENSG00000124224	ENSG00000101160
ENSG00000124224	ENSG00000101162
ENSG00000229873	ENSG00000101189
ENSG00000229873	ENSG00000101194
ENSG00000232442	ENSG00000101216
ENSG00000234684	ENSG00000101255
ENSG00000225377	ENSG00000101266
ENSG00000234684	ENSG00000101266
ENSG00000225377	ENSG00000101276
ENSG00000149531	ENSG00000101294
ENSG00000234684	ENSG00000101298
ENSG00000088340	ENSG00000101335

ENSG00000232907	ENSG00000101335
ENSG00000198547	ENSG00000101346
ENSG00000235217	ENSG00000101346
ENSG00000232907	ENSG00000101347
ENSG00000198547	ENSG00000101391
ENSG00000230753	ENSG00000101400
ENSG00000275223	ENSG00000101400
ENSG00000272945	ENSG00000101421
ENSG00000224635	ENSG00000101442
ENSG00000269549	ENSG00000101473
ENSG00000274414	ENSG00000101474
ENSG00000263812	ENSG00000101493
ENSG00000265778	ENSG00000101493
ENSG00000266256	ENSG00000101493
ENSG00000261126	ENSG00000101546
ENSG00000267270	ENSG00000101546
ENSG00000266049	ENSG00000101574
ENSG00000264235	ENSG00000101596
ENSG00000266049	ENSG00000101596
ENSG00000266401	ENSG00000101596
ENSG00000266405	ENSG00000101596
ENSG00000264235	ENSG00000101605
ENSG00000265399	ENSG00000101605
ENSG00000266401	ENSG00000101605
ENSG00000263878	ENSG00000101608
ENSG00000264235	ENSG00000101608
ENSG00000265399	ENSG00000101608
ENSG00000272688	ENSG00000101608
ENSG00000270112	ENSG00000101638
ENSG00000264269	ENSG00000101665
ENSG00000265496	ENSG00000101665
ENSG00000205664	ENSG00000101825
ENSG00000236188	ENSG00000101825
ENSG00000233382	ENSG00000101856
ENSG00000258545	ENSG00000101856
ENSG00000226971	ENSG00000101940
ENSG00000214653	ENSG00000101974
ENSG00000232725	ENSG00000101986
ENSG00000226971	ENSG00000101997
ENSG00000226971	ENSG00000102001
ENSG00000271826	ENSG00000102024
ENSG00000232725	ENSG00000102032
ENSG00000232828	ENSG00000102103
ENSG00000232725	ENSG00000102119
ENSG00000197180	ENSG00000102125
ENSG00000232725	ENSG00000102125
ENSG00000280195	ENSG00000102125
ENSG00000197180	ENSG00000102178

ENSG00000232725	ENSG00000102178
ENSG00000235703	ENSG00000102181
ENSG00000245904	ENSG00000102189
ENSG00000257242	ENSG00000102189
ENSG00000235636	ENSG00000102225
ENSG00000237623	ENSG00000102265
ENSG00000226971	ENSG00000102312
ENSG00000235636	ENSG00000102312
ENSG00000204620	ENSG00000102317
ENSG00000226971	ENSG00000102317
ENSG00000227486	ENSG00000102349
ENSG00000168852	ENSG00000102743
ENSG00000215483	ENSG00000102743
ENSG00000233672	ENSG00000102786
ENSG00000236778	ENSG00000102786
ENSG00000281106	ENSG00000102786
ENSG00000231856	ENSG00000102796
ENSG00000233672	ENSG00000102796
ENSG00000243406	ENSG00000102796
ENSG00000281106	ENSG00000102796
ENSG00000224743	ENSG00000102802
ENSG00000170919	ENSG00000102804
ENSG00000227258	ENSG00000102804
ENSG00000183604	ENSG00000102870
ENSG00000261346	ENSG00000102870
ENSG00000246898	ENSG00000102871
ENSG00000270049	ENSG00000102871
ENSG00000246898	ENSG00000102878
ENSG00000270049	ENSG00000102878
ENSG00000183604	ENSG00000102879
ENSG00000198106	ENSG00000102879
ENSG00000205534	ENSG00000102879
ENSG00000198106	ENSG00000102882
ENSG00000205534	ENSG00000102882
ENSG00000232748	ENSG00000102882
ENSG00000260517	ENSG00000102882
ENSG00000277999	ENSG00000102882
ENSG00000198106	ENSG00000102886
ENSG00000205534	ENSG00000102886
ENSG00000260083	ENSG00000102886
ENSG00000260517	ENSG00000102886
ENSG00000262721	ENSG00000102886
ENSG00000277999	ENSG00000102886
ENSG00000270049	ENSG00000102890
ENSG00000180747	ENSG00000102897
ENSG00000189149	ENSG00000102897
ENSG00000248124	ENSG00000102897
ENSG00000260510	ENSG00000102897

ENSG00000270049	ENSG00000102901
ENSG00000263276	ENSG00000102904
ENSG00000196696	ENSG00000102908
ENSG00000261777	ENSG00000102908
ENSG00000260467	ENSG00000102931
ENSG00000260467	ENSG00000102934
ENSG00000259843	ENSG00000102935
ENSG00000260616	ENSG00000102935
ENSG00000263276	ENSG00000102974
ENSG00000260467	ENSG00000102978
ENSG00000260467	ENSG00000102996
ENSG00000276259	ENSG00000102996
ENSG00000196696	ENSG00000103018
ENSG00000196696	ENSG00000103043
ENSG00000260999	ENSG00000103047
ENSG00000196696	ENSG00000103051
ENSG00000263276	ENSG00000103061
ENSG00000270049	ENSG00000103066
ENSG00000261396	ENSG00000103067
ENSG00000270049	ENSG00000103067
ENSG00000261063	ENSG00000103111
ENSG00000259780	ENSG00000103197
ENSG00000259933	ENSG00000103197
ENSG00000261123	ENSG00000103197
ENSG00000236829	ENSG00000103202
ENSG00000250251	ENSG00000103222
ENSG00000260316	ENSG00000103227
ENSG00000260807	ENSG00000103227
ENSG00000276931	ENSG00000103227
ENSG00000261659	ENSG00000103245
ENSG00000236829	ENSG00000103254
ENSG00000260316	ENSG00000103254
ENSG00000261659	ENSG00000103254
ENSG00000261691	ENSG00000103254
ENSG00000182376	ENSG00000103257
ENSG00000260617	ENSG00000103257
ENSG00000261273	ENSG00000103257
ENSG00000261744	ENSG00000103257
ENSG00000269901	ENSG00000103264
ENSG00000270006	ENSG00000103264
ENSG00000236829	ENSG00000103266
ENSG00000261659	ENSG00000103266
ENSG00000182376	ENSG00000103335
ENSG00000224888	ENSG00000103335
ENSG00000260121	ENSG00000103335
ENSG00000260630	ENSG00000103335
ENSG00000261273	ENSG00000103335
ENSG00000263307	ENSG00000103342

ENSG00000260136	ENSG00000103356
ENSG00000259780	ENSG00000103363
ENSG00000262370	ENSG00000103363
ENSG00000267072	ENSG00000103423
ENSG00000103472	ENSG00000103485
ENSG00000198106	ENSG00000103485
ENSG00000260517	ENSG00000103485
ENSG00000260953	ENSG00000103485
ENSG00000230267	ENSG00000103490
ENSG00000261346	ENSG00000103490
ENSG00000103472	ENSG00000103495
ENSG00000198106	ENSG00000103495
ENSG00000238045	ENSG00000103495
ENSG00000250616	ENSG00000103495
ENSG00000260517	ENSG00000103495
ENSG00000260853	ENSG00000103495
ENSG00000260908	ENSG00000103495
ENSG00000261067	ENSG00000103495
ENSG00000261346	ENSG00000103495
ENSG00000260911	ENSG00000103496
ENSG00000261346	ENSG00000103496
ENSG00000103472	ENSG00000103502
ENSG00000198106	ENSG00000103502
ENSG00000238045	ENSG00000103502
ENSG00000260367	ENSG00000103502
ENSG00000260517	ENSG00000103502
ENSG00000260908	ENSG00000103502
ENSG00000261067	ENSG00000103502
ENSG00000261346	ENSG00000103502
ENSG00000261552	ENSG00000103502
ENSG00000260083	ENSG00000103507
ENSG00000260219	ENSG00000103507
ENSG00000260628	ENSG00000103507
ENSG00000261346	ENSG00000103507
ENSG00000262721	ENSG00000103507
ENSG00000278133	ENSG00000103510
ENSG00000250251	ENSG00000103512
ENSG00000260735	ENSG00000103512
ENSG00000262454	ENSG00000103512
ENSG00000277749	ENSG00000103653
ENSG00000259924	ENSG00000103710
ENSG00000259666	ENSG00000103740
ENSG00000260776	ENSG00000103740
ENSG00000259475	ENSG00000103852
ENSG00000260469	ENSG00000103855
ENSG00000259234	ENSG00000103876
ENSG00000259495	ENSG00000103888
ENSG00000174171	ENSG00000103994

ENSG00000206149	ENSG00000104059
ENSG00000259211	ENSG00000104081
ENSG00000259345	ENSG00000104081
ENSG00000259678	ENSG00000104093
ENSG00000259709	ENSG00000104093
ENSG00000259479	ENSG00000104131
ENSG00000259539	ENSG00000104131
ENSG00000259595	ENSG00000104131
ENSG00000179523	ENSG00000104133
ENSG00000205771	ENSG00000104133
ENSG00000259352	ENSG00000104133
ENSG00000259479	ENSG00000104133
ENSG00000259539	ENSG00000104133
ENSG00000247556	ENSG00000104142
ENSG00000248508	ENSG00000104142
ENSG00000259536	ENSG00000104142
ENSG00000259479	ENSG00000104164
ENSG00000259539	ENSG00000104164
ENSG00000259488	ENSG00000104177
ENSG00000249258	ENSG00000104219
ENSG00000253161	ENSG00000104221
ENSG00000253356	ENSG00000104221
ENSG00000253549	ENSG00000104267
ENSG00000251136	ENSG00000104325
ENSG00000253948	ENSG00000104356
ENSG00000253948	ENSG00000104375
ENSG00000259891	ENSG00000104472
ENSG00000254859	ENSG00000104518
ENSG00000247317	ENSG00000104522
ENSG00000247317	ENSG00000104524
ENSG00000253931	ENSG00000104524
ENSG00000255050	ENSG00000104524
ENSG00000177335	ENSG00000104529
ENSG00000247317	ENSG00000104529
ENSG00000254859	ENSG00000104529
ENSG00000254973	ENSG00000104529
ENSG00000246477	ENSG00000104643
ENSG00000248896	ENSG00000104643
ENSG00000251034	ENSG00000104679
ENSG00000253125	ENSG00000104679
ENSG00000253837	ENSG00000104679
ENSG00000246582	ENSG00000104689
ENSG00000250714	ENSG00000104689
ENSG00000253125	ENSG00000104689
ENSG00000260671	ENSG00000104731
ENSG00000235191	ENSG00000104804
ENSG00000268583	ENSG00000104804
ENSG00000267898	ENSG00000104812

ENSG00000269688	ENSG00000104824
ENSG00000269486	ENSG00000104825
ENSG00000266983	ENSG00000104833
ENSG00000266903	ENSG00000104853
ENSG00000267348	ENSG00000104853
ENSG00000186019	ENSG00000104856
ENSG00000266903	ENSG00000104856
ENSG00000267348	ENSG00000104856
ENSG00000269148	ENSG00000104856
ENSG00000186019	ENSG00000104859
ENSG00000266903	ENSG00000104859
ENSG00000267282	ENSG00000104859
ENSG00000267348	ENSG00000104859
ENSG00000266903	ENSG00000104866
ENSG00000267348	ENSG00000104866
ENSG00000267757	ENSG00000104866
ENSG00000232871	ENSG00000104872
ENSG00000243829	ENSG00000104872
ENSG00000269386	ENSG00000104880
ENSG00000214855	ENSG00000104881
ENSG00000266903	ENSG00000104881
ENSG00000267348	ENSG00000104881
ENSG00000269386	ENSG00000104883
ENSG00000267551	ENSG00000104885
ENSG00000232871	ENSG00000104894
ENSG00000268006	ENSG00000104894
ENSG00000280486	ENSG00000104897
ENSG00000267633	ENSG00000104907
ENSG00000267348	ENSG00000104936
ENSG00000267395	ENSG00000104936
ENSG00000268460	ENSG00000104936
ENSG00000131401	ENSG00000104946
ENSG00000268006	ENSG00000104946
ENSG00000180279	ENSG00000104951
ENSG00000235191	ENSG00000104951
ENSG00000267512	ENSG00000104957
ENSG00000235191	ENSG00000104960
ENSG00000268006	ENSG00000104960
ENSG00000267469	ENSG00000104964
ENSG00000275719	ENSG00000104967
ENSG00000267649	ENSG00000104974
ENSG00000269139	ENSG00000104980
ENSG00000161643	ENSG00000105053
ENSG00000268047	ENSG00000105053
ENSG00000267033	ENSG00000105058
ENSG00000267265	ENSG00000105063
ENSG00000267577	ENSG00000105063
ENSG00000266978	ENSG00000105088

ENSG00000267650	ENSG00000105088
ENSG00000268564	ENSG00000105135
ENSG00000248830	ENSG00000105220
ENSG00000256612	ENSG00000105221
ENSG00000237118	ENSG00000105223
ENSG00000130612	ENSG00000105227
ENSG00000256612	ENSG00000105227
ENSG00000130612	ENSG00000105245
ENSG00000256612	ENSG00000105245
ENSG00000205786	ENSG00000105270
ENSG00000267260	ENSG00000105270
ENSG00000267698	ENSG00000105270
ENSG00000268583	ENSG00000105321
ENSG00000268027	ENSG00000105323
ENSG00000270164	ENSG00000105323
ENSG00000267138	ENSG00000105325
ENSG00000267205	ENSG00000105325
ENSG00000267436	ENSG00000105325
ENSG00000256612	ENSG00000105329
ENSG00000268027	ENSG00000105329
ENSG00000239736	ENSG00000105341
ENSG00000267107	ENSG00000105341
ENSG00000232871	ENSG00000105373
ENSG00000267984	ENSG00000105379
ENSG00000269388	ENSG00000105379
ENSG00000182310	ENSG00000105383
ENSG00000269235	ENSG00000105383
ENSG00000269388	ENSG00000105383
ENSG00000269481	ENSG00000105393
ENSG00000266978	ENSG00000105397
ENSG00000232871	ENSG00000105398
ENSG00000267106	ENSG00000105401
ENSG00000267277	ENSG00000105401
ENSG00000266983	ENSG00000105426
ENSG00000269807	ENSG00000105426
ENSG00000267107	ENSG00000105429
ENSG00000270164	ENSG00000105429
ENSG00000268093	ENSG00000105438
ENSG00000232871	ENSG00000105443
ENSG00000268530	ENSG00000105443
ENSG00000232871	ENSG00000105447
ENSG00000268530	ENSG00000105447
ENSG00000180279	ENSG00000105472
ENSG00000182310	ENSG00000105472
ENSG00000269959	ENSG00000105472
ENSG00000268001	ENSG00000105483
ENSG00000268583	ENSG00000105483
ENSG00000269102	ENSG00000105497

ENSG00000235191	ENSG00000105516
ENSG00000268583	ENSG00000105516
ENSG00000266983	ENSG00000105519
ENSG00000267571	ENSG00000105519
ENSG00000232871	ENSG00000105538
ENSG00000235191	ENSG00000105559
ENSG00000213801	ENSG00000105568
ENSG00000268015	ENSG00000105568
ENSG00000269102	ENSG00000105568
ENSG00000226696	ENSG00000105609
ENSG00000227407	ENSG00000105609
ENSG00000228323	ENSG00000105609
ENSG00000267265	ENSG00000105609
ENSG00000267577	ENSG00000105609
ENSG00000267735	ENSG00000105612
ENSG00000268945	ENSG00000105612
ENSG00000237017	ENSG00000105619
ENSG00000269439	ENSG00000105642
ENSG00000268056	ENSG00000105643
ENSG00000269439	ENSG00000105643
ENSG00000269815	ENSG00000105643
ENSG00000273654	ENSG00000105643
ENSG00000269439	ENSG00000105650
ENSG00000269439	ENSG00000105655
ENSG00000268030	ENSG00000105656
ENSG00000269019	ENSG00000105662
ENSG00000273654	ENSG00000105662
ENSG00000266904	ENSG00000105671
ENSG00000267419	ENSG00000105671
ENSG00000275540	ENSG00000105671
ENSG00000266904	ENSG00000105676
ENSG00000267565	ENSG00000105676
ENSG00000275540	ENSG00000105676
ENSG00000269553	ENSG00000105679
ENSG00000205786	ENSG00000105699
ENSG00000262497	ENSG00000105699
ENSG00000268030	ENSG00000105700
ENSG00000269191	ENSG00000105701
ENSG00000267419	ENSG00000105705
ENSG00000275540	ENSG00000105705
ENSG00000271032	ENSG00000105707
ENSG00000205786	ENSG00000105711
ENSG00000271032	ENSG00000105711
ENSG00000271366	ENSG00000105711
ENSG00000213904	ENSG00000105722
ENSG00000267107	ENSG00000105722
ENSG00000213904	ENSG00000105723
ENSG00000231561	ENSG00000105723

ENSG00000267107	ENSG00000105723
ENSG00000268027	ENSG00000105723
ENSG00000231205	ENSG00000105726
ENSG00000213904	ENSG00000105737
ENSG00000231561	ENSG00000105737
ENSG00000239736	ENSG00000105737
ENSG00000266903	ENSG00000105771
ENSG00000205763	ENSG00000105778
ENSG00000229358	ENSG00000105778
ENSG00000170629	ENSG00000105808
ENSG00000170629	ENSG00000105819
ENSG00000236861	ENSG00000105829
ENSG00000243193	ENSG00000105835
ENSG00000223500	ENSG00000105875
ENSG00000231794	ENSG00000105875
ENSG00000231764	ENSG00000105880
ENSG00000226329	ENSG00000105889
ENSG00000214783	ENSG00000105953
ENSG00000273432	ENSG00000105953
ENSG00000214188	ENSG00000105967
ENSG00000226367	ENSG00000105967
ENSG00000243243	ENSG00000105967
ENSG00000214765	ENSG00000105968
ENSG00000272556	ENSG00000105968
ENSG00000214188	ENSG00000105971
ENSG00000226367	ENSG00000105971
ENSG00000243243	ENSG00000105971
ENSG00000226367	ENSG00000105974
ENSG00000243243	ENSG00000105974
ENSG00000243243	ENSG00000105976
ENSG00000224903	ENSG00000105982
ENSG00000233038	ENSG00000105983
ENSG00000230487	ENSG00000106003
ENSG00000230487	ENSG00000106009
ENSG00000236708	ENSG00000106009
ENSG00000228204	ENSG00000106070
ENSG00000225264	ENSG00000106080
ENSG00000227014	ENSG00000106080
ENSG00000196295	ENSG00000106086
ENSG00000225264	ENSG00000106086
ENSG00000227014	ENSG00000106086
ENSG00000232415	ENSG00000106089
ENSG00000232729	ENSG00000106089
ENSG00000229153	ENSG00000106123
ENSG00000231840	ENSG00000106123
ENSG00000253882	ENSG00000106123
ENSG00000281404	ENSG00000106125
ENSG00000229153	ENSG00000106144

ENSG00000231840	ENSG00000106144
ENSG00000244198	ENSG00000106144
ENSG00000253882	ENSG00000106144
ENSG00000078319	ENSG00000106244
ENSG00000242294	ENSG00000106244
ENSG00000244219	ENSG00000106244
ENSG00000272752	ENSG00000106244
ENSG00000078319	ENSG00000106258
ENSG00000224729	ENSG00000106258
ENSG00000242294	ENSG00000106258
ENSG00000272752	ENSG00000106258
ENSG00000242798	ENSG00000106261
ENSG00000272752	ENSG00000106261
ENSG00000274272	ENSG00000106261
ENSG00000236708	ENSG00000106266
ENSG00000273230	ENSG00000106266
ENSG00000224729	ENSG00000106333
ENSG00000242798	ENSG00000106333
ENSG00000244219	ENSG00000106333
ENSG00000274272	ENSG00000106333
ENSG00000280828	ENSG00000106344
ENSG00000187953	ENSG00000106346
ENSG00000240758	ENSG00000106348
ENSG00000242588	ENSG00000106348
ENSG00000280828	ENSG00000106348
ENSG00000281490	ENSG00000106348
ENSG00000240211	ENSG00000106351
ENSG00000244219	ENSG00000106351
ENSG00000274272	ENSG00000106351
ENSG00000078319	ENSG00000106397
ENSG00000242294	ENSG00000106397
ENSG00000272752	ENSG00000106397
ENSG00000230626	ENSG00000106459
ENSG00000242902	ENSG00000106459
ENSG00000273329	ENSG00000106459
ENSG00000204934	ENSG00000106479
ENSG00000235837	ENSG00000106524
ENSG00000237773	ENSG00000106524
ENSG00000235837	ENSG00000106546
ENSG00000224116	ENSG00000106571
ENSG00000214783	ENSG00000106605
ENSG00000228903	ENSG00000106605
ENSG00000273432	ENSG00000106605
ENSG00000214783	ENSG00000106608
ENSG00000272655	ENSG00000106608
ENSG00000273432	ENSG00000106608
ENSG00000214783	ENSG00000106624
ENSG00000228903	ENSG00000106624

ENSG00000273432	ENSG00000106624
ENSG00000214544	ENSG00000106635
ENSG00000274080	ENSG00000106635
ENSG00000214544	ENSG00000106638
ENSG00000232729	ENSG00000106665
ENSG00000205578	ENSG00000106682
ENSG00000214544	ENSG00000106682
ENSG00000214544	ENSG00000106683
ENSG00000232104	ENSG00000106688
ENSG00000226334	ENSG00000106692
ENSG00000237461	ENSG00000106799
ENSG00000187984	ENSG00000106823
ENSG00000259953	ENSG00000106868
ENSG00000204054	ENSG00000107021
ENSG00000204055	ENSG00000107021
ENSG00000257524	ENSG00000107021
ENSG00000228739	ENSG00000107077
ENSG00000204054	ENSG00000107130
ENSG00000227388	ENSG00000107140
ENSG00000230074	ENSG00000107140
ENSG00000225361	ENSG00000107147
ENSG00000235448	ENSG00000107165
ENSG00000227388	ENSG00000107185
ENSG00000235448	ENSG00000107186
ENSG00000226562	ENSG00000107262
ENSG00000228352	ENSG00000107262
ENSG00000236986	ENSG00000107263
ENSG00000204706	ENSG00000107282
ENSG00000275549	ENSG00000107317
ENSG00000180539	ENSG00000107331
ENSG00000260190	ENSG00000107331
ENSG00000225434	ENSG00000107372
ENSG00000177757	ENSG00000107404
ENSG00000217801	ENSG00000107404
ENSG00000224870	ENSG00000107404
ENSG00000268575	ENSG00000107404
ENSG00000239665	ENSG00000107537
ENSG00000224812	ENSG00000107551
ENSG00000237840	ENSG00000107551
ENSG00000227695	ENSG00000107554
ENSG00000177640	ENSG00000107560
ENSG00000258114	ENSG00000107560
ENSG00000224812	ENSG00000107562
ENSG00000237512	ENSG00000107731
ENSG00000237512	ENSG00000107736
ENSG00000259267	ENSG00000107736
ENSG00000259267	ENSG00000107738
ENSG00000236756	ENSG00000107745

ENSG00000236756	ENSG00000107758
ENSG00000237768	ENSG00000107758
ENSG00000271848	ENSG00000107758
ENSG00000196566	ENSG00000107789
ENSG00000223482	ENSG00000107789
ENSG00000225913	ENSG00000107789
ENSG00000227896	ENSG00000107789
ENSG00000180139	ENSG00000107796
ENSG00000232229	ENSG00000107796
ENSG00000232229	ENSG00000107798
ENSG00000226009	ENSG00000107874
ENSG00000233334	ENSG00000107902
ENSG00000273599	ENSG00000107902
ENSG00000232656	ENSG00000107929
ENSG00000232656	ENSG00000107937
ENSG00000260461	ENSG00000107960
ENSG00000224597	ENSG00000107968
ENSG00000259994	ENSG00000107968
ENSG00000228651	ENSG00000107984
ENSG00000273312	ENSG00000108100
ENSG00000224596	ENSG00000108175
ENSG00000225484	ENSG00000108175
ENSG00000244733	ENSG00000108175
ENSG00000224596	ENSG00000108179
ENSG00000225484	ENSG00000108179
ENSG00000230091	ENSG00000108179
ENSG00000235426	ENSG00000108179
ENSG00000242600	ENSG00000108179
ENSG00000244733	ENSG00000108179
ENSG00000225484	ENSG00000108219
ENSG00000264031	ENSG00000108256
ENSG00000264290	ENSG00000108256
ENSG00000264808	ENSG00000108256
ENSG00000265845	ENSG00000108256
ENSG00000227543	ENSG00000108262
ENSG00000264031	ENSG00000108262
ENSG00000264808	ENSG00000108262
ENSG00000188825	ENSG00000108312
ENSG00000224505	ENSG00000108312
ENSG00000267002	ENSG00000108312
ENSG00000267080	ENSG00000108312
ENSG00000267121	ENSG00000108312
ENSG00000267340	ENSG00000108312
ENSG00000267394	ENSG00000108312
ENSG00000215769	ENSG00000108370
ENSG00000265148	ENSG00000108375
ENSG00000265542	ENSG00000108375
ENSG00000213326	ENSG00000108379

ENSG00000262879	ENSG00000108379
ENSG00000265148	ENSG00000108387
ENSG00000265542	ENSG00000108387
ENSG00000265148	ENSG00000108389
ENSG00000265542	ENSG00000108389
ENSG00000240898	ENSG00000108405
ENSG00000266002	ENSG00000108406
ENSG00000267095	ENSG00000108406
ENSG00000228782	ENSG00000108424
ENSG00000262879	ENSG00000108424
ENSG00000263142	ENSG00000108424
ENSG00000263412	ENSG00000108424
ENSG00000264019	ENSG00000108424
ENSG00000264920	ENSG00000108424
ENSG00000262879	ENSG00000108433
ENSG00000263142	ENSG00000108433
ENSG00000228782	ENSG00000108439
ENSG00000234494	ENSG00000108439
ENSG00000263412	ENSG00000108439
ENSG00000264019	ENSG00000108439
ENSG00000266601	ENSG00000108439
ENSG00000259349	ENSG00000108443
ENSG00000231625	ENSG00000108448
ENSG00000240445	ENSG00000108448
ENSG00000273018	ENSG00000108448
ENSG00000264701	ENSG00000108465
ENSG00000266036	ENSG00000108469
ENSG00000267546	ENSG00000108479
ENSG00000266066	ENSG00000108506
ENSG00000235085	ENSG00000108509
ENSG00000261879	ENSG00000108515
ENSG00000261879	ENSG00000108518
ENSG00000263164	ENSG00000108518
ENSG00000263219	ENSG00000108518
ENSG00000261879	ENSG00000108523
ENSG00000235085	ENSG00000108528
ENSG00000188933	ENSG00000108551
ENSG00000154874	ENSG00000108557
ENSG00000273018	ENSG00000108557
ENSG00000263272	ENSG00000108561
ENSG00000214719	ENSG00000108578
ENSG00000248121	ENSG00000108578
ENSG00000263657	ENSG00000108578
ENSG00000264031	ENSG00000108578
ENSG00000264538	ENSG00000108578
ENSG00000214719	ENSG00000108582
ENSG00000264031	ENSG00000108582
ENSG00000264538	ENSG00000108582

ENSG00000214719	ENSG00000108587
ENSG00000264031	ENSG00000108587
ENSG00000264538	ENSG00000108587
ENSG00000266865	ENSG00000108587
ENSG00000233635	ENSG00000108588
ENSG00000215067	ENSG00000108590
ENSG00000256806	ENSG00000108590
ENSG00000227685	ENSG00000108599
ENSG00000228983	ENSG00000108599
ENSG00000233098	ENSG00000108599
ENSG00000262769	ENSG00000108599
ENSG00000214176	ENSG00000108604
ENSG00000233635	ENSG00000108604
ENSG00000215769	ENSG00000108654
ENSG00000266970	ENSG00000108669
ENSG00000266970	ENSG00000108679
ENSG00000267765	ENSG00000108773
ENSG00000260105	ENSG00000108774
ENSG00000266962	ENSG00000108786
ENSG00000267042	ENSG00000108786
ENSG00000267765	ENSG00000108786
ENSG00000236383	ENSG00000108797
ENSG00000267765	ENSG00000108797
ENSG00000267002	ENSG00000108799
ENSG00000229980	ENSG00000108819
ENSG00000236472	ENSG00000108819
ENSG00000249176	ENSG00000108819
ENSG00000204584	ENSG00000108821
ENSG00000229980	ENSG00000108826
ENSG00000250286	ENSG00000108826
ENSG00000188825	ENSG00000108828
ENSG00000197291	ENSG00000108828
ENSG00000213373	ENSG00000108828
ENSG00000236383	ENSG00000108828
ENSG00000229980	ENSG00000108829
ENSG00000188825	ENSG00000108830
ENSG00000267002	ENSG00000108830
ENSG00000215067	ENSG00000108839
ENSG00000264772	ENSG00000108839
ENSG00000267394	ENSG00000108840
ENSG00000251239	ENSG00000108846
ENSG00000236383	ENSG00000108861
ENSG00000267121	ENSG00000108883
ENSG00000267278	ENSG00000108883
ENSG00000231749	ENSG00000108932
ENSG00000237854	ENSG00000108932
ENSG00000231749	ENSG00000108946
ENSG00000215067	ENSG00000108947

ENSG00000231749	ENSG00000108950
ENSG00000267023	ENSG00000108950
ENSG00000267731	ENSG00000108950
ENSG00000227685	ENSG00000109016
ENSG00000233098	ENSG00000109016
ENSG00000239223	ENSG00000109046
ENSG00000264486	ENSG00000109046
ENSG00000265683	ENSG00000109046
ENSG00000266036	ENSG00000109065
ENSG00000265168	ENSG00000109079
ENSG00000227543	ENSG00000109103
ENSG00000264808	ENSG00000109103
ENSG00000265254	ENSG00000109103
ENSG00000266527	ENSG00000109103
ENSG00000227543	ENSG00000109107
ENSG00000264031	ENSG00000109107
ENSG00000264808	ENSG00000109107
ENSG00000265168	ENSG00000109107
ENSG00000264031	ENSG00000109111
ENSG00000264486	ENSG00000109111
ENSG00000266642	ENSG00000109111
ENSG00000227543	ENSG00000109113
ENSG00000264031	ENSG00000109113
ENSG00000264304	ENSG00000109113
ENSG00000267259	ENSG00000109113
ENSG00000260777	ENSG00000109118
ENSG00000264031	ENSG00000109118
ENSG00000265845	ENSG00000109118
ENSG00000226950	ENSG00000109184
ENSG00000248115	ENSG00000109189
ENSG00000249341	ENSG00000109220
ENSG00000248161	ENSG00000109320
ENSG00000248161	ENSG00000109323
ENSG00000251596	ENSG00000109472
ENSG00000245293	ENSG00000109475
ENSG00000247950	ENSG00000109534
ENSG00000249955	ENSG00000109572
ENSG00000237125	ENSG00000109586
ENSG00000245213	ENSG00000109586
ENSG00000248174	ENSG00000109586
ENSG00000249767	ENSG00000109667
ENSG00000261490	ENSG00000109667
ENSG00000249673	ENSG00000109758
ENSG00000231160	ENSG00000109787
ENSG00000249207	ENSG00000109790
ENSG00000205794	ENSG00000109814
ENSG00000231160	ENSG00000109814
ENSG00000249348	ENSG00000109814

ENSG00000255197	ENSG00000109919
ENSG00000255248	ENSG00000109929
ENSG00000245498	ENSG00000110013
ENSG00000229719	ENSG00000110025
ENSG00000245532	ENSG00000110046
ENSG00000255478	ENSG00000110046
ENSG00000251562	ENSG00000110047
ENSG00000160172	ENSG00000110057
ENSG00000255306	ENSG00000110057
ENSG00000255062	ENSG00000110074
ENSG00000254607	ENSG00000110080
ENSG00000257271	ENSG00000110080
ENSG00000280832	ENSG00000110080
ENSG00000254604	ENSG00000110092
ENSG00000243742	ENSG00000110104
ENSG00000256813	ENSG00000110104
ENSG00000243742	ENSG00000110107
ENSG00000256813	ENSG00000110107
ENSG00000255929	ENSG00000110218
ENSG00000256469	ENSG00000110218
ENSG00000254851	ENSG00000110274
ENSG00000246273	ENSG00000110315
ENSG00000246308	ENSG00000110315
ENSG00000247271	ENSG00000110315
ENSG00000255125	ENSG00000110315
ENSG00000254528	ENSG00000110324
ENSG00000255422	ENSG00000110324
ENSG00000278768	ENSG00000110324
ENSG00000255422	ENSG00000110367
ENSG00000278376	ENSG00000110367
ENSG00000255422	ENSG00000110375
ENSG00000255422	ENSG00000110400
ENSG00000254708	ENSG00000110429
ENSG00000124915	ENSG00000110446
ENSG00000243742	ENSG00000110446
ENSG00000254639	ENSG00000110497
ENSG00000255197	ENSG00000110514
ENSG00000238184	ENSG00000110651
ENSG00000269821	ENSG00000110651
ENSG00000226416	ENSG00000110665
ENSG00000238184	ENSG00000110665
ENSG00000184669	ENSG00000110696
ENSG00000160172	ENSG00000110717
ENSG00000255306	ENSG00000110717
ENSG00000160172	ENSG00000110721
ENSG00000255306	ENSG00000110721
ENSG00000215039	ENSG00000110799
ENSG00000256377	ENSG00000110841

ENSG00000257378	ENSG00000110844
ENSG00000257588	ENSG00000110844
ENSG00000258017	ENSG00000110844
ENSG00000258057	ENSG00000110844
ENSG00000272368	ENSG00000110844
ENSG00000111788	ENSG00000110852
ENSG00000214776	ENSG00000110852
ENSG00000245648	ENSG00000110852
ENSG00000256673	ENSG00000110852
ENSG00000256888	ENSG00000110852
ENSG00000257221	ENSG00000110876
ENSG00000256139	ENSG00000110880
ENSG00000258017	ENSG00000110881
ENSG00000258101	ENSG00000110881
ENSG00000272368	ENSG00000110881
ENSG00000226472	ENSG00000110900
ENSG00000255867	ENSG00000110900
ENSG00000272368	ENSG00000110911
ENSG00000255650	ENSG00000110921
ENSG00000272368	ENSG00000110925
ENSG00000224713	ENSG00000110955
ENSG00000257337	ENSG00000111057
ENSG00000257467	ENSG00000111058
ENSG00000257337	ENSG00000111077
ENSG00000224713	ENSG00000111087
ENSG00000177406	ENSG00000111181
ENSG00000226210	ENSG00000111181
ENSG00000249695	ENSG00000111181
ENSG00000256667	ENSG00000111196
ENSG00000250899	ENSG00000111203
ENSG00000256712	ENSG00000111215
ENSG00000255650	ENSG00000111229
ENSG00000258210	ENSG00000111229
ENSG00000258210	ENSG00000111237
ENSG00000234608	ENSG00000111271
ENSG00000258099	ENSG00000111271
ENSG00000240370	ENSG00000111319
ENSG00000256433	ENSG00000111321
ENSG00000235423	ENSG00000111325
ENSG00000256249	ENSG00000111325
ENSG00000235423	ENSG00000111328
ENSG00000249550	ENSG00000111331
ENSG00000249550	ENSG00000111335
ENSG00000257286	ENSG00000111335
ENSG00000249550	ENSG00000111344
ENSG00000257261	ENSG00000111371
ENSG00000257735	ENSG00000111424
ENSG00000248265	ENSG00000111481

ENSG00000250742	ENSG00000111481
ENSG00000258086	ENSG00000111481
ENSG00000179899	ENSG00000111540
ENSG00000237493	ENSG00000111540
ENSG00000215039	ENSG00000111639
ENSG00000256967	ENSG00000111640
ENSG00000256433	ENSG00000111641
ENSG00000256967	ENSG00000111641
ENSG00000215039	ENSG00000111644
ENSG00000205885	ENSG00000111652
ENSG00000256433	ENSG00000111652
ENSG00000215039	ENSG00000111653
ENSG00000256967	ENSG00000111667
ENSG00000269892	ENSG00000111667
ENSG00000240370	ENSG00000111669
ENSG00000281344	ENSG00000111670
ENSG00000240370	ENSG00000111671
ENSG00000248593	ENSG00000111671
ENSG00000240370	ENSG00000111674
ENSG00000275703	ENSG00000111674
ENSG00000240370	ENSG00000111676
ENSG00000248593	ENSG00000111676
ENSG00000275703	ENSG00000111676
ENSG00000215039	ENSG00000111684
ENSG00000240370	ENSG00000111684
ENSG00000257681	ENSG00000111696
ENSG00000277283	ENSG00000111737
ENSG00000226711	ENSG00000111752
ENSG00000237248	ENSG00000111752
ENSG00000256069	ENSG00000111752
ENSG00000245311	ENSG00000111790
ENSG00000246695	ENSG00000111790
ENSG00000255750	ENSG00000111790
ENSG00000255968	ENSG00000111790
ENSG00000256234	ENSG00000111790
ENSG00000272558	ENSG00000111801
ENSG00000247925	ENSG00000111859
ENSG00000236548	ENSG00000111912
ENSG00000227627	ENSG00000112031
ENSG00000232909	ENSG00000112062
ENSG00000246982	ENSG00000112062
ENSG00000230845	ENSG00000112144
ENSG00000231683	ENSG00000112144
ENSG00000231683	ENSG00000112146
ENSG00000226803	ENSG00000112200
ENSG00000226803	ENSG00000112208
ENSG00000231441	ENSG00000112210
ENSG00000224384	ENSG00000112218

ENSG00000228439	ENSG00000112234
ENSG00000227803	ENSG00000112242
ENSG00000227220	ENSG00000112282
ENSG00000227954	ENSG00000112299
ENSG00000234484	ENSG00000112303
ENSG00000232295	ENSG00000112305
ENSG00000233237	ENSG00000112305
ENSG00000168405	ENSG00000112308
ENSG00000232295	ENSG00000112309
ENSG00000227954	ENSG00000112319
ENSG00000237596	ENSG00000112339
ENSG00000168405	ENSG00000112343
ENSG00000272053	ENSG00000112343
ENSG00000237499	ENSG00000112378
ENSG00000235652	ENSG00000112425
ENSG00000204188	ENSG00000112473
ENSG00000229391	ENSG00000112473
ENSG00000204188	ENSG00000112511
ENSG00000225339	ENSG00000112511
ENSG00000204188	ENSG00000112514
ENSG00000225339	ENSG00000112514
ENSG00000261068	ENSG00000112578
ENSG00000261003	ENSG00000112584
ENSG00000245261	ENSG00000112624
ENSG00000231113	ENSG00000112640
ENSG00000237686	ENSG00000112655
ENSG00000261068	ENSG00000112655
ENSG00000237686	ENSG00000112658
ENSG00000237686	ENSG00000112659
ENSG00000237686	ENSG00000112667
ENSG00000245261	ENSG00000112667
ENSG00000272463	ENSG00000112685
ENSG00000225793	ENSG00000112697
ENSG00000225793	ENSG00000112701
ENSG00000237174	ENSG00000112701
ENSG00000237686	ENSG00000112715
ENSG00000228170	ENSG00000112739
ENSG00000224843	ENSG00000112763
ENSG00000231889	ENSG00000112769
ENSG00000255992	ENSG00000112787
ENSG00000272154	ENSG00000112852
ENSG00000278936	ENSG00000112852
ENSG00000280029	ENSG00000112852
ENSG00000249637	ENSG00000112855
ENSG00000254363	ENSG00000112855
ENSG00000272154	ENSG00000112855
ENSG00000280029	ENSG00000112855
ENSG00000249476	ENSG00000112893

ENSG00000177738	ENSG00000112964
ENSG00000215068	ENSG00000112964
ENSG00000177738	ENSG00000112972
ENSG00000215068	ENSG00000112972
ENSG00000249593	ENSG00000112981
ENSG00000177738	ENSG00000112992
ENSG00000215068	ENSG00000112992
ENSG00000248464	ENSG00000112996
ENSG00000251141	ENSG00000112996
ENSG00000249593	ENSG00000113013
ENSG00000254363	ENSG00000113068
ENSG00000278936	ENSG00000113068
ENSG00000280029	ENSG00000113068
ENSG00000249637	ENSG00000113070
ENSG00000278936	ENSG00000113070
ENSG00000280029	ENSG00000113070
ENSG00000249637	ENSG00000113108
ENSG00000254363	ENSG00000113108
ENSG00000280029	ENSG00000113108
ENSG00000278936	ENSG00000113119
ENSG00000280029	ENSG00000113119
ENSG00000197083	ENSG00000113140
ENSG00000249035	ENSG00000113140
ENSG00000260581	ENSG00000113140
ENSG00000270978	ENSG00000113140
ENSG00000248596	ENSG00000113194
ENSG00000262096	ENSG00000113205
ENSG00000272154	ENSG00000113205
ENSG00000278936	ENSG00000113205
ENSG00000280029	ENSG00000113205
ENSG00000272154	ENSG00000113209
ENSG00000278936	ENSG00000113209
ENSG00000280029	ENSG00000113209
ENSG00000254099	ENSG00000113211
ENSG00000272154	ENSG00000113211
ENSG00000280029	ENSG00000113211
ENSG00000272154	ENSG00000113212
ENSG00000280029	ENSG00000113212
ENSG00000146001	ENSG00000113248
ENSG00000262096	ENSG00000113248
ENSG00000272070	ENSG00000113248
ENSG00000278936	ENSG00000113248
ENSG00000280029	ENSG00000113248
ENSG00000254138	ENSG00000113361
ENSG00000250764	ENSG00000113384
ENSG00000254138	ENSG00000113384
ENSG00000215158	ENSG00000113407
ENSG00000249572	ENSG00000113407

ENSG00000247121	ENSG00000113441
ENSG00000248734	ENSG00000113441
ENSG00000251314	ENSG00000113441
ENSG00000278936	ENSG00000113555
ENSG00000280029	ENSG00000113555
ENSG00000273345	ENSG00000113583
ENSG00000244968	ENSG00000113594
ENSG00000249740	ENSG00000113594
ENSG00000224186	ENSG00000113621
ENSG00000224186	ENSG00000113648
ENSG00000224186	ENSG00000113658
ENSG00000230551	ENSG00000113712
ENSG00000253686	ENSG00000113719
ENSG00000254211	ENSG00000113719
ENSG00000253406	ENSG00000113721
ENSG00000253686	ENSG00000113732
ENSG00000254211	ENSG00000113732
ENSG00000253955	ENSG00000113739
ENSG00000254211	ENSG00000113739
ENSG00000253686	ENSG00000113742
ENSG00000246596	ENSG00000113758
ENSG00000247679	ENSG00000113758
ENSG00000248996	ENSG00000113758
ENSG00000223358	ENSG00000113790
ENSG00000239959	ENSG00000113805
ENSG00000163915	ENSG00000113838
ENSG00000197099	ENSG00000113838
ENSG00000197099	ENSG00000113916
ENSG00000228804	ENSG00000113916
ENSG00000240882	ENSG00000113924
ENSG00000248724	ENSG00000113971
ENSG00000272758	ENSG00000114013
ENSG00000242337	ENSG00000114019
ENSG00000224078	ENSG00000114062
ENSG00000259905	ENSG00000114062
ENSG00000248932	ENSG00000114115
ENSG00000249417	ENSG00000114125
ENSG00000241570	ENSG00000114126
ENSG00000249417	ENSG00000114126
ENSG00000228350	ENSG00000114268
ENSG00000223343	ENSG00000114270
ENSG00000271973	ENSG00000114270
ENSG00000233058	ENSG00000114315
ENSG00000223343	ENSG00000114316
ENSG00000235016	ENSG00000114316
ENSG00000224769	ENSG00000114331
ENSG00000225822	ENSG00000114331
ENSG00000233058	ENSG00000114331

ENSG00000235016	ENSG00000114353
ENSG00000235016	ENSG00000114388
ENSG00000230454	ENSG00000114395
ENSG00000239828	ENSG00000114439
ENSG00000243701	ENSG00000114439
ENSG00000273125	ENSG00000114439
ENSG00000214135	ENSG00000114473
ENSG00000246022	ENSG00000114520
ENSG00000250934	ENSG00000114520
ENSG00000239482	ENSG00000114529
ENSG00000261488	ENSG00000114529
ENSG00000250218	ENSG00000114544
ENSG00000250934	ENSG00000114544
ENSG00000246022	ENSG00000114554
ENSG00000251448	ENSG00000114554
ENSG00000243795	ENSG00000114573
ENSG00000259976	ENSG00000114573
ENSG00000244300	ENSG00000114626
ENSG00000227398	ENSG00000114646
ENSG00000227398	ENSG00000114650
ENSG00000228252	ENSG00000114670
ENSG00000235016	ENSG00000114735
ENSG00000235016	ENSG00000114738
ENSG00000240429	ENSG00000114757
ENSG00000272699	ENSG00000114757
ENSG00000240602	ENSG00000114771
ENSG00000243305	ENSG00000114771
ENSG00000250271	ENSG00000114771
ENSG00000233441	ENSG00000114796
ENSG00000234617	ENSG00000114812
ENSG00000242797	ENSG00000114841
ENSG00000240875	ENSG00000114850
ENSG00000243176	ENSG00000114850
ENSG00000241163	ENSG00000114861
ENSG00000223358	ENSG00000114867
ENSG00000223725	ENSG00000114948
ENSG00000228577	ENSG00000114948
ENSG00000279220	ENSG00000114948
ENSG00000163016	ENSG00000114978
ENSG00000225439	ENSG00000114978
ENSG00000257800	ENSG00000114978
ENSG00000228486	ENSG00000114982
ENSG00000163016	ENSG00000114993
ENSG00000243389	ENSG00000114999
ENSG00000243389	ENSG00000115008
ENSG00000230606	ENSG00000115073
ENSG00000189223	ENSG00000115084
ENSG00000240356	ENSG00000115084

ENSG00000228486	ENSG00000115085
ENSG00000228857	ENSG00000115091
ENSG00000240356	ENSG00000115091
ENSG00000224165	ENSG00000115129
ENSG00000223642	ENSG00000115183
ENSG00000234072	ENSG00000115207
ENSG00000234072	ENSG00000115216
ENSG00000227938	ENSG00000115226
ENSG00000227403	ENSG00000115233
ENSG00000235724	ENSG00000115233
ENSG00000234072	ENSG00000115234
ENSG00000266933	ENSG00000115255
ENSG00000267317	ENSG00000115257
ENSG00000226121	ENSG00000115263
ENSG00000235724	ENSG00000115263
ENSG00000267283	ENSG00000115266
ENSG00000267317	ENSG00000115266
ENSG00000272183	ENSG00000115274
ENSG00000163016	ENSG00000115282
ENSG00000225439	ENSG00000115282
ENSG00000267122	ENSG00000115286
ENSG00000280486	ENSG00000115286
ENSG00000163016	ENSG00000115307
ENSG00000236209	ENSG00000115307
ENSG00000257702	ENSG00000115307
ENSG00000272180	ENSG00000115355
ENSG00000234281	ENSG00000115365
ENSG00000233251	ENSG00000115380
ENSG00000272180	ENSG00000115380
ENSG00000228509	ENSG00000115419
ENSG00000226791	ENSG00000115446
ENSG00000235444	ENSG00000115457
ENSG00000236886	ENSG00000115457
ENSG00000235042	ENSG00000115461
ENSG00000236886	ENSG00000115461
ENSG00000271889	ENSG00000115484
ENSG00000228363	ENSG00000115525
ENSG00000281162	ENSG00000115590
ENSG00000226925	ENSG00000115594
ENSG00000238273	ENSG00000115641
ENSG00000231802	ENSG00000115661
ENSG00000223374	ENSG00000115677
ENSG00000218416	ENSG00000115685
ENSG00000227479	ENSG00000115685
ENSG00000233806	ENSG00000115685
ENSG00000224272	ENSG00000115694
ENSG00000233806	ENSG00000115694
ENSG00000235092	ENSG00000115738

ENSG00000235665	ENSG00000115738
ENSG00000236790	ENSG00000115738
ENSG00000232973	ENSG00000115825
ENSG00000235848	ENSG00000115825
ENSG00000232973	ENSG00000115828
ENSG00000236213	ENSG00000115828
ENSG00000224043	ENSG00000115839
ENSG00000225205	ENSG00000115840
ENSG00000231367	ENSG00000115841
ENSG00000232973	ENSG00000115841
ENSG00000235848	ENSG00000115841
ENSG00000224043	ENSG00000115850
ENSG00000231890	ENSG00000115850
ENSG00000236204	ENSG00000115884
ENSG00000231367	ENSG00000115904
ENSG00000232973	ENSG00000115904
ENSG00000237798	ENSG00000115935
ENSG00000230408	ENSG00000115942
ENSG00000237380	ENSG00000115966
ENSG00000228384	ENSG00000115998
ENSG00000228384	ENSG00000116005
ENSG00000266933	ENSG00000116017
ENSG00000163016	ENSG00000116096
ENSG00000278811	ENSG00000116128
ENSG00000231407	ENSG00000116132
ENSG00000175147	ENSG00000116138
ENSG00000224459	ENSG00000116138
ENSG00000269896	ENSG00000116151
ENSG00000232993	ENSG00000116171
ENSG00000242193	ENSG00000116191
ENSG00000254154	ENSG00000116191
ENSG00000242193	ENSG00000116194
ENSG00000254154	ENSG00000116194
ENSG00000242193	ENSG00000116199
ENSG00000254154	ENSG00000116199
ENSG00000219102	ENSG00000116205
ENSG00000241720	ENSG00000116299
ENSG00000224699	ENSG00000116337
ENSG00000227963	ENSG00000116337
ENSG00000235407	ENSG00000116337
ENSG00000240194	ENSG00000116337
ENSG00000241720	ENSG00000116337
ENSG00000235407	ENSG00000116455
ENSG00000240194	ENSG00000116455
ENSG00000227811	ENSG00000116459
ENSG00000236065	ENSG00000116478
ENSG00000224167	ENSG00000116489
ENSG00000226419	ENSG00000116489

ENSG00000238198	ENSG00000116489
ENSG00000225313	ENSG00000116514
ENSG00000282111	ENSG00000116574
ENSG00000236947	ENSG00000116584
ENSG00000246203	ENSG00000116584
ENSG00000272405	ENSG00000116604
ENSG00000235545	ENSG00000116641
ENSG00000232860	ENSG00000116698
ENSG00000229956	ENSG00000116761
ENSG00000238198	ENSG00000116774
ENSG00000175147	ENSG00000116786
ENSG00000224459	ENSG00000116786
ENSG00000237938	ENSG00000116786
ENSG00000080947	ENSG00000116809
ENSG00000175147	ENSG00000116809
ENSG00000186715	ENSG00000116809
ENSG00000226029	ENSG00000116809
ENSG00000268869	ENSG00000116809
ENSG00000236137	ENSG00000116815
ENSG00000116883	ENSG00000116863
ENSG00000224238	ENSG00000116874
ENSG00000198857	ENSG00000116882
ENSG00000224238	ENSG00000116882
ENSG00000231365	ENSG00000116882
ENSG00000228436	ENSG00000116954
ENSG00000230325	ENSG00000116977
ENSG00000228436	ENSG00000116981
ENSG00000225333	ENSG00000116983
ENSG00000182109	ENSG00000116990
ENSG00000237899	ENSG00000116990
ENSG00000243970	ENSG00000116990
ENSG00000237899	ENSG00000117013
ENSG00000281207	ENSG00000117016
ENSG00000219201	ENSG00000117069
ENSG00000228863	ENSG00000117091
ENSG00000226526	ENSG00000117115
ENSG00000268869	ENSG00000117115
ENSG00000122432	ENSG00000117133
ENSG00000223653	ENSG00000117133
ENSG00000243323	ENSG00000117139
ENSG00000232995	ENSG00000117143
ENSG00000122432	ENSG00000117151
ENSG00000223653	ENSG00000117151
ENSG00000240520	ENSG00000117151
ENSG00000223653	ENSG00000117174
ENSG00000261737	ENSG00000117174
ENSG00000282057	ENSG00000117174
ENSG00000230735	ENSG00000117228

ENSG00000231999	ENSG00000117228
ENSG00000237505	ENSG00000117228
ENSG00000225603	ENSG00000117262
ENSG00000234222	ENSG00000117262
ENSG00000237188	ENSG00000117262
ENSG00000278811	ENSG00000117262
ENSG00000281406	ENSG00000117266
ENSG00000231105	ENSG00000117298
ENSG00000225315	ENSG00000117305
ENSG00000236810	ENSG00000117305
ENSG00000225315	ENSG00000117308
ENSG00000264443	ENSG00000117308
ENSG00000203709	ENSG00000117335
ENSG00000227533	ENSG00000117385
ENSG00000228192	ENSG00000117385
ENSG00000227533	ENSG00000117394
ENSG00000227533	ENSG00000117395
ENSG00000230615	ENSG00000117395
ENSG00000230615	ENSG00000117407
ENSG00000229348	ENSG00000117408
ENSG00000230615	ENSG00000117408
ENSG00000230615	ENSG00000117411
ENSG00000230615	ENSG00000117419
ENSG00000230615	ENSG00000117425
ENSG00000232022	ENSG00000117448
ENSG00000232022	ENSG00000117472
ENSG00000232022	ENSG00000117480
ENSG00000232022	ENSG00000117481
ENSG00000223745	ENSG00000117500
ENSG00000223745	ENSG00000117505
ENSG00000224081	ENSG00000117525
ENSG00000226026	ENSG00000117525
ENSG00000224081	ENSG00000117528
ENSG00000228852	ENSG00000117528
ENSG00000235501	ENSG00000117528
ENSG00000233184	ENSG00000117543
ENSG00000232878	ENSG00000117569
ENSG00000264443	ENSG00000117616
ENSG00000228172	ENSG00000117640
ENSG00000236155	ENSG00000117682
ENSG00000230461	ENSG00000117707
ENSG00000227742	ENSG00000117862
ENSG00000266993	ENSG00000117862
ENSG00000233038	ENSG00000117868
ENSG00000259495	ENSG00000117899
ENSG00000259581	ENSG00000117906
ENSG00000260776	ENSG00000117906
ENSG00000233930	ENSG00000117984

ENSG00000269821	ENSG00000117984
ENSG00000255422	ENSG00000118058
ENSG00000255435	ENSG00000118058
ENSG00000235910	ENSG00000118137
ENSG00000268001	ENSG00000118162
ENSG00000225166	ENSG00000118242
ENSG00000232485	ENSG00000118242
ENSG00000236886	ENSG00000118242
ENSG00000225216	ENSG00000118257
ENSG00000223725	ENSG00000118260
ENSG00000223725	ENSG00000118263
ENSG00000279220	ENSG00000118263
ENSG00000225793	ENSG00000118407
ENSG00000237174	ENSG00000118407
ENSG00000229956	ENSG00000118454
ENSG00000229036	ENSG00000118495
ENSG00000278206	ENSG00000118495
ENSG00000227220	ENSG00000118507
ENSG00000227678	ENSG00000118507
ENSG00000220494	ENSG00000118508
ENSG00000233452	ENSG00000118508
ENSG00000235652	ENSG00000118508
ENSG00000231028	ENSG00000118513
ENSG00000236700	ENSG00000118513
ENSG00000237596	ENSG00000118513
ENSG00000227954	ENSG00000118515
ENSG00000236700	ENSG00000118515
ENSG00000227945	ENSG00000118518
ENSG00000227220	ENSG00000118523
ENSG00000227954	ENSG00000118526
ENSG00000231205	ENSG00000118620
ENSG00000268278	ENSG00000118620
ENSG00000268555	ENSG00000118620
ENSG00000268658	ENSG00000118620
ENSG00000243150	ENSG00000118849
ENSG00000267072	ENSG00000118894
ENSG00000267072	ENSG00000118900
ENSG00000232800	ENSG00000118960
ENSG00000270100	ENSG00000118961
ENSG00000236204	ENSG00000118965
ENSG00000247121	ENSG00000118985
ENSG00000251314	ENSG00000118985
ENSG00000204706	ENSG00000119138
ENSG00000235092	ENSG00000119203
ENSG00000239300	ENSG00000119203
ENSG00000271855	ENSG00000119203
ENSG00000214135	ENSG00000119231
ENSG00000270130	ENSG00000119242

ENSG00000225684	ENSG00000119314
ENSG00000228623	ENSG00000119314
ENSG00000230185	ENSG00000119314
ENSG00000259953	ENSG00000119314
ENSG00000228623	ENSG00000119321
ENSG00000230185	ENSG00000119321
ENSG00000204055	ENSG00000119383
ENSG00000228395	ENSG00000119392
ENSG00000226752	ENSG00000119403
ENSG00000228623	ENSG00000119411
ENSG00000230185	ENSG00000119411
ENSG00000228623	ENSG00000119457
ENSG00000228623	ENSG00000119471
ENSG00000230185	ENSG00000119471
ENSG00000237461	ENSG00000119508
ENSG00000237461	ENSG00000119509
ENSG00000233621	ENSG00000119535
ENSG00000267390	ENSG00000119537
ENSG00000267787	ENSG00000119547
ENSG00000266933	ENSG00000119559
ENSG00000213753	ENSG00000119574
ENSG00000232098	ENSG00000119574
ENSG00000267858	ENSG00000119574
ENSG00000268230	ENSG00000119574
ENSG00000224721	ENSG00000119616
ENSG00000258891	ENSG00000119616
ENSG00000258603	ENSG00000119636
ENSG00000259138	ENSG00000119638
ENSG00000246548	ENSG00000119669
ENSG00000258569	ENSG00000119669
ENSG00000273729	ENSG00000119669
ENSG00000259319	ENSG00000119684
ENSG00000259687	ENSG00000119684
ENSG00000224721	ENSG00000119689
ENSG00000258891	ENSG00000119689
ENSG00000258301	ENSG00000119699
ENSG00000259138	ENSG00000119703
ENSG00000259319	ENSG00000119703
ENSG00000251393	ENSG00000119711
ENSG00000258875	ENSG00000119714
ENSG00000258891	ENSG00000119725
ENSG00000259065	ENSG00000119725
ENSG00000234072	ENSG00000119760
ENSG00000223935	ENSG00000119844
ENSG00000260101	ENSG00000119844
ENSG00000223935	ENSG00000119862
ENSG00000271889	ENSG00000119866
ENSG00000250116	ENSG00000119878

ENSG00000232295	ENSG00000119900
ENSG00000233237	ENSG00000119900
ENSG00000227695	ENSG00000119906
ENSG00000226688	ENSG00000119977
ENSG00000226009	ENSG00000120029
ENSG00000282772	ENSG00000120029
ENSG00000235823	ENSG00000120053
ENSG00000231970	ENSG00000120057
ENSG00000264057	ENSG00000120063
ENSG00000253686	ENSG00000120129
ENSG00000225408	ENSG00000120215
ENSG00000225489	ENSG00000120215
ENSG00000228739	ENSG00000120217
ENSG00000268592	ENSG00000120256
ENSG00000278936	ENSG00000120318
ENSG00000280029	ENSG00000120318
ENSG00000272154	ENSG00000120324
ENSG00000278936	ENSG00000120324
ENSG00000280029	ENSG00000120324
ENSG00000272154	ENSG00000120327
ENSG00000278936	ENSG00000120327
ENSG00000280029	ENSG00000120327
ENSG00000272154	ENSG00000120328
ENSG00000278936	ENSG00000120328
ENSG00000280029	ENSG00000120328
ENSG00000231407	ENSG00000120370
ENSG00000245498	ENSG00000120458
ENSG00000249695	ENSG00000120645
ENSG00000250132	ENSG00000120645
ENSG00000168852	ENSG00000120662
ENSG00000215483	ENSG00000120662
ENSG00000223685	ENSG00000120686
ENSG00000235550	ENSG00000120686
ENSG00000224743	ENSG00000120694
ENSG00000120664	ENSG00000120697
ENSG00000224186	ENSG00000120708
ENSG00000249593	ENSG00000120725
ENSG00000257681	ENSG00000120820
ENSG00000251191	ENSG00000120875
ENSG00000259607	ENSG00000120875
ENSG00000253125	ENSG00000120889
ENSG00000253837	ENSG00000120889
ENSG00000253125	ENSG00000120896
ENSG00000253200	ENSG00000120896
ENSG00000253837	ENSG00000120896
ENSG00000254230	ENSG00000120896
ENSG00000251034	ENSG00000120910
ENSG00000253125	ENSG00000120910

ENSG00000253837	ENSG00000120910
ENSG00000265542	ENSG00000121060
ENSG00000265542	ENSG00000121064
ENSG00000187013	ENSG00000121068
ENSG00000213753	ENSG00000121410
ENSG00000232098	ENSG00000121410
ENSG00000267858	ENSG00000121410
ENSG00000268230	ENSG00000121410
ENSG00000268895	ENSG00000121410
ENSG00000269473	ENSG00000121410
ENSG00000242622	ENSG00000121577
ENSG00000243795	ENSG00000121579
ENSG00000259976	ENSG00000121579
ENSG00000240254	ENSG00000121594
ENSG00000254427	ENSG00000121653
ENSG00000078319	ENSG00000121716
ENSG00000242294	ENSG00000121716
ENSG00000272752	ENSG00000121716
ENSG00000235790	ENSG00000121764
ENSG00000220785	ENSG00000121775
ENSG00000236065	ENSG00000121775
ENSG00000227398	ENSG00000121807
ENSG00000272699	ENSG00000121864
ENSG00000205794	ENSG00000121892
ENSG00000249348	ENSG00000121892
ENSG00000236065	ENSG00000121903
ENSG00000241720	ENSG00000121940
ENSG00000232377	ENSG00000121964
ENSG00000224043	ENSG00000121988
ENSG00000250889	ENSG00000122008
ENSG00000275294	ENSG00000122026
ENSG00000179141	ENSG00000122042
ENSG00000214135	ENSG00000122068
ENSG00000225361	ENSG00000122140
ENSG00000226706	ENSG00000122140
ENSG00000237339	ENSG00000122140
ENSG00000248596	ENSG00000122203
ENSG00000251458	ENSG00000122203
ENSG00000224985	ENSG00000122218
ENSG00000228863	ENSG00000122218
ENSG00000260448	ENSG00000122257
ENSG00000225484	ENSG00000122359
ENSG00000278616	ENSG00000122359
ENSG00000196566	ENSG00000122367
ENSG00000225913	ENSG00000122367
ENSG00000272631	ENSG00000122367
ENSG00000223482	ENSG00000122376
ENSG00000224914	ENSG00000122376

ENSG00000225484	ENSG00000122378
ENSG00000260565	ENSG00000122386
ENSG00000261093	ENSG00000122386
ENSG00000262370	ENSG00000122386
ENSG00000263072	ENSG00000122386
ENSG00000260565	ENSG00000122390
ENSG00000235927	ENSG00000122420
ENSG00000233184	ENSG00000122477
ENSG00000226026	ENSG00000122481
ENSG00000228852	ENSG00000122481
ENSG00000233593	ENSG00000122482
ENSG00000261126	ENSG00000122490
ENSG00000267270	ENSG00000122490
ENSG00000214783	ENSG00000122515
ENSG00000239556	ENSG00000122515
ENSG00000273432	ENSG00000122515
ENSG00000228878	ENSG00000122557
ENSG00000271122	ENSG00000122557
ENSG00000214870	ENSG00000122565
ENSG00000214870	ENSG00000122566
ENSG00000227855	ENSG00000122574
ENSG00000226329	ENSG00000122591
ENSG00000205763	ENSG00000122642
ENSG00000214783	ENSG00000122678
ENSG00000228903	ENSG00000122678
ENSG00000273432	ENSG00000122678
ENSG00000235453	ENSG00000122692
ENSG00000227388	ENSG00000122707
ENSG00000230074	ENSG00000122733
ENSG00000261215	ENSG00000122733
ENSG00000281205	ENSG00000122733
ENSG00000231794	ENSG00000122783
ENSG00000272941	ENSG00000122783
ENSG00000273297	ENSG00000122783
ENSG00000273297	ENSG00000122786
ENSG00000221817	ENSG00000122861
ENSG00000271848	ENSG00000122861
ENSG00000229261	ENSG00000122862
ENSG00000236756	ENSG00000122882
ENSG00000242338	ENSG00000122882
ENSG00000271816	ENSG00000122882
ENSG00000271848	ENSG00000122882
ENSG00000272630	ENSG00000122882
ENSG00000236756	ENSG00000122884
ENSG00000271848	ENSG00000122884
ENSG00000258210	ENSG00000122970
ENSG00000227742	ENSG00000123080
ENSG00000227742	ENSG00000123091

ENSG00000246695	ENSG00000123094
ENSG00000255750	ENSG00000123094
ENSG00000256234	ENSG00000123094
ENSG00000255750	ENSG00000123095
ENSG00000255968	ENSG00000123095
ENSG00000256234	ENSG00000123095
ENSG00000255968	ENSG00000123096
ENSG00000255750	ENSG00000123104
ENSG00000255968	ENSG00000123104
ENSG00000256234	ENSG00000123104
ENSG00000256377	ENSG00000123104
ENSG00000254231	ENSG00000123124
ENSG00000219665	ENSG00000123144
ENSG00000257588	ENSG00000123268
ENSG00000248265	ENSG00000123338
ENSG00000258086	ENSG00000123338
ENSG00000182796	ENSG00000123342
ENSG00000257735	ENSG00000123352
ENSG00000258017	ENSG00000123352
ENSG00000258101	ENSG00000123352
ENSG00000257616	ENSG00000123358
ENSG00000250451	ENSG00000123360
ENSG00000258086	ENSG00000123360
ENSG00000258317	ENSG00000123374
ENSG00000248265	ENSG00000123415
ENSG00000250742	ENSG00000123415
ENSG00000257346	ENSG00000123416
ENSG00000257735	ENSG00000123416
ENSG00000257913	ENSG00000123416
ENSG00000258017	ENSG00000123416
ENSG00000258057	ENSG00000123416
ENSG00000255197	ENSG00000123444
ENSG00000225762	ENSG00000123472
ENSG00000232022	ENSG00000123472
ENSG00000240050	ENSG00000123500
ENSG00000231154	ENSG00000123562
ENSG00000231154	ENSG00000123570
ENSG00000229083	ENSG00000123595
ENSG00000234350	ENSG00000123600
ENSG00000224490	ENSG00000123607
ENSG00000223642	ENSG00000123636
ENSG00000226266	ENSG00000123636
ENSG00000249035	ENSG00000123643
ENSG00000153363	ENSG00000123684
ENSG00000203706	ENSG00000123689
ENSG00000232160	ENSG00000123728
ENSG00000247950	ENSG00000123739
ENSG00000256612	ENSG00000123810

ENSG00000237118	ENSG00000123815
ENSG00000256612	ENSG00000123815
ENSG00000267107	ENSG00000123815
ENSG00000162888	ENSG00000123843
ENSG00000244558	ENSG00000124120
ENSG00000244558	ENSG00000124134
ENSG00000271984	ENSG00000124145
ENSG00000275894	ENSG00000124145
ENSG00000204044	ENSG00000124155
ENSG00000244558	ENSG00000124155
ENSG00000269549	ENSG00000124155
ENSG00000273828	ENSG00000124155
ENSG00000269549	ENSG00000124160
ENSG00000223891	ENSG00000124193
ENSG00000124224	ENSG00000124225
ENSG00000244558	ENSG00000124249
ENSG00000244558	ENSG00000124251
ENSG00000269549	ENSG00000124251
ENSG00000273828	ENSG00000124251
ENSG00000124224	ENSG00000124253
ENSG00000204044	ENSG00000124257
ENSG00000269549	ENSG00000124257
ENSG00000273828	ENSG00000124257
ENSG00000232593	ENSG00000124313
ENSG00000228384	ENSG00000124370
ENSG00000233098	ENSG00000124422
ENSG00000266903	ENSG00000124459
ENSG00000236276	ENSG00000124479
ENSG00000224843	ENSG00000124508
ENSG00000237686	ENSG00000124541
ENSG00000224846	ENSG00000124570
ENSG00000228170	ENSG00000124570
ENSG00000230438	ENSG00000124570
ENSG00000244041	ENSG00000124570
ENSG00000271361	ENSG00000124570
ENSG00000231881	ENSG00000124571
ENSG00000237686	ENSG00000124571
ENSG00000245261	ENSG00000124571
ENSG00000237686	ENSG00000124574
ENSG00000181524	ENSG00000124587
ENSG00000231113	ENSG00000124587
ENSG00000224846	ENSG00000124588
ENSG00000244041	ENSG00000124588
ENSG00000261068	ENSG00000124593
ENSG00000237686	ENSG00000124608
ENSG00000271755	ENSG00000124613
ENSG00000224843	ENSG00000124635
ENSG00000281706	ENSG00000124635

ENSG00000203362	ENSG00000124733
ENSG00000245261	ENSG00000124733
ENSG00000216775	ENSG00000124743
ENSG00000227885	ENSG00000124743
ENSG00000231683	ENSG00000124743
ENSG00000224666	ENSG00000124762
ENSG00000238221	ENSG00000124783
ENSG00000271978	ENSG00000124787
ENSG00000272341	ENSG00000124788
ENSG00000249942	ENSG00000124875
ENSG00000249942	ENSG00000124882
ENSG00000124915	ENSG00000124920
ENSG00000243742	ENSG00000124942
ENSG00000255717	ENSG00000124942
ENSG00000249767	ENSG00000125089
ENSG00000270049	ENSG00000125122
ENSG00000260240	ENSG00000125166
ENSG00000260467	ENSG00000125170
ENSG00000258545	ENSG00000125354
ENSG00000233382	ENSG00000125355
ENSG00000237356	ENSG00000125384
ENSG00000249673	ENSG00000125388
ENSG00000227036	ENSG00000125398
ENSG00000236088	ENSG00000125430
ENSG00000203761	ENSG00000125459
ENSG00000246203	ENSG00000125459
ENSG00000267265	ENSG00000125505
ENSG00000267838	ENSG00000125505
ENSG00000277496	ENSG00000125531
ENSG00000189223	ENSG00000125538
ENSG00000240356	ENSG00000125538
ENSG00000240356	ENSG00000125637
ENSG00000266983	ENSG00000125648
ENSG00000268758	ENSG00000125648
ENSG00000266983	ENSG00000125650
ENSG00000269802	ENSG00000125650
ENSG00000269802	ENSG00000125656
ENSG00000214546	ENSG00000125686
ENSG00000265460	ENSG00000125686
ENSG00000266983	ENSG00000125730
ENSG00000269680	ENSG00000125731
ENSG00000266983	ENSG00000125733
ENSG00000269680	ENSG00000125733
ENSG00000269802	ENSG00000125733
ENSG00000266903	ENSG00000125740
ENSG00000266903	ENSG00000125741
ENSG00000267348	ENSG00000125741
ENSG00000266903	ENSG00000125744

ENSG00000267348	ENSG00000125744
ENSG00000267757	ENSG00000125744
ENSG00000266903	ENSG00000125746
ENSG00000267348	ENSG00000125746
ENSG00000266903	ENSG00000125753
ENSG00000267348	ENSG00000125753
ENSG00000275632	ENSG00000125772
ENSG00000278719	ENSG00000125772
ENSG00000234684	ENSG00000125775
ENSG00000235958	ENSG00000125779
ENSG00000235958	ENSG00000125817
ENSG00000229766	ENSG00000125827
ENSG00000225377	ENSG00000125841
ENSG00000234684	ENSG00000125875
ENSG00000225377	ENSG00000125895
ENSG00000267551	ENSG00000125910
ENSG00000225315	ENSG00000125944
ENSG00000236810	ENSG00000125945
ENSG00000249087	ENSG00000125945
ENSG00000270050	ENSG00000125962
ENSG00000271147	ENSG00000125962
ENSG00000088340	ENSG00000125966
ENSG00000230753	ENSG00000125970
ENSG00000230753	ENSG00000125971
ENSG00000088340	ENSG00000125991
ENSG00000260032	ENSG00000125991
ENSG00000232593	ENSG00000126012
ENSG00000235016	ENSG00000126062
ENSG00000230615	ENSG00000126088
ENSG00000227533	ENSG00000126091
ENSG00000230615	ENSG00000126091
ENSG00000230615	ENSG00000126106
ENSG00000230615	ENSG00000126107
ENSG00000281912	ENSG00000126107
ENSG00000235280	ENSG00000126217
ENSG00000233695	ENSG00000126218
ENSG00000267874	ENSG00000126243
ENSG00000271032	ENSG00000126243
ENSG00000271366	ENSG00000126243
ENSG00000205786	ENSG00000126247
ENSG00000267698	ENSG00000126247
ENSG00000271032	ENSG00000126261
ENSG00000271366	ENSG00000126261
ENSG00000267049	ENSG00000126262
ENSG00000267698	ENSG00000126262
ENSG00000265460	ENSG00000126368
ENSG00000245532	ENSG00000126391
ENSG00000251562	ENSG00000126391

ENSG00000235191	ENSG00000126457
ENSG00000243829	ENSG00000126457
ENSG00000268006	ENSG00000126457
ENSG00000268854	ENSG00000126457
ENSG00000161643	ENSG00000126458
ENSG00000268006	ENSG00000126458
ENSG00000273189	ENSG00000126458
ENSG00000268006	ENSG00000126461
ENSG00000164669	ENSG00000126522
ENSG00000224316	ENSG00000126522
ENSG00000226002	ENSG00000126522
ENSG00000230189	ENSG00000126522
ENSG00000234185	ENSG00000126522
ENSG00000272693	ENSG00000126522
ENSG00000226824	ENSG00000126524
ENSG00000229180	ENSG00000126524
ENSG00000230189	ENSG00000126524
ENSG00000232546	ENSG00000126524
ENSG00000234500	ENSG00000126524
ENSG00000213373	ENSG00000126561
ENSG00000267765	ENSG00000126561
ENSG00000267072	ENSG00000126603
ENSG00000205885	ENSG00000126749
ENSG00000240370	ENSG00000126749
ENSG00000204620	ENSG00000126759
ENSG00000204620	ENSG00000126767
ENSG00000259868	ENSG00000126777
ENSG00000234911	ENSG00000126804
ENSG00000258824	ENSG00000126804
ENSG00000259116	ENSG00000126804
ENSG00000272909	ENSG00000126804
ENSG00000258824	ENSG00000126821
ENSG00000214719	ENSG00000126860
ENSG00000264538	ENSG00000126860
ENSG00000266865	ENSG00000126860
ENSG00000236986	ENSG00000126883
ENSG00000261101	ENSG00000126947
ENSG00000254035	ENSG00000127022
ENSG00000187984	ENSG00000127081
ENSG00000224809	ENSG00000127081
ENSG00000225511	ENSG00000127081
ENSG00000275318	ENSG00000127081
ENSG00000230638	ENSG00000127124
ENSG00000235872	ENSG00000127314
ENSG00000247131	ENSG00000127328
ENSG00000256248	ENSG00000127334
ENSG00000196810	ENSG00000127418
ENSG00000267106	ENSG00000127445

ENSG00000230424	ENSG00000127463
ENSG00000272084	ENSG00000127463
ENSG00000230424	ENSG00000127481
ENSG00000117242	ENSG00000127483
ENSG00000187952	ENSG00000127483
ENSG00000269481	ENSG00000127511
ENSG00000268087	ENSG00000127526
ENSG00000269439	ENSG00000127526
ENSG00000269481	ENSG00000127526
ENSG00000269085	ENSG00000127533
ENSG00000269439	ENSG00000127533
ENSG00000215154	ENSG00000127554
ENSG00000261790	ENSG00000127554
ENSG00000236829	ENSG00000127580
ENSG00000261641	ENSG00000127580
ENSG00000261659	ENSG00000127580
ENSG00000260425	ENSG00000127586
ENSG00000182109	ENSG00000127603
ENSG00000243970	ENSG00000127603
ENSG00000266978	ENSG00000127616
ENSG00000267274	ENSG00000127616
ENSG00000267571	ENSG00000127663
ENSG00000269807	ENSG00000127663
ENSG00000223923	ENSG00000127837
ENSG00000223923	ENSG00000127838
ENSG00000275294	ENSG00000127870
ENSG00000166917	ENSG00000127884
ENSG00000214279	ENSG00000127884
ENSG00000188693	ENSG00000127914
ENSG00000236861	ENSG00000127920
ENSG00000231764	ENSG00000127922
ENSG00000205485	ENSG00000127946
ENSG00000186704	ENSG00000127947
ENSG00000214293	ENSG00000127947
ENSG00000205485	ENSG00000127951
ENSG00000214439	ENSG00000127951
ENSG00000227038	ENSG00000127952
ENSG00000244055	ENSG00000127980
ENSG00000188693	ENSG00000127989
ENSG00000236861	ENSG00000127990
ENSG00000233622	ENSG00000128000
ENSG00000256612	ENSG00000128000
ENSG00000248115	ENSG00000128045
ENSG00000249341	ENSG00000128045
ENSG00000245067	ENSG00000128050
ENSG00000184319	ENSG00000128159
ENSG00000273253	ENSG00000128159
ENSG00000161132	ENSG00000128185

ENSG00000243762	ENSG00000128191
ENSG00000225465	ENSG00000128250
ENSG00000178248	ENSG00000128266
ENSG00000215478	ENSG00000128266
ENSG00000234353	ENSG00000128266
ENSG00000280623	ENSG00000128266
ENSG00000128262	ENSG00000128271
ENSG00000178803	ENSG00000128271
ENSG00000218537	ENSG00000128271
ENSG00000272578	ENSG00000128271
ENSG00000272733	ENSG00000128271
ENSG00000273000	ENSG00000128271
ENSG00000279467	ENSG00000128271
ENSG00000280623	ENSG00000128271
ENSG00000172250	ENSG00000128274
ENSG00000235237	ENSG00000128283
ENSG00000244627	ENSG00000128283
ENSG00000279010	ENSG00000128283
ENSG00000183822	ENSG00000128284
ENSG00000225783	ENSG00000128294
ENSG00000233360	ENSG00000128309
ENSG00000279010	ENSG00000128309
ENSG00000183822	ENSG00000128311
ENSG00000244627	ENSG00000128346
ENSG00000279010	ENSG00000128346
ENSG00000279738	ENSG00000128346
ENSG00000244627	ENSG00000128383
ENSG00000279010	ENSG00000128383
ENSG00000233739	ENSG00000128394
ENSG00000244627	ENSG00000128394
ENSG00000224897	ENSG00000128513
ENSG00000224899	ENSG00000128513
ENSG00000242593	ENSG00000128513
ENSG00000078319	ENSG00000128564
ENSG00000226380	ENSG00000128585
ENSG00000231721	ENSG00000128585
ENSG00000233559	ENSG00000128585
ENSG00000236753	ENSG00000128585
ENSG00000230626	ENSG00000128591
ENSG00000243302	ENSG00000128591
ENSG00000280828	ENSG00000128591
ENSG00000240758	ENSG00000128595
ENSG00000272899	ENSG00000128595
ENSG00000280828	ENSG00000128595
ENSG00000240571	ENSG00000128602
ENSG00000243302	ENSG00000128602
ENSG00000272899	ENSG00000128602
ENSG00000226380	ENSG00000128607

ENSG00000231721	ENSG00000128607
ENSG00000232716	ENSG00000128607
ENSG00000233559	ENSG00000128607
ENSG00000242593	ENSG00000128609
ENSG00000272686	ENSG00000128609
ENSG00000230626	ENSG00000128617
ENSG00000273270	ENSG00000128617
ENSG00000227542	ENSG00000128641
ENSG00000206149	ENSG00000128731
ENSG00000248334	ENSG00000128731
ENSG00000224078	ENSG00000128739
ENSG00000259905	ENSG00000128739
ENSG00000226576	ENSG00000128815
ENSG00000248508	ENSG00000128829
ENSG00000259345	ENSG00000128829
ENSG00000258461	ENSG00000128881
ENSG00000259536	ENSG00000128891
ENSG00000157021	ENSG00000128908
ENSG00000261183	ENSG00000128928
ENSG00000260469	ENSG00000129009
ENSG00000277749	ENSG00000129009
ENSG00000242337	ENSG00000129055
ENSG00000248980	ENSG00000129128
ENSG00000254966	ENSG00000129167
ENSG00000177822	ENSG00000129187
ENSG00000215067	ENSG00000129195
ENSG00000256806	ENSG00000129195
ENSG00000235085	ENSG00000129197
ENSG00000263272	ENSG00000129197
ENSG00000261879	ENSG00000129204
ENSG00000215067	ENSG00000129214
ENSG00000256806	ENSG00000129214
ENSG00000261879	ENSG00000129219
ENSG00000215067	ENSG00000129244
ENSG00000256806	ENSG00000129244
ENSG00000215067	ENSG00000129245
ENSG00000256806	ENSG00000129245
ENSG00000265749	ENSG00000129245
ENSG00000261879	ENSG00000129250
ENSG00000215067	ENSG00000129255
ENSG00000256806	ENSG00000129255
ENSG00000257735	ENSG00000129315
ENSG00000269514	ENSG00000129315
ENSG00000266936	ENSG00000129351
ENSG00000266978	ENSG00000129351
ENSG00000266978	ENSG00000129355
ENSG00000267984	ENSG00000129450
ENSG00000269235	ENSG00000129450

ENSG00000215256	ENSG00000129465
ENSG00000157306	ENSG00000129467
ENSG00000258441	ENSG00000129472
ENSG00000259102	ENSG00000129472
ENSG00000157306	ENSG00000129473
ENSG00000215256	ENSG00000129473
ENSG00000251002	ENSG00000129473
ENSG00000275552	ENSG00000129473
ENSG00000157306	ENSG00000129474
ENSG00000251002	ENSG00000129474
ENSG00000258458	ENSG00000129474
ENSG00000258580	ENSG00000129480
ENSG00000215256	ENSG00000129535
ENSG00000260669	ENSG00000129535
ENSG00000258441	ENSG00000129538
ENSG00000258451	ENSG00000129538
ENSG00000248445	ENSG00000129596
ENSG00000270067	ENSG00000129625
ENSG00000267078	ENSG00000129646
ENSG00000267546	ENSG00000129646
ENSG00000253161	ENSG00000129691
ENSG00000261526	ENSG00000129911
ENSG00000236829	ENSG00000129925
ENSG00000267551	ENSG00000129932
ENSG00000266904	ENSG00000129933
ENSG00000266933	ENSG00000129946
ENSG00000267265	ENSG00000129991
ENSG00000267577	ENSG00000129991
ENSG00000276570	ENSG00000129991
ENSG00000248015	ENSG00000130005
ENSG00000226445	ENSG00000130024
ENSG00000227704	ENSG00000130024
ENSG00000272848	ENSG00000130024
ENSG00000219665	ENSG00000130158
ENSG00000267082	ENSG00000130158
ENSG00000219665	ENSG00000130159
ENSG00000266978	ENSG00000130164
ENSG00000267274	ENSG00000130165
ENSG00000234773	ENSG00000130173
ENSG00000266978	ENSG00000130173
ENSG00000267082	ENSG00000130173
ENSG00000267082	ENSG00000130175
ENSG00000266903	ENSG00000130202
ENSG00000266903	ENSG00000130203
ENSG00000267282	ENSG00000130203
ENSG00000214855	ENSG00000130204
ENSG00000266903	ENSG00000130204
ENSG00000267348	ENSG00000130204

ENSG00000266903	ENSG00000130208
ENSG00000267348	ENSG00000130208
ENSG00000271826	ENSG00000130224
ENSG00000253125	ENSG00000130227
ENSG00000266983	ENSG00000130254
ENSG00000269802	ENSG00000130254
ENSG00000268087	ENSG00000130299
ENSG00000269439	ENSG00000130303
ENSG00000269481	ENSG00000130303
ENSG00000269439	ENSG00000130304
ENSG00000268056	ENSG00000130307
ENSG00000273654	ENSG00000130307
ENSG00000273654	ENSG00000130309
ENSG00000268087	ENSG00000130313
ENSG00000269439	ENSG00000130313
ENSG00000273654	ENSG00000130313
ENSG00000271913	ENSG00000130338
ENSG00000271913	ENSG00000130363
ENSG00000269191	ENSG00000130477
ENSG00000273654	ENSG00000130477
ENSG00000268112	ENSG00000130479
ENSG00000273253	ENSG00000130489
ENSG00000273654	ENSG00000130511
ENSG00000269439	ENSG00000130522
ENSG00000232871	ENSG00000130529
ENSG00000235191	ENSG00000130529
ENSG00000232442	ENSG00000130589
ENSG00000226416	ENSG00000130592
ENSG00000233930	ENSG00000130592
ENSG00000238184	ENSG00000130592
ENSG00000269821	ENSG00000130592
ENSG00000225361	ENSG00000130635
ENSG00000197182	ENSG00000130638
ENSG00000280383	ENSG00000130638
ENSG00000214279	ENSG00000130640
ENSG00000213742	ENSG00000130684
ENSG00000230289	ENSG00000130714
ENSG00000213753	ENSG00000130724
ENSG00000267858	ENSG00000130724
ENSG00000268516	ENSG00000130724
ENSG00000213753	ENSG00000130725
ENSG00000268201	ENSG00000130725
ENSG00000268230	ENSG00000130725
ENSG00000268516	ENSG00000130725
ENSG00000268895	ENSG00000130725
ENSG00000269473	ENSG00000130725
ENSG00000213753	ENSG00000130726
ENSG00000232098	ENSG00000130726

ENSG00000268516	ENSG00000130726
ENSG00000266978	ENSG00000130733
ENSG00000266978	ENSG00000130734
ENSG00000268621	ENSG00000130748
ENSG00000268810	ENSG00000130749
ENSG00000205041	ENSG00000130758
ENSG00000266936	ENSG00000130810
ENSG00000266978	ENSG00000130810
ENSG00000274425	ENSG00000130810
ENSG00000266936	ENSG00000130811
ENSG00000266978	ENSG00000130813
ENSG00000232725	ENSG00000130821
ENSG00000232725	ENSG00000130822
ENSG00000260081	ENSG00000130822
ENSG00000197180	ENSG00000130827
ENSG00000232725	ENSG00000130827
ENSG00000228986	ENSG00000130830
ENSG00000242779	ENSG00000130844
ENSG00000228526	ENSG00000130939
ENSG00000175611	ENSG00000130958
ENSG00000225194	ENSG00000130958
ENSG00000226679	ENSG00000130962
ENSG00000259977	ENSG00000130962
ENSG00000215284	ENSG00000130985
ENSG00000224401	ENSG00000130985
ENSG00000268592	ENSG00000131015
ENSG00000281021	ENSG00000131015
ENSG00000268592	ENSG00000131023
ENSG00000235081	ENSG00000131037
ENSG00000267265	ENSG00000131037
ENSG00000267649	ENSG00000131037
ENSG00000267689	ENSG00000131037
ENSG00000228323	ENSG00000131042
ENSG00000237017	ENSG00000131042
ENSG00000267265	ENSG00000131042
ENSG00000267649	ENSG00000131042
ENSG00000088340	ENSG00000131043
ENSG00000232907	ENSG00000131043
ENSG00000260032	ENSG00000131043
ENSG00000088340	ENSG00000131051
ENSG00000232907	ENSG00000131051
ENSG00000230753	ENSG00000131061
ENSG00000088340	ENSG00000131067
ENSG00000228265	ENSG00000131069
ENSG00000235437	ENSG00000131089
ENSG00000236754	ENSG00000131100
ENSG00000280007	ENSG00000131100
ENSG00000196810	ENSG00000131127

ENSG00000222019	ENSG00000131165
ENSG00000224888	ENSG00000131165
ENSG00000261373	ENSG00000131165
ENSG00000278530	ENSG00000131171
ENSG00000237899	ENSG00000131236
ENSG00000260920	ENSG00000131236
ENSG00000281207	ENSG00000131236
ENSG00000225470	ENSG00000131263
ENSG00000230590	ENSG00000131263
ENSG00000270641	ENSG00000131263
ENSG00000271430	ENSG00000131263
ENSG00000225733	ENSG00000131368
ENSG00000249786	ENSG00000131368
ENSG00000224660	ENSG00000131381
ENSG00000225733	ENSG00000131381
ENSG00000249786	ENSG00000131386
ENSG00000272529	ENSG00000131386
ENSG00000131401	ENSG00000131398
ENSG00000273189	ENSG00000131398
ENSG00000223442	ENSG00000131435
ENSG00000233937	ENSG00000131446
ENSG00000245060	ENSG00000131446
ENSG00000250222	ENSG00000131446
ENSG00000233937	ENSG00000131459
ENSG00000245317	ENSG00000131459
ENSG00000248367	ENSG00000131459
ENSG00000188825	ENSG00000131471
ENSG00000197291	ENSG00000131471
ENSG00000213373	ENSG00000131471
ENSG00000249637	ENSG00000131503
ENSG00000272154	ENSG00000131503
ENSG00000280029	ENSG00000131503
ENSG00000254099	ENSG00000131504
ENSG00000272154	ENSG00000131504
ENSG00000278936	ENSG00000131504
ENSG00000280029	ENSG00000131504
ENSG00000249593	ENSG00000131508
ENSG00000249637	ENSG00000131508
ENSG00000177757	ENSG00000131584
ENSG00000268575	ENSG00000131584
ENSG00000261691	ENSG00000131634
ENSG00000205913	ENSG00000131653
ENSG00000262370	ENSG00000131653
ENSG00000225511	ENSG00000131669
ENSG00000236717	ENSG00000131669
ENSG00000214546	ENSG00000131748
ENSG00000265460	ENSG00000131748
ENSG00000277182	ENSG00000131748

ENSG00000237188	ENSG00000131778
ENSG00000278811	ENSG00000131778
ENSG00000234222	ENSG00000131779
ENSG00000237188	ENSG00000131781
ENSG00000278811	ENSG00000131781
ENSG00000233396	ENSG00000131788
ENSG00000234222	ENSG00000131788
ENSG00000237188	ENSG00000131791
ENSG00000278811	ENSG00000131791
ENSG00000185596	ENSG00000131871
ENSG00000259172	ENSG00000131871
ENSG00000272808	ENSG00000131871
ENSG00000259583	ENSG00000131873
ENSG00000270127	ENSG00000131876
ENSG00000272808	ENSG00000131876
ENSG00000258413	ENSG00000131981
ENSG00000267169	ENSG00000132000
ENSG00000267169	ENSG00000132002
ENSG00000267169	ENSG00000132003
ENSG00000267379	ENSG00000132003
ENSG00000267062	ENSG00000132004
ENSG00000267169	ENSG00000132005
ENSG00000234773	ENSG00000132010
ENSG00000267274	ENSG00000132010
ENSG00000236204	ENSG00000132031
ENSG00000255367	ENSG00000132109
ENSG00000232022	ENSG00000132128
ENSG00000227252	ENSG00000132330
ENSG00000223528	ENSG00000132334
ENSG00000262903	ENSG00000132359
ENSG00000263345	ENSG00000132359
ENSG00000231305	ENSG00000132394
ENSG00000228439	ENSG00000132423
ENSG00000224057	ENSG00000132434
ENSG00000163597	ENSG00000132471
ENSG00000267546	ENSG00000132471
ENSG00000267342	ENSG00000132478
ENSG00000163597	ENSG00000132481
ENSG00000267801	ENSG00000132481
ENSG00000229956	ENSG00000132485
ENSG00000256806	ENSG00000132507
ENSG00000215067	ENSG00000132510
ENSG00000265749	ENSG00000132510
ENSG00000215067	ENSG00000132522
ENSG00000256806	ENSG00000132522
ENSG00000215067	ENSG00000132530
ENSG00000256806	ENSG00000132530
ENSG00000215067	ENSG00000132535

ENSG00000256806	ENSG00000132535
ENSG00000224186	ENSG00000132570
ENSG00000273345	ENSG00000132570
ENSG00000227543	ENSG00000132581
ENSG00000264031	ENSG00000132581
ENSG00000263657	ENSG00000132589
ENSG00000276075	ENSG00000132600
ENSG00000196696	ENSG00000132612
ENSG00000196696	ENSG00000132613
ENSG00000214353	ENSG00000132613
ENSG00000260111	ENSG00000132613
ENSG00000235958	ENSG00000132635
ENSG00000225988	ENSG00000132639
ENSG00000236526	ENSG00000132640
ENSG00000233895	ENSG00000132669
ENSG00000225855	ENSG00000132676
ENSG00000236947	ENSG00000132676
ENSG00000246203	ENSG00000132676
ENSG00000229953	ENSG00000132677
ENSG00000160766	ENSG00000132680
ENSG00000229953	ENSG00000132680
ENSG00000246203	ENSG00000132680
ENSG00000229953	ENSG00000132688
ENSG00000272405	ENSG00000132688
ENSG00000229953	ENSG00000132692
ENSG00000224259	ENSG00000132716
ENSG00000228863	ENSG00000132716
ENSG00000160766	ENSG00000132718
ENSG00000246203	ENSG00000132718
ENSG00000272068	ENSG00000132718
ENSG00000272405	ENSG00000132718
ENSG00000232022	ENSG00000132763
ENSG00000224635	ENSG00000132821
ENSG00000223891	ENSG00000132823
ENSG00000244558	ENSG00000132823
ENSG00000223891	ENSG00000132824
ENSG00000244558	ENSG00000132824
ENSG00000225407	ENSG00000132846
ENSG00000250802	ENSG00000132846
ENSG00000186301	ENSG00000132881
ENSG00000237276	ENSG00000132881
ENSG00000268869	ENSG00000132881
ENSG00000249035	ENSG00000132912
ENSG00000260581	ENSG00000132912
ENSG00000179141	ENSG00000132963
ENSG00000215808	ENSG00000133019
ENSG00000263388	ENSG00000133028
ENSG00000263400	ENSG00000133028

ENSG00000264016	ENSG00000133028
ENSG00000281406	ENSG00000133065
ENSG00000231871	ENSG00000133067
ENSG00000120664	ENSG00000133101
ENSG00000120664	ENSG00000133104
ENSG00000120664	ENSG00000133111
ENSG00000170919	ENSG00000133114
ENSG00000227258	ENSG00000133114
ENSG00000236581	ENSG00000133116
ENSG00000236581	ENSG00000133119
ENSG00000236581	ENSG00000133121
ENSG00000239407	ENSG00000133134
ENSG00000270050	ENSG00000133169
ENSG00000271147	ENSG00000133169
ENSG00000226101	ENSG00000133195
ENSG00000227036	ENSG00000133195
ENSG00000249592	ENSG00000133256
ENSG00000256116	ENSG00000133321
ENSG00000250764	ENSG00000133401
ENSG00000228839	ENSG00000133422
ENSG00000225282	ENSG00000133433
ENSG00000183822	ENSG00000133466
ENSG00000235237	ENSG00000133466
ENSG00000250318	ENSG00000133488
ENSG00000240449	ENSG00000133612
ENSG00000133624	ENSG00000133619
ENSG00000240449	ENSG00000133619
ENSG00000245904	ENSG00000133639
ENSG00000257242	ENSG00000133639
ENSG00000229334	ENSG00000133657
ENSG00000231770	ENSG00000133657
ENSG00000233058	ENSG00000133657
ENSG00000244675	ENSG00000133657
ENSG00000225484	ENSG00000133678
ENSG00000230091	ENSG00000133678
ENSG00000242600	ENSG00000133678
ENSG00000251556	ENSG00000133706
ENSG00000254208	ENSG00000133739
ENSG00000247271	ENSG00000133800
ENSG00000255125	ENSG00000133800
ENSG00000246273	ENSG00000133812
ENSG00000247271	ENSG00000133812
ENSG00000251191	ENSG00000133872
ENSG00000272256	ENSG00000133872
ENSG00000258875	ENSG00000133943
ENSG00000253125	ENSG00000134013
ENSG00000253837	ENSG00000134013
ENSG00000227115	ENSG00000134042

ENSG00000179978	ENSG00000134056
ENSG00000248664	ENSG00000134056
ENSG00000231249	ENSG00000134109
ENSG00000233912	ENSG00000134109
ENSG00000235978	ENSG00000134109
ENSG00000234661	ENSG00000134121
ENSG00000259287	ENSG00000134152
ENSG00000228703	ENSG00000134184
ENSG00000241720	ENSG00000134184
ENSG00000228703	ENSG00000134186
ENSG00000241720	ENSG00000134201
ENSG00000241720	ENSG00000134202
ENSG00000224167	ENSG00000134242
ENSG00000226167	ENSG00000134242
ENSG00000238198	ENSG00000134242
ENSG00000227963	ENSG00000134243
ENSG00000224167	ENSG00000134245
ENSG00000203865	ENSG00000134247
ENSG00000187481	ENSG00000134250
ENSG00000203855	ENSG00000134250
ENSG00000227082	ENSG00000134250
ENSG00000227712	ENSG00000134250
ENSG00000231365	ENSG00000134250
ENSG00000224699	ENSG00000134255
ENSG00000235407	ENSG00000134255
ENSG00000240194	ENSG00000134255
ENSG00000224950	ENSG00000134256
ENSG00000236137	ENSG00000134256
ENSG00000267108	ENSG00000134278
ENSG00000267199	ENSG00000134278
ENSG00000257735	ENSG00000134285
ENSG00000257588	ENSG00000134287
ENSG00000257735	ENSG00000134287
ENSG00000258017	ENSG00000134287
ENSG00000258101	ENSG00000134287
ENSG00000257735	ENSG00000134291
ENSG00000257261	ENSG00000134294
ENSG00000235092	ENSG00000134313
ENSG00000235665	ENSG00000134313
ENSG00000236790	ENSG00000134313
ENSG00000228203	ENSG00000134321
ENSG00000223884	ENSG00000134326
ENSG00000254966	ENSG00000134333
ENSG00000248898	ENSG00000134363
ENSG00000249899	ENSG00000134363
ENSG00000223396	ENSG00000134369
ENSG00000231871	ENSG00000134369
ENSG00000235121	ENSG00000134369

ENSG00000223396	ENSG00000134375
ENSG00000234678	ENSG00000134375
ENSG00000243323	ENSG00000134375
ENSG00000267040	ENSG00000134440
ENSG00000267787	ENSG00000134440
ENSG00000215244	ENSG00000134461
ENSG00000264745	ENSG00000134508
ENSG00000264825	ENSG00000134508
ENSG00000247498	ENSG00000134531
ENSG00000214776	ENSG00000134539
ENSG00000256673	ENSG00000134539
ENSG00000256712	ENSG00000134539
ENSG00000257027	ENSG00000134539
ENSG00000247675	ENSG00000134569
ENSG00000247675	ENSG00000134574
ENSG00000255197	ENSG00000134574
ENSG00000223749	ENSG00000134590
ENSG00000232160	ENSG00000134602
ENSG00000255929	ENSG00000134627
ENSG00000235790	ENSG00000134668
ENSG00000225313	ENSG00000134684
ENSG00000225313	ENSG00000134686
ENSG00000266993	ENSG00000134717
ENSG00000223390	ENSG00000134744
ENSG00000227742	ENSG00000134748
ENSG00000260552	ENSG00000134759
ENSG00000274184	ENSG00000134759
ENSG00000268573	ENSG00000134769
ENSG00000268873	ENSG00000134769
ENSG00000243742	ENSG00000134780
ENSG00000243742	ENSG00000134824
ENSG00000249700	ENSG00000134851
ENSG00000249341	ENSG00000134853
ENSG00000227105	ENSG00000134871
ENSG00000255874	ENSG00000134871
ENSG00000275741	ENSG00000134905
ENSG00000257271	ENSG00000134910
ENSG00000248445	ENSG00000134970
ENSG00000249249	ENSG00000134970
ENSG00000246859	ENSG00000134986
ENSG00000246859	ENSG00000134987
ENSG00000225434	ENSG00000135046
ENSG00000226237	ENSG00000135047
ENSG00000234424	ENSG00000135049
ENSG00000226237	ENSG00000135070
ENSG00000258285	ENSG00000135108
ENSG00000212694	ENSG00000135114
ENSG00000249550	ENSG00000135144

ENSG00000230626	ENSG00000135245
ENSG00000280828	ENSG00000135245
ENSG00000281896	ENSG00000135245
ENSG00000197258	ENSG00000135250
ENSG00000228393	ENSG00000135250
ENSG00000237513	ENSG00000135250
ENSG00000272918	ENSG00000135250
ENSG00000272899	ENSG00000135253
ENSG00000273270	ENSG00000135253
ENSG00000280828	ENSG00000135253
ENSG00000226367	ENSG00000135269
ENSG00000243243	ENSG00000135269
ENSG00000225535	ENSG00000135272
ENSG00000229862	ENSG00000135297
ENSG00000217060	ENSG00000135316
ENSG00000183242	ENSG00000135378
ENSG00000255252	ENSG00000135378
ENSG00000182796	ENSG00000135392
ENSG00000257809	ENSG00000135392
ENSG00000248265	ENSG00000135409
ENSG00000250742	ENSG00000135409
ENSG00000257337	ENSG00000135409
ENSG00000257913	ENSG00000135436
ENSG00000258057	ENSG00000135436
ENSG00000258101	ENSG00000135436
ENSG00000257342	ENSG00000135439
ENSG00000257342	ENSG00000135446
ENSG00000248265	ENSG00000135447
ENSG00000258086	ENSG00000135447
ENSG00000260470	ENSG00000135447
ENSG00000259125	ENSG00000135452
ENSG00000224713	ENSG00000135454
ENSG00000257342	ENSG00000135454
ENSG00000259125	ENSG00000135454
ENSG00000259125	ENSG00000135469
ENSG00000258199	ENSG00000135482
ENSG00000224713	ENSG00000135502
ENSG00000257342	ENSG00000135502
ENSG00000257342	ENSG00000135506
ENSG00000219565	ENSG00000135537
ENSG00000272476	ENSG00000135537
ENSG00000231028	ENSG00000135541
ENSG00000234084	ENSG00000135541
ENSG00000163016	ENSG00000135624
ENSG00000272702	ENSG00000135624
ENSG00000225439	ENSG00000135625
ENSG00000163016	ENSG00000135631
ENSG00000163016	ENSG00000135637

ENSG00000257354	ENSG00000135655
ENSG00000243024	ENSG00000135677
ENSG00000250748	ENSG00000135677
ENSG00000247131	ENSG00000135679
ENSG00000257181	ENSG00000135679
ENSG00000261519	ENSG00000135723
ENSG00000270049	ENSG00000135723
ENSG00000276075	ENSG00000135723
ENSG00000276075	ENSG00000135740
ENSG00000177788	ENSG00000135763
ENSG00000183929	ENSG00000135776
ENSG00000224407	ENSG00000135776
ENSG00000232586	ENSG00000135835
ENSG00000243155	ENSG00000135835
ENSG00000232586	ENSG00000135837
ENSG00000243155	ENSG00000135837
ENSG00000236432	ENSG00000135917
ENSG00000229754	ENSG00000135929
ENSG00000237126	ENSG00000135930
ENSG00000225963	ENSG00000135932
ENSG00000281162	ENSG00000135953
ENSG00000204685	ENSG00000135956
ENSG00000197927	ENSG00000136002
ENSG00000246985	ENSG00000136040
ENSG00000257283	ENSG00000136040
ENSG00000257999	ENSG00000136051
ENSG00000235257	ENSG00000136059
ENSG00000242428	ENSG00000136068
ENSG00000244161	ENSG00000136068
ENSG00000244383	ENSG00000136068
ENSG00000236778	ENSG00000136098
ENSG00000243406	ENSG00000136098
ENSG00000281106	ENSG00000136098
ENSG00000236778	ENSG00000136100
ENSG00000250299	ENSG00000136100
ENSG00000233672	ENSG00000136104
ENSG00000261553	ENSG00000136111
ENSG00000260388	ENSG00000136143
ENSG00000235903	ENSG00000136152
ENSG00000170919	ENSG00000136167
ENSG00000235903	ENSG00000136167
ENSG00000225264	ENSG00000136193
ENSG00000227014	ENSG00000136193
ENSG00000227855	ENSG00000136193
ENSG00000281404	ENSG00000136193
ENSG00000214783	ENSG00000136206
ENSG00000272655	ENSG00000136206
ENSG00000273432	ENSG00000136206

ENSG00000214783	ENSG00000136271
ENSG00000273432	ENSG00000136271
ENSG00000225705	ENSG00000136273
ENSG00000232956	ENSG00000136274
ENSG00000272556	ENSG00000136274
ENSG00000272768	ENSG00000136274
ENSG00000214783	ENSG00000136279
ENSG00000228903	ENSG00000136279
ENSG00000235314	ENSG00000136279
ENSG00000272655	ENSG00000136279
ENSG00000273432	ENSG00000136279
ENSG00000214765	ENSG00000136280
ENSG00000228903	ENSG00000136280
ENSG00000232956	ENSG00000136280
ENSG00000272556	ENSG00000136280
ENSG00000214765	ENSG00000136286
ENSG00000272556	ENSG00000136286
ENSG00000236708	ENSG00000136295
ENSG00000157306	ENSG00000136305
ENSG00000215256	ENSG00000136305
ENSG00000157306	ENSG00000136367
ENSG00000215256	ENSG00000136367
ENSG00000259234	ENSG00000136371
ENSG00000259495	ENSG00000136371
ENSG00000259642	ENSG00000136371
ENSG00000278600	ENSG00000136371
ENSG00000259234	ENSG00000136378
ENSG00000260776	ENSG00000136378
ENSG00000259234	ENSG00000136381
ENSG00000260776	ENSG00000136381
ENSG00000261143	ENSG00000136381
ENSG00000189136	ENSG00000136383
ENSG00000225151	ENSG00000136383
ENSG00000235370	ENSG00000136383
ENSG00000230532	ENSG00000136436
ENSG00000275897	ENSG00000136444
ENSG00000224505	ENSG00000136448
ENSG00000251239	ENSG00000136449
ENSG00000265148	ENSG00000136450
ENSG00000265542	ENSG00000136451
ENSG00000275897	ENSG00000136457
ENSG00000265702	ENSG00000136463
ENSG00000233635	ENSG00000136490
ENSG00000263644	ENSG00000136490
ENSG00000265702	ENSG00000136490
ENSG00000197099	ENSG00000136514
ENSG00000197099	ENSG00000136527
ENSG00000223642	ENSG00000136536

ENSG00000226266	ENSG00000136536
ENSG00000227403	ENSG00000136560
ENSG00000235724	ENSG00000136560
ENSG00000248896	ENSG00000136574
ENSG00000255052	ENSG00000136574
ENSG00000269889	ENSG00000136603
ENSG00000237781	ENSG00000136631
ENSG00000230461	ENSG00000136643
ENSG00000189223	ENSG00000136682
ENSG00000240356	ENSG00000136682
ENSG00000243389	ENSG00000136682
ENSG00000189223	ENSG00000136689
ENSG00000240356	ENSG00000136689
ENSG00000178162	ENSG00000136710
ENSG00000230646	ENSG00000136710
ENSG00000272667	ENSG00000136720
ENSG00000204054	ENSG00000136802
ENSG00000228395	ENSG00000136807
ENSG00000257524	ENSG00000136807
ENSG00000204055	ENSG00000136816
ENSG00000232850	ENSG00000136830
ENSG00000257524	ENSG00000136830
ENSG00000257524	ENSG00000136840
ENSG00000257524	ENSG00000136859
ENSG00000226752	ENSG00000136861
ENSG00000228623	ENSG00000136866
ENSG00000228623	ENSG00000136867
ENSG00000230185	ENSG00000136867
ENSG00000228623	ENSG00000136868
ENSG00000257524	ENSG00000136877
ENSG00000237461	ENSG00000136891
ENSG00000249859	ENSG00000136997
ENSG00000228352	ENSG00000137070
ENSG00000230074	ENSG00000137070
ENSG00000261215	ENSG00000137070
ENSG00000226562	ENSG00000137073
ENSG00000235481	ENSG00000137073
ENSG00000226562	ENSG00000137074
ENSG00000227388	ENSG00000137094
ENSG00000228352	ENSG00000137094
ENSG00000227388	ENSG00000137098
ENSG00000227388	ENSG00000137103
ENSG00000227388	ENSG00000137133
ENSG00000235387	ENSG00000137133
ENSG00000245261	ENSG00000137161
ENSG00000237686	ENSG00000137171
ENSG00000261068	ENSG00000137171
ENSG00000237686	ENSG00000137207

ENSG00000237686	ENSG00000137216
ENSG00000228170	ENSG00000137267
ENSG00000250903	ENSG00000137267
ENSG00000228170	ENSG00000137274
ENSG00000250903	ENSG00000137275
ENSG00000272277	ENSG00000137275
ENSG00000228170	ENSG00000137285
ENSG00000250903	ENSG00000137285
ENSG00000206337	ENSG00000137310
ENSG00000181126	ENSG00000137312
ENSG00000206337	ENSG00000137312
ENSG00000214894	ENSG00000137312
ENSG00000224328	ENSG00000137312
ENSG00000270604	ENSG00000137312
ENSG00000271821	ENSG00000137312
ENSG00000206337	ENSG00000137331
ENSG00000214894	ENSG00000137331
ENSG00000270604	ENSG00000137331
ENSG00000271581	ENSG00000137331
ENSG00000206337	ENSG00000137337
ENSG00000227766	ENSG00000137337
ENSG00000230795	ENSG00000137337
ENSG00000233902	ENSG00000137337
ENSG00000226314	ENSG00000137338
ENSG00000269293	ENSG00000137338
ENSG00000206337	ENSG00000137343
ENSG00000206341	ENSG00000137343
ENSG00000214922	ENSG00000137343
ENSG00000270604	ENSG00000137343
ENSG00000206337	ENSG00000137404
ENSG00000214894	ENSG00000137404
ENSG00000270604	ENSG00000137404
ENSG00000214894	ENSG00000137411
ENSG00000256007	ENSG00000137478
ENSG00000260401	ENSG00000137478
ENSG00000254963	ENSG00000137486
ENSG00000241170	ENSG00000137491
ENSG00000255234	ENSG00000137500
ENSG00000255234	ENSG00000137509
ENSG00000245148	ENSG00000137522
ENSG00000255248	ENSG00000137642
ENSG00000282728	ENSG00000137672
ENSG00000282728	ENSG00000137693
ENSG00000254416	ENSG00000137710
ENSG00000196167	ENSG00000137714
ENSG00000254416	ENSG00000137714
ENSG00000255422	ENSG00000137726
ENSG00000254416	ENSG00000137727

ENSG00000278768	ENSG00000137747
ENSG00000259479	ENSG00000137770
ENSG00000259539	ENSG00000137770
ENSG00000259771	ENSG00000137776
ENSG00000259345	ENSG00000137801
ENSG00000258461	ENSG00000137814
ENSG00000261822	ENSG00000137814
ENSG00000261460	ENSG00000137817
ENSG00000248508	ENSG00000137841
ENSG00000205771	ENSG00000137842
ENSG00000258461	ENSG00000137842
ENSG00000259250	ENSG00000137845
ENSG00000259479	ENSG00000137857
ENSG00000259539	ENSG00000137857
ENSG00000259327	ENSG00000137869
ENSG00000223745	ENSG00000137936
ENSG00000224081	ENSG00000137936
ENSG00000224093	ENSG00000137936
ENSG00000260464	ENSG00000137936
ENSG00000240520	ENSG00000137941
ENSG00000237505	ENSG00000137944
ENSG00000235927	ENSG00000137965
ENSG00000233184	ENSG00000137992
ENSG00000224616	ENSG00000137996
ENSG00000231367	ENSG00000138061
ENSG00000232973	ENSG00000138061
ENSG00000235848	ENSG00000138061
ENSG00000236213	ENSG00000138061
ENSG00000172974	ENSG00000138069
ENSG00000234072	ENSG00000138073
ENSG00000214465	ENSG00000138080
ENSG00000225850	ENSG00000138131
ENSG00000196566	ENSG00000138138
ENSG00000223482	ENSG00000138138
ENSG00000225913	ENSG00000138138
ENSG00000226688	ENSG00000138185
ENSG00000244578	ENSG00000138231
ENSG00000227542	ENSG00000138378
ENSG00000231858	ENSG00000138378
ENSG00000228509	ENSG00000138386
ENSG00000229056	ENSG00000138411
ENSG00000224063	ENSG00000138448
ENSG00000227227	ENSG00000138448
ENSG00000242622	ENSG00000138495
ENSG00000259678	ENSG00000138594
ENSG00000259709	ENSG00000138594
ENSG00000259479	ENSG00000138606
ENSG00000259539	ENSG00000138606

ENSG00000259595	ENSG00000138606
ENSG00000260266	ENSG00000138623
ENSG00000271474	ENSG00000138696
ENSG00000246090	ENSG00000138698
ENSG00000260641	ENSG00000138698
ENSG00000245958	ENSG00000138735
ENSG00000250412	ENSG00000138735
ENSG00000251442	ENSG00000138756
ENSG00000249717	ENSG00000138757
ENSG00000251442	ENSG00000138759
ENSG00000245293	ENSG00000138796
ENSG00000247950	ENSG00000138802
ENSG00000248161	ENSG00000138821
ENSG00000245322	ENSG00000138823
ENSG00000246090	ENSG00000138823
ENSG00000248676	ENSG00000138823
ENSG00000260807	ENSG00000138834
ENSG00000228623	ENSG00000138835
ENSG00000228839	ENSG00000138942
ENSG00000226210	ENSG00000139044
ENSG00000214776	ENSG00000139112
ENSG00000214826	ENSG00000139112
ENSG00000245648	ENSG00000139112
ENSG00000256069	ENSG00000139112
ENSG00000225342	ENSG00000139116
ENSG00000226472	ENSG00000139146
ENSG00000255867	ENSG00000139146
ENSG00000253284	ENSG00000139154
ENSG00000215039	ENSG00000139178
ENSG00000256433	ENSG00000139178
ENSG00000205885	ENSG00000139182
ENSG00000215039	ENSG00000139182
ENSG00000205885	ENSG00000139190
ENSG00000205885	ENSG00000139193
ENSG00000215039	ENSG00000139193
ENSG00000219410	ENSG00000139193
ENSG00000205885	ENSG00000139194
ENSG00000257261	ENSG00000139209
ENSG00000257261	ENSG00000139211
ENSG00000257261	ENSG00000139218
ENSG00000257735	ENSG00000139219
ENSG00000197301	ENSG00000139233
ENSG00000241749	ENSG00000139233
ENSG00000250748	ENSG00000139233
ENSG00000256248	ENSG00000139233
ENSG00000257342	ENSG00000139266
ENSG00000258302	ENSG00000139318
ENSG00000257242	ENSG00000139329

ENSG00000257732	ENSG00000139372
ENSG00000257286	ENSG00000139405
ENSG00000249550	ENSG00000139410
ENSG00000255650	ENSG00000139428
ENSG00000275294	ENSG00000139517
ENSG00000257809	ENSG00000139540
ENSG00000248265	ENSG00000139546
ENSG00000258086	ENSG00000139546
ENSG00000257735	ENSG00000139549
ENSG00000258283	ENSG00000139549
ENSG00000248265	ENSG00000139572
ENSG00000250451	ENSG00000139572
ENSG00000250742	ENSG00000139572
ENSG00000258086	ENSG00000139572
ENSG00000236581	ENSG00000139597
ENSG00000272368	ENSG00000139624
ENSG00000248265	ENSG00000139625
ENSG00000250742	ENSG00000139625
ENSG00000257337	ENSG00000139625
ENSG00000258086	ENSG00000139625
ENSG00000248265	ENSG00000139626
ENSG00000248265	ENSG00000139631
ENSG00000257337	ENSG00000139631
ENSG00000257790	ENSG00000139631
ENSG00000257346	ENSG00000139636
ENSG00000257588	ENSG00000139636
ENSG00000257735	ENSG00000139636
ENSG00000258017	ENSG00000139636
ENSG00000258283	ENSG00000139636
ENSG00000248265	ENSG00000139637
ENSG00000250451	ENSG00000139637
ENSG00000250742	ENSG00000139637
ENSG00000257616	ENSG00000139637
ENSG00000258554	ENSG00000139641
ENSG00000258101	ENSG00000139644
ENSG00000272368	ENSG00000139644
ENSG00000248265	ENSG00000139651
ENSG00000257790	ENSG00000139651
ENSG00000257808	ENSG00000139651
ENSG00000233672	ENSG00000139668
ENSG00000236778	ENSG00000139668
ENSG00000281106	ENSG00000139668
ENSG00000275202	ENSG00000139679
ENSG00000256249	ENSG00000139718
ENSG00000256249	ENSG00000139719
ENSG00000212694	ENSG00000139725
ENSG00000256249	ENSG00000139725
ENSG00000272849	ENSG00000139725

ENSG00000274292	ENSG00000139725
ENSG00000212694	ENSG00000139726
ENSG00000235423	ENSG00000139726
ENSG00000256249	ENSG00000139726
ENSG00000272849	ENSG00000139726
ENSG00000236242	ENSG00000139826
ENSG00000233695	ENSG00000139842
ENSG00000260910	ENSG00000139842
ENSG00000157306	ENSG00000139880
ENSG00000215256	ENSG00000139880
ENSG00000251002	ENSG00000139880
ENSG00000251002	ENSG00000139890
ENSG00000157306	ENSG00000139908
ENSG00000276698	ENSG00000139908
ENSG00000259868	ENSG00000139946
ENSG00000258867	ENSG00000140030
ENSG00000258891	ENSG00000140043
ENSG00000259065	ENSG00000140043
ENSG00000227051	ENSG00000140057
ENSG00000214548	ENSG00000140105
ENSG00000223403	ENSG00000140105
ENSG00000247970	ENSG00000140105
ENSG00000258663	ENSG00000140105
ENSG00000258666	ENSG00000140105
ENSG00000276172	ENSG00000140157
ENSG00000179523	ENSG00000140254
ENSG00000259479	ENSG00000140254
ENSG00000259539	ENSG00000140254
ENSG00000259479	ENSG00000140263
ENSG00000259659	ENSG00000140263
ENSG00000259479	ENSG00000140274
ENSG00000259539	ENSG00000140274
ENSG00000259352	ENSG00000140279
ENSG00000259479	ENSG00000140279
ENSG00000259539	ENSG00000140279
ENSG00000259678	ENSG00000140280
ENSG00000259656	ENSG00000140285
ENSG00000259705	ENSG00000140285
ENSG00000259238	ENSG00000140299
ENSG00000259211	ENSG00000140320
ENSG00000259536	ENSG00000140320
ENSG00000259345	ENSG00000140323
ENSG00000258461	ENSG00000140326
ENSG00000278769	ENSG00000140326
ENSG00000259175	ENSG00000140379
ENSG00000259234	ENSG00000140379
ENSG00000259495	ENSG00000140379
ENSG00000260776	ENSG00000140391

ENSG00000261143	ENSG00000140391
ENSG00000246877	ENSG00000140398
ENSG00000260266	ENSG00000140398
ENSG00000260269	ENSG00000140398
ENSG00000260269	ENSG00000140400
ENSG00000259234	ENSG00000140403
ENSG00000259562	ENSG00000140403
ENSG00000261143	ENSG00000140403
ENSG00000259175	ENSG00000140406
ENSG00000259495	ENSG00000140406
ENSG00000259475	ENSG00000140443
ENSG00000261821	ENSG00000140459
ENSG00000259205	ENSG00000140471
ENSG00000259540	ENSG00000140471
ENSG00000260269	ENSG00000140474
ENSG00000259205	ENSG00000140479
ENSG00000259660	ENSG00000140479
ENSG00000270127	ENSG00000140479
ENSG00000272808	ENSG00000140479
ENSG00000255571	ENSG00000140526
ENSG00000255571	ENSG00000140545
ENSG00000259736	ENSG00000140548
ENSG00000224441	ENSG00000140553
ENSG00000258384	ENSG00000140553
ENSG00000259736	ENSG00000140575
ENSG00000259314	ENSG00000140577
ENSG00000259736	ENSG00000140577
ENSG00000260276	ENSG00000140650
ENSG00000230267	ENSG00000140675
ENSG00000232748	ENSG00000140675
ENSG00000260494	ENSG00000140675
ENSG00000261346	ENSG00000140678
ENSG00000230267	ENSG00000140682
ENSG00000261346	ENSG00000140682
ENSG00000230267	ENSG00000140688
ENSG00000235560	ENSG00000140688
ENSG00000239791	ENSG00000140688
ENSG00000260267	ENSG00000140691
ENSG00000260911	ENSG00000140691
ENSG00000183458	ENSG00000140694
ENSG00000188599	ENSG00000140694
ENSG00000250251	ENSG00000140694
ENSG00000262454	ENSG00000140694
ENSG00000270580	ENSG00000140694
ENSG00000275910	ENSG00000140694
ENSG00000180747	ENSG00000140740
ENSG00000189149	ENSG00000140740
ENSG00000248124	ENSG00000140740

ENSG00000180747	ENSG00000140749
ENSG00000260448	ENSG00000140750
ENSG00000262587	ENSG00000140750
ENSG00000260281	ENSG00000140795
ENSG00000255986	ENSG00000140848
ENSG00000260549	ENSG00000140848
ENSG00000277978	ENSG00000140931
ENSG00000246898	ENSG00000140939
ENSG00000261519	ENSG00000140939
ENSG00000236829	ENSG00000140983
ENSG00000262686	ENSG00000140987
ENSG00000267048	ENSG00000140995
ENSG00000223959	ENSG00000141002
ENSG00000259006	ENSG00000141002
ENSG00000267048	ENSG00000141002
ENSG00000261273	ENSG00000141012
ENSG00000222019	ENSG00000141013
ENSG00000175061	ENSG00000141040
ENSG00000261386	ENSG00000141086
ENSG00000263276	ENSG00000141086
ENSG00000227036	ENSG00000141219
ENSG00000265010	ENSG00000141219
ENSG00000263015	ENSG00000141252
ENSG00000263345	ENSG00000141258
ENSG00000228782	ENSG00000141279
ENSG00000234494	ENSG00000141279
ENSG00000262879	ENSG00000141279
ENSG00000263142	ENSG00000141279
ENSG00000263766	ENSG00000141279
ENSG00000214719	ENSG00000141298
ENSG00000248121	ENSG00000141298
ENSG00000264031	ENSG00000141298
ENSG00000264538	ENSG00000141298
ENSG00000237854	ENSG00000141337
ENSG00000231749	ENSG00000141338
ENSG00000267023	ENSG00000141338
ENSG00000188825	ENSG00000141349
ENSG00000259349	ENSG00000141367
ENSG00000267108	ENSG00000141385
ENSG00000267108	ENSG00000141391
ENSG00000267199	ENSG00000141391
ENSG00000268573	ENSG00000141429
ENSG00000235085	ENSG00000141456
ENSG00000261879	ENSG00000141456
ENSG00000235085	ENSG00000141480
ENSG00000261879	ENSG00000141497
ENSG00000215067	ENSG00000141499
ENSG00000265749	ENSG00000141499

ENSG00000261879	ENSG00000141503
ENSG00000264772	ENSG00000141504
ENSG00000215067	ENSG00000141510
ENSG00000256806	ENSG00000141510
ENSG00000263301	ENSG00000141510
ENSG00000262580	ENSG00000141527
ENSG00000226137	ENSG00000141543
ENSG00000263321	ENSG00000141556
ENSG00000279066	ENSG00000141562
ENSG00000261924	ENSG00000141564
ENSG00000263069	ENSG00000141564
ENSG00000163597	ENSG00000141569
ENSG00000267342	ENSG00000141569
ENSG00000267801	ENSG00000141569
ENSG00000260005	ENSG00000141577
ENSG00000262223	ENSG00000141577
ENSG00000263069	ENSG00000141577
ENSG00000227115	ENSG00000141642
ENSG00000227115	ENSG00000141646
ENSG00000236194	ENSG00000141696
ENSG00000267221	ENSG00000141696
ENSG00000188825	ENSG00000141699
ENSG00000267765	ENSG00000141699
ENSG00000261126	ENSG00000141759
ENSG00000266924	ENSG00000141759
ENSG00000261342	ENSG00000141873
ENSG00000267436	ENSG00000141873
ENSG00000267436	ENSG00000141905
ENSG00000184441	ENSG00000141959
ENSG00000267484	ENSG00000141965
ENSG00000267571	ENSG00000141968
ENSG00000254503	ENSG00000141971
ENSG00000269053	ENSG00000141971
ENSG00000269439	ENSG00000141971
ENSG00000269481	ENSG00000141971
ENSG00000269807	ENSG00000142002
ENSG00000256612	ENSG00000142039
ENSG00000267107	ENSG00000142039
ENSG00000268475	ENSG00000142039
ENSG00000130612	ENSG00000142046
ENSG00000268475	ENSG00000142046
ENSG00000270164	ENSG00000142046
ENSG00000251661	ENSG00000142082
ENSG00000250397	ENSG00000142089
ENSG00000251661	ENSG00000142089
ENSG00000255108	ENSG00000142089
ENSG00000255026	ENSG00000142102
ENSG00000228404	ENSG00000142156

ENSG00000238197	ENSG00000142166
ENSG00000215424	ENSG00000142173
ENSG00000229719	ENSG00000142186
ENSG00000245532	ENSG00000142186
ENSG00000273492	ENSG00000142192
ENSG00000232871	ENSG00000142233
ENSG00000267348	ENSG00000142252
ENSG00000267219	ENSG00000142279
ENSG00000227479	ENSG00000142327
ENSG00000267858	ENSG00000142396
ENSG00000268230	ENSG00000142396
ENSG00000268895	ENSG00000142396
ENSG00000267549	ENSG00000142409
ENSG00000266936	ENSG00000142444
ENSG00000266978	ENSG00000142453
ENSG00000154874	ENSG00000142494
ENSG00000228983	ENSG00000142494
ENSG00000262769	ENSG00000142494
ENSG00000232871	ENSG00000142552
ENSG00000235191	ENSG00000142552
ENSG00000123870	ENSG00000142556
ENSG00000213793	ENSG00000142556
ENSG00000242779	ENSG00000142556
ENSG00000260160	ENSG00000142556
ENSG00000269235	ENSG00000142556
ENSG00000269388	ENSG00000142556
ENSG00000228526	ENSG00000142599
ENSG00000232912	ENSG00000142599
ENSG00000234546	ENSG00000142599
ENSG00000080947	ENSG00000142627
ENSG00000226029	ENSG00000142627
ENSG00000237276	ENSG00000142627
ENSG00000237938	ENSG00000142627
ENSG00000268869	ENSG00000142627
ENSG00000237276	ENSG00000142632
ENSG00000175147	ENSG00000142634
ENSG00000225315	ENSG00000142661
ENSG00000225315	ENSG00000142677
ENSG00000264443	ENSG00000142677
ENSG00000227409	ENSG00000142686
ENSG00000116883	ENSG00000142694
ENSG00000248802	ENSG00000142731
ENSG00000251432	ENSG00000142731
ENSG00000235912	ENSG00000142784
ENSG00000228431	ENSG00000142864
ENSG00000282057	ENSG00000142871
ENSG00000122432	ENSG00000142875
ENSG00000240520	ENSG00000142875

ENSG00000186056	ENSG00000142910
ENSG00000223382	ENSG00000142910
ENSG00000235790	ENSG00000142910
ENSG00000225099	ENSG00000142949
ENSG00000230615	ENSG00000142949
ENSG00000230615	ENSG00000142959
ENSG00000232022	ENSG00000142961
ENSG00000224081	ENSG00000143036
ENSG00000236137	ENSG00000143061
ENSG00000198857	ENSG00000143067
ENSG00000224238	ENSG00000143067
ENSG00000231365	ENSG00000143067
ENSG00000240194	ENSG00000143110
ENSG00000224699	ENSG00000143119
ENSG00000227963	ENSG00000143119
ENSG00000231346	ENSG00000143119
ENSG00000224699	ENSG00000143125
ENSG00000240194	ENSG00000143125
ENSG00000225171	ENSG00000143157
ENSG00000198358	ENSG00000143224
ENSG00000228863	ENSG00000143224
ENSG00000198358	ENSG00000143226
ENSG00000224985	ENSG00000143226
ENSG00000228863	ENSG00000143226
ENSG00000232995	ENSG00000143248
ENSG00000228863	ENSG00000143257
ENSG00000236947	ENSG00000143314
ENSG00000198358	ENSG00000143315
ENSG00000224259	ENSG00000143315
ENSG00000228863	ENSG00000143315
ENSG00000232188	ENSG00000143315
ENSG00000224259	ENSG00000143318
ENSG00000228863	ENSG00000143318
ENSG00000229953	ENSG00000143319
ENSG00000236947	ENSG00000143319
ENSG00000272405	ENSG00000143320
ENSG00000236539	ENSG00000143340
ENSG00000203288	ENSG00000143367
ENSG00000229021	ENSG00000143367
ENSG00000237975	ENSG00000143367
ENSG00000249602	ENSG00000143367
ENSG00000237975	ENSG00000143373
ENSG00000249602	ENSG00000143373
ENSG00000203804	ENSG00000143379
ENSG00000249602	ENSG00000143379
ENSG00000237781	ENSG00000143382
ENSG00000224645	ENSG00000143384
ENSG00000237781	ENSG00000143387

ENSG00000203804	ENSG00000143390
ENSG00000229021	ENSG00000143390
ENSG00000234614	ENSG00000143390
ENSG00000237781	ENSG00000143390
ENSG00000237975	ENSG00000143390
ENSG00000237975	ENSG00000143398
ENSG00000249602	ENSG00000143398
ENSG00000237781	ENSG00000143401
ENSG00000237781	ENSG00000143409
ENSG00000237975	ENSG00000143416
ENSG00000249602	ENSG00000143416
ENSG00000203804	ENSG00000143420
ENSG00000249602	ENSG00000143434
ENSG00000234614	ENSG00000143436
ENSG00000237975	ENSG00000143436
ENSG00000249602	ENSG00000143436
ENSG00000249602	ENSG00000143437
ENSG00000203804	ENSG00000143442
ENSG00000224645	ENSG00000143442
ENSG00000237975	ENSG00000143442
ENSG00000249602	ENSG00000143442
ENSG00000229021	ENSG00000143450
ENSG00000234614	ENSG00000143450
ENSG00000237975	ENSG00000143450
ENSG00000249602	ENSG00000143450
ENSG00000224645	ENSG00000143458
ENSG00000229021	ENSG00000143458
ENSG00000234614	ENSG00000143458
ENSG00000153363	ENSG00000143493
ENSG00000230461	ENSG00000143494
ENSG00000230461	ENSG00000143499
ENSG00000237975	ENSG00000143546
ENSG00000272030	ENSG00000143549
ENSG00000273026	ENSG00000143578
ENSG00000246203	ENSG00000143622
ENSG00000229021	ENSG00000143631
ENSG00000237975	ENSG00000143631
ENSG00000224407	ENSG00000143641
ENSG00000227006	ENSG00000143641
ENSG00000223519	ENSG00000143653
ENSG00000227953	ENSG00000143653
ENSG00000228643	ENSG00000143727
ENSG00000227496	ENSG00000143742
ENSG00000185495	ENSG00000143753
ENSG00000272645	ENSG00000143753
ENSG00000272645	ENSG00000143786
ENSG00000227496	ENSG00000143811
ENSG00000223396	ENSG00000143851

ENSG00000231871	ENSG00000143851
ENSG00000234678	ENSG00000143851
ENSG00000243323	ENSG00000143851
ENSG00000223396	ENSG00000143862
ENSG00000231871	ENSG00000143862
ENSG00000234678	ENSG00000143862
ENSG00000243323	ENSG00000143862
ENSG00000231367	ENSG00000143889
ENSG00000232973	ENSG00000143889
ENSG00000235848	ENSG00000143889
ENSG00000236213	ENSG00000143889
ENSG00000231367	ENSG00000143891
ENSG00000232973	ENSG00000143891
ENSG00000235848	ENSG00000143891
ENSG00000223935	ENSG00000143952
ENSG00000224165	ENSG00000143970
ENSG00000204685	ENSG00000144021
ENSG00000232931	ENSG00000144029
ENSG00000228384	ENSG00000144031
ENSG00000163016	ENSG00000144036
ENSG00000163016	ENSG00000144040
ENSG00000228384	ENSG00000144043
ENSG00000204581	ENSG00000144063
ENSG00000224789	ENSG00000144118
ENSG00000243389	ENSG00000144130
ENSG00000234199	ENSG00000144134
ENSG00000240356	ENSG00000144134
ENSG00000189223	ENSG00000144136
ENSG00000240356	ENSG00000144136
ENSG00000243389	ENSG00000144136
ENSG00000272667	ENSG00000144230
ENSG00000274010	ENSG00000144231
ENSG00000224063	ENSG00000144366
ENSG00000229127	ENSG00000144445
ENSG00000234281	ENSG00000144445
ENSG00000227252	ENSG00000144476
ENSG00000223923	ENSG00000144580
ENSG00000249786	ENSG00000144597
ENSG00000213842	ENSG00000144635
ENSG00000261572	ENSG00000144635
ENSG00000235904	ENSG00000144642
ENSG00000198590	ENSG00000144668
ENSG00000230067	ENSG00000144668
ENSG00000235257	ENSG00000144668
ENSG00000236411	ENSG00000144668
ENSG00000213943	ENSG00000144712
ENSG00000241472	ENSG00000144724
ENSG00000241163	ENSG00000144736

ENSG00000244513	ENSG00000144746
ENSG00000244513	ENSG00000144747
ENSG00000236869	ENSG00000144792
ENSG00000272077	ENSG00000144792
ENSG00000239462	ENSG00000144810
ENSG00000242622	ENSG00000144840
ENSG00000240254	ENSG00000144852
ENSG00000239482	ENSG00000144857
ENSG00000243795	ENSG00000144857
ENSG00000242337	ENSG00000144867
ENSG00000246022	ENSG00000144908
ENSG00000250934	ENSG00000144908
ENSG00000241570	ENSG00000144935
ENSG00000229334	ENSG00000145014
ENSG00000231770	ENSG00000145014
ENSG00000233058	ENSG00000145014
ENSG00000214135	ENSG00000145016
ENSG00000272989	ENSG00000145016
ENSG00000225399	ENSG00000145022
ENSG00000235016	ENSG00000145022
ENSG00000223343	ENSG00000145029
ENSG00000235016	ENSG00000145029
ENSG00000224769	ENSG00000145113
ENSG00000231443	ENSG00000145113
ENSG00000233441	ENSG00000145194
ENSG00000233441	ENSG00000145198
ENSG00000196810	ENSG00000145217
ENSG00000248416	ENSG00000145217
ENSG00000250846	ENSG00000145242
ENSG00000248254	ENSG00000145246
ENSG00000245322	ENSG00000145331
ENSG00000246090	ENSG00000145331
ENSG00000248676	ENSG00000145331
ENSG00000251095	ENSG00000145335
ENSG00000248161	ENSG00000145354
ENSG00000154608	ENSG00000145390
ENSG00000245958	ENSG00000145390
ENSG00000250412	ENSG00000145390
ENSG00000196951	ENSG00000145391
ENSG00000273247	ENSG00000145391
ENSG00000250056	ENSG00000145545
ENSG00000215196	ENSG00000145555
ENSG00000244968	ENSG00000145623
ENSG00000249740	ENSG00000145623
ENSG00000247572	ENSG00000145687
ENSG00000225407	ENSG00000145708
ENSG00000250802	ENSG00000145708
ENSG00000248664	ENSG00000145740

ENSG00000249476	ENSG00000145743
ENSG00000246859	ENSG00000145777
ENSG00000249249	ENSG00000145780
ENSG00000248445	ENSG00000145782
ENSG00000224186	ENSG00000145824
ENSG00000247699	ENSG00000145861
ENSG00000253406	ENSG00000145882
ENSG00000253618	ENSG00000145882
ENSG00000249035	ENSG00000145907
ENSG00000197083	ENSG00000145908
ENSG00000249035	ENSG00000145908
ENSG00000246596	ENSG00000145911
ENSG00000216657	ENSG00000145945
ENSG00000230438	ENSG00000145945
ENSG00000250903	ENSG00000145949
ENSG00000233937	ENSG00000146054
ENSG00000250900	ENSG00000146054
ENSG00000233937	ENSG00000146063
ENSG00000247049	ENSG00000146063
ENSG00000250900	ENSG00000146063
ENSG00000248996	ENSG00000146067
ENSG00000246334	ENSG00000146083
ENSG00000247679	ENSG00000146094
ENSG00000224843	ENSG00000146109
ENSG00000206337	ENSG00000146112
ENSG00000214894	ENSG00000146112
ENSG00000270604	ENSG00000146112
ENSG00000272221	ENSG00000146112
ENSG00000246982	ENSG00000146192
ENSG00000237686	ENSG00000146216
ENSG00000237686	ENSG00000146232
ENSG00000236548	ENSG00000146373
ENSG00000231329	ENSG00000146386
ENSG00000227954	ENSG00000146409
ENSG00000227954	ENSG00000146411
ENSG00000236700	ENSG00000146411
ENSG00000233452	ENSG00000146414
ENSG00000270638	ENSG00000146414
ENSG00000224460	ENSG00000146416
ENSG00000218226	ENSG00000146433
ENSG00000236708	ENSG00000146555
ENSG00000176826	ENSG00000146648
ENSG00000214765	ENSG00000146676
ENSG00000214783	ENSG00000146676
ENSG00000228903	ENSG00000146676
ENSG00000273432	ENSG00000146676
ENSG00000186704	ENSG00000146700
ENSG00000205485	ENSG00000146700

ENSG00000214439	ENSG00000146700
ENSG00000227038	ENSG00000146700
ENSG00000186704	ENSG00000146701
ENSG00000205485	ENSG00000146701
ENSG00000227038	ENSG00000146701
ENSG00000176826	ENSG00000146731
ENSG00000176826	ENSG00000146733
ENSG00000226851	ENSG00000146802
ENSG00000242798	ENSG00000146826
ENSG00000242798	ENSG00000146828
ENSG00000078319	ENSG00000146834
ENSG00000242294	ENSG00000146834
ENSG00000272752	ENSG00000146834
ENSG00000226380	ENSG00000146842
ENSG00000231721	ENSG00000146842
ENSG00000233559	ENSG00000146842
ENSG00000240571	ENSG00000146842
ENSG00000273329	ENSG00000146842
ENSG00000223500	ENSG00000146859
ENSG00000229153	ENSG00000146904
ENSG00000232533	ENSG00000146904
ENSG00000244198	ENSG00000146904
ENSG00000244479	ENSG00000146904
ENSG00000253882	ENSG00000146904
ENSG00000226679	ENSG00000147036
ENSG00000235806	ENSG00000147041
ENSG00000225470	ENSG00000147100
ENSG00000230590	ENSG00000147100
ENSG00000269904	ENSG00000147100
ENSG00000229563	ENSG00000147121
ENSG00000270069	ENSG00000147121
ENSG00000215284	ENSG00000147123
ENSG00000235636	ENSG00000147123
ENSG00000232611	ENSG00000147274
ENSG00000173295	ENSG00000147324
ENSG00000253981	ENSG00000147324
ENSG00000182366	ENSG00000147364
ENSG00000249868	ENSG00000147364
ENSG00000232725	ENSG00000147394
ENSG00000260081	ENSG00000147400
ENSG00000253125	ENSG00000147454
ENSG00000253476	ENSG00000147459
ENSG00000253161	ENSG00000147465
ENSG00000253161	ENSG00000147475
ENSG00000253161	ENSG00000147535
ENSG00000253356	ENSG00000147535
ENSG00000272092	ENSG00000147535
ENSG00000253161	ENSG00000147548

ENSG00000253356	ENSG00000147548
ENSG00000246366	ENSG00000147592
ENSG00000253258	ENSG00000147689
ENSG00000232600	ENSG00000147789
ENSG00000255050	ENSG00000147813
ENSG00000225472	ENSG00000147862
ENSG00000171889	ENSG00000147883
ENSG00000236739	ENSG00000147883
ENSG00000233262	ENSG00000148057
ENSG00000228623	ENSG00000148158
ENSG00000230185	ENSG00000148225
ENSG00000235106	ENSG00000148297
ENSG00000119440	ENSG00000148308
ENSG00000204054	ENSG00000148331
ENSG00000204055	ENSG00000148331
ENSG00000234789	ENSG00000148331
ENSG00000257524	ENSG00000148334
ENSG00000204054	ENSG00000148335
ENSG00000204055	ENSG00000148335
ENSG00000234789	ENSG00000148335
ENSG00000204055	ENSG00000148337
ENSG00000234771	ENSG00000148339
ENSG00000204054	ENSG00000148341
ENSG00000204054	ENSG00000148343
ENSG00000204055	ENSG00000148343
ENSG00000228395	ENSG00000148343
ENSG00000234789	ENSG00000148344
ENSG00000196366	ENSG00000148362
ENSG00000237886	ENSG00000148362
ENSG00000196366	ENSG00000148396
ENSG00000237339	ENSG00000148396
ENSG00000203993	ENSG00000148399
ENSG00000237339	ENSG00000148400
ENSG00000180539	ENSG00000148411
ENSG00000264527	ENSG00000148411
ENSG00000224215	ENSG00000148450
ENSG00000196566	ENSG00000148671
ENSG00000223482	ENSG00000148671
ENSG00000225913	ENSG00000148671
ENSG00000272631	ENSG00000148671
ENSG00000196566	ENSG00000148672
ENSG00000223482	ENSG00000148672
ENSG00000225913	ENSG00000148672
ENSG00000272734	ENSG00000148672
ENSG00000282772	ENSG00000148795
ENSG00000214279	ENSG00000148803
ENSG00000234311	ENSG00000148803
ENSG00000270075	ENSG00000148834

ENSG00000273485	ENSG00000148843
ENSG00000223528	ENSG00000148848
ENSG00000246273	ENSG00000148926
ENSG00000246250	ENSG00000149084
ENSG00000183242	ENSG00000149100
ENSG00000255252	ENSG00000149100
ENSG00000254602	ENSG00000149115
ENSG00000255929	ENSG00000149212
ENSG00000255929	ENSG00000149218
ENSG00000254459	ENSG00000149262
ENSG00000254433	ENSG00000149313
ENSG00000256633	ENSG00000149357
ENSG00000124915	ENSG00000149476
ENSG00000256813	ENSG00000149485
ENSG00000243742	ENSG00000149489
ENSG00000280832	ENSG00000149557
ENSG00000257271	ENSG00000149571
ENSG00000280832	ENSG00000149571
ENSG00000254851	ENSG00000149577
ENSG00000278768	ENSG00000149577
ENSG00000198547	ENSG00000149599
ENSG00000236772	ENSG00000149599
ENSG00000269549	ENSG00000149634
ENSG00000273828	ENSG00000149634
ENSG00000173727	ENSG00000149792
ENSG00000251562	ENSG00000149798
ENSG00000173727	ENSG00000149809
ENSG00000245532	ENSG00000149809
ENSG00000255200	ENSG00000149823
ENSG00000198106	ENSG00000149925
ENSG00000260517	ENSG00000149925
ENSG00000261346	ENSG00000149925
ENSG00000261416	ENSG00000149925
ENSG00000183604	ENSG00000149927
ENSG00000198106	ENSG00000149927
ENSG00000205534	ENSG00000149927
ENSG00000250616	ENSG00000149927
ENSG00000260494	ENSG00000149927
ENSG00000260517	ENSG00000149927
ENSG00000260953	ENSG00000149927
ENSG00000103472	ENSG00000149930
ENSG00000198106	ENSG00000149930
ENSG00000260517	ENSG00000149930
ENSG00000260908	ENSG00000149930
ENSG00000261346	ENSG00000149930
ENSG00000183604	ENSG00000149932
ENSG00000198106	ENSG00000149932
ENSG00000205534	ENSG00000149932

ENSG00000260517	ENSG00000149932
ENSG00000250748	ENSG00000149948
ENSG00000229656	ENSG00000150076
ENSG00000229656	ENSG00000150093
ENSG00000226067	ENSG00000150337
ENSG00000237781	ENSG00000150337
ENSG00000237233	ENSG00000150347
ENSG00000276248	ENSG00000150401
ENSG00000254607	ENSG00000150455
ENSG00000255062	ENSG00000150455
ENSG00000280832	ENSG00000150455
ENSG00000248692	ENSG00000150471
ENSG00000248968	ENSG00000150753
ENSG00000271980	ENSG00000150756
ENSG00000223528	ENSG00000150760
ENSG00000196167	ENSG00000150764
ENSG00000247416	ENSG00000150768
ENSG00000213234	ENSG00000150779
ENSG00000196167	ENSG00000150782
ENSG00000223685	ENSG00000150893
ENSG00000168852	ENSG00000150907
ENSG00000215483	ENSG00000150907
ENSG00000230710	ENSG00000150907
ENSG00000154608	ENSG00000150961
ENSG00000250412	ENSG00000150961
ENSG00000256249	ENSG00000150977
ENSG00000235978	ENSG00000150995
ENSG00000261346	ENSG00000151006
ENSG00000273247	ENSG00000151014
ENSG00000235049	ENSG00000151062
ENSG00000235049	ENSG00000151065
ENSG00000246627	ENSG00000151067
ENSG00000254966	ENSG00000151116
ENSG00000212789	ENSG00000151117
ENSG00000255650	ENSG00000151148
ENSG00000228748	ENSG00000151208
ENSG00000236467	ENSG00000151208
ENSG00000223652	ENSG00000151292
ENSG00000254409	ENSG00000151348
ENSG00000246174	ENSG00000151366
ENSG00000258580	ENSG00000151413
ENSG00000258569	ENSG00000151445
ENSG00000261324	ENSG00000151490
ENSG00000251226	ENSG00000151502
ENSG00000232934	ENSG00000151532
ENSG00000231858	ENSG00000151690
ENSG00000233110	ENSG00000151726
ENSG00000249096	ENSG00000151726

ENSG00000250410	ENSG00000151726
ENSG00000251139	ENSG00000151726
ENSG00000235902	ENSG00000151729
ENSG00000257530	ENSG00000151746
ENSG00000227258	ENSG00000151778
ENSG00000177738	ENSG00000151881
ENSG00000248464	ENSG00000151881
ENSG00000260572	ENSG00000151967
ENSG00000178162	ENSG00000152076
ENSG00000178162	ENSG00000152082
ENSG00000242193	ENSG00000152092
ENSG00000254154	ENSG00000152092
ENSG00000152117	ENSG00000152102
ENSG00000178162	ENSG00000152102
ENSG00000230461	ENSG00000152104
ENSG00000250536	ENSG00000152104
ENSG00000224043	ENSG00000152127
ENSG00000213144	ENSG00000152137
ENSG00000231367	ENSG00000152147
ENSG00000232973	ENSG00000152147
ENSG00000235848	ENSG00000152147
ENSG00000271443	ENSG00000152147
ENSG00000274776	ENSG00000152242
ENSG00000246575	ENSG00000152291
ENSG00000267858	ENSG00000152443
ENSG00000268205	ENSG00000152443
ENSG00000268230	ENSG00000152443
ENSG00000272853	ENSG00000152457
ENSG00000268379	ENSG00000152467
ENSG00000268516	ENSG00000152467
ENSG00000232098	ENSG00000152475
ENSG00000268230	ENSG00000152475
ENSG00000269054	ENSG00000152475
ENSG00000275294	ENSG00000152484
ENSG00000257735	ENSG00000152556
ENSG00000243305	ENSG00000152601
ENSG00000250271	ENSG00000152601
ENSG00000224186	ENSG00000152700
ENSG00000273345	ENSG00000152700
ENSG00000180139	ENSG00000152766
ENSG00000226002	ENSG00000152926
ENSG00000234585	ENSG00000152926
ENSG00000179978	ENSG00000152939
ENSG00000243415	ENSG00000152952
ENSG00000232586	ENSG00000153029
ENSG00000243155	ENSG00000153029
ENSG00000260276	ENSG00000153048
ENSG00000244968	ENSG00000153071

ENSG00000249740	ENSG00000153071
ENSG00000251314	ENSG00000153113
ENSG00000196951	ENSG00000153132
ENSG00000249661	ENSG00000153132
ENSG00000236296	ENSG00000153147
ENSG00000250326	ENSG00000153147
ENSG00000251600	ENSG00000153147
ENSG00000172965	ENSG00000153208
ENSG00000243389	ENSG00000153214
ENSG00000226266	ENSG00000153250
ENSG00000227403	ENSG00000153250
ENSG00000235724	ENSG00000153250
ENSG00000263823	ENSG00000153339
ENSG00000185986	ENSG00000153395
ENSG00000251532	ENSG00000153395
ENSG00000267072	ENSG00000153443
ENSG00000227105	ENSG00000153487
ENSG00000232814	ENSG00000153487
ENSG00000222041	ENSG00000153563
ENSG00000280721	ENSG00000153563
ENSG00000235448	ENSG00000153714
ENSG00000236432	ENSG00000153820
ENSG00000225963	ENSG00000153827
ENSG00000277013	ENSG00000153885
ENSG00000223653	ENSG00000153904
ENSG00000282057	ENSG00000153904
ENSG00000267272	ENSG00000153936
ENSG00000265542	ENSG00000153944
ENSG00000141028	ENSG00000153976
ENSG00000236088	ENSG00000153976
ENSG00000265148	ENSG00000153982
ENSG00000267248	ENSG00000153982
ENSG00000154874	ENSG00000154025
ENSG00000262769	ENSG00000154025
ENSG00000273018	ENSG00000154025
ENSG00000232295	ENSG00000154079
ENSG00000233237	ENSG00000154079
ENSG00000227742	ENSG00000154222
ENSG00000215023	ENSG00000154252
ENSG00000215692	ENSG00000154252
ENSG00000224272	ENSG00000154252
ENSG00000233806	ENSG00000154252
ENSG00000231749	ENSG00000154258
ENSG00000231749	ENSG00000154262
ENSG00000267023	ENSG00000154262
ENSG00000231749	ENSG00000154263
ENSG00000267603	ENSG00000154263
ENSG00000227220	ENSG00000154269

ENSG00000282111	ENSG00000154358
ENSG00000227496	ENSG00000154380
ENSG00000270598	ENSG00000154380
ENSG00000231999	ENSG00000154451
ENSG00000235902	ENSG00000154556
ENSG00000215386	ENSG00000154639
ENSG00000244676	ENSG00000154642
ENSG00000244676	ENSG00000154645
ENSG00000265487	ENSG00000154655
ENSG00000234883	ENSG00000154721
ENSG00000273492	ENSG00000154721
ENSG00000273492	ENSG00000154727
ENSG00000267745	ENSG00000154760
ENSG00000263627	ENSG00000154845
ENSG00000265962	ENSG00000154845
ENSG00000229980	ENSG00000154945
ENSG00000251239	ENSG00000154945
ENSG00000224057	ENSG00000154978
ENSG00000204685	ENSG00000155066
ENSG00000232717	ENSG00000155066
ENSG00000253320	ENSG00000155090
ENSG00000254281	ENSG00000155090
ENSG00000253320	ENSG00000155096
ENSG00000254236	ENSG00000155096
ENSG00000254281	ENSG00000155096
ENSG00000253320	ENSG00000155097
ENSG00000253669	ENSG00000155097
ENSG00000254281	ENSG00000155097
ENSG00000231889	ENSG00000155111
ENSG00000231889	ENSG00000155115
[truncated: 172,804 more chars]
